# Differential impact of COVID-19 non-pharmaceutical interventions on the epidemiological dynamics of respiratory syncytial virus subtypes A and B

**DOI:** 10.1038/s41598-024-64624-1

**Published:** 2024-06-24

**Authors:** Inga Holmdahl, Samantha J. Bents, Rachel E. Baker, Jean-Sebastien Casalegno, Nídia Sequeira Trovão, Sang Woo Park, Jessica E. Metcalf, Cécile Viboud, Bryan Grenfell

**Affiliations:** 1 Department of Ecology and Evolutionary Biology, Princeton University, Princeton, NJ USA; 2 Fogarty International Center, National Institutes of Health, Bethesda, MD USA; 3 School of Public Health, Brown University, Providence, RI USA; 4 Hospices Civils de Lyon, Hôpital de la Croix-Rousse, Centre de Biologie Nord, Institut des Agents Infectieux, Laboratoire de Virologie, Lyon, France

**Keywords:** Viral infection, Computational models

## Abstract

Nonpharmaceutical interventions (NPIs) implemented during the COVID-19 pandemic have disrupted the dynamics of respiratory syncytial virus (RSV) on a global scale; however, the cycling of RSV subtypes in the pre- and post-pandemic period remains poorly understood. Here, we used a two subtype RSV model supplemented with epidemiological data to study the impact of NPIs on the two circulating subtypes, RSV-A and RSV-B. The model is calibrated to historic RSV subtype data from the United Kingdom and Finland and predicts a tendency for RSV-A dominance over RSV-B immediately following the implementation of NPIs. Using a global genetic dataset, we confirm that RSV-A has prevailed over RSV-B in the post-pandemic period, consistent with a higher R_0_ for RSV-A. With new RSV infant monoclonals and maternal and elderly vaccines becoming widely available, these results may have important implications for understanding intervention effectiveness in the context of disrupted subtype dynamics.

## Introduction

During the COVID-19 pandemic, non-pharmaceutical interventions (NPIs), such as social distancing, mask use, travel restrictions, and school closures, were imposed to reduce the transmission of SARS-COV-2^1^. These measures were effective in preventing transmission of other endemic pathogens such as influenza, respiratory syncytial virus (RSV), and other common respiratory diseases^2,3^. The typical annual or biennial seasonality of these endemic pathogens was interrupted abruptly during the intervention period (2020–2023)^4,5^. In the case of RSV, there was a global decline in cases followed by strong, out-of-season resurgences as COVID-19 NPIs were lifted. In the subsequent winter season of 2022–23, there was a notably large and early surge in RSV cases in the northern hemisphere, suggesting RSV circulation had not yet returned to pre-pandemic dynamics^6^. Simple models explain these patterns, with major outbreaks driven by a buildup of primary susceptible individuals during the NPI period^7^.

RSV primarily causes severe infection in infants, young children, and the elderly^8^. There are two antigenically distinct subtypes of RSV, A and B, that can cocirculate both within and between epidemic seasons^9^. Severity rates are comparable between the two subtypes, but some studies have reported that RSV-A may spread quicker and be responsible for more severe infections in young children due to higher transmissibility and genetic variability^10–12^. RSV A and B have been shown to cause similar host immune responses, and household transmission studies have indicated that homotypic immunity is stronger than heterotypic immunity after infection with both subtypes^13^.

Historical subtype dynamics explored by a simple two subtype model successfully captured complex recurrent epidemics, where A and B patterns repeat on a 10–12-year cycle and show slight dominance of subtype A across locations^14^. The model estimates a higher reproductive number R_0_ for subtype A, indicating that those infected with RSV-A transmit the disease to a high number of secondary contacts than those infected with RSV-B. The COVID-19 disruption constitutes a unique natural experiment to revisit existing hypotheses about the endemic dynamics of respiratory pathogens, including the cycling between RSV subtypes. Here, we used a mathematical model previously developed by White et al.^14^ and calibrated to historical data from the UK and Finland to make predictions about the transmission dynamics of RSV subtypes throughout the implementation of COVID-19 NPIs. We then compared our model predictions to empirical patterns of subtype-specific RSV resurgence from a global dataset over 2019–2023. With new RSV interventions becoming available, it will be important to understand how baseline RSV transmission dynamics have changed in order to interpret perceived benefits of the interventions^15–18^.

## Results

### Modeling results

**Figure 1:**
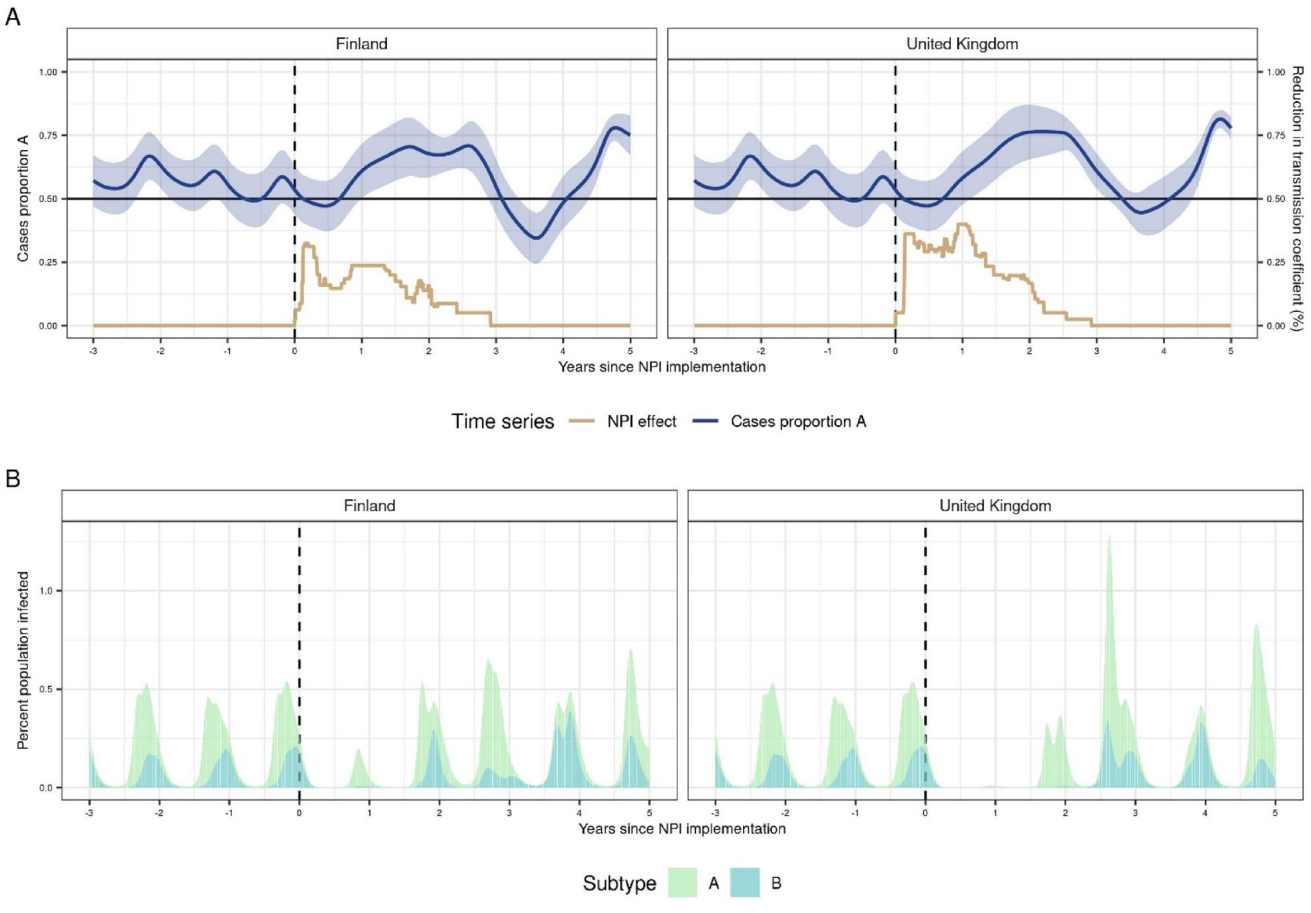
(**A**) Mean subtype dynamics over the first 5 years after NPI implementation. The start of NPIs in all figures is indicated with a dashed black line. Top: The mean proportion of all cases caused by subtype A over time. Ribbon shows the 95% confidence interval. The tan line below shows NPI strength over time, as a proportion reduction in the transmission coefficient. NPI strength is higher in the first year than in year 2 or year 3 and varies between locations. (**B**) The mean proportion of the population infected with RSV over time across simulations. Subtype A is shown in green and subtype B in blue. In Finland-like scenarios (left), the first RSV season that occurs during the COVID-19 pandemic has a very small epidemic, made up almost entirely of subtype A. The second is more typical, and still primarily subtype A. In the UK-like scenarios (right) there is no RSV epidemic in the first season after NPIs are imposed, and a smaller than typical epidemic, made up of entirely subtype A, in the second season. The third is much larger, but still primarily subtype A.

We adapted a deterministic, two-subtype model originally parameterized to historical data from Finland and the UK to explore the impact of COVID-19 NPIs on subtype-specific RSV transmission for up to 75 years after the pandemic. The original model incorporated seasonality and partial cross-immunity between RSV A and B subtypes and found a 10–12-year periodicity in the dynamics between the two subtypes^14^. Accordingly, we simulated the introduction of NPIs over an 11-year period in the UK and Finland to test how RSV subtype dynamics may respond across a plausible range of background subtype dynamics. In Fig. 1, we show the mean simulated subtype dynamics over the 5 years following implementation of NPIs in the UK-like and Finland-like scenarios. Consistent with White et al., subtype A caused a higher proportion of cases on average in the pre-pandemic period, although individual simulations showed that A and B predominate in alternating seasons (Figure S3). Due to cross-immunity and a higher R_0_ in subtype A, RSV outbreaks during subtype A seasons tended to be larger than those during subtype B seasons.

We allowed NPIs to reduce transmission by a maximum of 40%, corresponding to a maximum impact of 39% in the UK and 32% in Finland. In our model projections, there was either a reduction in cases or a delayed outbreak the following season after NPIs were imposed. In Finland-like scenarios, due to weaker estimated NPI stringency, a large resurgence occurred during the second year of NPIs; in the UK-like scenarios however, where NPI stringency remained higher over time, a large resurgence did not occur until the third year of NPIs. This first RSV resurgence after NPI onset was dominated by subtype A in both locations. Empirical data from Finland and the UK showed that RSV rebounded in late 2020 and early 2021 respectively, generally aligning with our model projections. In sensitivity analyses, we varied the maximum impact of NPIs between 30 and 50% and found that the model results were robust to a range of perturbation (Figures S1–S2).

**Figure 2:**
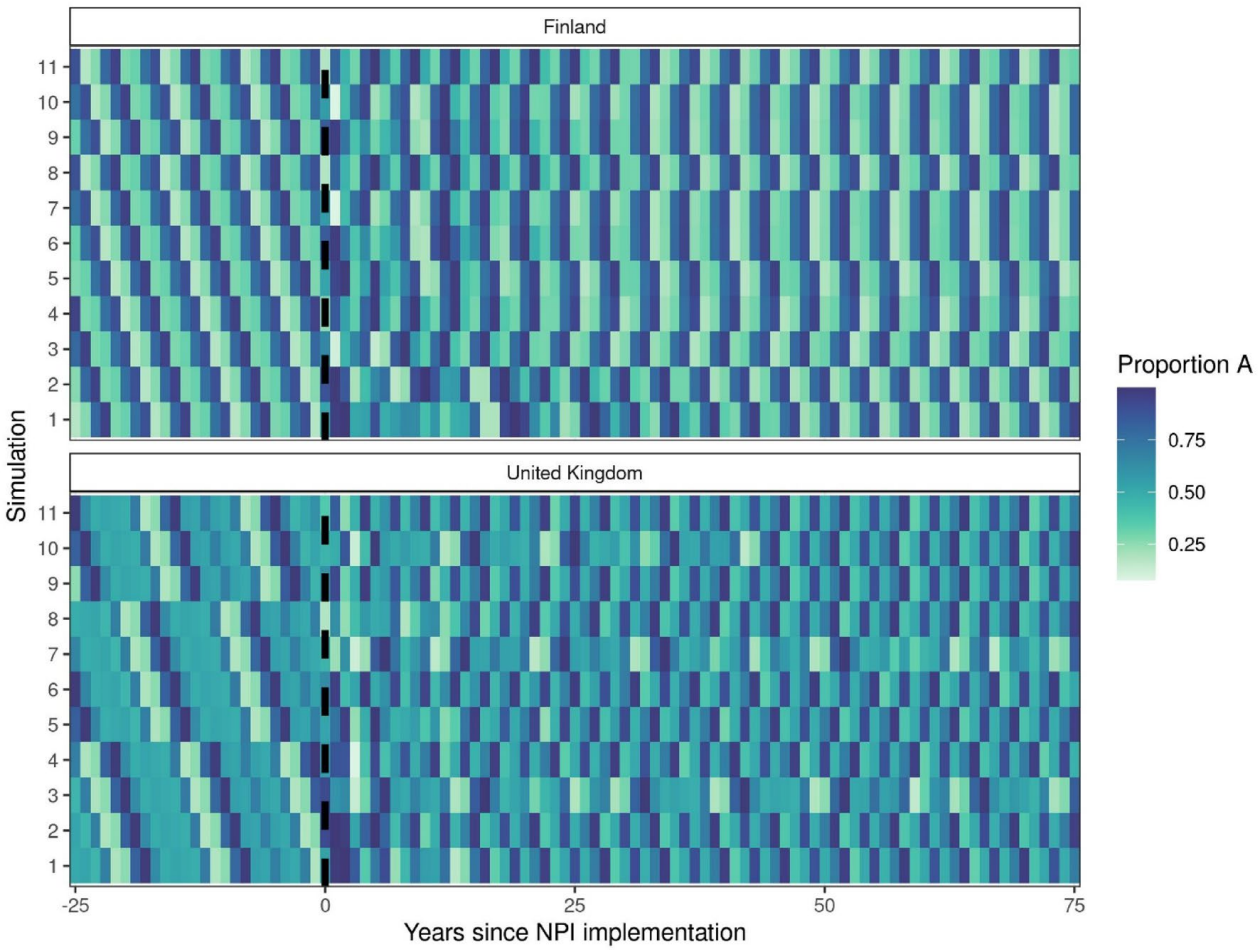
Heatmap showing the long-term mean subtype dynamics before and after NPI onset in Finland-like and UK-like scenarios. The mean proportion of RSV cases caused by subtype A are shown for each year in each location, ranging from 25 years before NPIs were implemented to 75 years after. (**A**) In Finland-like scenarios, the dynamic of switches between subtypes A and B are fairly regular following a 4-year cycle, and do not show any long-term change following the lift of NPIs. (**B**) In UK-like scenarios, the pattern of switching between A and B subtypes changes after NPIs are lifted in most simulations, showing more frequent changes between subtypes, and lasting for the full 75 years after NPIs are lifted.

Overall, we predicted that the pandemic interruption caused subtype dynamics to switch out of pre-pandemic periodicity, resulting in a stronger than normal pattern of subtype A dominance that lasted for several years in both Finland-like and UK-like scenarios following NPI implementation. In the Finland-like scenario, dynamics eventually returned to the pre-pandemic state (Fig. 1). However, in the UK-like scenarios, we predicted that dominance fluctuates between subtypes A and B more frequently in the post-pandemic period, and that this change in periodicity may persist for several decades (Fig. 2).

**Figure 3:**
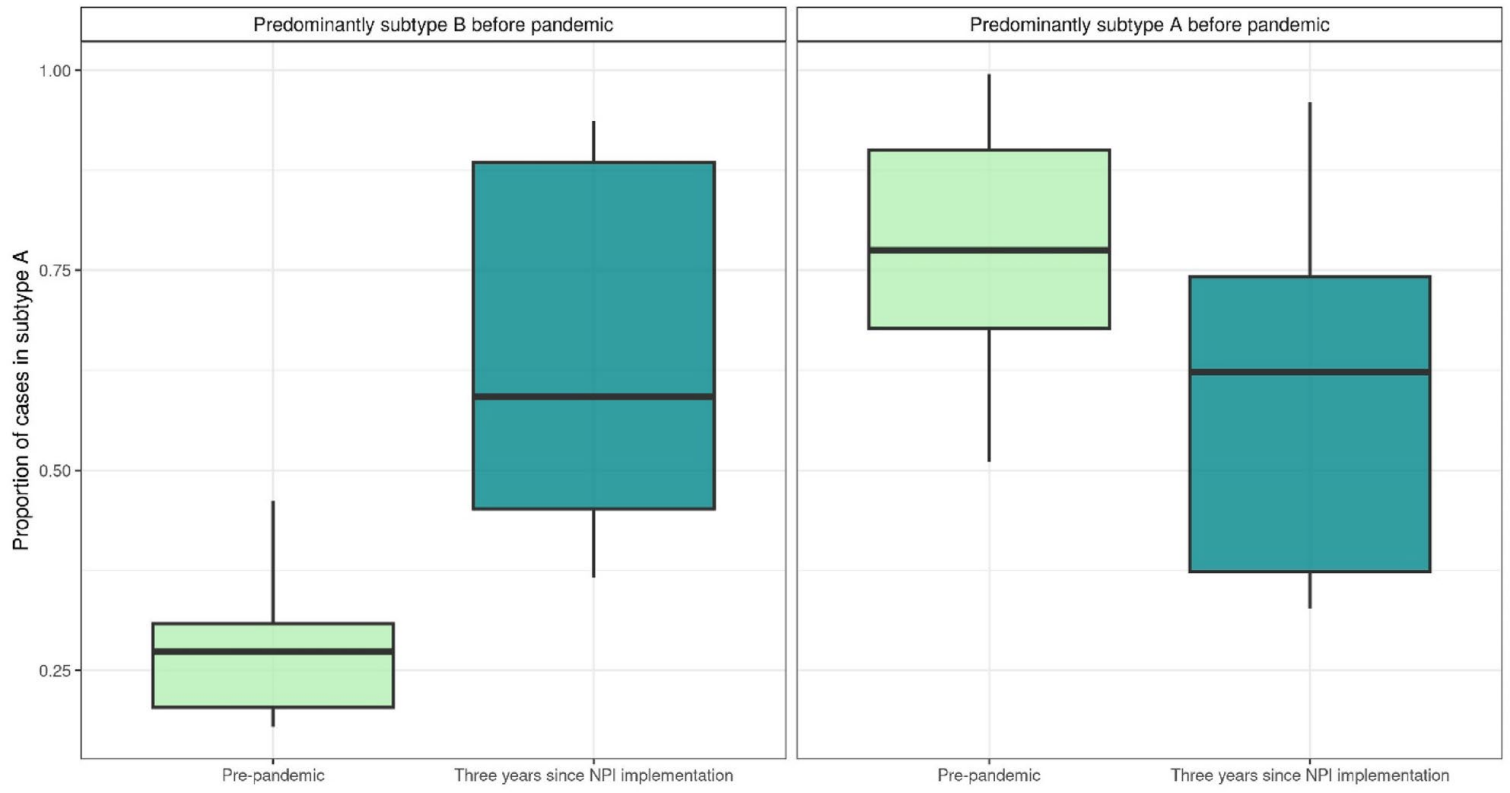
Box plots show, across simulations, the distribution of proportion of cases in subtype A in the year before (green) and 3 years after (blue) NPIs are put in place. Simulations are stratified based on which subtype dominated pre-NPIs. On the left are the average proportion of cases caused by subtype A in simulations in which cases were caused predominantly by subtype B before the pandemic. On the right, simulations in which cases were caused primarily by subtype A. Regardless of which subtype caused the majority of cases in the year before NPIs were put in place, subtype A causes the majority of infections on average over the 3 years after.

To determine whether the predominant post-NPI subtype was primarily dependent on the subtype that circulated just before NPIs started, we stratified simulations based on which subtype dominated in the year leading up to NPI start. The proportion of cases in subtype A over the three years after pandemic NPIs were imposed was relatively consistent regardless of which subtype dominated before (Fig. 3). When subtype B was more prevalent in the year preceding the implementation of NPIs, most post-pandemic years were subtype A, as expected. However, in simulations that were dominated by subtype A in one year pre-pandemic, there was not as pronounced a shift towards subtype B in the years following, and on average, most cases remained subtype A. This is likely due to the relative transmission advantage of subtype A over subtype B, combined with waning immunity during the first period of NPIs, when RSV transmission was either completely or mostly interrupted.

### Comparison between model projections and observed subtype dynamics

To evaluate the model findings, we used genomic data collected through the Global Initiative on Sharing Avian Influenza Data (GISAID) for twenty locations across the globe. We assessed the impact of the COVID-19 NPIs on subtype dynamics by classifying each location by whether it was A or B dominant one season before and three seasons after the implementation of NPIs, where a dominant season is defined as ≥ 50% of sequences belonging to a given subtype (Table 1). We found evidence of RSV-A dominance both before and after the pandemic, whereby 85% and 80% of locations were RSV-A dominant in the pre- and post-pandemic period, respectively. This aligns with the direction of our model predictions in which we estimated that RSV-A would be dominant in 64% and 59% of the pre- and post-pandemic seasons.

**Table 1:** Frequency of locations that were subtype A or B dominant in the pre- and post-pandemic period.

	Post-A dominance—N (%)	Post-B dominance—N (%)
Pre-A dominance—N (%)	14 (70)	3 (15)
Pre-B dominance—N (%)	2 (10)	1 (5)

Pre-pandemic is defined as one season before the pandemic and post-pandemic refers to three post-pandemic seasons. Subtype dominance is defined as ≥ 50% of sequences belonging to a given subtype.

**Figure 4:**
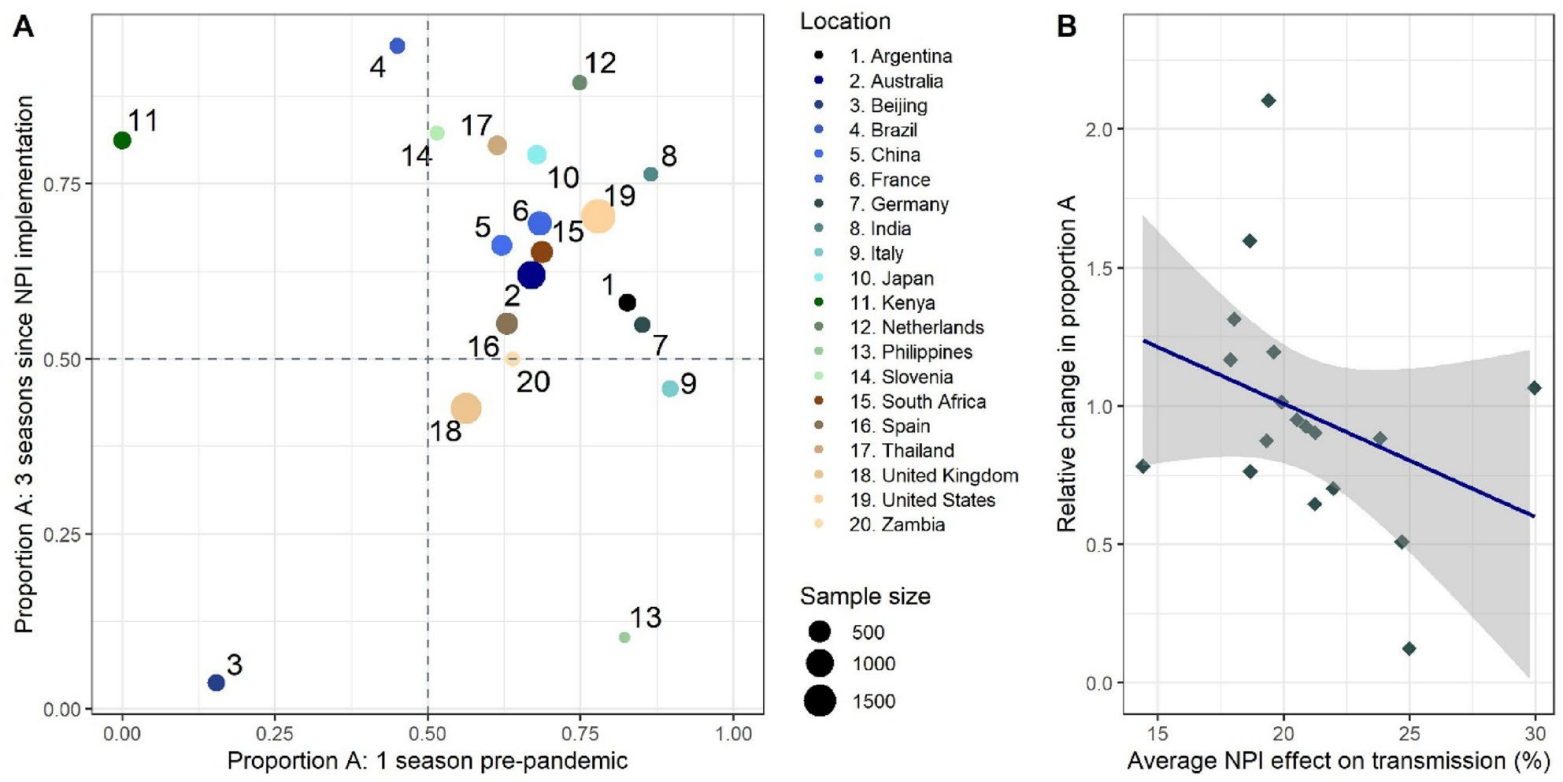
(**A**) The proportion of RSV-A reported in one pre-pandemic season against three seasons post-pandemic (3/2020–3/2023) for 20 locations. Here, we only show locations in which there are at > 30 subtyped cases in both the pre- and post-pandemic period. The dashed lines show 50% proportion A. The size of the point represents the number of post-pandemic RSV samples, where the locations that had less than 500 samples are shown by the smallest points. A more detailed figure showing the sample sizes and distribution of RSV-A by location is provided in the supplementary materials (Figure S4). (**B**) The average NPI strength by location against the ratio of proportion A in the post-pandemic compared to the pre-pandemic. Average NPI strength is calculated by taking the average adjusted stringency index (ranging from 0 to 40%) across the period March 2020–December 2022, where the stringency should be interpreted as the average impact on transmission. The plot shows the linear fit to the data points with a loess spline, where each point represents one of the 19 countries included in the analysis.

Three locations showed subtype B dominance before the introduction of NPIs, and of these only Beijing remained subtype B dominant in the post-pandemic period, while Kenya and Brazil observed pronounced switches to subtype A dominance (Fig. 4A). Conversely, 14 of the 17 locations which were subtype A dominant before the pandemic remained subtype A dominant during the post-pandemic period. We also assessed the relationship between average NPI strength and change in proportion A in the pre- and post-pandemic period. We found that as the average strength of NPIs increased, as measured by the Oxford Stringency Index, the ratio of the proportion A in the post-pandemic period to the proportion A in the pre-pandemic period generally declined, suggesting that NPI strength may modulate the extent of subtype A dominance in the initial RSV rebound (Fig. 4B).

## Discussion

Results based on a two-strain RSV model predicted subtype A dominance in the post-pandemic period, regardless of which subtype was circulating before the implementation of NPIs and across two different locations. We acknowledge that the global connectivity of viral transmission patterns could result in an intermediate situation existing somewhere in between the modeled scenarios. Regardless, modeling across different settings allowed us to consider how different baseline transmission dynamics could theoretically be impacted by NPIs^19^. Notably, in the UK-like scenario, we observed a shift in the pattern of change between the two subtypes that lasts for decades following the lift of NPIs.

Although surveillance data on RSV subtypes are sparse, our model findings were generally supported by the empirical genetic data available through GISAID. The 20 locations included in this dataset are geographically diverse and instituted varying levels of NPIs, yet showed a similar tendency for RSV-A to dominate the post-pandemic period. In individual simulations of the model, we see some of the transmission patterns flipping to extreme RSV-A proportion after NPIs are implemented, potentially aligning with empirical patterns (Figure S3). However, more viral genetic data is needed to understand the extent to which these predictions may be playing out in the real world.

Although the empirical genetic data generally agreed with our projections, Beijing was a notable exception, showing low RSV-A proportion before and after the pandemic period. China, and Beijing in particular, instituted some of the world’s most stringent NPIs, severely curtailing local pathogen circulation^20^. Because RSV circulation was primarily subtype B before the pandemic, strict NPIs may have influenced a reduction in viral introductions of RSV relative to other locations. In individual model simulations (Figure S3), we observed several simulations in which subtype B dominates both in the pre- and post- pandemic. Notably, these simulations only occurred in the UK, which had higher NPI stringency than Finland, potentially supporting the hypothesis that stricter NPIs may enable subtype B dominance. This is further supported by the empirical trend for switching to subtype A dominance to be more pronounced among countries with less stringent NPIs. Additionally, recently published surveillance data from Beijing shows a strong subtype switch to A in the second post-pandemic year following the initial rebound of RSV-B, consistent with a delay in RSV reintroductions and aligning with the subtype switching pattern predicted by our model^21^.

There are subtype-specific features of RSV-A that may drive higher estimated R_0_ in modeling analyses and explain the observed dominance during the post-pandemic period^14^. RSV-A is associated with more rapid viral replication upon infection, which could explain why RSV-A may cause more lower-tract respiratory infections whereas RSV-B comprises a higher portion of less severe, upper-tract respiratory infections^22^. Abnormal pathogen circulation and increased susceptibility throughout the pandemic period may have altered the likelihood of experiencing severe respiratory infections as compared to mild, causing populations to be more vulnerable to RSV-A epidemics.

New vaccines recently approved for 60 + year olds and pregnant women have demonstrated similar efficacy against RSV-A and RSV-B^15,16,18^. The United States Food and Drug Administration also recently approved a monoclonal antibody immunization for infants to receive ahead of RSV seasons, but clinical trials have not reported whether efficacy is subtype dependent^17^. With these new immunizations becoming widely available, understanding the dynamics between RSV-A and RSV-B is a timely public health issue^23^. If COVID-19 interventions have permanently altered baseline RSV dynamics, this may impact the perceived effectiveness of new interventions. For example, if RSV-A does indeed cause more severe infections, and UK-like scenarios switch more frequently to higher proportion A seasons in the post-pandemic period, interventions could be perceived as less effective compared to other settings^23^. Thus, it will be important to understand the relative contribution of RSV A and B to overall disease burden in the post-pandemic period.

### Limitations

These theoretical expectations are based on previous estimates of core RSV epidemiological features, such as subtype-specific reproduction numbers, homotypic and heterotypic immunity, and cross-reactivity^14^. Unfortunately, current RSV data resolved at the subtype level remains scarce, and when it is collected, it is rarely made publicly available. Interrogating these epidemiological parameters via more detailed modeling analyses will be an important extension of this work if more granular genomic time series data becomes publicly available.

The data for NPIs are approximate and based on an assumption that the reduction in transmission is proportionate to the NPI policies that were put in place. This relationship is modified by human behavior, and we do not account for changes in NPI adherence over time. In addition to dynamic human behavior, the model for NPI effect that we have used may not track linearly onto the effect of NPIs on RSV transmission. However, this is a simplifying assumption that we have made to approximate the impact of many different NPIs with varying effects that were also lifted and reinstated over the course of the COVID-19 pandemic.

We conducted a sensitivity analysis in which we varied the maximum impact of NPIs between 30 and 50% and found that the model results were robust to a range of perturbation. At a maximum NPI reduction of 30%, the model predicted a typical RSV season in 2020 in both the UK and Finland (Figure S1). At a maximum NPI reduction of 50%, the model predicted that RSV did not rebound until late 2021 in both locations (Figure S2). Empirical data from Finland and the UK showed that RSV rebounded in late 2020 and early 2021 respectively, suggesting that our sensitivity analysis bracketed the plausible range of scenarios.

Our analysis underlines the potential of COVID-19 NPIs, and other perturbations, to explore subtype-specific intricacies in seasonal pathogen dynamics. Further improving pathogen surveillance and sequencing would greatly improve the power of such studies, reinforcing calls for systematic serosurveillance^24,25^.

## Methods

### Model structure

**Figure 5:**
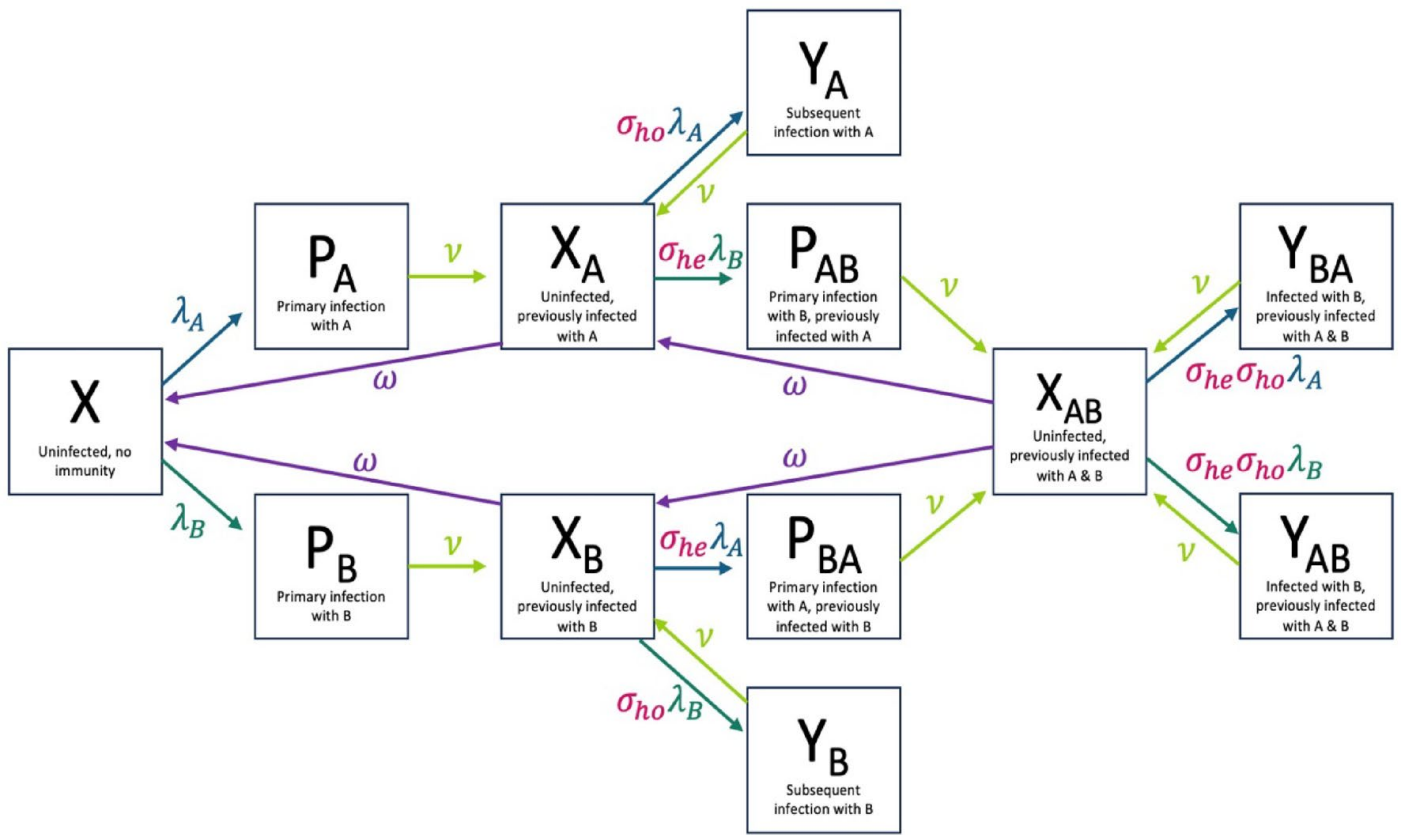
Schematic representation of the deterministic two-subtype model^14^. The boxes correspond to the possible infection states, ranging from uninfected to infected with immunity to both subtypes. The arrows represent the rate at which individuals move between the infection state compartments. The light green arrows represent recovery from infection, the purple lines represent immunity loss after infection, and the dark green lines show the rate of infection. The pink text indicates immunity status after infection.

We adapted a two-subtype RSV model developed by White et al.^14^. The model is a deterministic ordinary differential equation (ODE) with two subtypes and incomplete immunity following infection. It is governed by a set of deterministic equations (Supplementary Methods), and a schematic representation of the deterministic model is shown in Fig. 5. There are eight different infection compartments in the model, and four uninfected compartments, defined in Table S1. Infections are considered “primary” if there is no immunity to the infecting subtype (i.e. homotypic immunity), even if there is immunity through infection from the other subtype (i.e. heterotypic immunity). Infections are considered “subsequent” if there is homotypic immunity to the infecting subtype. Thus, previously infected individuals can experience a reinfection with either subtype. The number of infections an individual can experience is not limited, so subsequent infections with either subtype can happen repeatedly. In the absence of reinfection, immunity also wanes at rate $\omega .$ For those with immunity to both subtypes, it takes two full “waning periods” ($1/\omega$) to lose all immunity, where half of individuals lose immunity to subtype A first and half lose immunity to subtype B, regardless of infection history. We conducted an additional sensitivity analysis in which we assume that all individuals lose immunity at rate 2 $\omega$ and our model results were not qualitatively changed (Figure S5). All infections are the same duration, regardless of immunity, and individuals recover uniformly at rate $\nu$*.*

Immunity from infection is incorporated into the model in several different ways: homotypic immunity (σ_ho_, reduced susceptibility to homologous infections), heterotypic immunity (σ_he,_ reduced susceptibility to heterologous infections), and reduced infectiousness from subsequent (i.e. non-primary) infections. Individuals previously infected with both subtypes experience reduced susceptibility by a proportion of σ_ho_σ_he._ The rate that susceptible individuals become infected with RSV from infected compartments (labeled P and Y) on uninfected compartments (labeled X) is modified by these different types of immunity. It is also modified by a seasonal transmission parameter, which varies between the two locations modeled. This transmission coefficient $\beta$ follows a seasonal pattern and is given by Eq. (1):

1 $$\beta ={\beta }_{0} *a(cos(2\pi (t - \phi )) +1)$$

where α represents the amplitude of the RSV seasonal outbreak, *t* is time in years, and $\phi$ represents the peak timing of seasonal forcing, fit in a prior study to be between January 1st-December 31st. The timing and magnitude of seasonal outbreaks was allowed to vary between locations.

To estimate the impact of NPIs on RSV subtype dynamics, we incorporated a reduction in the transmission coefficient, β. The reduction in transmission represents the stringency of NPIs in place during the pandemic, which varied over time and between countries. To measure this reduction, we used an NPI *Stringency Index* generated by Mathieu et al.^26^, which provided a daily numerical value representing the strength of NPIs based on nine policy measures governments used to prevent COVID-19 transmission. The final values range from 0 to 100 for each day and are generated at the country level. In our model, we set the maximum possible effect of NPIs on transmission probability to 40%^25^, so the Stringency Index time series was transformed from the original 0–100 scale to a 0.0–0.4 scale. The maximum impact of NPIs is allowed to vary by country, where countries with less stringent NPI measures may not reach a stringency level of 100. To incorporate this NPI effect, $\rho$, in the compartmental model, the transmission coefficient at each time point is multiplied by $1-\rho$, shown by Eq. (2).

2 $$\beta ={\beta }_{0}(1- \rho ) *a(cos(2\pi (t - \phi )) +1)$$

The NPI effect for all dates before the pandemic and after December 31st, 2022, when the *NPI Stringency Index* stopped tracking government COVID-19 policies, are set to zero. Because the original model was fit to Finland and the UK separately, we ran the model for the two locations individually and use NPI stringency data specific to Finland and the UK, respectively. After scaling to the 40% maximum, the mean effect of NPIs over the first year was 29.1% in the UK and 19.6% in Finland. We additionally conducted a sensitivity analysis in which the maximum possible effect of NPIs is set to 30% and 50% (Figures S1–S2).

For the remaining parameters, we used the estimates that White et al. generated by fitting this model to historical time series data from Finland (1980–2000) and England & Wales (1989–2001) in their original analysis (Table S2). When this model was originally fit, the average duration of infection and average duration of immunity were considered global parameters, and thus were fixed between the two locations. The effect of homologous and heterologous immunity as well as the reduced infectiousness from subsequent infections were also fixed across two locations. The original study was notable for identifying a higher R_0_ for RSV A which in turn explained the predominance of this subtype in the studied locations.

### Model simulations

We ran the model for 100 years to reach a steady state before implementing NPIs. The original model identified a 10–12-year periodicity in the dynamics between the two strains. To account for the slow periodicity that ranges between 10 and 12 years in the pre-pandemic model^14^, we ran 11 model simulations in each location, in which NPIs are initialized every April over a 11-year period, aligning with the approximate onset of COVID-19 interventions in 2020. This allowed us to explore the disruption of subtype dynamics over a range of time points within the typical cycling pattern. We presented results as the mean of these simulations by day (in relation to NPI onset) and location, along with 95% confidence intervals at each time point. We ran the models to explore dynamics for a range of time from 5 to 75 years after NPIs were imposed.

### Epidemiological data

Next, to assess the validity of our model predictions, we sourced empirical data on subtype-specific RSV case data in the post-pandemic period. Subtype-specific RSV surveillance is scarce as subtyping is rarely conducted in routine in clinical settings. Instead, we used RSV genetic sequence data collected through the Global Initiative on Sharing Avian Influenza Data (GISAID)^27,28^ to estimate subtype prevalence over time. GISAID represents the most complete collection of global genetic sequence data available for influenza, SARS-CoV-2, and various other respiratory viruses, with over 170 locations submitting genetic sequences to the database since its inception. On November 23rd, 2023, RSV subtype data was available through GISAID for 89 locations; however, counts for most locations were very low (Supplementary S4). For this reason, we only included locations present in the dataset that had at least 30 subtyped cases in both one pre-pandemic and three post-pandemic seasons. The locations meeting this qualification were Argentina, Australia, Beijing, Brazil, China, England, France, Germany, India, Italy, Japan, Kenya, Netherlands, Philippines, Slovenia, South Africa, Spain, Thailand, the US, and Zambia. We tabulated monthly time series of RSV A and B sequence counts in each location and calculated the proportion of A in one season pre-pandemic compared to three seasons post-pandemic. Lastly, we compared the average NPI strength, as measured by the *NPI Stringency Index*, to changes in RSV-A proportion in the pre- and post-pandemic periods for each location.

### Supplementary Information

Supplementary Information 1.

Supplementary Information 2.

## Data Availability

The respiratory syncytial virus datasets used in this analysis are publicly available through the Global Initiative on Sharing Avian Influenza Data. The code for the analysis is publicly available at https://github.com/sbents/RSV_Subtypes.
