# Supplementary material for: Differential impact of COVID-19 non-pharmaceutical interventions on the epidemiological dynamics of respiratory syncytial virus subtypes A and B
Source: Sci Rep. 2024 Jun 24;14:14527. doi: 10.1038/s41598-024-64624-1 (PMC11196647; doi:10.1038/s41598-024-64624-1)
Supplement: Supplementary file 1 — Supplementary Information 1. [file 41598_2024_64624_MOESM1_ESM.pdf]

We gratefully acknowledge the following Authors from the Originating laboratories responsible for obtaining the specimens, as well as the Submitting laboratories where the genome data were generated and shared via GISAID, on which this research is based.

All Submitters of data may be contacted directly via www.gisaid.org

Authors are sorted alphabetically.

Accession ID	Originating Laboratory	Submitting Laboratory	Authors
EPI_ISL_15004433, EPI_ISL_15004435	National Institute of Hygiene, virology departement, National Influenza Center	Centers for Disease Control and Prevention, Coronavirus and Other Respiratory Viruses Division	Megha Aggarwal, Lijuan Wang, Ji In Park, Amanda Smith and Everardo Vega
EPI_ISL_15750102, EPI_ISL_15750103, EPI_ISL_15750110, EPI_ISL_15750111, EPI_ISL_15750112, EPI_ISL_15750113, EPI_ISL_15750114, EPI_ISL_15750116, EPI_ISL_15750117, EPI_ISL_15750118, EPI_ISL_15750125			
see above	Virology, Research Institute for Tropical Medicine	Virology, Research Institute for Tropical Medicine	Calaor-Morin,J., Arguelles,V.L., Foronda,J.L., Tan,A., Lagamayo,E., Dapat,C., Lupisan,S., Morin,J.C., Foronda,J.L.M.
EPI_ISL_15751208, EPI_ISL_15751209, EPI_ISL_15751210, EPI_ISL_15751211, EPI_ISL_15751212, EPI_ISL_15751213, EPI_ISL_15751214, EPI_ISL_15751215	Molecular Virology, Johns Hopkins School of Medicine	Molecular Virology, Johns Hopkins School of Medicine	Fall A, Elawar F, Hodcroft EB, Jallow MM, Toure CT, Barry MA, Kiori DE, Sy S, Diaw Y, Goudiaby D, Niang MN, Dia N, Fall,A., Jallow,M.M., Toure,C.T., Kiori,D.E., sy,S., Diaw,Y., Goudiaby,D., Niang,M.N., Dia,N.
EPI_ISL_15751228, EPI_ISL_15751229, EPI_ISL_15751230, EPI_ISL_15751231, EPI_ISL_15751234, EPI_ISL_15751235, EPI_ISL_15751242, EPI_ISL_15751243	Influenza Group, National Institute of Virology	Influenza Group, National Institute of Virology	Choudhary,M.L., Chaudhary,U., Choudhary,M.L., Chaudhary,U.
EPI_ISL_15751253, EPI_ISL_15751254, EPI_ISL_15751255, EPI_ISL_15751256, EPI_ISL_15751257, EPI_ISL_15751258, EPI_ISL_15751259, EPI_ISL_15751260, EPI_ISL_15751261, EPI_ISL_15751262, EPI_ISL_15751263, EPI_ISL_15751264, EPI_ISL_15751265, EPI_ISL_15751266, EPI_ISL_15751267, EPI_ISL_15751268, EPI_ISL_15751269, EPI_ISL_15751270, EPI_ISL_15751271, EPI_ISL_15751272, EPI_ISL_15751273, EPI_ISL_15751274, EPI_ISL_15751275, EPI_ISL_15751276, EPI_ISL_15751277, EPI_ISL_15751278, EPI_ISL_15751279, EPI_ISL_15751280, EPI_ISL_15751281, EPI_ISL_15751282, EPI_ISL_15751284, EPI_ISL_15751286, EPI_ISL_15751287, EPI_ISL_15751288, EPI_ISL_15751289, EPI_ISL_15751290, EPI_ISL_15751294, EPI_ISL_15751295, EPI_ISL_15751296, EPI_ISL_15751297, EPI_ISL_15751298, EPI_ISL_15751299, EPI_ISL_15751300, EPI_ISL_15751301, EPI_ISL_15751303, EPI_ISL_15751304, EPI_ISL_15751311, EPI_ISL_15751315, EPI_ISL_15751323, EPI_ISL_15751324, EPI_ISL_15751325, EPI_ISL_15751326, EPI_ISL_15751327, EPI_ISL_15751328, EPI_ISL_15751329, EPI_ISL_15751330, EPI_ISL_15751332, EPI_ISL_15751333, EPI_ISL_15751334, EPI_ISL_15751336, EPI_ISL_15751342, EPI_ISL_15751343, EPI_ISL_15751344			
see above	Center for Virus Research, KEMRI	Center for Virus Research, KEMRI	Wangui,J.M., Nokes,J.D., Opot,B.H., Opanda,S.M., Agoti,C.N., Otieno,J.R., Bulimo,W.D., Wangui,J.M., Nokes,J.D., Opot,B.H., Opanda,S.M., Agoti,C.N., Otieno,J.R., Bulimo,W.D.
EPI_ISL_15752007, EPI_ISL_15752008, EPI_ISL_15752009, EPI_ISL_15752018, EPI_ISL_15752019, EPI_ISL_15752020, EPI_ISL_15752021, EPI_ISL_15752022, EPI_ISL_15752023, EPI_ISL_15752024, EPI_ISL_15752025, EPI_ISL_15752026, EPI_ISL_15752027, EPI_ISL_15752028, EPI_ISL_15752029, EPI_ISL_15752030, EPI_ISL_15752031, EPI_ISL_15752032, EPI_ISL_15752033, EPI_ISL_15752034, EPI_ISL_15752035			
see above	Respiratory Viruses Branch, Division of Viral Diseases	Respiratory Viruses Branch, Division of Viral Diseases	Wang,L., Ng,T.F.F., Castro,C.J., Marine,R.L., Magana,L.C., Esona,M., Peret,T.C.T., Thornburg,N.J., Wang,L., Ng,T.F.F., Castro,C.J., Marine,R.L., Magana,L.C., Esona,M., Peret,T.C.T., Thornburg,N.J.
EPI_ISL_15752098, EPI_ISL_15752099, EPI_ISL_15752100, EPI_ISL_15752101, EPI_ISL_15752102, EPI_ISL_15752103, EPI_ISL_15752104, EPI_ISL_15752105, EPI_ISL_15752106, EPI_ISL_15752107, EPI_ISL_15752108, EPI_ISL_15752109, EPI_ISL_15752110, EPI_ISL_15752111, EPI_ISL_15752112, EPI_ISL_15752113, EPI_ISL_15752114, EPI_ISL_15752116, EPI_ISL_15752117, EPI_ISL_15752118, EPI_ISL_15752119, EPI_ISL_15752121, EPI_ISL_15752122, EPI_ISL_15752123, EPI_ISL_15752124, EPI_ISL_15752125, EPI_ISL_15752126, EPI_ISL_15752127, EPI_ISL_15752128, EPI_ISL_15752129, EPI_ISL_15752130, EPI_ISL_15752131, EPI_ISL_15752133, EPI_ISL_15752134, EPI_ISL_15752135			
see above	Virology, ICDDR	Virology, ICDDR	Rahman,M.Z., Hossain,M.E., Sumiya,M.K., Islam,M.M., Begum,M.N., Hoque,A.F., Uddin,K.M.M., Hassan,M.Z., Rahman,M., Goswami,D.R., Brooks,W.A., Rahman,M.Z., Hossain,M.E., Sumiya,M.K., Islam,M.M., Begum,M.N., Hoque,A.F., Uddin,K.M.M., Hassan,M.Z., Rahman,M., Goswami,D.R., Brooks,W.A.
EPI_ISL_17673340, EPI_ISL_17673341, EPI_ISL_17673342, EPI_ISL_17673343, EPI_ISL_17673344, EPI_ISL_17673345, EPI_ISL_17673346, EPI_ISL_17673347, EPI_ISL_17673348, EPI_ISL_17673349, EPI_ISL_17673350, EPI_ISL_17673351, EPI_ISL_17673352, EPI_ISL_17673353, EPI_ISL_17782659			
see above	University of Washington, Virology	University of Washington, Virology	Sereewit,J., Hajian,P., Xie,H. and Greninger,A.L.
EPI_ISL_17995625, EPI_ISL_17995626, EPI_ISL_17995627, EPI_ISL_17995628, EPI_ISL_17995629, EPI_ISL_17995630, EPI_ISL_17995631, EPI_ISL_17995632, EPI_ISL_17995633, EPI_ISL_17995666, EPI_ISL_17995667			
see above	Robert Koch-Institute Nationales Referenzzentrum für Influenza	Robert Koch-Institute Nationales Referenzzentrum für Influenza	Sophie Kondgen, Janine Reiche
EPI_ISL_18334166, EPI_ISL_18334167, EPI_ISL_18334178, EPI_ISL_18334180, EPI_ISL_18334204, EPI_ISL_18334215, EPI_ISL_18334220, EPI_ISL_18334231, EPI_ISL_18334232, EPI_ISL_18334236	Baylor College of Medicine, Division of Pediatric Tropical Medicine	Baylor College of Medicine, Division of Pediatric Tropical Medicine	Avadhanula,V., Agustinho,D.P., Menon,V.K., Chemaly,R.F., Shah,D.P., Qin,X., Surathu,A., Doddapaneni,H., Muzny,D.M., Metcalf,G.A., Gregeen,S.J., Gibbs,R.A., Petrosino,J.J., Sedlazeck,F.J. and Piedra,P.A.
EPI_ISL_18401453	Niigata University (IDRC) Infectious Diseases Research Center	Niigata University (IDRC) Infectious Diseases Research Center	Wint Wint Phyu, Reiko Saito
EPI_ISL_2543922	KEMRI Wellcome Trust Research Programme	KEMRI Wellcome Trust Research Programme	Agoti,C.N., Otieno,J.R., Munywoki,P.K., Mwihuri,A.G., Cane,P.A., Nokes,D.J., Kellam,P. and Cotten,M.L.
EPI_ISL_2543930	Laboratory of Virology, Capital Institute of Pediatrics	Laboratory of Virology, Capital Institute of Pediatrics	Cui,G., Deng,J., Zhu,R., Qian,Y., Sun,Y., Zhao,L. and Wang,F.
EPI_ISL_2543932	Microbiology Department, The University of Hong Kong, Queen Mary Hospital, University Pathology Building	Microbiology Department, The University of Hong Kong, Queen Mary Hospital, University Pathology Building	Zhang,K., He,J., Cheng,Z., Zhou,J., Bose,M., Henrickson,K.J. and Zheng,B.
EPI_ISL_2543933	Influenza Group, National Institute of Virology	Influenza Group, National Institute of Virology	Choudhary,M.L., Wadhwa,B., Jadhav,S.M., Chadha,M.S. and Mourya,D.T.
EPI_ISL_2543935, EPI_ISL_2543936	J. Craig Venter Institute	J. Craig Venter Institute	Shabman,R., Das,S.R., Puri,V., Fedorova,N., Amedeo,P., Williams,M., Shrivastava,S. and Halasa,N.
EPI_ISL_2543937	Virology, Graduate School of Medicine, Tohoku University	Virology, Graduate School of Medicine, Tohoku University	Malasao,R., Furuse,Y., Okamoto,M., Dapat,C., Saito,M., Saito-Obata,M., Tamaki,R., Segubre-Mercado,E., Lupisan,S. and Oshitani,H.
EPI_ISL_2543941	Division of Biosafety Evaluation and Control, Korea National Institute of Health, Korea Centers for Disease Control and Prevention	Division of Biosafety Evaluation and Control, Korea National Institute of Health, Korea Centers for Disease Control and Prevention	Yun,M.-R., Lee,W.-J., Kim,A.-R., Lee,H.S., Kim,K., Kim,S.S., Kim,Y.-J. and Kim,D.-W.
EPI_ISL_2543943	Emerging Viral Infections, Oxford University Clinical Research Unit	Emerging Viral Infections, Oxford University Clinical Research Unit	Do,L.A.H., Wilm,A., van Doorn,H.R., Lam,H.M., Sukumaran,R., Tran,A.T., Nguyen,B.H., Tran,T.T.L., Tran,Q.H., Vo,Q.B., Tran Dac,N.A., Trinh,H.N., Nguyen,t.T.H., Le Binh,B.T., Le,K., Nguyen,M.T., Thai,Q.T., Vo,T.V., Ngo,N.Q.M., Dang,t.K.H., Cao,N.H., Tran,T.V., Ho,L.V., Farrar,J., de Jong,M.D., Chen,S., Nagaranjan,N., Bryant,J.E. and Hibberd,M.L.
EPI_ISL_2543952, EPI_ISL_2543953, EPI_ISL_2543954	J. Craig Venter Institute	J. Craig Venter Institute	Lorenzi,H., Town,C., Halpin,R., Bera,J., Ransier,A., Fedorova,N., Stockwell,T., Amedeo,P., Appalla,L., Bishop,B., Edworthy,P., Gupta,N., Hoover,J., Katzel,D., Li,K., Schobel,S., Shrivastava,S., Thovarai,V., Wang,S., Rebuffo-Scheer,C., Fan,J., He,J., Kehl,S.C., Lederboer,N., Jurgens,L.A., Bose,M.E., Beck,E.T., Kumar,S., Gerna,G., Wentworth,D.E. and Henrickson,K.J.
EPI_ISL_2543966	Molecular Research, Ontario Agency for Health Protection and Promotion	Molecular Research, Ontario Agency for Health Protection and Promotion	Eshaghi,A., Lai,R., Nadarajah,J.T., Li,A., Patel,S.N., Gubbay,J.B. and Low,D.E.
EPI_ISL_2543968	Investigacion en Virologia y Biotecnologia, Instituto Conmemorativo Gorgas de Estudios de la Salud	Investigacion en Virologia y Biotecnologia, Instituto Conmemorativo Gorgas de Estudios de la Salud	Abrego,L., Delfraro,A., Franco,D., Castillo,C., Cano,M., Castillo,M., Castillo,J., Pascale,J. and Arbiza,J.
EPI_ISL_2543973, EPI_ISL_2543979, EPI_ISL_2543980, EPI_ISL_2543981, EPI_ISL_2543982	J. Craig Venter Institute	J. Craig Venter Institute	Das,S.R., Halpin,R.A., Shilts,M., Puri,V., Akopov,A., Fedorova,N., Stockwell,T., Amedeo,P., Bishop,B., Katzel,D., Schobel,S., Shrivastava,S. and Hartert,T.
EPI_ISL_2544030	Pediatrics - Infectious Diseases, Medical College of Wisconsin	Pediatrics - Infectious Diseases, Medical College of Wisconsin	Rebuffo-Scheer,C., Bose,M.E., He,J., Khaja,S., Ulatowski,M., Beck,E.T., Fan,J., Kumar,S., Nelson,M.I. and Henrickson,K.J.
EPI_ISL_2544035, EPI_ISL_2544037, EPI_ISL_2544040	J. Craig Venter Institute	J. Craig Venter Institute	Shabman,R., Das,S.R., Shilts,M., Fedorova,N., Puri,V., Shrivastava,S., Amedeo,P., Williams,M., Barratt,K., Mitchell,J. and Jennings,L.
EPI_ISL_2544047, EPI_ISL_2544048	Virology Laboratory, Dr. Ricardo Gutierrez Children Hospital	Virology Laboratory, Dr. Ricardo Gutierrez Children Hospital	Viegas,M., Goya,S. and Mistchenko,A.S.
EPI_ISL_2544055, EPI_ISL_2544056, EPI_ISL_2544057, EPI_ISL_2544058, EPI_ISL_2544060, EPI_ISL_2544062, EPI_ISL_2544063, EPI_ISL_2544064	J. Craig Venter Institute	J. Craig Venter Institute	Wentworth,D.E., Halpin,R.A., Bera,J., Lin,X., Fedorova,N., Tsitrin,T., McLellan,M., Stockwell,T., Amedeo,P., Bishop,B., Gupta,N., Hoover,J., Katzel,D., Schobel,S., Shrivastava,S., Garcia,J., Laguna-Torres,V.A., Leguia,M., Benavides,J.G. and Halsey,E.
EPI_ISL_2570814, EPI_ISL_2570815, EPI_ISL_2570816, EPI_ISL_2570817	Investigacion en Virologia y Biotecnologia, Instituto Conmemorativo Gorgas de Estudios de la Salud	Investigacion en Virologia y Biotecnologia, Instituto Conmemorativo Gorgas de Estudios de la Salud	Abrego,L., Delfraro,A., Franco,D., Castillo,C., Cano,M., Castillo,M., Castillo,J., Pascale,J. and Arbiza,J.
EPI_ISL_2570819	Department of Clinical Laboratory, Fujian Provincial Hospital	Department of Clinical Laboratory, Fujian Provincial Hospital	Su,Y., Wu,Y., Tian,R. and Liang,G.
EPI_ISL_2570822, EPI_ISL_2570823, EPI_ISL_2570824, EPI_ISL_2570825, EPI_ISL_2570826, EPI_ISL_2570827	WHO WPRO Measles Regional Reference Lab, Key Laboratory of Medical Virology Ministry of Health, National Institute for Viral Disease Control and Prevention, China Center for Disease Control and Prevention	WHO WPRO Measles Regional Reference Lab, Key Laboratory of Medical Virology Ministry of Health, National Institute for Viral Disease Control and Prevention, China Center for Disease Control and Prevention	Song,J., Zhang,Y. and Xu,W.
EPI_ISL_2570841	Health Science, Universidade Federal Do Parana	Health Science, Universidade Federal Do Parana	Moreira,F.B., Santos,J.S., Avanzi,V.M., Nogueira,M.B., Vidal,L.R.R. and Raboni,S.M.
EPI_ISL_2570846, EPI_ISL_2570848	Virology Section, Department of Microbiology, King George's Medical University	Virology Section, Department of Microbiology, King George's Medical University	Singh,A.K., Jain,A., Jain,B., Dangi,T., Verma,A.K., Dwivedi,M. and Kushwaha,R.
EPI_ISL_2570857	Virology Department, Hospices Civils de Lyon	Virology Department, Hospices Civils de Lyon	Gaymard,A., Pichon,M. and Morfin,F.
EPI_ISL_2570862	Centre de Recherche en Infectiologie, Universite Laval, CHUL	Centre de Recherche en Infectiologie, Universite Laval, CHUL	Papenburg,J., Carbonneau,J., Hamelin,M.-E., Isabel,S., Ohoumanne,N., Dery,P., Paes,B.A., Corbeil,J., Bergeron,M.G., De Serres,G. and Boivin,G.
EPI_ISL_2570863	Miho Kobayashi Gunma Prefectural Institute of Public Health and Environmental Sciences	Miho Kobayashi Gunma Prefectural Institute of Public Health and Environmental Sciences	Kobayashi,M. and Kimura,H.
EPI_ISL_2570871, EPI_ISL_2570872	Epidemiology and Demography, Kenya Medical Research Institute (KEMRI) - Wellcome Trust Research Programme	Epidemiology and Demography, Kenya Medical Research Institute (KEMRI) - Wellcome Trust Research Programme	Agoti,C.N., Otieno,J.R., Gitahi,C.W., Cane,P.A. and Nokes,D.J.
EPI_ISL_2570874	Department of Clinical Laboratory, Fujian Provincial Hospital	Department of Clinical Laboratory, Fujian Provincial Hospital	Su,Y., Wu,Y., Tian,R. and Liang,G.
EPI_ISL_2570876, EPI_ISL_2570877	Department of Epidemiology and Demography, Kenya Medical Research Institute (KEMRI) - Wellcome Trust Research Programme	Department of Epidemiology and Demography, Kenya Medical Research Institute (KEMRI) - Wellcome Trust Research Programme	Otieno,J.R., Agoti,C.N., Gitahi,C.W., Bett,A., Ngama,M., Medley,G.F., Cane,P.A. and Nokes,J.D.
EPI_ISL_2570916, EPI_ISL_2570917, EPI_ISL_2570918	Epidemiology and Demography, KEMRI - Wellcome Trust Research Programme, Centre for Geographic Medicine Research	Epidemiology and Demography, KEMRI - Wellcome Trust Research Programme, Centre for Geographic Medicine Research	Oketch,J.W., Kamau,E., Otieno,J.R., Mwema,A., Lewa,C., Agoti,C.N. and Nokes,J.D.
EPI_ISL_2570920	Department of Respiratory Medicine, Children's Hospital of Chongqing Medical University	Department of Respiratory Medicine, Children's Hospital of Chongqing Medical University	Ren,L. and Liu,E.
EPI_ISL_2570946	Pediatrics, Show-Chwan Memorial Hospital	Pediatrics, Show-Chwan Memorial Hospital	Lee,C.-Y.
EPI_ISL_2570968	Laboratory Medicine and Pathobiology, University of Toronto	Laboratory Medicine and Pathobiology, University of Toronto	Granados,A., Duvvuri,V., Rosenfeld,P., Eshaghi,A. and Gubbay,J.B.

EPI_ISL_2570969, EPI_ISL_2570970, EPI_ISL_2570971	Laboratory Medicine and Pathobiology, University of Toronto	Laboratory Medicine and Pathobiology, University of Toronto	Granados,A., Duvvuri,V., Rosenfeld,P., Eshaghi,A. and Gubbay,J.
EPI_ISL_2570972	Laboratory Medicine and Pathobiology, University of Toronto	Laboratory Medicine and Pathobiology, University of Toronto	Granados,A., Duvvuri,V., Rosenfeld,P., Eshaghi,A. and Gubbay,J.B.
EPI_ISL_2570973, EPI_ISL_2570974, EPI_ISL_2570975, EPI_ISL_2570976, EPI_ISL_2570977, EPI_ISL_2570978, EPI_ISL_2570979, EPI_ISL_2570980, EPI_ISL_2570981, EPI_ISL_2570982, EPI_ISL_2570983			
see above	Laboratory Medicine and Pathobiology, University of Toronto	Laboratory Medicine and Pathobiology, University of Toronto	Granados,A., Duvvuri,V., Rosenfeld,P., Eshaghi,A. and Gubbay,J.
EPI_ISL_2570984	Laboratory Medicine and Pathobiology, University of Toronto	Laboratory Medicine and Pathobiology, University of Toronto	Granados,A., Duvvuri,V., Rosenfeld,P., Eshaghi,A. and Gubbay,J.B.
EPI_ISL_2570985, EPI_ISL_2570986	Laboratory Medicine and Pathobiology, University of Toronto	Laboratory Medicine and Pathobiology, University of Toronto	Granados,A., Duvvuri,V., Rosenfeld,P., Eshaghi,A. and Gubbay,J.
EPI_ISL_2570987, EPI_ISL_2570988, EPI_ISL_2570989, EPI_ISL_2570990	Laboratory Medicine and Pathobiology, University of Toronto	Laboratory Medicine and Pathobiology, University of Toronto	Granados,A., Duvvuri,V., Rosenfeld,P., Eshaghi,A. and Gubbay,J.B.
EPI_ISL_2571097, EPI_ISL_2571128, EPI_ISL_2571130, EPI_ISL_2571143	University of Wuerzburg, Institute of Virology and Immunobiology	University of Wuerzburg, Institute of Virology and Immunobiology	Prifert,C., Hofmann,D. and Weissbrich,B.
EPI_ISL_2571194	Pediatric Clinic 1, Department of Pathophysiology and Transplantation, University of Milan and Fondazione IRCCS Ca Granda	Pediatric Clinic 1, Department of Pathophysiology and Transplantation, University of Milan and Fondazione IRCCS Ca Granda	Esposito,S., Zampiero,A., Piralla,A. and Principi,N.
EPI_ISL_2571227	Virology, Tohoku University Graduate School of Medicine	Virology, Tohoku University Graduate School of Medicine	Malasao,R., Okamoto,M., Chaimongkol,N., Imamura,T., Tohma,K., Dapat,I., Dapat,C., Suzuki,A., Saito,M., Saito,M., Tamaki,R., Segubre-Mercado,E., Igoy,M.A.U., Lupisan,S., Olveda,R. and Oshitani,H.
EPI_ISL_2571364, EPI_ISL_2571365, EPI_ISL_2571366, EPI_ISL_2571367, EPI_ISL_2571368, EPI_ISL_2571369, EPI_ISL_2571370, EPI_ISL_2571371, EPI_ISL_2571372, EPI_ISL_2571373, EPI_ISL_2571374, EPI_ISL_2571375, EPI_ISL_2571376, EPI_ISL_2571377			
see above	Department of Paediatric Infectious Diseases, Institute of Tropical Medicine, Nagasaki University	Department of Paediatric Infectious Diseases, Institute of Tropical Medicine, Nagasaki University	Yoshihara,K., Nhat Le,M., Nagasawa,K., Tsukagoshi,H., Nguyen,H.A., Toizumi,M., Moriuchi,H., Hashizume,M., Ariyoshi,K., Dang,D.A., Kimura,H. and Yoshida,L.-M.
EPI_ISL_2571390, EPI_ISL_2571391	Department of Clinical Laboratory, Fujian Provincial Hospital	Department of Clinical Laboratory, Fujian Provincial Hospital	Su,Y., Wu,Y., Tian,R. and Liang,G.
EPI_ISL_2571397, EPI_ISL_2571398	Virology, Tropical Medicine Institute Pedro Kouri	Virology, Tropical Medicine Institute Pedro Kouri	Valdes,O., Corso,M., Pinon,A., Acosta,B., Savon,C., Gonzalez,G., Mune,M., Gonzalez,G., Hernandez,B., Echevarria,Y. and Oropesa,S.
EPI_ISL_2571399	Influenza Group, National Institute of Virology	Influenza Group, National Institute of Virology	Choudhary,M.L., Anand,S.P., Wadhwa,B.S. and Chadha,M.S.
EPI_ISL_2571536	Investigacion en Virologia y Biotecnologia, Instituto Conmemorativo Gorgas de Estudios de la Salud	Investigacion en Virologia y Biotecnologia, Instituto Conmemorativo Gorgas de Estudios de la Salud	Abrego,L., Delfraro,A., Franco,D., Castillo,C., Cano,M., Castillo,M., Castillo,J., Pascale,J. and Arbiza,J.
EPI_ISL_2571543, EPI_ISL_2571544, EPI_ISL_2571545, EPI_ISL_2571546, EPI_ISL_2571547, EPI_ISL_2571548, EPI_ISL_2571565, EPI_ISL_2571566, EPI_ISL_2571567	Department of Clinical Laboratory, Fujian Provincial Hospital	Department of Clinical Laboratory, Fujian Provincial Hospital	Su,Y., Wu,Y., Tian,R. and Liang,G.
EPI_ISL_2571568	West China School of Public Health, Sichuan University	West China School of Public Health, Sichuan University	Zhang,M.
EPI_ISL_2571622	Pediatrics, Show-Chwan Memorial Hospital	Pediatrics, Show-Chwan Memorial Hospital	Lee,C.-Y.
EPI_ISL_2571634	Depto Microbiologia, Instituto de Ciencias Biomedicas, Universidade de Sao Paulo	Depto Microbiologia, Instituto de Ciencias Biomedicas, Universidade de Sao Paulo	Moura,F.E.A., Thomazelli,L.M., Campelo,F.S., Delfraro,A., Arbiza,J. and Durigon,E.L.
EPI_ISL_2571651	Microbiology Department, Virology Division, College of Medicine, Taif University	Microbiology Department, Virology Division, College of Medicine, Taif University	Al Aboud,D.M., Al Aboud,N.M., Al-Malky,M.I.R. and Abdel-Moneim,A.S.
EPI_ISL_2571659	Centre for Research And Knowledge Transfer in Biotechnology, University of Zagreb	Centre for Research And Knowledge Transfer in Biotechnology, University of Zagreb	Ivancic-Jelecki,J., Slovic,A., Ljubin-Sternak,S., Mlinaric-Galinovic,G. and Forcic,D.
EPI_ISL_2571668	Department of Clinical Laboratory, Fujian Provincial Hospital	Department of Clinical Laboratory, Fujian Provincial Hospital	Su,Y., Wu,Y., Tian,R. and Liang,G.
EPI_ISL_2571687	Depto Microbiologia, Instituto de Ciencias Biomedicas, Universidade de Sao Paulo	Depto Microbiologia, Instituto de Ciencias Biomedicas, Universidade de Sao Paulo	Moura,F.E.A., Thomazelli,L.M., Campelo,F.S., Delfraro,A., Arbiza,J. and Durigon,E.L.
EPI_ISL_2571708	Laboratory Medicine and Pathobiology, University of Toronto	Laboratory Medicine and Pathobiology, University of Toronto	Granados,A., Duvvuri,V., Rosenfeld,P., Eshaghi,A. and Gubbay,J.B.
EPI_ISL_2571769	University of Wuerzburg, Institute of Virology and Immunobiology	University of Wuerzburg, Institute of Virology and Immunobiology	Prifert,C., Hofmann,D. and Weissbrich,B.
EPI_ISL_2571893, EPI_ISL_2571894, EPI_ISL_2571895, EPI_ISL_2571896	Department of Paediatric Infectious Diseases, Institute of Tropical Medicine, Nagasaki University	Department of Paediatric Infectious Diseases, Institute of Tropical Medicine, Nagasaki University	Yoshihara,K., Nhat Le,M., Nagasawa,K., Tsukagoshi,H., Nguyen,H.A., Toizumi,M., Moriuchi,H., Hashizume,M., Ariyoshi,K., Dang,D.A., Kimura,H. and Yoshida,L.-M.
EPI_ISL_2571908	Pathogen Diagnostic Center, Institut Pasteur of Shanghai	Pathogen Diagnostic Center, Institut Pasteur of Shanghai	Liu,J., Mu,Y., Dong,W., Yao,F., Yan,H., Lan,K. and Zhang,C.
EPI_ISL_2571953, EPI_ISL_2571954	Microbiology Department, Virology Division, College of Medicine, Taif University	Microbiology Department, Virology Division, College of Medicine, Taif University	Al Aboud,D.M., Al Aboud,N.M., Al-Malky,M.I.R. and Abdel-Moneim,A.S.
EPI_ISL_2571969	Epidemiology and Demography, KEMRI - Wellcome Trust Research Programme, Centre for Geographic Medicine Research	Epidemiology and Demography, KEMRI - Wellcome Trust Research Programme, Centre for Geographic Medicine Research	Oketch,J.W., Kamau,E., Otieno,J.R., Mwema,A., Lewa,C., Agoti,C.N. and Nokes,J.D.
EPI_ISL_2571972	Virology Department, Hospices Civils de Lyon	Virology Department, Hospices Civils de Lyon	Gaymard,A., Pichon,M. and Morfin,F.
EPI_ISL_2571977, EPI_ISL_2571980	Bashir Salim Faculty of Veterinary Medicine, University of Khartoum, Parasitology	Bashir Salim Faculty of Veterinary Medicine, University of Khartoum, Parasitology	Salim,B. and Khalil,S.O.
EPI_ISL_2572038, EPI_ISL_2572039	Department of Paediatric Infectious Diseases, Institute of Tropical Medicine, Nagasaki University	Department of Paediatric Infectious Diseases, Institute of Tropical Medicine, Nagasaki University	Yoshihara,K., Nhat Le,M., Nagasawa,K., Tsukagoshi,H., Nguyen,H.A., Toizumi,M., Moriuchi,H., Hashizume,M., Ariyoshi,K., Dang,D.A., Kimura,H. and Yoshida,L.-M.
EPI_ISL_2572048	Depto Microbiologia, Instituto de Ciencias Biomedicas, Universidade de Sao Paulo	Depto Microbiologia, Instituto de Ciencias Biomedicas, Universidade de Sao Paulo	Moura,F.E.A., Thomazelli,L.M., Campelo,F.S., Delfraro,A., Arbiza,J. and Durigon,E.L.
EPI_ISL_2572050	School of Medicine, University of Washington	School of Medicine, University of Washington	Scott,E.M., Magaret,A., Kuypers,J., Stewart,L., Shrestha,L., Tielsch,J.M., Steinhoff,M., Katz,J., Khatry,S.K., LeClerq,S.C., Englund,J.A. and Chu,H.Y.
EPI_ISL_2572051	Epidemiology and Demography, KEMRI - Wellcome Trust Research Programme, Centre for Geographic Medicine Research	Epidemiology and Demography, KEMRI - Wellcome Trust Research Programme, Centre for Geographic Medicine Research	Oketch,J.W., Kamau,E., Otieno,J.R., Mwema,A., Lewa,C., Agoti,C.N. and Nokes,J.D.
EPI_ISL_2572052	Institute of Endemic Diseases, University of Khartoum	Institute of Endemic Diseases, University of Khartoum	Khalil,S.O., Altayb,H.N., Anan,K.A., H,A.Y., Elkhidir,I.M. and Hassan,M.A.
EPI_ISL_2572054	Microbiology Department, Virology Division, College of Medicine, Taif University	Microbiology Department, Virology Division, College of Medicine, Taif University	Al Aboud,D.M., Al Aboud,N.M., Al-Malky,M.I.R. and Abdel-Moneim,A.S.
EPI_ISL_2572081, EPI_ISL_2572082	Department of Clinical Laboratory, Fujian Provincial Hospital	Department of Clinical Laboratory, Fujian Provincial Hospital	Su,Y., Wu,Y., Tian,R. and Liang,G.
EPI_ISL_2572087	Depto Microbiologia, Instituto de Ciencias Biomedicas, Universidade de Sao Paulo	Depto Microbiologia, Instituto de Ciencias Biomedicas, Universidade de Sao Paulo	Moura,F.E.A., Thomazelli,L.M., Campelo,F.S., Delfraro,A., Arbiza,J. and Durigon,E.L.
EPI_ISL_2572099	Department of Paediatric Infectious Diseases, Institute of Tropical Medicine, Nagasaki University	Department of Paediatric Infectious Diseases, Institute of Tropical Medicine, Nagasaki University	Yoshihara,K., Nhat Le,M., Nagasawa,K., Tsukagoshi,H., Nguyen,H.A., Toizumi,M., Moriuchi,H., Hashizume,M., Ariyoshi,K., Dang,D.A., Kimura,H. and Yoshida,L.-M.
EPI_ISL_2572102	Manipal Centre for Virus Research, Manipal University	Manipal Centre for Virus Research, Manipal University	Anjali,A., Aswathy,R., Akhil,C., Giselle,D., Revti,B., Hindol,M., Suresh,P. and Arunkumar,G.
EPI_ISL_2572112	School of Medicine, University of Washington	School of Medicine, University of Washington	Scott,E.M., Magaret,A., Kuypers,J., Stewart,L., Shrestha,L., Tielsch,J.M., Steinhoff,M., Katz,J., Khatry,S.K., LeClerq,S.C., Englund,J.A. and Chu,H.Y.
EPI_ISL_2572132, EPI_ISL_2572133, EPI_ISL_2572134, EPI_ISL_2572135	Health Science, Universidade Federal Do Parana	Health Science, Universidade Federal Do Parana	Moreira,F.B., Santos,J.S., Avanzi,V.M., Nogueira,M.B., Vidal,L.R.R. and Raboni,S.M.
EPI_ISL_2572168, EPI_ISL_2572169, EPI_ISL_2572170, EPI_ISL_2572171, EPI_ISL_2572172, EPI_ISL_2572173, EPI_ISL_2572174	Centre de Recherche en Infectiologie, Universite Laval, CHUL	Centre de Recherche en Infectiologie, Universite Laval, CHUL	Papenburg,J., Carbonneau,J., Hamelin,M.-E., Isabel,S., Ohoumanne,N., Dery,P., Paes,B.A., Corbeil,J., Bergeron,M.G., De Serres,G. and Boivin,G.
EPI_ISL_2572175, EPI_ISL_2572176, EPI_ISL_2572177, EPI_ISL_2572178	Laboratory Medicine and Pathobiology, University of Toronto	Laboratory Medicine and Pathobiology, University of Toronto	Granados,A., Duvvuri,V., Rosenfeld,P., Eshaghi,A. and Gubbay,J.
EPI_ISL_2572179	Laboratory Medicine and Pathobiology, University of Toronto	Laboratory Medicine and Pathobiology, University of Toronto	Granados,A., Duvvuri,V., Rosenfeld,P., Eshaghi,A. and Gubbay,J.B.
EPI_ISL_2572180, EPI_ISL_2572181, EPI_ISL_2572182	Laboratory Medicine and Pathobiology, University of Toronto	Laboratory Medicine and Pathobiology, University of Toronto	Granados,A., Duvvuri,V., Rosenfeld,P., Eshaghi,A. and Gubbay,J.
EPI_ISL_2572183	Laboratory Medicine and Pathobiology, University of Toronto	Laboratory Medicine and Pathobiology, University of Toronto	Granados,A., Duvvuri,V., Rosenfeld,P., Eshaghi,A. and Gubbay,J.B.
EPI_ISL_2572184	Laboratory Medicine and Pathobiology, University of Toronto	Laboratory Medicine and Pathobiology, University of Toronto	Granados,A., Duvvuri,V., Rosenfeld,P., Eshaghi,A. and Gubbay,J.
EPI_ISL_2572185	Laboratory Medicine and Pathobiology, University of Toronto	Laboratory Medicine and Pathobiology, University of Toronto	Granados,A., Duvvuri,V., Rosenfeld,P., Eshaghi,A. and Gubbay,J.B.
EPI_ISL_2572186, EPI_ISL_2572187, EPI_ISL_2572188	Laboratory Medicine and Pathobiology, University of Toronto	Laboratory Medicine and Pathobiology, University of Toronto	Granados,A., Duvvuri,V., Rosenfeld,P., Eshaghi,A. and Gubbay,J.
EPI_ISL_2572189	Laboratory Medicine and Pathobiology, University of Toronto	Laboratory Medicine and Pathobiology, University of Toronto	Granados,A., Duvvuri,V., Rosenfeld,P., Eshaghi,A. and Gubbay,J.B.
EPI_ISL_2572190, EPI_ISL_2572191	Laboratory Medicine and Pathobiology, University of Toronto	Laboratory Medicine and Pathobiology, University of Toronto	Granados,A., Duvvuri,V., Rosenfeld,P., Eshaghi,A. and Gubbay,J.
EPI_ISL_2572192	Laboratory Medicine and Pathobiology, University of Toronto	Laboratory Medicine and Pathobiology, University of Toronto	Granados,A., Duvvuri,V., Rosenfeld,P., Eshaghi,A. and Gubbay,J.B.
EPI_ISL_2572193, EPI_ISL_2572194	Laboratory Medicine and Pathobiology, University of Toronto	Laboratory Medicine and Pathobiology, University of Toronto	Granados,A., Duvvuri,V., Rosenfeld,P., Eshaghi,A. and Gubbay,J.
EPI_ISL_2572195, EPI_ISL_2572196, EPI_ISL_2572197	Laboratory Medicine and Pathobiology, University of Toronto	Laboratory Medicine and Pathobiology, University of Toronto	Granados,A., Duvvuri,V., Rosenfeld,P., Eshaghi,A. and Gubbay,J.B.
EPI_ISL_2572198, EPI_ISL_2572199	Laboratory Medicine and Pathobiology, University of Toronto	Laboratory Medicine and Pathobiology, University of Toronto	Granados,A., Duvvuri,V., Rosenfeld,P., Eshaghi,A. and Gubbay,J.
EPI_ISL_2572227	Central Laboratory, Guangzhou Women and Children's Medical Center	Central Laboratory, Guangzhou Women and Children's Medical Center	Xie,J.H., Zhu,B., Zhong,J.Y., Chen,Y. and Zhang,Y.Y.
EPI_ISL_2572229	WHO WPRO Measles Regional Reference Lab, Key Laboratory of Medical Virology Ministry of Health, National Institute for Viral Disease Control and Prevention, China Center for Disease Control and Prevention	WHO WPRO Measles Regional Reference Lab, Key Laboratory of Medical Virology Ministry of Health, National Institute for Viral Disease Control and Prevention, China Center for Disease Control and Prevention	Song,J., Zhang,Y. and Xu,W.
EPI_ISL_2572238	Laboratory of Virology, Capital Institute of Pediatrics	Laboratory of Virology, Capital Institute of Pediatrics	Cui,G., Deng,J., Zhu,R., Qian,Y., Sun,Y., Zhao,L. and Wang,F.
EPI_ISL_2572262	WHO WPRO Measles Regional Reference Lab, Key Laboratory of Medical Virology Ministry of Health, National Institute for Viral Disease Control and Prevention, China Center for Disease Control and Prevention	WHO WPRO Measles Regional Reference Lab, Key Laboratory of Medical Virology Ministry of Health, National Institute for Viral Disease Control and Prevention, China Center for Disease Control and Prevention	Song,J., Zhang,Y. and Xu,W.
EPI_ISL_2572264	WHO WPRO Measles Regional Reference Lab, Key Laboratory of Medical Virology Ministry of Health, National Institute for Viral Disease Control and Prevention, China Center for Disease Control and Prevention	WHO WPRO Measles Regional Reference Lab, Key Laboratory of Medical Virology Ministry of Health, National Institute for Viral Disease Control and Prevention, China Center for Disease Control and Prevention	Zhang,Y., Song,J. and Xu,W.
EPI_ISL_2572265, EPI_ISL_2572266, EPI_ISL_2572267	WHO WPRO Measles Regional Reference Lab, Key Laboratory of Medical Virology	WHO WPRO Measles Regional Reference Lab, Key Laboratory of Medical Virology	Song,J., Zhang,Y. and Xu,W.

	Ministry of Health, National Institute for Viral Disease Control and Prevention, China Center for Disease Control and Prevention	Ministry of Health, National Institute for Viral Disease Control and Prevention, China Center for Disease Control and Prevention	
EPI_ISL_2572288, EPI_ISL_2572290, EPI_ISL_2572303	University of Wuerzburg, Institute of Virology and Immunobiology	University of Wuerzburg, Institute of Virology and Immunobiology	Prifert,C., Hofmann,D. and Weissbrich,B.
EPI_ISL_2572354	Pediatric Clinic 1, Department of Pathophysiology and Transplantation, University of Milan and Fondazione IRCCS Ca Granda	Pediatric Clinic 1, Department of Pathophysiology and Transplantation, University of Milan and Fondazione IRCCS Ca Granda	Esposito,S., Zampiero,A., Piralla,A. and Principi,N.
EPI_ISL_2572387	Virology, Tohoku University Graduate School of Medicine	Virology, Tohoku University Graduate School of Medicine	Malasao,R., Okamoto,M., Chaimongkol,N., Imamura,T., Tohma,K., Dapat,I., Dapat,C., Suzuki,A., Saito,M., Saito,M., Tamaki,R., Segubre-Mercado,E., Igoy,M.A.U., Lupisan,S., Olveda,R. and Oshitani,H.
EPI_ISL_2572524, EPI_ISL_2572525, EPI_ISL_2572526, EPI_ISL_2572527, EPI_ISL_2572528, EPI_ISL_2572529, EPI_ISL_2572530, EPI_ISL_2572531, EPI_ISL_2572532, EPI_ISL_2572533, EPI_ISL_2572534, EPI_ISL_2572535, EPI_ISL_2572536, EPI_ISL_2572537			
see above	Department of Paediatric Infectious Diseases, Institute of Tropical Medicine, Nagasaki University	Department of Paediatric Infectious Diseases, Institute of Tropical Medicine, Nagasaki University	Yoshihara,K., Nhat Le,M., Nagasawa,K., Tsukagoshi,H., Nguyen,H.A., Toizumi,M., Moriuchi,H., Hashizume,M., Ariyoshi,K., Dang,D.A., Kimura,H. and Yoshida,L.-M.
EPI_ISL_2572550, EPI_ISL_2572551	Department of Clinical Laboratory, Fujian Provincial Hospital	Department of Clinical Laboratory, Fujian Provincial Hospital	Su,Y., Wu,Y., Tian,R. and Liang,G.
EPI_ISL_2572557, EPI_ISL_2572558	Virology, Tropical Medicine Institute Pedro Kouri	Virology, Tropical Medicine Institute Pedro Kouri	Valdes,O., Corso,M., Pinon,A., Acosta,B., Savon,C., Gonzalez,G., Mune,M., Gonzalez,G., Hernandez,B., Echevarria,Y. and Oropesa,S.
EPI_ISL_2572559	Influenza Group, National Institute of Virology	Influenza Group, National Institute of Virology	Choudhary,M.L., Anand,S.P., Wadhwa,B.S. and Chadha,M.S.
EPI_ISL_2572696	Investigacion en Virologia y Biotecnologia, Instituto Conmemorativo Gorgas de Estudios de la Salud	Investigacion en Virologia y Biotecnologia, Instituto Conmemorativo Gorgas de Estudios de la Salud	Abrego,L., Delfraro,A., Franco,D., Castillo,C., Cano,M., Castillo,M., Castillo,J., Pascale,J. and Arbiza,J.
EPI_ISL_2572703, EPI_ISL_2572704, EPI_ISL_2572705, EPI_ISL_2572706, EPI_ISL_2572707, EPI_ISL_2572708, EPI_ISL_2572725, EPI_ISL_2572726, EPI_ISL_2572727	Department of Clinical Laboratory, Fujian Provincial Hospital	Department of Clinical Laboratory, Fujian Provincial Hospital	Su,Y., Wu,Y., Tian,R. and Liang,G.
EPI_ISL_2572728	West China School of Public Health, Sichuan University	West China School of Public Health, Sichuan University	Zhang,M.
EPI_ISL_2572782	Pediatrics, Show-Chwan Memorial Hospital	Pediatrics, Show-Chwan Memorial Hospital	Lee,C.-Y.
EPI_ISL_2572811	Microbiology Department, Virology Division, College of Medicine, Taif University	Microbiology Department, Virology Division, College of Medicine, Taif University	Al Aboud,D.M., Al Aboud,N.M., Al-Malky,M.I.R. and Abdel-Moneim,A.S.
EPI_ISL_2572819	Centre for Research And Knowledge Transfer in Biotechnology, University of Zagreb	Centre for Research And Knowledge Transfer in Biotechnology, University of Zagreb	Ivancic-Jelecki,J., Slovic,A., Ljubin-Sternak,S., Mlinaric-Galinovic,G. and Forcic,D.
EPI_ISL_2572828	Department of Clinical Laboratory, Fujian Provincial Hospital	Department of Clinical Laboratory, Fujian Provincial Hospital	Su,Y., Wu,Y., Tian,R. and Liang,G.
EPI_ISL_2572868	Laboratory Medicine and Pathobiology, University of Toronto	Laboratory Medicine and Pathobiology, University of Toronto	Granados,A., Duvvuri,V., Rosenfeld,P., Eshaghi,A. and Gubbay,J.B.
EPI_ISL_2572929	University of Wuerzburg, Institute of Virology and Immunobiology	University of Wuerzburg, Institute of Virology and Immunobiology	Prifert,C., Hofmann,D. and Weissbrich,B.
EPI_ISL_2573053, EPI_ISL_2573054, EPI_ISL_2573055, EPI_ISL_2573056	Department of Paediatric Infectious Diseases, Institute of Tropical Medicine, Nagasaki University	Department of Paediatric Infectious Diseases, Institute of Tropical Medicine, Nagasaki University	Yoshihara,K., Nhat Le,M., Nagasawa,K., Tsukagoshi,H., Nguyen,H.A., Toizumi,M., Moriuchi,H., Hashizume,M., Ariyoshi,K., Dang,D.A., Kimura,H. and Yoshida,L.-M.
EPI_ISL_2573068	Pathogen Diagnostic Center, Institut Pasteur of Shanghai	Pathogen Diagnostic Center, Institut Pasteur of Shanghai	Liu,J., Mu,Y., Dong,W., Yao,F., Yan,H., Lan,K. and Zhang,C.
EPI_ISL_2573113, EPI_ISL_2573114	Microbiology Department, Virology Division, College of Medicine, Taif University	Microbiology Department, Virology Division, College of Medicine, Taif University	Al Aboud,D.M., Al Aboud,N.M., Al-Malky,M.I.R. and Abdel-Moneim,A.S.
EPI_ISL_2573129	Epidemiology and Demography, KEMRI - Wellcome Trust Research Programme, Centre for Geographic Medicine Research	Epidemiology and Demography, KEMRI - Wellcome Trust Research Programme, Centre for Geographic Medicine Research	Oketch,J.W., Kamau,E., Otieno,J.R., Mwema,A., Lewa,C., Agoti,C.N. and Nokes,J.D.
EPI_ISL_2573132	Virology Department, Hospices Civils de Lyon	Virology Department, Hospices Civils de Lyon	Gaymard,A., Pichon,M. and Morfin,F.
EPI_ISL_2573137, EPI_ISL_2573140	Bashir Salim Faculty of Veterinary Medicine, University of Khartoum, Parasitology	Bashir Salim Faculty of Veterinary Medicine, University of Khartoum, Parasitology	Salim,B. and Khalil,S.O.
EPI_ISL_2573198, EPI_ISL_2573199	Department of Paediatric Infectious Diseases, Institute of Tropical Medicine, Nagasaki University	Department of Paediatric Infectious Diseases, Institute of Tropical Medicine, Nagasaki University	Yoshihara,K., Nhat Le,M., Nagasawa,K., Tsukagoshi,H., Nguyen,H.A., Toizumi,M., Moriuchi,H., Hashizume,M., Ariyoshi,K., Dang,D.A., Kimura,H. and Yoshida,L.-M.
EPI_ISL_2573210	School of Medicine, University of Washington	School of Medicine, University of Washington	Scott,E.M., Magaret,A., Kuypers,J., Stewart,L., Shrestha,L., Tielsch,J.M., Steinhoff,M., Katz,J., Khatry,S.K., LeClerq,S.C., Englund,J.A. and Chu,H.Y.
EPI_ISL_2573211	Epidemiology and Demography, KEMRI - Wellcome Trust Research Programme, Centre for Geographic Medicine Research	Epidemiology and Demography, KEMRI - Wellcome Trust Research Programme, Centre for Geographic Medicine Research	Oketch,J.W., Kamau,E., Otieno,J.R., Mwema,A., Lewa,C., Agoti,C.N. and Nokes,J.D.
EPI_ISL_2573212	Institute of Endemic Diseases, University of Khartoum	Institute of Endemic Diseases, University of Khartoum	Khalil,S.O., Altayb,H.N., Anan,K.A., H,A.Y., Elkhidir,I.M. and Hassan,M.A.
EPI_ISL_2573214	Microbiology Department, Virology Division, College of Medicine, Taif University	Microbiology Department, Virology Division, College of Medicine, Taif University	Al Aboud,D.M., Al Aboud,N.M., Al-Malky,M.I.R. and Abdel-Moneim,A.S.
EPI_ISL_2573241, EPI_ISL_2573242	Department of Clinical Laboratory, Fujian Provincial Hospital	Department of Clinical Laboratory, Fujian Provincial Hospital	Su,Y., Wu,Y., Tian,R. and Liang,G.
EPI_ISL_2573259	Department of Paediatric Infectious Diseases, Institute of Tropical Medicine, Nagasaki University	Department of Paediatric Infectious Diseases, Institute of Tropical Medicine, Nagasaki University	Yoshihara,K., Nhat Le,M., Nagasawa,K., Tsukagoshi,H., Nguyen,H.A., Toizumi,M., Moriuchi,H., Hashizume,M., Ariyoshi,K., Dang,D.A., Kimura,H. and Yoshida,L.-M.
EPI_ISL_2573262	Manipal Centre for Virus Research, Manipal University	Manipal Centre for Virus Research, Manipal University	Anjali,A., Aswathy,R., Akhil,C., Giselle,D., Revti,B., Hindol,M., Suresh,P. and Arunkumar,G.
EPI_ISL_2573272	School of Medicine, University of Washington	School of Medicine, University of Washington	Scott,E.M., Magaret,A., Kuypers,J., Stewart,L., Shrestha,L., Tielsch,J.M., Steinhoff,M., Katz,J., Khatry,S.K., LeClerq,S.C., Englund,J.A. and Chu,H.Y.
EPI_ISL_2573328, EPI_ISL_2573329, EPI_ISL_2573330, EPI_ISL_2573331, EPI_ISL_2573332, EPI_ISL_2573333, EPI_ISL_2573334	Centre de Recherche en Infectiologie, Universite Laval, CHUL	Centre de Recherche en Infectiologie, Universite Laval, CHUL	Papenburg,J., Carbonneau,J., Hamelin,M.-E., Isabel,S., Ohoumanne,N., Dery,P., Paes,B.A., Corbeil,J., Bergeron,M.G., De Serres,G. and Boivin,G.
EPI_ISL_2573335, EPI_ISL_2573336, EPI_ISL_2573337, EPI_ISL_2573338	Laboratory Medicine and Pathobiology, University of Toronto	Laboratory Medicine and Pathobiology, University of Toronto	Granados,A., Duvvuri,V., Rosenfeld,P., Eshaghi,A. and Gubbay,J.
EPI_ISL_2573339	Laboratory Medicine and Pathobiology, University of Toronto	Laboratory Medicine and Pathobiology, University of Toronto	Granados,A., Duvvuri,V., Rosenfeld,P., Eshaghi,A. and Gubbay,J.B.
EPI_ISL_2573340, EPI_ISL_2573341, EPI_ISL_2573342	Laboratory Medicine and Pathobiology, University of Toronto	Laboratory Medicine and Pathobiology, University of Toronto	Granados,A., Duvvuri,V., Rosenfeld,P., Eshaghi,A. and Gubbay,J.
EPI_ISL_2573343	Laboratory Medicine and Pathobiology, University of Toronto	Laboratory Medicine and Pathobiology, University of Toronto	Granados,A., Duvvuri,V., Rosenfeld,P., Eshaghi,A. and Gubbay,J.B.
EPI_ISL_2573344	Laboratory Medicine and Pathobiology, University of Toronto	Laboratory Medicine and Pathobiology, University of Toronto	Granados,A., Duvvuri,V., Rosenfeld,P., Eshaghi,A. and Gubbay,J.
EPI_ISL_2573345	Laboratory Medicine and Pathobiology, University of Toronto	Laboratory Medicine and Pathobiology, University of Toronto	Granados,A., Duvvuri,V., Rosenfeld,P., Eshaghi,A. and Gubbay,J.B.
EPI_ISL_2573346, EPI_ISL_2573347, EPI_ISL_2573348	Laboratory Medicine and Pathobiology, University of Toronto	Laboratory Medicine and Pathobiology, University of Toronto	Granados,A., Duvvuri,V., Rosenfeld,P., Eshaghi,A. and Gubbay,J.
EPI_ISL_2573349	Laboratory Medicine and Pathobiology, University of Toronto	Laboratory Medicine and Pathobiology, University of Toronto	Granados,A., Duvvuri,V., Rosenfeld,P., Eshaghi,A. and Gubbay,J.B.
EPI_ISL_2573350, EPI_ISL_2573351	Laboratory Medicine and Pathobiology, University of Toronto	Laboratory Medicine and Pathobiology, University of Toronto	Granados,A., Duvvuri,V., Rosenfeld,P., Eshaghi,A. and Gubbay,J.
EPI_ISL_2573352	Laboratory Medicine and Pathobiology, University of Toronto	Laboratory Medicine and Pathobiology, University of Toronto	Granados,A., Duvvuri,V., Rosenfeld,P., Eshaghi,A. and Gubbay,J.B.
EPI_ISL_2573353, EPI_ISL_2573354	Laboratory Medicine and Pathobiology, University of Toronto	Laboratory Medicine and Pathobiology, University of Toronto	Granados,A., Duvvuri,V., Rosenfeld,P., Eshaghi,A. and Gubbay,J.
EPI_ISL_2573355, EPI_ISL_2573356, EPI_ISL_2573357	Laboratory Medicine and Pathobiology, University of Toronto	Laboratory Medicine and Pathobiology, University of Toronto	Granados,A., Duvvuri,V., Rosenfeld,P., Eshaghi,A. and Gubbay,J.B.
EPI_ISL_2573358, EPI_ISL_2573359	Laboratory Medicine and Pathobiology, University of Toronto	Laboratory Medicine and Pathobiology, University of Toronto	Granados,A., Duvvuri,V., Rosenfeld,P., Eshaghi,A. and Gubbay,J.
EPI_ISL_2573387	Central Laboratory, Guangzhou Women and Children's Medical Center	Central Laboratory, Guangzhou Women and Children's Medical Center	Xie,J.H., Zhu,B., Zhong,J.Y., Chen,Y. and Zhang,Y.Y.
EPI_ISL_2573389	WHO WPRO Measles Regional Reference Lab, Key Laboratory of Medical Virology Ministry of Health, National Institute for Viral Disease Control and Prevention, China Center for Disease Control and Prevention	WHO WPRO Measles Regional Reference Lab, Key Laboratory of Medical Virology Ministry of Health, National Institute for Viral Disease Control and Prevention, China Center for Disease Control and Prevention	Song,J., Zhang,Y. and Xu,W.
EPI_ISL_2573398	Laboratory of Virology, Capital Institute of Pediatrics	Laboratory of Virology, Capital Institute of Pediatrics	Cui,G., Deng,J., Zhu,R., Qian,Y., Sun,Y., Zhao,L. and Wang,F.
EPI_ISL_2573422	WHO WPRO Measles Regional Reference Lab, Key Laboratory of Medical Virology Ministry of Health, National Institute for Viral Disease Control and Prevention, China Center for Disease Control and Prevention	WHO WPRO Measles Regional Reference Lab, Key Laboratory of Medical Virology Ministry of Health, National Institute for Viral Disease Control and Prevention, China Center for Disease Control and Prevention	Song,J., Zhang,Y. and Xu,W.
EPI_ISL_2573424	WHO WPRO Measles Regional Reference Lab, Key Laboratory of Medical Virology Ministry of Health, National Institute for Viral Disease Control and Prevention, China Center for Disease Control and Prevention	WHO WPRO Measles Regional Reference Lab, Key Laboratory of Medical Virology Ministry of Health, National Institute for Viral Disease Control and Prevention, China Center for Disease Control and Prevention	Zhang,Y., Song,J. and Xu,W.
EPI_ISL_2573425, EPI_ISL_2573426, EPI_ISL_2573427	WHO WPRO Measles Regional Reference Lab, Key Laboratory of Medical Virology Ministry of Health, National Institute for Viral Disease Control and Prevention, China Center for Disease Control and Prevention	WHO WPRO Measles Regional Reference Lab, Key Laboratory of Medical Virology Ministry of Health, National Institute for Viral Disease Control and Prevention, China Center for Disease Control and Prevention	Song,J., Zhang,Y. and Xu,W.
EPI_ISL_2573497	Department of Respiratory Medicine, Children's Hospital of Chongqing Medical University	Department of Respiratory Medicine, Children's Hospital of Chongqing Medical University	Ren,L. and Liu,E.
EPI_ISL_2573505, EPI_ISL_2573506, EPI_ISL_2573507, EPI_ISL_2573508, EPI_ISL_2573509, EPI_ISL_2573510, EPI_ISL_2573511, EPI_ISL_2573513, EPI_ISL_2573514, EPI_ISL_2573515, EPI_ISL_2573516, EPI_ISL_2573517, EPI_ISL_2573518, EPI_ISL_2573529, EPI_ISL_2573533, EPI_ISL_2573534, EPI_ISL_2573541, EPI_ISL_2573542, EPI_ISL_2573543, EPI_ISL_2573544			
see above	WHO WPRO Measles Regional Reference Lab, Key Laboratory of Medical Virology Ministry of Health, National Institute for Viral Disease Control and Prevention, China Center for Disease Control and Prevention	WHO WPRO Measles Regional Reference Lab, Key Laboratory of Medical Virology Ministry of Health, National Institute for Viral Disease Control and Prevention, China Center for Disease Control and Prevention	Song,J., Zhang,Y. and Xu,W.
EPI_ISL_2573556, EPI_ISL_2573563, EPI_ISL_2573564	Centre for Research And Knowledge Transfer in Biotechnology, University of Zagreb	Centre for Research And Knowledge Transfer in Biotechnology, University of Zagreb	Jagusic,M., Slovic,A., Santak,M., Kosutic-Gulija,T., Ivancic-Jelecki,J. and Forcic,D.
EPI_ISL_2573565	Centre for Research And Knowledge Transfer in Biotechnology, University of Zagreb	Centre for Research And Knowledge Transfer in Biotechnology, University of Zagreb	Ivancic-Jelecki,J., Slovic,A., Ljubin-Sternak,S., Mlinaric-Galinovic,G. and Forcic,D.
EPI_ISL_2573566	Centre for Research And Knowledge Transfer in Biotechnology, University of Zagreb	Centre for Research And Knowledge Transfer in Biotechnology, University of Zagreb	Jagusic,M., Slovic,A., Santak,M., Kosutic-Gulija,T., Ivancic-Jelecki,J. and Forcic,D.
EPI_ISL_2573567	Centre for Research And Knowledge Transfer in Biotechnology, University of Zagreb	Centre for Research And Knowledge Transfer in Biotechnology, University of Zagreb	Ivancic-Jelecki,J., Slovic,A., Ljubin-Sternak,S., Mlinaric-Galinovic,G. and Forcic,D.

EPI_ISL_2573583, EPI_ISL_2573584, EPI_ISL_2573585, EPI_ISL_2573615	Centre for Research And Knowledge Transfer in Biotechnology, University of Zagreb	Centre for Research And Knowledge Transfer in Biotechnology, University of Zagreb	Jagusic,M., Slovic,A., Santak,M., Kosutic-Gulija,T., Ivancic-Jelecki,J. and Forcic,D.
EPI_ISL_2573616	Centre for Research And Knowledge Transfer in Biotechnology, University of Zagreb	Centre for Research And Knowledge Transfer in Biotechnology, University of Zagreb	Ivancic-Jelecki,J., Slovic,A., Ljubin-Sternak,S., Mlinaric-Galinovic,G. and Forcic,D.
EPI_ISL_2573619, EPI_ISL_2573620, EPI_ISL_2573621, EPI_ISL_2573622, EPI_ISL_2573623, EPI_ISL_2573624, EPI_ISL_2573625, EPI_ISL_2573626, EPI_ISL_2573627, EPI_ISL_2573628, EPI_ISL_2573629			
see above	Virology, Tropical Medicine Institute Pedro Kouri	Virology, Tropical Medicine Institute Pedro Kouri	Valdes,O., Corso,M., Pinon,A., Acosta,B., Savon,C., Gonzalez,G., Mune,M., Gonzalez,G., Hernandez,B., Echevarria,Y. and Oropesa,S.
EPI_ISL_2573633, EPI_ISL_2573634, EPI_ISL_2573635, EPI_ISL_2573636, EPI_ISL_2573637, EPI_ISL_2573638, EPI_ISL_2573639, EPI_ISL_2573640, EPI_ISL_2573641, EPI_ISL_2573642, EPI_ISL_2573643			
see above	Virology Department, Hospices Civils de Lyon	Virology Department, Hospices Civils de Lyon	Gaymard,A., Pichon,M. and Morfin,F.
EPI_ISL_2573717, EPI_ISL_2573721, EPI_ISL_2573722, EPI_ISL_2573743	University of Wuerzburg, Institute of Virology and Immunobiology	University of Wuerzburg, Institute of Virology and Immunobiology	Prifert,C., Hofmann,D. and Weissbrich,B.
EPI_ISL_2573813	Microbiology, King George Medical University	Microbiology, King George Medical University	Singh,A.K., Jain,B., Dangi,T., Verma,A., Dwivedi,M., Prakash,S. and Jain,A.
EPI_ISL_2573817, EPI_ISL_2573818, EPI_ISL_2573819, EPI_ISL_2573820, EPI_ISL_2573821, EPI_ISL_2573822, EPI_ISL_2573823, EPI_ISL_2573824, EPI_ISL_2573825, EPI_ISL_2573826, EPI_ISL_2573827, EPI_ISL_2573828, EPI_ISL_2573829, EPI_ISL_2573830, EPI_ISL_2573832			
see above	Virology Section, Department of Microbiology, King George's Medical University	Virology Section, Department of Microbiology, King George's Medical University	Singh,A.K., Jain,A., Jain,B., Dangi,T., Verma,A.K., Dwivedi,M. and Kushwaha,R.
EPI_ISL_2573885	Molecular Medicine Laboratory of Virology, Sapienza University	Molecular Medicine Laboratory of Virology, Sapienza University	Pierangeli,A.
EPI_ISL_2573946	Toshimitsu Tanaka Chiba City Institute of Health and Environment	Toshimitsu Tanaka Chiba City Institute of Health and Environment	Tanaka,T. and Tanaka,M.
EPI_ISL_2573947, EPI_ISL_2573948, EPI_ISL_2573949, EPI_ISL_2573950, EPI_ISL_2573951, EPI_ISL_2573952, EPI_ISL_2573953, EPI_ISL_2573954, EPI_ISL_2573955, EPI_ISL_2573956, EPI_ISL_2573957, EPI_ISL_2573958, EPI_ISL_2573959, EPI_ISL_2573960, EPI_ISL_2573961			
see above	Miho Kobayashi Gunma Prefectural Institute of Public Health and Environmental Sciences	Miho Kobayashi Gunma Prefectural Institute of Public Health and Environmental Sciences	Kobayashi,M. and Kimura,H.
EPI_ISL_2573968	Akinobu Hibino Niigata University, International Health, Public Health	Akinobu Hibino Niigata University, International Health, Public Health	Hibino,A., Saito,R., Shoubugawa,Y. and Takei,T.
EPI_ISL_2573997, EPI_ISL_2573998, EPI_ISL_2573999, EPI_ISL_2574000	Akinobu Hibino Niigata University, International Health, Public Health	Akinobu Hibino Niigata University, International Health, Public Health	Hibino,A., Saito,R., Shoubugawa,Y. and Sano,Y.
EPI_ISL_2574170, EPI_ISL_2574174, EPI_ISL_2574175, EPI_ISL_2574198	University of Wuerzburg, Institute of Virology and Immunobiology	University of Wuerzburg, Institute of Virology and Immunobiology	Prifert,C., Hofmann,D. and Weissbrich,B.
EPI_ISL_2574274	Microbiology, King George Medical University	Microbiology, King George Medical University	Singh,A.K., Jain,B., Dangi,T., Verma,A., Dwivedi,M., Prakash,S. and Jain,A.
EPI_ISL_2574278, EPI_ISL_2574279, EPI_ISL_2574280, EPI_ISL_2574281, EPI_ISL_2574282, EPI_ISL_2574284, EPI_ISL_2574285, EPI_ISL_2574286, EPI_ISL_2574287, EPI_ISL_2574288, EPI_ISL_2574289, EPI_ISL_2574290, EPI_ISL_2574291, EPI_ISL_2574292, EPI_ISL_2574294			
see above	Virology Section, Department of Microbiology, King George's Medical University	Virology Section, Department of Microbiology, King George's Medical University	Singh,A.K., Jain,A., Jain,B., Dangi,T., Verma,A.K., Dwivedi,M. and Kushwaha,R.
EPI_ISL_2574352	Molecular Medicine Laboratory of Virology, Sapienza University	Molecular Medicine Laboratory of Virology, Sapienza University	Pierangeli,A.
EPI_ISL_2574416	Toshimitsu Tanaka Chiba City Institute of Health and Environment	Toshimitsu Tanaka Chiba City Institute of Health and Environment	Tanaka,T. and Tanaka,M.
EPI_ISL_2574417, EPI_ISL_2574418, EPI_ISL_2574419, EPI_ISL_2574420, EPI_ISL_2574421, EPI_ISL_2574422, EPI_ISL_2574423, EPI_ISL_2574424, EPI_ISL_2574425, EPI_ISL_2574426, EPI_ISL_2574427, EPI_ISL_2574428, EPI_ISL_2574429, EPI_ISL_2574430, EPI_ISL_2574431			
see above	Miho Kobayashi Gunma Prefectural Institute of Public Health and Environmental Sciences	Miho Kobayashi Gunma Prefectural Institute of Public Health and Environmental Sciences	Kobayashi,M. and Kimura,H.
EPI_ISL_2574438	Akinobu Hibino Niigata University, International Health, Public Health	Akinobu Hibino Niigata University, International Health, Public Health	Hibino,A., Saito,R., Shoubugawa,Y. and Takei,T.
EPI_ISL_2574468, EPI_ISL_2574469, EPI_ISL_2574470, EPI_ISL_2574471	Akinobu Hibino Niigata University, International Health, Public Health	Akinobu Hibino Niigata University, International Health, Public Health	Hibino,A., Saito,R., Shoubugawa,Y. and Sano,Y.
EPI_ISL_2574506, EPI_ISL_2574507	Miho Kobayashi Gunma Prefectural Institute of Public Health and Environmental Sciences	Miho Kobayashi Gunma Prefectural Institute of Public Health and Environmental Sciences	Kobayashi,M. and Kimura,H.
EPI_ISL_2574518, EPI_ISL_2574519	Epidemiology and Demography, KEMRI - Wellcome Trust Research Programme, Centre for Geographic Medicine Research	Epidemiology and Demography, KEMRI - Wellcome Trust Research Programme, Centre for Geographic Medicine Research	Oketch,J.W., Kamau,E., Otieno,J.R., Mwema,A., Lewa,C., Agoti,C.N. and Nokes,J.D.
EPI_ISL_2574521, EPI_ISL_2574522, EPI_ISL_2574523, EPI_ISL_2574524, EPI_ISL_2574525, EPI_ISL_2574526, EPI_ISL_2574527, EPI_ISL_2574528, EPI_ISL_2574529, EPI_ISL_2574530, EPI_ISL_2574531, EPI_ISL_2574532, EPI_ISL_2574533, EPI_ISL_2574534, EPI_ISL_2574535, EPI_ISL_2574536, EPI_ISL_2574537, EPI_ISL_2574538, EPI_ISL_2574539, EPI_ISL_2574540, EPI_ISL_2574541, EPI_ISL_2574542, EPI_ISL_2574543, EPI_ISL_2574544, EPI_ISL_2574545, EPI_ISL_2574546, EPI_ISL_2574547, EPI_ISL_2574548, EPI_ISL_2574549, EPI_ISL_2574550, EPI_ISL_2574551, EPI_ISL_2574552, EPI_ISL_2574553, EPI_ISL_2574554, EPI_ISL_2574555, EPI_ISL_2574556, EPI_ISL_2574557, EPI_ISL_2574558, EPI_ISL_2574559, EPI_ISL_2574560, EPI_ISL_2574561, EPI_ISL_2574562, EPI_ISL_2574563, EPI_ISL_2574564, EPI_ISL_2574565, EPI_ISL_2574566, EPI_ISL_2574567, EPI_ISL_2574568, EPI_ISL_2574569, EPI_ISL_2574570, EPI_ISL_2574571, EPI_ISL_2574572			
see above	Department of Epidemiology and Demography, Kenya Medical Research Institute (KEMRI) - Wellcome Trust Research Programme	Department of Epidemiology and Demography, Kenya Medical Research Institute (KEMRI) - Wellcome Trust Research Programme	Otieno,J.R., Agoti,C.N., Gitahi,C.W., Bett,A., Ngama,M., Medley,G.F., Cane,P.A. and Nokes,J.D.
EPI_ISL_2574574, EPI_ISL_2574578, EPI_ISL_2574579, EPI_ISL_2574580, EPI_ISL_2574582, EPI_ISL_2574584, EPI_ISL_2574585, EPI_ISL_2574587, EPI_ISL_2574588, EPI_ISL_2574591, EPI_ISL_2574592, EPI_ISL_2574596, EPI_ISL_2574599, EPI_ISL_2574602, EPI_ISL_2574604, EPI_ISL_2574605, EPI_ISL_2574607, EPI_ISL_2574608, EPI_ISL_2574610, EPI_ISL_2574612, EPI_ISL_2574619, EPI_ISL_2574620, EPI_ISL_2574622, EPI_ISL_2574623, EPI_ISL_2574624, EPI_ISL_2574626, EPI_ISL_2574627, EPI_ISL_2574628, EPI_ISL_2574629, EPI_ISL_2574630, EPI_ISL_2574631, EPI_ISL_2574632, EPI_ISL_2574633, EPI_ISL_2574634, EPI_ISL_2574635, EPI_ISL_2574636, EPI_ISL_2574637, EPI_ISL_2574638, EPI_ISL_2574639, EPI_ISL_2574640, EPI_ISL_2574641, EPI_ISL_2574642, EPI_ISL_2574643, EPI_ISL_2574644, EPI_ISL_2574645, EPI_ISL_2574646, EPI_ISL_2574648, EPI_ISL_2574649, EPI_ISL_2574650, EPI_ISL_2574651, EPI_ISL_2574652, EPI_ISL_2574653			
see above	Epidemiology and Demography, Kenya Medical Research Institute (KEMRI) - Wellcome Trust Research Programme	Epidemiology and Demography, Kenya Medical Research Institute (KEMRI) - Wellcome Trust Research Programme	Agoti,C.N., Otieno,J.R., Gitahi,C.W., Cane,P.A. and Nokes,D.J.
EPI_ISL_2574728	Epidemiology and Demography Department, KEMRI-Wellcome Trust Research Programme	Epidemiology and Demography Department, KEMRI-Wellcome Trust Research Programme	Otieno,J.R., Kamau,E.M., Oketch,J.W., Ngoi,J.M., Agoti,C.N., Gichuki,A.M., Otieno,G.P., Ngama,M., Cane,P.A., Kellam,P., Cotten,M., Lemey,P. and Nokes,D.J.
EPI_ISL_2574729	Department of Protein Engineering, Latvian Biomedical Research and Study Centre	Department of Protein Engineering, Latvian Biomedical Research and Study Centre	Balmaks,R., Ribakova,I., Gardovska,D. and Kazaks,A.
EPI_ISL_2574809	Epidemiology and Demography, KEMRI - Wellcome Trust Research Programme, Centre for Geographic Medicine Research	Epidemiology and Demography, KEMRI - Wellcome Trust Research Programme, Centre for Geographic Medicine Research	Oketch,J.W., Kamau,E., Otieno,J.R., Mwema,A., Lewa,C., Agoti,C.N. and Nokes,J.D.
EPI_ISL_2574856, EPI_ISL_2574857, EPI_ISL_2574858, EPI_ISL_2574859, EPI_ISL_2574860, EPI_ISL_2574861, EPI_ISL_2574862, EPI_ISL_2574863, EPI_ISL_2574864, EPI_ISL_2574865, EPI_ISL_2574866, EPI_ISL_2574867, EPI_ISL_2574868, EPI_ISL_2574869, EPI_ISL_2574870			
see above	Investigacion en Virologia y Biotecnologia, Instituto Conmemorativo Gorgas de Estudios de la Salud	Investigacion en Virologia y Biotecnologia, Instituto Conmemorativo Gorgas de Estudios de la Salud	Abrego,L., Delfraro,A., Franco,D., Castillo,C., Cano,M., Castillo,M., Castillo,J., Pascale,J. and Arbiza,J.
EPI_ISL_2574875, EPI_ISL_2574876, EPI_ISL_2574877, EPI_ISL_2574885, EPI_ISL_2574886	Virology, Tohoku University Graduate School of Medicine	Virology, Tohoku University Graduate School of Medicine	Malasao,R., Okamoto,M., Chaimongkol,N., Imamura,T., Tohma,K., Dapat,I., Dapat,C., Suzuki,A., Saito,M., Saito,M., Tamaki,R., Segubre-Mercado,E., Igoy,M.A.U., Lupisan,S., Olveda,R. and Oshitani,H.
EPI_ISL_2574935, EPI_ISL_2574937	Department of Infectious Diseases, National Health Institute Doutor Ricardo Jorge	Department of Infectious Diseases, National Health Institute Doutor Ricardo Jorge	Saez-Lopez,E., Cristovao,P., Costa,I., Pechirra,P., Conde,P. and Guiomar,R.
EPI_ISL_2574999, EPI_ISL_2575000	Center for Medical Biotechnology, VIB-UGent	Center for Medical Biotechnology, VIB-UGent	Rossey,I., Schepens,B., Van Cauwenberghe,S., Saelens,X. and Melero,J.A.
EPI_ISL_2575119, EPI_ISL_2575120, EPI_ISL_2575121, EPI_ISL_2575122, EPI_ISL_2575123	Bashir Salim Faculty of Veterinary Medicine, University of Khartoum, Parasitology	Bashir Salim Faculty of Veterinary Medicine, University of Khartoum, Parasitology	Salim,B. and Khalil,S.O.
EPI_ISL_2575150, EPI_ISL_2575151, EPI_ISL_2575152, EPI_ISL_2575153	Pediatrics, Mackay Memorial Hospital	Pediatrics, Mackay Memorial Hospital	Chi,H., Liu,H.F., Weng,L.C., Wang,N.Y., Chiang,C.L., Chiu,Y.Y., Huang,G.J., Cheng,Y.Y., Chiu,N.C., Hsieh,W.S. and Huang,L.M.
EPI_ISL_2575180	Pediatrics, Show-Chwan Memorial Hospital	Pediatrics, Show-Chwan Memorial Hospital	Lee,C.-Y.
EPI_ISL_2575182, EPI_ISL_2575191, EPI_ISL_2575192, EPI_ISL_2575193, EPI_ISL_2575194, EPI_ISL_2575195, EPI_ISL_2575212, EPI_ISL_2575213, EPI_ISL_2575214, EPI_ISL_2575215, EPI_ISL_2575216, EPI_ISL_2575217, EPI_ISL_2575218, EPI_ISL_2575219, EPI_ISL_2575220, EPI_ISL_2575221			
see above	Department of Pediatrics, Center of Excellence in Clinical Virology, Faculty of Medicine, Chulalongkorn University	Department of Pediatrics, Center of Excellence in Clinical Virology, Faculty of Medicine, Chulalongkorn University	Thongpan,I., Mauleekoonphairoj,J., Vichiwattana,P., Korkong,S., Vongpunsawad,S. and Poovorawan,Y.
EPI_ISL_2575248, EPI_ISL_2575249, EPI_ISL_2575250, EPI_ISL_2575251, EPI_ISL_2575252, EPI_ISL_2575253, EPI_ISL_2575254, EPI_ISL_2575255, EPI_ISL_2575256, EPI_ISL_2575257, EPI_ISL_2575258, EPI_ISL_2575259, EPI_ISL_2575260, EPI_ISL_2575261, EPI_ISL_2575262, EPI_ISL_2575263, EPI_ISL_2575264, EPI_ISL_2575265, EPI_ISL_2575266, EPI_ISL_2575267, EPI_ISL_2575268, EPI_ISL_2575269, EPI_ISL_2575270, EPI_ISL_2575271, EPI_ISL_2575272, EPI_ISL_2575273, EPI_ISL_2575274, EPI_ISL_2575275, EPI_ISL_2575276, EPI_ISL_2575277, EPI_ISL_2575278, EPI_ISL_2575279, EPI_ISL_2575280, EPI_ISL_2575281, EPI_ISL_2575282, EPI_ISL_2575283, EPI_ISL_2575284, EPI_ISL_2575285, EPI_ISL_2575286, EPI_ISL_2575287, EPI_ISL_2575288, EPI_ISL_2575289, EPI_ISL_2575290, EPI_ISL_2575291, EPI_ISL_2575292, EPI_ISL_2575293, EPI_ISL_2575294, EPI_ISL_2575295, EPI_ISL_2575296, EPI_ISL_2575297, EPI_ISL_2575298, EPI_ISL_2575299, EPI_ISL_2575300, EPI_ISL_2575301, EPI_ISL_2575302, EPI_ISL_2575303, EPI_ISL_2575304, EPI_ISL_2575305, EPI_ISL_2575306, EPI_ISL_2575307, EPI_ISL_2575308, EPI_ISL_2575309, EPI_ISL_2575310, EPI_ISL_2575311, EPI_ISL_2575312, EPI_ISL_2575313, EPI_ISL_2575314, EPI_ISL_2575315, EPI_ISL_2575316, EPI_ISL_2575317, EPI_ISL_2575318, EPI_ISL_2575319, EPI_ISL_2575320, EPI_ISL_2575321, EPI_ISL_2575322, EPI_ISL_2575323, EPI_ISL_2575324, EPI_ISL_2575325, EPI_ISL_2575326, EPI_ISL_2575327, EPI_ISL_2575328, EPI_ISL_2575329, EPI_ISL_2575330, EPI_ISL_2575331, EPI_ISL_2575332, EPI_ISL_2575333, EPI_ISL_2575334, EPI_ISL_2575335, EPI_ISL_2575336, EPI_ISL_2575337, EPI_ISL_2575338, EPI_ISL_2575339, EPI_ISL_2575340, EPI_ISL_2575341, EPI_ISL_2575342, EPI_ISL_2575343, EPI_ISL_2575344, EPI_ISL_2575345, EPI_ISL_2575346, EPI_ISL_2575347, EPI_ISL_2575348			
see above	Department of Paediatric Infectious Diseases, Institute of Tropical Medicine, Nagasaki University	Department of Paediatric Infectious Diseases, Institute of Tropical Medicine, Nagasaki University	Yoshihara,K., Nhat Le,M., Nagasawa,K., Tsukagoshi,H., Nguyen,H.A., Toizumi,M., Moriuchi,H., Hashizume,M., Ariyoshi,K., Dang,D.A., Kimura,H. and Yoshida,L.-M.
EPI_ISL_2575349, EPI_ISL_2575350, EPI_ISL_2575351, EPI_ISL_2575352, EPI_ISL_2575353, EPI_ISL_2575354, EPI_ISL_2575355, EPI_ISL_2575356, EPI_ISL_2575357, EPI_ISL_2575358, EPI_ISL_2575359, EPI_ISL_2575360			
see above	Epidemiology and Demography, KEMRI - Wellcome Trust Research Programme, Centre for Geographic Medicine Research	Epidemiology and Demography, KEMRI - Wellcome Trust Research Programme, Centre for Geographic Medicine Research	Oketch,J.W., Kamau,E., Otieno,J.R., Mwema,A., Lewa,C., Agoti,C.N. and Nokes,J.D.
EPI_ISL_2578663, EPI_ISL_2578664, EPI_ISL_2578665, EPI_ISL_2578666, EPI_ISL_2578667, EPI_ISL_2578669, EPI_ISL_2578670, EPI_ISL_2578671, EPI_ISL_2578672	Epidemiology and Demography Department, KEMRI-Wellcome Trust Research Programme	Epidemiology and Demography Department, KEMRI-Wellcome Trust Research Programme	Otieno,J.R., Kamau,E.M., Oketch,J.W., Ngoi,J.M., Agoti,C.N., Gichuki,A.M., Otieno,G.P., Ngama,M., Cane,P.A., Kellam,P., Cotten,M., Lemey,P. and Nokes,D.J.
EPI_ISL_2578677, EPI_ISL_2578678	Center for Infectious Diseases, School of Public Health, University of Texas Health Science Center	Center for Infectious Diseases, School of Public Health, University of Texas Health Science Center	Bahl,J., Hixson,J., Kim,D.-K., Qiu,X., Piedra,P.A., Piedra,F.-A., Avadhanula,V. and Machado,A.A.
EPI_ISL_2578680, EPI_ISL_2578681, EPI_ISL_2578682, EPI_ISL_2578683, EPI_ISL_2578684, EPI_ISL_2578685, EPI_ISL_2578686, EPI_ISL_2578687, EPI_ISL_2578688, EPI_ISL_2578689	Epidemiology and Demography Department, KEMRI-Wellcome Trust Research Programme	Epidemiology and Demography Department, KEMRI-Wellcome Trust Research Programme	Otieno,J.R., Kamau,E.M., Oketch,J.W., Ngoi,J.M., Agoti,C.N., Gichuki,A.M., Otieno,G.P., Ngama,M., Cane,P.A., Kellam,P., Cotten,M., Lemey,P. and Nokes,D.J.
EPI_ISL_2578692	Center for Infectious Diseases, School of Public Health, University of Texas Health Science Center	Center for Infectious Diseases, School of Public Health, University of Texas Health Science Center	Bahl,J., Hixson,J., Kim,D.-K., Qiu,X., Piedra,P.A., Piedra,F.-A., Avadhanula,V. and Machado,A.A.
EPI_ISL_2578693, EPI_ISL_2578694, EPI_ISL_2578695, EPI_ISL_2578696, EPI_ISL_2578697, EPI_ISL_2578698, EPI_ISL_2578699, EPI_ISL_2578700, EPI_ISL_2578701, EPI_ISL_2578702, EPI_ISL_2578703, EPI_ISL_2578704, EPI_ISL_2578705, EPI_ISL_2578706, EPI_ISL_2578707, EPI_ISL_2578718, EPI_ISL_2578719, EPI_ISL_2578720, EPI_ISL_2578721, EPI_ISL_2578722, EPI_ISL_2578723, EPI_ISL_2578724, EPI_ISL_2578725, EPI_ISL_2578726, EPI_ISL_2578727, EPI_ISL_2578728			
see above	Epidemiology and Demography Department, KEMRI-Wellcome Trust Research Programme	Epidemiology and Demography Department, KEMRI-Wellcome Trust Research Programme	Otieno,J.R., Kamau,E.M., Oketch,J.W., Ngoi,J.M., Agoti,C.N., Gichuki,A.M., Otieno,G.P., Ngama,M., Cane,P.A., Kellam,P., Cotten,M., Lemey,P. and Nokes,D.J.

EPI_ISL_2578736, EPI_ISL_2578737	WHO WPRO Measles Regional Reference Lab, Key Laboratory of Medical Virology Ministry of Health, National Institute for Viral Disease Control and Prevention, China Center for Disease Control and Prevention	WHO WPRO Measles Regional Reference Lab, Key Laboratory of Medical Virology Ministry of Health, National Institute for Viral Disease Control and Prevention, China Center for Disease Control and Prevention	Song,J., Zhang,Y. and Xu,W.
EPI_ISL_2578740	Department of Clinical Laboratory, Fujian Provincial Hospital	Department of Clinical Laboratory, Fujian Provincial Hospital	Su,Y., Wu,Y., Tian,R. and Liang,G.
EPI_ISL_2578744, EPI_ISL_2578745, EPI_ISL_2578747, EPI_ISL_2578748, EPI_ISL_2578749, EPI_ISL_2578750, EPI_ISL_2578757, EPI_ISL_2578758	WHO WPRO Measles Regional Reference Lab, Key Laboratory of Medical Virology Ministry of Health, National Institute for Viral Disease Control and Prevention, China Center for Disease Control and Prevention	WHO WPRO Measles Regional Reference Lab, Key Laboratory of Medical Virology Ministry of Health, National Institute for Viral Disease Control and Prevention, China Center for Disease Control and Prevention	Song,J., Zhang,Y. and Xu,W.
EPI_ISL_2578766	Center for Medical Biotechnology, VIB-UGent	Center for Medical Biotechnology, VIB-UGent	Rossey,I., Schepens,B., Van Cauwenberghe,S., Saelens,X. and Melero,J.A.
EPI_ISL_2578769, EPI_ISL_2578770, EPI_ISL_2578771	Epidemiology and Demography Department, KEMRI-Wellcome Trust Research Programme	Epidemiology and Demography Department, KEMRI-Wellcome Trust Research Programme	Otieno,J.R., Kamau,E.M., Oketch,J.W., Ngoi,J.M., Agoti,C.N., Gichuki,A.M., Otieno,G.P., Ngama,M., Cane,P.A., Kellam,P., Cotten,M., Lemey,P. and Nokes,D.J.
EPI_ISL_2578787	Department of Pediatrics, Center of Excellence in Clinical Virology, Chulalongkorn	Department of Pediatrics, Center of Excellence in Clinical Virology, Chulalongkorn	Thongpan,I.
EPI_ISL_2578790, EPI_ISL_2578791, EPI_ISL_2578792, EPI_ISL_2578793, EPI_ISL_2578794, EPI_ISL_2578798	Centre de Recherche en Infectiologie, Universite Laval, CHUL	Centre de Recherche en Infectiologie, Universite Laval, CHUL	Papenburg,J., Carbonneau,J., Hamelin,M.-E., Isabel,S., Ohoumanne,N., Dery,P., Paes,B.A., Corbeil,J., Bergeron,M.G., De Serres,G. and Boivin,G.
EPI_ISL_2578799	Epidemiology and Demography Department, KEMRI-Wellcome Trust Research Programme	Epidemiology and Demography Department, KEMRI-Wellcome Trust Research Programme	Otieno,J.R., Kamau,E.M., Oketch,J.W., Ngoi,J.M., Agoti,C.N., Gichuki,A.M., Otieno,G.P., Ngama,M., Cane,P.A., Kellam,P., Cotten,M., Lemey,P. and Nokes,D.J.
EPI_ISL_2578809	Virology, Graduate School of Medicine, Tohoku University	Virology, Graduate School of Medicine, Tohoku University	Malasao,R., Furuse,Y., Okamoto,M., Dapat,C., Saito,M., Saito-Obata,M., Tamaki,R., Segubre-Mercado,E., Lupisan,S. and Oshitani,H.
EPI_ISL_2578817	Department of Pediatrics, Center of Excellence in Clinical Virology, Chulalongkorn	Department of Pediatrics, Center of Excellence in Clinical Virology, Chulalongkorn	Thongpan,I.
EPI_ISL_2578870, EPI_ISL_2578871, EPI_ISL_2578872, EPI_ISL_2578873, EPI_ISL_2578874, EPI_ISL_2578875, EPI_ISL_2578876, EPI_ISL_2578877, EPI_ISL_2578878, EPI_ISL_2578879	Department of Clinical Laboratory, Fujian Provincial Hospital	Department of Clinical Laboratory, Fujian Provincial Hospital	Su,Y., Wu,Y., Tian,R. and Liang,G.
EPI_ISL_2578892, EPI_ISL_2578893, EPI_ISL_2578894, EPI_ISL_2578895, EPI_ISL_2578896, EPI_ISL_2578897, EPI_ISL_2578898, EPI_ISL_2578899, EPI_ISL_2578900, EPI_ISL_2578901, EPI_ISL_2578902, EPI_ISL_2578903, EPI_ISL_2578904, EPI_ISL_2578905, EPI_ISL_2578906, EPI_ISL_2578907			
see above	Virology, Tropical Medicine Institute Pedro Kouri	Virology, Tropical Medicine Institute Pedro Kouri	Valdes,O., Corso,M., Pinon,A., Acosta,B., Savon,C., Gonzalez,G., Mune,M., Gonzalez,G., Hernandez,B., Echevarria,Y. and Oropesa,S.
EPI_ISL_2578909, EPI_ISL_2578910	Influenza Group, National Institute of Virology	Influenza Group, National Institute of Virology	Choudhary,M.L., Anand,S.P., Wadhwa,B.S. and Chadha,M.S.
EPI_ISL_2578966, EPI_ISL_2578970	Pediatrics, Mackay Memorial Hospital	Pediatrics, Mackay Memorial Hospital	Chi,H., Liu,H.F., Weng,L.C., Wang,N.Y., Chiang,C.L., Chiu,Y.Y., Huang,G.J., Cheng,Y.Y., Chiu,N.C., Hsieh,W.S. and Huang,L.M.
EPI_ISL_2578972, EPI_ISL_2578973, EPI_ISL_2578974, EPI_ISL_2578975, EPI_ISL_2578976, EPI_ISL_2578977, EPI_ISL_2578978, EPI_ISL_2578979, EPI_ISL_2578980, EPI_ISL_2578981, EPI_ISL_2578982, EPI_ISL_2578983, EPI_ISL_2578984, EPI_ISL_2578985, EPI_ISL_2578986, EPI_ISL_2578987, EPI_ISL_2578988, EPI_ISL_2578989, EPI_ISL_2578990, EPI_ISL_2578991			
see above	Investigacion en Virologia y Biotecnologia, Instituto Conmemorativo Gorgas de Estudios de la Salud	Investigacion en Virologia y Biotecnologia, Instituto Conmemorativo Gorgas de Estudios de la Salud	Abrego,L., Delfraro,A., Franco,D., Castillo,C., Cano,M., Castillo,M., Castillo,J., Pascale,J. and Arbiza,J.
EPI_ISL_2578994, EPI_ISL_2578995, EPI_ISL_2578996, EPI_ISL_2578997, EPI_ISL_2578998, EPI_ISL_2578999, EPI_ISL_2579000	Department of Clinical Laboratory, Fujian Provincial Hospital	Department of Clinical Laboratory, Fujian Provincial Hospital	Su,Y., Wu,Y., Tian,R. and Liang,G.
EPI_ISL_2579013	Influenza Group, National Institute of Virology	Influenza Group, National Institute of Virology	Choudhary,M.L., Anand,S.P., Wadhwa,B.S. and Chadha,M.S.
EPI_ISL_2579021	Center for Medical Biotechnology, VIB-UGent	Center for Medical Biotechnology, VIB-UGent	Rossey,I., Schepens,B., Van Cauwenberghe,S., Saelens,X. and Melero,J.A.
EPI_ISL_2579037, EPI_ISL_2579038	Centre de Recherche en Infectiologie, Universite Laval, CHUL	Centre de Recherche en Infectiologie, Universite Laval, CHUL	Papenburg,J., Carbonneau,J., Hamelin,M.-E., Isabel,S., Ohoumanne,N., Dery,P., Paes,B.A., Corbeil,J., Bergeron,M.G., De Serres,G. and Boivin,G.
EPI_ISL_2579041, EPI_ISL_2579042, EPI_ISL_2579043, EPI_ISL_2579044, EPI_ISL_2579045, EPI_ISL_2579046, EPI_ISL_2579047, EPI_ISL_2579048, EPI_ISL_2579049, EPI_ISL_2579050, EPI_ISL_2579051, EPI_ISL_2579052			
see above	Epidemiology and Demography, Kenya Medical Research Institute (KEMRI) - Wellcome Trust Research Programme	Epidemiology and Demography, Kenya Medical Research Institute (KEMRI) - Wellcome Trust Research Programme	Agoti,C.N., Otieno,J.R., Gitahi,C.W., Cane,P.A. and Nokes,D.J.
EPI_ISL_2579053	Epidemiology and Demography, KEMRI - Wellcome Trust Research Programme, Centre for Geographic Medicine Research	Epidemiology and Demography, KEMRI - Wellcome Trust Research Programme, Centre for Geographic Medicine Research	Oketch,J.W., Kamau,E., Otieno,J.R., Mwema,A., Lewa,C., Agoti,C.N. and Nokes,J.D.
EPI_ISL_2579056, EPI_ISL_2579057, EPI_ISL_2579058, EPI_ISL_2579059, EPI_ISL_2579060, EPI_ISL_2579061, EPI_ISL_2579062, EPI_ISL_2579063, EPI_ISL_2579064, EPI_ISL_2579065, EPI_ISL_2579066, EPI_ISL_2579067			
see above	Department of Epidemiology and Demography, Kenya Medical Research Institute (KEMRI) - Wellcome Trust Research Programme	Department of Epidemiology and Demography, Kenya Medical Research Institute (KEMRI) - Wellcome Trust Research Programme	Otieno,J.R., Agoti,C.N., Gitahi,C.W., Bett,A., Ngama,M., Medley,G.F., Cane,P.A. and Nokes,J.D.
EPI_ISL_2579162	Epidemiology and Demography, KEMRI - Wellcome Trust Research Programme, Centre for Geographic Medicine Research	Epidemiology and Demography, KEMRI - Wellcome Trust Research Programme, Centre for Geographic Medicine Research	Oketch,J.W., Kamau,E., Otieno,J.R., Mwema,A., Lewa,C., Agoti,C.N. and Nokes,J.D.
EPI_ISL_2579191, EPI_ISL_2579192, EPI_ISL_2579193	Pediatrics, Mackay Memorial Hospital	Pediatrics, Mackay Memorial Hospital	Chi,H., Hsiao,K.-L., Weng,L.-C., Chiu,N.-C., Huang,L.-M., Chiu,Y.-Y., Liu,C.-P. and Liu,H.-F.
EPI_ISL_2579217	Pediatrics, Show-Chwan Memorial Hospital	Pediatrics, Show-Chwan Memorial Hospital	Lee,C.-Y.
EPI_ISL_2579221	Department of Pediatrics, Center of Excellence in Clinical Virology, Faculty of Medicine, Chulalongkorn University	Department of Pediatrics, Center of Excellence in Clinical Virology, Faculty of Medicine, Chulalongkorn University	Thongpan,I., Mauleekoonphairoj,J., Vichiwattana,P., Korkong,S., Vongpunsawad,S. and Poovorawan,Y.
EPI_ISL_2579238, EPI_ISL_2579239, EPI_ISL_2579240, EPI_ISL_2579241, EPI_ISL_2579242, EPI_ISL_2579243, EPI_ISL_2579244, EPI_ISL_2579245, EPI_ISL_2579246, EPI_ISL_2579247, EPI_ISL_2579248			
see above	Department of Clinical Laboratory, Fujian Provincial Hospital	Department of Clinical Laboratory, Fujian Provincial Hospital	Su,Y., Wu,Y., Tian,R. and Liang,G.
EPI_ISL_2579257, EPI_ISL_2579258, EPI_ISL_2579259, EPI_ISL_2579260	WHO WPRO Measles Regional Reference Lab, Key Laboratory of Medical Virology Ministry of Health, National Institute for Viral Disease Control and Prevention, China Center for Disease Control and Prevention	WHO WPRO Measles Regional Reference Lab, Key Laboratory of Medical Virology Ministry of Health, National Institute for Viral Disease Control and Prevention, China Center for Disease Control and Prevention	Song,J., Zhang,Y. and Xu,W.
EPI_ISL_2579262	West China School of Public Health, Sichuan University	West China School of Public Health, Sichuan University	Zhang,M.
EPI_ISL_2579271, EPI_ISL_2579272	Centre de Recherche en Infectiologie, Universite Laval, CHUL	Centre de Recherche en Infectiologie, Universite Laval, CHUL	Papenburg,J., Carbonneau,J., Hamelin,M.-E., Isabel,S., Ohoumanne,N., Dery,P., Paes,B.A., Corbeil,J., Bergeron,M.G., De Serres,G. and Boivin,G.
EPI_ISL_2579297, EPI_ISL_2579298, EPI_ISL_2579299	Laboratory of Virology, Capital Institute of Pediatrics	Laboratory of Virology, Capital Institute of Pediatrics	Cui,G., Deng,J., Zhu,R., Qian,Y., Sun,Y., Zhao,L. and Wang,F.
EPI_ISL_2579308, EPI_ISL_2579309, EPI_ISL_2579310, EPI_ISL_2579311, EPI_ISL_2579312	Department of Clinical Laboratory, Fujian Provincial Hospital	Department of Clinical Laboratory, Fujian Provincial Hospital	Su,Y., Wu,Y., Tian,R. and Liang,G.
EPI_ISL_2579324, EPI_ISL_2579325, EPI_ISL_2579326	Virology, Tropical Medicine Institute Pedro Kouri	Virology, Tropical Medicine Institute Pedro Kouri	Valdes,O., Corso,M., Pinon,A., Acosta,B., Savon,C., Gonzalez,G., Mune,M., Gonzalez,G., Hernandez,B., Echevarria,Y. and Oropesa,S.
EPI_ISL_2579330	Influenza Group, National Institute of Virology	Influenza Group, National Institute of Virology	Choudhary,M.L., Anand,S.P., Wadhwa,B.S. and Chadha,M.S.
EPI_ISL_2579416	Investigacion en Virologia y Biotecnologia, Instituto Conmemorativo Gorgas de Estudios de la Salud	Investigacion en Virologia y Biotecnologia, Instituto Conmemorativo Gorgas de Estudios de la Salud	Abrego,L., Delfraro,A., Franco,D., Castillo,C., Cano,M., Castillo,M., Castillo,J., Pascale,J. and Arbiza,J.
EPI_ISL_2579417, EPI_ISL_2579418, EPI_ISL_2579419, EPI_ISL_2579420, EPI_ISL_2579421	Pediatric Clinic 1, Department of Pathophysiology and Transplantation, University of Milan and Fondazione IRCCS Ca Granda	Pediatric Clinic 1, Department of Pathophysiology and Transplantation, University of Milan and Fondazione IRCCS Ca Granda	Esposito,S., Zampiero,A., Piralla,A. and Principi,N.
EPI_ISL_2579457	Department of Respiratory Medicine, Children's Hospital of Chongqing Medical University	Department of Respiratory Medicine, Children's Hospital of Chongqing Medical University	Ren,L. and Liu,E.
EPI_ISL_2579458	WHO WPRO Measles Regional Reference Lab, Key Laboratory of Medical Virology Ministry of Health, National Institute for Viral Disease Control and Prevention, China Center for Disease Control and Prevention	WHO WPRO Measles Regional Reference Lab, Key Laboratory of Medical Virology Ministry of Health, National Institute for Viral Disease Control and Prevention, China Center for Disease Control and Prevention	Zhang,Y., Song,J. and Xu,W.
EPI_ISL_2579459, EPI_ISL_2579460	Laboratory Medicine and Pathobiology, University of Toronto	Laboratory Medicine and Pathobiology, University of Toronto	Granados,A., Duvvuri,V., Rosenfeld,P., Eshaghi,A. and Gubbay,J.B.
EPI_ISL_2579477	WHO WPRO Measles Regional Reference Lab, Key Laboratory of Medical Virology Ministry of Health, National Institute for Viral Disease Control and Prevention, China Center for Disease Control and Prevention	WHO WPRO Measles Regional Reference Lab, Key Laboratory of Medical Virology Ministry of Health, National Institute for Viral Disease Control and Prevention, China Center for Disease Control and Prevention	Zhang,Y., Song,J. and Xu,W.
EPI_ISL_2579480	Laboratory Medicine and Pathobiology, University of Toronto	Laboratory Medicine and Pathobiology, University of Toronto	Granados,A., Duvvuri,V., Rosenfeld,P., Eshaghi,A. and Gubbay,J.B.
EPI_ISL_2579487	Israel National Influenza Center, Central Virology Laboratory	Israel National Influenza Center, Central Virology Laboratory	Hirsh,S., MNorth America / USA,H. and Michal,M.
EPI_ISL_2579490	University of Wuerzburg, Institute of Virology and Immunobiology	University of Wuerzburg, Institute of Virology and Immunobiology	Prifert,C., Hofmann,D. and Weissbrich,B.
EPI_ISL_2579496, EPI_ISL_2579497	Department of Respiratory Medicine, Children's Hospital of Chongqing Medical University	Department of Respiratory Medicine, Children's Hospital of Chongqing Medical University	Ren,L. and Liu,E.
EPI_ISL_2579499, EPI_ISL_2579500	Chinese Academy of Medical Sciences & Peking Union Medical College	Chinese Academy of Medical Sciences & Peking Union Medical College	Jia,B., Xiao,Y., Wang,Y., Chen,L., Zhang,J., Ren,L. and Wang,J.
EPI_ISL_2579501, EPI_ISL_2579502, EPI_ISL_2579503, EPI_ISL_2579504, EPI_ISL_2579505, EPI_ISL_2579506, EPI_ISL_2579507, EPI_ISL_2579508, EPI_ISL_2579509, EPI_ISL_2579510, EPI_ISL_2579511, EPI_ISL_2579512, EPI_ISL_2579513, EPI_ISL_2579514, EPI_ISL_2579515, EPI_ISL_2579516, EPI_ISL_2579517, EPI_ISL_2579518, EPI_ISL_2579519, EPI_ISL_2579520, EPI_ISL_2579521, EPI_ISL_2579522, EPI_ISL_2579523, EPI_ISL_2579524, EPI_ISL_2579525, EPI_ISL_2579526, EPI_ISL_2579527, EPI_ISL_2579528, EPI_ISL_2579529, EPI_ISL_2579530, EPI_ISL_2579531, EPI_ISL_2579532			
see above	WHO WPRO Measles Regional Reference Lab, Key Laboratory of Medical Virology Ministry of Health, National Institute for Viral Disease Control and Prevention, China Center for Disease Control and Prevention	WHO WPRO Measles Regional Reference Lab, Key Laboratory of Medical Virology Ministry of Health, National Institute for Viral Disease Control and Prevention, China Center for Disease Control and Prevention	Zhang,Y., Song,J. and Xu,W.
EPI_ISL_2579533, EPI_ISL_2579534	Department of Paediatric Infectious Diseases, Institute of Tropical Medicine, Nagasaki University	Department of Paediatric Infectious Diseases, Institute of Tropical Medicine, Nagasaki University	Yoshihara,K., Nhat Le,M., Nagasawa,K., Tsukagoshi,H., Nguyen,H.A., Toizumi,M., Moriuchi,H., Hashizume,M., Ariyoshi,K., Dang,D.A., Kimura,H. and Yoshida,L.-M.
EPI_ISL_2579563	Chinese Academy of Medical Sciences & Peking Union Medical College	Chinese Academy of Medical Sciences & Peking Union Medical College	Jia,B., Xiao,Y., Wang,Y., Chen,L., Zhang,J., Ren,L. and Wang,J.
EPI_ISL_2579564, EPI_ISL_2579565, EPI_ISL_2579566, EPI_ISL_2579567, EPI_ISL_2579568, EPI_ISL_2579569, EPI_ISL_2579570, EPI_ISL_2579571, EPI_ISL_2579572, EPI_ISL_2579573, EPI_ISL_2579574, EPI_ISL_2579575, EPI_ISL_2579585, EPI_ISL_2579586			

see above	WHO WPRO Measles Regional Reference Lab, Key Laboratory of Medical Virology Ministry of Health, National Institute for Viral Disease Control and Prevention, China Center for Disease Control and Prevention	WHO WPRO Measles Regional Reference Lab, Key Laboratory of Medical Virology Ministry of Health, National Institute for Viral Disease Control and Prevention, China Center for Disease Control and Prevention	Zhang,Y., Song,J. and Xu,W.
EPI_ISL_2579599, EPI_ISL_2579681	Department of Respiratory Medicine, Children's Hospital of Chongqing Medical University	Department of Respiratory Medicine, Children's Hospital of Chongqing Medical University	Ren,L. and Liu,E.
EPI_ISL_2579682, EPI_ISL_2579683	Chinese Academy of Medical Sciences & Peking Union Medical College	Chinese Academy of Medical Sciences & Peking Union Medical College	Jia,B., Xiao,Y., Wang,Y., Chen,L., Zhang,J., Ren,L. and Wang,J.
EPI_ISL_2579689, EPI_ISL_2579693	WHO WPRO Measles Regional Reference Lab, Key Laboratory of Medical Virology Ministry of Health, National Institute for Viral Disease Control and Prevention, China Center for Disease Control and Prevention	WHO WPRO Measles Regional Reference Lab, Key Laboratory of Medical Virology Ministry of Health, National Institute for Viral Disease Control and Prevention, China Center for Disease Control and Prevention	Zhang,Y., Song,J. and Xu,W.
EPI_ISL_2579752, EPI_ISL_2579753	Department of Respiratory Medicine, Children's Hospital of Chongqing Medical University	Department of Respiratory Medicine, Children's Hospital of Chongqing Medical University	Ren,L. and Liu,E.
EPI_ISL_2579754	Minoru Nidaira Okinawa Prefectural Institute of Health and Environment, Department of Biological Science	Minoru Nidaira Okinawa Prefectural Institute of Health and Environment, Department of Biological Science	Nidaira,M. and Taira,K.
EPI_ISL_2579760, EPI_ISL_2579761	Laboratory Medicine and Pathobiology, University of Toronto	Laboratory Medicine and Pathobiology, University of Toronto	Granados,A., Duvvuri,V., Rosenfeld,P., Eshaghi,A. and Gubbay,J.B.
EPI_ISL_2579775	Manipal Centre for Virus Research, Manipal University	Manipal Centre for Virus Research, Manipal University	Anjali,A., Aswathy,R., Akhil,C., Giselle,D., Revti,B., Hindol,M., Suresh,P. and Arunkumar,G.
EPI_ISL_2579787, EPI_ISL_2579791, EPI_ISL_2579792, EPI_ISL_2579793, EPI_ISL_2579794, EPI_ISL_2579795, EPI_ISL_2579796, EPI_ISL_2579797, EPI_ISL_2579799, EPI_ISL_2579800, EPI_ISL_2579801, EPI_ISL_2579802, EPI_ISL_2579803, EPI_ISL_2579804, EPI_ISL_2579805, EPI_ISL_2579806, EPI_ISL_2579807, EPI_ISL_2579808, EPI_ISL_2579809, EPI_ISL_2579810, EPI_ISL_2579811, EPI_ISL_2579812, EPI_ISL_2579814, EPI_ISL_2579815, EPI_ISL_2579817, EPI_ISL_2579819, EPI_ISL_2579820, EPI_ISL_2579823, EPI_ISL_2579839, EPI_ISL_2579845, EPI_ISL_2579846, EPI_ISL_2579847, EPI_ISL_2579848			
see above	Centre for Research And Knowledge Transfer in Biotechnology, University of Zagreb	Centre for Research And Knowledge Transfer in Biotechnology, University of Zagreb	Ivancic-Jelecki,J., Slovic,A., Ljubin-Sternak,S., Mlinaric-Galinovic,G. and Forcic,D.
EPI_ISL_2579857	Influenza Group, National Institute of Virology	Influenza Group, National Institute of Virology	Choudhary,M.L., Wadhwa,B., Jadhav,S.M., Chadha,M.S. and Mourya,D.T.
EPI_ISL_2579898, EPI_ISL_2579899	Influenza Group, National Institute of Virology	Influenza Group, National Institute of Virology	Choudhary,M.L., Anand,S.P., Wadhwa,B.S. and Chadha,M.S.
EPI_ISL_2579907	Pediatric Clinic 1, Department of Pathophysiology and Transplantation, University of Milan and Fondazione IRCCS Ca Granda	Pediatric Clinic 1, Department of Pathophysiology and Transplantation, University of Milan and Fondazione IRCCS Ca Granda	Esposito,S., Zampiero,A., Piralla,A. and Principi,N.
EPI_ISL_2579917, EPI_ISL_2579918, EPI_ISL_2579920	Virology, Tohoku University Graduate School of Medicine	Virology, Tohoku University Graduate School of Medicine	Malasao,R., Okamoto,M., Chaimongkol,N., Imamura,T., Tohma,K., Dapat,I., Dapat,C., Suzuki,A., Saito,M., Saito,M., Tamaki,R., Segubre-Mercado,E., Igoy,M.A.U., Lupisan,S., Olveda,R. and Oshitani,H.
EPI_ISL_2579937, EPI_ISL_2579938	Department of Infectious Diseases, National Health Institute Doutor Ricardo Jorge	Department of Infectious Diseases, National Health Institute Doutor Ricardo Jorge	Saez-Lopez,E., Cristovao,P., Costa,I., Pechirra,P., Conde,P. and Guiomar,R.
EPI_ISL_2579970, EPI_ISL_2579971, EPI_ISL_2579973, EPI_ISL_2579975, EPI_ISL_2579976, EPI_ISL_2579978, EPI_ISL_2579980, EPI_ISL_2579982, EPI_ISL_2579983, EPI_ISL_2579985, EPI_ISL_2579987, EPI_ISL_2579989, EPI_ISL_2579990, EPI_ISL_2579992, EPI_ISL_2579994, EPI_ISL_2579995, EPI_ISL_2579997, EPI_ISL_2579999, EPI_ISL_2580001, EPI_ISL_2580002, EPI_ISL_2580004, EPI_ISL_2580006, EPI_ISL_2580007, EPI_ISL_2580009, EPI_ISL_2580011, EPI_ISL_2580013, EPI_ISL_2580014, EPI_ISL_2580016, EPI_ISL_2580018			
see above	Department of Paediatric Infectious Diseases, Institute of Tropical Medicine, Nagasaki University	Department of Paediatric Infectious Diseases, Institute of Tropical Medicine, Nagasaki University	Yoshihara,K., Nhat Le,M., Nagasawa,K., Tsukagoshi,H., Nguyen,H.A., Toizumi,M., Moriuchi,H., Hashizume,M., Ariyoshi,K., Dang,D.A., Kimura,H. and Yoshida,L.-M.
EPI_ISL_2580020	School of Medicine, University of Washington	School of Medicine, University of Washington	Scott,E.M., Magaret,A., Kuypers,J., Stewart,L., Shrestha,L., Tielsch,J.M., Steinhoff,M., Katz,J., Khatry,S.K., LeClerq,S.C., Englund,J.A. and Chu,H.Y.
EPI_ISL_2580021, EPI_ISL_2580023	Israel National Influenza Center, Central Virology Laboratory	Israel National Influenza Center, Central Virology Laboratory	Hirsh,S., MNorth America / USA,H. and Michal,M.
EPI_ISL_2580025	WHO WPRO Measles Regional Reference Lab, Key Laboratory of Medical Virology Ministry of Health, National Institute for Viral Disease Control and Prevention, China Center for Disease Control and Prevention	WHO WPRO Measles Regional Reference Lab, Key Laboratory of Medical Virology Ministry of Health, National Institute for Viral Disease Control and Prevention, China Center for Disease Control and Prevention	Zhang,Y., Song,J. and Xu,W.
EPI_ISL_2580030	School of Medicine, University of Washington	School of Medicine, University of Washington	Scott,E.M., Magaret,A., Kuypers,J., Stewart,L., Shrestha,L., Tielsch,J.M., Steinhoff,M., Katz,J., Khatry,S.K., LeClerq,S.C., Englund,J.A. and Chu,H.Y.
EPI_ISL_2580033, EPI_ISL_2580035, EPI_ISL_2580037, EPI_ISL_2580039, EPI_ISL_2580041, EPI_ISL_2580042, EPI_ISL_2580044, EPI_ISL_2580046, EPI_ISL_2580048, EPI_ISL_2580049, EPI_ISL_2580051, EPI_ISL_2580053			
see above	Department of Respiratory Medicine, Children's Hospital of Chongqing Medical University	Department of Respiratory Medicine, Children's Hospital of Chongqing Medical University	Ren,L. and Liu,E.
EPI_ISL_2580071, EPI_ISL_2580074	School of Medicine, University of Washington	School of Medicine, University of Washington	Scott,E.M., Magaret,A., Kuypers,J., Stewart,L., Shrestha,L., Tielsch,J.M., Steinhoff,M., Katz,J., Khatry,S.K., LeClerq,S.C., Englund,J.A. and Chu,H.Y.
EPI_ISL_2580099, EPI_ISL_2580100, EPI_ISL_2580115, EPI_ISL_2580117, EPI_ISL_2580118, EPI_ISL_2580120, EPI_ISL_2580121, EPI_ISL_2580123, EPI_ISL_2580125, EPI_ISL_2580128, EPI_ISL_2580130, EPI_ISL_2580132, EPI_ISL_2580134, EPI_ISL_2580135, EPI_ISL_2580137, EPI_ISL_2580139, EPI_ISL_2580140, EPI_ISL_2580142, EPI_ISL_2580144, EPI_ISL_2580146, EPI_ISL_2580147, EPI_ISL_2580149, EPI_ISL_2580151, EPI_ISL_2580153, EPI_ISL_2580155, EPI_ISL_2580156, EPI_ISL_2580158			
see above	Chinese Academy of Medical Sciences & Peking Union Medical College	Chinese Academy of Medical Sciences & Peking Union Medical College	Jia,B., Xiao,Y., Wang,Y., Chen,L., Zhang,J., Ren,L. and Wang,J.
EPI_ISL_2580220, EPI_ISL_2580222	WHO WPRO Measles Regional Reference Lab, Key Laboratory of Medical Virology Ministry of Health, National Institute for Viral Disease Control and Prevention, China Center for Disease Control and Prevention	WHO WPRO Measles Regional Reference Lab, Key Laboratory of Medical Virology Ministry of Health, National Institute for Viral Disease Control and Prevention, China Center for Disease Control and Prevention	Zhang,Y., Song,J. and Xu,W.
EPI_ISL_2580230, EPI_ISL_2580232	Department of Public Health Laboratory Sciences, West China School of Public Health, Sichuan University	Department of Public Health Laboratory Sciences, West China School of Public Health, Sichuan University	Hu,W.P. and Pei,F.X.
EPI_ISL_2580249	Division of Immunology, Children's Hospital	Division of Immunology, Children's Hospital	Qin,X., Zhang,C., Zhao,Y. and Zhao,X.
EPI_ISL_2580251, EPI_ISL_2580252, EPI_ISL_2580254	WHO WPRO Measles Regional Reference Lab, Key Laboratory of Medical Virology Ministry of Health, National Institute for Viral Disease Control and Prevention, China Center for Disease Control and Prevention	WHO WPRO Measles Regional Reference Lab, Key Laboratory of Medical Virology Ministry of Health, National Institute for Viral Disease Control and Prevention, China Center for Disease Control and Prevention	Zhang,Y., Song,J. and Xu,W.
EPI_ISL_2580256	Chinese Academy of Medical Sciences & Peking Union Medical College	Chinese Academy of Medical Sciences & Peking Union Medical College	Jia,B., Xiao,Y., Wang,Y., Chen,L., Zhang,J., Ren,L. and Wang,J.
EPI_ISL_2580258, EPI_ISL_2580259, EPI_ISL_2580261, EPI_ISL_2580263, EPI_ISL_2580265	WHO WPRO Measles Regional Reference Lab, Key Laboratory of Medical Virology Ministry of Health, National Institute for Viral Disease Control and Prevention, China Center for Disease Control and Prevention	WHO WPRO Measles Regional Reference Lab, Key Laboratory of Medical Virology Ministry of Health, National Institute for Viral Disease Control and Prevention, China Center for Disease Control and Prevention	Zhang,Y., Song,J. and Xu,W.
EPI_ISL_2580271, EPI_ISL_2580273, EPI_ISL_2580276, EPI_ISL_2580278, EPI_ISL_2580280	University of Wuerzburg, Institute of Virology and Immunobiology	University of Wuerzburg, Institute of Virology and Immunobiology	Prifert,C., Hofmann,D. and Weissbrich,B.
EPI_ISL_2580290	Virology Division, Public Health Laboratory Services Branch, Centre for Health Protection, Department of Health	Virology Division, Public Health Laboratory Services Branch, Centre for Health Protection, Department of Health	Mak,G.C., Lau,C.S., Chiu,D.M.Y. and Lim,W.
EPI_ISL_2580291, EPI_ISL_2580293, EPI_ISL_2580295, EPI_ISL_2580296, EPI_ISL_2580299, EPI_ISL_2580300, EPI_ISL_2580302	Virology, School of Public Health, Tehran University of Medical Sciences	Virology, School of Public Health, Tehran University of Medical Sciences	Yavarian,J., Faghihloo,E. and Mokhtari Azad,T.
EPI_ISL_2580304, EPI_ISL_2580305, EPI_ISL_2580307, EPI_ISL_2580309, EPI_ISL_2580310, EPI_ISL_2580312, EPI_ISL_2580314, EPI_ISL_2580316, EPI_ISL_2580317, EPI_ISL_2580319, EPI_ISL_2580321, EPI_ISL_2580323, EPI_ISL_2580324, EPI_ISL_2580326, EPI_ISL_2580327, EPI_ISL_2580329, EPI_ISL_2580331, EPI_ISL_2580333, EPI_ISL_2580334, EPI_ISL_2580336, EPI_ISL_2580338			
see above	Pediatric Clinic 1, Department of Pathophysiology and Transplantation, University of Milan and Fondazione IRCCS Ca Granda	Pediatric Clinic 1, Department of Pathophysiology and Transplantation, University of Milan and Fondazione IRCCS Ca Granda	Esposito,S., Zampiero,A., Piralla,A. and Principi,N.
EPI_ISL_2580340, EPI_ISL_2580342	Virology, National Institute of Health-Pakistan, Park Road, Chak Shahzad, Islamabad	Virology, National Institute of Health-Pakistan, Park Road, Chak Shahzad, Islamabad	Aamir,U.B., Alam,M.M., Sadia,H. and Zaidi,S.S.Z.
EPI_ISL_2580350, EPI_ISL_2580352, EPI_ISL_2580354	Department of Paediatric Infectious Diseases, Institute of Tropical Medicine, Nagasaki University	Department of Paediatric Infectious Diseases, Institute of Tropical Medicine, Nagasaki University	Yoshihara,K., Nhat Le,M., Nagasawa,K., Tsukagoshi,H., Nguyen,H.A., Toizumi,M., Moriuchi,H., Hashizume,M., Ariyoshi,K., Dang,D.A., Kimura,H. and Yoshida,L.-M.
EPI_ISL_2580360	WHO WPRO Measles Regional Reference Lab, Key Laboratory of Medical Virology Ministry of Health, National Institute for Viral Disease Control and Prevention, China Center for Disease Control and Prevention	WHO WPRO Measles Regional Reference Lab, Key Laboratory of Medical Virology Ministry of Health, National Institute for Viral Disease Control and Prevention, China Center for Disease Control and Prevention	Zhang,Y., Song,J. and Xu,W.
EPI_ISL_2580363	Israel National Influenza Center, Central Virology Laboratory	Israel National Influenza Center, Central Virology Laboratory	Hirsh,S., MNorth America / USA,H. and Michal,M.
EPI_ISL_2580365, EPI_ISL_2580366, EPI_ISL_2580368, EPI_ISL_2580369, EPI_ISL_2580370, EPI_ISL_2580372, EPI_ISL_2580373, EPI_ISL_2580375, EPI_ISL_2580377, EPI_ISL_2580378	Department of Respiratory Medicine, Children's Hospital of Chongqing Medical University	Department of Respiratory Medicine, Children's Hospital of Chongqing Medical University	Ren,L. and Liu,E.
EPI_ISL_2580380, EPI_ISL_2580382, EPI_ISL_2580384	Akinobu Hibino Niigata University, International Health, Public Health	Akinobu Hibino Niigata University, International Health, Public Health	Hibino,A., Saito,R., Shoubugawa,Y. and Seo,T.
EPI_ISL_2580386	Akinobu Hibino Niigata University, International Health, Public Health	Akinobu Hibino Niigata University, International Health, Public Health	Hibino,A., Saito,R., Shoubugawa,Y. and Tomimoto,K.
EPI_ISL_2580396, EPI_ISL_2580398	Akinobu Hibino Niigata University, International Health, Public Health	Akinobu Hibino Niigata University, International Health, Public Health	Hibino,A., Saito,R., Shoubugawa,Y. and Saito,T.
EPI_ISL_2580403, EPI_ISL_2580405, EPI_ISL_2580407, EPI_ISL_2580413, EPI_ISL_2580415, EPI_ISL_2580416, EPI_ISL_2580418, EPI_ISL_2580420, EPI_ISL_2580422, EPI_ISL_2580423, EPI_ISL_2580427, EPI_ISL_2580429, EPI_ISL_2580430, EPI_ISL_2580432, EPI_ISL_2580434, EPI_ISL_2580436, EPI_ISL_2580438, EPI_ISL_2580440, EPI_ISL_2580442, EPI_ISL_2580443			
see above	Eiko Hirano Fukui Prefectural Institute of Public Health and Environmental Science	Eiko Hirano Fukui Prefectural Institute of Public Health and Environmental Science	Hirano,E. and Kobayashi,M.
EPI_ISL_2580445, EPI_ISL_2580447, EPI_ISL_2580449	Akinobu Hibino Niigata University, International Health, Public Health	Akinobu Hibino Niigata University, International Health, Public Health	Hibino,A., Saito,R., Shoubugawa,Y. and Sugimura,T.
EPI_ISL_2580452, EPI_ISL_2580454	Akinobu Hibino Niigata University, International Health, Public Health	Akinobu Hibino Niigata University, International Health, Public Health	Hibino,A., Saito,R., Shoubugawa,Y. and Nakamura,Y.
EPI_ISL_2580456, EPI_ISL_2580457, EPI_ISL_2580459, EPI_ISL_2580461, EPI_ISL_2580462	Akinobu Hibino Niigata University, International Health, Public Health	Akinobu Hibino Niigata University, International Health, Public Health	Hibino,A., Saito,R., Shoubugawa,Y. and Shirakawa,K.
EPI_ISL_2580464	Akinobu Hibino Niigata University, International Health, Public Health	Akinobu Hibino Niigata University, International Health, Public Health	Hibino,A., Saito,R., Shoubugawa,Y. and Takei,T.
EPI_ISL_2580490, EPI_ISL_2580492, EPI_ISL_2580493, EPI_ISL_2580495	Akinobu Hibino Niigata University, International Health, Public Health	Akinobu Hibino Niigata University, International Health, Public Health	Hibino,A., Saito,R., Shoubugawa,Y. and Ikezawa,S.
EPI_ISL_2580504, EPI_ISL_2580506, EPI_ISL_2580508, EPI_ISL_2580510, EPI_ISL_2580512, EPI_ISL_2580514, EPI_ISL_2580515, EPI_ISL_2580517, EPI_ISL_2580519, EPI_ISL_2580521, EPI_ISL_2580523, EPI_ISL_2580525, EPI_ISL_2580526, EPI_ISL_2580528, EPI_ISL_2580530, EPI_ISL_2580531, EPI_ISL_2580533, EPI_ISL_2580535, EPI_ISL_2580537, EPI_ISL_2580539, EPI_ISL_2580541, EPI_ISL_2580543, EPI_ISL_2580545, EPI_ISL_2580547, EPI_ISL_2580548, EPI_ISL_2580551, EPI_ISL_2580553, EPI_ISL_2580555, EPI_ISL_2580557, EPI_ISL_2580559, EPI_ISL_2580560, EPI_ISL_2580562, EPI_ISL_2580563			

see above	Akinobu Hibino Niigata University, International Health, Public Health	Akinobu Hibino Niigata University, International Health, Public Health	Hibino,A., Saito,R., Shoubugawa,Y. and Sano,Y.
EPI_ISL_2580647, EPI_ISL_2580649, EPI_ISL_2580652, EPI_ISL_2580653, EPI_ISL_2580655	Akinobu Hibino Niigata University, International Health, Public Health	Akinobu Hibino Niigata University, International Health, Public Health	Hibino,A., Saito,R., Shoubugawa,Y. and Kaji,H.
EPI_ISL_2580701, EPI_ISL_2580703, EPI_ISL_2580704, EPI_ISL_2580707, EPI_ISL_2580709, EPI_ISL_2580711, EPI_ISL_2580713, EPI_ISL_2580715, EPI_ISL_2580717, EPI_ISL_2580719, EPI_ISL_2580721, EPI_ISL_2580723, EPI_ISL_2580725, EPI_ISL_2580727, EPI_ISL_2580729, EPI_ISL_2580731, EPI_ISL_2580733, EPI_ISL_2580737, EPI_ISL_2580739, EPI_ISL_2580741, EPI_ISL_2580743, EPI_ISL_2580745, EPI_ISL_2580747, EPI_ISL_2580749, EPI_ISL_2580751, EPI_ISL_2580753, EPI_ISL_2580755, EPI_ISL_2580757, EPI_ISL_2580759, EPI_ISL_2580761, EPI_ISL_2580763, EPI_ISL_2580766, EPI_ISL_2580767, EPI_ISL_2580769, EPI_ISL_2580772, EPI_ISL_2580773, EPI_ISL_2580775, EPI_ISL_2580777, EPI_ISL_2580779, EPI_ISL_2580781, EPI_ISL_2580785, EPI_ISL_2580787, EPI_ISL_2580789, EPI_ISL_2580791, EPI_ISL_2580793, EPI_ISL_2580796, EPI_ISL_2580797, EPI_ISL_2580799, EPI_ISL_2580801, EPI_ISL_2580803, EPI_ISL_2580805			
see above	Chinese Academy of Medical Sciences & Peking Union Medical College	Chinese Academy of Medical Sciences & Peking Union Medical College	Jia,B., Xiao,Y., Wang,Y., Chen,L., Zhang,J., Ren,L. and Wang,J.
EPI_ISL_2580831	West China School of Public Health, Sichuan University	West China School of Public Health, Sichuan University	Zhang,M.
EPI_ISL_2580836, EPI_ISL_2580837, EPI_ISL_2580839	WHO WPRO Measles Regional Reference Lab, Key Laboratory of Medical Virology Ministry of Health, National Institute for Viral Disease Control and Prevention, China Center for Disease Control and Prevention	WHO WPRO Measles Regional Reference Lab, Key Laboratory of Medical Virology Ministry of Health, National Institute for Viral Disease Control and Prevention, China Center for Disease Control and Prevention	Zhang,Y., Song,J. and Xu,W.
EPI_ISL_2580857, EPI_ISL_2580859, EPI_ISL_2580861	Department of Public Health Laboratory Sciences, West China School of Public Health, Sichuan University	Department of Public Health Laboratory Sciences, West China School of Public Health, Sichuan University	Hu,W.P. and Pei,F.X.
EPI_ISL_2580869, EPI_ISL_2580871, EPI_ISL_2580875, EPI_ISL_2580877, EPI_ISL_2580879	WHO WPRO Measles Regional Reference Lab, Key Laboratory of Medical Virology Ministry of Health, National Institute for Viral Disease Control and Prevention, China Center for Disease Control and Prevention	WHO WPRO Measles Regional Reference Lab, Key Laboratory of Medical Virology Ministry of Health, National Institute for Viral Disease Control and Prevention, China Center for Disease Control and Prevention	Zhang,Y., Song,J. and Xu,W.
EPI_ISL_2580885, EPI_ISL_2580889, EPI_ISL_2580895, EPI_ISL_2580897, EPI_ISL_2580901	University of Wuerzburg, Institute of Virology and Immunobiology	University of Wuerzburg, Institute of Virology and Immunobiology	Prifert,C., Hofmann,D. and Weissbrich,B.
EPI_ISL_2580903, EPI_ISL_2580906, EPI_ISL_2580907	Virology Division, Public Health Laboratory Services Branch, Centre for Health Protection, Department of Health	Virology Division, Public Health Laboratory Services Branch, Centre for Health Protection, Department of Health	Mak,G.C., Lau,C.S., Chiu,D.M.Y. and Lim,W.
EPI_ISL_2580922, EPI_ISL_2580924, EPI_ISL_2580928, EPI_ISL_2580929, EPI_ISL_2580931, EPI_ISL_2580933, EPI_ISL_2580935	Virology, School of Public Health, Tehran University of Medical Sciences	Virology, School of Public Health, Tehran University of Medical Sciences	Yavarian,J., Faghihloo,E. and Mokhtari Azad,T.
EPI_ISL_2580938, EPI_ISL_2580939, EPI_ISL_2580941, EPI_ISL_2580943, EPI_ISL_2580944	Pediatric Clinic 1, Department of Pathophysiology and Transplantation, University of Milan and Fondazione IRCCS Ca Granda	Pediatric Clinic 1, Department of Pathophysiology and Transplantation, University of Milan and Fondazione IRCCS Ca Granda	Esposito,S., Zampiero,A., Piralla,A. and Principi,N.
EPI_ISL_2580949	Virology, Tohoku University Graduate School of Medicine	Virology, Tohoku University Graduate School of Medicine	Malasao,R., Okamoto,M., Chaimongkol,N., Imamura,T., Tohma,K., Dapat,I., Dapat,C., Suzuki,A., Saito,M., Saito,M., Tamaki,R., Segubre-Mercado,E., Igoy,M.A.U., Lupisan,S., Olveda,R. and Oshitani,H.
EPI_ISL_2580953, EPI_ISL_2580954, EPI_ISL_2580955, EPI_ISL_2580956, EPI_ISL_2580957, EPI_ISL_2580958, EPI_ISL_2580959, EPI_ISL_2580960, EPI_ISL_2580961, EPI_ISL_2580962, EPI_ISL_2580963, EPI_ISL_2580964, EPI_ISL_2580965, EPI_ISL_2580966, EPI_ISL_2580967, EPI_ISL_2580968, EPI_ISL_2580969, EPI_ISL_2580970, EPI_ISL_2580972, EPI_ISL_2580973, EPI_ISL_2580974, EPI_ISL_2580975, EPI_ISL_2580976, EPI_ISL_2580977, EPI_ISL_2580978, EPI_ISL_2580979, EPI_ISL_2580980, EPI_ISL_2580981, EPI_ISL_2580982, EPI_ISL_2580983, EPI_ISL_2580984, EPI_ISL_2580985, EPI_ISL_2580986, EPI_ISL_2580987, EPI_ISL_2580989, EPI_ISL_2580990, EPI_ISL_2580992, EPI_ISL_2580993, EPI_ISL_2580994, EPI_ISL_2580995, EPI_ISL_2580996, EPI_ISL_2580998, EPI_ISL_2580999, EPI_ISL_2581000, EPI_ISL_2581001, EPI_ISL_2581003, EPI_ISL_2581004, EPI_ISL_2581005, EPI_ISL_2581006, EPI_ISL_2581007, EPI_ISL_2581008, EPI_ISL_2581009, EPI_ISL_2581010, EPI_ISL_2581012, EPI_ISL_2581013, EPI_ISL_2581014, EPI_ISL_2581015, EPI_ISL_2581016			
see above	Department of Paediatric Infectious Diseases, Institute of Tropical Medicine, Nagasaki University	Department of Paediatric Infectious Diseases, Institute of Tropical Medicine, Nagasaki University	Yoshihara,K., Nhat Le,M., Nagasawa,K., Tsukagoshi,H., Nguyen,H.A., Toizumi,M., Moriuchi,H., Hashizume,M., Ariyoshi,K., Dang,D.A., Kimura,H. and Yoshida,L.-M.
EPI_ISL_2581026	School of Medicine, University of Washington	School of Medicine, University of Washington	Scott,E.M., Magaret,A., Kuypers,J., Stewart,L., Shrestha,L., Tielsch,J.M., Steinhoff,M., Katz,J., Khatry,S.K., LeClerq,S.C., Englund,J.A. and Chu,H.Y.
EPI_ISL_2581027	Virology, National Institute of Health-Pakistan, Park Road, Chak Shahzad, Islamabad	Virology, National Institute of Health-Pakistan, Park Road, Chak Shahzad, Islamabad	Aamir,U.B., Alam,M.M., Sadia,H. and Zaidi,S.S.Z.
EPI_ISL_2581029	Israel National Influenza Center, Central Virology Laboratory	Israel National Influenza Center, Central Virology Laboratory	Hirsh,S., MNorth America / USA,H. and Michal,M.
EPI_ISL_2581038	Department of Respiratory Medicine, Children's Hospital of Chongqing Medical University	Department of Respiratory Medicine, Children's Hospital of Chongqing Medical University	Ren,L. and Liu,E.
EPI_ISL_2581039	School of Medicine, University of Washington	School of Medicine, University of Washington	Scott,E.M., Magaret,A., Kuypers,J., Stewart,L., Shrestha,L., Tielsch,J.M., Steinhoff,M., Katz,J., Khatry,S.K., LeClerq,S.C., Englund,J.A. and Chu,H.Y.
EPI_ISL_2581040	Akinobu Hibino Niigata University, International Health, Public Health	Akinobu Hibino Niigata University, International Health, Public Health	Hibino,A., Saito,R., Shoubugawa,Y. and Seo,T.
EPI_ISL_2581061, EPI_ISL_2581064, EPI_ISL_2581095, EPI_ISL_2581097, EPI_ISL_2581100, EPI_ISL_2581103, EPI_ISL_2581111, EPI_ISL_2581114	Eiko Hirano Fukui Prefectural Institute of Public Health and Environmental Science	Eiko Hirano Fukui Prefectural Institute of Public Health and Environmental Science	Hirano,E. and Kobayashi,M.
EPI_ISL_2581133, EPI_ISL_2581135, EPI_ISL_2581138	Akinobu Hibino Niigata University, International Health, Public Health	Akinobu Hibino Niigata University, International Health, Public Health	Hibino,A., Saito,R., Shoubugawa,Y. and Nakamura,Y.
EPI_ISL_2581139	Akinobu Hibino Niigata University, International Health, Public Health	Akinobu Hibino Niigata University, International Health, Public Health	Hibino,A., Saito,R., Shoubugawa,Y. and Shirakawa,K.
EPI_ISL_2581141	Akinobu Hibino Niigata University, International Health, Public Health	Akinobu Hibino Niigata University, International Health, Public Health	Hibino,A., Saito,R., Shoubugawa,Y. and Ikezawa,S.
EPI_ISL_2581143, EPI_ISL_2581146, EPI_ISL_2581147, EPI_ISL_2581149, EPI_ISL_2581151, EPI_ISL_2581153, EPI_ISL_2581156, EPI_ISL_2581157, EPI_ISL_2581160, EPI_ISL_2581161, EPI_ISL_2581164, EPI_ISL_2581165, EPI_ISL_2581167, EPI_ISL_2581169, EPI_ISL_2581171, EPI_ISL_2581173, EPI_ISL_2581175, EPI_ISL_2581177, EPI_ISL_2581179			
see above	Akinobu Hibino Niigata University, International Health, Public Health	Akinobu Hibino Niigata University, International Health, Public Health	Hibino,A., Saito,R., Shoubugawa,Y. and Sano,Y.
EPI_ISL_2581194	Virology, National Institute of Health-Pakistan, Park Road, Chak Shahzad, Islamabad	Virology, National Institute of Health-Pakistan, Park Road, Chak Shahzad, Islamabad	Aamir,U.B., Alam,M.M., Sadia,H. and Zaidi,S.S.Z.
EPI_ISL_2581201, EPI_ISL_2581202, EPI_ISL_2581204, EPI_ISL_2581205, EPI_ISL_2581208, EPI_ISL_2581209, EPI_ISL_2581211	Chinese Academy of Medical Sciences & Peking Union Medical College	Chinese Academy of Medical Sciences & Peking Union Medical College	Jia,B., Xiao,Y., Wang,Y., Chen,L., Zhang,J., Ren,L. and Wang,J.
EPI_ISL_2581237	Department of Public Health Laboratory Sciences, West China School of Public Health, Sichuan University	Department of Public Health Laboratory Sciences, West China School of Public Health, Sichuan University	Hu,W.P. and Pei,F.X.
EPI_ISL_2581242	Department of Respiratory Medicine, Children's Hospital, Chongqing Medical University	Department of Respiratory Medicine, Children's Hospital, Chongqing Medical University	Ren,L. and Liu,E.
EPI_ISL_2581244, EPI_ISL_2581245, EPI_ISL_2581247, EPI_ISL_2581250	WHO WPRO Measles Regional Reference Lab, Key Laboratory of Medical Virology Ministry of Health, National Institute for Viral Disease Control and Prevention, China Center for Disease Control and Prevention	WHO WPRO Measles Regional Reference Lab, Key Laboratory of Medical Virology Ministry of Health, National Institute for Viral Disease Control and Prevention, China Center for Disease Control and Prevention	Zhang,Y., Song,J. and Xu,W.
EPI_ISL_2581251, EPI_ISL_2581253, EPI_ISL_2581256, EPI_ISL_2581257, EPI_ISL_2581258, EPI_ISL_2581261, EPI_ISL_2581264	University of Wuerzburg, Institute of Virology and Immunobiology	University of Wuerzburg, Institute of Virology and Immunobiology	Prifert,C., Hofmann,D. and Weissbrich,B.
EPI_ISL_2581267	Virology, School of Public Health, Tehran University of Medical Sciences	Virology, School of Public Health, Tehran University of Medical Sciences	Yavarian,J., Faghihloo,E. and Mokhtari Azad,T.
EPI_ISL_2581269, EPI_ISL_2581271	Pediatric Clinic 1, Department of Pathophysiology and Transplantation, University of Milan and Fondazione IRCCS Ca Granda	Pediatric Clinic 1, Department of Pathophysiology and Transplantation, University of Milan and Fondazione IRCCS Ca Granda	Esposito,S., Zampiero,A., Piralla,A. and Principi,N.
EPI_ISL_2581292, EPI_ISL_2581293, EPI_ISL_2581295, EPI_ISL_2581297, EPI_ISL_2581299, EPI_ISL_2581302, EPI_ISL_2581303, EPI_ISL_2581305, EPI_ISL_2581307, EPI_ISL_2581309, EPI_ISL_2581311			
see above	Department of Paediatric Infectious Diseases, Institute of Tropical Medicine, Nagasaki University	Department of Paediatric Infectious Diseases, Institute of Tropical Medicine, Nagasaki University	Yoshihara,K., Nhat Le,M., Nagasawa,K., Tsukagoshi,H., Nguyen,H.A., Toizumi,M., Moriuchi,H., Hashizume,M., Ariyoshi,K., Dang,D.A., Kimura,H. and Yoshida,L.-M.
EPI_ISL_2581313	Virology, National Institute of Health-Pakistan, Park Road, Chak Shahzad, Islamabad	Virology, National Institute of Health-Pakistan, Park Road, Chak Shahzad, Islamabad	Aamir,U.B., Alam,M.M., Sadia,H. and Zaidi,S.S.Z.
EPI_ISL_2581315	School of Medicine, University of Washington	School of Medicine, University of Washington	Scott,E.M., Magaret,A., Kuypers,J., Stewart,L., Shrestha,L., Tielsch,J.M., Steinhoff,M., Katz,J., Khatry,S.K., LeClerq,S.C., Englund,J.A. and Chu,H.Y.
EPI_ISL_2581328	Virology Dep, School of Public Health and Institute of Public Health Research, Tehran University of Medical Sciences	Virology Dep, School of Public Health and Institute of Public Health Research, Tehran University of Medical Sciences	Mokhtari-Azad,T., Faghihloo,E., Salimi,V. and Rezaei,F.
EPI_ISL_2581329, EPI_ISL_2581331	Akinobu Hibino Niigata University, International Health, Public Health	Akinobu Hibino Niigata University, International Health, Public Health	Hibino,A., Saito,R., Shoubugawa,Y. and Tomimoto,K.
EPI_ISL_2581333	Akinobu Hibino Niigata University, International Health, Public Health	Akinobu Hibino Niigata University, International Health, Public Health	Hibino,A., Saito,R., Shoubugawa,Y. and Saito,T.
EPI_ISL_2581336, EPI_ISL_2581337	Eiko Hirano Fukui Prefectural Institute of Public Health and Environmental Science	Eiko Hirano Fukui Prefectural Institute of Public Health and Environmental Science	Hirano,E. and Kobayashi,M.
EPI_ISL_2581339	Akinobu Hibino Niigata University, International Health, Public Health	Akinobu Hibino Niigata University, International Health, Public Health	Hibino,A., Saito,R., Shoubugawa,Y. and Shirakawa,K.
EPI_ISL_2581341, EPI_ISL_2581344, EPI_ISL_2581345, EPI_ISL_2581347, EPI_ISL_2581349, EPI_ISL_2581350, EPI_ISL_2581352, EPI_ISL_2581353, EPI_ISL_2581355, EPI_ISL_2581357, EPI_ISL_2581359, EPI_ISL_2581361			
see above	Akinobu Hibino Niigata University, International Health, Public Health	Akinobu Hibino Niigata University, International Health, Public Health	Hibino,A., Saito,R., Shoubugawa,Y. and Sano,Y.
EPI_ISL_2581383	Chinese Academy of Medical Sciences & Peking Union Medical College	Chinese Academy of Medical Sciences & Peking Union Medical College	Jia,B., Xiao,Y., Wang,Y., Chen,L., Zhang,J., Ren,L. and Wang,J.
EPI_ISL_2581394	Department of Public Health Laboratory Sciences, West China School of Public Health, Sichuan University	Department of Public Health Laboratory Sciences, West China School of Public Health, Sichuan University	Hu,W.P. and Pei,F.X.
EPI_ISL_2581396, EPI_ISL_2581397, EPI_ISL_2581399, EPI_ISL_2581402	University of Wuerzburg, Institute of Virology and Immunobiology	University of Wuerzburg, Institute of Virology and Immunobiology	Prifert,C., Hofmann,D. and Weissbrich,B.
EPI_ISL_2581410, EPI_ISL_2581411, EPI_ISL_2581412, EPI_ISL_2581413	Virology, School of Public Health, Tehran University of Medical Sciences	Virology, School of Public Health, Tehran University of Medical Sciences	Yavarian,J., Faghihloo,E. and Mokhtari Azad,T.
EPI_ISL_2581417	Pediatric Clinic 1, Department of Pathophysiology and Transplantation, University of Milan and Fondazione IRCCS Ca Granda	Pediatric Clinic 1, Department of Pathophysiology and Transplantation, University of Milan and Fondazione IRCCS Ca Granda	Esposito,S., Zampiero,A., Piralla,A. and Principi,N.
EPI_ISL_2581431, EPI_ISL_2581433, EPI_ISL_2581434, EPI_ISL_2581435, EPI_ISL_2581437, EPI_ISL_2581439	Department of Paediatric Infectious Diseases, Institute of Tropical Medicine, Nagasaki University	Department of Paediatric Infectious Diseases, Institute of Tropical Medicine, Nagasaki University	Yoshihara,K., Nhat Le,M., Nagasawa,K., Tsukagoshi,H., Nguyen,H.A., Toizumi,M., Moriuchi,H., Hashizume,M., Ariyoshi,K., Dang,D.A., Kimura,H. and Yoshida,L.-M.
EPI_ISL_2581459	Chinese Academy of Medical Sciences & Peking Union Medical College	Chinese Academy of Medical Sciences & Peking Union Medical College	Jia,B., Xiao,Y., Wang,Y., Chen,L., Zhang,J., Ren,L. and Wang,J.
EPI_ISL_2581463, EPI_ISL_2581466	Virology, School of Public Health, Tehran University of Medical Sciences	Virology, School of Public Health, Tehran University of Medical Sciences	Yavarian,J., Faghihloo,E. and Mokhtari Azad,T.
EPI_ISL_2581467	Virology, National Institute of Health-Pakistan, Park Road, Chak Shahzad, Islamabad	Virology, National Institute of Health-Pakistan, Park Road, Chak Shahzad, Islamabad	Aamir,U.B., Alam,M.M., Sadia,H. and Zaidi,S.S.Z.

EPI_ISL_2581471, EPI_ISL_2581473	Eiko Hirano Fukui Prefectural Institute of Public Health and Environmental Science	Eiko Hirano Fukui Prefectural Institute of Public Health and Environmental Science	Hirano,E. and Kobayashi,M.
EPI_ISL_2581480, EPI_ISL_2581482, EPI_ISL_2581483	Centre for Interdisciplinary Research in Basic Science	Centre for Interdisciplinary Research in Basic Science	Haider,M.S.H., Naqvi,I.H., Broor,S., Ahmed,A. and Parveen,S.
EPI_ISL_2581493	University of Wuerzburg, Institute of Virology and Immunobiology	University of Wuerzburg, Institute of Virology and Immunobiology	Prifert,C., Hofmann,D. and Weissbrich,B.
EPI_ISL_2581497	Virology, School of Public Health, Tehran University of Medical Sciences	Virology, School of Public Health, Tehran University of Medical Sciences	Yavarian,J., Faghihloo,E. and Mokhtari Azad,T.
EPI_ISL_2581501, EPI_ISL_2581503	Pediatric Clinic 1, Department of Pathophysiology and Transplantation, University of Milan and Fondazione IRCCS Ca Granda	Pediatric Clinic 1, Department of Pathophysiology and Transplantation, University of Milan and Fondazione IRCCS Ca Granda	Esposito,S., Zampiero,A., Piralla,A. and Principi,N.
EPI_ISL_2581505	Department of Paediatric Infectious Diseases, Institute of Tropical Medicine, Nagasaki University	Department of Paediatric Infectious Diseases, Institute of Tropical Medicine, Nagasaki University	Yoshihara,K., Nhat Le,M., Nagasawa,K., Tsukagoshi,H., Nguyen,H.A., Toizumi,M., Moriuchi,H., Hashizume,M., Ariyoshi,K., Dang,D.A., Kimura,H. and Yoshida,L.-M.
EPI_ISL_2581507, EPI_ISL_2581509	Virology, Centre Pasteur du Cameroun	Virology, Centre Pasteur du Cameroun	Kenmoe,S., Vernet,M.-A., Miszczak,F., Dina,J., Penlap,V.B., Vabret,A. and Njouom,R.
EPI_ISL_2581524	Akinobu Hibino Niigata University, International Health, Public Health	Akinobu Hibino Niigata University, International Health, Public Health	Hibino,A., Saito,R., Shoubugawa,Y. and Sano,Y.
EPI_ISL_2581531	University of Wuerzburg, Institute of Virology and Immunobiology	University of Wuerzburg, Institute of Virology and Immunobiology	Prifert,C., Hofmann,D. and Weissbrich,B.
EPI_ISL_2581533	Centre for Interdisciplinary Research in Basic Science	Centre for Interdisciplinary Research in Basic Science	Haider,M.S.H., Naqvi,I.H., Broor,S., Ahmed,A. and Parveen,S.
EPI_ISL_2581543	Virology, National Institute of Health-Pakistan, Park Road, Chak Shahzad, Islamabad	Virology, National Institute of Health-Pakistan, Park Road, Chak Shahzad, Islamabad	Aamir,U.B., Alam,M.M., Sadia,H. and Zaidi,S.S.Z.
EPI_ISL_2581546	Virology, Centre Pasteur du Cameroun	Virology, Centre Pasteur du Cameroun	Kenmoe,S., Vernet,M.-A., Miszczak,F., Dina,J., Penlap,V.B., Vabret,A. and Njouom,R.
EPI_ISL_2581547	West China School of Public Health, Sichuan University	West China School of Public Health, Sichuan University	Zhang,M.
EPI_ISL_2581554, EPI_ISL_2581555	Centre for Interdisciplinary Research in Basic Science	Centre for Interdisciplinary Research in Basic Science	Haider,M.S.H., Naqvi,I.H., Broor,S., Ahmed,A. and Parveen,S.
EPI_ISL_2581575	West China School of Public Health, Sichuan University	West China School of Public Health, Sichuan University	Zhang,M.
EPI_ISL_2581577	Division of Immunology, Children's Hospital	Division of Immunology, Children's Hospital	Qin,X., Zhang,C., Zhao,Y. and Zhao,X.
EPI_ISL_2581581	Virology, School of Public Health, Tehran University of Medical Sciences	Virology, School of Public Health, Tehran University of Medical Sciences	Yavarian,J., Faghihloo,E. and Mokhtari Azad,T.
EPI_ISL_2581585, EPI_ISL_2581588	Chinese Academy of Medical Sciences & Peking Union Medical College	Chinese Academy of Medical Sciences & Peking Union Medical College	Jia,B., Xiao,Y., Wang,Y., Chen,L., Zhang,J., Ren,L. and Wang,J.
EPI_ISL_2581589, EPI_ISL_2581591, EPI_ISL_2581593, EPI_ISL_2581596, EPI_ISL_2581597	Laboratory of Virology, Capital Institute of Pediatrics	Laboratory of Virology, Capital Institute of Pediatrics	Cui,G., Deng,J., Zhu,R., Qian,Y., Sun,Y., Zhao,L. and Wang,F.
EPI_ISL_2581603, EPI_ISL_2581608, EPI_ISL_2581609, EPI_ISL_2581610, EPI_ISL_2581612	Chinese Academy of Medical Sciences & Peking Union Medical College	Chinese Academy of Medical Sciences & Peking Union Medical College	Jia,B., Xiao,Y., Wang,Y., Chen,L., Zhang,J., Ren,L. and Wang,J.
EPI_ISL_2581619, EPI_ISL_2581622	Central Laboratory, Guangzhou Women and Children's Medical Center	Central Laboratory, Guangzhou Women and Children's Medical Center	Xie,J.H., Zhu,B., Zhong,J.Y., Chen,Y. and Zhang,Y.Y.
EPI_ISL_2581623, EPI_ISL_2581625, EPI_ISL_2581627, EPI_ISL_2581629, EPI_ISL_2581631, EPI_ISL_2581633, EPI_ISL_2581635, EPI_ISL_2581637	Laboratory of Virology, Capital Institute of Pediatrics	Laboratory of Virology, Capital Institute of Pediatrics	Cui,G., Deng,J., Zhu,R., Qian,Y., Sun,Y., Zhao,L. and Wang,F.
EPI_ISL_2581641, EPI_ISL_2581642	Virology, Tropical Medicine Institute Pedro Kouri	Virology, Tropical Medicine Institute Pedro Kouri	Valdes,O., Corso,M., Pinon,A., Acosta,B., Savon,C., Gonzalez,G., Mune,M., Gonzalez,G., Hernandez,B., Echevarria,Y. and Oropesa,S.
EPI_ISL_2581644, EPI_ISL_2581646	Investigacion en Virologia y Biotecnologia, Instituto Conmemorativo Gorgas de Estudios de la Salud	Investigacion en Virologia y Biotecnologia, Instituto Conmemorativo Gorgas de Estudios de la Salud	Abrego,L., Delfraro,A., Franco,D., Castillo,C., Cano,M., Castillo,M., Castillo,J., Pascale,J. and Arbiza,J.
EPI_ISL_2581649, EPI_ISL_2581651, EPI_ISL_2581653, EPI_ISL_2581655, EPI_ISL_2581658, EPI_ISL_2581660, EPI_ISL_2581662, EPI_ISL_2581664, EPI_ISL_2581665, EPI_ISL_2581668, EPI_ISL_2581669, EPI_ISL_2581672, EPI_ISL_2581673, EPI_ISL_2581674, EPI_ISL_2581676, EPI_ISL_2581678, EPI_ISL_2581679, EPI_ISL_2581681, EPI_ISL_2581683, EPI_ISL_2581685, EPI_ISL_2581687			
see above	Chinese Academy of Medical Sciences & Peking Union Medical College	Chinese Academy of Medical Sciences & Peking Union Medical College	Jia,B., Xiao,Y., Wang,Y., Chen,L., Zhang,J., Ren,L. and Wang,J.
EPI_ISL_2581693, EPI_ISL_2581695, EPI_ISL_2581697, EPI_ISL_2581699, EPI_ISL_2581701, EPI_ISL_2581704, EPI_ISL_2581705	Central Laboratory, Guangzhou Women and Children's Medical Center	Central Laboratory, Guangzhou Women and Children's Medical Center	Xie,J.H., Zhu,B., Zhong,J.Y., Chen,Y. and Zhang,Y.Y.
EPI_ISL_2581706, EPI_ISL_2581707, EPI_ISL_2581709, EPI_ISL_2581711, EPI_ISL_2581713, EPI_ISL_2581715, EPI_ISL_2581716, EPI_ISL_2581717, EPI_ISL_2581718, EPI_ISL_2581719, EPI_ISL_2581721, EPI_ISL_2581724, EPI_ISL_2581725, EPI_ISL_2581727, EPI_ISL_2581729, EPI_ISL_2581731, EPI_ISL_2581733, EPI_ISL_2581735, EPI_ISL_2581737, EPI_ISL_2581739, EPI_ISL_2581741, EPI_ISL_2581743, EPI_ISL_2581745, EPI_ISL_2581747, EPI_ISL_2581749, EPI_ISL_2581751, EPI_ISL_2581753, EPI_ISL_2581756, EPI_ISL_2581757, EPI_ISL_2581759, EPI_ISL_2581762, EPI_ISL_2581763, EPI_ISL_2581765, EPI_ISL_2581768			
see above	Laboratory of Virology, Capital Institute of Pediatrics	Laboratory of Virology, Capital Institute of Pediatrics	Cui,G., Deng,J., Zhu,R., Qian,Y., Sun,Y., Zhao,L. and Wang,F.
EPI_ISL_2581784, EPI_ISL_2581785, EPI_ISL_2581787, EPI_ISL_2581789, EPI_ISL_2581790	Virology, Tropical Medicine Institute Pedro Kouri	Virology, Tropical Medicine Institute Pedro Kouri	Valdes,O., Corso,M., Pinon,A., Acosta,B., Savon,C., Gonzalez,G., Mune,M., Gonzalez,G., Hernandez,B., Echevarria,Y. and Oropesa,S.
EPI_ISL_2581794	Investigacion en Virologia y Biotecnologia, Instituto Conmemorativo Gorgas de Estudios de la Salud	Investigacion en Virologia y Biotecnologia, Instituto Conmemorativo Gorgas de Estudios de la Salud	Abrego,L., Delfraro,A., Franco,D., Castillo,C., Cano,M., Castillo,M., Castillo,J., Pascale,J. and Arbiza,J.
EPI_ISL_2581795	Virology, Tropical Medicine Institute Pedro Kouri	Virology, Tropical Medicine Institute Pedro Kouri	Valdes,O., Corso,M., Pinon,A., Acosta,B., Savon,C., Gonzalez,G., Mune,M., Gonzalez,G., Hernandez,B., Echevarria,Y. and Oropesa,S.
EPI_ISL_2581799, EPI_ISL_2581802, EPI_ISL_2581803, EPI_ISL_2581805, EPI_ISL_2581807, EPI_ISL_2581816, EPI_ISL_2581817, EPI_ISL_2581819, EPI_ISL_2581822, EPI_ISL_2581823	Chinese Academy of Medical Sciences & Peking Union Medical College	Chinese Academy of Medical Sciences & Peking Union Medical College	Jia,B., Xiao,Y., Wang,Y., Chen,L., Zhang,J., Ren,L. and Wang,J.
EPI_ISL_2581851, EPI_ISL_2581854	Central Laboratory, Guangzhou Women and Children's Medical Center	Central Laboratory, Guangzhou Women and Children's Medical Center	Xie,J.H., Zhu,B., Zhong,J.Y., Chen,Y. and Zhang,Y.Y.
EPI_ISL_2581861, EPI_ISL_2581863, EPI_ISL_2581865, EPI_ISL_2581868, EPI_ISL_2581869, EPI_ISL_2581872, EPI_ISL_2581873, EPI_ISL_2581875, EPI_ISL_2581877, EPI_ISL_2581880, EPI_ISL_2581881, EPI_ISL_2581884, EPI_ISL_2581885, EPI_ISL_2581887			
see above	Laboratory of Virology, Capital Institute of Pediatrics	Laboratory of Virology, Capital Institute of Pediatrics	Cui,G., Deng,J., Zhu,R., Qian,Y., Sun,Y., Zhao,L. and Wang,F.
EPI_ISL_2581913, EPI_ISL_2581915	Virology, Tropical Medicine Institute Pedro Kouri	Virology, Tropical Medicine Institute Pedro Kouri	Valdes,O., Corso,M., Pinon,A., Acosta,B., Savon,C., Gonzalez,G., Mune,M., Gonzalez,G., Hernandez,B., Echevarria,Y. and Oropesa,S.
EPI_ISL_2581920	Investigacion en Virologia y Biotecnologia, Instituto Conmemorativo Gorgas de Estudios de la Salud	Investigacion en Virologia y Biotecnologia, Instituto Conmemorativo Gorgas de Estudios de la Salud	Abrego,L., Delfraro,A., Franco,D., Castillo,C., Cano,M., Castillo,M., Castillo,J., Pascale,J. and Arbiza,J.
EPI_ISL_2581922	College of Medicine and Medical Research Institute, Chungbuk National University	College of Medicine and Medical Research Institute, Chungbuk National University	Baek,Y.H., Choi,E.H., Park,S.-J., Song,M.-S. and Choi,Y.-K.
EPI_ISL_2581941, EPI_ISL_2581945, EPI_ISL_2581949, EPI_ISL_2581952, EPI_ISL_2581956, EPI_ISL_2581957, EPI_ISL_2581961, EPI_ISL_2581977, EPI_ISL_2581985, EPI_ISL_2581988, EPI_ISL_2581995, EPI_ISL_2581998, EPI_ISL_2582012, EPI_ISL_2582022, EPI_ISL_2582023, EPI_ISL_2582026, EPI_ISL_2582027, EPI_ISL_2582030, EPI_ISL_2582031			
see above	Depto Microbiologia, Instituto de Ciencias Biomedicas, Universidade de Sao Paulo	Depto Microbiologia, Instituto de Ciencias Biomedicas, Universidade de Sao Paulo	Moura,F.E.A., Thomazelli,L.M., Candido,E.D.O., Florencio,C.M.G.D., Pereira,S.A.R., Oliveira,F.M.S., Alves,A.A., Ocadaque,C.J. and Durigon,E.L.
EPI_ISL_2582084, EPI_ISL_2582085, EPI_ISL_2582087, EPI_ISL_2582090, EPI_ISL_2582091, EPI_ISL_2582093	Virology, National Institute for Research in Tribal Health	Virology, National Institute for Research in Tribal Health	Sahu,M., Barde,P.V. and Shukla,M.K.
EPI_ISL_2582096, EPI_ISL_2582098	Manipal Centre for Virus Research, Manipal University	Manipal Centre for Virus Research, Manipal University	Anjali,A., Aswathy,R., Akhil,C., Giselle,D., Revti,B., Hindol,M., Suresh,P. and Arunkumar,G.
EPI_ISL_2582099, EPI_ISL_2582101, EPI_ISL_2582104, EPI_ISL_2582105, EPI_ISL_2582108, EPI_ISL_2582109, EPI_ISL_2582111	Influenza Group, National Institute of Virology	Influenza Group, National Institute of Virology	Choudhary,M.L., Anand,S.P., Wadhwa,B.S. and Chadha,M.S.
EPI_ISL_2582114, EPI_ISL_2582115, EPI_ISL_2582117, EPI_ISL_2582120, EPI_ISL_2582121, EPI_ISL_2582124, EPI_ISL_2582125, EPI_ISL_2582127, EPI_ISL_2582130, EPI_ISL_2582131, EPI_ISL_2582134, EPI_ISL_2582135, EPI_ISL_2582137, EPI_ISL_2582140, EPI_ISL_2582141, EPI_ISL_2582143, EPI_ISL_2582146			
see above	Microbiology, Chungnam Health and Environment Research Institute	Microbiology, Chungnam Health and Environment Research Institute	Hong,J., Kim,D., Park,S. and Park,K.
EPI_ISL_2582149, EPI_ISL_2582152, EPI_ISL_2582153, EPI_ISL_2582155, EPI_ISL_2582163	Marie Bashir Institute for Infectious Diseases and Biosecurity & Sydney Medical School, The University of Sydney, Westmead Institute for Medical Research	Marie Bashir Institute for Infectious Diseases and Biosecurity & Sydney Medical School, The University of Sydney, Westmead Institute for Medical Research	Eden,J.-S., Kok,J., Dwyer,D.E., Fernandez,M., Carter,I. and Holmes,E.C.
EPI_ISL_2582166	Virology, Graduate School of Medicine, Tohoku University	Virology, Graduate School of Medicine, Tohoku University	Malasao,R., Furuse,Y., Okamoto,M., Dapat,C., Saito,M., Saito-Obata,M., Tamaki,R., Segubre-Mercado,E., Lupisan,S. and Oshitani,H.
EPI_ISL_2582169	Pediatrics - Infectious Diseases, Medical College of Wisconsin	Pediatrics - Infectious Diseases, Medical College of Wisconsin	Rebuffo-Scheer,C., Bose,M.E., He,J., Khaja,S., Ulatowski,M., Beck,E.T., Fan,J., Kumar,S., Nelson,M.I. and Henrickson,K.J.
EPI_ISL_2582172	Marie Bashir Institute for Infectious Diseases and Biosecurity & Sydney Medical School, The University of Sydney, Westmead Institute for Medical Research	Marie Bashir Institute for Infectious Diseases and Biosecurity & Sydney Medical School, The University of Sydney, Westmead Institute for Medical Research	Eden,J.-S., Kok,J., Dwyer,D.E., Fernandez,M., Carter,I. and Holmes,E.C.
EPI_ISL_2582173, EPI_ISL_2582181	Virology, Graduate School of Medicine, Tohoku University	Virology, Graduate School of Medicine, Tohoku University	Malasao,R., Furuse,Y., Okamoto,M., Dapat,C., Saito,M., Saito-Obata,M., Tamaki,R., Segubre-Mercado,E., Lupisan,S. and Oshitani,H.
EPI_ISL_2582186	Marie Bashir Institute for Infectious Diseases and Biosecurity & Sydney Medical School, The University of Sydney, Westmead Institute for Medical Research	Marie Bashir Institute for Infectious Diseases and Biosecurity & Sydney Medical School, The University of Sydney, Westmead Institute for Medical Research	Eden,J.-S., Kok,J., Dwyer,D.E., Fernandez,M., Carter,I. and Holmes,E.C.
EPI_ISL_2582187	J. Craig Venter Institute	J. Craig Venter Institute	Tan,G., Pickett,B., Fedorova,N., Amedeo,P., Isom,R., Hu,L., Christensen,J., Miller,J., Novotny,M., Durbin,A., Rocchi,I., Williams,T., Arumemi,F. and Das,S.
EPI_ISL_2582190, EPI_ISL_2582193	Marie Bashir Institute for Infectious Diseases and Biosecurity & Sydney Medical School, The University of Sydney, Westmead Institute for Medical Research	Marie Bashir Institute for Infectious Diseases and Biosecurity & Sydney Medical School, The University of Sydney, Westmead Institute for Medical Research	Eden,J.-S., Kok,J., Dwyer,D.E., Fernandez,M., Carter,I. and Holmes,E.C.
EPI_ISL_2582196	J. Craig Venter Institute	J. Craig Venter Institute	Tan,G., Pickett,B., Fedorova,N., Amedeo,P., Isom,R., Hu,L., Christensen,J., Miller,J., Novotny,M., Durbin,A., Rocchi,I., Williams,T., Arumemi,F. and Das,S.
EPI_ISL_2582203	Marie Bashir Institute for Infectious Diseases and Biosecurity & Sydney Medical School, The University of Sydney, Westmead Institute for Medical Research	Marie Bashir Institute for Infectious Diseases and Biosecurity & Sydney Medical School, The University of Sydney, Westmead Institute for Medical Research	Eden,J.-S., Kok,J., Dwyer,D.E., Fernandez,M., Carter,I. and Holmes,E.C.
EPI_ISL_2582206	Laboratory of Virology, Capital Institute of Pediatrics	Laboratory of Virology, Capital Institute of Pediatrics	Cui,G., Deng,J., Zhu,R., Qian,Y., Sun,Y., Zhao,L. and Wang,F.
EPI_ISL_2582207	Pediatrics, Mackay Memorial Hospital	Pediatrics, Mackay Memorial Hospital	Chi,H., Hsiao,K.-L., Weng,L.-C., Chiu,N.-C., Huang,L.-M., Chiu,Y.-Y., Liu,C.-P. and Liu,H.-F.
EPI_ISL_2582217	Influenza Group, National Institute of Virology	Influenza Group, National Institute of Virology	Choudhary,M.L., Anand,S.P., Wadhwa,B.S. and Chadha,M.S.
EPI_ISL_2582226	Marie Bashir Institute for Infectious Diseases and Biosecurity & Sydney Medical School, The University of Sydney, Westmead Institute for Medical Research	Marie Bashir Institute for Infectious Diseases and Biosecurity & Sydney Medical School, The University of Sydney, Westmead Institute for Medical Research	Eden,J.-S., Kok,J., Dwyer,D.E., Fernandez,M., Carter,I. and Holmes,E.C.
EPI_ISL_2582267	Epidemiology and Demography Department, KEMRI-Wellcome Trust Research	Epidemiology and Demography Department, KEMRI-Wellcome Trust Research	Otieno,J.R., Kamau,E.M., Oketch,J.W., Ngoi,J.M., Agoti,C.N., Gichuki,A.M., Otieno,G.P., Ngama,M., Cane,P.A., Kellam,P., Cotten,M., Lemey,P. and

	Programme	Programme	Nokes,D.J.
EPI_ISL_2582473	Marie Bashir Institute for Infectious Diseases and Biosecurity & Sydney Medical School, The University of Sydney, Westmead Institute for Medical Research	Marie Bashir Institute for Infectious Diseases and Biosecurity & Sydney Medical School, The University of Sydney, Westmead Institute for Medical Research	Eden,J.-S., Kok,J., Dwyer,D.E., Fernandez,M., Carter,I. and Holmes,E.C.
EPI_ISL_2582527, EPI_ISL_2582529, EPI_ISL_2582535	Depto Microbiologia, Instituto de Ciencias Biomedicas, Universidade de Sao Paulo	Depto Microbiologia, Instituto de Ciencias Biomedicas, Universidade de Sao Paulo	Moura,F.E.A., Thomazelli,L.M., Candido,E.D.O., Florencio,C.M.G.D., Pereira,S.A.R., Oliveira,F.M.S., Alves,A.A., Ocadaque,C.J. and Durigon,E.L.
EPI_ISL_2582543, EPI_ISL_2582545, EPI_ISL_2582547	J. Craig Venter Institute	J. Craig Venter Institute	Tan,G., Pickett,B., Fedorova,N., Amedeo,P., Isom,R., Hu,L., Christensen,J., Miller,J., Novotny,M., Durbin,A., Rocchi,I., Williams,T., Arumemi,F. and Das,S.
EPI_ISL_2582549	Epidemiology and Demography Department, KEMRI-Wellcome Trust Research Programme	Epidemiology and Demography Department, KEMRI-Wellcome Trust Research Programme	Otieno,J.R., Kamau,E.M., Oketch,J.W., Ngoi,J.M., Agoti,C.N., Gichuki,A.M., Otieno,G.P., Ngama,M., Cane,P.A., Kellam,P., Cotten,M., Lemey,P. and Nokes,D.J.
EPI_ISL_2582553	J. Craig Venter Institute	J. Craig Venter Institute	Tan,G., Pickett,B., Fedorova,N., Amedeo,P., Isom,R., Hu,L., Christensen,J., Miller,J., Novotny,M., Durbin,A., Rocchi,I., Williams,T., Arumemi,F. and Das,S.
EPI_ISL_2582556	Epidemiology and Demography Department, KEMRI-Wellcome Trust Research Programme	Epidemiology and Demography Department, KEMRI-Wellcome Trust Research Programme	Otieno,J.R., Kamau,E.M., Oketch,J.W., Ngoi,J.M., Agoti,C.N., Gichuki,A.M., Otieno,G.P., Ngama,M., Cane,P.A., Kellam,P., Cotten,M., Lemey,P. and Nokes,D.J.
EPI_ISL_2582559	Depto Microbiologia, Instituto de Ciencias Biomedicas, Universidade de Sao Paulo	Depto Microbiologia, Instituto de Ciencias Biomedicas, Universidade de Sao Paulo	Moura,F.E.A., Thomazelli,L.M., Candido,E.D.O., Florencio,C.M.G.D., Pereira,S.A.R., Oliveira,F.M.S., Alves,A.A., Ocadaque,C.J. and Durigon,E.L.
EPI_ISL_2582561	Central Laboratory, Guangzhou Women and Children's Medical Center	Central Laboratory, Guangzhou Women and Children's Medical Center	Xie,J.H., Zhu,B., Zhong,J.Y., Chen,Y. and Zhang,Y.Y.
EPI_ISL_2582563	The Second Department, Lanzhou Institute of Biological Products Co	The Second Department, Lanzhou Institute of Biological Products Co	Zhu,C., Fu,S., Yu,L. and Zhou,X.
EPI_ISL_2582569, EPI_ISL_2582571	J. Craig Venter Institute	J. Craig Venter Institute	Tan,G., Pickett,B., Fedorova,N., Amedeo,P., Isom,R., Hu,L., Christensen,J., Miller,J., Novotny,M., Durbin,A., Rocchi,I., Williams,T., Arumemi,F. and Das,S.
EPI_ISL_2582573	Pediatrics - Infectious Diseases, Medical College of Wisconsin	Pediatrics - Infectious Diseases, Medical College of Wisconsin	Rebuffo-Scheer,C., Bose,M.E., He,J., Khaja,S., Ulatowski,M., Beck,E.T., Fan,J., Kumar,S., Nelson,M.I. and Henrickson,K.J.
EPI_ISL_2582576	Marie Bashir Institute for Infectious Diseases and Biosecurity & Sydney Medical School, The University of Sydney, Westmead Institute for Medical Research	Marie Bashir Institute for Infectious Diseases and Biosecurity & Sydney Medical School, The University of Sydney, Westmead Institute for Medical Research	Eden,J.-S., Kok,J., Dwyer,D.E., Fernandez,M., Carter,I. and Holmes,E.C.
EPI_ISL_2582619	Microbiology, Chungnam Health and Environment Research Institute	Microbiology, Chungnam Health and Environment Research Institute	Hong,J., Kim,D., Park,S. and Park,K.
EPI_ISL_2582621	Academy of Military Medical Sciences, Institute of Microbiology and Epidemiology	Academy of Military Medical Sciences, Institute of Microbiology and Epidemiology	Gu,H.J., Sun,S.J., Chen,R. and Yang,P.H.
EPI_ISL_2582627	Influenza Group, National Institute of Virology	Influenza Group, National Institute of Virology	Choudhary,M.L., Anand,S.P., Wadhwa,B.S. and Chadha,M.S.
EPI_ISL_2582637	Institute of Endemic Diseases, University of Khartoum	Institute of Endemic Diseases, University of Khartoum	Khalil,S.O., Altayb,H.N., Anan,K.A., H,A.Y., Elkhidir,I.M. and Hassan,M.A.
EPI_ISL_2582762	Broad Institute of MIT & Harvard	Broad Institute of MIT & Harvard	Newman,R.M., Zody,M.C., DeVincenzo,J.P., Grad,Y., Lipsitch,M., Murphy,R., Fitzgerald,M., Young,S., Gargeya,S., Poon,T.W., Charlebois,P., Weiner,B., Yang,X., Piper,M.E., McCowan,C., Ireland,A., Levin,J., Malboeuf,C., Qu,J., Chapman,S.B., Murphy,C., Wortman,J., Nusbaum,C. and Birren,B.
EPI_ISL_2582789, EPI_ISL_2582793	Epidemiology and Demography Department, KEMRI-Wellcome Trust Research Programme	Epidemiology and Demography Department, KEMRI-Wellcome Trust Research Programme	Otieno,J.R., Kamau,E.M., Oketch,J.W., Ngoi,J.M., Agoti,C.N., Gichuki,A.M., Otieno,G.P., Ngama,M., Cane,P.A., Kellam,P., Cotten,M., Lemey,P. and Nokes,D.J.
EPI_ISL_2582796	Central Laboratory, Guangzhou Women and Children's Medical Center	Central Laboratory, Guangzhou Women and Children's Medical Center	Xie,J.H., Zhu,B., Zhong,J.Y., Chen,Y. and Zhang,Y.Y.
EPI_ISL_2582801	Department of Laboratory Medicine, Lin-Kou Chang-Gung Memorial Hospital	Department of Laboratory Medicine, Lin-Kou Chang-Gung Memorial Hospital	Tsao,K.-C., Gong,Y.-N., Yang,S.-L., Chen,G.-W., Chen,Y.-W., Huang,Y.-C. and Liu,Y.-C.
EPI_ISL_2582804	Epidemiology and Demography Department, KEMRI-Wellcome Trust Research Programme	Epidemiology and Demography Department, KEMRI-Wellcome Trust Research Programme	Otieno,J.R., Kamau,E.M., Oketch,J.W., Ngoi,J.M., Agoti,C.N., Gichuki,A.M., Otieno,G.P., Ngama,M., Cane,P.A., Kellam,P., Cotten,M., Lemey,P. and Nokes,D.J.
EPI_ISL_2582805	J. Craig Venter Institute	J. Craig Venter Institute	Tan,G., Pickett,B., Fedorova,N., Amedeo,P., Isom,R., Hu,L., Christensen,J., Miller,J., Novotny,M., Durbin,A., Rocchi,I., Williams,T., Arumemi,F. and Das,S.
EPI_ISL_2582871	Epidemiology and Demography Department, KEMRI-Wellcome Trust Research Programme	Epidemiology and Demography Department, KEMRI-Wellcome Trust Research Programme	Otieno,J.R., Kamau,E.M., Oketch,J.W., Ngoi,J.M., Agoti,C.N., Gichuki,A.M., Otieno,G.P., Ngama,M., Cane,P.A., Kellam,P., Cotten,M., Lemey,P. and Nokes,D.J.
EPI_ISL_2582880	Medicine, University of Washington, 300 9th Ave, Harborview Research & Training Building	Medicine, University of Washington, 300 9th Ave, Harborview Research & Training Building	Chu,H., Scott,E. and Roychoudhury,P.
EPI_ISL_2582889, EPI_ISL_2582896	Epidemiology and Demography Department, KEMRI-Wellcome Trust Research Programme	Epidemiology and Demography Department, KEMRI-Wellcome Trust Research Programme	Otieno,J.R., Kamau,E.M., Oketch,J.W., Ngoi,J.M., Agoti,C.N., Gichuki,A.M., Otieno,G.P., Ngama,M., Cane,P.A., Kellam,P., Cotten,M., Lemey,P. and Nokes,D.J.
EPI_ISL_2582898	Marie Bashir Institute for Infectious Diseases and Biosecurity & Sydney Medical School, The University of Sydney, Westmead Institute for Medical Research	Marie Bashir Institute for Infectious Diseases and Biosecurity & Sydney Medical School, The University of Sydney, Westmead Institute for Medical Research	Eden,J.-S., Kok,J., Dwyer,D.E., Fernandez,M., Carter,I. and Holmes,E.C.
EPI_ISL_2582908, EPI_ISL_2582910, EPI_ISL_2582913, EPI_ISL_2582916, EPI_ISL_2582918, EPI_ISL_2583023, EPI_ISL_2583024	Epidemiology and Demography Department, KEMRI-Wellcome Trust Research Programme	Epidemiology and Demography Department, KEMRI-Wellcome Trust Research Programme	Otieno,J.R., Kamau,E.M., Oketch,J.W., Ngoi,J.M., Agoti,C.N., Gichuki,A.M., Otieno,G.P., Ngama,M., Cane,P.A., Kellam,P., Cotten,M., Lemey,P. and Nokes,D.J.
EPI_ISL_2583031	Pediatrics - Infectious Diseases, Medical College of Wisconsin	Pediatrics - Infectious Diseases, Medical College of Wisconsin	Rebuffo-Scheer,C., Bose,M.E., He,J., Khaja,S., Ulatowski,M., Beck,E.T., Fan,J., Kumar,S., Nelson,M.I. and Henrickson,K.J.
EPI_ISL_2583059	Epidemiology and Demography Department, KEMRI-Wellcome Trust Research Programme	Epidemiology and Demography Department, KEMRI-Wellcome Trust Research Programme	Otieno,J.R., Kamau,E.M., Oketch,J.W., Ngoi,J.M., Agoti,C.N., Gichuki,A.M., Otieno,G.P., Ngama,M., Cane,P.A., Kellam,P., Cotten,M., Lemey,P. and Nokes,D.J.
EPI_ISL_2583124	Virology, National Institute for Research in Tribal Health	Virology, National Institute for Research in Tribal Health	Sahu,M., Barde,P.V. and Shukla,M.K.
EPI_ISL_2583137, EPI_ISL_2583139, EPI_ISL_2583149, EPI_ISL_2583150, EPI_ISL_2583152, EPI_ISL_2583154, EPI_ISL_2583156	Microbiology, Chungnam Health and Environment Research Institute	Microbiology, Chungnam Health and Environment Research Institute	Hong,J., Kim,D., Park,S. and Park,K.
EPI_ISL_2583159	Virology, National Institute for Research in Tribal Health	Virology, National Institute for Research in Tribal Health	Sahu,M., Barde,P.V. and Shukla,M.K.
EPI_ISL_2583160, EPI_ISL_2583163, EPI_ISL_2583165, EPI_ISL_2583170	Microbiology, Chungnam Health and Environment Research Institute	Microbiology, Chungnam Health and Environment Research Institute	Hong,J., Kim,D., Park,S. and Park,K.
EPI_ISL_2585201	Epidemiology and Demography Department, KEMRI - Wellcome Trust Research Collaborative Programme	Epidemiology and Demography Department, KEMRI - Wellcome Trust Research Collaborative Programme	Agoti,C.N., Munywoki,P.K., Phan,M.V.T., Otieno,J.R., Kamau,E., Bett,A., Githinji,G., Medley,G.F., Cane,P.A., Kellam,P., Cotten,M.L. and Nokes,D.J.
EPI_ISL_2585202	KEMRI Wellcome Trust Research Programme	KEMRI Wellcome Trust Research Programme	Agoti,C.N., Otieno,J.R., Munywoki,P.K., Mwihuri,A.G., Cane,P.A., Nokes,D.J., Kellam,P. and Cotten,M.L.
EPI_ISL_2585203, EPI_ISL_2585204, EPI_ISL_2585206	Epidemiology and Demography Department, KEMRI - Wellcome Trust Research Collaborative Programme	Epidemiology and Demography Department, KEMRI - Wellcome Trust Research Collaborative Programme	Agoti,C.N., Munywoki,P.K., Phan,M.V.T., Otieno,J.R., Kamau,E., Bett,A., Githinji,G., Medley,G.F., Cane,P.A., Kellam,P., Cotten,M.L. and Nokes,D.J.
EPI_ISL_2585213, EPI_ISL_2585214, EPI_ISL_2585215, EPI_ISL_2585216, EPI_ISL_2585217, EPI_ISL_2585218	Pediatrics, Center of Excellence in Clinical Virology, Faculty of Medicine, Chulalongkorn University	Pediatrics, Center of Excellence in Clinical Virology, Faculty of Medicine, Chulalongkorn University	Auksornkitti,V., Kamprasert,N., Thongkomplew,S., Suwannakarn,K., Theamboonlers,A., Samransamruajkij,R. and Poovorawan,Y.
EPI_ISL_2585219	Lab. Virologia. Depto. Microbiologia, Universidad Autonoma de San Luis Potosi	Lab. Virologia. Depto. Microbiologia, Universidad Autonoma de San Luis Potosi	Comas-Garcia,A., Noyola,D.E., Cadena-Mota,S., Rico-Hernandez,M. and Bernal-Silva,S.
EPI_ISL_2585221, EPI_ISL_2585222, EPI_ISL_2585223, EPI_ISL_2585224	Pediatrics, Center of Excellence in Clinical Virology, Faculty of Medicine, Chulalongkorn University	Pediatrics, Center of Excellence in Clinical Virology, Faculty of Medicine, Chulalongkorn University	Auksornkitti,V., Kamprasert,N., Thongkomplew,S., Suwannakarn,K., Theamboonlers,A., Samransamruajkij,R. and Poovorawan,Y.
EPI_ISL_2585225	Epidemiology and Demography Department, KEMRI - Wellcome Trust Research Collaborative Programme	Epidemiology and Demography Department, KEMRI - Wellcome Trust Research Collaborative Programme	Agoti,C.N., Munywoki,P.K., Phan,M.V.T., Otieno,J.R., Kamau,E., Bett,A., Githinji,G., Medley,G.F., Cane,P.A., Kellam,P., Cotten,M.L. and Nokes,D.J.
EPI_ISL_2585258	J. Craig Venter Institute	J. Craig Venter Institute	Shabman,R., Das,S.R., Shilts,M., Fedorova,N., Puri,V., Shrivastava,S., Amedeo,P., Williams,M., Barratt,K., Mitchell,J. and Jennings,L.
EPI_ISL_2585260	J. Craig Venter Institute	J. Craig Venter Institute	Das,S.R., Halpin,R.A., Shilts,M., Puri,V., Akopov,A., Fedorova,N., Stockwell,T., Amedeo,P., Bishop,B., Katzel,D., Schobel,S., Shrivastava,S. and Hartert,T.
EPI_ISL_2585261	J. Craig Venter Institute	J. Craig Venter Institute	Shabman,R., Das,S.R., Shilts,M., Fedorova,N., Puri,V., Shrivastava,S., Amedeo,P., Williams,M., Barratt,K., Mitchell,J. and Jennings,L.
EPI_ISL_2585263, EPI_ISL_2585264	KEMRI Wellcome Trust Research Programme	KEMRI Wellcome Trust Research Programme	Agoti,C.N., Otieno,J.R., Munywoki,P.K., Mwihuri,A.G., Cane,P.A., Nokes,D.J., Kellam,P. and Cotten,M.L.
EPI_ISL_2585265	J. Craig Venter Institute	J. Craig Venter Institute	Das,S.R., Halpin,R.A., Shilts,M., Puri,V., Akopov,A., Fedorova,N., Stockwell,T., Amedeo,P., Bishop,B., Katzel,D., Schobel,S., Shrivastava,S. and Hartert,T.
EPI_ISL_2585271, EPI_ISL_2585272	Epidemiology and Demography Department, KEMRI - Wellcome Trust Research Collaborative Programme	Epidemiology and Demography Department, KEMRI - Wellcome Trust Research Collaborative Programme	Agoti,C.N., Munywoki,P.K., Phan,M.V.T., Otieno,J.R., Kamau,E., Bett,A., Githinji,G., Medley,G.F., Cane,P.A., Kellam,P., Cotten,M.L. and Nokes,D.J.
EPI_ISL_2585275, EPI_ISL_2585276, EPI_ISL_2585277, EPI_ISL_2585279	J. Craig Venter Institute	J. Craig Venter Institute	Shabman,R., Das,S.R., Shilts,M., Fedorova,N., Puri,V., Shrivastava,S., Amedeo,P., Williams,M., Barratt,K., Mitchell,J. and Jennings,L.
EPI_ISL_2585285, EPI_ISL_2585288	J. Craig Venter Institute	J. Craig Venter Institute	Das,S.R., Halpin,R.A., Shilts,M., Puri,V., Akopov,A., Fedorova,N., Stockwell,T., Amedeo,P., Bishop,B., Katzel,D., Schobel,S., Shrivastava,S. and Hartert,T.
EPI_ISL_2585291	J. Craig Venter Institute	J. Craig Venter Institute	Shabman,R., Das,S.R., Shilts,M., Fedorova,N., Puri,V., Shrivastava,S., Amedeo,P., Williams,M., Barratt,K., Mitchell,J. and Jennings,L.
EPI_ISL_2585294	Lab. Virologia. Depto. Microbiologia, Universidad Autonoma de San Luis Potosi	Lab. Virologia. Depto. Microbiologia, Universidad Autonoma de San Luis Potosi	Comas-Garcia,A., Noyola,D.E., Cadena-Mota,S., Rico-Hernandez,M. and Bernal-Silva,S.
EPI_ISL_2585299	Epidemiology and Demography Department, KEMRI - Wellcome Trust Research Collaborative Programme	Epidemiology and Demography Department, KEMRI - Wellcome Trust Research Collaborative Programme	Agoti,C.N., Munywoki,P.K., Phan,M.V.T., Otieno,J.R., Kamau,E., Bett,A., Githinji,G., Medley,G.F., Cane,P.A., Kellam,P., Cotten,M.L. and Nokes,D.J.
EPI_ISL_2585309	J. Craig Venter Institute	J. Craig Venter Institute	Das,S.R., Halpin,R.A., Shilts,M., Puri,V., Akopov,A., Fedorova,N., Stockwell,T., Amedeo,P., Bishop,B., Katzel,D., Schobel,S., Shrivastava,S. and Hartert,T.
EPI_ISL_2585311	J. Craig Venter Institute	J. Craig Venter Institute	Shabman,R., Das,S.R., Shilts,M., Fedorova,N., Puri,V., Shrivastava,S., Amedeo,P., Williams,M., Barratt,K., Mitchell,J. and Jennings,L.
EPI_ISL_2585320, EPI_ISL_2585323	J. Craig Venter Institute	J. Craig Venter Institute	Das,S.R., Halpin,R.A., Shilts,M., Puri,V., Akopov,A., Fedorova,N., Stockwell,T., Amedeo,P., Bishop,B., Katzel,D., Schobel,S., Shrivastava,S. and Hartert,T.

EPI_ISL_2585333, EPI_ISL_2585334	Centre for Respiratory Diseases and Meningitis, National Institute for Communicable Diseases	Centre for Respiratory Diseases and Meningitis, National Institute for Communicable Diseases	Pretorius,M.A., van Niekerk,S., Tempia,S., Moyes,J., Cohen,C., Madhi,S.A. and Venter,M.
EPI_ISL_2585336	KEMRI Wellcome Trust Research Programme	KEMRI Wellcome Trust Research Programme	Agoti,C.N., Otieno,J.R., Munywoki,P.K., Mwihuri,A.G., Cane,P.A., Nokes,D.J., Kellam,P. and Cotten,M.L.
EPI_ISL_2585338	J. Craig Venter Institute	J. Craig Venter Institute	Lorenzi,H., Town,C., Halpin,R., Bera,J., Ransier,A., Fedorova,N., Stockwell,T., Amedeo,P., Appalla,L., Bishop,B., Edworthy,P., Gupta,N., Hoover,J., Katzel,D., Schobel,S., Shrivastava,S., Thovarai,V., Wang,S., Rebuffo-Scheer,C., Fan,J., He,J., Kehl,S.C., Lederboer,N., Jurgens,L.A., Bose,M.E., Beck,E.T., Kumar,S., Noyola,D.E., Wentworth,D.E. and Henrickson,K.J.
EPI_ISL_2585352	Botany and Microbiology, College of Science, King Saud University	Botany and Microbiology, College of Science, King Saud University	Farrag,M.A., Amer,H.M. and Almajhdi,F.N.
EPI_ISL_2585354, EPI_ISL_2585355, EPI_ISL_2585356, EPI_ISL_2585358, EPI_ISL_2585359, EPI_ISL_2585362, EPI_ISL_2585363	Wan Ji Lee Korea National Institute of Health, Division of Respiratory Viruses, Center for Infectious Diseases	Wan Ji Lee Korea National Institute of Health, Division of Respiratory Viruses, Center for Infectious Diseases	Lee,W.J., Kim,Y.J., Kim,D.W., Lee,H.Y., Yun,M.R., Lee,H.S., Jung,H.D. and Kim,K.
EPI_ISL_2585364, EPI_ISL_2585366, EPI_ISL_2585367, EPI_ISL_2585368, EPI_ISL_2585370, EPI_ISL_2585371, EPI_ISL_2585372, EPI_ISL_2585375, EPI_ISL_2585378, EPI_ISL_2585379, EPI_ISL_2585380, EPI_ISL_2585382, EPI_ISL_2585383, EPI_ISL_2585385, EPI_ISL_2585386, EPI_ISL_2585387, EPI_ISL_2585389, EPI_ISL_2585390, EPI_ISL_2585391, EPI_ISL_2585393, EPI_ISL_2585394, EPI_ISL_2585395, EPI_ISL_2585397, EPI_ISL_2585398, EPI_ISL_2585399, EPI_ISL_2585401, EPI_ISL_2585402, EPI_ISL_2585403, EPI_ISL_2585405, EPI_ISL_2585406, EPI_ISL_2585407, EPI_ISL_2585408, EPI_ISL_2585410, EPI_ISL_2585412, EPI_ISL_2585414, EPI_ISL_2585416, EPI_ISL_2585418, EPI_ISL_2585419, EPI_ISL_2585421, EPI_ISL_2585423, EPI_ISL_2585425, EPI_ISL_2585427, EPI_ISL_2585430, EPI_ISL_2585432, EPI_ISL_2585434, EPI_ISL_2585436, EPI_ISL_2585437, EPI_ISL_2585439, EPI_ISL_2585441, EPI_ISL_2585443, EPI_ISL_2585445, EPI_ISL_2585447, EPI_ISL_2585448, EPI_ISL_2585450, EPI_ISL_2585452, EPI_ISL_2585454, EPI_ISL_2585455, EPI_ISL_2585457, EPI_ISL_2585459, EPI_ISL_2585461, EPI_ISL_2585463, EPI_ISL_2585465, EPI_ISL_2585467, EPI_ISL_2585468, EPI_ISL_2585470, EPI_ISL_2585472, EPI_ISL_2585474, EPI_ISL_2585476, EPI_ISL_2585479, EPI_ISL_2585481, EPI_ISL_2585483, EPI_ISL_2585484, EPI_ISL_2585486, EPI_ISL_2585488, EPI_ISL_2585489, EPI_ISL_2585491, EPI_ISL_2585493, EPI_ISL_2585495, EPI_ISL_2585496, EPI_ISL_2585499, EPI_ISL_2585501, EPI_ISL_2585503, EPI_ISL_2585505, EPI_ISL_2585506, EPI_ISL_2585508, EPI_ISL_2585510, EPI_ISL_2585512, EPI_ISL_2585514			
see above	Pediatrics, Center of Excellence in Clinical Virology, Faculty of Medicine, Chulalongkorn University	Pediatrics, Center of Excellence in Clinical Virology, Faculty of Medicine, Chulalongkorn University	Auksornkitti,V., Kamprasert,N., Thongkomplew,S., Suwannakarn,K., Theamboonlers,A., Samransamruajkij,R. and Poovorawan,Y.
EPI_ISL_2585532, EPI_ISL_2585534, EPI_ISL_2585535	Centre for Infectious Diseases, University of Edinburgh	Centre for Infectious Diseases, University of Edinburgh	Gaunt,E.R.
EPI_ISL_2585546	Epidemiology and Demography Department, KEMRI - Wellcome Trust Research Collaborative Programme	Epidemiology and Demography Department, KEMRI - Wellcome Trust Research Collaborative Programme	Agoti,C.N., Munywoki,P.K., Phan,M.V.T., Otieno,J.R., Kamau,E., Bett,A., Githinji,G., Medley,G.F., Cane,P.A., Kellam,P., Cotten,M.L. and Nokes,D.J.
EPI_ISL_2585550, EPI_ISL_2585551, EPI_ISL_2585553, EPI_ISL_2585555, EPI_ISL_2585557, EPI_ISL_2585559	Centre for Respiratory Diseases and Meningitis, National Institute for Communicable Diseases	Centre for Respiratory Diseases and Meningitis, National Institute for Communicable Diseases	Pretorius,M.A., van Niekerk,S., Tempia,S., Moyes,J., Cohen,C., Madhi,S.A. and Venter,M.
EPI_ISL_2585561, EPI_ISL_2585563, EPI_ISL_2585565, EPI_ISL_2585567, EPI_ISL_2585569	Influenza and Other Respiratory Viruses Unit, National Center for Microbiology, Instituto de Salud Carlos III	Influenza and Other Respiratory Viruses Unit, National Center for Microbiology, Instituto de Salud Carlos III	Casas,I., Calderon,A., Gonzalez,M., Molinero,M. and Pozo,F.
EPI_ISL_2585574, EPI_ISL_2585578, EPI_ISL_2585580, EPI_ISL_2585581	Centre for Respiratory Diseases and Meningitis, National Institute for Communicable Diseases	Centre for Respiratory Diseases and Meningitis, National Institute for Communicable Diseases	Pretorius,M.A., van Niekerk,S., Tempia,S., Moyes,J., Cohen,C., Madhi,S.A. and Venter,M.
EPI_ISL_2585583, EPI_ISL_2585585, EPI_ISL_2585587, EPI_ISL_2585588, EPI_ISL_2585590, EPI_ISL_2585592, EPI_ISL_2585594, EPI_ISL_2585595, EPI_ISL_2585597, EPI_ISL_2585599, EPI_ISL_2585601, EPI_ISL_2585603, EPI_ISL_2585605, EPI_ISL_2585607, EPI_ISL_2585609, EPI_ISL_2585611, EPI_ISL_2585613, EPI_ISL_2585614, EPI_ISL_2585616, EPI_ISL_2585618, EPI_ISL_2585620, EPI_ISL_2585622, EPI_ISL_2585624, EPI_ISL_2585626			
see above	Influenza and Other Respiratory Viruses Unit, National Center for Microbiology, Instituto de Salud Carlos III	Influenza and Other Respiratory Viruses Unit, National Center for Microbiology, Instituto de Salud Carlos III	Casas,I., Calderon,A., Gonzalez,M., Molinero,M. and Pozo,F.
EPI_ISL_2585631	Centre for Respiratory Diseases and Meningitis, National Institute for Communicable Diseases	Centre for Respiratory Diseases and Meningitis, National Institute for Communicable Diseases	Pretorius,M.A., van Niekerk,S., Tempia,S., Moyes,J., Cohen,C., Madhi,S.A. and Venter,M.
EPI_ISL_2585635, EPI_ISL_2585637, EPI_ISL_2585639, EPI_ISL_2585641, EPI_ISL_2585642, EPI_ISL_2585643	Influenza and Other Respiratory Viruses Unit, National Center for Microbiology, Instituto de Salud Carlos III	Influenza and Other Respiratory Viruses Unit, National Center for Microbiology, Instituto de Salud Carlos III	Casas,I., Calderon,A., Gonzalez,M., Molinero,M. and Pozo,F.
EPI_ISL_2585644, EPI_ISL_2585651, EPI_ISL_2585682, EPI_ISL_2585688, EPI_ISL_2585697, EPI_ISL_2585698, EPI_ISL_2585733, EPI_ISL_2585737, EPI_ISL_2585742, EPI_ISL_2585744, EPI_ISL_2585749, EPI_ISL_2585754, EPI_ISL_2585761, EPI_ISL_2585793, EPI_ISL_2585794, EPI_ISL_2585810			
see above	Virology, Public Health Institution of Turkey	Virology, Public Health Institution of Turkey	Bayrakdar,F.
EPI_ISL_2585824, EPI_ISL_2585826	Izumi Kushibuchi Tochigi prefectural Institute of Public Health and Enviromental Science	Izumi Kushibuchi Tochigi prefectural Institute of Public Health and Enviromental Science	Kushibuchi,I. and Kobayashi,M.
EPI_ISL_2585844, EPI_ISL_2585854	Virology, Public Health Institution of Turkey	Virology, Public Health Institution of Turkey	Bayrakdar,F.
EPI_ISL_2585900, EPI_ISL_2585905	Department of Medical Microbiology, Faculty of Medicine, University Malaya, Lembah Pantai, Kuala Lumpur	Department of Medical Microbiology, Faculty of Medicine, University Malaya, Lembah Pantai, Kuala Lumpur	Khor,C.S., Sam,I.C. and Chan,Y.F.
EPI_ISL_2585918, EPI_ISL_2585928, EPI_ISL_2585930	Izumi Kushibuchi Tochigi prefectural Institute of Public Health and Enviromental Science	Izumi Kushibuchi Tochigi prefectural Institute of Public Health and Enviromental Science	Kushibuchi,I. and Kobayashi,M.
EPI_ISL_2585948	Virology, Public Health Institution of Turkey	Virology, Public Health Institution of Turkey	Bayrakdar,F.
EPI_ISL_2585961	Centre for Respiratory Diseases and Meningitis, National Institute for Communicable Diseases	Centre for Respiratory Diseases and Meningitis, National Institute for Communicable Diseases	Pretorius,M.A., van Niekerk,S., Tempia,S., Moyes,J., Cohen,C., Madhi,S.A. and Venter,M.
EPI_ISL_2585970, EPI_ISL_2585971, EPI_ISL_2585973	Department of Medical Microbiology, Faculty of Medicine, University Malaya, Lembah Pantai, Kuala Lumpur	Department of Medical Microbiology, Faculty of Medicine, University Malaya, Lembah Pantai, Kuala Lumpur	Khor,C.S., Sam,I.C. and Chan,Y.F.
EPI_ISL_2585977, EPI_ISL_2585979, EPI_ISL_2586005, EPI_ISL_2586007, EPI_ISL_2586008	Izumi Kushibuchi Tochigi prefectural Institute of Public Health and Enviromental Science	Izumi Kushibuchi Tochigi prefectural Institute of Public Health and Enviromental Science	Kushibuchi,I. and Kobayashi,M.
EPI_ISL_2586036, EPI_ISL_2586038, EPI_ISL_2586041, EPI_ISL_2586050	Centre for Respiratory Diseases and Meningitis, National Institute for Communicable Diseases	Centre for Respiratory Diseases and Meningitis, National Institute for Communicable Diseases	Pretorius,M.A., van Niekerk,S., Tempia,S., Moyes,J., Cohen,C., Madhi,S.A. and Venter,M.
EPI_ISL_2586071, EPI_ISL_2586073, EPI_ISL_2586075, EPI_ISL_2586077, EPI_ISL_2586079, EPI_ISL_2586081, EPI_ISL_2586082, EPI_ISL_2586084, EPI_ISL_2586086, EPI_ISL_2586088, EPI_ISL_2586089			
see above	Department of Medical Microbiology, Faculty of Medicine, University Malaya, Lembah Pantai, Kuala Lumpur	Department of Medical Microbiology, Faculty of Medicine, University Malaya, Lembah Pantai, Kuala Lumpur	Khor,C.S., Sam,I.C. and Chan,Y.F.
EPI_ISL_2586095, EPI_ISL_2586096, EPI_ISL_2586098, EPI_ISL_2586101, EPI_ISL_2586103	Izumi Kushibuchi Tochigi prefectural Institute of Public Health and Enviromental Science	Izumi Kushibuchi Tochigi prefectural Institute of Public Health and Enviromental Science	Kushibuchi,I. and Kobayashi,M.
EPI_ISL_2586112, EPI_ISL_2586114, EPI_ISL_2586116, EPI_ISL_2586118, EPI_ISL_2586120, EPI_ISL_2586122, EPI_ISL_2586124, EPI_ISL_2586145, EPI_ISL_2586147, EPI_ISL_2586148, EPI_ISL_2586150			
see above	Centre for Respiratory Diseases and Meningitis, National Institute for Communicable Diseases	Centre for Respiratory Diseases and Meningitis, National Institute for Communicable Diseases	Pretorius,M.A., van Niekerk,S., Tempia,S., Moyes,J., Cohen,C., Madhi,S.A. and Venter,M.
EPI_ISL_2586198, EPI_ISL_2586200	Department of Medical Microbiology, Faculty of Medicine, University Malaya, Lembah Pantai, Kuala Lumpur	Department of Medical Microbiology, Faculty of Medicine, University Malaya, Lembah Pantai, Kuala Lumpur	Khor,C.S., Sam,I.C. and Chan,Y.F.
EPI_ISL_2586202, EPI_ISL_2586204	Michiko Okamoto Department of Virology, Tohoku university Graduate school of medicine	Michiko Okamoto Department of Virology, Tohoku university Graduate school of medicine	Suzuki,A., Ohno,A., Oshitani,H. and Okamoto,M.
EPI_ISL_2586238, EPI_ISL_2586243	Akira Suzuki Touhoku university, Dpartment of virology	Akira Suzuki Touhoku university, Dpartment of virology	Ohno,A., Suzuki,A. and Oshitani,H.
EPI_ISL_2586245, EPI_ISL_2586247, EPI_ISL_2586249, EPI_ISL_2586251, EPI_ISL_2586252, EPI_ISL_2586254, EPI_ISL_2586256, EPI_ISL_2586257, EPI_ISL_2586259, EPI_ISL_2586261, EPI_ISL_2586263, EPI_ISL_2586265, EPI_ISL_2586267, EPI_ISL_2586268, EPI_ISL_2586270, EPI_ISL_2586273, EPI_ISL_2586275, EPI_ISL_2586277, EPI_ISL_2586278, EPI_ISL_2586311, EPI_ISL_2586313, EPI_ISL_2586315, EPI_ISL_2586317, EPI_ISL_2586319, EPI_ISL_2586320, EPI_ISL_2586322, EPI_ISL_2586324, EPI_ISL_2586326, EPI_ISL_2586328, EPI_ISL_2586330, EPI_ISL_2586332, EPI_ISL_2586334, EPI_ISL_2586336, EPI_ISL_2586338, EPI_ISL_2586340, EPI_ISL_2586342, EPI_ISL_2586344			
see above	Centre for Respiratory Diseases and Meningitis, National Institute for Communicable Diseases	Centre for Respiratory Diseases and Meningitis, National Institute for Communicable Diseases	Pretorius,M.A., van Niekerk,S., Tempia,S., Moyes,J., Cohen,C., Madhi,S.A. and Venter,M.
EPI_ISL_2586384, EPI_ISL_2586386, EPI_ISL_2586387, EPI_ISL_2586389, EPI_ISL_2586391, EPI_ISL_2586393	Department of Medical Microbiology, Faculty of Medicine, University Malaya, Lembah Pantai, Kuala Lumpur	Department of Medical Microbiology, Faculty of Medicine, University Malaya, Lembah Pantai, Kuala Lumpur	Khor,C.S., Sam,I.C. and Chan,Y.F.
EPI_ISL_2586397	Lab. Virologia. Depto. Microbiologia, Universidad Autonoma de San Luis Potosi	Lab. Virologia. Depto. Microbiologia, Universidad Autonoma de San Luis Potosi	Comas-Garcia,A., Noyola,D.E., Cadena-Mota,S., Rico-Hernandez,M. and Bernal-Silva,S.
EPI_ISL_2586399, EPI_ISL_2586400, EPI_ISL_2586402, EPI_ISL_2586404, EPI_ISL_2586406, EPI_ISL_2586408, EPI_ISL_2586409, EPI_ISL_2586411, EPI_ISL_2586413, EPI_ISL_2586415, EPI_ISL_2586417, EPI_ISL_2586419, EPI_ISL_2586421, EPI_ISL_2586423, EPI_ISL_2586425, EPI_ISL_2586426, EPI_ISL_2586428, EPI_ISL_2586430, EPI_ISL_2586432, EPI_ISL_2586433, EPI_ISL_2586435, EPI_ISL_2586437, EPI_ISL_2586439, EPI_ISL_2586441, EPI_ISL_2586442			
see above	Michiko Okamoto Department of Virology, Tohoku university Graduate school of medicine	Michiko Okamoto Department of Virology, Tohoku university Graduate school of medicine	Suzuki,A., Ohno,A., Oshitani,H. and Okamoto,M.
EPI_ISL_2586537, EPI_ISL_2586540, EPI_ISL_2586541, EPI_ISL_2586543, EPI_ISL_2586546, EPI_ISL_2586547, EPI_ISL_2586549, EPI_ISL_2586552, EPI_ISL_2586553	Akira Suzuki Touhoku university, Dpartment of virology	Akira Suzuki Touhoku university, Dpartment of virology	Ohno,A., Suzuki,A. and Oshitani,H.
EPI_ISL_2586555	Department of Medical Microbiology, Faculty of Medicine, University Malaya, Lembah Pantai, Kuala Lumpur	Department of Medical Microbiology, Faculty of Medicine, University Malaya, Lembah Pantai, Kuala Lumpur	Khor,C.S., Sam,I.C. and Chan,Y.F.
EPI_ISL_2586558, EPI_ISL_2586560, EPI_ISL_2586562, EPI_ISL_2586564, EPI_ISL_2586566, EPI_ISL_2586569, EPI_ISL_2586571, EPI_ISL_2586573, EPI_ISL_2586575, EPI_ISL_2586577	Centre for Respiratory Diseases and Meningitis, National Institute for Communicable Diseases	Centre for Respiratory Diseases and Meningitis, National Institute for Communicable Diseases	Pretorius,M.A., van Niekerk,S., Tempia,S., Moyes,J., Cohen,C., Madhi,S.A. and Venter,M.
EPI_ISL_2586587, EPI_ISL_2586589, EPI_ISL_2586591, EPI_ISL_2586592, EPI_ISL_2586594, EPI_ISL_2586596, EPI_ISL_2586598, EPI_ISL_2586600, EPI_ISL_2586602, EPI_ISL_2586604, EPI_ISL_2586608, EPI_ISL_2586610, EPI_ISL_2586612			
see above	Department of Medical Microbiology, Faculty of Medicine, University Malaya, Lembah Pantai, Kuala Lumpur	Department of Medical Microbiology, Faculty of Medicine, University Malaya, Lembah Pantai, Kuala Lumpur	Khor,C.S., Sam,I.C. and Chan,Y.F.
EPI_ISL_2586614, EPI_ISL_2586616, EPI_ISL_2586618, EPI_ISL_2586620, EPI_ISL_2586622, EPI_ISL_2586623, EPI_ISL_2586625, EPI_ISL_2586627, EPI_ISL_2586629, EPI_ISL_2586631, EPI_ISL_2586632, EPI_ISL_2586634			
see above	Akira Suzuki Touhoku university, Dpartment of virology	Akira Suzuki Touhoku university, Dpartment of virology	Ohno,A., Suzuki,A. and Oshitani,H.
EPI_ISL_2586636, EPI_ISL_2586638, EPI_ISL_2586640, EPI_ISL_2586642, EPI_ISL_2586643, EPI_ISL_2586645, EPI_ISL_2586649, EPI_ISL_2586651, EPI_ISL_2586653, EPI_ISL_2586655, EPI_ISL_2586656, EPI_ISL_2586658, EPI_ISL_2586660, EPI_ISL_2586662, EPI_ISL_2586663, EPI_ISL_2586665, EPI_ISL_2586667, EPI_ISL_2586669, EPI_ISL_2586671, EPI_ISL_2586673, EPI_ISL_2586674, EPI_ISL_2586676,			

EPI_ISL_2586677, EPI_ISL_2586679, EPI_ISL_2586680, EPI_ISL_2586682, EPI_ISL_2586684, EPI_ISL_2586686			
see above	Centre for Respiratory Diseases and Meningitis, National Institute for Communicable Diseases	Centre for Respiratory Diseases and Meningitis, National Institute for Communicable Diseases	Pretorius,M.A., van Niekerk,S., Tempia,S., Moyes,J., Cohen,C., Madhi,S.A. and Venter,M.
EPI_ISL_2586708, EPI_ISL_2586710, EPI_ISL_2586719, EPI_ISL_2586721, EPI_ISL_2586723, EPI_ISL_2586727, EPI_ISL_2586728, EPI_ISL_2586730, EPI_ISL_2586733, EPI_ISL_2586735	Naoko Kiyota Kumamoto Prefectural Institute of Public-Health and Environmental Science, Department of Microbiology	Naoko Kiyota Kumamoto Prefectural Institute of Public-Health and Environmental Science, Department of Microbiology	Kiyota,N., Yoshida,A. and Kobayashi,M.
EPI_ISL_2586744	Pediatrics, Center of Excellence in Clinical Virology, Faculty of Medicine, Chulalongkorn University	Pediatrics, Center of Excellence in Clinical Virology, Faculty of Medicine, Chulalongkorn University	Auksornkitti,V., Kamprasert,N., Thongkomplew,S., Suwannakarn,K., Theamboonlers,A., Samransamruajkij,R. and Poovorawan,Y.
EPI_ISL_2586817	Division of Respiratory Viruses, Center for Infectious Diseases, Korea National Institute of Health	Division of Respiratory Viruses, Center for Infectious Diseases, Korea National Institute of Health	Kim,Y.-J., Lee,W.J., Lee,H.S., Lee,H.Y. and Kim,K.
EPI_ISL_2586834	Influenza and Other Respiratory Viruses Unit, National Center for Microbiology, Instituto de Salud Carlos III	Influenza and Other Respiratory Viruses Unit, National Center for Microbiology, Instituto de Salud Carlos III	Casas,I., Calderon,A., Gonzalez,M., Molinero,M. and Pozo,F.
EPI_ISL_2586848	Department of Botany and Microbiology, King Saud University, College of Science	Department of Botany and Microbiology, King Saud University, College of Science	Ali,G., Amer,H.M. and Almajhdi,F.N.
EPI_ISL_2586853	Medical Microbiology (Interdisciplinary Program), Graduate School, Chulalongkorn University	Medical Microbiology (Interdisciplinary Program), Graduate School, Chulalongkorn University	Nilwong,O., Bhattarakosol,P. and Kowitdamrong,E.
EPI_ISL_2586855	Department of Protein Engineering, Latvian Biomedical Research and Study Centre	Department of Protein Engineering, Latvian Biomedical Research and Study Centre	Balmaks,R., Gardovska,D. and Kazaks,A.
EPI_ISL_2586858	Centre for Respiratory Diseases and Meningitis, National Institute for Communicable Diseases	Centre for Respiratory Diseases and Meningitis, National Institute for Communicable Diseases	Pretorius,M.A., van Niekerk,S., Tempia,S., Moyes,J., Cohen,C., Madhi,S.A. and Venter,M.
EPI_ISL_2586860, EPI_ISL_2586862	Influenza and Other Respiratory Viruses Unit, National Center for Microbiology, Instituto de Salud Carlos III	Influenza and Other Respiratory Viruses Unit, National Center for Microbiology, Instituto de Salud Carlos III	Casas,I., Calderon,A., Gonzalez,M., Molinero,M. and Pozo,F.
EPI_ISL_2586868	Paediatrics and Child Health / Biological and Biomedical Sciences, Aga Khan University Hospital	Paediatrics and Child Health / Biological and Biomedical Sciences, Aga Khan University Hospital	Aziz,F., Ali,S., Kazmi,S.U., Ali,S.A. and Abidi,S.H.
EPI_ISL_2586870	Department of Protein Engineering, Latvian Biomedical Research and Study Centre	Department of Protein Engineering, Latvian Biomedical Research and Study Centre	Balmaks,R., Gardovska,D. and Kazaks,A.
EPI_ISL_2586874, EPI_ISL_2586875, EPI_ISL_2586877	Centre for Respiratory Diseases and Meningitis, National Institute for Communicable Diseases	Centre for Respiratory Diseases and Meningitis, National Institute for Communicable Diseases	Pretorius,M.A., van Niekerk,S., Tempia,S., Moyes,J., Cohen,C., Madhi,S.A. and Venter,M.
EPI_ISL_2586879	Influenza and Other Respiratory Viruses Unit, National Center for Microbiology, Instituto de Salud Carlos III	Influenza and Other Respiratory Viruses Unit, National Center for Microbiology, Instituto de Salud Carlos III	Casas,I., Calderon,A., Gonzalez,M., Molinero,M. and Pozo,F.
EPI_ISL_2586893	Botany and Microbiology, College of Science, King Saud University	Botany and Microbiology, College of Science, King Saud University	Farrag,M.A., Amer,H.M. and Almajhdi,F.N.
EPI_ISL_2586897, EPI_ISL_2586898, EPI_ISL_2586900, EPI_ISL_2586902, EPI_ISL_2586904, EPI_ISL_2586906, EPI_ISL_2586908, EPI_ISL_2586910, EPI_ISL_2586912, EPI_ISL_2586914, EPI_ISL_2586916			
see above	Centre for Respiratory Diseases and Meningitis, National Institute for Communicable Diseases	Centre for Respiratory Diseases and Meningitis, National Institute for Communicable Diseases	Pretorius,M.A., van Niekerk,S., Tempia,S., Moyes,J., Cohen,C., Madhi,S.A. and Venter,M.
EPI_ISL_2586918, EPI_ISL_2586920, EPI_ISL_2586922, EPI_ISL_2586924, EPI_ISL_2586925, EPI_ISL_2586927	Influenza and Other Respiratory Viruses Unit, National Center for Microbiology, Instituto de Salud Carlos III	Influenza and Other Respiratory Viruses Unit, National Center for Microbiology, Instituto de Salud Carlos III	Casas,I., Calderon,A., Gonzalez,M., Molinero,M. and Pozo,F.
EPI_ISL_2586940, EPI_ISL_2586941	Medical Microbiology (Interdisciplinary Program), Graduate School, Chulalongkorn University	Medical Microbiology (Interdisciplinary Program), Graduate School, Chulalongkorn University	Nilwong,O., Bhattarakosol,P. and Kowitdamrong,E.
EPI_ISL_2586948, EPI_ISL_2586949, EPI_ISL_2586951, EPI_ISL_2586953	Department of Protein Engineering, Latvian Biomedical Research and Study Centre	Department of Protein Engineering, Latvian Biomedical Research and Study Centre	Balmaks,R., Gardovska,D. and Kazaks,A.
EPI_ISL_2586955	Departament of Microbiology, University of Sao Paulo Institute of Biomedical Science	Departament of Microbiology, University of Sao Paulo Institute of Biomedical Science	Espinola,E.E.
EPI_ISL_2586957	Department of Botany and Microbiology, King Saud University, College of Science	Department of Botany and Microbiology, King Saud University, College of Science	Ali,G., Amer,H.M. and Almajhdi,F.N.
EPI_ISL_2586962, EPI_ISL_2586964, EPI_ISL_2586966, EPI_ISL_2586968, EPI_ISL_2586970, EPI_ISL_2586972, EPI_ISL_2586974, EPI_ISL_2586976, EPI_ISL_2586978, EPI_ISL_2586979, EPI_ISL_2586981, EPI_ISL_2586983, EPI_ISL_2586985, EPI_ISL_2586987, EPI_ISL_2586989, EPI_ISL_2586990, EPI_ISL_2586992, EPI_ISL_2586994, EPI_ISL_2586995, EPI_ISL_2586997, EPI_ISL_2586999, EPI_ISL_2587000, EPI_ISL_2587002, EPI_ISL_2587004, EPI_ISL_2587006, EPI_ISL_2587007, EPI_ISL_2587009, EPI_ISL_2587010, EPI_ISL_2587012, EPI_ISL_2587014, EPI_ISL_2587016, EPI_ISL_2587018, EPI_ISL_2587020, EPI_ISL_2587022, EPI_ISL_2587028, EPI_ISL_2587030, EPI_ISL_2587032, EPI_ISL_2587034, EPI_ISL_2587036, EPI_ISL_2587038, EPI_ISL_2587040			
see above	Centre for Respiratory Diseases and Meningitis, National Institute for Communicable Diseases	Centre for Respiratory Diseases and Meningitis, National Institute for Communicable Diseases	Pretorius,M.A., van Niekerk,S., Tempia,S., Moyes,J., Cohen,C., Madhi,S.A. and Venter,M.
EPI_ISL_2587049, EPI_ISL_2587050, EPI_ISL_2587052, EPI_ISL_2587053, EPI_ISL_2587055, EPI_ISL_2587056, EPI_ISL_2587058, EPI_ISL_2587060, EPI_ISL_2587062, EPI_ISL_2587064, EPI_ISL_2587066, EPI_ISL_2587068			
see above	Influenza and Other Respiratory Viruses Unit, National Center for Microbiology, Instituto de Salud Carlos III	Influenza and Other Respiratory Viruses Unit, National Center for Microbiology, Instituto de Salud Carlos III	Casas,I., Calderon,A., Gonzalez,M., Molinero,M. and Pozo,F.
EPI_ISL_2587087, EPI_ISL_2587088, EPI_ISL_2587090, EPI_ISL_2587092	Medical Microbiology (Interdisciplinary Program), Graduate School, Chulalongkorn University	Medical Microbiology (Interdisciplinary Program), Graduate School, Chulalongkorn University	Nilwong,O., Bhattarakosol,P. and Kowitdamrong,E.
EPI_ISL_2587094, EPI_ISL_2587096, EPI_ISL_2587098	Department of Protein Engineering, Latvian Biomedical Research and Study Centre	Department of Protein Engineering, Latvian Biomedical Research and Study Centre	Balmaks,R., Gardovska,D. and Kazaks,A.
EPI_ISL_2587100	Department of Botany and Microbiology, King Saud University, College of Science	Department of Botany and Microbiology, King Saud University, College of Science	Ali,G., Amer,H.M. and Almajhdi,F.N.
EPI_ISL_2587102, EPI_ISL_2587104, EPI_ISL_2587106	Botany and Microbiology, College of Science, King Saud University	Botany and Microbiology, College of Science, King Saud University	Farrag,M.A., Amer,H.M. and Almajhdi,F.N.
EPI_ISL_2587107, EPI_ISL_2587109, EPI_ISL_2587111, EPI_ISL_2587114, EPI_ISL_2587116, EPI_ISL_2587117, EPI_ISL_2587119, EPI_ISL_2587121	Centre for Respiratory Diseases and Meningitis, National Institute for Communicable Diseases	Centre for Respiratory Diseases and Meningitis, National Institute for Communicable Diseases	Pretorius,M.A., van Niekerk,S., Tempia,S., Moyes,J., Cohen,C., Madhi,S.A. and Venter,M.
EPI_ISL_2587124, EPI_ISL_2587125, EPI_ISL_2587127, EPI_ISL_2587129, EPI_ISL_2587130	Influenza and Other Respiratory Viruses Unit, National Center for Microbiology, Instituto de Salud Carlos III	Influenza and Other Respiratory Viruses Unit, National Center for Microbiology, Instituto de Salud Carlos III	Casas,I., Calderon,A., Gonzalez,M., Molinero,M. and Pozo,F.
EPI_ISL_2587141, EPI_ISL_2587152, EPI_ISL_2587154, EPI_ISL_2587155	Medical Microbiology (Interdisciplinary Program), Graduate School, Chulalongkorn University	Medical Microbiology (Interdisciplinary Program), Graduate School, Chulalongkorn University	Nilwong,O., Bhattarakosol,P. and Kowitdamrong,E.
EPI_ISL_2587157	Departament of Microbiology, University of Sao Paulo Institute of Biomedical Science	Departament of Microbiology, University of Sao Paulo Institute of Biomedical Science	Espinola,E.E.
EPI_ISL_2587159	J. Craig Venter Institute	J. Craig Venter Institute	Das,S.R., Halpin,R.A., Shilts,M., Puri,V., Akopov,A., Fedorova,N., Stockwell,T., Amedeo,P., Bishop,B., Katzel,D., Schobel,S., Shrivastava,S. and Hartert,T.
EPI_ISL_2587163	J. Craig Venter Institute	J. Craig Venter Institute	Shabman,R., Das,S.R., Puri,V., Fedorova,N., Amedeo,P., Williams,M., Shrivastava,S. and Halasa,N.
EPI_ISL_2587340	Wan Ji Lee Korea National Institute of Health, Division of Respiratory Viruses, Center for Infectious Diseases	Wan Ji Lee Korea National Institute of Health, Division of Respiratory Viruses, Center for Infectious Diseases	Lee,W.J., Kim,Y.J., Kim,D.W., Lee,H.Y., Yun,M.R., Lee,H.S., Jung,H.D. and Kim,K.
EPI_ISL_2587364	J. Craig Venter Institute	J. Craig Venter Institute	Das,S.R., Halpin,R.A., Shilts,M., Puri,V., Akopov,A., Fedorova,N., Stockwell,T., Amedeo,P., Bishop,B., Katzel,D., Schobel,S., Shrivastava,S. and Hartert,T.
EPI_ISL_2587373	Epidemiology and Demography Department, KEMRI - Wellcome Trust Research Collaborative Programme	Epidemiology and Demography Department, KEMRI - Wellcome Trust Research Collaborative Programme	Agoti,C.N., Munywoki,P.K., Phan,M.V.T., Otieno,J.R., Kamau,E., Bett,A., Githinji,G., Medley,G.F., Cane,P.A., Kellam,P., Cotten,M.L. and Nokes,D.J.
EPI_ISL_2587389	J. Craig Venter Institute	J. Craig Venter Institute	Shabman,R., Das,S.R., Puri,V., Fedorova,N., Amedeo,P., Williams,M., Shrivastava,S. and Halasa,N.
EPI_ISL_2587471, EPI_ISL_2587473	J. Craig Venter Institute	J. Craig Venter Institute	Das,S.R., Halpin,R.A., Shilts,M., Puri,V., Akopov,A., Fedorova,N., Stockwell,T., Amedeo,P., Bishop,B., Katzel,D., Schobel,S., Shrivastava,S. and Hartert,T.
EPI_ISL_2587590	ARI Unit, Center for Virus Research, Kenya Medical Research Institute	ARI Unit, Center for Virus Research, Kenya Medical Research Institute	Symekher,S.M.L., Gachara,G.M., Gikera,C., Magana,J.M., Simwa,J.M., Otieno,M.N. and Ochieng,W.O.
EPI_ISL_2587595	Centre for Respiratory Diseases and Meningitis, National Institute for Communicable Diseases	Centre for Respiratory Diseases and Meningitis, National Institute for Communicable Diseases	Pretorius,M.A., van Niekerk,S., Tempia,S., Moyes,J., Cohen,C., Madhi,S.A. and Venter,M.
EPI_ISL_2587624	Departament of Microbiology, University of Sao Paulo Institute of Biomedical Science	Departament of Microbiology, University of Sao Paulo Institute of Biomedical Science	Espinola,E.E.
EPI_ISL_2587626	Centre for Respiratory Diseases and Meningitis, National Institute for Communicable Diseases	Centre for Respiratory Diseases and Meningitis, National Institute for Communicable Diseases	Pretorius,M.A., van Niekerk,S., Tempia,S., Moyes,J., Cohen,C., Madhi,S.A. and Venter,M.
EPI_ISL_2587630, EPI_ISL_2587632	ARI Unit, Center for Virus Research, Kenya Medical Research Institute	ARI Unit, Center for Virus Research, Kenya Medical Research Institute	Symekher,S.M.L., Gachara,G.M., Gikera,C., Magana,J.M., Simwa,J.M., Otieno,M.N. and Ochieng,W.O.
EPI_ISL_2587643	Centre for Respiratory Diseases and Meningitis, National Institute for Communicable Diseases	Centre for Respiratory Diseases and Meningitis, National Institute for Communicable Diseases	Pretorius,M.A., van Niekerk,S., Tempia,S., Moyes,J., Cohen,C., Madhi,S.A. and Venter,M.
EPI_ISL_2587900	Virology, NAMRU-40	Virology, NAMRU-40	Garcia,J., Sovero,M. and Halsey,E.S.
EPI_ISL_2587905	ARI Unit, Center for Virus Research, Kenya Medical Research Institute	ARI Unit, Center for Virus Research, Kenya Medical Research Institute	Symekher,S.M.L., Gachara,G.M., Gikera,C., Magana,J.M., Simwa,J.M., Otieno,M.N. and Ochieng,W.O.
EPI_ISL_2587919	Virology, NAMRU-21	Virology, NAMRU-21	Garcia,J., Sovero,M. and Halsey,E.S.
EPI_ISL_2587920	Virology, NAMRU-11	Virology, NAMRU-11	Garcia,J., Sovero,M. and Halsey,E.S.
EPI_ISL_2587924	Virology, NAMRU-31	Virology, NAMRU-31	Garcia,J., Sovero,M. and Halsey,E.S.
EPI_ISL_2587940, EPI_ISL_2587941	Department of Medical Microbiology, Faculty of Medicine, University Malaya, Lembah Pantai, Kuala Lumpur	Department of Medical Microbiology, Faculty of Medicine, University Malaya, Lembah Pantai, Kuala Lumpur	Khor,C.S., Sam,I.C. and Chan,Y.F.
EPI_ISL_2587950, EPI_ISL_2587951, EPI_ISL_2587954	ARI Unit, Center for Virus Research, Kenya Medical Research Institute	ARI Unit, Center for Virus Research, Kenya Medical Research Institute	Symekher,S.M.L., Gachara,G.M., Gikera,C., Magana,J.M., Simwa,J.M., Otieno,M.N. and Ochieng,W.O.

EPI_ISL_2587963, EPI_ISL_2587965	Centre for Respiratory Diseases and Meningitis, National Institute for Communicable Diseases	Centre for Respiratory Diseases and Meningitis, National Institute for Communicable Diseases	Pretorius,M.A., van Niekerk,S., Tempia,S., Moyes,J., Cohen,C., Madhi,S.A. and Venter,M.
EPI_ISL_2587967	Biology, Gilead Sciences	Biology, Gilead Sciences	Stray,K.
EPI_ISL_2587989	Virology, NAMRU-14	Virology, NAMRU-14	Garcia,J., Sovero,M. and Halsey,E.S.
EPI_ISL_2587990	Virology, NAMRU-37	Virology, NAMRU-37	Garcia,J., Sovero,M. and Halsey,E.S.
EPI_ISL_2587991	ARI Unit, Center for Virus Research, Kenya Medical Research Institute	ARI Unit, Center for Virus Research, Kenya Medical Research Institute	Symekher,S.M.L., Gachara,G.M., Gikera,C., Magana,J.M., Simwa,J.M., Otieno,M.N. and Ochieng,W.O.
EPI_ISL_2587992	Center for Virus Research, ARI Unit, Kenya Medical Research Institute	Center for Virus Research, ARI Unit, Kenya Medical Research Institute	Symekher,S.M.L., Muthoni,S.M., Ochieng,W.O., Simwa,J.M. and Gachara,G.M.
EPI_ISL_2587994	ARI Unit, Center for Virus Research, Kenya Medical Research Institute	ARI Unit, Center for Virus Research, Kenya Medical Research Institute	Symekher,S.M.L., Gachara,G.M., Gikera,C., Magana,J.M., Simwa,J.M., Otieno,M.N. and Ochieng,W.O.
EPI_ISL_2587998	Department of Medical Microbiology, Faculty of Medicine, University Malaya, Lembah Pantai, Kuala Lumpur	Department of Medical Microbiology, Faculty of Medicine, University Malaya, Lembah Pantai, Kuala Lumpur	Khor,C.S., Sam,I.C. and Chan,Y.F.
EPI_ISL_2587999, EPI_ISL_2588002	ARI Unit, Center for Virus Research, Kenya Medical Research Institute	ARI Unit, Center for Virus Research, Kenya Medical Research Institute	Symekher,S.M.L., Gachara,G.M., Gikera,C., Magana,J.M., Simwa,J.M., Otieno,M.N. and Ochieng,W.O.
EPI_ISL_2588003	Centre for Respiratory Diseases and Meningitis, National Institute for Communicable Diseases	Centre for Respiratory Diseases and Meningitis, National Institute for Communicable Diseases	Pretorius,M.A., van Niekerk,S., Tempia,S., Moyes,J., Cohen,C., Madhi,S.A. and Venter,M.
EPI_ISL_2588016	ARI Unit, Center for Virus Research, Kenya Medical Research Institute	ARI Unit, Center for Virus Research, Kenya Medical Research Institute	Symekher,S.M.L., Gachara,G.M., Gikera,C., Magana,J.M., Simwa,J.M., Otieno,M.N. and Ochieng,W.O.
EPI_ISL_2588036	Virology, NAMRU-18	Virology, NAMRU-18	Garcia,J., Sovero,M. and Halsey,E.S.
EPI_ISL_2588037	Virology, NAMRU-49	Virology, NAMRU-49	Garcia,J., Sovero,M. and Halsey,E.S.
EPI_ISL_2588038	Virology, NAMRU-23	Virology, NAMRU-23	Garcia,J., Sovero,M. and Halsey,E.S.
EPI_ISL_2588039	Virology, NAMRU-52	Virology, NAMRU-52	Garcia,J., Sovero,M. and Halsey,E.S.
EPI_ISL_2588040	Virology, NAMRU-29	Virology, NAMRU-29	Garcia,J., Sovero,M. and Halsey,E.S.
EPI_ISL_2588041	Biology, Gilead Sciences	Biology, Gilead Sciences	Stray,K.
EPI_ISL_2588048, EPI_ISL_2588049, EPI_ISL_2588050, EPI_ISL_2588051, EPI_ISL_2588052	Centre for Respiratory Diseases and Meningitis, National Institute for Communicable Diseases	Centre for Respiratory Diseases and Meningitis, National Institute for Communicable Diseases	Pretorius,M.A., van Niekerk,S., Tempia,S., Moyes,J., Cohen,C., Madhi,S.A. and Venter,M.
EPI_ISL_2588089	Virology, Public Health Institution of Turkey	Virology, Public Health Institution of Turkey	Bayrakdar,F.
EPI_ISL_2588092	Virology, NAMRU-51	Virology, NAMRU-51	Garcia,J., Sovero,M. and Halsey,E.S.
EPI_ISL_2588093	Virology, NAMRU-19	Virology, NAMRU-19	Garcia,J., Sovero,M. and Halsey,E.S.
EPI_ISL_2588094	Virology, NAMRU-20	Virology, NAMRU-20	Garcia,J., Sovero,M. and Halsey,E.S.
EPI_ISL_2588109, EPI_ISL_2588110, EPI_ISL_2588111, EPI_ISL_2588112	Microbiology Department, Virology Division, College of Medicine, Taif University	Microbiology Department, Virology Division, College of Medicine, Taif University	Abdel-Moneim,A.S., Shehab,G.M., Alsulaimani,A.A., Al-Malky,M.I.R. and Kamel,M.M.
EPI_ISL_2588145	Departament of Microbiology, University of Sao Paulo Institute of Biomedical Science	Departament of Microbiology, University of Sao Paulo Institute of Biomedical Science	Espinola,E.E.
EPI_ISL_2588150, EPI_ISL_2588151, EPI_ISL_2588152, EPI_ISL_2588153	Influenza and Other Respiratory Viruses Unit, National Center for Microbiology, Instituto de Salud Carlos III	Influenza and Other Respiratory Viruses Unit, National Center for Microbiology, Instituto de Salud Carlos III	Casas,I., Calderon,A., Gonzalez,M., Molinero,M. and Pozo,F.
EPI_ISL_2588165, EPI_ISL_2588198, EPI_ISL_2588199, EPI_ISL_2588200, EPI_ISL_2588201, EPI_ISL_2588202	Miwako SaikNorth America / USA Yokohama City Institute of Public Health	Miwako SaikNorth America / USA Yokohama City Institute of Public Health	SaikNorth America / USA,M., Kawakami,C., Usuku,S., Sasao,T. and Okubo,I.
EPI_ISL_2588217	Botany and Microbiology, College of Science, King Saud University	Botany and Microbiology, College of Science, King Saud University	Farrag,M.A., Amer,H.M. and Almajhdi,F.N.
EPI_ISL_2588218	Biology, Gilead Sciences	Biology, Gilead Sciences	Stray,K.
EPI_ISL_2588219	Miwako SaikNorth America / USA Yokohama City Institute of Health	Miwako SaikNorth America / USA Yokohama City Institute of Health	SaikNorth America / USA,M., Momoki,T., Kawakami,C., Usuku,S., Morita,M. and Mizuno,T.
EPI_ISL_2588220, EPI_ISL_2588221	Miwako SaikNorth America / USA Yokohama City Institute of Health	Miwako SaikNorth America / USA Yokohama City Institute of Health	SaikNorth America / USA,M., Kawakami,C., Usuku,S., Morita,M. and Mizuno,T.
EPI_ISL_2588223	Centre for Respiratory Diseases and Meningitis, National Institute for Communicable Diseases	Centre for Respiratory Diseases and Meningitis, National Institute for Communicable Diseases	Pretorius,M.A., van Niekerk,S., Tempia,S., Moyes,J., Cohen,C., Madhi,S.A. and Venter,M.
EPI_ISL_2588224, EPI_ISL_2588225, EPI_ISL_2588226, EPI_ISL_2588227, EPI_ISL_2588228, EPI_ISL_2588229, EPI_ISL_2588230	Influenza and Other Respiratory Viruses Unit, National Center for Microbiology, Instituto de Salud Carlos III	Influenza and Other Respiratory Viruses Unit, National Center for Microbiology, Instituto de Salud Carlos III	Casas,I., Calderon,A., Gonzalez,M., Molinero,M. and Pozo,F.
EPI_ISL_2588257, EPI_ISL_2588259, EPI_ISL_2588260, EPI_ISL_2588261	Laboratorio de Biologia Viral, Instituto de Salud Carlos III	Laboratorio de Biologia Viral, Instituto de Salud Carlos III	Trento,A., Abrego,L., Rodriguez-Fernandez,R., Gonzalez-Sanchez,M.I., Gonzalez-Martinez,F., Delfaro,A., Pascale,J.M., Arbiza,J. and Melero,J.A.
EPI_ISL_2588296, EPI_ISL_2588299, EPI_ISL_2588300	Medical Microbiology (Interdisciplinary Program), Graduate School, Chulalongkorn University	Medical Microbiology (Interdisciplinary Program), Graduate School, Chulalongkorn University	Nilwong,O., Bhattarakosol,P. and Kowitdamrong,E.
EPI_ISL_2588301, EPI_ISL_2588305, EPI_ISL_2588306	J. Craig Venter Institute	J. Craig Venter Institute	Das,S.R., Halpin,R.A., Shilts,M., Puri,V., Akopov,A., Fedorova,N., Stockwell,T., Amedeo,P., Bishop,B., Katzel,D., Schobel,S., Shrivastava,S. and Hartert,T.
EPI_ISL_2588307, EPI_ISL_2588308, EPI_ISL_2588309, EPI_ISL_2588310	Miwako SaikNorth America / USA Yokohama City Institute of Public Health	Miwako SaikNorth America / USA Yokohama City Institute of Public Health	SaikNorth America / USA,M., Kawakami,C., Usuku,S., Sasao,T. and Okubo,I.
EPI_ISL_2588348	J. Craig Venter Institute	J. Craig Venter Institute	Das,S., Halpin,R.A., Bera,J., Puri,V., Fedorova,N., Tsitrin,T., Stockwell,T., Amedeo,P., Bishop,B., Katzel,D., Schobel,S., Shrivastava,S., Hartert,T., Moore,M., Chappell,J., Larkin,E., Wentworth,D.E. and Anderson,L.J.
EPI_ISL_2588353, EPI_ISL_2588354, EPI_ISL_2588355	J. Craig Venter Institute	J. Craig Venter Institute	Das,S.R., Halpin,R.A., Shilts,M., Puri,V., Akopov,A., Fedorova,N., Stockwell,T., Amedeo,P., Bishop,B., Katzel,D., Schobel,S., Shrivastava,S. and Hartert,T.
EPI_ISL_2588357	J. Craig Venter Institute	J. Craig Venter Institute	Das,S., Halpin,R.A., Bera,J., Fedorova,N., Tsitrin,T., Stockwell,T., Amedeo,P., Bishop,B., Gupta,N., Hoover,J., Katzel,D., Schobel,S., Shrivastava,S., Hartert,T., Moore,M., Chappell,J., Larkin,E., Wentworth,D.E. and Anderson,L.J.
EPI_ISL_2588363, EPI_ISL_2588364, EPI_ISL_2588365, EPI_ISL_2588367, EPI_ISL_2588372, EPI_ISL_2588373	J. Craig Venter Institute	J. Craig Venter Institute	Das,S.R., Halpin,R.A., Shilts,M., Puri,V., Akopov,A., Fedorova,N., Stockwell,T., Amedeo,P., Bishop,B., Katzel,D., Schobel,S., Shrivastava,S. and Hartert,T.
EPI_ISL_2588379	J. Craig Venter Institute	J. Craig Venter Institute	Wentworth,D.E., Halpin,R.A., Bera,J., Lin,X., Fedorova,N., Tsitrin,T., McLellan,M., Stockwell,T., Amedeo,P., Bishop,B., Gupta,N., Hoover,J., Katzel,D., Schobel,S., Shrivastava,S., Garcia,J., Laguna-Torres,V.A., Leguia,M., Benavides,J.G. and Halsey,E.
EPI_ISL_2588381, EPI_ISL_2588382, EPI_ISL_2588383, EPI_ISL_2588384, EPI_ISL_2588385	Miwako SaikNorth America / USA Yokohama City Institute of Public Health	Miwako SaikNorth America / USA Yokohama City Institute of Public Health	SaikNorth America / USA,M., Kawakami,C., Usuku,S., Sasao,T. and Okubo,I.
EPI_ISL_2588436	Shinichi Takao Hiroshima Prefectural Technology Research Institute, Center for Public Health and Environment	Shinichi Takao Hiroshima Prefectural Technology Research Institute, Center for Public Health and Environment	Takao,S. and Shimazu,Y.
EPI_ISL_2588437	J. Craig Venter Institute	J. Craig Venter Institute	Shabman,R., Das,S.R., Puri,V., Fedorova,N., Amedeo,P., Williams,M., Shrivastava,S. and Halasa,N.
EPI_ISL_2588438	Department of Medical Microbiology, Faculty of Medicine, University Malaya, Lembah Pantai, Kuala Lumpur	Department of Medical Microbiology, Faculty of Medicine, University Malaya, Lembah Pantai, Kuala Lumpur	Khor,C.S., Sam,I.C. and Chan,Y.F.
EPI_ISL_2588447	J. Craig Venter Institute	J. Craig Venter Institute	Wentworth,D.E., Halpin,R.A., Bera,J., Lin,X., Fedorova,N., Tsitrin,T., McLellan,M., Stockwell,T., Amedeo,P., Bishop,B., Gupta,N., Hoover,J., Katzel,D., Schobel,S., Shrivastava,S., Garcia,J., Laguna-Torres,V.A., Leguia,M., Benavides,J.G. and Halsey,E.
EPI_ISL_2588458, EPI_ISL_2588459	Department of Botany and Microbiology, King Saud University, College of Science	Department of Botany and Microbiology, King Saud University, College of Science	Ali,G., Amer,H.M. and Almajhdi,F.N.
EPI_ISL_2588461, EPI_ISL_2588462, EPI_ISL_2588463, EPI_ISL_2588464, EPI_ISL_2588465, EPI_ISL_2588466, EPI_ISL_2588467, EPI_ISL_2588468, EPI_ISL_2588469, EPI_ISL_2588470, EPI_ISL_2588471, EPI_ISL_2588472, EPI_ISL_2588473, EPI_ISL_2588474, EPI_ISL_2588475, EPI_ISL_2588476, EPI_ISL_2588477, EPI_ISL_2588478, EPI_ISL_2588479, EPI_ISL_2588480, EPI_ISL_2588481, EPI_ISL_2588482, EPI_ISL_2588483, EPI_ISL_2588484, EPI_ISL_2588485, EPI_ISL_2588486, EPI_ISL_2588487, EPI_ISL_2588488, EPI_ISL_2588489, EPI_ISL_2588490, EPI_ISL_2588491, EPI_ISL_2588493, EPI_ISL_2588494, EPI_ISL_2588495, EPI_ISL_2588496, EPI_ISL_2588497, EPI_ISL_2588498, EPI_ISL_2588499, EPI_ISL_2588500, EPI_ISL_2588501, EPI_ISL_2588502, EPI_ISL_2588503, EPI_ISL_2588504, EPI_ISL_2588505, EPI_ISL_2588506, EPI_ISL_2588507, EPI_ISL_2588508, EPI_ISL_2588509, EPI_ISL_2588510, EPI_ISL_2588511, EPI_ISL_2588512, EPI_ISL_2588513, EPI_ISL_2588514, EPI_ISL_2588515, EPI_ISL_2588516, EPI_ISL_2588517, EPI_ISL_2588518, EPI_ISL_2588519			
see above	Centre for Respiratory Diseases and Meningitis, National Institute for Communicable Diseases	Centre for Respiratory Diseases and Meningitis, National Institute for Communicable Diseases	Pretorius,M.A., van Niekerk,S., Tempia,S., Moyes,J., Cohen,C., Madhi,S.A. and Venter,M.
EPI_ISL_2588520	Medical Virology, University of Pretoria	Medical Virology, University of Pretoria	Pretorius,M.A., van Niekerk,S., Tempia,S., Moyes,J., Cohen,C., Madhi,S.A. and Venter,M.
EPI_ISL_2588532, EPI_ISL_2588533, EPI_ISL_2588535, EPI_ISL_2588536, EPI_ISL_2588545, EPI_ISL_2588546, EPI_ISL_2588547, EPI_ISL_2588548, EPI_ISL_2588549	Medical Microbiology (Interdisciplinary Program), Graduate School, Chulalongkorn University	Medical Microbiology (Interdisciplinary Program), Graduate School, Chulalongkorn University	Nilwong,O., Bhattarakosol,P. and Kowitdamrong,E.
EPI_ISL_2588550, EPI_ISL_2588551, EPI_ISL_2588554, EPI_ISL_2588557, EPI_ISL_2588558, EPI_ISL_2588563, EPI_ISL_2588564, EPI_ISL_2588565, EPI_ISL_2588566, EPI_ISL_2588568, EPI_ISL_2588575, EPI_ISL_2588577, EPI_ISL_2588578, EPI_ISL_2588579, EPI_ISL_2588581, EPI_ISL_2588588, EPI_ISL_2588589, EPI_ISL_2588590, EPI_ISL_2588592			
see above	Virology, Public Health Institution of Turkey	Virology, Public Health Institution of Turkey	Bayrakdar,F.
EPI_ISL_2588594	Biology, Gilead Sciences	Biology, Gilead Sciences	Stray,K.
EPI_ISL_2588598, EPI_ISL_2588599	J. Craig Venter Institute	J. Craig Venter Institute	Das,S.R., Halpin,R.A., Shilts,M., Puri,V., Akopov,A., Fedorova,N., Stockwell,T., Amedeo,P., Bishop,B., Katzel,D., Schobel,S., Shrivastava,S. and Hartert,T.
EPI_ISL_2588797	Virology Laboratory, Dr. Ricardo Gutierrez Children Hospital	Virology Laboratory, Dr. Ricardo Gutierrez Children Hospital	Viegas,M., Goya,S. and Mistchenko,A.S.
EPI_ISL_2588870, EPI_ISL_2588871, EPI_ISL_2588872	Virology, Capital Institute of Pediatrics	Virology, Capital Institute of Pediatrics	Deng,J., Zhu,R., Qian,Y., Sun,Y., Zhao,L., Wang,F., Wu,H., Shan,M. and Deji,M.
EPI_ISL_2588873, EPI_ISL_2588874, EPI_ISL_2588876	Molecular Research, Ontario Agency for Health Protection and Promotion	Molecular Research, Ontario Agency for Health Protection and Promotion	Eshaghi,A., Lai,R., Nadarajah,J.T., Li,A., Patel,S.N., Gubbay,J.B. and Low,D.E.
EPI_ISL_2588993, EPI_ISL_2588994	Virology, RMRC, NE Region (ICMR)	Virology, RMRC, NE Region (ICMR)	Biswas,D., Yadav,K., Borkakoty,B. and Mahanta,J.

EPI_ISL_2588995, EPI_ISL_2588996, EPI_ISL_2588997, EPI_ISL_2588998, EPI_ISL_2588999, EPI_ISL_2589000, EPI_ISL_2589001	Virology, Regional Medical Research Centre, NE (ICMR) Dibrugarh	Virology, Regional Medical Research Centre, NE (ICMR) Dibrugarh	Biswas,D., Yadav,K., Borkakoty,B. and Mahanta,J.
EPI_ISL_2589002	Virology, School of Public Health, Tehran University of Medical Sciences	Virology, School of Public Health, Tehran University of Medical Sciences	Yavarian,J., Faghihloo,E. and Mokhtari Azad,T.
EPI_ISL_2589051, EPI_ISL_2589052, EPI_ISL_2589053, EPI_ISL_2589054, EPI_ISL_2589055	Laboratory for Molecular Biomedicine, Centre for Research and Knowledge Transfer in Biotechnology, University of Zagreb	Laboratory for Molecular Biomedicine, Centre for Research and Knowledge Transfer in Biotechnology, University of Zagreb	Slovic,A., Forcic,D., Ivancic-Jelecki,J., Ljubin Sternak,S. and Mlinaric-Galinovic,G.
EPI_ISL_2589070	Virology, School of Public Health, Tehran University of Medical Sciences	Virology, School of Public Health, Tehran University of Medical Sciences	Yavarian,J., Faghihloo,E. and Mokhtari Azad,T.
EPI_ISL_2589074, EPI_ISL_2589076	Clinical Microbiology, Trinity College Dublin	Clinical Microbiology, Trinity College Dublin	Salter,A.C., Crowley,B. and Ni Laoi,B.
EPI_ISL_2589165, EPI_ISL_2589166, EPI_ISL_2589167	Laboratory for Molecular Biomedicine, Centre for Research and Knowledge Transfer in Biotechnology, University of Zagreb	Laboratory for Molecular Biomedicine, Centre for Research and Knowledge Transfer in Biotechnology, University of Zagreb	Slovic,A., Forcic,D., Ivancic-Jelecki,J., Ljubin Sternak,S. and Mlinaric-Galinovic,G.
EPI_ISL_2589213	Virology, Regional Medical Research Centre, NE (ICMR) Dibrugarh	Virology, Regional Medical Research Centre, NE (ICMR) Dibrugarh	Biswas,D., Yadav,K., Borkakoty,B. and Mahanta,J.
EPI_ISL_2589240	Laboratory for Molecular Biomedicine, Centre for Research and Knowledge Transfer in Biotechnology, University of Zagreb	Laboratory for Molecular Biomedicine, Centre for Research and Knowledge Transfer in Biotechnology, University of Zagreb	Slovic,A., Forcic,D., Ivancic-Jelecki,J., Ljubin Sternak,S. and Mlinaric-Galinovic,G.
EPI_ISL_2589252	Virology, RMRC, NE Region (ICMR)	Virology, RMRC, NE Region (ICMR)	Biswas,D., Yadav,K., Borkakoty,B. and Mahanta,J.
EPI_ISL_2589253	Virology, School of Public Health, Tehran University of Medical Sciences	Virology, School of Public Health, Tehran University of Medical Sciences	Yavarian,J., Faghihloo,E. and Mokhtari Azad,T.
EPI_ISL_2589257, EPI_ISL_2589258, EPI_ISL_2589259, EPI_ISL_2589260, EPI_ISL_2589261	Servico de Patologia Clinica, Hospital de Clinicas de Porto Alegre	Servico de Patologia Clinica, Hospital de Clinicas de Porto Alegre	de-Paris,F., Beck,C., Nunes,L.S., Machado,A.B.M.P., Paiva,R.M., Menezes,D.S., Pires,M., Santos,R.P., Kuchenbecker,R.S. and Barth,A.L.
EPI_ISL_2589303, EPI_ISL_2589309, EPI_ISL_2589311, EPI_ISL_2589312, EPI_ISL_2589314	Laboratory for Molecular Biomedicine, Centre for Research and Knowledge Transfer in Biotechnology, University of Zagreb	Laboratory for Molecular Biomedicine, Centre for Research and Knowledge Transfer in Biotechnology, University of Zagreb	Slovic,A., Forcic,D., Ivancic-Jelecki,J., Ljubin Sternak,S. and Mlinaric-Galinovic,G.
EPI_ISL_2589326, EPI_ISL_2589327, EPI_ISL_2589328, EPI_ISL_2589329	Virology, RMRC, NE Region (ICMR)	Virology, RMRC, NE Region (ICMR)	Biswas,D., Yadav,K., Borkakoty,B. and Mahanta,J.
EPI_ISL_2589330	Virology, School of Public Health, Tehran University of Medical Sciences	Virology, School of Public Health, Tehran University of Medical Sciences	Yavarian,J., Faghihloo,E. and Mokhtari Azad,T.
EPI_ISL_2589331	Virology, Faculty of Public Health, Tehran University of Medical Sciences	Virology, Faculty of Public Health, Tehran University of Medical Sciences	Mokhtari-Azad,T., Faghihloo,E., Rezaei,F. and Salimi,V.
EPI_ISL_2589334, EPI_ISL_2589335, EPI_ISL_2589336, EPI_ISL_2589337, EPI_ISL_2589338, EPI_ISL_2589339	Servico de Patologia Clinica, Hospital de Clinicas de Porto Alegre	Servico de Patologia Clinica, Hospital de Clinicas de Porto Alegre	de-Paris,F., Beck,C., Nunes,L.S., Machado,A.B.M.P., Paiva,R.M., Menezes,D.S., Pires,M., Santos,R.P., Kuchenbecker,R.S. and Barth,A.L.
EPI_ISL_2589345, EPI_ISL_2589346, EPI_ISL_2589347, EPI_ISL_2589349	Laboratory for Molecular Biomedicine, Centre for Research and Knowledge Transfer in Biotechnology, University of Zagreb	Laboratory for Molecular Biomedicine, Centre for Research and Knowledge Transfer in Biotechnology, University of Zagreb	Slovic,A., Forcic,D., Ivancic-Jelecki,J., Ljubin Sternak,S. and Mlinaric-Galinovic,G.
EPI_ISL_2589351, EPI_ISL_2589352	Virology, School of Public Health, Tehran University of Medical Sciences	Virology, School of Public Health, Tehran University of Medical Sciences	Yavarian,J., Faghihloo,E. and Mokhtari Azad,T.
EPI_ISL_2589353, EPI_ISL_2589354, EPI_ISL_2589355, EPI_ISL_2589356, EPI_ISL_2589357, EPI_ISL_2589358, EPI_ISL_2589359, EPI_ISL_2589360, EPI_ISL_2589361, EPI_ISL_2589362, EPI_ISL_2589363, EPI_ISL_2589364, EPI_ISL_2589365, EPI_ISL_2589366, EPI_ISL_2589367, EPI_ISL_2589368, EPI_ISL_2589369, EPI_ISL_2589370, EPI_ISL_2589371, EPI_ISL_2589372, EPI_ISL_2589373, EPI_ISL_2589374, EPI_ISL_2589375, EPI_ISL_2589376, EPI_ISL_2589377, EPI_ISL_2589378, EPI_ISL_2589379			
see above	Servico de Patologia Clinica, Hospital de Clinicas de Porto Alegre	Servico de Patologia Clinica, Hospital de Clinicas de Porto Alegre	de-Paris,F., Beck,C., Nunes,L.S., Machado,A.B.M.P., Paiva,R.M., Menezes,D.S., Pires,M., Santos,R.P., Kuchenbecker,R.S. and Barth,A.L.
EPI_ISL_2589383	Laboratory for Molecular Biomedicine, Centre for Research and Knowledge Transfer in Biotechnology, University of Zagreb	Laboratory for Molecular Biomedicine, Centre for Research and Knowledge Transfer in Biotechnology, University of Zagreb	Slovic,A., Forcic,D., Ivancic-Jelecki,J., Ljubin Sternak,S. and Mlinaric-Galinovic,G.
EPI_ISL_2589384	Department for Infectious Diseases Virology, University of Heidelberg	Department for Infectious Diseases Virology, University of Heidelberg	Tabatabai,J., Prifert,C., Pfeil,J., Grulich-Henn,J. and Schnitzler,P.
EPI_ISL_2589387	Servico de Patologia Clinica, Hospital de Clinicas de Porto Alegre	Servico de Patologia Clinica, Hospital de Clinicas de Porto Alegre	de-Paris,F., Beck,C., Nunes,L.S., Machado,A.B.M.P., Paiva,R.M., Menezes,D.S., Pires,M., Santos,R.P., Kuchenbecker,R.S. and Barth,A.L.
EPI_ISL_2589394	Virology, Regional Medical Research Centre, NE (ICMR) Dibrugarh	Virology, Regional Medical Research Centre, NE (ICMR) Dibrugarh	Biswas,D., Yadav,K., Borkakoty,B. and Mahanta,J.
EPI_ISL_2589400, EPI_ISL_2589401, EPI_ISL_2589402, EPI_ISL_2589403, EPI_ISL_2589404, EPI_ISL_2589405, EPI_ISL_2589406, EPI_ISL_2589407, EPI_ISL_2589408, EPI_ISL_2589409, EPI_ISL_2589410, EPI_ISL_2589411, EPI_ISL_2589415, EPI_ISL_2589416, EPI_ISL_2589417, EPI_ISL_2589418, EPI_ISL_2589419, EPI_ISL_2589420, EPI_ISL_2589421, EPI_ISL_2589422			
see above	Dept of Biomedical Sciences and Public Health, Virology Unit, Marche Politechnic University Medical School	Dept of Biomedical Sciences and Public Health, Virology Unit, Marche Politechnic University Medical School	Bagnarelli,P., Trotta,D., Ferreri,M.L. and Pierangeli,A.
EPI_ISL_2589424, EPI_ISL_2589425, EPI_ISL_2589426, EPI_ISL_2589427, EPI_ISL_2589428, EPI_ISL_2589429, EPI_ISL_2589430, EPI_ISL_2589431	Naoko Kiyota Kumamoto Prefectural Institute of Public-Health and Environmental Science, Department of Microbiology	Naoko Kiyota Kumamoto Prefectural Institute of Public-Health and Environmental Science, Department of Microbiology	Kiyota,N., Yoshida,A. and Kobayashi,M.
EPI_ISL_2589433, EPI_ISL_2589434, EPI_ISL_2589435, EPI_ISL_2589436, EPI_ISL_2589437, EPI_ISL_2589438, EPI_ISL_2589439, EPI_ISL_2589440, EPI_ISL_2589441, EPI_ISL_2589442, EPI_ISL_2589443, EPI_ISL_2589444, EPI_ISL_2589445, EPI_ISL_2589446, EPI_ISL_2589447, EPI_ISL_2589448, EPI_ISL_2589451, EPI_ISL_2589456, EPI_ISL_2589457, EPI_ISL_2589458, EPI_ISL_2589459, EPI_ISL_2589460, EPI_ISL_2589461, EPI_ISL_2589466, EPI_ISL_2589467, EPI_ISL_2589468, EPI_ISL_2589469, EPI_ISL_2589470			
see above	Dept of Biomedical Sciences and Public Health, Virology Unit, Marche Politechnic University Medical School	Dept of Biomedical Sciences and Public Health, Virology Unit, Marche Politechnic University Medical School	Bagnarelli,P., Trotta,D., Ferreri,M.L. and Pierangeli,A.
EPI_ISL_2589472, EPI_ISL_2589473, EPI_ISL_2589474, EPI_ISL_2589475, EPI_ISL_2589476, EPI_ISL_2589477, EPI_ISL_2589478, EPI_ISL_2589479, EPI_ISL_2589480, EPI_ISL_2589481, EPI_ISL_2589482			
see above	Naoko Kiyota Kumamoto Prefectural Institute of Public-Health and Environmental Science, Department of Microbiology	Naoko Kiyota Kumamoto Prefectural Institute of Public-Health and Environmental Science, Department of Microbiology	Kiyota,N., Yoshida,A. and Kobayashi,M.
EPI_ISL_2589485, EPI_ISL_2589486, EPI_ISL_2589487, EPI_ISL_2589488, EPI_ISL_2589489, EPI_ISL_2589490, EPI_ISL_2589491, EPI_ISL_2589492, EPI_ISL_2589493, EPI_ISL_2589494	Dept of Biomedical Sciences and Public Health, Virology Unit, Marche Politechnic University Medical School	Dept of Biomedical Sciences and Public Health, Virology Unit, Marche Politechnic University Medical School	Bagnarelli,P., Trotta,D., Ferreri,M.L. and Pierangeli,A.
EPI_ISL_2589495, EPI_ISL_2589496, EPI_ISL_2589497, EPI_ISL_2589498	Naoko Kiyota Kumamoto Prefectural Institute of Public-Health and Environmental Science, Department of Microbiology	Naoko Kiyota Kumamoto Prefectural Institute of Public-Health and Environmental Science, Department of Microbiology	Kiyota,N., Yoshida,A. and Kobayashi,M.
EPI_ISL_2589504, EPI_ISL_2589505, EPI_ISL_2589506, EPI_ISL_2589507, EPI_ISL_2589508, EPI_ISL_2589509, EPI_ISL_2589510, EPI_ISL_2589511, EPI_ISL_2589512, EPI_ISL_2589513, EPI_ISL_2589514, EPI_ISL_2589515, EPI_ISL_2589516, EPI_ISL_2589517, EPI_ISL_2589518, EPI_ISL_2589522, EPI_ISL_2589523, EPI_ISL_2589524, EPI_ISL_2589525, EPI_ISL_2589526, EPI_ISL_2589527, EPI_ISL_2589528, EPI_ISL_2589529, EPI_ISL_2589530, EPI_ISL_2589531, EPI_ISL_2589532, EPI_ISL_2589533, EPI_ISL_2589534, EPI_ISL_2589535			
see above	Dept of Biomedical Sciences and Public Health, Virology Unit, Marche Politechnic University Medical School	Dept of Biomedical Sciences and Public Health, Virology Unit, Marche Politechnic University Medical School	Bagnarelli,P., Trotta,D., Ferreri,M.L. and Pierangeli,A.
EPI_ISL_2589563, EPI_ISL_2589598	Virology Laboratory, Dr. Ricardo Gutierrez Children Hospital	Virology Laboratory, Dr. Ricardo Gutierrez Children Hospital	Viegas,M., Goya,S. and Mistchenko,A.S.
EPI_ISL_2589618	Immunology and Microbiology, Rega Institute for Medical Research	Immunology and Microbiology, Rega Institute for Medical Research	Houspie,L., Lemey,P., Keyaerts,E., Reijmen,E., Vergote,V., Vankeerberghen,A., Vaeyens,F., De Beenhouwer,H. and Van Ranst,M.
EPI_ISL_2589620	Virology Laboratory, Dr. Ricardo Gutierrez Children Hospital	Virology Laboratory, Dr. Ricardo Gutierrez Children Hospital	Viegas,M., Goya,S. and Mistchenko,A.S.
EPI_ISL_2589650, EPI_ISL_2589651, EPI_ISL_2589652, EPI_ISL_2589653, EPI_ISL_2589654, EPI_ISL_2589656, EPI_ISL_2589657, EPI_ISL_2589658	Immunology and Microbiology, Rega Institute for Medical Research	Immunology and Microbiology, Rega Institute for Medical Research	Houspie,L., Lemey,P., Keyaerts,E., Reijmen,E., Vergote,V., Vankeerberghen,A., Vaeyens,F., De Beenhouwer,H. and Van Ranst,M.
EPI_ISL_2589659, EPI_ISL_2589660, EPI_ISL_2589661, EPI_ISL_2589662, EPI_ISL_2589664	Virology Laboratory, Dr. Ricardo Gutierrez Children Hospital	Virology Laboratory, Dr. Ricardo Gutierrez Children Hospital	Viegas,M., Goya,S. and Mistchenko,A.S.
EPI_ISL_2589683, EPI_ISL_2589686, EPI_ISL_2589687, EPI_ISL_2589688	Immunology and Microbiology, Rega Institute for Medical Research	Immunology and Microbiology, Rega Institute for Medical Research	Houspie,L., Lemey,P., Keyaerts,E., Reijmen,E., Vergote,V., Vankeerberghen,A., Vaeyens,F., De Beenhouwer,H. and Van Ranst,M.
EPI_ISL_2589689, EPI_ISL_2589690, EPI_ISL_2589694, EPI_ISL_2589695, EPI_ISL_2589696	Virology Laboratory, Dr. Ricardo Gutierrez Children Hospital	Virology Laboratory, Dr. Ricardo Gutierrez Children Hospital	Viegas,M., Goya,S. and Mistchenko,A.S.
EPI_ISL_2589697	Department of Biomedical Sciences for Health, University of Milan	Department of Biomedical Sciences for Health, University of Milan	Martinelli,M., Frati,E.R., Zappa,A., Ebranati,E., Bianchi,S., Pariani,E., Amendola,A., Zehender,G. and Tanzi,E.
EPI_ISL_2589702, EPI_ISL_2589704	Immunology and Microbiology, Rega Institute for Medical Research	Immunology and Microbiology, Rega Institute for Medical Research	Houspie,L., Lemey,P., Keyaerts,E., Reijmen,E., Vergote,V., Vankeerberghen,A., Vaeyens,F., De Beenhouwer,H. and Van Ranst,M.
EPI_ISL_2589707, EPI_ISL_2589708, EPI_ISL_2589709, EPI_ISL_2589710, EPI_ISL_2589716, EPI_ISL_2589717	Virology Laboratory, Dr. Ricardo Gutierrez Children Hospital	Virology Laboratory, Dr. Ricardo Gutierrez Children Hospital	Viegas,M., Goya,S. and Mistchenko,A.S.
EPI_ISL_2589721, EPI_ISL_2589725	Immunology and Microbiology, Rega Institute for Medical Research	Immunology and Microbiology, Rega Institute for Medical Research	Houspie,L., Lemey,P., Keyaerts,E., Reijmen,E., Vergote,V., Vankeerberghen,A., Vaeyens,F., De Beenhouwer,H. and Van Ranst,M.
EPI_ISL_2589729, EPI_ISL_2589730, EPI_ISL_2589731	Virology Laboratory, Dr. Ricardo Gutierrez Children Hospital	Virology Laboratory, Dr. Ricardo Gutierrez Children Hospital	Viegas,M., Goya,S. and Mistchenko,A.S.
EPI_ISL_2589732, EPI_ISL_2589733, EPI_ISL_2589734, EPI_ISL_2589738, EPI_ISL_2589740, EPI_ISL_2589741, EPI_ISL_2589745, EPI_ISL_2589747, EPI_ISL_2589748, EPI_ISL_2589751	Immunology and Microbiology, Rega Institute for Medical Research	Immunology and Microbiology, Rega Institute for Medical Research	Houspie,L., Lemey,P., Keyaerts,E., Reijmen,E., Vergote,V., Vankeerberghen,A., Vaeyens,F., De Beenhouwer,H. and Van Ranst,M.
EPI_ISL_2589756, EPI_ISL_2589758, EPI_ISL_2589760, EPI_ISL_2589762	Virology Laboratory, Dr. Ricardo Gutierrez Children Hospital	Virology Laboratory, Dr. Ricardo Gutierrez Children Hospital	Viegas,M., Goya,S. and Mistchenko,A.S.
EPI_ISL_2589766, EPI_ISL_2589773	Immunology and Microbiology, Rega Institute for Medical Research	Immunology and Microbiology, Rega Institute for Medical Research	Houspie,L., Lemey,P., Keyaerts,E., Reijmen,E., Vergote,V., Vankeerberghen,A., Vaeyens,F., De Beenhouwer,H. and Van Ranst,M.
EPI_ISL_2589793, EPI_ISL_2589798, EPI_ISL_2589800	Virology Laboratory, Dr. Ricardo Gutierrez Children Hospital	Virology Laboratory, Dr. Ricardo Gutierrez Children Hospital	Viegas,M., Goya,S. and Mistchenko,A.S.
EPI_ISL_2589804, EPI_ISL_2589809, EPI_ISL_2589832	Immunology and Microbiology, Rega Institute for Medical Research	Immunology and Microbiology, Rega Institute for Medical Research	Houspie,L., Lemey,P., Keyaerts,E., Reijmen,E., Vergote,V., Vankeerberghen,A., Vaeyens,F., De Beenhouwer,H. and Van Ranst,M.
EPI_ISL_2589840, EPI_ISL_2589842	Virology Laboratory, Dr. Ricardo Gutierrez Children Hospital	Virology Laboratory, Dr. Ricardo Gutierrez Children Hospital	Viegas,M., Goya,S. and Mistchenko,A.S.
EPI_ISL_2589846, EPI_ISL_2589848, EPI_ISL_2589851, EPI_ISL_2589853	Immunology and Microbiology, Rega Institute for Medical Research	Immunology and Microbiology, Rega Institute for Medical Research	Houspie,L., Lemey,P., Keyaerts,E., Reijmen,E., Vergote,V., Vankeerberghen,A., Vaeyens,F., De Beenhouwer,H. and Van Ranst,M.
EPI_ISL_2589855, EPI_ISL_2589857	Virology Laboratory, Dr. Ricardo Gutierrez Children Hospital	Virology Laboratory, Dr. Ricardo Gutierrez Children Hospital	Viegas,M., Goya,S. and Mistchenko,A.S.
EPI_ISL_2589859, EPI_ISL_2589861, EPI_ISL_2589863, EPI_ISL_2589869	Immunology and Microbiology, Rega Institute for Medical Research	Immunology and Microbiology, Rega Institute for Medical Research	Houspie,L., Lemey,P., Keyaerts,E., Reijmen,E., Vergote,V., Vankeerberghen,A., Vaeyens,F., De Beenhouwer,H. and Van Ranst,M.
EPI_ISL_2589871, EPI_ISL_2589873	Molecular Research, Ontario Agency for Health Protection and Promotion	Molecular Research, Ontario Agency for Health Protection and Promotion	Eshaghi,A., Lai,R., Nadarajah,J.T., Li,A., Patel,S.N., Gubbay,J.B. and Low,D.E.
EPI_ISL_2589875	Virology, Capital Institute of Pediatrics	Virology, Capital Institute of Pediatrics	Deng,J., Zhu,R., Qian,Y., Sun,Y., Zhao,L., Wang,F., Wu,H., Shan,M. and Deji,M.

EPI_ISL_2589879	Molecular Research, Ontario Agency for Health Protection and Promotion	Molecular Research, Ontario Agency for Health Protection and Promotion	Eshaghi,A., Lai,R., Nadarajah,J.T., Li,A., Patel,S.N., Gubbay,J.B. and Low,D.E.
EPI_ISL_2589889	Virology, Capital Institute of Pediatrics	Virology, Capital Institute of Pediatrics	Deng,J., Zhu,R., Qian,Y., Sun,Y., Zhao,L., Wang,F., Wu,H., Shan,M. and Deji,M.
EPI_ISL_2589896	Molecular Research, Ontario Agency for Health Protection and Promotion	Molecular Research, Ontario Agency for Health Protection and Promotion	Eshaghi,A., Lai,R., Nadarajah,J.T., Li,A., Patel,S.N., Gubbay,J.B. and Low,D.E.
EPI_ISL_2589909	Virology, Capital Institute of Pediatrics	Virology, Capital Institute of Pediatrics	Deng,J., Zhu,R., Qian,Y., Sun,Y., Zhao,L., Wang,F., Wu,H., Shan,M. and Deji,M.
EPI_ISL_2589910	Molecular Research, Ontario Agency for Health Protection and Promotion	Molecular Research, Ontario Agency for Health Protection and Promotion	Eshaghi,A., Lai,R., Nadarajah,J.T., Li,A., Patel,S.N., Gubbay,J.B. and Low,D.E.
EPI_ISL_2589912	West China School of Public Health, Sichuan University	West China School of Public Health, Sichuan University	Zhang,M.
EPI_ISL_2589925	Microbiology, Jawaharlal Institute of Postgraduate Medical Education & Research (JIPMER)	Microbiology, Jawaharlal Institute of Postgraduate Medical Education & Research (JIPMER)	Nandhini,G. and Sujatha,S.
EPI_ISL_2589976	Virology, Capital Institute of Pediatrics	Virology, Capital Institute of Pediatrics	Deng,J., Zhu,R., Qian,Y., Sun,Y., Zhao,L., Wang,F., Wu,H., Shan,M. and Deji,M.
EPI_ISL_2589990	Virology Laboratory, Dr. Ricardo Gutierrez Children Hospital	Virology Laboratory, Dr. Ricardo Gutierrez Children Hospital	Viegas,M., Goya,S. and Mistchenko,A.S.
EPI_ISL_2590226	Molecular Research, Ontario Agency for Health Protection and Promotion	Molecular Research, Ontario Agency for Health Protection and Promotion	Eshaghi,A., Lai,R., Nadarajah,J.T., Li,A., Patel,S.N., Gubbay,J.B. and Low,D.E.
EPI_ISL_2590299, EPI_ISL_2590301, EPI_ISL_2590302	Virology Laboratory, Dr. Ricardo Gutierrez Children Hospital	Virology Laboratory, Dr. Ricardo Gutierrez Children Hospital	Viegas,M., Goya,S. and Mistchenko,A.S.
EPI_ISL_2590826	Virology, NAMRU-36	Virology, NAMRU-36	Garcia,J., Sovero,M. and Halsey,E.S.
EPI_ISL_2590916	Dept of Biomedical Sciences and Public Health, Virology Unit, Marche Politechnic University Medical School	Dept of Biomedical Sciences and Public Health, Virology Unit, Marche Politechnic University Medical School	Bagnarelli,P., Trotta,D., Ferreri,M.L. and Pierangeli,A.
EPI_ISL_2590953	Virology, NAMRU-22	Virology, NAMRU-22	Garcia,J., Sovero,M. and Halsey,E.S.
EPI_ISL_2591105	West China School of Public Health, Sichuan University	West China School of Public Health, Sichuan University	Zhang,M.
EPI_ISL_2591117, EPI_ISL_2591119	Virology, Faculty of Public Health, Tehran University of Medical Sciences	Virology, Faculty of Public Health, Tehran University of Medical Sciences	Mokhtari-Azad,T., Faghihloo,E., Rezaei,F. and Salimi,V.
EPI_ISL_2591135	Virology, School of Public Health, Tehran University of Medical Sciences	Virology, School of Public Health, Tehran University of Medical Sciences	Yavarian,J., Faghihloo,E. and Mokhtari Azad,T.
EPI_ISL_2591188	Virology, Faculty of Public Health, Tehran University of Medical Sciences	Virology, Faculty of Public Health, Tehran University of Medical Sciences	Mokhtari-Azad,T., Faghihloo,E., Rezaei,F. and Salimi,V.
EPI_ISL_2591202	Virology, NAMRU-16	Virology, NAMRU-16	Garcia,J., Sovero,M. and Halsey,E.S.
EPI_ISL_2591212	Virology, NAMRU-45	Virology, NAMRU-45	Garcia,J., Sovero,M. and Halsey,E.S.
EPI_ISL_2591237, EPI_ISL_2591239	Virology, Regional Medical Research Centre, NE (ICMR) Dibrugarh	Virology, Regional Medical Research Centre, NE (ICMR) Dibrugarh	Biswas,D., Yadav,K., Borkakoty,B. and Mahanta,J.
EPI_ISL_2591241	Immunology and Microbiology, Rega Institute for Medical Research	Immunology and Microbiology, Rega Institute for Medical Research	Houspie,L., Lemey,P., Keyaerts,E., Reijmen,E., Vergote,V., Vankeerberghen,A., Vaeyens,F., De Beenhouwer,H. and Van Ranst,M.
EPI_ISL_2591276, EPI_ISL_2591284	Virology Laboratory, Dr. Ricardo Gutierrez Children Hospital	Virology Laboratory, Dr. Ricardo Gutierrez Children Hospital	Viegas,M., Goya,S. and Mistchenko,A.S.
EPI_ISL_2591474	Department of Respiratory Medicine, Children's Hospital of Chongqing Medical University	Department of Respiratory Medicine, Children's Hospital of Chongqing Medical University	Xia,Q. and Liu,E.
EPI_ISL_2591478	Molecular Research, Ontario Agency for Health Protection and Promotion	Molecular Research, Ontario Agency for Health Protection and Promotion	Eshaghi,A., Lai,R., Nadarajah,J.T., Li,A., Patel,S.N., Gubbay,J.B. and Low,D.E.
EPI_ISL_2591502	Virology, NAMRU-38	Virology, NAMRU-38	Garcia,J., Sovero,M. and Halsey,E.S.
EPI_ISL_2591531	University of Wuerzburg, Institute of Virology and Immunobiology	University of Wuerzburg, Institute of Virology and Immunobiology	Prifert,C., Hofmann,D. and Weissbrich,B.
EPI_ISL_2591533, EPI_ISL_2591535, EPI_ISL_2591537, EPI_ISL_2591541, EPI_ISL_2591543, EPI_ISL_2591545	Dept of Biomedical Sciences and Public Health, Virology Unit, Marche Politechnic University Medical School	Dept of Biomedical Sciences and Public Health, Virology Unit, Marche Politechnic University Medical School	Bagnarelli,P., Trotta,D., Ferreri,M.L. and Pierangeli,A.
EPI_ISL_2591625, EPI_ISL_2591627	Virology, School of Public Health, Tehran University of Medical Sciences	Virology, School of Public Health, Tehran University of Medical Sciences	Yavarian,J., Faghihloo,E. and Mokhtari Azad,T.
EPI_ISL_2591631, EPI_ISL_2591633	Virology, Faculty of Public Health, Tehran University of Medical Sciences	Virology, Faculty of Public Health, Tehran University of Medical Sciences	Mokhtari-Azad,T., Faghihloo,E., Rezaei,F. and Salimi,V.
EPI_ISL_2591715, EPI_ISL_2591716, EPI_ISL_2591717, EPI_ISL_2591718	Virology Laboratory, Dr. Ricardo Gutierrez Children Hospital	Virology Laboratory, Dr. Ricardo Gutierrez Children Hospital	Viegas,M., Goya,S. and Mistchenko,A.S.
EPI_ISL_2591768	Department for Infectious Diseases Virology, University of Heidelberg	Department for Infectious Diseases Virology, University of Heidelberg	Tabatabai,J., Prifert,C., Pfeil,J., Grulich-Henn,J. and Schnitzler,P.
EPI_ISL_2591784, EPI_ISL_2591785	University of Wuerzburg, Institute of Virology and Immunobiology	University of Wuerzburg, Institute of Virology and Immunobiology	Prifert,C., Hofmann,D. and Weissbrich,B.
EPI_ISL_2591789, EPI_ISL_2591790, EPI_ISL_2591791	Dept of Biomedical Sciences and Public Health, Virology Unit, Marche Politechnic University Medical School	Dept of Biomedical Sciences and Public Health, Virology Unit, Marche Politechnic University Medical School	Bagnarelli,P., Trotta,D., Ferreri,M.L. and Pierangeli,A.
EPI_ISL_2591815	Department of Respiratory Medicine, Children's Hospital of Chongqing Medical University	Department of Respiratory Medicine, Children's Hospital of Chongqing Medical University	Xia,Q. and Liu,E.
EPI_ISL_2591819	Molecular Virology Unit, Virology and Microbiology Department, Fondazione IRCCS Policlinico San Matteo	Molecular Virology Unit, Virology and Microbiology Department, Fondazione IRCCS Policlinico San Matteo	Piralla,A., Percivalle,E. and Baldanti,F.
EPI_ISL_2591833, EPI_ISL_2591834, EPI_ISL_2591835, EPI_ISL_2591836, EPI_ISL_2591837	Miwako SaikNorth America / USA Yokohama City Institute of Public Health	Miwako SaikNorth America / USA Yokohama City Institute of Public Health	SaikNorth America / USA,M., Kawakami,C., Usuku,S., Sasao,T. and Okubo,I.
EPI_ISL_2591838	Virology, NAMRU-30	Virology, NAMRU-30	Garcia,J., Sovero,M. and Halsey,E.S.
EPI_ISL_2591862	Virology Laboratory, Dr. Ricardo Gutierrez Children Hospital	Virology Laboratory, Dr. Ricardo Gutierrez Children Hospital	Viegas,M., Goya,S. and Mistchenko,A.S.
EPI_ISL_2591875, EPI_ISL_2591876, EPI_ISL_2591877, EPI_ISL_2591878, EPI_ISL_2591879	Department of Respiratory Medicine, Children's Hospital of Chongqing Medical University	Department of Respiratory Medicine, Children's Hospital of Chongqing Medical University	Xia,Q. and Liu,E.
EPI_ISL_2591896, EPI_ISL_2591897, EPI_ISL_2591898, EPI_ISL_2591899, EPI_ISL_2591900, EPI_ISL_2591901	Miwako SaikNorth America / USA Yokohama City Institute of Public Health	Miwako SaikNorth America / USA Yokohama City Institute of Public Health	SaikNorth America / USA,M., Kawakami,C., Usuku,S., Sasao,T. and Okubo,I.
EPI_ISL_2591903, EPI_ISL_2591904, EPI_ISL_2591905, EPI_ISL_2591906, EPI_ISL_2591907, EPI_ISL_2591908	Molecular Research, Ontario Agency for Health Protection and Promotion	Molecular Research, Ontario Agency for Health Protection and Promotion	Eshaghi,A., Lai,R., Nadarajah,J.T., Li,A., Patel,S.N., Gubbay,J.B. and Low,D.E.
EPI_ISL_2591919, EPI_ISL_2591921, EPI_ISL_2591922, EPI_ISL_2591923, EPI_ISL_2591924, EPI_ISL_2591925, EPI_ISL_2591926, EPI_ISL_2591927, EPI_ISL_2591928, EPI_ISL_2591929, EPI_ISL_2591930, EPI_ISL_2591931, EPI_ISL_2591932, EPI_ISL_2591933, EPI_ISL_2591934, EPI_ISL_2591935, EPI_ISL_2591936, EPI_ISL_2591937, EPI_ISL_2591938, EPI_ISL_2591939			
see above	Department of Respiratory Medicine, Children's Hospital of Chongqing Medical University	Department of Respiratory Medicine, Children's Hospital of Chongqing Medical University	Xia,Q. and Liu,E.
EPI_ISL_2591975, EPI_ISL_2591976, EPI_ISL_2591990, EPI_ISL_2591991, EPI_ISL_2591992, EPI_ISL_2591993, EPI_ISL_2591994, EPI_ISL_2591995, EPI_ISL_2591996, EPI_ISL_2591997, EPI_ISL_2591998, EPI_ISL_2591999, EPI_ISL_2592000, EPI_ISL_2592001, EPI_ISL_2592002, EPI_ISL_2592003, EPI_ISL_2592004, EPI_ISL_2592005, EPI_ISL_2592006, EPI_ISL_2592007, EPI_ISL_2592008			
see above	Miwako SaikNorth America / USA Yokohama City Institute of Public Health	Miwako SaikNorth America / USA Yokohama City Institute of Public Health	SaikNorth America / USA,M., Kawakami,C., Usuku,S., Sasao,T. and Okubo,I.
EPI_ISL_2592090, EPI_ISL_2592091, EPI_ISL_2592092, EPI_ISL_2592093, EPI_ISL_2592094, EPI_ISL_2592095	Molecular Research, Ontario Agency for Health Protection and Promotion	Molecular Research, Ontario Agency for Health Protection and Promotion	Eshaghi,A., Lai,R., Nadarajah,J.T., Li,A., Patel,S.N., Gubbay,J.B. and Low,D.E.
EPI_ISL_2592096, EPI_ISL_2592097, EPI_ISL_2592098, EPI_ISL_2592099	Virology, Institut Pasteur de Bangui	Virology, Institut Pasteur de Bangui	Tricou,V.
EPI_ISL_2592100	West China School of Public Health, Sichuan University	West China School of Public Health, Sichuan University	Zhang,M.
EPI_ISL_2592115, EPI_ISL_2592116, EPI_ISL_2592117, EPI_ISL_2592118, EPI_ISL_2592119, EPI_ISL_2592120, EPI_ISL_2592121, EPI_ISL_2592122, EPI_ISL_2592123, EPI_ISL_2592124, EPI_ISL_2592125, EPI_ISL_2592126, EPI_ISL_2592127, EPI_ISL_2592128, EPI_ISL_2592129, EPI_ISL_2592130, EPI_ISL_2592131, EPI_ISL_2592132, EPI_ISL_2592133, EPI_ISL_2592134, EPI_ISL_2592135, EPI_ISL_2592136, EPI_ISL_2592137, EPI_ISL_2592138, EPI_ISL_2592139, EPI_ISL_2592140, EPI_ISL_2592141, EPI_ISL_2592142, EPI_ISL_2592143, EPI_ISL_2592144, EPI_ISL_2592145, EPI_ISL_2592146, EPI_ISL_2592147, EPI_ISL_2592148, EPI_ISL_2592149, EPI_ISL_2592150, EPI_ISL_2592151, EPI_ISL_2592152, EPI_ISL_2592153, EPI_ISL_2592154, EPI_ISL_2592155, EPI_ISL_2592156, EPI_ISL_2592157, EPI_ISL_2592158, EPI_ISL_2592159, EPI_ISL_2592160, EPI_ISL_2592161, EPI_ISL_2592162, EPI_ISL_2592163, EPI_ISL_2592164, EPI_ISL_2592165, EPI_ISL_2592167, EPI_ISL_2592168, EPI_ISL_2592169, EPI_ISL_2592170, EPI_ISL_2592171, EPI_ISL_2592172, EPI_ISL_2592173, EPI_ISL_2592174, EPI_ISL_2592175, EPI_ISL_2592176, EPI_ISL_2592177, EPI_ISL_2592178, EPI_ISL_2592179, EPI_ISL_2592180, EPI_ISL_2592181, EPI_ISL_2592182, EPI_ISL_2592183, EPI_ISL_2592184, EPI_ISL_2592185, EPI_ISL_2592186, EPI_ISL_2592187, EPI_ISL_2592188, EPI_ISL_2592189			
see above	Department of Respiratory Medicine, Children's Hospital of Chongqing Medical University	Department of Respiratory Medicine, Children's Hospital of Chongqing Medical University	Xia,Q. and Liu,E.
EPI_ISL_2592301, EPI_ISL_2592302	Laboratory for Molecular Biomedicine, Centre for Research and Knowledge Transfer in Biotechnology, University of Zagreb	Laboratory for Molecular Biomedicine, Centre for Research and Knowledge Transfer in Biotechnology, University of Zagreb	Slovic,A., Forcic,D., Ivancic-Jelecki,J., Ljubin Sternak,S. and Mlinaric-Galinovic,G.
EPI_ISL_2592362, EPI_ISL_2592363, EPI_ISL_2592364, EPI_ISL_2592365, EPI_ISL_2592366, EPI_ISL_2592367, EPI_ISL_2592368	University of Wuerzburg, Institute of Virology and Immunobiology	University of Wuerzburg, Institute of Virology and Immunobiology	Prifert,C., Hofmann,D. and Weissbrich,B.
EPI_ISL_2592389	Microbiology, Jawaharlal Institute of Postgraduate Medical Education & Research (JIPMER)	Microbiology, Jawaharlal Institute of Postgraduate Medical Education & Research (JIPMER)	Nandhini,G. and Sujatha,S.
EPI_ISL_2592391, EPI_ISL_2592392	virology, king institute of preventive medicine	virology, king institute of preventive medicine	Gunasekharan,P., Kaveri,K., Mohan,K., Salma Sulthana,S., Senthil Raju,K., Senthil Kumar,V., Kiruba,R., Padma Priya,P., Saravana Murali,V. and Suresh Babu,V.
EPI_ISL_2592393	Virology, School of Public Health, Tehran University of Medical Sciences	Virology, School of Public Health, Tehran University of Medical Sciences	Yavarian,J., Faghihloo,E. and Mokhtari Azad,T.
EPI_ISL_2592394, EPI_ISL_2592395, EPI_ISL_2592396, EPI_ISL_2592422, EPI_ISL_2592423	Dept of Biomedical Sciences and Public Health, Virology Unit, Marche Politechnic University Medical School	Dept of Biomedical Sciences and Public Health, Virology Unit, Marche Politechnic University Medical School	Bagnarelli,P., Trotta,D., Ferreri,M.L. and Pierangeli,A.
EPI_ISL_2592425, EPI_ISL_2592426	Molecular Virology Unit, Virology and Microbiology Department, Fondazione IRCCS Policlinico San Matteo	Molecular Virology Unit, Virology and Microbiology Department, Fondazione IRCCS Policlinico San Matteo	Piralla,A., Percivalle,E. and Baldanti,F.
EPI_ISL_2592436, EPI_ISL_2592450, EPI_ISL_2592451, EPI_ISL_2592452, EPI_ISL_2592453, EPI_ISL_2592454, EPI_ISL_2592455, EPI_ISL_2592456, EPI_ISL_2592457, EPI_ISL_2592458, EPI_ISL_2592459, EPI_ISL_2592460, EPI_ISL_2592461, EPI_ISL_2592462, EPI_ISL_2592463, EPI_ISL_2592464, EPI_ISL_2592465, EPI_ISL_2592466, EPI_ISL_2592467, EPI_ISL_2592468, EPI_ISL_2592469, EPI_ISL_2592470, EPI_ISL_2592471, EPI_ISL_2592472, EPI_ISL_2592473, EPI_ISL_2592474, EPI_ISL_2592475, EPI_ISL_2592476, EPI_ISL_2592477, EPI_ISL_2592478, EPI_ISL_2592479, EPI_ISL_2592480, EPI_ISL_2592481, EPI_ISL_2592482, EPI_ISL_2592483, EPI_ISL_2592484, EPI_ISL_2592485			
see above	Miwako SaikNorth America / USA Yokohama City Institute of Public Health	Miwako SaikNorth America / USA Yokohama City Institute of Public Health	SaikNorth America / USA,M., Kawakami,C., Usuku,S., Sasao,T. and Okubo,I.

EPI_ISL_2593329, EPI_ISL_2593330, EPI_ISL_2593331, EPI_ISL_2593332	Epidemiology and Demography, KEMRI-Wellcome Trust Research Collaborative Programme	Epidemiology and Demography, KEMRI-Wellcome Trust Research Collaborative Programme	Agoti,C.N., Mayieka,L., Otieno,J.R., Ahmed,J.A., Fields,B., Waiboci,L.B., Nyoka,R., Eidex,R., Morano,N., Burton,W., Montgomery,J., Breiman,R. and Nokes,D.J.
EPI_ISL_2593333	Department of Clinical Laboratory, Fujian Provincial Hospital	Department of Clinical Laboratory, Fujian Provincial Hospital	Su,Y., Wu,Y., Tian,R. and Liang,G.
EPI_ISL_2593334, EPI_ISL_2593335, EPI_ISL_2593336, EPI_ISL_2593337	Epidemiology and Demography, KEMRI-Wellcome Trust Research Collaborative Programme	Epidemiology and Demography, KEMRI-Wellcome Trust Research Collaborative Programme	Agoti,C.N., Mayieka,L., Otieno,J.R., Ahmed,J.A., Fields,B., Waiboci,L.B., Nyoka,R., Eidex,R., Morano,N., Burton,W., Montgomery,J., Breiman,R. and Nokes,D.J.
EPI_ISL_2593338, EPI_ISL_2593339, EPI_ISL_2593340, EPI_ISL_2593341, EPI_ISL_2593342	Department of Epidemiology and Demography, Kenya Medical Research Institute (KEMRI) - Wellcome Trust Research Programme	Department of Epidemiology and Demography, Kenya Medical Research Institute (KEMRI) - Wellcome Trust Research Programme	Otieno,J.R., Agoti,C.N., Gitahi,C.W., Bett,A., Ngama,M., Medley,G.F., Cane,P.A. and Nokes,J.D.
EPI_ISL_2593343, EPI_ISL_2593344, EPI_ISL_2593345	Epidemiology and Demography, KEMRI-Wellcome Trust Research Collaborative Programme	Epidemiology and Demography, KEMRI-Wellcome Trust Research Collaborative Programme	Agoti,C.N., Mayieka,L., Otieno,J.R., Ahmed,J.A., Fields,B., Waiboci,L.B., Nyoka,R., Eidex,R., Morano,N., Burton,W., Montgomery,J., Breiman,R. and Nokes,D.J.
EPI_ISL_2593346	Department of Epidemiology and Demography, Kenya Medical Research Institute (KEMRI) - Wellcome Trust Research Programme	Department of Epidemiology and Demography, Kenya Medical Research Institute (KEMRI) - Wellcome Trust Research Programme	Otieno,J.R., Agoti,C.N., Gitahi,C.W., Bett,A., Ngama,M., Medley,G.F., Cane,P.A. and Nokes,J.D.
EPI_ISL_2593347	Virology, Tropical Medicine Institute Pedro Kouri	Virology, Tropical Medicine Institute Pedro Kouri	Valdes,O., Corso,M., Pinon,A., Acosta,B., Savon,C., Gonzalez,G., Mune,M., Gonzalez,G., Hernandez,B., Echevarria,Y. and Oropesa,S.
EPI_ISL_2593348	Department of Epidemiology and Demography, Kenya Medical Research Institute (KEMRI) - Wellcome Trust Research Programme	Department of Epidemiology and Demography, Kenya Medical Research Institute (KEMRI) - Wellcome Trust Research Programme	Otieno,J.R., Agoti,C.N., Gitahi,C.W., Bett,A., Ngama,M., Medley,G.F., Cane,P.A. and Nokes,J.D.
EPI_ISL_2593349, EPI_ISL_2593350	Epidemiology and Demography, KEMRI-Wellcome Trust Research Collaborative Programme	Epidemiology and Demography, KEMRI-Wellcome Trust Research Collaborative Programme	Agoti,C.N., Mayieka,L., Otieno,J.R., Ahmed,J.A., Fields,B., Waiboci,L.B., Nyoka,R., Eidex,R., Morano,N., Burton,W., Montgomery,J., Breiman,R. and Nokes,D.J.
EPI_ISL_2593351	Department of Epidemiology and Demography, Kenya Medical Research Institute (KEMRI) - Wellcome Trust Research Programme	Department of Epidemiology and Demography, Kenya Medical Research Institute (KEMRI) - Wellcome Trust Research Programme	Otieno,J.R., Agoti,C.N., Gitahi,C.W., Bett,A., Ngama,M., Medley,G.F., Cane,P.A. and Nokes,J.D.
EPI_ISL_2593352	Epidemiology and Demography, KEMRI-Wellcome Trust Research Collaborative Programme	Epidemiology and Demography, KEMRI-Wellcome Trust Research Collaborative Programme	Agoti,C.N., Mayieka,L., Otieno,J.R., Ahmed,J.A., Fields,B., Waiboci,L.B., Nyoka,R., Eidex,R., Morano,N., Burton,W., Montgomery,J., Breiman,R. and Nokes,D.J.
EPI_ISL_2593353	Department of Epidemiology and Demography, Kenya Medical Research Institute (KEMRI) - Wellcome Trust Research Programme	Department of Epidemiology and Demography, Kenya Medical Research Institute (KEMRI) - Wellcome Trust Research Programme	Otieno,J.R., Agoti,C.N., Gitahi,C.W., Bett,A., Ngama,M., Medley,G.F., Cane,P.A. and Nokes,J.D.
EPI_ISL_2593354, EPI_ISL_2593355, EPI_ISL_2593356	Epidemiology and Demography, KEMRI-Wellcome Trust Research Collaborative Programme	Epidemiology and Demography, KEMRI-Wellcome Trust Research Collaborative Programme	Agoti,C.N., Mayieka,L., Otieno,J.R., Ahmed,J.A., Fields,B., Waiboci,L.B., Nyoka,R., Eidex,R., Morano,N., Burton,W., Montgomery,J., Breiman,R. and Nokes,D.J.
EPI_ISL_2593357	Department of Epidemiology and Demography, Kenya Medical Research Institute (KEMRI) - Wellcome Trust Research Programme	Department of Epidemiology and Demography, Kenya Medical Research Institute (KEMRI) - Wellcome Trust Research Programme	Otieno,J.R., Agoti,C.N., Gitahi,C.W., Bett,A., Ngama,M., Medley,G.F., Cane,P.A. and Nokes,J.D.
EPI_ISL_2593358	Epidemiology and Demography, KEMRI-Wellcome Trust Research Collaborative Programme	Epidemiology and Demography, KEMRI-Wellcome Trust Research Collaborative Programme	Agoti,C.N., Mayieka,L., Otieno,J.R., Ahmed,J.A., Fields,B., Waiboci,L.B., Nyoka,R., Eidex,R., Morano,N., Burton,W., Montgomery,J., Breiman,R. and Nokes,D.J.
EPI_ISL_2593359	Department of Epidemiology and Demography, Kenya Medical Research Institute (KEMRI) - Wellcome Trust Research Programme	Department of Epidemiology and Demography, Kenya Medical Research Institute (KEMRI) - Wellcome Trust Research Programme	Otieno,J.R., Agoti,C.N., Gitahi,C.W., Bett,A., Ngama,M., Medley,G.F., Cane,P.A. and Nokes,J.D.
EPI_ISL_2593360	Epidemiology and Demography, KEMRI-Wellcome Trust Research Collaborative Programme	Epidemiology and Demography, KEMRI-Wellcome Trust Research Collaborative Programme	Agoti,C.N., Mayieka,L., Otieno,J.R., Ahmed,J.A., Fields,B., Waiboci,L.B., Nyoka,R., Eidex,R., Morano,N., Burton,W., Montgomery,J., Breiman,R. and Nokes,D.J.
EPI_ISL_2593361	Department of Epidemiology and Demography, Kenya Medical Research Institute (KEMRI) - Wellcome Trust Research Programme	Department of Epidemiology and Demography, Kenya Medical Research Institute (KEMRI) - Wellcome Trust Research Programme	Otieno,J.R., Agoti,C.N., Gitahi,C.W., Bett,A., Ngama,M., Medley,G.F., Cane,P.A. and Nokes,J.D.
EPI_ISL_2593362	Epidemiology and Demography, KEMRI-Wellcome Trust Research Collaborative Programme	Epidemiology and Demography, KEMRI-Wellcome Trust Research Collaborative Programme	Agoti,C.N., Mayieka,L., Otieno,J.R., Ahmed,J.A., Fields,B., Waiboci,L.B., Nyoka,R., Eidex,R., Morano,N., Burton,W., Montgomery,J., Breiman,R. and Nokes,D.J.
EPI_ISL_2593363	College of Medicine and Medical Research Institute, Chungbuk National University	College of Medicine and Medical Research Institute, Chungbuk National University	Baek,Y.H., Choi,E.H., Park,S.-J., Song,M.-S. and Choi,Y.-K.
EPI_ISL_2593364	Virology Division, Public Health Laboratory Services Branch, Centre for Health Protection, Department of Health	Virology Division, Public Health Laboratory Services Branch, Centre for Health Protection, Department of Health	Mak,G.C., Lau,C.S., Chiu,D.M.Y. and Lim,W.
EPI_ISL_2593365, EPI_ISL_2593366, EPI_ISL_2593367	Department of Epidemiology and Demography, Kenya Medical Research Institute (KEMRI) - Wellcome Trust Research Programme	Department of Epidemiology and Demography, Kenya Medical Research Institute (KEMRI) - Wellcome Trust Research Programme	Otieno,J.R., Agoti,C.N., Gitahi,C.W., Bett,A., Ngama,M., Medley,G.F., Cane,P.A. and Nokes,J.D.
EPI_ISL_2593368	Epidemiology and Demography, KEMRI-Wellcome Trust Research Collaborative Programme	Epidemiology and Demography, KEMRI-Wellcome Trust Research Collaborative Programme	Agoti,C.N., Mayieka,L., Otieno,J.R., Ahmed,J.A., Fields,B., Waiboci,L.B., Nyoka,R., Eidex,R., Morano,N., Burton,W., Montgomery,J., Breiman,R. and Nokes,D.J.
EPI_ISL_2593369	Department of Epidemiology and Demography, Kenya Medical Research Institute (KEMRI) - Wellcome Trust Research Programme	Department of Epidemiology and Demography, Kenya Medical Research Institute (KEMRI) - Wellcome Trust Research Programme	Otieno,J.R., Agoti,C.N., Gitahi,C.W., Bett,A., Ngama,M., Medley,G.F., Cane,P.A. and Nokes,J.D.
EPI_ISL_2593370	Epidemiology and Demography, KEMRI-Wellcome Trust Research Collaborative Programme	Epidemiology and Demography, KEMRI-Wellcome Trust Research Collaborative Programme	Agoti,C.N., Mayieka,L., Otieno,J.R., Ahmed,J.A., Fields,B., Waiboci,L.B., Nyoka,R., Eidex,R., Morano,N., Burton,W., Montgomery,J., Breiman,R. and Nokes,D.J.
EPI_ISL_2593371, EPI_ISL_2593372, EPI_ISL_2593373, EPI_ISL_2593374	Department of Epidemiology and Demography, Kenya Medical Research Institute (KEMRI) - Wellcome Trust Research Programme	Department of Epidemiology and Demography, Kenya Medical Research Institute (KEMRI) - Wellcome Trust Research Programme	Otieno,J.R., Agoti,C.N., Gitahi,C.W., Bett,A., Ngama,M., Medley,G.F., Cane,P.A. and Nokes,J.D.
EPI_ISL_2593375, EPI_ISL_2593376, EPI_ISL_2593377, EPI_ISL_2593378	Epidemiology and Demography, KEMRI-Wellcome Trust Research Collaborative Programme	Epidemiology and Demography, KEMRI-Wellcome Trust Research Collaborative Programme	Agoti,C.N., Mayieka,L., Otieno,J.R., Ahmed,J.A., Fields,B., Waiboci,L.B., Nyoka,R., Eidex,R., Morano,N., Burton,W., Montgomery,J., Breiman,R. and Nokes,D.J.
EPI_ISL_2593379	Department of Epidemiology and Demography, Kenya Medical Research Institute (KEMRI) - Wellcome Trust Research Programme	Department of Epidemiology and Demography, Kenya Medical Research Institute (KEMRI) - Wellcome Trust Research Programme	Otieno,J.R., Agoti,C.N., Gitahi,C.W., Bett,A., Ngama,M., Medley,G.F., Cane,P.A. and Nokes,J.D.
EPI_ISL_2593380, EPI_ISL_2593381	Virology, Tropical Medicine Institute Pedro Kouri	Virology, Tropical Medicine Institute Pedro Kouri	Valdes,O., Corso,M., Pinon,A., Acosta,B., Savon,C., Gonzalez,G., Mune,M., Gonzalez,G., Hernandez,B., Echevarria,Y. and Oropesa,S.
EPI_ISL_2593382, EPI_ISL_2593383, EPI_ISL_2593384, EPI_ISL_2593385, EPI_ISL_2593386, EPI_ISL_2593387, EPI_ISL_2593388	Department of Epidemiology and Demography, Kenya Medical Research Institute (KEMRI) - Wellcome Trust Research Programme	Department of Epidemiology and Demography, Kenya Medical Research Institute (KEMRI) - Wellcome Trust Research Programme	Otieno,J.R., Agoti,C.N., Gitahi,C.W., Bett,A., Ngama,M., Medley,G.F., Cane,P.A. and Nokes,J.D.
EPI_ISL_2593389	Epidemiology and Demography, KEMRI-Wellcome Trust Research Collaborative Programme	Epidemiology and Demography, KEMRI-Wellcome Trust Research Collaborative Programme	Agoti,C.N., Mayieka,L., Otieno,J.R., Ahmed,J.A., Fields,B., Waiboci,L.B., Nyoka,R., Eidex,R., Morano,N., Burton,W., Montgomery,J., Breiman,R. and Nokes,D.J.
EPI_ISL_2593390	College of Medicine and Medical Research Institute, Chungbuk National University	College of Medicine and Medical Research Institute, Chungbuk National University	Baek,Y.H., Choi,E.H., Park,S.-J., Song,M.-S. and Choi,Y.-K.
EPI_ISL_2593391, EPI_ISL_2593392	Department of Epidemiology and Demography, Kenya Medical Research Institute (KEMRI) - Wellcome Trust Research Programme	Department of Epidemiology and Demography, Kenya Medical Research Institute (KEMRI) - Wellcome Trust Research Programme	Otieno,J.R., Agoti,C.N., Gitahi,C.W., Bett,A., Ngama,M., Medley,G.F., Cane,P.A. and Nokes,J.D.
EPI_ISL_2593393	Epidemiology and Demography, KEMRI-Wellcome Trust Research Collaborative Programme	Epidemiology and Demography, KEMRI-Wellcome Trust Research Collaborative Programme	Agoti,C.N., Mayieka,L., Otieno,J.R., Ahmed,J.A., Fields,B., Waiboci,L.B., Nyoka,R., Eidex,R., Morano,N., Burton,W., Montgomery,J., Breiman,R. and Nokes,D.J.
EPI_ISL_2593394	Department of Clinical Laboratory, Fujian Provincial Hospital	Department of Clinical Laboratory, Fujian Provincial Hospital	Su,Y., Wu,Y., Tian,R. and Liang,G.
EPI_ISL_2593395	Epidemiology and Demography, KEMRI-Wellcome Trust Research Collaborative Programme	Epidemiology and Demography, KEMRI-Wellcome Trust Research Collaborative Programme	Agoti,C.N., Mayieka,L., Otieno,J.R., Ahmed,J.A., Fields,B., Waiboci,L.B., Nyoka,R., Eidex,R., Morano,N., Burton,W., Montgomery,J., Breiman,R. and Nokes,D.J.
EPI_ISL_2593396, EPI_ISL_2593397, EPI_ISL_2593398, EPI_ISL_2593399, EPI_ISL_2593400, EPI_ISL_2593401	Department of Epidemiology and Demography, Kenya Medical Research Institute (KEMRI) - Wellcome Trust Research Programme	Department of Epidemiology and Demography, Kenya Medical Research Institute (KEMRI) - Wellcome Trust Research Programme	Otieno,J.R., Agoti,C.N., Gitahi,C.W., Bett,A., Ngama,M., Medley,G.F., Cane,P.A. and Nokes,J.D.
EPI_ISL_2593402, EPI_ISL_2593403, EPI_ISL_2593404, EPI_ISL_2593405	Virology, Tropical Medicine Institute Pedro Kouri	Virology, Tropical Medicine Institute Pedro Kouri	Valdes,O., Corso,M., Pinon,A., Acosta,B., Savon,C., Gonzalez,G., Mune,M., Gonzalez,G., Hernandez,B., Echevarria,Y. and Oropesa,S.
EPI_ISL_2593406	Epidemiology and Demography, KEMRI-Wellcome Trust Research Collaborative Programme	Epidemiology and Demography, KEMRI-Wellcome Trust Research Collaborative Programme	Agoti,C.N., Mayieka,L., Otieno,J.R., Ahmed,J.A., Fields,B., Waiboci,L.B., Nyoka,R., Eidex,R., Morano,N., Burton,W., Montgomery,J., Breiman,R. and Nokes,D.J.
EPI_ISL_2593407	College of Medicine and Medical Research Institute, Chungbuk National University	College of Medicine and Medical Research Institute, Chungbuk National University	Baek,Y.H., Choi,E.H., Park,S.-J., Song,M.-S. and Choi,Y.-K.
EPI_ISL_2593408, EPI_ISL_2593409, EPI_ISL_2593410	Department of Epidemiology and Demography, Kenya Medical Research Institute (KEMRI) - Wellcome Trust Research Programme	Department of Epidemiology and Demography, Kenya Medical Research Institute (KEMRI) - Wellcome Trust Research Programme	Otieno,J.R., Agoti,C.N., Gitahi,C.W., Bett,A., Ngama,M., Medley,G.F., Cane,P.A. and Nokes,J.D.
EPI_ISL_2593411, EPI_ISL_2593412, EPI_ISL_2593413, EPI_ISL_2593414, EPI_ISL_2593415	Epidemiology and Demography, KEMRI-Wellcome Trust Research Collaborative Programme	Epidemiology and Demography, KEMRI-Wellcome Trust Research Collaborative Programme	Agoti,C.N., Mayieka,L., Otieno,J.R., Ahmed,J.A., Fields,B., Waiboci,L.B., Nyoka,R., Eidex,R., Morano,N., Burton,W., Montgomery,J., Breiman,R. and Nokes,D.J.
EPI_ISL_2593416	Department of Epidemiology and Demography, Kenya Medical Research Institute (KEMRI) - Wellcome Trust Research Programme	Department of Epidemiology and Demography, Kenya Medical Research Institute (KEMRI) - Wellcome Trust Research Programme	Otieno,J.R., Agoti,C.N., Gitahi,C.W., Bett,A., Ngama,M., Medley,G.F., Cane,P.A. and Nokes,J.D.
EPI_ISL_2593417	Epidemiology and Demography, KEMRI-Wellcome Trust Research Collaborative Programme	Epidemiology and Demography, KEMRI-Wellcome Trust Research Collaborative Programme	Agoti,C.N., Mayieka,L., Otieno,J.R., Ahmed,J.A., Fields,B., Waiboci,L.B., Nyoka,R., Eidex,R., Morano,N., Burton,W., Montgomery,J., Breiman,R. and Nokes,D.J.
EPI_ISL_2593418	Department of Epidemiology and Demography, Kenya Medical Research Institute (KEMRI) - Wellcome Trust Research Programme	Department of Epidemiology and Demography, Kenya Medical Research Institute (KEMRI) - Wellcome Trust Research Programme	Otieno,J.R., Agoti,C.N., Gitahi,C.W., Bett,A., Ngama,M., Medley,G.F., Cane,P.A. and Nokes,J.D.
EPI_ISL_2593419, EPI_ISL_2593420, EPI_ISL_2593421, EPI_ISL_2593422, EPI_ISL_2593423, EPI_ISL_2593424, EPI_ISL_2593425, EPI_ISL_2593426, EPI_ISL_2593427	Epidemiology and Demography, KEMRI-Wellcome Trust Research Collaborative Programme	Epidemiology and Demography, KEMRI-Wellcome Trust Research Collaborative Programme	Agoti,C.N., Mayieka,L., Otieno,J.R., Ahmed,J.A., Fields,B., Waiboci,L.B., Nyoka,R., Eidex,R., Morano,N., Burton,W., Montgomery,J., Breiman,R. and Nokes,D.J.
EPI_ISL_2593428	Department of Respiratory Medicine, Children's Hospital, Chongqing Medical University	Department of Respiratory Medicine, Children's Hospital, Chongqing Medical University	Ren,L. and Liu,E.
EPI_ISL_2593429	Depto. de Virologia, Instituto Oswaldo Cruz, FIOCRUZ, Laboratorio de Virus Respiratorios e do Sarampo	Depto. de Virologia, Instituto Oswaldo Cruz, FIOCRUZ, Laboratorio de Virus Respiratorios e do Sarampo	Machado,D.B.B., Motta,F.C., Mesquita,M.M.A., Souza,T.M.L. and Siqueira,M.M.

EPI_ISL_2593430	Virology, Tropical Medicine Institute Pedro Kouri	Virology, Tropical Medicine Institute Pedro Kouri	Valdes,O., Corso,M., Pinon,A., Acosta,B., Savon,C., Gonzalez,G., Mune,M., Gonzalez,G., Hernandez,B., Echevarria,Y. and Oropesa,S.
EPI_ISL_2593431	Epidemiology and Demography, KEMRI-Wellcome Trust Research Collaborative Programme	Epidemiology and Demography, KEMRI-Wellcome Trust Research Collaborative Programme	Agoti,C.N., Mayieka,L., Otieno,J.R., Ahmed,J.A., Fields,B., Waiboci,L.B., Nyoka,R., Eidex,R., Morano,N., Burton,W., Montgomery,J., Breiman,R. and Nokes,D.J.
EPI_ISL_2593432, EPI_ISL_2593433	Virology, Tropical Medicine Institute Pedro Kouri	Virology, Tropical Medicine Institute Pedro Kouri	Valdes,O., Corso,M., Pinon,A., Acosta,B., Savon,C., Gonzalez,G., Mune,M., Gonzalez,G., Hernandez,B., Echevarria,Y. and Oropesa,S.
EPI_ISL_2593434, EPI_ISL_2593435	Epidemiology and Demography, KEMRI-Wellcome Trust Research Collaborative Programme	Epidemiology and Demography, KEMRI-Wellcome Trust Research Collaborative Programme	Agoti,C.N., Mayieka,L., Otieno,J.R., Ahmed,J.A., Fields,B., Waiboci,L.B., Nyoka,R., Eidex,R., Morano,N., Burton,W., Montgomery,J., Breiman,R. and Nokes,D.J.
EPI_ISL_2593436	Depto. de Virologia, Instituto Oswaldo Cruz, FIOCRUZ, Laboratorio de Virus Respiratorios e do Sarampo	Depto. de Virologia, Instituto Oswaldo Cruz, FIOCRUZ, Laboratorio de Virus Respiratorios e do Sarampo	Machado,D.B.B., Motta,F.C., Mesquita,M.M.A., Souza,T.M.L. and Siqueira,M.M.
EPI_ISL_2593437, EPI_ISL_2593438	Epidemiology and Demography, KEMRI-Wellcome Trust Research Collaborative Programme	Epidemiology and Demography, KEMRI-Wellcome Trust Research Collaborative Programme	Agoti,C.N., Mayieka,L., Otieno,J.R., Ahmed,J.A., Fields,B., Waiboci,L.B., Nyoka,R., Eidex,R., Morano,N., Burton,W., Montgomery,J., Breiman,R. and Nokes,D.J.
EPI_ISL_2593439, EPI_ISL_2593440	Depto. de Virologia, Instituto Oswaldo Cruz, FIOCRUZ, Laboratorio de Virus Respiratorios e do Sarampo	Depto. de Virologia, Instituto Oswaldo Cruz, FIOCRUZ, Laboratorio de Virus Respiratorios e do Sarampo	Machado,D.B.B., Motta,F.C., Mesquita,M.M.A., Souza,T.M.L. and Siqueira,M.M.
EPI_ISL_2593441	Virology, Tropical Medicine Institute Pedro Kouri	Virology, Tropical Medicine Institute Pedro Kouri	Valdes,O., Corso,M., Pinon,A., Acosta,B., Savon,C., Gonzalez,G., Mune,M., Gonzalez,G., Hernandez,B., Echevarria,Y. and Oropesa,S.
EPI_ISL_2593442, EPI_ISL_2593443, EPI_ISL_2593444, EPI_ISL_2593445, EPI_ISL_2593446, EPI_ISL_2593447	Depto. de Virologia, Instituto Oswaldo Cruz, FIOCRUZ, Laboratorio de Virus Respiratorios e do Sarampo	Depto. de Virologia, Instituto Oswaldo Cruz, FIOCRUZ, Laboratorio de Virus Respiratorios e do Sarampo	Machado,D.B.B., Motta,F.C., Mesquita,M.M.A., Souza,T.M.L. and Siqueira,M.M.
EPI_ISL_2593448	Epidemiology and Demography, KEMRI-Wellcome Trust Research Collaborative Programme	Epidemiology and Demography, KEMRI-Wellcome Trust Research Collaborative Programme	Agoti,C.N., Mayieka,L., Otieno,J.R., Ahmed,J.A., Fields,B., Waiboci,L.B., Nyoka,R., Eidex,R., Morano,N., Burton,W., Montgomery,J., Breiman,R. and Nokes,D.J.
EPI_ISL_2593449	Depto. de Virologia, Instituto Oswaldo Cruz, FIOCRUZ, Laboratorio de Virus Respiratorios e do Sarampo	Depto. de Virologia, Instituto Oswaldo Cruz, FIOCRUZ, Laboratorio de Virus Respiratorios e do Sarampo	Machado,D.B.B., Motta,F.C., Mesquita,M.M.A., Souza,T.M.L. and Siqueira,M.M.
EPI_ISL_2593450	Epidemiology and Demography, KEMRI-Wellcome Trust Research Collaborative Programme	Epidemiology and Demography, KEMRI-Wellcome Trust Research Collaborative Programme	Agoti,C.N., Mayieka,L., Otieno,J.R., Ahmed,J.A., Fields,B., Waiboci,L.B., Nyoka,R., Eidex,R., Morano,N., Burton,W., Montgomery,J., Breiman,R. and Nokes,D.J.
EPI_ISL_2593451, EPI_ISL_2593452, EPI_ISL_2593453, EPI_ISL_2593454	Depto. de Virologia, Instituto Oswaldo Cruz, FIOCRUZ, Laboratorio de Virus Respiratorios e do Sarampo	Depto. de Virologia, Instituto Oswaldo Cruz, FIOCRUZ, Laboratorio de Virus Respiratorios e do Sarampo	Machado,D.B.B., Motta,F.C., Mesquita,M.M.A., Souza,T.M.L. and Siqueira,M.M.
EPI_ISL_2593455, EPI_ISL_2593456	Epidemiology and Demography, KEMRI-Wellcome Trust Research Collaborative Programme	Epidemiology and Demography, KEMRI-Wellcome Trust Research Collaborative Programme	Agoti,C.N., Mayieka,L., Otieno,J.R., Ahmed,J.A., Fields,B., Waiboci,L.B., Nyoka,R., Eidex,R., Morano,N., Burton,W., Montgomery,J., Breiman,R. and Nokes,D.J.
EPI_ISL_2593457	Virology, Tropical Medicine Institute Pedro Kouri	Virology, Tropical Medicine Institute Pedro Kouri	Valdes,O., Corso,M., Pinon,A., Acosta,B., Savon,C., Gonzalez,G., Mune,M., Gonzalez,G., Hernandez,B., Echevarria,Y. and Oropesa,S.
EPI_ISL_2593458	WHO WPRO Measles Regional Reference Lab, Key Laboratory of Medical Virology Ministry of Health, National Institute for Viral Disease Control and Prevention, China Center for Disease Control and Prevention	WHO WPRO Measles Regional Reference Lab, Key Laboratory of Medical Virology Ministry of Health, National Institute for Viral Disease Control and Prevention, China Center for Disease Control and Prevention	Zhang,Y., Song,J. and Xu,W.
EPI_ISL_2593459	Virology, Faculty of Public Health, Tehran University of Medical Sciences	Virology, Faculty of Public Health, Tehran University of Medical Sciences	Mokhtari-Azad,T., Faghihloo,E., Rezaei,F. and Salimi,V.
EPI_ISL_2593460	Virology, Tropical Medicine Institute Pedro Kouri	Virology, Tropical Medicine Institute Pedro Kouri	Valdes,O., Corso,M., Pinon,A., Acosta,B., Savon,C., Gonzalez,G., Mune,M., Gonzalez,G., Hernandez,B., Echevarria,Y. and Oropesa,S.
EPI_ISL_2593461, EPI_ISL_2593462, EPI_ISL_2593463, EPI_ISL_2593464	College of Medicine and Medical Research Institute, Chungbuk National University	College of Medicine and Medical Research Institute, Chungbuk National University	Baek,Y.H., Choi,E.H., Park,S.-J., Song,M.-S. and Choi,Y.-K.
EPI_ISL_2593465, EPI_ISL_2593466, EPI_ISL_2593467, EPI_ISL_2593468, EPI_ISL_2593469, EPI_ISL_2593470	Virology, Faculty of Public Health, Tehran University of Medical Sciences	Virology, Faculty of Public Health, Tehran University of Medical Sciences	Mokhtari-Azad,T., Faghihloo,E., Rezaei,F. and Salimi,V.
EPI_ISL_2593471	Virology, Tropical Medicine Institute Pedro Kouri	Virology, Tropical Medicine Institute Pedro Kouri	Valdes,O., Corso,M., Pinon,A., Acosta,B., Savon,C., Gonzalez,G., Mune,M., Gonzalez,G., Hernandez,B., Echevarria,Y. and Oropesa,S.
EPI_ISL_2593472	Department of Clinical Laboratory, Fujian Provincial Hospital	Department of Clinical Laboratory, Fujian Provincial Hospital	Su,Y., Wu,Y., Tian,R. and Liang,G.
EPI_ISL_2593473, EPI_ISL_2593474	Pathogen Diagnostic Center, Institut Pasteur of Shanghai	Pathogen Diagnostic Center, Institut Pasteur of Shanghai	Liu,J., Mu,Y., Dong,W., Yao,F., Yan,H., Lan,K. and Zhang,C.
EPI_ISL_2593475, EPI_ISL_2593476, EPI_ISL_2593477	Epidemiology and Demography, KEMRI-Wellcome Trust Research Collaborative Programme	Epidemiology and Demography, KEMRI-Wellcome Trust Research Collaborative Programme	Agoti,C.N., Mayieka,L., Otieno,J.R., Ahmed,J.A., Fields,B., Waiboci,L.B., Nyoka,R., Eidex,R., Morano,N., Burton,W., Montgomery,J., Breiman,R. and Nokes,D.J.
EPI_ISL_2593478	Division of Public Health Research, Gyeonggi Province institute of Health and Environment	Division of Public Health Research, Gyeonggi Province institute of Health and Environment	Park,E., Park,P., Huh,J., Yun,H., Lee,H., Yoon,M., Lee,S. and Ko,G.
EPI_ISL_2593479, EPI_ISL_2593480, EPI_ISL_2593481, EPI_ISL_2593482, EPI_ISL_2593483	Pediatric Clinic 1, Department of Pathophysiology and Transplantation, University of Milan and Fondazione IRCCS Ca Granda	Pediatric Clinic 1, Department of Pathophysiology and Transplantation, University of Milan and Fondazione IRCCS Ca Granda	Esposito,S., Zampiero,A., Piralla,A. and Principi,N.
EPI_ISL_2593484, EPI_ISL_2593485, EPI_ISL_2593486	College of Medicine and Medical Research Institute, Chungbuk National University	College of Medicine and Medical Research Institute, Chungbuk National University	Baek,Y.H., Choi,E.H., Park,S.-J., Song,M.-S. and Choi,Y.-K.
EPI_ISL_2593487, EPI_ISL_2593488, EPI_ISL_2593489	Virology, Faculty of Public Health, Tehran University of Medical Sciences	Virology, Faculty of Public Health, Tehran University of Medical Sciences	Mokhtari-Azad,T., Faghihloo,E., Rezaei,F. and Salimi,V.
EPI_ISL_2593490, EPI_ISL_2593491, EPI_ISL_2593492, EPI_ISL_2593493, EPI_ISL_2593494	Epidemiology and Demography, KEMRI-Wellcome Trust Research Collaborative Programme	Epidemiology and Demography, KEMRI-Wellcome Trust Research Collaborative Programme	Agoti,C.N., Mayieka,L., Otieno,J.R., Ahmed,J.A., Fields,B., Waiboci,L.B., Nyoka,R., Eidex,R., Morano,N., Burton,W., Montgomery,J., Breiman,R. and Nokes,D.J.
EPI_ISL_2593495	Department of Epidemiology and Demography, Kenya Medical Research Institute (KEMRI) - Wellcome Trust Research Programme	Department of Epidemiology and Demography, Kenya Medical Research Institute (KEMRI) - Wellcome Trust Research Programme	Otieno,J.R., Agoti,C.N., Gitahi,C.W., Bett,A., Ngama,M., Medley,G.F., Cane,P.A. and Nokes,J.D.
EPI_ISL_2593496, EPI_ISL_2593497, EPI_ISL_2593498, EPI_ISL_2593499, EPI_ISL_2593500, EPI_ISL_2593501	Epidemiology and Demography, KEMRI-Wellcome Trust Research Collaborative Programme	Epidemiology and Demography, KEMRI-Wellcome Trust Research Collaborative Programme	Agoti,C.N., Mayieka,L., Otieno,J.R., Ahmed,J.A., Fields,B., Waiboci,L.B., Nyoka,R., Eidex,R., Morano,N., Burton,W., Montgomery,J., Breiman,R. and Nokes,D.J.
EPI_ISL_2593502	Division of Public Health Research, Gyeonggi Province institute of Health and Environment	Division of Public Health Research, Gyeonggi Province institute of Health and Environment	Park,E., Park,P., Huh,J., Yun,H., Lee,H., Yoon,M., Lee,S. and Ko,G.
EPI_ISL_2593503, EPI_ISL_2593504	Epidemiology and Demography, KEMRI-Wellcome Trust Research Collaborative Programme	Epidemiology and Demography, KEMRI-Wellcome Trust Research Collaborative Programme	Agoti,C.N., Mayieka,L., Otieno,J.R., Ahmed,J.A., Fields,B., Waiboci,L.B., Nyoka,R., Eidex,R., Morano,N., Burton,W., Montgomery,J., Breiman,R. and Nokes,D.J.
EPI_ISL_2593505	Influenza Group, National Institute of Virology	Influenza Group, National Institute of Virology	Choudhary,M.L., Anand,S.P., Wadhwa,B.S. and Chadha,M.S.
EPI_ISL_2593506, EPI_ISL_2593507, EPI_ISL_2593508, EPI_ISL_2593509, EPI_ISL_2593510, EPI_ISL_2593511, EPI_ISL_2593512, EPI_ISL_2593513, EPI_ISL_2593514, EPI_ISL_2593515	Epidemiology and Demography, KEMRI-Wellcome Trust Research Collaborative Programme	Epidemiology and Demography, KEMRI-Wellcome Trust Research Collaborative Programme	Agoti,C.N., Mayieka,L., Otieno,J.R., Ahmed,J.A., Fields,B., Waiboci,L.B., Nyoka,R., Eidex,R., Morano,N., Burton,W., Montgomery,J., Breiman,R. and Nokes,D.J.
EPI_ISL_2593516	Department of Epidemiology and Demography, Kenya Medical Research Institute (KEMRI) - Wellcome Trust Research Programme	Department of Epidemiology and Demography, Kenya Medical Research Institute (KEMRI) - Wellcome Trust Research Programme	Otieno,J.R., Agoti,C.N., Gitahi,C.W., Bett,A., Ngama,M., Medley,G.F., Cane,P.A. and Nokes,J.D.
EPI_ISL_2593517	Pathogen Diagnostic Center, Institut Pasteur of Shanghai	Pathogen Diagnostic Center, Institut Pasteur of Shanghai	Liu,J., Mu,Y., Dong,W., Yao,F., Yan,H., Lan,K. and Zhang,C.
EPI_ISL_2593518, EPI_ISL_2593519	Epidemiology and Demography, KEMRI-Wellcome Trust Research Collaborative Programme	Epidemiology and Demography, KEMRI-Wellcome Trust Research Collaborative Programme	Agoti,C.N., Mayieka,L., Otieno,J.R., Ahmed,J.A., Fields,B., Waiboci,L.B., Nyoka,R., Eidex,R., Morano,N., Burton,W., Montgomery,J., Breiman,R. and Nokes,D.J.
EPI_ISL_2593520	Department of Clinical Laboratory, Fujian Provincial Hospital	Department of Clinical Laboratory, Fujian Provincial Hospital	Su,Y., Wu,Y., Tian,R. and Liang,G.
EPI_ISL_2593521, EPI_ISL_2593522, EPI_ISL_2593523, EPI_ISL_2593524, EPI_ISL_2593525, EPI_ISL_2593526, EPI_ISL_2593527	Epidemiology and Demography, KEMRI-Wellcome Trust Research Collaborative Programme	Epidemiology and Demography, KEMRI-Wellcome Trust Research Collaborative Programme	Agoti,C.N., Mayieka,L., Otieno,J.R., Ahmed,J.A., Fields,B., Waiboci,L.B., Nyoka,R., Eidex,R., Morano,N., Burton,W., Montgomery,J., Breiman,R. and Nokes,D.J.
EPI_ISL_2593528	Influenza Group, National Institute of Virology	Influenza Group, National Institute of Virology	Choudhary,M.L., Anand,S.P., Wadhwa,B.S. and Chadha,M.S.
EPI_ISL_2593529, EPI_ISL_2593530, EPI_ISL_2593531, EPI_ISL_2593532	Department of Epidemiology and Demography, Kenya Medical Research Institute (KEMRI) - Wellcome Trust Research Programme	Department of Epidemiology and Demography, Kenya Medical Research Institute (KEMRI) - Wellcome Trust Research Programme	Otieno,J.R., Agoti,C.N., Gitahi,C.W., Bett,A., Ngama,M., Medley,G.F., Cane,P.A. and Nokes,J.D.
EPI_ISL_2593533, EPI_ISL_2593534, EPI_ISL_2593535	Epidemiology and Demography, KEMRI-Wellcome Trust Research Collaborative Programme	Epidemiology and Demography, KEMRI-Wellcome Trust Research Collaborative Programme	Agoti,C.N., Mayieka,L., Otieno,J.R., Ahmed,J.A., Fields,B., Waiboci,L.B., Nyoka,R., Eidex,R., Morano,N., Burton,W., Montgomery,J., Breiman,R. and Nokes,D.J.
EPI_ISL_2593536, EPI_ISL_2593537, EPI_ISL_2593538, EPI_ISL_2593539, EPI_ISL_2593540, EPI_ISL_2593541, EPI_ISL_2593542, EPI_ISL_2593543	Chinese Academy of Medical Sciences & Peking Union Medical College	Chinese Academy of Medical Sciences & Peking Union Medical College	Jia,B., Xiao,Y., Wang,Y., Chen,L., Zhang,J., Ren,L. and Wang,J.
EPI_ISL_2593544	WHO WPRO Measles Regional Reference Lab, Key Laboratory of Medical Virology Ministry of Health, National Institute for Viral Disease Control and Prevention, China Center for Disease Control and Prevention	WHO WPRO Measles Regional Reference Lab, Key Laboratory of Medical Virology Ministry of Health, National Institute for Viral Disease Control and Prevention, China Center for Disease Control and Prevention	Zhang,Y., Song,J. and Xu,W.
EPI_ISL_2593545, EPI_ISL_2593546, EPI_ISL_2593547	University of Wuerzburg, Institute of Virology and Immunobiology	University of Wuerzburg, Institute of Virology and Immunobiology	Prifert,C., Hofmann,D. and Weissbrich,B.
EPI_ISL_2593548	Chinese Academy of Medical Sciences & Peking Union Medical College	Chinese Academy of Medical Sciences & Peking Union Medical College	Jia,B., Xiao,Y., Wang,Y., Chen,L., Zhang,J., Ren,L. and Wang,J.
EPI_ISL_2593549, EPI_ISL_2593550	University of Wuerzburg, Institute of Virology and Immunobiology	University of Wuerzburg, Institute of Virology and Immunobiology	Prifert,C., Hofmann,D. and Weissbrich,B.
EPI_ISL_2593551	Department of Infectious Diseases, National Health Institute Doutor Ricardo Jorge	Department of Infectious Diseases, National Health Institute Doutor Ricardo Jorge	Saez-Lopez,E., Cristovao,P., Costa,I., Pechirra,P., Conde,P. and Guiomar,R.
EPI_ISL_2593552, EPI_ISL_2593553	Chinese Academy of Medical Sciences & Peking Union Medical College	Chinese Academy of Medical Sciences & Peking Union Medical College	Jia,B., Xiao,Y., Wang,Y., Chen,L., Zhang,J., Ren,L. and Wang,J.
EPI_ISL_2593554	WHO WPRO Measles Regional Reference Lab, Key Laboratory of Medical Virology Ministry of Health, National Institute for Viral Disease Control and Prevention, China Center for Disease Control and Prevention	WHO WPRO Measles Regional Reference Lab, Key Laboratory of Medical Virology Ministry of Health, National Institute for Viral Disease Control and Prevention, China Center for Disease Control and Prevention	Zhang,Y., Song,J. and Xu,W.
EPI_ISL_2593555, EPI_ISL_2593556, EPI_ISL_2593557	University of Wuerzburg, Institute of Virology and Immunobiology	University of Wuerzburg, Institute of Virology and Immunobiology	Prifert,C., Hofmann,D. and Weissbrich,B.
EPI_ISL_2593558	Department of Biomedical Sciences for Health, University of Milan	Department of Biomedical Sciences for Health, University of Milan	Martinelli,M., Frati,E.R., Zappa,A., Ebranati,E., Bianchi,S., Pariani,E., Amendola,A., Zehender,G. and Tanzi,E.

EPI_ISL_2593559, EPI_ISL_2593560, EPI_ISL_2593561, EPI_ISL_2593562, EPI_ISL_2593563	Pediatrics, Mackay Memorial Hospital	Pediatrics, Mackay Memorial Hospital	Chi,H., Hsiao,K.-L., Weng,L.-C., Chiu,N.-C., Huang,L.-M., Chiu,Y.-Y., Liu,C.-P. and Liu,H.-F.
EPI_ISL_2593564	Pediatrics, Show-Chwan Memorial Hospital	Pediatrics, Show-Chwan Memorial Hospital	Lee,C.-Y.
EPI_ISL_2593565, EPI_ISL_2593566, EPI_ISL_2593567	Pediatrics, Mackay Memorial Hospital	Pediatrics, Mackay Memorial Hospital	Chi,H., Hsiao,K.-L., Weng,L.-C., Chiu,N.-C., Huang,L.-M., Chiu,Y.-Y., Liu,C.-P. and Liu,H.-F.
EPI_ISL_2593568	Pediatrics, Show-Chwan Memorial Hospital	Pediatrics, Show-Chwan Memorial Hospital	Lee,C.-Y.
EPI_ISL_2593569, EPI_ISL_2593570, EPI_ISL_2593571	Pediatrics, Mackay Memorial Hospital	Pediatrics, Mackay Memorial Hospital	Chi,H., Hsiao,K.-L., Weng,L.-C., Chiu,N.-C., Huang,L.-M., Chiu,Y.-Y., Liu,C.-P. and Liu,H.-F.
EPI_ISL_2593572, EPI_ISL_2593573, EPI_ISL_2593574	Pediatrics, Show-Chwan Memorial Hospital	Pediatrics, Show-Chwan Memorial Hospital	Lee,C.-Y.
EPI_ISL_2593575, EPI_ISL_2593576, EPI_ISL_2593577, EPI_ISL_2593578, EPI_ISL_2593579, EPI_ISL_2593580, EPI_ISL_2593581, EPI_ISL_2593582	Pediatrics, Mackay Memorial Hospital	Pediatrics, Mackay Memorial Hospital	Chi,H., Hsiao,K.-L., Weng,L.-C., Chiu,N.-C., Huang,L.-M., Chiu,Y.-Y., Liu,C.-P. and Liu,H.-F.
EPI_ISL_2593583	Pediatrics, Show-Chwan Memorial Hospital	Pediatrics, Show-Chwan Memorial Hospital	Lee,C.-Y.
EPI_ISL_2593584	Department of Biomedical Sciences for Health, University of Milan	Department of Biomedical Sciences for Health, University of Milan	Martinelli,M., Frati,E.R., Zappa,A., Ebranati,E., Bianchi,S., Pariani,E., Amendola,A., Zehender,G. and Tanzi,E.
EPI_ISL_2593585	Pediatrics, Show-Chwan Memorial Hospital	Pediatrics, Show-Chwan Memorial Hospital	Lee,C.-Y.
EPI_ISL_2593586	Laboratory of Virology, Capital Institute of Pediatrics	Laboratory of Virology, Capital Institute of Pediatrics	Cui,G., Deng,J., Zhu,R., Qian,Y., Sun,Y., Zhao,L. and Wang,F.
EPI_ISL_2593587	Chinese Academy of Medical Sciences & Peking Union Medical College	Chinese Academy of Medical Sciences & Peking Union Medical College	Jia,B., Xiao,Y., Wang,Y., Chen,L., Zhang,J., Ren,L. and Wang,J.
EPI_ISL_2593588	Laboratory of Virology, Capital Institute of Pediatrics	Laboratory of Virology, Capital Institute of Pediatrics	Cui,G., Deng,J., Zhu,R., Qian,Y., Sun,Y., Zhao,L. and Wang,F.
EPI_ISL_2593589, EPI_ISL_2593590, EPI_ISL_2593591, EPI_ISL_2593592, EPI_ISL_2593593, EPI_ISL_2593594, EPI_ISL_2593595, EPI_ISL_2593596, EPI_ISL_2593597, EPI_ISL_2593598	Chinese Academy of Medical Sciences & Peking Union Medical College	Chinese Academy of Medical Sciences & Peking Union Medical College	Jia,B., Xiao,Y., Wang,Y., Chen,L., Zhang,J., Ren,L. and Wang,J.
EPI_ISL_2593599, EPI_ISL_2593600, EPI_ISL_2593601, EPI_ISL_2593602	Laboratory of Virology, Capital Institute of Pediatrics	Laboratory of Virology, Capital Institute of Pediatrics	Cui,G., Deng,J., Zhu,R., Qian,Y., Sun,Y., Zhao,L. and Wang,F.
EPI_ISL_2593603, EPI_ISL_2593604, EPI_ISL_2593605, EPI_ISL_2593606, EPI_ISL_2593607, EPI_ISL_2593608, EPI_ISL_2593609, EPI_ISL_2593610, EPI_ISL_2593611, EPI_ISL_2593612, EPI_ISL_2593613, EPI_ISL_2593614, EPI_ISL_2593615			
see above	Chinese Academy of Medical Sciences & Peking Union Medical College	Chinese Academy of Medical Sciences & Peking Union Medical College	Jia,B., Xiao,Y., Wang,Y., Chen,L., Zhang,J., Ren,L. and Wang,J.
EPI_ISL_2593616, EPI_ISL_2593617, EPI_ISL_2593618, EPI_ISL_2593619	Laboratory of Virology, Capital Institute of Pediatrics	Laboratory of Virology, Capital Institute of Pediatrics	Cui,G., Deng,J., Zhu,R., Qian,Y., Sun,Y., Zhao,L. and Wang,F.
EPI_ISL_2593620, EPI_ISL_2593621, EPI_ISL_2593622, EPI_ISL_2593623	Chinese Academy of Medical Sciences & Peking Union Medical College	Chinese Academy of Medical Sciences & Peking Union Medical College	Jia,B., Xiao,Y., Wang,Y., Chen,L., Zhang,J., Ren,L. and Wang,J.
EPI_ISL_2593624	Laboratory of Virology, Capital Institute of Pediatrics	Laboratory of Virology, Capital Institute of Pediatrics	Cui,G., Deng,J., Zhu,R., Qian,Y., Sun,Y., Zhao,L. and Wang,F.
EPI_ISL_2593625, EPI_ISL_2593626	Chinese Academy of Medical Sciences & Peking Union Medical College	Chinese Academy of Medical Sciences & Peking Union Medical College	Jia,B., Xiao,Y., Wang,Y., Chen,L., Zhang,J., Ren,L. and Wang,J.
EPI_ISL_2593627	Laboratory of Virology, Capital Institute of Pediatrics	Laboratory of Virology, Capital Institute of Pediatrics	Cui,G., Deng,J., Zhu,R., Qian,Y., Sun,Y., Zhao,L. and Wang,F.
EPI_ISL_2593628, EPI_ISL_2593629, EPI_ISL_2593630	Chinese Academy of Medical Sciences & Peking Union Medical College	Chinese Academy of Medical Sciences & Peking Union Medical College	Jia,B., Xiao,Y., Wang,Y., Chen,L., Zhang,J., Ren,L. and Wang,J.
EPI_ISL_2593631, EPI_ISL_2593632	Laboratory of Virology, Capital Institute of Pediatrics	Laboratory of Virology, Capital Institute of Pediatrics	Cui,G., Deng,J., Zhu,R., Qian,Y., Sun,Y., Zhao,L. and Wang,F.
EPI_ISL_2593633, EPI_ISL_2593634, EPI_ISL_2593635, EPI_ISL_2593636, EPI_ISL_2593637, EPI_ISL_2593638	Pediatric Clinic 1, Department of Pathophysiology and Transplantation, University of Milan and Fondazione IRCCS Ca Granda	Pediatric Clinic 1, Department of Pathophysiology and Transplantation, University of Milan and Fondazione IRCCS Ca Granda	Esposito,S., Zampiero,A., Piralla,A. and Principi,N.
EPI_ISL_2593639, EPI_ISL_2593640, EPI_ISL_2593641, EPI_ISL_2593642, EPI_ISL_2593643	College of Medicine and Medical Research Institute, Chungbuk National University	College of Medicine and Medical Research Institute, Chungbuk National University	Baek,Y.H., Choi,E.H., Park,S.-J., Song,M.-S. and Choi,Y.-K.
EPI_ISL_2593644, EPI_ISL_2593645, EPI_ISL_2593646, EPI_ISL_2593647	Epidemiology and Demography, KEMRI-Wellcome Trust Research Collaborative Programme	Epidemiology and Demography, KEMRI-Wellcome Trust Research Collaborative Programme	Agoti,C.N., Mayieka,L., Otieno,J.R., Ahmed,J.A., Fields,B., Waiboci,L.B., Nyoka,R., Eidex,R., Morano,N., Burton,W., Montgomery,J., Breiman,R. and Nokes,D.J.
EPI_ISL_2593648	Department of Epidemiology and Demography, Kenya Medical Research Institute (KEMRI) - Wellcome Trust Research Programme	Department of Epidemiology and Demography, Kenya Medical Research Institute (KEMRI) - Wellcome Trust Research Programme	Otieno,J.R., Agoti,C.N., Gitahi,C.W., Bett,A., Ngama,M., Medley,G.F., Cane,P.A. and Nokes,J.D.
EPI_ISL_2593649	Epidemiology and Demography, KEMRI-Wellcome Trust Research Collaborative Programme	Epidemiology and Demography, KEMRI-Wellcome Trust Research Collaborative Programme	Agoti,C.N., Mayieka,L., Otieno,J.R., Ahmed,J.A., Fields,B., Waiboci,L.B., Nyoka,R., Eidex,R., Morano,N., Burton,W., Montgomery,J., Breiman,R. and Nokes,D.J.
EPI_ISL_2593650	Division of Immunology, Children's Hospital	Division of Immunology, Children's Hospital	Qin,X., Zhang,C., Zhao,Y. and Zhao,X.
EPI_ISL_2593651	Department of Epidemiology and Demography, Kenya Medical Research Institute (KEMRI) - Wellcome Trust Research Programme	Department of Epidemiology and Demography, Kenya Medical Research Institute (KEMRI) - Wellcome Trust Research Programme	Otieno,J.R., Agoti,C.N., Gitahi,C.W., Bett,A., Ngama,M., Medley,G.F., Cane,P.A. and Nokes,J.D.
EPI_ISL_2593652	Epidemiology and Demography, KEMRI-Wellcome Trust Research Collaborative Programme	Epidemiology and Demography, KEMRI-Wellcome Trust Research Collaborative Programme	Agoti,C.N., Mayieka,L., Otieno,J.R., Ahmed,J.A., Fields,B., Waiboci,L.B., Nyoka,R., Eidex,R., Morano,N., Burton,W., Montgomery,J., Breiman,R. and Nokes,D.J.
EPI_ISL_2593653, EPI_ISL_2593654	Department of Epidemiology and Demography, Kenya Medical Research Institute (KEMRI) - Wellcome Trust Research Programme	Department of Epidemiology and Demography, Kenya Medical Research Institute (KEMRI) - Wellcome Trust Research Programme	Otieno,J.R., Agoti,C.N., Gitahi,C.W., Bett,A., Ngama,M., Medley,G.F., Cane,P.A. and Nokes,J.D.
EPI_ISL_2593655, EPI_ISL_2593656, EPI_ISL_2593657	Epidemiology and Demography, KEMRI-Wellcome Trust Research Collaborative Programme	Epidemiology and Demography, KEMRI-Wellcome Trust Research Collaborative Programme	Agoti,C.N., Mayieka,L., Otieno,J.R., Ahmed,J.A., Fields,B., Waiboci,L.B., Nyoka,R., Eidex,R., Morano,N., Burton,W., Montgomery,J., Breiman,R. and Nokes,D.J.
EPI_ISL_2593658, EPI_ISL_2593659	Department of Epidemiology and Demography, Kenya Medical Research Institute (KEMRI) - Wellcome Trust Research Programme	Department of Epidemiology and Demography, Kenya Medical Research Institute (KEMRI) - Wellcome Trust Research Programme	Otieno,J.R., Agoti,C.N., Gitahi,C.W., Bett,A., Ngama,M., Medley,G.F., Cane,P.A. and Nokes,J.D.
EPI_ISL_2593660, EPI_ISL_2593661, EPI_ISL_2593662	Epidemiology and Demography, KEMRI-Wellcome Trust Research Collaborative Programme	Epidemiology and Demography, KEMRI-Wellcome Trust Research Collaborative Programme	Agoti,C.N., Mayieka,L., Otieno,J.R., Ahmed,J.A., Fields,B., Waiboci,L.B., Nyoka,R., Eidex,R., Morano,N., Burton,W., Montgomery,J., Breiman,R. and Nokes,D.J.
EPI_ISL_2593663, EPI_ISL_2593664, EPI_ISL_2593665	Department of Epidemiology and Demography, Kenya Medical Research Institute (KEMRI) - Wellcome Trust Research Programme	Department of Epidemiology and Demography, Kenya Medical Research Institute (KEMRI) - Wellcome Trust Research Programme	Otieno,J.R., Agoti,C.N., Gitahi,C.W., Bett,A., Ngama,M., Medley,G.F., Cane,P.A. and Nokes,J.D.
EPI_ISL_2593666	Epidemiology and Demography, KEMRI-Wellcome Trust Research Collaborative Programme	Epidemiology and Demography, KEMRI-Wellcome Trust Research Collaborative Programme	Agoti,C.N., Mayieka,L., Otieno,J.R., Ahmed,J.A., Fields,B., Waiboci,L.B., Nyoka,R., Eidex,R., Morano,N., Burton,W., Montgomery,J., Breiman,R. and Nokes,D.J.
EPI_ISL_2593667, EPI_ISL_2593668, EPI_ISL_2593669, EPI_ISL_2593670, EPI_ISL_2593671, EPI_ISL_2593672	Department of Epidemiology and Demography, Kenya Medical Research Institute (KEMRI) - Wellcome Trust Research Programme	Department of Epidemiology and Demography, Kenya Medical Research Institute (KEMRI) - Wellcome Trust Research Programme	Otieno,J.R., Agoti,C.N., Gitahi,C.W., Bett,A., Ngama,M., Medley,G.F., Cane,P.A. and Nokes,J.D.
EPI_ISL_2593673	Epidemiology and Demography, KEMRI-Wellcome Trust Research Collaborative Programme	Epidemiology and Demography, KEMRI-Wellcome Trust Research Collaborative Programme	Agoti,C.N., Mayieka,L., Otieno,J.R., Ahmed,J.A., Fields,B., Waiboci,L.B., Nyoka,R., Eidex,R., Morano,N., Burton,W., Montgomery,J., Breiman,R. and Nokes,D.J.
EPI_ISL_2593674	Pathogen Diagnostic Center, Institut Pasteur of Shanghai	Pathogen Diagnostic Center, Institut Pasteur of Shanghai	Liu,J., Mu,Y., Dong,W., Yao,F., Yan,H., Lan,K. and Zhang,C.
EPI_ISL_2593675, EPI_ISL_2593676	Department of Epidemiology and Demography, Kenya Medical Research Institute (KEMRI) - Wellcome Trust Research Programme	Department of Epidemiology and Demography, Kenya Medical Research Institute (KEMRI) - Wellcome Trust Research Programme	Otieno,J.R., Agoti,C.N., Gitahi,C.W., Bett,A., Ngama,M., Medley,G.F., Cane,P.A. and Nokes,J.D.
EPI_ISL_2593677, EPI_ISL_2593678, EPI_ISL_2593679, EPI_ISL_2593680	Pediatric Clinic 1, Department of Pathophysiology and Transplantation, University of Milan and Fondazione IRCCS Ca Granda	Pediatric Clinic 1, Department of Pathophysiology and Transplantation, University of Milan and Fondazione IRCCS Ca Granda	Esposito,S., Zampiero,A., Piralla,A. and Principi,N.
EPI_ISL_2593681, EPI_ISL_2593682	College of Medicine and Medical Research Institute, Chungbuk National University	College of Medicine and Medical Research Institute, Chungbuk National University	Baek,Y.H., Choi,E.H., Park,S.-J., Song,M.-S. and Choi,Y.-K.
EPI_ISL_2593683, EPI_ISL_2593684, EPI_ISL_2593685, EPI_ISL_2593686, EPI_ISL_2593687, EPI_ISL_2593688, EPI_ISL_2593689, EPI_ISL_2593690, EPI_ISL_2593691, EPI_ISL_2593692, EPI_ISL_2593693, EPI_ISL_2593694, EPI_ISL_2593695, EPI_ISL_2593696, EPI_ISL_2593697			
see above	Department of Epidemiology and Demography, Kenya Medical Research Institute (KEMRI) - Wellcome Trust Research Programme	Department of Epidemiology and Demography, Kenya Medical Research Institute (KEMRI) - Wellcome Trust Research Programme	Otieno,J.R., Agoti,C.N., Gitahi,C.W., Bett,A., Ngama,M., Medley,G.F., Cane,P.A. and Nokes,J.D.
EPI_ISL_2593698	College of Medicine and Medical Research Institute, Chungbuk National University	College of Medicine and Medical Research Institute, Chungbuk National University	Baek,Y.H., Choi,E.H., Park,S.-J., Song,M.-S. and Choi,Y.-K.
EPI_ISL_2593699, EPI_ISL_2593700, EPI_ISL_2593701, EPI_ISL_2593702	Department of Epidemiology and Demography, Kenya Medical Research Institute (KEMRI) - Wellcome Trust Research Programme	Department of Epidemiology and Demography, Kenya Medical Research Institute (KEMRI) - Wellcome Trust Research Programme	Otieno,J.R., Agoti,C.N., Gitahi,C.W., Bett,A., Ngama,M., Medley,G.F., Cane,P.A. and Nokes,J.D.
EPI_ISL_2593703	Epidemiology and Demography, KEMRI-Wellcome Trust Research Collaborative Programme	Epidemiology and Demography, KEMRI-Wellcome Trust Research Collaborative Programme	Agoti,C.N., Mayieka,L., Otieno,J.R., Ahmed,J.A., Fields,B., Waiboci,L.B., Nyoka,R., Eidex,R., Morano,N., Burton,W., Montgomery,J., Breiman,R. and Nokes,D.J.
EPI_ISL_2593704, EPI_ISL_2593705, EPI_ISL_2593706, EPI_ISL_2593707, EPI_ISL_2593708, EPI_ISL_2593709, EPI_ISL_2593710, EPI_ISL_2593711	Department of Epidemiology and Demography, Kenya Medical Research Institute (KEMRI) - Wellcome Trust Research Programme	Department of Epidemiology and Demography, Kenya Medical Research Institute (KEMRI) - Wellcome Trust Research Programme	Otieno,J.R., Agoti,C.N., Gitahi,C.W., Bett,A., Ngama,M., Medley,G.F., Cane,P.A. and Nokes,J.D.
EPI_ISL_2593712	Depto. de Virologia, Instituto Oswaldo Cruz, FIOCRUZ, Laboratorio de Virus Respiratorios e do Sarampo	Depto. de Virologia, Instituto Oswaldo Cruz, FIOCRUZ, Laboratorio de Virus Respiratorios e do Sarampo	Machado,D.B.B., Motta,F.C., Mesquita,M.M.A., Souza,T.M.L. and Siqueira,M.M.
EPI_ISL_2593713, EPI_ISL_2593714, EPI_ISL_2593715, EPI_ISL_2593716, EPI_ISL_2593717, EPI_ISL_2593718	Department of Epidemiology and Demography, Kenya Medical Research Institute (KEMRI) - Wellcome Trust Research Programme	Department of Epidemiology and Demography, Kenya Medical Research Institute (KEMRI) - Wellcome Trust Research Programme	Otieno,J.R., Agoti,C.N., Gitahi,C.W., Bett,A., Ngama,M., Medley,G.F., Cane,P.A. and Nokes,J.D.
EPI_ISL_2593719	Epidemiology and Demography, KEMRI-Wellcome Trust Research Collaborative Programme	Epidemiology and Demography, KEMRI-Wellcome Trust Research Collaborative Programme	Agoti,C.N., Mayieka,L., Otieno,J.R., Ahmed,J.A., Fields,B., Waiboci,L.B., Nyoka,R., Eidex,R., Morano,N., Burton,W., Montgomery,J., Breiman,R. and Nokes,D.J.
EPI_ISL_2593720, EPI_ISL_2593721, EPI_ISL_2593722, EPI_ISL_2593723, EPI_ISL_2593724, EPI_ISL_2593725, EPI_ISL_2593726	Department of Epidemiology and Demography, Kenya Medical Research Institute (KEMRI) - Wellcome Trust Research Programme	Department of Epidemiology and Demography, Kenya Medical Research Institute (KEMRI) - Wellcome Trust Research Programme	Otieno,J.R., Agoti,C.N., Gitahi,C.W., Bett,A., Ngama,M., Medley,G.F., Cane,P.A. and Nokes,J.D.
EPI_ISL_2593727	Department of Respiratory Medicine, Children's Hospital, Chongqing Medical University	Department of Respiratory Medicine, Children's Hospital, Chongqing Medical University	Ren,L. and Liu,E.

EPI_ISL_2593728, EPI_ISL_2593729, EPI_ISL_2593730, EPI_ISL_2593731, EPI_ISL_2593732	Department of Epidemiology and Demography, Kenya Medical Research Institute (KEMRI) - Wellcome Trust Research Programme	Department of Epidemiology and Demography, Kenya Medical Research Institute (KEMRI) - Wellcome Trust Research Programme	Otieno,J.R., Agoti,C.N., Gitahi,C.W., Bett,A., Ngama,M., Medley,G.F., Cane,P.A. and Nokes,J.D.
EPI_ISL_2593733	Depto. de Virologia, Instituto Oswaldo Cruz, FIOCRUZ, Laboratorio de Virus Respiratorios e do Sarampo	Depto. de Virologia, Instituto Oswaldo Cruz, FIOCRUZ, Laboratorio de Virus Respiratorios e do Sarampo	Machado,D.B.B., Motta,F.C., Mesquita,M.M.A., Souza,T.M.L. and Siqueira,M.M.
EPI_ISL_2593734	Epidemiology and Demography, KEMRI-Wellcome Trust Research Collaborative Programme	Epidemiology and Demography, KEMRI-Wellcome Trust Research Collaborative Programme	Agoti,C.N., Mayieka,L., Otieno,J.R., Ahmed,J.A., Fields,B., Waiboci,L.B., Nyoka,R., Eidex,R., Morano,N., Burton,W., Montgomery,J., Breiman,R. and Nokes,D.J.
EPI_ISL_2593735	Department of Epidemiology and Demography, Kenya Medical Research Institute (KEMRI) - Wellcome Trust Research Programme	Department of Epidemiology and Demography, Kenya Medical Research Institute (KEMRI) - Wellcome Trust Research Programme	Otieno,J.R., Agoti,C.N., Gitahi,C.W., Bett,A., Ngama,M., Medley,G.F., Cane,P.A. and Nokes,J.D.
EPI_ISL_2593736	Depto. de Virologia, Instituto Oswaldo Cruz, FIOCRUZ, Laboratorio de Virus Respiratorios e do Sarampo	Depto. de Virologia, Instituto Oswaldo Cruz, FIOCRUZ, Laboratorio de Virus Respiratorios e do Sarampo	Machado,D.B.B., Motta,F.C., Mesquita,M.M.A., Souza,T.M.L. and Siqueira,M.M.
EPI_ISL_2593737, EPI_ISL_2593738	Department of Epidemiology and Demography, Kenya Medical Research Institute (KEMRI) - Wellcome Trust Research Programme	Department of Epidemiology and Demography, Kenya Medical Research Institute (KEMRI) - Wellcome Trust Research Programme	Otieno,J.R., Agoti,C.N., Gitahi,C.W., Bett,A., Ngama,M., Medley,G.F., Cane,P.A. and Nokes,J.D.
EPI_ISL_2593739	Depto. de Virologia, Instituto Oswaldo Cruz, FIOCRUZ, Laboratorio de Virus Respiratorios e do Sarampo	Depto. de Virologia, Instituto Oswaldo Cruz, FIOCRUZ, Laboratorio de Virus Respiratorios e do Sarampo	Machado,D.B.B., Motta,F.C., Mesquita,M.M.A., Souza,T.M.L. and Siqueira,M.M.
EPI_ISL_2593740	Department of Epidemiology and Demography, Kenya Medical Research Institute (KEMRI) - Wellcome Trust Research Programme	Department of Epidemiology and Demography, Kenya Medical Research Institute (KEMRI) - Wellcome Trust Research Programme	Otieno,J.R., Agoti,C.N., Gitahi,C.W., Bett,A., Ngama,M., Medley,G.F., Cane,P.A. and Nokes,J.D.
EPI_ISL_2593741	Naoko Kiyota Kumamoto Prefectural Institute of Public-Health and Environmental Science, Department of Microbiology	Naoko Kiyota Kumamoto Prefectural Institute of Public-Health and Environmental Science, Department of Microbiology	Kiyota,N., Yoshida,A. and Kobayashi,M.
EPI_ISL_2593742, EPI_ISL_2593743, EPI_ISL_2593744	Department of Epidemiology and Demography, Kenya Medical Research Institute (KEMRI) - Wellcome Trust Research Programme	Department of Epidemiology and Demography, Kenya Medical Research Institute (KEMRI) - Wellcome Trust Research Programme	Otieno,J.R., Agoti,C.N., Gitahi,C.W., Bett,A., Ngama,M., Medley,G.F., Cane,P.A. and Nokes,J.D.
EPI_ISL_2593745, EPI_ISL_2593746	Depto. de Virologia, Instituto Oswaldo Cruz, FIOCRUZ, Laboratorio de Virus Respiratorios e do Sarampo	Depto. de Virologia, Instituto Oswaldo Cruz, FIOCRUZ, Laboratorio de Virus Respiratorios e do Sarampo	Machado,D.B.B., Motta,F.C., Mesquita,M.M.A., Souza,T.M.L. and Siqueira,M.M.
EPI_ISL_2593747, EPI_ISL_2593748	Department of Epidemiology and Demography, Kenya Medical Research Institute (KEMRI) - Wellcome Trust Research Programme	Department of Epidemiology and Demography, Kenya Medical Research Institute (KEMRI) - Wellcome Trust Research Programme	Otieno,J.R., Agoti,C.N., Gitahi,C.W., Bett,A., Ngama,M., Medley,G.F., Cane,P.A. and Nokes,J.D.
EPI_ISL_2593749	Division of Immunology, Children's Hospital	Division of Immunology, Children's Hospital	Qin,X., Zhang,C., Zhao,Y. and Zhao,X.
EPI_ISL_2593750, EPI_ISL_2593751	Department of Epidemiology and Demography, Kenya Medical Research Institute (KEMRI) - Wellcome Trust Research Programme	Department of Epidemiology and Demography, Kenya Medical Research Institute (KEMRI) - Wellcome Trust Research Programme	Otieno,J.R., Agoti,C.N., Gitahi,C.W., Bett,A., Ngama,M., Medley,G.F., Cane,P.A. and Nokes,J.D.
EPI_ISL_2593752, EPI_ISL_2593753	Epidemiology and Demography, KEMRI-Wellcome Trust Research Collaborative Programme	Epidemiology and Demography, KEMRI-Wellcome Trust Research Collaborative Programme	Agoti,C.N., Mayieka,L., Otieno,J.R., Ahmed,J.A., Fields,B., Waiboci,L.B., Nyoka,R., Eidex,R., Morano,N., Burton,W., Montgomery,J., Breiman,R. and Nokes,D.J.
EPI_ISL_2593754	Department of Epidemiology and Demography, Kenya Medical Research Institute (KEMRI) - Wellcome Trust Research Programme	Department of Epidemiology and Demography, Kenya Medical Research Institute (KEMRI) - Wellcome Trust Research Programme	Otieno,J.R., Agoti,C.N., Gitahi,C.W., Bett,A., Ngama,M., Medley,G.F., Cane,P.A. and Nokes,J.D.
EPI_ISL_2593755, EPI_ISL_2593756	Depto. de Virologia, Instituto Oswaldo Cruz, FIOCRUZ, Laboratorio de Virus Respiratorios e do Sarampo	Depto. de Virologia, Instituto Oswaldo Cruz, FIOCRUZ, Laboratorio de Virus Respiratorios e do Sarampo	Machado,D.B.B., Motta,F.C., Mesquita,M.M.A., Souza,T.M.L. and Siqueira,M.M.
EPI_ISL_2593757, EPI_ISL_2593758	Department of Epidemiology and Demography, Kenya Medical Research Institute (KEMRI) - Wellcome Trust Research Programme	Department of Epidemiology and Demography, Kenya Medical Research Institute (KEMRI) - Wellcome Trust Research Programme	Otieno,J.R., Agoti,C.N., Gitahi,C.W., Bett,A., Ngama,M., Medley,G.F., Cane,P.A. and Nokes,J.D.
EPI_ISL_2593759, EPI_ISL_2593760	Depto. de Virologia, Instituto Oswaldo Cruz, FIOCRUZ, Laboratorio de Virus Respiratorios e do Sarampo	Depto. de Virologia, Instituto Oswaldo Cruz, FIOCRUZ, Laboratorio de Virus Respiratorios e do Sarampo	Machado,D.B.B., Motta,F.C., Mesquita,M.M.A., Souza,T.M.L. and Siqueira,M.M.
EPI_ISL_2593761	Department of Epidemiology and Demography, Kenya Medical Research Institute (KEMRI) - Wellcome Trust Research Programme	Department of Epidemiology and Demography, Kenya Medical Research Institute (KEMRI) - Wellcome Trust Research Programme	Otieno,J.R., Agoti,C.N., Gitahi,C.W., Bett,A., Ngama,M., Medley,G.F., Cane,P.A. and Nokes,J.D.
EPI_ISL_2593762, EPI_ISL_2593763, EPI_ISL_2593765	Depto. de Virologia, Instituto Oswaldo Cruz, FIOCRUZ, Laboratorio de Virus Respiratorios e do Sarampo	Depto. de Virologia, Instituto Oswaldo Cruz, FIOCRUZ, Laboratorio de Virus Respiratorios e do Sarampo	Machado,D.B.B., Motta,F.C., Mesquita,M.M.A., Souza,T.M.L. and Siqueira,M.M.
EPI_ISL_2593766	West China School of Public Health, Sichuan University	West China School of Public Health, Sichuan University	Zhang,M.
EPI_ISL_2593767, EPI_ISL_2593768, EPI_ISL_2593769	Minoru Nidaira Okinawa Prefectural Institute of Health and Environment, Department of Biological Science	Minoru Nidaira Okinawa Prefectural Institute of Health and Environment, Department of Biological Science	Nidaira,M. and Taira,K.
EPI_ISL_2593770	Depto. de Virologia, Instituto Oswaldo Cruz, FIOCRUZ, Laboratorio de Virus Respiratorios e do Sarampo	Depto. de Virologia, Instituto Oswaldo Cruz, FIOCRUZ, Laboratorio de Virus Respiratorios e do Sarampo	Machado,D.B.B., Motta,F.C., Mesquita,M.M.A., Souza,T.M.L. and Siqueira,M.M.
EPI_ISL_2593771	Epidemiology and Demography, KEMRI-Wellcome Trust Research Collaborative Programme	Epidemiology and Demography, KEMRI-Wellcome Trust Research Collaborative Programme	Agoti,C.N., Mayieka,L., Otieno,J.R., Ahmed,J.A., Fields,B., Waiboci,L.B., Nyoka,R., Eidex,R., Morano,N., Burton,W., Montgomery,J., Breiman,R. and Nokes,D.J.
EPI_ISL_2593772	Department of Epidemiology and Demography, Kenya Medical Research Institute (KEMRI) - Wellcome Trust Research Programme	Department of Epidemiology and Demography, Kenya Medical Research Institute (KEMRI) - Wellcome Trust Research Programme	Otieno,J.R., Agoti,C.N., Gitahi,C.W., Bett,A., Ngama,M., Medley,G.F., Cane,P.A. and Nokes,J.D.
EPI_ISL_2593773	Depto. de Virologia, Instituto Oswaldo Cruz, FIOCRUZ, Laboratorio de Virus Respiratorios e do Sarampo	Depto. de Virologia, Instituto Oswaldo Cruz, FIOCRUZ, Laboratorio de Virus Respiratorios e do Sarampo	Machado,D.B.B., Motta,F.C., Mesquita,M.M.A., Souza,T.M.L. and Siqueira,M.M.
EPI_ISL_2593774, EPI_ISL_2593775	Minoru Nidaira Okinawa Prefectural Institute of Health and Environment, Department of Biological Science	Minoru Nidaira Okinawa Prefectural Institute of Health and Environment, Department of Biological Science	Nidaira,M. and Taira,K.
EPI_ISL_2593776	Depto. de Virologia, Instituto Oswaldo Cruz, FIOCRUZ, Laboratorio de Virus Respiratorios e do Sarampo	Depto. de Virologia, Instituto Oswaldo Cruz, FIOCRUZ, Laboratorio de Virus Respiratorios e do Sarampo	Machado,D.B.B., Motta,F.C., Mesquita,M.M.A., Souza,T.M.L. and Siqueira,M.M.
EPI_ISL_2593777	West China School of Public Health, Sichuan University	West China School of Public Health, Sichuan University	Zhang,M.
EPI_ISL_2593778	Depto. de Virologia, Instituto Oswaldo Cruz, FIOCRUZ, Laboratorio de Virus Respiratorios e do Sarampo	Depto. de Virologia, Instituto Oswaldo Cruz, FIOCRUZ, Laboratorio de Virus Respiratorios e do Sarampo	Machado,D.B.B., Motta,F.C., Mesquita,M.M.A., Souza,T.M.L. and Siqueira,M.M.
EPI_ISL_2593779	WHO WPRO Measles Regional Reference Lab, Key Laboratory of Medical Virology Ministry of Health, National Institute for Viral Disease Control and Prevention, China Center for Disease Control and Prevention	WHO WPRO Measles Regional Reference Lab, Key Laboratory of Medical Virology Ministry of Health, National Institute for Viral Disease Control and Prevention, China Center for Disease Control and Prevention	Zhang,Y., Song,J. and Xu,W.
EPI_ISL_2593780	Department of Respiratory Medicine, Children's Hospital, Chongqing Medical University	Department of Respiratory Medicine, Children's Hospital, Chongqing Medical University	Ren,L. and Liu,E.
EPI_ISL_2593781	Minoru Nidaira Okinawa Prefectural Institute of Health and Environment, Department of Biological Science	Minoru Nidaira Okinawa Prefectural Institute of Health and Environment, Department of Biological Science	Nidaira,M. and Taira,K.
EPI_ISL_2593782	WHO WPRO Measles Regional Reference Lab, Key Laboratory of Medical Virology Ministry of Health, National Institute for Viral Disease Control and Prevention, China Center for Disease Control and Prevention	WHO WPRO Measles Regional Reference Lab, Key Laboratory of Medical Virology Ministry of Health, National Institute for Viral Disease Control and Prevention, China Center for Disease Control and Prevention	Zhang,Y., Song,J. and Xu,W.
EPI_ISL_2593783	Division of Immunology, Children's Hospital	Division of Immunology, Children's Hospital	Qin,X., Zhang,C., Zhao,Y. and Zhao,X.
EPI_ISL_2593784, EPI_ISL_2593785	Influenza Group, National Institute of Virology	Influenza Group, National Institute of Virology	Choudhary,M.L., Anand,S.P., Wadhwa,B.S. and Chadha,M.S.
EPI_ISL_2593786	Division of Immunology, Children's Hospital	Division of Immunology, Children's Hospital	Qin,X., Zhang,C., Zhao,Y. and Zhao,X.
EPI_ISL_2593787	Department of Epidemiology and Demography, Kenya Medical Research Institute (KEMRI) - Wellcome Trust Research Programme	Department of Epidemiology and Demography, Kenya Medical Research Institute (KEMRI) - Wellcome Trust Research Programme	Otieno,J.R., Agoti,C.N., Gitahi,C.W., Bett,A., Ngama,M., Medley,G.F., Cane,P.A. and Nokes,J.D.
EPI_ISL_2593788	Influenza Group, National Institute of Virology	Influenza Group, National Institute of Virology	Choudhary,M.L., Anand,S.P., Wadhwa,B.S. and Chadha,M.S.
EPI_ISL_2593789, EPI_ISL_2593790	WHO WPRO Measles Regional Reference Lab, Key Laboratory of Medical Virology Ministry of Health, National Institute for Viral Disease Control and Prevention, China Center for Disease Control and Prevention	WHO WPRO Measles Regional Reference Lab, Key Laboratory of Medical Virology Ministry of Health, National Institute for Viral Disease Control and Prevention, China Center for Disease Control and Prevention	Zhang,Y., Song,J. and Xu,W.
EPI_ISL_2593791	Influenza Group, National Institute of Virology	Influenza Group, National Institute of Virology	Choudhary,M.L., Anand,S.P., Wadhwa,B.S. and Chadha,M.S.
EPI_ISL_2593792, EPI_ISL_2593793	Department of Epidemiology and Demography, Kenya Medical Research Institute (KEMRI) - Wellcome Trust Research Programme	Department of Epidemiology and Demography, Kenya Medical Research Institute (KEMRI) - Wellcome Trust Research Programme	Otieno,J.R., Agoti,C.N., Gitahi,C.W., Bett,A., Ngama,M., Medley,G.F., Cane,P.A. and Nokes,J.D.
EPI_ISL_2593794, EPI_ISL_2593795	Department of Respiratory Medicine, Children's Hospital, Chongqing Medical University	Department of Respiratory Medicine, Children's Hospital, Chongqing Medical University	Ren,L. and Liu,E.
EPI_ISL_2593796	Minoru Nidaira Okinawa Prefectural Institute of Health and Environment, Department of Biological Science	Minoru Nidaira Okinawa Prefectural Institute of Health and Environment, Department of Biological Science	Nidaira,M. and Taira,K.
EPI_ISL_2593797, EPI_ISL_2593798	Influenza Group, National Institute of Virology	Influenza Group, National Institute of Virology	Choudhary,M.L., Anand,S.P., Wadhwa,B.S. and Chadha,M.S.
EPI_ISL_2593799	Department of Epidemiology and Demography, Kenya Medical Research Institute (KEMRI) - Wellcome Trust Research Programme	Department of Epidemiology and Demography, Kenya Medical Research Institute (KEMRI) - Wellcome Trust Research Programme	Otieno,J.R., Agoti,C.N., Gitahi,C.W., Bett,A., Ngama,M., Medley,G.F., Cane,P.A. and Nokes,J.D.
EPI_ISL_2593800	Department of Respiratory Medicine, Children's Hospital of Chongqing Medical	Department of Respiratory Medicine, Children's Hospital of Chongqing Medical	Ren,L. and Liu,E.

	University	University	
EPI_ISL_2593801	Department of Respiratory Medicine, Children's Hospital, Chongqing Medical University	Department of Respiratory Medicine, Children's Hospital, Chongqing Medical University	Ren,L. and Liu,E.
EPI_ISL_2593802	Department of Respiratory Medicine, Children's Hospital of Chongqing Medical University	Department of Respiratory Medicine, Children's Hospital of Chongqing Medical University	Ren,L. and Liu,E.
EPI_ISL_2593803	Minoru Nidaira Okinawa Prefectural Institute of Health and Environment, Department of Biological Science	Minoru Nidaira Okinawa Prefectural Institute of Health and Environment, Department of Biological Science	Nidaira,M. and Taira,K.
EPI_ISL_2593804	Epidemiology and Demography, KEMRI-Wellcome Trust Research Collaborative Programme	Epidemiology and Demography, KEMRI-Wellcome Trust Research Collaborative Programme	Agoti,C.N., Mayieka,L., Otieno,J.R., Ahmed,J.A., Fields,B., Waiboci,L.B., Nyoka,R., Eidex,R., Morano,N., Burton,W., Montgomery,J., Breiman,R. and Nokes,D.J.
EPI_ISL_2593805	Department of Clinical Laboratory, Fujian Provincial Hospital	Department of Clinical Laboratory, Fujian Provincial Hospital	Su,Y., Wu,Y., Tian,R. and Liang,G.
EPI_ISL_2593806, EPI_ISL_2593807	WHO WPRO Measles Regional Reference Lab, Key Laboratory of Medical Virology Ministry of Health, National Institute for Viral Disease Control and Prevention, China Center for Disease Control and Prevention	WHO WPRO Measles Regional Reference Lab, Key Laboratory of Medical Virology Ministry of Health, National Institute for Viral Disease Control and Prevention, China Center for Disease Control and Prevention	Zhang,Y., Song,J. and Xu,W.
EPI_ISL_2593808	Virology, Tropical Medicine Institute Pedro Kouri	Virology, Tropical Medicine Institute Pedro Kouri	Valdes,O., Corso,M., Pinon,A., Acosta,B., Savon,C., Gonzalez,G., Mune,M., Gonzalez,G., Hernandez,B., Echevarria,Y. and Oropesa,S.
EPI_ISL_2593809	Department of Clinical Laboratory, Fujian Provincial Hospital	Department of Clinical Laboratory, Fujian Provincial Hospital	Su,Y., Wu,Y., Tian,R. and Liang,G.
EPI_ISL_2593810	Pathogen Diagnostic Center, Institut Pasteur of Shanghai	Pathogen Diagnostic Center, Institut Pasteur of Shanghai	Liu,J., Mu,Y., Dong,W., Yao,F., Yan,H., Lan,K. and Zhang,C.
EPI_ISL_2593811	Virology, Tropical Medicine Institute Pedro Kouri	Virology, Tropical Medicine Institute Pedro Kouri	Valdes,O., Corso,M., Pinon,A., Acosta,B., Savon,C., Gonzalez,G., Mune,M., Gonzalez,G., Hernandez,B., Echevarria,Y. and Oropesa,S.
EPI_ISL_2593812	WHO WPRO Measles Regional Reference Lab, Key Laboratory of Medical Virology Ministry of Health, National Institute for Viral Disease Control and Prevention, China Center for Disease Control and Prevention	WHO WPRO Measles Regional Reference Lab, Key Laboratory of Medical Virology Ministry of Health, National Institute for Viral Disease Control and Prevention, China Center for Disease Control and Prevention	Zhang,Y., Song,J. and Xu,W.
EPI_ISL_2593813	Department of Respiratory Medicine, Children's Hospital of Chongqing Medical University	Department of Respiratory Medicine, Children's Hospital of Chongqing Medical University	Ren,L. and Liu,E.
EPI_ISL_2593814	Department of Respiratory Medicine, Children's Hospital, Chongqing Medical University	Department of Respiratory Medicine, Children's Hospital, Chongqing Medical University	Ren,L. and Liu,E.
EPI_ISL_2593815, EPI_ISL_2593816	Department of Respiratory Medicine, Children's Hospital of Chongqing Medical University	Department of Respiratory Medicine, Children's Hospital of Chongqing Medical University	Ren,L. and Liu,E.
EPI_ISL_2593817	Division of Immunology, Children's Hospital	Division of Immunology, Children's Hospital	Qin,X., Zhang,C., Zhao,Y. and Zhao,X.
EPI_ISL_2593818, EPI_ISL_2593819	WHO WPRO Measles Regional Reference Lab, Key Laboratory of Medical Virology Ministry of Health, National Institute for Viral Disease Control and Prevention, China Center for Disease Control and Prevention	WHO WPRO Measles Regional Reference Lab, Key Laboratory of Medical Virology Ministry of Health, National Institute for Viral Disease Control and Prevention, China Center for Disease Control and Prevention	Zhang,Y., Song,J. and Xu,W.
EPI_ISL_2593820	Department of Epidemiology and Demography, Kenya Medical Research Institute (KEMRI) - Wellcome Trust Research Programme	Department of Epidemiology and Demography, Kenya Medical Research Institute (KEMRI) - Wellcome Trust Research Programme	Otieno,J.R., Agoti,C.N., Gitahi,C.W., Bett,A., Ngama,M., Medley,G.F., Cane,P.A. and Nokes,J.D.
EPI_ISL_2593821	Department of Infection and Immunity, Luxembourg Institute of Health	Department of Infection and Immunity, Luxembourg Institute of Health	Snoeck,C.J., Ponghsavath,V., Luetteke,N., Buch,S., Sausy,A., Samountry,B., Jutavijittum,P., Weber,B. and Muller,C.P.
EPI_ISL_2593822	Division of Immunology, Children's Hospital	Division of Immunology, Children's Hospital	Qin,X., Zhang,C., Zhao,Y. and Zhao,X.
EPI_ISL_2593823	Department of Epidemiology and Demography, Kenya Medical Research Institute (KEMRI) - Wellcome Trust Research Programme	Department of Epidemiology and Demography, Kenya Medical Research Institute (KEMRI) - Wellcome Trust Research Programme	Otieno,J.R., Agoti,C.N., Gitahi,C.W., Bett,A., Ngama,M., Medley,G.F., Cane,P.A. and Nokes,J.D.
EPI_ISL_2593824	Department of Clinical Laboratory, Fujian Provincial Hospital	Department of Clinical Laboratory, Fujian Provincial Hospital	Su,Y., Wu,Y., Tian,R. and Liang,G.
EPI_ISL_2593825	Department of Epidemiology and Demography, Kenya Medical Research Institute (KEMRI) - Wellcome Trust Research Programme	Department of Epidemiology and Demography, Kenya Medical Research Institute (KEMRI) - Wellcome Trust Research Programme	Otieno,J.R., Agoti,C.N., Gitahi,C.W., Bett,A., Ngama,M., Medley,G.F., Cane,P.A. and Nokes,J.D.
EPI_ISL_2593826	Division of Immunology, Children's Hospital	Division of Immunology, Children's Hospital	Qin,X., Zhang,C., Zhao,Y. and Zhao,X.
EPI_ISL_2593827, EPI_ISL_2593828, EPI_ISL_2593829	Department of Epidemiology and Demography, Kenya Medical Research Institute (KEMRI) - Wellcome Trust Research Programme	Department of Epidemiology and Demography, Kenya Medical Research Institute (KEMRI) - Wellcome Trust Research Programme	Otieno,J.R., Agoti,C.N., Gitahi,C.W., Bett,A., Ngama,M., Medley,G.F., Cane,P.A. and Nokes,J.D.
EPI_ISL_2593830, EPI_ISL_2593831	WHO WPRO Measles Regional Reference Lab, Key Laboratory of Medical Virology Ministry of Health, National Institute for Viral Disease Control and Prevention, China Center for Disease Control and Prevention	WHO WPRO Measles Regional Reference Lab, Key Laboratory of Medical Virology Ministry of Health, National Institute for Viral Disease Control and Prevention, China Center for Disease Control and Prevention	Zhang,Y., Song,J. and Xu,W.
EPI_ISL_2593832	Pediatric Clinic 1, Department of Pathophysiology and Transplantation, University of Milan and Fondazione IRCCS Ca Granda	Pediatric Clinic 1, Department of Pathophysiology and Transplantation, University of Milan and Fondazione IRCCS Ca Granda	Esposito,S., Zampiero,A., Piralla,A. and Principi,N.
EPI_ISL_2593833, EPI_ISL_2593834, EPI_ISL_2593835, EPI_ISL_2593836, EPI_ISL_2593837	Department of Respiratory Medicine, Children's Hospital of Chongqing Medical University	Department of Respiratory Medicine, Children's Hospital of Chongqing Medical University	Ren,L. and Liu,E.
EPI_ISL_2593838	Department of Epidemiology and Demography, Kenya Medical Research Institute (KEMRI) - Wellcome Trust Research Programme	Department of Epidemiology and Demography, Kenya Medical Research Institute (KEMRI) - Wellcome Trust Research Programme	Otieno,J.R., Agoti,C.N., Gitahi,C.W., Bett,A., Ngama,M., Medley,G.F., Cane,P.A. and Nokes,J.D.
EPI_ISL_2593839	Virology, Tropical Medicine Institute Pedro Kouri	Virology, Tropical Medicine Institute Pedro Kouri	Valdes,O., Corso,M., Pinon,A., Acosta,B., Savon,C., Gonzalez,G., Mune,M., Gonzalez,G., Hernandez,B., Echevarria,Y. and Oropesa,S.
EPI_ISL_2593840	WHO WPRO Measles Regional Reference Lab, Key Laboratory of Medical Virology Ministry of Health, National Institute for Viral Disease Control and Prevention, China Center for Disease Control and Prevention	WHO WPRO Measles Regional Reference Lab, Key Laboratory of Medical Virology Ministry of Health, National Institute for Viral Disease Control and Prevention, China Center for Disease Control and Prevention	Zhang,Y., Song,J. and Xu,W.
EPI_ISL_2593841	Virology, School of Public Health, Tehran University of Medical Sciences	Virology, School of Public Health, Tehran University of Medical Sciences	Yavarian,J., Faghihloo,E. and Mokhtari Azad,T.
EPI_ISL_2593842, EPI_ISL_2593843	Department of Epidemiology and Demography, Kenya Medical Research Institute (KEMRI) - Wellcome Trust Research Programme	Department of Epidemiology and Demography, Kenya Medical Research Institute (KEMRI) - Wellcome Trust Research Programme	Otieno,J.R., Agoti,C.N., Gitahi,C.W., Bett,A., Ngama,M., Medley,G.F., Cane,P.A. and Nokes,J.D.
EPI_ISL_2593844	WHO WPRO Measles Regional Reference Lab, Key Laboratory of Medical Virology Ministry of Health, National Institute for Viral Disease Control and Prevention, China Center for Disease Control and Prevention	WHO WPRO Measles Regional Reference Lab, Key Laboratory of Medical Virology Ministry of Health, National Institute for Viral Disease Control and Prevention, China Center for Disease Control and Prevention	Zhang,Y., Song,J. and Xu,W.
EPI_ISL_2593845	Department of Epidemiology and Demography, Kenya Medical Research Institute (KEMRI) - Wellcome Trust Research Programme	Department of Epidemiology and Demography, Kenya Medical Research Institute (KEMRI) - Wellcome Trust Research Programme	Otieno,J.R., Agoti,C.N., Gitahi,C.W., Bett,A., Ngama,M., Medley,G.F., Cane,P.A. and Nokes,J.D.
EPI_ISL_2593846, EPI_ISL_2593847, EPI_ISL_2593848	Virology Division, Public Health Laboratory Services Branch, Centre for Health Protection, Department of Health	Virology Division, Public Health Laboratory Services Branch, Centre for Health Protection, Department of Health	Mak,G.C., Lau,C.S., Chiu,D.M.Y. and Lim,W.
EPI_ISL_2593849	Department of Epidemiology and Demography, Kenya Medical Research Institute (KEMRI) - Wellcome Trust Research Programme	Department of Epidemiology and Demography, Kenya Medical Research Institute (KEMRI) - Wellcome Trust Research Programme	Otieno,J.R., Agoti,C.N., Gitahi,C.W., Bett,A., Ngama,M., Medley,G.F., Cane,P.A. and Nokes,J.D.
EPI_ISL_2593850	Department of Clinical Laboratory, Fujian Provincial Hospital	Department of Clinical Laboratory, Fujian Provincial Hospital	Su,Y., Wu,Y., Tian,R. and Liang,G.
EPI_ISL_2593851, EPI_ISL_2593852	Department of Epidemiology and Demography, Kenya Medical Research Institute (KEMRI) - Wellcome Trust Research Programme	Department of Epidemiology and Demography, Kenya Medical Research Institute (KEMRI) - Wellcome Trust Research Programme	Otieno,J.R., Agoti,C.N., Gitahi,C.W., Bett,A., Ngama,M., Medley,G.F., Cane,P.A. and Nokes,J.D.
EPI_ISL_2593853	WHO WPRO Measles Regional Reference Lab, Key Laboratory of Medical Virology Ministry of Health, National Institute for Viral Disease Control and Prevention, China Center for Disease Control and Prevention	WHO WPRO Measles Regional Reference Lab, Key Laboratory of Medical Virology Ministry of Health, National Institute for Viral Disease Control and Prevention, China Center for Disease Control and Prevention	Zhang,Y., Song,J. and Xu,W.
EPI_ISL_2593854	Department of Epidemiology and Demography, Kenya Medical Research Institute (KEMRI) - Wellcome Trust Research Programme	Department of Epidemiology and Demography, Kenya Medical Research Institute (KEMRI) - Wellcome Trust Research Programme	Otieno,J.R., Agoti,C.N., Gitahi,C.W., Bett,A., Ngama,M., Medley,G.F., Cane,P.A. and Nokes,J.D.
EPI_ISL_2593855	West China School of Public Health, Sichuan University	West China School of Public Health, Sichuan University	Zhang,M.
EPI_ISL_2593856	Virology, School of Public Health, Tehran University of Medical Sciences	Virology, School of Public Health, Tehran University of Medical Sciences	Yavarian,J., Faghihloo,E. and Mokhtari Azad,T.
EPI_ISL_2593857, EPI_ISL_2593858	Department of Epidemiology and Demography, Kenya Medical Research Institute (KEMRI) - Wellcome Trust Research Programme	Department of Epidemiology and Demography, Kenya Medical Research Institute (KEMRI) - Wellcome Trust Research Programme	Otieno,J.R., Agoti,C.N., Gitahi,C.W., Bett,A., Ngama,M., Medley,G.F., Cane,P.A. and Nokes,J.D.
EPI_ISL_2593859	Department of Clinical Laboratory, Fujian Provincial Hospital	Department of Clinical Laboratory, Fujian Provincial Hospital	Su,Y., Wu,Y., Tian,R. and Liang,G.
EPI_ISL_2593860	Virology, School of Public Health, Tehran University of Medical Sciences	Virology, School of Public Health, Tehran University of Medical Sciences	Yavarian,J., Faghihloo,E. and Mokhtari Azad,T.
EPI_ISL_2593861	Division of Public Health Research, Gyeonggi Province institute of Health and Environment	Division of Public Health Research, Gyeonggi Province institute of Health and Environment	Park,E., Park,P., Huh,J., Yun,H., Lee,H., Yoon,M., Lee,S. and Ko,G.
EPI_ISL_2593862	Department of Clinical Laboratory, Fujian Provincial Hospital	Department of Clinical Laboratory, Fujian Provincial Hospital	Su,Y., Wu,Y., Tian,R. and Liang,G.
EPI_ISL_2593863	Virology, School of Public Health, Tehran University of Medical Sciences	Virology, School of Public Health, Tehran University of Medical Sciences	Yavarian,J., Faghihloo,E. and Mokhtari Azad,T.
EPI_ISL_2593864	Department of Epidemiology and Demography, Kenya Medical Research Institute (KEMRI) - Wellcome Trust Research Programme	Department of Epidemiology and Demography, Kenya Medical Research Institute (KEMRI) - Wellcome Trust Research Programme	Otieno,J.R., Agoti,C.N., Gitahi,C.W., Bett,A., Ngama,M., Medley,G.F., Cane,P.A. and Nokes,J.D.
EPI_ISL_2593865	Department of Protein Engineering, Latvian Biomedical Research and Study Centre	Department of Protein Engineering, Latvian Biomedical Research and Study Centre	Balmaks,R., Ribakova,I., Gardovska,D. and Kazaks,A.

EPI_ISL_2593866	Department of Epidemiology and Demography, Kenya Medical Research Institute (KEMRI) - Wellcome Trust Research Programme	Department of Epidemiology and Demography, Kenya Medical Research Institute (KEMRI) - Wellcome Trust Research Programme	Otieno,J.R., Agoti,C.N., Gitahi,C.W., Bett,A., Ngama,M., Medley,G.F., Cane,P.A. and Nokes,J.D.
EPI_ISL_2593867	Department of Clinical Laboratory, Fujian Provincial Hospital	Department of Clinical Laboratory, Fujian Provincial Hospital	Su,Y., Wu,Y., Tian,R. and Liang,G.
EPI_ISL_2593868	Virology, School of Public Health, Tehran University of Medical Sciences	Virology, School of Public Health, Tehran University of Medical Sciences	Yavarian,J., Faghihloo,E. and Mokhtari Azad,T.
EPI_ISL_2593869	Department of Epidemiology and Demography, Kenya Medical Research Institute (KEMRI) - Wellcome Trust Research Programme	Department of Epidemiology and Demography, Kenya Medical Research Institute (KEMRI) - Wellcome Trust Research Programme	Otieno,J.R., Agoti,C.N., Gitahi,C.W., Bett,A., Ngama,M., Medley,G.F., Cane,P.A. and Nokes,J.D.
EPI_ISL_2593870, EPI_ISL_2593871, EPI_ISL_2593872, EPI_ISL_2593873, EPI_ISL_2593874	Depto Microbiologia, Instituto de Ciencias Biomedicas, Universidade de Sao Paulo	Depto Microbiologia, Instituto de Ciencias Biomedicas, Universidade de Sao Paulo	Moura,F.E.A., Thomazelli,L.M., Candido,E.D.O., Florencio,C.M.G.D., Pereira,S.A.R., Oliveira,F.M.S., Alves,A.A., Ocadaque,C.J. and Durigon,E.L.
EPI_ISL_2593875, EPI_ISL_2593876, EPI_ISL_2593877, EPI_ISL_2593878, EPI_ISL_2593879, EPI_ISL_2593880, EPI_ISL_2593881, EPI_ISL_2593882, EPI_ISL_2593883, EPI_ISL_2593884, EPI_ISL_2593885, EPI_ISL_2593886, EPI_ISL_2593887, EPI_ISL_2593888, EPI_ISL_2593889, EPI_ISL_2593890, EPI_ISL_2593891, EPI_ISL_2593892, EPI_ISL_2593893, EPI_ISL_2593894, EPI_ISL_2593895, EPI_ISL_2593896, EPI_ISL_2593897, EPI_ISL_2593898, EPI_ISL_2593899, EPI_ISL_2593900, EPI_ISL_2593901, EPI_ISL_2593902, EPI_ISL_2593903, EPI_ISL_2593904, EPI_ISL_2593905, EPI_ISL_2593906			
see above	Chinese Academy of Medical Sciences & Peking Union Medical College	Chinese Academy of Medical Sciences & Peking Union Medical College	Jia,B., Xiao,Y., Wang,Y., Chen,L., Zhang,J., Ren,L. and Wang,J.
EPI_ISL_2593907, EPI_ISL_2593908, EPI_ISL_2593909	WHO WPRO Measles Regional Reference Lab, Key Laboratory of Medical Virology Ministry of Health, National Institute for Viral Disease Control and Prevention, China Center for Disease Control and Prevention	WHO WPRO Measles Regional Reference Lab, Key Laboratory of Medical Virology Ministry of Health, National Institute for Viral Disease Control and Prevention, China Center for Disease Control and Prevention	Zhang,Y., Song,J. and Xu,W.
EPI_ISL_2593910, EPI_ISL_2593911, EPI_ISL_2593912, EPI_ISL_2593913, EPI_ISL_2593914	University of Wuerzburg, Institute of Virology and Immunobiology	University of Wuerzburg, Institute of Virology and Immunobiology	Prifert,C., Hofmann,D. and Weissbrich,B.
EPI_ISL_2593915	Virology Department, Hospices Civils de Lyon	Virology Department, Hospices Civils de Lyon	Gaymard,A., Pichon,M. and Morfin,F.
EPI_ISL_2593916, EPI_ISL_2593917, EPI_ISL_2593918, EPI_ISL_2593919, EPI_ISL_2593920, EPI_ISL_2593921, EPI_ISL_2593922, EPI_ISL_2593923, EPI_ISL_2593924, EPI_ISL_2593925, EPI_ISL_2593926, EPI_ISL_2593927, EPI_ISL_2593928, EPI_ISL_2593929, EPI_ISL_2593930, EPI_ISL_2593931			
see above	Chinese Academy of Medical Sciences & Peking Union Medical College	Chinese Academy of Medical Sciences & Peking Union Medical College	Jia,B., Xiao,Y., Wang,Y., Chen,L., Zhang,J., Ren,L. and Wang,J.
EPI_ISL_2593932, EPI_ISL_2593933, EPI_ISL_2593934, EPI_ISL_2593935, EPI_ISL_2593936, EPI_ISL_2593937, EPI_ISL_2593938	WHO WPRO Measles Regional Reference Lab, Key Laboratory of Medical Virology Ministry of Health, National Institute for Viral Disease Control and Prevention, China Center for Disease Control and Prevention	WHO WPRO Measles Regional Reference Lab, Key Laboratory of Medical Virology Ministry of Health, National Institute for Viral Disease Control and Prevention, China Center for Disease Control and Prevention	Zhang,Y., Song,J. and Xu,W.
EPI_ISL_2593939, EPI_ISL_2593940, EPI_ISL_2593941, EPI_ISL_2593942, EPI_ISL_2593943, EPI_ISL_2593944, EPI_ISL_2593945	University of Wuerzburg, Institute of Virology and Immunobiology	University of Wuerzburg, Institute of Virology and Immunobiology	Prifert,C., Hofmann,D. and Weissbrich,B.
EPI_ISL_2593946	Department of Infectious Diseases, National Health Institute Doutor Ricardo Jorge	Department of Infectious Diseases, National Health Institute Doutor Ricardo Jorge	Saez-Lopez,E., Cristovao,P., Costa,I., Pechirra,P., Conde,P. and Guiomar,R.
EPI_ISL_2593947, EPI_ISL_2593948, EPI_ISL_2593949, EPI_ISL_2593950	Chinese Academy of Medical Sciences & Peking Union Medical College	Chinese Academy of Medical Sciences & Peking Union Medical College	Jia,B., Xiao,Y., Wang,Y., Chen,L., Zhang,J., Ren,L. and Wang,J.
EPI_ISL_2593951, EPI_ISL_2593952, EPI_ISL_2593953, EPI_ISL_2593954	University of Wuerzburg, Institute of Virology and Immunobiology	University of Wuerzburg, Institute of Virology and Immunobiology	Prifert,C., Hofmann,D. and Weissbrich,B.
EPI_ISL_2593955, EPI_ISL_2593956, EPI_ISL_2593957, EPI_ISL_2593958, EPI_ISL_2593959, EPI_ISL_2593960, EPI_ISL_2593961, EPI_ISL_2593962, EPI_ISL_2593963, EPI_ISL_2593964, EPI_ISL_2593965, EPI_ISL_2593966, EPI_ISL_2593967, EPI_ISL_2593968			
see above	Virology Department, Hospices Civils de Lyon	Virology Department, Hospices Civils de Lyon	Gaymard,A., Pichon,M. and Morfin,F.
EPI_ISL_2593969	Health Science, Universidade Federal Do Parana	Health Science, Universidade Federal Do Parana	Moreira,F.B., Santos,J.S., Avanzi,V.M., Nogueira,M.B., Vidal,L.R.R. and Raboni,S.M.
EPI_ISL_2593970, EPI_ISL_2593971, EPI_ISL_2593972, EPI_ISL_2593973	Virology Department, Hospices Civils de Lyon	Virology Department, Hospices Civils de Lyon	Gaymard,A., Pichon,M. and Morfin,F.
EPI_ISL_2593974, EPI_ISL_2593975, EPI_ISL_2593976	Health Science, Universidade Federal Do Parana	Health Science, Universidade Federal Do Parana	Moreira,F.B., Santos,J.S., Avanzi,V.M., Nogueira,M.B., Vidal,L.R.R. and Raboni,S.M.
EPI_ISL_2593977, EPI_ISL_2593978, EPI_ISL_2593979, EPI_ISL_2593980, EPI_ISL_2593981, EPI_ISL_2593982, EPI_ISL_2593983	Virology Department, Hospices Civils de Lyon	Virology Department, Hospices Civils de Lyon	Gaymard,A., Pichon,M. and Morfin,F.
EPI_ISL_2593984, EPI_ISL_2593985	Health Science, Universidade Federal Do Parana	Health Science, Universidade Federal Do Parana	Moreira,F.B., Santos,J.S., Avanzi,V.M., Nogueira,M.B., Vidal,L.R.R. and Raboni,S.M.
EPI_ISL_2593986, EPI_ISL_2593987, EPI_ISL_2593988	Virology Department, Hospices Civils de Lyon	Virology Department, Hospices Civils de Lyon	Gaymard,A., Pichon,M. and Morfin,F.
EPI_ISL_2593989, EPI_ISL_2593990	Health Science, Universidade Federal Do Parana	Health Science, Universidade Federal Do Parana	Moreira,F.B., Santos,J.S., Avanzi,V.M., Nogueira,M.B., Vidal,L.R.R. and Raboni,S.M.
EPI_ISL_2593991, EPI_ISL_2593992, EPI_ISL_2593993, EPI_ISL_2593994, EPI_ISL_2593995	Virology Department, Hospices Civils de Lyon	Virology Department, Hospices Civils de Lyon	Gaymard,A., Pichon,M. and Morfin,F.
EPI_ISL_2593996	Health Science, Universidade Federal Do Parana	Health Science, Universidade Federal Do Parana	Moreira,F.B., Santos,J.S., Avanzi,V.M., Nogueira,M.B., Vidal,L.R.R. and Raboni,S.M.
EPI_ISL_2593997, EPI_ISL_2593998, EPI_ISL_2593999, EPI_ISL_2594000, EPI_ISL_2594001, EPI_ISL_2594002, EPI_ISL_2594003, EPI_ISL_2594004, EPI_ISL_2594005, EPI_ISL_2594006	Virology Department, Hospices Civils de Lyon	Virology Department, Hospices Civils de Lyon	Gaymard,A., Pichon,M. and Morfin,F.
EPI_ISL_2594007	Pediatrics, Mackay Memorial Hospital	Pediatrics, Mackay Memorial Hospital	Chi,H., Hsiao,K.-L., Weng,L.-C., Chiu,N.-C., Huang,L.-M., Chiu,Y.-Y., Liu,C.-P. and Liu,H.-F.
EPI_ISL_2594008	Pediatrics, Show-Chwan Memorial Hospital	Pediatrics, Show-Chwan Memorial Hospital	Lee,C.-Y.
EPI_ISL_2594009, EPI_ISL_2594010, EPI_ISL_2594011	Pediatrics, Mackay Memorial Hospital	Pediatrics, Mackay Memorial Hospital	Chi,H., Hsiao,K.-L., Weng,L.-C., Chiu,N.-C., Huang,L.-M., Chiu,Y.-Y., Liu,C.-P. and Liu,H.-F.
EPI_ISL_2594012	Pediatrics, Show-Chwan Memorial Hospital	Pediatrics, Show-Chwan Memorial Hospital	Lee,C.-Y.
EPI_ISL_2594013, EPI_ISL_2594014, EPI_ISL_2594015, EPI_ISL_2594016, EPI_ISL_2594017, EPI_ISL_2594018, EPI_ISL_2594019, EPI_ISL_2594020, EPI_ISL_2594021, EPI_ISL_2594022, EPI_ISL_2594023			
see above	Pediatrics, Mackay Memorial Hospital	Pediatrics, Mackay Memorial Hospital	Chi,H., Hsiao,K.-L., Weng,L.-C., Chiu,N.-C., Huang,L.-M., Chiu,Y.-Y., Liu,C.-P. and Liu,H.-F.
EPI_ISL_2594024	Pediatrics, Show-Chwan Memorial Hospital	Pediatrics, Show-Chwan Memorial Hospital	Lee,C.-Y.
EPI_ISL_2594025	Laboratory of Virology, Capital Institute of Pediatrics	Laboratory of Virology, Capital Institute of Pediatrics	Cui,G., Deng,J., Zhu,R., Qian,Y., Sun,Y., Zhao,L. and Wang,F.
EPI_ISL_2594026, EPI_ISL_2594027, EPI_ISL_2594028, EPI_ISL_2594029	Pediatrics, Mackay Memorial Hospital	Pediatrics, Mackay Memorial Hospital	Chi,H., Hsiao,K.-L., Weng,L.-C., Chiu,N.-C., Huang,L.-M., Chiu,Y.-Y., Liu,C.-P. and Liu,H.-F.
EPI_ISL_2594030, EPI_ISL_2594031, EPI_ISL_2594032, EPI_ISL_2594033	Pediatrics, Show-Chwan Memorial Hospital	Pediatrics, Show-Chwan Memorial Hospital	Lee,C.-Y.
EPI_ISL_2594034	Department of Biomedical Sciences for Health, University of Milan	Department of Biomedical Sciences for Health, University of Milan	Martinelli,M., Frati,E.R., Zappa,A., Ebranati,E., Bianchi,S., Pariani,E., Amendola,A., Zehender,G. and Tanzi,E.
EPI_ISL_2594035	Pediatrics, Mackay Memorial Hospital	Pediatrics, Mackay Memorial Hospital	Chi,H., Hsiao,K.-L., Weng,L.-C., Chiu,N.-C., Huang,L.-M., Chiu,Y.-Y., Liu,C.-P. and Liu,H.-F.
EPI_ISL_2594036	Pediatrics, Show-Chwan Memorial Hospital	Pediatrics, Show-Chwan Memorial Hospital	Lee,C.-Y.
EPI_ISL_2594037	Depto Microbiologia, Instituto de Ciencias Biomedicas, Universidade de Sao Paulo	Depto Microbiologia, Instituto de Ciencias Biomedicas, Universidade de Sao Paulo	Moura,F.E.A., Thomazelli,L.M., Candido,E.D.O., Florencio,C.M.G.D., Pereira,S.A.R., Oliveira,F.M.S., Alves,A.A., Ocadaque,C.J. and Durigon,E.L.
EPI_ISL_2594038	Department of Biomedical Sciences for Health, University of Milan	Department of Biomedical Sciences for Health, University of Milan	Martinelli,M., Frati,E.R., Zappa,A., Ebranati,E., Bianchi,S., Pariani,E., Amendola,A., Zehender,G. and Tanzi,E.
EPI_ISL_2594039	Laboratory of Virology, Capital Institute of Pediatrics	Laboratory of Virology, Capital Institute of Pediatrics	Cui,G., Deng,J., Zhu,R., Qian,Y., Sun,Y., Zhao,L. and Wang,F.
EPI_ISL_2594040	Chinese Academy of Medical Sciences & Peking Union Medical College	Chinese Academy of Medical Sciences & Peking Union Medical College	Jia,B., Xiao,Y., Wang,Y., Chen,L., Zhang,J., Ren,L. and Wang,J.
EPI_ISL_2594041, EPI_ISL_2594042	Laboratory of Virology, Capital Institute of Pediatrics	Laboratory of Virology, Capital Institute of Pediatrics	Cui,G., Deng,J., Zhu,R., Qian,Y., Sun,Y., Zhao,L. and Wang,F.
EPI_ISL_2594043, EPI_ISL_2594044, EPI_ISL_2594045, EPI_ISL_2594046, EPI_ISL_2594047, EPI_ISL_2594048, EPI_ISL_2594049, EPI_ISL_2594050, EPI_ISL_2594051, EPI_ISL_2594052, EPI_ISL_2594053			
see above	Chinese Academy of Medical Sciences & Peking Union Medical College	Chinese Academy of Medical Sciences & Peking Union Medical College	Jia,B., Xiao,Y., Wang,Y., Chen,L., Zhang,J., Ren,L. and Wang,J.
EPI_ISL_2594054, EPI_ISL_2594055, EPI_ISL_2594056, EPI_ISL_2594057, EPI_ISL_2594058, EPI_ISL_2594059, EPI_ISL_2594060, EPI_ISL_2594061, EPI_ISL_2594062, EPI_ISL_2594063, EPI_ISL_2594064			
see above	Laboratory of Virology, Capital Institute of Pediatrics	Laboratory of Virology, Capital Institute of Pediatrics	Cui,G., Deng,J., Zhu,R., Qian,Y., Sun,Y., Zhao,L. and Wang,F.
EPI_ISL_2594065, EPI_ISL_2594066, EPI_ISL_2594067, EPI_ISL_2594068, EPI_ISL_2594069, EPI_ISL_2594070, EPI_ISL_2594071	Chinese Academy of Medical Sciences & Peking Union Medical College	Chinese Academy of Medical Sciences & Peking Union Medical College	Jia,B., Xiao,Y., Wang,Y., Chen,L., Zhang,J., Ren,L. and Wang,J.
EPI_ISL_2594072, EPI_ISL_2594073, EPI_ISL_2594074, EPI_ISL_2594075, EPI_ISL_2594076, EPI_ISL_2594077, EPI_ISL_2594078	Laboratory of Virology, Capital Institute of Pediatrics	Laboratory of Virology, Capital Institute of Pediatrics	Cui,G., Deng,J., Zhu,R., Qian,Y., Sun,Y., Zhao,L. and Wang,F.
EPI_ISL_2594079, EPI_ISL_2594080, EPI_ISL_2594081, EPI_ISL_2594082, EPI_ISL_2594083, EPI_ISL_2594084, EPI_ISL_2594085, EPI_ISL_2594086, EPI_ISL_2594087, EPI_ISL_2594088, EPI_ISL_2594089, EPI_ISL_2594090			
see above	Chinese Academy of Medical Sciences & Peking Union Medical College	Chinese Academy of Medical Sciences & Peking Union Medical College	Jia,B., Xiao,Y., Wang,Y., Chen,L., Zhang,J., Ren,L. and Wang,J.
EPI_ISL_2594091, EPI_ISL_2594092, EPI_ISL_2594093, EPI_ISL_2594094, EPI_ISL_2594095, EPI_ISL_2594096, EPI_ISL_2594097	Laboratory of Virology, Capital Institute of Pediatrics	Laboratory of Virology, Capital Institute of Pediatrics	Cui,G., Deng,J., Zhu,R., Qian,Y., Sun,Y., Zhao,L. and Wang,F.
EPI_ISL_2594098, EPI_ISL_2594099, EPI_ISL_2594100, EPI_ISL_2594101, EPI_ISL_2594102, EPI_ISL_2594103, EPI_ISL_2594105, EPI_ISL_2594106	Chinese Academy of Medical Sciences & Peking Union Medical College	Chinese Academy of Medical Sciences & Peking Union Medical College	Jia,B., Xiao,Y., Wang,Y., Chen,L., Zhang,J., Ren,L. and Wang,J.
EPI_ISL_2594107, EPI_ISL_2594109, EPI_ISL_2594110, EPI_ISL_2594111, EPI_ISL_2594113, EPI_ISL_2594114, EPI_ISL_2594115, EPI_ISL_2594116	Laboratory of Virology, Capital Institute of Pediatrics	Laboratory of Virology, Capital Institute of Pediatrics	Cui,G., Deng,J., Zhu,R., Qian,Y., Sun,Y., Zhao,L. and Wang,F.
EPI_ISL_2594118, EPI_ISL_2594119, EPI_ISL_2594120, EPI_ISL_2594121	Chinese Academy of Medical Sciences & Peking Union Medical College	Chinese Academy of Medical Sciences & Peking Union Medical College	Jia,B., Xiao,Y., Wang,Y., Chen,L., Zhang,J., Ren,L. and Wang,J.
EPI_ISL_2594123, EPI_ISL_2594124, EPI_ISL_2594125	Laboratory of Virology, Capital Institute of Pediatrics	Laboratory of Virology, Capital Institute of Pediatrics	Cui,G., Deng,J., Zhu,R., Qian,Y., Sun,Y., Zhao,L. and Wang,F.
EPI_ISL_2594126, EPI_ISL_2594128	Depto Microbiologia, Instituto de Ciencias Biomedicas, Universidade de Sao Paulo	Depto Microbiologia, Instituto de Ciencias Biomedicas, Universidade de Sao Paulo	Moura,F.E.A., Thomazelli,L.M., Candido,E.D.O., Florencio,C.M.G.D., Pereira,S.A.R., Oliveira,F.M.S., Alves,A.A., Ocadaque,C.J. and Durigon,E.L.
EPI_ISL_2594129	Health Science, Universidade Federal Do Parana	Health Science, Universidade Federal Do Parana	Moreira,F.B., Santos,J.S., Avanzi,V.M., Nogueira,M.B., Vidal,L.R.R. and Raboni,S.M.
EPI_ISL_2594130	Virology Department, Hospices Civils de Lyon	Virology Department, Hospices Civils de Lyon	Gaymard,A., Pichon,M. and Morfin,F.

EPI_ISL_2594131	Department of Respiratory Medicine, Children's Hospital, Chongqing Medical University	Department of Respiratory Medicine, Children's Hospital, Chongqing Medical University	Ren,L. and Liu,E.
EPI_ISL_2594133	Department of Respiratory Medicine, Children's Hospital of Chongqing Medical University	Department of Respiratory Medicine, Children's Hospital of Chongqing Medical University	Ren,L. and Liu,E.
EPI_ISL_2594134	Department of Clinical Laboratory, Fujian Provincial Hospital	Department of Clinical Laboratory, Fujian Provincial Hospital	Su,Y., Wu,Y., Tian,R. and Liang,G.
EPI_ISL_2594135	Virology Division, Public Health Laboratory Services Branch, Centre for Health Protection, Department of Health	Virology Division, Public Health Laboratory Services Branch, Centre for Health Protection, Department of Health	Mak,G.C., Lau,C.S., Chiu,D.M.Y. and Lim,W.
EPI_ISL_2594136, EPI_ISL_2594138	Department of Epidemiology and Demography, Kenya Medical Research Institute (KEMRI) - Wellcome Trust Research Programme	Department of Epidemiology and Demography, Kenya Medical Research Institute (KEMRI) - Wellcome Trust Research Programme	Otieno,J.R., Agoti,C.N., Gitahi,C.W., Bett,A., Ngama,M., Medley,G.F., Cane,P.A. and Nokes,J.D.
EPI_ISL_2594139	WHO WPRO Measles Regional Reference Lab, Key Laboratory of Medical Virology Ministry of Health, National Institute for Viral Disease Control and Prevention, China Center for Disease Control and Prevention	WHO WPRO Measles Regional Reference Lab, Key Laboratory of Medical Virology Ministry of Health, National Institute for Viral Disease Control and Prevention, China Center for Disease Control and Prevention	Zhang,Y., Song,J. and Xu,W.
EPI_ISL_2594140	Pathogen Diagnostic Center, Institut Pasteur of Shanghai	Pathogen Diagnostic Center, Institut Pasteur of Shanghai	Liu,J., Mu,Y., Dong,W., Yao,F., Yan,H., Lan,K. and Zhang,C.
EPI_ISL_2594142	Department of Clinical Laboratory, Fujian Provincial Hospital	Department of Clinical Laboratory, Fujian Provincial Hospital	Su,Y., Wu,Y., Tian,R. and Liang,G.
EPI_ISL_2594143	Department of Epidemiology and Demography, Kenya Medical Research Institute (KEMRI) - Wellcome Trust Research Programme	Department of Epidemiology and Demography, Kenya Medical Research Institute (KEMRI) - Wellcome Trust Research Programme	Otieno,J.R., Agoti,C.N., Gitahi,C.W., Bett,A., Ngama,M., Medley,G.F., Cane,P.A. and Nokes,J.D.
EPI_ISL_2594144	Pathogen Diagnostic Center, Institut Pasteur of Shanghai	Pathogen Diagnostic Center, Institut Pasteur of Shanghai	Liu,J., Mu,Y., Dong,W., Yao,F., Yan,H., Lan,K. and Zhang,C.
EPI_ISL_2594145, EPI_ISL_2594147, EPI_ISL_2594148, EPI_ISL_2594149, EPI_ISL_2594151	Department of Epidemiology and Demography, Kenya Medical Research Institute (KEMRI) - Wellcome Trust Research Programme	Department of Epidemiology and Demography, Kenya Medical Research Institute (KEMRI) - Wellcome Trust Research Programme	Otieno,J.R., Agoti,C.N., Gitahi,C.W., Bett,A., Ngama,M., Medley,G.F., Cane,P.A. and Nokes,J.D.
EPI_ISL_2594152	Department of Protein Engineering, Latvian Biomedical Research and Study Centre	Department of Protein Engineering, Latvian Biomedical Research and Study Centre	Balmaks,R., Ribakova,I., Gardovska,D. and Kazaks,A.
EPI_ISL_2594153	Department of Clinical Laboratory, Fujian Provincial Hospital	Department of Clinical Laboratory, Fujian Provincial Hospital	Su,Y., Wu,Y., Tian,R. and Liang,G.
EPI_ISL_2594155, EPI_ISL_2594156, EPI_ISL_2594157, EPI_ISL_2594158, EPI_ISL_2594160, EPI_ISL_2594161	Department of Epidemiology and Demography, Kenya Medical Research Institute (KEMRI) - Wellcome Trust Research Programme	Department of Epidemiology and Demography, Kenya Medical Research Institute (KEMRI) - Wellcome Trust Research Programme	Otieno,J.R., Agoti,C.N., Gitahi,C.W., Bett,A., Ngama,M., Medley,G.F., Cane,P.A. and Nokes,J.D.
EPI_ISL_2594162	Israel National Influenza Center, Central Virology Laboratory	Israel National Influenza Center, Central Virology Laboratory	Hirsh,S., MNorth America / USA,H. and Michal,M.
EPI_ISL_2594164	Department of Epidemiology and Demography, Kenya Medical Research Institute (KEMRI) - Wellcome Trust Research Programme	Department of Epidemiology and Demography, Kenya Medical Research Institute (KEMRI) - Wellcome Trust Research Programme	Otieno,J.R., Agoti,C.N., Gitahi,C.W., Bett,A., Ngama,M., Medley,G.F., Cane,P.A. and Nokes,J.D.
EPI_ISL_2594165	Department of Protein Engineering, Latvian Biomedical Research and Study Centre	Department of Protein Engineering, Latvian Biomedical Research and Study Centre	Balmaks,R., Ribakova,I., Gardovska,D. and Kazaks,A.
EPI_ISL_2594166, EPI_ISL_2594167	Department of Respiratory Medicine, Children's Hospital of Chongqing Medical University	Department of Respiratory Medicine, Children's Hospital of Chongqing Medical University	Ren,L. and Liu,E.
EPI_ISL_2594169, EPI_ISL_2594170, EPI_ISL_2594171, EPI_ISL_2594172	Department of Protein Engineering, Latvian Biomedical Research and Study Centre	Department of Protein Engineering, Latvian Biomedical Research and Study Centre	Balmaks,R., Ribakova,I., Gardovska,D. and Kazaks,A.
EPI_ISL_2594174	Pathogen Diagnostic Center, Institut Pasteur of Shanghai	Pathogen Diagnostic Center, Institut Pasteur of Shanghai	Liu,J., Mu,Y., Dong,W., Yao,F., Yan,H., Lan,K. and Zhang,C.
EPI_ISL_2594175	Virology Division, Public Health Laboratory Services Branch, Centre for Health Protection, Department of Health	Virology Division, Public Health Laboratory Services Branch, Centre for Health Protection, Department of Health	Mak,G.C., Lau,C.S., Chiu,D.M.Y. and Lim,W.
EPI_ISL_2594176	Virology, Tropical Medicine Institute Pedro Kouri	Virology, Tropical Medicine Institute Pedro Kouri	Valdes,O., Corso,M., Pinon,A., Acosta,B., Savon,C., Gonzalez,G., Mune,M., Gonzalez,G., Hernandez,B., Echevarria,Y. and Oropesa,S.
EPI_ISL_2594177	Department of Protein Engineering, Latvian Biomedical Research and Study Centre	Department of Protein Engineering, Latvian Biomedical Research and Study Centre	Balmaks,R., Ribakova,I., Gardovska,D. and Kazaks,A.
EPI_ISL_2594179, EPI_ISL_2594180, EPI_ISL_2594181, EPI_ISL_2594183	Department of Epidemiology and Demography, Kenya Medical Research Institute (KEMRI) - Wellcome Trust Research Programme	Department of Epidemiology and Demography, Kenya Medical Research Institute (KEMRI) - Wellcome Trust Research Programme	Otieno,J.R., Agoti,C.N., Gitahi,C.W., Bett,A., Ngama,M., Medley,G.F., Cane,P.A. and Nokes,J.D.
EPI_ISL_2594184, EPI_ISL_2594185	Department of Protein Engineering, Latvian Biomedical Research and Study Centre	Department of Protein Engineering, Latvian Biomedical Research and Study Centre	Balmaks,R., Ribakova,I., Gardovska,D. and Kazaks,A.
EPI_ISL_2594186, EPI_ISL_2594188, EPI_ISL_2594189	Department of Epidemiology and Demography, Kenya Medical Research Institute (KEMRI) - Wellcome Trust Research Programme	Department of Epidemiology and Demography, Kenya Medical Research Institute (KEMRI) - Wellcome Trust Research Programme	Otieno,J.R., Agoti,C.N., Gitahi,C.W., Bett,A., Ngama,M., Medley,G.F., Cane,P.A. and Nokes,J.D.
EPI_ISL_2594190	Department of Protein Engineering, Latvian Biomedical Research and Study Centre	Department of Protein Engineering, Latvian Biomedical Research and Study Centre	Balmaks,R., Ribakova,I., Gardovska,D. and Kazaks,A.
EPI_ISL_2594191	Department of Epidemiology and Demography, Kenya Medical Research Institute (KEMRI) - Wellcome Trust Research Programme	Department of Epidemiology and Demography, Kenya Medical Research Institute (KEMRI) - Wellcome Trust Research Programme	Otieno,J.R., Agoti,C.N., Gitahi,C.W., Bett,A., Ngama,M., Medley,G.F., Cane,P.A. and Nokes,J.D.
EPI_ISL_2594193	Department of Protein Engineering, Latvian Biomedical Research and Study Centre	Department of Protein Engineering, Latvian Biomedical Research and Study Centre	Balmaks,R., Ribakova,I., Gardovska,D. and Kazaks,A.
EPI_ISL_2594194, EPI_ISL_2594195	Department of Epidemiology and Demography, Kenya Medical Research Institute (KEMRI) - Wellcome Trust Research Programme	Department of Epidemiology and Demography, Kenya Medical Research Institute (KEMRI) - Wellcome Trust Research Programme	Otieno,J.R., Agoti,C.N., Gitahi,C.W., Bett,A., Ngama,M., Medley,G.F., Cane,P.A. and Nokes,J.D.
EPI_ISL_2594196, EPI_ISL_2594197, EPI_ISL_2594199	Department of Respiratory Medicine, Children's Hospital of Chongqing Medical University	Department of Respiratory Medicine, Children's Hospital of Chongqing Medical University	Ren,L. and Liu,E.
EPI_ISL_2594200	Virology, School of Public Health, Tehran University of Medical Sciences	Virology, School of Public Health, Tehran University of Medical Sciences	Yavarian,J., Faghihloo,E. and Mokhtari Azad,T.
EPI_ISL_2594201	Epidemiology and Demography, KEMRI-Wellcome Trust Research Collaborative Programme	Epidemiology and Demography, KEMRI-Wellcome Trust Research Collaborative Programme	Agoti,C.N., Mayieka,L., Otieno,J.R., Ahmed,J.A., Fields,B., Waiboci,L.B., Nyoka,R., Eidex,R., Morano,N., Burton,W., Montgomery,J., Breiman,R. and Nokes,D.J.
EPI_ISL_2594202	Virology, School of Public Health, Tehran University of Medical Sciences	Virology, School of Public Health, Tehran University of Medical Sciences	Yavarian,J., Faghihloo,E. and Mokhtari Azad,T.
EPI_ISL_2594204, EPI_ISL_2594205, EPI_ISL_2594207, EPI_ISL_2594208	Department of Epidemiology and Demography, Kenya Medical Research Institute (KEMRI) - Wellcome Trust Research Programme	Department of Epidemiology and Demography, Kenya Medical Research Institute (KEMRI) - Wellcome Trust Research Programme	Otieno,J.R., Agoti,C.N., Gitahi,C.W., Bett,A., Ngama,M., Medley,G.F., Cane,P.A. and Nokes,J.D.
EPI_ISL_2594209	Epidemiology and Demography, KEMRI-Wellcome Trust Research Collaborative Programme	Epidemiology and Demography, KEMRI-Wellcome Trust Research Collaborative Programme	Agoti,C.N., Mayieka,L., Otieno,J.R., Ahmed,J.A., Fields,B., Waiboci,L.B., Nyoka,R., Eidex,R., Morano,N., Burton,W., Montgomery,J., Breiman,R. and Nokes,D.J.
EPI_ISL_2594210, EPI_ISL_2594212, EPI_ISL_2594213, EPI_ISL_2594214	Department of Epidemiology and Demography, Kenya Medical Research Institute (KEMRI) - Wellcome Trust Research Programme	Department of Epidemiology and Demography, Kenya Medical Research Institute (KEMRI) - Wellcome Trust Research Programme	Otieno,J.R., Agoti,C.N., Gitahi,C.W., Bett,A., Ngama,M., Medley,G.F., Cane,P.A. and Nokes,J.D.
EPI_ISL_2594216	Epidemiology and Demography, KEMRI-Wellcome Trust Research Collaborative Programme	Epidemiology and Demography, KEMRI-Wellcome Trust Research Collaborative Programme	Agoti,C.N., Mayieka,L., Otieno,J.R., Ahmed,J.A., Fields,B., Waiboci,L.B., Nyoka,R., Eidex,R., Morano,N., Burton,W., Montgomery,J., Breiman,R. and Nokes,D.J.
EPI_ISL_2594217, EPI_ISL_2594218	Department of Epidemiology and Demography, Kenya Medical Research Institute (KEMRI) - Wellcome Trust Research Programme	Department of Epidemiology and Demography, Kenya Medical Research Institute (KEMRI) - Wellcome Trust Research Programme	Otieno,J.R., Agoti,C.N., Gitahi,C.W., Bett,A., Ngama,M., Medley,G.F., Cane,P.A. and Nokes,J.D.
EPI_ISL_2594219	Department of Clinical Laboratory, Fujian Provincial Hospital	Department of Clinical Laboratory, Fujian Provincial Hospital	Su,Y., Wu,Y., Tian,R. and Liang,G.
EPI_ISL_2594221, EPI_ISL_2594222	Department of Epidemiology and Demography, Kenya Medical Research Institute (KEMRI) - Wellcome Trust Research Programme	Department of Epidemiology and Demography, Kenya Medical Research Institute (KEMRI) - Wellcome Trust Research Programme	Otieno,J.R., Agoti,C.N., Gitahi,C.W., Bett,A., Ngama,M., Medley,G.F., Cane,P.A. and Nokes,J.D.
EPI_ISL_2594223	Department of Protein Engineering, Latvian Biomedical Research and Study Centre	Department of Protein Engineering, Latvian Biomedical Research and Study Centre	Balmaks,R., Ribakova,I., Gardovska,D. and Kazaks,A.
EPI_ISL_2594225, EPI_ISL_2594226, EPI_ISL_2594227, EPI_ISL_2594229, EPI_ISL_2594230, EPI_ISL_2594231, EPI_ISL_2594232, EPI_ISL_2594234	Department of Epidemiology and Demography, Kenya Medical Research Institute (KEMRI) - Wellcome Trust Research Programme	Department of Epidemiology and Demography, Kenya Medical Research Institute (KEMRI) - Wellcome Trust Research Programme	Otieno,J.R., Agoti,C.N., Gitahi,C.W., Bett,A., Ngama,M., Medley,G.F., Cane,P.A. and Nokes,J.D.
EPI_ISL_2594235	Department of Protein Engineering, Latvian Biomedical Research and Study Centre	Department of Protein Engineering, Latvian Biomedical Research and Study Centre	Balmaks,R., Ribakova,I., Gardovska,D. and Kazaks,A.
EPI_ISL_2594236, EPI_ISL_2594238	Department of Epidemiology and Demography, Kenya Medical Research Institute (KEMRI) - Wellcome Trust Research Programme	Department of Epidemiology and Demography, Kenya Medical Research Institute (KEMRI) - Wellcome Trust Research Programme	Otieno,J.R., Agoti,C.N., Gitahi,C.W., Bett,A., Ngama,M., Medley,G.F., Cane,P.A. and Nokes,J.D.
EPI_ISL_2594239	Pathogen Diagnostic Center, Institut Pasteur of Shanghai	Pathogen Diagnostic Center, Institut Pasteur of Shanghai	Liu,J., Mu,Y., Dong,W., Yao,F., Yan,H., Lan,K. and Zhang,C.
EPI_ISL_2594240	Department of Epidemiology and Demography, Kenya Medical Research Institute (KEMRI) - Wellcome Trust Research Programme	Department of Epidemiology and Demography, Kenya Medical Research Institute (KEMRI) - Wellcome Trust Research Programme	Otieno,J.R., Agoti,C.N., Gitahi,C.W., Bett,A., Ngama,M., Medley,G.F., Cane,P.A. and Nokes,J.D.
EPI_ISL_2594242	Virology Division, Public Health Laboratory Services Branch, Centre for Health Protection, Department of Health	Virology Division, Public Health Laboratory Services Branch, Centre for Health Protection, Department of Health	Mak,G.C., Lau,C.S., Chiu,D.M.Y. and Lim,W.
EPI_ISL_2594243, EPI_ISL_2594244, EPI_ISL_2594245, EPI_ISL_2594246, EPI_ISL_2594248, EPI_ISL_2594249, EPI_ISL_2594250	Department of Epidemiology and Demography, Kenya Medical Research Institute (KEMRI) - Wellcome Trust Research Programme	Department of Epidemiology and Demography, Kenya Medical Research Institute (KEMRI) - Wellcome Trust Research Programme	Otieno,J.R., Agoti,C.N., Gitahi,C.W., Bett,A., Ngama,M., Medley,G.F., Cane,P.A. and Nokes,J.D.
EPI_ISL_2594251	Department of Protein Engineering, Latvian Biomedical Research and Study Centre	Department of Protein Engineering, Latvian Biomedical Research and Study Centre	Balmaks,R., Ribakova,I., Gardovska,D. and Kazaks,A.
EPI_ISL_2594253, EPI_ISL_2594254, EPI_ISL_2594255, EPI_ISL_2594256	Department of Epidemiology and Demography, Kenya Medical Research Institute (KEMRI) - Wellcome Trust Research Programme	Department of Epidemiology and Demography, Kenya Medical Research Institute (KEMRI) - Wellcome Trust Research Programme	Otieno,J.R., Agoti,C.N., Gitahi,C.W., Bett,A., Ngama,M., Medley,G.F., Cane,P.A. and Nokes,J.D.
EPI_ISL_2594258	Virology, Tropical Medicine Institute Pedro Kouri	Virology, Tropical Medicine Institute Pedro Kouri	Valdes,O., Corso,M., Pinon,A., Acosta,B., Savon,C., Gonzalez,G., Mune,M., Gonzalez,G., Hernandez,B., Echevarria,Y. and Oropesa,S.
EPI_ISL_2594259, EPI_ISL_2594260, EPI_ISL_2594261	Department of Epidemiology and Demography, Kenya Medical Research Institute (KEMRI) - Wellcome Trust Research Programme	Department of Epidemiology and Demography, Kenya Medical Research Institute (KEMRI) - Wellcome Trust Research Programme	Otieno,J.R., Agoti,C.N., Gitahi,C.W., Bett,A., Ngama,M., Medley,G.F., Cane,P.A. and Nokes,J.D.
EPI_ISL_2594263	Department of Clinical Laboratory, Fujian Provincial Hospital	Department of Clinical Laboratory, Fujian Provincial Hospital	Su,Y., Wu,Y., Tian,R. and Liang,G.
EPI_ISL_2594264, EPI_ISL_2594265, EPI_ISL_2594266	Department of Epidemiology and Demography, Kenya Medical Research Institute	Department of Epidemiology and Demography, Kenya Medical Research Institute	Otieno,J.R., Agoti,C.N., Gitahi,C.W., Bett,A., Ngama,M., Medley,G.F., Cane,P.A. and Nokes,J.D.

	(KEMRI) - Wellcome Trust Research Programme	(KEMRI) - Wellcome Trust Research Programme	
EPI_ISL_2594268, EPI_ISL_2594269	Department of Respiratory Medicine, Children's Hospital of Chongqing Medical University	Department of Respiratory Medicine, Children's Hospital of Chongqing Medical University	Ren,L. and Liu,E.
EPI_ISL_2594270, EPI_ISL_2594271	Department of Respiratory Medicine, Children's Hospital, Chongqing Medical University	Department of Respiratory Medicine, Children's Hospital, Chongqing Medical University	Ren,L. and Liu,E.
EPI_ISL_2594273	Department of Respiratory Medicine, Children's Hospital of Chongqing Medical University	Department of Respiratory Medicine, Children's Hospital of Chongqing Medical University	Ren,L. and Liu,E.
EPI_ISL_2594274, EPI_ISL_2594275, EPI_ISL_2594277, EPI_ISL_2594278, EPI_ISL_2594279, EPI_ISL_2594280, EPI_ISL_2594282, EPI_ISL_2594283, EPI_ISL_2594284, EPI_ISL_2594285	Department of Epidemiology and Demography, Kenya Medical Research Institute (KEMRI) - Wellcome Trust Research Programme	Department of Epidemiology and Demography, Kenya Medical Research Institute (KEMRI) - Wellcome Trust Research Programme	Otieno,J.R., Agoti,C.N., Gitahi,C.W., Bett,A., Ngama,M., Medley,G.F., Cane,P.A. and Nokes,J.D.
EPI_ISL_2594287	WHO WPRO Measles Regional Reference Lab, Key Laboratory of Medical Virology Ministry of Health, National Institute for Viral Disease Control and Prevention, China Center for Disease Control and Prevention	WHO WPRO Measles Regional Reference Lab, Key Laboratory of Medical Virology Ministry of Health, National Institute for Viral Disease Control and Prevention, China Center for Disease Control and Prevention	Zhang,Y., Song,J. and Xu,W.
EPI_ISL_2594288, EPI_ISL_2594289, EPI_ISL_2594290, EPI_ISL_2594292	Department of Epidemiology and Demography, Kenya Medical Research Institute (KEMRI) - Wellcome Trust Research Programme	Department of Epidemiology and Demography, Kenya Medical Research Institute (KEMRI) - Wellcome Trust Research Programme	Otieno,J.R., Agoti,C.N., Gitahi,C.W., Bett,A., Ngama,M., Medley,G.F., Cane,P.A. and Nokes,J.D.
EPI_ISL_2594293	Department of Protein Engineering, Latvian Biomedical Research and Study Centre	Department of Protein Engineering, Latvian Biomedical Research and Study Centre	Balmaks,R., Ribakova,I., Gardovska,D. and Kazaks,A.
EPI_ISL_2594294, EPI_ISL_2594295, EPI_ISL_2594297, EPI_ISL_2594298, EPI_ISL_2594299, EPI_ISL_2594300, EPI_ISL_2594302, EPI_ISL_2594303, EPI_ISL_2594304, EPI_ISL_2594306	Department of Epidemiology and Demography, Kenya Medical Research Institute (KEMRI) - Wellcome Trust Research Programme	Department of Epidemiology and Demography, Kenya Medical Research Institute (KEMRI) - Wellcome Trust Research Programme	Otieno,J.R., Agoti,C.N., Gitahi,C.W., Bett,A., Ngama,M., Medley,G.F., Cane,P.A. and Nokes,J.D.
EPI_ISL_2594307	WHO WPRO Measles Regional Reference Lab, Key Laboratory of Medical Virology Ministry of Health, National Institute for Viral Disease Control and Prevention, China Center for Disease Control and Prevention	WHO WPRO Measles Regional Reference Lab, Key Laboratory of Medical Virology Ministry of Health, National Institute for Viral Disease Control and Prevention, China Center for Disease Control and Prevention	Zhang,Y., Song,J. and Xu,W.
EPI_ISL_2594308, EPI_ISL_2594309, EPI_ISL_2594311, EPI_ISL_2594312, EPI_ISL_2594313, EPI_ISL_2594314, EPI_ISL_2594316, EPI_ISL_2594317	Department of Epidemiology and Demography, Kenya Medical Research Institute (KEMRI) - Wellcome Trust Research Programme	Department of Epidemiology and Demography, Kenya Medical Research Institute (KEMRI) - Wellcome Trust Research Programme	Otieno,J.R., Agoti,C.N., Gitahi,C.W., Bett,A., Ngama,M., Medley,G.F., Cane,P.A. and Nokes,J.D.
EPI_ISL_2594318	Epidemiology and Demography, KEMRI-Wellcome Trust Research Collaborative Programme	Epidemiology and Demography, KEMRI-Wellcome Trust Research Collaborative Programme	Agoti,C.N., Mayieka,L., Otieno,J.R., Ahmed,J.A., Fields,B., Waiboci,L.B., Nyoka,R., Eidex,R., Morano,N., Burton,W., Montgomery,J., Breiman,R. and Nokes,D.J.
EPI_ISL_2594320, EPI_ISL_2594321, EPI_ISL_2594322	Department of Epidemiology and Demography, Kenya Medical Research Institute (KEMRI) - Wellcome Trust Research Programme	Department of Epidemiology and Demography, Kenya Medical Research Institute (KEMRI) - Wellcome Trust Research Programme	Otieno,J.R., Agoti,C.N., Gitahi,C.W., Bett,A., Ngama,M., Medley,G.F., Cane,P.A. and Nokes,J.D.
EPI_ISL_2594323	Department of Respiratory Medicine, Children's Hospital of Chongqing Medical University	Department of Respiratory Medicine, Children's Hospital of Chongqing Medical University	Ren,L. and Liu,E.
EPI_ISL_2594325	Department of Respiratory Medicine, Children's Hospital, Chongqing Medical University	Department of Respiratory Medicine, Children's Hospital, Chongqing Medical University	Ren,L. and Liu,E.
EPI_ISL_2594326	Department of Respiratory Medicine, Children's Hospital of Chongqing Medical University	Department of Respiratory Medicine, Children's Hospital of Chongqing Medical University	Ren,L. and Liu,E.
EPI_ISL_2594327, EPI_ISL_2594329	Department of Epidemiology and Demography, Kenya Medical Research Institute (KEMRI) - Wellcome Trust Research Programme	Department of Epidemiology and Demography, Kenya Medical Research Institute (KEMRI) - Wellcome Trust Research Programme	Otieno,J.R., Agoti,C.N., Gitahi,C.W., Bett,A., Ngama,M., Medley,G.F., Cane,P.A. and Nokes,J.D.
EPI_ISL_2594330	Virology Division, Public Health Laboratory Services Branch, Centre for Health Protection, Department of Health	Virology Division, Public Health Laboratory Services Branch, Centre for Health Protection, Department of Health	Mak,G.C., Lau,C.S., Chiu,D.M.Y. and Lim,W.
EPI_ISL_2594331	Department of Epidemiology and Demography, Kenya Medical Research Institute (KEMRI) - Wellcome Trust Research Programme	Department of Epidemiology and Demography, Kenya Medical Research Institute (KEMRI) - Wellcome Trust Research Programme	Otieno,J.R., Agoti,C.N., Gitahi,C.W., Bett,A., Ngama,M., Medley,G.F., Cane,P.A. and Nokes,J.D.
EPI_ISL_2594332	Virology Division, Public Health Laboratory Services Branch, Centre for Health Protection, Department of Health	Virology Division, Public Health Laboratory Services Branch, Centre for Health Protection, Department of Health	Mak,G.C., Lau,C.S., Chiu,D.M.Y. and Lim,W.
EPI_ISL_2594334, EPI_ISL_2594335, EPI_ISL_2594336, EPI_ISL_2594337, EPI_ISL_2594339	Department of Epidemiology and Demography, Kenya Medical Research Institute (KEMRI) - Wellcome Trust Research Programme	Department of Epidemiology and Demography, Kenya Medical Research Institute (KEMRI) - Wellcome Trust Research Programme	Otieno,J.R., Agoti,C.N., Gitahi,C.W., Bett,A., Ngama,M., Medley,G.F., Cane,P.A. and Nokes,J.D.
EPI_ISL_2594340, EPI_ISL_2594341, EPI_ISL_2594342, EPI_ISL_2594344, EPI_ISL_2594345	Virology Division, Public Health Laboratory Services Branch, Centre for Health Protection, Department of Health	Virology Division, Public Health Laboratory Services Branch, Centre for Health Protection, Department of Health	Mak,G.C., Lau,C.S., Chiu,D.M.Y. and Lim,W.
EPI_ISL_2594346	WHO WPRO Measles Regional Reference Lab, Key Laboratory of Medical Virology Ministry of Health, National Institute for Viral Disease Control and Prevention, China Center for Disease Control and Prevention	WHO WPRO Measles Regional Reference Lab, Key Laboratory of Medical Virology Ministry of Health, National Institute for Viral Disease Control and Prevention, China Center for Disease Control and Prevention	Zhang,Y., Song,J. and Xu,W.
EPI_ISL_2594347	Epidemiology and Demography, KEMRI-Wellcome Trust Research Collaborative Programme	Epidemiology and Demography, KEMRI-Wellcome Trust Research Collaborative Programme	Agoti,C.N., Mayieka,L., Otieno,J.R., Ahmed,J.A., Fields,B., Waiboci,L.B., Nyoka,R., Eidex,R., Morano,N., Burton,W., Montgomery,J., Breiman,R. and Nokes,D.J.
EPI_ISL_2594349, EPI_ISL_2594350, EPI_ISL_2594351, EPI_ISL_2594353, EPI_ISL_2594354, EPI_ISL_2594355, EPI_ISL_2594357, EPI_ISL_2594358, EPI_ISL_2594359, EPI_ISL_2594360, EPI_ISL_2594362, EPI_ISL_2594363			
see above	Department of Epidemiology and Demography, Kenya Medical Research Institute (KEMRI) - Wellcome Trust Research Programme	Department of Epidemiology and Demography, Kenya Medical Research Institute (KEMRI) - Wellcome Trust Research Programme	Otieno,J.R., Agoti,C.N., Gitahi,C.W., Bett,A., Ngama,M., Medley,G.F., Cane,P.A. and Nokes,J.D.
EPI_ISL_2594364	Department of Respiratory Medicine, Children's Hospital of Chongqing Medical University	Department of Respiratory Medicine, Children's Hospital of Chongqing Medical University	Ren,L. and Liu,E.
EPI_ISL_2594365	Minoru Nidaira Okinawa Prefectural Institute of Health and Environment, Department of Biological Science	Minoru Nidaira Okinawa Prefectural Institute of Health and Environment, Department of Biological Science	Nidaira,M. and Taira,K.
EPI_ISL_2594367	Virology Division, Public Health Laboratory Services Branch, Centre for Health Protection, Department of Health	Virology Division, Public Health Laboratory Services Branch, Centre for Health Protection, Department of Health	Mak,G.C., Lau,C.S., Chiu,D.M.Y. and Lim,W.
EPI_ISL_2594368, EPI_ISL_2594369	Department of Epidemiology and Demography, Kenya Medical Research Institute (KEMRI) - Wellcome Trust Research Programme	Department of Epidemiology and Demography, Kenya Medical Research Institute (KEMRI) - Wellcome Trust Research Programme	Otieno,J.R., Agoti,C.N., Gitahi,C.W., Bett,A., Ngama,M., Medley,G.F., Cane,P.A. and Nokes,J.D.
EPI_ISL_2594371	Epidemiology and Demography, KEMRI-Wellcome Trust Research Collaborative Programme	Epidemiology and Demography, KEMRI-Wellcome Trust Research Collaborative Programme	Agoti,C.N., Mayieka,L., Otieno,J.R., Ahmed,J.A., Fields,B., Waiboci,L.B., Nyoka,R., Eidex,R., Morano,N., Burton,W., Montgomery,J., Breiman,R. and Nokes,D.J.
EPI_ISL_2594372	Department of Epidemiology and Demography, Kenya Medical Research Institute (KEMRI) - Wellcome Trust Research Programme	Department of Epidemiology and Demography, Kenya Medical Research Institute (KEMRI) - Wellcome Trust Research Programme	Otieno,J.R., Agoti,C.N., Gitahi,C.W., Bett,A., Ngama,M., Medley,G.F., Cane,P.A. and Nokes,J.D.
EPI_ISL_2594374	Epidemiology and Demography, KEMRI-Wellcome Trust Research Collaborative Programme	Epidemiology and Demography, KEMRI-Wellcome Trust Research Collaborative Programme	Agoti,C.N., Mayieka,L., Otieno,J.R., Ahmed,J.A., Fields,B., Waiboci,L.B., Nyoka,R., Eidex,R., Morano,N., Burton,W., Montgomery,J., Breiman,R. and Nokes,D.J.
EPI_ISL_2594375	Department of Epidemiology and Demography, Kenya Medical Research Institute (KEMRI) - Wellcome Trust Research Programme	Department of Epidemiology and Demography, Kenya Medical Research Institute (KEMRI) - Wellcome Trust Research Programme	Otieno,J.R., Agoti,C.N., Gitahi,C.W., Bett,A., Ngama,M., Medley,G.F., Cane,P.A. and Nokes,J.D.
EPI_ISL_2594376	Virology, Tropical Medicine Institute Pedro Kouri	Virology, Tropical Medicine Institute Pedro Kouri	Valdes,O., Corso,M., Pinon,A., Acosta,B., Savon,C., Gonzalez,G., Mune,M., Gonzalez,G., Hernandez,B., Echevarria,Y. and Oropesa,S.
EPI_ISL_2594377	Department of Epidemiology and Demography, Kenya Medical Research Institute (KEMRI) - Wellcome Trust Research Programme	Department of Epidemiology and Demography, Kenya Medical Research Institute (KEMRI) - Wellcome Trust Research Programme	Otieno,J.R., Agoti,C.N., Gitahi,C.W., Bett,A., Ngama,M., Medley,G.F., Cane,P.A. and Nokes,J.D.
EPI_ISL_2594379	Virology, Tropical Medicine Institute Pedro Kouri	Virology, Tropical Medicine Institute Pedro Kouri	Valdes,O., Corso,M., Pinon,A., Acosta,B., Savon,C., Gonzalez,G., Mune,M., Gonzalez,G., Hernandez,B., Echevarria,Y. and Oropesa,S.
EPI_ISL_2594380, EPI_ISL_2594381	Department of Epidemiology and Demography, Kenya Medical Research Institute (KEMRI) - Wellcome Trust Research Programme	Department of Epidemiology and Demography, Kenya Medical Research Institute (KEMRI) - Wellcome Trust Research Programme	Otieno,J.R., Agoti,C.N., Gitahi,C.W., Bett,A., Ngama,M., Medley,G.F., Cane,P.A. and Nokes,J.D.
EPI_ISL_2594382	Department of Public Health Laboratory Sciences, West China School of Public Health, Sichuan University	Department of Public Health Laboratory Sciences, West China School of Public Health, Sichuan University	Hu,W.P. and Pei,F.X.
EPI_ISL_2594384	Department of Epidemiology and Demography, Kenya Medical Research Institute (KEMRI) - Wellcome Trust Research Programme	Department of Epidemiology and Demography, Kenya Medical Research Institute (KEMRI) - Wellcome Trust Research Programme	Otieno,J.R., Agoti,C.N., Gitahi,C.W., Bett,A., Ngama,M., Medley,G.F., Cane,P.A. and Nokes,J.D.
EPI_ISL_2594385	WHO WPRO Measles Regional Reference Lab, Key Laboratory of Medical Virology Ministry of Health, National Institute for Viral Disease Control and Prevention, China Center for Disease Control and Prevention	WHO WPRO Measles Regional Reference Lab, Key Laboratory of Medical Virology Ministry of Health, National Institute for Viral Disease Control and Prevention, China Center for Disease Control and Prevention	Zhang,Y., Song,J. and Xu,W.
EPI_ISL_2594386, EPI_ISL_2594388	Department of Epidemiology and Demography, Kenya Medical Research Institute (KEMRI) - Wellcome Trust Research Programme	Department of Epidemiology and Demography, Kenya Medical Research Institute (KEMRI) - Wellcome Trust Research Programme	Otieno,J.R., Agoti,C.N., Gitahi,C.W., Bett,A., Ngama,M., Medley,G.F., Cane,P.A. and Nokes,J.D.
EPI_ISL_2594389	WHO WPRO Measles Regional Reference Lab, Key Laboratory of Medical Virology Ministry of Health, National Institute for Viral Disease Control and Prevention, China Center for Disease Control and Prevention	WHO WPRO Measles Regional Reference Lab, Key Laboratory of Medical Virology Ministry of Health, National Institute for Viral Disease Control and Prevention, China Center for Disease Control and Prevention	Zhang,Y., Song,J. and Xu,W.
EPI_ISL_2594390	Department of Respiratory Medicine, Children's Hospital, Chongqing Medical University	Department of Respiratory Medicine, Children's Hospital, Chongqing Medical University	Ren,L. and Liu,E.
EPI_ISL_2594391, EPI_ISL_2594393	Minoru Nidaira Okinawa Prefectural Institute of Health and Environment, Department	Minoru Nidaira Okinawa Prefectural Institute of Health and Environment, Department	Nidaira,M. and Taira,K.

	of Biological Science	of Biological Science	
EPI_ISL_2594394, EPI_ISL_2594395	Department of Epidemiology and Demography, Kenya Medical Research Institute (KEMRI) - Wellcome Trust Research Programme	Department of Epidemiology and Demography, Kenya Medical Research Institute (KEMRI) - Wellcome Trust Research Programme	Otieno,J.R., Agoti,C.N., Gitahi,C.W., Bett,A., Ngama,M., Medley,G.F., Cane,P.A. and Nokes,J.D.
EPI_ISL_2594396	Manipal Centre for Virus Research, Manipal University	Manipal Centre for Virus Research, Manipal University	Anjali,A., Aswathy,R., Akhil,C., Giselle,D., Revti,B., Hindol,M., Suresh,P. and Arunkumar,G.
EPI_ISL_2594397, EPI_ISL_2594399	WHO WPRO Measles Regional Reference Lab, Key Laboratory of Medical Virology Ministry of Health, National Institute for Viral Disease Control and Prevention, China Center for Disease Control and Prevention	WHO WPRO Measles Regional Reference Lab, Key Laboratory of Medical Virology Ministry of Health, National Institute for Viral Disease Control and Prevention, China Center for Disease Control and Prevention	Zhang,Y., Song,J. and Xu,W.
EPI_ISL_2594400, EPI_ISL_2594401	Department of Epidemiology and Demography, Kenya Medical Research Institute (KEMRI) - Wellcome Trust Research Programme	Department of Epidemiology and Demography, Kenya Medical Research Institute (KEMRI) - Wellcome Trust Research Programme	Otieno,J.R., Agoti,C.N., Gitahi,C.W., Bett,A., Ngama,M., Medley,G.F., Cane,P.A. and Nokes,J.D.
EPI_ISL_2594403	Department of Public Health Laboratory Sciences, West China School of Public Health, Sichuan University	Department of Public Health Laboratory Sciences, West China School of Public Health, Sichuan University	Hu,W.P. and Pei,F.X.
EPI_ISL_2594404	Central Laboratory, Guangzhou Women and Children's Medical Center	Central Laboratory, Guangzhou Women and Children's Medical Center	Xie,J.H., Zhu,B., Zhong,J.Y., Chen,Y. and Zhang,Y.Y.
EPI_ISL_2594405	Department of Respiratory Medicine, Children's Hospital of Chongqing Medical University	Department of Respiratory Medicine, Children's Hospital of Chongqing Medical University	Ren,L. and Liu,E.
EPI_ISL_2594406, EPI_ISL_2594408, EPI_ISL_2594409	Department of Paediatric Infectious Diseases, Institute of Tropical Medicine, Nagasaki University	Department of Paediatric Infectious Diseases, Institute of Tropical Medicine, Nagasaki University	Yoshihara,K., Nhat Le,M., Nagasawa,K., Tsukagoshi,H., Nguyen,H.A., Toizumi,M., Moriuchi,H., Hashizume,M., Ariyoshi,K., Dang,D.A., Kimura,H. and Yoshida,L.-M.
EPI_ISL_2594410	Minoru Nidaira Okinawa Prefectural Institute of Health and Environment, Department of Biological Science	Minoru Nidaira Okinawa Prefectural Institute of Health and Environment, Department of Biological Science	Nidaira,M. and Taira,K.
EPI_ISL_2594412	Department of Public Health Laboratory Sciences, West China School of Public Health, Sichuan University	Department of Public Health Laboratory Sciences, West China School of Public Health, Sichuan University	Hu,W.P. and Pei,F.X.
EPI_ISL_2594413	Epidemiology and Demography, KEMRI-Wellcome Trust Research Collaborative Programme	Epidemiology and Demography, KEMRI-Wellcome Trust Research Collaborative Programme	Agoti,C.N., Mayieka,L., Otieno,J.R., Ahmed,J.A., Fields,B., Waiboci,L.B., Nyoka,R., Eidex,R., Morano,N., Burton,W., Montgomery,J., Breiman,R. and Nokes,D.J.
EPI_ISL_2594414	Pathogen Diagnostic Center, Institut Pasteur of Shanghai	Pathogen Diagnostic Center, Institut Pasteur of Shanghai	Liu,J., Mu,Y., Dong,W., Yao,F., Yan,H., Lan,K. and Zhang,C.
EPI_ISL_2594415	Virology, Tropical Medicine Institute Pedro Kouri	Virology, Tropical Medicine Institute Pedro Kouri	Valdes,O., Corso,M., Pinon,A., Acosta,B., Savon,C., Gonzalez,G., Mune,M., Gonzalez,G., Hernandez,B., Echevarria,Y. and Oropesa,S.
EPI_ISL_2594417	WHO WPRO Measles Regional Reference Lab, Key Laboratory of Medical Virology Ministry of Health, National Institute for Viral Disease Control and Prevention, China Center for Disease Control and Prevention	WHO WPRO Measles Regional Reference Lab, Key Laboratory of Medical Virology Ministry of Health, National Institute for Viral Disease Control and Prevention, China Center for Disease Control and Prevention	Zhang,Y., Song,J. and Xu,W.
EPI_ISL_2594418	Virology, Tropical Medicine Institute Pedro Kouri	Virology, Tropical Medicine Institute Pedro Kouri	Valdes,O., Corso,M., Pinon,A., Acosta,B., Savon,C., Gonzalez,G., Mune,M., Gonzalez,G., Hernandez,B., Echevarria,Y. and Oropesa,S.
EPI_ISL_2594419	Epidemiology and Demography, KEMRI - Wellcome Trust Research Programme, Centre for Geographic Medicine Research	Epidemiology and Demography, KEMRI - Wellcome Trust Research Programme, Centre for Geographic Medicine Research	Oketch,J.W., Kamau,E., Otieno,J.R., Mwema,A., Lewa,C., Agoti,C.N. and Nokes,J.D.
EPI_ISL_2594421	Virology, Tropical Medicine Institute Pedro Kouri	Virology, Tropical Medicine Institute Pedro Kouri	Valdes,O., Corso,M., Pinon,A., Acosta,B., Savon,C., Gonzalez,G., Mune,M., Gonzalez,G., Hernandez,B., Echevarria,Y. and Oropesa,S.
EPI_ISL_2594422, EPI_ISL_2594423	Department of Respiratory Medicine, Children's Hospital of Chongqing Medical University	Department of Respiratory Medicine, Children's Hospital of Chongqing Medical University	Ren,L. and Liu,E.
EPI_ISL_2594424	Department of Respiratory Medicine, Children's Hospital, Chongqing Medical University	Department of Respiratory Medicine, Children's Hospital, Chongqing Medical University	Ren,L. and Liu,E.
EPI_ISL_2594426, EPI_ISL_2594427, EPI_ISL_2594428	Department of Paediatric Infectious Diseases, Institute of Tropical Medicine, Nagasaki University	Department of Paediatric Infectious Diseases, Institute of Tropical Medicine, Nagasaki University	Yoshihara,K., Nhat Le,M., Nagasawa,K., Tsukagoshi,H., Nguyen,H.A., Toizumi,M., Moriuchi,H., Hashizume,M., Ariyoshi,K., Dang,D.A., Kimura,H. and Yoshida,L.-M.
EPI_ISL_2594429	Department of Respiratory Medicine, Children's Hospital of Chongqing Medical University	Department of Respiratory Medicine, Children's Hospital of Chongqing Medical University	Ren,L. and Liu,E.
EPI_ISL_2594431, EPI_ISL_2594432, EPI_ISL_2594433, EPI_ISL_2594434, EPI_ISL_2594436, EPI_ISL_2594437, EPI_ISL_2594438, EPI_ISL_2594439	School of Medicine, University of Washington	School of Medicine, University of Washington	Scott,E.M., Magaret,A., Kuypers,J., Stewart,L., Shrestha,L., Tielsch,J.M., Steinhoff,M., Katz,J., Khatry,S.K., LeClerq,S.C., Englund,J.A. and Chu,H.Y.
EPI_ISL_2594441	Pathogen Diagnostic Center, Institut Pasteur of Shanghai	Pathogen Diagnostic Center, Institut Pasteur of Shanghai	Liu,J., Mu,Y., Dong,W., Yao,F., Yan,H., Lan,K. and Zhang,C.
EPI_ISL_2594442	WHO WPRO Measles Regional Reference Lab, Key Laboratory of Medical Virology Ministry of Health, National Institute for Viral Disease Control and Prevention, China Center for Disease Control and Prevention	WHO WPRO Measles Regional Reference Lab, Key Laboratory of Medical Virology Ministry of Health, National Institute for Viral Disease Control and Prevention, China Center for Disease Control and Prevention	Zhang,Y., Song,J. and Xu,W.
EPI_ISL_2594443	Manipal Centre for Virus Research, Manipal University	Manipal Centre for Virus Research, Manipal University	Anjali,A., Aswathy,R., Akhil,C., Giselle,D., Revti,B., Hindol,M., Suresh,P. and Arunkumar,G.
EPI_ISL_2594445	Virology, Tropical Medicine Institute Pedro Kouri	Virology, Tropical Medicine Institute Pedro Kouri	Valdes,O., Corso,M., Pinon,A., Acosta,B., Savon,C., Gonzalez,G., Mune,M., Gonzalez,G., Hernandez,B., Echevarria,Y. and Oropesa,S.
EPI_ISL_2594446	Laboratory Medicine and Pathobiology, University of Toronto	Laboratory Medicine and Pathobiology, University of Toronto	Granados,A., Duvvuri,V., Rosenfeld,P., Eshaghi,A. and Gubbay,J.B.
EPI_ISL_2594447	Epidemiology and Demography, KEMRI - Wellcome Trust Research Programme, Centre for Geographic Medicine Research	Epidemiology and Demography, KEMRI - Wellcome Trust Research Programme, Centre for Geographic Medicine Research	Oketch,J.W., Kamau,E., Otieno,J.R., Mwema,A., Lewa,C., Agoti,C.N. and Nokes,J.D.
EPI_ISL_2594448	Virology, Tropical Medicine Institute Pedro Kouri	Virology, Tropical Medicine Institute Pedro Kouri	Valdes,O., Corso,M., Pinon,A., Acosta,B., Savon,C., Gonzalez,G., Mune,M., Gonzalez,G., Hernandez,B., Echevarria,Y. and Oropesa,S.
EPI_ISL_2594449, EPI_ISL_2594451	Department of Respiratory Medicine, Children's Hospital of Chongqing Medical University	Department of Respiratory Medicine, Children's Hospital of Chongqing Medical University	Ren,L. and Liu,E.
EPI_ISL_2594452	Department of Respiratory Medicine, Children's Hospital, Chongqing Medical University	Department of Respiratory Medicine, Children's Hospital, Chongqing Medical University	Ren,L. and Liu,E.
EPI_ISL_2594453, EPI_ISL_2594454	Department of Respiratory Medicine, Children's Hospital of Chongqing Medical University	Department of Respiratory Medicine, Children's Hospital of Chongqing Medical University	Ren,L. and Liu,E.
EPI_ISL_2594456, EPI_ISL_2594457, EPI_ISL_2594458, EPI_ISL_2594459	School of Medicine, University of Washington	School of Medicine, University of Washington	Scott,E.M., Magaret,A., Kuypers,J., Stewart,L., Shrestha,L., Tielsch,J.M., Steinhoff,M., Katz,J., Khatry,S.K., LeClerq,S.C., Englund,J.A. and Chu,H.Y.
EPI_ISL_2594461	WHO WPRO Measles Regional Reference Lab, Key Laboratory of Medical Virology Ministry of Health, National Institute for Viral Disease Control and Prevention, China Center for Disease Control and Prevention	WHO WPRO Measles Regional Reference Lab, Key Laboratory of Medical Virology Ministry of Health, National Institute for Viral Disease Control and Prevention, China Center for Disease Control and Prevention	Zhang,Y., Song,J. and Xu,W.
EPI_ISL_2594462	Virology, Tropical Medicine Institute Pedro Kouri	Virology, Tropical Medicine Institute Pedro Kouri	Valdes,O., Corso,M., Pinon,A., Acosta,B., Savon,C., Gonzalez,G., Mune,M., Gonzalez,G., Hernandez,B., Echevarria,Y. and Oropesa,S.
EPI_ISL_2594463	Central Laboratory, Guangzhou Women and Children's Medical Center	Central Laboratory, Guangzhou Women and Children's Medical Center	Xie,J.H., Zhu,B., Zhong,J.Y., Chen,Y. and Zhang,Y.Y.
EPI_ISL_2594464	Virology, Tropical Medicine Institute Pedro Kouri	Virology, Tropical Medicine Institute Pedro Kouri	Valdes,O., Corso,M., Pinon,A., Acosta,B., Savon,C., Gonzalez,G., Mune,M., Gonzalez,G., Hernandez,B., Echevarria,Y. and Oropesa,S.
EPI_ISL_2594466	Epidemiology and Demography, KEMRI - Wellcome Trust Research Programme, Centre for Geographic Medicine Research	Epidemiology and Demography, KEMRI - Wellcome Trust Research Programme, Centre for Geographic Medicine Research	Oketch,J.W., Kamau,E., Otieno,J.R., Mwema,A., Lewa,C., Agoti,C.N. and Nokes,J.D.
EPI_ISL_2594467	WHO WPRO Measles Regional Reference Lab, Key Laboratory of Medical Virology Ministry of Health, National Institute for Viral Disease Control and Prevention, China Center for Disease Control and Prevention	WHO WPRO Measles Regional Reference Lab, Key Laboratory of Medical Virology Ministry of Health, National Institute for Viral Disease Control and Prevention, China Center for Disease Control and Prevention	Zhang,Y., Song,J. and Xu,W.
EPI_ISL_2594468	Virology, Tropical Medicine Institute Pedro Kouri	Virology, Tropical Medicine Institute Pedro Kouri	Valdes,O., Corso,M., Pinon,A., Acosta,B., Savon,C., Gonzalez,G., Mune,M., Gonzalez,G., Hernandez,B., Echevarria,Y. and Oropesa,S.
EPI_ISL_2594469	Epidemiology and Demography, KEMRI - Wellcome Trust Research Programme, Centre for Geographic Medicine Research	Epidemiology and Demography, KEMRI - Wellcome Trust Research Programme, Centre for Geographic Medicine Research	Oketch,J.W., Kamau,E., Otieno,J.R., Mwema,A., Lewa,C., Agoti,C.N. and Nokes,J.D.
EPI_ISL_2594471	Pathogen Diagnostic Center, Institut Pasteur of Shanghai	Pathogen Diagnostic Center, Institut Pasteur of Shanghai	Liu,J., Mu,Y., Dong,W., Yao,F., Yan,H., Lan,K. and Zhang,C.
EPI_ISL_2594472, EPI_ISL_2594473	Department of Respiratory Medicine, Children's Hospital of Chongqing Medical University	Department of Respiratory Medicine, Children's Hospital of Chongqing Medical University	Ren,L. and Liu,E.
EPI_ISL_2594474, EPI_ISL_2594476	Department of Respiratory Medicine, Children's Hospital, Chongqing Medical University	Department of Respiratory Medicine, Children's Hospital, Chongqing Medical University	Ren,L. and Liu,E.
EPI_ISL_2594477	Department of Respiratory Medicine, Children's Hospital of Chongqing Medical University	Department of Respiratory Medicine, Children's Hospital of Chongqing Medical University	Ren,L. and Liu,E.
EPI_ISL_2594478, EPI_ISL_2594479, EPI_ISL_2594480, EPI_ISL_2594482, EPI_ISL_2594483, EPI_ISL_2594484	School of Medicine, University of Washington	School of Medicine, University of Washington	Scott,E.M., Magaret,A., Kuypers,J., Stewart,L., Shrestha,L., Tielsch,J.M., Steinhoff,M., Katz,J., Khatry,S.K., LeClerq,S.C., Englund,J.A. and Chu,H.Y.
EPI_ISL_2594485, EPI_ISL_2594487	Epidemiology and Demography, KEMRI - Wellcome Trust Research Programme, Centre for Geographic Medicine Research	Epidemiology and Demography, KEMRI - Wellcome Trust Research Programme, Centre for Geographic Medicine Research	Oketch,J.W., Kamau,E., Otieno,J.R., Mwema,A., Lewa,C., Agoti,C.N. and Nokes,J.D.
EPI_ISL_2594488	Virology, Tropical Medicine Institute Pedro Kouri	Virology, Tropical Medicine Institute Pedro Kouri	Valdes,O., Corso,M., Pinon,A., Acosta,B., Savon,C., Gonzalez,G., Mune,M., Gonzalez,G., Hernandez,B., Echevarria,Y. and Oropesa,S.
EPI_ISL_2594489	Epidemiology and Demography, KEMRI - Wellcome Trust Research Programme, Centre for Geographic Medicine Research	Epidemiology and Demography, KEMRI - Wellcome Trust Research Programme, Centre for Geographic Medicine Research	Oketch,J.W., Kamau,E., Otieno,J.R., Mwema,A., Lewa,C., Agoti,C.N. and Nokes,J.D.
EPI_ISL_2594490	Virology Section, Department of Microbiology, King George's Medical University	Virology Section, Department of Microbiology, King George's Medical University	Singh,A.K., Jain,A., Jain,B., Dangi,T., Verma,A.K., Dwivedi,M. and Kushwaha,R.
EPI_ISL_2594492	University of Wuerzburg, Institute of Virology and Immunobiology	University of Wuerzburg, Institute of Virology and Immunobiology	Prifert,C., Hofmann,D., Schnitzler,P. and Weissbrich,B.

EPI_ISL_2594493	Epidemiology and Demography, KEMRI - Wellcome Trust Research Programme, Centre for Geographic Medicine Research	Epidemiology and Demography, KEMRI - Wellcome Trust Research Programme, Centre for Geographic Medicine Research	Oketch,J.W., Kamau,E., Otieno,J.R., Mwema,A., Lewa,C., Agoti,C.N. and Nokes,J.D.
EPI_ISL_2594494, EPI_ISL_2594495	Department of Public Health Laboratory Sciences, West China School of Public Health, Sichuan University	Department of Public Health Laboratory Sciences, West China School of Public Health, Sichuan University	Hu,W.P. and Pei,F.X.
EPI_ISL_2594497, EPI_ISL_2594498	Pathogen Diagnostic Center, Institut Pasteur of Shanghai	Pathogen Diagnostic Center, Institut Pasteur of Shanghai	Liu,J., Mu,Y., Dong,W., Yao,F., Yan,H., Lan,K. and Zhang,C.
EPI_ISL_2594499	WHO WPRO Measles Regional Reference Lab, Key Laboratory of Medical Virology Ministry of Health, National Institute for Viral Disease Control and Prevention, China Center for Disease Control and Prevention	WHO WPRO Measles Regional Reference Lab, Key Laboratory of Medical Virology Ministry of Health, National Institute for Viral Disease Control and Prevention, China Center for Disease Control and Prevention	Zhang,Y., Song,J. and Xu,W.
EPI_ISL_2594500	Epidemiology and Demography, KEMRI - Wellcome Trust Research Programme, Centre for Geographic Medicine Research	Epidemiology and Demography, KEMRI - Wellcome Trust Research Programme, Centre for Geographic Medicine Research	Oketch,J.W., Kamau,E., Otieno,J.R., Mwema,A., Lewa,C., Agoti,C.N. and Nokes,J.D.
EPI_ISL_2594502	Virology Section, Department of Microbiology, King George's Medical University	Virology Section, Department of Microbiology, King George's Medical University	Singh,A.K., Jain,A., Jain,B., Dangi,T., Verma,A.K., Dwivedi,M. and Kushwaha,R.
EPI_ISL_2594503, EPI_ISL_2594505	Department of Public Health Laboratory Sciences, West China School of Public Health, Sichuan University	Department of Public Health Laboratory Sciences, West China School of Public Health, Sichuan University	Hu,W.P. and Pei,F.X.
EPI_ISL_2594506, EPI_ISL_2594507	WHO WPRO Measles Regional Reference Lab, Key Laboratory of Medical Virology Ministry of Health, National Institute for Viral Disease Control and Prevention, China Center for Disease Control and Prevention	WHO WPRO Measles Regional Reference Lab, Key Laboratory of Medical Virology Ministry of Health, National Institute for Viral Disease Control and Prevention, China Center for Disease Control and Prevention	Zhang,Y., Song,J. and Xu,W.
EPI_ISL_2594508, EPI_ISL_2594510	Virology Section, Department of Microbiology, King George's Medical University	Virology Section, Department of Microbiology, King George's Medical University	Singh,A.K., Jain,A., Jain,B., Dangi,T., Verma,A.K., Dwivedi,M. and Kushwaha,R.
EPI_ISL_2594511	Laboratory Medicine and Pathobiology, University of Toronto	Laboratory Medicine and Pathobiology, University of Toronto	Granados,A., Duvvuri,V., Rosenfeld,P., Eshaghi,A. and Gubbay,J.B.
EPI_ISL_2594512, EPI_ISL_2594514	Pediatric Clinic 1, Department of Pathophysiology and Transplantation, University of Milan and Fondazione IRCCS Ca Granda	Pediatric Clinic 1, Department of Pathophysiology and Transplantation, University of Milan and Fondazione IRCCS Ca Granda	Esposito,S., Zampiero,A., Piralla,A. and Principi,N.
EPI_ISL_2594515, EPI_ISL_2594516, EPI_ISL_2594517	Department of Respiratory Medicine, Children's Hospital of Chongqing Medical University	Department of Respiratory Medicine, Children's Hospital of Chongqing Medical University	Ren,L. and Liu,E.
EPI_ISL_2594519	Department of Respiratory Medicine, Children's Hospital, Chongqing Medical University	Department of Respiratory Medicine, Children's Hospital, Chongqing Medical University	Ren,L. and Liu,E.
EPI_ISL_2594520, EPI_ISL_2594521, EPI_ISL_2594522	Pediatric Clinic 1, Department of Pathophysiology and Transplantation, University of Milan and Fondazione IRCCS Ca Granda	Pediatric Clinic 1, Department of Pathophysiology and Transplantation, University of Milan and Fondazione IRCCS Ca Granda	Esposito,S., Zampiero,A., Piralla,A. and Principi,N.
EPI_ISL_2594524, EPI_ISL_2594525, EPI_ISL_2594526, EPI_ISL_2594527	Department of Respiratory Medicine, Children's Hospital of Chongqing Medical University	Department of Respiratory Medicine, Children's Hospital of Chongqing Medical University	Ren,L. and Liu,E.
EPI_ISL_2594528	Pediatric Clinic 1, Department of Pathophysiology and Transplantation, University of Milan and Fondazione IRCCS Ca Granda	Pediatric Clinic 1, Department of Pathophysiology and Transplantation, University of Milan and Fondazione IRCCS Ca Granda	Esposito,S., Zampiero,A., Piralla,A. and Principi,N.
EPI_ISL_2594530, EPI_ISL_2594531	Virology Section, Department of Microbiology, King George's Medical University	Virology Section, Department of Microbiology, King George's Medical University	Singh,A.K., Jain,A., Jain,B., Dangi,T., Verma,A.K., Dwivedi,M. and Kushwaha,R.
EPI_ISL_2594532	Division of Public Health Research, Gyeonggi Province institute of Health and Environment	Division of Public Health Research, Gyeonggi Province institute of Health and Environment	Park,E., Park,P., Huh,J., Yun,H., Lee,H., Yoon,M., Lee,S. and Ko,G.
EPI_ISL_2594534	Department of Public Health Laboratory Sciences, West China School of Public Health, Sichuan University	Department of Public Health Laboratory Sciences, West China School of Public Health, Sichuan University	Hu,W.P. and Pei,F.X.
EPI_ISL_2594535	WHO WPRO Measles Regional Reference Lab, Key Laboratory of Medical Virology Ministry of Health, National Institute for Viral Disease Control and Prevention, China Center for Disease Control and Prevention	WHO WPRO Measles Regional Reference Lab, Key Laboratory of Medical Virology Ministry of Health, National Institute for Viral Disease Control and Prevention, China Center for Disease Control and Prevention	Zhang,Y., Song,J. and Xu,W.
EPI_ISL_2594536, EPI_ISL_2594537, EPI_ISL_2594538, EPI_ISL_2594540	Department of Public Health Laboratory Sciences, West China School of Public Health, Sichuan University	Department of Public Health Laboratory Sciences, West China School of Public Health, Sichuan University	Hu,W.P. and Pei,F.X.
EPI_ISL_2594541	Division of Public Health Research, Gyeonggi Province institute of Health and Environment	Division of Public Health Research, Gyeonggi Province institute of Health and Environment	Park,E., Park,P., Huh,J., Yun,H., Lee,H., Yoon,M., Lee,S. and Ko,G.
EPI_ISL_2594542	Department of Clinical Laboratory, Fujian Provincial Hospital	Department of Clinical Laboratory, Fujian Provincial Hospital	Su,Y., Wu,Y., Tian,R. and Liang,G.
EPI_ISL_2594544, EPI_ISL_2594545	Virology Section, Department of Microbiology, King George's Medical University	Virology Section, Department of Microbiology, King George's Medical University	Singh,A.K., Jain,A., Jain,B., Dangi,T., Verma,A.K., Dwivedi,M. and Kushwaha,R.
EPI_ISL_2594546	Central Laboratory, Guangzhou Women and Children's Medical Center	Central Laboratory, Guangzhou Women and Children's Medical Center	Xie,J.H., Zhu,B., Zhong,J.Y., Chen,Y. and Zhang,Y.Y.
EPI_ISL_2594547	Department of Public Health Laboratory Sciences, West China School of Public Health, Sichuan University	Department of Public Health Laboratory Sciences, West China School of Public Health, Sichuan University	Hu,W.P. and Pei,F.X.
EPI_ISL_2594549	Virology Section, Department of Microbiology, King George's Medical University	Virology Section, Department of Microbiology, King George's Medical University	Singh,A.K., Jain,A., Jain,B., Dangi,T., Verma,A.K., Dwivedi,M. and Kushwaha,R.
EPI_ISL_2594550	Epidemiology and Demography, KEMRI - Wellcome Trust Research Programme, Centre for Geographic Medicine Research	Epidemiology and Demography, KEMRI - Wellcome Trust Research Programme, Centre for Geographic Medicine Research	Oketch,J.W., Kamau,E., Otieno,J.R., Mwema,A., Lewa,C., Agoti,C.N. and Nokes,J.D.
EPI_ISL_2594551, EPI_ISL_2594552	Laboratory Medicine and Pathobiology, University of Toronto	Laboratory Medicine and Pathobiology, University of Toronto	Granados,A., Duvvuri,V., Rosenfeld,P., Eshaghi,A. and Gubbay,J.B.
EPI_ISL_2594554	Pathogen Diagnostic Center, Institut Pasteur of Shanghai	Pathogen Diagnostic Center, Institut Pasteur of Shanghai	Liu,J., Mu,Y., Dong,W., Yao,F., Yan,H., Lan,K. and Zhang,C.
EPI_ISL_2594555	Epidemiology and Demography, KEMRI - Wellcome Trust Research Programme, Centre for Geographic Medicine Research	Epidemiology and Demography, KEMRI - Wellcome Trust Research Programme, Centre for Geographic Medicine Research	Oketch,J.W., Kamau,E., Otieno,J.R., Mwema,A., Lewa,C., Agoti,C.N. and Nokes,J.D.
EPI_ISL_2594557, EPI_ISL_2594558	Laboratory Medicine and Pathobiology, University of Toronto	Laboratory Medicine and Pathobiology, University of Toronto	Granados,A., Duvvuri,V., Rosenfeld,P., Eshaghi,A. and Gubbay,J.B.
EPI_ISL_2594559	Department of Epidemiology and Demography, Kenya Medical Research Institute (KEMRI) - Wellcome Trust Research Programme	Department of Epidemiology and Demography, Kenya Medical Research Institute (KEMRI) - Wellcome Trust Research Programme	Otieno,J.R., Agoti,C.N., Gitahi,C.W., Bett,A., Ngama,M., Medley,G.F., Cane,P.A. and Nokes,J.D.
EPI_ISL_2594560	Laboratory Medicine and Pathobiology, University of Toronto	Laboratory Medicine and Pathobiology, University of Toronto	Granados,A., Duvvuri,V., Rosenfeld,P., Eshaghi,A. and Gubbay,J.B.
EPI_ISL_2594562, EPI_ISL_2594563, EPI_ISL_2594564	Virology Section, Department of Microbiology, King George's Medical University	Virology Section, Department of Microbiology, King George's Medical University	Singh,A.K., Jain,A., Jain,B., Dangi,T., Verma,A.K., Dwivedi,M. and Kushwaha,R.
EPI_ISL_2594566, EPI_ISL_2594567, EPI_ISL_2594568, EPI_ISL_2594570, EPI_ISL_2594571, EPI_ISL_2594572, EPI_ISL_2594573, EPI_ISL_2594575, EPI_ISL_2594576, EPI_ISL_2594577	Chinese Academy of Medical Sciences & Peking Union Medical College	Chinese Academy of Medical Sciences & Peking Union Medical College	Jia,B., Xiao,Y., Wang,Y., Chen,L., Zhang,J., Ren,L. and Wang,J.
EPI_ISL_2594579, EPI_ISL_2594580, EPI_ISL_2594581, EPI_ISL_2594582, EPI_ISL_2594584, EPI_ISL_2594585, EPI_ISL_2594586, EPI_ISL_2594587, EPI_ISL_2594589	University of Wuerzburg, Institute of Virology and Immunobiology	University of Wuerzburg, Institute of Virology and Immunobiology	Prifert,C., Hofmann,D. and Weissbrich,B.
EPI_ISL_2594590, EPI_ISL_2594591, EPI_ISL_2594593, EPI_ISL_2594594, EPI_ISL_2594595, EPI_ISL_2594596, EPI_ISL_2594598, EPI_ISL_2594599, EPI_ISL_2594600, EPI_ISL_2594602, EPI_ISL_2594603, EPI_ISL_2594604, EPI_ISL_2594606, EPI_ISL_2594607, EPI_ISL_2594608			
see above	Chinese Academy of Medical Sciences & Peking Union Medical College	Chinese Academy of Medical Sciences & Peking Union Medical College	Jia,B., Xiao,Y., Wang,Y., Chen,L., Zhang,J., Ren,L. and Wang,J.
EPI_ISL_2594609, EPI_ISL_2594611, EPI_ISL_2594612, EPI_ISL_2594613, EPI_ISL_2594614, EPI_ISL_2594615, EPI_ISL_2594617, EPI_ISL_2594618, EPI_ISL_2594619, EPI_ISL_2594621	University of Wuerzburg, Institute of Virology and Immunobiology	University of Wuerzburg, Institute of Virology and Immunobiology	Prifert,C., Hofmann,D. and Weissbrich,B.
EPI_ISL_2594622, EPI_ISL_2594623, EPI_ISL_2594624, EPI_ISL_2594626, EPI_ISL_2594627, EPI_ISL_2594628, EPI_ISL_2594629, EPI_ISL_2594631, EPI_ISL_2594632, EPI_ISL_2594633, EPI_ISL_2594635			
see above	Health Science, Universidade Federal Do Parana	Health Science, Universidade Federal Do Parana	Moreira,F.B., Santos,J.S., Avanzi,V.M., Nogueira,M.B., Vidal,L.R.R. and Raboni,S.M.
EPI_ISL_2594636, EPI_ISL_2594637, EPI_ISL_2594640, EPI_ISL_2594641	Virology Department, Hospices Civils de Lyon	Virology Department, Hospices Civils de Lyon	Gaymard,A., Pichon,M. and Morfin,F.
EPI_ISL_2594643	Pediatrics, Mackay Memorial Hospital	Pediatrics, Mackay Memorial Hospital	Chi,H., Hsiao,K.-L., Weng,L.-C., Chiu,N.-C., Huang,L.-M., Chiu,Y.-Y., Liu,C.-P. and Liu,H.-F.
EPI_ISL_2594644, EPI_ISL_2594645, EPI_ISL_2594647	Health Science, Universidade Federal Do Parana	Health Science, Universidade Federal Do Parana	Moreira,F.B., Santos,J.S., Avanzi,V.M., Nogueira,M.B., Vidal,L.R.R. and Raboni,S.M.
EPI_ISL_2594648	Virology Department, Hospices Civils de Lyon	Virology Department, Hospices Civils de Lyon	Gaymard,A., Pichon,M. and Morfin,F.
EPI_ISL_2594649	Pediatrics, Mackay Memorial Hospital	Pediatrics, Mackay Memorial Hospital	Chi,H., Hsiao,K.-L., Weng,L.-C., Chiu,N.-C., Huang,L.-M., Chiu,Y.-Y., Liu,C.-P. and Liu,H.-F.
EPI_ISL_2594650, EPI_ISL_2594652	Pediatrics, Show-Chwan Memorial Hospital	Pediatrics, Show-Chwan Memorial Hospital	Lee,C.-Y.
EPI_ISL_2594653	Department of Biomedical Sciences for Health, University of Milan	Department of Biomedical Sciences for Health, University of Milan	Martinelli,M., Frati,E.R., Zappa,A., Ebranati,E., Bianchi,S., Pariani,E., Amendola,A., Zehender,G. and Tanzi,E.
EPI_ISL_2594654, EPI_ISL_2594656, EPI_ISL_2594657	Pediatrics, Show-Chwan Memorial Hospital	Pediatrics, Show-Chwan Memorial Hospital	Lee,C.-Y.
EPI_ISL_2594658, EPI_ISL_2594659, EPI_ISL_2594661	Pediatrics, Mackay Memorial Hospital	Pediatrics, Mackay Memorial Hospital	Chi,H., Hsiao,K.-L., Weng,L.-C., Chiu,N.-C., Huang,L.-M., Chiu,Y.-Y., Liu,C.-P. and Liu,H.-F.
EPI_ISL_2594662, EPI_ISL_2594663, EPI_ISL_2594665	Pediatrics, Show-Chwan Memorial Hospital	Pediatrics, Show-Chwan Memorial Hospital	Lee,C.-Y.
EPI_ISL_2594666, EPI_ISL_2594667	Pediatrics, Mackay Memorial Hospital	Pediatrics, Mackay Memorial Hospital	Chi,H., Hsiao,K.-L., Weng,L.-C., Chiu,N.-C., Huang,L.-M., Chiu,Y.-Y., Liu,C.-P. and Liu,H.-F.
EPI_ISL_2594668, EPI_ISL_2594670, EPI_ISL_2594672	Pediatrics, Show-Chwan Memorial Hospital	Pediatrics, Show-Chwan Memorial Hospital	Lee,C.-Y.
EPI_ISL_2594673, EPI_ISL_2594675	Laboratory of Virology, Capital Institute of Pediatrics	Laboratory of Virology, Capital Institute of Pediatrics	Cui,G., Deng,J., Zhu,R., Qian,Y., Sun,Y., Zhao,L. and Wang,F.
EPI_ISL_2594677, EPI_ISL_2594678, EPI_ISL_2594680	Pediatrics, Mackay Memorial Hospital	Pediatrics, Mackay Memorial Hospital	Chi,H., Hsiao,K.-L., Weng,L.-C., Chiu,N.-C., Huang,L.-M., Chiu,Y.-Y., Liu,C.-P. and Liu,H.-F.

EPI_ISL_2594682, EPI_ISL_2594684	Pediatrics, Show-Chwan Memorial Hospital	Pediatrics, Show-Chwan Memorial Hospital	Lee,C.-Y.
EPI_ISL_2594686, EPI_ISL_2594688	Chinese Academy of Medical Sciences & Peking Union Medical College	Chinese Academy of Medical Sciences & Peking Union Medical College	Jia,B., Xiao,Y., Wang,Y., Chen,L., Zhang,J., Ren,L. and Wang,J.
EPI_ISL_2594689	Laboratory of Virology, Capital Institute of Pediatrics	Laboratory of Virology, Capital Institute of Pediatrics	Cui,G., Deng,J., Zhu,R., Qian,Y., Sun,Y., Zhao,L. and Wang,F.
EPI_ISL_2594691, EPI_ISL_2594693, EPI_ISL_2594695	Chinese Academy of Medical Sciences & Peking Union Medical College	Chinese Academy of Medical Sciences & Peking Union Medical College	Jia,B., Xiao,Y., Wang,Y., Chen,L., Zhang,J., Ren,L. and Wang,J.
EPI_ISL_2594696, EPI_ISL_2594698	Laboratory of Virology, Capital Institute of Pediatrics	Laboratory of Virology, Capital Institute of Pediatrics	Cui,G., Deng,J., Zhu,R., Qian,Y., Sun,Y., Zhao,L. and Wang,F.
EPI_ISL_2594700, EPI_ISL_2594702	Chinese Academy of Medical Sciences & Peking Union Medical College	Chinese Academy of Medical Sciences & Peking Union Medical College	Jia,B., Xiao,Y., Wang,Y., Chen,L., Zhang,J., Ren,L. and Wang,J.
EPI_ISL_2594703	Laboratory of Virology, Capital Institute of Pediatrics	Laboratory of Virology, Capital Institute of Pediatrics	Cui,G., Deng,J., Zhu,R., Qian,Y., Sun,Y., Zhao,L. and Wang,F.
EPI_ISL_2594705, EPI_ISL_2594707, EPI_ISL_2594709	Chinese Academy of Medical Sciences & Peking Union Medical College	Chinese Academy of Medical Sciences & Peking Union Medical College	Jia,B., Xiao,Y., Wang,Y., Chen,L., Zhang,J., Ren,L. and Wang,J.
EPI_ISL_2594711, EPI_ISL_2594713, EPI_ISL_2594714, EPI_ISL_2594716, EPI_ISL_2594718, EPI_ISL_2594719	Laboratory of Virology, Capital Institute of Pediatrics	Laboratory of Virology, Capital Institute of Pediatrics	Cui,G., Deng,J., Zhu,R., Qian,Y., Sun,Y., Zhao,L. and Wang,F.
EPI_ISL_2594721	Health Science, Universidade Federal Do Parana	Health Science, Universidade Federal Do Parana	Moreira,F.B., Santos,J.S., Avanzi,V.M., Nogueira,M.B., Vidal,L.R.R. and Raboni,S.M.
EPI_ISL_2594723, EPI_ISL_2594725, EPI_ISL_2594727, EPI_ISL_2594728	Chinese Academy of Medical Sciences & Peking Union Medical College	Chinese Academy of Medical Sciences & Peking Union Medical College	Jia,B., Xiao,Y., Wang,Y., Chen,L., Zhang,J., Ren,L. and Wang,J.
EPI_ISL_2594730, EPI_ISL_2594732, EPI_ISL_2594734	Laboratory of Virology, Capital Institute of Pediatrics	Laboratory of Virology, Capital Institute of Pediatrics	Cui,G., Deng,J., Zhu,R., Qian,Y., Sun,Y., Zhao,L. and Wang,F.
EPI_ISL_2594736, EPI_ISL_2594738, EPI_ISL_2594739, EPI_ISL_2594741, EPI_ISL_2594743	Chinese Academy of Medical Sciences & Peking Union Medical College	Chinese Academy of Medical Sciences & Peking Union Medical College	Jia,B., Xiao,Y., Wang,Y., Chen,L., Zhang,J., Ren,L. and Wang,J.
EPI_ISL_2594745, EPI_ISL_2594746, EPI_ISL_2594748, EPI_ISL_2594750, EPI_ISL_2594752, EPI_ISL_2594754	Laboratory of Virology, Capital Institute of Pediatrics	Laboratory of Virology, Capital Institute of Pediatrics	Cui,G., Deng,J., Zhu,R., Qian,Y., Sun,Y., Zhao,L. and Wang,F.
EPI_ISL_2594755	Depto Microbiologia, Instituto de Ciencias Biomedicas, Universidade de Sao Paulo	Depto Microbiologia, Instituto de Ciencias Biomedicas, Universidade de Sao Paulo	Moura,F.E.A., Thomazelli,L.M., Campelo,F.S., Delfraro,A., Arbiza,J. and Durigon,E.L.
EPI_ISL_2594757	Pediatric Clinic 1, Department of Pathophysiology and Transplantation, University of Milan and Fondazione IRCCS Ca Granda	Pediatric Clinic 1, Department of Pathophysiology and Transplantation, University of Milan and Fondazione IRCCS Ca Granda	Esposito,S., Zampiero,A., Piralla,A. and Principi,N.
EPI_ISL_2594759, EPI_ISL_2594761, EPI_ISL_2594762, EPI_ISL_2594764, EPI_ISL_2594766, EPI_ISL_2594768, EPI_ISL_2594770, EPI_ISL_2594771, EPI_ISL_2594773, EPI_ISL_2594775	Department of Respiratory Medicine, Children's Hospital of Chongqing Medical University	Department of Respiratory Medicine, Children's Hospital of Chongqing Medical University	Ren,L. and Liu,E.
EPI_ISL_2594777	Pediatric Clinic 1, Department of Pathophysiology and Transplantation, University of Milan and Fondazione IRCCS Ca Granda	Pediatric Clinic 1, Department of Pathophysiology and Transplantation, University of Milan and Fondazione IRCCS Ca Granda	Esposito,S., Zampiero,A., Piralla,A. and Principi,N.
EPI_ISL_2594779, EPI_ISL_2594780	School of Medicine, University of Washington	School of Medicine, University of Washington	Scott,E.M., Magaret,A., Kuypers,J., Stewart,L., Shrestha,L., Tielsch,J.M., Steinhoff,M., Katz,J., Khatry,S.K., LeClerq,S.C., Englund,J.A. and Chu,H.Y.
EPI_ISL_2594782	Division of Public Health Research, Gyeonggi Province institute of Health and Environment	Division of Public Health Research, Gyeonggi Province institute of Health and Environment	Park,E., Park,P., Huh,J., Yun,H., Lee,H., Yoon,M., Lee,S. and Ko,G.
EPI_ISL_2594784, EPI_ISL_2594786, EPI_ISL_2594787, EPI_ISL_2594789	Department of Public Health Laboratory Sciences, West China School of Public Health, Sichuan University	Department of Public Health Laboratory Sciences, West China School of Public Health, Sichuan University	Hu,W.P. and Pei,F.X.
EPI_ISL_2594791	WHO WPRO Measles Regional Reference Lab, Key Laboratory of Medical Virology Ministry of Health, National Institute for Viral Disease Control and Prevention, China Center for Disease Control and Prevention	WHO WPRO Measles Regional Reference Lab, Key Laboratory of Medical Virology Ministry of Health, National Institute for Viral Disease Control and Prevention, China Center for Disease Control and Prevention	Zhang,Y., Song,J. and Xu,W.
EPI_ISL_2594793	Department of Public Health Laboratory Sciences, West China School of Public Health, Sichuan University	Department of Public Health Laboratory Sciences, West China School of Public Health, Sichuan University	Hu,W.P. and Pei,F.X.
EPI_ISL_2594794	Laboratory Medicine and Pathobiology, University of Toronto	Laboratory Medicine and Pathobiology, University of Toronto	Granados,A., Duvvuri,V., Rosenfeld,P., Eshaghi,A. and Gubbay,J.B.
EPI_ISL_2594796, EPI_ISL_2594798	Virology Section, Department of Microbiology, King George's Medical University	Virology Section, Department of Microbiology, King George's Medical University	Singh,A.K., Jain,A., Jain,B., Dangi,T., Verma,A.K., Dwivedi,M. and Kushwaha,R.
EPI_ISL_2594800	Department of Public Health Laboratory Sciences, West China School of Public Health, Sichuan University	Department of Public Health Laboratory Sciences, West China School of Public Health, Sichuan University	Hu,W.P. and Pei,F.X.
EPI_ISL_2594801	Laboratory Medicine and Pathobiology, University of Toronto	Laboratory Medicine and Pathobiology, University of Toronto	Granados,A., Duvvuri,V., Rosenfeld,P., Eshaghi,A. and Gubbay,J.B.
EPI_ISL_2594804	Microbiology Department, Virology Division, College of Medicine, Taif University	Microbiology Department, Virology Division, College of Medicine, Taif University	Al Aboud,D.M., Al Aboud,N.M., Al-Malky,M.I.R. and Abdel-Moneim,A.S.
EPI_ISL_2594806	WHO WPRO Measles Regional Reference Lab, Key Laboratory of Medical Virology Ministry of Health, National Institute for Viral Disease Control and Prevention, China Center for Disease Control and Prevention	WHO WPRO Measles Regional Reference Lab, Key Laboratory of Medical Virology Ministry of Health, National Institute for Viral Disease Control and Prevention, China Center for Disease Control and Prevention	Zhang,Y., Song,J. and Xu,W.
EPI_ISL_2594807, EPI_ISL_2594809	Virology Section, Department of Microbiology, King George's Medical University	Virology Section, Department of Microbiology, King George's Medical University	Singh,A.K., Jain,A., Jain,B., Dangi,T., Verma,A.K., Dwivedi,M. and Kushwaha,R.
EPI_ISL_2594811	Pathogen Diagnostic Center, Institut Pasteur of Shanghai	Pathogen Diagnostic Center, Institut Pasteur of Shanghai	Liu,J., Mu,Y., Dong,W., Yao,F., Yan,H., Lan,K. and Zhang,C.
EPI_ISL_2594813	Department for Infectious Diseases Virology, University of Heidelberg	Department for Infectious Diseases Virology, University of Heidelberg	Tabatabai,J., Thielen,A., Lehners,N., Daeumer,M. and Schnitzler,P.
EPI_ISL_2594815	Laboratory Medicine and Pathobiology, University of Toronto	Laboratory Medicine and Pathobiology, University of Toronto	Granados,A., Duvvuri,V., Rosenfeld,P., Eshaghi,A. and Gubbay,J.B.
EPI_ISL_2594817, EPI_ISL_2594819	Virology Section, Department of Microbiology, King George's Medical University	Virology Section, Department of Microbiology, King George's Medical University	Singh,A.K., Jain,A., Jain,B., Dangi,T., Verma,A.K., Dwivedi,M. and Kushwaha,R.
EPI_ISL_2594821	WHO WPRO Measles Regional Reference Lab, Key Laboratory of Medical Virology Ministry of Health, National Institute for Viral Disease Control and Prevention, China Center for Disease Control and Prevention	WHO WPRO Measles Regional Reference Lab, Key Laboratory of Medical Virology Ministry of Health, National Institute for Viral Disease Control and Prevention, China Center for Disease Control and Prevention	Zhang,Y., Song,J. and Xu,W.
EPI_ISL_2594823	Central Laboratory, Guangzhou Women and Children's Medical Center	Central Laboratory, Guangzhou Women and Children's Medical Center	Xie,J.H., Zhu,B., Zhong,J.Y., Chen,Y. and Zhang,Y.Y.
EPI_ISL_2594825, EPI_ISL_2594826	Virology, School of Public Health, Tehran University of Medical Sciences	Virology, School of Public Health, Tehran University of Medical Sciences	Yavarian,J., Faghihloo,E. and Mokhtari Azad,T.
EPI_ISL_2594828	Virology, Tropical Medicine Institute Pedro Kouri	Virology, Tropical Medicine Institute Pedro Kouri	Valdes,O., Corso,M., Pinon,A., Acosta,B., Savon,C., Gonzalez,G., Mune,M., Gonzalez,G., Hernandez,B., Echevarria,Y. and Oropesa,S.
EPI_ISL_2594830	Department of Clinical Laboratory, Fujian Provincial Hospital	Department of Clinical Laboratory, Fujian Provincial Hospital	Su,Y., Wu,Y., Tian,R. and Liang,G.
EPI_ISL_2594832	Epidemiology and Demography, KEMRI - Wellcome Trust Research Programme, Centre for Geographic Medicine Research	Epidemiology and Demography, KEMRI - Wellcome Trust Research Programme, Centre for Geographic Medicine Research	Oketch,J.W., Kamau,E., Otieno,J.R., Mwema,A., Lewa,C., Agoti,C.N. and Nokes,J.D.
EPI_ISL_2594833, EPI_ISL_2594835	WHO WPRO Measles Regional Reference Lab, Key Laboratory of Medical Virology Ministry of Health, National Institute for Viral Disease Control and Prevention, China Center for Disease Control and Prevention	WHO WPRO Measles Regional Reference Lab, Key Laboratory of Medical Virology Ministry of Health, National Institute for Viral Disease Control and Prevention, China Center for Disease Control and Prevention	Zhang,Y., Song,J. and Xu,W.
EPI_ISL_2594837	Laboratory Medicine and Pathobiology, University of Toronto	Laboratory Medicine and Pathobiology, University of Toronto	Granados,A., Duvvuri,V., Rosenfeld,P., Eshaghi,A. and Gubbay,J.B.
EPI_ISL_2594838	University of Wuerzburg, Institute of Virology and Immunobiology	University of Wuerzburg, Institute of Virology and Immunobiology	Prifert,C., Hofmann,D., Schnitzler,P. and Weissbrich,B.
EPI_ISL_2594840	Department of Respiratory Medicine, Children's Hospital of Chongqing Medical University	Department of Respiratory Medicine, Children's Hospital of Chongqing Medical University	Ren,L. and Liu,E.
EPI_ISL_2594842	Department of Respiratory Medicine, Children's Hospital, Chongqing Medical University	Department of Respiratory Medicine, Children's Hospital, Chongqing Medical University	Ren,L. and Liu,E.
EPI_ISL_2594844	Pediatric Clinic 1, Department of Pathophysiology and Transplantation, University of Milan and Fondazione IRCCS Ca Granda	Pediatric Clinic 1, Department of Pathophysiology and Transplantation, University of Milan and Fondazione IRCCS Ca Granda	Esposito,S., Zampiero,A., Piralla,A. and Principi,N.
EPI_ISL_2594846	Department of Respiratory Medicine, Children's Hospital of Chongqing Medical University	Department of Respiratory Medicine, Children's Hospital of Chongqing Medical University	Ren,L. and Liu,E.
EPI_ISL_2594847, EPI_ISL_2594848	Minoru Nidaira Okinawa Prefectural Institute of Health and Environment, Department of Biological Science	Minoru Nidaira Okinawa Prefectural Institute of Health and Environment, Department of Biological Science	Nidaira,M. and Taira,K.
EPI_ISL_2594849, EPI_ISL_2594852	Department of Pediatrics, Center of Excellence in Clinical Virology, Faculty of Medicine, Chulalongkorn University	Department of Pediatrics, Center of Excellence in Clinical Virology, Faculty of Medicine, Chulalongkorn University	Thongpan,I., Mauleekoonphairoj,J., Vichiwattana,P., Korkong,S., Vongpunsawad,S. and Poovorawan,Y.
EPI_ISL_2594853	Department of Epidemiology and Demography, Kenya Medical Research Institute (KEMRI) - Wellcome Trust Research Programme	Department of Epidemiology and Demography, Kenya Medical Research Institute (KEMRI) - Wellcome Trust Research Programme	Otieno,J.R., Agoti,C.N., Gitahi,C.W., Bett,A., Ngama,M., Medley,G.F., Cane,P.A. and Nokes,J.D.
EPI_ISL_2594855	Epidemiology and Demography, Kenya Medical Research Institute (KEMRI) - Wellcome Trust Research Programme	Epidemiology and Demography, Kenya Medical Research Institute (KEMRI) - Wellcome Trust Research Programme	Agoti,C.N., Otieno,J.R., Gitahi,C.W., Cane,P.A. and Nokes,D.J.
EPI_ISL_2594857	Virology Section, Department of Microbiology, King George's Medical University	Virology Section, Department of Microbiology, King George's Medical University	Singh,A.K., Jain,A., Jain,B., Dangi,T., Verma,A.K., Dwivedi,M. and Kushwaha,R.
EPI_ISL_2594859	Microbiology Department, Virology Division, College of Medicine, Taif University	Microbiology Department, Virology Division, College of Medicine, Taif University	Al Aboud,D.M., Al Aboud,N.M., Al-Malky,M.I.R. and Abdel-Moneim,A.S.
EPI_ISL_2594861, EPI_ISL_2594863	WHO WPRO Measles Regional Reference Lab, Key Laboratory of Medical Virology Ministry of Health, National Institute for Viral Disease Control and Prevention, China Center for Disease Control and Prevention	WHO WPRO Measles Regional Reference Lab, Key Laboratory of Medical Virology Ministry of Health, National Institute for Viral Disease Control and Prevention, China Center for Disease Control and Prevention	Zhang,Y., Song,J. and Xu,W.
EPI_ISL_2594864, EPI_ISL_2594866	University of Wuerzburg, Institute of Virology and Immunobiology	University of Wuerzburg, Institute of Virology and Immunobiology	Prifert,C., Hofmann,D., Schnitzler,P. and Weissbrich,B.

EPI_ISL_2594868	Department of Epidemiology and Demography, Kenya Medical Research Institute (KEMRI) - Wellcome Trust Research Programme	Department of Epidemiology and Demography, Kenya Medical Research Institute (KEMRI) - Wellcome Trust Research Programme	Otieno,J.R., Agoti,C.N., Gitahi,C.W., Bett,A., Ngama,M., Medley,G.F., Cane,P.A. and Nokes,J.D.
EPI_ISL_2594870	Epidemiology and Demography, Kenya Medical Research Institute (KEMRI) - Wellcome Trust Research Programme	Epidemiology and Demography, Kenya Medical Research Institute (KEMRI) - Wellcome Trust Research Programme	Agoti,C.N., Otieno,J.R., Gitahi,C.W., Cane,P.A. and Nokes,D.J.
EPI_ISL_2594871	University of Wuerzburg, Institute of Virology and Immunobiology	University of Wuerzburg, Institute of Virology and Immunobiology	Prifert,C., Hofmann,D., Schnitzler,P. and Weissbrich,B.
EPI_ISL_2594873	Laboratory Medicine and Pathobiology, University of Toronto	Laboratory Medicine and Pathobiology, University of Toronto	Granados,A., Duvvuri,V., Rosenfeld,P., Eshaghi,A. and Gubbay,J.B.
EPI_ISL_2594876	University of Wuerzburg, Institute of Virology and Immunobiology	University of Wuerzburg, Institute of Virology and Immunobiology	Prifert,C., Hofmann,D., Schnitzler,P. and Weissbrich,B.
EPI_ISL_2594878, EPI_ISL_2594879	Center for Infectious Diseases, School of Public Health, University of Texas Health Science Center	Center for Infectious Diseases, School of Public Health, University of Texas Health Science Center	Bahl,J., Hixson,J., Kim,D.-K., Qiu,X., Piedra,P.A., Piedra,F.-A., Avadhanula,V. and Machado,A.A.
EPI_ISL_2594881	Department for Infectious Diseases Virology, University of Heidelberg	Department for Infectious Diseases Virology, University of Heidelberg	Tabatabai,J., Thielen,A., Lehners,N., Daeumer,M. and Schnitzler,P.
EPI_ISL_2594883	University of Wuerzburg, Institute of Virology and Immunobiology	University of Wuerzburg, Institute of Virology and Immunobiology	Prifert,C., Hofmann,D., Schnitzler,P. and Weissbrich,B.
EPI_ISL_2594885	Department of Epidemiology and Demography, Kenya Medical Research Institute (KEMRI) - Wellcome Trust Research Programme	Department of Epidemiology and Demography, Kenya Medical Research Institute (KEMRI) - Wellcome Trust Research Programme	Otieno,J.R., Agoti,C.N., Gitahi,C.W., Bett,A., Ngama,M., Medley,G.F., Cane,P.A. and Nokes,J.D.
EPI_ISL_2594887	Epidemiology and Demography, Kenya Medical Research Institute (KEMRI) - Wellcome Trust Research Programme	Epidemiology and Demography, Kenya Medical Research Institute (KEMRI) - Wellcome Trust Research Programme	Agoti,C.N., Otieno,J.R., Gitahi,C.W., Cane,P.A. and Nokes,D.J.
EPI_ISL_2594889	WHO WPRO Measles Regional Reference Lab, Key Laboratory of Medical Virology Ministry of Health, National Institute for Viral Disease Control and Prevention, China Center for Disease Control and Prevention	WHO WPRO Measles Regional Reference Lab, Key Laboratory of Medical Virology Ministry of Health, National Institute for Viral Disease Control and Prevention, China Center for Disease Control and Prevention	Zhang,Y., Song,J. and Xu,W.
EPI_ISL_2594890	Epidemiology and Demography, KEMRI - Wellcome Trust Research Programme, Centre for Geographic Medicine Research	Epidemiology and Demography, KEMRI - Wellcome Trust Research Programme, Centre for Geographic Medicine Research	Oketch,J.W., Kamau,E., Otieno,J.R., Mwema,A., Lewa,C., Agoti,C.N. and Nokes,J.D.
EPI_ISL_2594892, EPI_ISL_2594894	Microbiology Department, Virology Division, College of Medicine, Taif University	Microbiology Department, Virology Division, College of Medicine, Taif University	Al Aboud,D.M., Al Aboud,N.M., Al-Malky,M.I.R. and Abdel-Moneim,A.S.
EPI_ISL_2594896	WHO WPRO Measles Regional Reference Lab, Key Laboratory of Medical Virology Ministry of Health, National Institute for Viral Disease Control and Prevention, China Center for Disease Control and Prevention	WHO WPRO Measles Regional Reference Lab, Key Laboratory of Medical Virology Ministry of Health, National Institute for Viral Disease Control and Prevention, China Center for Disease Control and Prevention	Zhang,Y., Song,J. and Xu,W.
EPI_ISL_2594898	University of Wuerzburg, Institute of Virology and Immunobiology	University of Wuerzburg, Institute of Virology and Immunobiology	Prifert,C., Hofmann,D., Schnitzler,P. and Weissbrich,B.
EPI_ISL_2594899	Epidemiology and Demography, Kenya Medical Research Institute (KEMRI) - Wellcome Trust Research Programme	Epidemiology and Demography, Kenya Medical Research Institute (KEMRI) - Wellcome Trust Research Programme	Agoti,C.N., Otieno,J.R., Gitahi,C.W., Cane,P.A. and Nokes,D.J.
EPI_ISL_2594901	WHO WPRO Measles Regional Reference Lab, Key Laboratory of Medical Virology Ministry of Health, National Institute for Viral Disease Control and Prevention, China Center for Disease Control and Prevention	WHO WPRO Measles Regional Reference Lab, Key Laboratory of Medical Virology Ministry of Health, National Institute for Viral Disease Control and Prevention, China Center for Disease Control and Prevention	Zhang,Y., Song,J. and Xu,W.
EPI_ISL_2594903	Department of Public Health Laboratory Sciences, West China School of Public Health, Sichuan University	Department of Public Health Laboratory Sciences, West China School of Public Health, Sichuan University	Hu,W.P. and Pei,F.X.
EPI_ISL_2594905	Pathogen Diagnostic Center, Institut Pasteur of Shanghai	Pathogen Diagnostic Center, Institut Pasteur of Shanghai	Liu,J., Mu,Y., Dong,W., Yao,F., Yan,H., Lan,K. and Zhang,C.
EPI_ISL_2594906	Department for Infectious Diseases Virology, University of Heidelberg	Department for Infectious Diseases Virology, University of Heidelberg	Tabatabai,J., Thielen,A., Lehners,N., Daeumer,M. and Schnitzler,P.
EPI_ISL_2594908	University of Wuerzburg, Institute of Virology and Immunobiology	University of Wuerzburg, Institute of Virology and Immunobiology	Prifert,C., Hofmann,D., Schnitzler,P. and Weissbrich,B.
EPI_ISL_2594910	Epidemiology and Demography, KEMRI - Wellcome Trust Research Programme, Centre for Geographic Medicine Research	Epidemiology and Demography, KEMRI - Wellcome Trust Research Programme, Centre for Geographic Medicine Research	Oketch,J.W., Kamau,E., Otieno,J.R., Mwema,A., Lewa,C., Agoti,C.N. and Nokes,J.D.
EPI_ISL_2594911	Department of Respiratory Medicine, Children's Hospital, Chongqing Medical University	Department of Respiratory Medicine, Children's Hospital, Chongqing Medical University	Ren,L. and Liu,E.
EPI_ISL_2594913, EPI_ISL_2594915, EPI_ISL_2594917	Department of Respiratory Medicine, Children's Hospital of Chongqing Medical University	Department of Respiratory Medicine, Children's Hospital of Chongqing Medical University	Ren,L. and Liu,E.
EPI_ISL_2594919	Department of Epidemiology and Demography, Kenya Medical Research Institute (KEMRI) - Wellcome Trust Research Programme	Department of Epidemiology and Demography, Kenya Medical Research Institute (KEMRI) - Wellcome Trust Research Programme	Otieno,J.R., Agoti,C.N., Gitahi,C.W., Bett,A., Ngama,M., Medley,G.F., Cane,P.A. and Nokes,J.D.
EPI_ISL_2594920	Epidemiology and Demography, Kenya Medical Research Institute (KEMRI) - Wellcome Trust Research Programme	Epidemiology and Demography, Kenya Medical Research Institute (KEMRI) - Wellcome Trust Research Programme	Agoti,C.N., Otieno,J.R., Gitahi,C.W., Cane,P.A. and Nokes,D.J.
EPI_ISL_2594922	Department of Epidemiology and Demography, Kenya Medical Research Institute (KEMRI) - Wellcome Trust Research Programme	Department of Epidemiology and Demography, Kenya Medical Research Institute (KEMRI) - Wellcome Trust Research Programme	Otieno,J.R., Agoti,C.N., Gitahi,C.W., Bett,A., Ngama,M., Medley,G.F., Cane,P.A. and Nokes,J.D.
EPI_ISL_2594924	Epidemiology and Demography, Kenya Medical Research Institute (KEMRI) - Wellcome Trust Research Programme	Epidemiology and Demography, Kenya Medical Research Institute (KEMRI) - Wellcome Trust Research Programme	Agoti,C.N., Otieno,J.R., Gitahi,C.W., Cane,P.A. and Nokes,D.J.
EPI_ISL_2594926	Department of Epidemiology and Demography, Kenya Medical Research Institute (KEMRI) - Wellcome Trust Research Programme	Department of Epidemiology and Demography, Kenya Medical Research Institute (KEMRI) - Wellcome Trust Research Programme	Otieno,J.R., Agoti,C.N., Gitahi,C.W., Bett,A., Ngama,M., Medley,G.F., Cane,P.A. and Nokes,J.D.
EPI_ISL_2594928	Epidemiology and Demography, Kenya Medical Research Institute (KEMRI) - Wellcome Trust Research Programme	Epidemiology and Demography, Kenya Medical Research Institute (KEMRI) - Wellcome Trust Research Programme	Agoti,C.N., Otieno,J.R., Gitahi,C.W., Cane,P.A. and Nokes,D.J.
EPI_ISL_2594929	Department of Epidemiology and Demography, Kenya Medical Research Institute (KEMRI) - Wellcome Trust Research Programme	Department of Epidemiology and Demography, Kenya Medical Research Institute (KEMRI) - Wellcome Trust Research Programme	Otieno,J.R., Agoti,C.N., Gitahi,C.W., Bett,A., Ngama,M., Medley,G.F., Cane,P.A. and Nokes,J.D.
EPI_ISL_2594931	Epidemiology and Demography, Kenya Medical Research Institute (KEMRI) - Wellcome Trust Research Programme	Epidemiology and Demography, Kenya Medical Research Institute (KEMRI) - Wellcome Trust Research Programme	Agoti,C.N., Otieno,J.R., Gitahi,C.W., Cane,P.A. and Nokes,D.J.
EPI_ISL_2594933	Pathogen Diagnostic Center, Institut Pasteur of Shanghai	Pathogen Diagnostic Center, Institut Pasteur of Shanghai	Liu,J., Mu,Y., Dong,W., Yao,F., Yan,H., Lan,K. and Zhang,C.
EPI_ISL_2594934	Department of Epidemiology and Demography, Kenya Medical Research Institute (KEMRI) - Wellcome Trust Research Programme	Department of Epidemiology and Demography, Kenya Medical Research Institute (KEMRI) - Wellcome Trust Research Programme	Otieno,J.R., Agoti,C.N., Gitahi,C.W., Bett,A., Ngama,M., Medley,G.F., Cane,P.A. and Nokes,J.D.
EPI_ISL_2594935	Epidemiology and Demography, Kenya Medical Research Institute (KEMRI) - Wellcome Trust Research Programme	Epidemiology and Demography, Kenya Medical Research Institute (KEMRI) - Wellcome Trust Research Programme	Agoti,C.N., Otieno,J.R., Gitahi,C.W., Cane,P.A. and Nokes,D.J.
EPI_ISL_2594936	WHO WPRO Measles Regional Reference Lab, Key Laboratory of Medical Virology Ministry of Health, National Institute for Viral Disease Control and Prevention, China Center for Disease Control and Prevention	WHO WPRO Measles Regional Reference Lab, Key Laboratory of Medical Virology Ministry of Health, National Institute for Viral Disease Control and Prevention, China Center for Disease Control and Prevention	Zhang,Y., Song,J. and Xu,W.
EPI_ISL_2594937	Department of Protein Engineering, Latvian Biomedical Research and Study Centre	Department of Protein Engineering, Latvian Biomedical Research and Study Centre	Balmaks,R., Ribakova,I., Gardovska,D. and Kazaks,A.
EPI_ISL_2594938	Laboratory Medicine and Pathobiology, University of Toronto	Laboratory Medicine and Pathobiology, University of Toronto	Granados,A., Duvvuri,V., Rosenfeld,P., Eshaghi,A. and Gubbay,J.B.
EPI_ISL_2594939, EPI_ISL_2594940	Epidemiology and Demography, KEMRI - Wellcome Trust Research Programme, Centre for Geographic Medicine Research	Epidemiology and Demography, KEMRI - Wellcome Trust Research Programme, Centre for Geographic Medicine Research	Oketch,J.W., Kamau,E., Otieno,J.R., Mwema,A., Lewa,C., Agoti,C.N. and Nokes,J.D.
EPI_ISL_2594941	Pathogen Diagnostic Center, Institut Pasteur of Shanghai	Pathogen Diagnostic Center, Institut Pasteur of Shanghai	Liu,J., Mu,Y., Dong,W., Yao,F., Yan,H., Lan,K. and Zhang,C.
EPI_ISL_2594942, EPI_ISL_2594943	WHO WPRO Measles Regional Reference Lab, Key Laboratory of Medical Virology Ministry of Health, National Institute for Viral Disease Control and Prevention, China Center for Disease Control and Prevention	WHO WPRO Measles Regional Reference Lab, Key Laboratory of Medical Virology Ministry of Health, National Institute for Viral Disease Control and Prevention, China Center for Disease Control and Prevention	Zhang,Y., Song,J. and Xu,W.
EPI_ISL_2594944	Laboratory Medicine and Pathobiology, University of Toronto	Laboratory Medicine and Pathobiology, University of Toronto	Granados,A., Duvvuri,V., Rosenfeld,P., Eshaghi,A. and Gubbay,J.B.
EPI_ISL_2594945	Department of Public Health Laboratory Sciences, West China School of Public Health, Sichuan University	Department of Public Health Laboratory Sciences, West China School of Public Health, Sichuan University	Hu,W.P. and Pei,F.X.
EPI_ISL_2594946	Department of Epidemiology and Demography, Kenya Medical Research Institute (KEMRI) - Wellcome Trust Research Programme	Department of Epidemiology and Demography, Kenya Medical Research Institute (KEMRI) - Wellcome Trust Research Programme	Otieno,J.R., Agoti,C.N., Gitahi,C.W., Bett,A., Ngama,M., Medley,G.F., Cane,P.A. and Nokes,J.D.
EPI_ISL_2594947	Epidemiology and Demography, Kenya Medical Research Institute (KEMRI) - Wellcome Trust Research Programme	Epidemiology and Demography, Kenya Medical Research Institute (KEMRI) - Wellcome Trust Research Programme	Agoti,C.N., Otieno,J.R., Gitahi,C.W., Cane,P.A. and Nokes,D.J.
EPI_ISL_2594948	WHO WPRO Measles Regional Reference Lab, Key Laboratory of Medical Virology Ministry of Health, National Institute for Viral Disease Control and Prevention, China Center for Disease Control and Prevention	WHO WPRO Measles Regional Reference Lab, Key Laboratory of Medical Virology Ministry of Health, National Institute for Viral Disease Control and Prevention, China Center for Disease Control and Prevention	Zhang,Y., Song,J. and Xu,W.
EPI_ISL_2594949	Department of Epidemiology and Demography, Kenya Medical Research Institute (KEMRI) - Wellcome Trust Research Programme	Department of Epidemiology and Demography, Kenya Medical Research Institute (KEMRI) - Wellcome Trust Research Programme	Otieno,J.R., Agoti,C.N., Gitahi,C.W., Bett,A., Ngama,M., Medley,G.F., Cane,P.A. and Nokes,J.D.
EPI_ISL_2594950	Epidemiology and Demography, Kenya Medical Research Institute (KEMRI) - Wellcome Trust Research Programme	Epidemiology and Demography, Kenya Medical Research Institute (KEMRI) - Wellcome Trust Research Programme	Agoti,C.N., Otieno,J.R., Gitahi,C.W., Cane,P.A. and Nokes,D.J.
EPI_ISL_2594951	Department of Protein Engineering, Latvian Biomedical Research and Study Centre	Department of Protein Engineering, Latvian Biomedical Research and Study Centre	Balmaks,R., Ribakova,I., Gardovska,D. and Kazaks,A.

EPI_ISL_2594952	Department for Infectious Diseases Virology, University of Heidelberg	Department for Infectious Diseases Virology, University of Heidelberg	Tabatabai,J., Thielen,A., Lehners,N., Daeumer,M. and Schnitzler,P.
EPI_ISL_2594953	Epidemiology and Demography, KEMRI - Wellcome Trust Research Programme, Centre for Geographic Medicine Research	Epidemiology and Demography, KEMRI - Wellcome Trust Research Programme, Centre for Geographic Medicine Research	Oketch,J.W., Kamau,E., Otieno,J.R., Mwema,A., Lewa,C., Agoti,C.N. and Nokes,J.D.
EPI_ISL_2594954	Department of Respiratory Medicine, Children's Hospital of Chongqing Medical University	Department of Respiratory Medicine, Children's Hospital of Chongqing Medical University	Ren,L. and Liu,E.
EPI_ISL_2594955	Minoru Nidaira Okinawa Prefectural Institute of Health and Environment, Department of Biological Science	Minoru Nidaira Okinawa Prefectural Institute of Health and Environment, Department of Biological Science	Nidaira,M. and Taira,K.
EPI_ISL_2594956	Department of Pediatrics, Center of Excellence in Clinical Virology, Faculty of Medicine, Chulalongkorn University	Department of Pediatrics, Center of Excellence in Clinical Virology, Faculty of Medicine, Chulalongkorn University	Thongpan,I., Mauleekoonphairoj,J., Vichiwattana,P., Korkong,S., Vongpunsawad,S. and Poovorawan,Y.
EPI_ISL_2594957	Epidemiology and Demography, KEMRI - Wellcome Trust Research Programme, Centre for Geographic Medicine Research	Epidemiology and Demography, KEMRI - Wellcome Trust Research Programme, Centre for Geographic Medicine Research	Oketch,J.W., Kamau,E., Otieno,J.R., Mwema,A., Lewa,C., Agoti,C.N. and Nokes,J.D.
EPI_ISL_2594958	Pathogen Diagnostic Center, Institut Pasteur of Shanghai	Pathogen Diagnostic Center, Institut Pasteur of Shanghai	Liu,J., Mu,Y., Dong,W., Yao,F., Yan,H., Lan,K. and Zhang,C.
EPI_ISL_2594959	Centre for Research And Knowledge Transfer in Biotechnology, University of Zagreb	Centre for Research And Knowledge Transfer in Biotechnology, University of Zagreb	Ivancic-Jelecki,J., Slovic,A., Ljubin-Sternak,S., Mlinaric-Galinovic,G. and Forcic,D.
EPI_ISL_2594960	Epidemiology and Demography, KEMRI - Wellcome Trust Research Programme, Centre for Geographic Medicine Research	Epidemiology and Demography, KEMRI - Wellcome Trust Research Programme, Centre for Geographic Medicine Research	Oketch,J.W., Kamau,E., Otieno,J.R., Mwema,A., Lewa,C., Agoti,C.N. and Nokes,J.D.
EPI_ISL_2594961	Department of Epidemiology and Demography, Kenya Medical Research Institute (KEMRI) - Wellcome Trust Research Programme	Department of Epidemiology and Demography, Kenya Medical Research Institute (KEMRI) - Wellcome Trust Research Programme	Otieno,J.R., Agoti,C.N., Gitahi,C.W., Bett,A., Ngama,M., Medley,G.F., Cane,P.A. and Nokes,J.D.
EPI_ISL_2594962	Epidemiology and Demography, Kenya Medical Research Institute (KEMRI) - Wellcome Trust Research Programme	Epidemiology and Demography, Kenya Medical Research Institute (KEMRI) - Wellcome Trust Research Programme	Agoti,C.N., Otieno,J.R., Gitahi,C.W., Cane,P.A. and Nokes,D.J.
EPI_ISL_2594963, EPI_ISL_2594964, EPI_ISL_2594965, EPI_ISL_2594966	Epidemiology and Demography, KEMRI - Wellcome Trust Research Programme, Centre for Geographic Medicine Research	Epidemiology and Demography, KEMRI - Wellcome Trust Research Programme, Centre for Geographic Medicine Research	Oketch,J.W., Kamau,E., Otieno,J.R., Mwema,A., Lewa,C., Agoti,C.N. and Nokes,J.D.
EPI_ISL_2594967	WHO WPRO Measles Regional Reference Lab, Key Laboratory of Medical Virology Ministry of Health, National Institute for Viral Disease Control and Prevention, China Center for Disease Control and Prevention	WHO WPRO Measles Regional Reference Lab, Key Laboratory of Medical Virology Ministry of Health, National Institute for Viral Disease Control and Prevention, China Center for Disease Control and Prevention	Zhang,Y., Song,J. and Xu,W.
EPI_ISL_2594968	Department of Epidemiology and Demography, Kenya Medical Research Institute (KEMRI) - Wellcome Trust Research Programme	Department of Epidemiology and Demography, Kenya Medical Research Institute (KEMRI) - Wellcome Trust Research Programme	Otieno,J.R., Agoti,C.N., Gitahi,C.W., Bett,A., Ngama,M., Medley,G.F., Cane,P.A. and Nokes,J.D.
EPI_ISL_2594969	Epidemiology and Demography, Kenya Medical Research Institute (KEMRI) - Wellcome Trust Research Programme	Epidemiology and Demography, Kenya Medical Research Institute (KEMRI) - Wellcome Trust Research Programme	Agoti,C.N., Otieno,J.R., Gitahi,C.W., Cane,P.A. and Nokes,D.J.
EPI_ISL_2594970	Centre for Research And Knowledge Transfer in Biotechnology, University of Zagreb	Centre for Research And Knowledge Transfer in Biotechnology, University of Zagreb	Ivancic-Jelecki,J., Slovic,A., Ljubin-Sternak,S., Mlinaric-Galinovic,G. and Forcic,D.
EPI_ISL_2594971	Department of Epidemiology and Demography, Kenya Medical Research Institute (KEMRI) - Wellcome Trust Research Programme	Department of Epidemiology and Demography, Kenya Medical Research Institute (KEMRI) - Wellcome Trust Research Programme	Otieno,J.R., Agoti,C.N., Gitahi,C.W., Bett,A., Ngama,M., Medley,G.F., Cane,P.A. and Nokes,J.D.
EPI_ISL_2594972	Epidemiology and Demography, KEMRI - Wellcome Trust Research Programme, Centre for Geographic Medicine Research	Epidemiology and Demography, KEMRI - Wellcome Trust Research Programme, Centre for Geographic Medicine Research	Oketch,J.W., Kamau,E., Otieno,J.R., Mwema,A., Lewa,C., Agoti,C.N. and Nokes,J.D.
EPI_ISL_2594973	Epidemiology and Demography, Kenya Medical Research Institute (KEMRI) - Wellcome Trust Research Programme	Epidemiology and Demography, Kenya Medical Research Institute (KEMRI) - Wellcome Trust Research Programme	Agoti,C.N., Otieno,J.R., Gitahi,C.W., Cane,P.A. and Nokes,D.J.
EPI_ISL_2594974	Department of Epidemiology and Demography, Kenya Medical Research Institute (KEMRI) - Wellcome Trust Research Programme	Department of Epidemiology and Demography, Kenya Medical Research Institute (KEMRI) - Wellcome Trust Research Programme	Otieno,J.R., Agoti,C.N., Gitahi,C.W., Bett,A., Ngama,M., Medley,G.F., Cane,P.A. and Nokes,J.D.
EPI_ISL_2594975	Epidemiology and Demography, Kenya Medical Research Institute (KEMRI) - Wellcome Trust Research Programme	Epidemiology and Demography, Kenya Medical Research Institute (KEMRI) - Wellcome Trust Research Programme	Agoti,C.N., Otieno,J.R., Gitahi,C.W., Cane,P.A. and Nokes,D.J.
EPI_ISL_2594976	Department of Protein Engineering, Latvian Biomedical Research and Study Centre	Department of Protein Engineering, Latvian Biomedical Research and Study Centre	Balmaks,R., Ribakova,I., Gardovska,D. and Kazaks,A.
EPI_ISL_2594977	Department of Epidemiology and Demography, Kenya Medical Research Institute (KEMRI) - Wellcome Trust Research Programme	Department of Epidemiology and Demography, Kenya Medical Research Institute (KEMRI) - Wellcome Trust Research Programme	Otieno,J.R., Agoti,C.N., Gitahi,C.W., Bett,A., Ngama,M., Medley,G.F., Cane,P.A. and Nokes,J.D.
EPI_ISL_2594978, EPI_ISL_2594979	Epidemiology and Demography, KEMRI - Wellcome Trust Research Programme, Centre for Geographic Medicine Research	Epidemiology and Demography, KEMRI - Wellcome Trust Research Programme, Centre for Geographic Medicine Research	Oketch,J.W., Kamau,E., Otieno,J.R., Mwema,A., Lewa,C., Agoti,C.N. and Nokes,J.D.
EPI_ISL_2594980	Epidemiology and Demography, Kenya Medical Research Institute (KEMRI) - Wellcome Trust Research Programme	Epidemiology and Demography, Kenya Medical Research Institute (KEMRI) - Wellcome Trust Research Programme	Agoti,C.N., Otieno,J.R., Gitahi,C.W., Cane,P.A. and Nokes,D.J.
EPI_ISL_2594981	Department of Epidemiology and Demography, Kenya Medical Research Institute (KEMRI) - Wellcome Trust Research Programme	Department of Epidemiology and Demography, Kenya Medical Research Institute (KEMRI) - Wellcome Trust Research Programme	Otieno,J.R., Agoti,C.N., Gitahi,C.W., Bett,A., Ngama,M., Medley,G.F., Cane,P.A. and Nokes,J.D.
EPI_ISL_2594982	Epidemiology and Demography, Kenya Medical Research Institute (KEMRI) - Wellcome Trust Research Programme	Epidemiology and Demography, Kenya Medical Research Institute (KEMRI) - Wellcome Trust Research Programme	Agoti,C.N., Otieno,J.R., Gitahi,C.W., Cane,P.A. and Nokes,D.J.
EPI_ISL_2594983	Department of Protein Engineering, Latvian Biomedical Research and Study Centre	Department of Protein Engineering, Latvian Biomedical Research and Study Centre	Balmaks,R., Ribakova,I., Gardovska,D. and Kazaks,A.
EPI_ISL_2594984, EPI_ISL_2594985	Epidemiology and Demography, KEMRI - Wellcome Trust Research Programme, Centre for Geographic Medicine Research	Epidemiology and Demography, KEMRI - Wellcome Trust Research Programme, Centre for Geographic Medicine Research	Oketch,J.W., Kamau,E., Otieno,J.R., Mwema,A., Lewa,C., Agoti,C.N. and Nokes,J.D.
EPI_ISL_2594986	Laboratory Medicine and Pathobiology, University of Toronto	Laboratory Medicine and Pathobiology, University of Toronto	Granados,A., Duvvuri,V., Rosenfeld,P., Eshaghi,A. and Gubbay,J.B.
EPI_ISL_2594987	Department of Epidemiology and Demography, Kenya Medical Research Institute (KEMRI) - Wellcome Trust Research Programme	Department of Epidemiology and Demography, Kenya Medical Research Institute (KEMRI) - Wellcome Trust Research Programme	Otieno,J.R., Agoti,C.N., Gitahi,C.W., Bett,A., Ngama,M., Medley,G.F., Cane,P.A. and Nokes,J.D.
EPI_ISL_2594988	Epidemiology and Demography, Kenya Medical Research Institute (KEMRI) - Wellcome Trust Research Programme	Epidemiology and Demography, Kenya Medical Research Institute (KEMRI) - Wellcome Trust Research Programme	Agoti,C.N., Otieno,J.R., Gitahi,C.W., Cane,P.A. and Nokes,D.J.
EPI_ISL_2594989	Epidemiology and Demography, KEMRI - Wellcome Trust Research Programme, Centre for Geographic Medicine Research	Epidemiology and Demography, KEMRI - Wellcome Trust Research Programme, Centre for Geographic Medicine Research	Oketch,J.W., Kamau,E., Otieno,J.R., Mwema,A., Lewa,C., Agoti,C.N. and Nokes,J.D.
EPI_ISL_2594990	Laboratory Medicine and Pathobiology, University of Toronto	Laboratory Medicine and Pathobiology, University of Toronto	Granados,A., Duvvuri,V., Rosenfeld,P., Eshaghi,A. and Gubbay,J.B.
EPI_ISL_2594991	Epidemiology and Demography, KEMRI - Wellcome Trust Research Programme, Centre for Geographic Medicine Research	Epidemiology and Demography, KEMRI - Wellcome Trust Research Programme, Centre for Geographic Medicine Research	Oketch,J.W., Kamau,E., Otieno,J.R., Mwema,A., Lewa,C., Agoti,C.N. and Nokes,J.D.
EPI_ISL_2594992, EPI_ISL_2594993, EPI_ISL_2594994	Department of Paediatric Infectious Diseases, Institute of Tropical Medicine, Nagasaki University	Department of Paediatric Infectious Diseases, Institute of Tropical Medicine, Nagasaki University	Yoshihara,K., Nhat Le,M., Nagasawa,K., Tsukagoshi,H., Nguyen,H.A., Toizumi,M., Moriuchi,H., Hashizume,M., Ariyoshi,K., Dang,D.A., Kimura,H. and Yoshida,L.-M.
EPI_ISL_2594995	Minoru Nidaira Okinawa Prefectural Institute of Health and Environment, Department of Biological Science	Minoru Nidaira Okinawa Prefectural Institute of Health and Environment, Department of Biological Science	Nidaira,M. and Taira,K.
EPI_ISL_2594996	Department of Pediatrics, Center of Excellence in Clinical Virology, Faculty of Medicine, Chulalongkorn University	Department of Pediatrics, Center of Excellence in Clinical Virology, Faculty of Medicine, Chulalongkorn University	Thongpan,I., Mauleekoonphairoj,J., Vichiwattana,P., Korkong,S., Vongpunsawad,S. and Poovorawan,Y.
EPI_ISL_2594997	Department of Protein Engineering, Latvian Biomedical Research and Study Centre	Department of Protein Engineering, Latvian Biomedical Research and Study Centre	Balmaks,R., Ribakova,I., Gardovska,D. and Kazaks,A.
EPI_ISL_2594998	Epidemiology and Demography, KEMRI - Wellcome Trust Research Programme, Centre for Geographic Medicine Research	Epidemiology and Demography, KEMRI - Wellcome Trust Research Programme, Centre for Geographic Medicine Research	Oketch,J.W., Kamau,E., Otieno,J.R., Mwema,A., Lewa,C., Agoti,C.N. and Nokes,J.D.
EPI_ISL_2594999	WHO WPRO Measles Regional Reference Lab, Key Laboratory of Medical Virology Ministry of Health, National Institute for Viral Disease Control and Prevention, China Center for Disease Control and Prevention	WHO WPRO Measles Regional Reference Lab, Key Laboratory of Medical Virology Ministry of Health, National Institute for Viral Disease Control and Prevention, China Center for Disease Control and Prevention	Zhang,Y., Song,J. and Xu,W.
EPI_ISL_2595000	Microbiology Department, Virology Division, College of Medicine, Taif University	Microbiology Department, Virology Division, College of Medicine, Taif University	Al Aboud,D.M., Al Aboud,N.M., Al-Malky,M.I.R. and Abdel-Moneim,A.S.
EPI_ISL_2595001, EPI_ISL_2595002, EPI_ISL_2595003, EPI_ISL_2595004, EPI_ISL_2595005, EPI_ISL_2595006, EPI_ISL_2595007, EPI_ISL_2595008, EPI_ISL_2595009, EPI_ISL_2595010, EPI_ISL_2595011, EPI_ISL_2595012, EPI_ISL_2595013, EPI_ISL_2595014, EPI_ISL_2595015, EPI_ISL_2595016			
see above	Epidemiology and Demography, KEMRI - Wellcome Trust Research Programme, Centre for Geographic Medicine Research	Epidemiology and Demography, KEMRI - Wellcome Trust Research Programme, Centre for Geographic Medicine Research	Oketch,J.W., Kamau,E., Otieno,J.R., Mwema,A., Lewa,C., Agoti,C.N. and Nokes,J.D.
EPI_ISL_2595017	Department of Paediatric Infectious Diseases, Institute of Tropical Medicine, Nagasaki University	Department of Paediatric Infectious Diseases, Institute of Tropical Medicine, Nagasaki University	Yoshihara,K., Nhat Le,M., Nagasawa,K., Tsukagoshi,H., Nguyen,H.A., Toizumi,M., Moriuchi,H., Hashizume,M., Ariyoshi,K., Dang,D.A., Kimura,H. and Yoshida,L.-M.
EPI_ISL_2595018, EPI_ISL_2595019	Minoru Nidaira Okinawa Prefectural Institute of Health and Environment, Department of Biological Science	Minoru Nidaira Okinawa Prefectural Institute of Health and Environment, Department of Biological Science	Nidaira,M. and Taira,K.
EPI_ISL_2595020, EPI_ISL_2595021, EPI_ISL_2595022	Department of Pediatrics, Center of Excellence in Clinical Virology, Faculty of Medicine, Chulalongkorn University	Department of Pediatrics, Center of Excellence in Clinical Virology, Faculty of Medicine, Chulalongkorn University	Thongpan,I., Mauleekoonphairoj,J., Vichiwattana,P., Korkong,S., Vongpunsawad,S. and Poovorawan,Y.
EPI_ISL_2595023, EPI_ISL_2595024, EPI_ISL_2595025, EPI_ISL_2595026, EPI_ISL_2595027, EPI_ISL_2595028, EPI_ISL_2595029, EPI_ISL_2595030	Epidemiology and Demography, KEMRI - Wellcome Trust Research Programme, Centre for Geographic Medicine Research	Epidemiology and Demography, KEMRI - Wellcome Trust Research Programme, Centre for Geographic Medicine Research	Oketch,J.W., Kamau,E., Otieno,J.R., Mwema,A., Lewa,C., Agoti,C.N. and Nokes,J.D.
EPI_ISL_2595031	Department of Epidemiology and Demography, Kenya Medical Research Institute	Department of Epidemiology and Demography, Kenya Medical Research Institute	Otieno,J.R., Agoti,C.N., Gitahi,C.W., Bett,A., Ngama,M., Medley,G.F., Cane,P.A. and Nokes,J.D.

	(KEMRI) - Wellcome Trust Research Programme	(KEMRI) - Wellcome Trust Research Programme	
EPI_ISL_2595032	Epidemiology and Demography, Kenya Medical Research Institute (KEMRI) - Wellcome Trust Research Programme	Epidemiology and Demography, Kenya Medical Research Institute (KEMRI) - Wellcome Trust Research Programme	Agoti,C.N., Otieno,J.R., Gitahi,C.W., Cane,P.A. and Nokes,D.J.
EPI_ISL_2595033, EPI_ISL_2595034, EPI_ISL_2595035, EPI_ISL_2595036, EPI_ISL_2595037, EPI_ISL_2595038	Epidemiology and Demography, KEMRI - Wellcome Trust Research Programme, Centre for Geographic Medicine Research	Epidemiology and Demography, KEMRI - Wellcome Trust Research Programme, Centre for Geographic Medicine Research	Oketch,J.W., Kamau,E., Otieno,J.R., Mwema,A., Lewa,C., Agoti,C.N. and Nokes,J.D.
EPI_ISL_2595039	Department of Epidemiology and Demography, Kenya Medical Research Institute (KEMRI) - Wellcome Trust Research Programme	Department of Epidemiology and Demography, Kenya Medical Research Institute (KEMRI) - Wellcome Trust Research Programme	Otieno,J.R., Agoti,C.N., Gitahi,C.W., Bett,A., Ngama,M., Medley,G.F., Cane,P.A. and Nokes,J.D.
EPI_ISL_2595040	Epidemiology and Demography, Kenya Medical Research Institute (KEMRI) - Wellcome Trust Research Programme	Epidemiology and Demography, Kenya Medical Research Institute (KEMRI) - Wellcome Trust Research Programme	Agoti,C.N., Otieno,J.R., Gitahi,C.W., Cane,P.A. and Nokes,D.J.
EPI_ISL_2595041	Department of Paediatric Infectious Diseases, Institute of Tropical Medicine, Nagasaki University	Department of Paediatric Infectious Diseases, Institute of Tropical Medicine, Nagasaki University	Yoshihara,K., Nhat Le,M., Nagasawa,K., Tsukagoshi,H., Nguyen,H.A., Toizumi,M., Moriuchi,H., Hashizume,M., Ariyoshi,K., Dang,D.A., Kimura,H. and Yoshida,L.-M.
EPI_ISL_2595042, EPI_ISL_2595043, EPI_ISL_2595044	Minoru Nidaira Okinawa Prefectural Institute of Health and Environment, Department of Biological Science	Minoru Nidaira Okinawa Prefectural Institute of Health and Environment, Department of Biological Science	Nidaira,M. and Taira,K.
EPI_ISL_2595045, EPI_ISL_2595046, EPI_ISL_2595047, EPI_ISL_2595048, EPI_ISL_2595049	Department of Pediatrics, Center of Excellence in Clinical Virology, Faculty of Medicine, Chulalongkorn University	Department of Pediatrics, Center of Excellence in Clinical Virology, Faculty of Medicine, Chulalongkorn University	Thongpan,I., Mauleekoonphairoj,J., Vichiwattana,P., Korkong,S., Vongpunsawad,S. and Poovorawan,Y.
EPI_ISL_2595050, EPI_ISL_2595051, EPI_ISL_2595052, EPI_ISL_2595053, EPI_ISL_2595054	Epidemiology and Demography, KEMRI - Wellcome Trust Research Programme, Centre for Geographic Medicine Research	Epidemiology and Demography, KEMRI - Wellcome Trust Research Programme, Centre for Geographic Medicine Research	Oketch,J.W., Kamau,E., Otieno,J.R., Mwema,A., Lewa,C., Agoti,C.N. and Nokes,J.D.
EPI_ISL_2595055	Investigacion en Virologia y Biotecnologia, Instituto Conmemorativo Gorgas de Estudios de la Salud	Investigacion en Virologia y Biotecnologia, Instituto Conmemorativo Gorgas de Estudios de la Salud	Abrego,L., Delfraro,A., Franco,D., Castillo,C., Cano,M., Castillo,M., Castillo,J., Pascale,J. and Arbiza,J.
EPI_ISL_2595056, EPI_ISL_2595057, EPI_ISL_2595058, EPI_ISL_2595059, EPI_ISL_2595060, EPI_ISL_2595061, EPI_ISL_2595062, EPI_ISL_2595063	Epidemiology and Demography, KEMRI - Wellcome Trust Research Programme, Centre for Geographic Medicine Research	Epidemiology and Demography, KEMRI - Wellcome Trust Research Programme, Centre for Geographic Medicine Research	Oketch,J.W., Kamau,E., Otieno,J.R., Mwema,A., Lewa,C., Agoti,C.N. and Nokes,J.D.
EPI_ISL_2595064	Manipal Centre for Virus Research, Manipal University	Manipal Centre for Virus Research, Manipal University	Anjali,A., Aswathy,R., Akhil,C., Giselle,D., Revti,B., Hindol,M., Suresh,P. and Arunkumar,G.
EPI_ISL_2595065, EPI_ISL_2595066, EPI_ISL_2595067	Epidemiology and Demography, KEMRI - Wellcome Trust Research Programme, Centre for Geographic Medicine Research	Epidemiology and Demography, KEMRI - Wellcome Trust Research Programme, Centre for Geographic Medicine Research	Oketch,J.W., Kamau,E., Otieno,J.R., Mwema,A., Lewa,C., Agoti,C.N. and Nokes,J.D.
EPI_ISL_2595068	Manipal Centre for Virus Research, Manipal University	Manipal Centre for Virus Research, Manipal University	Anjali,A., Aswathy,R., Akhil,C., Giselle,D., Revti,B., Hindol,M., Suresh,P. and Arunkumar,G.
EPI_ISL_2595069, EPI_ISL_2595070, EPI_ISL_2595071, EPI_ISL_2595072	Epidemiology and Demography, KEMRI - Wellcome Trust Research Programme, Centre for Geographic Medicine Research	Epidemiology and Demography, KEMRI - Wellcome Trust Research Programme, Centre for Geographic Medicine Research	Oketch,J.W., Kamau,E., Otieno,J.R., Mwema,A., Lewa,C., Agoti,C.N. and Nokes,J.D.
EPI_ISL_2595073	Virology, Tohoku University Graduate School of Medicine	Virology, Tohoku University Graduate School of Medicine	Malasao,R., Okamoto,M., Chaimongkol,N., Imamura,T., Tohma,K., Dapat,I., Dapat,C., Suzuki,A., Saito,M., Saito,M., Tamaki,R., Segubre-Mercado,E., Igoy,M.A.U., Lupisan,S., Olveda,R. and Oshitani,H.
EPI_ISL_2595074, EPI_ISL_2595075, EPI_ISL_2595076, EPI_ISL_2595077, EPI_ISL_2595078, EPI_ISL_2595079, EPI_ISL_2595080, EPI_ISL_2595081, EPI_ISL_2595082	Department of Paediatric Infectious Diseases, Institute of Tropical Medicine, Nagasaki University	Department of Paediatric Infectious Diseases, Institute of Tropical Medicine, Nagasaki University	Yoshihara,K., Nhat Le,M., Nagasawa,K., Tsukagoshi,H., Nguyen,H.A., Toizumi,M., Moriuchi,H., Hashizume,M., Ariyoshi,K., Dang,D.A., Kimura,H. and Yoshida,L.-M.
EPI_ISL_2595083, EPI_ISL_2595084, EPI_ISL_2595085, EPI_ISL_2595086, EPI_ISL_2595087, EPI_ISL_2595088, EPI_ISL_2595089, EPI_ISL_2595090, EPI_ISL_2595091, EPI_ISL_2595092, EPI_ISL_2595093, EPI_ISL_2595094, EPI_ISL_2595095, EPI_ISL_2595096, EPI_ISL_2595097, EPI_ISL_2595098			
see above	Department of Pediatrics, Center of Excellence in Clinical Virology, Faculty of Medicine, Chulalongkorn University	Department of Pediatrics, Center of Excellence in Clinical Virology, Faculty of Medicine, Chulalongkorn University	Thongpan,I., Mauleekoonphairoj,J., Vichiwattana,P., Korkong,S., Vongpunsawad,S. and Poovorawan,Y.
EPI_ISL_2595099	Manipal Centre for Virus Research, Manipal University	Manipal Centre for Virus Research, Manipal University	Anjali,A., Aswathy,R., Akhil,C., Giselle,D., Revti,B., Hindol,M., Suresh,P. and Arunkumar,G.
EPI_ISL_2595100, EPI_ISL_2595101, EPI_ISL_2595102, EPI_ISL_2595103, EPI_ISL_2595104	Epidemiology and Demography, KEMRI - Wellcome Trust Research Programme, Centre for Geographic Medicine Research	Epidemiology and Demography, KEMRI - Wellcome Trust Research Programme, Centre for Geographic Medicine Research	Oketch,J.W., Kamau,E., Otieno,J.R., Mwema,A., Lewa,C., Agoti,C.N. and Nokes,J.D.
EPI_ISL_2595105	Influenza Group, National Institute of Virology	Influenza Group, National Institute of Virology	Choudhary,M.L., Anand,S.P., Wadhwa,B.S. and Chadha,M.S.
EPI_ISL_2595106	Manipal Centre for Virus Research, Manipal University	Manipal Centre for Virus Research, Manipal University	Anjali,A., Aswathy,R., Akhil,C., Giselle,D., Revti,B., Hindol,M., Suresh,P. and Arunkumar,G.
EPI_ISL_2595107	Epidemiology and Demography, KEMRI - Wellcome Trust Research Programme, Centre for Geographic Medicine Research	Epidemiology and Demography, KEMRI - Wellcome Trust Research Programme, Centre for Geographic Medicine Research	Oketch,J.W., Kamau,E., Otieno,J.R., Mwema,A., Lewa,C., Agoti,C.N. and Nokes,J.D.
EPI_ISL_2595108	Department of Pediatrics, Center of Excellence in Clinical Virology, Faculty of Medicine, Chulalongkorn University	Department of Pediatrics, Center of Excellence in Clinical Virology, Faculty of Medicine, Chulalongkorn University	Thongpan,I., Mauleekoonphairoj,J., Vichiwattana,P., Korkong,S., Vongpunsawad,S. and Poovorawan,Y.
EPI_ISL_2595109, EPI_ISL_2595110	Manipal Centre for Virus Research, Manipal University	Manipal Centre for Virus Research, Manipal University	Anjali,A., Aswathy,R., Akhil,C., Giselle,D., Revti,B., Hindol,M., Suresh,P. and Arunkumar,G.
EPI_ISL_2595111, EPI_ISL_2595112, EPI_ISL_2595113, EPI_ISL_2595114	Epidemiology and Demography, KEMRI - Wellcome Trust Research Programme, Centre for Geographic Medicine Research	Epidemiology and Demography, KEMRI - Wellcome Trust Research Programme, Centre for Geographic Medicine Research	Oketch,J.W., Kamau,E., Otieno,J.R., Mwema,A., Lewa,C., Agoti,C.N. and Nokes,J.D.
EPI_ISL_2595115	Manipal Centre for Virus Research, Manipal University	Manipal Centre for Virus Research, Manipal University	Anjali,A., Aswathy,R., Akhil,C., Giselle,D., Revti,B., Hindol,M., Suresh,P. and Arunkumar,G.
EPI_ISL_2595116	Virology, Tohoku University Graduate School of Medicine	Virology, Tohoku University Graduate School of Medicine	Malasao,R., Okamoto,M., Chaimongkol,N., Imamura,T., Tohma,K., Dapat,I., Dapat,C., Suzuki,A., Saito,M., Saito,M., Tamaki,R., Segubre-Mercado,E., Igoy,M.A.U., Lupisan,S., Olveda,R. and Oshitani,H.
EPI_ISL_2595117, EPI_ISL_2595118, EPI_ISL_2595119	Department of Paediatric Infectious Diseases, Institute of Tropical Medicine, Nagasaki University	Department of Paediatric Infectious Diseases, Institute of Tropical Medicine, Nagasaki University	Yoshihara,K., Nhat Le,M., Nagasawa,K., Tsukagoshi,H., Nguyen,H.A., Toizumi,M., Moriuchi,H., Hashizume,M., Ariyoshi,K., Dang,D.A., Kimura,H. and Yoshida,L.-M.
EPI_ISL_2595120, EPI_ISL_2595121, EPI_ISL_2595122, EPI_ISL_2595123, EPI_ISL_2595124, EPI_ISL_2595125, EPI_ISL_2595126, EPI_ISL_2595127, EPI_ISL_2595128, EPI_ISL_2595129, EPI_ISL_2595130, EPI_ISL_2595131, EPI_ISL_2595132, EPI_ISL_2595133, EPI_ISL_2595134			
see above	Department of Pediatrics, Center of Excellence in Clinical Virology, Faculty of Medicine, Chulalongkorn University	Department of Pediatrics, Center of Excellence in Clinical Virology, Faculty of Medicine, Chulalongkorn University	Thongpan,I., Mauleekoonphairoj,J., Vichiwattana,P., Korkong,S., Vongpunsawad,S. and Poovorawan,Y.
EPI_ISL_2595135, EPI_ISL_2595136, EPI_ISL_2595137, EPI_ISL_2595138, EPI_ISL_2595139	Epidemiology and Demography, KEMRI - Wellcome Trust Research Programme, Centre for Geographic Medicine Research	Epidemiology and Demography, KEMRI - Wellcome Trust Research Programme, Centre for Geographic Medicine Research	Oketch,J.W., Kamau,E., Otieno,J.R., Mwema,A., Lewa,C., Agoti,C.N. and Nokes,J.D.
EPI_ISL_2595140	Virology Section, Department of Microbiology, King George's Medical University	Virology Section, Department of Microbiology, King George's Medical University	Singh,A.K., Jain,A., Jain,B., Dangi,T., Verma,A.K., Dwivedi,M. and Kushwaha,R.
EPI_ISL_2595141	Epidemiology and Demography, KEMRI - Wellcome Trust Research Programme, Centre for Geographic Medicine Research	Epidemiology and Demography, KEMRI - Wellcome Trust Research Programme, Centre for Geographic Medicine Research	Oketch,J.W., Kamau,E., Otieno,J.R., Mwema,A., Lewa,C., Agoti,C.N. and Nokes,J.D.
EPI_ISL_2595142	Virology, Tohoku University Graduate School of Medicine	Virology, Tohoku University Graduate School of Medicine	Malasao,R., Okamoto,M., Chaimongkol,N., Imamura,T., Tohma,K., Dapat,I., Dapat,C., Suzuki,A., Saito,M., Saito,M., Tamaki,R., Segubre-Mercado,E., Igoy,M.A.U., Lupisan,S., Olveda,R. and Oshitani,H.
EPI_ISL_2595143, EPI_ISL_2595144, EPI_ISL_2595145	Department of Paediatric Infectious Diseases, Institute of Tropical Medicine, Nagasaki University	Department of Paediatric Infectious Diseases, Institute of Tropical Medicine, Nagasaki University	Yoshihara,K., Nhat Le,M., Nagasawa,K., Tsukagoshi,H., Nguyen,H.A., Toizumi,M., Moriuchi,H., Hashizume,M., Ariyoshi,K., Dang,D.A., Kimura,H. and Yoshida,L.-M.
EPI_ISL_2595146, EPI_ISL_2595147, EPI_ISL_2595148	Department of Pediatrics, Center of Excellence in Clinical Virology, Faculty of Medicine, Chulalongkorn University	Department of Pediatrics, Center of Excellence in Clinical Virology, Faculty of Medicine, Chulalongkorn University	Thongpan,I., Mauleekoonphairoj,J., Vichiwattana,P., Korkong,S., Vongpunsawad,S. and Poovorawan,Y.
EPI_ISL_2595149	Investigacion en Virologia y Biotecnologia, Instituto Conmemorativo Gorgas de Estudios de la Salud	Investigacion en Virologia y Biotecnologia, Instituto Conmemorativo Gorgas de Estudios de la Salud	Abrego,L., Delfraro,A., Franco,D., Castillo,C., Cano,M., Castillo,M., Castillo,J., Pascale,J. and Arbiza,J.
EPI_ISL_2595150	Department of Clinical Laboratory, Fujian Provincial Hospital	Department of Clinical Laboratory, Fujian Provincial Hospital	Su,Y., Wu,Y., Tian,R. and Liang,G.
EPI_ISL_2595151	Department of Epidemiology and Demography, Kenya Medical Research Institute (KEMRI) - Wellcome Trust Research Programme	Department of Epidemiology and Demography, Kenya Medical Research Institute (KEMRI) - Wellcome Trust Research Programme	Otieno,J.R., Agoti,C.N., Gitahi,C.W., Bett,A., Ngama,M., Medley,G.F., Cane,P.A. and Nokes,J.D.
EPI_ISL_2595152	Epidemiology and Demography, Kenya Medical Research Institute (KEMRI) - Wellcome Trust Research Programme	Epidemiology and Demography, Kenya Medical Research Institute (KEMRI) - Wellcome Trust Research Programme	Agoti,C.N., Otieno,J.R., Gitahi,C.W., Cane,P.A. and Nokes,D.J.
EPI_ISL_2595153	Department of Epidemiology and Demography, Kenya Medical Research Institute (KEMRI) - Wellcome Trust Research Programme	Department of Epidemiology and Demography, Kenya Medical Research Institute (KEMRI) - Wellcome Trust Research Programme	Otieno,J.R., Agoti,C.N., Gitahi,C.W., Bett,A., Ngama,M., Medley,G.F., Cane,P.A. and Nokes,J.D.
EPI_ISL_2595154	Epidemiology and Demography, Kenya Medical Research Institute (KEMRI) - Wellcome Trust Research Programme	Epidemiology and Demography, Kenya Medical Research Institute (KEMRI) - Wellcome Trust Research Programme	Agoti,C.N., Otieno,J.R., Gitahi,C.W., Cane,P.A. and Nokes,D.J.
EPI_ISL_2595155	Virology Section, Department of Microbiology, King George's Medical University	Virology Section, Department of Microbiology, King George's Medical University	Singh,A.K., Jain,A., Jain,B., Dangi,T., Verma,A.K., Dwivedi,M. and Kushwaha,R.
EPI_ISL_2595156, EPI_ISL_2595157	Epidemiology and Demography, KEMRI - Wellcome Trust Research Programme, Centre for Geographic Medicine Research	Epidemiology and Demography, KEMRI - Wellcome Trust Research Programme, Centre for Geographic Medicine Research	Oketch,J.W., Kamau,E., Otieno,J.R., Mwema,A., Lewa,C., Agoti,C.N. and Nokes,J.D.
EPI_ISL_2595158	Pathogen Diagnostic Center, Institut Pasteur of Shanghai	Pathogen Diagnostic Center, Institut Pasteur of Shanghai	Liu,J., Mu,Y., Dong,W., Yao,F., Yan,H., Lan,K. and Zhang,C.
EPI_ISL_2595159, EPI_ISL_2595160	Center for Infectious Diseases, School of Public Health, University of Texas Health Science Center	Center for Infectious Diseases, School of Public Health, University of Texas Health Science Center	Bahl,J., Hixson,J., Kim,D.-K., Qiu,X., Piedra,P.A., Piedra,F.-A., Avadhanula,V. and Machado,A.A.
EPI_ISL_2595161	Epidemiology and Demography, KEMRI - Wellcome Trust Research Programme, Centre for Geographic Medicine Research	Epidemiology and Demography, KEMRI - Wellcome Trust Research Programme, Centre for Geographic Medicine Research	Oketch,J.W., Kamau,E., Otieno,J.R., Mwema,A., Lewa,C., Agoti,C.N. and Nokes,J.D.
EPI_ISL_2595162	Virology, School of Public Health, Tehran University of Medical Sciences	Virology, School of Public Health, Tehran University of Medical Sciences	Yavarian,J., Faghihloo,E. and Mokhtari Azad,T.
EPI_ISL_2595163, EPI_ISL_2595164, EPI_ISL_2595165, EPI_ISL_2595166, EPI_ISL_2595167, EPI_ISL_2595168	Virology, Tohoku University Graduate School of Medicine	Virology, Tohoku University Graduate School of Medicine	Malasao,R., Okamoto,M., Chaimongkol,N., Imamura,T., Tohma,K., Dapat,I., Dapat,C., Suzuki,A., Saito,M., Saito,M., Tamaki,R., Segubre-Mercado,E., Igoy,M.A.U., Lupisan,S., Olveda,R. and Oshitani,H.

EPI_ISL_2595169, EPI_ISL_2595170, EPI_ISL_2595171, EPI_ISL_2595172	Laboratory of Virology, Capital Institute of Pediatrics	Laboratory of Virology, Capital Institute of Pediatrics	Cui,G., Deng,J., Zhu,R., Qian,Y., Sun,Y., Zhao,L. and Wang,F.
EPI_ISL_2595173	Beijing Key Laboratory of Etiology of Viral Diseases in Children; Laboratory of Virology, Capital Institute of Pediatrics	Beijing Key Laboratory of Etiology of Viral Diseases in Children; Laboratory of Virology, Capital Institute of Pediatrics	Cui,G., Zhu,R., Deng,J., Zhao,L., Sun,Y., Wang,F. and Qian,Y.
EPI_ISL_2595174, EPI_ISL_2595175	Department of Pediatrics, Center of Excellence in Clinical Virology, Faculty of Medicine, Chulalongkorn University	Department of Pediatrics, Center of Excellence in Clinical Virology, Faculty of Medicine, Chulalongkorn University	Thongpan,I., Mauleekoonphairoj,J., Vichiwattana,P., Korkong,S., Vongpunsawad,S. and Poovorawan,Y.
EPI_ISL_2595176	Department of Clinical Laboratory, Fujian Provincial Hospital	Department of Clinical Laboratory, Fujian Provincial Hospital	Su,Y., Wu,Y., Tian,R. and Liang,G.
EPI_ISL_2595177, EPI_ISL_2595178, EPI_ISL_2595179, EPI_ISL_2595180	Epidemiology and Demography, KEMRI - Wellcome Trust Research Programme, Centre for Geographic Medicine Research	Epidemiology and Demography, KEMRI - Wellcome Trust Research Programme, Centre for Geographic Medicine Research	Oketch,J.W., Kamau,E., Otieno,J.R., Mwema,A., Lewa,C., Agoti,C.N. and Nokes,J.D.
EPI_ISL_2595181, EPI_ISL_2595182	Center for Infectious Diseases, School of Public Health, University of Texas Health Science Center	Center for Infectious Diseases, School of Public Health, University of Texas Health Science Center	Bahl,J., Hixson,J., Kim,D.-K., Qiu,X., Piedra,P.A., Piedra,F.-A., Avadhanula,V. and Machado,A.A.
EPI_ISL_2595183	Epidemiology and Demography, KEMRI - Wellcome Trust Research Programme, Centre for Geographic Medicine Research	Epidemiology and Demography, KEMRI - Wellcome Trust Research Programme, Centre for Geographic Medicine Research	Oketch,J.W., Kamau,E., Otieno,J.R., Mwema,A., Lewa,C., Agoti,C.N. and Nokes,J.D.
EPI_ISL_2595184	Department for Infectious Diseases Virology, University of Heidelberg	Department for Infectious Diseases Virology, University of Heidelberg	Tabatabai,J., Thielen,A., Lehners,N., Daeumer,M. and Schnitzler,P.
EPI_ISL_2595185, EPI_ISL_2595186, EPI_ISL_2595187	Epidemiology and Demography, KEMRI - Wellcome Trust Research Programme, Centre for Geographic Medicine Research	Epidemiology and Demography, KEMRI - Wellcome Trust Research Programme, Centre for Geographic Medicine Research	Oketch,J.W., Kamau,E., Otieno,J.R., Mwema,A., Lewa,C., Agoti,C.N. and Nokes,J.D.
EPI_ISL_2595188	Epidemiology and Demography Department, KEMRI-Wellcome Trust Research Programme	Epidemiology and Demography Department, KEMRI-Wellcome Trust Research Programme	Otieno,J.R., Kamau,E.M., Oketch,J.W., Ngoi,J.M., Agoti,C.N., Gichuki,A.M., Otieno,G.P., Ngama,M., Cane,P.A., Kellam,P., Cotten,M., Lemey,P. and Nokes,D.J.
EPI_ISL_2595189	Department of Public Health Laboratory Sciences, West China School of Public Health, Sichuan University	Department of Public Health Laboratory Sciences, West China School of Public Health, Sichuan University	Hu,W.P. and Pei,F.X.
EPI_ISL_2595190, EPI_ISL_2595191	Epidemiology and Demography, KEMRI - Wellcome Trust Research Programme, Centre for Geographic Medicine Research	Epidemiology and Demography, KEMRI - Wellcome Trust Research Programme, Centre for Geographic Medicine Research	Oketch,J.W., Kamau,E., Otieno,J.R., Mwema,A., Lewa,C., Agoti,C.N. and Nokes,J.D.
EPI_ISL_2595192	Epidemiology and Demography Department, KEMRI-Wellcome Trust Research Programme	Epidemiology and Demography Department, KEMRI-Wellcome Trust Research Programme	Otieno,J.R., Kamau,E.M., Oketch,J.W., Ngoi,J.M., Agoti,C.N., Gichuki,A.M., Otieno,G.P., Ngama,M., Cane,P.A., Kellam,P., Cotten,M., Lemey,P. and Nokes,D.J.
EPI_ISL_2595193, EPI_ISL_2595194, EPI_ISL_2595195	Epidemiology and Demography, KEMRI - Wellcome Trust Research Programme, Centre for Geographic Medicine Research	Epidemiology and Demography, KEMRI - Wellcome Trust Research Programme, Centre for Geographic Medicine Research	Oketch,J.W., Kamau,E., Otieno,J.R., Mwema,A., Lewa,C., Agoti,C.N. and Nokes,J.D.
EPI_ISL_2595196, EPI_ISL_2595197, EPI_ISL_2595198	Epidemiology and Demography Department, KEMRI-Wellcome Trust Research Programme	Epidemiology and Demography Department, KEMRI-Wellcome Trust Research Programme	Otieno,J.R., Kamau,E.M., Oketch,J.W., Ngoi,J.M., Agoti,C.N., Gichuki,A.M., Otieno,G.P., Ngama,M., Cane,P.A., Kellam,P., Cotten,M., Lemey,P. and Nokes,D.J.
EPI_ISL_2595199	Department of Epidemiology and Demography, Kenya Medical Research Institute (KEMRI) - Wellcome Trust Research Programme	Department of Epidemiology and Demography, Kenya Medical Research Institute (KEMRI) - Wellcome Trust Research Programme	Otieno,J.R., Agoti,C.N., Gitahi,C.W., Bett,A., Ngama,M., Medley,G.F., Cane,P.A. and Nokes,J.D.
EPI_ISL_2595200	Epidemiology and Demography, Kenya Medical Research Institute (KEMRI) - Wellcome Trust Research Programme	Epidemiology and Demography, Kenya Medical Research Institute (KEMRI) - Wellcome Trust Research Programme	Agoti,C.N., Otieno,J.R., Gitahi,C.W., Cane,P.A. and Nokes,D.J.
EPI_ISL_2595201	WHO WPRO Measles Regional Reference Lab, Key Laboratory of Medical Virology Ministry of Health, National Institute for Viral Disease Control and Prevention, China Center for Disease Control and Prevention	WHO WPRO Measles Regional Reference Lab, Key Laboratory of Medical Virology Ministry of Health, National Institute for Viral Disease Control and Prevention, China Center for Disease Control and Prevention	Zhang,Y., Song,J. and Xu,W.
EPI_ISL_2595202	Department of Epidemiology and Demography, Kenya Medical Research Institute (KEMRI) - Wellcome Trust Research Programme	Department of Epidemiology and Demography, Kenya Medical Research Institute (KEMRI) - Wellcome Trust Research Programme	Otieno,J.R., Agoti,C.N., Gitahi,C.W., Bett,A., Ngama,M., Medley,G.F., Cane,P.A. and Nokes,J.D.
EPI_ISL_2595203	Epidemiology and Demography, Kenya Medical Research Institute (KEMRI) - Wellcome Trust Research Programme	Epidemiology and Demography, Kenya Medical Research Institute (KEMRI) - Wellcome Trust Research Programme	Agoti,C.N., Otieno,J.R., Gitahi,C.W., Cane,P.A. and Nokes,D.J.
EPI_ISL_2595204	Epidemiology and Demography, KEMRI - Wellcome Trust Research Programme, Centre for Geographic Medicine Research	Epidemiology and Demography, KEMRI - Wellcome Trust Research Programme, Centre for Geographic Medicine Research	Oketch,J.W., Kamau,E., Otieno,J.R., Mwema,A., Lewa,C., Agoti,C.N. and Nokes,J.D.
EPI_ISL_2595205	Epidemiology and Demography Department, KEMRI-Wellcome Trust Research Programme	Epidemiology and Demography Department, KEMRI-Wellcome Trust Research Programme	Otieno,J.R., Kamau,E.M., Oketch,J.W., Ngoi,J.M., Agoti,C.N., Gichuki,A.M., Otieno,G.P., Ngama,M., Cane,P.A., Kellam,P., Cotten,M., Lemey,P. and Nokes,D.J.
EPI_ISL_2595206	Department of Epidemiology and Demography, Kenya Medical Research Institute (KEMRI) - Wellcome Trust Research Programme	Department of Epidemiology and Demography, Kenya Medical Research Institute (KEMRI) - Wellcome Trust Research Programme	Otieno,J.R., Agoti,C.N., Gitahi,C.W., Bett,A., Ngama,M., Medley,G.F., Cane,P.A. and Nokes,J.D.
EPI_ISL_2595207	Epidemiology and Demography, Kenya Medical Research Institute (KEMRI) - Wellcome Trust Research Programme	Epidemiology and Demography, Kenya Medical Research Institute (KEMRI) - Wellcome Trust Research Programme	Agoti,C.N., Otieno,J.R., Gitahi,C.W., Cane,P.A. and Nokes,D.J.
EPI_ISL_412905	Centro de Investigação em Saúde de Manhiça	Barcelona Institute of Global Health	Bassat, Q,, Tan,G., Pickett,B., Fedorova,N., Amedeo,P., Hu,L., Christensen,J., Miller,J., Durbin,A., Williams,T., Arumemi,F., Cadiz,C., Williams, T.
EPI_ISL_6494909, EPI_ISL_6494911, EPI_ISL_6494935, EPI_ISL_6494953, EPI_ISL_6494954, EPI_ISL_6494955, EPI_ISL_6494957, EPI_ISL_6494958, EPI_ISL_6494960, EPI_ISL_6494962, EPI_ISL_6494963, EPI_ISL_6494965			
see above	Respiratory Virus Unit, National Infection Service, Public Health England	National Infection Service, Public Health England	Zambon M, Talts T, Ellis J, Miah S, Platt S

We gratefully acknowledge the following Authors from the Originating laboratories responsible for obtaining the specimens, as well as the Submitting laboratories where the genome data were generated and shared via GISAID, on which this research is based.

All Submitters of data may be contacted directly via www.gisaid.org

Authors are sorted alphabetically.

Accession ID	Originating Laboratory	Submitting Laboratory	Authors
EPI_ISL_1074029, EPI_ISL_1074035, EPI_ISL_1074036, EPI_ISL_1074037, EPI_ISL_1074038, EPI_ISL_1074039, EPI_ISL_1074040, EPI_ISL_1074041, EPI_ISL_1074042, EPI_ISL_1074043, EPI_ISL_1074044, EPI_ISL_1074045, EPI_ISL_1074046, EPI_ISL_1074047, EPI_ISL_1074088, EPI_ISL_1074090, EPI_ISL_1074098, EPI_ISL_1074104, EPI_ISL_1074105, EPI_ISL_1074108, EPI_ISL_1074111, EPI_ISL_1074113, EPI_ISL_1074114, EPI_ISL_1074124, EPI_ISL_1074135, EPI_ISL_1074136, EPI_ISL_1074145, EPI_ISL_1074150, EPI_ISL_1074151, EPI_ISL_1074164, EPI_ISL_1074165, EPI_ISL_1074166, EPI_ISL_1074167, EPI_ISL_1074183, EPI_ISL_1074184, EPI_ISL_1074187, EPI_ISL_1074190, EPI_ISL_1074194, EPI_ISL_1074208, EPI_ISL_1074209, EPI_ISL_1074210, EPI_ISL_1074211, EPI_ISL_1074227, EPI_ISL_1074228, EPI_ISL_1074233, EPI_ISL_1074243, EPI_ISL_1074244, EPI_ISL_1074248, EPI_ISL_1074249, EPI_ISL_1074250, EPI_ISL_1074260, EPI_ISL_1074262, EPI_ISL_1074267, EPI_ISL_1074270, EPI_ISL_1074271, EPI_ISL_1074272			
see above	Virology Laboratory, Ricardo Gutiérrez Children's Hospital	Vanderbilt University Medical Center / Virology Laboratory, Ricardo Gutiérrez Children's Hospital	Goya, Stephanie; Lucion, Maria Florencia; Juarez, Maria del Valle; Shilts, Meghan; Gentile, Angela; Mistchenko, Alicia S.; Das, Suman# & Viegas, Mariana# (#contributed equally to this study)
EPI_ISL_15120665, EPI_ISL_15120666, EPI_ISL_15120667, EPI_ISL_15120668, EPI_ISL_15120669, EPI_ISL_15120670, EPI_ISL_15120671, EPI_ISL_15120672, EPI_ISL_15120673, EPI_ISL_15120674, EPI_ISL_15120675, EPI_ISL_15120676, EPI_ISL_15120677, EPI_ISL_15120678, EPI_ISL_15120679, EPI_ISL_15120680, EPI_ISL_15120681, EPI_ISL_15120682, EPI_ISL_15120683, EPI_ISL_15120688, EPI_ISL_15120689, EPI_ISL_15120690			
see above	National Institute of Hygiene, virology departement, National Influenza Center	Centers for Disease Control and Prevention, Coronavirus and Other Respiratory Viruses Division	Megha Aggarwal, Lijuan Wang, Ji In Park, Amanda Smith and Everardo Vega
EPI_ISL_15750115, EPI_ISL_15750120, EPI_ISL_15750121, EPI_ISL_15750122	Virology, Research Institute for Tropical Medicine	Virology, Research Institute for Tropical Medicine	Calaor-Morin,J., Arguelles,V.L., Foronda,J.L., Tan,A., Lagamayo,E., Dapat,C., Lupisan,S., Morin,J.C., Foronda,J.L.M.
EPI_ISL_15750680, EPI_ISL_15750681, EPI_ISL_15750752, EPI_ISL_15750753, EPI_ISL_15750754, EPI_ISL_15750755, EPI_ISL_15750756, EPI_ISL_15750757, EPI_ISL_15750758, EPI_ISL_15750759, EPI_ISL_15750760, EPI_ISL_15750761, EPI_ISL_15750762, EPI_ISL_15750763, EPI_ISL_15750764, EPI_ISL_15750765, EPI_ISL_15750767			
see above	Beijing Center for Disease Prevention and Control, Immunological Prevention	Beijing Center for Disease Prevention and Control, Immunological Prevention	Luo,Q., Li,M., Huang,F.
EPI_ISL_15750768, EPI_ISL_15750804, EPI_ISL_15750805, EPI_ISL_15750806, EPI_ISL_15750807, EPI_ISL_15750808, EPI_ISL_15750809, EPI_ISL_15750810, EPI_ISL_15750811, EPI_ISL_15750812, EPI_ISL_15750813, EPI_ISL_15750814, EPI_ISL_15750815, EPI_ISL_15750816, EPI_ISL_15750817, EPI_ISL_15750818, EPI_ISL_15750819, EPI_ISL_15750820, EPI_ISL_15750821, EPI_ISL_15750822, EPI_ISL_15750823, EPI_ISL_15750824, EPI_ISL_15750825			
see above	Department of Virology, Post Graduate Institute of Medical Education & Research	Department of Virology, Post Graduate Institute of Medical Education & Research	Sarkar,S., Ratho,R.K., Singh,M.
EPI_ISL_15750955, EPI_ISL_15750956, EPI_ISL_15750957, EPI_ISL_15750958, EPI_ISL_15750959, EPI_ISL_15750960, EPI_ISL_15750961, EPI_ISL_15750962, EPI_ISL_15750963, EPI_ISL_15750964, EPI_ISL_15750965, EPI_ISL_15750966, EPI_ISL_15750967, EPI_ISL_15750968, EPI_ISL_15750969, EPI_ISL_15750970, EPI_ISL_15750971, EPI_ISL_15750972, EPI_ISL_15750973, EPI_ISL_15750974, EPI_ISL_15750975, EPI_ISL_15750976, EPI_ISL_15750977, EPI_ISL_15751092, EPI_ISL_15751093, EPI_ISL_15751094, EPI_ISL_15751095, EPI_ISL_15751096, EPI_ISL_15751097, EPI_ISL_15751098, EPI_ISL_15751099, EPI_ISL_15751100, EPI_ISL_15751101, EPI_ISL_15751102, EPI_ISL_15751103, EPI_ISL_15751104, EPI_ISL_15751105, EPI_ISL_15751106, EPI_ISL_15751107, EPI_ISL_15751108, EPI_ISL_15751109, EPI_ISL_15751110, EPI_ISL_15751111, EPI_ISL_15751112, EPI_ISL_15751113, EPI_ISL_15751114, EPI_ISL_15751115, EPI_ISL_15751116			
see above	Beijing Center for Disease Prevention and Control, Immunological Prevention	Beijing Center for Disease Prevention and Control, Immunological Prevention	Luo,Q., Li,M., Huang,F.
EPI_ISL_15751172, EPI_ISL_15751173, EPI_ISL_15751174, EPI_ISL_15751175, EPI_ISL_15751176, EPI_ISL_15751177, EPI_ISL_15751178, EPI_ISL_15751179, EPI_ISL_15751180, EPI_ISL_15751181, EPI_ISL_15751182, EPI_ISL_15751183, EPI_ISL_15751184, EPI_ISL_15751185, EPI_ISL_15751186, EPI_ISL_15751187, EPI_ISL_15751188, EPI_ISL_15751189, EPI_ISL_15751190, EPI_ISL_15751191, EPI_ISL_15751192, EPI_ISL_15751193, EPI_ISL_15751194, EPI_ISL_15751195, EPI_ISL_15751196, EPI_ISL_15751197, EPI_ISL_15751198, EPI_ISL_15751199, EPI_ISL_15751200, EPI_ISL_15751201, EPI_ISL_15751202, EPI_ISL_15751203, EPI_ISL_15751204, EPI_ISL_15751205, EPI_ISL_15751206, EPI_ISL_15751207			
see above	Molecular Virology, Johns Hopkins School of Medicine	Molecular Virology, Johns Hopkins School of Medicine	Fall A, Elawar F, Hodcroft EB, Jallow MM, Toure CT, Barry MA, Kiori DE, Sy S, Diaw Y, Goudiaby D, Niang MN, Dia N, Fall,A., Jallow,M.M., Toure,C.T., Kiori,D.E., sy,S., Diaw,Y., Goudiaby,D., Niang,M.N., Dia,N.
EPI_ISL_15752005, EPI_ISL_15752006	Respiratory Viruses Branch, Division of Viral Diseases	Respiratory Viruses Branch, Division of Viral Diseases	Wang,L., Ng,T.F.F., Castro,C.J., Marine,R.L., Magana,L.C., Esona,M., Peret,T.C.T., Thornburg,N.J., Wang,L., Ng,T.F.F., Castro,C.J., Marine,R.L., Magana,L.C., Esona,M., Peret,T.C.T., Thornburg,N.J.
EPI_ISL_15820237	University of Ghana, Medical Laboratory Sciences	University of Ghana, Medical Laboratory Sciences	Kafintu-Kwashie,A.A., Nii-Trebi,N.I., Obodai,E., Neizer,M., Adiku,T.K., Odoom,J.K., Nii-Trebi,N.I., Kafintu-Kwashie,A.A., Obodai,E., Neizer,M., Adiku,T.K., Odoom,J.K.
EPI_ISL_15820257, EPI_ISL_15820258, EPI_ISL_15820259, EPI_ISL_15820264	Beijing Center for Disease Prevention and Control, Immunological Prevention	Beijing Center for Disease Prevention and Control, Immunological Prevention	Luo,Q., Li,M., Huang,F.
EPI_ISL_16982626, EPI_ISL_16982629, EPI_ISL_16982630, EPI_ISL_16982631	Chinese Center for Disease Control and Prevention	Chinese Center for Disease Control and Prevention	Jiang,Y.
EPI_ISL_17212969, EPI_ISL_17212970, EPI_ISL_17212971, EPI_ISL_17212972, EPI_ISL_17212973, EPI_ISL_17212974, EPI_ISL_17212975, EPI_ISL_17212976, EPI_ISL_17212977, EPI_ISL_17212978, EPI_ISL_17212979, EPI_ISL_17212980, EPI_ISL_17212981, EPI_ISL_17212982, EPI_ISL_17212983, EPI_ISL_17212984, EPI_ISL_17212985, EPI_ISL_17212986, EPI_ISL_17212987			
see above	Molecular Biology/Microbiology Research Laboratory. Universidade Federal do Paraná	Molecular Biology/Microbiology Research Laboratory. Universidade Federal do Paraná	Jucelia Stadinicki, Maria do Carmo Debur, Heloisa Ihle Garcia Giamberardino, Sonia Mara Raboni
EPI_ISL_17215964, EPI_ISL_17216082, EPI_ISL_17216185, EPI_ISL_17216395, EPI_ISL_17216545, EPI_ISL_17216606, EPI_ISL_17216784, EPI_ISL_17216845, EPI_ISL_17217036, EPI_ISL_17217100, EPI_ISL_17217118, EPI_ISL_17217552, EPI_ISL_17217556, EPI_ISL_17217563, EPI_ISL_17217572, EPI_ISL_17217611, EPI_ISL_17217662, EPI_ISL_17217909, EPI_ISL_17217945, EPI_ISL_17218122, EPI_ISL_17218152, EPI_ISL_17218156, EPI_ISL_17218229, EPI_ISL_17218235, EPI_ISL_17218285, EPI_ISL_17218649, EPI_ISL_17218657, EPI_ISL_17218669, EPI_ISL_17218711, EPI_ISL_17218772, EPI_ISL_17218827, EPI_ISL_17218870, EPI_ISL_17219029, EPI_ISL_17219168, EPI_ISL_17219173, EPI_ISL_17219241, EPI_ISL_17219249, EPI_ISL_17219529, EPI_ISL_17219687, EPI_ISL_17219692, EPI_ISL_17219948, EPI_ISL_17219983, EPI_ISL_17220028, EPI_ISL_17220225, EPI_ISL_17220272, EPI_ISL_17220279, EPI_ISL_17220365, EPI_ISL_17220570, EPI_ISL_17220677, EPI_ISL_17220910, EPI_ISL_17221221, EPI_ISL_17221389			
see above	Astrazeneca Laboratory	Astrazeneca Laboratory	Wilkins,D., Langedijk,A.C., Lebbink,R.J., Morehouse,C.A., Abram,M.E., Ahani,B., Aksyuk,A.A., Baraldi,E., Brady,T., Chen,A.T., Chi,H., Choi,E.H., Cohen,R., Danilenko,D.M., Gopalakrishnan,V., Greenough,A., Heikkinen,T., Hosoya,M., Keller,C., Kelly,E.J., Kragten-Tabatabaie,L., Martinon-Torres,F., Mascarenas-de Los Santos,A.H., Nunes,M.C., Palomino,M.A., Papenburg,J., Pernica,J.M., Richmond,P., Stein,R.T., Tuffy,K.M., Verwey,C., Esser,M.T., Tabor,D.E. and Bont,L.J.
EPI_ISL_17308723, EPI_ISL_17308726, EPI_ISL_17308727	Laboratório de Virologia - Instituto Nacional de Saúde	Laboratório de Vírus Respiratórios e do Sarampo - Instituto Oswaldo Cruz, Laboratório de Virologia - Instituto Nacional de Saúde	Almiro Tivane, Paola Resende, Neuza Nguenha, Loira Machalele, Sadia Alí, Délcio Muteto, Aunésia Marrurele, Mirela Pale, Félix Gundane, Judite Salência, Marilda Siqueira
EPI_ISL_17508474	University of Washington	University of Washington	Greninger,A.L., Makhsous,N., Kuypers,J., Shean,R.C. and Jerome,K.R.
EPI_ISL_17594669, EPI_ISL_17594677, EPI_ISL_17594685, EPI_ISL_17594687, EPI_ISL_17594690, EPI_ISL_17594696, EPI_ISL_17594701, EPI_ISL_17594704, EPI_ISL_17594707, EPI_ISL_17594709, EPI_ISL_17594712, EPI_ISL_17594713, EPI_ISL_17594759			
see above	Institute for Infectious Disease Control and Prevention, Henan Center for Disease Control and Prevention	Institute for Infectious Disease Control and Prevention, Henan Center for Disease Control and Prevention	Zhao,X., Teng,Z. and Zhang,X.
EPI_ISL_17995634, EPI_ISL_17995635, EPI_ISL_17995636, EPI_ISL_17995638, EPI_ISL_17995639, EPI_ISL_17995640, EPI_ISL_17995668, EPI_ISL_17995669, EPI_ISL_17995670, EPI_ISL_17995671, EPI_ISL_17995672, EPI_ISL_17995673, EPI_ISL_17995674, EPI_ISL_17995675			
see above	Robert Koch-Institute Nationales Referenzzentrum für Influenza	Robert Koch-Institute Nationales Referenzzentrum für Influenza	Sophie Kondgen, Janine Reiche
EPI_ISL_18334163, EPI_ISL_18334169, EPI_ISL_18334171, EPI_ISL_18334173, EPI_ISL_18334175, EPI_ISL_18334176, EPI_ISL_18334177, EPI_ISL_18334179, EPI_ISL_18334185, EPI_ISL_18334187, EPI_ISL_18334190, EPI_ISL_18334191, EPI_ISL_18334194, EPI_ISL_18334195, EPI_ISL_18334196, EPI_ISL_18334199, EPI_ISL_18334201, EPI_ISL_18334205, EPI_ISL_18334206, EPI_ISL_18334214, EPI_ISL_18334216, EPI_ISL_18334221, EPI_ISL_18334222, EPI_ISL_18334225, EPI_ISL_18334230			
see above	Baylor College of Medicine, Division of Pediatric Tropical Medicine	Baylor College of Medicine, Division of Pediatric Tropical Medicine	Avadhanula,V., Agustinho,D.P., Menon,V.K., Chemaly,R.F., Shah,D.P., Qin,X., Surathu,A., Doddapaneni,H., Muzny,D.M., Metcalf,G.A., Gregeen,S.J., Gibbs,R.A., Petrosino,J.J., Sedlazeck,F.J. and Piedra,P.A.
EPI_ISL_18396054, EPI_ISL_18396056, EPI_ISL_18396057, EPI_ISL_18396058, EPI_ISL_18396059, EPI_ISL_18396060, EPI_ISL_18396061, EPI_ISL_18396062, EPI_ISL_18396063, EPI_ISL_18396064, EPI_ISL_18396065, EPI_ISL_18396066, EPI_ISL_18396067, EPI_ISL_18396068, EPI_ISL_18396069, EPI_ISL_18396070, EPI_ISL_18396071, EPI_ISL_18396072, EPI_ISL_18396073, EPI_ISL_18396074, EPI_ISL_18396075, EPI_ISL_18396076			
see above	Niigata University (IDRC) Infectious Diseases Research Center	Niigata University (IDRC) Infectious Diseases Research Center	Wint Wint Phyu, Reiko Saito
EPI_ISL_18507286	Victorian Infectious Diseases Reference Laboratory, Translational Diagnostics	Victorian Infectious Diseases Reference Laboratory, Translational Diagnostics	Steinig JOACHIM,E.
EPI_ISL_2543926, EPI_ISL_2543927, EPI_ISL_2543928, EPI_ISL_2543929	Department of Epidemiology and Demography, Kenya Medical Research Institute (KEMRI) - Wellcome Trust Research Programme	Department of Epidemiology and Demography, Kenya Medical Research Institute (KEMRI) - Wellcome Trust Research Programme	Otieno,J.R., Kamau,E.M., Agoti,C.N., Lewa,C., Bett,A., Ngama,M., Cane,P.A. and Nokes,J.D.
EPI_ISL_2543931	Pathogen Diagnostic Center, Institut Pasteur of Shanghai, Chinese Academy of Sciences	Pathogen Diagnostic Center, Institut Pasteur of Shanghai, Chinese Academy of Sciences	Fu,X., He,Z., Lan,K., Zhang,C., Dong,W., Cheng,Y. and Hu,Y.
EPI_ISL_2543934	J. Craig Venter Institute	J. Craig Venter Institute	Shabman,R., Das,S.R., Puri,V., Fedorova,N., Amedeo,P., Williams,M., Shrivastava,S. and Halasa,N.
EPI_ISL_2543938, EPI_ISL_2543939, EPI_ISL_2543940	Virology, Graduate School of Medicine, Tohoku University	Virology, Graduate School of Medicine, Tohoku University	Malasao,R., Furuse,Y., Okamoto,M., Dapat,C., Saito,M., Saito-Obata,M., Tamaki,R., Segubre-Mercado,E., Lupisan,S. and Oshitani,H.
EPI_ISL_2543961, EPI_ISL_2543962	Influenza and Other Respiratory Viruses Unit, National Center for Microbiology, Instituto de Salud Carlos III	Influenza and Other Respiratory Viruses Unit, National Center for Microbiology, Instituto de Salud Carlos III	Calderon,A., Pozo,F., Calvo,C., Garcia-Garcia,M., Gonzalez,M., Molinero,M. and Casas,I.
EPI_ISL_2543970, EPI_ISL_2543971, EPI_ISL_2543972, EPI_ISL_2543974, EPI_ISL_2543975, EPI_ISL_2543977, EPI_ISL_2543978	J. Craig Venter Institute	J. Craig Venter Institute	Das,S.R., Halpin,R.A., Shilts,M., Puri,V., Akopov,A., Fedorova,N., Stockwell,T., Amedeo,P., Bishop,B., Katzel,D., Schobel,S., Shrivastava,S. and Hartert,T.
EPI_ISL_2543983	J. Craig Venter Institute	J. Craig Venter Institute	Shabman,R., Das,S.R., Shilts,M., Fedorova,N., Puri,V., Shrivastava,S., Amedeo,P., Hu,L., Durbin,A., Rocchi,I., Williams,T. and Hartert,T.
EPI_ISL_2543984, EPI_ISL_2543985, EPI_ISL_2543986, EPI_ISL_2543987, EPI_ISL_2543988, EPI_ISL_2543989, EPI_ISL_2543990	Lab Medicine, UW	Lab Medicine, UW	Greninger,A.L., Makhsous,N., Kuypers,J.M., Shean,R.C. and Jerome,K.R.
EPI_ISL_2544021, EPI_ISL_2544022, EPI_ISL_2544023, EPI_ISL_2544024	J. Craig Venter Institute	J. Craig Venter Institute	Das,S., Halpin,R.A., Bera,J., Fedorova,N., Tsitrin,T., Stockwell,T., Amedeo,P., Bishop,B., Gupta,N., Hoover,J., Katzel,D., Schobel,S., Shrivastava,S., Hartert,T., Moore,M., Chappell,J., Larkin,E., Wentworth,D.E. and Anderson,L.J.
EPI_ISL_2544034, EPI_ISL_2544036, EPI_ISL_2544038, EPI_ISL_2544039, EPI_ISL_2544041	J. Craig Venter Institute	J. Craig Venter Institute	Shabman,R., Das,S.R., Shilts,M., Fedorova,N., Puri,V., Shrivastava,S., Amedeo,P., Williams,M., Barratt,K., Mitchell,J. and Jennings,L.
EPI_ISL_2544050	Virology Laboratory, Dr. Ricardo Gutierrez Children Hospital	Virology Laboratory, Dr. Ricardo Gutierrez Children Hospital	Rojo,G.L., Goya,S., Orellana,M., Sancilio,A., Rodriguez Perez,A., Montali,C., Garcia,C., Sosa,L., Musto,A., Hamilton,G., Alvarez,D., Castello,A. and Viegas,M.
EPI_ISL_2544051	Virology Laboratory, Dr. Ricardo Gutierrez Children Hospital	Virology Laboratory, Dr. Ricardo Gutierrez Children Hospital	Goya,S., Valinotto,L.E., Tittarelli,E., Rojo,G.L., Greninger,A., Luso,S., Natale,M., Mistchenko,A.S. and Viegas,M.
EPI_ISL_2558776	Centro de Estudios en Salud, Universidad Del Valle De Guatemala	Centro de Estudios en Salud, Universidad Del Valle De Guatemala	Lupo,P., Jara,J., Alvis,J.P., Pennington,P., Castillo,L., Moreno,B. and Albrego,L.

EPI_ISL_2570820, EPI_ISL_2570821	Department of Clinical Laboratory, Fujian Provincial Hospital	Department of Clinical Laboratory, Fujian Provincial Hospital	Su,Y., Wu,Y., Tian,R. and Liang,G.
EPI_ISL_2570829, EPI_ISL_2570830, EPI_ISL_2570831, EPI_ISL_2570832	Laboratory of molecular virology, Research Institute of Influenza, Ministry of Healthcare of the Russian Federation	Laboratory of molecular virology, Research Institute of Influenza, Ministry of Healthcare of the Russian Federation	Komissarova,K.S., Krivitskaya,V.Z., Sverlova,M.V., Petrova,E.R., Fadeev,A.V. and Komissarov,A.B.
EPI_ISL_2570833, EPI_ISL_2570834, EPI_ISL_2570835, EPI_ISL_2570836, EPI_ISL_2570837, EPI_ISL_2570838, EPI_ISL_2570839, EPI_ISL_2570840, EPI_ISL_2570842	Health Science, Universidade Federal Do Parana	Health Science, Universidade Federal Do Parana	Moreira,F.B., Santos,J.S., Avanzi,V.M., Nogueira,M.B., Vidal,L.R.R. and Raboni,S.M.
EPI_ISL_2570843	Virology Department, Hospices Civils de Lyon	Virology Department, Hospices Civils de Lyon	Gaymard,A., Pichon,M. and Morfin,F.
EPI_ISL_2570845	Health Science, Universidade Federal Do Parana	Health Science, Universidade Federal Do Parana	Moreira,F.B., Santos,J.S., Avanzi,V.M., Nogueira,M.B., Vidal,L.R.R. and Raboni,S.M.
EPI_ISL_2570847	Virology Section, Department of Microbiology, King George's Medical University	Virology Section, Department of Microbiology, King George's Medical University	Singh,A.K., Jain,A., Jain,B., Dangi,T., Verma,A.K., Dwivedi,M. and Kushwaha,R.
EPI_ISL_2570849	Centro de Estudios en Salud, Universidad Del Valle De Guatemala	Centro de Estudios en Salud, Universidad Del Valle De Guatemala	Lupo,P., Jara,J., Alvis,J.P., Pennington,P., Castillo,L., Moreno,B. and Albrego,L.
EPI_ISL_2570850, EPI_ISL_2570851	Centre for Infectious Diseases Research, Diagnostics and laboratory Surveillance, National Institute for Public Health and the Environment	Centre for Infectious Diseases Research, Diagnostics and laboratory Surveillance, National Institute for Public Health and the Environment	Meijer,A. and Overduin,P.
EPI_ISL_2570852, EPI_ISL_2570853, EPI_ISL_2570854, EPI_ISL_2570855, EPI_ISL_2570856, EPI_ISL_2570858, EPI_ISL_2570859	Virology Department, Hospices Civils de Lyon	Virology Department, Hospices Civils de Lyon	Gaymard,A., Pichon,M. and Morfin,F.
EPI_ISL_2570860	Centro de Estudios en Salud, Universidad Del Valle De Guatemala	Centro de Estudios en Salud, Universidad Del Valle De Guatemala	Lupo,P., Jara,J., Alvis,J.P., Pennington,P., Castillo,L., Moreno,B. and Albrego,L.
EPI_ISL_2570861	Department of Medical Research, Ministry of Health and Sports, No. 5, Ziwaka Road, Dagon Township	Department of Medical Research, Ministry of Health and Sports, No. 5, Ziwaka Road, Dagon Township	Latt,A.Z., Aye,K.T. and Thwe,H.H.
EPI_ISL_2570864	Virology, University of Ibadan, College of Medicine (UCH)	Virology, University of Ibadan, College of Medicine (UCH)	Ogunsemowo,O.S., Odaibo,G.N. and Olaleye,D.O.
EPI_ISL_2570865	Virology Department, Hospices Civils de Lyon	Virology Department, Hospices Civils de Lyon	Gaymard,A., Pichon,M. and Morfin,F.
EPI_ISL_2570866, EPI_ISL_2570867, EPI_ISL_2570868, EPI_ISL_2570869, EPI_ISL_2570870	Centro de Estudios en Salud, Universidad Del Valle De Guatemala	Centro de Estudios en Salud, Universidad Del Valle De Guatemala	Lupo,P., Jara,J., Alvis,J.P., Pennington,P., Castillo,L., Moreno,B. and Albrego,L.
EPI_ISL_2570875	Virology, University of Ibadan, College of Medicine (UCH)	Virology, University of Ibadan, College of Medicine (UCH)	Ogunsemowo,O., Odaibo,G.N. and Olaleye,D.O.
EPI_ISL_2570878, EPI_ISL_2570879, EPI_ISL_2570880, EPI_ISL_2570881, EPI_ISL_2570882, EPI_ISL_2570883, EPI_ISL_2570884, EPI_ISL_2570885, EPI_ISL_2570886, EPI_ISL_2570887, EPI_ISL_2570888, EPI_ISL_2570889, EPI_ISL_2570890, EPI_ISL_2570891, EPI_ISL_2570892, EPI_ISL_2570893, EPI_ISL_2570894, EPI_ISL_2570895, EPI_ISL_2570896, EPI_ISL_2570897, EPI_ISL_2570898, EPI_ISL_2570899, EPI_ISL_2570900, EPI_ISL_2570901, EPI_ISL_2570902, EPI_ISL_2570903, EPI_ISL_2570904, EPI_ISL_2570905, EPI_ISL_2570906, EPI_ISL_2570907, EPI_ISL_2570908, EPI_ISL_2570909			
see above	Department of Epidemiology and Demography, Kenya Medical Research Institute (KEMRI) - Wellcome Trust Research Programme	Department of Epidemiology and Demography, Kenya Medical Research Institute (KEMRI) - Wellcome Trust Research Programme	Otieno,J.R., Kamau,E.M., Agoti,C.N., Lewa,C., Bett,A., Ngama,M., Cane,P.A. and Nokes,J.D.
EPI_ISL_2570910, EPI_ISL_2570911	Epidemiology and Demography, KEMRI - Wellcome Trust Research Programme, Centre for Geographic Medicine Research	Epidemiology and Demography, KEMRI - Wellcome Trust Research Programme, Centre for Geographic Medicine Research	Oketch,J.W., Kamau,E., Otieno,J.R., Mwema,A., Lewa,C., Agoti,C.N. and Nokes,J.D.
EPI_ISL_2570912	Virology, University of Ibadan, College of Medicine (UCH)	Virology, University of Ibadan, College of Medicine (UCH)	Ogunsemowo,O., Odaibo,G.N. and Olaleye,D.O.
EPI_ISL_2570913	Botany and Microbiology, College of Science, King Saud University, King Saud University	Botany and Microbiology, College of Science, King Saud University, King Saud University	Amer,H.M., Farrag,M.A., Hamad,M.E., Aziz,I.M. and Almajhdi,F.N.
EPI_ISL_2570914, EPI_ISL_2570915	Epidemiology and Demography, KEMRI - Wellcome Trust Research Programme, Centre for Geographic Medicine Research	Epidemiology and Demography, KEMRI - Wellcome Trust Research Programme, Centre for Geographic Medicine Research	Oketch,J.W., Kamau,E., Otieno,J.R., Mwema,A., Lewa,C., Agoti,C.N. and Nokes,J.D.
EPI_ISL_2570922, EPI_ISL_2570923	Department of Respiratory Medicine, Children's Hospital of Chongqing Medical University	Department of Respiratory Medicine, Children's Hospital of Chongqing Medical University	Ren,L. and Liu,E.
EPI_ISL_2570924, EPI_ISL_2570925, EPI_ISL_2570926	Department for Infectious Diseases Virology, University of Heidelberg	Department for Infectious Diseases Virology, University of Heidelberg	Tabatabai,J., Thielen,A., Lehners,N., Daeumer,M. and Schnitzler,P.
EPI_ISL_2570927	Centre for Infectious Diseases Virology, University of Heidelberg	Centre for Infectious Diseases Virology, University of Heidelberg	Ihling,C.M., Schnitzler,P., Heinrich,N., Mangu,C., Sudi,L., Souares,A., Gies,S., Sie,A., Coulibaly,B., Ouedraogo,A.T., Mordmueller,B., Held,J., Adegnika,A.A., Fernandez,J.F., Eckerle,I., May,J., Hogan,B., Eibach,D., Owusu-Dabo,E. and Tabatabai,J.
EPI_ISL_2570930, EPI_ISL_2570931, EPI_ISL_2570932, EPI_ISL_2570933, EPI_ISL_2570934, EPI_ISL_2570935	Pediatrics, Mackay Memorial Hospital	Pediatrics, Mackay Memorial Hospital	Chi,H., Hsiao,K.-L., Weng,L.-C., Chiu,N.-C., Huang,L.-M., Chiu,Y.-Y., Liu,C.-P. and Liu,H.-F.
EPI_ISL_2570936, EPI_ISL_2570937, EPI_ISL_2570938, EPI_ISL_2570939, EPI_ISL_2570940, EPI_ISL_2570941, EPI_ISL_2570942, EPI_ISL_2570943, EPI_ISL_2570944	Pediatrics, Show-Chwan Memorial Hospital	Pediatrics, Show-Chwan Memorial Hospital	Lee,C.-Y.
EPI_ISL_2570948, EPI_ISL_2570949, EPI_ISL_2570950, EPI_ISL_2570951, EPI_ISL_2570952, EPI_ISL_2570953, EPI_ISL_2570954, EPI_ISL_2570955, EPI_ISL_2570956, EPI_ISL_2570957, EPI_ISL_2570958, EPI_ISL_2570959, EPI_ISL_2570960, EPI_ISL_2570961, EPI_ISL_2570962, EPI_ISL_2570963			
see above	Virology Laboratory, Dr. Ricardo Gutierrez Children Hospital	Virology Laboratory, Dr. Ricardo Gutierrez Children Hospital	Rojo,G.L., Goya,S., Orellana,M., Sancilio,A., Rodriguez Perez,A., Montali,C., Garcia,C., Sosa,L., Musto,A., Hamilton,G., Alvarez,D., Castello,A. and Viegas,M.
EPI_ISL_2570964, EPI_ISL_2570965, EPI_ISL_2570966, EPI_ISL_2570967	Depto Microbiologia, Instituto de Ciencias Biomedicas, Universidade de Sao Paulo	Depto Microbiologia, Instituto de Ciencias Biomedicas, Universidade de Sao Paulo	Thomazelli,L.M., Oliveira,D.B.L., Colmanetti,T., Vieira,S.E., Paulis,M., Ferronato,A.E., Martinez,M.B. and Durigon,E.L.
EPI_ISL_2570991, EPI_ISL_2570992, EPI_ISL_2570993, EPI_ISL_2570994, EPI_ISL_2570995, EPI_ISL_2570996	Chinese Academy of Medical Sciences & Peking Union Medical College	Chinese Academy of Medical Sciences & Peking Union Medical College	Jia,B., Xiao,Y., Wang,Y., Chen,L., Zhang,J., Ren,L. and Wang,J.
EPI_ISL_2570997, EPI_ISL_2570998, EPI_ISL_2570999, EPI_ISL_2571000, EPI_ISL_2571001	WHO WPRO Measles Regional Reference Lab, Key Laboratory of Medical Virology Ministry of Health, National Institute for Viral Disease Control and Prevention, China Center for Disease Control and Prevention	WHO WPRO Measles Regional Reference Lab, Key Laboratory of Medical Virology Ministry of Health, National Institute for Viral Disease Control and Prevention, China Center for Disease Control and Prevention	Zhang,Y., Song,J. and Xu,W.
EPI_ISL_2571002, EPI_ISL_2571003, EPI_ISL_2571004, EPI_ISL_2571005	Institute of Virology and AIDS Research, First Hospital of Jilin University	Institute of Virology and AIDS Research, First Hospital of Jilin University	Zheng,Y., Liu,L., Wang,S., Li,Z., Hou,M., Li,J., Yu,X.-F., Zhang,W. and Hua,S.
EPI_ISL_2571006, EPI_ISL_2571007, EPI_ISL_2571008, EPI_ISL_2571009, EPI_ISL_2571010, EPI_ISL_2571011, EPI_ISL_2571012, EPI_ISL_2571013, EPI_ISL_2571014, EPI_ISL_2571015, EPI_ISL_2571016, EPI_ISL_2571017, EPI_ISL_2571018, EPI_ISL_2571019, EPI_ISL_2571020, EPI_ISL_2571021, EPI_ISL_2571022, EPI_ISL_2571023, EPI_ISL_2571024, EPI_ISL_2571025, EPI_ISL_2571026, EPI_ISL_2571027, EPI_ISL_2571028, EPI_ISL_2571029, EPI_ISL_2571030, EPI_ISL_2571031, EPI_ISL_2571032, EPI_ISL_2571033, EPI_ISL_2571034			
see above	WHO WPRO Measles Regional Reference Lab, Key Laboratory of Medical Virology Ministry of Health, National Institute for Viral Disease Control and Prevention, China Center for Disease Control and Prevention	WHO WPRO Measles Regional Reference Lab, Key Laboratory of Medical Virology Ministry of Health, National Institute for Viral Disease Control and Prevention, China Center for Disease Control and Prevention	Zhang,Y., Song,J. and Xu,W.
EPI_ISL_2571035	Institute of Virology and AIDS Research, First Hospital of Jilin University	Institute of Virology and AIDS Research, First Hospital of Jilin University	Zheng,Y., Liu,L., Wang,S., Li,Z., Hou,M., Li,J., Yu,X.-F., Zhang,W. and Hua,S.
EPI_ISL_2571036, EPI_ISL_2571037	WHO WPRO Measles Regional Reference Lab, Key Laboratory of Medical Virology Ministry of Health, National Institute for Viral Disease Control and Prevention, China Center for Disease Control and Prevention	WHO WPRO Measles Regional Reference Lab, Key Laboratory of Medical Virology Ministry of Health, National Institute for Viral Disease Control and Prevention, China Center for Disease Control and Prevention	Zhang,Y., Song,J. and Xu,W.
EPI_ISL_2571038, EPI_ISL_2571039, EPI_ISL_2571040, EPI_ISL_2571041	Institute of Virology and AIDS Research, First Hospital of Jilin University	Institute of Virology and AIDS Research, First Hospital of Jilin University	Zheng,Y., Liu,L., Wang,S., Li,Z., Hou,M., Li,J., Yu,X.-F., Zhang,W. and Hua,S.
EPI_ISL_2571042, EPI_ISL_2571043	Department of Public Health Laboratory Sciences, West China School of Public Health, Sichuan University	Department of Public Health Laboratory Sciences, West China School of Public Health, Sichuan University	Hu,W.P. and Pei,F.X.
EPI_ISL_2571044, EPI_ISL_2571045, EPI_ISL_2571046, EPI_ISL_2571047, EPI_ISL_2571048, EPI_ISL_2571049, EPI_ISL_2571050, EPI_ISL_2571051, EPI_ISL_2571052, EPI_ISL_2571053, EPI_ISL_2571054, EPI_ISL_2571055			
see above	WHO WPRO Measles Regional Reference Lab, Key Laboratory of Medical Virology Ministry of Health, National Institute for Viral Disease Control and Prevention, China Center for Disease Control and Prevention	WHO WPRO Measles Regional Reference Lab, Key Laboratory of Medical Virology Ministry of Health, National Institute for Viral Disease Control and Prevention, China Center for Disease Control and Prevention	Zhang,Y., Song,J. and Xu,W.
EPI_ISL_2571083, EPI_ISL_2571084, EPI_ISL_2571085, EPI_ISL_2571086, EPI_ISL_2571087, EPI_ISL_2571088, EPI_ISL_2571089, EPI_ISL_2571090, EPI_ISL_2571091, EPI_ISL_2571092, EPI_ISL_2571093, EPI_ISL_2571096, EPI_ISL_2571099, EPI_ISL_2571101, EPI_ISL_2571102, EPI_ISL_2571103, EPI_ISL_2571104, EPI_ISL_2571105, EPI_ISL_2571106, EPI_ISL_2571107, EPI_ISL_2571108, EPI_ISL_2571110, EPI_ISL_2571113, EPI_ISL_2571114, EPI_ISL_2571115, EPI_ISL_2571116, EPI_ISL_2571117, EPI_ISL_2571118, EPI_ISL_2571120, EPI_ISL_2571121, EPI_ISL_2571124, EPI_ISL_2571126, EPI_ISL_2571129, EPI_ISL_2571132, EPI_ISL_2571133, EPI_ISL_2571134, EPI_ISL_2571135, EPI_ISL_2571137, EPI_ISL_2571140, EPI_ISL_2571141, EPI_ISL_2571142, EPI_ISL_2571148, EPI_ISL_2571149, EPI_ISL_2571150, EPI_ISL_2571151, EPI_ISL_2571152, EPI_ISL_2571154, EPI_ISL_2571156, EPI_ISL_2571157, EPI_ISL_2571158, EPI_ISL_2571160, EPI_ISL_2571161, EPI_ISL_2571162, EPI_ISL_2571163, EPI_ISL_2571164, EPI_ISL_2571165, EPI_ISL_2571166, EPI_ISL_2571167			
see above	University of Wuerzburg, Institute of Virology and Immunobiology	University of Wuerzburg, Institute of Virology and Immunobiology	Prifert,C., Hofmann,D. and Weissbrich,B.
EPI_ISL_2571168, EPI_ISL_2571169, EPI_ISL_2571170	Virology, Noguchi Memorial Institute for Medical Research	Virology, Noguchi Memorial Institute for Medical Research	Obodai,E., Odoom,J.K., Adiku,T., Goka,B., Biere,B., Wolff,T., Schweiger,B. and Reiche,J.
EPI_ISL_2571181, EPI_ISL_2571182	Department of Virology, School of Public Health, Tehran University of Medical Sciences	Department of Virology, School of Public Health, Tehran University of Medical Sciences	Salimi,V., Samieipoor,Y., Ghavami,N. and Mokhtariazad,T.
EPI_ISL_2571187, EPI_ISL_2571188, EPI_ISL_2571189, EPI_ISL_2571190, EPI_ISL_2571191, EPI_ISL_2571192, EPI_ISL_2571193, EPI_ISL_2571195, EPI_ISL_2571196, EPI_ISL_2571197, EPI_ISL_2571198, EPI_ISL_2571199, EPI_ISL_2571200, EPI_ISL_2571201, EPI_ISL_2571202, EPI_ISL_2571203, EPI_ISL_2571204, EPI_ISL_2571205, EPI_ISL_2571206, EPI_ISL_2571207, EPI_ISL_2571208, EPI_ISL_2571209, EPI_ISL_2571210, EPI_ISL_2571211, EPI_ISL_2571212, EPI_ISL_2571213, EPI_ISL_2571214, EPI_ISL_2571215, EPI_ISL_2571216, EPI_ISL_2571217, EPI_ISL_2571218, EPI_ISL_2571219, EPI_ISL_2571220, EPI_ISL_2571221			
see above	Pediatric Clinic 1, Department of Pathophysiology and Transplantation, University of Milan and Fondazione IRCCS Ca Granda	Pediatric Clinic 1, Department of Pathophysiology and Transplantation, University of Milan and Fondazione IRCCS Ca Granda	Esposito,S., Zampiero,A., Piralla,A. and Principi,N.
EPI_ISL_2571222, EPI_ISL_2571223, EPI_ISL_2571224, EPI_ISL_2571225, EPI_ISL_2571226, EPI_ISL_2571229, EPI_ISL_2571230, EPI_ISL_2571231, EPI_ISL_2571232, EPI_ISL_2571233	Virology, Tohoku University Graduate School of Medicine	Virology, Tohoku University Graduate School of Medicine	Malasao,R., Okamoto,M., Chaimongkol,N., Imamura,T., Tohma,K., Dapat,I., Dapat,C., Suzuki,A., Saito,M., Saito,M., Tamaki,R., Segubre-Mercado,E., Igoy,M.A.U., Lupisan,S., Olveda,R. and Oshitani,H.
EPI_ISL_2571276, EPI_ISL_2571277, EPI_ISL_2571278, EPI_ISL_2571279, EPI_ISL_2571280, EPI_ISL_2571281, EPI_ISL_2571282, EPI_ISL_2571283, EPI_ISL_2571284, EPI_ISL_2571285, EPI_ISL_2571286, EPI_ISL_2571287, EPI_ISL_2571288, EPI_ISL_2571289, EPI_ISL_2571290, EPI_ISL_2571291, EPI_ISL_2571292, EPI_ISL_2571330, EPI_ISL_2571331, EPI_ISL_2571332, EPI_ISL_2571333, EPI_ISL_2571334,			

EPI_ISL_2571335			
see above	Microbiology, Hospital Universitari Vall d'Hebron	Microbiology, Hospital Universitari Vall d'Hebron	Gimferrer,L., Martin,Md.C., Pumarola,T. and Anton,A.
EPI_ISL_2571361	Epidemiology and Immunogenetic of Viral Infections LR14SP02, University Hospital Sahloul	Epidemiology and Immunogenetic of Viral Infections LR14SP02, University Hospital Sahloul	Ataoui,I., Jerbi,A., BenHamida-Rebai,M., Ben Hadj Fredj,M., Fodha,I., Bennour,H., Hammouda,H., Khlifa,M., Brini,I., Boussoffara,R., Boussetta,K., Abroug,S. and Trabelsi,A.
EPI_ISL_2571378	Virology Laboratory, Dr. Ricardo Gutierrez Children Hospital	Virology Laboratory, Dr. Ricardo Gutierrez Children Hospital	Rojo,G.L., Goya,S., Orellana,M., Sancilio,A., Rodriguez Perez,A., Montali,C., Garcia,C., Sosa,L., Musto,A., Hamilton,G., Alvarez,D., Castello,A. and Viegas,M.
EPI_ISL_2571379	Depto Microbiologia, Instituto de Ciencias Biomedicas, Universidade de Sao Paulo	Depto Microbiologia, Instituto de Ciencias Biomedicas, Universidade de Sao Paulo	Thomazelli,L.M., Oliveira,D.B.L., Colmanetti,T., Vieira,S.E., Paulis,M., Ferronato,A.E., Martinez,M.B. and Durigon,E.L.
EPI_ISL_2571380	Virology, Noguchi Memorial Institute for Medical Research	Virology, Noguchi Memorial Institute for Medical Research	Obodai,E., Odoom,J.K., Adiku,T., Goka,B., Biere,B., Wolff,T., Schweiger,B. and Reiche,J.
EPI_ISL_2571383, EPI_ISL_2571387	Chinese Academy of Medical Sciences & Peking Union Medical College	Chinese Academy of Medical Sciences & Peking Union Medical College	Jia,B., Xiao,Y., Wang,Y., Chen,L., Zhang,J., Ren,L. and Wang,J.
EPI_ISL_2571392, EPI_ISL_2571393, EPI_ISL_2571394, EPI_ISL_2571395, EPI_ISL_2571396	Department of Clinical Laboratory, Fujian Provincial Hospital	Department of Clinical Laboratory, Fujian Provincial Hospital	Su,Y., Wu,Y., Tian,R. and Liang,G.
EPI_ISL_2571401, EPI_ISL_2571402, EPI_ISL_2571403	Lab. of Molecular Virology, Research Institute of Influenza	Lab. of Molecular Virology, Research Institute of Influenza	Sintsova,K.S., Krivitskaya,V.Z., Sverlova,M.V., Petrova,E.R., Fadeev,A.V. and Komissarov,A.B.
EPI_ISL_2571408, EPI_ISL_2571409, EPI_ISL_2571410	Influenza and Other Respiratory Viruses Unit, National Center for Microbiology, Instituto de Salud Carlos III	Influenza and Other Respiratory Viruses Unit, National Center for Microbiology, Instituto de Salud Carlos III	Calderon,A., Pozo,F., Calvo,C., Garcia-Garcia,M., Gonzalez,M., Molinero,M. and Casas,I.
EPI_ISL_2571411, EPI_ISL_2571412, EPI_ISL_2571413, EPI_ISL_2571414, EPI_ISL_2571415, EPI_ISL_2571416, EPI_ISL_2571417, EPI_ISL_2571418, EPI_ISL_2571419, EPI_ISL_2571420, EPI_ISL_2571421			
see above	Department of Pediatrics, Center of Excellence in Clinical Virology, Faculty of Medicine, Chulalongkorn University	Department of Pediatrics, Center of Excellence in Clinical Virology, Faculty of Medicine, Chulalongkorn University	Thongpan,I., Mauleekoonphairoj,J., Vichiwattana,P., Korkong,S., Vongpunsawad,S. and Poovorawan,Y.
EPI_ISL_2571432, EPI_ISL_2571433, EPI_ISL_2571434	Miho Kobayashi Gunma Prefectural Institute of Public Health and Environmental Sciences	Miho Kobayashi Gunma Prefectural Institute of Public Health and Environmental Sciences	Kobayashi,M. and Kimura,H.
EPI_ISL_2571435, EPI_ISL_2571436, EPI_ISL_2571437, EPI_ISL_2571438, EPI_ISL_2571439, EPI_ISL_2571440, EPI_ISL_2571441, EPI_ISL_2571442, EPI_ISL_2571443, EPI_ISL_2571444, EPI_ISL_2571445, EPI_ISL_2571446, EPI_ISL_2571447, EPI_ISL_2571448, EPI_ISL_2571449, EPI_ISL_2571450, EPI_ISL_2571451, EPI_ISL_2571452, EPI_ISL_2571453, EPI_ISL_2571454, EPI_ISL_2571455, EPI_ISL_2571456			
see above	Eiko Hirano Fukui Prefectural Institute of Public Health and Environmental Science	Eiko Hirano Fukui Prefectural Institute of Public Health and Environmental Science	Hirano,E. and Kobayashi,M.
EPI_ISL_2571457	Akinobu Hibino Niigata University, International Health, Public Health	Akinobu Hibino Niigata University, International Health, Public Health	Hibino,A., Saito,R., Shoubugawa,Y. and Takei,T.
EPI_ISL_2571458, EPI_ISL_2571459, EPI_ISL_2571460, EPI_ISL_2571461, EPI_ISL_2571462, EPI_ISL_2571463	Akinobu Hibino Niigata University, International Health, Public Health	Akinobu Hibino Niigata University, International Health, Public Health	Hibino,A., Saito,R., Shoubugawa,Y. and Shimada,Y.
EPI_ISL_2571464, EPI_ISL_2571465	Akinobu Hibino Niigata University, International Health, Public Health	Akinobu Hibino Niigata University, International Health, Public Health	Hibino,A., Saito,R., Shoubugawa,Y. and Taniguchi,K.
EPI_ISL_2571466	Akinobu Hibino Niigata University, International Health, Public Health	Akinobu Hibino Niigata University, International Health, Public Health	Hibino,A., Saito,R., Shoubugawa,Y. and Sato,I.
EPI_ISL_2571467, EPI_ISL_2571468, EPI_ISL_2571469, EPI_ISL_2571470	Akinobu Hibino Niigata University, International Health, Public Health	Akinobu Hibino Niigata University, International Health, Public Health	Hibino,A., Saito,R., Shoubugawa,Y. and Sano,Y.
EPI_ISL_2571471, EPI_ISL_2571472, EPI_ISL_2571473, EPI_ISL_2571474, EPI_ISL_2571475, EPI_ISL_2571476, EPI_ISL_2571477, EPI_ISL_2571478, EPI_ISL_2571479, EPI_ISL_2571480, EPI_ISL_2571481, EPI_ISL_2571482, EPI_ISL_2571483			
see above	Akinobu Hibino Niigata University, International Health, Public Health	Akinobu Hibino Niigata University, International Health, Public Health	Hibino,A., Saito,R., Shoubugawa,Y., Makiya,T. and Takefuta,K.
EPI_ISL_2571484, EPI_ISL_2571485, EPI_ISL_2571486, EPI_ISL_2571487, EPI_ISL_2571488, EPI_ISL_2571489, EPI_ISL_2571490, EPI_ISL_2571491, EPI_ISL_2571492	Akinobu Hibino Niigata University, International Health, Public Health	Akinobu Hibino Niigata University, International Health, Public Health	Hibino,A., Saito,R., Shoubugawa,Y., Htay,H.T., Khin,Y.O., Yi,Y.M. and Yadanar,K.
EPI_ISL_2571508	Lab. of Molecular Virology, Research Institute of Influenza	Lab. of Molecular Virology, Research Institute of Influenza	Sintsova,K.S., Krivitskaya,V.Z., Sverlova,M.V., Petrova,E.R., Fadeev,A.V. and Komissarov,A.B.
EPI_ISL_2571509, EPI_ISL_2571510, EPI_ISL_2571511, EPI_ISL_2571512, EPI_ISL_2571513, EPI_ISL_2571514	Medicine, American University of Beirut	Medicine, American University of Beirut	Abou-El-Hassan,H. and Zaraket,H.
EPI_ISL_2571534	Department of Pediatrics, Center of Excellence in Clinical Virology, Faculty of Medicine, Chulalongkorn University	Department of Pediatrics, Center of Excellence in Clinical Virology, Faculty of Medicine, Chulalongkorn University	Thongpan,I., Mauleekoonphairoj,J., Vichiwattana,P., Korkong,S., Vongpunsawad,S. and Poovorawan,Y.
EPI_ISL_2571549, EPI_ISL_2571550, EPI_ISL_2571551	Department of Clinical Laboratory, Fujian Provincial Hospital	Department of Clinical Laboratory, Fujian Provincial Hospital	Su,Y., Wu,Y., Tian,R. and Liang,G.
EPI_ISL_2571558	Virology, Evandro Chagas Institute	Virology, Evandro Chagas Institute	Santos,V.M., Ferreira,J.A., Lima,J.F., Barbagelata,L.S., Souza,E.M.A., Goncalves,M.S., SoNorth America / USA,E.C. Jr., Costa,I.B., Santos,M.C., SoNorth America / USA,R.C.M. and Mello,W.A.
EPI_ISL_2571572	Chinese Academy of Medical Sciences & Peking Union Medical College	Chinese Academy of Medical Sciences & Peking Union Medical College	Jia,B., Xiao,Y., Wang,Y., Chen,L., Zhang,J., Ren,L. and Wang,J.
EPI_ISL_2571576	Laboratory of Virology, Capital Institute of Pediatrics	Laboratory of Virology, Capital Institute of Pediatrics	Cui,G., Zhu,R., Qian,Y., Deng,J., Zhao,L., Sun,Y. and Wang,F.
EPI_ISL_2571577, EPI_ISL_2571579	Lab. of Molecular Virology, Research Institute of Influenza	Lab. of Molecular Virology, Research Institute of Influenza	Sintsova,K.S., Krivitskaya,V.Z., Sverlova,M.V., Petrova,E.R., Fadeev,A.V. and Komissarov,A.B.
EPI_ISL_2571585, EPI_ISL_2571586	Influenza and Other Respiratory Viruses Unit, National Center for Microbiology, Instituto de Salud Carlos III	Influenza and Other Respiratory Viruses Unit, National Center for Microbiology, Instituto de Salud Carlos III	Calderon,A., Pozo,F., Calvo,C., Garcia-Garcia,M., Gonzalez,M., Molinero,M. and Casas,I.
EPI_ISL_2571590	Division of Public Health Research, Gyeonggi Province institute of Health and Environment	Division of Public Health Research, Gyeonggi Province institute of Health and Environment	Park,E., Park,P., Huh,J., Yun,H., Lee,H., Yoon,M., Lee,S. and Ko,G.
EPI_ISL_2571593, EPI_ISL_2571594, EPI_ISL_2571595, EPI_ISL_2571596	Department of Epidemiology and Demography, Kenya Medical Research Institute (KEMRI) - Wellcome Trust Research Programme	Department of Epidemiology and Demography, Kenya Medical Research Institute (KEMRI) - Wellcome Trust Research Programme	Otieno,J.R., Kamau,E.M., Agoti,C.N., Lewa,C., Bett,A., Ngama,M., Cane,P.A. and Nokes,J.D.
EPI_ISL_2571597	Botany and Microbiology, College of Science, King Saud University, King Saud University	Botany and Microbiology, College of Science, King Saud University, King Saud University	Amer,H.M., Farrag,M.A., Hamad,M.E., Aziz,I.M. and Almajhdi,F.N.
EPI_ISL_2571599	Epidemiology and Demography, KEMRI - Wellcome Trust Research Programme, Centre for Geographic Medicine Research	Epidemiology and Demography, KEMRI - Wellcome Trust Research Programme, Centre for Geographic Medicine Research	Oketch,J.W., Kamau,E., Otieno,J.R., Mwema,A., Lewa,C., Agoti,C.N. and Nokes,J.D.
EPI_ISL_2571600, EPI_ISL_2571601, EPI_ISL_2571602, EPI_ISL_2571603, EPI_ISL_2571604	Molecular Medicine Laboratory of Virology, Sapienza University	Molecular Medicine Laboratory of Virology, Sapienza University	Pierangeli,A.
EPI_ISL_2571608, EPI_ISL_2571609, EPI_ISL_2571610	Pediatrics, Show-Chwan Memorial Hospital	Pediatrics, Show-Chwan Memorial Hospital	Lee,C.-Y.
EPI_ISL_2571616, EPI_ISL_2571617	Pediatrics, Mackay Memorial Hospital	Pediatrics, Mackay Memorial Hospital	Chi,H., Hsiao,K.-L., Weng,L.-C., Chiu,N.-C., Huang,L.-M., Chiu,Y.-Y., Liu,C.-P. and Liu,H.-F.
EPI_ISL_2571618	Pediatrics, Show-Chwan Memorial Hospital	Pediatrics, Show-Chwan Memorial Hospital	Lee,C.-Y.
EPI_ISL_2571623, EPI_ISL_2571624, EPI_ISL_2571625, EPI_ISL_2571626, EPI_ISL_2571627, EPI_ISL_2571628, EPI_ISL_2571629, EPI_ISL_2571630, EPI_ISL_2571631, EPI_ISL_2571632, EPI_ISL_2571633			
see above	Virology Laboratory, Dr. Ricardo Gutierrez Children Hospital	Virology Laboratory, Dr. Ricardo Gutierrez Children Hospital	Rojo,G.L., Goya,S., Orellana,M., Sancilio,A., Rodriguez Perez,A., Montali,C., Garcia,C., Sosa,L., Musto,A., Hamilton,G., Alvarez,D., Castello,A. and Viegas,M.
EPI_ISL_2571637	Department of Pediatrics, Center of Excellence in Clinical Virology, Faculty of Medicine, Chulalongkorn University	Department of Pediatrics, Center of Excellence in Clinical Virology, Faculty of Medicine, Chulalongkorn University	Thongpan,I., Mauleekoonphairoj,J., Vichiwattana,P., Korkong,S., Vongpunsawad,S. and Poovorawan,Y.
EPI_ISL_2571638, EPI_ISL_2571639, EPI_ISL_2571640, EPI_ISL_2571641, EPI_ISL_2571642	Health Science, Universidade Federal Do Parana	Health Science, Universidade Federal Do Parana	Moreira,F.B., Santos,J.S., Avanzi,V.M., Nogueira,M.B., Vidal,L.R.R. and Raboni,S.M.
EPI_ISL_2571644	Depto Microbiologia, Instituto de Ciencias Biomedicas, Universidade de Sao Paulo	Depto Microbiologia, Instituto de Ciencias Biomedicas, Universidade de Sao Paulo	Thomazelli,L.M., Oliveira,D.B.L., Colmanetti,T., Vieira,S.E., Paulis,M., Ferronato,A.E., Martinez,M.B. and Durigon,E.L.
EPI_ISL_2571645	Centro de Estudios en Salud, Universidad Del Valle De Guatemala	Centro de Estudios en Salud, Universidad Del Valle De Guatemala	Lupo,P., Jara,J., Alvis,J.P., Pennington,P., Castillo,L., Moreno,B. and Albrego,L.
EPI_ISL_2571646	Centre for Infectious Diseases Research, Diagnostics and laboratory Surveillance, National Institute for Public Health and the Environment	Centre for Infectious Diseases Research, Diagnostics and laboratory Surveillance, National Institute for Public Health and the Environment	Meijer,A. and Overduin,P.
EPI_ISL_2571647	Depto Microbiologia, Instituto de Ciencias Biomedicas, Universidade de Sao Paulo	Depto Microbiologia, Instituto de Ciencias Biomedicas, Universidade de Sao Paulo	Moura,F.E.A., Thomazelli,L.M., Campelo,F.S., Delfraro,A., Arbiza,J. and Durigon,E.L.
EPI_ISL_2571648	Virology Department, Hospices Civils de Lyon	Virology Department, Hospices Civils de Lyon	Gaymard,A., Pichon,M. and Morfin,F.
EPI_ISL_2571649, EPI_ISL_2571650	Centro de Estudios en Salud, Universidad Del Valle De Guatemala	Centro de Estudios en Salud, Universidad Del Valle De Guatemala	Lupo,P., Jara,J., Alvis,J.P., Pennington,P., Castillo,L., Moreno,B. and Albrego,L.
EPI_ISL_2571652, EPI_ISL_2571653, EPI_ISL_2571654	Chinese Academy of Medical Sciences & Peking Union Medical College	Chinese Academy of Medical Sciences & Peking Union Medical College	Jia,B., Xiao,Y., Wang,Y., Chen,L., Zhang,J., Ren,L. and Wang,J.
EPI_ISL_2571657	Centre for Research And Knowledge Transfer in Biotechnology, University of Zagreb	Centre for Research And Knowledge Transfer in Biotechnology, University of Zagreb	Jagusic,M., Slovic,A., Santak,M., Kosutic-Gulija,T., Ivancic-Jelecki,J. and Forcic,D.
EPI_ISL_2571658	Centre for Research And Knowledge Transfer in Biotechnology, University of Zagreb	Centre for Research And Knowledge Transfer in Biotechnology, University of Zagreb	Ivancic-Jelecki,J., Slovic,A., Ljubin-Sternak,S., Mlinaric-Galinovic,G. and Forcic,D.
EPI_ISL_2571660	Department of Medical Research, Ministry of Health and Sports, No. 5, Ziwaka Road, Dagon Township	Department of Medical Research, Ministry of Health and Sports, No. 5, Ziwaka Road, Dagon Township	Aye,K.T., Latt,A.Z. and Thwe,H.H.
EPI_ISL_2571661	Department of Medical Research, Ministry of Health and Sports, No. 5, Ziwaka Road, Dagon Township	Department of Medical Research, Ministry of Health and Sports, No. 5, Ziwaka Road, Dagon Township	Latt,A.Z., Aye,K.T. and Thwe,H.H.
EPI_ISL_2571662	Department of Medical Research, Ministry of Health and Sports, No. 5, Ziwaka	Department of Medical Research, Ministry of Health and Sports, No. 5, Ziwaka	Aye,K.T.

	Road, Dagon Township	Road, Dagon Township	
EPI_ISL_2571663	Lab. of Molecular Virology, Research Institute of Influenza	Lab. of Molecular Virology, Research Institute of Influenza	Sintsova,K.S., Krivitskaya,V.Z., Sverlova,M.V., Petrova,E.R., Fadeev,A.V. and Komissarov,A.B.
EPI_ISL_2571666	Virology Department, National Center of Microbiology, Instituto de Salud Carlos III	Virology Department, National Center of Microbiology, Instituto de Salud Carlos III	Casas,I., Calderon,A., Pozo,F., Calvo,C., Garcia-Garcia,M., Gonzalez,M. and Molinero,M.
EPI_ISL_2571669	WHO WPRO Measles Regional Reference Lab, Key Laboratory of Medical Virology Ministry of Health, National Institute for Viral Disease Control and Prevention, China Center for Disease Control and Prevention	WHO WPRO Measles Regional Reference Lab, Key Laboratory of Medical Virology Ministry of Health, National Institute for Viral Disease Control and Prevention, China Center for Disease Control and Prevention	Song,J., Zhang,Y. and Xu,W.
EPI_ISL_2571671	Laboratory of molecular virology, Research Institute of Influenza, Ministry of Healthcare of the Russian Federation	Laboratory of molecular virology, Research Institute of Influenza, Ministry of Healthcare of the Russian Federation	Komissarova,K.S., Krivitskaya,V.Z., Sverlova,M.V., Petrova,E.R., Fadeev,A.V. and Komissarov,A.B.
EPI_ISL_2571672	Virology, University of Ibadan, College of Medicine (UCH)	Virology, University of Ibadan, College of Medicine (UCH)	Ogunsemowo,O.S., Odaibo,G.N. and Olaleye,D.O.
EPI_ISL_2571673	Virology, University of Ibadan, College of Medicine (UCH)	Virology, University of Ibadan, College of Medicine (UCH)	Ogunsemowo,O.O., Odaibo,G.N. and Olaleye,D.O.
EPI_ISL_2571674	Lab. of Molecular Virology, Research Institute of Influenza	Lab. of Molecular Virology, Research Institute of Influenza	Sintsova,K.S., Krivitskaya,V.Z., Sverlova,M.V., Petrova,E.R., Fadeev,A.V. and Komissarov,A.B.
EPI_ISL_2571676	Centro de Estudios en Salud, Universidad Del Valle De Guatemala	Centro de Estudios en Salud, Universidad Del Valle De Guatemala	Lupo,P., Jara,J., Alvis,J.P., Pennington,P., Castillo,L., Moreno,B. and Albrego,L.
EPI_ISL_2571677	Virology, University of Ibadan, College of Medicine (UCH)	Virology, University of Ibadan, College of Medicine (UCH)	Ogunsemowo,O.S., Odaibo,G.N. and Olaleye,D.O.
EPI_ISL_2571682, EPI_ISL_2571683, EPI_ISL_2571684, EPI_ISL_2571685, EPI_ISL_2571686	Epidemiology and Demography, KEMRI - Wellcome Trust Research Programme, Centre for Geographic Medicine Research	Epidemiology and Demography, KEMRI - Wellcome Trust Research Programme, Centre for Geographic Medicine Research	Oketch,J.W., Kamau,E., Otieno,J.R., Mwema,A., Lewa,C., Agoti,C.N. and Nokes,J.D.
EPI_ISL_2571688, EPI_ISL_2571689	Pediatrics, Mackay Memorial Hospital	Pediatrics, Mackay Memorial Hospital	Chi,H., Hsiao,K.-L., Weng,L.-C., Chiu,N.-C., Huang,L.-M., Chiu,Y.-Y., Liu,C.-P. and Liu,H.-F.
EPI_ISL_2571690, EPI_ISL_2571691, EPI_ISL_2571692	Pediatrics, Show-Chwan Memorial Hospital	Pediatrics, Show-Chwan Memorial Hospital	Lee,C.-Y.
EPI_ISL_2571698	Epidemiology and Demography, KEMRI - Wellcome Trust Research Programme, Centre for Geographic Medicine Research	Epidemiology and Demography, KEMRI - Wellcome Trust Research Programme, Centre for Geographic Medicine Research	Oketch,J.W., Kamau,E., Otieno,J.R., Mwema,A., Lewa,C., Agoti,C.N. and Nokes,J.D.
EPI_ISL_2571699, EPI_ISL_2571700, EPI_ISL_2571701, EPI_ISL_2571702	Virology Laboratory, Dr. Ricardo Gutierrez Children Hospital	Virology Laboratory, Dr. Ricardo Gutierrez Children Hospital	Rojo,G.L., Goya,S., Orellana,M., Sancilio,A., Rodriguez Perez,A., Montali,C., Garcia,C., Sosa,L., Musto,A., Hamilton,G., Alvarez,D., Castello,A. and Viegas,M.
EPI_ISL_2571703, EPI_ISL_2571704, EPI_ISL_2571705, EPI_ISL_2571706, EPI_ISL_2571707	Depto Microbiologia, Instituto de Ciencias Biomedicas, Universidade de Sao Paulo	Depto Microbiologia, Instituto de Ciencias Biomedicas, Universidade de Sao Paulo	Thomazelli,L.M., Oliveira,D.B.L., Colmanetti,T., Vieira,S.E., Paulis,M., Ferronato,A.E., Martinez,M.B. and Durigon,E.L.
EPI_ISL_2571709, EPI_ISL_2571710	Chinese Academy of Medical Sciences & Peking Union Medical College	Chinese Academy of Medical Sciences & Peking Union Medical College	Jia,B., Xiao,Y., Wang,Y., Chen,L., Zhang,J., Ren,L. and Wang,J.
EPI_ISL_2571711, EPI_ISL_2571712, EPI_ISL_2571713, EPI_ISL_2571714, EPI_ISL_2571715, EPI_ISL_2571716, EPI_ISL_2571717	WHO WPRO Measles Regional Reference Lab, Key Laboratory of Medical Virology Ministry of Health, National Institute for Viral Disease Control and Prevention, China Center for Disease Control and Prevention	WHO WPRO Measles Regional Reference Lab, Key Laboratory of Medical Virology Ministry of Health, National Institute for Viral Disease Control and Prevention, China Center for Disease Control and Prevention	Zhang,Y., Song,J. and Xu,W.
EPI_ISL_2571718	Institute of Virology and AIDS Research, First Hospital of Jilin University	Institute of Virology and AIDS Research, First Hospital of Jilin University	Zheng,Y., Liu,L., Wang,S., Li,Z., Hou,M., Li,J., Yu,X.-F., Zhang,W. and Hua,S.
EPI_ISL_2571719, EPI_ISL_2571720, EPI_ISL_2571721, EPI_ISL_2571722, EPI_ISL_2571723, EPI_ISL_2571724, EPI_ISL_2571725, EPI_ISL_2571726, EPI_ISL_2571727, EPI_ISL_2571728	WHO WPRO Measles Regional Reference Lab, Key Laboratory of Medical Virology Ministry of Health, National Institute for Viral Disease Control and Prevention, China Center for Disease Control and Prevention	WHO WPRO Measles Regional Reference Lab, Key Laboratory of Medical Virology Ministry of Health, National Institute for Viral Disease Control and Prevention, China Center for Disease Control and Prevention	Zhang,Y., Song,J. and Xu,W.
EPI_ISL_2571729, EPI_ISL_2571730	Institute of Virology and AIDS Research, First Hospital of Jilin University	Institute of Virology and AIDS Research, First Hospital of Jilin University	Zheng,Y., Liu,L., Wang,S., Li,Z., Hou,M., Li,J., Yu,X.-F., Zhang,W. and Hua,S.
EPI_ISL_2571731, EPI_ISL_2571732, EPI_ISL_2571733, EPI_ISL_2571734, EPI_ISL_2571735, EPI_ISL_2571736, EPI_ISL_2571737, EPI_ISL_2571738	WHO WPRO Measles Regional Reference Lab, Key Laboratory of Medical Virology Ministry of Health, National Institute for Viral Disease Control and Prevention, China Center for Disease Control and Prevention	WHO WPRO Measles Regional Reference Lab, Key Laboratory of Medical Virology Ministry of Health, National Institute for Viral Disease Control and Prevention, China Center for Disease Control and Prevention	Zhang,Y., Song,J. and Xu,W.
EPI_ISL_2571756, EPI_ISL_2571757, EPI_ISL_2571758, EPI_ISL_2571759, EPI_ISL_2571760, EPI_ISL_2571761, EPI_ISL_2571763, EPI_ISL_2571767, EPI_ISL_2571774, EPI_ISL_2571775, EPI_ISL_2571776, EPI_ISL_2571778, EPI_ISL_2571780, EPI_ISL_2571781, EPI_ISL_2571784, EPI_ISL_2571785, EPI_ISL_2571786, EPI_ISL_2571788, EPI_ISL_2571789, EPI_ISL_2571790, EPI_ISL_2571792, EPI_ISL_2571793, EPI_ISL_2571796, EPI_ISL_2571797, EPI_ISL_2571798, EPI_ISL_2571799			
see above	University of Wuerzburg, Institute of Virology and Immunobiology	University of Wuerzburg, Institute of Virology and Immunobiology	Prifert,C., Hofmann,D. and Weissbrich,B.
EPI_ISL_2571817, EPI_ISL_2571818, EPI_ISL_2571819, EPI_ISL_2571820, EPI_ISL_2571821	Pediatric Clinic 1, Department of Pathophysiology and Transplantation, University of Milan and Fondazione IRCCS Ca Granda	Pediatric Clinic 1, Department of Pathophysiology and Transplantation, University of Milan and Fondazione IRCCS Ca Granda	Esposito,S., Zampiero,A., Piralla,A. and Principi,N.
EPI_ISL_2571822, EPI_ISL_2571823	Virology, University of Ibadan, College of Medicine (UCH)	Virology, University of Ibadan, College of Medicine (UCH)	Ogunsemowo,O.S., Odaibo,G.N. and Olaleye,D.O.
EPI_ISL_2571825, EPI_ISL_2571826, EPI_ISL_2571827, EPI_ISL_2571828, EPI_ISL_2571829, EPI_ISL_2571830, EPI_ISL_2571831, EPI_ISL_2571832, EPI_ISL_2571833, EPI_ISL_2571834, EPI_ISL_2571835, EPI_ISL_2571836			
see above	Virology, Tohoku University Graduate School of Medicine	Virology, Tohoku University Graduate School of Medicine	Malasao,R., Okamoto,M., Chaimongkol,N., Imamura,T., Tohma,K., Dapat,I., Dapat,C., Suzuki,A., Saito,M., Saito,M., Tamaki,R., Segubre-Mercado,E., Igoy,M.A.U., Lupisan,S., Olveda,R. and Oshitani,H.
EPI_ISL_2571838	Department of Infectious Diseases, National Health Institute Doutor Ricardo Jorge	Department of Infectious Diseases, National Health Institute Doutor Ricardo Jorge	Saez-Lopez,E., Cristovao,P., Costa,I., Pechirra,P., Conde,P. and Guiomar,R.
EPI_ISL_2571855, EPI_ISL_2571856	Microbiology, Hospital Universitari Vall d'Hebron	Microbiology, Hospital Universitari Vall d'Hebron	Gimferrer,L., Martin,Md.C., Pumarola,T. and Anton,A.
EPI_ISL_2571889	Epidemiology and Immunogenetic of Viral Infections LR14SP02, University Hospital Sahloul	Epidemiology and Immunogenetic of Viral Infections LR14SP02, University Hospital Sahloul	Ataoui,I., Jerbi,A., BenHamida-Rebai,M., Ben Hadj Fredj,M., Fodha,I., Bennour,H., Hammouda,H., Khlifa,M., Brini,I., Boussoffara,R., Boussetta,K., Abroug,S. and Trabelsi,A.
EPI_ISL_2571897, EPI_ISL_2571898	Virology Laboratory, Dr. Ricardo Gutierrez Children Hospital	Virology Laboratory, Dr. Ricardo Gutierrez Children Hospital	Rojo,G.L., Goya,S., Orellana,M., Sancilio,A., Rodriguez Perez,A., Montali,C., Garcia,C., Sosa,L., Musto,A., Hamilton,G., Alvarez,D., Castello,A. and Viegas,M.
EPI_ISL_2571899	WHO WPRO Measles Regional Reference Lab, Key Laboratory of Medical Virology Ministry of Health, National Institute for Viral Disease Control and Prevention, China Center for Disease Control and Prevention	WHO WPRO Measles Regional Reference Lab, Key Laboratory of Medical Virology Ministry of Health, National Institute for Viral Disease Control and Prevention, China Center for Disease Control and Prevention	Zhang,Y., Song,J. and Xu,W.
EPI_ISL_2571900	University of Wuerzburg, Institute of Virology and Immunobiology	University of Wuerzburg, Institute of Virology and Immunobiology	Prifert,C., Hofmann,D. and Weissbrich,B.
EPI_ISL_2571904	Influenza and Other Respiratory Viruses Unit, National Center for Microbiology, Instituto de Salud Carlos III	Influenza and Other Respiratory Viruses Unit, National Center for Microbiology, Instituto de Salud Carlos III	Calderon,A., Pozo,F., Calvo,C., Garcia-Garcia,M., Gonzalez,M., Molinero,M. and Casas,I.
EPI_ISL_2571905, EPI_ISL_2571906	Depto Microbiologia, Instituto de Ciencias Biomedicas, Universidade de Sao Paulo	Depto Microbiologia, Instituto de Ciencias Biomedicas, Universidade de Sao Paulo	Moura,F.E.A., Thomazelli,L.M., Campelo,F.S., Delfraro,A., Arbiza,J. and Durigon,E.L.
EPI_ISL_2571909	Laboratory of molecular virology, Research Institute of Influenza, Ministry of Healthcare of the Russian Federation	Laboratory of molecular virology, Research Institute of Influenza, Ministry of Healthcare of the Russian Federation	Komissarova,K.S., Krivitskaya,V.Z., Sverlova,M.V., Petrova,E.R., Fadeev,A.V. and Komissarov,A.B.
EPI_ISL_2571910	Department of Pediatrics, Center of Excellence in Clinical Virology, Faculty of Medicine, Chulalongkorn University	Department of Pediatrics, Center of Excellence in Clinical Virology, Faculty of Medicine, Chulalongkorn University	Thongpan,I., Mauleekoonphairoj,J., Vichiwattana,P., Korkong,S., Vongpunsawad,S. and Poovorawan,Y.
EPI_ISL_2571911	Akinobu Hibino Niigata University, International Health, Public Health	Akinobu Hibino Niigata University, International Health, Public Health	Hibino,A., Saito,R., Shoubugawa,Y. and Saito,T.
EPI_ISL_2571912, EPI_ISL_2571913, EPI_ISL_2571914, EPI_ISL_2571915	Akinobu Hibino Niigata University, International Health, Public Health	Akinobu Hibino Niigata University, International Health, Public Health	Hibino,A., Saito,R., Shoubugawa,Y. and Sano,Y.
EPI_ISL_2571916, EPI_ISL_2571917, EPI_ISL_2571918, EPI_ISL_2571919, EPI_ISL_2571920	Akinobu Hibino Niigata University, International Health, Public Health	Akinobu Hibino Niigata University, International Health, Public Health	Hibino,A., Saito,R., Shoubugawa,Y., Makiya,T. and Takefuta,K.
EPI_ISL_2571921	Akinobu Hibino Niigata University, International Health, Public Health	Akinobu Hibino Niigata University, International Health, Public Health	Hibino,A., Saito,R., Shoubugawa,Y., Azhar,S. and Rohaizat,H.
EPI_ISL_2571922, EPI_ISL_2571923, EPI_ISL_2571924, EPI_ISL_2571925	Akinobu Hibino Niigata University, International Health, Public Health	Akinobu Hibino Niigata University, International Health, Public Health	Hibino,A., Saito,R., Shoubugawa,Y., Htay,H.T., Khin,Y.O., Yi,Y.M. and Yadanar,K.
EPI_ISL_2571926	Wint Wint Phyu Niigat University, International Health, Public Health	Wint Wint Phyu Niigat University, International Health, Public Health	Phyu,W.W. and Saito,R.
EPI_ISL_2571944	Medicine, American University of Beirut	Medicine, American University of Beirut	Abou-El-Hassan,H. and Zaraket,H.
EPI_ISL_2571947	Microbiology, Sanjay Gandhi Post Graduate Institute of Medical Science	Microbiology, Sanjay Gandhi Post Graduate Institute of Medical Science	Saxena,S., Singh,D., Tripathi,R., Dhole,T.N. and Kushwaha,R.
EPI_ISL_2571951	Depto Microbiologia, Instituto de Ciencias Biomedicas, Universidade de Sao Paulo	Depto Microbiologia, Instituto de Ciencias Biomedicas, Universidade de Sao Paulo	Thomazelli,L.M., Oliveira,D.B.L., Colmanetti,T., Vieira,S.E., Paulis,M., Ferronato,A.E., Martinez,M.B. and Durigon,E.L.
EPI_ISL_2571952	Microbiology Department, Virology Division, College of Medicine, Taif University	Microbiology Department, Virology Division, College of Medicine, Taif University	Al Aboud,D.M., Al Aboud,N.M., Al-Malky,M.I.R. and Abdel-Moneim,A.S.
EPI_ISL_2571962, EPI_ISL_2571964, EPI_ISL_2571965	Epidemiology and Demography, KEMRI - Wellcome Trust Research Programme, Centre for Geographic Medicine Research	Epidemiology and Demography, KEMRI - Wellcome Trust Research Programme, Centre for Geographic Medicine Research	Oketch,J.W., Kamau,E., Otieno,J.R., Mwema,A., Lewa,C., Agoti,C.N. and Nokes,J.D.
EPI_ISL_2571966	Virology, Evandro Chagas Institute	Virology, Evandro Chagas Institute	Santos,V.M., Ferreira,J.A., Lima,J.F., Barbagelata,L.S., Souza,E.M.A., Goncalves,M.S., SoNorth America / USA,E.C. Jr., Costa,I.B., Santos,M.C., SoNorth America / USA,R.C.M. and Mello,W.A.
EPI_ISL_2571968	Department of Medical Research, Ministry of Health and Sports, No. 5, Ziwaka Road, Dagon Township	Department of Medical Research, Ministry of Health and Sports, No. 5, Ziwaka Road, Dagon Township	Latt,A.Z., Aye,K.T. and Thwe,H.H.
EPI_ISL_2571970	Pediatrics, Show-Chwan Memorial Hospital	Pediatrics, Show-Chwan Memorial Hospital	Lee,C.-Y.
EPI_ISL_2571973	Department of Pediatrics, Center of Excellence in Clinical Virology, Faculty of	Department of Pediatrics, Center of Excellence in Clinical Virology, Faculty of	Thongpan,I., Mauleekoonphairoj,J., Vichiwattana,P., Korkong,S., Vongpunsawad,S. and Poovorawan,Y.

	Medicine, Chulalongkorn University	Medicine, Chulalongkorn University	
EPI_ISL_2571978, EPI_ISL_2571979	Virology, University of Ibadan, College of Medicine (UCH)	Virology, University of Ibadan, College of Medicine (UCH)	Ogunsemowo,O.S., Odaibo,G.N. and Olaleye,D.O.
EPI_ISL_2571981	Depto Microbiologia, Instituto de Ciencias Biomedicas, Universidade de Sao Paulo	Depto Microbiologia, Instituto de Ciencias Biomedicas, Universidade de Sao Paulo	Moura,F.E.A., Thomazelli,L.M., Campelo,F.S., Delfraro,A., Arbiza,J. and Durigon,E.L.
EPI_ISL_2571982	Depto Microbiologia, Instituto de Ciencias Biomedicas, Universidade de Sao Paulo	Depto Microbiologia, Instituto de Ciencias Biomedicas, Universidade de Sao Paulo	Thomazelli,L.M., Oliveira,D.B.L., Colmanetti,T., Vieira,S.E., Paulis,M., Ferronato,A.E., Martinez,M.B. and Durigon,E.L.
EPI_ISL_2571984, EPI_ISL_2571985	Chinese Academy of Medical Sciences & Peking Union Medical College	Chinese Academy of Medical Sciences & Peking Union Medical College	Jia,B., Xiao,Y., Wang,Y., Chen,L., Zhang,J., Ren,L. and Wang,J.
EPI_ISL_2571986, EPI_ISL_2571987	Institute of Virology and AIDS Research, First Hospital of Jilin University	Institute of Virology and AIDS Research, First Hospital of Jilin University	Zheng,Y., Liu,L., Wang,S., Li,Z., Hou,M., Li,J., Yu,X.-F., Zhang,W. and Hua,S.
EPI_ISL_2571988, EPI_ISL_2571989, EPI_ISL_2571990, EPI_ISL_2571991, EPI_ISL_2571992, EPI_ISL_2571993	WHO WPRO Measles Regional Reference Lab, Key Laboratory of Medical Virology Ministry of Health, National Institute for Viral Disease Control and Prevention, China Center for Disease Control and Prevention	WHO WPRO Measles Regional Reference Lab, Key Laboratory of Medical Virology Ministry of Health, National Institute for Viral Disease Control and Prevention, China Center for Disease Control and Prevention	Zhang,Y., Song,J. and Xu,W.
EPI_ISL_2571994, EPI_ISL_2571995	Institute of Virology and AIDS Research, First Hospital of Jilin University	Institute of Virology and AIDS Research, First Hospital of Jilin University	Zheng,Y., Liu,L., Wang,S., Li,Z., Hou,M., Li,J., Yu,X.-F., Zhang,W. and Hua,S.
EPI_ISL_2571996, EPI_ISL_2571997	WHO WPRO Measles Regional Reference Lab, Key Laboratory of Medical Virology Ministry of Health, National Institute for Viral Disease Control and Prevention, China Center for Disease Control and Prevention	WHO WPRO Measles Regional Reference Lab, Key Laboratory of Medical Virology Ministry of Health, National Institute for Viral Disease Control and Prevention, China Center for Disease Control and Prevention	Zhang,Y., Song,J. and Xu,W.
EPI_ISL_2571998, EPI_ISL_2571999, EPI_ISL_2572003, EPI_ISL_2572007, EPI_ISL_2572008	University of Wuerzburg, Institute of Virology and Immunobiology	University of Wuerzburg, Institute of Virology and Immunobiology	Prifert,C., Hofmann,D. and Weissbrich,B.
EPI_ISL_2572009, EPI_ISL_2572010	Virology, Noguchi Memorial Institute for Medical Research	Virology, Noguchi Memorial Institute for Medical Research	Obodai,E., Odoom,J.K., Adiku,T., Goka,B., Biere,B., Wolff,T., Schweiger,B. and Reiche,J.
EPI_ISL_2572012	Department of Virology, School of Public Health, Tehran University of Medical Sciences	Department of Virology, School of Public Health, Tehran University of Medical Sciences	Salimi,V., Samieipoor,Y., Ghavami,N. and Mokhtariazad,T.
EPI_ISL_2572013	Virology, University of Ibadan, College of Medicine (UCH)	Virology, University of Ibadan, College of Medicine (UCH)	Ogunsemowo,O.S., Odaibo,G.N. and Olaleye,D.O.
EPI_ISL_2572014	Virology, Tohoku University Graduate School of Medicine	Virology, Tohoku University Graduate School of Medicine	Malasao,R., Okamoto,M., Chaimongkol,N., Imamura,T., Tohma,K., Dapat,I., Dapat,C., Suzuki,A., Saito,M., Saito,M., Tamaki,R., Segubre-Mercado,E., Igoy,M.A.U., Lupisan,S., Olveda,R. and Oshitani,H.
EPI_ISL_2572024, EPI_ISL_2572025, EPI_ISL_2572026, EPI_ISL_2572027, EPI_ISL_2572028, EPI_ISL_2572034	Microbiology, Hospital Universitari Vall d'Hebron	Microbiology, Hospital Universitari Vall d'Hebron	Gimferrer,L., Martin,Md.C., Pumarola,T. and Anton,A.
EPI_ISL_2572040	Depto Microbiologia, Instituto de Ciencias Biomedicas, Universidade de Sao Paulo	Depto Microbiologia, Instituto de Ciencias Biomedicas, Universidade de Sao Paulo	Thomazelli,L.M., Oliveira,D.B.L., Colmanetti,T., Vieira,S.E., Paulis,M., Ferronato,A.E., Martinez,M.B. and Durigon,E.L.
EPI_ISL_2572041, EPI_ISL_2572042, EPI_ISL_2572043	Akinobu Hibino Niigata University, International Health, Public Health	Akinobu Hibino Niigata University, International Health, Public Health	Hibino,A., Saito,R., Shoubugawa,Y. and Sano,Y.
EPI_ISL_2572053	Virology, University of Ibadan, College of Medicine (UCH)	Virology, University of Ibadan, College of Medicine (UCH)	Ogunsemowo,O.O., Odaibo,G.N. and Olaleye,D.O.
EPI_ISL_2572055	WHO WPRO Measles Regional Reference Lab, Key Laboratory of Medical Virology Ministry of Health, National Institute for Viral Disease Control and Prevention, China Center for Disease Control and Prevention	WHO WPRO Measles Regional Reference Lab, Key Laboratory of Medical Virology Ministry of Health, National Institute for Viral Disease Control and Prevention, China Center for Disease Control and Prevention	Zhang,Y., Song,J. and Xu,W.
EPI_ISL_2572057	University of Wuerzburg, Institute of Virology and Immunobiology	University of Wuerzburg, Institute of Virology and Immunobiology	Prifert,C., Hofmann,D. and Weissbrich,B.
EPI_ISL_2572064	Virology, Noguchi Memorial Institute for Medical Research	Virology, Noguchi Memorial Institute for Medical Research	Obodai,E., Odoom,J.K., Adiku,T., Goka,B., Biere,B., Wolff,T., Schweiger,B. and Reiche,J.
EPI_ISL_2572065, EPI_ISL_2572066, EPI_ISL_2572067	Akinobu Hibino Niigata University, International Health, Public Health	Akinobu Hibino Niigata University, International Health, Public Health	Hibino,A., Saito,R., Shoubugawa,Y. and Sano,Y.
EPI_ISL_2572068	Akinobu Hibino Niigata University, International Health, Public Health	Akinobu Hibino Niigata University, International Health, Public Health	Hibino,A., Saito,R., Shoubugawa,Y., Ghassan,D. and Hassan,Z.
EPI_ISL_2572069, EPI_ISL_2572070, EPI_ISL_2572071, EPI_ISL_2572072, EPI_ISL_2572073	Akinobu Hibino Niigata University, International Health, Public Health	Akinobu Hibino Niigata University, International Health, Public Health	Hibino,A., Saito,R., Shoubugawa,Y., Htay,H.T., Khin,Y.O., Yi,Y.M. and Yadanar,K.
EPI_ISL_2572075, EPI_ISL_2572076	Medicine, American University of Beirut	Medicine, American University of Beirut	Abou-El-Hassan,H. and Zaraket,H.
EPI_ISL_2572078	Virology, Noguchi Memorial Institute for Medical Research	Virology, Noguchi Memorial Institute for Medical Research	Obodai,E., Odoom,J.K., Adiku,T., Goka,B., Biere,B., Wolff,T., Schweiger,B. and Reiche,J.
EPI_ISL_2572080	Epidemiology and Immunogenetic of Viral Infections LR14SP02, University Hospital Sahloul	Epidemiology and Immunogenetic of Viral Infections LR14SP02, University Hospital Sahloul	Ataoui,I., Jerbi,A., BenHamida-Rebai,M., Ben Hadj Fredj,M., Fodha,I., Bennour,H., Hammouda,H., Khlifa,M., Brini,I., Boussoffara,R., Boussetta,K., Abroug,S. and Trabelsi,A.
EPI_ISL_2572083, EPI_ISL_2572084, EPI_ISL_2572085, EPI_ISL_2572086, EPI_ISL_2572088, EPI_ISL_2572089	Department of Clinical Laboratory, Fujian Provincial Hospital	Department of Clinical Laboratory, Fujian Provincial Hospital	Su,Y., Wu,Y., Tian,R. and Liang,G.
EPI_ISL_2572090	Akinobu Hibino Niigata University, International Health, Public Health	Akinobu Hibino Niigata University, International Health, Public Health	Hibino,A., Saito,R., Shoubugawa,Y. and Sano,Y.
EPI_ISL_2572091	Akinobu Hibino Niigata University, International Health, Public Health	Akinobu Hibino Niigata University, International Health, Public Health	Hibino,A., Saito,R., Shoubugawa,Y., Htay,H.T., Khin,Y.O., Yi,Y.M. and Yadanar,K.
EPI_ISL_2572097	Chinese Academy of Medical Sciences & Peking Union Medical College	Chinese Academy of Medical Sciences & Peking Union Medical College	Jia,B., Xiao,Y., Wang,Y., Chen,L., Zhang,J., Ren,L. and Wang,J.
EPI_ISL_2572098	Microbiology, Hospital Universitari Vall d'Hebron	Microbiology, Hospital Universitari Vall d'Hebron	Gimferrer,L., Martin,Md.C., Pumarola,T. and Anton,A.
EPI_ISL_2572100, EPI_ISL_2572101, EPI_ISL_2572107	Virology, Evandro Chagas Institute	Virology, Evandro Chagas Institute	Santos,V.M., Ferreira,J.A., Lima,J.F., Barbagelata,L.S., Souza,E.M.A., Goncalves,M.S., SoNorth America / USA,E.C. Jr., Costa,I.B., Santos,M.C., SoNorth America / USA,R.C.M. and Mello,W.A.
EPI_ISL_2572110	Akinobu Hibino Niigata University, International Health, Public Health	Akinobu Hibino Niigata University, International Health, Public Health	Hibino,A., Saito,R., Shoubugawa,Y., Ghassan,D. and Hassan,Z.
EPI_ISL_2572111	Medicine, American University of Beirut	Medicine, American University of Beirut	Abou-El-Hassan,H. and Zaraket,H.
EPI_ISL_2572113	Akinobu Hibino Niigata University, International Health, Public Health	Akinobu Hibino Niigata University, International Health, Public Health	Hibino,A., Saito,R., Shoubugawa,Y., Ghassan,D. and Hassan,Z.
EPI_ISL_2572114	Medicine, American University of Beirut	Medicine, American University of Beirut	Abou-El-Hassan,H. and Zaraket,H.
EPI_ISL_2572116	Akinobu Hibino Niigata University, International Health, Public Health	Akinobu Hibino Niigata University, International Health, Public Health	Hibino,A., Saito,R., Shoubugawa,Y., Ghassan,D. and Hassan,Z.
EPI_ISL_2572117	Medicine, American University of Beirut	Medicine, American University of Beirut	Abou-El-Hassan,H. and Zaraket,H.
EPI_ISL_2572118, EPI_ISL_2572119, EPI_ISL_2572120, EPI_ISL_2572121, EPI_ISL_2572122, EPI_ISL_2572123, EPI_ISL_2572124	Virology Laboratory, Dr. Ricardo Gutierrez Children Hospital	Virology Laboratory, Dr. Ricardo Gutierrez Children Hospital	Rojo,G.L., Goya,S., Orellana,M., Sancilio,A., Rodriguez Perez,A., Montali,C., Garcia,C., Sosa,L., Musto,A., Hamilton,G., Alvarez,D., Castello,A. and Viegas,M.
EPI_ISL_2572125, EPI_ISL_2572126, EPI_ISL_2572127, EPI_ISL_2572128, EPI_ISL_2572129, EPI_ISL_2572130, EPI_ISL_2572131	Health Science, Universidade Federal Do Parana	Health Science, Universidade Federal Do Parana	Moreira,F.B., Santos,J.S., Avanzi,V.M., Nogueira,M.B., Vidal,L.R.R. and Raboni,S.M.
EPI_ISL_2572136, EPI_ISL_2572137, EPI_ISL_2572138, EPI_ISL_2572139, EPI_ISL_2572140, EPI_ISL_2572141, EPI_ISL_2572142, EPI_ISL_2572143, EPI_ISL_2572144	Depto Microbiologia, Instituto de Ciencias Biomedicas, Universidade de Sao Paulo	Depto Microbiologia, Instituto de Ciencias Biomedicas, Universidade de Sao Paulo	Moura,F.E.A., Thomazelli,L.M., Campelo,F.S., Delfraro,A., Arbiza,J. and Durigon,E.L.
EPI_ISL_2572145, EPI_ISL_2572146, EPI_ISL_2572147, EPI_ISL_2572148, EPI_ISL_2572149, EPI_ISL_2572150, EPI_ISL_2572151, EPI_ISL_2572152, EPI_ISL_2572153, EPI_ISL_2572154, EPI_ISL_2572155, EPI_ISL_2572156, EPI_ISL_2572157, EPI_ISL_2572158, EPI_ISL_2572159, EPI_ISL_2572160, EPI_ISL_2572161, EPI_ISL_2572162, EPI_ISL_2572163, EPI_ISL_2572164, EPI_ISL_2572165, EPI_ISL_2572166, EPI_ISL_2572167			
see above	Depto Microbiologia, Instituto de Ciencias Biomedicas, Universidade de Sao Paulo	Depto Microbiologia, Instituto de Ciencias Biomedicas, Universidade de Sao Paulo	Thomazelli,L.M., Oliveira,D.B.L., Colmanetti,T., Vieira,S.E., Paulis,M., Ferronato,A.E., Martinez,M.B. and Durigon,E.L.
EPI_ISL_2572200, EPI_ISL_2572201, EPI_ISL_2572202, EPI_ISL_2572203, EPI_ISL_2572204, EPI_ISL_2572209, EPI_ISL_2572210, EPI_ISL_2572211, EPI_ISL_2572212, EPI_ISL_2572213, EPI_ISL_2572214, EPI_ISL_2572215, EPI_ISL_2572216, EPI_ISL_2572217, EPI_ISL_2572219, EPI_ISL_2572220, EPI_ISL_2572221, EPI_ISL_2572222, EPI_ISL_2572223, EPI_ISL_2572224			
see above	Chinese Academy of Medical Sciences & Peking Union Medical College	Chinese Academy of Medical Sciences & Peking Union Medical College	Jia,B., Xiao,Y., Wang,Y., Chen,L., Zhang,J., Ren,L. and Wang,J.
EPI_ISL_2572230, EPI_ISL_2572231, EPI_ISL_2572232, EPI_ISL_2572233, EPI_ISL_2572234, EPI_ISL_2572235, EPI_ISL_2572236, EPI_ISL_2572237	WHO WPRO Measles Regional Reference Lab, Key Laboratory of Medical Virology Ministry of Health, National Institute for Viral Disease Control and Prevention, China Center for Disease Control and Prevention	WHO WPRO Measles Regional Reference Lab, Key Laboratory of Medical Virology Ministry of Health, National Institute for Viral Disease Control and Prevention, China Center for Disease Control and Prevention	Zhang,Y., Song,J. and Xu,W.
EPI_ISL_2572239, EPI_ISL_2572240, EPI_ISL_2572241, EPI_ISL_2572242, EPI_ISL_2572243, EPI_ISL_2572244, EPI_ISL_2572245, EPI_ISL_2572246, EPI_ISL_2572247, EPI_ISL_2572248, EPI_ISL_2572249, EPI_ISL_2572250, EPI_ISL_2572251, EPI_ISL_2572252, EPI_ISL_2572253, EPI_ISL_2572254, EPI_ISL_2572255, EPI_ISL_2572256, EPI_ISL_2572257			
see above	Laboratory of Virology, Capital Institute of Pediatrics	Laboratory of Virology, Capital Institute of Pediatrics	Cui,G., Zhu,R., Qian,Y., Deng,J., Zhao,L., Sun,Y. and Wang,F.
EPI_ISL_2572258, EPI_ISL_2572259, EPI_ISL_2572260, EPI_ISL_2572261, EPI_ISL_2572263, EPI_ISL_2572268	Institute of Virology and AIDS Research, First Hospital of Jilin University	Institute of Virology and AIDS Research, First Hospital of Jilin University	Zheng,Y., Liu,L., Wang,S., Li,Z., Hou,M., Li,J., Yu,X.-F., Zhang,W. and Hua,S.
EPI_ISL_2572269, EPI_ISL_2572270	WHO WPRO Measles Regional Reference Lab, Key Laboratory of Medical Virology Ministry of Health, National Institute for Viral Disease Control and Prevention, China Center for Disease Control and Prevention	WHO WPRO Measles Regional Reference Lab, Key Laboratory of Medical Virology Ministry of Health, National Institute for Viral Disease Control and Prevention, China Center for Disease Control and Prevention	Zhang,Y., Song,J. and Xu,W.
EPI_ISL_2572271	WHO WPRO Measles Regional Reference Lab, Key Laboratory of Medical Virology Ministry of Health, National Institute for Viral Disease Control and Prevention, China Center for Disease Control and Prevention	WHO WPRO Measles Regional Reference Lab, Key Laboratory of Medical Virology Ministry of Health, National Institute for Viral Disease Control and Prevention, China Center for Disease Control and Prevention	Song,J., Zhang,Y. and Xu,W.
EPI_ISL_2572272, EPI_ISL_2572273, EPI_ISL_2572274, EPI_ISL_2572275	WHO WPRO Measles Regional Reference Lab, Key Laboratory of Medical Virology Ministry of Health, National Institute for Viral Disease Control and Prevention, China Center for Disease Control and Prevention	WHO WPRO Measles Regional Reference Lab, Key Laboratory of Medical Virology Ministry of Health, National Institute for Viral Disease Control and Prevention, China Center for Disease Control and Prevention	Zhang,Y., Song,J. and Xu,W.

EPI_ISL_2572276	WHO WPRO Measles Regional Reference Lab, Key Laboratory of Medical Virology Ministry of Health, National Institute for Viral Disease Control and Prevention, China Center for Disease Control and Prevention	WHO WPRO Measles Regional Reference Lab, Key Laboratory of Medical Virology Ministry of Health, National Institute for Viral Disease Control and Prevention, China Center for Disease Control and Prevention	Song,J., Zhang,Y. and Xu,W.
EPI_ISL_2572277	WHO WPRO Measles Regional Reference Lab, Key Laboratory of Medical Virology Ministry of Health, National Institute for Viral Disease Control and Prevention, China Center for Disease Control and Prevention	WHO WPRO Measles Regional Reference Lab, Key Laboratory of Medical Virology Ministry of Health, National Institute for Viral Disease Control and Prevention, China Center for Disease Control and Prevention	Zhang,Y., Song,J. and Xu,W.
EPI_ISL_2572278	WHO WPRO Measles Regional Reference Lab, Key Laboratory of Medical Virology Ministry of Health, National Institute for Viral Disease Control and Prevention, China Center for Disease Control and Prevention	WHO WPRO Measles Regional Reference Lab, Key Laboratory of Medical Virology Ministry of Health, National Institute for Viral Disease Control and Prevention, China Center for Disease Control and Prevention	Song,J., Zhang,Y. and Xu,W.
EPI_ISL_2572279, EPI_ISL_2572280, EPI_ISL_2572281	WHO WPRO Measles Regional Reference Lab, Key Laboratory of Medical Virology Ministry of Health, National Institute for Viral Disease Control and Prevention, China Center for Disease Control and Prevention	WHO WPRO Measles Regional Reference Lab, Key Laboratory of Medical Virology Ministry of Health, National Institute for Viral Disease Control and Prevention, China Center for Disease Control and Prevention	Zhang,Y., Song,J. and Xu,W.
EPI_ISL_2572282	Institute of Virology and AIDS Research, First Hospital of Jilin University	Institute of Virology and AIDS Research, First Hospital of Jilin University	Zheng,Y., Liu,L., Wang,S., Li,Z., Hou,M., Li,J., Yu,X.-F., Zhang,W. and Hua,S.
EPI_ISL_2572283, EPI_ISL_2572284	WHO WPRO Measles Regional Reference Lab, Key Laboratory of Medical Virology Ministry of Health, National Institute for Viral Disease Control and Prevention, China Center for Disease Control and Prevention	WHO WPRO Measles Regional Reference Lab, Key Laboratory of Medical Virology Ministry of Health, National Institute for Viral Disease Control and Prevention, China Center for Disease Control and Prevention	Zhang,Y., Song,J. and Xu,W.
EPI_ISL_2572286, EPI_ISL_2572289, EPI_ISL_2572292, EPI_ISL_2572293, EPI_ISL_2572294, EPI_ISL_2572295, EPI_ISL_2572297, EPI_ISL_2572300, EPI_ISL_2572301, EPI_ISL_2572302, EPI_ISL_2572308, EPI_ISL_2572309, EPI_ISL_2572310, EPI_ISL_2572311, EPI_ISL_2572312, EPI_ISL_2572314, EPI_ISL_2572316, EPI_ISL_2572317, EPI_ISL_2572318, EPI_ISL_2572320, EPI_ISL_2572321, EPI_ISL_2572322, EPI_ISL_2572323, EPI_ISL_2572324, EPI_ISL_2572325, EPI_ISL_2572326, EPI_ISL_2572327			
see above	University of Wuerzburg, Institute of Virology and Immunobiology	University of Wuerzburg, Institute of Virology and Immunobiology	Prifert,C., Hofmann,D. and Weissbrich,B.
EPI_ISL_2572328, EPI_ISL_2572329, EPI_ISL_2572330	Virology, Noguchi Memorial Institute for Medical Research	Virology, Noguchi Memorial Institute for Medical Research	Obodai,E., Odoom,J.K., Adiku,T., Goka,B., Biere,B., Wolff,T., Schweiger,B. and Reiche,J.
EPI_ISL_2572341, EPI_ISL_2572342	Department of Virology, School of Public Health, Tehran University of Medical Sciences	Department of Virology, School of Public Health, Tehran University of Medical Sciences	Salimi,V., Samieipoor,Y., Ghavami,N. and Mokhtariazad,T.
EPI_ISL_2572347, EPI_ISL_2572348, EPI_ISL_2572349, EPI_ISL_2572350, EPI_ISL_2572351, EPI_ISL_2572352, EPI_ISL_2572353, EPI_ISL_2572355, EPI_ISL_2572356, EPI_ISL_2572357, EPI_ISL_2572358, EPI_ISL_2572359, EPI_ISL_2572360, EPI_ISL_2572361, EPI_ISL_2572362, EPI_ISL_2572363, EPI_ISL_2572364, EPI_ISL_2572365, EPI_ISL_2572366, EPI_ISL_2572367, EPI_ISL_2572368, EPI_ISL_2572369, EPI_ISL_2572370, EPI_ISL_2572371, EPI_ISL_2572372, EPI_ISL_2572373, EPI_ISL_2572374, EPI_ISL_2572375, EPI_ISL_2572376, EPI_ISL_2572377, EPI_ISL_2572378, EPI_ISL_2572379, EPI_ISL_2572380, EPI_ISL_2572381			
see above	Pediatric Clinic 1, Department of Pathophysiology and Transplantation, University of Milan and Fondazione IRCCS Ca Granda	Pediatric Clinic 1, Department of Pathophysiology and Transplantation, University of Milan and Fondazione IRCCS Ca Granda	Esposito,S., Zampiero,A., Piralla,A. and Principi,N.
EPI_ISL_2572382, EPI_ISL_2572383, EPI_ISL_2572384, EPI_ISL_2572385, EPI_ISL_2572386, EPI_ISL_2572389, EPI_ISL_2572390, EPI_ISL_2572391, EPI_ISL_2572392, EPI_ISL_2572393	Virology, Tohoku University Graduate School of Medicine	Virology, Tohoku University Graduate School of Medicine	Malasao,R., Okamoto,M., Chaimongkol,N., Imamura,T., Tohma,K., Dapat,I., Dapat,C., Suzuki,A., Saito,M., Saito,M., Tamaki,R., Segubre-Mercado,E., Igoy,M.A.U., Lupisan,S., Olveda,R. and Oshitani,H.
EPI_ISL_2572436, EPI_ISL_2572437, EPI_ISL_2572438, EPI_ISL_2572439, EPI_ISL_2572440, EPI_ISL_2572441, EPI_ISL_2572442, EPI_ISL_2572443, EPI_ISL_2572444, EPI_ISL_2572445, EPI_ISL_2572446, EPI_ISL_2572447, EPI_ISL_2572448, EPI_ISL_2572449, EPI_ISL_2572450, EPI_ISL_2572451, EPI_ISL_2572452, EPI_ISL_2572490, EPI_ISL_2572491, EPI_ISL_2572492, EPI_ISL_2572493, EPI_ISL_2572494, EPI_ISL_2572495			
see above	Microbiology, Hospital Universitari Vall d'Hebron	Microbiology, Hospital Universitari Vall d'Hebron	Gimferrer,L., Martin,Md.C., Pumarola,T. and Anton,A.
EPI_ISL_2572521	Epidemiology and Immunogenetic of Viral Infections LR14SP02, University Hospital Sahloul	Epidemiology and Immunogenetic of Viral Infections LR14SP02, University Hospital Sahloul	Ataoui,I., Jerbi,A., BenHamida-Rebai,M., Ben Hadj Fredj,M., Fodha,I., Bennour,H., Hammouda,H., Khlifa,M., Brini,I., Boussoffara,R., Boussetta,K., Abroug,S. and Trabelsi,A.
EPI_ISL_2572538	Virology Laboratory, Dr. Ricardo Gutierrez Children Hospital	Virology Laboratory, Dr. Ricardo Gutierrez Children Hospital	Rojo,G.L., Goya,S., Orellana,M., Sancilio,A., Rodriguez Perez,A., Montali,C., Garcia,C., Sosa,L., Musto,A., Hamilton,G., Alvarez,D., Castello,A. and Viegas,M.
EPI_ISL_2572540	Virology, Noguchi Memorial Institute for Medical Research	Virology, Noguchi Memorial Institute for Medical Research	Obodai,E., Odoom,J.K., Adiku,T., Goka,B., Biere,B., Wolff,T., Schweiger,B. and Reiche,J.
EPI_ISL_2572543, EPI_ISL_2572547	Chinese Academy of Medical Sciences & Peking Union Medical College	Chinese Academy of Medical Sciences & Peking Union Medical College	Jia,B., Xiao,Y., Wang,Y., Chen,L., Zhang,J., Ren,L. and Wang,J.
EPI_ISL_2572552, EPI_ISL_2572553, EPI_ISL_2572554, EPI_ISL_2572555, EPI_ISL_2572556	Department of Clinical Laboratory, Fujian Provincial Hospital	Department of Clinical Laboratory, Fujian Provincial Hospital	Su,Y., Wu,Y., Tian,R. and Liang,G.
EPI_ISL_2572561, EPI_ISL_2572562, EPI_ISL_2572563	Lab. of Molecular Virology, Research Institute of Influenza	Lab. of Molecular Virology, Research Institute of Influenza	Sintsova,K.S., Krivitskaya,V.Z., Sverlova,M.V., Petrova,E.R., Fadeev,A.V. and Komissarov,A.B.
EPI_ISL_2572568, EPI_ISL_2572569, EPI_ISL_2572570	Influenza and Other Respiratory Viruses Unit, National Center for Microbiology, Instituto de Salud Carlos III	Influenza and Other Respiratory Viruses Unit, National Center for Microbiology, Instituto de Salud Carlos III	Calderon,A., Pozo,F., Calvo,C., Garcia-Garcia,M., Gonzalez,M., Molinero,M. and Casas,I.
EPI_ISL_2572571, EPI_ISL_2572572, EPI_ISL_2572573, EPI_ISL_2572574, EPI_ISL_2572575, EPI_ISL_2572576, EPI_ISL_2572577, EPI_ISL_2572578, EPI_ISL_2572579, EPI_ISL_2572580, EPI_ISL_2572581			
see above	Department of Pediatrics, Center of Excellence in Clinical Virology, Faculty of Medicine, Chulalongkorn University	Department of Pediatrics, Center of Excellence in Clinical Virology, Faculty of Medicine, Chulalongkorn University	Thongpan,I., Mauleekoonphairoj,J., Vichiwattana,P., Korkong,S., Vongpunsawad,S. and Poovorawan,Y.
EPI_ISL_2572592, EPI_ISL_2572593, EPI_ISL_2572594	Miho Kobayashi Gunma Prefectural Institute of Public Health and Environmental Sciences	Miho Kobayashi Gunma Prefectural Institute of Public Health and Environmental Sciences	Kobayashi,M. and Kimura,H.
EPI_ISL_2572595, EPI_ISL_2572596, EPI_ISL_2572597, EPI_ISL_2572598, EPI_ISL_2572599, EPI_ISL_2572600, EPI_ISL_2572601, EPI_ISL_2572602, EPI_ISL_2572603, EPI_ISL_2572604, EPI_ISL_2572605, EPI_ISL_2572606, EPI_ISL_2572607, EPI_ISL_2572608, EPI_ISL_2572609, EPI_ISL_2572610, EPI_ISL_2572611, EPI_ISL_2572612, EPI_ISL_2572613, EPI_ISL_2572614, EPI_ISL_2572615, EPI_ISL_2572616			
see above	Eiko Hirano Fukui Prefectural Institute of Public Health and Environmental Science	Eiko Hirano Fukui Prefectural Institute of Public Health and Environmental Science	Hirano,E. and Kobayashi,M.
EPI_ISL_2572617	Akinobu Hibino Niigata University, International Health, Public Health	Akinobu Hibino Niigata University, International Health, Public Health	Hibino,A., Saito,R., Shoubugawa,Y. and Takei,T.
EPI_ISL_2572618, EPI_ISL_2572619, EPI_ISL_2572620, EPI_ISL_2572621, EPI_ISL_2572622, EPI_ISL_2572623	Akinobu Hibino Niigata University, International Health, Public Health	Akinobu Hibino Niigata University, International Health, Public Health	Hibino,A., Saito,R., Shoubugawa,Y. and Shimada,Y.
EPI_ISL_2572624, EPI_ISL_2572625	Akinobu Hibino Niigata University, International Health, Public Health	Akinobu Hibino Niigata University, International Health, Public Health	Hibino,A., Saito,R., Shoubugawa,Y. and Taniguchi,K.
EPI_ISL_2572626	Akinobu Hibino Niigata University, International Health, Public Health	Akinobu Hibino Niigata University, International Health, Public Health	Hibino,A., Saito,R., Shoubugawa,Y. and Sato,I.
EPI_ISL_2572627, EPI_ISL_2572628, EPI_ISL_2572629, EPI_ISL_2572630	Akinobu Hibino Niigata University, International Health, Public Health	Akinobu Hibino Niigata University, International Health, Public Health	Hibino,A., Saito,R., Shoubugawa,Y. and Sano,Y.
EPI_ISL_2572631, EPI_ISL_2572632, EPI_ISL_2572633, EPI_ISL_2572634, EPI_ISL_2572635, EPI_ISL_2572636, EPI_ISL_2572637, EPI_ISL_2572638, EPI_ISL_2572639, EPI_ISL_2572640, EPI_ISL_2572641, EPI_ISL_2572642, EPI_ISL_2572643			
see above	Akinobu Hibino Niigata University, International Health, Public Health	Akinobu Hibino Niigata University, International Health, Public Health	Hibino,A., Saito,R., Shoubugawa,Y., Makiya,T. and Takefuta,K.
EPI_ISL_2572644, EPI_ISL_2572645, EPI_ISL_2572646, EPI_ISL_2572647, EPI_ISL_2572648, EPI_ISL_2572649, EPI_ISL_2572650, EPI_ISL_2572651, EPI_ISL_2572652	Akinobu Hibino Niigata University, International Health, Public Health	Akinobu Hibino Niigata University, International Health, Public Health	Hibino,A., Saito,R., Shoubugawa,Y., Htay,H.T., Khin,Y.O., Yi,Y.M. and Yadanar,K.
EPI_ISL_2572668	Lab. of Molecular Virology, Research Institute of Influenza	Lab. of Molecular Virology, Research Institute of Influenza	Sintsova,K.S., Krivitskaya,V.Z., Sverlova,M.V., Petrova,E.R., Fadeev,A.V. and Komissarov,A.B.
EPI_ISL_2572669, EPI_ISL_2572670, EPI_ISL_2572671, EPI_ISL_2572672, EPI_ISL_2572673, EPI_ISL_2572674	Medicine, American University of Beirut	Medicine, American University of Beirut	Abou-El-Hassan,H. and Zaraket,H.
EPI_ISL_2572694	Department of Pediatrics, Center of Excellence in Clinical Virology, Faculty of Medicine, Chulalongkorn University	Department of Pediatrics, Center of Excellence in Clinical Virology, Faculty of Medicine, Chulalongkorn University	Thongpan,I., Mauleekoonphairoj,J., Vichiwattana,P., Korkong,S., Vongpunsawad,S. and Poovorawan,Y.
EPI_ISL_2572709, EPI_ISL_2572710, EPI_ISL_2572711	Department of Clinical Laboratory, Fujian Provincial Hospital	Department of Clinical Laboratory, Fujian Provincial Hospital	Su,Y., Wu,Y., Tian,R. and Liang,G.
EPI_ISL_2572718	Virology, Evandro Chagas Institute	Virology, Evandro Chagas Institute	Santos,V.M., Ferreira,J.A., Lima,J.F., Barbagelata,L.S., Souza,E.M.A., Goncalves,M.S., SoNorth America / USA,E.C. Jr., Costa,I.B., Santos,M.C., SoNorth America / USA,R.C.M. and Mello,W.A.
EPI_ISL_2572732	Chinese Academy of Medical Sciences & Peking Union Medical College	Chinese Academy of Medical Sciences & Peking Union Medical College	Jia,B., Xiao,Y., Wang,Y., Chen,L., Zhang,J., Ren,L. and Wang,J.
EPI_ISL_2572736	Laboratory of Virology, Capital Institute of Pediatrics	Laboratory of Virology, Capital Institute of Pediatrics	Cui,G., Zhu,R., Qian,Y., Deng,J., Zhao,L., Sun,Y. and Wang,F.
EPI_ISL_2572737, EPI_ISL_2572739	Lab. of Molecular Virology, Research Institute of Influenza	Lab. of Molecular Virology, Research Institute of Influenza	Sintsova,K.S., Krivitskaya,V.Z., Sverlova,M.V., Petrova,E.R., Fadeev,A.V. and Komissarov,A.B.
EPI_ISL_2572745, EPI_ISL_2572746	Influenza and Other Respiratory Viruses Unit, National Center for Microbiology, Instituto de Salud Carlos III	Influenza and Other Respiratory Viruses Unit, National Center for Microbiology, Instituto de Salud Carlos III	Calderon,A., Pozo,F., Calvo,C., Garcia-Garcia,M., Gonzalez,M., Molinero,M. and Casas,I.
EPI_ISL_2572750	Division of Public Health Research, Gyeonggi Province institute of Health and Environment	Division of Public Health Research, Gyeonggi Province institute of Health and Environment	Park,E., Park,P., Huh,J., Yun,H., Lee,H., Yoon,M., Lee,S. and Ko,G.
EPI_ISL_2572753, EPI_ISL_2572754, EPI_ISL_2572755, EPI_ISL_2572756	Department of Epidemiology and Demography, Kenya Medical Research Institute (KEMRI) - Wellcome Trust Research Programme	Department of Epidemiology and Demography, Kenya Medical Research Institute (KEMRI) - Wellcome Trust Research Programme	Otieno,J.R., Kamau,E.M., Agoti,C.N., Lewa,C., Bett,A., Ngama,M., Cane,P.A. and Nokes,J.D.
EPI_ISL_2572757	Botany and Microbiology, College of Science, King Saud University, King Saud University	Botany and Microbiology, College of Science, King Saud University, King Saud University	Amer,H.M., Farrag,M.A., Hamad,M.E., Aziz,I.M. and Almajhdi,F.N.
EPI_ISL_2572759	Epidemiology and Demography, KEMRI - Wellcome Trust Research Programme, Centre for Geographic Medicine Research	Epidemiology and Demography, KEMRI - Wellcome Trust Research Programme, Centre for Geographic Medicine Research	Oketch,J.W., Kamau,E., Otieno,J.R., Mwema,A., Lewa,C., Agoti,C.N. and Nokes,J.D.
EPI_ISL_2572760, EPI_ISL_2572761, EPI_ISL_2572762, EPI_ISL_2572763, EPI_ISL_2572764	Molecular Medicine Laboratory of Virology, Sapienza University	Molecular Medicine Laboratory of Virology, Sapienza University	Pierangeli,A.
EPI_ISL_2572768, EPI_ISL_2572769, EPI_ISL_2572770	Pediatrics, Show-Chwan Memorial Hospital	Pediatrics, Show-Chwan Memorial Hospital	Lee,C.-Y.

EPI_ISL_2572776, EPI_ISL_2572777	Pediatrics, Mackay Memorial Hospital	Pediatrics, Mackay Memorial Hospital	Chi,H., Hsiao,K.-L., Weng,L.-C., Chiu,N.-C., Huang,L.-M., Chiu,Y.-Y., Liu,C.-P. and Liu,H.-F.
EPI_ISL_2572778	Pediatrics, Show-Chwan Memorial Hospital	Pediatrics, Show-Chwan Memorial Hospital	Lee,C.-Y.
EPI_ISL_2572783, EPI_ISL_2572784, EPI_ISL_2572785, EPI_ISL_2572786, EPI_ISL_2572787, EPI_ISL_2572788, EPI_ISL_2572789, EPI_ISL_2572790, EPI_ISL_2572791, EPI_ISL_2572792, EPI_ISL_2572793			
see above	Virology Laboratory, Dr. Ricardo Gutierrez Children Hospital	Virology Laboratory, Dr. Ricardo Gutierrez Children Hospital	Rojo,G.L., Goya,S., Orellana,M., Sancilio,A., Rodriguez Perez,A., Montali,C., Garcia,C., Sosa,L., Musto,A., Hamilton,G., Alvarez,D., Castello,A. and Viegas,M.
EPI_ISL_2572797	Department of Pediatrics, Center of Excellence in Clinical Virology, Faculty of Medicine, Chulalongkorn University	Department of Pediatrics, Center of Excellence in Clinical Virology, Faculty of Medicine, Chulalongkorn University	Thongpan,I., Mauleekoonphairoj,J., Vichiwattana,P., Korkong,S., Vongpunsawad,S. and Poovorawan,Y.
EPI_ISL_2572805	Centro de Estudios en Salud, Universidad Del Valle De Guatemala	Centro de Estudios en Salud, Universidad Del Valle De Guatemala	Lupo,P., Jara,J., Alvis,J.P., Pennington,P., Castillo,L., Moreno,B. and Albrego,L.
EPI_ISL_2572806	Centre for Infectious Diseases Research, Diagnostics and laboratory Surveillance, National Institute for Public Health and the Environment	Centre for Infectious Diseases Research, Diagnostics and laboratory Surveillance, National Institute for Public Health and the Environment	Meijer,A. and Overduin,P.
EPI_ISL_2572808	Virology Department, Hospices Civils de Lyon	Virology Department, Hospices Civils de Lyon	Gaymard,A., Pichon,M. and Morfin,F.
EPI_ISL_2572809, EPI_ISL_2572810	Centro de Estudios en Salud, Universidad Del Valle De Guatemala	Centro de Estudios en Salud, Universidad Del Valle De Guatemala	Lupo,P., Jara,J., Alvis,J.P., Pennington,P., Castillo,L., Moreno,B. and Albrego,L.
EPI_ISL_2572812, EPI_ISL_2572813, EPI_ISL_2572814	Chinese Academy of Medical Sciences & Peking Union Medical College	Chinese Academy of Medical Sciences & Peking Union Medical College	Jia,B., Xiao,Y., Wang,Y., Chen,L., Zhang,J., Ren,L. and Wang,J.
EPI_ISL_2572817	Centre for Research And Knowledge Transfer in Biotechnology, University of Zagreb	Centre for Research And Knowledge Transfer in Biotechnology, University of Zagreb	Jagusic,M., Slovic,A., Santak,M., Kosutic-Gulija,T., Ivancic-Jelecki,J. and Forcic,D.
EPI_ISL_2572818	Centre for Research And Knowledge Transfer in Biotechnology, University of Zagreb	Centre for Research And Knowledge Transfer in Biotechnology, University of Zagreb	Ivancic-Jelecki,J., Slovic,A., Ljubin-Sternak,S., Mlinaric-Galinovic,G. and Forcic,D.
EPI_ISL_2572820	Department of Medical Research, Ministry of Health and Sports, No. 5, Ziwaka Road, Dagon Township	Department of Medical Research, Ministry of Health and Sports, No. 5, Ziwaka Road, Dagon Township	Aye,K.T., Latt,A.Z. and Thwe,H.H.
EPI_ISL_2572821	Department of Medical Research, Ministry of Health and Sports, No. 5, Ziwaka Road, Dagon Township	Department of Medical Research, Ministry of Health and Sports, No. 5, Ziwaka Road, Dagon Township	Latt,A.Z., Aye,K.T. and Thwe,H.H.
EPI_ISL_2572822	Department of Medical Research, Ministry of Health and Sports, No. 5, Ziwaka Road, Dagon Township	Department of Medical Research, Ministry of Health and Sports, No. 5, Ziwaka Road, Dagon Township	Aye,K.T.
EPI_ISL_2572823	Lab. of Molecular Virology, Research Institute of Influenza	Lab. of Molecular Virology, Research Institute of Influenza	Sintsova,K.S., Krivitskaya,V.Z., Sverlova,M.V., Petrova,E.R., Fadeev,A.V. and Komissarov,A.B.
EPI_ISL_2572826	Virology Department, National Center of Microbiology, Instituto de Salud Carlos III	Virology Department, National Center of Microbiology, Instituto de Salud Carlos III	Casas,I., Calderon,A., Pozo,F., Calvo,C., Garcia-Garcia,M., Gonzalez,M. and Molinero,M.
EPI_ISL_2572829	WHO WPRO Measles Regional Reference Lab, Key Laboratory of Medical Virology Ministry of Health, National Institute for Viral Disease Control and Prevention, China Center for Disease Control and Prevention	WHO WPRO Measles Regional Reference Lab, Key Laboratory of Medical Virology Ministry of Health, National Institute for Viral Disease Control and Prevention, China Center for Disease Control and Prevention	Song,J., Zhang,Y. and Xu,W.
EPI_ISL_2572831	Laboratory of molecular virology, Research Institute of Influenza, Ministry of Healthcare of the Russian Federation	Laboratory of molecular virology, Research Institute of Influenza, Ministry of Healthcare of the Russian Federation	Komissarova,K.S., Krivitskaya,V.Z., Sverlova,M.V., Petrova,E.R., Fadeev,A.V. and Komissarov,A.B.
EPI_ISL_2572832	Virology, University of Ibadan, College of Medicine (UCH)	Virology, University of Ibadan, College of Medicine (UCH)	Ogunsemowo,O.S., Odaibo,G.N. and Olaleye,D.O.
EPI_ISL_2572833	Virology, University of Ibadan, College of Medicine (UCH)	Virology, University of Ibadan, College of Medicine (UCH)	Ogunsemowo,O.O., Odaibo,G.N. and Olaleye,D.O.
EPI_ISL_2572834	Lab. of Molecular Virology, Research Institute of Influenza	Lab. of Molecular Virology, Research Institute of Influenza	Sintsova,K.S., Krivitskaya,V.Z., Sverlova,M.V., Petrova,E.R., Fadeev,A.V. and Komissarov,A.B.
EPI_ISL_2572836	Centro de Estudios en Salud, Universidad Del Valle De Guatemala	Centro de Estudios en Salud, Universidad Del Valle De Guatemala	Lupo,P., Jara,J., Alvis,J.P., Pennington,P., Castillo,L., Moreno,B. and Albrego,L.
EPI_ISL_2572837	Virology, University of Ibadan, College of Medicine (UCH)	Virology, University of Ibadan, College of Medicine (UCH)	Ogunsemowo,O.S., Odaibo,G.N. and Olaleye,D.O.
EPI_ISL_2572842, EPI_ISL_2572843, EPI_ISL_2572844, EPI_ISL_2572845, EPI_ISL_2572846	Epidemiology and Demography, KEMRI - Wellcome Trust Research Programme, Centre for Geographic Medicine Research	Epidemiology and Demography, KEMRI - Wellcome Trust Research Programme, Centre for Geographic Medicine Research	Oketch,J.W., Kamau,E., Otieno,J.R., Mwema,A., Lewa,C., Agoti,C.N. and Nokes,J.D.
EPI_ISL_2572848, EPI_ISL_2572849	Pediatrics, Mackay Memorial Hospital	Pediatrics, Mackay Memorial Hospital	Chi,H., Hsiao,K.-L., Weng,L.-C., Chiu,N.-C., Huang,L.-M., Chiu,Y.-Y., Liu,C.-P. and Liu,H.-F.
EPI_ISL_2572850, EPI_ISL_2572851, EPI_ISL_2572852	Pediatrics, Show-Chwan Memorial Hospital	Pediatrics, Show-Chwan Memorial Hospital	Lee,C.-Y.
EPI_ISL_2572858	Epidemiology and Demography, KEMRI - Wellcome Trust Research Programme, Centre for Geographic Medicine Research	Epidemiology and Demography, KEMRI - Wellcome Trust Research Programme, Centre for Geographic Medicine Research	Oketch,J.W., Kamau,E., Otieno,J.R., Mwema,A., Lewa,C., Agoti,C.N. and Nokes,J.D.
EPI_ISL_2572859, EPI_ISL_2572860, EPI_ISL_2572861, EPI_ISL_2572862	Virology Laboratory, Dr. Ricardo Gutierrez Children Hospital	Virology Laboratory, Dr. Ricardo Gutierrez Children Hospital	Rojo,G.L., Goya,S., Orellana,M., Sancilio,A., Rodriguez Perez,A., Montali,C., Garcia,C., Sosa,L., Musto,A., Hamilton,G., Alvarez,D., Castello,A. and Viegas,M.
EPI_ISL_2572869, EPI_ISL_2572870	Chinese Academy of Medical Sciences & Peking Union Medical College	Chinese Academy of Medical Sciences & Peking Union Medical College	Jia,B., Xiao,Y., Wang,Y., Chen,L., Zhang,J., Ren,L. and Wang,J.
EPI_ISL_2572871, EPI_ISL_2572872, EPI_ISL_2572873, EPI_ISL_2572874, EPI_ISL_2572875, EPI_ISL_2572876, EPI_ISL_2572877	WHO WPRO Measles Regional Reference Lab, Key Laboratory of Medical Virology Ministry of Health, National Institute for Viral Disease Control and Prevention, China Center for Disease Control and Prevention	WHO WPRO Measles Regional Reference Lab, Key Laboratory of Medical Virology Ministry of Health, National Institute for Viral Disease Control and Prevention, China Center for Disease Control and Prevention	Zhang,Y., Song,J. and Xu,W.
EPI_ISL_2572878	Institute of Virology and AIDS Research, First Hospital of Jilin University	Institute of Virology and AIDS Research, First Hospital of Jilin University	Zheng,Y., Liu,L., Wang,S., Li,Z., Hou,M., Li,J., Yu,X.-F., Zhang,W. and Hua,S.
EPI_ISL_2572879, EPI_ISL_2572880, EPI_ISL_2572881, EPI_ISL_2572882, EPI_ISL_2572883, EPI_ISL_2572884, EPI_ISL_2572885, EPI_ISL_2572886, EPI_ISL_2572887, EPI_ISL_2572888	WHO WPRO Measles Regional Reference Lab, Key Laboratory of Medical Virology Ministry of Health, National Institute for Viral Disease Control and Prevention, China Center for Disease Control and Prevention	WHO WPRO Measles Regional Reference Lab, Key Laboratory of Medical Virology Ministry of Health, National Institute for Viral Disease Control and Prevention, China Center for Disease Control and Prevention	Zhang,Y., Song,J. and Xu,W.
EPI_ISL_2572889, EPI_ISL_2572890	Institute of Virology and AIDS Research, First Hospital of Jilin University	Institute of Virology and AIDS Research, First Hospital of Jilin University	Zheng,Y., Liu,L., Wang,S., Li,Z., Hou,M., Li,J., Yu,X.-F., Zhang,W. and Hua,S.
EPI_ISL_2572891, EPI_ISL_2572892, EPI_ISL_2572893, EPI_ISL_2572894, EPI_ISL_2572895, EPI_ISL_2572896, EPI_ISL_2572897, EPI_ISL_2572898	WHO WPRO Measles Regional Reference Lab, Key Laboratory of Medical Virology Ministry of Health, National Institute for Viral Disease Control and Prevention, China Center for Disease Control and Prevention	WHO WPRO Measles Regional Reference Lab, Key Laboratory of Medical Virology Ministry of Health, National Institute for Viral Disease Control and Prevention, China Center for Disease Control and Prevention	Zhang,Y., Song,J. and Xu,W.
EPI_ISL_2572916, EPI_ISL_2572917, EPI_ISL_2572918, EPI_ISL_2572919, EPI_ISL_2572920, EPI_ISL_2572921, EPI_ISL_2572923, EPI_ISL_2572927, EPI_ISL_2572934, EPI_ISL_2572935, EPI_ISL_2572936, EPI_ISL_2572938, EPI_ISL_2572940, EPI_ISL_2572941, EPI_ISL_2572944, EPI_ISL_2572945, EPI_ISL_2572946, EPI_ISL_2572948, EPI_ISL_2572949, EPI_ISL_2572950, EPI_ISL_2572952, EPI_ISL_2572953, EPI_ISL_2572956, EPI_ISL_2572957, EPI_ISL_2572958, EPI_ISL_2572959			
see above	University of Wuerzburg, Institute of Virology and Immunobiology	University of Wuerzburg, Institute of Virology and Immunobiology	Prifert,C., Hofmann,D. and Weissbrich,B.
EPI_ISL_2572977, EPI_ISL_2572978, EPI_ISL_2572979, EPI_ISL_2572980, EPI_ISL_2572981	Pediatric Clinic 1, Department of Pathophysiology and Transplantation, University of Milan and Fondazione IRCCS Ca Granda	Pediatric Clinic 1, Department of Pathophysiology and Transplantation, University of Milan and Fondazione IRCCS Ca Granda	Esposito,S., Zampiero,A., Piralla,A. and Principi,N.
EPI_ISL_2572982, EPI_ISL_2572983	Virology, University of Ibadan, College of Medicine (UCH)	Virology, University of Ibadan, College of Medicine (UCH)	Ogunsemowo,O.S., Odaibo,G.N. and Olaleye,D.O.
EPI_ISL_2572985, EPI_ISL_2572986, EPI_ISL_2572987, EPI_ISL_2572988, EPI_ISL_2572989, EPI_ISL_2572990, EPI_ISL_2572991, EPI_ISL_2572992, EPI_ISL_2572993, EPI_ISL_2572994, EPI_ISL_2572995, EPI_ISL_2572996			
see above	Virology, Tohoku University Graduate School of Medicine	Virology, Tohoku University Graduate School of Medicine	Malasao,R., Okamoto,M., Chaimongkol,N., Imamura,T., Tohma,K., Dapat,I., Dapat,C., Suzuki,A., Saito,M., Saito,M., Tamaki,R., Segubre-Mercado,E., Igoy,M.A.U., Lupisan,S., Olveda,R. and Oshitani,H.
EPI_ISL_2572998	Department of Infectious Diseases, National Health Institute Doutor Ricardo Jorge	Department of Infectious Diseases, National Health Institute Doutor Ricardo Jorge	Saez-Lopez,E., Cristovao,P., Costa,I., Pechirra,P., Conde,P. and Guiomar,R.
EPI_ISL_2573015, EPI_ISL_2573016	Microbiology, Hospital Universitari Vall d'Hebron	Microbiology, Hospital Universitari Vall d'Hebron	Gimferrer,L., Martin,Md.C., Pumarola,T. and Anton,A.
EPI_ISL_2573049	Epidemiology and Immunogenetic of Viral Infections LR14SP02, University Hospital Sahloul	Epidemiology and Immunogenetic of Viral Infections LR14SP02, University Hospital Sahloul	Ataoui,I., Jerbi,A., BenHamida-Rebai,M., Ben Hadj Fredj,M., Fodha,I., Bennour,H., Hammouda,H., Khlifa,M., Brini,I., Boussoffara,R., Boussetta,K., Abroug,S. and Trabelsi,A.
EPI_ISL_2573057, EPI_ISL_2573058	Virology Laboratory, Dr. Ricardo Gutierrez Children Hospital	Virology Laboratory, Dr. Ricardo Gutierrez Children Hospital	Rojo,G.L., Goya,S., Orellana,M., Sancilio,A., Rodriguez Perez,A., Montali,C., Garcia,C., Sosa,L., Musto,A., Hamilton,G., Alvarez,D., Castello,A. and Viegas,M.
EPI_ISL_2573059	WHO WPRO Measles Regional Reference Lab, Key Laboratory of Medical Virology Ministry of Health, National Institute for Viral Disease Control and Prevention, China Center for Disease Control and Prevention	WHO WPRO Measles Regional Reference Lab, Key Laboratory of Medical Virology Ministry of Health, National Institute for Viral Disease Control and Prevention, China Center for Disease Control and Prevention	Zhang,Y., Song,J. and Xu,W.
EPI_ISL_2573060	University of Wuerzburg, Institute of Virology and Immunobiology	University of Wuerzburg, Institute of Virology and Immunobiology	Prifert,C., Hofmann,D. and Weissbrich,B.
EPI_ISL_2573064	Influenza and Other Respiratory Viruses Unit, National Center for Microbiology, Instituto de Salud Carlos III	Influenza and Other Respiratory Viruses Unit, National Center for Microbiology, Instituto de Salud Carlos III	Calderon,A., Pozo,F., Calvo,C., Garcia-Garcia,M., Gonzalez,M., Molinero,M. and Casas,I.
EPI_ISL_2573069	Laboratory of molecular virology, Research Institute of Influenza, Ministry of Healthcare of the Russian Federation	Laboratory of molecular virology, Research Institute of Influenza, Ministry of Healthcare of the Russian Federation	Komissarova,K.S., Krivitskaya,V.Z., Sverlova,M.V., Petrova,E.R., Fadeev,A.V. and Komissarov,A.B.
EPI_ISL_2573070	Department of Pediatrics, Center of Excellence in Clinical Virology, Faculty of Medicine, Chulalongkorn University	Department of Pediatrics, Center of Excellence in Clinical Virology, Faculty of Medicine, Chulalongkorn University	Thongpan,I., Mauleekoonphairoj,J., Vichiwattana,P., Korkong,S., Vongpunsawad,S. and Poovorawan,Y.
EPI_ISL_2573071	Akinobu Hibino Niigata University, International Health, Public Health	Akinobu Hibino Niigata University, International Health, Public Health	Hibino,A., Saito,R., Shoubugawa,Y. and Saito,T.
EPI_ISL_2573072, EPI_ISL_2573073, EPI_ISL_2573074, EPI_ISL_2573075	Akinobu Hibino Niigata University, International Health, Public Health	Akinobu Hibino Niigata University, International Health, Public Health	Hibino,A., Saito,R., Shoubugawa,Y. and Sano,Y.
EPI_ISL_2573076, EPI_ISL_2573077, EPI_ISL_2573078, EPI_ISL_2573079, EPI_ISL_2573080	Akinobu Hibino Niigata University, International Health, Public Health	Akinobu Hibino Niigata University, International Health, Public Health	Hibino,A., Saito,R., Shoubugawa,Y., Makiya,T. and Takefuta,K.

EPI_ISL_2573081	Akinobu Hibino Niigata University, International Health, Public Health	Akinobu Hibino Niigata University, International Health, Public Health	Hibino,A., Saito,R., Shoubugawa,Y., Azhar,S. and Rohaizat,H.
EPI_ISL_2573082, EPI_ISL_2573083, EPI_ISL_2573084, EPI_ISL_2573085	Akinobu Hibino Niigata University, International Health, Public Health	Akinobu Hibino Niigata University, International Health, Public Health	Hibino,A., Saito,R., Shoubugawa,Y., Htay,H.T., Khin,Y.O., Yi,Y.M. and Yadanar,K.
EPI_ISL_2573086	Wint Wint Phyu Niigat University, International Health, Public Health	Wint Wint Phyu Niigat University, International Health, Public Health	Phyu,W.W. and Saito,R.
EPI_ISL_2573104	Medicine, American University of Beirut	Medicine, American University of Beirut	Abou-El-Hassan,H. and Zaraket,H.
EPI_ISL_2573107	Microbiology, Sanjay Gandhi Post Graduate Institute of Medical Science	Microbiology, Sanjay Gandhi Post Graduate Institute of Medical Science	Saxena,S., Singh,D., Tripathi,R., Dhole,T.N. and Kushwaha,R.
EPI_ISL_2573112	Microbiology Department, Virology Division, College of Medicine, Taif University	Microbiology Department, Virology Division, College of Medicine, Taif University	Al Aboud,D.M., Al Aboud,N.M., Al-Malky,M.I.R. and Abdel-Moneim,A.S.
EPI_ISL_2573122, EPI_ISL_2573124, EPI_ISL_2573125	Epidemiology and Demography, KEMRI - Wellcome Trust Research Programme, Centre for Geographic Medicine Research	Epidemiology and Demography, KEMRI - Wellcome Trust Research Programme, Centre for Geographic Medicine Research	Oketch,J.W., Kamau,E., Otieno,J.R., Mwema,A., Lewa,C., Agoti,C.N. and Nokes,J.D.
EPI_ISL_2573126	Virology, Evandro Chagas Institute	Virology, Evandro Chagas Institute	Santos,V.M., Ferreira,J.A., Lima,J.F., Barbagelata,L.S., Souza,E.M.A., Goncalves,M.S., SoNorth America / USA,E.C. Jr., Costa,I.B., Santos,M.C., SoNorth America / USA,R.C.M. and Mello,W.A.
EPI_ISL_2573128	Department of Medical Research, Ministry of Health and Sports, No. 5, Ziwaka Road, Dagon Township	Department of Medical Research, Ministry of Health and Sports, No. 5, Ziwaka Road, Dagon Township	Latt,A.Z., Aye,K.T. and Thwe,H.H.
EPI_ISL_2573130	Pediatrics, Show-Chwan Memorial Hospital	Pediatrics, Show-Chwan Memorial Hospital	Lee,C.-Y.
EPI_ISL_2573133	Department of Pediatrics, Center of Excellence in Clinical Virology, Faculty of Medicine, Chulalongkorn University	Department of Pediatrics, Center of Excellence in Clinical Virology, Faculty of Medicine, Chulalongkorn University	Thongpan,I., Mauleekoonphairoj,J., Vichiwattana,P., Korkong,S., Vongpunsawad,S. and Poovorawan,Y.
EPI_ISL_2573138, EPI_ISL_2573139	Virology, University of Ibadan, College of Medicine (UCH)	Virology, University of Ibadan, College of Medicine (UCH)	Ogunsemowo,O.S., Odaibo,G.N. and Olaleye,D.O.
EPI_ISL_2573144, EPI_ISL_2573145	Chinese Academy of Medical Sciences & Peking Union Medical College	Chinese Academy of Medical Sciences & Peking Union Medical College	Jia,B., Xiao,Y., Wang,Y., Chen,L., Zhang,J., Ren,L. and Wang,J.
EPI_ISL_2573146, EPI_ISL_2573147	Institute of Virology and AIDS Research, First Hospital of Jilin University	Institute of Virology and AIDS Research, First Hospital of Jilin University	Zheng,Y., Liu,L., Wang,S., Li,Z., Hou,M., Li,J., Yu,X.-F., Zhang,W. and Hua,S.
EPI_ISL_2573148, EPI_ISL_2573149, EPI_ISL_2573150, EPI_ISL_2573151, EPI_ISL_2573152, EPI_ISL_2573153	WHO WPRO Measles Regional Reference Lab, Key Laboratory of Medical Virology Ministry of Health, National Institute for Viral Disease Control and Prevention, China Center for Disease Control and Prevention	WHO WPRO Measles Regional Reference Lab, Key Laboratory of Medical Virology Ministry of Health, National Institute for Viral Disease Control and Prevention, China Center for Disease Control and Prevention	Zhang,Y., Song,J. and Xu,W.
EPI_ISL_2573154, EPI_ISL_2573155	Institute of Virology and AIDS Research, First Hospital of Jilin University	Institute of Virology and AIDS Research, First Hospital of Jilin University	Zheng,Y., Liu,L., Wang,S., Li,Z., Hou,M., Li,J., Yu,X.-F., Zhang,W. and Hua,S.
EPI_ISL_2573156, EPI_ISL_2573157	WHO WPRO Measles Regional Reference Lab, Key Laboratory of Medical Virology Ministry of Health, National Institute for Viral Disease Control and Prevention, China Center for Disease Control and Prevention	WHO WPRO Measles Regional Reference Lab, Key Laboratory of Medical Virology Ministry of Health, National Institute for Viral Disease Control and Prevention, China Center for Disease Control and Prevention	Zhang,Y., Song,J. and Xu,W.
EPI_ISL_2573158, EPI_ISL_2573159, EPI_ISL_2573163, EPI_ISL_2573167, EPI_ISL_2573168	University of Wuerzburg, Institute of Virology and Immunobiology	University of Wuerzburg, Institute of Virology and Immunobiology	Prifert,C., Hofmann,D. and Weissbrich,B.
EPI_ISL_2573169, EPI_ISL_2573170	Virology, Noguchi Memorial Institute for Medical Research	Virology, Noguchi Memorial Institute for Medical Research	Obodai,E., Odoom,J.K., Adiku,T., Goka,B., Biere,B., Wolff,T., Schweiger,B. and Reiche,J.
EPI_ISL_2573172	Department of Virology, School of Public Health, Tehran University of Medical Sciences	Department of Virology, School of Public Health, Tehran University of Medical Sciences	Salimi,V., Samieipoor,Y., Ghavami,N. and Mokhtariazad,T.
EPI_ISL_2573173	Virology, University of Ibadan, College of Medicine (UCH)	Virology, University of Ibadan, College of Medicine (UCH)	Ogunsemowo,O.S., Odaibo,G.N. and Olaleye,D.O.
EPI_ISL_2573174	Virology, Tohoku University Graduate School of Medicine	Virology, Tohoku University Graduate School of Medicine	Malasao,R., Okamoto,M., Chaimongkol,N., Imamura,T., Tohma,K., Dapat,I., Dapat,C., Suzuki,A., Saito,M., Saito,M., Tamaki,R., Segubre-Mercado,E., Igoy,M.A.U., Lupisan,S., Olveda,R. and Oshitani,H.
EPI_ISL_2573184, EPI_ISL_2573185, EPI_ISL_2573186, EPI_ISL_2573187, EPI_ISL_2573188, EPI_ISL_2573194	Microbiology, Hospital Universitari Vall d'Hebron	Microbiology, Hospital Universitari Vall d'Hebron	Gimferrer,L., Martin,Md.C., Pumarola,T. and Anton,A.
EPI_ISL_2573201, EPI_ISL_2573202, EPI_ISL_2573203	Akinobu Hibino Niigata University, International Health, Public Health	Akinobu Hibino Niigata University, International Health, Public Health	Hibino,A., Saito,R., Shoubugawa,Y. and Sano,Y.
EPI_ISL_2573213	Virology, University of Ibadan, College of Medicine (UCH)	Virology, University of Ibadan, College of Medicine (UCH)	Ogunsemowo,O.O., Odaibo,G.N. and Olaleye,D.O.
EPI_ISL_2573215	WHO WPRO Measles Regional Reference Lab, Key Laboratory of Medical Virology Ministry of Health, National Institute for Viral Disease Control and Prevention, China Center for Disease Control and Prevention	WHO WPRO Measles Regional Reference Lab, Key Laboratory of Medical Virology Ministry of Health, National Institute for Viral Disease Control and Prevention, China Center for Disease Control and Prevention	Zhang,Y., Song,J. and Xu,W.
EPI_ISL_2573217	University of Wuerzburg, Institute of Virology and Immunobiology	University of Wuerzburg, Institute of Virology and Immunobiology	Prifert,C., Hofmann,D. and Weissbrich,B.
EPI_ISL_2573224	Virology, Noguchi Memorial Institute for Medical Research	Virology, Noguchi Memorial Institute for Medical Research	Obodai,E., Odoom,J.K., Adiku,T., Goka,B., Biere,B., Wolff,T., Schweiger,B. and Reiche,J.
EPI_ISL_2573225, EPI_ISL_2573226, EPI_ISL_2573227	Akinobu Hibino Niigata University, International Health, Public Health	Akinobu Hibino Niigata University, International Health, Public Health	Hibino,A., Saito,R., Shoubugawa,Y. and Sano,Y.
EPI_ISL_2573228	Akinobu Hibino Niigata University, International Health, Public Health	Akinobu Hibino Niigata University, International Health, Public Health	Hibino,A., Saito,R., Shoubugawa,Y., Ghassan,D. and Hassan,Z.
EPI_ISL_2573229, EPI_ISL_2573230, EPI_ISL_2573231, EPI_ISL_2573232, EPI_ISL_2573233	Akinobu Hibino Niigata University, International Health, Public Health	Akinobu Hibino Niigata University, International Health, Public Health	Hibino,A., Saito,R., Shoubugawa,Y., Htay,H.T., Khin,Y.O., Yi,Y.M. and Yadanar,K.
EPI_ISL_2573235, EPI_ISL_2573236	Medicine, American University of Beirut	Medicine, American University of Beirut	Abou-El-Hassan,H. and Zaraket,H.
EPI_ISL_2573238	Virology, Noguchi Memorial Institute for Medical Research	Virology, Noguchi Memorial Institute for Medical Research	Obodai,E., Odoom,J.K., Adiku,T., Goka,B., Biere,B., Wolff,T., Schweiger,B. and Reiche,J.
EPI_ISL_2573240	Epidemiology and Immunogenetic of Viral Infections LR14SP02, University Hospital Sahloul	Epidemiology and Immunogenetic of Viral Infections LR14SP02, University Hospital Sahloul	Ataoui,I., Jerbi,A., BenHamida-Rebai,M., Ben Hadj Fredj,M., Fodha,I., Bennour,H., Hammouda,H., Khlifa,M., Brini,I., Boussoffara,R., Boussetta,K., Abroug,S. and Trabelsi,A.
EPI_ISL_2573243, EPI_ISL_2573244, EPI_ISL_2573245, EPI_ISL_2573246, EPI_ISL_2573248, EPI_ISL_2573249	Department of Clinical Laboratory, Fujian Provincial Hospital	Department of Clinical Laboratory, Fujian Provincial Hospital	Su,Y., Wu,Y., Tian,R. and Liang,G.
EPI_ISL_2573250	Akinobu Hibino Niigata University, International Health, Public Health	Akinobu Hibino Niigata University, International Health, Public Health	Hibino,A., Saito,R., Shoubugawa,Y. and Sano,Y.
EPI_ISL_2573251	Akinobu Hibino Niigata University, International Health, Public Health	Akinobu Hibino Niigata University, International Health, Public Health	Hibino,A., Saito,R., Shoubugawa,Y., Htay,H.T., Khin,Y.O., Yi,Y.M. and Yadanar,K.
EPI_ISL_2573257	Chinese Academy of Medical Sciences & Peking Union Medical College	Chinese Academy of Medical Sciences & Peking Union Medical College	Jia,B., Xiao,Y., Wang,Y., Chen,L., Zhang,J., Ren,L. and Wang,J.
EPI_ISL_2573258	Microbiology, Hospital Universitari Vall d'Hebron	Microbiology, Hospital Universitari Vall d'Hebron	Gimferrer,L., Martin,Md.C., Pumarola,T. and Anton,A.
EPI_ISL_2573260, EPI_ISL_2573261, EPI_ISL_2573267	Virology, Evandro Chagas Institute	Virology, Evandro Chagas Institute	Santos,V.M., Ferreira,J.A., Lima,J.F., Barbagelata,L.S., Souza,E.M.A., Goncalves,M.S., SoNorth America / USA,E.C. Jr., Costa,I.B., Santos,M.C., SoNorth America / USA,R.C.M. and Mello,W.A.
EPI_ISL_2573270	Akinobu Hibino Niigata University, International Health, Public Health	Akinobu Hibino Niigata University, International Health, Public Health	Hibino,A., Saito,R., Shoubugawa,Y., Ghassan,D. and Hassan,Z.
EPI_ISL_2573271	Medicine, American University of Beirut	Medicine, American University of Beirut	Abou-El-Hassan,H. and Zaraket,H.
EPI_ISL_2573273	Akinobu Hibino Niigata University, International Health, Public Health	Akinobu Hibino Niigata University, International Health, Public Health	Hibino,A., Saito,R., Shoubugawa,Y., Ghassan,D. and Hassan,Z.
EPI_ISL_2573274	Medicine, American University of Beirut	Medicine, American University of Beirut	Abou-El-Hassan,H. and Zaraket,H.
EPI_ISL_2573276	Akinobu Hibino Niigata University, International Health, Public Health	Akinobu Hibino Niigata University, International Health, Public Health	Hibino,A., Saito,R., Shoubugawa,Y., Ghassan,D. and Hassan,Z.
EPI_ISL_2573277	Medicine, American University of Beirut	Medicine, American University of Beirut	Abou-El-Hassan,H. and Zaraket,H.
EPI_ISL_2573278, EPI_ISL_2573279, EPI_ISL_2573280, EPI_ISL_2573281, EPI_ISL_2573282, EPI_ISL_2573283, EPI_ISL_2573284	Virology Laboratory, Dr. Ricardo Gutierrez Children Hospital	Virology Laboratory, Dr. Ricardo Gutierrez Children Hospital	Rojo,G.L., Goya,S., Orellana,M., Sancilio,A., Rodriguez Perez,A., Montali,C., Garcia,C., Sosa,L., Musto,A., Hamilton,G., Alvarez,D., Castello,A. and Viegas,M.
EPI_ISL_2573360, EPI_ISL_2573361, EPI_ISL_2573362, EPI_ISL_2573363, EPI_ISL_2573364, EPI_ISL_2573369, EPI_ISL_2573370, EPI_ISL_2573371, EPI_ISL_2573372, EPI_ISL_2573373, EPI_ISL_2573374, EPI_ISL_2573375, EPI_ISL_2573376, EPI_ISL_2573377, EPI_ISL_2573379, EPI_ISL_2573380, EPI_ISL_2573381, EPI_ISL_2573382, EPI_ISL_2573383, EPI_ISL_2573384			
see above	Chinese Academy of Medical Sciences & Peking Union Medical College	Chinese Academy of Medical Sciences & Peking Union Medical College	Jia,B., Xiao,Y., Wang,Y., Chen,L., Zhang,J., Ren,L. and Wang,J.
EPI_ISL_2573390, EPI_ISL_2573391, EPI_ISL_2573392, EPI_ISL_2573393, EPI_ISL_2573394, EPI_ISL_2573395, EPI_ISL_2573396, EPI_ISL_2573397	WHO WPRO Measles Regional Reference Lab, Key Laboratory of Medical Virology Ministry of Health, National Institute for Viral Disease Control and Prevention, China Center for Disease Control and Prevention	WHO WPRO Measles Regional Reference Lab, Key Laboratory of Medical Virology Ministry of Health, National Institute for Viral Disease Control and Prevention, China Center for Disease Control and Prevention	Zhang,Y., Song,J. and Xu,W.
EPI_ISL_2573399, EPI_ISL_2573400, EPI_ISL_2573401, EPI_ISL_2573402, EPI_ISL_2573403, EPI_ISL_2573404, EPI_ISL_2573405, EPI_ISL_2573406, EPI_ISL_2573407, EPI_ISL_2573408, EPI_ISL_2573409, EPI_ISL_2573410, EPI_ISL_2573411, EPI_ISL_2573412, EPI_ISL_2573413, EPI_ISL_2573414, EPI_ISL_2573415, EPI_ISL_2573416, EPI_ISL_2573417			
see above	Laboratory of Virology, Capital Institute of Pediatrics	Laboratory of Virology, Capital Institute of Pediatrics	Cui,G., Zhu,R., Qian,Y., Deng,J., Zhao,L., Sun,Y. and Wang,F.
EPI_ISL_2573418, EPI_ISL_2573419, EPI_ISL_2573420, EPI_ISL_2573421, EPI_ISL_2573423, EPI_ISL_2573428	Institute of Virology and AIDS Research, First Hospital of Jilin University	Institute of Virology and AIDS Research, First Hospital of Jilin University	Zheng,Y., Liu,L., Wang,S., Li,Z., Hou,M., Li,J., Yu,X.-F., Zhang,W. and Hua,S.
EPI_ISL_2573429, EPI_ISL_2573430	WHO WPRO Measles Regional Reference Lab, Key Laboratory of Medical Virology Ministry of Health, National Institute for Viral Disease Control and Prevention, China Center for Disease Control and Prevention	WHO WPRO Measles Regional Reference Lab, Key Laboratory of Medical Virology Ministry of Health, National Institute for Viral Disease Control and Prevention, China Center for Disease Control and Prevention	Zhang,Y., Song,J. and Xu,W.
EPI_ISL_2573431	WHO WPRO Measles Regional Reference Lab, Key Laboratory of Medical Virology Ministry of Health, National Institute for Viral Disease Control and Prevention, China Center for Disease Control and Prevention	WHO WPRO Measles Regional Reference Lab, Key Laboratory of Medical Virology Ministry of Health, National Institute for Viral Disease Control and Prevention, China Center for Disease Control and Prevention	Song,J., Zhang,Y. and Xu,W.
EPI_ISL_2573432, EPI_ISL_2573433, EPI_ISL_2573434, EPI_ISL_2573435	WHO WPRO Measles Regional Reference Lab, Key Laboratory of Medical Virology Ministry of Health, National Institute for Viral Disease Control and	WHO WPRO Measles Regional Reference Lab, Key Laboratory of Medical Virology Ministry of Health, National Institute for Viral Disease Control and	Zhang,Y., Song,J. and Xu,W.

	Prevention, China Center for Disease Control and Prevention	Prevention, China Center for Disease Control and Prevention	
EPI_ISL_2573436	WHO WPRO Measles Regional Reference Lab, Key Laboratory of Medical Virology Ministry of Health, National Institute for Viral Disease Control and Prevention, China Center for Disease Control and Prevention	WHO WPRO Measles Regional Reference Lab, Key Laboratory of Medical Virology Ministry of Health, National Institute for Viral Disease Control and Prevention, China Center for Disease Control and Prevention	Song,J., Zhang,Y. and Xu,W.
EPI_ISL_2573437	WHO WPRO Measles Regional Reference Lab, Key Laboratory of Medical Virology Ministry of Health, National Institute for Viral Disease Control and Prevention, China Center for Disease Control and Prevention	WHO WPRO Measles Regional Reference Lab, Key Laboratory of Medical Virology Ministry of Health, National Institute for Viral Disease Control and Prevention, China Center for Disease Control and Prevention	Zhang,Y., Song,J. and Xu,W.
EPI_ISL_2573438	WHO WPRO Measles Regional Reference Lab, Key Laboratory of Medical Virology Ministry of Health, National Institute for Viral Disease Control and Prevention, China Center for Disease Control and Prevention	WHO WPRO Measles Regional Reference Lab, Key Laboratory of Medical Virology Ministry of Health, National Institute for Viral Disease Control and Prevention, China Center for Disease Control and Prevention	Song,J., Zhang,Y. and Xu,W.
EPI_ISL_2573439, EPI_ISL_2573440, EPI_ISL_2573441	WHO WPRO Measles Regional Reference Lab, Key Laboratory of Medical Virology Ministry of Health, National Institute for Viral Disease Control and Prevention, China Center for Disease Control and Prevention	WHO WPRO Measles Regional Reference Lab, Key Laboratory of Medical Virology Ministry of Health, National Institute for Viral Disease Control and Prevention, China Center for Disease Control and Prevention	Zhang,Y., Song,J. and Xu,W.
EPI_ISL_2573442	Institute of Virology and AIDS Research, First Hospital of Jilin University	Institute of Virology and AIDS Research, First Hospital of Jilin University	Zheng,Y., Liu,L., Wang,S., Li,Z., Hou,M., Li,J., Yu,X.-F., Zhang,W. and Hua,S.
EPI_ISL_2573443, EPI_ISL_2573444, EPI_ISL_2573445, EPI_ISL_2573446, EPI_ISL_2573447, EPI_ISL_2573448, EPI_ISL_2573449, EPI_ISL_2573450, EPI_ISL_2573451, EPI_ISL_2573452, EPI_ISL_2573453, EPI_ISL_2573454, EPI_ISL_2573455, EPI_ISL_2573456, EPI_ISL_2573457			
see above	WHO WPRO Measles Regional Reference Lab, Key Laboratory of Medical Virology Ministry of Health, National Institute for Viral Disease Control and Prevention, China Center for Disease Control and Prevention	WHO WPRO Measles Regional Reference Lab, Key Laboratory of Medical Virology Ministry of Health, National Institute for Viral Disease Control and Prevention, China Center for Disease Control and Prevention	Zhang,Y., Song,J. and Xu,W.
EPI_ISL_2573458	WHO WPRO Measles Regional Reference Lab, Key Laboratory of Medical Virology Ministry of Health, National Institute for Viral Disease Control and Prevention, China Center for Disease Control and Prevention	WHO WPRO Measles Regional Reference Lab, Key Laboratory of Medical Virology Ministry of Health, National Institute for Viral Disease Control and Prevention, China Center for Disease Control and Prevention	Song,J., Zhang,Y. and Xu,W.
EPI_ISL_2573459, EPI_ISL_2573460, EPI_ISL_2573461, EPI_ISL_2573462, EPI_ISL_2573463, EPI_ISL_2573464, EPI_ISL_2573465, EPI_ISL_2573466, EPI_ISL_2573467, EPI_ISL_2573468, EPI_ISL_2573469, EPI_ISL_2573470, EPI_ISL_2573471, EPI_ISL_2573472, EPI_ISL_2573473			
see above	WHO WPRO Measles Regional Reference Lab, Key Laboratory of Medical Virology Ministry of Health, National Institute for Viral Disease Control and Prevention, China Center for Disease Control and Prevention	WHO WPRO Measles Regional Reference Lab, Key Laboratory of Medical Virology Ministry of Health, National Institute for Viral Disease Control and Prevention, China Center for Disease Control and Prevention	Zhang,Y., Song,J. and Xu,W.
EPI_ISL_2573474	WHO WPRO Measles Regional Reference Lab, Key Laboratory of Medical Virology Ministry of Health, National Institute for Viral Disease Control and Prevention, China Center for Disease Control and Prevention	WHO WPRO Measles Regional Reference Lab, Key Laboratory of Medical Virology Ministry of Health, National Institute for Viral Disease Control and Prevention, China Center for Disease Control and Prevention	Song,J., Zhang,Y. and Xu,W.
EPI_ISL_2573475, EPI_ISL_2573476, EPI_ISL_2573477, EPI_ISL_2573478, EPI_ISL_2573479, EPI_ISL_2573480, EPI_ISL_2573481, EPI_ISL_2573482, EPI_ISL_2573483, EPI_ISL_2573484, EPI_ISL_2573485, EPI_ISL_2573486, EPI_ISL_2573487, EPI_ISL_2573488, EPI_ISL_2573489, EPI_ISL_2573490, EPI_ISL_2573491, EPI_ISL_2573492, EPI_ISL_2573493, EPI_ISL_2573494			
see above	WHO WPRO Measles Regional Reference Lab, Key Laboratory of Medical Virology Ministry of Health, National Institute for Viral Disease Control and Prevention, China Center for Disease Control and Prevention	WHO WPRO Measles Regional Reference Lab, Key Laboratory of Medical Virology Ministry of Health, National Institute for Viral Disease Control and Prevention, China Center for Disease Control and Prevention	Zhang,Y., Song,J. and Xu,W.
EPI_ISL_2573495, EPI_ISL_2573496	Institute of Virology and AIDS Research, First Hospital of Jilin University	Institute of Virology and AIDS Research, First Hospital of Jilin University	Zheng,Y., Liu,L., Wang,S., Li,Z., Hou,M., Li,J., Yu,X.-F., Zhang,W. and Hua,S.
EPI_ISL_2573499, EPI_ISL_2573500, EPI_ISL_2573501	Department of Respiratory Medicine, Children's Hospital of Chongqing Medical University	Department of Respiratory Medicine, Children's Hospital of Chongqing Medical University	Ren,L. and Liu,E.
EPI_ISL_2573502, EPI_ISL_2573503, EPI_ISL_2573504	Department of Clinical Laboratory, Fujian Provincial Hospital	Department of Clinical Laboratory, Fujian Provincial Hospital	Su,Y., Wu,Y., Tian,R. and Liang,G.
EPI_ISL_2573526	WHO WPRO Measles Regional Reference Lab, Key Laboratory of Medical Virology Ministry of Health, National Institute for Viral Disease Control and Prevention, China Center for Disease Control and Prevention	WHO WPRO Measles Regional Reference Lab, Key Laboratory of Medical Virology Ministry of Health, National Institute for Viral Disease Control and Prevention, China Center for Disease Control and Prevention	Zhang,Y., Song,J. and Xu,W.
EPI_ISL_2573527, EPI_ISL_2573528	WHO WPRO Measles Regional Reference Lab, Key Laboratory of Medical Virology Ministry of Health, National Institute for Viral Disease Control and Prevention, China Center for Disease Control and Prevention	WHO WPRO Measles Regional Reference Lab, Key Laboratory of Medical Virology Ministry of Health, National Institute for Viral Disease Control and Prevention, China Center for Disease Control and Prevention	Song,J., Zhang,Y. and Xu,W.
EPI_ISL_2573535, EPI_ISL_2573536, EPI_ISL_2573537, EPI_ISL_2573538, EPI_ISL_2573539, EPI_ISL_2573540	WHO WPRO Measles Regional Reference Lab, Key Laboratory of Medical Virology Ministry of Health, National Institute for Viral Disease Control and Prevention, China Center for Disease Control and Prevention	WHO WPRO Measles Regional Reference Lab, Key Laboratory of Medical Virology Ministry of Health, National Institute for Viral Disease Control and Prevention, China Center for Disease Control and Prevention	Zhang,Y., Song,J. and Xu,W.
EPI_ISL_2573546	Centre for Research And Knowledge Transfer in Biotechnology, University of Zagreb	Centre for Research And Knowledge Transfer in Biotechnology, University of Zagreb	Ivancic-Jelecki,J., Slovic,A., Ljubin-Sternak,S., Mlinaric-Galinovic,G. and Forcic,D.
EPI_ISL_2573549	Centre for Research And Knowledge Transfer in Biotechnology, University of Zagreb	Centre for Research And Knowledge Transfer in Biotechnology, University of Zagreb	Jagusic,M., Slovic,A., Santak,M., Kosutic-Gulija,T., Ivancic-Jelecki,J. and Forcic,D.
EPI_ISL_2573553	Centre for Research And Knowledge Transfer in Biotechnology, University of Zagreb	Centre for Research And Knowledge Transfer in Biotechnology, University of Zagreb	Ivancic-Jelecki,J., Slovic,A., Ljubin-Sternak,S., Mlinaric-Galinovic,G. and Forcic,D.
EPI_ISL_2573554	Centre for Research And Knowledge Transfer in Biotechnology, University of Zagreb	Centre for Research And Knowledge Transfer in Biotechnology, University of Zagreb	Jagusic,M., Slovic,A., Santak,M., Kosutic-Gulija,T., Ivancic-Jelecki,J. and Forcic,D.
EPI_ISL_2573557	Centre for Research And Knowledge Transfer in Biotechnology, University of Zagreb	Centre for Research And Knowledge Transfer in Biotechnology, University of Zagreb	Ivancic-Jelecki,J., Slovic,A., Ljubin-Sternak,S., Mlinaric-Galinovic,G. and Forcic,D.
EPI_ISL_2573559	Centre for Research And Knowledge Transfer in Biotechnology, University of Zagreb	Centre for Research And Knowledge Transfer in Biotechnology, University of Zagreb	Jagusic,M., Slovic,A., Santak,M., Kosutic-Gulija,T., Ivancic-Jelecki,J. and Forcic,D.
EPI_ISL_2573560	Centre for Research And Knowledge Transfer in Biotechnology, University of Zagreb	Centre for Research And Knowledge Transfer in Biotechnology, University of Zagreb	Ivancic-Jelecki,J., Slovic,A., Ljubin-Sternak,S., Mlinaric-Galinovic,G. and Forcic,D.
EPI_ISL_2573561, EPI_ISL_2573562	Centre for Research And Knowledge Transfer in Biotechnology, University of Zagreb	Centre for Research And Knowledge Transfer in Biotechnology, University of Zagreb	Jagusic,M., Slovic,A., Santak,M., Kosutic-Gulija,T., Ivancic-Jelecki,J. and Forcic,D.
EPI_ISL_2573568, EPI_ISL_2573569, EPI_ISL_2573571, EPI_ISL_2573572, EPI_ISL_2573573, EPI_ISL_2573574, EPI_ISL_2573575, EPI_ISL_2573576	Centre for Research And Knowledge Transfer in Biotechnology, University of Zagreb	Centre for Research And Knowledge Transfer in Biotechnology, University of Zagreb	Ivancic-Jelecki,J., Slovic,A., Ljubin-Sternak,S., Mlinaric-Galinovic,G. and Forcic,D.
EPI_ISL_2573577	Centre for Research And Knowledge Transfer in Biotechnology, University of Zagreb	Centre for Research And Knowledge Transfer in Biotechnology, University of Zagreb	Jagusic,M., Slovic,A., Santak,M., Kosutic-Gulija,T., Ivancic-Jelecki,J. and Forcic,D.
EPI_ISL_2573578, EPI_ISL_2573579	Centre for Research And Knowledge Transfer in Biotechnology, University of Zagreb	Centre for Research And Knowledge Transfer in Biotechnology, University of Zagreb	Ivancic-Jelecki,J., Slovic,A., Ljubin-Sternak,S., Mlinaric-Galinovic,G. and Forcic,D.
EPI_ISL_2573580, EPI_ISL_2573581	Centre for Research And Knowledge Transfer in Biotechnology, University of Zagreb	Centre for Research And Knowledge Transfer in Biotechnology, University of Zagreb	Jagusic,M., Slovic,A., Santak,M., Kosutic-Gulija,T., Ivancic-Jelecki,J. and Forcic,D.
EPI_ISL_2573582, EPI_ISL_2573587, EPI_ISL_2573589, EPI_ISL_2573590, EPI_ISL_2573591, EPI_ISL_2573592, EPI_ISL_2573593, EPI_ISL_2573594, EPI_ISL_2573595, EPI_ISL_2573596	Centre for Research And Knowledge Transfer in Biotechnology, University of Zagreb	Centre for Research And Knowledge Transfer in Biotechnology, University of Zagreb	Ivancic-Jelecki,J., Slovic,A., Ljubin-Sternak,S., Mlinaric-Galinovic,G. and Forcic,D.
EPI_ISL_2573598, EPI_ISL_2573599	Centre for Research And Knowledge Transfer in Biotechnology, University of Zagreb	Centre for Research And Knowledge Transfer in Biotechnology, University of Zagreb	Jagusic,M., Slovic,A., Santak,M., Kosutic-Gulija,T., Ivancic-Jelecki,J. and Forcic,D.
EPI_ISL_2573601, EPI_ISL_2573603, EPI_ISL_2573604, EPI_ISL_2573609	Centre for Research And Knowledge Transfer in Biotechnology, University of Zagreb	Centre for Research And Knowledge Transfer in Biotechnology, University of Zagreb	Ivancic-Jelecki,J., Slovic,A., Ljubin-Sternak,S., Mlinaric-Galinovic,G. and Forcic,D.
EPI_ISL_2573610, EPI_ISL_2573611	Centre for Research And Knowledge Transfer in Biotechnology, University of Zagreb	Centre for Research And Knowledge Transfer in Biotechnology, University of Zagreb	Jagusic,M., Slovic,A., Santak,M., Kosutic-Gulija,T., Ivancic-Jelecki,J. and Forcic,D.
EPI_ISL_2573612	Centre for Research And Knowledge Transfer in Biotechnology, University of Zagreb	Centre for Research And Knowledge Transfer in Biotechnology, University of Zagreb	Ivancic-Jelecki,J., Slovic,A., Ljubin-Sternak,S., Mlinaric-Galinovic,G. and Forcic,D.
EPI_ISL_2573618	Centre for Research And Knowledge Transfer in Biotechnology, University of Zagreb	Centre for Research And Knowledge Transfer in Biotechnology, University of Zagreb	Jagusic,M., Slovic,A., Santak,M., Kosutic-Gulija,T., Ivancic-Jelecki,J. and Forcic,D.
EPI_ISL_2573630, EPI_ISL_2573631, EPI_ISL_2573632, EPI_ISL_2573644	Virology Department, Hospices Civils de Lyon	Virology Department, Hospices Civils de Lyon	Gaymard,A., Pichon,M. and Morfin,F.
EPI_ISL_2573660	Epidemiology and Demography, KEMRI - Wellcome Trust Research Programme, Centre for Geographic Medicine Research	Epidemiology and Demography, KEMRI - Wellcome Trust Research Programme, Centre for Geographic Medicine Research	Oketch,J.W., Kamau,E., Otieno,J.R., Mwema,A., Lewa,C., Agoti,C.N. and Nokes,J.D.
EPI_ISL_2573661, EPI_ISL_2573662, EPI_ISL_2573663, EPI_ISL_2573664, EPI_ISL_2573665, EPI_ISL_2573666, EPI_ISL_2573667, EPI_ISL_2573668, EPI_ISL_2573669, EPI_ISL_2573670, EPI_ISL_2573671, EPI_ISL_2573672, EPI_ISL_2573673			
see above	Centre for Infectious Diseases Research, Diagnostics and laboratory Surveillance, National Institute for Public Health and the Environment	Centre for Infectious Diseases Research, Diagnostics and laboratory Surveillance, National Institute for Public Health and the Environment	Meijer,A. and Overduin,P.

EPI_ISL_2573674, EPI_ISL_2573675, EPI_ISL_2573676, EPI_ISL_2573677, EPI_ISL_2573678	Department for Infectious Diseases Virology, University of Heidelberg	Department for Infectious Diseases Virology, University of Heidelberg	Tabatabai,J., Thielen,A., Lehners,N., Daeumer,M. and Schnitzler,P.
EPI_ISL_2573679, EPI_ISL_2573682, EPI_ISL_2573683, EPI_ISL_2573685, EPI_ISL_2573686, EPI_ISL_2573689, EPI_ISL_2573690, EPI_ISL_2573691, EPI_ISL_2573692, EPI_ISL_2573694, EPI_ISL_2573695, EPI_ISL_2573696, EPI_ISL_2573698, EPI_ISL_2573701, EPI_ISL_2573702, EPI_ISL_2573703, EPI_ISL_2573704, EPI_ISL_2573705, EPI_ISL_2573707, EPI_ISL_2573708, EPI_ISL_2573709, EPI_ISL_2573711, EPI_ISL_2573712, EPI_ISL_2573713, EPI_ISL_2573714, EPI_ISL_2573715, EPI_ISL_2573719, EPI_ISL_2573720, EPI_ISL_2573724, EPI_ISL_2573726, EPI_ISL_2573727, EPI_ISL_2573729, EPI_ISL_2573730, EPI_ISL_2573732, EPI_ISL_2573733, EPI_ISL_2573734, EPI_ISL_2573736, EPI_ISL_2573737, EPI_ISL_2573740, EPI_ISL_2573741, EPI_ISL_2573742, EPI_ISL_2573744, EPI_ISL_2573745, EPI_ISL_2573746, EPI_ISL_2573747, EPI_ISL_2573748, EPI_ISL_2573749, EPI_ISL_2573751, EPI_ISL_2573752, EPI_ISL_2573753, EPI_ISL_2573754, EPI_ISL_2573756, EPI_ISL_2573758, EPI_ISL_2573760, EPI_ISL_2573761, EPI_ISL_2573762, EPI_ISL_2573763, EPI_ISL_2573764, EPI_ISL_2573765, EPI_ISL_2573766			
see above	University of Wuerzburg, Institute of Virology and Immunobiology	University of Wuerzburg, Institute of Virology and Immunobiology	Prifert,C., Hofmann,D. and Weissbrich,B.
EPI_ISL_2573770, EPI_ISL_2573771, EPI_ISL_2573772, EPI_ISL_2573773, EPI_ISL_2573774	Centre for Infectious Diseases Virology, University of Heidelberg	Centre for Infectious Diseases Virology, University of Heidelberg	Ihling,C.M., Schnitzler,P., Heinrich,N., Mangu,C., Sudi,L., Souares,A., Gies,S., Sie,A., Coulibaly,B., Ouedraogo,A.T., Mordmueller,B., Held,J., Adegnika,A.A., Fernandez,J.F., Eckerle,I., May,J., Hogan,B., Eibach,D., Owusu-Dabo,E. and Tabatabai,J.
EPI_ISL_2573775, EPI_ISL_2573776, EPI_ISL_2573777, EPI_ISL_2573778, EPI_ISL_2573779, EPI_ISL_2573780, EPI_ISL_2573781, EPI_ISL_2573782, EPI_ISL_2573783, EPI_ISL_2573784, EPI_ISL_2573785, EPI_ISL_2573786, EPI_ISL_2573787, EPI_ISL_2573788, EPI_ISL_2573789, EPI_ISL_2573790, EPI_ISL_2573791, EPI_ISL_2573792, EPI_ISL_2573793, EPI_ISL_2573794, EPI_ISL_2573795, EPI_ISL_2573796, EPI_ISL_2573797, EPI_ISL_2573798, EPI_ISL_2573799, EPI_ISL_2573800, EPI_ISL_2573801, EPI_ISL_2573802, EPI_ISL_2573803, EPI_ISL_2573804, EPI_ISL_2573805, EPI_ISL_2573806			
see above	Virology, Noguchi Memorial Institute for Medical Research	Virology, Noguchi Memorial Institute for Medical Research	Obodai,E., Odoom,J.K., Adiku,T., Goka,B., Biere,B., Wolff,T., Schweiger,B. and Reiche,J.
EPI_ISL_2573807, EPI_ISL_2573808, EPI_ISL_2573809, EPI_ISL_2573810, EPI_ISL_2573811, EPI_ISL_2573812	Centro de Estudios en Salud, Universidad Del Valle De Guatemala	Centro de Estudios en Salud, Universidad Del Valle De Guatemala	Lupo,P., Jara,J., Alvis,J.P., Pennington,P., Castillo,L., Moreno,B. and Albrego,L.
EPI_ISL_2573814, EPI_ISL_2573815	Microbiology, King George Medical University	Microbiology, King George Medical University	Singh,A.K., Jain,B., Dangi,T., Verma,A., Dwivedi,M., Prakash,S. and Jain,A.
EPI_ISL_2573816	Centre for Interdisciplinary Research in Basic Science	Centre for Interdisciplinary Research in Basic Science	Haider,M.S.H., Naqvi,I.H., Broor,S., Ahmed,A. and Parveen,S.
EPI_ISL_2573831, EPI_ISL_2573833, EPI_ISL_2573834, EPI_ISL_2573835, EPI_ISL_2573836, EPI_ISL_2573837, EPI_ISL_2573838, EPI_ISL_2573839, EPI_ISL_2573840, EPI_ISL_2573841, EPI_ISL_2573842, EPI_ISL_2573843			
see above	Virology Section, Department of Microbiology, King George's Medical University	Virology Section, Department of Microbiology, King George's Medical University	Singh,A.K., Jain,A., Jain,B., Dangi,T., Verma,A.K., Dwivedi,M. and Kushwaha,R.
EPI_ISL_2573844, EPI_ISL_2573845, EPI_ISL_2573846, EPI_ISL_2573847, EPI_ISL_2573848	Pushpagiri Research Centre, Pushpagiri Institute of Medical Science and Research Centre, Pushpagir Medical College Hospital	Pushpagiri Research Centre, Pushpagiri Institute of Medical Science and Research Centre, Pushpagir Medical College Hospital	George,S., Jagan,O.A., Jose,S., John,N.S., Bai,S., Chandy,S. and Manoharan,A.
EPI_ISL_2573878	Department of Virology, Tehran University of Medical Sciences	Department of Virology, Tehran University of Medical Sciences	Salimi,V., Shatizadeh Malekshahi,S., Ghavami,N. and Mokhtari Azad,T.
EPI_ISL_2573879	Department of Virology, School of Public Health, Tehran University of Medical Sciences	Department of Virology, School of Public Health, Tehran University of Medical Sciences	Salimi,V., Samieipoor,Y., Ghavami,N. and Mokhtariazad,T.
EPI_ISL_2573880	Department of Virology, Tehran University of Medical Sciences	Department of Virology, Tehran University of Medical Sciences	Salimi,V., Shatizadeh Malekshahi,S., Ghavami,N. and Mokhtari Azad,T.
EPI_ISL_2573889, EPI_ISL_2573890, EPI_ISL_2573891, EPI_ISL_2573892, EPI_ISL_2573893, EPI_ISL_2573894, EPI_ISL_2573895, EPI_ISL_2573896, EPI_ISL_2573897	Molecular Medicine Laboratory of Virology, Sapienza University	Molecular Medicine Laboratory of Virology, Sapienza University	Pierangeli,A.
EPI_ISL_2573911, EPI_ISL_2573912, EPI_ISL_2573913, EPI_ISL_2573914, EPI_ISL_2573921, EPI_ISL_2573922, EPI_ISL_2573923, EPI_ISL_2573924, EPI_ISL_2573925, EPI_ISL_2573926, EPI_ISL_2573927, EPI_ISL_2573928, EPI_ISL_2573929, EPI_ISL_2573930, EPI_ISL_2573931, EPI_ISL_2573932, EPI_ISL_2573933, EPI_ISL_2573934, EPI_ISL_2573935, EPI_ISL_2573936, EPI_ISL_2573937, EPI_ISL_2573938, EPI_ISL_2573939			
see above	Pediatric Clinic 1, Department of Pathophysiology and Transplantation, University of Milan and Fondazione IRCCS Ca Granda	Pediatric Clinic 1, Department of Pathophysiology and Transplantation, University of Milan and Fondazione IRCCS Ca Granda	Esposito,S., Zampiero,A., Piralla,A. and Principi,N.
EPI_ISL_2573940, EPI_ISL_2573941, EPI_ISL_2573942	Akinobu Hibino Niigata University, International Health, Public Health	Akinobu Hibino Niigata University, International Health, Public Health	Hibino,A., Saito,R., Shoubugawa,Y. and Tomimoto,K.
EPI_ISL_2573943, EPI_ISL_2573944	Miho Kobayashi Gunma Prefectural Institute of Public Health and Environmental Sciences	Miho Kobayashi Gunma Prefectural Institute of Public Health and Environmental Sciences	Kobayashi,M. and Kimura,H.
EPI_ISL_2573945	Akinobu Hibino Niigata University, International Health, Public Health	Akinobu Hibino Niigata University, International Health, Public Health	Hibino,A., Saito,R., Shoubugawa,Y. and Saito,T.
EPI_ISL_2573962, EPI_ISL_2573963, EPI_ISL_2573964, EPI_ISL_2573965, EPI_ISL_2573966, EPI_ISL_2573967	Akinobu Hibino Niigata University, International Health, Public Health	Akinobu Hibino Niigata University, International Health, Public Health	Hibino,A., Saito,R., Shoubugawa,Y. and Nagata,N.
EPI_ISL_2573969, EPI_ISL_2573970	Akinobu Hibino Niigata University, International Health, Public Health	Akinobu Hibino Niigata University, International Health, Public Health	Hibino,A., Saito,R., Shoubugawa,Y. and Takei,T.
EPI_ISL_2573971, EPI_ISL_2573972, EPI_ISL_2573973, EPI_ISL_2573974, EPI_ISL_2573975, EPI_ISL_2573976, EPI_ISL_2573977, EPI_ISL_2573978, EPI_ISL_2573979, EPI_ISL_2573980, EPI_ISL_2573981, EPI_ISL_2573982, EPI_ISL_2573983			
see above	Akinobu Hibino Niigata University, International Health, Public Health	Akinobu Hibino Niigata University, International Health, Public Health	Hibino,A., Saito,R., Shoubugawa,Y. and Shimada,Y.
EPI_ISL_2573984, EPI_ISL_2573985, EPI_ISL_2573986, EPI_ISL_2573987, EPI_ISL_2573988, EPI_ISL_2573989, EPI_ISL_2573990, EPI_ISL_2573991, EPI_ISL_2573992, EPI_ISL_2573993, EPI_ISL_2573994, EPI_ISL_2573995, EPI_ISL_2573996			
see above	Akinobu Hibino Niigata University, International Health, Public Health	Akinobu Hibino Niigata University, International Health, Public Health	Hibino,A., Saito,R., Shoubugawa,Y. and Taniguchi,K.
EPI_ISL_2574001, EPI_ISL_2574002, EPI_ISL_2574003, EPI_ISL_2574004, EPI_ISL_2574005, EPI_ISL_2574007	Akinobu Hibino Niigata University, International Health, Public Health	Akinobu Hibino Niigata University, International Health, Public Health	Hibino,A., Saito,R., Shoubugawa,Y. and Sano,Y.
EPI_ISL_2574129, EPI_ISL_2574133, EPI_ISL_2574134, EPI_ISL_2574136, EPI_ISL_2574137, EPI_ISL_2574140, EPI_ISL_2574141, EPI_ISL_2574142, EPI_ISL_2574143, EPI_ISL_2574145, EPI_ISL_2574147, EPI_ISL_2574148, EPI_ISL_2574150, EPI_ISL_2574153, EPI_ISL_2574154, EPI_ISL_2574155, EPI_ISL_2574156, EPI_ISL_2574157, EPI_ISL_2574159, EPI_ISL_2574160, EPI_ISL_2574162, EPI_ISL_2574164, EPI_ISL_2574165, EPI_ISL_2574166, EPI_ISL_2574167, EPI_ISL_2574168, EPI_ISL_2574172, EPI_ISL_2574173, EPI_ISL_2574178, EPI_ISL_2574180, EPI_ISL_2574181, EPI_ISL_2574183, EPI_ISL_2574184, EPI_ISL_2574186, EPI_ISL_2574187, EPI_ISL_2574188, EPI_ISL_2574190, EPI_ISL_2574192, EPI_ISL_2574195, EPI_ISL_2574196, EPI_ISL_2574197, EPI_ISL_2574199, EPI_ISL_2574200, EPI_ISL_2574201, EPI_ISL_2574202, EPI_ISL_2574203, EPI_ISL_2574204, EPI_ISL_2574207, EPI_ISL_2574208, EPI_ISL_2574209, EPI_ISL_2574210, EPI_ISL_2574212, EPI_ISL_2574214, EPI_ISL_2574216, EPI_ISL_2574217, EPI_ISL_2574218, EPI_ISL_2574219, EPI_ISL_2574221, EPI_ISL_2574222, EPI_ISL_2574223			
see above	University of Wuerzburg, Institute of Virology and Immunobiology	University of Wuerzburg, Institute of Virology and Immunobiology	Prifert,C., Hofmann,D. and Weissbrich,B.
EPI_ISL_2574227, EPI_ISL_2574228, EPI_ISL_2574229, EPI_ISL_2574230, EPI_ISL_2574231	Centre for Infectious Diseases Virology, University of Heidelberg	Centre for Infectious Diseases Virology, University of Heidelberg	Ihling,C.M., Schnitzler,P., Heinrich,N., Mangu,C., Sudi,L., Souares,A., Gies,S., Sie,A., Coulibaly,B., Ouedraogo,A.T., Mordmueller,B., Held,J., Adegnika,A.A., Fernandez,J.F., Eckerle,I., May,J., Hogan,B., Eibach,D., Owusu-Dabo,E. and Tabatabai,J.
EPI_ISL_2574232, EPI_ISL_2574234, EPI_ISL_2574235, EPI_ISL_2574236, EPI_ISL_2574237, EPI_ISL_2574238, EPI_ISL_2574239, EPI_ISL_2574240, EPI_ISL_2574241, EPI_ISL_2574242, EPI_ISL_2574243, EPI_ISL_2574244, EPI_ISL_2574245, EPI_ISL_2574247, EPI_ISL_2574248, EPI_ISL_2574249, EPI_ISL_2574250, EPI_ISL_2574251, EPI_ISL_2574252, EPI_ISL_2574253, EPI_ISL_2574254, EPI_ISL_2574256, EPI_ISL_2574257, EPI_ISL_2574258, EPI_ISL_2574259, EPI_ISL_2574260, EPI_ISL_2574261, EPI_ISL_2574262, EPI_ISL_2574263, EPI_ISL_2574264, EPI_ISL_2574265, EPI_ISL_2574266			
see above	Virology, Noguchi Memorial Institute for Medical Research	Virology, Noguchi Memorial Institute for Medical Research	Obodai,E., Odoom,J.K., Adiku,T., Goka,B., Biere,B., Wolff,T., Schweiger,B. and Reiche,J.
EPI_ISL_2574267, EPI_ISL_2574268, EPI_ISL_2574270, EPI_ISL_2574271, EPI_ISL_2574272, EPI_ISL_2574273	Centro de Estudios en Salud, Universidad Del Valle De Guatemala	Centro de Estudios en Salud, Universidad Del Valle De Guatemala	Lupo,P., Jara,J., Alvis,J.P., Pennington,P., Castillo,L., Moreno,B. and Albrego,L.
EPI_ISL_2574275, EPI_ISL_2574276	Microbiology, King George Medical University	Microbiology, King George Medical University	Singh,A.K., Jain,B., Dangi,T., Verma,A., Dwivedi,M., Prakash,S. and Jain,A.
EPI_ISL_2574277	Centre for Interdisciplinary Research in Basic Science	Centre for Interdisciplinary Research in Basic Science	Haider,M.S.H., Naqvi,I.H., Broor,S., Ahmed,A. and Parveen,S.
EPI_ISL_2574293, EPI_ISL_2574296, EPI_ISL_2574297, EPI_ISL_2574298, EPI_ISL_2574299, EPI_ISL_2574300, EPI_ISL_2574301, EPI_ISL_2574302, EPI_ISL_2574303, EPI_ISL_2574304, EPI_ISL_2574305, EPI_ISL_2574306			
see above	Virology Section, Department of Microbiology, King George's Medical University	Virology Section, Department of Microbiology, King George's Medical University	Singh,A.K., Jain,A., Jain,B., Dangi,T., Verma,A.K., Dwivedi,M. and Kushwaha,R.
EPI_ISL_2574308, EPI_ISL_2574309, EPI_ISL_2574310, EPI_ISL_2574311, EPI_ISL_2574312	Pushpagiri Research Centre, Pushpagiri Institute of Medical Science and Research Centre, Pushpagir Medical College Hospital	Pushpagiri Research Centre, Pushpagiri Institute of Medical Science and Research Centre, Pushpagir Medical College Hospital	George,S., Jagan,O.A., Jose,S., John,N.S., Bai,S., Chandy,S. and Manoharan,A.
EPI_ISL_2574344	Department of Virology, Tehran University of Medical Sciences	Department of Virology, Tehran University of Medical Sciences	Salimi,V., Shatizadeh Malekshahi,S., Ghavami,N. and Mokhtari Azad,T.
EPI_ISL_2574345	Department of Virology, School of Public Health, Tehran University of Medical Sciences	Department of Virology, School of Public Health, Tehran University of Medical Sciences	Salimi,V., Samieipoor,Y., Ghavami,N. and Mokhtariazad,T.
EPI_ISL_2574346	Department of Virology, Tehran University of Medical Sciences	Department of Virology, Tehran University of Medical Sciences	Salimi,V., Shatizadeh Malekshahi,S., Ghavami,N. and Mokhtari Azad,T.
EPI_ISL_2574356, EPI_ISL_2574357, EPI_ISL_2574358, EPI_ISL_2574359, EPI_ISL_2574361, EPI_ISL_2574362, EPI_ISL_2574363, EPI_ISL_2574364, EPI_ISL_2574365	Molecular Medicine Laboratory of Virology, Sapienza University	Molecular Medicine Laboratory of Virology, Sapienza University	Pierangeli,A.
EPI_ISL_2574381, EPI_ISL_2574382, EPI_ISL_2574383, EPI_ISL_2574384, EPI_ISL_2574391, EPI_ISL_2574392, EPI_ISL_2574393, EPI_ISL_2574394, EPI_ISL_2574395, EPI_ISL_2574396, EPI_ISL_2574397, EPI_ISL_2574398, EPI_ISL_2574399, EPI_ISL_2574400, EPI_ISL_2574401, EPI_ISL_2574402, EPI_ISL_2574403, EPI_ISL_2574404, EPI_ISL_2574405, EPI_ISL_2574406, EPI_ISL_2574407, EPI_ISL_2574408, EPI_ISL_2574409			
see above	Pediatric Clinic 1, Department of Pathophysiology and Transplantation, University of Milan and Fondazione IRCCS Ca Granda	Pediatric Clinic 1, Department of Pathophysiology and Transplantation, University of Milan and Fondazione IRCCS Ca Granda	Esposito,S., Zampiero,A., Piralla,A. and Principi,N.
EPI_ISL_2574410, EPI_ISL_2574411, EPI_ISL_2574412	Akinobu Hibino Niigata University, International Health, Public Health	Akinobu Hibino Niigata University, International Health, Public Health	Hibino,A., Saito,R., Shoubugawa,Y. and Tomimoto,K.
EPI_ISL_2574413, EPI_ISL_2574414	Miho Kobayashi Gunma Prefectural Institute of Public Health and Environmental Sciences	Miho Kobayashi Gunma Prefectural Institute of Public Health and Environmental Sciences	Kobayashi,M. and Kimura,H.
EPI_ISL_2574415	Akinobu Hibino Niigata University, International Health, Public Health	Akinobu Hibino Niigata University, International Health, Public Health	Hibino,A., Saito,R., Shoubugawa,Y. and Saito,T.
EPI_ISL_2574432, EPI_ISL_2574433, EPI_ISL_2574434, EPI_ISL_2574435, EPI_ISL_2574436, EPI_ISL_2574437	Akinobu Hibino Niigata University, International Health, Public Health	Akinobu Hibino Niigata University, International Health, Public Health	Hibino,A., Saito,R., Shoubugawa,Y. and Nagata,N.
EPI_ISL_2574439, EPI_ISL_2574440	Akinobu Hibino Niigata University, International Health, Public Health	Akinobu Hibino Niigata University, International Health, Public Health	Hibino,A., Saito,R., Shoubugawa,Y. and Takei,T.
EPI_ISL_2574441, EPI_ISL_2574442, EPI_ISL_2574443, EPI_ISL_2574444, EPI_ISL_2574445, EPI_ISL_2574446, EPI_ISL_2574447, EPI_ISL_2574448, EPI_ISL_2574449, EPI_ISL_2574450, EPI_ISL_2574451, EPI_ISL_2574452, EPI_ISL_2574453			
see above	Akinobu Hibino Niigata University, International Health, Public Health	Akinobu Hibino Niigata University, International Health, Public Health	Hibino,A., Saito,R., Shoubugawa,Y. and Shimada,Y.
EPI_ISL_2574454, EPI_ISL_2574455, EPI_ISL_2574456, EPI_ISL_2574457, EPI_ISL_2574458, EPI_ISL_2574459, EPI_ISL_2574460, EPI_ISL_2574461, EPI_ISL_2574462, EPI_ISL_2574463, EPI_ISL_2574464, EPI_ISL_2574466, EPI_ISL_2574467			
see above	Akinobu Hibino Niigata University, International Health, Public Health	Akinobu Hibino Niigata University, International Health, Public Health	Hibino,A., Saito,R., Shoubugawa,Y. and Taniguchi,K.
EPI_ISL_2574472, EPI_ISL_2574473, EPI_ISL_2574474, EPI_ISL_2574475, EPI_ISL_2574476, EPI_ISL_2574477	Akinobu Hibino Niigata University, International Health, Public Health	Akinobu Hibino Niigata University, International Health, Public Health	Hibino,A., Saito,R., Shoubugawa,Y. and Sano,Y.

EPI_ISL_2574478, EPI_ISL_2574479, EPI_ISL_2574480, EPI_ISL_2574481	Akinobu Hibino Niigata University, International Health, Public Health	Akinobu Hibino Niigata University, International Health, Public Health	Hibino,A., Saito,R., Shoubugawa,Y., Makiya,T. and Takefuta,K.
EPI_ISL_2574482	Minoru Nidaira Okinawa Prefectural Institute of Health and Environment, Department of Biological Science	Minoru Nidaira Okinawa Prefectural Institute of Health and Environment, Department of Biological Science	Nidaira,M., Kato,T. and Arakaki,E.
EPI_ISL_2574483, EPI_ISL_2574484, EPI_ISL_2574485, EPI_ISL_2574486, EPI_ISL_2574487, EPI_ISL_2574488	Akinobu Hibino Niigata University, International Health, Public Health	Akinobu Hibino Niigata University, International Health, Public Health	Hibino,A., Saito,R., Shoubugawa,Y. and Saito,N.
EPI_ISL_2574489, EPI_ISL_2574490, EPI_ISL_2574491, EPI_ISL_2574492, EPI_ISL_2574493, EPI_ISL_2574494, EPI_ISL_2574495, EPI_ISL_2574496, EPI_ISL_2574497	Akinobu Hibino Niigata University, International Health, Public Health	Akinobu Hibino Niigata University, International Health, Public Health	Hibino,A., Saito,R., Shoubugawa,Y. and Tanaka,T.
EPI_ISL_2574498, EPI_ISL_2574499, EPI_ISL_2574501, EPI_ISL_2574502	Akinobu Hibino Niigata University, International Health, Public Health	Akinobu Hibino Niigata University, International Health, Public Health	Hibino,A., Saito,R., Shoubugawa,Y. and Kaji,H.
EPI_ISL_2574503, EPI_ISL_2574504	Akinobu Hibino Niigata University, International Health, Public Health	Akinobu Hibino Niigata University, International Health, Public Health	Hibino,A., Saito,R., Shoubugawa,Y. and Suzuki,E.
EPI_ISL_2574505	Miho Kobayashi Gunma Prefectural Institute of Public Health and Environmental Sciences	Miho Kobayashi Gunma Prefectural Institute of Public Health and Environmental Sciences	Kobayashi,M. and Kimura,H.
EPI_ISL_2574508, EPI_ISL_2574509, EPI_ISL_2574510, EPI_ISL_2574511, EPI_ISL_2574512, EPI_ISL_2574513, EPI_ISL_2574514	Epidemiology and Demography, KEMRI-Wellcome Trust Research Programme	Epidemiology and Demography, KEMRI-Wellcome Trust Research Programme	Kamau,E.M., Agoti,C.N., Owor,B., Lewa,C., Bett,A., Oketch,J., Cane,P.A. and Nokes,J.D.
EPI_ISL_2574517, EPI_ISL_2574520	Epidemiology and Demography, KEMRI - Wellcome Trust Research Programme, Centre for Geographic Medicine Research	Epidemiology and Demography, KEMRI - Wellcome Trust Research Programme, Centre for Geographic Medicine Research	Oketch,J.W., Kamau,E., Otieno,J.R., Mwema,A., Lewa,C., Agoti,C.N. and Nokes,J.D.
EPI_ISL_2574573, EPI_ISL_2574575, EPI_ISL_2574576, EPI_ISL_2574577, EPI_ISL_2574581, EPI_ISL_2574583, EPI_ISL_2574586, EPI_ISL_2574589, EPI_ISL_2574590, EPI_ISL_2574593, EPI_ISL_2574594			
see above	Department of Epidemiology and Demography, Kenya Medical Research Institute (KEMRI) - Wellcome Trust Research Programme	Department of Epidemiology and Demography, Kenya Medical Research Institute (KEMRI) - Wellcome Trust Research Programme	Otieno,J.R., Agoti,C.N., Gitahi,C.W., Bett,A., Ngama,M., Medley,G.F., Cane,P.A. and Nokes,J.D.
EPI_ISL_2574595, EPI_ISL_2574597, EPI_ISL_2574598, EPI_ISL_2574600, EPI_ISL_2574601, EPI_ISL_2574603, EPI_ISL_2574606, EPI_ISL_2574609, EPI_ISL_2574611, EPI_ISL_2574613, EPI_ISL_2574614, EPI_ISL_2574615, EPI_ISL_2574616, EPI_ISL_2574617, EPI_ISL_2574618, EPI_ISL_2574621, EPI_ISL_2574625, EPI_ISL_2574647, EPI_ISL_2574654			
see above	Department of Epidemiology and Demography, Kenya Medical Research Institute (KEMRI) - Wellcome Trust Research Programme	Department of Epidemiology and Demography, Kenya Medical Research Institute (KEMRI) - Wellcome Trust Research Programme	Otieno,J.R., Kamau,E.M., Agoti,C.N., Lewa,C., Bett,A., Ngama,M., Cane,P.A. and Nokes,J.D.
EPI_ISL_2574655, EPI_ISL_2574656, EPI_ISL_2574657, EPI_ISL_2574658, EPI_ISL_2574659, EPI_ISL_2574660, EPI_ISL_2574661, EPI_ISL_2574662, EPI_ISL_2574663, EPI_ISL_2574664, EPI_ISL_2574665			
see above	Epidemiology and Demography, Kenya Medical Research Institute (KEMRI) - Wellcome Trust Research Programme	Epidemiology and Demography, Kenya Medical Research Institute (KEMRI) - Wellcome Trust Research Programme	Agoti,C.N., Otieno,J.R., Gitahi,C.W., Cane,P.A. and Nokes,D.J.
EPI_ISL_2574666, EPI_ISL_2574667, EPI_ISL_2574668, EPI_ISL_2574669, EPI_ISL_2574670, EPI_ISL_2574671, EPI_ISL_2574672, EPI_ISL_2574673, EPI_ISL_2574674, EPI_ISL_2574675, EPI_ISL_2574676, EPI_ISL_2574677, EPI_ISL_2574678, EPI_ISL_2574679, EPI_ISL_2574680, EPI_ISL_2574681, EPI_ISL_2574682, EPI_ISL_2574683, EPI_ISL_2574684, EPI_ISL_2574685, EPI_ISL_2574686, EPI_ISL_2574687, EPI_ISL_2574688, EPI_ISL_2574689, EPI_ISL_2574690, EPI_ISL_2574691, EPI_ISL_2574692, EPI_ISL_2574693, EPI_ISL_2574694, EPI_ISL_2574695, EPI_ISL_2574696, EPI_ISL_2574697, EPI_ISL_2574698, EPI_ISL_2574699, EPI_ISL_2574700, EPI_ISL_2574701, EPI_ISL_2574702, EPI_ISL_2574703, EPI_ISL_2574704, EPI_ISL_2574705, EPI_ISL_2574706, EPI_ISL_2574707, EPI_ISL_2574708, EPI_ISL_2574709, EPI_ISL_2574710, EPI_ISL_2574711, EPI_ISL_2574712, EPI_ISL_2574713, EPI_ISL_2574714, EPI_ISL_2574715, EPI_ISL_2574716, EPI_ISL_2574717, EPI_ISL_2574718, EPI_ISL_2574719, EPI_ISL_2574720, EPI_ISL_2574721, EPI_ISL_2574722, EPI_ISL_2574723, EPI_ISL_2574724, EPI_ISL_2574725, EPI_ISL_2574726, EPI_ISL_2574727			
see above	Department of Epidemiology and Demography, Kenya Medical Research Institute (KEMRI) - Wellcome Trust Research Programme	Department of Epidemiology and Demography, Kenya Medical Research Institute (KEMRI) - Wellcome Trust Research Programme	Otieno,J.R., Kamau,E.M., Agoti,C.N., Lewa,C., Bett,A., Ngama,M., Cane,P.A. and Nokes,J.D.
EPI_ISL_2574730, EPI_ISL_2574731, EPI_ISL_2574732, EPI_ISL_2574733, EPI_ISL_2574734, EPI_ISL_2574735, EPI_ISL_2574736, EPI_ISL_2574737, EPI_ISL_2574738, EPI_ISL_2574739, EPI_ISL_2574740, EPI_ISL_2574741, EPI_ISL_2574742, EPI_ISL_2574743, EPI_ISL_2574744, EPI_ISL_2574745, EPI_ISL_2574746, EPI_ISL_2574747, EPI_ISL_2574748, EPI_ISL_2574749, EPI_ISL_2574750, EPI_ISL_2574751, EPI_ISL_2574752, EPI_ISL_2574753, EPI_ISL_2574754, EPI_ISL_2574755, EPI_ISL_2574756, EPI_ISL_2574757, EPI_ISL_2574758, EPI_ISL_2574759, EPI_ISL_2574760			
see above	Medicine, American University of Beirut	Medicine, American University of Beirut	Abou-El-Hassan,H. and Zaraket,H.
EPI_ISL_2574808	Epidemiology and Demography, KEMRI - Wellcome Trust Research Programme, Centre for Geographic Medicine Research	Epidemiology and Demography, KEMRI - Wellcome Trust Research Programme, Centre for Geographic Medicine Research	Oketch,J.W., Kamau,E., Otieno,J.R., Mwema,A., Lewa,C., Agoti,C.N. and Nokes,J.D.
EPI_ISL_2574810	Department of Medical Research, Ministry of Health and Sports, No. 5, Ziwaka Road, Dagon Township	Department of Medical Research, Ministry of Health and Sports, No. 5, Ziwaka Road, Dagon Township	Aye,K.T., Latt,A.Z. and Thwe,H.H.
EPI_ISL_2574811, EPI_ISL_2574812	Department of Medical Research, Ministry of Health and Sports, No. 5, Ziwaka Road, Dagon Township	Department of Medical Research, Ministry of Health and Sports, No. 5, Ziwaka Road, Dagon Township	Aye,K.T.
EPI_ISL_2574813, EPI_ISL_2574814, EPI_ISL_2574815, EPI_ISL_2574816	Akinobu Hibino Niigata University, International Health, Public Health	Akinobu Hibino Niigata University, International Health, Public Health	Hibino,A., Saito,R., Shoubugawa,Y., Htay,H.T., Khin,Y.O., Yi,Y.M. and Yadanar,K.
EPI_ISL_2574817, EPI_ISL_2574818	Wint Wint Phyu Niigat University, International Health, Public Health	Wint Wint Phyu Niigat University, International Health, Public Health	Phyu,W.W. and Saito,R.
EPI_ISL_2574835, EPI_ISL_2574836	MedImmune	MedImmune	Moldt,B.
EPI_ISL_2574837, EPI_ISL_2574838, EPI_ISL_2574839, EPI_ISL_2574840, EPI_ISL_2574841	Centre for Infectious Diseases Research, Diagnostics and laboratory Surveillance, National Institute for Public Health and the Environment	Centre for Infectious Diseases Research, Diagnostics and laboratory Surveillance, National Institute for Public Health and the Environment	Meijer,A. and Overduin,P.
EPI_ISL_2574842	Virology, University of Ibadan, College of Medicine (UCH)	Virology, University of Ibadan, College of Medicine (UCH)	Ogunsemowo,O.S., Odaibo,G.N. and Olaleye,D.O.
EPI_ISL_2574843	Virology, University of Ibadan, College of Medicine (UCH)	Virology, University of Ibadan, College of Medicine (UCH)	Ogunsemowo,O., Odaibo,G.N. and Olaleye,D.O.
EPI_ISL_2574844, EPI_ISL_2574845	Virology, University of Ibadan, College of Medicine (UCH)	Virology, University of Ibadan, College of Medicine (UCH)	Ogunsemowo,O.S., Odaibo,G.N. and Olaleye,D.O.
EPI_ISL_2574846	Virology, University of Ibadan, College of Medicine (UCH)	Virology, University of Ibadan, College of Medicine (UCH)	Ogunsemowo,O., Odaibo,G.N. and Olaleye,D.O.
EPI_ISL_2574847, EPI_ISL_2574848, EPI_ISL_2574849, EPI_ISL_2574850, EPI_ISL_2574851, EPI_ISL_2574852, EPI_ISL_2574853, EPI_ISL_2574854, EPI_ISL_2574855	Centro de Estudios en Salud, Universidad Del Valle De Guatemala	Centro de Estudios en Salud, Universidad Del Valle De Guatemala	Lupo,P., Jara,J., Alvis,J.P., Pennington,P., Castillo,L., Moreno,B. and Albrego,L.
EPI_ISL_2574871, EPI_ISL_2574872	Michiko Okamoto Tohoku University Graduate School of Medicine, Virology	Michiko Okamoto Tohoku University Graduate School of Medicine, Virology	Okamoto,M. and Oshitani,H.
EPI_ISL_2574873, EPI_ISL_2574874, EPI_ISL_2574879, EPI_ISL_2574880, EPI_ISL_2574881, EPI_ISL_2574882, EPI_ISL_2574883, EPI_ISL_2574884, EPI_ISL_2574891, EPI_ISL_2574892, EPI_ISL_2574893, EPI_ISL_2574894, EPI_ISL_2574895, EPI_ISL_2574896, EPI_ISL_2574897, EPI_ISL_2574898			
see above	Virology, Tohoku University Graduate School of Medicine	Virology, Tohoku University Graduate School of Medicine	Malasao,R., Okamoto,M., Chaimongkol,N., Imamura,T., Tohma,K., Dapat,I., Dapat,C., Suzuki,A., Saito,M., Saito,M., Tamaki,R., Segubre-Mercado,E., Igoy,M.A.U., Lupisan,S., Olveda,R. and Oshitani,H.
EPI_ISL_2574936	Department of Infectious Diseases, National Health Institute Doutor Ricardo Jorge	Department of Infectious Diseases, National Health Institute Doutor Ricardo Jorge	Saez-Lopez,E., Cristovao,P., Costa,I., Pechirra,P., Conde,P. and Guiomar,R.
EPI_ISL_2574942	Lab. of Molecular Virology, Research Institute of Influenza	Lab. of Molecular Virology, Research Institute of Influenza	Sintsova,K.S., Krivitskaya,V.Z., Sverlova,M.V., Petrova,E.R., Fadeev,A.V. and Komissarov,A.B.
EPI_ISL_2574943, EPI_ISL_2574950, EPI_ISL_2574954	Laboratory of molecular virology, Research Institute of Influenza, Ministry of Healthcare of the Russian Federation	Laboratory of molecular virology, Research Institute of Influenza, Ministry of Healthcare of the Russian Federation	Komissarova,K.S., Krivitskaya,V.Z., Sverlova,M.V., Petrova,E.R., Fadeev,A.V. and Komissarov,A.B.
EPI_ISL_2574955	Lab. of Molecular Virology, Research Institute of Influenza	Lab. of Molecular Virology, Research Institute of Influenza	Sintsova,K.S., Krivitskaya,V.Z., Sverlova,M.V., Petrova,E.R., Fadeev,A.V. and Komissarov,A.B.
EPI_ISL_2574956	Etiology and Epidemiology Department, Smorodintsev Research Institute of Influenza	Etiology and Epidemiology Department, Smorodintsev Research Institute of Influenza	Komissarova,K.S., Krivitskaya,V.Z., Sverlova,M.V., Petrova,E.R. and Ivanova,A.A.
EPI_ISL_2574959	Laboratory of molecular virology, Research Institute of Influenza, Ministry of Healthcare of the Russian Federation	Laboratory of molecular virology, Research Institute of Influenza, Ministry of Healthcare of the Russian Federation	Komissarova,K.S., Krivitskaya,V.Z., Sverlova,M.V., Petrova,E.R., Fadeev,A.V. and Komissarov,A.B.
EPI_ISL_2574960	Etiology and Epidemiology Department, Smorodintsev Research Institute of Influenza	Etiology and Epidemiology Department, Smorodintsev Research Institute of Influenza	Komissarova,K.S., Krivitskaya,V.Z., Sverlova,M.V., Petrova,E.R. and Ivanova,A.A.
EPI_ISL_2574961	Lab. of Molecular Virology, Research Institute of Influenza	Lab. of Molecular Virology, Research Institute of Influenza	Sintsova,K.S., Krivitskaya,V.Z., Sverlova,M.V., Petrova,E.R., Fadeev,A.V. and Komissarov,A.B.
EPI_ISL_2574962	Laboratory of molecular virology, Research Institute of Influenza, Ministry of Healthcare of the Russian Federation	Laboratory of molecular virology, Research Institute of Influenza, Ministry of Healthcare of the Russian Federation	Komissarova,K.S., Krivitskaya,V.Z., Sverlova,M.V., Petrova,E.R., Fadeev,A.V. and Komissarov,A.B.
EPI_ISL_2574964	Lab. of Molecular Virology, Research Institute of Influenza	Lab. of Molecular Virology, Research Institute of Influenza	Sintsova,K.S., Krivitskaya,V.Z., Sverlova,M.V., Petrova,E.R., Fadeev,A.V. and Komissarov,A.B.
EPI_ISL_2574967	Laboratory of molecular virology, Research Institute of Influenza, Ministry of Healthcare of the Russian Federation	Laboratory of molecular virology, Research Institute of Influenza, Ministry of Healthcare of the Russian Federation	Komissarova,K.S., Krivitskaya,V.Z., Sverlova,M.V., Petrova,E.R., Fadeev,A.V. and Komissarov,A.B.
EPI_ISL_2574970, EPI_ISL_2574972, EPI_ISL_2574973, EPI_ISL_2574974	Botany and Microbiology, College of Science, King Saud University, King Saud University	Botany and Microbiology, College of Science, King Saud University, King Saud University	Amer,H.M., Farrag,M.A., Hamad,M.E., Aziz,I.M. and Almajhdi,F.N.
EPI_ISL_2574992, EPI_ISL_2574993, EPI_ISL_2574994, EPI_ISL_2574995	Protein Research Chair, Department of Biochemistry, College of Science, King Saud University	Protein Research Chair, Department of Biochemistry, College of Science, King Saud University	Ahmed,A., Haider,M.S.H., Arshad,M., Baaboud,A.O., AlSenaidy,A.M., AlSaadi,M.M., Mobaireek,K.F., Sullender,W.M. and Parveen,S.
EPI_ISL_2574996	Epidemiology and Demography, KEMRI - Wellcome Trust Research Programme, Centre for Geographic Medicine Research	Epidemiology and Demography, KEMRI - Wellcome Trust Research Programme, Centre for Geographic Medicine Research	Oketch,J.W., Kamau,E., Otieno,J.R., Mwema,A., Lewa,C., Agoti,C.N. and Nokes,J.D.
EPI_ISL_2574997, EPI_ISL_2574998	Influenza and Other Respiratory Viruses Unit, National Center for Microbiology, Instituto de Salud Carlos III	Influenza and Other Respiratory Viruses Unit, National Center for Microbiology, Instituto de Salud Carlos III	Calderon,A., Pozo,F., Calvo,C., Garcia-Garcia,M., Gonzalez,M., Molinero,M. and Casas,I.
EPI_ISL_2575001, EPI_ISL_2575002, EPI_ISL_2575003, EPI_ISL_2575004, EPI_ISL_2575005, EPI_ISL_2575006, EPI_ISL_2575007, EPI_ISL_2575008, EPI_ISL_2575009, EPI_ISL_2575010, EPI_ISL_2575011, EPI_ISL_2575012, EPI_ISL_2575013, EPI_ISL_2575014, EPI_ISL_2575015, EPI_ISL_2575016, EPI_ISL_2575017, EPI_ISL_2575018, EPI_ISL_2575019, EPI_ISL_2575020, EPI_ISL_2575093, EPI_ISL_2575094, EPI_ISL_2575095, EPI_ISL_2575096, EPI_ISL_2575097, EPI_ISL_2575098, EPI_ISL_2575099, EPI_ISL_2575100			
see above	Microbiology, Hospital Universitari Vall d'Hebron	Microbiology, Hospital Universitari Vall d'Hebron	Gimferrer,L., Martin,Md.C., Pumarola,T. and Anton,A.

EPI_ISL_2575154, EPI_ISL_2575155, EPI_ISL_2575156, EPI_ISL_2575157, EPI_ISL_2575158	Pediatrics, Show-Chwan Memorial Hospital	Pediatrics, Show-Chwan Memorial Hospital	Lee,C.-Y.
EPI_ISL_2575159, EPI_ISL_2575160, EPI_ISL_2575161, EPI_ISL_2575162, EPI_ISL_2575163, EPI_ISL_2575164, EPI_ISL_2575165, EPI_ISL_2575166, EPI_ISL_2575167, EPI_ISL_2575168, EPI_ISL_2575169, EPI_ISL_2575170			
see above	Pediatrics, Mackay Memorial Hospital	Pediatrics, Mackay Memorial Hospital	Chi,H., Hsiao,K.-L., Weng,L.-C., Chiu,N.-C., Huang,L.-M., Chiu,Y.-Y., Liu,C.-P. and Liu,H.-F.
EPI_ISL_2575171, EPI_ISL_2575172, EPI_ISL_2575173, EPI_ISL_2575174, EPI_ISL_2575175, EPI_ISL_2575176	Pediatrics, Show-Chwan Memorial Hospital	Pediatrics, Show-Chwan Memorial Hospital	Lee,C.-Y.
EPI_ISL_2575183, EPI_ISL_2575184, EPI_ISL_2575185, EPI_ISL_2575196, EPI_ISL_2575197, EPI_ISL_2575198, EPI_ISL_2575199, EPI_ISL_2575200, EPI_ISL_2575201, EPI_ISL_2575202, EPI_ISL_2575203, EPI_ISL_2575204, EPI_ISL_2575205, EPI_ISL_2575206, EPI_ISL_2575207, EPI_ISL_2575208, EPI_ISL_2575209, EPI_ISL_2575210, EPI_ISL_2575211, EPI_ISL_2575222, EPI_ISL_2575223, EPI_ISL_2575224			
see above	Department of Pediatrics, Center of Excellence in Clinical Virology, Faculty of Medicine, Chulalongkorn University	Department of Pediatrics, Center of Excellence in Clinical Virology, Faculty of Medicine, Chulalongkorn University	Thongpan,I., Mauleekoonphairoj,J., Vichiwattana,P., Korkong,S., Vongpunsawad,S. and Poovorawan,Y.
EPI_ISL_2575225, EPI_ISL_2575226, EPI_ISL_2575227, EPI_ISL_2575228, EPI_ISL_2575229, EPI_ISL_2575230, EPI_ISL_2575231, EPI_ISL_2575232, EPI_ISL_2575233, EPI_ISL_2575234, EPI_ISL_2575236, EPI_ISL_2575237, EPI_ISL_2575238, EPI_ISL_2575239, EPI_ISL_2575241, EPI_ISL_2575243, EPI_ISL_2575244, EPI_ISL_2575247			
see above	Epidemiology and Immunogenetic of Viral Infections LR14SP02, University Hospital Sahloul	Epidemiology and Immunogenetic of Viral Infections LR14SP02, University Hospital Sahloul	Ataoui,I., Jerbi,A., BenHamida-Rebai,M., Ben Hadj Fredj,M., Fodha,I., Bennour,H., Hammouda,H., Khlifa,M., Brini,I., Boussoffara,R., Boussetta,K., Abroug,S. and Trabelsi,A.
EPI_ISL_2575361, EPI_ISL_2575362	Epidemiology and Demography, KEMRI - Wellcome Trust Research Programme, Centre for Geographic Medicine Research	Epidemiology and Demography, KEMRI - Wellcome Trust Research Programme, Centre for Geographic Medicine Research	Oketch,J.W., Kamau,E., Otieno,J.R., Mwema,A., Lewa,C., Agoti,C.N. and Nokes,J.D.
EPI_ISL_2575363, EPI_ISL_2575364	Epidemiology and Demography, KEMRI-Wellcome Trust Research Programme	Epidemiology and Demography, KEMRI-Wellcome Trust Research Programme	Kamau,E.M., Agoti,C.N., Owor,B., Lewa,C., Bett,A., Oketch,J., Cane,P.A. and Nokes,J.D.
EPI_ISL_2578662	Beijing Key Laboratory of Etiology of Viral Diseases in Children; Laboratory of Virology, Capital Institute of Pediatrics	Beijing Key Laboratory of Etiology of Viral Diseases in Children; Laboratory of Virology, Capital Institute of Pediatrics	Cui,G., Zhu,R., Deng,J., Zhao,L., Sun,Y., Wang,F. and Qian,Y.
EPI_ISL_2578668, EPI_ISL_2578673, EPI_ISL_2578674, EPI_ISL_2578675, EPI_ISL_2578676	Epidemiology and Demography Department, KEMRI-Wellcome Trust Research Programme	Epidemiology and Demography Department, KEMRI-Wellcome Trust Research Programme	Otieno,J.R., Kamau,E.M., Oketch,J.W., Ngoi,J.M., Agoti,C.N., Gichuki,A.M., Otieno,G.P., Ngama,M., Cane,P.A., Kellam,P., Cotten,M., Lemey,P. and Nokes,D.J.
EPI_ISL_2578679	Marie Bashir Institute for Infectious Diseases and Biosecurity & Sydney Medical School, The University of Sydney, Westmead Institute for Medical Research	Marie Bashir Institute for Infectious Diseases and Biosecurity & Sydney Medical School, The University of Sydney, Westmead Institute for Medical Research	Eden,J.-S., Kok,J., Dwyer,D.E., Fernandez,M., Carter,I. and Holmes,E.C.
EPI_ISL_2578690	Epidemiology and Demography Department, KEMRI-Wellcome Trust Research Programme	Epidemiology and Demography Department, KEMRI-Wellcome Trust Research Programme	Otieno,J.R., Kamau,E.M., Oketch,J.W., Ngoi,J.M., Agoti,C.N., Gichuki,A.M., Otieno,G.P., Ngama,M., Cane,P.A., Kellam,P., Cotten,M., Lemey,P. and Nokes,D.J.
EPI_ISL_2578691	Department of Pediatrics, Center of Excellence in Clinical Virology, Chulalongkorn	Department of Pediatrics, Center of Excellence in Clinical Virology, Chulalongkorn	Thongpan,I.
EPI_ISL_2578708, EPI_ISL_2578709, EPI_ISL_2578710, EPI_ISL_2578711	Epidemiology and Demography Department, KEMRI-Wellcome Trust Research Programme	Epidemiology and Demography Department, KEMRI-Wellcome Trust Research Programme	Otieno,J.R., Kamau,E.M., Oketch,J.W., Ngoi,J.M., Agoti,C.N., Gichuki,A.M., Otieno,G.P., Ngama,M., Cane,P.A., Kellam,P., Cotten,M., Lemey,P. and Nokes,D.J.
EPI_ISL_2578713, EPI_ISL_2578714	Center for Infectious Diseases, School of Public Health, University of Texas Health Science Center	Center for Infectious Diseases, School of Public Health, University of Texas Health Science Center	Bahl,J., Hixson,J., Kim,D.-K., Qiu,X., Piedra,P.A., Piedra,F.-A., Avadhanula,V. and Machado,A.A.
EPI_ISL_2578729, EPI_ISL_2578730, EPI_ISL_2578731, EPI_ISL_2578732, EPI_ISL_2578733	Epidemiology and Demography Department, KEMRI-Wellcome Trust Research Programme	Epidemiology and Demography Department, KEMRI-Wellcome Trust Research Programme	Otieno,J.R., Kamau,E.M., Oketch,J.W., Ngoi,J.M., Agoti,C.N., Gichuki,A.M., Otieno,G.P., Ngama,M., Cane,P.A., Kellam,P., Cotten,M., Lemey,P. and Nokes,D.J.
EPI_ISL_2578738, EPI_ISL_2578739	WHO WPRO Measles Regional Reference Lab, Key Laboratory of Medical Virology Ministry of Health, National Institute for Viral Disease Control and Prevention, China Center for Disease Control and Prevention	WHO WPRO Measles Regional Reference Lab, Key Laboratory of Medical Virology Ministry of Health, National Institute for Viral Disease Control and Prevention, China Center for Disease Control and Prevention	Song,J., Zhang,Y. and Xu,W.
EPI_ISL_2578741, EPI_ISL_2578742, EPI_ISL_2578743	Department of Clinical Laboratory, Fujian Provincial Hospital	Department of Clinical Laboratory, Fujian Provincial Hospital	Su,Y., Wu,Y., Tian,R. and Liang,G.
EPI_ISL_2578759	WHO WPRO Measles Regional Reference Lab, Key Laboratory of Medical Virology Ministry of Health, National Institute for Viral Disease Control and Prevention, China Center for Disease Control and Prevention	WHO WPRO Measles Regional Reference Lab, Key Laboratory of Medical Virology Ministry of Health, National Institute for Viral Disease Control and Prevention, China Center for Disease Control and Prevention	Song,J., Zhang,Y. and Xu,W.
EPI_ISL_2578772, EPI_ISL_2578773, EPI_ISL_2578774, EPI_ISL_2578775, EPI_ISL_2578776	Epidemiology and Demography Department, KEMRI-Wellcome Trust Research Programme	Epidemiology and Demography Department, KEMRI-Wellcome Trust Research Programme	Otieno,J.R., Kamau,E.M., Oketch,J.W., Ngoi,J.M., Agoti,C.N., Gichuki,A.M., Otieno,G.P., Ngama,M., Cane,P.A., Kellam,P., Cotten,M., Lemey,P. and Nokes,D.J.
EPI_ISL_2578779, EPI_ISL_2578780	Virology, Graduate School of Medicine, Tohoku University	Virology, Graduate School of Medicine, Tohoku University	Malasao,R., Furuse,Y., Okamoto,M., Dapat,C., Saito,M., Saito-Obata,M., Tamaki,R., Segubre-Mercado,E., Lupisan,S. and Oshitani,H.
EPI_ISL_2578781, EPI_ISL_2578782, EPI_ISL_2578783	Laboratory of molecular virology, Research Institute of Influenza, Ministry of Healthcare of the Russian Federation	Laboratory of molecular virology, Research Institute of Influenza, Ministry of Healthcare of the Russian Federation	Komissarova,K.S., Krivitskaya,V.Z., Sverlova,M.V., Petrova,E.R., Fadeev,A.V. and Komissarov,A.B.
EPI_ISL_2578784	Lab. of Molecular Virology, Research Institute of Influenza	Lab. of Molecular Virology, Research Institute of Influenza	Sintsova,K.S., Krivitskaya,V.Z., Sverlova,M.V., Petrova,E.R., Fadeev,A.V. and Komissarov,A.B.
EPI_ISL_2578785	Laboratory of molecular virology, Research Institute of Influenza, Ministry of Healthcare of the Russian Federation	Laboratory of molecular virology, Research Institute of Influenza, Ministry of Healthcare of the Russian Federation	Komissarova,K.S., Krivitskaya,V.Z., Sverlova,M.V., Petrova,E.R., Fadeev,A.V. and Komissarov,A.B.
EPI_ISL_2578786	Etiology and Epidemiology Department, Smorodintsev Research Institute of Influenza	Etiology and Epidemiology Department, Smorodintsev Research Institute of Influenza	Komissarova,K.S., Krivitskaya,V.Z., Sverlova,M.V., Petrova,E.R. and Ivanova,A.A.
EPI_ISL_2578800, EPI_ISL_2578801	Epidemiology and Demography Department, KEMRI-Wellcome Trust Research Programme	Epidemiology and Demography Department, KEMRI-Wellcome Trust Research Programme	Otieno,J.R., Kamau,E.M., Oketch,J.W., Ngoi,J.M., Agoti,C.N., Gichuki,A.M., Otieno,G.P., Ngama,M., Cane,P.A., Kellam,P., Cotten,M., Lemey,P. and Nokes,D.J.
EPI_ISL_2578802	Virology, Graduate School of Medicine, Tohoku University	Virology, Graduate School of Medicine, Tohoku University	Malasao,R., Furuse,Y., Okamoto,M., Dapat,C., Saito,M., Saito-Obata,M., Tamaki,R., Segubre-Mercado,E., Lupisan,S. and Oshitani,H.
EPI_ISL_2578804, EPI_ISL_2578805, EPI_ISL_2578806, EPI_ISL_2578807, EPI_ISL_2578808	Epidemiology and Demography Department, KEMRI-Wellcome Trust Research Programme	Epidemiology and Demography Department, KEMRI-Wellcome Trust Research Programme	Otieno,J.R., Kamau,E.M., Oketch,J.W., Ngoi,J.M., Agoti,C.N., Gichuki,A.M., Otieno,G.P., Ngama,M., Cane,P.A., Kellam,P., Cotten,M., Lemey,P. and Nokes,D.J.
EPI_ISL_2578810	Virology, Graduate School of Medicine, Tohoku University	Virology, Graduate School of Medicine, Tohoku University	Malasao,R., Furuse,Y., Okamoto,M., Dapat,C., Saito,M., Saito-Obata,M., Tamaki,R., Segubre-Mercado,E., Lupisan,S. and Oshitani,H.
EPI_ISL_2578814, EPI_ISL_2578815, EPI_ISL_2578818, EPI_ISL_2578819, EPI_ISL_2578820, EPI_ISL_2578821	Epidemiology and Demography Department, KEMRI-Wellcome Trust Research Programme	Epidemiology and Demography Department, KEMRI-Wellcome Trust Research Programme	Otieno,J.R., Kamau,E.M., Oketch,J.W., Ngoi,J.M., Agoti,C.N., Gichuki,A.M., Otieno,G.P., Ngama,M., Cane,P.A., Kellam,P., Cotten,M., Lemey,P. and Nokes,D.J.
EPI_ISL_2578822, EPI_ISL_2578823	Centre for Infectious Diseases Research, Diagnostics and laboratory Surveillance, National Institute for Public Health and the Environment	Centre for Infectious Diseases Research, Diagnostics and laboratory Surveillance, National Institute for Public Health and the Environment	Meijer,A. and Overduin,P.
EPI_ISL_2578824	Epidemiology and Demography Department, KEMRI-Wellcome Trust Research Programme	Epidemiology and Demography Department, KEMRI-Wellcome Trust Research Programme	Otieno,J.R., Kamau,E.M., Oketch,J.W., Ngoi,J.M., Agoti,C.N., Gichuki,A.M., Otieno,G.P., Ngama,M., Cane,P.A., Kellam,P., Cotten,M., Lemey,P. and Nokes,D.J.
EPI_ISL_2578839, EPI_ISL_2578840, EPI_ISL_2578841, EPI_ISL_2578842, EPI_ISL_2578843, EPI_ISL_2578844, EPI_ISL_2578845	Chinese Academy of Medical Sciences & Peking Union Medical College	Chinese Academy of Medical Sciences & Peking Union Medical College	Jia,B., Xiao,Y., Wang,Y., Chen,L., Zhang,J., Ren,L. and Wang,J.
EPI_ISL_2578851	Central Laboratory, Guangzhou Women and Children's Medical Center	Central Laboratory, Guangzhou Women and Children's Medical Center	Xie,J.H., Zhu,B., Zhong,J.Y., Chen,Y. and Zhang,Y.Y.
EPI_ISL_2578852, EPI_ISL_2578853, EPI_ISL_2578854, EPI_ISL_2578855, EPI_ISL_2578856, EPI_ISL_2578857, EPI_ISL_2578858, EPI_ISL_2578859, EPI_ISL_2578860, EPI_ISL_2578861, EPI_ISL_2578862, EPI_ISL_2578863, EPI_ISL_2578864, EPI_ISL_2578865, EPI_ISL_2578866, EPI_ISL_2578867, EPI_ISL_2578868, EPI_ISL_2578869			
see above	Laboratory of Virology, Capital Institute of Pediatrics	Laboratory of Virology, Capital Institute of Pediatrics	Cui,G., Zhu,R., Qian,Y., Deng,J., Zhao,L., Sun,Y. and Wang,F.
EPI_ISL_2578880, EPI_ISL_2578881, EPI_ISL_2578882, EPI_ISL_2578883, EPI_ISL_2578884, EPI_ISL_2578885, EPI_ISL_2578886, EPI_ISL_2578887, EPI_ISL_2578888, EPI_ISL_2578889, EPI_ISL_2578890			
see above	Department of Clinical Laboratory, Fujian Provincial Hospital	Department of Clinical Laboratory, Fujian Provincial Hospital	Su,Y., Wu,Y., Tian,R. and Liang,G.
EPI_ISL_2578891, EPI_ISL_2578908	Virology, Tropical Medicine Institute Pedro Kouri	Virology, Tropical Medicine Institute Pedro Kouri	Valdes,O., Corso,M., Pinon,A., Acosta,B., Savon,C., Gonzalez,G., Mune,M., Gonzalez,G., Hernandez,B., Echevarria,Y. and Oropesa,S.
EPI_ISL_2578912, EPI_ISL_2578913	Lab. of Molecular Virology, Research Institute of Influenza	Lab. of Molecular Virology, Research Institute of Influenza	Sintsova,K.S., Krivitskaya,V.Z., Sverlova,M.V., Petrova,E.R., Fadeev,A.V. and Komissarov,A.B.
EPI_ISL_2578918	Laboratory of molecular virology, Research Institute of Influenza, Ministry of Healthcare of the Russian Federation	Laboratory of molecular virology, Research Institute of Influenza, Ministry of Healthcare of the Russian Federation	Komissarova,K.S., Krivitskaya,V.Z., Sverlova,M.V., Petrova,E.R., Fadeev,A.V. and Komissarov,A.B.
EPI_ISL_2578921, EPI_ISL_2578923	Lab. of Molecular Virology, Research Institute of Influenza	Lab. of Molecular Virology, Research Institute of Influenza	Sintsova,K.S., Krivitskaya,V.Z., Sverlova,M.V., Petrova,E.R., Fadeev,A.V. and Komissarov,A.B.
EPI_ISL_2578931, EPI_ISL_2578932	Virology Department, National Center of Microbiology, Instituto de Salud Carlos III	Virology Department, National Center of Microbiology, Instituto de Salud Carlos III	Casas,I., Calderon,A., Pozo,F., Calvo,C., Garcia-Garcia,M., Gonzalez,M. and Molinero,M.
EPI_ISL_2578933, EPI_ISL_2578934, EPI_ISL_2578935, EPI_ISL_2578936, EPI_ISL_2578937, EPI_ISL_2578938, EPI_ISL_2578939, EPI_ISL_2578940, EPI_ISL_2578941, EPI_ISL_2578942, EPI_ISL_2578943, EPI_ISL_2578944, EPI_ISL_2578945, EPI_ISL_2578946, EPI_ISL_2578947, EPI_ISL_2578948, EPI_ISL_2578949, EPI_ISL_2578950, EPI_ISL_2578951			
see above	Influenza and Other Respiratory Viruses Unit, National Center for Microbiology, Instituto de Salud Carlos III	Influenza and Other Respiratory Viruses Unit, National Center for Microbiology, Instituto de Salud Carlos III	Calderon,A., Pozo,F., Calvo,C., Garcia-Garcia,M., Gonzalez,M., Molinero,M. and Casas,I.
EPI_ISL_2578953, EPI_ISL_2578954	Virology, Tropical Medicine Institute Pedro Kouri	Virology, Tropical Medicine Institute Pedro Kouri	Valdes,O., Corso,M., Pinon,A., Acosta,B., Savon,C., Gonzalez,G., Mune,M., Gonzalez,G., Hernandez,B., Echevarria,Y. and Oropesa,S.
EPI_ISL_2578955	Etiology and Epidemiology Department, Smorodintsev Research Institute of Influenza	Etiology and Epidemiology Department, Smorodintsev Research Institute of Influenza	Komissarova,K.S., Krivitskaya,V.Z., Sverlova,M.V., Petrova,E.R. and Ivanova,A.A.
EPI_ISL_2578956	Lab. of Molecular Virology, Research Institute of Influenza	Lab. of Molecular Virology, Research Institute of Influenza	Sintsova,K.S., Krivitskaya,V.Z., Sverlova,M.V., Petrova,E.R., Fadeev,A.V. and Komissarov,A.B.
EPI_ISL_2578993	WHO WPRO Measles Regional Reference Lab, Key Laboratory of Medical Virology Ministry of Health, National Institute for Viral Disease Control and Prevention, China Center for Disease Control and Prevention	WHO WPRO Measles Regional Reference Lab, Key Laboratory of Medical Virology Ministry of Health, National Institute for Viral Disease Control and Prevention, China Center for Disease Control and Prevention	Song,J., Zhang,Y. and Xu,W.
EPI_ISL_2579001, EPI_ISL_2579002, EPI_ISL_2579003,	Department of Clinical Laboratory, Fujian Provincial Hospital	Department of Clinical Laboratory, Fujian Provincial Hospital	Su,Y., Wu,Y., Tian,R. and Liang,G.

EPI_ISL_2579004, EPI_ISL_2579005, EPI_ISL_2579006, EPI_ISL_2579007, EPI_ISL_2579008, EPI_ISL_2579009, EPI_ISL_2579010			
EPI_ISL_2579012	WHO WPRO Measles Regional Reference Lab, Key Laboratory of Medical Virology Ministry of Health, National Institute for Viral Disease Control and Prevention, China Center for Disease Control and Prevention	WHO WPRO Measles Regional Reference Lab, Key Laboratory of Medical Virology Ministry of Health, National Institute for Viral Disease Control and Prevention, China Center for Disease Control and Prevention	Song,J., Zhang,Y. and Xu,W.
EPI_ISL_2579017, EPI_ISL_2579018	Lab. of Molecular Virology, Research Institute of Influenza	Lab. of Molecular Virology, Research Institute of Influenza	Sintsova,K.S., Krivitskaya,V.Z., Sverlova,M.V., Petrova,E.R., Fadeev,A.V. and Komissarov,A.B.
EPI_ISL_2579019	Etiology and Epidemiology Department, Smorodintsev Research Institute of Influenza	Etiology and Epidemiology Department, Smorodintsev Research Institute of Influenza	Komissarova,K.S., Krivitskaya,V.Z., Sverlova,M.V., Petrova,E.R. and Ivanova,A.A.
EPI_ISL_2579020	Lab. of Molecular Virology, Research Institute of Influenza	Lab. of Molecular Virology, Research Institute of Influenza	Sintsova,K.S., Krivitskaya,V.Z., Sverlova,M.V., Petrova,E.R., Fadeev,A.V. and Komissarov,A.B.
EPI_ISL_2579022, EPI_ISL_2579023, EPI_ISL_2579024, EPI_ISL_2579025	Centre for Infectious Diseases Research, Diagnostics and laboratory Surveillance, National Institute for Public Health and the Environment	Centre for Infectious Diseases Research, Diagnostics and laboratory Surveillance, National Institute for Public Health and the Environment	Meijer,A. and Overduin,P.
EPI_ISL_2579039	Centre for Research And Knowledge Transfer in Biotechnology, University of Zagreb	Centre for Research And Knowledge Transfer in Biotechnology, University of Zagreb	Jagusic,M., Slovic,A., Santak,M., Kosutic-Gulija,T., Ivancic-Jelecki,J. and Forcic,D.
EPI_ISL_2579040	Depto Microbiologia, Instituto de Ciencias Biomedicas, Universidade de Sao Paulo	Depto Microbiologia, Instituto de Ciencias Biomedicas, Universidade de Sao Paulo	Moura,F.E.A., Thomazelli,L.M., Campelo,F.S., Delfraro,A., Arbiza,J. and Durigon,E.L.
EPI_ISL_2579054, EPI_ISL_2579055	Pushpagiri Research Centre, Pushpagiri Institute of Medical Science and Research Centre, Pushpagir Medical College Hospital	Pushpagiri Research Centre, Pushpagiri Institute of Medical Science and Research Centre, Pushpagir Medical College Hospital	George,S., Jagan,O.A., Jose,S., John,N.S., Bai,S., Chandy,S. and Manoharan,A.
EPI_ISL_2579068, EPI_ISL_2579069, EPI_ISL_2579070, EPI_ISL_2579071, EPI_ISL_2579072, EPI_ISL_2579073, EPI_ISL_2579074, EPI_ISL_2579075, EPI_ISL_2579076, EPI_ISL_2579077, EPI_ISL_2579078, EPI_ISL_2579079, EPI_ISL_2579080, EPI_ISL_2579081, EPI_ISL_2579082, EPI_ISL_2579083, EPI_ISL_2579084, EPI_ISL_2579085, EPI_ISL_2579086, EPI_ISL_2579087, EPI_ISL_2579088, EPI_ISL_2579089, EPI_ISL_2579090, EPI_ISL_2579091, EPI_ISL_2579092, EPI_ISL_2579093, EPI_ISL_2579094, EPI_ISL_2579095, EPI_ISL_2579096, EPI_ISL_2579097, EPI_ISL_2579098, EPI_ISL_2579099, EPI_ISL_2579100, EPI_ISL_2579101, EPI_ISL_2579102, EPI_ISL_2579103, EPI_ISL_2579104, EPI_ISL_2579105, EPI_ISL_2579106, EPI_ISL_2579107, EPI_ISL_2579108, EPI_ISL_2579109, EPI_ISL_2579110, EPI_ISL_2579111, EPI_ISL_2579112, EPI_ISL_2579113, EPI_ISL_2579114, EPI_ISL_2579115, EPI_ISL_2579116, EPI_ISL_2579117, EPI_ISL_2579118, EPI_ISL_2579119, EPI_ISL_2579120, EPI_ISL_2579121, EPI_ISL_2579122, EPI_ISL_2579123, EPI_ISL_2579124, EPI_ISL_2579125, EPI_ISL_2579126, EPI_ISL_2579127, EPI_ISL_2579128, EPI_ISL_2579129, EPI_ISL_2579130, EPI_ISL_2579131, EPI_ISL_2579132, EPI_ISL_2579133, EPI_ISL_2579134, EPI_ISL_2579135, EPI_ISL_2579136, EPI_ISL_2579137, EPI_ISL_2579138, EPI_ISL_2579139, EPI_ISL_2579140, EPI_ISL_2579141, EPI_ISL_2579142, EPI_ISL_2579143, EPI_ISL_2579144, EPI_ISL_2579145, EPI_ISL_2579146, EPI_ISL_2579147, EPI_ISL_2579149			
see above	Department of Epidemiology and Demography, Kenya Medical Research Institute (KEMRI) - Wellcome Trust Research Programme	Department of Epidemiology and Demography, Kenya Medical Research Institute (KEMRI) - Wellcome Trust Research Programme	Otieno,J.R., Kamau,E.M., Agoti,C.N., Lewa,C., Bett,A., Ngama,M., Cane,P.A. and Nokes,J.D.
EPI_ISL_2579150	Epidemiology and Demography, KEMRI - Wellcome Trust Research Programme, Centre for Geographic Medicine Research	Epidemiology and Demography, KEMRI - Wellcome Trust Research Programme, Centre for Geographic Medicine Research	Oketch,J.W., Kamau,E., Otieno,J.R., Mwema,A., Lewa,C., Agoti,C.N. and Nokes,J.D.
EPI_ISL_2579151, EPI_ISL_2579152, EPI_ISL_2579153	Botany and Microbiology, College of Science, King Saud University, King Saud University	Botany and Microbiology, College of Science, King Saud University, King Saud University	Amer,H.M., Farrag,M.A., Hamad,M.E., Aziz,I.M. and Almajhdi,F.N.
EPI_ISL_2579155, EPI_ISL_2579156, EPI_ISL_2579157, EPI_ISL_2579158, EPI_ISL_2579159, EPI_ISL_2579160, EPI_ISL_2579161, EPI_ISL_2579163, EPI_ISL_2579164, EPI_ISL_2579165, EPI_ISL_2579166, EPI_ISL_2579167			
see above	Epidemiology and Demography, KEMRI - Wellcome Trust Research Programme, Centre for Geographic Medicine Research	Epidemiology and Demography, KEMRI - Wellcome Trust Research Programme, Centre for Geographic Medicine Research	Oketch,J.W., Kamau,E., Otieno,J.R., Mwema,A., Lewa,C., Agoti,C.N. and Nokes,J.D.
EPI_ISL_2579168, EPI_ISL_2579169, EPI_ISL_2579170, EPI_ISL_2579171	Virology Laboratory, Dr. Ricardo Gutierrez Children Hospital	Virology Laboratory, Dr. Ricardo Gutierrez Children Hospital	Rojo,G.L., Goya,S., Orellana,M., Sancilio,A., Rodriguez Perez,A., Montali,C., Garcia,C., Sosa,L., Musto,A., Hamilton,G., Alvarez,D., Castello,A. and Viegas,M.
EPI_ISL_2579172	Pediatrics, Show-Chwan Memorial Hospital	Pediatrics, Show-Chwan Memorial Hospital	Lee,C.-Y.
EPI_ISL_2579179, EPI_ISL_2579180, EPI_ISL_2579181, EPI_ISL_2579182, EPI_ISL_2579183, EPI_ISL_2579184, EPI_ISL_2579185, EPI_ISL_2579186, EPI_ISL_2579187, EPI_ISL_2579188, EPI_ISL_2579189, EPI_ISL_2579190, EPI_ISL_2579194, EPI_ISL_2579195, EPI_ISL_2579196, EPI_ISL_2579197, EPI_ISL_2579198, EPI_ISL_2579199, EPI_ISL_2579200, EPI_ISL_2579201			
see above	Pediatrics, Mackay Memorial Hospital	Pediatrics, Mackay Memorial Hospital	Chi,H., Hsiao,K.-L., Weng,L.-C., Chiu,N.-C., Huang,L.-M., Chiu,Y.-Y., Liu,C.-P. and Liu,H.-F.
EPI_ISL_2579204, EPI_ISL_2579205, EPI_ISL_2579206, EPI_ISL_2579207, EPI_ISL_2579208, EPI_ISL_2579209, EPI_ISL_2579210, EPI_ISL_2579211, EPI_ISL_2579212, EPI_ISL_2579213, EPI_ISL_2579214, EPI_ISL_2579215, EPI_ISL_2579216			
see above	Pediatrics, Show-Chwan Memorial Hospital	Pediatrics, Show-Chwan Memorial Hospital	Lee,C.-Y.
EPI_ISL_2579218, EPI_ISL_2579219	Virology Laboratory, Dr. Ricardo Gutierrez Children Hospital	Virology Laboratory, Dr. Ricardo Gutierrez Children Hospital	Rojo,G.L., Goya,S., Orellana,M., Sancilio,A., Rodriguez Perez,A., Montali,C., Garcia,C., Sosa,L., Musto,A., Hamilton,G., Alvarez,D., Castello,A. and Viegas,M.
EPI_ISL_2579220	Epidemiology and Demography, KEMRI - Wellcome Trust Research Programme, Centre for Geographic Medicine Research	Epidemiology and Demography, KEMRI - Wellcome Trust Research Programme, Centre for Geographic Medicine Research	Oketch,J.W., Kamau,E., Otieno,J.R., Mwema,A., Lewa,C., Agoti,C.N. and Nokes,J.D.
EPI_ISL_2579222, EPI_ISL_2579223, EPI_ISL_2579224, EPI_ISL_2579225, EPI_ISL_2579226, EPI_ISL_2579227, EPI_ISL_2579228, EPI_ISL_2579229, EPI_ISL_2579230, EPI_ISL_2579231, EPI_ISL_2579232, EPI_ISL_2579233, EPI_ISL_2579234, EPI_ISL_2579235, EPI_ISL_2579236			
see above	Department of Pediatrics, Center of Excellence in Clinical Virology, Faculty of Medicine, Chulalongkorn University	Department of Pediatrics, Center of Excellence in Clinical Virology, Faculty of Medicine, Chulalongkorn University	Thongpan,I., Mauleekoonphairoj,J., Vichiwattana,P., Korkong,S., Vongpunsawad,S. and Poovorawan,Y.
EPI_ISL_2579237	Epidemiology and Demography, KEMRI - Wellcome Trust Research Programme, Centre for Geographic Medicine Research	Epidemiology and Demography, KEMRI - Wellcome Trust Research Programme, Centre for Geographic Medicine Research	Oketch,J.W., Kamau,E., Otieno,J.R., Mwema,A., Lewa,C., Agoti,C.N. and Nokes,J.D.
EPI_ISL_2579249, EPI_ISL_2579250, EPI_ISL_2579251, EPI_ISL_2579252, EPI_ISL_2579253, EPI_ISL_2579254, EPI_ISL_2579255, EPI_ISL_2579256	Department of Clinical Laboratory, Fujian Provincial Hospital	Department of Clinical Laboratory, Fujian Provincial Hospital	Su,Y., Wu,Y., Tian,R. and Liang,G.
EPI_ISL_2579263	Centre for Infectious Diseases Research, Diagnostics and laboratory Surveillance, National Institute for Public Health and the Environment	Centre for Infectious Diseases Research, Diagnostics and laboratory Surveillance, National Institute for Public Health and the Environment	Meijer,A. and Overduin,P.
EPI_ISL_2579281, EPI_ISL_2579283, EPI_ISL_2579284, EPI_ISL_2579285, EPI_ISL_2579286, EPI_ISL_2579287, EPI_ISL_2579288, EPI_ISL_2579290	Chinese Academy of Medical Sciences & Peking Union Medical College	Chinese Academy of Medical Sciences & Peking Union Medical College	Jia,B., Xiao,Y., Wang,Y., Chen,L., Zhang,J., Ren,L. and Wang,J.
EPI_ISL_2579300, EPI_ISL_2579301, EPI_ISL_2579302, EPI_ISL_2579303, EPI_ISL_2579304, EPI_ISL_2579305, EPI_ISL_2579306, EPI_ISL_2579307	Laboratory of Virology, Capital Institute of Pediatrics	Laboratory of Virology, Capital Institute of Pediatrics	Cui,G., Zhu,R., Qian,Y., Deng,J., Zhao,L., Sun,Y. and Wang,F.
EPI_ISL_2579313, EPI_ISL_2579314, EPI_ISL_2579315, EPI_ISL_2579316, EPI_ISL_2579317, EPI_ISL_2579318, EPI_ISL_2579319, EPI_ISL_2579320, EPI_ISL_2579321, EPI_ISL_2579322, EPI_ISL_2579323			
see above	Department of Clinical Laboratory, Fujian Provincial Hospital	Department of Clinical Laboratory, Fujian Provincial Hospital	Su,Y., Wu,Y., Tian,R. and Liang,G.
EPI_ISL_2579331, EPI_ISL_2579332	Lab. of Molecular Virology, Research Institute of Influenza	Lab. of Molecular Virology, Research Institute of Influenza	Sintsova,K.S., Krivitskaya,V.Z., Sverlova,M.V., Petrova,E.R., Fadeev,A.V. and Komissarov,A.B.
EPI_ISL_2579333	Laboratory of molecular virology, Research Institute of Influenza, Ministry of Healthcare of the Russian Federation	Laboratory of molecular virology, Research Institute of Influenza, Ministry of Healthcare of the Russian Federation	Komissarova,K.S., Krivitskaya,V.Z., Sverlova,M.V., Petrova,E.R., Fadeev,A.V. and Komissarov,A.B.
EPI_ISL_2579337, EPI_ISL_2579338, EPI_ISL_2579341	Lab. of Molecular Virology, Research Institute of Influenza	Lab. of Molecular Virology, Research Institute of Influenza	Sintsova,K.S., Krivitskaya,V.Z., Sverlova,M.V., Petrova,E.R., Fadeev,A.V. and Komissarov,A.B.
EPI_ISL_2579351	Virology Department, National Center of Microbiology, Instituto de Salud Carlos III	Virology Department, National Center of Microbiology, Instituto de Salud Carlos III	Casas,I., Calderon,A., Pozo,F., Calvo,C., Garcia-Garcia,M., Gonzalez,M. and Molinero,M.
EPI_ISL_2579352, EPI_ISL_2579353, EPI_ISL_2579354, EPI_ISL_2579355, EPI_ISL_2579356, EPI_ISL_2579357, EPI_ISL_2579358, EPI_ISL_2579359, EPI_ISL_2579360, EPI_ISL_2579361, EPI_ISL_2579362, EPI_ISL_2579363, EPI_ISL_2579364, EPI_ISL_2579365, EPI_ISL_2579366			
see above	Influenza and Other Respiratory Viruses Unit, National Center for Microbiology, Instituto de Salud Carlos III	Influenza and Other Respiratory Viruses Unit, National Center for Microbiology, Instituto de Salud Carlos III	Calderon,A., Pozo,F., Calvo,C., Garcia-Garcia,M., Gonzalez,M., Molinero,M. and Casas,I.
EPI_ISL_2579367	Virology, University of Ibadan, College of Medicine (UCH)	Virology, University of Ibadan, College of Medicine (UCH)	Ogunsemowo,O.O., Odaibo,G.N. and Olaleye,D.O.
EPI_ISL_2579368	Etiology and Epidemiology Department, Smorodintsev Research Institute of Influenza	Etiology and Epidemiology Department, Smorodintsev Research Institute of Influenza	Komissarova,K.S., Krivitskaya,V.Z., Sverlova,M.V., Petrova,E.R. and Ivanova,A.A.
EPI_ISL_2579376, EPI_ISL_2579380	Depto Microbiologia, Instituto de Ciencias Biomedicas, Universidade de Sao Paulo	Depto Microbiologia, Instituto de Ciencias Biomedicas, Universidade de Sao Paulo	Moura,F.E.A., Thomazelli,L.M., Campelo,F.S., Delfraro,A., Arbiza,J. and Durigon,E.L.
EPI_ISL_2579384	Division of Public Health Research, Gyeonggi Province institute of Health and Environment	Division of Public Health Research, Gyeonggi Province institute of Health and Environment	Park,E., Park,P., Huh,J., Yun,H., Lee,H., Yoon,M., Lee,S. and Ko,G.
EPI_ISL_2579387	Depto Microbiologia, Instituto de Ciencias Biomedicas, Universidade de Sao Paulo	Depto Microbiologia, Instituto de Ciencias Biomedicas, Universidade de Sao Paulo	Moura,F.E.A., Thomazelli,L.M., Campelo,F.S., Delfraro,A., Arbiza,J. and Durigon,E.L.
EPI_ISL_2579388	Virology, University of Ibadan, College of Medicine (UCH)	Virology, University of Ibadan, College of Medicine (UCH)	Ogunsemowo,O.O., Odaibo,G.N. and Olaleye,D.O.
EPI_ISL_2579392	Depto Microbiologia, Instituto de Ciencias Biomedicas, Universidade de Sao Paulo	Depto Microbiologia, Instituto de Ciencias Biomedicas, Universidade de Sao Paulo	Moura,F.E.A., Thomazelli,L.M., Campelo,F.S., Delfraro,A., Arbiza,J. and Durigon,E.L.
EPI_ISL_2579393, EPI_ISL_2579394, EPI_ISL_2579395, EPI_ISL_2579396, EPI_ISL_2579397, EPI_ISL_2579398	Molecular Medicine Laboratory of Virology, Sapienza University	Molecular Medicine Laboratory of Virology, Sapienza University	Pierangeli,A.
EPI_ISL_2579413, EPI_ISL_2579415	Depto Microbiologia, Instituto de Ciencias Biomedicas, Universidade de Sao Paulo	Depto Microbiologia, Instituto de Ciencias Biomedicas, Universidade de Sao Paulo	Moura,F.E.A., Thomazelli,L.M., Campelo,F.S., Delfraro,A., Arbiza,J. and Durigon,E.L.
EPI_ISL_2579447, EPI_ISL_2579448	University of Wuerzburg, Institute of Virology and Immunobiology	University of Wuerzburg, Institute of Virology and Immunobiology	Prifert,C., Hofmann,D. and Weissbrich,B.
EPI_ISL_2579483	WHO WPRO Measles Regional Reference Lab, Key Laboratory of Medical Virology Ministry of Health, National Institute for Viral Disease Control and Prevention, China Center for Disease Control and Prevention	WHO WPRO Measles Regional Reference Lab, Key Laboratory of Medical Virology Ministry of Health, National Institute for Viral Disease Control and Prevention, China Center for Disease Control and Prevention	Zhang,Y., Song,J. and Xu,W.

EPI_ISL_2579484, EPI_ISL_2579485	University of Wuerzburg, Institute of Virology and Immunobiology	University of Wuerzburg, Institute of Virology and Immunobiology	Prifert,C., Hofmann,D. and Weissbrich,B.
EPI_ISL_2579494	Centre for Interdisciplinary Research in Basic Science	Centre for Interdisciplinary Research in Basic Science	Haider,M.S.H., Naqvi,I.H., Broor,S., Ahmed,A. and Parveen,S.
EPI_ISL_2579495	Virology, School of Public Health, Tehran University of Medical Sciences	Virology, School of Public Health, Tehran University of Medical Sciences	Yavarian,J., Faghihloo,E. and Mokhtari Azad,T.
EPI_ISL_2579777, EPI_ISL_2579778, EPI_ISL_2579779, EPI_ISL_2579780, EPI_ISL_2579781, EPI_ISL_2579782, EPI_ISL_2579783, EPI_ISL_2579784, EPI_ISL_2579785, EPI_ISL_2579786, EPI_ISL_2579788, EPI_ISL_2579789, EPI_ISL_2579790, EPI_ISL_2579798, EPI_ISL_2579813			
see above	Centre for Research And Knowledge Transfer in Biotechnology, University of Zagreb	Centre for Research And Knowledge Transfer in Biotechnology, University of Zagreb	Ivancic-Jelecki,J., Slovic,A., Ljubin-Sternak,S., Mlinaric-Galinovic,G. and Forcic,D.
EPI_ISL_2579816	Centre for Research And Knowledge Transfer in Biotechnology, University of Zagreb	Centre for Research And Knowledge Transfer in Biotechnology, University of Zagreb	Jagusic,M., Slovic,A., Santak,M., Kosutic-Gulija,T., Ivancic-Jelecki,J. and Forcic,D.
EPI_ISL_2579818, EPI_ISL_2579822, EPI_ISL_2579824, EPI_ISL_2579825, EPI_ISL_2579826, EPI_ISL_2579827, EPI_ISL_2579828, EPI_ISL_2579829, EPI_ISL_2579830, EPI_ISL_2579831, EPI_ISL_2579832, EPI_ISL_2579833, EPI_ISL_2579834, EPI_ISL_2579835, EPI_ISL_2579836, EPI_ISL_2579837, EPI_ISL_2579838, EPI_ISL_2579840, EPI_ISL_2579841, EPI_ISL_2579842, EPI_ISL_2579843, EPI_ISL_2579844			
see above	Centre for Research And Knowledge Transfer in Biotechnology, University of Zagreb	Centre for Research And Knowledge Transfer in Biotechnology, University of Zagreb	Ivancic-Jelecki,J., Slovic,A., Ljubin-Sternak,S., Mlinaric-Galinovic,G. and Forcic,D.
EPI_ISL_2579849, EPI_ISL_2579850, EPI_ISL_2579851, EPI_ISL_2579852, EPI_ISL_2579853, EPI_ISL_2579854, EPI_ISL_2579855, EPI_ISL_2579856	Centre for Infectious Diseases Research, Diagnostics and laboratory Surveillance, National Institute for Public Health and the Environment	Centre for Infectious Diseases Research, Diagnostics and laboratory Surveillance, National Institute for Public Health and the Environment	Meijer,A. and Overduin,P.
EPI_ISL_2579858, EPI_ISL_2579859, EPI_ISL_2579860	Center for Infectious Diseases, School of Public Health, University of Texas Health Science Center	Center for Infectious Diseases, School of Public Health, University of Texas Health Science Center	Bahl,J., Hixson,J., Kim,D.-K., Qiu,X., Piedra,P.A., Piedra,F.-A., Avadhanula,V. and Machado,A.A.
EPI_ISL_2579900	J. Craig Venter Institute	J. Craig Venter Institute	Shrivastava,S., Halpin,R.A., Puri,V., Fedorova,N.B., Stockwell,T., Amedeo,P., Katzel,D., Schobel,S., Pickett,B.E., Moore,M., Chappell,J., Larkin,E., Wentworth,D.E., Anderson,L.J. and Hartert,T.
EPI_ISL_2579923, EPI_ISL_2579925, EPI_ISL_2579927, EPI_ISL_2579929, EPI_ISL_2579932	Virology, Tohoku University Graduate School of Medicine	Virology, Tohoku University Graduate School of Medicine	Malasao,R., Okamoto,M., Chaimongkol,N., Imamura,T., Tohma,K., Dapat,I., Dapat,C., Suzuki,A., Saito,M., Saito,M., Tamaki,R., Segubre-Mercado,E., Igoy,M.A.U., Lupisan,S., Olveda,R. and Oshitani,H.
EPI_ISL_2580055, EPI_ISL_2580057, EPI_ISL_2580058	Department of Respiratory Medicine, Children's Hospital of Chongqing Medical University	Department of Respiratory Medicine, Children's Hospital of Chongqing Medical University	Ren,L. and Liu,E.
EPI_ISL_2580060, EPI_ISL_2580062	Akinobu Hibino Niigata University, International Health, Public Health	Akinobu Hibino Niigata University, International Health, Public Health	Hibino,A., Saito,R., Shoubugawa,Y. and Tomimoto,K.
EPI_ISL_2580064	Akinobu Hibino Niigata University, International Health, Public Health	Akinobu Hibino Niigata University, International Health, Public Health	Hibino,A., Saito,R., Shoubugawa,Y. and Saito,T.
EPI_ISL_2580066, EPI_ISL_2580068, EPI_ISL_2580070	Akinobu Hibino Niigata University, International Health, Public Health	Akinobu Hibino Niigata University, International Health, Public Health	Hibino,A., Saito,R., Shoubugawa,Y. and Sano,Y.
EPI_ISL_2580075	Depto Microbiologia, Instituto de Ciencias Biomedicas, Universidade de Sao Paulo	Depto Microbiologia, Instituto de Ciencias Biomedicas, Universidade de Sao Paulo	Moura,F.E.A., Thomazelli,L.M., Campelo,F.S., Delfraro,A., Arbiza,J. and Durigon,E.L.
EPI_ISL_2580089	Depto Microbiologia, Instituto de Ciencias Biomedicas, Universidade de Sao Paulo	Depto Microbiologia, Instituto de Ciencias Biomedicas, Universidade de Sao Paulo	Thomazelli,L.M., Oliveira,D.B.L., Colmanetti,T., Vieira,S.E., Paulis,M., Ferronato,A.E., Martinez,M.B. and Durigon,E.L.
EPI_ISL_2580165, EPI_ISL_2580170, EPI_ISL_2580178	Chinese Academy of Medical Sciences & Peking Union Medical College	Chinese Academy of Medical Sciences & Peking Union Medical College	Jia,B., Xiao,Y., Wang,Y., Chen,L., Zhang,J., Ren,L. and Wang,J.
EPI_ISL_2580223, EPI_ISL_2580225, EPI_ISL_2580227, EPI_ISL_2580228	WHO WPRO Measles Regional Reference Lab, Key Laboratory of Medical Virology Ministry of Health, National Institute for Viral Disease Control and Prevention, China Center for Disease Control and Prevention	WHO WPRO Measles Regional Reference Lab, Key Laboratory of Medical Virology Ministry of Health, National Institute for Viral Disease Control and Prevention, China Center for Disease Control and Prevention	Zhang,Y., Song,J. and Xu,W.
EPI_ISL_2580233	Department of Public Health Laboratory Sciences, West China School of Public Health, Sichuan University	Department of Public Health Laboratory Sciences, West China School of Public Health, Sichuan University	Hu,W.P. and Pei,F.X.
EPI_ISL_2580266, EPI_ISL_2580268, EPI_ISL_2580269	WHO WPRO Measles Regional Reference Lab, Key Laboratory of Medical Virology Ministry of Health, National Institute for Viral Disease Control and Prevention, China Center for Disease Control and Prevention	WHO WPRO Measles Regional Reference Lab, Key Laboratory of Medical Virology Ministry of Health, National Institute for Viral Disease Control and Prevention, China Center for Disease Control and Prevention	Zhang,Y., Song,J. and Xu,W.
EPI_ISL_2580275	University of Wuerzburg, Institute of Virology and Immunobiology	University of Wuerzburg, Institute of Virology and Immunobiology	Prifert,C., Hofmann,D. and Weissbrich,B.
EPI_ISL_2580387, EPI_ISL_2580389, EPI_ISL_2580391, EPI_ISL_2580392, EPI_ISL_2580394	Akinobu Hibino Niigata University, International Health, Public Health	Akinobu Hibino Niigata University, International Health, Public Health	Hibino,A., Saito,R., Shoubugawa,Y. and Tomimoto,K.
EPI_ISL_2580399, EPI_ISL_2580400, EPI_ISL_2580401	Akinobu Hibino Niigata University, International Health, Public Health	Akinobu Hibino Niigata University, International Health, Public Health	Hibino,A., Saito,R., Shoubugawa,Y. and Saito,T.
EPI_ISL_2580402, EPI_ISL_2580404, EPI_ISL_2580408, EPI_ISL_2580409, EPI_ISL_2580411, EPI_ISL_2580425	Eiko Hirano Fukui Prefectural Institute of Public Health and Environmental Science	Eiko Hirano Fukui Prefectural Institute of Public Health and Environmental Science	Hirano,E. and Kobayashi,M.
EPI_ISL_2580450	Akinobu Hibino Niigata University, International Health, Public Health	Akinobu Hibino Niigata University, International Health, Public Health	Hibino,A., Saito,R., Shoubugawa,Y. and Sugimura,T.
EPI_ISL_2580466, EPI_ISL_2580468, EPI_ISL_2580470, EPI_ISL_2580472, EPI_ISL_2580474, EPI_ISL_2580475, EPI_ISL_2580477, EPI_ISL_2580479, EPI_ISL_2580481, EPI_ISL_2580482, EPI_ISL_2580484, EPI_ISL_2580487, EPI_ISL_2580488			
see above	Akinobu Hibino Niigata University, International Health, Public Health	Akinobu Hibino Niigata University, International Health, Public Health	Hibino,A., Saito,R., Shoubugawa,Y. and Takei,T.
EPI_ISL_2580497, EPI_ISL_2580499, EPI_ISL_2580501, EPI_ISL_2580502	Akinobu Hibino Niigata University, International Health, Public Health	Akinobu Hibino Niigata University, International Health, Public Health	Hibino,A., Saito,R., Shoubugawa,Y. and Taniguchi,K.
EPI_ISL_2580565, EPI_ISL_2580567, EPI_ISL_2580570, EPI_ISL_2580571, EPI_ISL_2580573, EPI_ISL_2580575, EPI_ISL_2580578, EPI_ISL_2580580, EPI_ISL_2580582, EPI_ISL_2580583, EPI_ISL_2580585, EPI_ISL_2580587, EPI_ISL_2580589, EPI_ISL_2580591, EPI_ISL_2580593, EPI_ISL_2580595, EPI_ISL_2580597, EPI_ISL_2580599, EPI_ISL_2580601, EPI_ISL_2580603, EPI_ISL_2580605, EPI_ISL_2580607, EPI_ISL_2580609, EPI_ISL_2580612, EPI_ISL_2580613, EPI_ISL_2580614, EPI_ISL_2580616			
see above	Akinobu Hibino Niigata University, International Health, Public Health	Akinobu Hibino Niigata University, International Health, Public Health	Hibino,A., Saito,R., Shoubugawa,Y. and Sano,Y.
EPI_ISL_2580617, EPI_ISL_2580619, EPI_ISL_2580621, EPI_ISL_2580623, EPI_ISL_2580625, EPI_ISL_2580627, EPI_ISL_2580629, EPI_ISL_2580631, EPI_ISL_2580633, EPI_ISL_2580636	Akinobu Hibino Niigata University, International Health, Public Health	Akinobu Hibino Niigata University, International Health, Public Health	Hibino,A., Saito,R., Shoubugawa,Y. and Nishimura,T.
EPI_ISL_2580637	Akinobu Hibino Niigata University, International Health, Public Health	Akinobu Hibino Niigata University, International Health, Public Health	Hibino,A., Saito,R., Shoubugawa,Y. and Saito,N.
EPI_ISL_2580639, EPI_ISL_2580641, EPI_ISL_2580643, EPI_ISL_2580645	Akinobu Hibino Niigata University, International Health, Public Health	Akinobu Hibino Niigata University, International Health, Public Health	Hibino,A., Saito,R., Shoubugawa,Y. and Tanaka,T.
EPI_ISL_2580657, EPI_ISL_2580659, EPI_ISL_2580661	Akinobu Hibino Niigata University, International Health, Public Health	Akinobu Hibino Niigata University, International Health, Public Health	Hibino,A., Saito,R., Shoubugawa,Y. and Kaji,H.
EPI_ISL_2580663, EPI_ISL_2580665, EPI_ISL_2580667, EPI_ISL_2580669, EPI_ISL_2580671, EPI_ISL_2580673, EPI_ISL_2580675, EPI_ISL_2580677, EPI_ISL_2580679, EPI_ISL_2580681, EPI_ISL_2580683, EPI_ISL_2580685, EPI_ISL_2580687, EPI_ISL_2580689			
see above	Akinobu Hibino Niigata University, International Health, Public Health	Akinobu Hibino Niigata University, International Health, Public Health	Hibino,A., Saito,R., Shoubugawa,Y. and Suzuki,E.
EPI_ISL_2580813, EPI_ISL_2580815	Chinese Academy of Medical Sciences & Peking Union Medical College	Chinese Academy of Medical Sciences & Peking Union Medical College	Jia,B., Xiao,Y., Wang,Y., Chen,L., Zhang,J., Ren,L. and Wang,J.
EPI_ISL_2580842, EPI_ISL_2580843, EPI_ISL_2580845, EPI_ISL_2580848, EPI_ISL_2580849, EPI_ISL_2580851, EPI_ISL_2580854, EPI_ISL_2580856	WHO WPRO Measles Regional Reference Lab, Key Laboratory of Medical Virology Ministry of Health, National Institute for Viral Disease Control and Prevention, China Center for Disease Control and Prevention	WHO WPRO Measles Regional Reference Lab, Key Laboratory of Medical Virology Ministry of Health, National Institute for Viral Disease Control and Prevention, China Center for Disease Control and Prevention	Zhang,Y., Song,J. and Xu,W.
EPI_ISL_2580863, EPI_ISL_2580865	Department of Public Health Laboratory Sciences, West China School of Public Health, Sichuan University	Department of Public Health Laboratory Sciences, West China School of Public Health, Sichuan University	Hu,W.P. and Pei,F.X.
EPI_ISL_2580873, EPI_ISL_2580881	WHO WPRO Measles Regional Reference Lab, Key Laboratory of Medical Virology Ministry of Health, National Institute for Viral Disease Control and Prevention, China Center for Disease Control and Prevention	WHO WPRO Measles Regional Reference Lab, Key Laboratory of Medical Virology Ministry of Health, National Institute for Viral Disease Control and Prevention, China Center for Disease Control and Prevention	Zhang,Y., Song,J. and Xu,W.
EPI_ISL_2580883, EPI_ISL_2580887, EPI_ISL_2580892, EPI_ISL_2580893, EPI_ISL_2580900	University of Wuerzburg, Institute of Virology and Immunobiology	University of Wuerzburg, Institute of Virology and Immunobiology	Prifert,C., Hofmann,D. and Weissbrich,B.
EPI_ISL_2580923	Virology, School of Public Health, Tehran University of Medical Sciences	Virology, School of Public Health, Tehran University of Medical Sciences	Yavarian,J., Faghihloo,E. and Mokhtari Azad,T.
EPI_ISL_2580947	Virology, Tohoku University Graduate School of Medicine	Virology, Tohoku University Graduate School of Medicine	Malasao,R., Okamoto,M., Chaimongkol,N., Imamura,T., Tohma,K., Dapat,I., Dapat,C., Suzuki,A., Saito,M., Saito,M., Tamaki,R., Segubre-Mercado,E., Igoy,M.A.U., Lupisan,S., Olveda,R. and Oshitani,H.
EPI_ISL_2581024	Medical Virology, Faculty of Public Health, Tehran University of Medical Sciences	Medical Virology, Faculty of Public Health, Tehran University of Medical Sciences	Arjeyni,Y., Faghihloo,E. and Mokhtari Azad,T.
EPI_ISL_2581031, EPI_ISL_2581032, EPI_ISL_2581033, EPI_ISL_2581034, EPI_ISL_2581036, EPI_ISL_2581037	Medicine, American University of Beirut	Medicine, American University of Beirut	Abou-El-Hassan,H. and Zaraket,H.
EPI_ISL_2581041	Akinobu Hibino Niigata University, International Health, Public Health	Akinobu Hibino Niigata University, International Health, Public Health	Hibino,A., Saito,R., Shoubugawa,Y. and Seo,T.
EPI_ISL_2581042, EPI_ISL_2581043, EPI_ISL_2581044, EPI_ISL_2581045, EPI_ISL_2581046, EPI_ISL_2581047, EPI_ISL_2581048, EPI_ISL_2581049, EPI_ISL_2581050, EPI_ISL_2581051, EPI_ISL_2581052, EPI_ISL_2581054, EPI_ISL_2581056, EPI_ISL_2581057, EPI_ISL_2581058, EPI_ISL_2581060			
see above	Akinobu Hibino Niigata University, International Health, Public Health	Akinobu Hibino Niigata University, International Health, Public Health	Hibino,A., Saito,R., Shoubugawa,Y. and Tomimoto,K.
EPI_ISL_2581065, EPI_ISL_2581068, EPI_ISL_2581069, EPI_ISL_2581072, EPI_ISL_2581073, EPI_ISL_2581075, EPI_ISL_2581077, EPI_ISL_2581079, EPI_ISL_2581082, EPI_ISL_2581083, EPI_ISL_2581085, EPI_ISL_2581088, EPI_ISL_2581090, EPI_ISL_2581091, EPI_ISL_2581093, EPI_ISL_2581101, EPI_ISL_2581105, EPI_ISL_2581108, EPI_ISL_2581109			
see above	Eiko Hirano Fukui Prefectural Institute of Public Health and Environmental Science	Eiko Hirano Fukui Prefectural Institute of Public Health and Environmental Science	Hirano,E. and Kobayashi,M.
EPI_ISL_2581115, EPI_ISL_2581117, EPI_ISL_2581119,	Akinobu Hibino Niigata University, International Health, Public Health	Akinobu Hibino Niigata University, International Health, Public Health	Hibino,A., Saito,R., Shoubugawa,Y. and Sugimura,T.

EPI_ISL_2581122, EPI_ISL_2581123, EPI_ISL_2581125, EPI_ISL_2581127, EPI_ISL_2581129, EPI_ISL_2581132			
EPI_ISL_2581182, EPI_ISL_2581183, EPI_ISL_2581185, EPI_ISL_2581188, EPI_ISL_2581189	Akinobu Hibino Niigata University, International Health, Public Health	Akinobu Hibino Niigata University, International Health, Public Health	Hibino,A., Saito,R., Shoubugawa,Y. and Sano,Y.
EPI_ISL_2581191	Akinobu Hibino Niigata University, International Health, Public Health	Akinobu Hibino Niigata University, International Health, Public Health	Hibino,A., Saito,R., Shoubugawa,Y., Htay,H.T., Khin,Y.O., Yi,Y.M. and Yadanar,K.
EPI_ISL_2581215	Chinese Academy of Medical Sciences & Peking Union Medical College	Chinese Academy of Medical Sciences & Peking Union Medical College	Jia,B., Xiao,Y., Wang,Y., Chen,L., Zhang,J., Ren,L. and Wang,J.
EPI_ISL_2581260	University of Wuerzburg, Institute of Virology and Immunobiology	University of Wuerzburg, Institute of Virology and Immunobiology	Prifert,C., Hofmann,D. and Weissbrich,B.
EPI_ISL_2581363, EPI_ISL_2581365	Akinobu Hibino Niigata University, International Health, Public Health	Akinobu Hibino Niigata University, International Health, Public Health	Hibino,A., Saito,R., Shoubugawa,Y. and Sano,Y.
EPI_ISL_2581367, EPI_ISL_2581369, EPI_ISL_2581372, EPI_ISL_2581373, EPI_ISL_2581375	Akinobu Hibino Niigata University, International Health, Public Health	Akinobu Hibino Niigata University, International Health, Public Health	Hibino,A., Saito,R., Shoubugawa,Y., Htay,H.T., Khin,Y.O., Yi,Y.M. and Yadanar,K.
EPI_ISL_2581389, EPI_ISL_2581391, EPI_ISL_2581392	WHO WPRO Measles Regional Reference Lab, Key Laboratory of Medical Virology Ministry of Health, National Institute for Viral Disease Control and Prevention, China Center for Disease Control and Prevention	WHO WPRO Measles Regional Reference Lab, Key Laboratory of Medical Virology Ministry of Health, National Institute for Viral Disease Control and Prevention, China Center for Disease Control and Prevention	Zhang,Y., Song,J. and Xu,W.
EPI_ISL_2581395	University of Wuerzburg, Institute of Virology and Immunobiology	University of Wuerzburg, Institute of Virology and Immunobiology	Prifert,C., Hofmann,D. and Weissbrich,B.
EPI_ISL_2581414, EPI_ISL_2581415	Medical Virology, Faculty of Public Health, Tehran University of Medical Sciences	Medical Virology, Faculty of Public Health, Tehran University of Medical Sciences	Arjeyni,Y., Faghihloo,E. and Mokhtari Azad,T.
EPI_ISL_2581443, EPI_ISL_2581445, EPI_ISL_2581447, EPI_ISL_2581449, EPI_ISL_2581451, EPI_ISL_2581454, EPI_ISL_2581455, EPI_ISL_2581458	Protein Research Chair, Department of Biochemistry, College of Science, King Saud University	Protein Research Chair, Department of Biochemistry, College of Science, King Saud University	Ahmed,A., Haider,M.S.H., Arshad,M., Baaboud,A.O., AlSenaidy,A.M., AlSaadi,M.M., Mobaireek,K.F., Sullender,W.M. and Parveen,S.
EPI_ISL_2581476	Eiko Hirano Fukui Prefectural Institute of Public Health and Environmental Science	Eiko Hirano Fukui Prefectural Institute of Public Health and Environmental Science	Hirano,E. and Kobayashi,M.
EPI_ISL_2581477	Akinobu Hibino Niigata University, International Health, Public Health	Akinobu Hibino Niigata University, International Health, Public Health	Hibino,A., Saito,R., Shoubugawa,Y., Htay,H.T., Khin,Y.O., Yi,Y.M. and Yadanar,K.
EPI_ISL_2581479	Virology, Centre Pasteur du Cameroun	Virology, Centre Pasteur du Cameroun	Kenmoe,S., Vernet,M.-A., Miszczak,F., Dina,J., Penlap,V.B., Vabret,A. and Njouom,R.
EPI_ISL_2581485, EPI_ISL_2581488	Centre for Interdisciplinary Research in Basic Science	Centre for Interdisciplinary Research in Basic Science	Haider,M.S.H., Naqvi,I.H., Broor,S., Ahmed,A. and Parveen,S.
EPI_ISL_2581489, EPI_ISL_2581491	Protein Research Chair, Department of Biochemistry, College of Science, King Saud University	Protein Research Chair, Department of Biochemistry, College of Science, King Saud University	Ahmed,A., Haider,M.S.H., Arshad,M., Baaboud,A.O., AlSenaidy,A.M., AlSaadi,M.M., Mobaireek,K.F., Sullender,W.M. and Parveen,S.
EPI_ISL_2581499	Medical Virology, Faculty of Public Health, Tehran University of Medical Sciences	Medical Virology, Faculty of Public Health, Tehran University of Medical Sciences	Arjeyni,Y., Faghihloo,E. and Mokhtari Azad,T.
EPI_ISL_2581511, EPI_ISL_2581513	Virology, Centre Pasteur du Cameroun	Virology, Centre Pasteur du Cameroun	Kenmoe,S., Vernet,M.-A., Miszczak,F., Dina,J., Penlap,V.B., Vabret,A. and Njouom,R.
EPI_ISL_2581521	Akinobu Hibino Niigata University, International Health, Public Health	Akinobu Hibino Niigata University, International Health, Public Health	Hibino,A., Saito,R., Shoubugawa,Y. and Takei,T.
EPI_ISL_2581525, EPI_ISL_2581528	Virology, Centre Pasteur du Cameroun	Virology, Centre Pasteur du Cameroun	Kenmoe,S., Vernet,M.-A., Miszczak,F., Dina,J., Penlap,V.B., Vabret,A. and Njouom,R.
EPI_ISL_2581536	Protein Research Chair, Department of Biochemistry, College of Science, King Saud University	Protein Research Chair, Department of Biochemistry, College of Science, King Saud University	Ahmed,A., Haider,M.S.H., Arshad,M., Baaboud,A.O., AlSenaidy,A.M., AlSaadi,M.M., Mobaireek,K.F., Sullender,W.M. and Parveen,S.
EPI_ISL_2581551	Centre for Interdisciplinary Research in Basic Science	Centre for Interdisciplinary Research in Basic Science	Haider,M.S.H., Naqvi,I.H., Broor,S., Ahmed,A. and Parveen,S.
EPI_ISL_2581557, EPI_ISL_2581560, EPI_ISL_2581561	Protein Research Chair, Department of Biochemistry, College of Science, King Saud University	Protein Research Chair, Department of Biochemistry, College of Science, King Saud University	Ahmed,A., Haider,M.S.H., Arshad,M., Baaboud,A.O., AlSenaidy,A.M., AlSaadi,M.M., Mobaireek,K.F., Sullender,W.M. and Parveen,S.
EPI_ISL_2581564	Centre for Interdisciplinary Research in Basic Science	Centre for Interdisciplinary Research in Basic Science	Haider,M.S.H., Naqvi,I.H., Broor,S., Ahmed,A. and Parveen,S.
EPI_ISL_2581565, EPI_ISL_2581567, EPI_ISL_2581569, EPI_ISL_2581571, EPI_ISL_2581574	Protein Research Chair, Department of Biochemistry, College of Science, King Saud University	Protein Research Chair, Department of Biochemistry, College of Science, King Saud University	Ahmed,A., Haider,M.S.H., Arshad,M., Baaboud,A.O., AlSenaidy,A.M., AlSaadi,M.M., Mobaireek,K.F., Sullender,W.M. and Parveen,S.
EPI_ISL_2581613, EPI_ISL_2581690	Chinese Academy of Medical Sciences & Peking Union Medical College	Chinese Academy of Medical Sciences & Peking Union Medical College	Jia,B., Xiao,Y., Wang,Y., Chen,L., Zhang,J., Ren,L. and Wang,J.
EPI_ISL_2581769, EPI_ISL_2581771, EPI_ISL_2581773, EPI_ISL_2581776, EPI_ISL_2581777, EPI_ISL_2581779	Laboratory of Virology, Capital Institute of Pediatrics	Laboratory of Virology, Capital Institute of Pediatrics	Cui,G., Zhu,R., Qian,Y., Deng,J., Zhao,L., Sun,Y. and Wang,F.
EPI_ISL_2581835	Chinese Academy of Medical Sciences & Peking Union Medical College	Chinese Academy of Medical Sciences & Peking Union Medical College	Jia,B., Xiao,Y., Wang,Y., Chen,L., Zhang,J., Ren,L. and Wang,J.
EPI_ISL_2581855, EPI_ISL_2581857	Central Laboratory, Guangzhou Women and Children's Medical Center	Central Laboratory, Guangzhou Women and Children's Medical Center	Xie,J.H., Zhu,B., Zhong,J.Y., Chen,Y. and Zhang,Y.Y.
EPI_ISL_2581891, EPI_ISL_2581893, EPI_ISL_2581896, EPI_ISL_2581897, EPI_ISL_2581899	Laboratory of Virology, Capital Institute of Pediatrics	Laboratory of Virology, Capital Institute of Pediatrics	Cui,G., Zhu,R., Qian,Y., Deng,J., Zhao,L., Sun,Y. and Wang,F.
EPI_ISL_2582147	Experimental Pathology, Immunology, and Microbiology, American University of Beirut	Experimental Pathology, Immunology, and Microbiology, American University of Beirut	Hamdan,F., Ezzeddine,A., Elbahesh,H. and Zaraket,H.
EPI_ISL_2582157, EPI_ISL_2582160, EPI_ISL_2582161	Epidemiology and Demography Department, KEMRI-Wellcome Trust Research Programme	Epidemiology and Demography Department, KEMRI-Wellcome Trust Research Programme	Otieno,J.R., Kamau,E.M., Oketch,J.W., Ngoi,J.M., Agoti,C.N., Gichuki,A.M., Otieno,G.P., Ngama,M., Cane,P.A., Kellam,P., Cotten,M., Lemey,P. and Nokes,D.J.
EPI_ISL_2582167	Lab Medicine, UW	Lab Medicine, UW	Greninger,A.L., Makhsous,N., Kuypers,J.M., Shean,R.C. and Jerome,K.R.
EPI_ISL_2582180	Broad Institute of MIT & Harvard	Broad Institute of MIT & Harvard	Newman,R.M., Zody,M.C., DeVincenzo,J.P., Grad,Y., Lipsitch,M., Murphy,R., Fitzgerald,M., Young,S., Gargeya,S., Poon,T.W., Charlebois,P., Weiner,B., Yang,X., Piper,M.E., McCowan,C., Ireland,A., Levin,J., Malboeuf,C., Qu,J., Chapman,S.B., Murphy,C., Wortman,J., Nusbaum,C. and Birren,B.
EPI_ISL_2582183	Virology, Graduate School of Medicine, Tohoku University	Virology, Graduate School of Medicine, Tohoku University	Malasao,R., Furuse,Y., Okamoto,M., Dapat,C., Saito,M., Saito-Obata,M., Tamaki,R., Segubre-Mercado,E., Lupisan,S. and Oshitani,H.
EPI_ISL_2582191	J. Craig Venter Institute	J. Craig Venter Institute	Shrivastava,S., Halpin,R.A., Puri,V., Fedorova,N.B., Stockwell,T., Amedeo,P., Katzel,D., Schobel,S., Pickett,B.E., Moore,M., Chappell,J., Larkin,E., Wentworth,D.E., Anderson,L.J. and Hartert,T.
EPI_ISL_2582197	Lab Medicine, UW	Lab Medicine, UW	Greninger,A.L., Makhsous,N., Kuypers,J.M., Shean,R.C. and Jerome,K.R.
EPI_ISL_2582199, EPI_ISL_2582201	Epidemiology and Demography Department, KEMRI-Wellcome Trust Research Programme	Epidemiology and Demography Department, KEMRI-Wellcome Trust Research Programme	Otieno,J.R., Kamau,E.M., Oketch,J.W., Ngoi,J.M., Agoti,C.N., Gichuki,A.M., Otieno,G.P., Ngama,M., Cane,P.A., Kellam,P., Cotten,M., Lemey,P. and Nokes,D.J.
EPI_ISL_2582209	Microbiology, Sanjay Gandhi Post Graduate Institute of Medical Science	Microbiology, Sanjay Gandhi Post Graduate Institute of Medical Science	Saxena,S., Singh,D., Tripathi,R. and Kushwaha,R.
EPI_ISL_2582214	Virology, King Institute of Preventive Medicine & Research	Virology, King Institute of Preventive Medicine & Research	Sureshbabu B,V., Gunasekaran,P., Kaveri,K., Venkataraman,P., Kiruba,R., Magesh,S. and SenthilRaja,R.
EPI_ISL_2582215	Microbiology, Sanjay Gandhi Post Graduate Institute of Medical Science	Microbiology, Sanjay Gandhi Post Graduate Institute of Medical Science	Saxena,S., Singh,D., Tripathi,R., Dhole,T.N. and Kushwaha,R.
EPI_ISL_2582220	Virology, King Institute of Preventive Medicine & Research	Virology, King Institute of Preventive Medicine & Research	Sureshbabu B,V., Gunasekaran,P., Kaveri,K., Venkataraman,P., Kiruba,R., Magesh,S. and SenthilRaja,R.
EPI_ISL_2582223, EPI_ISL_2582227	Marie Bashir Institute for Infectious Diseases and Biosecurity & Sydney Medical School, The University of Sydney, Westmead Institute for Medical Research	Marie Bashir Institute for Infectious Diseases and Biosecurity & Sydney Medical School, The University of Sydney, Westmead Institute for Medical Research	Eden,J.-S., Kok,J., Dwyer,D.E., Fernandez,M., Carter,I. and Holmes,E.C.
EPI_ISL_2582256, EPI_ISL_2582257, EPI_ISL_2582260, EPI_ISL_2582270, EPI_ISL_2582279, EPI_ISL_2582281, EPI_ISL_2582284	Epidemiology and Demography Department, KEMRI-Wellcome Trust Research Programme	Epidemiology and Demography Department, KEMRI-Wellcome Trust Research Programme	Otieno,J.R., Kamau,E.M., Oketch,J.W., Ngoi,J.M., Agoti,C.N., Gichuki,A.M., Otieno,G.P., Ngama,M., Cane,P.A., Kellam,P., Cotten,M., Lemey,P. and Nokes,D.J.
EPI_ISL_2582285	Department of Pediatrics, Center of Excellence in Clinical Virology, Chulalongkorn	Department of Pediatrics, Center of Excellence in Clinical Virology, Chulalongkorn	Thongpan,I.
EPI_ISL_2582292, EPI_ISL_2582293	Epidemiology and Demography Department, KEMRI-Wellcome Trust Research Programme	Epidemiology and Demography Department, KEMRI-Wellcome Trust Research Programme	Otieno,J.R., Kamau,E.M., Oketch,J.W., Ngoi,J.M., Agoti,C.N., Gichuki,A.M., Otieno,G.P., Ngama,M., Cane,P.A., Kellam,P., Cotten,M., Lemey,P. and Nokes,D.J.
EPI_ISL_2582296	Department of Pediatrics, Center of Excellence in Clinical Virology, Chulalongkorn	Department of Pediatrics, Center of Excellence in Clinical Virology, Chulalongkorn	Thongpan,I.
EPI_ISL_2582306, EPI_ISL_2582307, EPI_ISL_2582309, EPI_ISL_2582312, EPI_ISL_2582313, EPI_ISL_2582316, EPI_ISL_2582318, EPI_ISL_2582319, EPI_ISL_2582321, EPI_ISL_2582324, EPI_ISL_2582325, EPI_ISL_2582327, EPI_ISL_2582330, EPI_ISL_2582332, EPI_ISL_2582333, EPI_ISL_2582335, EPI_ISL_2582337, EPI_ISL_2582340, EPI_ISL_2582341, EPI_ISL_2582342, EPI_ISL_2582344, EPI_ISL_2582345, EPI_ISL_2582348, EPI_ISL_2582349, EPI_ISL_2582351, EPI_ISL_2582354, EPI_ISL_2582355, EPI_ISL_2582357, EPI_ISL_2582360, EPI_ISL_2582361, EPI_ISL_2582363, EPI_ISL_2582366, EPI_ISL_2582367, EPI_ISL_2582370, EPI_ISL_2582371, EPI_ISL_2582373, EPI_ISL_2582375, EPI_ISL_2582378, EPI_ISL_2582379, EPI_ISL_2582381, EPI_ISL_2582383, EPI_ISL_2582386, EPI_ISL_2582387, EPI_ISL_2582389, EPI_ISL_2582391, EPI_ISL_2582393, EPI_ISL_2582395, EPI_ISL_2582397, EPI_ISL_2582399, EPI_ISL_2582402, EPI_ISL_2582403, EPI_ISL_2582405, EPI_ISL_2582407, EPI_ISL_2582410, EPI_ISL_2582411, EPI_ISL_2582413, EPI_ISL_2582416, EPI_ISL_2582417, EPI_ISL_2582419, EPI_ISL_2582422, EPI_ISL_2582423, EPI_ISL_2582426, EPI_ISL_2582427, EPI_ISL_2582430, EPI_ISL_2582431, EPI_ISL_2582434, EPI_ISL_2582435, EPI_ISL_2582445, EPI_ISL_2582485, EPI_ISL_2582490, EPI_ISL_2582495, EPI_ISL_2582497, EPI_ISL_2582500			
see above	Broad Institute of MIT & Harvard	Broad Institute of MIT & Harvard	Newman,R.M., Zody,M.C., DeVincenzo,J.P., Grad,Y., Lipsitch,M., Murphy,R., Fitzgerald,M., Young,S., Gargeya,S., Poon,T.W., Charlebois,P., Weiner,B., Yang,X., Piper,M.E., McCowan,C., Ireland,A., Levin,J., Malboeuf,C., Qu,J., Chapman,S.B., Murphy,C., Wortman,J., Nusbaum,C. and Birren,B.
EPI_ISL_2582510, EPI_ISL_2582512	Marie Bashir Institute for Infectious Diseases and Biosecurity & Sydney Medical School, The University of Sydney, Westmead Institute for Medical Research	Marie Bashir Institute for Infectious Diseases and Biosecurity & Sydney Medical School, The University of Sydney, Westmead Institute for Medical Research	Eden,J.-S., Kok,J., Dwyer,D.E., Fernandez,M., Carter,I. and Holmes,E.C.
EPI_ISL_2582515, EPI_ISL_2582517	Broad Institute of MIT & Harvard	Broad Institute of MIT & Harvard	Newman,R.M., Zody,M.C., DeVincenzo,J.P., Grad,Y., Lipsitch,M., Murphy,R., Fitzgerald,M., Young,S., Gargeya,S., Poon,T.W., Charlebois,P., Weiner,B., Yang,X., Piper,M.E., McCowan,C., Ireland,A., Levin,J., Malboeuf,C., Qu,J., Chapman,S.B., Murphy,C., Wortman,J., Nusbaum,C. and Birren,B.
EPI_ISL_2582538, EPI_ISL_2582540, EPI_ISL_2582541, EPI_ISL_2582551	Lab Medicine, UW	Lab Medicine, UW	Greninger,A.L., Makhsous,N., Kuypers,J.M., Shean,R.C. and Jerome,K.R.

EPI_ISL_2582565, EPI_ISL_2582567	Epidemiology and Demography Department, KEMRI-Wellcome Trust Research Programme	Epidemiology and Demography Department, KEMRI-Wellcome Trust Research Programme	Otieno,J.R., Kamau,E.M., Oketch,J.W., Ngoi,J.M., Agoti,C.N., Gichuki,A.M., Otieno,G.P., Ngama,M., Cane,P.A., Kellam,P., Cotten,M., Lemey,P. and Nokes,D.J.
EPI_ISL_2582577, EPI_ISL_2582579, EPI_ISL_2582581, EPI_ISL_2582583, EPI_ISL_2582585, EPI_ISL_2582587, EPI_ISL_2582588, EPI_ISL_2582590, EPI_ISL_2582592, EPI_ISL_2582594, EPI_ISL_2582596, EPI_ISL_2582598, EPI_ISL_2582600, EPI_ISL_2582601, EPI_ISL_2582603, EPI_ISL_2582605, EPI_ISL_2582607, EPI_ISL_2582609, EPI_ISL_2582610, EPI_ISL_2582612, EPI_ISL_2582614, EPI_ISL_2582618			
see above	Broad Institute of MIT & Harvard	Broad Institute of MIT & Harvard	Newman,R.M., Zody,M.C., DeVincenzo,J.P., Grad,Y., Lipsitch,M., Murphy,R., Fitzgerald,M., Young,S., Gargeya,S., Poon,T.W., Charlebois,P., Weiner,B., Yang,X., Piper,M.E., McCowan,C., Ireland,A., Levin,J., Malboeuf,C., Qu,J., Chapman,S.B., Murphy,C., Wortman,J., Nusbaum,C. and Birren,B.
EPI_ISL_2582646	Marie Bashir Institute for Infectious Diseases and Biosecurity & Sydney Medical School, The University of Sydney, Westmead Institute for Medical Research	Marie Bashir Institute for Infectious Diseases and Biosecurity & Sydney Medical School, The University of Sydney, Westmead Institute for Medical Research	Eden,J.-S., Kok,J., Dwyer,D.E., Fernandez,M., Carter,I. and Holmes,E.C.
EPI_ISL_2582669, EPI_ISL_2582670, EPI_ISL_2582673	Epidemiology and Demography Department, KEMRI-Wellcome Trust Research Programme	Epidemiology and Demography Department, KEMRI-Wellcome Trust Research Programme	Otieno,J.R., Kamau,E.M., Oketch,J.W., Ngoi,J.M., Agoti,C.N., Gichuki,A.M., Otieno,G.P., Ngama,M., Cane,P.A., Kellam,P., Cotten,M., Lemey,P. and Nokes,D.J.
EPI_ISL_2582681	Experimental Pathology, Immunology, and Microbiology, American University of Beirut	Experimental Pathology, Immunology, and Microbiology, American University of Beirut	Ezzeddine,A.M.
EPI_ISL_2582683, EPI_ISL_2582686, EPI_ISL_2582780, EPI_ISL_2582784	Broad Institute of MIT & Harvard	Broad Institute of MIT & Harvard	Newman,R.M., Zody,M.C., DeVincenzo,J.P., Grad,Y., Lipsitch,M., Murphy,R., Fitzgerald,M., Young,S., Gargeya,S., Poon,T.W., Charlebois,P., Weiner,B., Yang,X., Piper,M.E., McCowan,C., Ireland,A., Levin,J., Malboeuf,C., Qu,J., Chapman,S.B., Murphy,C., Wortman,J., Nusbaum,C. and Birren,B.
EPI_ISL_2582787	Lab Medicine, UW	Lab Medicine, UW	Greninger,A.L., Makhsous,N., Kuypers,J.M., Shean,R.C. and Jerome,K.R.
EPI_ISL_2582791	Epidemiology and Demography Department, KEMRI-Wellcome Trust Research Programme	Epidemiology and Demography Department, KEMRI-Wellcome Trust Research Programme	Otieno,J.R., Kamau,E.M., Oketch,J.W., Ngoi,J.M., Agoti,C.N., Gichuki,A.M., Otieno,G.P., Ngama,M., Cane,P.A., Kellam,P., Cotten,M., Lemey,P. and Nokes,D.J.
EPI_ISL_2582798	Lab Medicine, UW	Lab Medicine, UW	Greninger,A.L., Makhsous,N., Kuypers,J.M., Shean,R.C. and Jerome,K.R.
EPI_ISL_2582808, EPI_ISL_2582810, EPI_ISL_2582813	Broad Institute of MIT & Harvard	Broad Institute of MIT & Harvard	Newman,R.M., Zody,M.C., DeVincenzo,J.P., Grad,Y., Lipsitch,M., Murphy,R., Fitzgerald,M., Young,S., Gargeya,S., Poon,T.W., Charlebois,P., Weiner,B., Yang,X., Piper,M.E., McCowan,C., Ireland,A., Levin,J., Malboeuf,C., Qu,J., Chapman,S.B., Murphy,C., Wortman,J., Nusbaum,C. and Birren,B.
EPI_ISL_2582816	Marie Bashir Institute for Infectious Diseases and Biosecurity & Sydney Medical School, The University of Sydney, Westmead Institute for Medical Research	Marie Bashir Institute for Infectious Diseases and Biosecurity & Sydney Medical School, The University of Sydney, Westmead Institute for Medical Research	Eden,J.-S., Kok,J., Dwyer,D.E., Fernandez,M., Carter,I. and Holmes,E.C.
EPI_ISL_2582819, EPI_ISL_2582820, EPI_ISL_2582823, EPI_ISL_2582826, EPI_ISL_2582829, EPI_ISL_2582844	Broad Institute of MIT & Harvard	Broad Institute of MIT & Harvard	Newman,R.M., Zody,M.C., DeVincenzo,J.P., Grad,Y., Lipsitch,M., Murphy,R., Fitzgerald,M., Young,S., Gargeya,S., Poon,T.W., Charlebois,P., Weiner,B., Yang,X., Piper,M.E., McCowan,C., Ireland,A., Levin,J., Malboeuf,C., Qu,J., Chapman,S.B., Murphy,C., Wortman,J., Nusbaum,C. and Birren,B.
EPI_ISL_2582849, EPI_ISL_2582851	Center for Infectious Diseases, School of Public Health, University of Texas Health Science Center	Center for Infectious Diseases, School of Public Health, University of Texas Health Science Center	Bahl,J., Hixson,J., Kim,D.-K., Qiu,X., Piedra,P.A., Piedra,F.-A., Avadhanula,V. and Machado,A.A.
EPI_ISL_2582854	Broad Institute of MIT & Harvard	Broad Institute of MIT & Harvard	Newman,R.M., Zody,M.C., DeVincenzo,J.P., Grad,Y., Lipsitch,M., Murphy,R., Fitzgerald,M., Young,S., Gargeya,S., Poon,T.W., Charlebois,P., Weiner,B., Yang,X., Piper,M.E., McCowan,C., Ireland,A., Levin,J., Malboeuf,C., Qu,J., Chapman,S.B., Murphy,C., Wortman,J., Nusbaum,C. and Birren,B.
EPI_ISL_2582866	Epidemiology and Demography Department, KEMRI-Wellcome Trust Research Programme	Epidemiology and Demography Department, KEMRI-Wellcome Trust Research Programme	Otieno,J.R., Kamau,E.M., Oketch,J.W., Ngoi,J.M., Agoti,C.N., Gichuki,A.M., Otieno,G.P., Ngama,M., Cane,P.A., Kellam,P., Cotten,M., Lemey,P. and Nokes,D.J.
EPI_ISL_2582883, EPI_ISL_2582904, EPI_ISL_2582951	Broad Institute of MIT & Harvard	Broad Institute of MIT & Harvard	Newman,R.M., Zody,M.C., DeVincenzo,J.P., Grad,Y., Lipsitch,M., Murphy,R., Fitzgerald,M., Young,S., Gargeya,S., Poon,T.W., Charlebois,P., Weiner,B., Yang,X., Piper,M.E., McCowan,C., Ireland,A., Levin,J., Malboeuf,C., Qu,J., Chapman,S.B., Murphy,C., Wortman,J., Nusbaum,C. and Birren,B.
EPI_ISL_2583026	Medicine, University of Washington, 300 9th Ave, Harborview Research & Training Building	Medicine, University of Washington, 300 9th Ave, Harborview Research & Training Building	Chu,H., Scott,E. and Roychoudhury,P.
EPI_ISL_2583027	Epidemiology and Demography Department, KEMRI-Wellcome Trust Research Programme	Epidemiology and Demography Department, KEMRI-Wellcome Trust Research Programme	Otieno,J.R., Kamau,E.M., Oketch,J.W., Ngoi,J.M., Agoti,C.N., Gichuki,A.M., Otieno,G.P., Ngama,M., Cane,P.A., Kellam,P., Cotten,M., Lemey,P. and Nokes,D.J.
EPI_ISL_2583034	Broad Institute of MIT & Harvard	Broad Institute of MIT & Harvard	Newman,R.M., Zody,M.C., DeVincenzo,J.P., Grad,Y., Lipsitch,M., Murphy,R., Fitzgerald,M., Young,S., Gargeya,S., Poon,T.W., Charlebois,P., Weiner,B., Yang,X., Piper,M.E., McCowan,C., Ireland,A., Levin,J., Malboeuf,C., Qu,J., Chapman,S.B., Murphy,C., Wortman,J., Nusbaum,C. and Birren,B.
EPI_ISL_2583036	Epidemiology and Demography Department, KEMRI-Wellcome Trust Research Programme	Epidemiology and Demography Department, KEMRI-Wellcome Trust Research Programme	Otieno,J.R., Kamau,E.M., Oketch,J.W., Ngoi,J.M., Agoti,C.N., Gichuki,A.M., Otieno,G.P., Ngama,M., Cane,P.A., Kellam,P., Cotten,M., Lemey,P. and Nokes,D.J.
EPI_ISL_2583040	Broad Institute of MIT & Harvard	Broad Institute of MIT & Harvard	Newman,R.M., Zody,M.C., DeVincenzo,J.P., Grad,Y., Lipsitch,M., Murphy,R., Fitzgerald,M., Young,S., Gargeya,S., Poon,T.W., Charlebois,P., Weiner,B., Yang,X., Piper,M.E., McCowan,C., Ireland,A., Levin,J., Malboeuf,C., Qu,J., Chapman,S.B., Murphy,C., Wortman,J., Nusbaum,C. and Birren,B.
EPI_ISL_2583043	Epidemiology and Demography Department, KEMRI-Wellcome Trust Research Programme	Epidemiology and Demography Department, KEMRI-Wellcome Trust Research Programme	Otieno,J.R., Kamau,E.M., Oketch,J.W., Ngoi,J.M., Agoti,C.N., Gichuki,A.M., Otieno,G.P., Ngama,M., Cane,P.A., Kellam,P., Cotten,M., Lemey,P. and Nokes,D.J.
EPI_ISL_2583045, EPI_ISL_2583050	Broad Institute of MIT & Harvard	Broad Institute of MIT & Harvard	Newman,R.M., Zody,M.C., DeVincenzo,J.P., Grad,Y., Lipsitch,M., Murphy,R., Fitzgerald,M., Young,S., Gargeya,S., Poon,T.W., Charlebois,P., Weiner,B., Yang,X., Piper,M.E., McCowan,C., Ireland,A., Levin,J., Malboeuf,C., Qu,J., Chapman,S.B., Murphy,C., Wortman,J., Nusbaum,C. and Birren,B.
EPI_ISL_2583052	Epidemiology and Demography Department, KEMRI-Wellcome Trust Research Programme	Epidemiology and Demography Department, KEMRI-Wellcome Trust Research Programme	Otieno,J.R., Kamau,E.M., Oketch,J.W., Ngoi,J.M., Agoti,C.N., Gichuki,A.M., Otieno,G.P., Ngama,M., Cane,P.A., Kellam,P., Cotten,M., Lemey,P. and Nokes,D.J.
EPI_ISL_2583055, EPI_ISL_2583068, EPI_ISL_2583072, EPI_ISL_2583078	Broad Institute of MIT & Harvard	Broad Institute of MIT & Harvard	Newman,R.M., Zody,M.C., DeVincenzo,J.P., Grad,Y., Lipsitch,M., Murphy,R., Fitzgerald,M., Young,S., Gargeya,S., Poon,T.W., Charlebois,P., Weiner,B., Yang,X., Piper,M.E., McCowan,C., Ireland,A., Levin,J., Malboeuf,C., Qu,J., Chapman,S.B., Murphy,C., Wortman,J., Nusbaum,C. and Birren,B.
EPI_ISL_2583126	Virology, National Institute for Research in Tribal Health	Virology, National Institute for Research in Tribal Health	Sahu,M., Barde,P.V. and Shukla,M.K.
EPI_ISL_2583127, EPI_ISL_2583129, EPI_ISL_2583131, EPI_ISL_2583132, EPI_ISL_2583133, EPI_ISL_2583135, EPI_ISL_2583138, EPI_ISL_2583141, EPI_ISL_2583143, EPI_ISL_2583144, EPI_ISL_2583146, EPI_ISL_2583148, EPI_ISL_2583153, EPI_ISL_2583158, EPI_ISL_2583162, EPI_ISL_2583164, EPI_ISL_2583166, EPI_ISL_2583168, EPI_ISL_2583169			
see above	Microbiology, Chungnam Health and Environment Research Institute	Microbiology, Chungnam Health and Environment Research Institute	Hong,J., Kim,D., Park,S. and Park,K.
EPI_ISL_2583171	J. Craig Venter Institute	J. Craig Venter Institute	Shrivastava,S., Halpin,R.A., Puri,V., Fedorova,N.B., Stockwell,T., Amedeo,P., Katzel,D., Schobel,S., Pickett,B.E., Moore,M., Chappell,J., Larkin,E., Wentworth,D.E., Anderson,L.J. and Hartert,T.
EPI_ISL_2585208, EPI_ISL_2585220	Lab. Virologia. Depto. Microbiologia, Universidad Autonoma de San Luis Potosi	Lab. Virologia. Depto. Microbiologia, Universidad Autonoma de San Luis Potosi	Comas-Garcia,A., Noyola,D.E., Cadena-Mota,S., Rico-Hernandez,M. and Bernal-Silva,S.
EPI_ISL_2585256	J. Craig Venter Institute	J. Craig Venter Institute	Shabman,R., Das,S.R., Shilts,M., Fedorova,N., Puri,V., Shrivastava,S., Amedeo,P., Williams,M., Barratt,K., Mitchell,J. and Jennings,L.
EPI_ISL_2585259	J. Craig Venter Institute	J. Craig Venter Institute	Das,S., Halpin,R.A., Bera,J., Puri,V., Fedorova,N., Tsitrin,T., Stockwell,T., Amedeo,P., Bishop,B., Katzel,D., Schobel,S., Shrivastava,S., Hartert,T., Moore,M., Chappell,J., Larkin,E., Wentworth,D.E. and Anderson,L.J.
EPI_ISL_2585267, EPI_ISL_2585268, EPI_ISL_2585269	J. Craig Venter Institute	J. Craig Venter Institute	Shabman,R., Das,S.R., Shilts,M., Fedorova,N., Puri,V., Shrivastava,S., Amedeo,P., Williams,M., Barratt,K., Mitchell,J. and Jennings,L.
EPI_ISL_2585280	J. Craig Venter Institute	J. Craig Venter Institute	Das,S., Halpin,R.A., Bera,J., Fedorova,N., Tsitrin,T., Stockwell,T., Amedeo,P., Bishop,B., Gupta,N., Hoover,J., Katzel,D., Schobel,S., Shrivastava,S., Hartert,T., Moore,M., Chappell,J., Larkin,E., Wentworth,D.E. and Anderson,L.J.
EPI_ISL_2585281	J. Craig Venter Institute	J. Craig Venter Institute	Das,S.R., Halpin,R.A., Shilts,M., Puri,V., Akopov,A., Fedorova,N., Stockwell,T., Amedeo,P., Bishop,B., Katzel,D., Schobel,S., Shrivastava,S. and Hartert,T.
EPI_ISL_2585284	J. Craig Venter Institute	J. Craig Venter Institute	Shabman,R., Das,S.R., Puri,V., Fedorova,N., Amedeo,P., Williams,M., Shrivastava,S. and Halasa,N.
EPI_ISL_2585286	J. Craig Venter Institute	J. Craig Venter Institute	Das,S., Halpin,R.A., Bera,J., Fedorova,N., Tsitrin,T., Stockwell,T., Amedeo,P., Bishop,B., Gupta,N., Hoover,J., Katzel,D., Schobel,S., Shrivastava,S., Hartert,T., Moore,M., Chappell,J., Larkin,E., Wentworth,D.E. and Anderson,L.J.
EPI_ISL_2585290	J. Craig Venter Institute	J. Craig Venter Institute	Das,S.R., Halpin,R.A., Shilts,M., Puri,V., Akopov,A., Fedorova,N., Stockwell,T., Amedeo,P., Bishop,B., Katzel,D., Schobel,S., Shrivastava,S. and Hartert,T.
EPI_ISL_2585302	J. Craig Venter Institute	J. Craig Venter Institute	Das,S., Halpin,R.A., Bera,J., Puri,V., Fedorova,N., Tsitrin,T., Stockwell,T., Amedeo,P., Bishop,B., Katzel,D., Schobel,S., Shrivastava,S., Hartert,T., Moore,M., Chappell,J., Larkin,E., Wentworth,D.E. and Anderson,L.J.
EPI_ISL_2585306	Departament of Microbiology, University of Sao Paulo Institute of Biomedical Science	Departament of Microbiology, University of Sao Paulo Institute of Biomedical Science	Espinola,E.E.
EPI_ISL_2585307, EPI_ISL_2585312, EPI_ISL_2585313, EPI_ISL_2585316, EPI_ISL_2585318	J. Craig Venter Institute	J. Craig Venter Institute	Shabman,R., Das,S.R., Shilts,M., Fedorova,N., Puri,V., Shrivastava,S., Amedeo,P., Williams,M., Barratt,K., Mitchell,J. and Jennings,L.
EPI_ISL_2585319	J. Craig Venter Institute	J. Craig Venter Institute	Das,S., Halpin,R.A., Bera,J., Fedorova,N., Tsitrin,T., Stockwell,T., Amedeo,P., Bishop,B., Gupta,N., Hoover,J., Katzel,D., Schobel,S., Shrivastava,S., Hartert,T., Moore,M., Chappell,J., Larkin,E., Wentworth,D.E. and Anderson,L.J.
EPI_ISL_2585325, EPI_ISL_2585327, EPI_ISL_2585329, EPI_ISL_2585332	J. Craig Venter Institute	J. Craig Venter Institute	Das,S.R., Halpin,R.A., Shilts,M., Puri,V., Akopov,A., Fedorova,N., Stockwell,T., Amedeo,P., Bishop,B., Katzel,D., Schobel,S., Shrivastava,S. and Hartert,T.
EPI_ISL_2585337	Microbiology, Hospital Universitari Vall d'Hebron	Microbiology, Hospital Universitari Vall d'Hebron	Gimferrer,L., Campins,M., Codina,M.G., Martin,M.D.C., Fuentes,F., Esperalba,J., Bruguera,A., Vilca,L.M., Armadans,L., Vall,A., Pumarola,T. and Anton,A.
EPI_ISL_2585570, EPI_ISL_2585628, EPI_ISL_2585630	Influenza and Other Respiratory Viruses Unit, National Center for Microbiology, Instituto de Salud Carlos III	Influenza and Other Respiratory Viruses Unit, National Center for Microbiology, Instituto de Salud Carlos III	Casas,I., Calderon,A., Gonzalez,M., Molinero,M. and Pozo,F.
EPI_ISL_2585647, EPI_ISL_2585648, EPI_ISL_2585653, EPI_ISL_2585655, EPI_ISL_2585657, EPI_ISL_2585661, EPI_ISL_2585665	Virology, Public Health Institution of Turkey	Virology, Public Health Institution of Turkey	Bayrakdar,F.
EPI_ISL_2585704	Yoshiko Sasaki Gunma Prefectural Institute of Public Health and Environmental Sciences	Yoshiko Sasaki Gunma Prefectural Institute of Public Health and Environmental Sciences	Sasaki,Y. and Kurai,D.
EPI_ISL_2586883, EPI_ISL_2586884, EPI_ISL_2586886, EPI_ISL_2586929, EPI_ISL_2586931, EPI_ISL_2586932, EPI_ISL_2587070	Influenza and Other Respiratory Viruses Unit, National Center for Microbiology, Instituto de Salud Carlos III	Influenza and Other Respiratory Viruses Unit, National Center for Microbiology, Instituto de Salud Carlos III	Casas,I., Calderon,A., Gonzalez,M., Molinero,M. and Pozo,F.

EPI_ISL_2587342	J. Craig Venter Institute	J. Craig Venter Institute	Shabman,R., Das,S.R., Shilts,M., Fedorova,N., Puri,V., Shrivastava,S., Amedeo,P., Williams,M., Barratt,K., Mitchell,J. and Jennings,L.
EPI_ISL_2587344	J. Craig Venter Institute	J. Craig Venter Institute	Shabman,R., Das,S.R., Shilts,M., Fedorova,N., Puri,V., Shrivastava,S., Amedeo,P., Hu,L., Durbin,A., Rocchi,I., Williams,T. and Hartert,T.
EPI_ISL_2587345	J. Craig Venter Institute	J. Craig Venter Institute	Das,S., Halpin,R.A., Bera,J., Fedorova,N., Tsitrin,T., Stockwell,T., Amedeo,P., Bishop,B., Gupta,N., Hoover,J., Katzel,D., Schobel,S., Shrivastava,S., Hartert,T., Moore,M., Chappell,J., Larkin,E., Wentworth,D.E. and Anderson,L.J.
EPI_ISL_2587349	J. Craig Venter Institute	J. Craig Venter Institute	Shabman,R., Das,S.R., Shilts,M., Fedorova,N., Puri,V., Shrivastava,S., Amedeo,P., Hu,L., Durbin,A., Rocchi,I., Williams,T. and Hartert,T.
EPI_ISL_2587351, EPI_ISL_2587353	J. Craig Venter Institute	J. Craig Venter Institute	Das,S., Halpin,R.A., Bera,J., Fedorova,N., Tsitrin,T., Stockwell,T., Amedeo,P., Bishop,B., Gupta,N., Hoover,J., Katzel,D., Schobel,S., Shrivastava,S., Hartert,T., Moore,M., Chappell,J., Larkin,E., Wentworth,D.E. and Anderson,L.J.
EPI_ISL_2587355, EPI_ISL_2587357	J. Craig Venter Institute	J. Craig Venter Institute	Das,S.R., Halpin,R.A., Shilts,M., Puri,V., Akopov,A., Fedorova,N., Stockwell,T., Amedeo,P., Bishop,B., Katzel,D., Schobel,S., Shrivastava,S. and Hartert,T.
EPI_ISL_2587358	J. Craig Venter Institute	J. Craig Venter Institute	Das,S., Halpin,R.A., Bera,J., Fedorova,N., Tsitrin,T., Stockwell,T., Amedeo,P., Bishop,B., Gupta,N., Hoover,J., Katzel,D., Schobel,S., Shrivastava,S., Hartert,T., Moore,M., Chappell,J., Larkin,E., Wentworth,D.E. and Anderson,L.J.
EPI_ISL_2587362	J. Craig Venter Institute	J. Craig Venter Institute	Das,S.R., Halpin,R.A., Shilts,M., Puri,V., Akopov,A., Fedorova,N., Stockwell,T., Amedeo,P., Bishop,B., Katzel,D., Schobel,S., Shrivastava,S. and Hartert,T.
EPI_ISL_2587366	J. Craig Venter Institute	J. Craig Venter Institute	Das,S., Halpin,R.A., Bera,J., Fedorova,N., Tsitrin,T., Stockwell,T., Amedeo,P., Bishop,B., Gupta,N., Hoover,J., Katzel,D., Schobel,S., Shrivastava,S., Hartert,T., Moore,M., Chappell,J., Larkin,E., Wentworth,D.E. and Anderson,L.J.
EPI_ISL_2587379, EPI_ISL_2587381, EPI_ISL_2587383, EPI_ISL_2587385, EPI_ISL_2587387, EPI_ISL_2587391, EPI_ISL_2587393, EPI_ISL_2587396, EPI_ISL_2587398, EPI_ISL_2587400, EPI_ISL_2587402, EPI_ISL_2587404			
see above	J. Craig Venter Institute	J. Craig Venter Institute	Shabman,R., Das,S.R., Shilts,M., Fedorova,N., Puri,V., Shrivastava,S., Amedeo,P., Williams,M., Barratt,K., Mitchell,J. and Jennings,L.
EPI_ISL_2587406	J. Craig Venter Institute	J. Craig Venter Institute	Das,S., Halpin,R.A., Bera,J., Puri,V., Fedorova,N., Tsitrin,T., Stockwell,T., Amedeo,P., Bishop,B., Katzel,D., Schobel,S., Shrivastava,S., Hartert,T., Moore,M., Chappell,J., Larkin,E., Wentworth,D.E. and Anderson,L.J.
EPI_ISL_2587408, EPI_ISL_2587410	J. Craig Venter Institute	J. Craig Venter Institute	Shabman,R., Das,S.R., Shilts,M., Fedorova,N., Puri,V., Shrivastava,S., Amedeo,P., Williams,M., Barratt,K., Mitchell,J. and Jennings,L.
EPI_ISL_2587412	J. Craig Venter Institute	J. Craig Venter Institute	Shabman,R., Das,S.R., Shilts,M., Fedorova,N., Puri,V., Shrivastava,S., Amedeo,P., Hu,L., Durbin,A., Rocchi,I., Williams,T. and Hartert,T.
EPI_ISL_2587414, EPI_ISL_2587415	J. Craig Venter Institute	J. Craig Venter Institute	Das,S., Halpin,R.A., Bera,J., Fedorova,N., Tsitrin,T., Stockwell,T., Amedeo,P., Bishop,B., Gupta,N., Hoover,J., Katzel,D., Schobel,S., Shrivastava,S., Hartert,T., Moore,M., Chappell,J., Larkin,E., Wentworth,D.E. and Anderson,L.J.
EPI_ISL_2587417, EPI_ISL_2587421, EPI_ISL_2587423, EPI_ISL_2587425, EPI_ISL_2587429, EPI_ISL_2587431, EPI_ISL_2587433	J. Craig Venter Institute	J. Craig Venter Institute	Das,S.R., Halpin,R.A., Shilts,M., Puri,V., Akopov,A., Fedorova,N., Stockwell,T., Amedeo,P., Bishop,B., Katzel,D., Schobel,S., Shrivastava,S. and Hartert,T.
EPI_ISL_2587434	J. Craig Venter Institute	J. Craig Venter Institute	Das,S., Halpin,R.A., Bera,J., Puri,V., Fedorova,N., Tsitrin,T., Stockwell,T., Amedeo,P., Bishop,B., Katzel,D., Schobel,S., Shrivastava,S., Hartert,T., Moore,M., Chappell,J., Larkin,E., Wentworth,D.E. and Anderson,L.J.
EPI_ISL_2587436, EPI_ISL_2587438	J. Craig Venter Institute	J. Craig Venter Institute	Das,S., Halpin,R.A., Bera,J., Fedorova,N., Tsitrin,T., Stockwell,T., Amedeo,P., Bishop,B., Gupta,N., Hoover,J., Katzel,D., Schobel,S., Shrivastava,S., Hartert,T., Moore,M., Chappell,J., Larkin,E., Wentworth,D.E. and Anderson,L.J.
EPI_ISL_2587440, EPI_ISL_2587442, EPI_ISL_2587444, EPI_ISL_2587446, EPI_ISL_2587447, EPI_ISL_2587451, EPI_ISL_2587452, EPI_ISL_2587454, EPI_ISL_2587456, EPI_ISL_2587458	J. Craig Venter Institute	J. Craig Venter Institute	Das,S.R., Halpin,R.A., Shilts,M., Puri,V., Akopov,A., Fedorova,N., Stockwell,T., Amedeo,P., Bishop,B., Katzel,D., Schobel,S., Shrivastava,S. and Hartert,T.
EPI_ISL_2587469	J. Craig Venter Institute	J. Craig Venter Institute	Das,S., Halpin,R.A., Bera,J., Fedorova,N., Tsitrin,T., Stockwell,T., Amedeo,P., Bishop,B., Gupta,N., Hoover,J., Katzel,D., Schobel,S., Shrivastava,S., Hartert,T., Moore,M., Chappell,J., Larkin,E., Wentworth,D.E. and Anderson,L.J.
EPI_ISL_2587488	Lab. Virologia. Depto. Microbiologia, Universidad Autonoma de San Luis Potosi	Lab. Virologia. Depto. Microbiologia, Universidad Autonoma de San Luis Potosi	Comas-Garcia,A., Noyola,D.E., Cadena-Mota,S., Rico-Hernandez,M. and Bernal-Silva,S.
EPI_ISL_2587494	J. Craig Venter Institute	J. Craig Venter Institute	Das,S.R., Halpin,R.A., Shilts,M., Puri,V., Akopov,A., Fedorova,N., Stockwell,T., Amedeo,P., Bishop,B., Katzel,D., Schobel,S., Shrivastava,S. and Hartert,T.
EPI_ISL_2587591, EPI_ISL_2587915	Virology, Public Health Institution of Turkey	Virology, Public Health Institution of Turkey	Bayrakdar,F.
EPI_ISL_2587935, EPI_ISL_2587936	Microbiology Department, Virology Division, College of Medicine, Taif University	Microbiology Department, Virology Division, College of Medicine, Taif University	Abdel-Moneim,A.S., Shehab,G.M., Alsulaimani,A.A., Al-Malky,M.I.R. and Kamel,M.M.
EPI_ISL_2587943, EPI_ISL_2587944	Virology, Public Health Institution of Turkey	Virology, Public Health Institution of Turkey	Bayrakdar,F.
EPI_ISL_2587953	Yoshiko Sasaki Gunma Prefectural Institute of Public Health and Environmental Sciences	Yoshiko Sasaki Gunma Prefectural Institute of Public Health and Environmental Sciences	Sasaki,Y. and Kurai,D.
EPI_ISL_2587968, EPI_ISL_2587971, EPI_ISL_2587972, EPI_ISL_2587973, EPI_ISL_2587975, EPI_ISL_2587977, EPI_ISL_2587981, EPI_ISL_2587984	Virology, Public Health Institution of Turkey	Virology, Public Health Institution of Turkey	Bayrakdar,F.
EPI_ISL_2588042, EPI_ISL_2588043, EPI_ISL_2588044, EPI_ISL_2588045	Microbiology, Hospital Universitari Vall d'Hebron	Microbiology, Hospital Universitari Vall d'Hebron	Gimferrer,L., Campins,M., Codina,M.G., Martin,M.D.C., Fuentes,F., Esperalba,J., Bruguera,A., Vilca,L.M., Armadans,L., Vall,A., Pumarola,T. and Anton,A.
EPI_ISL_2588047	Miwako SaikNorth America / USA Yokohama City Institute of Public Health	Miwako SaikNorth America / USA Yokohama City Institute of Public Health	SaikNorth America / USA,M., Kawakami,C., Usuku,S., Sasao,T. and Okubo,I.
EPI_ISL_2588056, EPI_ISL_2588064, EPI_ISL_2588068, EPI_ISL_2588069, EPI_ISL_2588070, EPI_ISL_2588071, EPI_ISL_2588073, EPI_ISL_2588083, EPI_ISL_2588087	Virology, Public Health Institution of Turkey	Virology, Public Health Institution of Turkey	Bayrakdar,F.
EPI_ISL_2588104	Departament of Microbiology, University of Sao Paulo Institute of Biomedical Science	Departament of Microbiology, University of Sao Paulo Institute of Biomedical Science	Espinola,E.E.
EPI_ISL_2588105, EPI_ISL_2588106	Michiko Okamoto Tohoku University Graduate School of Medicine, Virology	Michiko Okamoto Tohoku University Graduate School of Medicine, Virology	Okamoto,M., Malasaor,R. and Oshitani,H.
EPI_ISL_2588114, EPI_ISL_2588115, EPI_ISL_2588117, EPI_ISL_2588118, EPI_ISL_2588119, EPI_ISL_2588120, EPI_ISL_2588121, EPI_ISL_2588122, EPI_ISL_2588123, EPI_ISL_2588124, EPI_ISL_2588125, EPI_ISL_2588126, EPI_ISL_2588127			
see above	Microbiology, Hospital Universitari Vall d'Hebron	Microbiology, Hospital Universitari Vall d'Hebron	Gimferrer,L., Campins,M., Codina,M.G., Martin,M.D.C., Fuentes,F., Esperalba,J., Bruguera,A., Vilca,L.M., Armadans,L., Vall,A., Pumarola,T. and Anton,A.
EPI_ISL_2588132	Miwako SaikNorth America / USA Yokohama City Institute of Public Health	Miwako SaikNorth America / USA Yokohama City Institute of Public Health	SaikNorth America / USA,M., Kawakami,C., Usuku,S., Sasao,T. and Okubo,I.
EPI_ISL_2588143	Departament of Microbiology, University of Sao Paulo Institute of Biomedical Science	Departament of Microbiology, University of Sao Paulo Institute of Biomedical Science	Espinola,E.E.
EPI_ISL_2588154, EPI_ISL_2588155, EPI_ISL_2588156	Influenza and Other Respiratory Viruses Unit, National Center for Microbiology, Instituto de Salud Carlos III	Influenza and Other Respiratory Viruses Unit, National Center for Microbiology, Instituto de Salud Carlos III	Casas,I., Calderon,A., Gonzalez,M., Molinero,M. and Pozo,F.
EPI_ISL_2588157, EPI_ISL_2588158, EPI_ISL_2588159, EPI_ISL_2588160, EPI_ISL_2588161, EPI_ISL_2588163	Microbiology, Hospital Universitari Vall d'Hebron	Microbiology, Hospital Universitari Vall d'Hebron	Gimferrer,L., Campins,M., Codina,M.G., Martin,M.D.C., Fuentes,F., Esperalba,J., Bruguera,A., Vilca,L.M., Armadans,L., Vall,A., Pumarola,T. and Anton,A.
EPI_ISL_2588203, EPI_ISL_2588204, EPI_ISL_2588205, EPI_ISL_2588206, EPI_ISL_2588207, EPI_ISL_2588208, EPI_ISL_2588209, EPI_ISL_2588210	Miwako SaikNorth America / USA Yokohama City Institute of Public Health	Miwako SaikNorth America / USA Yokohama City Institute of Public Health	SaikNorth America / USA,M., Kawakami,C., Usuku,S., Sasao,T. and Okubo,I.
EPI_ISL_2588215, EPI_ISL_2588216	Michiko Okamoto Tohoku University Graduate School of Medicine, Virology	Michiko Okamoto Tohoku University Graduate School of Medicine, Virology	Okamoto,M., Malasaor,R. and Oshitani,H.
EPI_ISL_2588231, EPI_ISL_2588232, EPI_ISL_2588233, EPI_ISL_2588234, EPI_ISL_2588235, EPI_ISL_2588236, EPI_ISL_2588237, EPI_ISL_2588238, EPI_ISL_2588239, EPI_ISL_2588240, EPI_ISL_2588241, EPI_ISL_2588242, EPI_ISL_2588243, EPI_ISL_2588244, EPI_ISL_2588245, EPI_ISL_2588246, EPI_ISL_2588247, EPI_ISL_2588248, EPI_ISL_2588249, EPI_ISL_2588250, EPI_ISL_2588251, EPI_ISL_2588252, EPI_ISL_2588253, EPI_ISL_2588254			
see above	Influenza and Other Respiratory Viruses Unit, National Center for Microbiology, Instituto de Salud Carlos III	Influenza and Other Respiratory Viruses Unit, National Center for Microbiology, Instituto de Salud Carlos III	Casas,I., Calderon,A., Gonzalez,M., Molinero,M. and Pozo,F.
EPI_ISL_2588255, EPI_ISL_2588256, EPI_ISL_2588258, EPI_ISL_2588262, EPI_ISL_2588263, EPI_ISL_2588264, EPI_ISL_2588265, EPI_ISL_2588266	Laboratorio de Biologia Viral, Instituto de Salud Carlos III	Laboratorio de Biologia Viral, Instituto de Salud Carlos III	Trento,A., Abrego,L., Rodriguez-Fernandez,R., Gonzalez-Sanchez,M.I., Gonzalez-Martinez,F., Delfaro,A., Pascale,J.M., Arbiza,J. and Melero,J.A.
EPI_ISL_2588267, EPI_ISL_2588268, EPI_ISL_2588269, EPI_ISL_2588270, EPI_ISL_2588271, EPI_ISL_2588272, EPI_ISL_2588273, EPI_ISL_2588274, EPI_ISL_2588275, EPI_ISL_2588276, EPI_ISL_2588277, EPI_ISL_2588278, EPI_ISL_2588279, EPI_ISL_2588280, EPI_ISL_2588281, EPI_ISL_2588282, EPI_ISL_2588283, EPI_ISL_2588284, EPI_ISL_2588285, EPI_ISL_2588286, EPI_ISL_2588287, EPI_ISL_2588288, EPI_ISL_2588289			
see above	Microbiology, Hospital Universitari Vall d'Hebron	Microbiology, Hospital Universitari Vall d'Hebron	Gimferrer,L., Campins,M., Codina,M.G., Martin,M.D.C., Fuentes,F., Esperalba,J., Bruguera,A., Vilca,L.M., Armadans,L., Vall,A., Pumarola,T. and Anton,A.
EPI_ISL_2588290, EPI_ISL_2588292	J. Craig Venter Institute	J. Craig Venter Institute	Das,S., Halpin,R.A., Bera,J., Fedorova,N., Tsitrin,T., Stockwell,T., Amedeo,P., Bishop,B., Gupta,N., Hoover,J., Katzel,D., Schobel,S., Shrivastava,S., Hartert,T., Moore,M., Chappell,J., Larkin,E., Wentworth,D.E. and Anderson,L.J.
EPI_ISL_2588302, EPI_ISL_2588303	J. Craig Venter Institute	J. Craig Venter Institute	Das,S.R., Halpin,R.A., Shilts,M., Puri,V., Akopov,A., Fedorova,N., Stockwell,T., Amedeo,P., Bishop,B., Katzel,D., Schobel,S., Shrivastava,S. and Hartert,T.
EPI_ISL_2588304	J. Craig Venter Institute	J. Craig Venter Institute	Das,S., Halpin,R.A., Bera,J., Fedorova,N., Tsitrin,T., Stockwell,T., Amedeo,P., Bishop,B., Gupta,N., Hoover,J., Katzel,D., Schobel,S., Shrivastava,S., Hartert,T., Moore,M., Chappell,J., Larkin,E., Wentworth,D.E. and Anderson,L.J.
EPI_ISL_2588311, EPI_ISL_2588312, EPI_ISL_2588313, EPI_ISL_2588314, EPI_ISL_2588315, EPI_ISL_2588316, EPI_ISL_2588317, EPI_ISL_2588318, EPI_ISL_2588319, EPI_ISL_2588320, EPI_ISL_2588321, EPI_ISL_2588322, EPI_ISL_2588323, EPI_ISL_2588324, EPI_ISL_2588325, EPI_ISL_2588326, EPI_ISL_2588327, EPI_ISL_2588328, EPI_ISL_2588329, EPI_ISL_2588330, EPI_ISL_2588331, EPI_ISL_2588332, EPI_ISL_2588333, EPI_ISL_2588334, EPI_ISL_2588335, EPI_ISL_2588336, EPI_ISL_2588337			
see above	Miwako SaikNorth America / USA Yokohama City Institute of Public Health	Miwako SaikNorth America / USA Yokohama City Institute of Public Health	SaikNorth America / USA,M., Kawakami,C., Usuku,S., Sasao,T. and Okubo,I.
EPI_ISL_2588347, EPI_ISL_2588349, EPI_ISL_2588350, EPI_ISL_2588351	J. Craig Venter Institute	J. Craig Venter Institute	Das,S., Halpin,R.A., Bera,J., Fedorova,N., Tsitrin,T., Stockwell,T., Amedeo,P., Bishop,B., Gupta,N., Hoover,J., Katzel,D., Schobel,S., Shrivastava,S., Hartert,T., Moore,M., Chappell,J., Larkin,E., Wentworth,D.E. and Anderson,L.J.

EPI_ISL_2588352	J. Craig Venter Institute	J. Craig Venter Institute	Das,S., Halpin,R.A., Bera,J., Puri,V., Fedorova,N., Tsitrin,T., Stockwell,T., Amedeo,P., Bishop,B., Katzel,D., Schobel,S., Shrivastava,S., Hartert,T., Moore,M., Chappell,J., Larkin,E., Wentworth,D.E. and Anderson,L.J.
EPI_ISL_2588356	J. Craig Venter Institute	J. Craig Venter Institute	Shabman,R., Das,S.R., Shilts,M., Fedorova,N., Puri,V., Shrivastava,S., Amedeo,P., Williams,M., Barratt,K., Mitchell,J. and Jennings,L.
EPI_ISL_2588358, EPI_ISL_2588359, EPI_ISL_2588360, EPI_ISL_2588361, EPI_ISL_2588362	J. Craig Venter Institute	J. Craig Venter Institute	Das,S., Halpin,R.A., Bera,J., Fedorova,N., Tsitrin,T., Stockwell,T., Amedeo,P., Bishop,B., Gupta,N., Hoover,J., Katzel,D., Schobel,S., Shrivastava,S., Hartert,T., Moore,M., Chappell,J., Larkin,E., Wentworth,D.E. and Anderson,L.J.
EPI_ISL_2588366, EPI_ISL_2588368, EPI_ISL_2588369, EPI_ISL_2588370, EPI_ISL_2588371, EPI_ISL_2588374, EPI_ISL_2588375	J. Craig Venter Institute	J. Craig Venter Institute	Das,S.R., Halpin,R.A., Shilts,M., Puri,V., Akopov,A., Fedorova,N., Stockwell,T., Amedeo,P., Bishop,B., Katzel,D., Schobel,S., Shrivastava,S. and Hartert,T.
EPI_ISL_2588376	J. Craig Venter Institute	J. Craig Venter Institute	Shabman,R., Das,S.R., Puri,V., Fedorova,N., Amedeo,P., Williams,M., Shrivastava,S. and Halasa,N.
EPI_ISL_2588377, EPI_ISL_2588378, EPI_ISL_2588380	J. Craig Venter Institute	J. Craig Venter Institute	Das,S.R., Halpin,R.A., Shilts,M., Puri,V., Akopov,A., Fedorova,N., Stockwell,T., Amedeo,P., Bishop,B., Katzel,D., Schobel,S., Shrivastava,S. and Hartert,T.
EPI_ISL_2588386, EPI_ISL_2588387, EPI_ISL_2588388, EPI_ISL_2588389, EPI_ISL_2588390, EPI_ISL_2588391, EPI_ISL_2588392, EPI_ISL_2588393, EPI_ISL_2588394, EPI_ISL_2588395, EPI_ISL_2588396, EPI_ISL_2588397, EPI_ISL_2588398, EPI_ISL_2588399, EPI_ISL_2588400, EPI_ISL_2588401, EPI_ISL_2588402, EPI_ISL_2588403, EPI_ISL_2588404, EPI_ISL_2588405, EPI_ISL_2588406, EPI_ISL_2588407, EPI_ISL_2588408, EPI_ISL_2588409, EPI_ISL_2588410, EPI_ISL_2588411, EPI_ISL_2588412, EPI_ISL_2588413, EPI_ISL_2588414, EPI_ISL_2588415, EPI_ISL_2588416, EPI_ISL_2588417, EPI_ISL_2588418, EPI_ISL_2588419, EPI_ISL_2588420			
see above	Miwako SaikNorth America / USA Yokohama City Institute of Public Health	Miwako SaikNorth America / USA Yokohama City Institute of Public Health	SaikNorth America / USA,M., Kawakami,C., Usuku,S., Sasao,T. and Okubo,I.
EPI_ISL_2588452, EPI_ISL_2588456	Michiko Okamoto Tohoku University Graduate School of Medicine, Virology	Michiko Okamoto Tohoku University Graduate School of Medicine, Virology	Okamoto,M., Malasaor,R. and Oshitani,H.
EPI_ISL_2588521, EPI_ISL_2588522	Influenza and Other Respiratory Viruses Unit, National Center for Microbiology, Instituto de Salud Carlos III	Influenza and Other Respiratory Viruses Unit, National Center for Microbiology, Instituto de Salud Carlos III	Casas,I., Calderon,A., Gonzalez,M., Molinero,M. and Pozo,F.
EPI_ISL_2588523	Laboratorio de Biologia Viral, Instituto de Salud Carlos III	Laboratorio de Biologia Viral, Instituto de Salud Carlos III	Trento,A., Abrego,L., Rodriguez-Fernandez,R., Gonzalez-Sanchez,M.I., Gonzalez-Martinez,F., Delfaro,A., Pascale,J.M., Arbiza,J. and Melero,J.A.
EPI_ISL_2588524, EPI_ISL_2588525, EPI_ISL_2588526, EPI_ISL_2588527	Microbiology, Hospital Universitari Vall d'Hebron	Microbiology, Hospital Universitari Vall d'Hebron	Gimferrer,L., Campins,M., Codina,M.G., Martin,M.D.C., Fuentes,F., Esperalba,J., Bruguera,A., Vilca,L.M., Armadans,L., Vall,A., Pumarola,T. and Anton,A.
EPI_ISL_2588552, EPI_ISL_2588553, EPI_ISL_2588555, EPI_ISL_2588556, EPI_ISL_2588559, EPI_ISL_2588560, EPI_ISL_2588561, EPI_ISL_2588562, EPI_ISL_2588567, EPI_ISL_2588569, EPI_ISL_2588570, EPI_ISL_2588571, EPI_ISL_2588572, EPI_ISL_2588573, EPI_ISL_2588574, EPI_ISL_2588580, EPI_ISL_2588582, EPI_ISL_2588583, EPI_ISL_2588584, EPI_ISL_2588585, EPI_ISL_2588586, EPI_ISL_2588587, EPI_ISL_2588591			
see above	Virology, Public Health Institution of Turkey	Virology, Public Health Institution of Turkey	Bayrakdar,F.
EPI_ISL_2588597	J. Craig Venter Institute	J. Craig Venter Institute	Das,S., Halpin,R.A., Bera,J., Fedorova,N., Tsitrin,T., Stockwell,T., Amedeo,P., Bishop,B., Gupta,N., Hoover,J., Katzel,D., Schobel,S., Shrivastava,S., Hartert,T., Moore,M., Chappell,J., Larkin,E., Wentworth,D.E. and Anderson,L.J.
EPI_ISL_2588600	J. Craig Venter Institute	J. Craig Venter Institute	Das,S.R., Halpin,R.A., Shilts,M., Puri,V., Akopov,A., Fedorova,N., Stockwell,T., Amedeo,P., Bishop,B., Katzel,D., Schobel,S., Shrivastava,S. and Hartert,T.
EPI_ISL_2588796	Virology Laboratory, Dr. Ricardo Gutierrez Children Hospital	Virology Laboratory, Dr. Ricardo Gutierrez Children Hospital	Goya,S., Valinotto,L.E., Tittarelli,E., Rojo,G.L., Greninger,A., Luso,S., Natale,M., Mistchenko,A.S. and Viegas,M.
EPI_ISL_2588798, EPI_ISL_2588799, EPI_ISL_2588800	Research & Development, Institute of Immunology	Research & Development, Institute of Immunology	Ivancic-Jelecki,J., Forcic,D., Mlinaric-Galinovic,G. and Tesovic,G.
EPI_ISL_2588818	Virology Laboratory, Dr. Ricardo Gutierrez Children Hospital	Virology Laboratory, Dr. Ricardo Gutierrez Children Hospital	Goya,S., Valinotto,L.E., Tittarelli,E., Rojo,G.L., Greninger,A., Luso,S., Natale,M., Mistchenko,A.S. and Viegas,M.
EPI_ISL_2588820, EPI_ISL_2588826	Translational Medicine Institute, Affiliated the First People's Hospital of Chenzhou, University of South China	Translational Medicine Institute, Affiliated the First People's Hospital of Chenzhou, University of South China	Fan,R.Y., Qu,X.W. and Fan,C.P.
EPI_ISL_2588828	Virology Laboratory, Dr. Ricardo Gutierrez Children Hospital	Virology Laboratory, Dr. Ricardo Gutierrez Children Hospital	Goya,S., Rojo,G.L., Valinotto,L.E., Mistchenko,A.S. and Viegas,M.
EPI_ISL_2588864	Microbiology Department, Virology Division, College of Medicine, Taif University, Virology Department, Faculty of Veterinary Medicine	Microbiology Department, Virology Division, College of Medicine, Taif University, Virology Department, Faculty of Veterinary Medicine	Abdel-Moneim,A.S., Soliman,M.S., Kamel,M.M. and El-Kholy,A.A.
EPI_ISL_2588865, EPI_ISL_2588866, EPI_ISL_2588867, EPI_ISL_2588868, EPI_ISL_2588869	University of Zagreb, Centre for research and knowledge transfer in biotechnology	University of Zagreb, Centre for research and knowledge transfer in biotechnology	Ivancic-Jelecki,J., Forcic,D., Mlinaric-Galinovic,G. and Tesovic,G.
EPI_ISL_2588958	Microbiology Laboratory, Hangzhou Center for Disease Control and Prevention	Microbiology Laboratory, Hangzhou Center for Disease Control and Prevention	Yu,X., Kou,Y. and Li,J.
EPI_ISL_2588959	Laboratory for Molecular Biomedicine, Centre for Research and Knowledge Transfer in Biotechnology, University of Zagreb	Laboratory for Molecular Biomedicine, Centre for Research and Knowledge Transfer in Biotechnology, University of Zagreb	Slovic,A., Forcic,D., Ivancic-Jelecki,J., Ljubin Sternak,S. and Mlinaric-Galinovic,G.
EPI_ISL_2589050	Microbiology Laboratory, Hangzhou Center for Disease Control and Prevention	Microbiology Laboratory, Hangzhou Center for Disease Control and Prevention	Yu,X., Kou,Y. and Li,J.
EPI_ISL_2589072	Department for Infectious Diseases Virology, University of Heidelberg	Department for Infectious Diseases Virology, University of Heidelberg	Tabatabai,J., Prifert,C., Pfeil,J., Grulich-Henn,J. and Schnitzler,P.
EPI_ISL_2589162, EPI_ISL_2589163, EPI_ISL_2589164	Microbiology Laboratory, Hangzhou Center for Disease Control and Prevention	Microbiology Laboratory, Hangzhou Center for Disease Control and Prevention	Yu,X., Kou,Y. and Li,J.
EPI_ISL_2589168, EPI_ISL_2589169	Laboratory for Molecular Biomedicine, Centre for Research and Knowledge Transfer in Biotechnology, University of Zagreb	Laboratory for Molecular Biomedicine, Centre for Research and Knowledge Transfer in Biotechnology, University of Zagreb	Slovic,A., Forcic,D., Ivancic-Jelecki,J., Ljubin Sternak,S. and Mlinaric-Galinovic,G.
EPI_ISL_2589231, EPI_ISL_2589232	Microbiology Laboratory, Hangzhou Center for Disease Control and Prevention	Microbiology Laboratory, Hangzhou Center for Disease Control and Prevention	Yu,X., Kou,Y. and Li,J.
EPI_ISL_2589241	Laboratory for Molecular Biomedicine, Centre for Research and Knowledge Transfer in Biotechnology, University of Zagreb	Laboratory for Molecular Biomedicine, Centre for Research and Knowledge Transfer in Biotechnology, University of Zagreb	Slovic,A., Forcic,D., Ivancic-Jelecki,J., Ljubin Sternak,S. and Mlinaric-Galinovic,G.
EPI_ISL_2589256	Department for Infectious Diseases Virology, University of Heidelberg	Department for Infectious Diseases Virology, University of Heidelberg	Tabatabai,J., Prifert,C., Pfeil,J., Grulich-Henn,J. and Schnitzler,P.
EPI_ISL_2589288, EPI_ISL_2589289, EPI_ISL_2589290, EPI_ISL_2589291, EPI_ISL_2589292, EPI_ISL_2589293	Microbiology Laboratory, Hangzhou Center for Disease Control and Prevention	Microbiology Laboratory, Hangzhou Center for Disease Control and Prevention	Yu,X., Kou,Y. and Li,J.
EPI_ISL_2589304	University of Zagreb, Centre for research and knowledge transfer in biotechnology	University of Zagreb, Centre for research and knowledge transfer in biotechnology	Ivancic-Jelecki,J., Forcic,D., Mlinaric-Galinovic,G. and Tesovic,G.
EPI_ISL_2589315	Laboratory for Molecular Biomedicine, Centre for Research and Knowledge Transfer in Biotechnology, University of Zagreb	Laboratory for Molecular Biomedicine, Centre for Research and Knowledge Transfer in Biotechnology, University of Zagreb	Slovic,A., Forcic,D., Ivancic-Jelecki,J., Ljubin Sternak,S. and Mlinaric-Galinovic,G.
EPI_ISL_2589333	Department for Infectious Diseases Virology, University of Heidelberg	Department for Infectious Diseases Virology, University of Heidelberg	Tabatabai,J., Prifert,C., Pfeil,J., Grulich-Henn,J. and Schnitzler,P.
EPI_ISL_2589340, EPI_ISL_2589341, EPI_ISL_2589342, EPI_ISL_2589343, EPI_ISL_2589344	Microbiology Laboratory, Hangzhou Center for Disease Control and Prevention	Microbiology Laboratory, Hangzhou Center for Disease Control and Prevention	Yu,X., Kou,Y. and Li,J.
EPI_ISL_2589348	Laboratory for Molecular Biomedicine, Centre for Research and Knowledge Transfer in Biotechnology, University of Zagreb	Laboratory for Molecular Biomedicine, Centre for Research and Knowledge Transfer in Biotechnology, University of Zagreb	Slovic,A., Forcic,D., Ivancic-Jelecki,J., Ljubin Sternak,S. and Mlinaric-Galinovic,G.
EPI_ISL_2589380, EPI_ISL_2589381	Microbiology Laboratory, Hangzhou Center for Disease Control and Prevention	Microbiology Laboratory, Hangzhou Center for Disease Control and Prevention	Yu,X., Kou,Y. and Li,J.
EPI_ISL_2589382	Laboratory for Molecular Biomedicine, Centre for Research and Knowledge Transfer in Biotechnology, University of Zagreb	Laboratory for Molecular Biomedicine, Centre for Research and Knowledge Transfer in Biotechnology, University of Zagreb	Slovic,A., Forcic,D., Ivancic-Jelecki,J., Ljubin Sternak,S. and Mlinaric-Galinovic,G.
EPI_ISL_2589385, EPI_ISL_2589386	Department for Infectious Diseases Virology, University of Heidelberg	Department for Infectious Diseases Virology, University of Heidelberg	Tabatabai,J., Prifert,C., Pfeil,J., Grulich-Henn,J. and Schnitzler,P.
EPI_ISL_2589388, EPI_ISL_2589389	Laboratory for Molecular Biomedicine, Centre for Research and Knowledge Transfer in Biotechnology, University of Zagreb	Laboratory for Molecular Biomedicine, Centre for Research and Knowledge Transfer in Biotechnology, University of Zagreb	Slovic,A., Forcic,D., Ivancic-Jelecki,J., Ljubin Sternak,S. and Mlinaric-Galinovic,G.
EPI_ISL_2589390, EPI_ISL_2589391, EPI_ISL_2589392, EPI_ISL_2589393, EPI_ISL_2589432	Department for Infectious Diseases Virology, University of Heidelberg	Department for Infectious Diseases Virology, University of Heidelberg	Tabatabai,J., Prifert,C., Pfeil,J., Grulich-Henn,J. and Schnitzler,P.
EPI_ISL_2589449, EPI_ISL_2589450, EPI_ISL_2589452, EPI_ISL_2589453, EPI_ISL_2589454, EPI_ISL_2589455	Dept of Biomedical Sciences and Public Health, Virology Unit, Marche Politechnic University Medical School	Dept of Biomedical Sciences and Public Health, Virology Unit, Marche Politechnic University Medical School	Bagnarelli,P., Trotta,D., Ferreri,M.L. and Pierangeli,A.
EPI_ISL_2589463	Department for Infectious Diseases Virology, University of Heidelberg	Department for Infectious Diseases Virology, University of Heidelberg	Tabatabai,J., Prifert,C., Pfeil,J., Grulich-Henn,J. and Schnitzler,P.
EPI_ISL_2589464	Department for Infectious Diseases Virology, University of Heidelberg	Department for Infectious Diseases Virology, University of Heidelberg	Tabatabai,J., Lehners,N., Prifert,C., Wedde,M., Puthenparambil,J., Weissbrich,B., Schweiger,B., Egerer,G. and Schnitzler,P.
EPI_ISL_2589471	Dept of Biomedical Sciences and Public Health, Virology Unit, Marche Politechnic University Medical School	Dept of Biomedical Sciences and Public Health, Virology Unit, Marche Politechnic University Medical School	Bagnarelli,P., Trotta,D., Ferreri,M.L. and Pierangeli,A.
EPI_ISL_2589483	Department for Infectious Diseases Virology, University of Heidelberg	Department for Infectious Diseases Virology, University of Heidelberg	Tabatabai,J., Prifert,C., Pfeil,J., Grulich-Henn,J. and Schnitzler,P.
EPI_ISL_2589484, EPI_ISL_2589499, EPI_ISL_2589500, EPI_ISL_2589501	Department for Infectious Diseases Virology, University of Heidelberg	Department for Infectious Diseases Virology, University of Heidelberg	Tabatabai,J., Lehners,N., Prifert,C., Wedde,M., Puthenparambil,J., Weissbrich,B., Schweiger,B., Egerer,G. and Schnitzler,P.
EPI_ISL_2589844	Virology Laboratory, Dr. Ricardo Gutierrez Children Hospital	Virology Laboratory, Dr. Ricardo Gutierrez Children Hospital	Viegas,M., Goya,S. and Mistchenko,A.S.
EPI_ISL_2589885, EPI_ISL_2589887, EPI_ISL_2589890, EPI_ISL_2589894, EPI_ISL_2589898, EPI_ISL_2589901, EPI_ISL_2589903, EPI_ISL_2589905, EPI_ISL_2589907, EPI_ISL_2589914	Translational Medicine Institute, Affiliated the First People's Hospital of Chenzhou, University of South China	Translational Medicine Institute, Affiliated the First People's Hospital of Chenzhou, University of South China	Fan,R.Y., Qu,X.W. and Fan,C.P.
EPI_ISL_2589952, EPI_ISL_2589958, EPI_ISL_2589960, EPI_ISL_2589962, EPI_ISL_2589963, EPI_ISL_2589965, EPI_ISL_2589967, EPI_ISL_2589969, EPI_ISL_2589971	Microbiology Department, Virology Division, College of Medicine, Taif University, Virology Department, Faculty of Veterinary Medicine	Microbiology Department, Virology Division, College of Medicine, Taif University, Virology Department, Faculty of Veterinary Medicine	Abdel-Moneim,A.S., Soliman,M.S., Kamel,M.M. and El-Kholy,A.A.
EPI_ISL_2589982	Department for Infectious Diseases Virology, University of Heidelberg	Department for Infectious Diseases Virology, University of Heidelberg	Tabatabai,J., Prifert,C., Pfeil,J., Grulich-Henn,J. and Schnitzler,P.
EPI_ISL_2590993	Dept of Biomedical Sciences and Public Health, Virology Unit, Marche Politechnic University Medical School	Dept of Biomedical Sciences and Public Health, Virology Unit, Marche Politechnic University Medical School	Bagnarelli,P., Trotta,D., Ferreri,M.L. and Pierangeli,A.

EPI_ISL_2590995	Medical Virology, Faculty of Public Health, Tehran University of Medical Sciences	Medical Virology, Faculty of Public Health, Tehran University of Medical Sciences	Arjeyni,Y., Faghihloo,E. and Mokhtari Azad,T.
EPI_ISL_2591016, EPI_ISL_2591048	Dept of Biomedical Sciences and Public Health, Virology Unit, Marche Politechnic University Medical School	Dept of Biomedical Sciences and Public Health, Virology Unit, Marche Politechnic University Medical School	Bagnarelli,P., Trotta,D., Ferreri,M.L. and Pierangeli,A.
EPI_ISL_2591093	Laboratory for Molecular Biomedicine, Centre for Research and Knowledge Transfer in Biotechnology, University of Zagreb	Laboratory for Molecular Biomedicine, Centre for Research and Knowledge Transfer in Biotechnology, University of Zagreb	Slovic,A., Forcic,D., Ivancic-Jelecki,J., Ljubin Sternak,S. and Mlinaric-Galinovic,G.
EPI_ISL_2591109	Microbiology Laboratory, Hangzhou Center for Disease Control and Prevention	Microbiology Laboratory, Hangzhou Center for Disease Control and Prevention	Yu,X., Kou,Y. and Li,J.
EPI_ISL_2591159, EPI_ISL_2591161	Laboratory for Molecular Biomedicine, Centre for Research and Knowledge Transfer in Biotechnology, University of Zagreb	Laboratory for Molecular Biomedicine, Centre for Research and Knowledge Transfer in Biotechnology, University of Zagreb	Slovic,A., Forcic,D., Ivancic-Jelecki,J., Ljubin Sternak,S. and Mlinaric-Galinovic,G.
EPI_ISL_2591163, EPI_ISL_2591165	Dept of Biomedical Sciences and Public Health, Virology Unit, Marche Politechnic University Medical School	Dept of Biomedical Sciences and Public Health, Virology Unit, Marche Politechnic University Medical School	Bagnarelli,P., Trotta,D., Ferreri,M.L. and Pierangeli,A.
EPI_ISL_2591167, EPI_ISL_2591168	Department for Infectious Diseases Virology, University of Heidelberg	Department for Infectious Diseases Virology, University of Heidelberg	Tabatabai,J., Prifert,C., Pfeil,J., Grulich-Henn,J. and Schnitzler,P.
EPI_ISL_2591186	Virology, School of Public Health, Tehran University of Medical Sciences	Virology, School of Public Health, Tehran University of Medical Sciences	Yavarian,J., Faghihloo,E. and Mokhtari Azad,T.
EPI_ISL_2591189	Virology Laboratory, Dr. Ricardo Gutierrez Children Hospital	Virology Laboratory, Dr. Ricardo Gutierrez Children Hospital	Viegas,M., Goya,S. and Mistchenko,A.S.
EPI_ISL_2591197	Virology Laborotary, Beijing Pediatric Research Institute, Beijing Children's Hospital, Capital Medical University, National Center for Children's Health	Virology Laborotary, Beijing Pediatric Research Institute, Beijing Children's Hospital, Capital Medical University, National Center for Children's Health	Chen,X., Guo,J. and Xie,Z.
EPI_ISL_2591222	Department for Infectious Diseases Virology, University of Heidelberg	Department for Infectious Diseases Virology, University of Heidelberg	Tabatabai,J., Prifert,C., Pfeil,J., Grulich-Henn,J. and Schnitzler,P.
EPI_ISL_2591437, EPI_ISL_2591439	Dept of Biomedical Sciences and Public Health, Virology Unit, Marche Politechnic University Medical School	Dept of Biomedical Sciences and Public Health, Virology Unit, Marche Politechnic University Medical School	Bagnarelli,P., Trotta,D., Ferreri,M.L. and Pierangeli,A.
EPI_ISL_2591441	Microbiology Department, Virology Division, College of Medicine, Taif University, Virology Department, Faculty of Veterinary Medicine	Microbiology Department, Virology Division, College of Medicine, Taif University, Virology Department, Faculty of Veterinary Medicine	Abdel-Moneim,A.S., Soliman,M.S., Kamel,M.M. and El-Kholy,A.A.
EPI_ISL_2591454, EPI_ISL_2591456, EPI_ISL_2591458	Virology Laboratory, Dr. Ricardo Gutierrez Children Hospital	Virology Laboratory, Dr. Ricardo Gutierrez Children Hospital	Viegas,M., Goya,S. and Mistchenko,A.S.
EPI_ISL_2591460, EPI_ISL_2591462	Microbiology Laboratory, Hangzhou Center for Disease Control and Prevention	Microbiology Laboratory, Hangzhou Center for Disease Control and Prevention	Yu,X., Kou,Y. and Li,J.
EPI_ISL_2591464, EPI_ISL_2591466, EPI_ISL_2591468, EPI_ISL_2591470, EPI_ISL_2591472	Laboratory for Molecular Biomedicine, Centre for Research and Knowledge Transfer in Biotechnology, University of Zagreb	Laboratory for Molecular Biomedicine, Centre for Research and Knowledge Transfer in Biotechnology, University of Zagreb	Slovic,A., Forcic,D., Ivancic-Jelecki,J., Ljubin Sternak,S. and Mlinaric-Galinovic,G.
EPI_ISL_2591508	Microbiology Department, Virology Division, College of Medicine, Taif University, Virology Department, Faculty of Veterinary Medicine	Microbiology Department, Virology Division, College of Medicine, Taif University, Virology Department, Faculty of Veterinary Medicine	Abdel-Moneim,A.S., Soliman,M.S., Kamel,M.M. and El-Kholy,A.A.
EPI_ISL_2591523	Virology Laboratory, Dr. Ricardo Gutierrez Children Hospital	Virology Laboratory, Dr. Ricardo Gutierrez Children Hospital	Viegas,M., Goya,S. and Mistchenko,A.S.
EPI_ISL_2591525, EPI_ISL_2591527	Department for Infectious Diseases Virology, University of Heidelberg	Department for Infectious Diseases Virology, University of Heidelberg	Tabatabai,J., Prifert,C., Pfeil,J., Grulich-Henn,J. and Schnitzler,P.
EPI_ISL_2591529	Department for Infectious Diseases Virology, University of Heidelberg	Department for Infectious Diseases Virology, University of Heidelberg	Tabatabai,J., Lehners,N., Prifert,C., Wedde,M., Puthenparambil,J., Weissbrich,B., Schweiger,B., Egerer,G. and Schnitzler,P.
EPI_ISL_2591539	Dept of Biomedical Sciences and Public Health, Virology Unit, Marche Politechnic University Medical School	Dept of Biomedical Sciences and Public Health, Virology Unit, Marche Politechnic University Medical School	Bagnarelli,P., Trotta,D., Ferreri,M.L. and Pierangeli,A.
EPI_ISL_2591547	Translational Medicine Institute, Affiliated the First People's Hospital of Chenzhou, University of South China	Translational Medicine Institute, Affiliated the First People's Hospital of Chenzhou, University of South China	Fan,R.Y., Qu,X.W. and Fan,C.P.
EPI_ISL_2591549, EPI_ISL_2591550	Virology Laborotary, Beijing Pediatric Research Institute, Beijing Children's Hospital, Capital Medical University, National Center for Children's Health	Virology Laborotary, Beijing Pediatric Research Institute, Beijing Children's Hospital, Capital Medical University, National Center for Children's Health	Chen,X., Guo,J. and Xie,Z.
EPI_ISL_2591629	Virology, School of Public Health, Tehran University of Medical Sciences	Virology, School of Public Health, Tehran University of Medical Sciences	Yavarian,J., Faghihloo,E. and Mokhtari Azad,T.
EPI_ISL_2591691	Translational Medicine Institute, Affiliated the First People's Hospital of Chenzhou, University of South China	Translational Medicine Institute, Affiliated the First People's Hospital of Chenzhou, University of South China	Fan,R.Y., Qu,X.W. and Fan,C.P.
EPI_ISL_2591692, EPI_ISL_2591693, EPI_ISL_2591694, EPI_ISL_2591695, EPI_ISL_2591696, EPI_ISL_2591697	Microbiology Department, Virology Division, College of Medicine, Taif University, Virology Department, Faculty of Veterinary Medicine	Microbiology Department, Virology Division, College of Medicine, Taif University, Virology Department, Faculty of Veterinary Medicine	Abdel-Moneim,A.S., Soliman,M.S., Kamel,M.M. and El-Kholy,A.A.
EPI_ISL_2591698, EPI_ISL_2591699, EPI_ISL_2591700, EPI_ISL_2591719, EPI_ISL_2591720, EPI_ISL_2591721	Virology Laboratory, Dr. Ricardo Gutierrez Children Hospital	Virology Laboratory, Dr. Ricardo Gutierrez Children Hospital	Viegas,M., Goya,S. and Mistchenko,A.S.
EPI_ISL_2591722, EPI_ISL_2591723, EPI_ISL_2591724	Microbiology Laboratory, Hangzhou Center for Disease Control and Prevention	Microbiology Laboratory, Hangzhou Center for Disease Control and Prevention	Yu,X., Kou,Y. and Li,J.
EPI_ISL_2591725, EPI_ISL_2591726, EPI_ISL_2591727	Laboratory for Molecular Biomedicine, Centre for Research and Knowledge Transfer in Biotechnology, University of Zagreb	Laboratory for Molecular Biomedicine, Centre for Research and Knowledge Transfer in Biotechnology, University of Zagreb	Slovic,A., Forcic,D., Ivancic-Jelecki,J., Ljubin Sternak,S. and Mlinaric-Galinovic,G.
EPI_ISL_2591728	Centre for Research And Knowledge Transfer in Biotechnology, University of Zagreb	Centre for Research And Knowledge Transfer in Biotechnology, University of Zagreb	Slovic,A., Ljubin-Sternak,S., Mlinaric-Galinovic,G., Ivancic-Jelecki,J. and Forcic,D.
EPI_ISL_2591729, EPI_ISL_2591730, EPI_ISL_2591731, EPI_ISL_2591732	Laboratory for Molecular Biomedicine, Centre for Research and Knowledge Transfer in Biotechnology, University of Zagreb	Laboratory for Molecular Biomedicine, Centre for Research and Knowledge Transfer in Biotechnology, University of Zagreb	Slovic,A., Forcic,D., Ivancic-Jelecki,J., Ljubin Sternak,S. and Mlinaric-Galinovic,G.
EPI_ISL_2591733	Virology Laborotary, Beijing Pediatric Research Institute, Beijing Children's Hospital, Capital Medical University, National Center for Children's Health	Virology Laborotary, Beijing Pediatric Research Institute, Beijing Children's Hospital, Capital Medical University, National Center for Children's Health	Chen,X., Guo,J. and Xie,Z.
EPI_ISL_2591739, EPI_ISL_2591740, EPI_ISL_2591741, EPI_ISL_2591742, EPI_ISL_2591743, EPI_ISL_2591744, EPI_ISL_2591745, EPI_ISL_2591746	Department for Infectious Diseases Virology, University of Heidelberg	Department for Infectious Diseases Virology, University of Heidelberg	Tabatabai,J., Prifert,C., Pfeil,J., Grulich-Henn,J. and Schnitzler,P.
EPI_ISL_2591766, EPI_ISL_2591767	Translational Medicine Institute, Affiliated the First People's Hospital of Chenzhou, University of South China	Translational Medicine Institute, Affiliated the First People's Hospital of Chenzhou, University of South China	Fan,R.Y., Qu,X.W. and Fan,C.P.
EPI_ISL_2591769, EPI_ISL_2591770, EPI_ISL_2591771, EPI_ISL_2591772, EPI_ISL_2591773, EPI_ISL_2591774, EPI_ISL_2591775	Department for Infectious Diseases Virology, University of Heidelberg	Department for Infectious Diseases Virology, University of Heidelberg	Tabatabai,J., Prifert,C., Pfeil,J., Grulich-Henn,J. and Schnitzler,P.
EPI_ISL_2591776, EPI_ISL_2591777, EPI_ISL_2591778, EPI_ISL_2591779, EPI_ISL_2591780, EPI_ISL_2591781, EPI_ISL_2591782, EPI_ISL_2591783	Department for Infectious Diseases Virology, University of Heidelberg	Department for Infectious Diseases Virology, University of Heidelberg	Tabatabai,J., Lehners,N., Prifert,C., Wedde,M., Puthenparambil,J., Weissbrich,B., Schweiger,B., Egerer,G. and Schnitzler,P.
EPI_ISL_2591786, EPI_ISL_2591787, EPI_ISL_2591788	Translational Medicine Institute, Affiliated the First People's Hospital of Chenzhou, University of South China	Translational Medicine Institute, Affiliated the First People's Hospital of Chenzhou, University of South China	Fan,R.Y., Qu,X.W. and Fan,C.P.
EPI_ISL_2591792, EPI_ISL_2591793, EPI_ISL_2591794, EPI_ISL_2591795, EPI_ISL_2591796, EPI_ISL_2591797, EPI_ISL_2591798, EPI_ISL_2591799, EPI_ISL_2591800, EPI_ISL_2591801, EPI_ISL_2591802, EPI_ISL_2591803, EPI_ISL_2591804, EPI_ISL_2591805, EPI_ISL_2591806, EPI_ISL_2591807, EPI_ISL_2591808, EPI_ISL_2591809, EPI_ISL_2591810			
see above	Dept of Biomedical Sciences and Public Health, Virology Unit, Marche Politechnic University Medical School	Dept of Biomedical Sciences and Public Health, Virology Unit, Marche Politechnic University Medical School	Bagnarelli,P., Trotta,D., Ferreri,M.L. and Pierangeli,A.
EPI_ISL_2591811, EPI_ISL_2591812	Microbiology Department, Virology Division, College of Medicine, Taif University, Virology Department, Faculty of Veterinary Medicine	Microbiology Department, Virology Division, College of Medicine, Taif University, Virology Department, Faculty of Veterinary Medicine	Abdel-Moneim,A.S., Soliman,M.S., Kamel,M.M. and El-Kholy,A.A.
EPI_ISL_2591814	Translational Medicine Institute, Affiliated the First People's Hospital of Chenzhou, University of South China	Translational Medicine Institute, Affiliated the First People's Hospital of Chenzhou, University of South China	Fan,R.Y., Qu,X.W. and Fan,C.P.
EPI_ISL_2591816, EPI_ISL_2591817, EPI_ISL_2591818	Virology Laborotary, Beijing Pediatric Research Institute, Beijing Children's Hospital, Capital Medical University, National Center for Children's Health	Virology Laborotary, Beijing Pediatric Research Institute, Beijing Children's Hospital, Capital Medical University, National Center for Children's Health	Chen,X., Guo,J. and Xie,Z.
EPI_ISL_2591839, EPI_ISL_2591840, EPI_ISL_2591841, EPI_ISL_2591842, EPI_ISL_2591843, EPI_ISL_2591844, EPI_ISL_2591845, EPI_ISL_2591846, EPI_ISL_2591847, EPI_ISL_2591848, EPI_ISL_2591849, EPI_ISL_2591850, EPI_ISL_2591851, EPI_ISL_2591852, EPI_ISL_2591853, EPI_ISL_2591854, EPI_ISL_2591855, EPI_ISL_2591856, EPI_ISL_2591857, EPI_ISL_2591858, EPI_ISL_2591859, EPI_ISL_2591860, EPI_ISL_2591861			
see above	Microbiology Department, Virology Division, College of Medicine, Taif University, Virology Department, Faculty of Veterinary Medicine	Microbiology Department, Virology Division, College of Medicine, Taif University, Virology Department, Faculty of Veterinary Medicine	Abdel-Moneim,A.S., Soliman,M.S., Kamel,M.M. and El-Kholy,A.A.
EPI_ISL_2591863, EPI_ISL_2591864, EPI_ISL_2591865, EPI_ISL_2591866, EPI_ISL_2591867, EPI_ISL_2591868, EPI_ISL_2591869, EPI_ISL_2591870, EPI_ISL_2591871, EPI_ISL_2591872	Virology Laboratory, Dr. Ricardo Gutierrez Children Hospital	Virology Laboratory, Dr. Ricardo Gutierrez Children Hospital	Viegas,M., Goya,S. and Mistchenko,A.S.
EPI_ISL_2591874, EPI_ISL_2591880, EPI_ISL_2591881, EPI_ISL_2591882, EPI_ISL_2591883, EPI_ISL_2591884, EPI_ISL_2591885, EPI_ISL_2591886, EPI_ISL_2591887, EPI_ISL_2591888, EPI_ISL_2591889, EPI_ISL_2591890, EPI_ISL_2591891, EPI_ISL_2591892			
see above	Virology Laborotary, Beijing Pediatric Research Institute, Beijing Children's Hospital, Capital Medical University, National Center for Children's Health	Virology Laborotary, Beijing Pediatric Research Institute, Beijing Children's Hospital, Capital Medical University, National Center for Children's Health	Chen,X., Guo,J. and Xie,Z.
EPI_ISL_2591902	Microbiology Department, Virology Division, College of Medicine, Taif University, Virology Department, Faculty of Veterinary Medicine	Microbiology Department, Virology Division, College of Medicine, Taif University, Virology Department, Faculty of Veterinary Medicine	Abdel-Moneim,A.S., Soliman,M.S., Kamel,M.M. and El-Kholy,A.A.
EPI_ISL_2591909, EPI_ISL_2591910, EPI_ISL_2591911, EPI_ISL_2591912, EPI_ISL_2591913, EPI_ISL_2591914, EPI_ISL_2591916, EPI_ISL_2591917, EPI_ISL_2591918, EPI_ISL_2591920, EPI_ISL_2591941, EPI_ISL_2591942, EPI_ISL_2591943, EPI_ISL_2591944, EPI_ISL_2591945, EPI_ISL_2591946, EPI_ISL_2591948, EPI_ISL_2591949, EPI_ISL_2591950, EPI_ISL_2591951, EPI_ISL_2591952, EPI_ISL_2591953, EPI_ISL_2591954, EPI_ISL_2591955, EPI_ISL_2591956, EPI_ISL_2591957, EPI_ISL_2591958, EPI_ISL_2591959, EPI_ISL_2591960, EPI_ISL_2591961, EPI_ISL_2591962, EPI_ISL_2591963, EPI_ISL_2591964, EPI_ISL_2591965, EPI_ISL_2591966, EPI_ISL_2591967, EPI_ISL_2591968, EPI_ISL_2591969, EPI_ISL_2591970, EPI_ISL_2591971, EPI_ISL_2591972, EPI_ISL_2591973			

see above	Virology Laborotary, Beijing Pediatric Research Institute, Beijing Children's Hospital, Capital Medical University, National Center for Children's Health	Virology Laborotary, Beijing Pediatric Research Institute, Beijing Children's Hospital, Capital Medical University, National Center for Children's Health	Chen,X., Guo,J. and Xie,Z.
EPI_ISL_2591977, EPI_ISL_2591978, EPI_ISL_2591979, EPI_ISL_2591980, EPI_ISL_2591981, EPI_ISL_2591982, EPI_ISL_2591983	Miwako SaikNorth America / USA Yokohama City Institute of Public Health	Miwako SaikNorth America / USA Yokohama City Institute of Public Health	SaikNorth America / USA,M., Kawakami,C., Usuku,S., Sasao,T. and Okubo,I.
EPI_ISL_2592009, EPI_ISL_2592010, EPI_ISL_2592011, EPI_ISL_2592012, EPI_ISL_2592013, EPI_ISL_2592014, EPI_ISL_2592015, EPI_ISL_2592016, EPI_ISL_2592017, EPI_ISL_2592018, EPI_ISL_2592019, EPI_ISL_2592020, EPI_ISL_2592021, EPI_ISL_2592022, EPI_ISL_2592023			
see above	Virology Laboratory, Dr. Ricardo Gutierrez Children Hospital	Virology Laboratory, Dr. Ricardo Gutierrez Children Hospital	Viegas,M., Goya,S. and Mistchenko,A.S.
EPI_ISL_2592101, EPI_ISL_2592102, EPI_ISL_2592103, EPI_ISL_2592104, EPI_ISL_2592105, EPI_ISL_2592106, EPI_ISL_2592107, EPI_ISL_2592108, EPI_ISL_2592109, EPI_ISL_2592110, EPI_ISL_2592111, EPI_ISL_2592112, EPI_ISL_2592113, EPI_ISL_2592114, EPI_ISL_2592166, EPI_ISL_2592191, EPI_ISL_2592192, EPI_ISL_2592193, EPI_ISL_2592194, EPI_ISL_2592195, EPI_ISL_2592196, EPI_ISL_2592197, EPI_ISL_2592198, EPI_ISL_2592199, EPI_ISL_2592200, EPI_ISL_2592201, EPI_ISL_2592202, EPI_ISL_2592203, EPI_ISL_2592204, EPI_ISL_2592205, EPI_ISL_2592206, EPI_ISL_2592207, EPI_ISL_2592208, EPI_ISL_2592213			
see above	Virology Laborotary, Beijing Pediatric Research Institute, Beijing Children's Hospital, Capital Medical University, National Center for Children's Health	Virology Laborotary, Beijing Pediatric Research Institute, Beijing Children's Hospital, Capital Medical University, National Center for Children's Health	Chen,X., Guo,J. and Xie,Z.
EPI_ISL_2592214, EPI_ISL_2592215, EPI_ISL_2592216	Translational Medicine Institute, Affiliated the First People's Hospital of Chenzhou, University of South China	Translational Medicine Institute, Affiliated the First People's Hospital of Chenzhou, University of South China	Fan,R.Y., Qu,X.W. and Fan,C.P.
EPI_ISL_2592217	Microbiology Laboratory, Hangzhou Center for Disease Control and Prevention	Microbiology Laboratory, Hangzhou Center for Disease Control and Prevention	Yu,X., Kou,Y. and Li,J.
EPI_ISL_2592218, EPI_ISL_2592219, EPI_ISL_2592220, EPI_ISL_2592221, EPI_ISL_2592222, EPI_ISL_2592223, EPI_ISL_2592224, EPI_ISL_2592225, EPI_ISL_2592226, EPI_ISL_2592227, EPI_ISL_2592228, EPI_ISL_2592229, EPI_ISL_2592230, EPI_ISL_2592231, EPI_ISL_2592232, EPI_ISL_2592233, EPI_ISL_2592234, EPI_ISL_2592235, EPI_ISL_2592236, EPI_ISL_2592237, EPI_ISL_2592238, EPI_ISL_2592239, EPI_ISL_2592240, EPI_ISL_2592241, EPI_ISL_2592242, EPI_ISL_2592243, EPI_ISL_2592244, EPI_ISL_2592245, EPI_ISL_2592246, EPI_ISL_2592247, EPI_ISL_2592248, EPI_ISL_2592249, EPI_ISL_2592250, EPI_ISL_2592251, EPI_ISL_2592252, EPI_ISL_2592253, EPI_ISL_2592254, EPI_ISL_2592255, EPI_ISL_2592256, EPI_ISL_2592257, EPI_ISL_2592258, EPI_ISL_2592259, EPI_ISL_2592260, EPI_ISL_2592261, EPI_ISL_2592262, EPI_ISL_2592263, EPI_ISL_2592264, EPI_ISL_2592265, EPI_ISL_2592266, EPI_ISL_2592267, EPI_ISL_2592268, EPI_ISL_2592269, EPI_ISL_2592270, EPI_ISL_2592271, EPI_ISL_2592272, EPI_ISL_2592273, EPI_ISL_2592274, EPI_ISL_2592275, EPI_ISL_2592276, EPI_ISL_2592277, EPI_ISL_2592278, EPI_ISL_2592279, EPI_ISL_2592280, EPI_ISL_2592281, EPI_ISL_2592282, EPI_ISL_2592283, EPI_ISL_2592284, EPI_ISL_2592285, EPI_ISL_2592286, EPI_ISL_2592287, EPI_ISL_2592288, EPI_ISL_2592289, EPI_ISL_2592290, EPI_ISL_2592291, EPI_ISL_2592292, EPI_ISL_2592293, EPI_ISL_2592294, EPI_ISL_2592295			
see above	Virology Laborotary, Beijing Pediatric Research Institute, Beijing Children's Hospital, Capital Medical University, National Center for Children's Health	Virology Laborotary, Beijing Pediatric Research Institute, Beijing Children's Hospital, Capital Medical University, National Center for Children's Health	Chen,X., Guo,J. and Xie,Z.
EPI_ISL_2592296, EPI_ISL_2592297, EPI_ISL_2592298, EPI_ISL_2592299, EPI_ISL_2592300, EPI_ISL_2592303, EPI_ISL_2592304, EPI_ISL_2592305, EPI_ISL_2592306, EPI_ISL_2592307, EPI_ISL_2592308, EPI_ISL_2592309, EPI_ISL_2592310, EPI_ISL_2592311, EPI_ISL_2592312			
see above	Laboratory for Molecular Biomedicine, Centre for Research and Knowledge Transfer in Biotechnology, University of Zagreb	Laboratory for Molecular Biomedicine, Centre for Research and Knowledge Transfer in Biotechnology, University of Zagreb	Slovic,A., Forcic,D., Ivancic-Jelecki,J., Ljubin Sternak,S. and Mlinaric-Galinovic,G.
EPI_ISL_2592313, EPI_ISL_2592314	Microbiology Department, Virology Division, College of Medicine, Taif University, Virology Department, Faculty of Veterinary Medicine	Microbiology Department, Virology Division, College of Medicine, Taif University, Virology Department, Faculty of Veterinary Medicine	Abdel-Moneim,A.S., Soliman,M.S., Kamel,M.M. and El-Kholy,A.A.
EPI_ISL_2592315, EPI_ISL_2592316, EPI_ISL_2592317, EPI_ISL_2592318, EPI_ISL_2592319, EPI_ISL_2592320, EPI_ISL_2592321, EPI_ISL_2592322	Department for Infectious Diseases Virology, University of Heidelberg	Department for Infectious Diseases Virology, University of Heidelberg	Tabatabai,J., Prifert,C., Pfeil,J., Grulich-Henn,J. and Schnitzler,P.
EPI_ISL_2592323, EPI_ISL_2592324, EPI_ISL_2592325, EPI_ISL_2592326, EPI_ISL_2592327, EPI_ISL_2592328, EPI_ISL_2592329, EPI_ISL_2592330, EPI_ISL_2592331, EPI_ISL_2592332, EPI_ISL_2592333, EPI_ISL_2592334, EPI_ISL_2592335, EPI_ISL_2592336, EPI_ISL_2592337, EPI_ISL_2592338, EPI_ISL_2592339, EPI_ISL_2592340, EPI_ISL_2592341, EPI_ISL_2592342, EPI_ISL_2592343, EPI_ISL_2592344, EPI_ISL_2592345, EPI_ISL_2592346, EPI_ISL_2592347, EPI_ISL_2592348, EPI_ISL_2592349, EPI_ISL_2592350, EPI_ISL_2592351, EPI_ISL_2592352, EPI_ISL_2592353, EPI_ISL_2592354, EPI_ISL_2592355, EPI_ISL_2592356, EPI_ISL_2592357, EPI_ISL_2592358, EPI_ISL_2592359, EPI_ISL_2592360, EPI_ISL_2592361			
see above	Department for Infectious Diseases Virology, University of Heidelberg	Department for Infectious Diseases Virology, University of Heidelberg	Tabatabai,J., Lehners,N., Prifert,C., Wedde,M., Puthenparambil,J., Weissbrich,B., Schweiger,B., Egerer,G. and Schnitzler,P.
EPI_ISL_2592384, EPI_ISL_2592385, EPI_ISL_2592386, EPI_ISL_2592387, EPI_ISL_2592388	Microbiology, King George Medical University	Microbiology, King George Medical University	Singh,A.K., Jain,B., Dangi,T., Prakash,S., Verma,A., Dwivedi,M., Kumar,A. and Jain,A.
EPI_ISL_2592390	Microbiology, Jawaharlal Institute of Postgraduate Medical Education & Research (JIPMER)	Microbiology, Jawaharlal Institute of Postgraduate Medical Education & Research (JIPMER)	Nandhini,G. and Sujatha,S.
EPI_ISL_2592397, EPI_ISL_2592398, EPI_ISL_2592399, EPI_ISL_2592400, EPI_ISL_2592401, EPI_ISL_2592402, EPI_ISL_2592403, EPI_ISL_2592404, EPI_ISL_2592405, EPI_ISL_2592406, EPI_ISL_2592407, EPI_ISL_2592408, EPI_ISL_2592409, EPI_ISL_2592410, EPI_ISL_2592411, EPI_ISL_2592412, EPI_ISL_2592413, EPI_ISL_2592414, EPI_ISL_2592415, EPI_ISL_2592416, EPI_ISL_2592417, EPI_ISL_2592418, EPI_ISL_2592419, EPI_ISL_2592420, EPI_ISL_2592421			
see above	Dept of Biomedical Sciences and Public Health, Virology Unit, Marche Politechnic University Medical School	Dept of Biomedical Sciences and Public Health, Virology Unit, Marche Politechnic University Medical School	Bagnarelli,P., Trotta,D., Ferreri,M.L. and Pierangeli,A.
EPI_ISL_2595218	Department of Public Health Laboratory Sciences, West China School of Public Health, Sichuan University	Department of Public Health Laboratory Sciences, West China School of Public Health, Sichuan University	Hu,W.P. and Pei,F.X.
EPI_ISL_2595219, EPI_ISL_2595220	Department of Clinical Laboratory, Fujian Provincial Hospital	Department of Clinical Laboratory, Fujian Provincial Hospital	Su,Y., Wu,Y., Tian,R. and Liang,G.
EPI_ISL_2595221	Department of Public Health Laboratory Sciences, West China School of Public Health, Sichuan University	Department of Public Health Laboratory Sciences, West China School of Public Health, Sichuan University	Hu,W.P. and Pei,F.X.
EPI_ISL_2595222, EPI_ISL_2595223	Epidemiology and Demography, KEMRI - Wellcome Trust Research Programme, Centre for Geographic Medicine Research	Epidemiology and Demography, KEMRI - Wellcome Trust Research Programme, Centre for Geographic Medicine Research	Oketch,J.W., Kamau,E., Otieno,J.R., Mwema,A., Lewa,C., Agoti,C.N. and Nokes,J.D.
EPI_ISL_2595224	Department of Epidemiology and Demography, Kenya Medical Research Institute (KEMRI) - Wellcome Trust Research Programme	Department of Epidemiology and Demography, Kenya Medical Research Institute (KEMRI) - Wellcome Trust Research Programme	Otieno,J.R., Agoti,C.N., Gitahi,C.W., Bett,A., Ngama,M., Medley,G.F., Cane,P.A. and Nokes,J.D.
EPI_ISL_2595225	Epidemiology and Demography, Kenya Medical Research Institute (KEMRI) - Wellcome Trust Research Programme	Epidemiology and Demography, Kenya Medical Research Institute (KEMRI) - Wellcome Trust Research Programme	Agoti,C.N., Otieno,J.R., Gitahi,C.W., Cane,P.A. and Nokes,D.J.
EPI_ISL_2595226	Virology, School of Public Health, Tehran University of Medical Sciences	Virology, School of Public Health, Tehran University of Medical Sciences	Yavarian,J., Faghihloo,E. and Mokhtari Azad,T.
EPI_ISL_2595227	Epidemiology and Demography, KEMRI - Wellcome Trust Research Programme, Centre for Geographic Medicine Research	Epidemiology and Demography, KEMRI - Wellcome Trust Research Programme, Centre for Geographic Medicine Research	Oketch,J.W., Kamau,E., Otieno,J.R., Mwema,A., Lewa,C., Agoti,C.N. and Nokes,J.D.
EPI_ISL_2595228	Pathogen Diagnostic Center, Institut Pasteur of Shanghai	Pathogen Diagnostic Center, Institut Pasteur of Shanghai	Liu,J., Mu,Y., Dong,W., Yao,F., Yan,H., Lan,K. and Zhang,C.
EPI_ISL_2595229, EPI_ISL_2595230	Epidemiology and Demography, KEMRI - Wellcome Trust Research Programme, Centre for Geographic Medicine Research	Epidemiology and Demography, KEMRI - Wellcome Trust Research Programme, Centre for Geographic Medicine Research	Oketch,J.W., Kamau,E., Otieno,J.R., Mwema,A., Lewa,C., Agoti,C.N. and Nokes,J.D.
EPI_ISL_2595231	Virology Section, Department of Microbiology, King George's Medical University	Virology Section, Department of Microbiology, King George's Medical University	Singh,A.K., Jain,A., Jain,B., Dangi,T., Verma,A.K., Dwivedi,M. and Kushwaha,R.
EPI_ISL_2595232	Department of Epidemiology and Demography, Kenya Medical Research Institute (KEMRI) - Wellcome Trust Research Programme	Department of Epidemiology and Demography, Kenya Medical Research Institute (KEMRI) - Wellcome Trust Research Programme	Otieno,J.R., Agoti,C.N., Gitahi,C.W., Bett,A., Ngama,M., Medley,G.F., Cane,P.A. and Nokes,J.D.
EPI_ISL_2595233	Epidemiology and Demography, Kenya Medical Research Institute (KEMRI) - Wellcome Trust Research Programme	Epidemiology and Demography, Kenya Medical Research Institute (KEMRI) - Wellcome Trust Research Programme	Agoti,C.N., Otieno,J.R., Gitahi,C.W., Cane,P.A. and Nokes,D.J.
EPI_ISL_2595234	Epidemiology and Demography, KEMRI - Wellcome Trust Research Programme, Centre for Geographic Medicine Research	Epidemiology and Demography, KEMRI - Wellcome Trust Research Programme, Centre for Geographic Medicine Research	Oketch,J.W., Kamau,E., Otieno,J.R., Mwema,A., Lewa,C., Agoti,C.N. and Nokes,J.D.
EPI_ISL_2595235	Virology, School of Public Health, Tehran University of Medical Sciences	Virology, School of Public Health, Tehran University of Medical Sciences	Yavarian,J., Faghihloo,E. and Mokhtari Azad,T.
EPI_ISL_2595236	Broad Institute of MIT & Harvard	Broad Institute of MIT & Harvard	Newman,R.M., Zody,M.C., DeVincenzo,J.P., Grad,Y., Lipsitch,M., Murphy,R., Fitzgerald,M., Young,S., Gargeya,S., Poon,T.W., Charlebois,P., Weiner,B., Yang,X., Piper,M.E., McCowan,C., Ireland,A., Levin,J., Malboeuf,C., Qu,J., Chapman,S.B., Murphy,C., Wortman,J., Nusbaum,C. and Birren,B.
EPI_ISL_2595237	Chinese Academy of Medical Sciences & Peking Union Medical College	Chinese Academy of Medical Sciences & Peking Union Medical College	Jia,B., Xiao,Y., Wang,Y., Chen,L., Zhang,J., Ren,L. and Wang,J.
EPI_ISL_2595238, EPI_ISL_2595239, EPI_ISL_2595240, EPI_ISL_2595241, EPI_ISL_2595242	University of Wuerzburg, Institute of Virology and Immunobiology	University of Wuerzburg, Institute of Virology and Immunobiology	Prifert,C., Hofmann,D. and Weissbrich,B.
EPI_ISL_2595243	Depto Microbiologia, Instituto de Ciencias Biomedicas, Universidade de Sao Paulo	Depto Microbiologia, Instituto de Ciencias Biomedicas, Universidade de Sao Paulo	Moura,F.E.A., Thomazelli,L.M., Campelo,F.S., Delfraro,A., Arbiza,J. and Durigon,E.L.
EPI_ISL_2595244, EPI_ISL_2595245, EPI_ISL_2595246	Depto Microbiologia, Instituto de Ciencias Biomedicas, Universidade de Sao Paulo	Depto Microbiologia, Instituto de Ciencias Biomedicas, Universidade de Sao Paulo	Thomazelli,L.M., Oliveira,D.B.L., Colmanetti,T., Vieira,S.E., Paulis,M., Ferronato,A.E., Martinez,M.B. and Durigon,E.L.
EPI_ISL_2595247, EPI_ISL_2595248	Chinese Academy of Medical Sciences & Peking Union Medical College	Chinese Academy of Medical Sciences & Peking Union Medical College	Jia,B., Xiao,Y., Wang,Y., Chen,L., Zhang,J., Ren,L. and Wang,J.
EPI_ISL_2595249, EPI_ISL_2595250, EPI_ISL_2595251, EPI_ISL_2595252, EPI_ISL_2595253, EPI_ISL_2595254, EPI_ISL_2595255, EPI_ISL_2595256	University of Wuerzburg, Institute of Virology and Immunobiology	University of Wuerzburg, Institute of Virology and Immunobiology	Prifert,C., Hofmann,D. and Weissbrich,B.
EPI_ISL_2595257	Depto Microbiologia, Instituto de Ciencias Biomedicas, Universidade de Sao Paulo	Depto Microbiologia, Instituto de Ciencias Biomedicas, Universidade de Sao Paulo	Thomazelli,L.M., Oliveira,D.B.L., Colmanetti,T., Vieira,S.E., Paulis,M., Ferronato,A.E., Martinez,M.B. and Durigon,E.L.
EPI_ISL_2595258, EPI_ISL_2595259, EPI_ISL_2595260, EPI_ISL_2595261	University of Wuerzburg, Institute of Virology and Immunobiology	University of Wuerzburg, Institute of Virology and Immunobiology	Prifert,C., Hofmann,D. and Weissbrich,B.
EPI_ISL_2595262	Depto Microbiologia, Instituto de Ciencias Biomedicas, Universidade de Sao Paulo	Depto Microbiologia, Instituto de Ciencias Biomedicas, Universidade de Sao Paulo	Moura,F.E.A., Thomazelli,L.M., Campelo,F.S., Delfraro,A., Arbiza,J. and Durigon,E.L.
EPI_ISL_2595263	Virology Department, Hospices Civils de Lyon	Virology Department, Hospices Civils de Lyon	Gaymard,A., Pichon,M. and Morfin,F.
EPI_ISL_2595264	Depto Microbiologia, Instituto de Ciencias Biomedicas, Universidade de Sao Paulo	Depto Microbiologia, Instituto de Ciencias Biomedicas, Universidade de Sao Paulo	Moura,F.E.A., Thomazelli,L.M., Campelo,F.S., Delfraro,A., Arbiza,J. and Durigon,E.L.
EPI_ISL_2595265	Centro de Estudios en Salud, Universidad Del Valle De Guatemala	Centro de Estudios en Salud, Universidad Del Valle De Guatemala	Lupo,P., Jara,J., Alvis,J.P., Pennington,P., Castillo,L., Moreno,B. and Albrego,L.

EPI_ISL_2595266	Health Science, Universidade Federal Do Parana	Health Science, Universidade Federal Do Parana	Moreira,F.B., Santos,J.S., Avanzi,V.M., Nogueira,M.B., Vidal,L.R.R. and Raboni,S.M.
EPI_ISL_2595267, EPI_ISL_2595268, EPI_ISL_2595269, EPI_ISL_2595270, EPI_ISL_2595271, EPI_ISL_2595272, EPI_ISL_2595273, EPI_ISL_2595274, EPI_ISL_2595275	Depto Microbiologia, Instituto de Ciencias Biomedicas, Universidade de Sao Paulo	Depto Microbiologia, Instituto de Ciencias Biomedicas, Universidade de Sao Paulo	Moura,F.E.A., Thomazelli,L.M., Campelo,F.S., Delfraro,A., Arbiza,J. and Durigon,E.L.
EPI_ISL_2595276	Chinese Academy of Medical Sciences & Peking Union Medical College	Chinese Academy of Medical Sciences & Peking Union Medical College	Jia,B., Xiao,Y., Wang,Y., Chen,L., Zhang,J., Ren,L. and Wang,J.
EPI_ISL_2595277, EPI_ISL_2595278, EPI_ISL_2595279	Broad Institute of MIT & Harvard	Broad Institute of MIT & Harvard	Newman,R.M., Zody,M.C., DeVincenzo,J.P., Grad,Y., Lipsitch,M., Murphy,R., Fitzgerald,M., Young,S., Gargeya,S., Poon,T.W., Charlebois,P., Weiner,B., Yang,X., Piper,M.E., McCowan,C., Ireland,A., Levin,J., Malboeuf,C., Qu,J., Chapman,S.B., Murphy,C., Wortman,J., Nusbaum,C. and Birren,B.
EPI_ISL_2595280	Virology Department, Hospices Civils de Lyon	Virology Department, Hospices Civils de Lyon	Gaymard,A., Pichon,M. and Morfin,F.
EPI_ISL_2595281	Minoru Nidaira Okinawa Prefectural Institute of Health and Environment, Department of Biological Science	Minoru Nidaira Okinawa Prefectural Institute of Health and Environment, Department of Biological Science	Nidaira,M., Kato,T. and Arakaki,E.
EPI_ISL_2595282	Laboratory of Virology, Capital Institute of Pediatrics	Laboratory of Virology, Capital Institute of Pediatrics	Cui,G., Zhu,R., Qian,Y., Deng,J., Zhao,L., Sun,Y. and Wang,F.
EPI_ISL_2595283, EPI_ISL_2595284	Centre for Research And Knowledge Transfer in Biotechnology, University of Zagreb	Centre for Research And Knowledge Transfer in Biotechnology, University of Zagreb	Ivancic-Jelecki,J., Slovic,A., Ljubin-Sternak,S., Mlinaric-Galinovic,G. and Forcic,D.
EPI_ISL_2595285	Department of Epidemiology and Demography, Kenya Medical Research Institute (KEMRI) - Wellcome Trust Research Programme	Department of Epidemiology and Demography, Kenya Medical Research Institute (KEMRI) - Wellcome Trust Research Programme	Otieno,J.R., Kamau,E.M., Agoti,C.N., Lewa,C., Bett,A., Ngama,M., Cane,P.A. and Nokes,J.D.
EPI_ISL_2595286	Centre for Research And Knowledge Transfer in Biotechnology, University of Zagreb	Centre for Research And Knowledge Transfer in Biotechnology, University of Zagreb	Ivancic-Jelecki,J., Slovic,A., Ljubin-Sternak,S., Mlinaric-Galinovic,G. and Forcic,D.
EPI_ISL_2595287, EPI_ISL_2595288	Virology, School of Public Health, Tehran University of Medical Sciences	Virology, School of Public Health, Tehran University of Medical Sciences	Yavarian,J., Faghihloo,E. and Mokhtari Azad,T.
EPI_ISL_2595289	Epidemiology and Demography, KEMRI - Wellcome Trust Research Programme, Centre for Geographic Medicine Research	Epidemiology and Demography, KEMRI - Wellcome Trust Research Programme, Centre for Geographic Medicine Research	Oketch,J.W., Kamau,E., Otieno,J.R., Mwema,A., Lewa,C., Agoti,C.N. and Nokes,J.D.
EPI_ISL_2595290	Virology Section, Department of Microbiology, King George's Medical University	Virology Section, Department of Microbiology, King George's Medical University	Singh,A.K., Jain,A., Jain,B., Dangi,T., Verma,A.K., Dwivedi,M. and Kushwaha,R.
EPI_ISL_2595291, EPI_ISL_2595292, EPI_ISL_2595293	Epidemiology and Demography, KEMRI - Wellcome Trust Research Programme, Centre for Geographic Medicine Research	Epidemiology and Demography, KEMRI - Wellcome Trust Research Programme, Centre for Geographic Medicine Research	Oketch,J.W., Kamau,E., Otieno,J.R., Mwema,A., Lewa,C., Agoti,C.N. and Nokes,J.D.
EPI_ISL_2595294, EPI_ISL_2595295	Virology, School of Public Health, Tehran University of Medical Sciences	Virology, School of Public Health, Tehran University of Medical Sciences	Yavarian,J., Faghihloo,E. and Mokhtari Azad,T.
EPI_ISL_2595296	Department of Clinical Laboratory, Fujian Provincial Hospital	Department of Clinical Laboratory, Fujian Provincial Hospital	Su,Y., Wu,Y., Tian,R. and Liang,G.
EPI_ISL_2595297	Virology, School of Public Health, Tehran University of Medical Sciences	Virology, School of Public Health, Tehran University of Medical Sciences	Yavarian,J., Faghihloo,E. and Mokhtari Azad,T.
EPI_ISL_2595298, EPI_ISL_2595299	WHO WPRO Measles Regional Reference Lab, Key Laboratory of Medical Virology Ministry of Health, National Institute for Viral Disease Control and Prevention, China Center for Disease Control and Prevention	WHO WPRO Measles Regional Reference Lab, Key Laboratory of Medical Virology Ministry of Health, National Institute for Viral Disease Control and Prevention, China Center for Disease Control and Prevention	Zhang,Y., Song,J. and Xu,W.
EPI_ISL_2595300, EPI_ISL_2595301	Department of Epidemiology and Demography, Kenya Medical Research Institute (KEMRI) - Wellcome Trust Research Programme	Department of Epidemiology and Demography, Kenya Medical Research Institute (KEMRI) - Wellcome Trust Research Programme	Otieno,J.R., Kamau,E.M., Agoti,C.N., Lewa,C., Bett,A., Ngama,M., Cane,P.A. and Nokes,J.D.
EPI_ISL_2595302, EPI_ISL_2595303	Epidemiology and Demography, KEMRI - Wellcome Trust Research Programme, Centre for Geographic Medicine Research	Epidemiology and Demography, KEMRI - Wellcome Trust Research Programme, Centre for Geographic Medicine Research	Oketch,J.W., Kamau,E., Otieno,J.R., Mwema,A., Lewa,C., Agoti,C.N. and Nokes,J.D.
EPI_ISL_2595304	Department of Epidemiology and Demography, Kenya Medical Research Institute (KEMRI) - Wellcome Trust Research Programme	Department of Epidemiology and Demography, Kenya Medical Research Institute (KEMRI) - Wellcome Trust Research Programme	Otieno,J.R., Kamau,E.M., Agoti,C.N., Lewa,C., Bett,A., Ngama,M., Cane,P.A. and Nokes,J.D.
EPI_ISL_2595305, EPI_ISL_2595306, EPI_ISL_2595307	Epidemiology and Demography, KEMRI - Wellcome Trust Research Programme, Centre for Geographic Medicine Research	Epidemiology and Demography, KEMRI - Wellcome Trust Research Programme, Centre for Geographic Medicine Research	Oketch,J.W., Kamau,E., Otieno,J.R., Mwema,A., Lewa,C., Agoti,C.N. and Nokes,J.D.
EPI_ISL_2595308, EPI_ISL_2595309	Centre for Research And Knowledge Transfer in Biotechnology, University of Zagreb	Centre for Research And Knowledge Transfer in Biotechnology, University of Zagreb	Ivancic-Jelecki,J., Slovic,A., Ljubin-Sternak,S., Mlinaric-Galinovic,G. and Forcic,D.
EPI_ISL_2595310	WHO WPRO Measles Regional Reference Lab, Key Laboratory of Medical Virology Ministry of Health, National Institute for Viral Disease Control and Prevention, China Center for Disease Control and Prevention	WHO WPRO Measles Regional Reference Lab, Key Laboratory of Medical Virology Ministry of Health, National Institute for Viral Disease Control and Prevention, China Center for Disease Control and Prevention	Zhang,Y., Song,J. and Xu,W.
EPI_ISL_2595311	Epidemiology and Demography, KEMRI - Wellcome Trust Research Programme, Centre for Geographic Medicine Research	Epidemiology and Demography, KEMRI - Wellcome Trust Research Programme, Centre for Geographic Medicine Research	Oketch,J.W., Kamau,E., Otieno,J.R., Mwema,A., Lewa,C., Agoti,C.N. and Nokes,J.D.
EPI_ISL_2595312	Virology, School of Public Health, Tehran University of Medical Sciences	Virology, School of Public Health, Tehran University of Medical Sciences	Yavarian,J., Faghihloo,E. and Mokhtari Azad,T.
EPI_ISL_2595313	Centre for Research And Knowledge Transfer in Biotechnology, University of Zagreb	Centre for Research And Knowledge Transfer in Biotechnology, University of Zagreb	Ivancic-Jelecki,J., Slovic,A., Ljubin-Sternak,S., Mlinaric-Galinovic,G. and Forcic,D.
EPI_ISL_2595314	Epidemiology and Demography, KEMRI - Wellcome Trust Research Programme, Centre for Geographic Medicine Research	Epidemiology and Demography, KEMRI - Wellcome Trust Research Programme, Centre for Geographic Medicine Research	Oketch,J.W., Kamau,E., Otieno,J.R., Mwema,A., Lewa,C., Agoti,C.N. and Nokes,J.D.
EPI_ISL_2595315	Epidemiology and Demography Department, KEMRI-Wellcome Trust Research Programme	Epidemiology and Demography Department, KEMRI-Wellcome Trust Research Programme	Otieno,J.R., Kamau,E.M., Oketch,J.W., Ngoi,J.M., Agoti,C.N., Gichuki,A.M., Otieno,G.P., Ngama,M., Cane,P.A., Kellam,P., Cotten,M., Lemey,P. and Nokes,D.J.
EPI_ISL_2595316	Microbiology Department, Virology Division, College of Medicine, Taif University	Microbiology Department, Virology Division, College of Medicine, Taif University	Al Aboud,D.M., Al Aboud,N.M., Al-Malky,M.I.R. and Abdel-Moneim,A.S.
EPI_ISL_2595317, EPI_ISL_2595318	Epidemiology and Demography, KEMRI - Wellcome Trust Research Programme, Centre for Geographic Medicine Research	Epidemiology and Demography, KEMRI - Wellcome Trust Research Programme, Centre for Geographic Medicine Research	Oketch,J.W., Kamau,E., Otieno,J.R., Mwema,A., Lewa,C., Agoti,C.N. and Nokes,J.D.
EPI_ISL_2595319, EPI_ISL_2595320	Centre for Research And Knowledge Transfer in Biotechnology, University of Zagreb	Centre for Research And Knowledge Transfer in Biotechnology, University of Zagreb	Ivancic-Jelecki,J., Slovic,A., Ljubin-Sternak,S., Mlinaric-Galinovic,G. and Forcic,D.
EPI_ISL_2595321	J. Craig Venter Institute	J. Craig Venter Institute	Shrivastava,S., Halpin,R.A., Puri,V., Fedorova,N.B., Stockwell,T., Amedeo,P., Katzel,D., Schobel,S., Pickett,B.E., Moore,M., Chappell,J., Larkin,E., Wentworth,D.E., Anderson,L.J. and Hartert,T.
EPI_ISL_2595322	Epidemiology and Demography, KEMRI - Wellcome Trust Research Programme, Centre for Geographic Medicine Research	Epidemiology and Demography, KEMRI - Wellcome Trust Research Programme, Centre for Geographic Medicine Research	Oketch,J.W., Kamau,E., Otieno,J.R., Mwema,A., Lewa,C., Agoti,C.N. and Nokes,J.D.
EPI_ISL_2595323	Department of Clinical Laboratory, Fujian Provincial Hospital	Department of Clinical Laboratory, Fujian Provincial Hospital	Su,Y., Wu,Y., Tian,R. and Liang,G.
EPI_ISL_2595324, EPI_ISL_2595325, EPI_ISL_2595326	Epidemiology and Demography, KEMRI - Wellcome Trust Research Programme, Centre for Geographic Medicine Research	Epidemiology and Demography, KEMRI - Wellcome Trust Research Programme, Centre for Geographic Medicine Research	Oketch,J.W., Kamau,E., Otieno,J.R., Mwema,A., Lewa,C., Agoti,C.N. and Nokes,J.D.
EPI_ISL_2595327	Centre for Infectious Diseases Research, Diagnostics and laboratory Surveillance, National Institute for Public Health and the Environment	Centre for Infectious Diseases Research, Diagnostics and laboratory Surveillance, National Institute for Public Health and the Environment	Meijer,A. and Overduin,P.
EPI_ISL_2595328, EPI_ISL_2595329	Epidemiology and Demography, KEMRI - Wellcome Trust Research Programme, Centre for Geographic Medicine Research	Epidemiology and Demography, KEMRI - Wellcome Trust Research Programme, Centre for Geographic Medicine Research	Oketch,J.W., Kamau,E., Otieno,J.R., Mwema,A., Lewa,C., Agoti,C.N. and Nokes,J.D.
EPI_ISL_2595330	Centre for Research And Knowledge Transfer in Biotechnology, University of Zagreb	Centre for Research And Knowledge Transfer in Biotechnology, University of Zagreb	Ivancic-Jelecki,J., Slovic,A., Ljubin-Sternak,S., Mlinaric-Galinovic,G. and Forcic,D.
EPI_ISL_2595331	Epidemiology and Demography, KEMRI - Wellcome Trust Research Programme, Centre for Geographic Medicine Research	Epidemiology and Demography, KEMRI - Wellcome Trust Research Programme, Centre for Geographic Medicine Research	Oketch,J.W., Kamau,E., Otieno,J.R., Mwema,A., Lewa,C., Agoti,C.N. and Nokes,J.D.
EPI_ISL_2595332	Centre for Research And Knowledge Transfer in Biotechnology, University of Zagreb	Centre for Research And Knowledge Transfer in Biotechnology, University of Zagreb	Ivancic-Jelecki,J., Slovic,A., Ljubin-Sternak,S., Mlinaric-Galinovic,G. and Forcic,D.
EPI_ISL_2595333	Virology, School of Public Health, Tehran University of Medical Sciences	Virology, School of Public Health, Tehran University of Medical Sciences	Yavarian,J., Faghihloo,E. and Mokhtari Azad,T.
EPI_ISL_2595334	Department of Respiratory Medicine, Children's Hospital of Chongqing Medical University	Department of Respiratory Medicine, Children's Hospital of Chongqing Medical University	Ren,L. and Liu,E.
EPI_ISL_2595335, EPI_ISL_2595336	Virology, Tohoku University Graduate School of Medicine	Virology, Tohoku University Graduate School of Medicine	Malasao,R., Okamoto,M., Chaimongkol,N., Imamura,T., Tohma,K., Dapat,I., Dapat,C., Suzuki,A., Saito,M., Saito,M., Tamaki,R., Segubre-Mercado,E., Igoy,M.A.U., Lupisan,S., Olveda,R. and Oshitani,H.
EPI_ISL_2595337	Department of Respiratory Medicine, Children's Hospital of Chongqing Medical University	Department of Respiratory Medicine, Children's Hospital of Chongqing Medical University	Ren,L. and Liu,E.
EPI_ISL_2595338, EPI_ISL_2595339, EPI_ISL_2595340	Epidemiology and Demography, KEMRI - Wellcome Trust Research Programme, Centre for Geographic Medicine Research	Epidemiology and Demography, KEMRI - Wellcome Trust Research Programme, Centre for Geographic Medicine Research	Oketch,J.W., Kamau,E., Otieno,J.R., Mwema,A., Lewa,C., Agoti,C.N. and Nokes,J.D.
EPI_ISL_2595341	Department of Epidemiology and Demography, Kenya Medical Research Institute (KEMRI) - Wellcome Trust Research Programme	Department of Epidemiology and Demography, Kenya Medical Research Institute (KEMRI) - Wellcome Trust Research Programme	Otieno,J.R., Kamau,E.M., Agoti,C.N., Lewa,C., Bett,A., Ngama,M., Cane,P.A. and Nokes,J.D.
EPI_ISL_2595342	Epidemiology and Demography, KEMRI - Wellcome Trust Research Programme, Centre for Geographic Medicine Research	Epidemiology and Demography, KEMRI - Wellcome Trust Research Programme, Centre for Geographic Medicine Research	Oketch,J.W., Kamau,E., Otieno,J.R., Mwema,A., Lewa,C., Agoti,C.N. and Nokes,J.D.
EPI_ISL_2595343	WHO WPRO Measles Regional Reference Lab, Key Laboratory of Medical	WHO WPRO Measles Regional Reference Lab, Key Laboratory of Medical	Zhang,Y., Song,J. and Xu,W.

	Virology Ministry of Health, National Institute for Viral Disease Control and Prevention, China Center for Disease Control and Prevention	Virology Ministry of Health, National Institute for Viral Disease Control and Prevention, China Center for Disease Control and Prevention	
EPI_ISL_2595344, EPI_ISL_2595345	Epidemiology and Demography, KEMRI - Wellcome Trust Research Programme, Centre for Geographic Medicine Research	Epidemiology and Demography, KEMRI - Wellcome Trust Research Programme, Centre for Geographic Medicine Research	Oketch,J.W., Kamau,E., Otieno,J.R., Mwema,A., Lewa,C., Agoti,C.N. and Nokes,J.D.
EPI_ISL_2595346	Microbiology Department, Virology Division, College of Medicine, Taif University	Microbiology Department, Virology Division, College of Medicine, Taif University	Al Aboud,D.M., Al Aboud,N.M., Al-Malky,M.I.R. and Abdel-Moneim,A.S.
EPI_ISL_2595347, EPI_ISL_2595348, EPI_ISL_2595349, EPI_ISL_2595350	Epidemiology and Demography, KEMRI - Wellcome Trust Research Programme, Centre for Geographic Medicine Research	Epidemiology and Demography, KEMRI - Wellcome Trust Research Programme, Centre for Geographic Medicine Research	Oketch,J.W., Kamau,E., Otieno,J.R., Mwema,A., Lewa,C., Agoti,C.N. and Nokes,J.D.
EPI_ISL_2595351, EPI_ISL_2595352	Department of Epidemiology and Demography, Kenya Medical Research Institute (KEMRI) - Wellcome Trust Research Programme	Department of Epidemiology and Demography, Kenya Medical Research Institute (KEMRI) - Wellcome Trust Research Programme	Otieno,J.R., Kamau,E.M., Agoti,C.N., Lewa,C., Bett,A., Ngama,M., Cane,P.A. and Nokes,J.D.
EPI_ISL_2595353, EPI_ISL_2595354, EPI_ISL_2595355	Epidemiology and Demography, KEMRI - Wellcome Trust Research Programme, Centre for Geographic Medicine Research	Epidemiology and Demography, KEMRI - Wellcome Trust Research Programme, Centre for Geographic Medicine Research	Oketch,J.W., Kamau,E., Otieno,J.R., Mwema,A., Lewa,C., Agoti,C.N. and Nokes,J.D.
EPI_ISL_2595356	Department of Epidemiology and Demography, Kenya Medical Research Institute (KEMRI) - Wellcome Trust Research Programme	Department of Epidemiology and Demography, Kenya Medical Research Institute (KEMRI) - Wellcome Trust Research Programme	Otieno,J.R., Kamau,E.M., Agoti,C.N., Lewa,C., Bett,A., Ngama,M., Cane,P.A. and Nokes,J.D.
EPI_ISL_2595357	Epidemiology and Demography Department, KEMRI-Wellcome Trust Research Programme	Epidemiology and Demography Department, KEMRI-Wellcome Trust Research Programme	Otieno,J.R., Kamau,E.M., Oketch,J.W., Ngoi,J.M., Agoti,C.N., Gichuki,A.M., Otieno,G.P., Ngama,M., Cane,P.A., Kellam,P., Cotten,M., Lemey,P. and Nokes,D.J.
EPI_ISL_2595358, EPI_ISL_2595359, EPI_ISL_2595360, EPI_ISL_2595361, EPI_ISL_2595362, EPI_ISL_2595363	Epidemiology and Demography, KEMRI - Wellcome Trust Research Programme, Centre for Geographic Medicine Research	Epidemiology and Demography, KEMRI - Wellcome Trust Research Programme, Centre for Geographic Medicine Research	Oketch,J.W., Kamau,E., Otieno,J.R., Mwema,A., Lewa,C., Agoti,C.N. and Nokes,J.D.
EPI_ISL_2595364	WHO WPRO Measles Regional Reference Lab, Key Laboratory of Medical Virology Ministry of Health, National Institute for Viral Disease Control and Prevention, China Center for Disease Control and Prevention	WHO WPRO Measles Regional Reference Lab, Key Laboratory of Medical Virology Ministry of Health, National Institute for Viral Disease Control and Prevention, China Center for Disease Control and Prevention	Zhang,Y., Song,J. and Xu,W.
EPI_ISL_2595365, EPI_ISL_2595366, EPI_ISL_2595367	Epidemiology and Demography, KEMRI - Wellcome Trust Research Programme, Centre for Geographic Medicine Research	Epidemiology and Demography, KEMRI - Wellcome Trust Research Programme, Centre for Geographic Medicine Research	Oketch,J.W., Kamau,E., Otieno,J.R., Mwema,A., Lewa,C., Agoti,C.N. and Nokes,J.D.
EPI_ISL_2595368	Department of Clinical Laboratory, Fujian Provincial Hospital	Department of Clinical Laboratory, Fujian Provincial Hospital	Su,Y., Wu,Y., Tian,R. and Liang,G.
EPI_ISL_2595369	Centre for Research And Knowledge Transfer in Biotechnology, University of Zagreb	Centre for Research And Knowledge Transfer in Biotechnology, University of Zagreb	Ivancic-Jelecki,J., Slovic,A., Ljubin-Sternak,S., Mlinaric-Galinovic,G. and Forcic,D.
EPI_ISL_2595370	Department of Clinical Laboratory, Fujian Provincial Hospital	Department of Clinical Laboratory, Fujian Provincial Hospital	Su,Y., Wu,Y., Tian,R. and Liang,G.
EPI_ISL_2595371, EPI_ISL_2595372, EPI_ISL_2595373, EPI_ISL_2595374, EPI_ISL_2595375, EPI_ISL_2595376, EPI_ISL_2595377, EPI_ISL_2595378, EPI_ISL_2595379	Epidemiology and Demography, KEMRI - Wellcome Trust Research Programme, Centre for Geographic Medicine Research	Epidemiology and Demography, KEMRI - Wellcome Trust Research Programme, Centre for Geographic Medicine Research	Oketch,J.W., Kamau,E., Otieno,J.R., Mwema,A., Lewa,C., Agoti,C.N. and Nokes,J.D.
EPI_ISL_2595380	WHO WPRO Measles Regional Reference Lab, Key Laboratory of Medical Virology Ministry of Health, National Institute for Viral Disease Control and Prevention, China Center for Disease Control and Prevention	WHO WPRO Measles Regional Reference Lab, Key Laboratory of Medical Virology Ministry of Health, National Institute for Viral Disease Control and Prevention, China Center for Disease Control and Prevention	Zhang,Y., Song,J. and Xu,W.
EPI_ISL_2595381, EPI_ISL_2595382, EPI_ISL_2595383	Epidemiology and Demography, KEMRI - Wellcome Trust Research Programme, Centre for Geographic Medicine Research	Epidemiology and Demography, KEMRI - Wellcome Trust Research Programme, Centre for Geographic Medicine Research	Oketch,J.W., Kamau,E., Otieno,J.R., Mwema,A., Lewa,C., Agoti,C.N. and Nokes,J.D.
EPI_ISL_2595384	Minoru Nidaira Okinawa Prefectural Institute of Health and Environment, Department of Biological Science	Minoru Nidaira Okinawa Prefectural Institute of Health and Environment, Department of Biological Science	Nidaira,M., Kato,T. and Arakaki,E.
EPI_ISL_2595385	Department of Respiratory Medicine, Children's Hospital of Chongqing Medical University	Department of Respiratory Medicine, Children's Hospital of Chongqing Medical University	Ren,L. and Liu,E.
EPI_ISL_2595386	Central Laboratory, Guangzhou Women and Children's Medical Center	Central Laboratory, Guangzhou Women and Children's Medical Center	Xie,J.H., Zhu,B., Zhong,J.Y., Chen,Y. and Zhang,Y.Y.
EPI_ISL_2595387, EPI_ISL_2595388, EPI_ISL_2595389	WHO WPRO Measles Regional Reference Lab, Key Laboratory of Medical Virology Ministry of Health, National Institute for Viral Disease Control and Prevention, China Center for Disease Control and Prevention	WHO WPRO Measles Regional Reference Lab, Key Laboratory of Medical Virology Ministry of Health, National Institute for Viral Disease Control and Prevention, China Center for Disease Control and Prevention	Zhang,Y., Song,J. and Xu,W.
EPI_ISL_2595390, EPI_ISL_2595391, EPI_ISL_2595392, EPI_ISL_2595393, EPI_ISL_2595394, EPI_ISL_2595395	Epidemiology and Demography, KEMRI - Wellcome Trust Research Programme, Centre for Geographic Medicine Research	Epidemiology and Demography, KEMRI - Wellcome Trust Research Programme, Centre for Geographic Medicine Research	Oketch,J.W., Kamau,E., Otieno,J.R., Mwema,A., Lewa,C., Agoti,C.N. and Nokes,J.D.
EPI_ISL_2595396	WHO WPRO Measles Regional Reference Lab, Key Laboratory of Medical Virology Ministry of Health, National Institute for Viral Disease Control and Prevention, China Center for Disease Control and Prevention	WHO WPRO Measles Regional Reference Lab, Key Laboratory of Medical Virology Ministry of Health, National Institute for Viral Disease Control and Prevention, China Center for Disease Control and Prevention	Zhang,Y., Song,J. and Xu,W.
EPI_ISL_2595397, EPI_ISL_2595398, EPI_ISL_2595399	Epidemiology and Demography, KEMRI - Wellcome Trust Research Programme, Centre for Geographic Medicine Research	Epidemiology and Demography, KEMRI - Wellcome Trust Research Programme, Centre for Geographic Medicine Research	Oketch,J.W., Kamau,E., Otieno,J.R., Mwema,A., Lewa,C., Agoti,C.N. and Nokes,J.D.
EPI_ISL_2595400	WHO WPRO Measles Regional Reference Lab, Key Laboratory of Medical Virology Ministry of Health, National Institute for Viral Disease Control and Prevention, China Center for Disease Control and Prevention	WHO WPRO Measles Regional Reference Lab, Key Laboratory of Medical Virology Ministry of Health, National Institute for Viral Disease Control and Prevention, China Center for Disease Control and Prevention	Zhang,Y., Song,J. and Xu,W.
EPI_ISL_2595401, EPI_ISL_2595402, EPI_ISL_2595403, EPI_ISL_2595404, EPI_ISL_2595405, EPI_ISL_2595406, EPI_ISL_2595407, EPI_ISL_2595408, EPI_ISL_2595409, EPI_ISL_2595410, EPI_ISL_2595411, EPI_ISL_2595412, EPI_ISL_2595413, EPI_ISL_2595414, EPI_ISL_2595415, EPI_ISL_2595416, EPI_ISL_2595417, EPI_ISL_2595418, EPI_ISL_2595419, EPI_ISL_2595420, EPI_ISL_2595421			
see above	Epidemiology and Demography, KEMRI - Wellcome Trust Research Programme, Centre for Geographic Medicine Research	Epidemiology and Demography, KEMRI - Wellcome Trust Research Programme, Centre for Geographic Medicine Research	Oketch,J.W., Kamau,E., Otieno,J.R., Mwema,A., Lewa,C., Agoti,C.N. and Nokes,J.D.
EPI_ISL_2595422	Virology, Noguchi Memorial Institute for Medical Research	Virology, Noguchi Memorial Institute for Medical Research	Obodai,E., Odoom,J.K., Adiku,T., Goka,B., Biere,B., Wolff,T., Schweiger,B. and Reiche,J.
EPI_ISL_2595423, EPI_ISL_2595424, EPI_ISL_2595425, EPI_ISL_2595426, EPI_ISL_2595427	Epidemiology and Demography, KEMRI - Wellcome Trust Research Programme, Centre for Geographic Medicine Research	Epidemiology and Demography, KEMRI - Wellcome Trust Research Programme, Centre for Geographic Medicine Research	Oketch,J.W., Kamau,E., Otieno,J.R., Mwema,A., Lewa,C., Agoti,C.N. and Nokes,J.D.
EPI_ISL_2595428	Department of Respiratory Medicine, Children's Hospital of Chongqing Medical University	Department of Respiratory Medicine, Children's Hospital of Chongqing Medical University	Ren,L. and Liu,E.
EPI_ISL_2595429, EPI_ISL_2595430, EPI_ISL_2595431, EPI_ISL_2595432, EPI_ISL_2595433, EPI_ISL_2595434, EPI_ISL_2595435, EPI_ISL_2595436, EPI_ISL_2595437, EPI_ISL_2595438, EPI_ISL_2595439, EPI_ISL_2595440			
see above	Epidemiology and Demography, KEMRI - Wellcome Trust Research Programme, Centre for Geographic Medicine Research	Epidemiology and Demography, KEMRI - Wellcome Trust Research Programme, Centre for Geographic Medicine Research	Oketch,J.W., Kamau,E., Otieno,J.R., Mwema,A., Lewa,C., Agoti,C.N. and Nokes,J.D.
EPI_ISL_2595441	Department for Infectious Diseases Virology, University of Heidelberg	Department for Infectious Diseases Virology, University of Heidelberg	Tabatabai,J., Thielen,A., Lehners,N., Daeumer,M. and Schnitzler,P.
EPI_ISL_2595442	Epidemiology and Demography, KEMRI - Wellcome Trust Research Programme, Centre for Geographic Medicine Research	Epidemiology and Demography, KEMRI - Wellcome Trust Research Programme, Centre for Geographic Medicine Research	Oketch,J.W., Kamau,E., Otieno,J.R., Mwema,A., Lewa,C., Agoti,C.N. and Nokes,J.D.
EPI_ISL_2595443	Virology Section, Department of Microbiology, King George's Medical University	Virology Section, Department of Microbiology, King George's Medical University	Singh,A.K., Jain,A., Jain,B., Dangi,T., Verma,A.K., Dwivedi,M. and Kushwaha,R.
EPI_ISL_2595444	Epidemiology and Demography, KEMRI - Wellcome Trust Research Programme, Centre for Geographic Medicine Research	Epidemiology and Demography, KEMRI - Wellcome Trust Research Programme, Centre for Geographic Medicine Research	Oketch,J.W., Kamau,E., Otieno,J.R., Mwema,A., Lewa,C., Agoti,C.N. and Nokes,J.D.
EPI_ISL_2595445	Department of Epidemiology and Demography, Kenya Medical Research Institute (KEMRI) - Wellcome Trust Research Programme	Department of Epidemiology and Demography, Kenya Medical Research Institute (KEMRI) - Wellcome Trust Research Programme	Otieno,J.R., Kamau,E.M., Agoti,C.N., Lewa,C., Bett,A., Ngama,M., Cane,P.A. and Nokes,J.D.
EPI_ISL_2595446, EPI_ISL_2595447, EPI_ISL_2595448, EPI_ISL_2595449, EPI_ISL_2595450, EPI_ISL_2595451, EPI_ISL_2595452, EPI_ISL_2595453, EPI_ISL_2595454	Epidemiology and Demography, KEMRI - Wellcome Trust Research Programme, Centre for Geographic Medicine Research	Epidemiology and Demography, KEMRI - Wellcome Trust Research Programme, Centre for Geographic Medicine Research	Oketch,J.W., Kamau,E., Otieno,J.R., Mwema,A., Lewa,C., Agoti,C.N. and Nokes,J.D.
EPI_ISL_2595455, EPI_ISL_2595456	Department of Respiratory Medicine, Children's Hospital of Chongqing Medical University	Department of Respiratory Medicine, Children's Hospital of Chongqing Medical University	Ren,L. and Liu,E.
EPI_ISL_2595457	Virology, Tohoku University Graduate School of Medicine	Virology, Tohoku University Graduate School of Medicine	Malasao,R., Okamoto,M., Chaimongkol,N., Imamura,T., Tohma,K., Dapat,I., Dapat,C., Suzuki,A., Saito,M., Saito,M., Tamaki,R., Segubre-Mercado,E., Igoy,M.A.U., Lupisan,S., Olveda,R. and Oshitani,H.
EPI_ISL_2595458, EPI_ISL_2595459, EPI_ISL_2595460, EPI_ISL_2595461, EPI_ISL_2595462, EPI_ISL_2595463, EPI_ISL_2595464, EPI_ISL_2595465, EPI_ISL_2595466, EPI_ISL_2595467, EPI_ISL_2595468, EPI_ISL_2595469, EPI_ISL_2595470, EPI_ISL_2595471, EPI_ISL_2595472			
see above	Epidemiology and Demography, KEMRI - Wellcome Trust Research Programme, Centre for Geographic Medicine Research	Epidemiology and Demography, KEMRI - Wellcome Trust Research Programme, Centre for Geographic Medicine Research	Oketch,J.W., Kamau,E., Otieno,J.R., Mwema,A., Lewa,C., Agoti,C.N. and Nokes,J.D.
EPI_ISL_2595473	Department of Epidemiology and Demography, Kenya Medical Research Institute (KEMRI) - Wellcome Trust Research Programme	Department of Epidemiology and Demography, Kenya Medical Research Institute (KEMRI) - Wellcome Trust Research Programme	Otieno,J.R., Kamau,E.M., Agoti,C.N., Lewa,C., Bett,A., Ngama,M., Cane,P.A. and Nokes,J.D.
EPI_ISL_2595474, EPI_ISL_2595475	Epidemiology and Demography, KEMRI - Wellcome Trust Research Programme, Centre for Geographic Medicine Research	Epidemiology and Demography, KEMRI - Wellcome Trust Research Programme, Centre for Geographic Medicine Research	Oketch,J.W., Kamau,E., Otieno,J.R., Mwema,A., Lewa,C., Agoti,C.N. and Nokes,J.D.
EPI_ISL_2595476	Department of Epidemiology and Demography, Kenya Medical Research Institute (KEMRI) - Wellcome Trust Research Programme	Department of Epidemiology and Demography, Kenya Medical Research Institute (KEMRI) - Wellcome Trust Research Programme	Otieno,J.R., Kamau,E.M., Agoti,C.N., Lewa,C., Bett,A., Ngama,M., Cane,P.A. and Nokes,J.D.
EPI_ISL_2595477	Marie Bashir Institute for Infectious Diseases and Biosecurity & Sydney	Marie Bashir Institute for Infectious Diseases and Biosecurity & Sydney	Eden,J.-S., Kok,J., Dwyer,D.E., Fernandez,M., Carter,I. and Holmes,E.C.

	Medical School, The University of Sydney, Westmead Institute for Medical Research	Medical School, The University of Sydney, Westmead Institute for Medical Research	
EPI_ISL_2595478	Department of Respiratory Medicine, Children's Hospital of Chongqing Medical University	Department of Respiratory Medicine, Children's Hospital of Chongqing Medical University	Ren,L. and Liu,E.
EPI_ISL_2595479, EPI_ISL_2595480, EPI_ISL_2595481, EPI_ISL_2595482, EPI_ISL_2595483, EPI_ISL_2595484, EPI_ISL_2595485, EPI_ISL_2595486, EPI_ISL_2595487, EPI_ISL_2595488, EPI_ISL_2595489, EPI_ISL_2595490, EPI_ISL_2595491, EPI_ISL_2595492, EPI_ISL_2595493, EPI_ISL_2595494, EPI_ISL_2595495, EPI_ISL_2595496, EPI_ISL_2595497, EPI_ISL_2595498			
see above	Epidemiology and Demography, KEMRI - Wellcome Trust Research Programme, Centre for Geographic Medicine Research	Epidemiology and Demography, KEMRI - Wellcome Trust Research Programme, Centre for Geographic Medicine Research	Oketch,J.W., Kamau,E., Otieno,J.R., Mwema,A., Lewa,C., Agoti,C.N. and Nokes,J.D.
EPI_ISL_2595499	Department of Epidemiology and Demography, Kenya Medical Research Institute (KEMRI) - Wellcome Trust Research Programme	Department of Epidemiology and Demography, Kenya Medical Research Institute (KEMRI) - Wellcome Trust Research Programme	Otieno,J.R., Kamau,E.M., Agoti,C.N., Lewa,C., Bett,A., Ngama,M., Cane,P.A. and Nokes,J.D.
EPI_ISL_2595500	Virology, Tohoku University Graduate School of Medicine	Virology, Tohoku University Graduate School of Medicine	Malasao,R., Okamoto,M., Chaimongkol,N., Imamura,T., Tohma,K., Dapat,I., Dapat,C., Suzuki,A., Saito,M., Saito,M., Tamaki,R., Segubre-Mercado,E., Igoy,M.A.U., Lupisan,S., Olveda,R. and Oshitani,H.
EPI_ISL_2595501	Laboratory of Virology, Capital Institute of Pediatrics	Laboratory of Virology, Capital Institute of Pediatrics	Cui,G., Zhu,R., Qian,Y., Deng,J., Zhao,L., Sun,Y. and Wang,F.
EPI_ISL_2595502, EPI_ISL_2595503, EPI_ISL_2595504, EPI_ISL_2595505, EPI_ISL_2595506, EPI_ISL_2595507, EPI_ISL_2595508	Epidemiology and Demography, KEMRI - Wellcome Trust Research Programme, Centre for Geographic Medicine Research	Epidemiology and Demography, KEMRI - Wellcome Trust Research Programme, Centre for Geographic Medicine Research	Oketch,J.W., Kamau,E., Otieno,J.R., Mwema,A., Lewa,C., Agoti,C.N. and Nokes,J.D.
EPI_ISL_2595509	Virology, King Institute of Preventive Medicine & Research	Virology, King Institute of Preventive Medicine & Research	Sureshbabu B,V., Gunasekaran,P., Kaveri,K., Venkataraman,P., Kiruba,R., Magesh,S. and SenthilRaja,R.
EPI_ISL_2595510, EPI_ISL_2595511, EPI_ISL_2595512, EPI_ISL_2595513, EPI_ISL_2595514, EPI_ISL_2595515, EPI_ISL_2595516, EPI_ISL_2595517, EPI_ISL_2595518, EPI_ISL_2595519, EPI_ISL_2595520, EPI_ISL_2595521, EPI_ISL_2595522, EPI_ISL_2595523, EPI_ISL_2595524			
see above	Epidemiology and Demography, KEMRI - Wellcome Trust Research Programme, Centre for Geographic Medicine Research	Epidemiology and Demography, KEMRI - Wellcome Trust Research Programme, Centre for Geographic Medicine Research	Oketch,J.W., Kamau,E., Otieno,J.R., Mwema,A., Lewa,C., Agoti,C.N. and Nokes,J.D.
EPI_ISL_2595525	Central Laboratory, Guangzhou Women and Children's Medical Center	Central Laboratory, Guangzhou Women and Children's Medical Center	Xie,J.H., Zhu,B., Zhong,J.Y., Chen,Y. and Zhang,Y.Y.
EPI_ISL_2595526, EPI_ISL_2595527, EPI_ISL_2595528, EPI_ISL_2595529, EPI_ISL_2595530	Epidemiology and Demography, KEMRI - Wellcome Trust Research Programme, Centre for Geographic Medicine Research	Epidemiology and Demography, KEMRI - Wellcome Trust Research Programme, Centre for Geographic Medicine Research	Oketch,J.W., Kamau,E., Otieno,J.R., Mwema,A., Lewa,C., Agoti,C.N. and Nokes,J.D.
EPI_ISL_2595531	Virology, Noguchi Memorial Institute for Medical Research	Virology, Noguchi Memorial Institute for Medical Research	Obodai,E., Odoom,J.K., Adiku,T., Goka,B., Biere,B., Wolff,T., Schweiger,B. and Reiche,J.
EPI_ISL_2595532, EPI_ISL_2595533, EPI_ISL_2595534	Epidemiology and Demography, KEMRI - Wellcome Trust Research Programme, Centre for Geographic Medicine Research	Epidemiology and Demography, KEMRI - Wellcome Trust Research Programme, Centre for Geographic Medicine Research	Oketch,J.W., Kamau,E., Otieno,J.R., Mwema,A., Lewa,C., Agoti,C.N. and Nokes,J.D.
EPI_ISL_2595535	Virology, Noguchi Memorial Institute for Medical Research	Virology, Noguchi Memorial Institute for Medical Research	Obodai,E., Odoom,J.K., Adiku,T., Goka,B., Biere,B., Wolff,T., Schweiger,B. and Reiche,J.
EPI_ISL_2595536	Epidemiology and Demography, KEMRI - Wellcome Trust Research Programme, Centre for Geographic Medicine Research	Epidemiology and Demography, KEMRI - Wellcome Trust Research Programme, Centre for Geographic Medicine Research	Oketch,J.W., Kamau,E., Otieno,J.R., Mwema,A., Lewa,C., Agoti,C.N. and Nokes,J.D.
EPI_ISL_2595537	Department for Infectious Diseases Virology, University of Heidelberg	Department for Infectious Diseases Virology, University of Heidelberg	Tabatabai,J., Thielen,A., Lehners,N., Daeumer,M. and Schnitzler,P.
EPI_ISL_2595538, EPI_ISL_2595539	Department of Clinical Laboratory, Fujian Provincial Hospital	Department of Clinical Laboratory, Fujian Provincial Hospital	Su,Y., Wu,Y., Tian,R. and Liang,G.
EPI_ISL_2595540, EPI_ISL_2595541	Epidemiology and Demography Department, KEMRI-Wellcome Trust Research Programme	Epidemiology and Demography Department, KEMRI-Wellcome Trust Research Programme	Otieno,J.R., Kamau,E.M., Oketch,J.W., Ngoi,J.M., Agoti,C.N., Gichuki,A.M., Otieno,G.P., Ngama,M., Cane,P.A., Kellam,P., Cotten,M., Lemey,P. and Nokes,D.J.
EPI_ISL_2595542	Department for Infectious Diseases Virology, University of Heidelberg	Department for Infectious Diseases Virology, University of Heidelberg	Tabatabai,J., Thielen,A., Lehners,N., Daeumer,M. and Schnitzler,P.
EPI_ISL_2595543	Virology, Noguchi Memorial Institute for Medical Research	Virology, Noguchi Memorial Institute for Medical Research	Obodai,E., Odoom,J.K., Adiku,T., Goka,B., Biere,B., Wolff,T., Schweiger,B. and Reiche,J.
EPI_ISL_2595544, EPI_ISL_2595545	Epidemiology and Demography, KEMRI - Wellcome Trust Research Programme, Centre for Geographic Medicine Research	Epidemiology and Demography, KEMRI - Wellcome Trust Research Programme, Centre for Geographic Medicine Research	Oketch,J.W., Kamau,E., Otieno,J.R., Mwema,A., Lewa,C., Agoti,C.N. and Nokes,J.D.
EPI_ISL_2595546	Department of Pediatrics, Center of Excellence in Clinical Virology, Chulalongkorn	Department of Pediatrics, Center of Excellence in Clinical Virology, Chulalongkorn	Thongpan,I.
EPI_ISL_2595547	Virology Department, National Center of Microbiology, Instituto de Salud Carlos III	Virology Department, National Center of Microbiology, Instituto de Salud Carlos III	Casas,I., Calderon,A., Pozo,F., Calvo,C., Garcia-Garcia,M., Gonzalez,M. and Molinero,M.
EPI_ISL_2595548	Epidemiology and Demography Department, KEMRI-Wellcome Trust Research Programme	Epidemiology and Demography Department, KEMRI-Wellcome Trust Research Programme	Otieno,J.R., Kamau,E.M., Oketch,J.W., Ngoi,J.M., Agoti,C.N., Gichuki,A.M., Otieno,G.P., Ngama,M., Cane,P.A., Kellam,P., Cotten,M., Lemey,P. and Nokes,D.J.
EPI_ISL_2595549	Department of Epidemiology and Demography, Kenya Medical Research Institute (KEMRI) - Wellcome Trust Research Programme	Department of Epidemiology and Demography, Kenya Medical Research Institute (KEMRI) - Wellcome Trust Research Programme	Otieno,J.R., Kamau,E.M., Agoti,C.N., Lewa,C., Bett,A., Ngama,M., Cane,P.A. and Nokes,J.D.
EPI_ISL_2595550	Epidemiology and Demography Department, KEMRI-Wellcome Trust Research Programme	Epidemiology and Demography Department, KEMRI-Wellcome Trust Research Programme	Otieno,J.R., Kamau,E.M., Oketch,J.W., Ngoi,J.M., Agoti,C.N., Gichuki,A.M., Otieno,G.P., Ngama,M., Cane,P.A., Kellam,P., Cotten,M., Lemey,P. and Nokes,D.J.
EPI_ISL_2595551	Department of Epidemiology and Demography, Kenya Medical Research Institute (KEMRI) - Wellcome Trust Research Programme	Department of Epidemiology and Demography, Kenya Medical Research Institute (KEMRI) - Wellcome Trust Research Programme	Otieno,J.R., Kamau,E.M., Agoti,C.N., Lewa,C., Bett,A., Ngama,M., Cane,P.A. and Nokes,J.D.
EPI_ISL_2595552	Epidemiology and Demography Department, KEMRI-Wellcome Trust Research Programme	Epidemiology and Demography Department, KEMRI-Wellcome Trust Research Programme	Otieno,J.R., Kamau,E.M., Oketch,J.W., Ngoi,J.M., Agoti,C.N., Gichuki,A.M., Otieno,G.P., Ngama,M., Cane,P.A., Kellam,P., Cotten,M., Lemey,P. and Nokes,D.J.
EPI_ISL_2595553	Laboratory of Virology, Capital Institute of Pediatrics	Laboratory of Virology, Capital Institute of Pediatrics	Cui,G., Zhu,R., Qian,Y., Deng,J., Zhao,L., Sun,Y. and Wang,F.
EPI_ISL_2595554, EPI_ISL_2595555	Department of Clinical Laboratory, Fujian Provincial Hospital	Department of Clinical Laboratory, Fujian Provincial Hospital	Su,Y., Wu,Y., Tian,R. and Liang,G.
EPI_ISL_2595556	Virology, King Institute of Preventive Medicine & Research	Virology, King Institute of Preventive Medicine & Research	Sureshbabu B,V., Gunasekaran,P., Kaveri,K., Venkataraman,P., Kiruba,R., Magesh,S. and SenthilRaja,R.
EPI_ISL_2595557, EPI_ISL_2595558	Department of Clinical Laboratory, Fujian Provincial Hospital	Department of Clinical Laboratory, Fujian Provincial Hospital	Su,Y., Wu,Y., Tian,R. and Liang,G.
EPI_ISL_2595559, EPI_ISL_2595560, EPI_ISL_2595561, EPI_ISL_2595562	Chinese Academy of Medical Sciences & Peking Union Medical College	Chinese Academy of Medical Sciences & Peking Union Medical College	Jia,B., Xiao,Y., Wang,Y., Chen,L., Zhang,J., Ren,L. and Wang,J.
EPI_ISL_2595563	WHO WPRO Measles Regional Reference Lab, Key Laboratory of Medical Virology Ministry of Health, National Institute for Viral Disease Control and Prevention, China Center for Disease Control and Prevention	WHO WPRO Measles Regional Reference Lab, Key Laboratory of Medical Virology Ministry of Health, National Institute for Viral Disease Control and Prevention, China Center for Disease Control and Prevention	Zhang,Y., Song,J. and Xu,W.
EPI_ISL_2595564, EPI_ISL_2595565, EPI_ISL_2595566, EPI_ISL_2595567	University of Wuerzburg, Institute of Virology and Immunobiology	University of Wuerzburg, Institute of Virology and Immunobiology	Prifert,C., Hofmann,D. and Weissbrich,B.
EPI_ISL_2595568	Virology Department, Hospices Civils de Lyon	Virology Department, Hospices Civils de Lyon	Gaymard,A., Pichon,M. and Morfin,F.
EPI_ISL_2595569, EPI_ISL_2595570	Microbiology, Sanjay Gandhi Post Graduate Institute of Medical Science	Microbiology, Sanjay Gandhi Post Graduate Institute of Medical Science	Saxena,S., Singh,D., Tripathi,R. and Kushwaha,R.
EPI_ISL_2595571	Virology Department, Hospices Civils de Lyon	Virology Department, Hospices Civils de Lyon	Gaymard,A., Pichon,M. and Morfin,F.
EPI_ISL_2595572, EPI_ISL_2595573	Chinese Academy of Medical Sciences & Peking Union Medical College	Chinese Academy of Medical Sciences & Peking Union Medical College	Jia,B., Xiao,Y., Wang,Y., Chen,L., Zhang,J., Ren,L. and Wang,J.
EPI_ISL_2595574	Laboratory of Virology, Capital Institute of Pediatrics	Laboratory of Virology, Capital Institute of Pediatrics	Cui,G., Zhu,R., Qian,Y., Deng,J., Zhao,L., Sun,Y. and Wang,F.
EPI_ISL_2595575	Department of Epidemiology and Demography, Kenya Medical Research Institute (KEMRI) - Wellcome Trust Research Programme	Department of Epidemiology and Demography, Kenya Medical Research Institute (KEMRI) - Wellcome Trust Research Programme	Otieno,J.R., Kamau,E.M., Agoti,C.N., Lewa,C., Bett,A., Ngama,M., Cane,P.A. and Nokes,J.D.
EPI_ISL_2595576, EPI_ISL_2595577	Department of Clinical Laboratory, Fujian Provincial Hospital	Department of Clinical Laboratory, Fujian Provincial Hospital	Su,Y., Wu,Y., Tian,R. and Liang,G.
EPI_ISL_2595578	Department of Epidemiology and Demography, Kenya Medical Research Institute (KEMRI) - Wellcome Trust Research Programme	Department of Epidemiology and Demography, Kenya Medical Research Institute (KEMRI) - Wellcome Trust Research Programme	Otieno,J.R., Kamau,E.M., Agoti,C.N., Lewa,C., Bett,A., Ngama,M., Cane,P.A. and Nokes,J.D.
EPI_ISL_2595579	Laboratory of Virology, Capital Institute of Pediatrics	Laboratory of Virology, Capital Institute of Pediatrics	Cui,G., Zhu,R., Qian,Y., Deng,J., Zhao,L., Sun,Y. and Wang,F.
EPI_ISL_2595580	Akinobu Hibino Niigata University, International Health, Public Health	Akinobu Hibino Niigata University, International Health, Public Health	Hibino,A., Saito,R., Shoubugawa,Y. and Nakamura,Y.
EPI_ISL_2595581, EPI_ISL_2595582	Virology Department, National Center of Microbiology, Instituto de Salud Carlos III	Virology Department, National Center of Microbiology, Instituto de Salud Carlos III	Casas,I., Calderon,A., Pozo,F., Calvo,C., Garcia-Garcia,M., Gonzalez,M. and Molinero,M.
EPI_ISL_2595583	WHO WPRO Measles Regional Reference Lab, Key Laboratory of Medical Virology Ministry of Health, National Institute for Viral Disease Control and Prevention, China Center for Disease Control and Prevention	WHO WPRO Measles Regional Reference Lab, Key Laboratory of Medical Virology Ministry of Health, National Institute for Viral Disease Control and Prevention, China Center for Disease Control and Prevention	Zhang,Y., Song,J. and Xu,W.
EPI_ISL_2595584, EPI_ISL_2595585, EPI_ISL_2595586	Centre for Research And Knowledge Transfer in Biotechnology, University of Zagreb	Centre for Research And Knowledge Transfer in Biotechnology, University of Zagreb	Ivancic-Jelecki,J., Slovic,A., Ljubin-Sternak,S., Mlinaric-Galinovic,G. and Forcic,D.
EPI_ISL_2595587	Laboratory of Virology, Capital Institute of Pediatrics	Laboratory of Virology, Capital Institute of Pediatrics	Cui,G., Zhu,R., Qian,Y., Deng,J., Zhao,L., Sun,Y. and Wang,F.
EPI_ISL_2595588	Medical Virology, Faculty of Public Health, Tehran University of Medical Sciences	Medical Virology, Faculty of Public Health, Tehran University of Medical Sciences	Arjeyni,Y., Faghihloo,E. and Mokhtari Azad,T.
EPI_ISL_2595589	Laboratory of Virology, Capital Institute of Pediatrics	Laboratory of Virology, Capital Institute of Pediatrics	Cui,G., Zhu,R., Qian,Y., Deng,J., Zhao,L., Sun,Y. and Wang,F.
EPI_ISL_2595590	Department of Epidemiology and Demography, Kenya Medical Research Institute (KEMRI) - Wellcome Trust Research Programme	Department of Epidemiology and Demography, Kenya Medical Research Institute (KEMRI) - Wellcome Trust Research Programme	Otieno,J.R., Kamau,E.M., Agoti,C.N., Lewa,C., Bett,A., Ngama,M., Cane,P.A. and Nokes,J.D.

EPI_ISL_2595591, EPI_ISL_2595592	Kazuya Shirato National Institute of Infectious Diseases, Virology III	Kazuya Shirato National Institute of Infectious Diseases, Virology III	Shirato,K., Sato,K., Dapat,I., Nao,N., Omiya,S., Matsuyama,S., Takeda,M. and Nishimura,H.
EPI_ISL_2595593, EPI_ISL_2595594	Department of Epidemiology and Demography, Kenya Medical Research Institute (KEMRI) - Wellcome Trust Research Programme	Department of Epidemiology and Demography, Kenya Medical Research Institute (KEMRI) - Wellcome Trust Research Programme	Otieno,J.R., Kamau,E.M., Agoti,C.N., Lewa,C., Bett,A., Ngama,M., Cane,P.A. and Nokes,J.D.
EPI_ISL_2595595	Epidemiology and Demography Department, KEMRI-Wellcome Trust Research Programme	Epidemiology and Demography Department, KEMRI-Wellcome Trust Research Programme	Otieno,J.R., Kamau,E.M., Oketch,J.W., Ngoi,J.M., Agoti,C.N., Gichuki,A.M., Otieno,G.P., Ngama,M., Cane,P.A., Kellam,P., Cotten,M., Lemey,P. and Nokes,D.J.
EPI_ISL_2595596, EPI_ISL_2595597, EPI_ISL_2595598, EPI_ISL_2595599, EPI_ISL_2595600	Department of Epidemiology and Demography, Kenya Medical Research Institute (KEMRI) - Wellcome Trust Research Programme	Department of Epidemiology and Demography, Kenya Medical Research Institute (KEMRI) - Wellcome Trust Research Programme	Otieno,J.R., Kamau,E.M., Agoti,C.N., Lewa,C., Bett,A., Ngama,M., Cane,P.A. and Nokes,J.D.
EPI_ISL_2595601, EPI_ISL_2595602, EPI_ISL_2595603	Virology Laboratory, Dr. Ricardo Gutierrez Children Hospital	Virology Laboratory, Dr. Ricardo Gutierrez Children Hospital	Rojo,G.L., Goya,S., Orellana,M., Sancilio,A., Rodriguez Perez,A., Montali,C., Garcia,C., Sosa,L., Musto,A., Hamilton,G., Alvarez,D., Castello,A. and Viegas,M.
EPI_ISL_2595604	Kazuya Shirato National Institute of Infectious Diseases, Virology III	Kazuya Shirato National Institute of Infectious Diseases, Virology III	Shirato,K., Sato,K., Dapat,I., Nao,N., Omiya,S., Matsuyama,S., Takeda,M. and Nishimura,H.
EPI_ISL_2595605	Marie Bashir Institute for Infectious Diseases and Biosecurity & Sydney Medical School, The University of Sydney, Westmead Institute for Medical Research	Marie Bashir Institute for Infectious Diseases and Biosecurity & Sydney Medical School, The University of Sydney, Westmead Institute for Medical Research	Eden,J.-S., Kok,J., Dwyer,D.E., Fernandez,M., Carter,I. and Holmes,E.C.
EPI_ISL_2595606	Virology, King Institute of Preventive Medicine & Research	Virology, King Institute of Preventive Medicine & Research	Sureshbabu B,V., Gunasekaran,P., Kaveri,K., Venkataraman,P., Kiruba,R., Magesh,S. and SenthilRaja,R.
EPI_ISL_2595607, EPI_ISL_2595608, EPI_ISL_2595609	Marie Bashir Institute for Infectious Diseases and Biosecurity & Sydney Medical School, The University of Sydney, Westmead Institute for Medical Research	Marie Bashir Institute for Infectious Diseases and Biosecurity & Sydney Medical School, The University of Sydney, Westmead Institute for Medical Research	Eden,J.-S., Kok,J., Dwyer,D.E., Fernandez,M., Carter,I. and Holmes,E.C.
EPI_ISL_2595610	Akinobu Hibino Niigata University, International Health, Public Health	Akinobu Hibino Niigata University, International Health, Public Health	Hibino,A., Saito,R., Shoubugawa,Y. and Tanaka,T.
EPI_ISL_2595611	Division of Public Health Research, Gyeonggi Province institute of Health and Environment	Division of Public Health Research, Gyeonggi Province institute of Health and Environment	Park,E., Park,P., Huh,J., Yun,H., Lee,H., Yoon,M., Lee,S. and Ko,G.
EPI_ISL_2595612	Akinobu Hibino Niigata University, International Health, Public Health	Akinobu Hibino Niigata University, International Health, Public Health	Hibino,A., Saito,R., Shoubugawa,Y. and Nagata,N.
EPI_ISL_2595613	Akinobu Hibino Niigata University, International Health, Public Health	Akinobu Hibino Niigata University, International Health, Public Health	Hibino,A., Saito,R., Shoubugawa,Y. and Kaji,H.
EPI_ISL_2595614	Virology, Noguchi Memorial Institute for Medical Research	Virology, Noguchi Memorial Institute for Medical Research	Obodai,E., Odoom,J.K., Adiku,T., Goka,B., Biere,B., Wolff,T., Schweiger,B. and Reiche,J.
EPI_ISL_2595615, EPI_ISL_2595616	Akinobu Hibino Niigata University, International Health, Public Health	Akinobu Hibino Niigata University, International Health, Public Health	Hibino,A., Saito,R., Shoubugawa,Y. and Nagata,N.
EPI_ISL_2595617	Akinobu Hibino Niigata University, International Health, Public Health	Akinobu Hibino Niigata University, International Health, Public Health	Hibino,A., Saito,R., Shoubugawa,Y. and Kaji,H.
EPI_ISL_2595618	WHO WPRO Measles Regional Reference Lab, Key Laboratory of Medical Virology Ministry of Health, National Institute for Viral Disease Control and Prevention, China Center for Disease Control and Prevention	WHO WPRO Measles Regional Reference Lab, Key Laboratory of Medical Virology Ministry of Health, National Institute for Viral Disease Control and Prevention, China Center for Disease Control and Prevention	Zhang,Y., Song,J. and Xu,W.
EPI_ISL_2595619	Akinobu Hibino Niigata University, International Health, Public Health	Akinobu Hibino Niigata University, International Health, Public Health	Hibino,A., Saito,R., Shoubugawa,Y. and Nagata,N.
EPI_ISL_2595620	Akinobu Hibino Niigata University, International Health, Public Health	Akinobu Hibino Niigata University, International Health, Public Health	Hibino,A., Saito,R., Shoubugawa,Y. and Kaji,H.
EPI_ISL_2595621	Akinobu Hibino Niigata University, International Health, Public Health	Akinobu Hibino Niigata University, International Health, Public Health	Hibino,A., Saito,R., Shoubugawa,Y. and Nagata,N.
EPI_ISL_2595622, EPI_ISL_2595623, EPI_ISL_2595624	Akinobu Hibino Niigata University, International Health, Public Health	Akinobu Hibino Niigata University, International Health, Public Health	Hibino,A., Saito,R., Shoubugawa,Y. and Kaji,H.
EPI_ISL_2595625, EPI_ISL_2595626, EPI_ISL_2595627, EPI_ISL_2595628	Epidemiology and Demography Department, KEMRI-Wellcome Trust Research Programme	Epidemiology and Demography Department, KEMRI-Wellcome Trust Research Programme	Otieno,J.R., Kamau,E.M., Oketch,J.W., Ngoi,J.M., Agoti,C.N., Gichuki,A.M., Otieno,G.P., Ngama,M., Cane,P.A., Kellam,P., Cotten,M., Lemey,P. and Nokes,D.J.
EPI_ISL_2595629, EPI_ISL_2595630	Department of Clinical Laboratory, Fujian Provincial Hospital	Department of Clinical Laboratory, Fujian Provincial Hospital	Su,Y., Wu,Y., Tian,R. and Liang,G.
EPI_ISL_2595631	Epidemiology and Demography Department, KEMRI-Wellcome Trust Research Programme	Epidemiology and Demography Department, KEMRI-Wellcome Trust Research Programme	Otieno,J.R., Kamau,E.M., Oketch,J.W., Ngoi,J.M., Agoti,C.N., Gichuki,A.M., Otieno,G.P., Ngama,M., Cane,P.A., Kellam,P., Cotten,M., Lemey,P. and Nokes,D.J.
EPI_ISL_2595632	Akinobu Hibino Niigata University, International Health, Public Health	Akinobu Hibino Niigata University, International Health, Public Health	Hibino,A., Saito,R., Shoubugawa,Y. and Shimada,Y.
EPI_ISL_2595633, EPI_ISL_2595634	Epidemiology and Demography Department, KEMRI-Wellcome Trust Research Programme	Epidemiology and Demography Department, KEMRI-Wellcome Trust Research Programme	Otieno,J.R., Kamau,E.M., Oketch,J.W., Ngoi,J.M., Agoti,C.N., Gichuki,A.M., Otieno,G.P., Ngama,M., Cane,P.A., Kellam,P., Cotten,M., Lemey,P. and Nokes,D.J.
EPI_ISL_2595635	Akinobu Hibino Niigata University, International Health, Public Health	Akinobu Hibino Niigata University, International Health, Public Health	Hibino,A., Saito,R., Shoubugawa,Y. and Shimada,Y.
EPI_ISL_2595636	Epidemiology and Demography Department, KEMRI-Wellcome Trust Research Programme	Epidemiology and Demography Department, KEMRI-Wellcome Trust Research Programme	Otieno,J.R., Kamau,E.M., Oketch,J.W., Ngoi,J.M., Agoti,C.N., Gichuki,A.M., Otieno,G.P., Ngama,M., Cane,P.A., Kellam,P., Cotten,M., Lemey,P. and Nokes,D.J.
EPI_ISL_2595637	Akinobu Hibino Niigata University, International Health, Public Health	Akinobu Hibino Niigata University, International Health, Public Health	Hibino,A., Saito,R., Shoubugawa,Y. and Kaji,H.
EPI_ISL_2595638, EPI_ISL_2595639, EPI_ISL_2595640, EPI_ISL_2595641, EPI_ISL_2595642, EPI_ISL_2595643, EPI_ISL_2595644	Epidemiology and Demography Department, KEMRI-Wellcome Trust Research Programme	Epidemiology and Demography Department, KEMRI-Wellcome Trust Research Programme	Otieno,J.R., Kamau,E.M., Oketch,J.W., Ngoi,J.M., Agoti,C.N., Gichuki,A.M., Otieno,G.P., Ngama,M., Cane,P.A., Kellam,P., Cotten,M., Lemey,P. and Nokes,D.J.
EPI_ISL_2595645	University of Wuerzburg, Institute of Virology and Immunobiology	University of Wuerzburg, Institute of Virology and Immunobiology	Prifert,C., Hofmann,D. and Weissbrich,B.
EPI_ISL_2595646, EPI_ISL_2595647	Microbiology, Sanjay Gandhi Post Graduate Institute of Medical Science	Microbiology, Sanjay Gandhi Post Graduate Institute of Medical Science	Saxena,S., Singh,D., Tripathi,R., Dhole,T.N. and Kushwaha,R.
EPI_ISL_2595648	Pediatrics, Show-Chwan Memorial Hospital	Pediatrics, Show-Chwan Memorial Hospital	Lee,C.-Y.
EPI_ISL_2595649, EPI_ISL_2595650	Chinese Academy of Medical Sciences & Peking Union Medical College	Chinese Academy of Medical Sciences & Peking Union Medical College	Jia,B., Xiao,Y., Wang,Y., Chen,L., Zhang,J., Ren,L. and Wang,J.
EPI_ISL_2595651	Lab Medicine, UW	Lab Medicine, UW	Greninger,A.L., Makhsous,N., Kuypers,J.M., Shean,R.C. and Jerome,K.R.
EPI_ISL_2595652	Depto Microbiologia, Instituto de Ciencias Biomedicas, Universidade de Sao Paulo	Depto Microbiologia, Instituto de Ciencias Biomedicas, Universidade de Sao Paulo	Moura,F.E.A., Thomazelli,L.M., Campelo,F.S., Delfraro,A., Arbiza,J. and Durigon,E.L.
EPI_ISL_2595653, EPI_ISL_2595654, EPI_ISL_2595655	Epidemiology and Demography Department, KEMRI-Wellcome Trust Research Programme	Epidemiology and Demography Department, KEMRI-Wellcome Trust Research Programme	Otieno,J.R., Kamau,E.M., Oketch,J.W., Ngoi,J.M., Agoti,C.N., Gichuki,A.M., Otieno,G.P., Ngama,M., Cane,P.A., Kellam,P., Cotten,M., Lemey,P. and Nokes,D.J.
EPI_ISL_2595656	Microbiology, Hospital Universitari Vall d'Hebron	Microbiology, Hospital Universitari Vall d'Hebron	Gimferrer,L., Martin,Md.C., Pumarola,T. and Anton,A.
EPI_ISL_2595657, EPI_ISL_2595658	Epidemiology and Demography Department, KEMRI-Wellcome Trust Research Programme	Epidemiology and Demography Department, KEMRI-Wellcome Trust Research Programme	Otieno,J.R., Kamau,E.M., Oketch,J.W., Ngoi,J.M., Agoti,C.N., Gichuki,A.M., Otieno,G.P., Ngama,M., Cane,P.A., Kellam,P., Cotten,M., Lemey,P. and Nokes,D.J.
EPI_ISL_2595659	Department of Epidemiology and Demography, Kenya Medical Research Institute (KEMRI) - Wellcome Trust Research Programme	Department of Epidemiology and Demography, Kenya Medical Research Institute (KEMRI) - Wellcome Trust Research Programme	Otieno,J.R., Kamau,E.M., Agoti,C.N., Lewa,C., Bett,A., Ngama,M., Cane,P.A. and Nokes,J.D.
EPI_ISL_2595660, EPI_ISL_2595661, EPI_ISL_2595662, EPI_ISL_2595663, EPI_ISL_2595664, EPI_ISL_2595665	Epidemiology and Demography Department, KEMRI-Wellcome Trust Research Programme	Epidemiology and Demography Department, KEMRI-Wellcome Trust Research Programme	Otieno,J.R., Kamau,E.M., Oketch,J.W., Ngoi,J.M., Agoti,C.N., Gichuki,A.M., Otieno,G.P., Ngama,M., Cane,P.A., Kellam,P., Cotten,M., Lemey,P. and Nokes,D.J.
EPI_ISL_2595666	Department of Epidemiology and Demography, Kenya Medical Research Institute (KEMRI) - Wellcome Trust Research Programme	Department of Epidemiology and Demography, Kenya Medical Research Institute (KEMRI) - Wellcome Trust Research Programme	Otieno,J.R., Kamau,E.M., Agoti,C.N., Lewa,C., Bett,A., Ngama,M., Cane,P.A. and Nokes,J.D.
EPI_ISL_2595667	Centre for Infectious Diseases Research, Diagnostics and laboratory Surveillance, National Institute for Public Health and the Environment	Centre for Infectious Diseases Research, Diagnostics and laboratory Surveillance, National Institute for Public Health and the Environment	Meijer,A. and Overduin,P.
EPI_ISL_2595668	Department of Epidemiology and Demography, Kenya Medical Research Institute (KEMRI) - Wellcome Trust Research Programme	Department of Epidemiology and Demography, Kenya Medical Research Institute (KEMRI) - Wellcome Trust Research Programme	Otieno,J.R., Kamau,E.M., Agoti,C.N., Lewa,C., Bett,A., Ngama,M., Cane,P.A. and Nokes,J.D.
EPI_ISL_2595669, EPI_ISL_2595670	Department of Clinical Laboratory, Fujian Provincial Hospital	Department of Clinical Laboratory, Fujian Provincial Hospital	Su,Y., Wu,Y., Tian,R. and Liang,G.
EPI_ISL_2595671	Department of Epidemiology and Demography, Kenya Medical Research Institute (KEMRI) - Wellcome Trust Research Programme	Department of Epidemiology and Demography, Kenya Medical Research Institute (KEMRI) - Wellcome Trust Research Programme	Otieno,J.R., Kamau,E.M., Agoti,C.N., Lewa,C., Bett,A., Ngama,M., Cane,P.A. and Nokes,J.D.
EPI_ISL_2595672, EPI_ISL_2595673	Epidemiology and Demography Department, KEMRI-Wellcome Trust Research Programme	Epidemiology and Demography Department, KEMRI-Wellcome Trust Research Programme	Otieno,J.R., Kamau,E.M., Oketch,J.W., Ngoi,J.M., Agoti,C.N., Gichuki,A.M., Otieno,G.P., Ngama,M., Cane,P.A., Kellam,P., Cotten,M., Lemey,P. and Nokes,D.J.
EPI_ISL_2595674	Centre for Virology, Pushpagiri Institute of Medical Science and Research Centre	Centre for Virology, Pushpagiri Institute of Medical Science and Research Centre	Jose,S., Jagan,O.A., John,N.S., George,S., Chandy,S., Bai,S. and Manoharan,A.
EPI_ISL_2595675, EPI_ISL_2595676, EPI_ISL_2595677, EPI_ISL_2595678, EPI_ISL_2595679	Virology Laboratory, Dr. Ricardo Gutierrez Children Hospital	Virology Laboratory, Dr. Ricardo Gutierrez Children Hospital	Rojo,G.L., Goya,S., Orellana,M., Sancilio,A., Rodriguez Perez,A., Montali,C., Garcia,C., Sosa,L., Musto,A., Hamilton,G., Alvarez,D., Castello,A. and Viegas,M.
EPI_ISL_2595680	Department of Epidemiology and Demography, Kenya Medical Research Institute (KEMRI) - Wellcome Trust Research Programme	Department of Epidemiology and Demography, Kenya Medical Research Institute (KEMRI) - Wellcome Trust Research Programme	Otieno,J.R., Kamau,E.M., Agoti,C.N., Lewa,C., Bett,A., Ngama,M., Cane,P.A. and Nokes,J.D.
EPI_ISL_2595681	Marie Bashir Institute for Infectious Diseases and Biosecurity & Sydney Medical School, The University of Sydney, Westmead Institute for Medical Research	Marie Bashir Institute for Infectious Diseases and Biosecurity & Sydney Medical School, The University of Sydney, Westmead Institute for Medical Research	Eden,J.-S., Kok,J., Dwyer,D.E., Fernandez,M., Carter,I. and Holmes,E.C.
EPI_ISL_2595682, EPI_ISL_2595683	Virology, University of Ibadan, College of Medicine (UCH)	Virology, University of Ibadan, College of Medicine (UCH)	Ogunsemowo,O.S., Odaibo,G.N. and Olaleye,D.O.
EPI_ISL_2595684	Marie Bashir Institute for Infectious Diseases and Biosecurity & Sydney Medical School, The University of Sydney, Westmead Institute for Medical Research	Marie Bashir Institute for Infectious Diseases and Biosecurity & Sydney Medical School, The University of Sydney, Westmead Institute for Medical Research	Eden,J.-S., Kok,J., Dwyer,D.E., Fernandez,M., Carter,I. and Holmes,E.C.
EPI_ISL_2595685	Virology, University of Ibadan, College of Medicine (UCH)	Virology, University of Ibadan, College of Medicine (UCH)	Ogunsemowo,O.S., Odaibo,G.N. and Olaleye,D.O.

EPI_ISL_2595686, EPI_ISL_2595687	Centre for Infectious Diseases Research, Diagnostics and laboratory Surveillance, National Institute for Public Health and the Environment	Centre for Infectious Diseases Research, Diagnostics and laboratory Surveillance, National Institute for Public Health and the Environment	Meijer,A. and Overduin,P.
EPI_ISL_2595688	Virology, King Institute of Preventive Medicine & Research	Virology, King Institute of Preventive Medicine & Research	Sureshbabu B,V., Gunasekaran,P., Kaveri,K., Venkataraman,P., Kiruba,R., Magesh,S. and SenthilRaja,R.
EPI_ISL_2595689	Department of Epidemiology and Demography, Kenya Medical Research Institute (KEMRI) - Wellcome Trust Research Programme	Department of Epidemiology and Demography, Kenya Medical Research Institute (KEMRI) - Wellcome Trust Research Programme	Otieno,J.R., Kamau,E.M., Agoti,C.N., Lewa,C., Bett,A., Ngama,M., Cane,P.A. and Nokes,J.D.
EPI_ISL_2595690	Centre for Infectious Diseases Research, Diagnostics and laboratory Surveillance, National Institute for Public Health and the Environment	Centre for Infectious Diseases Research, Diagnostics and laboratory Surveillance, National Institute for Public Health and the Environment	Meijer,A. and Overduin,P.
EPI_ISL_2595691	J. Craig Venter Institute	J. Craig Venter Institute	Tan,G., Pickett,B., Fedorova,N., Amedeo,P., Hu,L., Christensen,J., Miller,J., Durbin,A., Williams,T., Arumemi,F., Cadiz,C., Alanis,R., Balmseda,A., Wiliams,T., Schiller,A., Patel,M., Kubale,J. and Gordon,A.
EPI_ISL_412858	Virology Laboratory, Ricardo Gutiérrez Children's Hospital	Virology Laboratory, Ricardo Gutiérrez Children's Hospital / Vanderbilt University Medical Center	Goya, Stephanie; Lucion, Maria Florencia; Juarez, Maria del Valle; Shilts, Meghan; Gentile, Angela; Mistchenko, Alicia S.; Das, Suman & Viegas, Mariana
EPI_ISL_413222	Institut Pasteur de Madagascar	Institut Pasteur de Madagascar	Jean-Michel HERAUD
EPI_ISL_413641	Bahrami Children Hospital, Tehran University of Medical Sciences	Virology Department, School of Public Health, Tehran University of Medical Sciences	Vahid Salimi, Somayeh Shatizadeh Malekshahi, Talat Mokhtari Azad
EPI_ISL_413642, EPI_ISL_413927	Bahrami Children Hospital, Tehran University of Medical Sciences	Virology Department, School of Public Health, Tehran University of Medical Sciences	Vahid Salimi, Somayeh Shatizadeh Malekshahi, Talat Mokhtari-Azad
EPI_ISL_6494783, EPI_ISL_6494784, EPI_ISL_6494785, EPI_ISL_6494787, EPI_ISL_6494788, EPI_ISL_6494792, EPI_ISL_6494793, EPI_ISL_6494794, EPI_ISL_6494796, EPI_ISL_6494800, EPI_ISL_6494805, EPI_ISL_6494806, EPI_ISL_6494807, EPI_ISL_6494808, EPI_ISL_6494809, EPI_ISL_6494966, EPI_ISL_6494967, EPI_ISL_6494970, EPI_ISL_6494971, EPI_ISL_6494973, EPI_ISL_6494974, EPI_ISL_6494975, EPI_ISL_6494976, EPI_ISL_6494977, EPI_ISL_6494979, EPI_ISL_6494980, EPI_ISL_6494981, EPI_ISL_6494983, EPI_ISL_6494984, EPI_ISL_6494985, EPI_ISL_6494988, EPI_ISL_6494991, EPI_ISL_6494992, EPI_ISL_6494993, EPI_ISL_6494995, EPI_ISL_6494996, EPI_ISL_6494997, EPI_ISL_6494998, EPI_ISL_6495001, EPI_ISL_6495003, EPI_ISL_6495004, EPI_ISL_6495007, EPI_ISL_6495008, EPI_ISL_6495009, EPI_ISL_6495010			
see above	Respiratory Virus Unit, National Infection Service, Public Health England	National Infection Service, Public Health England	Zambon M, Talts T, Ellis J, Miah S, Platt S

We gratefully acknowledge the following Authors from the Originating laboratories responsible for obtaining the specimens, as well as the Submitting laboratories where the genome data were generated and shared via GISAID, on which this research is based.

All Submitters of data may be contacted directly via www.gisaid.org

Authors are sorted alphabetically.

Accession ID	Originating Laboratory	Submitting Laboratory	Authors
EPI_ISL_11901829	UK NEQAS	UK NEQAS	Sanjiv Rughooputh
EPI_ISL_15750104, EPI_ISL_15750105, EPI_ISL_15750106, EPI_ISL_15750119, EPI_ISL_15750124	Virology, Research Institute for Tropical Medicine	Virology, Research Institute for Tropical Medicine	Calaor-Morin,J., Arguelles,V.L., Foronda,J.L., Tan,A., Lagamayo,E., Dapat,C., Lupisan,S., Morin,J.C., Foronda,J.L.M.
EPI_ISL_15751216, EPI_ISL_15751217	Molecular Virology, Johns Hopkins School of Medicine	Molecular Virology, Johns Hopkins School of Medicine	Fall A, Elawar F, Hodcroft EB, Jallow MM, Toure CT, Barry MA, Kiori DE, Sy S, Diaw Y, Goudiaby D, Niang MN, Dia N, Fall,A., Jallow,M.M., Toure,C.T., Kiori,D.E., sy,S., Diaw,Y., Goudiaby,D., Niang,M.N., Dia,N.
EPI_ISL_15751244, EPI_ISL_15751245, EPI_ISL_15751246, EPI_ISL_15751247, EPI_ISL_15751248, EPI_ISL_15751249, EPI_ISL_15751250, EPI_ISL_15751251, EPI_ISL_15751252, EPI_ISL_15751283, EPI_ISL_15751285, EPI_ISL_15751291, EPI_ISL_15751292, EPI_ISL_15751293, EPI_ISL_15751302, EPI_ISL_15751305, EPI_ISL_15751306, EPI_ISL_15751307, EPI_ISL_15751308, EPI_ISL_15751309, EPI_ISL_15751310, EPI_ISL_15751312, EPI_ISL_15751313, EPI_ISL_15751314, EPI_ISL_15751316, EPI_ISL_15751317, EPI_ISL_15751318, EPI_ISL_15751319, EPI_ISL_15751320, EPI_ISL_15751321, EPI_ISL_15751322, EPI_ISL_15751331, EPI_ISL_15751335, EPI_ISL_15751337, EPI_ISL_15751338, EPI_ISL_15751339, EPI_ISL_15751340, EPI_ISL_15751341, EPI_ISL_15751345, EPI_ISL_15751346, EPI_ISL_15751347, EPI_ISL_15751348, EPI_ISL_15751349, EPI_ISL_15751350, EPI_ISL_15751351, EPI_ISL_15751352, EPI_ISL_15751353, EPI_ISL_15751354, EPI_ISL_15751355, EPI_ISL_15751356, EPI_ISL_15751357, EPI_ISL_15751358, EPI_ISL_15751359, EPI_ISL_15751360			
see above	Center for Virus Research, KEMRI	Center for Virus Research, KEMRI	Wangui,J.M., Nokes,J.D., Opot,B.H., Opanda,S.M., Agoti,C.N., Otieno,J.R., Bulimo,W.D., Wangui,J.M., Nokes,J.D., Opot,B.H., Opanda,S.M., Agoti,C.N., Otieno,J.R., Bulimo,W.D.
EPI_ISL_15752010, EPI_ISL_15752011, EPI_ISL_15752012, EPI_ISL_15752013, EPI_ISL_15752014, EPI_ISL_15752015, EPI_ISL_15752016, EPI_ISL_15752017, EPI_ISL_15752043, EPI_ISL_15752044, EPI_ISL_15752045, EPI_ISL_15752046, EPI_ISL_15752047, EPI_ISL_15752048, EPI_ISL_15752049, EPI_ISL_15752050, EPI_ISL_15752051, EPI_ISL_15752052, EPI_ISL_15752053, EPI_ISL_15752054, EPI_ISL_15752055, EPI_ISL_15752056, EPI_ISL_15752057, EPI_ISL_15752058, EPI_ISL_15752059, EPI_ISL_15752060, EPI_ISL_15752061, EPI_ISL_15752062, EPI_ISL_15752063, EPI_ISL_15752064, EPI_ISL_15752065, EPI_ISL_15752066, EPI_ISL_15752067, EPI_ISL_15752068, EPI_ISL_15752069, EPI_ISL_15752070, EPI_ISL_15752071, EPI_ISL_15752072, EPI_ISL_15752073, EPI_ISL_15752074, EPI_ISL_15752075, EPI_ISL_15752076, EPI_ISL_15752077, EPI_ISL_15752078, EPI_ISL_15752079, EPI_ISL_15752080, EPI_ISL_15752081, EPI_ISL_15752082			
see above	Respiratory Viruses Branch, Division of Viral Diseases	Respiratory Viruses Branch, Division of Viral Diseases	Wang,L., Ng,T.F.F., Castro,C.J., Marine,R.L., Magana,L.C., Esona,M., Peret,T.C.T., Thornburg,N.J., Wang,L., Ng,T.F.F., Castro,C.J., Marine,R.L., Magana,L.C., Esona,M., Peret,T.C.T., Thornburg,N.J.
EPI_ISL_15752136, EPI_ISL_15752137, EPI_ISL_15752138, EPI_ISL_15752139	Virology, ICDDR	Virology, ICDDR	Rahman,M.Z., Hossain,M.E., Sumiya,M.K., Islam,M.M., Begum,M.N., Hoque,A.F., Uddin,K.M.M., Hassan,M.Z., Rahman,M., Goswami,D.R., Brooks,W.A., Rahman,M.Z., Hossain,M.E., Sumiya,M.K., Islam,M.M., Begum,M.N., Hoque,A.F., Uddin,K.M.M., Hassan,M.Z., Rahman,M., Goswami,D.R., Brooks,W.A.
EPI_ISL_15753583	Clinical Laboratory, Zhongshan Center for Disease Control and Prevention	Clinical Laboratory, Zhongshan Center for Disease Control and Prevention	Lin,J., Wu,Y., Shi,W., Yang,S., Xie,Y.
EPI_ISL_15771587, EPI_ISL_15771589, EPI_ISL_15771591, EPI_ISL_15771593, EPI_ISL_15771595, EPI_ISL_15771598, EPI_ISL_15771600, EPI_ISL_15771601, EPI_ISL_15771603, EPI_ISL_15771604, EPI_ISL_15771606, EPI_ISL_15771609, EPI_ISL_15771611, EPI_ISL_15771613, EPI_ISL_15771615			
see above	Respiratory Viruses Branch, Division of Viral Diseases, Centers for Disease Control and Prevention	Respiratory Viruses Branch, Division of Viral Diseases, Centers for Disease Control and Prevention	Wang,L., Ng,T.F.F., Castro,C.J., Marine,R.L., Magana,L.C., Esona,M., Peret,T.C.T. and Thornburg,N.J.
EPI_ISL_15820266, EPI_ISL_15820267, EPI_ISL_15820268	Respiratory Viruses Branch, Division of Viral Diseases	Respiratory Viruses Branch, Division of Viral Diseases	Wang,L., Ng,T.F.F., Castro,C.J., Marine,R.L., Magana,L.C., Esona,M., Peret,T.C.T., Thornburg,N.J., Wang,L., Ng,T.F.F., Castro,C.J., Marine,R.L., Magana,L.C., Esona,M., Peret,T.C.T., Thornburg,N.J.
EPI_ISL_17782655	Kitasato University, Infection Control and Immunology	Kitasato University, Infection Control and Immunology	Ito,T., Sawada,A., Saito,A., Ishikura,K., Kawashima,H., Nakayama,T. and Katayama,K.
EPI_ISL_17995589, EPI_ISL_17995590, EPI_ISL_17995591, EPI_ISL_17995592, EPI_ISL_17995593, EPI_ISL_17995594, EPI_ISL_17995595, EPI_ISL_17995596, EPI_ISL_17995597, EPI_ISL_17995598, EPI_ISL_17995599, EPI_ISL_17995600, EPI_ISL_17995601, EPI_ISL_17995602, EPI_ISL_17995603, EPI_ISL_17995604, EPI_ISL_17995605, EPI_ISL_17995606, EPI_ISL_17995607, EPI_ISL_17995608, EPI_ISL_17995609, EPI_ISL_17995610, EPI_ISL_17995611, EPI_ISL_17995612, EPI_ISL_17995613, EPI_ISL_17995614, EPI_ISL_17995615, EPI_ISL_17995616, EPI_ISL_17995617, EPI_ISL_17995618, EPI_ISL_17995619, EPI_ISL_17995620, EPI_ISL_17995621, EPI_ISL_17995622, EPI_ISL_17995623, EPI_ISL_17995624			
see above	Robert Koch-Institute Nationales Referenzzentrum für Influenza	Robert Koch-Institute Nationales Referenzzentrum für Influenza	Sophie Kondgen, Janine Reiche
EPI_ISL_18090606, EPI_ISL_18090678, EPI_ISL_18090682	Westmead Institute for Medical Research & Sydney Infectious Disease Institute	Westmead Institute for Medical Research & Sydney Infectious Disease Institute	Pangesti,K.N.A., Ansari,H.R., Bayoumi,A., Kesson,A.M., Hill-Cawthorne,G.A. and Abd El Ghany,M.
EPI_ISL_18334078	Kitasato University, Infection Control and Immunology, Yukie Katayama Omura Satoshi Memorial Institute	Kitasato University, Infection Control and Immunology, Yukie Katayama Omura Satoshi Memorial Institute	Takai-Todaka,R., Ito,T., Haga,K., Ishiyama,R., Igarashi,T., Katayama,Y., Nakayama,T. and Katayama,K.
EPI_ISL_2543921	Division of Immunity & Infection, The University of Birmingham, Medical School	Division of Immunity & Infection, The University of Birmingham, Medical School	Scott,P.D., Ochola,R., Ngama,M., Okiro,E.A., Nokes,D.J., Medley,G.F. and Cane,P.A.
EPI_ISL_2543923, EPI_ISL_2543924, EPI_ISL_2543925	Department of Epidemiology and Demography, Kenya Medical Research Institute (KEMRI) - Wellcome Trust Research Programme	Department of Epidemiology and Demography, Kenya Medical Research Institute (KEMRI) - Wellcome Trust Research Programme	Otieno,J.R., Agoti,C.N., Gitahi,C.W., Bett,A., Ngama,M., Medley,G.F., Cane,P.A. and Nokes,J.D.
EPI_ISL_2543942	Pediatrics, Mackay Memorial Hospital	Pediatrics, Mackay Memorial Hospital	Chi,H., Hsiao,K.-L., Weng,L.-C., Chiu,N.-C., Huang,L.-M., Chiu,Y.-Y., Liu,C.-P. and Liu,H.-F.
EPI_ISL_2543944, EPI_ISL_2543945, EPI_ISL_2543946	Department of Microbiology & Immunology, Rega Institute for Medical Research, University of Leuven, Laboratory of Clinical and Epidemiological Virology	Department of Microbiology & Immunology, Rega Institute for Medical Research, University of Leuven, Laboratory of Clinical and Epidemiological Virology	Zlateva,K.T., Lemey,P., Vandamme,A.-M. and Van Ranst,M.
EPI_ISL_2543947, EPI_ISL_2543948	Department of Microbiology and Immunology, Laboratory of Clinical and Epidemiological Virology, Rega Institute for Medical Research	Department of Microbiology and Immunology, Laboratory of Clinical and Epidemiological Virology, Rega Institute for Medical Research	Zlateva,K.T., Vijgen,L., Dekeersmaeker,N., Naranjo,C. and Van Ranst,M.
EPI_ISL_2543949	Medical Microbiology, University Medical Center Utrecht	Medical Microbiology, University Medical Center Utrecht	Tan,L., Lemey,P., Viveen,M. and Coenjaerts,F.E.J.
EPI_ISL_2543950	Immunology and Microbiology, Rega Institute for Medical Research	Immunology and Microbiology, Rega Institute for Medical Research	Houspie,L., Lemey,P., Keyaerts,E., Reijmen,E., Vergote,V., Vankeerberghen,A., Vaeyens,F., De Beenhouwer,H. and Van Ranst,M.
EPI_ISL_2543951	Department of Infectious, Parasitic and Immunomediated Diseases, Istituto Superiore di Sanita'	Department of Infectious, Parasitic and Immunomediated Diseases, Istituto Superiore di Sanita'	Montieri,S., Puzelli,S., Ciccozzi,M., Calzoletti,L. and Di Martino,A.
EPI_ISL_2543955	Medical Microbiology, University Medical Center Utrecht	Medical Microbiology, University Medical Center Utrecht	Tan,L., Lemey,P., Viveen,M. and Coenjaerts,F.
EPI_ISL_2543956	Medical Microbiology, University Medical Center Utrecht	Medical Microbiology, University Medical Center Utrecht	Tan,L., Lemey,P., Viveen,M. and Coenjaerts,F.E.J.
EPI_ISL_2543957	Medical Microbiology, University Medical Center Utrecht	Medical Microbiology, University Medical Center Utrecht	Tan,L., Lemey,P., Viveen,M. and Coenjaerts,F.
EPI_ISL_2543958, EPI_ISL_2543959, EPI_ISL_2543960	Medical Microbiology, University Medical Center Utrecht	Medical Microbiology, University Medical Center Utrecht	Tan,L., Lemey,P., Viveen,M. and Coenjaerts,F.E.J.
EPI_ISL_2543963	Microbiology and Clinical Microbiology, Cerrahpasa Medical Faculty of Istanbul University	Microbiology and Clinical Microbiology, Cerrahpasa Medical Faculty of Istanbul University	Midilli,K., Yilmaz,G., Turkoglu,S., Kuskucu,M.A., Iskonova,B., Yalcin,I.N. and Altas,K.
EPI_ISL_2543964, EPI_ISL_2543965	Centre for Infectious Diseases, University of Edinburgh	Centre for Infectious Diseases, University of Edinburgh	Gaunt,E.R.
EPI_ISL_2543967	J. Craig Venter Institute	J. Craig Venter Institute	Lorenzi,H., Town,C., Halpin,R., Bera,J., Ransier,A., Fedorova,N., Stockwell,T., Amedeo,P., Appalla,L., Bishop,B., Edworthy,P., Gupta,N., Hoover,J., Katzel,D., Li,K., Schobel,S., Shrivastava,S., Thovarai,V., Wang,S., Rebuffo-Scheer,C., Fan,J., He,J., Kehl,S.C., Lederboer,N., Jurgens,L.A., Bose,M.E., Beck,E.T., Kumar,S., Noyola,D.E., Wentworth,D.E. and Henrickson,K.J.
EPI_ISL_2543969	Mirus Bio Corporation	Mirus Bio Corporation	Brazas,R.M.
EPI_ISL_2543976	J. Craig Venter Institute	J. Craig Venter Institute	Das,S.R., Halpin,R.A., Shilts,M., Puri,V., Akopov,A., Fedorova,N., Stockwell,T., Amedeo,P., Bishop,B., Katzel,D., Schobel,S., Shrivastava,S. and Hartert,T.
EPI_ISL_2543991, EPI_ISL_2543992, EPI_ISL_2543993, EPI_ISL_2543994, EPI_ISL_2543995	J. Craig Venter Institute	J. Craig Venter Institute	Das,S.R., Halpin,R.A., Puri,V., Akopov,A., Fedorova,N., Tsitrin,T., Stockwell,T., Amedeo,P., Bishop,B., Gupta,N., Hoover,J., Katzel,D., Schobel,S., Shrivastava,S., Wentworth,D.E. and Caserta,M.
EPI_ISL_2543996, EPI_ISL_2543997, EPI_ISL_2543998, EPI_ISL_2543999, EPI_ISL_2544000, EPI_ISL_2544001, EPI_ISL_2544002, EPI_ISL_2544003	J. Craig Venter Institute	J. Craig Venter Institute	Das,S.R., Halpin,R.A., Puri,V., Akopov,A., Fedorova,N., Stockwell,T., Amedeo,P., Bishop,B., Katzel,D., Schobel,S., Shrivastava,S., Wentworth,D.E. and Caserta,M.
EPI_ISL_2544004, EPI_ISL_2544005, EPI_ISL_2544006, EPI_ISL_2544007, EPI_ISL_2544008, EPI_ISL_2544009, EPI_ISL_2544010, EPI_ISL_2544011, EPI_ISL_2544012, EPI_ISL_2544013	J. Craig Venter Institute	J. Craig Venter Institute	Das,S.R., Halpin,R.A., Puri,V., Akopov,A., Fedorova,N., Stockwell,T., Amedeo,P., Bishop,B., Katzel,D., Schobel,S., Shrivastava,S., Hall,C.B., Tesini,B.L., Schnabel,K.C., Walsh,E.E. and Caserta,M.
EPI_ISL_2544014, EPI_ISL_2544015, EPI_ISL_2544016, EPI_ISL_2544017, EPI_ISL_2544018, EPI_ISL_2544019	J. Craig Venter Institute	J. Craig Venter Institute	Shabman,R., Fedorova,N., Puri,V., Shrivastava,S., Amedeo,P., Isom,R., Hu,L., Pickett,B., Novotny,M., Durbin,A., Rocchi,I., Williams,T., Hall,C.B., Tesini,B.L., Schnabel,K.C., Walsh,E.E. and Caserta,M.
EPI_ISL_2544020	Broad Institute of MIT & Harvard	Broad Institute of MIT & Harvard	Newman,R.M., Zody,M.C., DeVincenzo,J.P., Grad,Y., Lipsitch,M., Murphy,R., Fitzgerald,M., Young,S., Gargeya,S., Poon,T.W., Charlebois,P., Weiner,B., Yang,X., Piper,M.E., McCowan,C., Ireland,A., Levin,J., Malboeuf,C., Qu,J., Chapman,S.B., Murphy,C., Wortman,J., Nusbaum,C. and Birren,B.
EPI_ISL_2544025	Infectious Diseases, St. Jude Children's Research Hospital	Infectious Diseases, St. Jude Children's Research Hospital	DeVincenzo,J., Jones,B.G., Kim,Y.-I. and Hurwitz,J.L.
EPI_ISL_2544026, EPI_ISL_2544027, EPI_ISL_2544028	J. Craig Venter Institute	J. Craig Venter Institute	Lorenzi,H., Halpin,R., Nguyen,A., Fedorova,N., Overton,L., Stockwell,T., Amedeo,P., Bishop,B., Chen,H., Edworthy,P., Gupta,N., Katzel,D., Li,K., Schobel,S., Shrivastava,S., Thovarai,V., Wang,S., Shapiro,C.A., Williams,J., Peebles,S., Chi,M., Wentworth,D.E. and Moore,M.L.
EPI_ISL_2544029	J. Craig Venter Institute	J. Craig Venter Institute	Lorenzi,H., Town,C., Halpin,R., Bera,J., Ransier,A., Fedorova,N., Stockwell,T., Amedeo,P., Appalla,L., Bishop,B., Edworthy,P., Gupta,N., Hoover,J., Katzel,D., Li,K., Schobel,S., Shrivastava,S., Thovarai,V., Wang,S., Rebuffo-Scheer,C., Fan,J., He,J., Kehl,S.C., Lederboer,N., Jurgens,L.A., Bose,M.E., Beck,E.T., Kumar,S., Wentworth,D.E. and Henrickson,K.J.
EPI_ISL_2544031	Pediatrics - Infectious Diseases, Medical College of Wisconsin	Pediatrics - Infectious Diseases, Medical College of Wisconsin	Rebuffo-Scheer,C., Bose,M.E., He,J., Khaja,S., Ulatowski,M., Beck,E.T., Fan,J., Kumar,S., Nelson,M.I. and Henrickson,K.J.
EPI_ISL_2544032	Molecular Virology and Microbiology, Baylor College of Medicine	Molecular Virology and Microbiology, Baylor College of Medicine	Tapia,L.I. and Piedra,P.A.
EPI_ISL_2544033	J. Craig Venter Institute	J. Craig Venter Institute	Lorenzi,H., Town,C., Halpin,R., Bera,J., Ransier,A., Fedorova,N., Stockwell,T., Amedeo,P., Appalla,L., Bishop,B., Edworthy,P., Gupta,N., Hoover,J., Katzel,D., Li,K., Schobel,S., Shrivastava,S., Thovarai,V., Wang,S., Rebuffo-Scheer,C., Fan,J., He,J., Kehl,S.C., Lederboer,N., Jurgens,L.A., Bose,M.E., Beck,E.T., Kumar,S., Kok,T., Wentworth,D.E. and Henrickson,K.J.
EPI_ISL_2544042, EPI_ISL_2544043	Virology Laboratory, Dr. Ricardo Gutierrez Children Hospital	Virology Laboratory, Dr. Ricardo Gutierrez Children Hospital	Viegas,M. and Mistchenko,A.S.
EPI_ISL_2544044	J. Craig Venter Institute	J. Craig Venter Institute	Lorenzi,H., Town,C., Halpin,R., Bera,J., Ransier,A., Fedorova,N., Stockwell,T., Amedeo,P., Appalla,L., Bishop,B., Edworthy,P., Gupta,N., Hoover,J.,

			Katzel,D., Schobel,S., Shrivastava,S., Thovarai,V., Wang,S., Rebuffo-Scheer,C., Fan,J., He,J., Kehl,S.C., Lederboer,N., Jurgens,L.A., Bose,M.E., Beck,E.T., Kumar,S., Videla,C., Wentworth,D.E. and Henrickson,K.J.
EPI_ISL_2544045, EPI_ISL_2544046	J. Craig Venter Institute	J. Craig Venter Institute	Lorenzi,H., Town,C., Halpin,R., Bera,J., Ransier,A., Fedorova,N., Stockwell,T., Amedeo,P., Appalla,L., Bishop,B., Edworthy,P., Gupta,N., Hoover,J., Katzel,D., Li,K., Schobel,S., Shrivastava,S., Thovarai,V., Wang,S., Rebuffo-Scheer,C., Fan,J., He,J., Kehl,S.C., Lederboer,N., Jurgens,L.A., Bose,M.E., Beck,E.T., Kumar,S., Videla,C., Wentworth,D.E. and Henrickson,K.J.
EPI_ISL_2544049	Virology Laboratory, Dr. Ricardo Gutierrez Children Hospital	Virology Laboratory, Dr. Ricardo Gutierrez Children Hospital	Viegas,M., Goya,S. and Mistchenko,A.S.
EPI_ISL_2544053	Depto. de Virologia, Instituto Oswaldo Cruz, FIOCRUZ, Laboratorio de Virus Respiratorios e do Sarampo	Depto. de Virologia, Instituto Oswaldo Cruz, FIOCRUZ, Laboratorio de Virus Respiratorios e do Sarampo	Machado,D.B.B., Motta,F.C., Mesquita,M.M.A., Souza,T.M.L. and Siqueira,M.M.
EPI_ISL_2544054	Depto Microbiologia, Instituto de Ciencias Biomedicas, Universidade de Sao Paulo	Depto Microbiologia, Instituto de Ciencias Biomedicas, Universidade de Sao Paulo	Moura,F.E.A., Thomazelli,L.M., Candido,E.D.O., Florencio,C.M.G.D., Pereira,S.A.R., Oliveira,F.M.S., Alves,A.A., Ocadaque,C.J. and Durigon,E.L.
EPI_ISL_2544059, EPI_ISL_2544061	J. Craig Venter Institute	J. Craig Venter Institute	Wentworth,D.E., Halpin,R.A., Bera,J., Lin,X., Fedorova,N., Tsitrin,T., McLellan,M., Stockwell,T., Amedeo,P., Bishop,B., Gupta,N., Hoover,J., Katzel,D., Schobel,S., Shrivastava,S., Garcia,J., Laguna-Torres,V.A., Leguia,M., Benavides,J.G. and Halsey,E.
EPI_ISL_2544065	Dopazo J., Centro Nacional de Biotecnologia - CSIC, BioInformatica, Universidad Autonoma	Dopazo J., Centro Nacional de Biotecnologia - CSIC, BioInformatica, Universidad Autonoma	Dopazo,J.
EPI_ISL_2558739, EPI_ISL_2558740, EPI_ISL_2558741, EPI_ISL_2558742, EPI_ISL_2558743, EPI_ISL_2558744, EPI_ISL_2558745, EPI_ISL_2558746, EPI_ISL_2558747, EPI_ISL_2558748, EPI_ISL_2558749, EPI_ISL_2558750, EPI_ISL_2558751, EPI_ISL_2558752, EPI_ISL_2558753, EPI_ISL_2558759, EPI_ISL_2558760, EPI_ISL_2558761, EPI_ISL_2558762, EPI_ISL_2558763, EPI_ISL_2558764, EPI_ISL_2558765, EPI_ISL_2558766, EPI_ISL_2558767, EPI_ISL_2558768, EPI_ISL_2558769, EPI_ISL_2558771, EPI_ISL_2558772, EPI_ISL_2558773, EPI_ISL_2558774			
see above	Clinical Virology Laboratory, CEMIC University Hospital	Clinical Virology Laboratory, CEMIC University Hospital	Galiano,M.C., Palomo,C. and Videla,C.M.
EPI_ISL_2558775	Dept Microbiology, Instituto de Ciencias Biomedicas, Universidade de Sao Paulo	Dept Microbiology, Instituto de Ciencias Biomedicas, Universidade de Sao Paulo	Moura,F.E.A., Perdigao,A.C.B., Ribeiro,J.F., Florencio,C.M.G.D., Oliveira,F.M.S., Pereira,S.A.R., Botosso,V.F., Siqueira,M.M., Thomazelli,L.M., Caldeira,R.N., Oliveira,D.B.L. and Durigon,E.L.
EPI_ISL_2570818	WHO WPRO Measles Regional Reference Lab, Key Laboratory of Medical Virology Ministry of Health, National Institute for Viral Disease Control and Prevention, China Center for Disease Control and Prevention	WHO WPRO Measles Regional Reference Lab, Key Laboratory of Medical Virology Ministry of Health, National Institute for Viral Disease Control and Prevention, China Center for Disease Control and Prevention	Song,J., Zhang,Y. and Xu,W.
EPI_ISL_2570844	Pediatrics, Mackay Memorial Hospital	Pediatrics, Mackay Memorial Hospital	Chi,H., Liu,H.F., Weng,L.C., Wang,N.Y., Chiang,C.L., Chiu,Y.Y., Huang,G.J., Cheng,Y.Y., Chiu,N.C., Hsieh,W.S. and Huang,L.M.
EPI_ISL_2570873	Department of Clinical Laboratory, Fujian Provincial Hospital	Department of Clinical Laboratory, Fujian Provincial Hospital	Su,Y., Wu,Y., Tian,R. and Liang,G.
EPI_ISL_2571422, EPI_ISL_2571423, EPI_ISL_2571424, EPI_ISL_2571425, EPI_ISL_2571426, EPI_ISL_2571427, EPI_ISL_2571428, EPI_ISL_2571429, EPI_ISL_2571430, EPI_ISL_2571431, EPI_ISL_2571533, EPI_ISL_2571535			
see above	Pediatrics, Mackay Memorial Hospital	Pediatrics, Mackay Memorial Hospital	Chi,H., Liu,H.F., Weng,L.C., Wang,N.Y., Chiang,C.L., Chiu,Y.Y., Huang,G.J., Cheng,Y.Y., Chiu,N.C., Hsieh,W.S. and Huang,L.M.
EPI_ISL_2571538, EPI_ISL_2571539, EPI_ISL_2571540, EPI_ISL_2571541	Dina J., University Hospital of Caen, Virology	Dina J., University Hospital of Caen, Virology	Dina,J.
EPI_ISL_2571542	WHO WPRO Measles Regional Reference Lab, Key Laboratory of Medical Virology Ministry of Health, National Institute for Viral Disease Control and Prevention, China Center for Disease Control and Prevention	WHO WPRO Measles Regional Reference Lab, Key Laboratory of Medical Virology Ministry of Health, National Institute for Viral Disease Control and Prevention, China Center for Disease Control and Prevention	Song,J., Zhang,Y. and Xu,W.
EPI_ISL_2571559	Centre de Recherche en Infectiologie, Universite Laval, CHUL	Centre de Recherche en Infectiologie, Universite Laval, CHUL	Papenburg,J., Carbonneau,J., Hamelin,M.-E., Isabel,S., Ohoumanne,N., Dery,P., Paes,B.A., Corbeil,J., Bergeron,M.G., De Serres,G. and Boivin,G.
EPI_ISL_2571561, EPI_ISL_2571562	Dina J., University Hospital of Caen, Virology	Dina J., University Hospital of Caen, Virology	Dina,J.
EPI_ISL_2571563, EPI_ISL_2571564	WHO WPRO Measles Regional Reference Lab, Key Laboratory of Medical Virology Ministry of Health, National Institute for Viral Disease Control and Prevention, China Center for Disease Control and Prevention	WHO WPRO Measles Regional Reference Lab, Key Laboratory of Medical Virology Ministry of Health, National Institute for Viral Disease Control and Prevention, China Center for Disease Control and Prevention	Song,J., Zhang,Y. and Xu,W.
EPI_ISL_2571587, EPI_ISL_2571588, EPI_ISL_2571589, EPI_ISL_2571591, EPI_ISL_2571592	Pediatrics, Mackay Memorial Hospital	Pediatrics, Mackay Memorial Hospital	Chi,H., Liu,H.F., Weng,L.C., Wang,N.Y., Chiang,C.L., Chiu,Y.Y., Huang,G.J., Cheng,Y.Y., Chiu,N.C., Hsieh,W.S. and Huang,L.M.
EPI_ISL_2571635, EPI_ISL_2571636	WHO WPRO Measles Regional Reference Lab, Key Laboratory of Medical Virology Ministry of Health, National Institute for Viral Disease Control and Prevention, China Center for Disease Control and Prevention	WHO WPRO Measles Regional Reference Lab, Key Laboratory of Medical Virology Ministry of Health, National Institute for Viral Disease Control and Prevention, China Center for Disease Control and Prevention	Song,J., Zhang,Y. and Xu,W.
EPI_ISL_2571643	Pediatrics, Mackay Memorial Hospital	Pediatrics, Mackay Memorial Hospital	Chi,H., Liu,H.F., Weng,L.C., Wang,N.Y., Chiang,C.L., Chiu,Y.Y., Huang,G.J., Cheng,Y.Y., Chiu,N.C., Hsieh,W.S. and Huang,L.M.
EPI_ISL_2571667	WHO WPRO Measles Regional Reference Lab, Key Laboratory of Medical Virology Ministry of Health, National Institute for Viral Disease Control and Prevention, China Center for Disease Control and Prevention	WHO WPRO Measles Regional Reference Lab, Key Laboratory of Medical Virology Ministry of Health, National Institute for Viral Disease Control and Prevention, China Center for Disease Control and Prevention	Song,J., Zhang,Y. and Xu,W.
EPI_ISL_2571670	Center for Medical Biotechnology, VIB-UGent	Center for Medical Biotechnology, VIB-UGent	Rossey,I., Schepens,B., Van Cauwenberghe,S., Saelens,X. and Melero,J.A.
EPI_ISL_2571678	Division of Virology, National Institute of Cholera and Enteric Diseases	Division of Virology, National Institute of Cholera and Enteric Diseases	Agrawal,A.S. and Naik,T.N.
EPI_ISL_2571679, EPI_ISL_2571680, EPI_ISL_2571681	WHO WPRO Measles Regional Reference Lab, Key Laboratory of Medical Virology Ministry of Health, National Institute for Viral Disease Control and Prevention, China Center for Disease Control and Prevention	WHO WPRO Measles Regional Reference Lab, Key Laboratory of Medical Virology Ministry of Health, National Institute for Viral Disease Control and Prevention, China Center for Disease Control and Prevention	Song,J., Zhang,Y. and Xu,W.
EPI_ISL_2571694, EPI_ISL_2571695, EPI_ISL_2571696, EPI_ISL_2571697	Dina J., University Hospital of Caen, Virology	Dina J., University Hospital of Caen, Virology	Dina,J.
EPI_ISL_2571907	WHO WPRO Measles Regional Reference Lab, Key Laboratory of Medical Virology Ministry of Health, National Institute for Viral Disease Control and Prevention, China Center for Disease Control and Prevention	WHO WPRO Measles Regional Reference Lab, Key Laboratory of Medical Virology Ministry of Health, National Institute for Viral Disease Control and Prevention, China Center for Disease Control and Prevention	Song,J., Zhang,Y. and Xu,W.
EPI_ISL_2571946, EPI_ISL_2571974, EPI_ISL_2571975, EPI_ISL_2571976	Dina J., University Hospital of Caen, Virology	Dina J., University Hospital of Caen, Virology	Dina,J.
EPI_ISL_2571983	Centre de Recherche en Infectiologie, Universite Laval, CHUL	Centre de Recherche en Infectiologie, Universite Laval, CHUL	Papenburg,J., Carbonneau,J., Hamelin,M.-E., Isabel,S., Ohoumanne,N., Dery,P., Paes,B.A., Corbeil,J., Bergeron,M.G., De Serres,G. and Boivin,G.
EPI_ISL_2572049, EPI_ISL_2572094, EPI_ISL_2572095, EPI_ISL_2572096, EPI_ISL_2572103, EPI_ISL_2572104	Dina J., University Hospital of Caen, Virology	Dina J., University Hospital of Caen, Virology	Dina,J.
EPI_ISL_2572108	Centre de Recherche en Infectiologie, Universite Laval, CHUL	Centre de Recherche en Infectiologie, Universite Laval, CHUL	Papenburg,J., Carbonneau,J., Hamelin,M.-E., Isabel,S., Ohoumanne,N., Dery,P., Paes,B.A., Corbeil,J., Bergeron,M.G., De Serres,G. and Boivin,G.
EPI_ISL_2572109, EPI_ISL_2572115	Dina J., University Hospital of Caen, Virology	Dina J., University Hospital of Caen, Virology	Dina,J.
EPI_ISL_2572228	WHO WPRO Measles Regional Reference Lab, Key Laboratory of Medical Virology Ministry of Health, National Institute for Viral Disease Control and Prevention, China Center for Disease Control and Prevention	WHO WPRO Measles Regional Reference Lab, Key Laboratory of Medical Virology Ministry of Health, National Institute for Viral Disease Control and Prevention, China Center for Disease Control and Prevention	Song,J., Zhang,Y. and Xu,W.
EPI_ISL_2572582, EPI_ISL_2572583, EPI_ISL_2572584, EPI_ISL_2572585, EPI_ISL_2572586, EPI_ISL_2572587, EPI_ISL_2572588, EPI_ISL_2572589, EPI_ISL_2572590, EPI_ISL_2572591, EPI_ISL_2572693, EPI_ISL_2572695			
see above	Pediatrics, Mackay Memorial Hospital	Pediatrics, Mackay Memorial Hospital	Chi,H., Liu,H.F., Weng,L.C., Wang,N.Y., Chiang,C.L., Chiu,Y.Y., Huang,G.J., Cheng,Y.Y., Chiu,N.C., Hsieh,W.S. and Huang,L.M.
EPI_ISL_2572698, EPI_ISL_2572699, EPI_ISL_2572700, EPI_ISL_2572701	Dina J., University Hospital of Caen, Virology	Dina J., University Hospital of Caen, Virology	Dina,J.
EPI_ISL_2572702	WHO WPRO Measles Regional Reference Lab, Key Laboratory of Medical Virology Ministry of Health, National Institute for Viral Disease Control and Prevention, China Center for Disease Control and Prevention	WHO WPRO Measles Regional Reference Lab, Key Laboratory of Medical Virology Ministry of Health, National Institute for Viral Disease Control and Prevention, China Center for Disease Control and Prevention	Song,J., Zhang,Y. and Xu,W.
EPI_ISL_2572719	Centre de Recherche en Infectiologie, Universite Laval, CHUL	Centre de Recherche en Infectiologie, Universite Laval, CHUL	Papenburg,J., Carbonneau,J., Hamelin,M.-E., Isabel,S., Ohoumanne,N., Dery,P., Paes,B.A., Corbeil,J., Bergeron,M.G., De Serres,G. and Boivin,G.
EPI_ISL_2572721, EPI_ISL_2572722	Dina J., University Hospital of Caen, Virology	Dina J., University Hospital of Caen, Virology	Dina,J.
EPI_ISL_2572723, EPI_ISL_2572724	WHO WPRO Measles Regional Reference Lab, Key Laboratory of Medical Virology Ministry of Health, National Institute for Viral Disease Control and Prevention, China Center for Disease Control and Prevention	WHO WPRO Measles Regional Reference Lab, Key Laboratory of Medical Virology Ministry of Health, National Institute for Viral Disease Control and Prevention, China Center for Disease Control and Prevention	Song,J., Zhang,Y. and Xu,W.
EPI_ISL_2572747, EPI_ISL_2572748, EPI_ISL_2572749, EPI_ISL_2572751, EPI_ISL_2572752	Pediatrics, Mackay Memorial Hospital	Pediatrics, Mackay Memorial Hospital	Chi,H., Liu,H.F., Weng,L.C., Wang,N.Y., Chiang,C.L., Chiu,Y.Y., Huang,G.J., Cheng,Y.Y., Chiu,N.C., Hsieh,W.S. and Huang,L.M.
EPI_ISL_2572795, EPI_ISL_2572796	WHO WPRO Measles Regional Reference Lab, Key Laboratory of Medical Virology Ministry of Health, National Institute for Viral Disease Control and Prevention, China Center for Disease Control and Prevention	WHO WPRO Measles Regional Reference Lab, Key Laboratory of Medical Virology Ministry of Health, National Institute for Viral Disease Control and Prevention, China Center for Disease Control and Prevention	Song,J., Zhang,Y. and Xu,W.
EPI_ISL_2572803	Pediatrics, Mackay Memorial Hospital	Pediatrics, Mackay Memorial Hospital	Chi,H., Liu,H.F., Weng,L.C., Wang,N.Y., Chiang,C.L., Chiu,Y.Y., Huang,G.J., Cheng,Y.Y., Chiu,N.C., Hsieh,W.S. and Huang,L.M.
EPI_ISL_2572827	WHO WPRO Measles Regional Reference Lab, Key Laboratory of Medical Virology Ministry of Health, National Institute for Viral Disease Control and Prevention, China Center for Disease Control and Prevention	WHO WPRO Measles Regional Reference Lab, Key Laboratory of Medical Virology Ministry of Health, National Institute for Viral Disease Control and Prevention, China Center for Disease Control and Prevention	Song,J., Zhang,Y. and Xu,W.
EPI_ISL_2572830	Center for Medical Biotechnology, VIB-UGent	Center for Medical Biotechnology, VIB-UGent	Rossey,I., Schepens,B., Van Cauwenberghe,S., Saelens,X. and Melero,J.A.
EPI_ISL_2572838	Division of Virology, National Institute of Cholera and Enteric Diseases	Division of Virology, National Institute of Cholera and Enteric Diseases	Agrawal,A.S. and Naik,T.N.
EPI_ISL_2572839, EPI_ISL_2572840, EPI_ISL_2572841	WHO WPRO Measles Regional Reference Lab, Key Laboratory of Medical Virology Ministry of Health, National Institute for Viral Disease Control and Prevention, China Center for Disease Control and Prevention	WHO WPRO Measles Regional Reference Lab, Key Laboratory of Medical Virology Ministry of Health, National Institute for Viral Disease Control and Prevention, China Center for Disease Control and Prevention	Song,J., Zhang,Y. and Xu,W.
EPI_ISL_2572854, EPI_ISL_2572855, EPI_ISL_2572856, EPI_ISL_2572857	Dina J., University Hospital of Caen, Virology	Dina J., University Hospital of Caen, Virology	Dina,J.

EPI_ISL_2573067	WHO WPRO Measles Regional Reference Lab, Key Laboratory of Medical Virology Ministry of Health, National Institute for Viral Disease Control and Prevention, China Center for Disease Control and Prevention	WHO WPRO Measles Regional Reference Lab, Key Laboratory of Medical Virology Ministry of Health, National Institute for Viral Disease Control and Prevention, China Center for Disease Control and Prevention	Song,J., Zhang,Y. and Xu,W.
EPI_ISL_2573106, EPI_ISL_2573134, EPI_ISL_2573135, EPI_ISL_2573136	Dina J., University Hospital of Caen, Virology	Dina J., University Hospital of Caen, Virology	Dina,J.
EPI_ISL_2573143	Centre de Recherche en Infectiologie, Universite Laval, CHUL	Centre de Recherche en Infectiologie, Universite Laval, CHUL	Papenburg,J., Carbonneau,J., Hamelin,M.-E., Isabel,S., Ohoumanne,N., Dery,P., Paes,B.A., Corbeil,J., Bergeron,M.G., De Serres,G. and Boivin,G.
EPI_ISL_2573209, EPI_ISL_2573254, EPI_ISL_2573255, EPI_ISL_2573256, EPI_ISL_2573263, EPI_ISL_2573264	Dina J., University Hospital of Caen, Virology	Dina J., University Hospital of Caen, Virology	Dina,J.
EPI_ISL_2573268	Centre de Recherche en Infectiologie, Universite Laval, CHUL	Centre de Recherche en Infectiologie, Universite Laval, CHUL	Papenburg,J., Carbonneau,J., Hamelin,M.-E., Isabel,S., Ohoumanne,N., Dery,P., Paes,B.A., Corbeil,J., Bergeron,M.G., De Serres,G. and Boivin,G.
EPI_ISL_2573269, EPI_ISL_2573275	Dina J., University Hospital of Caen, Virology	Dina J., University Hospital of Caen, Virology	Dina,J.
EPI_ISL_2573388, EPI_ISL_2573512, EPI_ISL_2573519, EPI_ISL_2573520, EPI_ISL_2573521, EPI_ISL_2573522, EPI_ISL_2573523, EPI_ISL_2573524, EPI_ISL_2573525, EPI_ISL_2573530, EPI_ISL_2573531, EPI_ISL_2573532			
see above	WHO WPRO Measles Regional Reference Lab, Key Laboratory of Medical Virology Ministry of Health, National Institute for Viral Disease Control and Prevention, China Center for Disease Control and Prevention	WHO WPRO Measles Regional Reference Lab, Key Laboratory of Medical Virology Ministry of Health, National Institute for Viral Disease Control and Prevention, China Center for Disease Control and Prevention	Song,J., Zhang,Y. and Xu,W.
EPI_ISL_2573555, EPI_ISL_2573558, EPI_ISL_2573602, EPI_ISL_2573608	Centre for Research And Knowledge Transfer in Biotechnology, University of Zagreb	Centre for Research And Knowledge Transfer in Biotechnology, University of Zagreb	Jagusic,M., Slovic,A., Santak,M., Kosutic-Gulija,T., Ivancic-Jelecki,J. and Forcic,D.
EPI_ISL_2573645, EPI_ISL_2573646, EPI_ISL_2573647, EPI_ISL_2573648, EPI_ISL_2573649, EPI_ISL_2573651, EPI_ISL_2573652, EPI_ISL_2573653, EPI_ISL_2573654, EPI_ISL_2573655, EPI_ISL_2573656, EPI_ISL_2573657, EPI_ISL_2573658, EPI_ISL_2573659			
see above	Dina J., University Hospital of Caen, Virology	Dina J., University Hospital of Caen, Virology	Dina,J.
EPI_ISL_2575124, EPI_ISL_2575125, EPI_ISL_2575126, EPI_ISL_2575127, EPI_ISL_2575128, EPI_ISL_2575129, EPI_ISL_2575130, EPI_ISL_2575131, EPI_ISL_2575132, EPI_ISL_2575133, EPI_ISL_2575134, EPI_ISL_2575135, EPI_ISL_2575136, EPI_ISL_2575137, EPI_ISL_2575138, EPI_ISL_2575139, EPI_ISL_2575140, EPI_ISL_2575141, EPI_ISL_2575142, EPI_ISL_2575143, EPI_ISL_2575144, EPI_ISL_2575145, EPI_ISL_2575146			
see above	Pediatrics, Mackay Memorial Hospital	Pediatrics, Mackay Memorial Hospital	Chi,H., Liu,H.F., Weng,L.C., Wang,N.Y., Chiang,C.L., Chiu,Y.Y., Huang,G.J., Cheng,Y.Y., Chiu,N.C., Hsieh,W.S. and Huang,L.M.
EPI_ISL_2578661	Pediatrics - Infectious Diseases, Medical College of Wisconsin	Pediatrics - Infectious Diseases, Medical College of Wisconsin	Rebuffo-Scheer,C., Bose,M.E., He,J., Khaja,S., Ulatowski,M., Beck,E.T., Fan,J., Kumar,S., Nelson,M.I. and Henrickson,K.J.
EPI_ISL_2578735, EPI_ISL_2578746, EPI_ISL_2578751, EPI_ISL_2578752, EPI_ISL_2578753, EPI_ISL_2578754, EPI_ISL_2578755, EPI_ISL_2578756	WHO WPRO Measles Regional Reference Lab, Key Laboratory of Medical Virology Ministry of Health, National Institute for Viral Disease Control and Prevention, China Center for Disease Control and Prevention	WHO WPRO Measles Regional Reference Lab, Key Laboratory of Medical Virology Ministry of Health, National Institute for Viral Disease Control and Prevention, China Center for Disease Control and Prevention	Song,J., Zhang,Y. and Xu,W.
EPI_ISL_2578795, EPI_ISL_2578796, EPI_ISL_2578797	Centre de Recherche en Infectiologie, Universite Laval, CHUL	Centre de Recherche en Infectiologie, Universite Laval, CHUL	Papenburg,J., Carbonneau,J., Hamelin,M.-E., Isabel,S., Ohoumanne,N., Dery,P., Paes,B.A., Corbeil,J., Bergeron,M.G., De Serres,G. and Boivin,G.
EPI_ISL_2578952, EPI_ISL_2578957, EPI_ISL_2578958, EPI_ISL_2578959, EPI_ISL_2578960, EPI_ISL_2578961, EPI_ISL_2578962, EPI_ISL_2578963, EPI_ISL_2578964, EPI_ISL_2578965, EPI_ISL_2578968, EPI_ISL_2578969			
see above	Pediatrics, Mackay Memorial Hospital	Pediatrics, Mackay Memorial Hospital	Chi,H., Liu,H.F., Weng,L.C., Wang,N.Y., Chiang,C.L., Chiu,Y.Y., Huang,G.J., Cheng,Y.Y., Chiu,N.C., Hsieh,W.S. and Huang,L.M.
EPI_ISL_2578992, EPI_ISL_2579011	WHO WPRO Measles Regional Reference Lab, Key Laboratory of Medical Virology Ministry of Health, National Institute for Viral Disease Control and Prevention, China Center for Disease Control and Prevention	WHO WPRO Measles Regional Reference Lab, Key Laboratory of Medical Virology Ministry of Health, National Institute for Viral Disease Control and Prevention, China Center for Disease Control and Prevention	Song,J., Zhang,Y. and Xu,W.
EPI_ISL_2579026, EPI_ISL_2579027, EPI_ISL_2579028, EPI_ISL_2579029, EPI_ISL_2579030, EPI_ISL_2579031, EPI_ISL_2579032, EPI_ISL_2579033, EPI_ISL_2579034, EPI_ISL_2579035, EPI_ISL_2579036, EPI_ISL_2579264, EPI_ISL_2579265, EPI_ISL_2579266, EPI_ISL_2579267, EPI_ISL_2579268, EPI_ISL_2579269, EPI_ISL_2579270			
see above	Centre de Recherche en Infectiologie, Universite Laval, CHUL	Centre de Recherche en Infectiologie, Universite Laval, CHUL	Papenburg,J., Carbonneau,J., Hamelin,M.-E., Isabel,S., Ohoumanne,N., Dery,P., Paes,B.A., Corbeil,J., Bergeron,M.G., De Serres,G. and Boivin,G.
EPI_ISL_2579273, EPI_ISL_2579327, EPI_ISL_2579328, EPI_ISL_2579329	Dina J., University Hospital of Caen, Virology	Dina J., University Hospital of Caen, Virology	Dina,J.
EPI_ISL_2579369, EPI_ISL_2579370, EPI_ISL_2579371, EPI_ISL_2579372, EPI_ISL_2579373, EPI_ISL_2579374, EPI_ISL_2579375, EPI_ISL_2579377, EPI_ISL_2579378, EPI_ISL_2579379, EPI_ISL_2579385, EPI_ISL_2579386, EPI_ISL_2579389, EPI_ISL_2579390, EPI_ISL_2579391, EPI_ISL_2579410, EPI_ISL_2579411, EPI_ISL_2579412, EPI_ISL_2579414			
see above	Pediatrics, Mackay Memorial Hospital	Pediatrics, Mackay Memorial Hospital	Chi,H., Liu,H.F., Weng,L.C., Wang,N.Y., Chiang,C.L., Chiu,Y.Y., Huang,G.J., Cheng,Y.Y., Chiu,N.C., Hsieh,W.S. and Huang,L.M.
EPI_ISL_2579422	Measles Section, National Institute for Viral Disease Control and Prevention, China CDC	Measles Section, National Institute for Viral Disease Control and Prevention, China CDC	Zhang,Y., Xu,W., Shen,K., Beeler,J.A., Xie,Z., Sun,L., Lu,Q., Liu,C., Liang,G. and Anderson,L.J.
EPI_ISL_2579423, EPI_ISL_2579424, EPI_ISL_2579425	Department of Microbiology, All India Institute of Medical Sciences	Department of Microbiology, All India Institute of Medical Sciences	Parveen,S., Sullender,W.M. and Broor,S.
EPI_ISL_2579426, EPI_ISL_2579427	Pediatrics and Microbiology, University of Alabama at Birmingham	Pediatrics and Microbiology, University of Alabama at Birmingham	Coggins,W.B., Lefkowitz,E.J. and Sullender,W.M.
EPI_ISL_2579428	Dept Microbiology, Instituto de Ciencias Biomedicas, Universidade de Sao Paulo	Dept Microbiology, Instituto de Ciencias Biomedicas, Universidade de Sao Paulo	Moura,F.E.A., Perdigao,A.C.B., Ribeiro,J.F., Florencio,C.M.G.D., Oliveira,F.M.S., Pereira,S.A.R., Botosso,V.F., Siqueira,M.M., Thomazelli,L.M., Caldeira,R.N., Oliveira,D.B.L. and Durigon,E.L.
EPI_ISL_2579429	Respiratory Viruses Branch, Centers for Disease Control and Prevention	Respiratory Viruses Branch, Centers for Disease Control and Prevention	Peret,T.C.T.
EPI_ISL_2579430, EPI_ISL_2579431, EPI_ISL_2579432, EPI_ISL_2579433, EPI_ISL_2579434, EPI_ISL_2579435, EPI_ISL_2579436, EPI_ISL_2579437, EPI_ISL_2579438, EPI_ISL_2579439, EPI_ISL_2579440, EPI_ISL_2579441			
see above	Dept Microbiology, Instituto de Ciencias Biomedicas, Universidade de Sao Paulo	Dept Microbiology, Instituto de Ciencias Biomedicas, Universidade de Sao Paulo	Moura,F.E.A., Perdigao,A.C.B., Ribeiro,J.F., Florencio,C.M.G.D., Oliveira,F.M.S., Pereira,S.A.R., Botosso,V.F., Siqueira,M.M., Thomazelli,L.M., Caldeira,R.N., Oliveira,D.B.L. and Durigon,E.L.
EPI_ISL_2579442	Microbiology, Adolfo Lutz Institut	Microbiology, Adolfo Lutz Institut	Souza,M.C.O., Matsumoto,T.K., Vaz-de-Lima,L.R.A., Sato,N.N., Salgado,M.M., Requejo,H.I.Z., Hong,M.A., Barbosa,M.L., Oliveira,C.A.F., Benelli,P.C., Shimokawa,R.Y., Pecchini,R., Berezin,E., Duarte,S.P., Schvartsman,C., Durigon,E.L. and Ueda,M.
EPI_ISL_2579444	Virology Division, Public Health Laboratory Services Branch, Centre for Health Protection, Department of Health	Virology Division, Public Health Laboratory Services Branch, Centre for Health Protection, Department of Health	Mak,G.C., Lau,C.S., Chiu,D.M.Y. and Lim,W.
EPI_ISL_2579445	Respiratory Viruses Branch, Centers for Disease Control and Prevention	Respiratory Viruses Branch, Centers for Disease Control and Prevention	Peret,T.C.T.
EPI_ISL_2579446	Microbiology, Adolfo Lutz Institut	Microbiology, Adolfo Lutz Institut	Souza,M.C.O., Matsumoto,T.K., Vaz-de-Lima,L.R.A., Sato,N.N., Salgado,M.M., Requejo,H.I.Z., Hong,M.A., Barbosa,M.L., Oliveira,C.A.F., Benelli,P.C., Shimokawa,R.Y., Pecchini,R., Berezin,E., Duarte,S.P., Schvartsman,C., Durigon,E.L. and Ueda,M.
EPI_ISL_2579449	Virology Division, Public Health Laboratory Services Branch, Centre for Health Protection, Department of Health	Virology Division, Public Health Laboratory Services Branch, Centre for Health Protection, Department of Health	Mak,G.C., Lau,C.S., Chiu,D.M.Y. and Lim,W.
EPI_ISL_2579450, EPI_ISL_2579451, EPI_ISL_2579452, EPI_ISL_2579453, EPI_ISL_2579454, EPI_ISL_2579455, EPI_ISL_2579456	Respiratory Viruses Branch, Centers for Disease Control and Prevention	Respiratory Viruses Branch, Centers for Disease Control and Prevention	Peret,T.C.T.
EPI_ISL_2579461, EPI_ISL_2579462, EPI_ISL_2579463, EPI_ISL_2579464, EPI_ISL_2579465, EPI_ISL_2579466	Department of Microbiology, All India Institute of Medical Sciences	Department of Microbiology, All India Institute of Medical Sciences	Parveen,S., Sullender,W.M. and Broor,S.
EPI_ISL_2579467, EPI_ISL_2579468, EPI_ISL_2579469, EPI_ISL_2579470, EPI_ISL_2579471	Dept Microbiology, Instituto de Ciencias Biomedicas, Universidade de Sao Paulo	Dept Microbiology, Instituto de Ciencias Biomedicas, Universidade de Sao Paulo	Moura,F.E.A., Perdigao,A.C.B., Ribeiro,J.F., Florencio,C.M.G.D., Oliveira,F.M.S., Pereira,S.A.R., Botosso,V.F., Siqueira,M.M., Thomazelli,L.M., Caldeira,R.N., Oliveira,D.B.L. and Durigon,E.L.
EPI_ISL_2579472	Microbiology, Adolfo Lutz Institut	Microbiology, Adolfo Lutz Institut	Souza,M.C.O., Matsumoto,T.K., Vaz-de-Lima,L.R.A., Sato,N.N., Salgado,M.M., Requejo,H.I.Z., Hong,M.A., Barbosa,M.L., Oliveira,C.A.F., Benelli,P.C., Shimokawa,R.Y., Pecchini,R., Berezin,E., Duarte,S.P., Schvartsman,C., Durigon,E.L. and Ueda,M.
EPI_ISL_2579473	Virology Division, Public Health Laboratory Services Branch, Centre for Health Protection, Department of Health	Virology Division, Public Health Laboratory Services Branch, Centre for Health Protection, Department of Health	Mak,G.C., Lau,C.S., Chiu,D.M.Y. and Lim,W.
EPI_ISL_2579474	Respiratory Viruses Branch, Centers for Disease Control and Prevention	Respiratory Viruses Branch, Centers for Disease Control and Prevention	Peret,T.C.T.
EPI_ISL_2579475	Measles Section, National Institute for Viral Disease Control and Prevention, China CDC	Measles Section, National Institute for Viral Disease Control and Prevention, China CDC	Zhang,Y., Xu,W., Shen,K., Beeler,J.A., Xie,Z., Sun,L., Lu,Q., Liu,C., Liang,G. and Anderson,L.J.
EPI_ISL_2579476	Pediatrics and Microbiology, University of Alabama at Birmingham	Pediatrics and Microbiology, University of Alabama at Birmingham	Coggins,W.B., Lefkowitz,E.J. and Sullender,W.M.
EPI_ISL_2579478, EPI_ISL_2579479	Division of Immunology, Children's Hospital of Chongqing Medical University	Division of Immunology, Children's Hospital of Chongqing Medical University	Zhang,Z.-Y., Du,L.-N., Chen,X., Zhao,Y., Liu,E.-M., Yang,X.-Q. and Zhao,X.-D.
EPI_ISL_2579481	Virologia, Facultad de Ciencias	Virologia, Facultad de Ciencias	Frabasile,S., Facal,L. and Arbiza,J.
EPI_ISL_2579482	Pediatrics and Microbiology, University of Alabama at Birmingham	Pediatrics and Microbiology, University of Alabama at Birmingham	Coggins,W.B., Lefkowitz,E.J. and Sullender,W.M.
EPI_ISL_2579486	Department of Microbiology, All India Institute of Medical Sciences	Department of Microbiology, All India Institute of Medical Sciences	Parveen,S., Sullender,W.M. and Broor,S.
EPI_ISL_2579488, EPI_ISL_2579489	Pediatrics and Microbiology, University of Alabama at Birmingham	Pediatrics and Microbiology, University of Alabama at Birmingham	Coggins,W.B., Lefkowitz,E.J. and Sullender,W.M.
EPI_ISL_2579491	Measles Section, National Institute for Viral Disease Control and Prevention, China CDC	Measles Section, National Institute for Viral Disease Control and Prevention, China CDC	Zhang,Y., Xu,W., Shen,K., Beeler,J.A., Xie,Z., Sun,L., Lu,Q., Liu,C., Liang,G. and Anderson,L.J.
EPI_ISL_2579492	Virology Division, Public Health Laboratory Services Branch, Centre for Health Protection, Department of Health	Virology Division, Public Health Laboratory Services Branch, Centre for Health Protection, Department of Health	Mak,G.C., Lau,C.S., Chiu,D.M.Y. and Lim,W.
EPI_ISL_2579493	Influenza Group, National Institute of Virology	Influenza Group, National Institute of Virology	Yeolekar,L.R., Peret,T.C., Erdman,D.D. and Rao,B.L.
EPI_ISL_2579498	Measles Section, National Institute for Viral Disease Control and Prevention, China CDC	Measles Section, National Institute for Viral Disease Control and Prevention, China CDC	Zhang,Y., Xu,W., Shen,K., Beeler,J.A., Xie,Z., Sun,L., Lu,Q., Liu,C., Liang,G. and Anderson,L.J.
EPI_ISL_2579535	Respiratory Viruses Branch, Centers for Disease Control and Prevention	Respiratory Viruses Branch, Centers for Disease Control and Prevention	Peret,T.C.T.
EPI_ISL_2579536, EPI_ISL_2579537	Virology Division, Public Health Laboratory Services Branch, Centre for Health Protection, Department of Health	Virology Division, Public Health Laboratory Services Branch, Centre for Health Protection, Department of Health	Mak,G.C., Lau,C.S., Chiu,D.M.Y. and Lim,W.
EPI_ISL_2579538, EPI_ISL_2579539	Microbiology, Adolfo Lutz Institut	Microbiology, Adolfo Lutz Institut	Souza,M.C.O., Matsumoto,T.K., Vaz-de-Lima,L.R.A., Sato,N.N., Salgado,M.M., Requejo,H.I.Z., Hong,M.A., Barbosa,M.L., Oliveira,C.A.F., Benelli,P.C., Shimokawa,R.Y., Pecchini,R., Berezin,E., Duarte,S.P., Schvartsman,C., Durigon,E.L. and Ueda,M.
EPI_ISL_2579540, EPI_ISL_2579541, EPI_ISL_2579542, EPI_ISL_2579543, EPI_ISL_2579544, EPI_ISL_2579545, EPI_ISL_2579546, EPI_ISL_2579547, EPI_ISL_2579548, EPI_ISL_2579549, EPI_ISL_2579550, EPI_ISL_2579551, EPI_ISL_2579552, EPI_ISL_2579553, EPI_ISL_2579554, EPI_ISL_2579555, EPI_ISL_2579556, EPI_ISL_2579557, EPI_ISL_2579558			
see above	Respiratory Viruses Branch, Centers for Disease Control and Prevention	Respiratory Viruses Branch, Centers for Disease Control and Prevention	Peret,T.C.T.
EPI_ISL_2579559, EPI_ISL_2579560, EPI_ISL_2579561	Pediatrics and Microbiology, University of Alabama at Birmingham	Pediatrics and Microbiology, University of Alabama at Birmingham	Coggins,W.B., Lefkowitz,E.J. and Sullender,W.M.
EPI_ISL_2579562, EPI_ISL_2579576	Measles Section, National Institute for Viral Disease Control and Prevention, China CDC	Measles Section, National Institute for Viral Disease Control and Prevention, China CDC	Zhang,Y., Xu,W., Shen,K., Beeler,J.A., Xie,Z., Sun,L., Lu,Q., Liu,C., Liang,G. and Anderson,L.J.

EPI_ISL_2579577	Virology Division, Public Health Laboratory Services Branch, Centre for Health Protection, Department of Health	Virology Division, Public Health Laboratory Services Branch, Centre for Health Protection, Department of Health	Mak,G.C., Lau,C.S., Chiu,D.M.Y. and Lim,W.
EPI_ISL_2579578	Respiratory Viruses Branch, Centers for Disease Control and Prevention	Respiratory Viruses Branch, Centers for Disease Control and Prevention	Peret,T.C.T.
EPI_ISL_2579579, EPI_ISL_2579580, EPI_ISL_2579581, EPI_ISL_2579582	Measles Section, National Institute for Viral Disease Control and Prevention, China CDC	Measles Section, National Institute for Viral Disease Control and Prevention, China CDC	Zhang,Y., Xu,W., Shen,K., Beeler,J.A., Xie,Z., Sun,L., Lu,Q., Liu,C., Liang,G. and Anderson,L.J.
EPI_ISL_2579583	Influenza Group, National Institute of Virology	Influenza Group, National Institute of Virology	Yeolekar,L.R., Peret,T.C., Erdman,D.D. and Rao,B.L.
EPI_ISL_2579584	Pediatrics and Microbiology, University of Alabama at Birmingham	Pediatrics and Microbiology, University of Alabama at Birmingham	Coggins,W.B., Lefkowitz,E.J. and Sullender,W.M.
EPI_ISL_2579587, EPI_ISL_2579588, EPI_ISL_2579589, EPI_ISL_2579590, EPI_ISL_2579591, EPI_ISL_2579592, EPI_ISL_2579593, EPI_ISL_2579594	Influenza Group, National Institute of Virology	Influenza Group, National Institute of Virology	Damle,R.G., Virdi,V.K. and Chadha,M.S.
EPI_ISL_2579595	Measles Section, National Institute for Viral Disease Control and Prevention, China CDC	Measles Section, National Institute for Viral Disease Control and Prevention, China CDC	Zhang,Y., Xu,W., Shen,K., Beeler,J.A., Xie,Z., Sun,L., Lu,Q., Liu,C., Liang,G. and Anderson,L.J.
EPI_ISL_2579596, EPI_ISL_2579597	Pediatrics and Microbiology, University of Alabama at Birmingham	Pediatrics and Microbiology, University of Alabama at Birmingham	Coggins,W.B., Lefkowitz,E.J. and Sullender,W.M.
EPI_ISL_2579598, EPI_ISL_2579600	Influenza Group, National Institute of Virology	Influenza Group, National Institute of Virology	Damle,R.G., Virdi,V.K. and Chadha,M.S.
EPI_ISL_2579601	WHO WPRO Measles Regional Reference Lab, Key Laboratory of Medical Virology Ministry of Health, National Institute for Viral Disease Control and Prevention, China Center for Disease Control and Prevention	WHO WPRO Measles Regional Reference Lab, Key Laboratory of Medical Virology Ministry of Health, National Institute for Viral Disease Control and Prevention, China Center for Disease Control and Prevention	Zhang,Y., Song,J. and Xu,W.
EPI_ISL_2579602	Virology Division, Public Health Laboratory Services Branch, Centre for Health Protection, Department of Health	Virology Division, Public Health Laboratory Services Branch, Centre for Health Protection, Department of Health	Mak,G.C., Lau,C.S., Chiu,D.M.Y. and Lim,W.
EPI_ISL_2579603	Respiratory Viruses Branch, Centers for Disease Control and Prevention	Respiratory Viruses Branch, Centers for Disease Control and Prevention	Peret,T.C.T.
EPI_ISL_2579604, EPI_ISL_2579605	Influenza Group, National Institute of Virology	Influenza Group, National Institute of Virology	Damle,R.G., Virdi,V.K. and Chadha,M.S.
EPI_ISL_2579606	Virologia, Facultad de Ciencias	Virologia, Facultad de Ciencias	Frabasile,S., Facal,L. and Arbiza,J.
EPI_ISL_2579607, EPI_ISL_2579608, EPI_ISL_2579609, EPI_ISL_2579610, EPI_ISL_2579611, EPI_ISL_2579612, EPI_ISL_2579613, EPI_ISL_2579614	Respiratory Viruses Branch, Centers for Disease Control and Prevention	Respiratory Viruses Branch, Centers for Disease Control and Prevention	Peret,T.C.T.
EPI_ISL_2579615, EPI_ISL_2579616, EPI_ISL_2579617, EPI_ISL_2579618, EPI_ISL_2579619	Virologia, Facultad de Ciencias	Virologia, Facultad de Ciencias	Frabasile,S., Facal,L. and Arbiza,J.
EPI_ISL_2579620	Pediatrics and Microbiology, University of Alabama at Birmingham	Pediatrics and Microbiology, University of Alabama at Birmingham	Coggins,W.B., Lefkowitz,E.J. and Sullender,W.M.
EPI_ISL_2579621, EPI_ISL_2579622, EPI_ISL_2579623, EPI_ISL_2579624, EPI_ISL_2579625, EPI_ISL_2579626, EPI_ISL_2579627, EPI_ISL_2579628, EPI_ISL_2579629, EPI_ISL_2579630, EPI_ISL_2579631, EPI_ISL_2579632, EPI_ISL_2579633, EPI_ISL_2579634, EPI_ISL_2579635, EPI_ISL_2579636, EPI_ISL_2579637, EPI_ISL_2579638, EPI_ISL_2579639, EPI_ISL_2579640, EPI_ISL_2579641, EPI_ISL_2579642, EPI_ISL_2579643			
see above	Respiratory Viruses Branch, Centers for Disease Control and Prevention	Respiratory Viruses Branch, Centers for Disease Control and Prevention	Peret,T.C.T.
EPI_ISL_2579644, EPI_ISL_2579645, EPI_ISL_2579646, EPI_ISL_2579647, EPI_ISL_2579648, EPI_ISL_2579649, EPI_ISL_2579650, EPI_ISL_2579651, EPI_ISL_2579652	Dept Microbiology, Instituto de Ciencias Biomedicas, Universidade de Sao Paulo	Dept Microbiology, Instituto de Ciencias Biomedicas, Universidade de Sao Paulo	Moura,F.E.A., Perdigao,A.C.B., Ribeiro,J.F., Florencio,C.M.G.D., Oliveira,F.M.S., Pereira,S.A.R., Botosso,V.F., Siqueira,M.M., Thomazelli,L.M., Caldeira,R.N., Oliveira,D.B.L. and Durigon,E.L.
EPI_ISL_2579653, EPI_ISL_2579654, EPI_ISL_2579655, EPI_ISL_2579656, EPI_ISL_2579657, EPI_ISL_2579658, EPI_ISL_2579659, EPI_ISL_2579660	Microbiology, Adolfo Lutz Institut	Microbiology, Adolfo Lutz Institut	Souza,M.C.O., Matsumoto,T.K., Vaz-de-Lima,L.R.A., Sato,N.N., Salgado,M.M., Requejo,H.I.Z., Hong,M.A., Barbosa,M.L., Oliveira,C.A.F., Benelli,P.C., Shimokawa,R.Y., Pecchini,R., Berezin,E., Duarte,S.P., Schvartsman,C., Durigon,E.L. and Ueda,M.
EPI_ISL_2579661, EPI_ISL_2579662	Virology Division, Public Health Laboratory Services Branch, Centre for Health Protection, Department of Health	Virology Division, Public Health Laboratory Services Branch, Centre for Health Protection, Department of Health	Mak,G.C., Lau,C.S., Chiu,D.M.Y. and Lim,W.
EPI_ISL_2579663	Influenza Group, National Institute of Virology	Influenza Group, National Institute of Virology	Damle,R.G., Virdi,V.K. and Chadha,M.S.
EPI_ISL_2579664	Influenza Group, National Institute of Virology	Influenza Group, National Institute of Virology	Yeolekar,L.R., Peret,T.C., Erdman,D.D. and Rao,B.L.
EPI_ISL_2579665, EPI_ISL_2579666, EPI_ISL_2579667, EPI_ISL_2579668, EPI_ISL_2579669, EPI_ISL_2579670, EPI_ISL_2579671	Respiratory Viruses Branch, Centers for Disease Control and Prevention	Respiratory Viruses Branch, Centers for Disease Control and Prevention	Peret,T.C.T.
EPI_ISL_2579672	Microbiology, Adolfo Lutz Institut	Microbiology, Adolfo Lutz Institut	Souza,M.C.O., Matsumoto,T.K., Vaz-de-Lima,L.R.A., Sato,N.N., Salgado,M.M., Requejo,H.I.Z., Hong,M.A., Barbosa,M.L., Oliveira,C.A.F., Benelli,P.C., Shimokawa,R.Y., Pecchini,R., Berezin,E., Duarte,S.P., Schvartsman,C., Durigon,E.L. and Ueda,M.
EPI_ISL_2579673	Influenza Group, National Institute of Virology	Influenza Group, National Institute of Virology	Damle,R.G., Virdi,V.K. and Chadha,M.S.
EPI_ISL_2579674, EPI_ISL_2579675, EPI_ISL_2579676, EPI_ISL_2579677, EPI_ISL_2579678, EPI_ISL_2579679, EPI_ISL_2579680	Pediatrics and Microbiology, University of Alabama at Birmingham	Pediatrics and Microbiology, University of Alabama at Birmingham	Coggins,W.B., Lefkowitz,E.J. and Sullender,W.M.
EPI_ISL_2579685, EPI_ISL_2579686, EPI_ISL_2579687	Measles Section, National Institute for Viral Disease Control and Prevention, China CDC	Measles Section, National Institute for Viral Disease Control and Prevention, China CDC	Zhang,Y., Xu,W., Shen,K., Beeler,J.A., Xie,Z., Sun,L., Lu,Q., Liu,C., Liang,G. and Anderson,L.J.
EPI_ISL_2579690, EPI_ISL_2579691, EPI_ISL_2579692	Division of Immunology, Children's Hospital of Chongqing Medical University	Division of Immunology, Children's Hospital of Chongqing Medical University	Zhang,Z.-Y., Du,L.-N., Chen,X., Zhao,Y., Liu,E.-M., Yang,X.-Q. and Zhao,X.-D.
EPI_ISL_2579694	Influenza Group, National Institute of Virology	Influenza Group, National Institute of Virology	Yeolekar,L.R., Peret,T.C., Erdman,D.D. and Rao,B.L.
EPI_ISL_2579695	Chinese Academy of Medical Sciences & Peking Union Medical College	Chinese Academy of Medical Sciences & Peking Union Medical College	Jia,B., Xiao,Y., Wang,Y., Chen,L., Zhang,J., Ren,L. and Wang,J.
EPI_ISL_2579697	Virology Division, Public Health Laboratory Services Branch, Centre for Health Protection, Department of Health	Virology Division, Public Health Laboratory Services Branch, Centre for Health Protection, Department of Health	Mak,G.C., Lau,C.S., Chiu,D.M.Y. and Lim,W.
EPI_ISL_2579698, EPI_ISL_2579699	Respiratory Viruses Branch, Centers for Disease Control and Prevention	Respiratory Viruses Branch, Centers for Disease Control and Prevention	Peret,T.C.T.
EPI_ISL_2579700, EPI_ISL_2579701, EPI_ISL_2579702, EPI_ISL_2579703, EPI_ISL_2579704, EPI_ISL_2579705, EPI_ISL_2579706, EPI_ISL_2579707, EPI_ISL_2579708, EPI_ISL_2579709, EPI_ISL_2579710, EPI_ISL_2579711, EPI_ISL_2579712, EPI_ISL_2579713, EPI_ISL_2579714			
see above	Department of Microbiology, All India Institute of Medical Sciences	Department of Microbiology, All India Institute of Medical Sciences	Parveen,S., Sullender,W.M. and Broor,S.
EPI_ISL_2579715	Respiratory Viruses Branch, Centers for Disease Control and Prevention	Respiratory Viruses Branch, Centers for Disease Control and Prevention	Peret,T.C.T.
EPI_ISL_2579716, EPI_ISL_2579717	Microbiology, Adolfo Lutz Institut	Microbiology, Adolfo Lutz Institut	Souza,M.C.O., Matsumoto,T.K., Vaz-de-Lima,L.R.A., Sato,N.N., Salgado,M.M., Requejo,H.I.Z., Hong,M.A., Barbosa,M.L., Oliveira,C.A.F., Benelli,P.C., Shimokawa,R.Y., Pecchini,R., Berezin,E., Duarte,S.P., Schvartsman,C., Durigon,E.L. and Ueda,M.
EPI_ISL_2579718, EPI_ISL_2579719, EPI_ISL_2579720, EPI_ISL_2579721, EPI_ISL_2579722, EPI_ISL_2579723, EPI_ISL_2579724, EPI_ISL_2579725, EPI_ISL_2579726, EPI_ISL_2579727, EPI_ISL_2579728, EPI_ISL_2579729, EPI_ISL_2579730, EPI_ISL_2579731			
see above	Respiratory Viruses Branch, Centers for Disease Control and Prevention	Respiratory Viruses Branch, Centers for Disease Control and Prevention	Peret,T.C.T.
EPI_ISL_2579732, EPI_ISL_2579733, EPI_ISL_2579734	Dept Microbiology, Instituto de Ciencias Biomedicas, Universidade de Sao Paulo	Dept Microbiology, Instituto de Ciencias Biomedicas, Universidade de Sao Paulo	Moura,F.E.A., Perdigao,A.C.B., Ribeiro,J.F., Florencio,C.M.G.D., Oliveira,F.M.S., Pereira,S.A.R., Botosso,V.F., Siqueira,M.M., Thomazelli,L.M., Caldeira,R.N., Oliveira,D.B.L. and Durigon,E.L.
EPI_ISL_2579735, EPI_ISL_2579736	Virology Division, Public Health Laboratory Services Branch, Centre for Health Protection, Department of Health	Virology Division, Public Health Laboratory Services Branch, Centre for Health Protection, Department of Health	Mak,G.C., Lau,C.S., Chiu,D.M.Y. and Lim,W.
EPI_ISL_2579737, EPI_ISL_2579738, EPI_ISL_2579739	Influenza Group, National Institute of Virology	Influenza Group, National Institute of Virology	Damle,R.G., Virdi,V.K. and Chadha,M.S.
EPI_ISL_2579740, EPI_ISL_2579741, EPI_ISL_2579742, EPI_ISL_2579743, EPI_ISL_2579744, EPI_ISL_2579745, EPI_ISL_2579746, EPI_ISL_2579747, EPI_ISL_2579748, EPI_ISL_2579749, EPI_ISL_2579750, EPI_ISL_2579751			
see above	Respiratory Viruses Branch, Centers for Disease Control and Prevention	Respiratory Viruses Branch, Centers for Disease Control and Prevention	Peret,T.C.T.
EPI_ISL_2579755, EPI_ISL_2579756, EPI_ISL_2579757	Chinese Academy of Medical Sciences & Peking Union Medical College	Chinese Academy of Medical Sciences & Peking Union Medical College	Jia,B., Xiao,Y., Wang,Y., Chen,L., Zhang,J., Ren,L. and Wang,J.
EPI_ISL_2579758	Measles Section, National Institute for Viral Disease Control and Prevention, China CDC	Measles Section, National Institute for Viral Disease Control and Prevention, China CDC	Zhang,Y., Xu,W., Shen,K., Beeler,J.A., Xie,Z., Sun,L., Lu,Q., Liu,C., Liang,G. and Anderson,L.J.
EPI_ISL_2579759	Virologia, Facultad de Ciencias	Virologia, Facultad de Ciencias	Frabasile,S., Facal,L. and Arbiza,J.
EPI_ISL_2579762, EPI_ISL_2579763, EPI_ISL_2579764, EPI_ISL_2579765, EPI_ISL_2579766, EPI_ISL_2579767, EPI_ISL_2579768, EPI_ISL_2579769, EPI_ISL_2579771, EPI_ISL_2579772, EPI_ISL_2579773			
see above	Dina J., University Hospital of Caen, Virology	Dina J., University Hospital of Caen, Virology	Dina,J.
EPI_ISL_2579774	Depto Microbiologia, Instituto de Ciencias Biomedicas, Universidade de Sao Paulo	Depto Microbiologia, Instituto de Ciencias Biomedicas, Universidade de Sao Paulo	Moura,F.E.A., Thomazelli,L.M., Candido,E.D.O., Florencio,C.M.G.D., Pereira,S.A.R., Oliveira,F.M.S., Alves,A.A., Ocadaque,C.J. and Durigon,E.L.
EPI_ISL_2579776	Center for Infectious Diseases, School of Public Health, University of Texas Health Science Center	Center for Infectious Diseases, School of Public Health, University of Texas Health Science Center	Bahl,J., Hixson,J., Kim,D.-K., Qiu,X., Piedra,P.A., Piedra,F.-A., Avadhanula,V. and Machado,A.A.
EPI_ISL_2579821	Centre for Research And Knowledge Transfer in Biotechnology, University of Zagreb	Centre for Research And Knowledge Transfer in Biotechnology, University of Zagreb	Jagusic,M., Slovic,A., Santak,M., Kosutic-Gulija,T., Ivancic-Jelecki,J. and Forcic,D.
EPI_ISL_2579861, EPI_ISL_2579862, EPI_ISL_2579863, EPI_ISL_2579864, EPI_ISL_2579865, EPI_ISL_2579866, EPI_ISL_2579867, EPI_ISL_2579868, EPI_ISL_2579869, EPI_ISL_2579870, EPI_ISL_2579871, EPI_ISL_2579872, EPI_ISL_2579873, EPI_ISL_2579874, EPI_ISL_2579875, EPI_ISL_2579876, EPI_ISL_2579877, EPI_ISL_2579878, EPI_ISL_2579879			
see above	Microbiology, Adolfo Lutz Institut	Microbiology, Adolfo Lutz Institut	Souza,M.C.O., Matsumoto,T.K., Vaz-de-Lima,L.R.A., Sato,N.N., Salgado,M.M., Requejo,H.I.Z., Hong,M.A., Barbosa,M.L., Oliveira,C.A.F., Benelli,P.C., Shimokawa,R.Y., Pecchini,R., Berezin,E., Duarte,S.P., Schvartsman,C., Durigon,E.L. and Ueda,M.
EPI_ISL_2579880, EPI_ISL_2579881	Depto Microbiologia, Instituto de Ciencias Biomedicas, Universidade de Sao Paulo	Depto Microbiologia, Instituto de Ciencias Biomedicas, Universidade de Sao Paulo	Moura,F.E.A., Thomazelli,L.M., Candido,E.D.O., Florencio,C.M.G.D., Pereira,S.A.R., Oliveira,F.M.S., Alves,A.A., Ocadaque,C.J. and Durigon,E.L.
EPI_ISL_2579882, EPI_ISL_2579883, EPI_ISL_2579884, EPI_ISL_2579885	Microbiology, Adolfo Lutz Institut	Microbiology, Adolfo Lutz Institut	Souza,M.C.O., Matsumoto,T.K., Vaz-de-Lima,L.R.A., Sato,N.N., Salgado,M.M., Requejo,H.I.Z., Hong,M.A., Barbosa,M.L., Oliveira,C.A.F., Benelli,P.C., Shimokawa,R.Y., Pecchini,R., Berezin,E., Duarte,S.P., Schvartsman,C., Durigon,E.L. and Ueda,M.
EPI_ISL_2579886, EPI_ISL_2579887, EPI_ISL_2579888, EPI_ISL_2579889, EPI_ISL_2579890, EPI_ISL_2579891, EPI_ISL_2579892, EPI_ISL_2579893, EPI_ISL_2579894, EPI_ISL_2579895, EPI_ISL_2579896, EPI_ISL_2579897			
see above	Broad Institute of MIT & Harvard	Broad Institute of MIT & Harvard	Newman,R.M., Zody,M.C., DeVincenzo,J.P., Grad,Y., Lipsitch,M., Murphy,R., Fitzgerald,M., Young,S., Gargeya,S., Poon,T.W., Charlebois,P., Weiner,B., Yang,X., Piper,M.E., McCowan,C., Ireland,A., Levin,J., Malboeuf,C., Qu,J., Chapman,S.B., Murphy,C., Wortman,J., Nusbaum,C. and Birren,B.
EPI_ISL_2579901	Department of Epidemiology and Demography, Kenya Medical Research Institute (KEMRI) - Wellcome Trust Research Programme	Department of Epidemiology and Demography, Kenya Medical Research Institute (KEMRI) - Wellcome Trust Research Programme	Otieno,J.R., Agoti,C.N., Gitahi,C.W., Bett,A., Ngama,M., Medley,G.F., Cane,P.A. and Nokes,J.D.

EPI_ISL_2579903	Microbiology, Adolfo Lutz Institut	Microbiology, Adolfo Lutz Institut	Souza,M.C.O., Matsumoto,T.K., Vaz-de-Lima,L.R.A., Sato,N.N., Salgado,M.M., Requejo,H.I.Z., Hong,M.A., Barbosa,M.L., Oliveira,C.A.F., Benelli,P.C., Shimokawa,R.Y., Pecchini,R., Berezin,E., Duarte,S.P., Schvartsman,C., Durigon,E.L. and Ueda,M.
EPI_ISL_2579905	Depto Microbiologia, Instituto de Ciencias Biomedicas, Universidade de Sao Paulo	Depto Microbiologia, Instituto de Ciencias Biomedicas, Universidade de Sao Paulo	Moura,F.E.A., Thomazelli,L.M., Candido,E.D.O., Florencio,C.M.G.D., Pereira,S.A.R., Oliveira,F.M.S., Alves,A.A., Ocadaque,C.J. and Durigon,E.L.
EPI_ISL_2579942, EPI_ISL_2579944, EPI_ISL_2579945, EPI_ISL_2579947, EPI_ISL_2579949, EPI_ISL_2579951, EPI_ISL_2579952, EPI_ISL_2579954, EPI_ISL_2579956, EPI_ISL_2579958, EPI_ISL_2579959, EPI_ISL_2579961, EPI_ISL_2579963, EPI_ISL_2579964, EPI_ISL_2579966, EPI_ISL_2579968			
see above	Respiratory Viruses Branch, Centers for Disease Control and Prevention	Respiratory Viruses Branch, Centers for Disease Control and Prevention	Peret,T.C.T.
EPI_ISL_2580032	Measles Section, National Institute for Viral Disease Control and Prevention, China CDC	Measles Section, National Institute for Viral Disease Control and Prevention, China CDC	Zhang,Y., Xu,W., Shen,K., Beeler,J.A., Xie,Z., Sun,L., Lu,Q., Liu,C., Liang,G. and Anderson,L.J.
EPI_ISL_2580073	Respiratory Viruses Branch, Centers for Disease Control and Prevention	Respiratory Viruses Branch, Centers for Disease Control and Prevention	Peret,T.C.T.
EPI_ISL_2580076, EPI_ISL_2580077, EPI_ISL_2580078, EPI_ISL_2580079, EPI_ISL_2580080, EPI_ISL_2580082, EPI_ISL_2580083, EPI_ISL_2580085, EPI_ISL_2580087	Dept Microbiology, Instituto de Ciencias Biomedicas, Universidade de Sao Paulo	Dept Microbiology, Instituto de Ciencias Biomedicas, Universidade de Sao Paulo	Moura,F.E.A., Perdigao,A.C.B., Ribeiro,J.F., Florencio,C.M.G.D., Oliveira,F.M.S., Pereira,S.A.R., Botosso,V.F., Siqueira,M.M., Thomazelli,L.M., Caldeira,R.N., Oliveira,D.B.L. and Durigon,E.L.
EPI_ISL_2580090, EPI_ISL_2580092, EPI_ISL_2580094	Microbiology, Adolfo Lutz Institut	Microbiology, Adolfo Lutz Institut	Souza,M.C.O., Matsumoto,T.K., Vaz-de-Lima,L.R.A., Sato,N.N., Salgado,M.M., Requejo,H.I.Z., Hong,M.A., Barbosa,M.L., Oliveira,C.A.F., Benelli,P.C., Shimokawa,R.Y., Pecchini,R., Berezin,E., Duarte,S.P., Schvartsman,C., Durigon,E.L. and Ueda,M.
EPI_ISL_2580095, EPI_ISL_2580097, EPI_ISL_2580104, EPI_ISL_2580106, EPI_ISL_2580109, EPI_ISL_2580110, EPI_ISL_2580112, EPI_ISL_2580114, EPI_ISL_2580126, EPI_ISL_2580160, EPI_ISL_2580162, EPI_ISL_2580163, EPI_ISL_2580166, EPI_ISL_2580168, EPI_ISL_2580171, EPI_ISL_2580173, EPI_ISL_2580175, EPI_ISL_2580177, EPI_ISL_2580180, EPI_ISL_2580182, EPI_ISL_2580184, EPI_ISL_2580186, EPI_ISL_2580187, EPI_ISL_2580189, EPI_ISL_2580191, EPI_ISL_2580193, EPI_ISL_2580194, EPI_ISL_2580196, EPI_ISL_2580198, EPI_ISL_2580200, EPI_ISL_2580201, EPI_ISL_2580203, EPI_ISL_2580205, EPI_ISL_2580207, EPI_ISL_2580208, EPI_ISL_2580210			
see above	Chinese Academy of Medical Sciences & Peking Union Medical College	Chinese Academy of Medical Sciences & Peking Union Medical College	Jia,B., Xiao,Y., Wang,Y., Chen,L., Zhang,J., Ren,L. and Wang,J.
EPI_ISL_2580212, EPI_ISL_2580213, EPI_ISL_2580215, EPI_ISL_2580235	Measles Section, National Institute for Viral Disease Control and Prevention, China CDC	Measles Section, National Institute for Viral Disease Control and Prevention, China CDC	Zhang,Y., Xu,W., Shen,K., Beeler,J.A., Xie,Z., Sun,L., Lu,Q., Liu,C., Liang,G. and Anderson,L.J.
EPI_ISL_2580237, EPI_ISL_2580239, EPI_ISL_2580240, EPI_ISL_2580242, EPI_ISL_2580244, EPI_ISL_2580246, EPI_ISL_2580247	Division of Immunology, Children's Hospital of Chongqing Medical University	Division of Immunology, Children's Hospital of Chongqing Medical University	Zhang,Z.-Y., Du,L.-N., Chen,X., Zhao,Y., Liu,E.-M., Yang,X.-Q. and Zhao,X.-D.
EPI_ISL_2580281	Virology, Noguchi Memorial Institute for Medical Research	Virology, Noguchi Memorial Institute for Medical Research	Obodai,E., Odoom,J.K., Adiku,T., Goka,B., Biere,B., Wolff,T., Schweiger,B. and Reiche,J.
EPI_ISL_2580283, EPI_ISL_2580285, EPI_ISL_2580286, EPI_ISL_2580288	Virology Division, Public Health Laboratory Services Branch, Centre for Health Protection, Department of Health	Virology Division, Public Health Laboratory Services Branch, Centre for Health Protection, Department of Health	Mak,G.C., Lau,C.S., Chiu,D.M.Y. and Lim,W.
EPI_ISL_2580297	Virology, School of Public Health and Institute of Public Health Research, Tehran University of Medical Sciences	Virology, School of Public Health and Institute of Public Health Research, Tehran University of Medical Sciences	Mokhtari-Azad,T., Faghihloo,E., Salimi,V. and Rezaei,F.
EPI_ISL_2580343, EPI_ISL_2580345, EPI_ISL_2580347, EPI_ISL_2580349	Respiratory Viruses Branch, Centers for Disease Control and Prevention	Respiratory Viruses Branch, Centers for Disease Control and Prevention	Peret,T.C.T.
EPI_ISL_2580355, EPI_ISL_2580357, EPI_ISL_2580358	Measles Section, National Institute for Viral Disease Control and Prevention, China CDC	Measles Section, National Institute for Viral Disease Control and Prevention, China CDC	Zhang,Y., Xu,W., Shen,K., Beeler,J.A., Xie,Z., Sun,L., Lu,Q., Liu,C., Liang,G. and Anderson,L.J.
EPI_ISL_2580362	Israel National Influenza Center, Central Virology Laboratory	Israel National Influenza Center, Central Virology Laboratory	Hirsh,S., MNorth America / USA,H. and Michal,M.
EPI_ISL_2580692, EPI_ISL_2580693, EPI_ISL_2580695	Dept Microbiology, Instituto de Ciencias Biomedicas, Universidade de Sao Paulo	Dept Microbiology, Instituto de Ciencias Biomedicas, Universidade de Sao Paulo	Moura,F.E.A., Perdigao,A.C.B., Ribeiro,J.F., Florencio,C.M.G.D., Oliveira,F.M.S., Pereira,S.A.R., Botosso,V.F., Siqueira,M.M., Thomazelli,L.M., Caldeira,R.N., Oliveira,D.B.L. and Durigon,E.L.
EPI_ISL_2580698, EPI_ISL_2580699, EPI_ISL_2580706, EPI_ISL_2580735, EPI_ISL_2580783, EPI_ISL_2580807, EPI_ISL_2580810, EPI_ISL_2580811, EPI_ISL_2580818, EPI_ISL_2580819, EPI_ISL_2580821, EPI_ISL_2580824, EPI_ISL_2580825, EPI_ISL_2580827, EPI_ISL_2580828, EPI_ISL_2580829			
see above	Chinese Academy of Medical Sciences & Peking Union Medical College	Chinese Academy of Medical Sciences & Peking Union Medical College	Jia,B., Xiao,Y., Wang,Y., Chen,L., Zhang,J., Ren,L. and Wang,J.
EPI_ISL_2580833	Measles Section, National Institute for Viral Disease Control and Prevention, China CDC	Measles Section, National Institute for Viral Disease Control and Prevention, China CDC	Zhang,Y., Xu,W., Shen,K., Beeler,J.A., Xie,Z., Sun,L., Lu,Q., Liu,C., Liang,G. and Anderson,L.J.
EPI_ISL_2580867	Division of Immunology, Children's Hospital of Chongqing Medical University	Division of Immunology, Children's Hospital of Chongqing Medical University	Zhang,Z.-Y., Du,L.-N., Chen,X., Zhao,Y., Liu,E.-M., Yang,X.-Q. and Zhao,X.-D.
EPI_ISL_2580909, EPI_ISL_2580912, EPI_ISL_2580913, EPI_ISL_2580916, EPI_ISL_2580917, EPI_ISL_2580919, EPI_ISL_2580921	Influenza Group, National Institute of Virology	Influenza Group, National Institute of Virology	Damle,R.G., Virdi,V.K. and Chadha,M.S.
EPI_ISL_2580925, EPI_ISL_2580927	Virology, School of Public Health and Institute of Public Health Research, Tehran University of Medical Sciences	Virology, School of Public Health and Institute of Public Health Research, Tehran University of Medical Sciences	Mokhtari-Azad,T., Faghihloo,E., Salimi,V. and Rezaei,F.
EPI_ISL_2580951	Respiratory Viruses Branch, Centers for Disease Control and Prevention	Respiratory Viruses Branch, Centers for Disease Control and Prevention	Peret,T.C.T.
EPI_ISL_2581017, EPI_ISL_2581018, EPI_ISL_2581019, EPI_ISL_2581020, EPI_ISL_2581021	Measles Section, National Institute for Viral Disease Control and Prevention, China CDC	Measles Section, National Institute for Viral Disease Control and Prevention, China CDC	Zhang,Y., Xu,W., Shen,K., Beeler,J.A., Xie,Z., Sun,L., Lu,Q., Liu,C., Liang,G. and Anderson,L.J.
EPI_ISL_2581022, EPI_ISL_2581023	Influenza Group, National Institute of Virology	Influenza Group, National Institute of Virology	Yeolekar,L.R., Peret,T.C., Erdman,D.D. and Rao,B.L.
EPI_ISL_2581028	Israel National Influenza Center, Central Virology Laboratory	Israel National Influenza Center, Central Virology Laboratory	Hirsh,S., MNorth America / USA,H. and Michal,M.
EPI_ISL_2581195, EPI_ISL_2581197, EPI_ISL_2581200	Dept Microbiology, Instituto de Ciencias Biomedicas, Universidade de Sao Paulo	Dept Microbiology, Instituto de Ciencias Biomedicas, Universidade de Sao Paulo	Moura,F.E.A., Perdigao,A.C.B., Ribeiro,J.F., Florencio,C.M.G.D., Oliveira,F.M.S., Pereira,S.A.R., Botosso,V.F., Siqueira,M.M., Thomazelli,L.M., Caldeira,R.N., Oliveira,D.B.L. and Durigon,E.L.
EPI_ISL_2581213, EPI_ISL_2581218, EPI_ISL_2581219, EPI_ISL_2581221, EPI_ISL_2581224, EPI_ISL_2581225, EPI_ISL_2581226	Chinese Academy of Medical Sciences & Peking Union Medical College	Chinese Academy of Medical Sciences & Peking Union Medical College	Jia,B., Xiao,Y., Wang,Y., Chen,L., Zhang,J., Ren,L. and Wang,J.
EPI_ISL_2581228, EPI_ISL_2581229, EPI_ISL_2581231, EPI_ISL_2581234, EPI_ISL_2581235	Measles Section, National Institute for Viral Disease Control and Prevention, China CDC	Measles Section, National Institute for Viral Disease Control and Prevention, China CDC	Zhang,Y., Xu,W., Shen,K., Beeler,J.A., Xie,Z., Sun,L., Lu,Q., Liu,C., Liang,G. and Anderson,L.J.
EPI_ISL_2581239	Division of Immunology, Children's Hospital of Chongqing Medical University	Division of Immunology, Children's Hospital of Chongqing Medical University	Zhang,Z.-Y., Du,L.-N., Chen,X., Zhao,Y., Liu,E.-M., Yang,X.-Q. and Zhao,X.-D.
EPI_ISL_2581265	Influenza Group, National Institute of Virology	Influenza Group, National Institute of Virology	Damle,R.G., Virdi,V.K. and Chadha,M.S.
EPI_ISL_2581273, EPI_ISL_2581275, EPI_ISL_2581277, EPI_ISL_2581279, EPI_ISL_2581281, EPI_ISL_2581284, EPI_ISL_2581285, EPI_ISL_2581287, EPI_ISL_2581289	Respiratory Viruses Branch, Centers for Disease Control and Prevention	Respiratory Viruses Branch, Centers for Disease Control and Prevention	Peret,T.C.T.
EPI_ISL_2581317, EPI_ISL_2581318, EPI_ISL_2581320, EPI_ISL_2581321, EPI_ISL_2581325	Measles Section, National Institute for Viral Disease Control and Prevention, China CDC	Measles Section, National Institute for Viral Disease Control and Prevention, China CDC	Zhang,Y., Xu,W., Shen,K., Beeler,J.A., Xie,Z., Sun,L., Lu,Q., Liu,C., Liang,G. and Anderson,L.J.
EPI_ISL_2581377, EPI_ISL_2581379, EPI_ISL_2581380	Dept Microbiology, Instituto de Ciencias Biomedicas, Universidade de Sao Paulo	Dept Microbiology, Instituto de Ciencias Biomedicas, Universidade de Sao Paulo	Moura,F.E.A., Perdigao,A.C.B., Ribeiro,J.F., Florencio,C.M.G.D., Oliveira,F.M.S., Pereira,S.A.R., Botosso,V.F., Siqueira,M.M., Thomazelli,L.M., Caldeira,R.N., Oliveira,D.B.L. and Durigon,E.L.
EPI_ISL_2581381	Microbiology, Adolfo Lutz Institut	Microbiology, Adolfo Lutz Institut	Souza,M.C.O., Matsumoto,T.K., Vaz-de-Lima,L.R.A., Sato,N.N., Salgado,M.M., Requejo,H.I.Z., Hong,M.A., Barbosa,M.L., Oliveira,C.A.F., Benelli,P.C., Shimokawa,R.Y., Pecchini,R., Berezin,E., Duarte,S.P., Schvartsman,C., Durigon,E.L. and Ueda,M.
EPI_ISL_2581382	Chinese Academy of Medical Sciences & Peking Union Medical College	Chinese Academy of Medical Sciences & Peking Union Medical College	Jia,B., Xiao,Y., Wang,Y., Chen,L., Zhang,J., Ren,L. and Wang,J.
EPI_ISL_2581384, EPI_ISL_2581385, EPI_ISL_2581386, EPI_ISL_2581388	Measles Section, National Institute for Viral Disease Control and Prevention, China CDC	Measles Section, National Institute for Viral Disease Control and Prevention, China CDC	Zhang,Y., Xu,W., Shen,K., Beeler,J.A., Xie,Z., Sun,L., Lu,Q., Liu,C., Liang,G. and Anderson,L.J.
EPI_ISL_2581403, EPI_ISL_2581405, EPI_ISL_2581407, EPI_ISL_2581409	Influenza Group, National Institute of Virology	Influenza Group, National Institute of Virology	Damle,R.G., Virdi,V.K. and Chadha,M.S.
EPI_ISL_2581419, EPI_ISL_2581422, EPI_ISL_2581423, EPI_ISL_2581425, EPI_ISL_2581428	Tsuguto Fujimoto National Institute of Infectious Diseases, Infectious Disease Surveillance Center	Tsuguto Fujimoto National Institute of Infectious Diseases, Infectious Disease Surveillance Center	Fujimoto,T., Hanaoka,N., Nakamura,M. and Kawabata,M.
EPI_ISL_2581429	Respiratory Viruses Branch, Centers for Disease Control and Prevention	Respiratory Viruses Branch, Centers for Disease Control and Prevention	Peret,T.C.T.
EPI_ISL_2581441	Dept Microbiology, Instituto de Ciencias Biomedicas, Universidade de Sao Paulo	Dept Microbiology, Instituto de Ciencias Biomedicas, Universidade de Sao Paulo	Moura,F.E.A., Perdigao,A.C.B., Ribeiro,J.F., Florencio,C.M.G.D., Oliveira,F.M.S., Pereira,S.A.R., Botosso,V.F., Siqueira,M.M., Thomazelli,L.M., Caldeira,R.N., Oliveira,D.B.L. and Durigon,E.L.
EPI_ISL_2581462	Measles Section, National Institute for Viral Disease Control and Prevention, China CDC	Measles Section, National Institute for Viral Disease Control and Prevention, China CDC	Zhang,Y., Xu,W., Shen,K., Beeler,J.A., Xie,Z., Sun,L., Lu,Q., Liu,C., Liang,G. and Anderson,L.J.
EPI_ISL_2581470	Department of Microbiology, All India Institute of Medical Sciences	Department of Microbiology, All India Institute of Medical Sciences	Parveen,S., Sullender,W.M. and Broor,S.
EPI_ISL_2581495	Virology Division, Public Health Laboratory Services Branch, Centre for Health Protection, Department of Health	Virology Division, Public Health Laboratory Services Branch, Centre for Health Protection, Department of Health	Mak,G.C., Lau,C.S., Chiu,D.M.Y. and Lim,W.
EPI_ISL_2581515, EPI_ISL_2581518, EPI_ISL_2581519	Department of Microbiology, All India Institute of Medical Sciences	Department of Microbiology, All India Institute of Medical Sciences	Parveen,S., Sullender,W.M. and Broor,S.
EPI_ISL_2581529	Measles Section, National Institute for Viral Disease Control and Prevention, China CDC	Measles Section, National Institute for Viral Disease Control and Prevention, China CDC	Zhang,Y., Xu,W., Shen,K., Beeler,J.A., Xie,Z., Sun,L., Lu,Q., Liu,C., Liang,G. and Anderson,L.J.
EPI_ISL_2581537, EPI_ISL_2581540, EPI_ISL_2581541, EPI_ISL_2581550	Department of Microbiology, All India Institute of Medical Sciences	Department of Microbiology, All India Institute of Medical Sciences	Parveen,S., Sullender,W.M. and Broor,S.
EPI_ISL_2581579	Pediatrics and Microbiology, University of Alabama at Birmingham	Pediatrics and Microbiology, University of Alabama at Birmingham	Coggins,W.B., Lefkowitz,E.J. and Sullender,W.M.
EPI_ISL_2581584	Depto Microbiologia, Instituto de Ciencias Biomedicas, Universidade de Sao Paulo	Depto Microbiologia, Instituto de Ciencias Biomedicas, Universidade de Sao Paulo	Moura,F.E.A., Thomazelli,L.M., Candido,E.D.O., Florencio,C.M.G.D., Pereira,S.A.R., Oliveira,F.M.S., Alves,A.A., Ocadaque,C.J. and Durigon,E.L.
EPI_ISL_2581599, EPI_ISL_2581602, EPI_ISL_2581615	Chinese Academy of Medical Sciences & Peking Union Medical College	Chinese Academy of Medical Sciences & Peking Union Medical College	Jia,B., Xiao,Y., Wang,Y., Chen,L., Zhang,J., Ren,L. and Wang,J.
EPI_ISL_2581639	Laboratory of Virology, Capital Institute of Pediatrics	Laboratory of Virology, Capital Institute of Pediatrics	Cui,G., Deng,J., Zhu,R., Qian,Y., Sun,Y., Zhao,L. and Wang,F.
EPI_ISL_2581647, EPI_ISL_2581691	Chinese Academy of Medical Sciences & Peking Union Medical College	Chinese Academy of Medical Sciences & Peking Union Medical College	Jia,B., Xiao,Y., Wang,Y., Chen,L., Zhang,J., Ren,L. and Wang,J.
EPI_ISL_2581781	Laboratory of Virology, Capital Institute of Pediatrics	Laboratory of Virology, Capital Institute of Pediatrics	Cui,G., Deng,J., Zhu,R., Qian,Y., Sun,Y., Zhao,L. and Wang,F.
EPI_ISL_2581797, EPI_ISL_2581810, EPI_ISL_2581811, EPI_ISL_2581813, EPI_ISL_2581824, EPI_ISL_2581825, EPI_ISL_2581828, EPI_ISL_2581829, EPI_ISL_2581832, EPI_ISL_2581833, EPI_ISL_2581837, EPI_ISL_2581840, EPI_ISL_2581841, EPI_ISL_2581844, EPI_ISL_2581845, EPI_ISL_2581846, EPI_ISL_2581848, EPI_ISL_2581849			
see above	Chinese Academy of Medical Sciences & Peking Union Medical College	Chinese Academy of Medical Sciences & Peking Union Medical College	Jia,B., Xiao,Y., Wang,Y., Chen,L., Zhang,J., Ren,L. and Wang,J.
EPI_ISL_2581902, EPI_ISL_2581903, EPI_ISL_2581906, EPI_ISL_2581907, EPI_ISL_2581909, EPI_ISL_2581912	Laboratory of Virology, Capital Institute of Pediatrics	Laboratory of Virology, Capital Institute of Pediatrics	Cui,G., Deng,J., Zhu,R., Qian,Y., Sun,Y., Zhao,L. and Wang,F.
EPI_ISL_2581923, EPI_ISL_2581927, EPI_ISL_2581930, EPI_ISL_2581938, EPI_ISL_2581959, EPI_ISL_2581965, EPI_ISL_2581972, EPI_ISL_2581973, EPI_ISL_2581974, EPI_ISL_2581980, EPI_ISL_2581981, EPI_ISL_2581983, EPI_ISL_2581989, EPI_ISL_2581991, EPI_ISL_2581994, EPI_ISL_2581999, EPI_ISL_2582001, EPI_ISL_2582003, EPI_ISL_2582006, EPI_ISL_2582007, EPI_ISL_2582015, EPI_ISL_2582035,			

EPI_ISL_2582043			
see above	Depto Microbiologia, Instituto de Ciencias Biomedicas, Universidade de Sao Paulo	Depto Microbiologia, Instituto de Ciencias Biomedicas, Universidade de Sao Paulo	Moura,F.E.A., Thomazelli,L.M., Candido,E.D.O., Florencio,C.M.G.D., Pereira,S.A.R., Oliveira,F.M.S., Alves,A.A., Ocadaque,C.J. and Durigon,E.L.
EPI_ISL_2582045, EPI_ISL_2582047, EPI_ISL_2582050, EPI_ISL_2582051	Microbiology, Adolfo Lutz Institut	Microbiology, Adolfo Lutz Institut	Souza,M.C.O., Matsumoto,T.K., Vaz-de-Lima,L.R.A., Sato,N.N., Salgado,M.M., Requejo,H.I.Z., Hong,M.A., Barbosa,M.L., Oliveira,C.A.F., Benelli,P.C., Shimokawa,R.Y., Pecchini,R., Berezin,E., Duarte,S.P., Schvartsman,C., Durigon,E.L. and Ueda,M.
EPI_ISL_2582053, EPI_ISL_2582056, EPI_ISL_2582057, EPI_ISL_2582060, EPI_ISL_2582061, EPI_ISL_2582064, EPI_ISL_2582065, EPI_ISL_2582067, EPI_ISL_2582070, EPI_ISL_2582071, EPI_ISL_2582074, EPI_ISL_2582075, EPI_ISL_2582077			
see above	Virologia, Universidad EL BOSQUE	Virologia, Universidad EL BOSQUE	Avila,V., Calvo,E.P., Barbosa,J., Velandia-Romero,M.L. and Castellanos,J.E.
EPI_ISL_2582211	Virologia, Facultad de Ciencias	Virologia, Facultad de Ciencias	Frabasile,S., Facal,L. and Arbiza,J.
EPI_ISL_2582298, EPI_ISL_2582299, EPI_ISL_2582302, EPI_ISL_2582303	J. Craig Venter Institute	J. Craig Venter Institute	Shabman,R., Fedorova,N., Puri,V., Shrivastava,S., Amedeo,P., Isom,R., Hu,L., Pickett,B., Novotny,M., Durbin,A., Rocchi,I., Williams,T., Hall,C.B., Tesini,B.L., Schnabel,K.C., Walsh,E.E. and Caserta,M.
EPI_ISL_2582438, EPI_ISL_2582439, EPI_ISL_2582442, EPI_ISL_2582443, EPI_ISL_2582448, EPI_ISL_2582449, EPI_ISL_2582451, EPI_ISL_2582454, EPI_ISL_2582456, EPI_ISL_2582457, EPI_ISL_2582460, EPI_ISL_2582461, EPI_ISL_2582463, EPI_ISL_2582465, EPI_ISL_2582468, EPI_ISL_2582469, EPI_ISL_2582471, EPI_ISL_2582476, EPI_ISL_2582477, EPI_ISL_2582479, EPI_ISL_2582481, EPI_ISL_2582484, EPI_ISL_2582487, EPI_ISL_2582491, EPI_ISL_2582493, EPI_ISL_2582502, EPI_ISL_2582503, EPI_ISL_2582505, EPI_ISL_2582508, EPI_ISL_2582509, EPI_ISL_2582513, EPI_ISL_2582519			
see above	Broad Institute of MIT & Harvard	Broad Institute of MIT & Harvard	Newman,R.M., Zody,M.C., DeVincenzo,J.P., Grad,Y., Lipsitch,M., Murphy,R., Fitzgerald,M., Young,S., Gargeya,S., Poon,T.W., Charlebois,P., Weiner,B., Yang,X., Piper,M.E., McCowan,C., Ireland,A., Levin,J., Malboeuf,C., Qu,J., Chapman,S.B., Murphy,C., Wortman,J., Nusbaum,C. and Birren,B.
EPI_ISL_2582521	J. Craig Venter Institute	J. Craig Venter Institute	Shabman,R., Fedorova,N., Puri,V., Shrivastava,S., Amedeo,P., Isom,R., Hu,L., Pickett,B., Novotny,M., Durbin,A., Rocchi,I., Williams,T., Hall,C.B., Tesini,B.L., Schnabel,K.C., Walsh,E.E. and Caserta,M.
EPI_ISL_2582523, EPI_ISL_2582533	Depto Microbiologia, Instituto de Ciencias Biomedicas, Universidade de Sao Paulo	Depto Microbiologia, Instituto de Ciencias Biomedicas, Universidade de Sao Paulo	Moura,F.E.A., Thomazelli,L.M., Candido,E.D.O., Florencio,C.M.G.D., Pereira,S.A.R., Oliveira,F.M.S., Alves,A.A., Ocadaque,C.J. and Durigon,E.L.
EPI_ISL_2582616, EPI_ISL_2582623, EPI_ISL_2582625	Broad Institute of MIT & Harvard	Broad Institute of MIT & Harvard	Newman,R.M., Zody,M.C., DeVincenzo,J.P., Grad,Y., Lipsitch,M., Murphy,R., Fitzgerald,M., Young,S., Gargeya,S., Poon,T.W., Charlebois,P., Weiner,B., Yang,X., Piper,M.E., McCowan,C., Ireland,A., Levin,J., Malboeuf,C., Qu,J., Chapman,S.B., Murphy,C., Wortman,J., Nusbaum,C. and Birren,B.
EPI_ISL_2582630	Pediatrics - Infectious Diseases, Medical College of Wisconsin	Pediatrics - Infectious Diseases, Medical College of Wisconsin	Rebuffo-Scheer,C., Bose,M.E., He,J., Khaja,S., Ulatowski,M., Beck,E.T., Fan,J., Kumar,S., Nelson,M.I. and Henrickson,K.J.
EPI_ISL_2582631, EPI_ISL_2582633	J. Craig Venter Institute	J. Craig Venter Institute	Shabman,R., Fedorova,N., Puri,V., Shrivastava,S., Amedeo,P., Isom,R., Hu,L., Pickett,B., Novotny,M., Durbin,A., Rocchi,I., Williams,T., Hall,C.B., Tesini,B.L., Schnabel,K.C., Walsh,E.E. and Caserta,M.
EPI_ISL_2582635	Broad Institute of MIT & Harvard	Broad Institute of MIT & Harvard	Newman,R.M., Zody,M.C., DeVincenzo,J.P., Grad,Y., Lipsitch,M., Murphy,R., Fitzgerald,M., Young,S., Gargeya,S., Poon,T.W., Charlebois,P., Weiner,B., Yang,X., Piper,M.E., McCowan,C., Ireland,A., Levin,J., Malboeuf,C., Qu,J., Chapman,S.B., Murphy,C., Wortman,J., Nusbaum,C. and Birren,B.
EPI_ISL_2582656, EPI_ISL_2582657, EPI_ISL_2582661, EPI_ISL_2582663, EPI_ISL_2582666	Dina J., University Hospital of Caen, Virology	Dina J., University Hospital of Caen, Virology	Dina,J.
EPI_ISL_2582688, EPI_ISL_2582691, EPI_ISL_2582694, EPI_ISL_2582696, EPI_ISL_2582699, EPI_ISL_2582701	Broad Institute of MIT & Harvard	Broad Institute of MIT & Harvard	Newman,R.M., Zody,M.C., DeVincenzo,J.P., Grad,Y., Lipsitch,M., Murphy,R., Fitzgerald,M., Young,S., Gargeya,S., Poon,T.W., Charlebois,P., Weiner,B., Yang,X., Piper,M.E., McCowan,C., Ireland,A., Levin,J., Malboeuf,C., Qu,J., Chapman,S.B., Murphy,C., Wortman,J., Nusbaum,C. and Birren,B.
EPI_ISL_2582703	J. Craig Venter Institute	J. Craig Venter Institute	Shabman,R., Fedorova,N., Puri,V., Shrivastava,S., Amedeo,P., Isom,R., Hu,L., Pickett,B., Novotny,M., Durbin,A., Rocchi,I., Williams,T., Hall,C.B., Tesini,B.L., Schnabel,K.C., Walsh,E.E. and Caserta,M.
EPI_ISL_2582706, EPI_ISL_2582709, EPI_ISL_2582711, EPI_ISL_2582714, EPI_ISL_2582716, EPI_ISL_2582719, EPI_ISL_2582722, EPI_ISL_2582724, EPI_ISL_2582727, EPI_ISL_2582729, EPI_ISL_2582732, EPI_ISL_2582735, EPI_ISL_2582738, EPI_ISL_2582740, EPI_ISL_2582743, EPI_ISL_2582745, EPI_ISL_2582747, EPI_ISL_2582750, EPI_ISL_2582751, EPI_ISL_2582756, EPI_ISL_2582758, EPI_ISL_2582760, EPI_ISL_2582764			
see above	Broad Institute of MIT & Harvard	Broad Institute of MIT & Harvard	Newman,R.M., Zody,M.C., DeVincenzo,J.P., Grad,Y., Lipsitch,M., Murphy,R., Fitzgerald,M., Young,S., Gargeya,S., Poon,T.W., Charlebois,P., Weiner,B., Yang,X., Piper,M.E., McCowan,C., Ireland,A., Levin,J., Malboeuf,C., Qu,J., Chapman,S.B., Murphy,C., Wortman,J., Nusbaum,C. and Birren,B.
EPI_ISL_2582766	Infectious Disease Initiative, Broad Institute	Infectious Disease Initiative, Broad Institute	Newman,R.M., Zody,M.C., DeVincenzo,J.P., Grad,Y., Lipsitch,M., Murphy,R., Fitzgerald,M., Young,S., Gargeya,S., Poon,T.W., Charlebois,P., Weiner,B., Yang,X., Piper,M.E., McCowan,C., Ireland,A., Levin,J., Malboeuf,C., Qu,J., Chapman,S.B., Murphy,C., Wortman,J., Nusbaum,C. and Birren,B.
EPI_ISL_2582768	Broad Institute of MIT & Harvard	Broad Institute of MIT & Harvard	Newman,R.M., Zody,M.C., DeVincenzo,J.P., Grad,Y., Lipsitch,M., Murphy,R., Fitzgerald,M., Young,S., Gargeya,S., Poon,T.W., Charlebois,P., Weiner,B., Yang,X., Piper,M.E., McCowan,C., Ireland,A., Levin,J., Malboeuf,C., Qu,J., Chapman,S.B., Murphy,C., Wortman,J., Nusbaum,C. and Birren,B.
EPI_ISL_2582775, EPI_ISL_2582778, EPI_ISL_2582782, EPI_ISL_2582847	J. Craig Venter Institute	J. Craig Venter Institute	Shabman,R., Fedorova,N., Puri,V., Shrivastava,S., Amedeo,P., Isom,R., Hu,L., Pickett,B., Novotny,M., Durbin,A., Rocchi,I., Williams,T., Hall,C.B., Tesini,B.L., Schnabel,K.C., Walsh,E.E. and Caserta,M.
EPI_ISL_2582857, EPI_ISL_2582859, EPI_ISL_2582861, EPI_ISL_2582869	Pediatrics - Infectious Diseases, Medical College of Wisconsin	Pediatrics - Infectious Diseases, Medical College of Wisconsin	Rebuffo-Scheer,C., Bose,M.E., He,J., Khaja,S., Ulatowski,M., Beck,E.T., Fan,J., Kumar,S., Nelson,M.I. and Henrickson,K.J.
EPI_ISL_2582873, EPI_ISL_2582875	J. Craig Venter Institute	J. Craig Venter Institute	Shabman,R., Fedorova,N., Puri,V., Shrivastava,S., Amedeo,P., Isom,R., Hu,L., Pickett,B., Novotny,M., Durbin,A., Rocchi,I., Williams,T., Hall,C.B., Tesini,B.L., Schnabel,K.C., Walsh,E.E. and Caserta,M.
EPI_ISL_2582878	Broad Institute of MIT & Harvard	Broad Institute of MIT & Harvard	Newman,R.M., Zody,M.C., DeVincenzo,J.P., Grad,Y., Lipsitch,M., Murphy,R., Fitzgerald,M., Young,S., Gargeya,S., Poon,T.W., Charlebois,P., Weiner,B., Yang,X., Piper,M.E., McCowan,C., Ireland,A., Levin,J., Malboeuf,C., Qu,J., Chapman,S.B., Murphy,C., Wortman,J., Nusbaum,C. and Birren,B.
EPI_ISL_2582886	J. Craig Venter Institute	J. Craig Venter Institute	Shabman,R., Fedorova,N., Puri,V., Shrivastava,S., Amedeo,P., Isom,R., Hu,L., Pickett,B., Novotny,M., Durbin,A., Rocchi,I., Williams,T., Hall,C.B., Tesini,B.L., Schnabel,K.C., Walsh,E.E. and Caserta,M.
EPI_ISL_2582892, EPI_ISL_2582893	Pediatrics - Infectious Diseases, Medical College of Wisconsin	Pediatrics - Infectious Diseases, Medical College of Wisconsin	Rebuffo-Scheer,C., Bose,M.E., He,J., Khaja,S., Ulatowski,M., Beck,E.T., Fan,J., Kumar,S., Nelson,M.I. and Henrickson,K.J.
EPI_ISL_2582901	J. Craig Venter Institute	J. Craig Venter Institute	Shabman,R., Fedorova,N., Puri,V., Shrivastava,S., Amedeo,P., Isom,R., Hu,L., Pickett,B., Novotny,M., Durbin,A., Rocchi,I., Williams,T., Hall,C.B., Tesini,B.L., Schnabel,K.C., Walsh,E.E. and Caserta,M.
EPI_ISL_2582920	Dopazo J., Centro Nacional de Biotecnologia - CSIC, BioInformatica, Universidad Autonoma	Dopazo J., Centro Nacional de Biotecnologia - CSIC, BioInformatica, Universidad Autonoma	Dopazo,J.
EPI_ISL_2582923, EPI_ISL_2582926, EPI_ISL_2582927, EPI_ISL_2582930, EPI_ISL_2582933	J. Craig Venter Institute	J. Craig Venter Institute	Shabman,R., Fedorova,N., Puri,V., Shrivastava,S., Amedeo,P., Isom,R., Hu,L., Pickett,B., Novotny,M., Durbin,A., Rocchi,I., Williams,T., Hall,C.B., Tesini,B.L., Schnabel,K.C., Walsh,E.E. and Caserta,M.
EPI_ISL_2582935, EPI_ISL_2582938, EPI_ISL_2582941, EPI_ISL_2582944	Broad Institute of MIT & Harvard	Broad Institute of MIT & Harvard	Newman,R.M., Zody,M.C., DeVincenzo,J.P., Grad,Y., Lipsitch,M., Murphy,R., Fitzgerald,M., Young,S., Gargeya,S., Poon,T.W., Charlebois,P., Weiner,B., Yang,X., Piper,M.E., McCowan,C., Ireland,A., Levin,J., Malboeuf,C., Qu,J., Chapman,S.B., Murphy,C., Wortman,J., Nusbaum,C. and Birren,B.
EPI_ISL_2582946, EPI_ISL_2582949, EPI_ISL_2582954	J. Craig Venter Institute	J. Craig Venter Institute	Shabman,R., Fedorova,N., Puri,V., Shrivastava,S., Amedeo,P., Isom,R., Hu,L., Pickett,B., Novotny,M., Durbin,A., Rocchi,I., Williams,T., Hall,C.B., Tesini,B.L., Schnabel,K.C., Walsh,E.E. and Caserta,M.
EPI_ISL_2583029, EPI_ISL_2583046, EPI_ISL_2583048, EPI_ISL_2583054	Broad Institute of MIT & Harvard	Broad Institute of MIT & Harvard	Newman,R.M., Zody,M.C., DeVincenzo,J.P., Grad,Y., Lipsitch,M., Murphy,R., Fitzgerald,M., Young,S., Gargeya,S., Poon,T.W., Charlebois,P., Weiner,B., Yang,X., Piper,M.E., McCowan,C., Ireland,A., Levin,J., Malboeuf,C., Qu,J., Chapman,S.B., Murphy,C., Wortman,J., Nusbaum,C. and Birren,B.
EPI_ISL_2583057, EPI_ISL_2583065	Pediatrics - Infectious Diseases, Medical College of Wisconsin	Pediatrics - Infectious Diseases, Medical College of Wisconsin	Rebuffo-Scheer,C., Bose,M.E., He,J., Khaja,S., Ulatowski,M., Beck,E.T., Fan,J., Kumar,S., Nelson,M.I. and Henrickson,K.J.
EPI_ISL_2583074, EPI_ISL_2583080, EPI_ISL_2583082	Broad Institute of MIT & Harvard	Broad Institute of MIT & Harvard	Newman,R.M., Zody,M.C., DeVincenzo,J.P., Grad,Y., Lipsitch,M., Murphy,R., Fitzgerald,M., Young,S., Gargeya,S., Poon,T.W., Charlebois,P., Weiner,B., Yang,X., Piper,M.E., McCowan,C., Ireland,A., Levin,J., Malboeuf,C., Qu,J., Chapman,S.B., Murphy,C., Wortman,J., Nusbaum,C. and Birren,B.
EPI_ISL_2583083	Pediatrics - Infectious Diseases, Medical College of Wisconsin	Pediatrics - Infectious Diseases, Medical College of Wisconsin	Rebuffo-Scheer,C., Bose,M.E., He,J., Khaja,S., Ulatowski,M., Beck,E.T., Fan,J., Kumar,S., Nelson,M.I. and Henrickson,K.J.
EPI_ISL_2583085, EPI_ISL_2583087, EPI_ISL_2583092	Broad Institute of MIT & Harvard	Broad Institute of MIT & Harvard	Newman,R.M., Zody,M.C., DeVincenzo,J.P., Grad,Y., Lipsitch,M., Murphy,R., Fitzgerald,M., Young,S., Gargeya,S., Poon,T.W., Charlebois,P., Weiner,B., Yang,X., Piper,M.E., McCowan,C., Ireland,A., Levin,J., Malboeuf,C., Qu,J., Chapman,S.B., Murphy,C., Wortman,J., Nusbaum,C. and Birren,B.
EPI_ISL_2583094, EPI_ISL_2583095, EPI_ISL_2583097, EPI_ISL_2583098, EPI_ISL_2583100, EPI_ISL_2583101, EPI_ISL_2583103, EPI_ISL_2583105, EPI_ISL_2583106, EPI_ISL_2583108, EPI_ISL_2583110, EPI_ISL_2583111, EPI_ISL_2583114, EPI_ISL_2583115, EPI_ISL_2583117, EPI_ISL_2583119, EPI_ISL_2583121			
see above	Depto Microbiologia, Instituto de Ciencias Biomedicas, Universidade de Sao Paulo	Depto Microbiologia, Instituto de Ciencias Biomedicas, Universidade de Sao Paulo	Moura,F.E.A., Thomazelli,L.M., Candido,E.D.O., Florencio,C.M.G.D., Pereira,S.A.R., Oliveira,F.M.S., Alves,A.A., Ocadaque,C.J. and Durigon,E.L.
EPI_ISL_2583122	Microbiology, Adolfo Lutz Institut	Microbiology, Adolfo Lutz Institut	Souza,M.C.O., Matsumoto,T.K., Vaz-de-Lima,L.R.A., Sato,N.N., Salgado,M.M., Requejo,H.I.Z., Hong,M.A., Barbosa,M.L., Oliveira,C.A.F., Benelli,P.C., Shimokawa,R.Y., Pecchini,R., Berezin,E., Duarte,S.P., Schvartsman,C., Durigon,E.L. and Ueda,M.
EPI_ISL_2585205	J. Craig Venter Institute	J. Craig Venter Institute	Lorenzi,H., Town,C., Halpin,R., Bera,J., Ransier,A., Fedorova,N., Stockwell,T., Amedeo,P., Appalla,L., Bishop,B., Edworthy,P., Gupta,N., Hoover,J., Katzel,D., Li,K., Schobel,S., Shrivastava,S., Thovarai,V., Wang,S., Rebuffo-Scheer,C., Fan,J., He,J., Kehl,S.C., Lederboer,N., Jurgens,L.A., Bose,M.E., Beck,E.T., Kumar,S., Noyola,D.E., Wentworth,D.E. and Henrickson,K.J.
EPI_ISL_2585209, EPI_ISL_2585210, EPI_ISL_2585211	Centre for Infectious Diseases, University of Edinburgh	Centre for Infectious Diseases, University of Edinburgh	Gaunt,E.R.
EPI_ISL_2585212	Centre for Infectious Diseases, University of Edinburgh	Centre for Infectious Diseases, University of Edinburgh	Gaunt,E.R., Jansen,R.R., Poovorawan,Y., Toms,G.L., Templeton,K.E. and Simmonds,P.
EPI_ISL_2585273, EPI_ISL_2585295, EPI_ISL_2585296	KEMRI Wellcome Trust Research Programme	KEMRI Wellcome Trust Research Programme	Agoti,C.N., Otieno,J.R., Munywoki,P.K., Mwihuri,A.G., Cane,P.A., Nokes,D.J., Kellam,P. and Cotten,M.L.
EPI_ISL_2585298	Epidemiology and Demography Department, KEMRI - Wellcome Trust Research Collaborative Programme	Epidemiology and Demography Department, KEMRI - Wellcome Trust Research Collaborative Programme	Agoti,C.N., Munywoki,P.K., Phan,M.V.T., Otieno,J.R., Kamau,E., Bett,A., Githinji,G., Medley,G.F., Cane,P.A., Kellam,P., Cotten,M.L. and Nokes,D.J.
EPI_ISL_2585303	KEMRI Wellcome Trust Research Programme	KEMRI Wellcome Trust Research Programme	Agoti,C.N., Otieno,J.R., Munywoki,P.K., Mwihuri,A.G., Cane,P.A., Nokes,D.J., Kellam,P. and Cotten,M.L.
EPI_ISL_2585304	Virology, NAMRU-61	Virology, NAMRU-61	Garcia,J., Sovero,M. and Halsey,E.S.
EPI_ISL_2585340, EPI_ISL_2585341, EPI_ISL_2585342, EPI_ISL_2585344, EPI_ISL_2585345, EPI_ISL_2585346, EPI_ISL_2585348, EPI_ISL_2585349	Infectious Diseases Group, Malaghan Institute of Medical Research	Infectious Diseases Group, Malaghan Institute of Medical Research	Rich,F.J., Matheson,J.W., Cohet,C., Grimwood,K., Penny,D., Hendy,M.D., Huang,Q.S. and Kirman,J.R.
EPI_ISL_2585350	J. Craig Venter Institute	J. Craig Venter Institute	Lorenzi,H., Town,C., Halpin,R., Bera,J., Ransier,A., Fedorova,N., Stockwell,T., Amedeo,P., Appalla,L., Bishop,B., Edworthy,P., Gupta,N., Hoover,J., Katzel,D., Li,K., Schobel,S., Shrivastava,S., Thovarai,V., Wang,S., Rebuffo-Scheer,C., Fan,J., He,J., Kehl,S.C., Lederboer,N., Jurgens,L.A., Bose,M.E., Beck,E.T., Kumar,S., Noyola,D.E., Wentworth,D.E. and Henrickson,K.J.
EPI_ISL_2585516, EPI_ISL_2585537, EPI_ISL_2585539, EPI_ISL_2585541, EPI_ISL_2585542, EPI_ISL_2585544	Centre for Infectious Diseases, University of Edinburgh	Centre for Infectious Diseases, University of Edinburgh	Gaunt,E.R.
EPI_ISL_2585548	Botany and Microbiology, College of Science, King Saud University	Botany and Microbiology, College of Science, King Saud University	Farrag,M.A., Amer,H.M. and Almajhdi,F.N.

EPI_ISL_2585572	Medical Microbiology (Interdisciplinary Program), Graduate School, Chulalongkorn University	Medical Microbiology (Interdisciplinary Program), Graduate School, Chulalongkorn University	Nilwong,O., Bhattarakosol,P. and Kowitdamrong,E.
EPI_ISL_2585649	Hiroyuki Tsukagoshi Gunma Prefectural Institute of Public Health and Environmental Sciences	Hiroyuki Tsukagoshi Gunma Prefectural Institute of Public Health and Environmental Sciences	Tsukagoshi,H., Kimura,H., Noda,M. and Mizuta,K.
EPI_ISL_2585666	Microbiology, Faculty of Medicine, Chulalongkorn University	Microbiology, Faculty of Medicine, Chulalongkorn University	Boonyasuppayakorn,S., Kowitdamrong,E. and Bhattarakosol,P.
EPI_ISL_2585668, EPI_ISL_2585670	Hiroyuki Tsukagoshi Gunma Prefectural Institute of Public Health and Environmental Sciences	Hiroyuki Tsukagoshi Gunma Prefectural Institute of Public Health and Environmental Sciences	Tsukagoshi,H., Kimura,H., Noda,M. and Mizuta,K.
EPI_ISL_2585672, EPI_ISL_2585674, EPI_ISL_2585676, EPI_ISL_2585678	Mika Arakawa tochigi prefectural institute of public health and environmental science	Mika Arakawa tochigi prefectural institute of public health and environmental science	Arakawa,M., Kimura,H., Noda,M. and Sugai,K.
EPI_ISL_2585701, EPI_ISL_2585702	Hiroyuki Tsukagoshi Gunma Prefectural Institute of Public Health and Environmental Sciences	Hiroyuki Tsukagoshi Gunma Prefectural Institute of Public Health and Environmental Sciences	Tsukagoshi,H., Kimura,H., Noda,M. and Mizuta,K.
EPI_ISL_2585706, EPI_ISL_2585707, EPI_ISL_2585709, EPI_ISL_2585710, EPI_ISL_2585712, EPI_ISL_2585713, EPI_ISL_2585715, EPI_ISL_2585717, EPI_ISL_2585719, EPI_ISL_2585720, EPI_ISL_2585722, EPI_ISL_2585724			
see above	Mika Arakawa tochigi prefectural institute of public health and environmental science	Mika Arakawa tochigi prefectural institute of public health and environmental science	Arakawa,M., Kimura,H., Noda,M. and Sugai,K.
EPI_ISL_2585725, EPI_ISL_2585727, EPI_ISL_2585729	Microbiology, Faculty of Medicine, Chulalongkorn University	Microbiology, Faculty of Medicine, Chulalongkorn University	Boonyasuppayakorn,S., Kowitdamrong,E. and Bhattarakosol,P.
EPI_ISL_2585763, EPI_ISL_2585765	Department of Medical Microbiology, Faculty of Medicine, University Malaya, Lembah Pantai, Kuala Lumpur	Department of Medical Microbiology, Faculty of Medicine, University Malaya, Lembah Pantai, Kuala Lumpur	Khor,C.S., Sam,I.C. and Chan,Y.F.
EPI_ISL_2585769, EPI_ISL_2585771, EPI_ISL_2585773, EPI_ISL_2585775, EPI_ISL_2585776, EPI_ISL_2585778, EPI_ISL_2585780, EPI_ISL_2585782, EPI_ISL_2585783, EPI_ISL_2585785, EPI_ISL_2585787			
see above	Microbiology, Faculty of Medicine, Chulalongkorn University	Microbiology, Faculty of Medicine, Chulalongkorn University	Boonyasuppayakorn,S., Kowitdamrong,E. and Bhattarakosol,P.
EPI_ISL_2585812, EPI_ISL_2585814	Medical Virology, University of Pretoria	Medical Virology, University of Pretoria	van Niekerk,S. and Venter,M.P.
EPI_ISL_2585815, EPI_ISL_2585817, EPI_ISL_2585819	Medical Virology, University of Pretoria	Medical Virology, University of Pretoria	van Niekerk,S. and Venter,M.
EPI_ISL_2585821, EPI_ISL_2585823	Medical Virology, University of Pretoria	Medical Virology, University of Pretoria	van Niekerk,S. and Venter,M.P.
EPI_ISL_2585828, EPI_ISL_2585830	Izumi Kushibuchi Tochigi prefectural Institute of Public Health and Enviromental Science	Izumi Kushibuchi Tochigi prefectural Institute of Public Health and Enviromental Science	Kushibuchi,I. and Kobayashi,M.
EPI_ISL_2585832, EPI_ISL_2585834	Mika Arakawa tochigi prefectural institute of public health and environmental science	Mika Arakawa tochigi prefectural institute of public health and environmental science	Arakawa,M., Kimura,H., Noda,M. and Sugai,K.
EPI_ISL_2585836, EPI_ISL_2585837	Masahiro Yamaguchi Niigata University, Graduate School of Medical and Dental Sciences, Division of Public Health, Department of Infectious Disease Control and Internal Medicine	Masahiro Yamaguchi Niigata University, Graduate School of Medical and Dental Sciences, Division of Public Health, Department of Infectious Disease Control and Internal Medicine	Yamaguchi,M., Sano,Y., Dapat,I.C., Saito,R., Suzuki,Y., Kumaki,A., Shobugawa,Y., Dapat,C., Uchiyama,M. and Suzuki,H.
EPI_ISL_2585839, EPI_ISL_2585841, EPI_ISL_2585863	Department of Medical Microbiology, Faculty of Medicine, University Malaya, Lembah Pantai, Kuala Lumpur	Department of Medical Microbiology, Faculty of Medicine, University Malaya, Lembah Pantai, Kuala Lumpur	Khor,C.S., Sam,I.C. and Chan,Y.F.
EPI_ISL_2585865, EPI_ISL_2585867	Medical Virology, University of Pretoria	Medical Virology, University of Pretoria	van Niekerk,S. and Venter,M.P.
EPI_ISL_2585869	Medical Virology, University of Pretoria	Medical Virology, University of Pretoria	van Niekerk,S. and Venter,M.
EPI_ISL_2585871, EPI_ISL_2585873, EPI_ISL_2585875, EPI_ISL_2585877, EPI_ISL_2585879, EPI_ISL_2585881, EPI_ISL_2585882, EPI_ISL_2585884, EPI_ISL_2585887	Medical Virology, University of Pretoria	Medical Virology, University of Pretoria	van Niekerk,S. and Venter,M.P.
EPI_ISL_2585888	Medical Virology, University of Pretoria	Medical Virology, University of Pretoria	van Niekerk,S. and Venter,M.
EPI_ISL_2585890, EPI_ISL_2585892	Medical Virology, University of Pretoria	Medical Virology, University of Pretoria	van Niekerk,S. and Venter,M.P.
EPI_ISL_2585894, EPI_ISL_2585896	Masahiro Yamaguchi Niigata University, Graduate School of Medical and Dental Sciences, Division of Public Health, Department of Infectious Disease Control and Internal Medicine	Masahiro Yamaguchi Niigata University, Graduate School of Medical and Dental Sciences, Division of Public Health, Department of Infectious Disease Control and Internal Medicine	Yamaguchi,M., Sano,Y., Dapat,I.C., Saito,R., Suzuki,Y., Kumaki,A., Shobugawa,Y., Dapat,C., Uchiyama,M. and Suzuki,H.
EPI_ISL_2585898	Yugo Shobugawa Niigata University Graduate School of Medical and Dental Sciences, Department of Public Health	Yugo Shobugawa Niigata University Graduate School of Medical and Dental Sciences, Department of Public Health	Shobugawa,Y., Saito,R. and Suzuki,H.
EPI_ISL_2585901, EPI_ISL_2585903, EPI_ISL_2585907, EPI_ISL_2585909, EPI_ISL_2585911	Department of Medical Microbiology, Faculty of Medicine, University Malaya, Lembah Pantai, Kuala Lumpur	Department of Medical Microbiology, Faculty of Medicine, University Malaya, Lembah Pantai, Kuala Lumpur	Khor,C.S., Sam,I.C. and Chan,Y.F.
EPI_ISL_2585912	Hiroyuki Tsukagoshi Gunma Prefectural Institute of Public Health and Environmental Sciences	Hiroyuki Tsukagoshi Gunma Prefectural Institute of Public Health and Environmental Sciences	Tsukagoshi,H., Kimura,H., Noda,M. and Mizuta,K.
EPI_ISL_2585914	Michiko Okamoto Tohoku University Graduate School of Medicine, Virology	Michiko Okamoto Tohoku University Graduate School of Medicine, Virology	Okamoto,M. and Nishimura,H.
EPI_ISL_2585916, EPI_ISL_2585920, EPI_ISL_2585921, EPI_ISL_2585923, EPI_ISL_2585925, EPI_ISL_2585927	Izumi Kushibuchi Tochigi prefectural Institute of Public Health and Enviromental Science	Izumi Kushibuchi Tochigi prefectural Institute of Public Health and Enviromental Science	Kushibuchi,I. and Kobayashi,M.
EPI_ISL_2585932, EPI_ISL_2585934, EPI_ISL_2585936, EPI_ISL_2585937, EPI_ISL_2585939, EPI_ISL_2585941, EPI_ISL_2585943, EPI_ISL_2585944	Mika Arakawa tochigi prefectural institute of public health and environmental science	Mika Arakawa tochigi prefectural institute of public health and environmental science	Arakawa,M., Kimura,H., Noda,M. and Sugai,K.
EPI_ISL_2585951, EPI_ISL_2585953	Department of Medical Microbiology, Faculty of Medicine, University Malaya, Lembah Pantai, Kuala Lumpur	Department of Medical Microbiology, Faculty of Medicine, University Malaya, Lembah Pantai, Kuala Lumpur	Khor,C.S., Sam,I.C. and Chan,Y.F.
EPI_ISL_2585956, EPI_ISL_2585958, EPI_ISL_2585959, EPI_ISL_2585965	Medical Virology, University of Pretoria	Medical Virology, University of Pretoria	van Niekerk,S. and Venter,M.P.
EPI_ISL_2585968	Department of Medical Microbiology, Faculty of Medicine, University Malaya, Lembah Pantai, Kuala Lumpur	Department of Medical Microbiology, Faculty of Medicine, University Malaya, Lembah Pantai, Kuala Lumpur	Khor,C.S., Sam,I.C. and Chan,Y.F.
EPI_ISL_2585975	Hiroyuki Tsukagoshi Gunma Prefectural Institute of Public Health and Environmental Sciences	Hiroyuki Tsukagoshi Gunma Prefectural Institute of Public Health and Environmental Sciences	Tsukagoshi,H., Kimura,H., Noda,M. and Mizuta,K.
EPI_ISL_2585981, EPI_ISL_2585982, EPI_ISL_2585984, EPI_ISL_2585986, EPI_ISL_2585988, EPI_ISL_2585990, EPI_ISL_2585992, EPI_ISL_2585994, EPI_ISL_2585996, EPI_ISL_2585997, EPI_ISL_2585999, EPI_ISL_2586001, EPI_ISL_2586003			
see above	Izumi Kushibuchi Tochigi prefectural Institute of Public Health and Enviromental Science	Izumi Kushibuchi Tochigi prefectural Institute of Public Health and Enviromental Science	Kushibuchi,I. and Kobayashi,M.
EPI_ISL_2586011, EPI_ISL_2586013, EPI_ISL_2586014, EPI_ISL_2586016, EPI_ISL_2586018, EPI_ISL_2586020, EPI_ISL_2586021, EPI_ISL_2586023, EPI_ISL_2586025, EPI_ISL_2586027, EPI_ISL_2586029, EPI_ISL_2586031, EPI_ISL_2586032			
see above	Mika Arakawa tochigi prefectural institute of public health and environmental science	Mika Arakawa tochigi prefectural institute of public health and environmental science	Arakawa,M., Kimura,H., Noda,M. and Sugai,K.
EPI_ISL_2586044, EPI_ISL_2586046, EPI_ISL_2586048, EPI_ISL_2586051	Medical Virology, University of Pretoria	Medical Virology, University of Pretoria	van Niekerk,S. and Venter,M.P.
EPI_ISL_2586053	Medical Virology, University of Pretoria	Medical Virology, University of Pretoria	van Niekerk,S. and Venter,M.
EPI_ISL_2586055	Medical Virology, University of Pretoria	Medical Virology, University of Pretoria	van Niekerk,S. and Venter,M.P.
EPI_ISL_2586057	Medical Virology, University of Pretoria	Medical Virology, University of Pretoria	van Niekerk,S. and Venter,M.
EPI_ISL_2586059, EPI_ISL_2586060, EPI_ISL_2586062	Medical Virology, University of Pretoria	Medical Virology, University of Pretoria	van Niekerk,S. and Venter,M.P.
EPI_ISL_2586064	Masahiro Yamaguchi Niigata University, Graduate School of Medical and Dental Sciences, Division of Public Health, Department of Infectious Disease Control and Internal Medicine	Masahiro Yamaguchi Niigata University, Graduate School of Medical and Dental Sciences, Division of Public Health, Department of Infectious Disease Control and Internal Medicine	Yamaguchi,M., Sano,Y., Dapat,I.C., Saito,R., Suzuki,Y., Kumaki,A., Shobugawa,Y., Dapat,C., Uchiyama,M. and Suzuki,H.
EPI_ISL_2586066, EPI_ISL_2586068, EPI_ISL_2586070	Department of Medical Microbiology, Faculty of Medicine, University Malaya, Lembah Pantai, Kuala Lumpur	Department of Medical Microbiology, Faculty of Medicine, University Malaya, Lembah Pantai, Kuala Lumpur	Khor,C.S., Sam,I.C. and Chan,Y.F.
EPI_ISL_2586091, EPI_ISL_2586093	Akira Suzuki Touhoku university, Dpartment of virology	Akira Suzuki Touhoku university, Dpartment of virology	Ohno,A., Suzuki,A. and Oshitani,H.
EPI_ISL_2586100	Izumi Kushibuchi Tochigi prefectural Institute of Public Health and Enviromental Science	Izumi Kushibuchi Tochigi prefectural Institute of Public Health and Enviromental Science	Kushibuchi,I. and Kobayashi,M.
EPI_ISL_2586105, EPI_ISL_2586107, EPI_ISL_2586109	Department of Medical Microbiology, Faculty of Medicine, University Malaya, Lembah Pantai, Kuala Lumpur	Department of Medical Microbiology, Faculty of Medicine, University Malaya, Lembah Pantai, Kuala Lumpur	Khor,C.S., Sam,I.C. and Chan,Y.F.
EPI_ISL_2586126, EPI_ISL_2586127	Medical Virology, University of Pretoria	Medical Virology, University of Pretoria	van Niekerk,S. and Venter,M.P.
EPI_ISL_2586129	Medical Virology, University of Pretoria	Medical Virology, University of Pretoria	van Niekerk,S. and Venter,M.
EPI_ISL_2586133, EPI_ISL_2586134, EPI_ISL_2586136, EPI_ISL_2586138	Medical Virology, University of Pretoria	Medical Virology, University of Pretoria	van Niekerk,S. and Venter,M.P.
EPI_ISL_2586152, EPI_ISL_2586154, EPI_ISL_2586156	Medical Virology, University of Pretoria	Medical Virology, University of Pretoria	van Niekerk,S. and Venter,M.
EPI_ISL_2586157, EPI_ISL_2586159	Medical Virology, University of Pretoria	Medical Virology, University of Pretoria	van Niekerk,S. and Venter,M.P.
EPI_ISL_2586161	Medical Virology, University of Pretoria	Medical Virology, University of Pretoria	van Niekerk,S. and Venter,M.
EPI_ISL_2586163, EPI_ISL_2586166, EPI_ISL_2586169	Medical Virology, University of Pretoria	Medical Virology, University of Pretoria	van Niekerk,S. and Venter,M.P.
EPI_ISL_2586170	Yugo Shobugawa Niigata University Graduate School of Medical and Dental Sciences, Department of Public Health	Yugo Shobugawa Niigata University Graduate School of Medical and Dental Sciences, Department of Public Health	Shobugawa,Y., Saito,R. and Suzuki,H.

EPI_ISL_2586172, EPI_ISL_2586174, EPI_ISL_2586176	Masahiro Yamaguchi Niigata University, Graduate School of Medical and Dental Sciences, Division of Public Health, Department of Infectious Disease Control and Internal Medicine	Masahiro Yamaguchi Niigata University, Graduate School of Medical and Dental Sciences, Division of Public Health, Department of Infectious Disease Control and Internal Medicine	Yamaguchi,M., Sano,Y., Dapat,I.C., Saito,R., Suzuki,Y., Kumaki,A., Shobugawa,Y., Dapat,C., Uchiyama,M. and Suzuki,H.
EPI_ISL_2586178	Hiroshi Suzuki, Niigata University Graduate School of Medical and Dental Sciences, Division of Public Health, Department of Infectious Control and International Medicine	Hiroshi Suzuki, Niigata University Graduate School of Medical and Dental Sciences, Division of Public Health, Department of Infectious Control and International Medicine	Sato,M., Saito,R., Sakai,T., Sano,Y., Nishikawa,M., Sasaki,A. and Suzuki,H.
EPI_ISL_2586180, EPI_ISL_2586181, EPI_ISL_2586183, EPI_ISL_2586184, EPI_ISL_2586186, EPI_ISL_2586187, EPI_ISL_2586189, EPI_ISL_2586191, EPI_ISL_2586196	Department of Medical Microbiology, Faculty of Medicine, University Malaya, Lembah Pantai, Kuala Lumpur	Department of Medical Microbiology, Faculty of Medicine, University Malaya, Lembah Pantai, Kuala Lumpur	Khor,C.S., Sam,I.C. and Chan,Y.F.
EPI_ISL_2586205, EPI_ISL_2586207, EPI_ISL_2586209, EPI_ISL_2586211, EPI_ISL_2586213, EPI_ISL_2586215, EPI_ISL_2586217, EPI_ISL_2586218, EPI_ISL_2586220, EPI_ISL_2586222, EPI_ISL_2586223, EPI_ISL_2586225, EPI_ISL_2586227, EPI_ISL_2586229, EPI_ISL_2586231, EPI_ISL_2586232, EPI_ISL_2586234			
see above	Akira Suzuki Touhoku university, Dpartment of virology	Akira Suzuki Touhoku university, Dpartment of virology	Ohno,A., Suzuki,A. and Oshitani,H.
EPI_ISL_2586240, EPI_ISL_2586241	Department of Medical Microbiology, Faculty of Medicine, University Malaya, Lembah Pantai, Kuala Lumpur	Department of Medical Microbiology, Faculty of Medicine, University Malaya, Lembah Pantai, Kuala Lumpur	Khor,C.S., Sam,I.C. and Chan,Y.F.
EPI_ISL_2586280	Medical Virology, University of Pretoria	Medical Virology, University of Pretoria	van Niekerk,S. and Venter,M.
EPI_ISL_2586282, EPI_ISL_2586284, EPI_ISL_2586286	Medical Virology, University of Pretoria	Medical Virology, University of Pretoria	van Niekerk,S. and Venter,M.P.
EPI_ISL_2586288	Medical Virology, University of Pretoria	Medical Virology, University of Pretoria	van Niekerk,S. and Venter,M.
EPI_ISL_2586289, EPI_ISL_2586290, EPI_ISL_2586291, EPI_ISL_2586292, EPI_ISL_2586293, EPI_ISL_2586296, EPI_ISL_2586297, EPI_ISL_2586298, EPI_ISL_2586299, EPI_ISL_2586301, EPI_ISL_2586303, EPI_ISL_2586305, EPI_ISL_2586307, EPI_ISL_2586309			
see above	Medical Virology, University of Pretoria	Medical Virology, University of Pretoria	van Niekerk,S. and Venter,M.P.
EPI_ISL_2586349, EPI_ISL_2586351	Medical Virology, University of Pretoria	Medical Virology, University of Pretoria	van Niekerk,S. and Venter,M.
EPI_ISL_2586352, EPI_ISL_2586354, EPI_ISL_2586358, EPI_ISL_2586360, EPI_ISL_2586361, EPI_ISL_2586363, EPI_ISL_2586365	Medical Virology, University of Pretoria	Medical Virology, University of Pretoria	van Niekerk,S. and Venter,M.P.
EPI_ISL_2586367, EPI_ISL_2586368, EPI_ISL_2586372, EPI_ISL_2586374, EPI_ISL_2586375	Masahiro Yamaguchi Niigata University, Graduate School of Medical and Dental Sciences, Division of Public Health, Department of Infectious Disease Control and Internal Medicine	Masahiro Yamaguchi Niigata University, Graduate School of Medical and Dental Sciences, Division of Public Health, Department of Infectious Disease Control and Internal Medicine	Yamaguchi,M., Sano,Y., Dapat,I.C., Saito,R., Suzuki,Y., Kumaki,A., Shobugawa,Y., Dapat,C., Uchiyama,M. and Suzuki,H.
EPI_ISL_2586377, EPI_ISL_2586378, EPI_ISL_2586380, EPI_ISL_2586382, EPI_ISL_2586395	Department of Medical Microbiology, Faculty of Medicine, University Malaya, Lembah Pantai, Kuala Lumpur	Department of Medical Microbiology, Faculty of Medicine, University Malaya, Lembah Pantai, Kuala Lumpur	Khor,C.S., Sam,I.C. and Chan,Y.F.
EPI_ISL_2586446, EPI_ISL_2586447, EPI_ISL_2586449, EPI_ISL_2586451, EPI_ISL_2586453, EPI_ISL_2586455, EPI_ISL_2586456, EPI_ISL_2586458, EPI_ISL_2586460, EPI_ISL_2586462, EPI_ISL_2586464, EPI_ISL_2586465, EPI_ISL_2586467, EPI_ISL_2586468, EPI_ISL_2586470, EPI_ISL_2586471, EPI_ISL_2586473, EPI_ISL_2586475, EPI_ISL_2586477, EPI_ISL_2586479, EPI_ISL_2586481, EPI_ISL_2586482, EPI_ISL_2586484, EPI_ISL_2586486, EPI_ISL_2586488, EPI_ISL_2586490, EPI_ISL_2586492, EPI_ISL_2586494, EPI_ISL_2586496, EPI_ISL_2586506, EPI_ISL_2586509, EPI_ISL_2586511, EPI_ISL_2586514, EPI_ISL_2586516, EPI_ISL_2586518, EPI_ISL_2586521, EPI_ISL_2586523, EPI_ISL_2586524, EPI_ISL_2586527, EPI_ISL_2586529, EPI_ISL_2586531, EPI_ISL_2586534			
see above	Akira Suzuki Touhoku university, Dpartment of virology	Akira Suzuki Touhoku university, Dpartment of virology	Ohno,A., Suzuki,A. and Oshitani,H.
EPI_ISL_2586581, EPI_ISL_2586583	Epidemiologia i Bioestadistica, IDIBAPS	Epidemiologia i Bioestadistica, IDIBAPS	Roca,A., Loscertales,M.P., Quinto,L., Perez-Brena,P., Vaz,N., Alonso,P.L. and Saiz,J.C.
EPI_ISL_2586585, EPI_ISL_2586606	Department of Medical Microbiology, Faculty of Medicine, University Malaya, Lembah Pantai, Kuala Lumpur	Department of Medical Microbiology, Faculty of Medicine, University Malaya, Lembah Pantai, Kuala Lumpur	Khor,C.S., Sam,I.C. and Chan,Y.F.
EPI_ISL_2586691, EPI_ISL_2586693, EPI_ISL_2586695, EPI_ISL_2586697	Epidemiologia i Bioestadistica, IDIBAPS	Epidemiologia i Bioestadistica, IDIBAPS	Roca,A., Loscertales,M.P., Quinto,L., Perez-Brena,P., Vaz,N., Alonso,P.L. and Saiz,J.C.
EPI_ISL_2586699	Masahiro Yamaguchi Niigata University, Graduate School of Medical and Dental Sciences, Division of Public Health, Department of Infectious Disease Control and Internal Medicine	Masahiro Yamaguchi Niigata University, Graduate School of Medical and Dental Sciences, Division of Public Health, Department of Infectious Disease Control and Internal Medicine	Yamaguchi,M., Sano,Y., Dapat,I.C., Saito,R., Suzuki,Y., Kumaki,A., Shobugawa,Y., Dapat,C., Uchiyama,M. and Suzuki,H.
EPI_ISL_2586700, EPI_ISL_2586704, EPI_ISL_2586706, EPI_ISL_2586712, EPI_ISL_2586714, EPI_ISL_2586716	Masaji Nakamura Okinawa Prefectural Institute of Health and Environment, Department of Biological Sciences	Masaji Nakamura Okinawa Prefectural Institute of Health and Environment, Department of Biological Sciences	Nakamura,M., Itokazu,K. and Taira,K.
EPI_ISL_2586725	Division of Immunity & Infection, The University of Birmingham, Medical School	Division of Immunity & Infection, The University of Birmingham, Medical School	Scott,P.D., Ochola,R., Ngama,M.J., Okiro,E.A., Nokes,D.J., Medley,G.F. and Cane,P.A.
EPI_ISL_2586737	Centre for Infectious Diseases, University of Edinburgh	Centre for Infectious Diseases, University of Edinburgh	Gaunt,E.R., Jansen,R.R., Poovorawan,Y., Toms,G.L., Templeton,K.E. and Simmonds,P.
EPI_ISL_2586738	Division of Immunity & Infection, The University of Birmingham, Medical School	Division of Immunity & Infection, The University of Birmingham, Medical School	Scott,P.D., Ochola,R., Ngama,M.J., Okiro,E.A., Nokes,D.J., Medley,G.F. and Cane,P.A.
EPI_ISL_2586740	Centre for Infectious Diseases, University of Edinburgh	Centre for Infectious Diseases, University of Edinburgh	Gaunt,E.R.
EPI_ISL_2586742	Division of Immunity & Infection, The University of Birmingham, Medical School	Division of Immunity & Infection, The University of Birmingham, Medical School	Scott,P.D., Ochola,R., Ngama,M.J., Okiro,E.A., Nokes,D.J., Medley,G.F. and Cane,P.A.
EPI_ISL_2586746	Centre for Infectious Diseases, University of Edinburgh	Centre for Infectious Diseases, University of Edinburgh	Gaunt,E.R.
EPI_ISL_2586748, EPI_ISL_2586750	Division of Immunity & Infection, The University of Birmingham, Medical School	Division of Immunity & Infection, The University of Birmingham, Medical School	Scott,P.D., Ochola,R., Ngama,M.J., Okiro,E.A., Nokes,D.J., Medley,G.F. and Cane,P.A.
EPI_ISL_2586753	Centre for Infectious Diseases, University of Edinburgh	Centre for Infectious Diseases, University of Edinburgh	Gaunt,E.R., Jansen,R.R., Poovorawan,Y., Toms,G.L., Templeton,K.E. and Simmonds,P.
EPI_ISL_2586755	Centre for Infectious Diseases, University of Edinburgh	Centre for Infectious Diseases, University of Edinburgh	Gaunt,E.R.
EPI_ISL_2586757, EPI_ISL_2586759, EPI_ISL_2586761	Division of Immunity & Infection, The University of Birmingham, Medical School	Division of Immunity & Infection, The University of Birmingham, Medical School	Scott,P.D., Ochola,R., Ngama,M.J., Okiro,E.A., Nokes,D.J., Medley,G.F. and Cane,P.A.
EPI_ISL_2586763	Division of Immunity & Infection, The University of Birmingham, Medical School	Division of Immunity & Infection, The University of Birmingham, Medical School	Scott,P.D., Ochola,R., Ngama,M., Okiro,E.A., Nokes,D.J., Medley,G.F. and Cane,P.A.
EPI_ISL_2586765, EPI_ISL_2586766, EPI_ISL_2586768, EPI_ISL_2586770, EPI_ISL_2586772, EPI_ISL_2586776, EPI_ISL_2586778, EPI_ISL_2586779, EPI_ISL_2586781, EPI_ISL_2586783, EPI_ISL_2586785, EPI_ISL_2586787, EPI_ISL_2586789, EPI_ISL_2586791, EPI_ISL_2586793, EPI_ISL_2586795, EPI_ISL_2586796, EPI_ISL_2586798, EPI_ISL_2586800, EPI_ISL_2586802, EPI_ISL_2586803, EPI_ISL_2586805, EPI_ISL_2586807, EPI_ISL_2586808, EPI_ISL_2586810			
see above	Division of Immunity & Infection, The University of Birmingham, Medical School	Division of Immunity & Infection, The University of Birmingham, Medical School	Scott,P.D., Ochola,R., Ngama,M.J., Okiro,E.A., Nokes,D.J., Medley,G.F. and Cane,P.A.
EPI_ISL_2586813	Centre for Infectious Diseases, University of Edinburgh	Centre for Infectious Diseases, University of Edinburgh	Gaunt,E.R., Jansen,R.R., Poovorawan,Y., Toms,G.L., Templeton,K.E. and Simmonds,P.
EPI_ISL_2586815, EPI_ISL_2586819	Centre for Infectious Diseases, University of Edinburgh	Centre for Infectious Diseases, University of Edinburgh	Gaunt,E.R.
EPI_ISL_2586821	Centre for Infectious Diseases, University of Edinburgh	Centre for Infectious Diseases, University of Edinburgh	Gaunt,E.R., Jansen,R.R., Poovorawan,Y., Toms,G.L., Templeton,K.E. and Simmonds,P.
EPI_ISL_2586823	Centre for Infectious Diseases, University of Edinburgh	Centre for Infectious Diseases, University of Edinburgh	Gaunt,E.R.
EPI_ISL_2586825, EPI_ISL_2586828	Division of Immunity & Infection, The University of Birmingham, Medical School	Division of Immunity & Infection, The University of Birmingham, Medical School	Scott,P.D., Ochola,R., Ngama,M.J., Okiro,E.A., Nokes,D.J., Medley,G.F. and Cane,P.A.
EPI_ISL_2586829	Centre for Infectious Diseases, University of Edinburgh	Centre for Infectious Diseases, University of Edinburgh	Gaunt,E.R.
EPI_ISL_2586830	Centre for Infectious Diseases, University of Edinburgh	Centre for Infectious Diseases, University of Edinburgh	Gaunt,E.R., Jansen,R.R., Poovorawan,Y., Toms,G.L., Templeton,K.E. and Simmonds,P.
EPI_ISL_2586833	University Graduate School of Medicine, Virology	University Graduate School of Medicine, Virology	Okamoto,M. and Nishimura,H.
EPI_ISL_2586838, EPI_ISL_2586842, EPI_ISL_2586844, EPI_ISL_2586846	Centre for Infectious Diseases, University of Edinburgh	Centre for Infectious Diseases, University of Edinburgh	Gaunt,E.R.
EPI_ISL_2586849, EPI_ISL_2586851	Medical Microbiology (Interdisciplinary Program), Graduate School, Chulalongkorn University	Medical Microbiology (Interdisciplinary Program), Graduate School, Chulalongkorn University	Nilwong,O., Bhattarakosol,P. and Kowitdamrong,E.
EPI_ISL_2586857	Botany and Microbiology, College of Science, King Saud University	Botany and Microbiology, College of Science, King Saud University	Farrag,M.A., Amer,H.M. and Almajhdi,F.N.
EPI_ISL_2586863, EPI_ISL_2586888, EPI_ISL_2586934, EPI_ISL_2586945, EPI_ISL_2586947	Medical Microbiology (Interdisciplinary Program), Graduate School, Chulalongkorn University	Medical Microbiology (Interdisciplinary Program), Graduate School, Chulalongkorn University	Nilwong,O., Bhattarakosol,P. and Kowitdamrong,E.
EPI_ISL_2586959	Botany and Microbiology, College of Science, King Saud University	Botany and Microbiology, College of Science, King Saud University	Farrag,M.A., Amer,H.M. and Almajhdi,F.N.
EPI_ISL_2587073, EPI_ISL_2587076	Laboratorio de Biologia Viral, Instituto de Salud Carlos III	Laboratorio de Biologia Viral, Instituto de Salud Carlos III	Trento,A., Abrego,L., Rodriguez-Fernandez,R., Gonzalez-Sanchez,M.I., Gonzalez-Martinez,F., Delfaro,A., Pascale,J.M., Arbiza,J. and Melero,J.A.
EPI_ISL_2587079, EPI_ISL_2587081, EPI_ISL_2587083, EPI_ISL_2587085	Medical Microbiology (Interdisciplinary Program), Graduate School, Chulalongkorn University	Medical Microbiology (Interdisciplinary Program), Graduate School, Chulalongkorn University	Nilwong,O., Bhattarakosol,P. and Kowitdamrong,E.
EPI_ISL_2587134, EPI_ISL_2587136	Laboratorio de Biologia Viral, Instituto de Salud Carlos III	Laboratorio de Biologia Viral, Instituto de Salud Carlos III	Trento,A., Abrego,L., Rodriguez-Fernandez,R., Gonzalez-Sanchez,M.I., Gonzalez-Martinez,F., Delfaro,A., Pascale,J.M., Arbiza,J. and Melero,J.A.
EPI_ISL_2587138, EPI_ISL_2587139, EPI_ISL_2587143, EPI_ISL_2587145, EPI_ISL_2587146, EPI_ISL_2587148, EPI_ISL_2587150	Medical Microbiology (Interdisciplinary Program), Graduate School, Chulalongkorn University	Medical Microbiology (Interdisciplinary Program), Graduate School, Chulalongkorn University	Nilwong,O., Bhattarakosol,P. and Kowitdamrong,E.
EPI_ISL_2587161	Division of Immunity & Infection, The University of Birmingham, Medical School	Division of Immunity & Infection, The University of Birmingham, Medical School	Scott,P.D., Ochola,R., Ngama,M.J., Okiro,E.A., Nokes,D.J., Medley,G.F. and Cane,P.A.
EPI_ISL_2587167, EPI_ISL_2587168, EPI_ISL_2587170, EPI_ISL_2587172, EPI_ISL_2587174, EPI_ISL_2587176, EPI_ISL_2587178, EPI_ISL_2587179, EPI_ISL_2587181, EPI_ISL_2587183, EPI_ISL_2587185, EPI_ISL_2587187, EPI_ISL_2587189, EPI_ISL_2587190, EPI_ISL_2587192, EPI_ISL_2587194, EPI_ISL_2587196, EPI_ISL_2587197, EPI_ISL_2587199, EPI_ISL_2587201, EPI_ISL_2587203, EPI_ISL_2587205, EPI_ISL_2587206, EPI_ISL_2587208, EPI_ISL_2587210, EPI_ISL_2587212, EPI_ISL_2587213, EPI_ISL_2587215, EPI_ISL_2587217, EPI_ISL_2587219, EPI_ISL_2587221, EPI_ISL_2587222, EPI_ISL_2587224, EPI_ISL_2587226, EPI_ISL_2587227, EPI_ISL_2587229, EPI_ISL_2587231, EPI_ISL_2587233, EPI_ISL_2587235, EPI_ISL_2587236, EPI_ISL_2587238, EPI_ISL_2587240, EPI_ISL_2587241, EPI_ISL_2587243, EPI_ISL_2587245, EPI_ISL_2587247, EPI_ISL_2587249, EPI_ISL_2587251, EPI_ISL_2587253, EPI_ISL_2587255, EPI_ISL_2587256, EPI_ISL_2587258, EPI_ISL_2587260, EPI_ISL_2587262, EPI_ISL_2587264, EPI_ISL_2587266, EPI_ISL_2587268, EPI_ISL_2587270, EPI_ISL_2587271, EPI_ISL_2587273, EPI_ISL_2587275, EPI_ISL_2587277, EPI_ISL_2587279, EPI_ISL_2587281, EPI_ISL_2587283, EPI_ISL_2587285, EPI_ISL_2587287, EPI_ISL_2587288, EPI_ISL_2587290, EPI_ISL_2587292, EPI_ISL_2587294, EPI_ISL_2587296, EPI_ISL_2587298, EPI_ISL_2587300, EPI_ISL_2587301, EPI_ISL_2587303, EPI_ISL_2587305, EPI_ISL_2587307, EPI_ISL_2587309, EPI_ISL_2587311, EPI_ISL_2587312, EPI_ISL_2587314, EPI_ISL_2587316, EPI_ISL_2587318, EPI_ISL_2587320, EPI_ISL_2587321, EPI_ISL_2587323, EPI_ISL_2587325, EPI_ISL_2587327, EPI_ISL_2587329, EPI_ISL_2587331, EPI_ISL_2587332, EPI_ISL_2587334, EPI_ISL_2587336, EPI_ISL_2587338			
see above	Infectious Diseases Group, Malaghan Institute of Medical Research	Infectious Diseases Group, Malaghan Institute of Medical Research	Rich,F.J., Matheson,J.W., Cohet,C., Grimwood,K., Penny,D., Hendy,M.D., Huang,Q.S. and Kirman,J.R.
EPI_ISL_2587367	KEMRI Wellcome Trust Research Programme	KEMRI Wellcome Trust Research Programme	Agoti,C.N., Otieno,J.R., Munywoki,P.K., Mwihuri,A.G., Cane,P.A., Nokes,D.J., Kellam,P. and Cotten,M.L.
EPI_ISL_2587477, EPI_ISL_2587479, EPI_ISL_2587481	Department of Medical Microbiology, Faculty of Medicine, University Malaya, Lembah Pantai, Kuala Lumpur	Department of Medical Microbiology, Faculty of Medicine, University Malaya, Lembah Pantai, Kuala Lumpur	Khor,C.S., Sam,I.C. and Chan,Y.F.
EPI_ISL_2587483, EPI_ISL_2587485	Infectious Diseases Group, Malaghan Institute of Medical Research	Infectious Diseases Group, Malaghan Institute of Medical Research	Rich,F.J., Matheson,J.W., Cohet,C., Grimwood,K., Penny,D., Hendy,M.D., Huang,Q.S. and Kirman,J.R.
EPI_ISL_2587487, EPI_ISL_2587490	Department of Medical Microbiology, Faculty of Medicine, University Malaya, Lembah Pantai, Kuala Lumpur	Department of Medical Microbiology, Faculty of Medicine, University Malaya, Lembah Pantai, Kuala Lumpur	Khor,C.S., Sam,I.C. and Chan,Y.F.

EPI_ISL_2587503	KEMRI Wellcome Trust Research Programme	KEMRI Wellcome Trust Research Programme	Agoti,C.N., Otieno,J.R., Munywoki,P.K., Mwihuri,A.G., Cane,P.A., Nokes,D.J., Kellam,P. and Cotten,M.L.
EPI_ISL_2587505	Medical Virology, University of Pretoria	Medical Virology, University of Pretoria	Venter,M.P., van Niekerk,S., Rakgantso,A. and Bent,N.
EPI_ISL_2587507	Medical Virology, University of Pretoria	Medical Virology, University of Pretoria	van Niekerk,S. and Venter,M.P.
EPI_ISL_2587508	Medical Virology, University of Pretoria	Medical Virology, University of Pretoria	van Niekerk,S. and Venter,M.
EPI_ISL_2587510, EPI_ISL_2587512, EPI_ISL_2587514, EPI_ISL_2587516	Medical Virology, University of Pretoria	Medical Virology, University of Pretoria	van Niekerk,S. and Venter,M.P.
EPI_ISL_2587517	Medical Virology, University of Pretoria	Medical Virology, University of Pretoria	van Niekerk,S. and Venter,M.
EPI_ISL_2587518	Medical Virology, University of Pretoria	Medical Virology, University of Pretoria	van Niekerk,S. and Venter,M.P.
EPI_ISL_2587520, EPI_ISL_2587522, EPI_ISL_2587523	Medical Virology, University of Pretoria	Medical Virology, University of Pretoria	van Niekerk,S. and Venter,M.
EPI_ISL_2587525, EPI_ISL_2587527, EPI_ISL_2587529, EPI_ISL_2587531, EPI_ISL_2587533, EPI_ISL_2587535, EPI_ISL_2587537	Medical Virology, University of Pretoria	Medical Virology, University of Pretoria	van Niekerk,S. and Venter,M.P.
EPI_ISL_2587538	Medical Virology, University of Pretoria	Medical Virology, University of Pretoria	van Niekerk,S. and Venter,M.
EPI_ISL_2587540, EPI_ISL_2587542	Medical Virology, University of Pretoria	Medical Virology, University of Pretoria	van Niekerk,S. and Venter,M.P.
EPI_ISL_2587543	Medical Virology, University of Pretoria	Medical Virology, University of Pretoria	van Niekerk,S. and Venter,M.
EPI_ISL_2587545	Medical Virology, University of Pretoria	Medical Virology, University of Pretoria	van Niekerk,S. and Venter,M.P.
EPI_ISL_2587547	Medical Virology, University of Pretoria	Medical Virology, University of Pretoria	van Niekerk,S. and Venter,M.
EPI_ISL_2587549	Medical Virology, University of Pretoria	Medical Virology, University of Pretoria	Venter,M., van Niekerk,S., Rakgantso,A. and Bent,N.
EPI_ISL_2587552, EPI_ISL_2587554	Medical Virology, University of Pretoria	Medical Virology, University of Pretoria	van Niekerk,S. and Venter,M.P.
EPI_ISL_2587556, EPI_ISL_2587558	Medical Virology, University of Pretoria	Medical Virology, University of Pretoria	van Niekerk,S. and Venter,M.
EPI_ISL_2587560, EPI_ISL_2587562, EPI_ISL_2587564, EPI_ISL_2587565, EPI_ISL_2587567, EPI_ISL_2587569	Medical Virology, University of Pretoria	Medical Virology, University of Pretoria	van Niekerk,S. and Venter,M.P.
EPI_ISL_2587571, EPI_ISL_2587573, EPI_ISL_2587574, EPI_ISL_2587578	Medical Virology, University of Pretoria	Medical Virology, University of Pretoria	van Niekerk,S. and Venter,M.
EPI_ISL_2587580, EPI_ISL_2587582	Medical Virology, University of Pretoria	Medical Virology, University of Pretoria	van Niekerk,S. and Venter,M.P.
EPI_ISL_2587584	Medical Virology, University of Pretoria	Medical Virology, University of Pretoria	van Niekerk,S. and Venter,M.
EPI_ISL_2587586, EPI_ISL_2587588	Medical Virology, University of Pretoria	Medical Virology, University of Pretoria	van Niekerk,S. and Venter,M.P.
EPI_ISL_2587593	Epidemiologia i Bioestadistica, IDIBAPS	Epidemiologia i Bioestadistica, IDIBAPS	Roca,A., Loscertales,M.P., Quinto,L., Perez-Brena,P., Vaz,N., Alonso,P.L. and Saiz,J.C.
EPI_ISL_2587597	Medical Virology, University of Pretoria	Medical Virology, University of Pretoria	van Niekerk,S. and Venter,M.
EPI_ISL_2587599, EPI_ISL_2587600, EPI_ISL_2587602	Medical Virology, University of Pretoria	Medical Virology, University of Pretoria	van Niekerk,S. and Venter,M.P.
EPI_ISL_2587604	Medical Virology, University of Pretoria	Medical Virology, University of Pretoria	van Niekerk,S. and Venter,M.
EPI_ISL_2587606, EPI_ISL_2587608	Medical Virology, University of Pretoria	Medical Virology, University of Pretoria	van Niekerk,S. and Venter,M.P.
EPI_ISL_2587609, EPI_ISL_2587611, EPI_ISL_2587613	Department of Medical Microbiology, Faculty of Medicine, University Malaya, Lembah Pantai, Kuala Lumpur	Department of Medical Microbiology, Faculty of Medicine, University Malaya, Lembah Pantai, Kuala Lumpur	Khor,C.S., Sam,I.C. and Chan,Y.F.
EPI_ISL_2587615, EPI_ISL_2587617	Taylor G.S., Virology laboratory, Bristol Public Health Laboratory	Taylor G.S., Virology laboratory, Bristol Public Health Laboratory	Taylor,G.S.
EPI_ISL_2587619	Mika Arakawa tochigi prefectural institute of public health and environmental science	Mika Arakawa tochigi prefectural institute of public health and environmental science	Arakawa,M., Kimura,H., Noda,M. and Sugai,K.
EPI_ISL_2587621	Taylor G.S., Virology laboratory, Bristol Public Health Laboratory	Taylor G.S., Virology laboratory, Bristol Public Health Laboratory	Taylor,G.S.
EPI_ISL_2587628	Division of Immunity & Infection, The University of Birmingham, Medical School	Division of Immunity & Infection, The University of Birmingham, Medical School	Scott,P.D., Ochola,R., Ngama,M.J., Okiro,E.A., Nokes,D.J., Medley,G.F. and Cane,P.A.
EPI_ISL_2587634	Rotzen Ostlund M., Microbiology, Tumor and Cell Biology, Karolinska Institutet, Karolinska University Hospital	Rotzen Ostlund M., Microbiology, Tumor and Cell Biology, Karolinska Institutet, Karolinska University Hospital	Rotzen Ostlund,M.
EPI_ISL_2587635	Taylor G.S., Virology laboratory, Bristol Public Health Laboratory	Taylor G.S., Virology laboratory, Bristol Public Health Laboratory	Taylor,G.S.
EPI_ISL_2587639	Epidemiologia i Bioestadistica, IDIBAPS	Epidemiologia i Bioestadistica, IDIBAPS	Roca,A., Loscertales,M.P., Quinto,L., Perez-Brena,P., Vaz,N., Alonso,P.L. and Saiz,J.C.
EPI_ISL_2587641	Taylor G.S., Virology laboratory, Bristol Public Health Laboratory	Taylor G.S., Virology laboratory, Bristol Public Health Laboratory	Taylor,G.S.
EPI_ISL_2587644	Division of Immunity & Infection, The University of Birmingham, Medical School	Division of Immunity & Infection, The University of Birmingham, Medical School	Scott,P.D., Ochola,R., Ngama,M.J., Okiro,E.A., Nokes,D.J., Medley,G.F. and Cane,P.A.
EPI_ISL_2587645	Hiroshi Suzuki, Niigata University Graduate School of Medical and Dental Sciences, Division of Public Health, Department of Infectious Control and International Medicine	Hiroshi Suzuki, Niigata University Graduate School of Medical and Dental Sciences, Division of Public Health, Department of Infectious Control and International Medicine	Sato,M., Saito,R., Sakai,T., Sano,Y., Nishikawa,M., Sasaki,A. and Suzuki,H.
EPI_ISL_2587646	Division of Immunity & Infection, The University of Birmingham, Medical School	Division of Immunity & Infection, The University of Birmingham, Medical School	Scott,P.D., Ochola,R., Ngama,M.J., Okiro,E.A., Nokes,D.J., Medley,G.F. and Cane,P.A.
EPI_ISL_2587647	Medical Virology, University of Pretoria	Medical Virology, University of Pretoria	van Niekerk,S. and Venter,M.P.
EPI_ISL_2587649, EPI_ISL_2587651	Centre for Infectious Diseases, University of Edinburgh	Centre for Infectious Diseases, University of Edinburgh	Gaunt,E.R.
EPI_ISL_2587653, EPI_ISL_2587655, EPI_ISL_2587656	Medical Virology, University of Pretoria	Medical Virology, University of Pretoria	van Niekerk,S. and Venter,M.P.
EPI_ISL_2587658	Medical Virology, University of Pretoria	Medical Virology, University of Pretoria	van Niekerk,S. and Venter,M.
EPI_ISL_2587662	Medical Virology, University of Pretoria	Medical Virology, University of Pretoria	van Niekerk,S. and Venter,M.P.
EPI_ISL_2587666, EPI_ISL_2587667	Medical Virology, University of Pretoria	Medical Virology, University of Pretoria	van Niekerk,S. and Venter,M.
EPI_ISL_2587669, EPI_ISL_2587671, EPI_ISL_2587673	Medical Virology, University of Pretoria	Medical Virology, University of Pretoria	van Niekerk,S. and Venter,M.P.
EPI_ISL_2587675	Medical Virology, University of Pretoria	Medical Virology, University of Pretoria	van Niekerk,S. and Venter,M.
EPI_ISL_2587677	Medical Virology, University of Pretoria	Medical Virology, University of Pretoria	van Niekerk,S. and Venter,M.P.
EPI_ISL_2587678	Medical Virology, University of Pretoria	Medical Virology, University of Pretoria	van Niekerk,S. and Venter,M.
EPI_ISL_2587680	Medical Virology, University of Pretoria	Medical Virology, University of Pretoria	van Niekerk,S. and Venter,M.P.
EPI_ISL_2587682, EPI_ISL_2587684	Medical Virology, University of Pretoria	Medical Virology, University of Pretoria	van Niekerk,S. and Venter,M.
EPI_ISL_2587686	Medical Virology, University of Pretoria	Medical Virology, University of Pretoria	van Niekerk,S. and Venter,M.P.
EPI_ISL_2587688, EPI_ISL_2587690, EPI_ISL_2587691	Medical Virology, University of Pretoria	Medical Virology, University of Pretoria	van Niekerk,S. and Venter,M.
EPI_ISL_2587693, EPI_ISL_2587695	Medical Virology, University of Pretoria	Medical Virology, University of Pretoria	van Niekerk,S. and Venter,M.P.
EPI_ISL_2587697, EPI_ISL_2587698	Medical Virology, University of Pretoria	Medical Virology, University of Pretoria	van Niekerk,S. and Venter,M.
EPI_ISL_2587700, EPI_ISL_2587702	Medical Virology, University of Pretoria	Medical Virology, University of Pretoria	van Niekerk,S. and Venter,M.P.
EPI_ISL_2587704	Medical Virology, University of Pretoria	Medical Virology, University of Pretoria	van Niekerk,S. and Venter,M.
EPI_ISL_2587705, EPI_ISL_2587708	Medical Virology, University of Pretoria	Medical Virology, University of Pretoria	van Niekerk,S. and Venter,M.P.
EPI_ISL_2587710	Medical Virology, University of Pretoria	Medical Virology, University of Pretoria	van Niekerk,S. and Venter,M.
EPI_ISL_2587711	Medical Virology, University of Pretoria	Medical Virology, University of Pretoria	van Niekerk,S. and Venter,M.P.
EPI_ISL_2587713, EPI_ISL_2587717, EPI_ISL_2587719	Medical Virology, University of Pretoria	Medical Virology, University of Pretoria	van Niekerk,S. and Venter,M.
EPI_ISL_2587721	Centre for Infectious Diseases, University of Edinburgh	Centre for Infectious Diseases, University of Edinburgh	Gaunt,E.R.
EPI_ISL_2587723	Masahiro Yamaguchi Niigata University, Graduate School of Medical and Dental Sciences, Division of Public Health, Department of Infectious Disease Control and Internal Medicine	Masahiro Yamaguchi Niigata University, Graduate School of Medical and Dental Sciences, Division of Public Health, Department of Infectious Disease Control and Internal Medicine	Yamaguchi,M., Sano,Y., Dapat,I.C., Saito,R., Suzuki,Y., Kumaki,A., Shobugawa,Y., Dapat,C., Uchiyama,M. and Suzuki,H.
EPI_ISL_2587725, EPI_ISL_2587726	Department of Medical Microbiology, Faculty of Medicine, University Malaya, Lembah Pantai, Kuala Lumpur	Department of Medical Microbiology, Faculty of Medicine, University Malaya, Lembah Pantai, Kuala Lumpur	Khor,C.S., Sam,I.C. and Chan,Y.F.
EPI_ISL_2587728	Centre for Infectious Diseases, University of Edinburgh	Centre for Infectious Diseases, University of Edinburgh	Gaunt,E.R.
EPI_ISL_2587730, EPI_ISL_2587732, EPI_ISL_2587734, EPI_ISL_2587735	Division of Immunity & Infection, The University of Birmingham, Medical School	Division of Immunity & Infection, The University of Birmingham, Medical School	Scott,P.D., Ochola,R., Ngama,M.J., Okiro,E.A., Nokes,D.J., Medley,G.F. and Cane,P.A.
EPI_ISL_2587737	Division of Immunity & Infection, The University of Birmingham, Medical School	Division of Immunity & Infection, The University of Birmingham, Medical School	Scott,P.D., Ochola,R., Ngama,M., Okiro,E.A., Nokes,D.J., Medley,G.F. and Cane,P.A.
EPI_ISL_2587739	Division of Immunity & Infection, The University of Birmingham, Medical School	Division of Immunity & Infection, The University of Birmingham, Medical School	Scott,P.D., Ochola,R., Ngama,M.J., Okiro,E.A., Nokes,D.J., Medley,G.F. and Cane,P.A.
EPI_ISL_2587741	Division of Immunity & Infection, The University of Birmingham, Medical School	Division of Immunity & Infection, The University of Birmingham, Medical School	Scott,P.D., Ochola,R., Ngama,M., Okiro,E.A., Nokes,D.J., Medley,G.F. and Cane,P.A.
EPI_ISL_2587743, EPI_ISL_2587744	Rotzen Ostlund M., Microbiology, Tumor and Cell Biology, Karolinska Institutet, Karolinska University Hospital	Rotzen Ostlund M., Microbiology, Tumor and Cell Biology, Karolinska Institutet, Karolinska University Hospital	Rotzen Ostlund,M.

EPI_ISL_2587746	Hiroyuki Tsukagoshi Gunma Prefectural Institute of Public Health and Environmental Sciences	Hiroyuki Tsukagoshi Gunma Prefectural Institute of Public Health and Environmental Sciences	Tsukagoshi,H., Kimura,H., Noda,M. and Mizuta,K.
EPI_ISL_2587748	Division of Immunity & Infection, The University of Birmingham, Medical School	Division of Immunity & Infection, The University of Birmingham, Medical School	Scott,P.D., Ochola,R., Ngama,M.J., Okiro,E.A., Nokes,D.J., Medley,G.F. and Cane,P.A.
EPI_ISL_2587750	Centre for Infectious Diseases, University of Edinburgh	Centre for Infectious Diseases, University of Edinburgh	Gaunt,E.R.
EPI_ISL_2587753	Centre for Infectious Diseases, University of Edinburgh	Centre for Infectious Diseases, University of Edinburgh	Gaunt,E.R., Jansen,R.R., Poovorawan,Y., Toms,G.L., Templeton,K.E. and Simmonds,P.
EPI_ISL_2587755, EPI_ISL_2587757, EPI_ISL_2587759, EPI_ISL_2587761, EPI_ISL_2587762, EPI_ISL_2587764, EPI_ISL_2587766, EPI_ISL_2587767, EPI_ISL_2587769, EPI_ISL_2587771	Division of Immunity & Infection, The University of Birmingham, Medical School	Division of Immunity & Infection, The University of Birmingham, Medical School	Scott,P.D., Ochola,R., Ngama,M.J., Okiro,E.A., Nokes,D.J., Medley,G.F. and Cane,P.A.
EPI_ISL_2587773	Division of Immunity & Infection, The University of Birmingham, Medical School	Division of Immunity & Infection, The University of Birmingham, Medical School	Scott,P.D., Ochola,R., Ngama,M., Okiro,E.A., Nokes,D.J., Medley,G.F. and Cane,P.A.
EPI_ISL_2587775, EPI_ISL_2587777, EPI_ISL_2587778, EPI_ISL_2587780	Division of Immunity & Infection, The University of Birmingham, Medical School	Division of Immunity & Infection, The University of Birmingham, Medical School	Scott,P.D., Ochola,R., Ngama,M.J., Okiro,E.A., Nokes,D.J., Medley,G.F. and Cane,P.A.
EPI_ISL_2587782	Division of Immunity & Infection, The University of Birmingham, Medical School	Division of Immunity & Infection, The University of Birmingham, Medical School	Scott,P.D., Ochola,R., Ngama,M., Okiro,E.A., Nokes,D.J., Medley,G.F. and Cane,P.A.
EPI_ISL_2587784	Epidemiologia i Bioestadistica, IDIBAPS	Epidemiologia i Bioestadistica, IDIBAPS	Roca,A., Loscertales,M.P., Quinto,L., Perez-Brena,P., Vaz,N., Alonso,P.L. and Saiz,J.C.
EPI_ISL_2587786	Division of Immunity & Infection, The University of Birmingham, Medical School	Division of Immunity & Infection, The University of Birmingham, Medical School	Scott,P.D., Ochola,R., Ngama,M.J., Okiro,E.A., Nokes,D.J., Medley,G.F. and Cane,P.A.
EPI_ISL_2587788, EPI_ISL_2587790, EPI_ISL_2587791, EPI_ISL_2587793	Medical Virology, University of Pretoria	Medical Virology, University of Pretoria	van Niekerk,S. and Venter,M.P.
EPI_ISL_2587795	Division of Immunity & Infection, The University of Birmingham, Medical School	Division of Immunity & Infection, The University of Birmingham, Medical School	Scott,P.D., Ochola,R., Ngama,M.J., Okiro,E.A., Nokes,D.J., Medley,G.F. and Cane,P.A.
EPI_ISL_2587797	Masahiro Yamaguchi Niigata University, Graduate School of Medical and Dental Sciences, Division of Public Health, Department of Infectious Disease Control and Internal Medicine	Masahiro Yamaguchi Niigata University, Graduate School of Medical and Dental Sciences, Division of Public Health, Department of Infectious Disease Control and Internal Medicine	Yamaguchi,M., Sano,Y., Dapat,I.C., Saito,R., Suzuki,Y., Kumaki,A., Shobugawa,Y., Dapat,C., Uchiyama,M. and Suzuki,H.
EPI_ISL_2587799, EPI_ISL_2587801, EPI_ISL_2587803, EPI_ISL_2587804, EPI_ISL_2587806, EPI_ISL_2587808	Division of Immunity & Infection, The University of Birmingham, Medical School	Division of Immunity & Infection, The University of Birmingham, Medical School	Scott,P.D., Ochola,R., Ngama,M.J., Okiro,E.A., Nokes,D.J., Medley,G.F. and Cane,P.A.
EPI_ISL_2587812	Centre for Infectious Diseases, University of Edinburgh	Centre for Infectious Diseases, University of Edinburgh	Gaunt,E.R.
EPI_ISL_2587814	Centre for Infectious Diseases, University of Edinburgh	Centre for Infectious Diseases, University of Edinburgh	Gaunt,E.R., Jansen,R.R., Poovorawan,Y., Toms,G.L., Templeton,K.E. and Simmonds,P.
EPI_ISL_2587815, EPI_ISL_2587817	Centre for Infectious Diseases, University of Edinburgh	Centre for Infectious Diseases, University of Edinburgh	Gaunt,E.R.
EPI_ISL_2587819, EPI_ISL_2587821, EPI_ISL_2587823, EPI_ISL_2587824, EPI_ISL_2587826	Division of Immunity & Infection, The University of Birmingham, Medical School	Division of Immunity & Infection, The University of Birmingham, Medical School	Scott,P.D., Ochola,R., Ngama,M.J., Okiro,E.A., Nokes,D.J., Medley,G.F. and Cane,P.A.
EPI_ISL_2587828	Division of Immunity & Infection, The University of Birmingham, Medical School	Division of Immunity & Infection, The University of Birmingham, Medical School	Scott,P.D., Ochola,R., Ngama,M., Okiro,E.A., Nokes,D.J., Medley,G.F. and Cane,P.A.
EPI_ISL_2587830, EPI_ISL_2587831, EPI_ISL_2587832, EPI_ISL_2587834, EPI_ISL_2587836, EPI_ISL_2587838, EPI_ISL_2587840	Division of Immunity & Infection, The University of Birmingham, Medical School	Division of Immunity & Infection, The University of Birmingham, Medical School	Scott,P.D., Ochola,R., Ngama,M.J., Okiro,E.A., Nokes,D.J., Medley,G.F. and Cane,P.A.
EPI_ISL_2587842, EPI_ISL_2587843	Division of Immunity & Infection, The University of Birmingham, Medical School	Division of Immunity & Infection, The University of Birmingham, Medical School	Scott,P.D., Ochola,R., Ngama,M., Okiro,E.A., Nokes,D.J., Medley,G.F. and Cane,P.A.
EPI_ISL_2587845	Division of Immunity & Infection, The University of Birmingham, Medical School	Division of Immunity & Infection, The University of Birmingham, Medical School	Scott,P.D., Ochola,R., Ngama,M.J., Okiro,E.A., Nokes,D.J., Medley,G.F. and Cane,P.A.
EPI_ISL_2587847	Division of Immunity & Infection, The University of Birmingham, Medical School	Division of Immunity & Infection, The University of Birmingham, Medical School	Scott,P.D., Ochola,R., Ngama,M., Okiro,E.A., Nokes,D.J., Medley,G.F. and Cane,P.A.
EPI_ISL_2587849, EPI_ISL_2587850, EPI_ISL_2587852	Division of Immunity & Infection, The University of Birmingham, Medical School	Division of Immunity & Infection, The University of Birmingham, Medical School	Scott,P.D., Ochola,R., Ngama,M.J., Okiro,E.A., Nokes,D.J., Medley,G.F. and Cane,P.A.
EPI_ISL_2587854	Division of Immunity & Infection, The University of Birmingham, Medical School	Division of Immunity & Infection, The University of Birmingham, Medical School	Scott,P.D., Ochola,R., Ngama,M., Okiro,E.A., Nokes,D.J., Medley,G.F. and Cane,P.A.
EPI_ISL_2587855	Division of Immunity & Infection, The University of Birmingham, Medical School	Division of Immunity & Infection, The University of Birmingham, Medical School	Scott,P.D., Ochola,R., Ngama,M.J., Okiro,E.A., Nokes,D.J., Medley,G.F. and Cane,P.A.
EPI_ISL_2587857, EPI_ISL_2587859	Division of Immunity & Infection, The University of Birmingham, Medical School	Division of Immunity & Infection, The University of Birmingham, Medical School	Scott,P.D., Ochola,R., Ngama,M., Okiro,E.A., Nokes,D.J., Medley,G.F. and Cane,P.A.
EPI_ISL_2587861, EPI_ISL_2587863, EPI_ISL_2587865, EPI_ISL_2587867, EPI_ISL_2587869, EPI_ISL_2587870, EPI_ISL_2587872, EPI_ISL_2587874, EPI_ISL_2587876, EPI_ISL_2587878, EPI_ISL_2587880, EPI_ISL_2587881, EPI_ISL_2587882, EPI_ISL_2587883			
see above	Division of Immunity & Infection, The University of Birmingham, Medical School	Division of Immunity & Infection, The University of Birmingham, Medical School	Scott,P.D., Ochola,R., Ngama,M.J., Okiro,E.A., Nokes,D.J., Medley,G.F. and Cane,P.A.
EPI_ISL_2587884	Division of Immunity & Infection, The University of Birmingham, Medical School	Division of Immunity & Infection, The University of Birmingham, Medical School	Scott,P.D., Ochola,R., Ngama,M., Okiro,E.A., Nokes,D.J., Medley,G.F. and Cane,P.A.
EPI_ISL_2587885, EPI_ISL_2587886	Laboratorio de Biologia Viral, Instituto de Salud Carlos III	Laboratorio de Biologia Viral, Instituto de Salud Carlos III	Trento,A., Abrego,L., Rodriguez-Fernandez,R., Gonzalez-Sanchez,M.I., Gonzalez-Martinez,F., Delfaro,A., Pascale,J.M., Arbiza,J. and Melero,J.A.
EPI_ISL_2587887	Department of Medical Microbiology, Faculty of Medicine, University Malaya, Lembah Pantai, Kuala Lumpur	Department of Medical Microbiology, Faculty of Medicine, University Malaya, Lembah Pantai, Kuala Lumpur	Khor,C.S., Sam,I.C. and Chan,Y.F.
EPI_ISL_2587888, EPI_ISL_2587889, EPI_ISL_2587890	Masahiro Yamaguchi Niigata University, Graduate School of Medical and Dental Sciences, Division of Public Health, Department of Infectious Disease Control and Internal Medicine	Masahiro Yamaguchi Niigata University, Graduate School of Medical and Dental Sciences, Division of Public Health, Department of Infectious Disease Control and Internal Medicine	Yamaguchi,M., Sano,Y., Dapat,I.C., Saito,R., Suzuki,Y., Kumaki,A., Shobugawa,Y., Dapat,C., Uchiyama,M. and Suzuki,H.
EPI_ISL_2587891	Hiroshi Suzuki, Niigata University Graduate School of Medical and Dental Sciences, Division of Public Health, Department of Infectious Control and International Medicine	Hiroshi Suzuki, Niigata University Graduate School of Medical and Dental Sciences, Division of Public Health, Department of Infectious Control and International Medicine	Sato,M., Saito,R., Sakai,T., Sano,Y., Nishikawa,M., Sasaki,A. and Suzuki,H.
EPI_ISL_2587892	Masahiro Yamaguchi Niigata University, Graduate School of Medical and Dental Sciences, Division of Public Health, Department of Infectious Disease Control and Internal Medicine	Masahiro Yamaguchi Niigata University, Graduate School of Medical and Dental Sciences, Division of Public Health, Department of Infectious Disease Control and Internal Medicine	Yamaguchi,M., Sano,Y., Dapat,I.C., Saito,R., Suzuki,Y., Kumaki,A., Shobugawa,Y., Dapat,C., Uchiyama,M. and Suzuki,H.
EPI_ISL_2587893	Division of Public Health, Department of Infectious Control and International Medicine, Niigata University Graduate School of Medical and Dental Sciences	Division of Public Health, Department of Infectious Control and International Medicine, Niigata University Graduate School of Medical and Dental Sciences	Dapat,I.C., Shobugawa,Y., Sano,Y., Saito,R., Sasaki,A., Suzuki,Y., Kumaki,A., Zaraket,H., Dapat,C., Oguma,T., Yamaguchi,M. and Suzuki,H.
EPI_ISL_2587894, EPI_ISL_2587895, EPI_ISL_2587896	Department of Medical Microbiology, Faculty of Medicine, University Malaya, Lembah Pantai, Kuala Lumpur	Department of Medical Microbiology, Faculty of Medicine, University Malaya, Lembah Pantai, Kuala Lumpur	Khor,C.S., Sam,I.C. and Chan,Y.F.
EPI_ISL_2587898	Centre for Infectious Diseases, University of Edinburgh	Centre for Infectious Diseases, University of Edinburgh	Gaunt,E.R., Jansen,R.R., Poovorawan,Y., Toms,G.L., Templeton,K.E. and Simmonds,P.
EPI_ISL_2587899	Virology, NAMRU-24	Virology, NAMRU-24	Garcia,J., Sovero,M. and Halsey,E.S.
EPI_ISL_2587901, EPI_ISL_2587903	Taylor G.S., Virology laboratory, Bristol Public Health Laboratory	Taylor G.S., Virology laboratory, Bristol Public Health Laboratory	Taylor,G.S.
EPI_ISL_2587904	Centre for Infectious Diseases, University of Edinburgh	Centre for Infectious Diseases, University of Edinburgh	Gaunt,E.R., Jansen,R.R., Poovorawan,Y., Toms,G.L., Templeton,K.E. and Simmonds,P.
EPI_ISL_2587907	Hiroyuki Tsukagoshi Gunma Prefectural Institute of Public Health and Environmental Sciences	Hiroyuki Tsukagoshi Gunma Prefectural Institute of Public Health and Environmental Sciences	Tsukagoshi,H., Kimura,H., Noda,M. and Mizuta,K.
EPI_ISL_2587908	Rotzen Ostlund M., Microbiology, Tumor and Cell Biology, Karolinska Institutet, Karolinska University Hospital	Rotzen Ostlund M., Microbiology, Tumor and Cell Biology, Karolinska Institutet, Karolinska University Hospital	Rotzen Ostlund,M.
EPI_ISL_2587909	Hiroyuki Tsukagoshi Gunma Prefectural Institute of Public Health and Environmental Sciences	Hiroyuki Tsukagoshi Gunma Prefectural Institute of Public Health and Environmental Sciences	Tsukagoshi,H., Kimura,H., Noda,M. and Mizuta,K.
EPI_ISL_2587910	Department of Medical Microbiology, Faculty of Medicine, University Malaya, Lembah Pantai, Kuala Lumpur	Department of Medical Microbiology, Faculty of Medicine, University Malaya, Lembah Pantai, Kuala Lumpur	Khor,C.S., Sam,I.C. and Chan,Y.F.
EPI_ISL_2587913	Centre for Infectious Diseases, University of Edinburgh	Centre for Infectious Diseases, University of Edinburgh	Gaunt,E.R., Jansen,R.R., Poovorawan,Y., Toms,G.L., Templeton,K.E. and Simmonds,P.
EPI_ISL_2587914	Hiroyuki Tsukagoshi Gunma Prefectural Institute of Public Health and Environmental Sciences	Hiroyuki Tsukagoshi Gunma Prefectural Institute of Public Health and Environmental Sciences	Tsukagoshi,H., Kimura,H., Noda,M. and Mizuta,K.
EPI_ISL_2587917, EPI_ISL_2587918	Mika Arakawa tochigi prefectural institute of public health and environmental science	Mika Arakawa tochigi prefectural institute of public health and environmental science	Arakawa,M., Kimura,H., Noda,M. and Sugai,K.
EPI_ISL_2587921	Medical Virology, University of Pretoria	Medical Virology, University of Pretoria	van Niekerk,S. and Venter,M.P.
EPI_ISL_2587922	Centre for Infectious Diseases, University of Edinburgh	Centre for Infectious Diseases, University of Edinburgh	Gaunt,E.R., Jansen,R.R., Poovorawan,Y., Toms,G.L., Templeton,K.E. and Simmonds,P.
EPI_ISL_2587923	Virology, NAMRU-59	Virology, NAMRU-59	Garcia,J., Sovero,M. and Halsey,E.S.
EPI_ISL_2587925	Virology, NAMRU-2	Virology, NAMRU-2	Garcia,J., Sovero,M. and Halsey,E.S.
EPI_ISL_2587927	Rotzen Ostlund M., Microbiology, Tumor and Cell Biology, Karolinska Institutet, Karolinska University Hospital	Rotzen Ostlund M., Microbiology, Tumor and Cell Biology, Karolinska Institutet, Karolinska University Hospital	Rotzen Ostlund,M.
EPI_ISL_2587930	Centre for Infectious Diseases, University of Edinburgh	Centre for Infectious Diseases, University of Edinburgh	Gaunt,E.R., Jansen,R.R., Poovorawan,Y., Toms,G.L., Templeton,K.E. and Simmonds,P.
EPI_ISL_2587932	Virology, NAMRU-13	Virology, NAMRU-13	Garcia,J., Sovero,M. and Halsey,E.S.
EPI_ISL_2587933	Virology, NAMRU-58	Virology, NAMRU-58	Garcia,J., Sovero,M. and Halsey,E.S.
EPI_ISL_2587934	Biological Sciences, University of Warwick	Biological Sciences, University of Warwick	Scott,P.D. and Cane,P.A.
EPI_ISL_2587938	Virology, NAMRU-26	Virology, NAMRU-26	Garcia,J., Sovero,M. and Halsey,E.S.
EPI_ISL_2587939	Virology, NAMRU-48	Virology, NAMRU-48	Garcia,J., Sovero,M. and Halsey,E.S.
EPI_ISL_2587942	Centre for Infectious Diseases, University of Edinburgh	Centre for Infectious Diseases, University of Edinburgh	Gaunt,E.R., Jansen,R.R., Poovorawan,Y., Toms,G.L., Templeton,K.E. and Simmonds,P.

EPI_ISL_2587945	Virology, NAMRU-12	Virology, NAMRU-12	Garcia,J., Sovero,M. and Halsey,E.S.
EPI_ISL_2587946	Virology, NAMRU-1	Virology, NAMRU-1	Garcia,J., Sovero,M. and Halsey,E.S.
EPI_ISL_2587947	Virology, NAMRU-54	Virology, NAMRU-54	Garcia,J., Sovero,M. and Halsey,E.S.
EPI_ISL_2587948	Virology, NAMRU-55	Virology, NAMRU-55	Garcia,J., Sovero,M. and Halsey,E.S.
EPI_ISL_2587949	Virology, NAMRU-3	Virology, NAMRU-3	Garcia,J., Sovero,M. and Halsey,E.S.
EPI_ISL_2587952	Centre for Infectious Diseases, University of Edinburgh	Centre for Infectious Diseases, University of Edinburgh	Gaunt,E.R., Jansen,R.R., Poovorawan,Y., Toms,G.L., Templeton,K.E. and Simmonds,P.
EPI_ISL_2587955	Hiroyuki Tsukagoshi Gunma Prefectural Institute of Public Health and Environmental Sciences	Hiroyuki Tsukagoshi Gunma Prefectural Institute of Public Health and Environmental Sciences	Tsukagoshi,H., Kimura,H., Noda,M. and Mizuta,K.
EPI_ISL_2587956	Biology, Gilead Sciences	Biology, Gilead Sciences	Stray,K.
EPI_ISL_2587957	Centre for Infectious Diseases, University of Edinburgh	Centre for Infectious Diseases, University of Edinburgh	Gaunt,E.R., Jansen,R.R., Poovorawan,Y., Toms,G.L., Templeton,K.E. and Simmonds,P.
EPI_ISL_2587958	Virology, NAMRU-57	Virology, NAMRU-57	Garcia,J., Sovero,M. and Halsey,E.S.
EPI_ISL_2587959	Virology, NAMRU-53	Virology, NAMRU-53	Garcia,J., Sovero,M. and Halsey,E.S.
EPI_ISL_2587960	Virology, NAMRU-32	Virology, NAMRU-32	Garcia,J., Sovero,M. and Halsey,E.S.
EPI_ISL_2587961	Virology, NAMRU-41	Virology, NAMRU-41	Garcia,J., Sovero,M. and Halsey,E.S.
EPI_ISL_2587962	Virology, NAMRU-35	Virology, NAMRU-35	Garcia,J., Sovero,M. and Halsey,E.S.
EPI_ISL_2587964	Department of Medical Microbiology, Faculty of Medicine, University Malaya, Lembah Pantai, Kuala Lumpur	Department of Medical Microbiology, Faculty of Medicine, University Malaya, Lembah Pantai, Kuala Lumpur	Khor,C.S., Sam,I.C. and Chan,Y.F.
EPI_ISL_2587966	Biology, Gilead Sciences	Biology, Gilead Sciences	Stray,K.
EPI_ISL_2587986	Centre for Infectious Diseases, University of Edinburgh	Centre for Infectious Diseases, University of Edinburgh	Gaunt,E.R.
EPI_ISL_2587987	Virology, NAMRU-60	Virology, NAMRU-60	Garcia,J., Sovero,M. and Halsey,E.S.
EPI_ISL_2587988	Virology, NAMRU-27	Virology, NAMRU-27	Garcia,J., Sovero,M. and Halsey,E.S.
EPI_ISL_2587993	Hiroyuki Tsukagoshi Gunma Prefectural Institute of Public Health and Environmental Sciences	Hiroyuki Tsukagoshi Gunma Prefectural Institute of Public Health and Environmental Sciences	Tsukagoshi,H., Kimura,H., Noda,M. and Mizuta,K.
EPI_ISL_2587995	Hiroshi Suzuki, Niigata University Graduate School of Medical and Dental Sciences, Division of Public Health, Department of Infectious Control and International Medicine	Hiroshi Suzuki, Niigata University Graduate School of Medical and Dental Sciences, Division of Public Health, Department of Infectious Control and International Medicine	Sato,M., Saito,R., Sakai,T., Sano,Y., Nishikawa,M., Sasaki,A. and Suzuki,H.
EPI_ISL_2587996	Masahiro Yamaguchi Niigata University, Graduate School of Medical and Dental Sciences, Division of Public Health, Department of Infectious Disease Control and Internal Medicine	Masahiro Yamaguchi Niigata University, Graduate School of Medical and Dental Sciences, Division of Public Health, Department of Infectious Disease Control and Internal Medicine	Yamaguchi,M., Sano,Y., Dapat,I.C., Saito,R., Suzuki,Y., Kumaki,A., Shobugawa,Y., Dapat,C., Uchiyama,M. and Suzuki,H.
EPI_ISL_2587997	Department of Medical Microbiology, Faculty of Medicine, University Malaya, Lembah Pantai, Kuala Lumpur	Department of Medical Microbiology, Faculty of Medicine, University Malaya, Lembah Pantai, Kuala Lumpur	Khor,C.S., Sam,I.C. and Chan,Y.F.
EPI_ISL_2588000	Rotzen Ostlund M., Microbiology, Tumor and Cell Biology, Karolinska Institutet, Karolinska University Hospital	Rotzen Ostlund M., Microbiology, Tumor and Cell Biology, Karolinska Institutet, Karolinska University Hospital	Rotzen Ostlund,M.
EPI_ISL_2588001	Microbiology, Faculty of Medicine, Chulalongkorn University	Microbiology, Faculty of Medicine, Chulalongkorn University	Boonyasuppayakorn,S., Kowitdamrong,E. and Bhattarakosol,P.
EPI_ISL_2588004, EPI_ISL_2588005, EPI_ISL_2588006, EPI_ISL_2588007	Medical Virology, University of Pretoria	Medical Virology, University of Pretoria	van Niekerk,S. and Venter,M.P.
EPI_ISL_2588008	Medical Virology, University of Pretoria	Medical Virology, University of Pretoria	van Niekerk,S. and Venter,M.
EPI_ISL_2588009, EPI_ISL_2588010	Medical Virology, University of Pretoria	Medical Virology, University of Pretoria	van Niekerk,S. and Venter,M.P.
EPI_ISL_2588011, EPI_ISL_2588012	Medical Virology, University of Pretoria	Medical Virology, University of Pretoria	van Niekerk,S. and Venter,M.
EPI_ISL_2588013	Medical Virology, University of Pretoria	Medical Virology, University of Pretoria	van Niekerk,S. and Venter,M.P.
EPI_ISL_2588014	Medical Virology, University of Pretoria	Medical Virology, University of Pretoria	van Niekerk,S. and Venter,M.
EPI_ISL_2588015	Medical Virology, University of Pretoria	Medical Virology, University of Pretoria	van Niekerk,S. and Venter,M.P.
EPI_ISL_2588017	Hiroyuki Tsukagoshi Gunma Prefectural Institute of Public Health and Environmental Sciences	Hiroyuki Tsukagoshi Gunma Prefectural Institute of Public Health and Environmental Sciences	Tsukagoshi,H., Kimura,H., Noda,M. and Mizuta,K.
EPI_ISL_2588018, EPI_ISL_2588019	Hiroshi Suzuki, Niigata University Graduate School of Medical and Dental Sciences, Division of Public Health, Department of Infectious Control and International Medicine	Hiroshi Suzuki, Niigata University Graduate School of Medical and Dental Sciences, Division of Public Health, Department of Infectious Control and International Medicine	Sato,M., Saito,R., Sakai,T., Sano,Y., Nishikawa,M., Sasaki,A. and Suzuki,H.
EPI_ISL_2588020	Masahiro Yamaguchi Niigata University, Graduate School of Medical and Dental Sciences, Division of Public Health, Department of Infectious Disease Control and Internal Medicine	Masahiro Yamaguchi Niigata University, Graduate School of Medical and Dental Sciences, Division of Public Health, Department of Infectious Disease Control and Internal Medicine	Yamaguchi,M., Sano,Y., Dapat,I.C., Saito,R., Suzuki,Y., Kumaki,A., Shobugawa,Y., Dapat,C., Uchiyama,M. and Suzuki,H.
EPI_ISL_2588021, EPI_ISL_2588022, EPI_ISL_2588023	Department of Medical Microbiology, Faculty of Medicine, University Malaya, Lembah Pantai, Kuala Lumpur	Department of Medical Microbiology, Faculty of Medicine, University Malaya, Lembah Pantai, Kuala Lumpur	Khor,C.S., Sam,I.C. and Chan,Y.F.
EPI_ISL_2588024	Epidemiologia i Bioestadistica, IDIBAPS	Epidemiologia i Bioestadistica, IDIBAPS	Roca,A., Loscertales,M.P., Quinto,L., Perez-Brena,P., Vaz,N., Alonso,P.L. and Saiz,J.C.
EPI_ISL_2588025	Microbiology, Faculty of Medicine, Chulalongkorn University	Microbiology, Faculty of Medicine, Chulalongkorn University	Boonyasuppayakorn,S., Kowitdamrong,E. and Bhattarakosol,P.
EPI_ISL_2588029	Centre for Infectious Diseases, University of Edinburgh	Centre for Infectious Diseases, University of Edinburgh	Gaunt,E.R., Jansen,R.R., Poovorawan,Y., Toms,G.L., Templeton,K.E. and Simmonds,P.
EPI_ISL_2588030, EPI_ISL_2588032, EPI_ISL_2588033	Centre for Infectious Diseases, University of Edinburgh	Centre for Infectious Diseases, University of Edinburgh	Gaunt,E.R.
EPI_ISL_2588034	Virology, NAMRU-64	Virology, NAMRU-64	Garcia,J., Sovero,M. and Halsey,E.S.
EPI_ISL_2588046	Biology, Gilead Sciences	Biology, Gilead Sciences	Stray,K.
EPI_ISL_2588055	Miwako SaikNorth America / USA Yokohama City Institute of Public Health	Miwako SaikNorth America / USA Yokohama City Institute of Public Health	SaikNorth America / USA,M., Kawakami,C., Usuku,S., Sasao,T. and Okubo,I.
EPI_ISL_2588076	Biology, Gilead Sciences	Biology, Gilead Sciences	Stray,K.
EPI_ISL_2588090	Centre for Infectious Diseases, University of Edinburgh	Centre for Infectious Diseases, University of Edinburgh	Gaunt,E.R., Jansen,R.R., Poovorawan,Y., Toms,G.L., Templeton,K.E. and Simmonds,P.
EPI_ISL_2588091	Virology, NAMRU-1	Virology, NAMRU-1	Garcia,J., Sovero,M. and Halsey,E.S.
EPI_ISL_2588095, EPI_ISL_2588096, EPI_ISL_2588097, EPI_ISL_2588098, EPI_ISL_2588099, EPI_ISL_2588101, EPI_ISL_2588102	Centre for Infectious Diseases, University of Edinburgh	Centre for Infectious Diseases, University of Edinburgh	Gaunt,E.R.
EPI_ISL_2588103	Biology, Gilead Sciences	Biology, Gilead Sciences	Stray,K.
EPI_ISL_2588128, EPI_ISL_2588129, EPI_ISL_2588130, EPI_ISL_2588131, EPI_ISL_2588133, EPI_ISL_2588134, EPI_ISL_2588135, EPI_ISL_2588136, EPI_ISL_2588137, EPI_ISL_2588138, EPI_ISL_2588139, EPI_ISL_2588140, EPI_ISL_2588141			
see above	Miwako SaikNorth America / USA Yokohama City Institute of Public Health	Miwako SaikNorth America / USA Yokohama City Institute of Public Health	SaikNorth America / USA,M., Kawakami,C., Usuku,S., Sasao,T. and Okubo,I.
EPI_ISL_2588144	Biology, Gilead Sciences	Biology, Gilead Sciences	Stray,K.
EPI_ISL_2588146	Medical Microbiology (Interdisciplinary Program), Graduate School, Chulalongkorn University	Medical Microbiology (Interdisciplinary Program), Graduate School, Chulalongkorn University	Nilwong,O., Bhattarakosol,P. and Kowitdamrong,E.
EPI_ISL_2588164	Biology, Gilead Sciences	Biology, Gilead Sciences	Stray,K.
EPI_ISL_2588166, EPI_ISL_2588167, EPI_ISL_2588168, EPI_ISL_2588169, EPI_ISL_2588170, EPI_ISL_2588171, EPI_ISL_2588172, EPI_ISL_2588173, EPI_ISL_2588174, EPI_ISL_2588175, EPI_ISL_2588176, EPI_ISL_2588177, EPI_ISL_2588178, EPI_ISL_2588179, EPI_ISL_2588180, EPI_ISL_2588181, EPI_ISL_2588182, EPI_ISL_2588183			
see above	Miwako SaikNorth America / USA Yokohama City Institute of Public Health	Miwako SaikNorth America / USA Yokohama City Institute of Public Health	SaikNorth America / USA,M., Kawakami,C., Usuku,S., Sasao,T. and Okubo,I.
EPI_ISL_2588184	Biology, Gilead Sciences	Biology, Gilead Sciences	Stray,K.
EPI_ISL_2588185, EPI_ISL_2588186, EPI_ISL_2588187, EPI_ISL_2588188, EPI_ISL_2588189, EPI_ISL_2588192	Centre for Infectious Diseases, University of Edinburgh	Centre for Infectious Diseases, University of Edinburgh	Gaunt,E.R., Jansen,R.R., Poovorawan,Y., Toms,G.L., Templeton,K.E. and Simmonds,P.
EPI_ISL_2588193, EPI_ISL_2588194, EPI_ISL_2588195, EPI_ISL_2588196	Centre for Infectious Diseases, University of Edinburgh	Centre for Infectious Diseases, University of Edinburgh	Gaunt,E.R.
EPI_ISL_2588213, EPI_ISL_2588214	Miwako SaikNorth America / USA Yokohama City Institute of Public Health	Miwako SaikNorth America / USA Yokohama City Institute of Public Health	SaikNorth America / USA,M., Kawakami,C., Usuku,S., Sasao,T. and Okubo,I.
EPI_ISL_2588293, EPI_ISL_2588294, EPI_ISL_2588295, EPI_ISL_2588297, EPI_ISL_2588298	Medical Microbiology (Interdisciplinary Program), Graduate School, Chulalongkorn University	Medical Microbiology (Interdisciplinary Program), Graduate School, Chulalongkorn University	Nilwong,O., Bhattarakosol,P. and Kowitdamrong,E.
EPI_ISL_2588346	Botany and Microbiology, College of Science, King Saud University	Botany and Microbiology, College of Science, King Saud University	Farrag,M.A., Amer,H.M. and Almajhdi,F.N.
EPI_ISL_2588439, EPI_ISL_2588440, EPI_ISL_2588441, EPI_ISL_2588442	Centre for Infectious Diseases, University of Edinburgh	Centre for Infectious Diseases, University of Edinburgh	Gaunt,E.R., Jansen,R.R., Poovorawan,Y., Toms,G.L., Templeton,K.E. and Simmonds,P.
EPI_ISL_2588528, EPI_ISL_2588529, EPI_ISL_2588530, EPI_ISL_2588531, EPI_ISL_2588534, EPI_ISL_2588537, EPI_ISL_2588538, EPI_ISL_2588539, EPI_ISL_2588540, EPI_ISL_2588541, EPI_ISL_2588542, EPI_ISL_2588543, EPI_ISL_2588544			
see above	Medical Microbiology (Interdisciplinary Program), Graduate School, Chulalongkorn University	Medical Microbiology (Interdisciplinary Program), Graduate School, Chulalongkorn University	Nilwong,O., Bhattarakosol,P. and Kowitdamrong,E.

EPI_ISL_2588595	Centre for Infectious Diseases, University of Edinburgh	Centre for Infectious Diseases, University of Edinburgh	Gaunt,E.R.
EPI_ISL_2588813	Department of Infectious, Parasitic and Immunomediated Diseases, Istituto Superiore di Sanita'	Department of Infectious, Parasitic and Immunomediated Diseases, Istituto Superiore di Sanita'	Montieri,S., Puzelli,S., Ciccozzi,M., Calzoletti,L. and Di Martino,A.
EPI_ISL_2588816, EPI_ISL_2588817	EPI, Henan Center for Disease Control and Prevention	EPI, Henan Center for Disease Control and Prevention	Feng,D., Xu,J. and Zhao,S.
EPI_ISL_2588819	Virology Laboratory, Dr. Ricardo Gutierrez Children Hospital	Virology Laboratory, Dr. Ricardo Gutierrez Children Hospital	Viegas,M. and Mistchenko,A.S.
EPI_ISL_2588822	Department of Microbiology, Institute of Biomedical Science	Department of Microbiology, Institute of Biomedical Science	Moraes,C.T.P., Botosso,V.F., Sacramento,P., Comone,P., Souza,T.S., Oliveira,D.B.L., Stewien,K.E., Sant'Anna,O.A.B.E. and Durigon,E.L.
EPI_ISL_2588823	Cell Biology, University of Sao Paulo School of Medicine	Cell Biology, University of Sao Paulo School of Medicine	Proenca-Modena,J.L., Paula,F.E., Iwamoto,M.A., Ansarah-Sobrinho,C., Saranzo,A.M., Santos,A.E., Cintra,O.A.L. and Arruda,E.
EPI_ISL_2588824, EPI_ISL_2588825	EPI, Henan Center for Disease Control and Prevention	EPI, Henan Center for Disease Control and Prevention	Feng,D., Xu,J. and Zhao,S.
EPI_ISL_2588829	Department of Microbiology, Institute of Biomedical Science, University of Sao Paulo	Department of Microbiology, Institute of Biomedical Science, University of Sao Paulo	Campos,A.C.A., Botosso,V.F., Souza,T.S., Moraes,C.T.P., Oliveira,D.B.L., Sant'Anna,O.A.B.E., Stewien,K.E. and Durigon,E.L.
EPI_ISL_2588830, EPI_ISL_2588831, EPI_ISL_2588832, EPI_ISL_2588833, EPI_ISL_2588834, EPI_ISL_2588835, EPI_ISL_2588836, EPI_ISL_2588837, EPI_ISL_2588838, EPI_ISL_2588839, EPI_ISL_2588840, EPI_ISL_2588841, EPI_ISL_2588842, EPI_ISL_2588843, EPI_ISL_2588844, EPI_ISL_2588845, EPI_ISL_2588846, EPI_ISL_2588847, EPI_ISL_2588848, EPI_ISL_2588849, EPI_ISL_2588850, EPI_ISL_2588851, EPI_ISL_2588852, EPI_ISL_2588853, EPI_ISL_2588854, EPI_ISL_2588855, EPI_ISL_2588856			
see above	Department of Microbiology, Institute of Biomedical Science, University of Sao Paulo	Department of Microbiology, Institute of Biomedical Science, University of Sao Paulo	Zukurov,J.P.L. and Durigon,E.L.
EPI_ISL_2588857	Virology Laboratory, Dr. Ricardo Gutierrez Children Hospital	Virology Laboratory, Dr. Ricardo Gutierrez Children Hospital	Viegas,M. and Mistchenko,A.S.
EPI_ISL_2588858	Institute of Virology, Zhejiang Provincial Center for Disease Control and Prevention	Institute of Virology, Zhejiang Provincial Center for Disease Control and Prevention	Mao,H.-Y., Lu,Y.-Y. and Yan,J.-Y.
EPI_ISL_2588859	Cell Biology, University of Sao Paulo School of Medicine	Cell Biology, University of Sao Paulo School of Medicine	Proenca-Modena,J.L., Paula,F.E., Iwamoto,M.A., Ansarah-Sobrinho,C., Saranzo,A.M., Santos,A.E., Cintra,O.A.L. and Arruda,E.
EPI_ISL_2588860	Virology Branch, Butantan Institute	Virology Branch, Butantan Institute	Botosso,V.F., Peret,T.C.T., Thomazelli,L.M., Stewien,K.E. and Durigon,E.L.
EPI_ISL_2588861, EPI_ISL_2588862, EPI_ISL_2588863	Institute of Virology, Zhejiang Provincial Center for Disease Control and Prevention	Institute of Virology, Zhejiang Provincial Center for Disease Control and Prevention	Mao,H.-Y., Lu,Y.-Y. and Yan,J.-Y.
EPI_ISL_2588875	Jiangxi Province Center for Disease Control and Prevention	Jiangxi Province Center for Disease Control and Prevention	Xiong,Y., Gong,T., Wang,F.X., Shi,Y. and Zhou,J.
EPI_ISL_2588877, EPI_ISL_2588878, EPI_ISL_2588879, EPI_ISL_2588880	Virology Unit, Institut Pasteur in Cambodia	Virology Unit, Institut Pasteur in Cambodia	Arnott,A., Vong,S., Sek,M., Chu,S., Naughtin,M., Sovann,L., Buecher,C., Beaute,J., Rith,S., Borand,L., Asgari,N., Guillard,B., Touch,S., Deubel,V. and Buchy,P.
EPI_ISL_2588881	Cell Biology, University of Sao Paulo School of Medicine	Cell Biology, University of Sao Paulo School of Medicine	Proenca-Modena,J.L., Paula,F.E., Iwamoto,M.A., Ansarah-Sobrinho,C., Saranzo,A.M., Santos,A.E., Cintra,O.A.L. and Arruda,E.
EPI_ISL_2588882, EPI_ISL_2588883, EPI_ISL_2588884, EPI_ISL_2588885, EPI_ISL_2588886, EPI_ISL_2588887, EPI_ISL_2588888, EPI_ISL_2588889, EPI_ISL_2588890, EPI_ISL_2588891, EPI_ISL_2588892, EPI_ISL_2588893, EPI_ISL_2588894, EPI_ISL_2588895, EPI_ISL_2588896, EPI_ISL_2588897, EPI_ISL_2588898, EPI_ISL_2588899, EPI_ISL_2588900, EPI_ISL_2588901, EPI_ISL_2588902			
see above	Virology Unit, Institut Pasteur in Cambodia	Virology Unit, Institut Pasteur in Cambodia	Arnott,A., Vong,S., Sek,M., Chu,S., Naughtin,M., Sovann,L., Buecher,C., Beaute,J., Rith,S., Borand,L., Asgari,N., Guillard,B., Touch,S., Deubel,V. and Buchy,P.
EPI_ISL_2588903	Virology, Institut Pasteur in Cambodia	Virology, Institut Pasteur in Cambodia	Chu,S., Buecher,C. and Buchy,P.
EPI_ISL_2588904	Division of Viral Infections, National Reference Center for Influenza, Robert Koch Institute	Division of Viral Infections, National Reference Center for Influenza, Robert Koch Institute	Reiche,J., Zadow,I., Lehmann,H., Tietze,S., Hafemann,S., Kluge,P. and Schweiger,B.
EPI_ISL_2588905	Clinical Microbiology, Trinity College Dublin	Clinical Microbiology, Trinity College Dublin	Salter,A.C., Crowley,B. and Ni Laoi,B.
EPI_ISL_2588906, EPI_ISL_2588907, EPI_ISL_2588908, EPI_ISL_2588909, EPI_ISL_2588910, EPI_ISL_2588911, EPI_ISL_2588912, EPI_ISL_2588913, EPI_ISL_2588914, EPI_ISL_2588915, EPI_ISL_2588916, EPI_ISL_2588917, EPI_ISL_2588918, EPI_ISL_2588919, EPI_ISL_2588920, EPI_ISL_2588921, EPI_ISL_2588922, EPI_ISL_2588923, EPI_ISL_2588924, EPI_ISL_2588925, EPI_ISL_2588926, EPI_ISL_2588927, EPI_ISL_2588928, EPI_ISL_2588929, EPI_ISL_2588930, EPI_ISL_2588931, EPI_ISL_2588932, EPI_ISL_2588933, EPI_ISL_2588934, EPI_ISL_2588935, EPI_ISL_2588936, EPI_ISL_2588937, EPI_ISL_2588938, EPI_ISL_2588939, EPI_ISL_2588940, EPI_ISL_2588941, EPI_ISL_2588942, EPI_ISL_2588943, EPI_ISL_2588944, EPI_ISL_2588945, EPI_ISL_2588946, EPI_ISL_2588947, EPI_ISL_2588948, EPI_ISL_2588949, EPI_ISL_2588950, EPI_ISL_2588951, EPI_ISL_2588952, EPI_ISL_2588953, EPI_ISL_2588954, EPI_ISL_2588955			
see above	Virology Unit, Institut Pasteur in Cambodia	Virology Unit, Institut Pasteur in Cambodia	Arnott,A., Vong,S., Sek,M., Chu,S., Naughtin,M., Sovann,L., Buecher,C., Beaute,J., Rith,S., Borand,L., Asgari,N., Guillard,B., Touch,S., Deubel,V. and Buchy,P.
EPI_ISL_2588956, EPI_ISL_2588957	Cell Biology, University of Sao Paulo School of Medicine	Cell Biology, University of Sao Paulo School of Medicine	Proenca-Modena,J.L., Paula,F.E., Iwamoto,M.A., Ansarah-Sobrinho,C., Saranzo,A.M., Santos,A.E., Cintra,O.A.L. and Arruda,E.
EPI_ISL_2588960, EPI_ISL_2588961, EPI_ISL_2588962, EPI_ISL_2588963, EPI_ISL_2588964, EPI_ISL_2588965, EPI_ISL_2588966, EPI_ISL_2588967, EPI_ISL_2588968	Division of Viral Infections, National Reference Center for Influenza, Robert Koch Institute	Division of Viral Infections, National Reference Center for Influenza, Robert Koch Institute	Reiche,J., Zadow,I., Lehmann,H., Tietze,S., Hafemann,S., Kluge,P. and Schweiger,B.
EPI_ISL_2588969, EPI_ISL_2588970, EPI_ISL_2588971, EPI_ISL_2588972, EPI_ISL_2588973	Clinical Microbiology, Trinity College Dublin	Clinical Microbiology, Trinity College Dublin	Salter,A.C., Crowley,B. and Ni Laoi,B.
EPI_ISL_2588974	Virology Branch, Butantan Institute	Virology Branch, Butantan Institute	Botosso,V.F., Peret,T.C.T., Thomazelli,L.M., Stewien,K.E. and Durigon,E.L.
EPI_ISL_2588975	Department of Microbiology, Institute of Biomedical Science	Department of Microbiology, Institute of Biomedical Science	Moraes,C.T.P., Botosso,V.F., Sacramento,P., Comone,P., Souza,T.S., Oliveira,D.B.L., Stewien,K.E., Sant'Anna,O.A.B.E. and Durigon,E.L.
EPI_ISL_2588976	Virology Branch, Butantan Institute	Virology Branch, Butantan Institute	Botosso,V.F., Sacramento,P., Comone,P., Oliveira,D.B.L., Souza,T.S., Moraes,C.T.P., Stewien,K.E., Sant'Anna,O.A.B.E. and Durigon,E.L.
EPI_ISL_2588977, EPI_ISL_2588978, EPI_ISL_2588979, EPI_ISL_2588980, EPI_ISL_2588981, EPI_ISL_2588982, EPI_ISL_2588983, EPI_ISL_2588984, EPI_ISL_2588985, EPI_ISL_2588986, EPI_ISL_2588987, EPI_ISL_2588988, EPI_ISL_2588989, EPI_ISL_2588990			
see above	Division of Viral Infections, National Reference Center for Influenza, Robert Koch Institute	Division of Viral Infections, National Reference Center for Influenza, Robert Koch Institute	Reiche,J., Zadow,I., Lehmann,H., Tietze,S., Hafemann,S., Kluge,P. and Schweiger,B.
EPI_ISL_2588991, EPI_ISL_2588992	Influenza Group, National Institute of Virology	Influenza Group, National Institute of Virology	Damle,R.G. and Chadha,M.S.
EPI_ISL_2589003, EPI_ISL_2589004, EPI_ISL_2589005, EPI_ISL_2589006, EPI_ISL_2589007, EPI_ISL_2589008, EPI_ISL_2589009	Tsuguto Fujimoto National Institute of Infectious Diseases, Infectious Disease Surveillance Center	Tsuguto Fujimoto National Institute of Infectious Diseases, Infectious Disease Surveillance Center	Fujimoto,T., Hanaoka,N., Nakamura,M. and Kawabata,M.
EPI_ISL_2589010, EPI_ISL_2589011, EPI_ISL_2589012	Clinical Microbiology, Trinity College Dublin	Clinical Microbiology, Trinity College Dublin	Salter,A.C., Crowley,B. and Ni Laoi,B.
EPI_ISL_2589014, EPI_ISL_2589015, EPI_ISL_2589016, EPI_ISL_2589017	Virology Branch, Butantan Institute	Virology Branch, Butantan Institute	Botosso,V.F., Peret,T.C.T., Thomazelli,L.M., Stewien,K.E. and Durigon,E.L.
EPI_ISL_2589018	Cell Biology, University of Sao Paulo School of Medicine	Cell Biology, University of Sao Paulo School of Medicine	Proenca-Modena,J.L., Paula,F.E., Iwamoto,M.A., Ansarah-Sobrinho,C., Saranzo,A.M., Santos,A.E., Cintra,O.A.L. and Arruda,E.
EPI_ISL_2589019	Virology Branch, Butantan Institute	Virology Branch, Butantan Institute	Botosso,V.F., Sacramento,P., Comone,P., Oliveira,D.B.L., Souza,T.S., Moraes,C.T.P., Stewien,K.E., Sant'Anna,O.A.B.E. and Durigon,E.L.
EPI_ISL_2589020, EPI_ISL_2589021, EPI_ISL_2589022, EPI_ISL_2589023	Department of Microbiology, Institute of Biomedical Science	Department of Microbiology, Institute of Biomedical Science	Moraes,C.T.P., Botosso,V.F., Sacramento,P., Comone,P., Souza,T.S., Oliveira,D.B.L., Stewien,K.E., Sant'Anna,O.A.B.E. and Durigon,E.L.
EPI_ISL_2589024	Virology Branch, Butantan Institute	Virology Branch, Butantan Institute	Botosso,V.F., Sacramento,P., Comone,P., Oliveira,D.B.L., Souza,T.S., Moraes,C.T.P., Stewien,K.E., Sant'Anna,O.A.B.E. and Durigon,E.L.
EPI_ISL_2589025	Department of Microbiology, Institute of Biomedical Science	Department of Microbiology, Institute of Biomedical Science	Moraes,C.T.P., Botosso,V.F., Sacramento,P., Comone,P., Souza,T.S., Oliveira,D.B.L., Stewien,K.E., Sant'Anna,O.A.B.E. and Durigon,E.L.
EPI_ISL_2589026, EPI_ISL_2589027	Department of Microbiology, Institute of Biomedical Science, University of Sao Paulo	Department of Microbiology, Institute of Biomedical Science, University of Sao Paulo	Campos,A.C.A., Botosso,V.F., Souza,T.S., Moraes,C.T.P., Oliveira,D.B.L., Sant'Anna,O.A.B.E., Stewien,K.E. and Durigon,E.L.
EPI_ISL_2589028	Department of Microbiology, Institute of Biomedical Science	Department of Microbiology, Institute of Biomedical Science	Moraes,C.T.P., Botosso,V.F., Sacramento,P., Comone,P., Souza,T.S., Oliveira,D.B.L., Stewien,K.E., Sant'Anna,O.A.B.E. and Durigon,E.L.
EPI_ISL_2589029, EPI_ISL_2589030, EPI_ISL_2589031, EPI_ISL_2589032, EPI_ISL_2589033, EPI_ISL_2589034	Virology Branch, Butantan Institute	Virology Branch, Butantan Institute	Botosso,V.F., Sacramento,P., Comone,P., Oliveira,D.B.L., Souza,T.S., Moraes,C.T.P., Stewien,K.E., Sant'Anna,O.A.B.E. and Durigon,E.L.
EPI_ISL_2589035, EPI_ISL_2589036	Department of Microbiology, Institute of Biomedical Science	Department of Microbiology, Institute of Biomedical Science	Moraes,C.T.P., Botosso,V.F., Sacramento,P., Comone,P., Souza,T.S., Oliveira,D.B.L., Stewien,K.E., Sant'Anna,O.A.B.E. and Durigon,E.L.
EPI_ISL_2589037, EPI_ISL_2589038, EPI_ISL_2589039, EPI_ISL_2589040, EPI_ISL_2589041, EPI_ISL_2589042, EPI_ISL_2589043, EPI_ISL_2589044, EPI_ISL_2589045, EPI_ISL_2589046, EPI_ISL_2589047, EPI_ISL_2589048			
see above	Cell Biology, University of Sao Paulo School of Medicine	Cell Biology, University of Sao Paulo School of Medicine	Proenca-Modena,J.L., Paula,F.E., Iwamoto,M.A., Ansarah-Sobrinho,C., Saranzo,A.M., Santos,A.E., Cintra,O.A.L. and Arruda,E.
EPI_ISL_2589049	Centre de Recherche en Infectiologie, Centre Hospitalier Universitaire de Quebec (Pavillon CHUL)	Centre de Recherche en Infectiologie, Centre Hospitalier Universitaire de Quebec (Pavillon CHUL)	Gilca,R., De Serres,G., Tremblay,M., Leblanc,E., Bergeron,M.G., Dery,P. and Boivin,G.
EPI_ISL_2589056, EPI_ISL_2589057, EPI_ISL_2589058, EPI_ISL_2589059, EPI_ISL_2589060, EPI_ISL_2589061, EPI_ISL_2589062, EPI_ISL_2589063, EPI_ISL_2589064, EPI_ISL_2589065, EPI_ISL_2589066, EPI_ISL_2589067, EPI_ISL_2589068, EPI_ISL_2589069			
see above	Division of Viral Infections, National Reference Center for Influenza, Robert Koch Institute	Division of Viral Infections, National Reference Center for Influenza, Robert Koch Institute	Reiche,J., Zadow,I., Lehmann,H., Tietze,S., Hafemann,S., Kluge,P. and Schweiger,B.
EPI_ISL_2589071	Virology, School of Public Health and Institute of Public Health Research, Tehran University of Medical Sciences	Virology, School of Public Health and Institute of Public Health Research, Tehran University of Medical Sciences	Mokhtari-Azad,T., Faghihloo,E., Salimi,V. and Rezaei,F.
EPI_ISL_2589077, EPI_ISL_2589078, EPI_ISL_2589079, EPI_ISL_2589080, EPI_ISL_2589081, EPI_ISL_2589082	Virology, Institut Pasteur in Cambodia	Virology, Institut Pasteur in Cambodia	Chu,S., Buecher,C. and Buchy,P.
EPI_ISL_2589083, EPI_ISL_2589084, EPI_ISL_2589085, EPI_ISL_2589086, EPI_ISL_2589087, EPI_ISL_2589088, EPI_ISL_2589089, EPI_ISL_2589090, EPI_ISL_2589091, EPI_ISL_2589092, EPI_ISL_2589093, EPI_ISL_2589094, EPI_ISL_2589095, EPI_ISL_2589096, EPI_ISL_2589097, EPI_ISL_2589098, EPI_ISL_2589099, EPI_ISL_2589100			
see above	Virology Branch, Butantan Institute	Virology Branch, Butantan Institute	Botosso,V.F., Peret,T.C.T., Thomazelli,L.M., Stewien,K.E. and Durigon,E.L.
EPI_ISL_2589101	Virology, Institut Pasteur in Cambodia	Virology, Institut Pasteur in Cambodia	Chu,S., Buecher,C. and Buchy,P.
EPI_ISL_2589102	Virology Branch, Butantan Institute	Virology Branch, Butantan Institute	Botosso,V.F., Sacramento,P., Comone,P., Oliveira,D.B.L., Souza,T.S., Moraes,C.T.P., Stewien,K.E., Sant'Anna,O.A.B.E. and Durigon,E.L.
EPI_ISL_2589103, EPI_ISL_2589104	Department of Microbiology, Institute of Biomedical Science, University of Sao Paulo	Department of Microbiology, Institute of Biomedical Science, University of Sao Paulo	Campos,A.C.A., Botosso,V.F., Souza,T.S., Moraes,C.T.P., Oliveira,D.B.L., Sant'Anna,O.A.B.E., Stewien,K.E. and Durigon,E.L.
EPI_ISL_2589105, EPI_ISL_2589106	Virology Branch, Butantan Institute	Virology Branch, Butantan Institute	Botosso,V.F., Sacramento,P., Comone,P., Oliveira,D.B.L., Souza,T.S., Moraes,C.T.P., Stewien,K.E., Sant'Anna,O.A.B.E. and Durigon,E.L.
EPI_ISL_2589107	Department of Microbiology, Institute of Biomedical Science, University of Sao Paulo	Department of Microbiology, Institute of Biomedical Science, University of Sao Paulo	Campos,A.C.A., Botosso,V.F., Souza,T.S., Moraes,C.T.P., Oliveira,D.B.L., Sant'Anna,O.A.B.E., Stewien,K.E. and Durigon,E.L.
EPI_ISL_2589108	Virology Branch, Butantan Institute	Virology Branch, Butantan Institute	Botosso,V.F., Sacramento,P., Comone,P., Oliveira,D.B.L., Souza,T.S., Moraes,C.T.P., Stewien,K.E., Sant'Anna,O.A.B.E. and Durigon,E.L.
EPI_ISL_2589109	Department of Microbiology, University of Sao Paulo Institute of Biomedical Science	Department of Microbiology, University of Sao Paulo Institute of Biomedical Science	Lima,H.N., Oliveira,D.B.L., Botosso,V.F., Stewien,K.E. and Durigon,E.L.
EPI_ISL_2589110	Virology Branch, Butantan Institute	Virology Branch, Butantan Institute	Botosso,V.F., Sacramento,P., Comone,P., Oliveira,D.B.L., Souza,T.S., Moraes,C.T.P., Stewien,K.E., Sant'Anna,O.A.B.E. and Durigon,E.L.
EPI_ISL_2589111	Department of Microbiology, University of Sao Paulo Institute of Biomedical Science	Department of Microbiology, University of Sao Paulo Institute of Biomedical Science	Lima,H.N., Oliveira,D.B.L., Botosso,V.F., Stewien,K.E. and Durigon,E.L.

EPI_ISL_2589112, EPI_ISL_2589113	Virology Branch, Butantan Institute	Virology Branch, Butantan Institute	Botosso,V.F., Peret,T.C.T., Thomazelli,L.M., Stewien,K.E. and Durigon,E.L.
EPI_ISL_2589114	Virology Branch, Butantan Institute	Virology Branch, Butantan Institute	Botosso,V.F., Sacramento,P., Comone,P., Oliveira,D.B.L., Souza,T.S., Moraes,C.T.P., Stewien,K.E., Sant'Anna,O.A.B.E. and Durigon,E.L.
EPI_ISL_2589115	Department of Microbiology, University of Sao Paulo Institute of Biomedical Science	Department of Microbiology, University of Sao Paulo Institute of Biomedical Science	Lima,H.N., Oliveira,D.B.L., Botosso,V.F., Stewien,K.E. and Durigon,E.L.
EPI_ISL_2589116	Department of Microbiology, Institute of Biomedical Science, University of Sao Paulo	Department of Microbiology, Institute of Biomedical Science, University of Sao Paulo	Campos,A.C.A., Botosso,V.F., Souza,T.S., Moraes,C.T.P., Oliveira,D.B.L., Sant'Anna,O.A.B.E., Stewien,K.E. and Durigon,E.L.
EPI_ISL_2589117	Virology Branch, Butantan Institute	Virology Branch, Butantan Institute	Botosso,V.F., Peret,T.C.T., Thomazelli,L.M., Stewien,K.E. and Durigon,E.L.
EPI_ISL_2589118, EPI_ISL_2589119, EPI_ISL_2589120	Department of Microbiology, Institute of Biomedical Science	Department of Microbiology, Institute of Biomedical Science	Moraes,C.T.P., Botosso,V.F., Sacramento,P., Comone,P., Souza,T.S., Oliveira,D.B.L., Stewien,K.E., Sant'Anna,O.A.B.E. and Durigon,E.L.
EPI_ISL_2589121	Department of Microbiology, University of Sao Paulo Institute of Biomedical Science	Department of Microbiology, University of Sao Paulo Institute of Biomedical Science	Lima,H.N., Oliveira,D.B.L., Botosso,V.F., Stewien,K.E. and Durigon,E.L.
EPI_ISL_2589122	Department of Microbiology, Institute of Biomedical Science	Department of Microbiology, Institute of Biomedical Science	Moraes,C.T.P., Botosso,V.F., Sacramento,P., Comone,P., Souza,T.S., Oliveira,D.B.L., Stewien,K.E., Sant'Anna,O.A.B.E. and Durigon,E.L.
EPI_ISL_2589123	Virology Branch, Butantan Institute	Virology Branch, Butantan Institute	Botosso,V.F., Sacramento,P., Comone,P., Oliveira,D.B.L., Souza,T.S., Moraes,C.T.P., Stewien,K.E., Sant'Anna,O.A.B.E. and Durigon,E.L.
EPI_ISL_2589124	Virology Branch, Butantan Institute	Virology Branch, Butantan Institute	Botosso,V.F., Peret,T.C.T., Thomazelli,L.M., Stewien,K.E. and Durigon,E.L.
EPI_ISL_2589125	Department of Microbiology, Institute of Biomedical Science, University of Sao Paulo	Department of Microbiology, Institute of Biomedical Science, University of Sao Paulo	Campos,A.C.A., Botosso,V.F., Souza,T.S., Moraes,C.T.P., Oliveira,D.B.L., Sant'Anna,O.A.B.E., Stewien,K.E. and Durigon,E.L.
EPI_ISL_2589126	Department of Microbiology, University of Sao Paulo Institute of Biomedical Science	Department of Microbiology, University of Sao Paulo Institute of Biomedical Science	Lima,H.N., Oliveira,D.B.L., Botosso,V.F., Stewien,K.E. and Durigon,E.L.
EPI_ISL_2589127, EPI_ISL_2589128	Virology Branch, Butantan Institute	Virology Branch, Butantan Institute	Botosso,V.F., Peret,T.C.T., Thomazelli,L.M., Stewien,K.E. and Durigon,E.L.
EPI_ISL_2589129	Virology Branch, Butantan Institute	Virology Branch, Butantan Institute	Botosso,V.F., Sacramento,P., Comone,P., Oliveira,D.B.L., Souza,T.S., Moraes,C.T.P., Stewien,K.E., Sant'Anna,O.A.B.E. and Durigon,E.L.
EPI_ISL_2589130, EPI_ISL_2589131	Department of Microbiology, Institute of Biomedical Science, University of Sao Paulo	Department of Microbiology, Institute of Biomedical Science, University of Sao Paulo	Campos,A.C.A., Botosso,V.F., Souza,T.S., Moraes,C.T.P., Oliveira,D.B.L., Sant'Anna,O.A.B.E., Stewien,K.E. and Durigon,E.L.
EPI_ISL_2589132	Department of Microbiology, University of Sao Paulo Institute of Biomedical Science	Department of Microbiology, University of Sao Paulo Institute of Biomedical Science	Lima,H.N., Oliveira,D.B.L., Botosso,V.F., Stewien,K.E. and Durigon,E.L.
EPI_ISL_2589133	Department of Microbiology, Institute of Biomedical Science, University of Sao Paulo	Department of Microbiology, Institute of Biomedical Science, University of Sao Paulo	Oliveira,D.B.L., Carvalho,A.C.L., Botosso,V.F., Sant' Anna,O.A.B.E., Stewien,K.E. and Durigon,E.L.
EPI_ISL_2589134	Department of Microbiology, Institute of Biomedical Science	Department of Microbiology, Institute of Biomedical Science	Moraes,C.T.P., Botosso,V.F., Sacramento,P., Comone,P., Souza,T.S., Oliveira,D.B.L., Stewien,K.E., Sant'Anna,O.A.B.E. and Durigon,E.L.
EPI_ISL_2589135	Department of Microbiology, University of Sao Paulo Institute of Biomedical Science	Department of Microbiology, University of Sao Paulo Institute of Biomedical Science	Lima,H.N., Oliveira,D.B.L., Botosso,V.F., Stewien,K.E. and Durigon,E.L.
EPI_ISL_2589136	Department of Microbiology, Institute of Biomedical Science, University of Sao Paulo	Department of Microbiology, Institute of Biomedical Science, University of Sao Paulo	Oliveira,D.B.L., Carvalho,A.C.L., Botosso,V.F., Sant' Anna,O.A.B.E., Stewien,K.E. and Durigon,E.L.
EPI_ISL_2589137	Department of Microbiology, Institute of Biomedical Science, University of Sao Paulo	Department of Microbiology, Institute of Biomedical Science, University of Sao Paulo	Campos,A.C.A., Botosso,V.F., Souza,T.S., Moraes,C.T.P., Oliveira,D.B.L., Sant'Anna,O.A.B.E., Stewien,K.E. and Durigon,E.L.
EPI_ISL_2589138, EPI_ISL_2589139, EPI_ISL_2589140	Virology Branch, Butantan Institute	Virology Branch, Butantan Institute	Botosso,V.F., Peret,T.C.T., Thomazelli,L.M., Stewien,K.E. and Durigon,E.L.
EPI_ISL_2589141	Department of Microbiology, Institute of Biomedical Science	Department of Microbiology, Institute of Biomedical Science	Moraes,C.T.P., Botosso,V.F., Sacramento,P., Comone,P., Souza,T.S., Oliveira,D.B.L., Stewien,K.E., Sant'Anna,O.A.B.E. and Durigon,E.L.
EPI_ISL_2589142, EPI_ISL_2589143	Virology Branch, Butantan Institute	Virology Branch, Butantan Institute	Botosso,V.F., Sacramento,P., Comone,P., Oliveira,D.B.L., Souza,T.S., Moraes,C.T.P., Stewien,K.E., Sant'Anna,O.A.B.E. and Durigon,E.L.
EPI_ISL_2589144	Department of Microbiology, Institute of Biomedical Science	Department of Microbiology, Institute of Biomedical Science	Moraes,C.T.P., Botosso,V.F., Sacramento,P., Comone,P., Souza,T.S., Oliveira,D.B.L., Stewien,K.E., Sant'Anna,O.A.B.E. and Durigon,E.L.
EPI_ISL_2589145, EPI_ISL_2589146, EPI_ISL_2589147, EPI_ISL_2589148	Virology Branch, Butantan Institute	Virology Branch, Butantan Institute	Botosso,V.F., Sacramento,P., Comone,P., Oliveira,D.B.L., Souza,T.S., Moraes,C.T.P., Stewien,K.E., Sant'Anna,O.A.B.E. and Durigon,E.L.
EPI_ISL_2589149	Virology Branch, Butantan Institute	Virology Branch, Butantan Institute	Botosso,V.F., Peret,T.C.T., Thomazelli,L.M., Stewien,K.E. and Durigon,E.L.
EPI_ISL_2589150, EPI_ISL_2589151	Department of Microbiology, Institute of Biomedical Science	Department of Microbiology, Institute of Biomedical Science	Moraes,C.T.P., Botosso,V.F., Sacramento,P., Comone,P., Souza,T.S., Oliveira,D.B.L., Stewien,K.E., Sant'Anna,O.A.B.E. and Durigon,E.L.
EPI_ISL_2589152	Virology Branch, Butantan Institute	Virology Branch, Butantan Institute	Botosso,V.F., Peret,T.C.T., Thomazelli,L.M., Stewien,K.E. and Durigon,E.L.
EPI_ISL_2589153	Virology Branch, Butantan Institute	Virology Branch, Butantan Institute	Botosso,V.F., Sacramento,P., Comone,P., Oliveira,D.B.L., Souza,T.S., Moraes,C.T.P., Stewien,K.E., Sant'Anna,O.A.B.E. and Durigon,E.L.
EPI_ISL_2589154, EPI_ISL_2589155	Microbiology, Adolfo Lutz Institut	Microbiology, Adolfo Lutz Institut	Souza,M.C.O., Matsumoto,T.K., Vaz-De-Lima,L.L.R.A., Sato,N.N., Salgado,M.M., Requejo,H.I.Z., Hong,M.A., Barbosa,M.L., Oliveira,C.A.F., Benelli,P.C., Shimokawa,R.Y., Pecchini,R., Berezin,E., Duarte,S.P., Schvartsman,C., Durigon,E.L. and Ueda,M.
EPI_ISL_2589156, EPI_ISL_2589157, EPI_ISL_2589158, EPI_ISL_2589159	Cell Biology, University of Sao Paulo School of Medicine	Cell Biology, University of Sao Paulo School of Medicine	Proenca-Modena,J.L., Paula,F.E., Iwamoto,M.A., Ansarah-Sobrinho,C., Saranzo,A.M., Santos,A.E., Cintra,O.A.L. and Arruda,E.
EPI_ISL_2589160	Microbiology, Adolfo Lutz Institut	Microbiology, Adolfo Lutz Institut	Souza,M.C.O., Matsumoto,T.K., Vaz-De-Lima,L.L.R.A., Sato,N.N., Salgado,M.M., Requejo,H.I.Z., Hong,M.A., Barbosa,M.L., Oliveira,C.A.F., Benelli,P.C., Shimokawa,R.Y., Pecchini,R., Berezin,E., Duarte,S.P., Schvartsman,C., Durigon,E.L. and Ueda,M.
EPI_ISL_2589161	Microbiology, Adolfo Lutz Institut	Microbiology, Adolfo Lutz Institut	Souza,M.C.O., Matsumoto,T.K., Vaz-de-Lima,L.L.R., Sato,N.N., Salgado,M.M., Requejo,H.I.Z., Hong,M.A., Barbosa,M.L., Oliveira,C.A.F., Benelli,P.C., Shimokawa,R.Y., Pecchini,R., Berezin,E., Duarte,S.P., Schvartsman,C., Durigon,E.L. and Ueda,M.
EPI_ISL_2589170, EPI_ISL_2589171, EPI_ISL_2589172, EPI_ISL_2589173, EPI_ISL_2589174, EPI_ISL_2589175, EPI_ISL_2589176, EPI_ISL_2589177, EPI_ISL_2589178	Division of Viral Infections, National Reference Center for Influenza, Robert Koch Institute	Division of Viral Infections, National Reference Center for Influenza, Robert Koch Institute	Reiche,J., Zadow,I., Lehmann,H., Tietze,S., Hafemann,S., Kluge,P. and Schweiger,B.
EPI_ISL_2589179	Influenza Group, National Institute of Virology	Influenza Group, National Institute of Virology	Damle,R.G. and Chadha,M.S.
EPI_ISL_2589180, EPI_ISL_2589181, EPI_ISL_2589182, EPI_ISL_2589183, EPI_ISL_2589184, EPI_ISL_2589185, EPI_ISL_2589186, EPI_ISL_2589187, EPI_ISL_2589188, EPI_ISL_2589189, EPI_ISL_2589190, EPI_ISL_2589191, EPI_ISL_2589192, EPI_ISL_2589193, EPI_ISL_2589194			
see above	Virology, Institut Pasteur in Cambodia	Virology, Institut Pasteur in Cambodia	Chu,S., Buecher,C. and Buchy,P.
EPI_ISL_2589195, EPI_ISL_2589196, EPI_ISL_2589197, EPI_ISL_2589198, EPI_ISL_2589199, EPI_ISL_2589200, EPI_ISL_2589201, EPI_ISL_2589202, EPI_ISL_2589203, EPI_ISL_2589204, EPI_ISL_2589205			
see above	Virology Branch, Butantan Institute	Virology Branch, Butantan Institute	Botosso,V.F., Peret,T.C.T., Thomazelli,L.M., Stewien,K.E. and Durigon,E.L.
EPI_ISL_2589206	Virology Branch, Butantan Institute	Virology Branch, Butantan Institute	Botosso,V.F., Sacramento,P., Comone,P., Oliveira,D.B.L., Souza,T.S., Moraes,C.T.P., Stewien,K.E., Sant'Anna,O.A.B.E. and Durigon,E.L.
EPI_ISL_2589207, EPI_ISL_2589208	Virology Branch, Butantan Institute	Virology Branch, Butantan Institute	Botosso,V.F., Peret,T.C.T., Thomazelli,L.M., Stewien,K.E. and Durigon,E.L.
EPI_ISL_2589209	Virology Branch, Butantan Institute	Virology Branch, Butantan Institute	Botosso,V.F., Sacramento,P., Comone,P., Oliveira,D.B.L., Souza,T.S., Moraes,C.T.P., Stewien,K.E., Sant'Anna,O.A.B.E. and Durigon,E.L.
EPI_ISL_2589210, EPI_ISL_2589211, EPI_ISL_2589212	Virology Branch, Butantan Institute	Virology Branch, Butantan Institute	Botosso,V.F., Peret,T.C.T., Thomazelli,L.M., Stewien,K.E. and Durigon,E.L.
EPI_ISL_2589214, EPI_ISL_2589215, EPI_ISL_2589216, EPI_ISL_2589217	Virology, Institut Pasteur in Cambodia	Virology, Institut Pasteur in Cambodia	Chu,S., Buecher,C. and Buchy,P.
EPI_ISL_2589218, EPI_ISL_2589219, EPI_ISL_2589220, EPI_ISL_2589221, EPI_ISL_2589222, EPI_ISL_2589223	Virology Branch, Butantan Institute	Virology Branch, Butantan Institute	Botosso,V.F., Peret,T.C.T., Thomazelli,L.M., Stewien,K.E. and Durigon,E.L.
EPI_ISL_2589224	Cell Biology, University of Sao Paulo School of Medicine	Cell Biology, University of Sao Paulo School of Medicine	Proenca-Modena,J.L., Paula,F.E., Iwamoto,M.A., Ansarah-Sobrinho,C., Saranzo,A.M., Santos,A.E., Cintra,O.A.L. and Arruda,E.
EPI_ISL_2589225, EPI_ISL_2589226, EPI_ISL_2589227, EPI_ISL_2589228	Microbiology, Adolfo Lutz Institut	Microbiology, Adolfo Lutz Institut	Souza,M.C.O., Matsumoto,T.K., Vaz-De-Lima,L.L.R.A., Sato,N.N., Salgado,M.M., Requejo,H.I.Z., Hong,M.A., Barbosa,M.L., Oliveira,C.A.F., Benelli,P.C., Shimokawa,R.Y., Pecchini,R., Berezin,E., Duarte,S.P., Schvartsman,C., Durigon,E.L. and Ueda,M.
EPI_ISL_2589229	Centre de Recherche en Infectiologie, Centre Hospitalier Universitaire de Quebec (Pavillon CHUL)	Centre de Recherche en Infectiologie, Centre Hospitalier Universitaire de Quebec (Pavillon CHUL)	Gilca,R., De Serres,G., Tremblay,M., Leblanc,E., Bergeron,M.G., Dery,P. and Boivin,G.
EPI_ISL_2589230	Chinese Academy of Medical Sciences & Peking Union Medical College	Chinese Academy of Medical Sciences & Peking Union Medical College	Jia,B., Xiao,Y., Wang,Y., Chen,L., Zhang,J., Ren,L. and Wang,J.
EPI_ISL_2589233, EPI_ISL_2589234, EPI_ISL_2589235, EPI_ISL_2589236, EPI_ISL_2589237, EPI_ISL_2589238, EPI_ISL_2589239	State Key Laboratory of Molecular Virology and Genetic Engineering, National Institute for Viral Disease Control and Prevention CDC	State Key Laboratory of Molecular Virology and Genetic Engineering, National Institute for Viral Disease Control and Prevention CDC	Zhang,R.F. and Duan,Z.J.
EPI_ISL_2589242	Research & Development, Institute of Immunology	Research & Development, Institute of Immunology	Ivancic-Jelecki,J. and Forcic,D.
EPI_ISL_2589243, EPI_ISL_2589244, EPI_ISL_2589245, EPI_ISL_2589246, EPI_ISL_2589247	Division of Viral Infections, National Reference Center for Influenza, Robert Koch Institute	Division of Viral Infections, National Reference Center for Influenza, Robert Koch Institute	Reiche,J., Zadow,I., Lehmann,H., Tietze,S., Hafemann,S., Kluge,P. and Schweiger,B.
EPI_ISL_2589248, EPI_ISL_2589249, EPI_ISL_2589250, EPI_ISL_2589251	Influenza Group, National Institute of Virology	Influenza Group, National Institute of Virology	Damle,R.G. and Chadha,M.S.
EPI_ISL_2589254, EPI_ISL_2589255	Virology, School of Public Health and Institute of Public Health Research, Tehran University of Medical Sciences	Virology, School of Public Health and Institute of Public Health Research, Tehran University of Medical Sciences	Mokhtari-Azad,T., Faghihloo,E., Salimi,V. and Rezaei,F.
EPI_ISL_2589262, EPI_ISL_2589263, EPI_ISL_2589264, EPI_ISL_2589265, EPI_ISL_2589266, EPI_ISL_2589267, EPI_ISL_2589268, EPI_ISL_2589269, EPI_ISL_2589270, EPI_ISL_2589271, EPI_ISL_2589272, EPI_ISL_2589273, EPI_ISL_2589274, EPI_ISL_2589275, EPI_ISL_2589276, EPI_ISL_2589277			
see above	Virology Branch, Butantan Institute	Virology Branch, Butantan Institute	Botosso,V.F., Peret,T.C.T., Thomazelli,L.M., Stewien,K.E. and Durigon,E.L.
EPI_ISL_2589278	Microbiology, Adolfo Lutz Institut	Microbiology, Adolfo Lutz Institut	Souza,M.C.O., Matsumoto,T.K., Vaz-De-Lima,L.L.R.A., Sato,N.N., Salgado,M.M., Requejo,H.I.Z., Hong,M.A., Barbosa,M.L., Oliveira,C.A.F., Benelli,P.C., Shimokawa,R.Y., Pecchini,R., Berezin,E., Duarte,S.P., Schvartsman,C., Durigon,E.L. and Ueda,M.
EPI_ISL_2589279, EPI_ISL_2589280, EPI_ISL_2589281, EPI_ISL_2589282, EPI_ISL_2589283	Cell Biology, University of Sao Paulo School of Medicine	Cell Biology, University of Sao Paulo School of Medicine	Proenca-Modena,J.L., Paula,F.E., Iwamoto,M.A., Ansarah-Sobrinho,C., Saranzo,A.M., Santos,A.E., Cintra,O.A.L. and Arruda,E.
EPI_ISL_2589284, EPI_ISL_2589285, EPI_ISL_2589286, EPI_ISL_2589287	Microbiology, Adolfo Lutz Institut	Microbiology, Adolfo Lutz Institut	Souza,M.C.O., Matsumoto,T.K., Vaz-De-Lima,L.L.R.A., Sato,N.N., Salgado,M.M., Requejo,H.I.Z., Hong,M.A., Barbosa,M.L., Oliveira,C.A.F., Benelli,P.C., Shimokawa,R.Y., Pecchini,R., Berezin,E., Duarte,S.P., Schvartsman,C., Durigon,E.L. and Ueda,M.
EPI_ISL_2589294, EPI_ISL_2589295, EPI_ISL_2589296, EPI_ISL_2589297, EPI_ISL_2589298, EPI_ISL_2589299	State Key Laboratory of Molecular Virology and Genetic Engineering, National Institute for Viral Disease Control and Prevention CDC	State Key Laboratory of Molecular Virology and Genetic Engineering, National Institute for Viral Disease Control and Prevention CDC	Zhang,R.F. and Duan,Z.J.
EPI_ISL_2589305, EPI_ISL_2589306, EPI_ISL_2589307, EPI_ISL_2589308, EPI_ISL_2589310, EPI_ISL_2589313	Research & Development, Institute of Immunology	Research & Development, Institute of Immunology	Ivancic-Jelecki,J. and Forcic,D.
EPI_ISL_2589316, EPI_ISL_2589317, EPI_ISL_2589318, EPI_ISL_2589319, EPI_ISL_2589320, EPI_ISL_2589321, EPI_ISL_2589322, EPI_ISL_2589323, EPI_ISL_2589324, EPI_ISL_2589325	Division of Viral Infections, National Reference Center for Influenza, Robert Koch Institute	Division of Viral Infections, National Reference Center for Influenza, Robert Koch Institute	Reiche,J., Zadow,I., Lehmann,H., Tietze,S., Hafemann,S., Kluge,P. and Schweiger,B.
EPI_ISL_2589332	Department of Microbiology and Immunolgy, Institute of Biology, State University of	Department of Microbiology and Immunolgy, Institute of Biology, State University of	da Silva,L.H.A., Spilki,F.R., Riccetto,A.G.L., de Almeida,R.S., Baracat,E.C.E. and Arns,C.W.

	Campinas (UNICAMP)	Campinas (UNICAMP)	
EPI_ISL_2589350	Division of Viral Infections, National Reference Center for Influenza, Robert Koch Institute	Division of Viral Infections, National Reference Center for Influenza, Robert Koch Institute	Reiche,J., Zadow,I., Lehmann,H., Tietze,S., Hafemann,S., Kluge,P. and Schweiger,B.
EPI_ISL_2589395, EPI_ISL_2589396	Department of Microbiology and Immunolgy, Institute of Biology, State University of Campinas (UNICAMP)	Department of Microbiology and Immunolgy, Institute of Biology, State University of Campinas (UNICAMP)	da Silva,L.H.A., Spilki,F.R., Riccetto,A.G.L., de Almeida,R.S., Baracat,E.C.E. and Arns,C.W.
EPI_ISL_2589397, EPI_ISL_2589398, EPI_ISL_2589399, EPI_ISL_2589412, EPI_ISL_2589413, EPI_ISL_2589414, EPI_ISL_2589423	Virology, Facultad de Ciencias	Virology, Facultad de Ciencias	Moura,F., Blanc,A. and Frabasile,S.
EPI_ISL_2589462, EPI_ISL_2589465	Virology Laboratory, Dr. Ricardo Gutierrez Children Hospital	Virology Laboratory, Dr. Ricardo Gutierrez Children Hospital	Viegas,M. and Mistchenko,A.S.
EPI_ISL_2589502	Department of Infectious, Parasitic and Immunomediated Diseases, Istituto Superiore di Sanita'	Department of Infectious, Parasitic and Immunomediated Diseases, Istituto Superiore di Sanita'	Montieri,S., Puzelli,S., Ciccozzi,M., Calzoletti,L. and Di Martino,A.
EPI_ISL_2589503	Immunology and Microbiology, Rega Institute for Medical Research	Immunology and Microbiology, Rega Institute for Medical Research	Houspie,L., Lemey,P., Keyaerts,E., Reijmen,E., Vergote,V., Vankeerberghen,A., Vaeyens,F., De Beenhouwer,H. and Van Ranst,M.
EPI_ISL_2589519	Virology Laboratory, Dr. Ricardo Gutierrez Children Hospital	Virology Laboratory, Dr. Ricardo Gutierrez Children Hospital	Viegas,M., Goya,S. and Mistchenko,A.S.
EPI_ISL_2589520	Virology Laboratory, Dr. Ricardo Gutierrez Children Hospital	Virology Laboratory, Dr. Ricardo Gutierrez Children Hospital	Viegas,M. and Mistchenko,A.S.
EPI_ISL_2589521	Virology Laboratory, Dr. Ricardo Gutierrez Children Hospital	Virology Laboratory, Dr. Ricardo Gutierrez Children Hospital	Viegas,M., Goya,S. and Mistchenko,A.S.
EPI_ISL_2589536, EPI_ISL_2589537, EPI_ISL_2589538, EPI_ISL_2589539	Virology Laboratory, Dr. Ricardo Gutierrez Children Hospital	Virology Laboratory, Dr. Ricardo Gutierrez Children Hospital	Viegas,M. and Mistchenko,A.S.
EPI_ISL_2589540, EPI_ISL_2589541, EPI_ISL_2589542, EPI_ISL_2589543, EPI_ISL_2589544	Virology Laboratory, Dr. Ricardo Gutierrez Children Hospital	Virology Laboratory, Dr. Ricardo Gutierrez Children Hospital	Viegas,M., Goya,S. and Mistchenko,A.S.
EPI_ISL_2589545	Department of Microbiology & Immunology, Rega Institute for Medical Research, University of Leuven, Laboratory of Clinical and Epidemiological Virology	Department of Microbiology & Immunology, Rega Institute for Medical Research, University of Leuven, Laboratory of Clinical and Epidemiological Virology	Zlateva,K.T., Lemey,P., Vandamme,A.-M. and Van Ranst,M.
EPI_ISL_2589546, EPI_ISL_2589547, EPI_ISL_2589548, EPI_ISL_2589549, EPI_ISL_2589550, EPI_ISL_2589551	Virology Laboratory, Dr. Ricardo Gutierrez Children Hospital	Virology Laboratory, Dr. Ricardo Gutierrez Children Hospital	Viegas,M. and Mistchenko,A.S.
EPI_ISL_2589552	Institute for Virology, University Duesseldorf	Institute for Virology, University Duesseldorf	Adams,O., Werczmircorwski,J. and Hengel,H.
EPI_ISL_2589553, EPI_ISL_2589554, EPI_ISL_2589555, EPI_ISL_2589556	Virology Laboratory, Dr. Ricardo Gutierrez Children Hospital	Virology Laboratory, Dr. Ricardo Gutierrez Children Hospital	Viegas,M. and Mistchenko,A.S.
EPI_ISL_2589557, EPI_ISL_2589558	Department of Microbiology & Immunology, Rega Institute for Medical Research, University of Leuven, Laboratory of Clinical and Epidemiological Virology	Department of Microbiology & Immunology, Rega Institute for Medical Research, University of Leuven, Laboratory of Clinical and Epidemiological Virology	Zlateva,K.T., Lemey,P., Vandamme,A.-M. and Van Ranst,M.
EPI_ISL_2589559	Department of Microbiology and Immunology, Laboratory of Clinical and Epidemiological Virology, Rega Institute for Medical Research	Department of Microbiology and Immunology, Laboratory of Clinical and Epidemiological Virology, Rega Institute for Medical Research	Zlateva,K.T., Vijgen,L., Dekeersmaeker,N., Naranjo,C. and Van Ranst,M.
EPI_ISL_2589560, EPI_ISL_2589561, EPI_ISL_2589562	Department of Microbiology & Immunology, Rega Institute for Medical Research, University of Leuven, Laboratory of Clinical and Epidemiological Virology	Department of Microbiology & Immunology, Rega Institute for Medical Research, University of Leuven, Laboratory of Clinical and Epidemiological Virology	Zlateva,K.T., Lemey,P., Vandamme,A.-M. and Van Ranst,M.
EPI_ISL_2589564	Virology Laboratory, Dr. Ricardo Gutierrez Children Hospital	Virology Laboratory, Dr. Ricardo Gutierrez Children Hospital	Viegas,M., Goya,S. and Mistchenko,A.S.
EPI_ISL_2589565	Department of Microbiology and Immunology, Laboratory of Clinical and Epidemiological Virology, Rega Institute for Medical Research	Department of Microbiology and Immunology, Laboratory of Clinical and Epidemiological Virology, Rega Institute for Medical Research	Zlateva,K.T., Vijgen,L., Dekeersmaeker,N., Naranjo,C. and Van Ranst,M.
EPI_ISL_2589566, EPI_ISL_2589567, EPI_ISL_2589568, EPI_ISL_2589569, EPI_ISL_2589570, EPI_ISL_2589571	Department of Infectious, Parasitic and Immunomediated Diseases, Istituto Superiore di Sanita'	Department of Infectious, Parasitic and Immunomediated Diseases, Istituto Superiore di Sanita'	Montieri,S., Puzelli,S., Ciccozzi,M., Calzoletti,L. and Di Martino,A.
EPI_ISL_2589572, EPI_ISL_2589573, EPI_ISL_2589574, EPI_ISL_2589575	Virology Laboratory, Dr. Ricardo Gutierrez Children Hospital	Virology Laboratory, Dr. Ricardo Gutierrez Children Hospital	Viegas,M. and Mistchenko,A.S.
EPI_ISL_2589576, EPI_ISL_2589577	Department of Microbiology & Immunology, Rega Institute for Medical Research, University of Leuven, Laboratory of Clinical and Epidemiological Virology	Department of Microbiology & Immunology, Rega Institute for Medical Research, University of Leuven, Laboratory of Clinical and Epidemiological Virology	Zlateva,K.T., Lemey,P., Vandamme,A.-M. and Van Ranst,M.
EPI_ISL_2589578, EPI_ISL_2589579	Virology Laboratory, Dr. Ricardo Gutierrez Children Hospital	Virology Laboratory, Dr. Ricardo Gutierrez Children Hospital	Viegas,M., Goya,S. and Mistchenko,A.S.
EPI_ISL_2589580	Immunology and Microbiology, Rega Institute for Medical Research	Immunology and Microbiology, Rega Institute for Medical Research	Houspie,L., Lemey,P., Keyaerts,E., Reijmen,E., Vergote,V., Vankeerberghen,A., Vaeyens,F., De Beenhouwer,H. and Van Ranst,M.
EPI_ISL_2589581, EPI_ISL_2589582	Virology Laboratory, Dr. Ricardo Gutierrez Children Hospital	Virology Laboratory, Dr. Ricardo Gutierrez Children Hospital	Viegas,M. and Mistchenko,A.S.
EPI_ISL_2589583	Department of Infectious, Parasitic and Immunomediated Diseases, Istituto Superiore di Sanita'	Department of Infectious, Parasitic and Immunomediated Diseases, Istituto Superiore di Sanita'	Montieri,S., Puzelli,S., Ciccozzi,M., Calzoletti,L. and Di Martino,A.
EPI_ISL_2589584, EPI_ISL_2589585, EPI_ISL_2589586, EPI_ISL_2589587, EPI_ISL_2589588, EPI_ISL_2589589, EPI_ISL_2589590	Virology Laboratory, Dr. Ricardo Gutierrez Children Hospital	Virology Laboratory, Dr. Ricardo Gutierrez Children Hospital	Viegas,M. and Mistchenko,A.S.
EPI_ISL_2589591, EPI_ISL_2589592, EPI_ISL_2589593, EPI_ISL_2589594, EPI_ISL_2589595	Department of Microbiology & Immunology, Rega Institute for Medical Research, University of Leuven, Laboratory of Clinical and Epidemiological Virology	Department of Microbiology & Immunology, Rega Institute for Medical Research, University of Leuven, Laboratory of Clinical and Epidemiological Virology	Zlateva,K.T., Lemey,P., Vandamme,A.-M. and Van Ranst,M.
EPI_ISL_2589596	Immunology and Microbiology, Rega Institute for Medical Research	Immunology and Microbiology, Rega Institute for Medical Research	Houspie,L., Lemey,P., Keyaerts,E., Reijmen,E., Vergote,V., Vankeerberghen,A., Vaeyens,F., De Beenhouwer,H. and Van Ranst,M.
EPI_ISL_2589597	Department of Microbiology & Immunology, Rega Institute for Medical Research, University of Leuven, Laboratory of Clinical and Epidemiological Virology	Department of Microbiology & Immunology, Rega Institute for Medical Research, University of Leuven, Laboratory of Clinical and Epidemiological Virology	Zlateva,K.T., Lemey,P., Vandamme,A.-M. and Van Ranst,M.
EPI_ISL_2589599	Virology Laboratory, Dr. Ricardo Gutierrez Children Hospital	Virology Laboratory, Dr. Ricardo Gutierrez Children Hospital	Viegas,M. and Mistchenko,A.S.
EPI_ISL_2589600	Immunology and Microbiology, Rega Institute for Medical Research	Immunology and Microbiology, Rega Institute for Medical Research	Houspie,L., Lemey,P., Keyaerts,E., Reijmen,E., Vergote,V., Vankeerberghen,A., Vaeyens,F., De Beenhouwer,H. and Van Ranst,M.
EPI_ISL_2589601, EPI_ISL_2589602	Department of Infectious, Parasitic and Immunomediated Diseases, Istituto Superiore di Sanita'	Department of Infectious, Parasitic and Immunomediated Diseases, Istituto Superiore di Sanita'	Montieri,S., Puzelli,S., Ciccozzi,M., Calzoletti,L. and Di Martino,A.
EPI_ISL_2589603, EPI_ISL_2589604, EPI_ISL_2589605, EPI_ISL_2589606, EPI_ISL_2589607, EPI_ISL_2589608, EPI_ISL_2589609	Virology Laboratory, Dr. Ricardo Gutierrez Children Hospital	Virology Laboratory, Dr. Ricardo Gutierrez Children Hospital	Viegas,M. and Mistchenko,A.S.
EPI_ISL_2589610	Institute for Virology, University Duesseldorf	Institute for Virology, University Duesseldorf	Adams,O., Werczmircorwski,J. and Hengel,H.
EPI_ISL_2589611	Department of Microbiology & Immunology, Rega Institute for Medical Research, University of Leuven, Laboratory of Clinical and Epidemiological Virology	Department of Microbiology & Immunology, Rega Institute for Medical Research, University of Leuven, Laboratory of Clinical and Epidemiological Virology	Zlateva,K.T., Lemey,P., Vandamme,A.-M. and Van Ranst,M.
EPI_ISL_2589612	Department of Microbiology and Immunology, Laboratory of Clinical and Epidemiological Virology, Rega Institute for Medical Research	Department of Microbiology and Immunology, Laboratory of Clinical and Epidemiological Virology, Rega Institute for Medical Research	Zlateva,K.T., Vijgen,L., Dekeersmaeker,N., Naranjo,C. and Van Ranst,M.
EPI_ISL_2589613	Immunology and Microbiology, Rega Institute for Medical Research	Immunology and Microbiology, Rega Institute for Medical Research	Houspie,L., Lemey,P., Keyaerts,E., Reijmen,E., Vergote,V., Vankeerberghen,A., Vaeyens,F., De Beenhouwer,H. and Van Ranst,M.
EPI_ISL_2589614, EPI_ISL_2589615, EPI_ISL_2589616	Department of Microbiology & Immunology, Rega Institute for Medical Research, University of Leuven, Laboratory of Clinical and Epidemiological Virology	Department of Microbiology & Immunology, Rega Institute for Medical Research, University of Leuven, Laboratory of Clinical and Epidemiological Virology	Zlateva,K.T., Lemey,P., Vandamme,A.-M. and Van Ranst,M.
EPI_ISL_2589617	Department of Infectious, Parasitic and Immunomediated Diseases, Istituto Superiore di Sanita'	Department of Infectious, Parasitic and Immunomediated Diseases, Istituto Superiore di Sanita'	Montieri,S., Puzelli,S., Ciccozzi,M., Calzoletti,L. and Di Martino,A.
EPI_ISL_2589619	Immunology and Microbiology, Rega Institute for Medical Research	Immunology and Microbiology, Rega Institute for Medical Research	Houspie,L., Lemey,P., Keyaerts,E., Reijmen,E., Vergote,V., Vankeerberghen,A., Vaeyens,F., De Beenhouwer,H. and Van Ranst,M.
EPI_ISL_2589621	Virology Laboratory, Dr. Ricardo Gutierrez Children Hospital	Virology Laboratory, Dr. Ricardo Gutierrez Children Hospital	Viegas,M. and Mistchenko,A.S.
EPI_ISL_2589622	Department of Infectious, Parasitic and Immunomediated Diseases, Istituto Superiore di Sanita'	Department of Infectious, Parasitic and Immunomediated Diseases, Istituto Superiore di Sanita'	Montieri,S., Puzelli,S., Ciccozzi,M., Calzoletti,L. and Di Martino,A.
EPI_ISL_2589623, EPI_ISL_2589624, EPI_ISL_2589625, EPI_ISL_2589626, EPI_ISL_2589627, EPI_ISL_2589628, EPI_ISL_2589629, EPI_ISL_2589630, EPI_ISL_2589631, EPI_ISL_2589632, EPI_ISL_2589633, EPI_ISL_2589634, EPI_ISL_2589635, EPI_ISL_2589636, EPI_ISL_2589637, EPI_ISL_2589638, EPI_ISL_2589639, EPI_ISL_2589640, EPI_ISL_2589641, EPI_ISL_2589642, EPI_ISL_2589643, EPI_ISL_2589644			
see above	Virology Laboratory, Dr. Ricardo Gutierrez Children Hospital	Virology Laboratory, Dr. Ricardo Gutierrez Children Hospital	Viegas,M. and Mistchenko,A.S.
EPI_ISL_2589645	Department of Microbiology and Immunology, Laboratory of Clinical and Epidemiological Virology, Rega Institute for Medical Research	Department of Microbiology and Immunology, Laboratory of Clinical and Epidemiological Virology, Rega Institute for Medical Research	Zlateva,K.T., Vijgen,L., Dekeersmaeker,N., Naranjo,C. and Van Ranst,M.
EPI_ISL_2589646, EPI_ISL_2589647, EPI_ISL_2589648	Department of Microbiology & Immunology, Rega Institute for Medical Research, University of Leuven, Laboratory of Clinical and Epidemiological Virology	Department of Microbiology & Immunology, Rega Institute for Medical Research, University of Leuven, Laboratory of Clinical and Epidemiological Virology	Zlateva,K.T., Lemey,P., Vandamme,A.-M. and Van Ranst,M.
EPI_ISL_2589649	Department of Infectious, Parasitic and Immunomediated Diseases, Istituto Superiore di Sanita'	Department of Infectious, Parasitic and Immunomediated Diseases, Istituto Superiore di Sanita'	Montieri,S., Puzelli,S., Ciccozzi,M., Calzoletti,L. and Di Martino,A.
EPI_ISL_2589663	Virology Laboratory, Dr. Ricardo Gutierrez Children Hospital	Virology Laboratory, Dr. Ricardo Gutierrez Children Hospital	Viegas,M., Goya,S. and Mistchenko,A.S.
EPI_ISL_2589665, EPI_ISL_2589666, EPI_ISL_2589667, EPI_ISL_2589668, EPI_ISL_2589669, EPI_ISL_2589670, EPI_ISL_2589671, EPI_ISL_2589672, EPI_ISL_2589673, EPI_ISL_2589674, EPI_ISL_2589675, EPI_ISL_2589676, EPI_ISL_2589677, EPI_ISL_2589678, EPI_ISL_2589679, EPI_ISL_2589680			
see above	Virology Laboratory, Dr. Ricardo Gutierrez Children Hospital	Virology Laboratory, Dr. Ricardo Gutierrez Children Hospital	Viegas,M. and Mistchenko,A.S.
EPI_ISL_2589681	Department of Microbiology and Immunology, Laboratory of Clinical and Epidemiological Virology, Rega Institute for Medical Research	Department of Microbiology and Immunology, Laboratory of Clinical and Epidemiological Virology, Rega Institute for Medical Research	Zlateva,K.T., Vijgen,L., Dekeersmaeker,N., Naranjo,C. and Van Ranst,M.
EPI_ISL_2589682, EPI_ISL_2589684, EPI_ISL_2589685	Department of Microbiology & Immunology, Rega Institute for Medical Research, University of Leuven, Laboratory of Clinical and Epidemiological Virology	Department of Microbiology & Immunology, Rega Institute for Medical Research, University of Leuven, Laboratory of Clinical and Epidemiological Virology	Zlateva,K.T., Lemey,P., Vandamme,A.-M. and Van Ranst,M.
EPI_ISL_2589691, EPI_ISL_2589692, EPI_ISL_2589693	Virology Laboratory, Dr. Ricardo Gutierrez Children Hospital	Virology Laboratory, Dr. Ricardo Gutierrez Children Hospital	Viegas,M., Goya,S. and Mistchenko,A.S.

EPI_ISL_2589698	Institute for Virology, University Duesseldorf	Institute for Virology, University Duesseldorf	Adams,O., Werczmircorwski,J. and Hengel,H.
EPI_ISL_2589699	Department of Microbiology & Immunology, Rega Institute for Medical Research, University of Leuven, Laboratory of Clinical and Epidemiological Virology	Department of Microbiology & Immunology, Rega Institute for Medical Research, University of Leuven, Laboratory of Clinical and Epidemiological Virology	Zlateva,K.T., Lemey,P., Vandamme,A.-M. and Van Ranst,M.
EPI_ISL_2589700	Department of Microbiology and Immunology, Laboratory of Clinical and Epidemiological Virology, Rega Institute for Medical Research	Department of Microbiology and Immunology, Laboratory of Clinical and Epidemiological Virology, Rega Institute for Medical Research	Zlateva,K.T., Vijgen,L., Dekeersmaeker,N., Naranjo,C. and Van Ranst,M.
EPI_ISL_2589701	Institute for Virology, University Duesseldorf	Institute for Virology, University Duesseldorf	Adams,O., Werczmircorwski,J. and Hengel,H.
EPI_ISL_2589703	Immunology and Microbiology, Rega Institute for Medical Research	Immunology and Microbiology, Rega Institute for Medical Research	Houspie,L., Lemey,P., Keyaerts,E., Reijmen,E., Vergote,V., Vankeerberghen,A., Vaeyens,F., De Beenhouwer,H. and Van Ranst,M.
EPI_ISL_2589705, EPI_ISL_2589706	Department of Microbiology & Immunology, Rega Institute for Medical Research, University of Leuven, Laboratory of Clinical and Epidemiological Virology	Department of Microbiology & Immunology, Rega Institute for Medical Research, University of Leuven, Laboratory of Clinical and Epidemiological Virology	Zlateva,K.T., Lemey,P., Vandamme,A.-M. and Van Ranst,M.
EPI_ISL_2589711, EPI_ISL_2589712, EPI_ISL_2589713, EPI_ISL_2589714, EPI_ISL_2589715	Virology Laboratory, Dr. Ricardo Gutierrez Children Hospital	Virology Laboratory, Dr. Ricardo Gutierrez Children Hospital	Viegas,M., Goya,S. and Mistchenko,A.S.
EPI_ISL_2589718, EPI_ISL_2589719, EPI_ISL_2589720	Department of Microbiology and Immunology, Laboratory of Clinical and Epidemiological Virology, Rega Institute for Medical Research	Department of Microbiology and Immunology, Laboratory of Clinical and Epidemiological Virology, Rega Institute for Medical Research	Zlateva,K.T., Vijgen,L., Dekeersmaeker,N., Naranjo,C. and Van Ranst,M.
EPI_ISL_2589722, EPI_ISL_2589723, EPI_ISL_2589724, EPI_ISL_2589726	Immunology and Microbiology, Rega Institute for Medical Research	Immunology and Microbiology, Rega Institute for Medical Research	Houspie,L., Lemey,P., Keyaerts,E., Reijmen,E., Vergote,V., Vankeerberghen,A., Vaeyens,F., De Beenhouwer,H. and Van Ranst,M.
EPI_ISL_2589727, EPI_ISL_2589728	Department of Microbiology & Immunology, Rega Institute for Medical Research, University of Leuven, Laboratory of Clinical and Epidemiological Virology	Department of Microbiology & Immunology, Rega Institute for Medical Research, University of Leuven, Laboratory of Clinical and Epidemiological Virology	Zlateva,K.T., Lemey,P., Vandamme,A.-M. and Van Ranst,M.
EPI_ISL_2589735, EPI_ISL_2589736, EPI_ISL_2589737, EPI_ISL_2589739, EPI_ISL_2589742, EPI_ISL_2589743, EPI_ISL_2589752	Immunology and Microbiology, Rega Institute for Medical Research	Immunology and Microbiology, Rega Institute for Medical Research	Houspie,L., Lemey,P., Keyaerts,E., Reijmen,E., Vergote,V., Vankeerberghen,A., Vaeyens,F., De Beenhouwer,H. and Van Ranst,M.
EPI_ISL_2589754	Department of Microbiology & Immunology, Rega Institute for Medical Research, University of Leuven, Laboratory of Clinical and Epidemiological Virology	Department of Microbiology & Immunology, Rega Institute for Medical Research, University of Leuven, Laboratory of Clinical and Epidemiological Virology	Zlateva,K.T., Lemey,P., Vandamme,A.-M. and Van Ranst,M.
EPI_ISL_2589763, EPI_ISL_2589767, EPI_ISL_2589769, EPI_ISL_2589771, EPI_ISL_2589775, EPI_ISL_2589776, EPI_ISL_2589778, EPI_ISL_2589780, EPI_ISL_2589781, EPI_ISL_2589783, EPI_ISL_2589785, EPI_ISL_2589787			
see above	Immunology and Microbiology, Rega Institute for Medical Research	Immunology and Microbiology, Rega Institute for Medical Research	Houspie,L., Lemey,P., Keyaerts,E., Reijmen,E., Vergote,V., Vankeerberghen,A., Vaeyens,F., De Beenhouwer,H. and Van Ranst,M.
EPI_ISL_2589789, EPI_ISL_2589791, EPI_ISL_2589794, EPI_ISL_2589796	Institute for Virology, University Duesseldorf	Institute for Virology, University Duesseldorf	Adams,O., Werczmircorwski,J. and Hengel,H.
EPI_ISL_2589802, EPI_ISL_2589805, EPI_ISL_2589807, EPI_ISL_2589811, EPI_ISL_2589813, EPI_ISL_2589815, EPI_ISL_2589817, EPI_ISL_2589819, EPI_ISL_2589821, EPI_ISL_2589822, EPI_ISL_2589824, EPI_ISL_2589826, EPI_ISL_2589828, EPI_ISL_2589830, EPI_ISL_2589834, EPI_ISL_2589836, EPI_ISL_2589838, EPI_ISL_2589850			
see above	Immunology and Microbiology, Rega Institute for Medical Research	Immunology and Microbiology, Rega Institute for Medical Research	Houspie,L., Lemey,P., Keyaerts,E., Reijmen,E., Vergote,V., Vankeerberghen,A., Vaeyens,F., De Beenhouwer,H. and Van Ranst,M.
EPI_ISL_2589865	Jiangxi Province Center for Disease Control and Prevention	Jiangxi Province Center for Disease Control and Prevention	Xiong,Y., Gong,T., Wang,F.X., Shi,Y. and Zhou,J.
EPI_ISL_2589867, EPI_ISL_2589877	Institute for Virology, University Duesseldorf	Institute for Virology, University Duesseldorf	Adams,O., Werczmircorwski,J. and Hengel,H.
EPI_ISL_2589881, EPI_ISL_2589883, EPI_ISL_2589892, EPI_ISL_2589900	Jiangxi Province Center for Disease Control and Prevention	Jiangxi Province Center for Disease Control and Prevention	Xiong,Y., Gong,T., Wang,F.X., Shi,Y. and Zhou,J.
EPI_ISL_2589916	Virology, Facultad de Ciencias	Virology, Facultad de Ciencias	Moura,F., Blanc,A. and Frabasile,S.
EPI_ISL_2589918, EPI_ISL_2589919, EPI_ISL_2589921, EPI_ISL_2589923	Virology Branch, Butantan Institute	Virology Branch, Butantan Institute	Botosso,V.F., Peret,T.C.T., Thomazelli,L.M., Stewien,K.E. and Durigon,E.L.
EPI_ISL_2589927, EPI_ISL_2589929, EPI_ISL_2589930, EPI_ISL_2589933, EPI_ISL_2589935, EPI_ISL_2589937, EPI_ISL_2589939, EPI_ISL_2589941, EPI_ISL_2589942, EPI_ISL_2589944, EPI_ISL_2589946, EPI_ISL_2589948			
see above	Department of Microbiology, Institute of Biomedical Science, University of Sao Paulo	Department of Microbiology, Institute of Biomedical Science, University of Sao Paulo	Zukurov,J.P.L. and Durigon,E.L.
EPI_ISL_2589950	Microbiology, Adolfo Lutz Institut	Microbiology, Adolfo Lutz Institut	Souza,M.C.O., Matsumoto,T.K., Vaz-de-Lima,L.L.R., Sato,N.N., Salgado,M.M., Requejo,H.I.Z., Hong,M.A., Barbosa,M.L., Oliveira,C.A.F., Benelli,P.C., Shimokawa,R.Y., Pecchini,R., Berezin,E., Duarte,S.P., Schvartsman,C., Durigon,E.L. and Ueda,M.
EPI_ISL_2589954	Virology Branch, Butantan Institute	Virology Branch, Butantan Institute	Botosso,V.F., Peret,T.C.T., Thomazelli,L.M., Stewien,K.E. and Durigon,E.L.
EPI_ISL_2589956	Microbiology, Adolfo Lutz Institut	Microbiology, Adolfo Lutz Institut	Souza,M.C.O., Matsumoto,T.K., Vaz-De-Lima,L.L.R.A., Sato,N.N., Salgado,M.M., Requejo,H.I.Z., Hong,M.A., Barbosa,M.L., Oliveira,C.A.F., Benelli,P.C., Shimokawa,R.Y., Pecchini,R., Berezin,E., Duarte,S.P., Schvartsman,C., Durigon,E.L. and Ueda,M.
EPI_ISL_2589972	Cell Biology, University of Sao Paulo School of Medicine	Cell Biology, University of Sao Paulo School of Medicine	Proenca-Modena,J.L., Paula,F.E., Iwamoto,M.A., Ansarah-Sobrinho,C., Saranzo,A.M., Santos,A.E., Cintra,O.A.L. and Arruda,E.
EPI_ISL_2589974	Department of Microbiology and Immunolgy, Institute of Biology, State University of Campinas (UNICAMP)	Department of Microbiology and Immunolgy, Institute of Biology, State University of Campinas (UNICAMP)	da Silva,L.H.A., Spilki,F.R., Riccetto,A.G.L., de Almeida,R.S., Baracat,E.C.E. and Arns,C.W.
EPI_ISL_2589978, EPI_ISL_2589980	Virology Branch, Butantan Institute	Virology Branch, Butantan Institute	Botosso,V.F., Peret,T.C.T., Thomazelli,L.M., Stewien,K.E. and Durigon,E.L.
EPI_ISL_2589984	Virology Laboratory, Dr. Ricardo Gutierrez Children Hospital	Virology Laboratory, Dr. Ricardo Gutierrez Children Hospital	Viegas,M. and Mistchenko,A.S.
EPI_ISL_2589986	Department of Microbiology, Institute of Biomedical Science, University of Sao Paulo	Department of Microbiology, Institute of Biomedical Science, University of Sao Paulo	Campos,A.C.A., Botosso,V.F., Souza,T.S., Moraes,C.T.P., Oliveira,D.B.L., Sant'Anna,O.A.B.E., Stewien,K.E. and Durigon,E.L.
EPI_ISL_2589988	Division of Viral Infections, National Reference Center for Influenza, Robert Koch Institute	Division of Viral Infections, National Reference Center for Influenza, Robert Koch Institute	Reiche,J., Zadow,I., Lehmann,H., Tietze,S., Hafemann,S., Kluge,P. and Schweiger,B.
EPI_ISL_2589992, EPI_ISL_2589994	Virology Branch, Butantan Institute	Virology Branch, Butantan Institute	Botosso,V.F., Peret,T.C.T., Thomazelli,L.M., Stewien,K.E. and Durigon,E.L.
EPI_ISL_2589996	Cell Biology, University of Sao Paulo School of Medicine	Cell Biology, University of Sao Paulo School of Medicine	Proenca-Modena,J.L., Paula,F.E., Iwamoto,M.A., Ansarah-Sobrinho,C., Saranzo,A.M., Santos,A.E., Cintra,O.A.L. and Arruda,E.
EPI_ISL_2589998, EPI_ISL_2590000	Department of Microbiology and Immunolgy, Institute of Biology, State University of Campinas (UNICAMP)	Department of Microbiology and Immunolgy, Institute of Biology, State University of Campinas (UNICAMP)	da Silva,L.H.A., Spilki,F.R., Riccetto,A.G.L., de Almeida,R.S., Baracat,E.C.E. and Arns,C.W.
EPI_ISL_2590002, EPI_ISL_2590004	Virology Laboratory, Dr. Ricardo Gutierrez Children Hospital	Virology Laboratory, Dr. Ricardo Gutierrez Children Hospital	Viegas,M., Goya,S. and Mistchenko,A.S.
EPI_ISL_2590006, EPI_ISL_2590008	Immunology and Microbiology, Rega Institute for Medical Research	Immunology and Microbiology, Rega Institute for Medical Research	Houspie,L., Lemey,P., Keyaerts,E., Reijmen,E., Vergote,V., Vankeerberghen,A., Vaeyens,F., De Beenhouwer,H. and Van Ranst,M.
EPI_ISL_2590010	Department of Infectious, Parasitic and Immunomediated Diseases, Istituto Superiore di Sanita'	Department of Infectious, Parasitic and Immunomediated Diseases, Istituto Superiore di Sanita'	Montieri,S., Puzelli,S., Ciccozzi,M., Calzoletti,L. and Di Martino,A.
EPI_ISL_2590012	Virology Branch, Butantan Institute	Virology Branch, Butantan Institute	Botosso,V.F., Peret,T.C.T., Thomazelli,L.M., Stewien,K.E. and Durigon,E.L.
EPI_ISL_2590014, EPI_ISL_2590016	Department of Microbiology and Immunology, Laboratory of Clinical and Epidemiological Virology, Rega Institute for Medical Research	Department of Microbiology and Immunology, Laboratory of Clinical and Epidemiological Virology, Rega Institute for Medical Research	Zlateva,K.T., Vijgen,L., Dekeersmaeker,N., Naranjo,C. and Van Ranst,M.
EPI_ISL_2590018	Virology Unit, Institut Pasteur in Cambodia	Virology Unit, Institut Pasteur in Cambodia	Arnott,A., Vong,S., Sek,M., Chu,S., Naughtin,M., Sovann,L., Buecher,C., Beaute,J., Rith,S., Borand,L., Asgari,N., Guillard,B., Touch,S., Deubel,V. and Buchy,P.
EPI_ISL_2590020	Institute for Virology, University Duesseldorf	Institute for Virology, University Duesseldorf	Adams,O., Werczmircorwski,J. and Hengel,H.
EPI_ISL_2590022	Virology Laboratory, Dr. Ricardo Gutierrez Children Hospital	Virology Laboratory, Dr. Ricardo Gutierrez Children Hospital	Viegas,M. and Mistchenko,A.S.
EPI_ISL_2590023	Immunology and Microbiology, Rega Institute for Medical Research	Immunology and Microbiology, Rega Institute for Medical Research	Houspie,L., Lemey,P., Keyaerts,E., Reijmen,E., Vergote,V., Vankeerberghen,A., Vaeyens,F., De Beenhouwer,H. and Van Ranst,M.
EPI_ISL_2590025	Department of Microbiology, University of Sao Paulo Institute of Biomedical Science	Department of Microbiology, University of Sao Paulo Institute of Biomedical Science	Lima,H.N., Oliveira,D.B.L., Botosso,V.F., Stewien,K.E. and Durigon,E.L.
EPI_ISL_2590027	Clinical Microbiology, Trinity College Dublin	Clinical Microbiology, Trinity College Dublin	Salter,A.C., Crowley,B. and Ni Laoi,B.
EPI_ISL_2590030	Department of Microbiology, University of Sao Paulo Institute of Biomedical Science	Department of Microbiology, University of Sao Paulo Institute of Biomedical Science	Lima,H.N., Oliveira,D.B.L., Botosso,V.F., Stewien,K.E. and Durigon,E.L.
EPI_ISL_2590031	Department of Microbiology, Institute of Biomedical Science, University of Sao Paulo	Department of Microbiology, Institute of Biomedical Science, University of Sao Paulo	Oliveira,D.B.L., Carvalho,A.C.L., Botosso,V.F., Sant' Anna,O.A.B.E., Stewien,K.E. and Durigon,E.L.
EPI_ISL_2590033	Department of Microbiology, University of Sao Paulo Institute of Biomedical Science	Department of Microbiology, University of Sao Paulo Institute of Biomedical Science	Lima,H.N., Oliveira,D.B.L., Botosso,V.F., Stewien,K.E. and Durigon,E.L.
EPI_ISL_2590035	Department of Microbiology, Institute of Biomedical Science	Department of Microbiology, Institute of Biomedical Science	Moraes,C.T.P., Botosso,V.F., Sacramento,P., Comone,P., Souza,T.S., Oliveira,D.B.L., Stewien,K.E., Sant'Anna,O.A.B.E. and Durigon,E.L.
EPI_ISL_2590037, EPI_ISL_2590039	Virology Branch, Butantan Institute	Virology Branch, Butantan Institute	Botosso,V.F., Peret,T.C.T., Thomazelli,L.M., Stewien,K.E. and Durigon,E.L.
EPI_ISL_2590041	Department of Microbiology, University of Sao Paulo Institute of Biomedical Science	Department of Microbiology, University of Sao Paulo Institute of Biomedical Science	Lima,H.N., Oliveira,D.B.L., Botosso,V.F., Stewien,K.E. and Durigon,E.L.
EPI_ISL_2590042, EPI_ISL_2590044	Virology Branch, Butantan Institute	Virology Branch, Butantan Institute	Botosso,V.F., Peret,T.C.T., Thomazelli,L.M., Stewien,K.E. and Durigon,E.L.
EPI_ISL_2590046	Virology Branch, Butantan Institute	Virology Branch, Butantan Institute	Botosso,V.F., Sacramento,P., Comone,P., Oliveira,D.B.L., Souza,T.S., Moraes,C.T.P., Stewien,K.E., Sant'Anna,O.A.B.E. and Durigon,E.L.
EPI_ISL_2590048, EPI_ISL_2590050, EPI_ISL_2590052, EPI_ISL_2590054, EPI_ISL_2590056, EPI_ISL_2590058, EPI_ISL_2590059, EPI_ISL_2590060	Virology Branch, Butantan Institute	Virology Branch, Butantan Institute	Botosso,V.F., Peret,T.C.T., Thomazelli,L.M., Stewien,K.E. and Durigon,E.L.
EPI_ISL_2590062	Department of Microbiology, Institute of Biomedical Science, University of Sao Paulo	Department of Microbiology, Institute of Biomedical Science, University of Sao Paulo	Campos,A.C.A., Botosso,V.F., Souza,T.S., Moraes,C.T.P., Oliveira,D.B.L., Sant'Anna,O.A.B.E., Stewien,K.E. and Durigon,E.L.
EPI_ISL_2590064	Department of Microbiology, Institute of Biomedical Science	Department of Microbiology, Institute of Biomedical Science	Moraes,C.T.P., Botosso,V.F., Sacramento,P., Comone,P., Souza,T.S., Oliveira,D.B.L., Stewien,K.E., Sant'Anna,O.A.B.E. and Durigon,E.L.
EPI_ISL_2590066, EPI_ISL_2590068, EPI_ISL_2590070	Virology Branch, Butantan Institute	Virology Branch, Butantan Institute	Botosso,V.F., Peret,T.C.T., Thomazelli,L.M., Stewien,K.E. and Durigon,E.L.
EPI_ISL_2590072	Department of Microbiology, Institute of Biomedical Science	Department of Microbiology, Institute of Biomedical Science	Moraes,C.T.P., Botosso,V.F., Sacramento,P., Comone,P., Souza,T.S., Oliveira,D.B.L., Stewien,K.E., Sant'Anna,O.A.B.E. and Durigon,E.L.
EPI_ISL_2590074	Virology Branch, Butantan Institute	Virology Branch, Butantan Institute	Botosso,V.F., Peret,T.C.T., Thomazelli,L.M., Stewien,K.E. and Durigon,E.L.
EPI_ISL_2590076	Department of Microbiology, Institute of Biomedical Science, University of Sao Paulo	Department of Microbiology, Institute of Biomedical Science, University of Sao Paulo	Oliveira,D.B.L., Carvalho,A.C.L., Botosso,V.F., Sant' Anna,O.A.B.E., Stewien,K.E. and Durigon,E.L.
EPI_ISL_2590078	Department of Microbiology, Institute of Biomedical Science, University of Sao Paulo	Department of Microbiology, Institute of Biomedical Science, University of Sao Paulo	Campos,A.C.A., Botosso,V.F., Souza,T.S., Moraes,C.T.P., Oliveira,D.B.L., Sant'Anna,O.A.B.E., Stewien,K.E. and Durigon,E.L.
EPI_ISL_2590080	Virology Branch, Butantan Institute	Virology Branch, Butantan Institute	Botosso,V.F., Peret,T.C.T., Thomazelli,L.M., Stewien,K.E. and Durigon,E.L.
EPI_ISL_2590081	Virology Branch, Butantan Institute	Virology Branch, Butantan Institute	Botosso,V.F., Sacramento,P., Comone,P., Oliveira,D.B.L., Souza,T.S., Moraes,C.T.P., Stewien,K.E., Sant'Anna,O.A.B.E. and Durigon,E.L.

EPI_ISL_2590083	Department of Microbiology, Institute of Biomedical Science	Department of Microbiology, Institute of Biomedical Science	Moraes,C.T.P., Botosso,V.F., Sacramento,P., Comone,P., Souza,T.S., Oliveira,D.B.L., Stewien,K.E., Sant'Anna,O.A.B.E. and Durigon,E.L.
EPI_ISL_2590085, EPI_ISL_2590087, EPI_ISL_2590089, EPI_ISL_2590091, EPI_ISL_2590093, EPI_ISL_2590095, EPI_ISL_2590097, EPI_ISL_2590099	Virology Branch, Butantan Institute	Virology Branch, Butantan Institute	Botosso,V.F., Peret,T.C.T., Thomazelli,L.M., Stewien,K.E. and Durigon,E.L.
EPI_ISL_2590100, EPI_ISL_2590102, EPI_ISL_2590104	Virology Branch, Butantan Institute	Virology Branch, Butantan Institute	Botosso,V.F., Sacramento,P., Comone,P., Oliveira,D.B.L., Souza,T.S., Moraes,C.T.P., Stewien,K.E., Sant'Anna,O.A.B.E. and Durigon,E.L.
EPI_ISL_2590106	Virology Branch, Butantan Institute	Virology Branch, Butantan Institute	Botosso,V.F., Peret,T.C.T., Thomazelli,L.M., Stewien,K.E. and Durigon,E.L.
EPI_ISL_2590107, EPI_ISL_2590109, EPI_ISL_2590111	Department of Microbiology, Institute of Biomedical Science	Department of Microbiology, Institute of Biomedical Science	Moraes,C.T.P., Botosso,V.F., Sacramento,P., Comone,P., Souza,T.S., Oliveira,D.B.L., Stewien,K.E., Sant'Anna,O.A.B.E. and Durigon,E.L.
EPI_ISL_2590113, EPI_ISL_2590115, EPI_ISL_2590119, EPI_ISL_2590121, EPI_ISL_2590122	Virology Branch, Butantan Institute	Virology Branch, Butantan Institute	Botosso,V.F., Peret,T.C.T., Thomazelli,L.M., Stewien,K.E. and Durigon,E.L.
EPI_ISL_2590124, EPI_ISL_2590126	Virology Branch, Butantan Institute	Virology Branch, Butantan Institute	Botosso,V.F., Sacramento,P., Comone,P., Oliveira,D.B.L., Souza,T.S., Moraes,C.T.P., Stewien,K.E., Sant'Anna,O.A.B.E. and Durigon,E.L.
EPI_ISL_2590128, EPI_ISL_2590130	Virology Branch, Butantan Institute	Virology Branch, Butantan Institute	Botosso,V.F., Peret,T.C.T., Thomazelli,L.M., Stewien,K.E. and Durigon,E.L.
EPI_ISL_2590132	Virology Branch, Butantan Institute	Virology Branch, Butantan Institute	Botosso,V.F., Sacramento,P., Comone,P., Oliveira,D.B.L., Souza,T.S., Moraes,C.T.P., Stewien,K.E., Sant'Anna,O.A.B.E. and Durigon,E.L.
EPI_ISL_2590133, EPI_ISL_2590135, EPI_ISL_2590137, EPI_ISL_2590139	Cell Biology, University of Sao Paulo School of Medicine	Cell Biology, University of Sao Paulo School of Medicine	Proenca-Modena,J.L., Paula,F.E., Iwamoto,M.A., Ansarah-Sobrinho,C., Saranzo,A.M., Santos,A.E., Cintra,O.A.L. and Arruda,E.
EPI_ISL_2590141	Microbiology, Adolfo Lutz Institut	Microbiology, Adolfo Lutz Institut	Souza,M.C.O., Matsumoto,T.K., Vaz-de-Lima,L.L.R., Sato,N.N., Salgado,M.M., Requejo,H.I.Z., Hong,M.A., Barbosa,M.L., Oliveira,C.A.F., Benelli,P.C., Shimokawa,R.Y., Pecchini,R., Berezin,E., Duarte,S.P., Schvartsman,C., Durigon,E.L. and Ueda,M.
EPI_ISL_2590143	Centre de Recherche en Infectiologie, Centre Hospitalier Universitaire de Quebec (Pavillon CHUL)	Centre de Recherche en Infectiologie, Centre Hospitalier Universitaire de Quebec (Pavillon CHUL)	Gilca,R., De Serres,G., Tremblay,M., Leblanc,E., Bergeron,M.G., Dery,P. and Boivin,G.
EPI_ISL_2590145, EPI_ISL_2590147, EPI_ISL_2590149, EPI_ISL_2590151, EPI_ISL_2590153, EPI_ISL_2590155, EPI_ISL_2590157, EPI_ISL_2590158, EPI_ISL_2590160, EPI_ISL_2590163, EPI_ISL_2590165, EPI_ISL_2590166, EPI_ISL_2590168, EPI_ISL_2590169, EPI_ISL_2590170, EPI_ISL_2590171, EPI_ISL_2590173, EPI_ISL_2590175, EPI_ISL_2590177, EPI_ISL_2590180, EPI_ISL_2590182, EPI_ISL_2590184, EPI_ISL_2590185, EPI_ISL_2590187, EPI_ISL_2590189, EPI_ISL_2590191, EPI_ISL_2590193, EPI_ISL_2590195, EPI_ISL_2590197, EPI_ISL_2590199, EPI_ISL_2590201, EPI_ISL_2590203, EPI_ISL_2590204, EPI_ISL_2590206, EPI_ISL_2590208			
see above	Division of Viral Infections, National Reference Center for Influenza, Robert Koch Institute	Division of Viral Infections, National Reference Center for Influenza, Robert Koch Institute	Reiche,J., Zadow,I., Lehmann,H., Tietze,S., Hafemann,S., Kluge,P. and Schweiger,B.
EPI_ISL_2590210	Institute for Virology, University Duesseldorf	Institute for Virology, University Duesseldorf	Adams,O., Werczmircorwski,J. and Hengel,H.
EPI_ISL_2590211	Virology, NAMRU-15	Virology, NAMRU-15	Garcia,J., Sovero,M. and Halsey,E.S.
EPI_ISL_2590213, EPI_ISL_2590215	Department of Microbiology & Immunology, Rega Institute for Medical Research, University of Leuven, Laboratory of Clinical and Epidemiological Virology	Department of Microbiology & Immunology, Rega Institute for Medical Research, University of Leuven, Laboratory of Clinical and Epidemiological Virology	Zlateva,K.T., Lemey,P., Vandamme,A.-M. and Van Ranst,M.
EPI_ISL_2590216	Department of Microbiology and Immunology, Laboratory of Clinical and Epidemiological Virology, Rega Institute for Medical Research	Department of Microbiology and Immunology, Laboratory of Clinical and Epidemiological Virology, Rega Institute for Medical Research	Zlateva,K.T., Vijgen,L., Dekeersmaeker,N., Naranjo,C. and Van Ranst,M.
EPI_ISL_2590218, EPI_ISL_2590220, EPI_ISL_2590221, EPI_ISL_2590224, EPI_ISL_2590228, EPI_ISL_2590229	Clinical Microbiology, Trinity College Dublin	Clinical Microbiology, Trinity College Dublin	Salter,A.C., Crowley,B. and Ni Laoi,B.
EPI_ISL_2590231	Virology Laboratory, Dr. Ricardo Gutierrez Children Hospital	Virology Laboratory, Dr. Ricardo Gutierrez Children Hospital	Viegas,M., Goya,S. and Mistchenko,A.S.
EPI_ISL_2590234, EPI_ISL_2590235, EPI_ISL_2590237, EPI_ISL_2590238, EPI_ISL_2590240, EPI_ISL_2590242, EPI_ISL_2590244	Department of Infectious, Parasitic and Immunomediated Diseases, Istituto Superiore di Sanita'	Department of Infectious, Parasitic and Immunomediated Diseases, Istituto Superiore di Sanita'	Montieri,S., Puzelli,S., Ciccozzi,M., Calzoletti,L. and Di Martino,A.
EPI_ISL_2590246, EPI_ISL_2590248, EPI_ISL_2590250, EPI_ISL_2590252, EPI_ISL_2590254	Clinical Microbiology, Trinity College Dublin	Clinical Microbiology, Trinity College Dublin	Salter,A.C., Crowley,B. and Ni Laoi,B.
EPI_ISL_2590256, EPI_ISL_2590258, EPI_ISL_2590260, EPI_ISL_2590262, EPI_ISL_2590264, EPI_ISL_2590266, EPI_ISL_2590268, EPI_ISL_2590270, EPI_ISL_2590272, EPI_ISL_2590274	Virology Laboratory, Dr. Ricardo Gutierrez Children Hospital	Virology Laboratory, Dr. Ricardo Gutierrez Children Hospital	Viegas,M. and Mistchenko,A.S.
EPI_ISL_2590276	Department of Microbiology and Immunology, Laboratory of Clinical and Epidemiological Virology, Rega Institute for Medical Research	Department of Microbiology and Immunology, Laboratory of Clinical and Epidemiological Virology, Rega Institute for Medical Research	Zlateva,K.T., Vijgen,L., Dekeersmaeker,N., Naranjo,C. and Van Ranst,M.
EPI_ISL_2590277	Department of Microbiology & Immunology, Rega Institute for Medical Research, University of Leuven, Laboratory of Clinical and Epidemiological Virology	Department of Microbiology & Immunology, Rega Institute for Medical Research, University of Leuven, Laboratory of Clinical and Epidemiological Virology	Zlateva,K.T., Lemey,P., Vandamme,A.-M. and Van Ranst,M.
EPI_ISL_2590279, EPI_ISL_2590281	Department of Microbiology and Immunology, Laboratory of Clinical and Epidemiological Virology, Rega Institute for Medical Research	Department of Microbiology and Immunology, Laboratory of Clinical and Epidemiological Virology, Rega Institute for Medical Research	Zlateva,K.T., Vijgen,L., Dekeersmaeker,N., Naranjo,C. and Van Ranst,M.
EPI_ISL_2590283	Department of Microbiology & Immunology, Rega Institute for Medical Research, University of Leuven, Laboratory of Clinical and Epidemiological Virology	Department of Microbiology & Immunology, Rega Institute for Medical Research, University of Leuven, Laboratory of Clinical and Epidemiological Virology	Zlateva,K.T., Lemey,P., Vandamme,A.-M. and Van Ranst,M.
EPI_ISL_2590285, EPI_ISL_2590287	Department of Microbiology and Immunology, Laboratory of Clinical and Epidemiological Virology, Rega Institute for Medical Research	Department of Microbiology and Immunology, Laboratory of Clinical and Epidemiological Virology, Rega Institute for Medical Research	Zlateva,K.T., Vijgen,L., Dekeersmaeker,N., Naranjo,C. and Van Ranst,M.
EPI_ISL_2590289	Virology Laboratory, Dr. Ricardo Gutierrez Children Hospital	Virology Laboratory, Dr. Ricardo Gutierrez Children Hospital	Viegas,M. and Mistchenko,A.S.
EPI_ISL_2590291	Department of Microbiology, Institute of Biomedical Science	Department of Microbiology, Institute of Biomedical Science	Moraes,C.T.P., Botosso,V.F., Sacramento,P., Comone,P., Souza,T.S., Oliveira,D.B.L., Stewien,K.E., Sant'Anna,O.A.B.E. and Durigon,E.L.
EPI_ISL_2590294	Division of Viral Infections, National Reference Center for Influenza, Robert Koch Institute	Division of Viral Infections, National Reference Center for Influenza, Robert Koch Institute	Reiche,J., Zadow,I., Lehmann,H., Tietze,S., Hafemann,S., Kluge,P. and Schweiger,B.
EPI_ISL_2590296, EPI_ISL_2590297	Immunology and Microbiology, Rega Institute for Medical Research	Immunology and Microbiology, Rega Institute for Medical Research	Houspie,L., Lemey,P., Keyaerts,E., Reijmen,E., Vergote,V., Vankeerberghen,A., Vaeyens,F., De Beenhouwer,H. and Van Ranst,M.
EPI_ISL_2590304, EPI_ISL_2590307, EPI_ISL_2590308, EPI_ISL_2590310, EPI_ISL_2590312, EPI_ISL_2590314	Virology Laboratory, Dr. Ricardo Gutierrez Children Hospital	Virology Laboratory, Dr. Ricardo Gutierrez Children Hospital	Viegas,M. and Mistchenko,A.S.
EPI_ISL_2590316	Virology Laboratory, Dr. Ricardo Gutierrez Children Hospital	Virology Laboratory, Dr. Ricardo Gutierrez Children Hospital	Viegas,M., Goya,S. and Mistchenko,A.S.
EPI_ISL_2590318, EPI_ISL_2590320, EPI_ISL_2590321, EPI_ISL_2590323, EPI_ISL_2590325, EPI_ISL_2590327, EPI_ISL_2590329	Virology Laboratory, Dr. Ricardo Gutierrez Children Hospital	Virology Laboratory, Dr. Ricardo Gutierrez Children Hospital	Viegas,M. and Mistchenko,A.S.
EPI_ISL_2590331, EPI_ISL_2590333, EPI_ISL_2590335	Virology Laboratory, Dr. Ricardo Gutierrez Children Hospital	Virology Laboratory, Dr. Ricardo Gutierrez Children Hospital	Viegas,M., Goya,S. and Mistchenko,A.S.
EPI_ISL_2590338	State Key Laboratory of Molecular Virology and Genetic Engineering, National Institute for Viral Disease Control and Prevention CDC	State Key Laboratory of Molecular Virology and Genetic Engineering, National Institute for Viral Disease Control and Prevention CDC	Zhang,R.F. and Duan,Z.J.
EPI_ISL_2590341, EPI_ISL_2590343, EPI_ISL_2590345, EPI_ISL_2590346, EPI_ISL_2590348, EPI_ISL_2590350, EPI_ISL_2590351, EPI_ISL_2590353, EPI_ISL_2590355, EPI_ISL_2590357, EPI_ISL_2590359			
see above	Division of Viral Infections, National Reference Center for Influenza, Robert Koch Institute	Division of Viral Infections, National Reference Center for Influenza, Robert Koch Institute	Reiche,J., Zadow,I., Lehmann,H., Tietze,S., Hafemann,S., Kluge,P. and Schweiger,B.
EPI_ISL_2590361, EPI_ISL_2590363, EPI_ISL_2590364	Department of Infectious, Parasitic and Immunomediated Diseases, Istituto Superiore di Sanita'	Department of Infectious, Parasitic and Immunomediated Diseases, Istituto Superiore di Sanita'	Montieri,S., Puzelli,S., Ciccozzi,M., Calzoletti,L. and Di Martino,A.
EPI_ISL_2590366, EPI_ISL_2590369, EPI_ISL_2590371, EPI_ISL_2590373, EPI_ISL_2590375, EPI_ISL_2590377	Department of Microbiology & Immunology, Rega Institute for Medical Research, University of Leuven, Laboratory of Clinical and Epidemiological Virology	Department of Microbiology & Immunology, Rega Institute for Medical Research, University of Leuven, Laboratory of Clinical and Epidemiological Virology	Zlateva,K.T., Lemey,P., Vandamme,A.-M. and Van Ranst,M.
EPI_ISL_2590379	Department of Microbiology and Immunology, Laboratory of Clinical and Epidemiological Virology, Rega Institute for Medical Research	Department of Microbiology and Immunology, Laboratory of Clinical and Epidemiological Virology, Rega Institute for Medical Research	Zlateva,K.T., Vijgen,L., Dekeersmaeker,N., Naranjo,C. and Van Ranst,M.
EPI_ISL_2590380	Department of Microbiology & Immunology, Rega Institute for Medical Research, University of Leuven, Laboratory of Clinical and Epidemiological Virology	Department of Microbiology & Immunology, Rega Institute for Medical Research, University of Leuven, Laboratory of Clinical and Epidemiological Virology	Zlateva,K.T., Lemey,P., Vandamme,A.-M. and Van Ranst,M.
EPI_ISL_2590382	Department of Microbiology and Immunology, Laboratory of Clinical and Epidemiological Virology, Rega Institute for Medical Research	Department of Microbiology and Immunology, Laboratory of Clinical and Epidemiological Virology, Rega Institute for Medical Research	Zlateva,K.T., Vijgen,L., Dekeersmaeker,N., Naranjo,C. and Van Ranst,M.
EPI_ISL_2590383	Department of Microbiology & Immunology, Rega Institute for Medical Research, University of Leuven, Laboratory of Clinical and Epidemiological Virology	Department of Microbiology & Immunology, Rega Institute for Medical Research, University of Leuven, Laboratory of Clinical and Epidemiological Virology	Zlateva,K.T., Lemey,P., Vandamme,A.-M. and Van Ranst,M.
EPI_ISL_2590384, EPI_ISL_2590386, EPI_ISL_2590388, EPI_ISL_2590390, EPI_ISL_2590392, EPI_ISL_2590394	Department of Microbiology and Immunology, Laboratory of Clinical and Epidemiological Virology, Rega Institute for Medical Research	Department of Microbiology and Immunology, Laboratory of Clinical and Epidemiological Virology, Rega Institute for Medical Research	Zlateva,K.T., Vijgen,L., Dekeersmaeker,N., Naranjo,C. and Van Ranst,M.
EPI_ISL_2590396, EPI_ISL_2590398, EPI_ISL_2590399, EPI_ISL_2590401	Department of Microbiology & Immunology, Rega Institute for Medical Research, University of Leuven, Laboratory of Clinical and Epidemiological Virology	Department of Microbiology & Immunology, Rega Institute for Medical Research, University of Leuven, Laboratory of Clinical and Epidemiological Virology	Zlateva,K.T., Lemey,P., Vandamme,A.-M. and Van Ranst,M.
EPI_ISL_2590403	Department of Microbiology and Immunology, Laboratory of Clinical and Epidemiological Virology, Rega Institute for Medical Research	Department of Microbiology and Immunology, Laboratory of Clinical and Epidemiological Virology, Rega Institute for Medical Research	Zlateva,K.T., Vijgen,L., Dekeersmaeker,N., Naranjo,C. and Van Ranst,M.
EPI_ISL_2590404	Department of Microbiology & Immunology, Rega Institute for Medical Research, University of Leuven, Laboratory of Clinical and Epidemiological Virology	Department of Microbiology & Immunology, Rega Institute for Medical Research, University of Leuven, Laboratory of Clinical and Epidemiological Virology	Zlateva,K.T., Lemey,P., Vandamme,A.-M. and Van Ranst,M.
EPI_ISL_2590406	Department of Microbiology and Immunology, Laboratory of Clinical and Epidemiological Virology, Rega Institute for Medical Research	Department of Microbiology and Immunology, Laboratory of Clinical and Epidemiological Virology, Rega Institute for Medical Research	Zlateva,K.T., Vijgen,L., Dekeersmaeker,N., Naranjo,C. and Van Ranst,M.
EPI_ISL_2590408, EPI_ISL_2590410, EPI_ISL_2590412	Virology Laboratory, Dr. Ricardo Gutierrez Children Hospital	Virology Laboratory, Dr. Ricardo Gutierrez Children Hospital	Viegas,M. and Mistchenko,A.S.
EPI_ISL_2590414, EPI_ISL_2590416	Department of Microbiology, University of Sao Paulo Institute of Biomedical Science	Department of Microbiology, University of Sao Paulo Institute of Biomedical Science	Lima,H.N., Oliveira,D.B.L., Botosso,V.F., Stewien,K.E. and Durigon,E.L.
EPI_ISL_2590417	Department of Microbiology, Institute of Biomedical Science, University of Sao Paulo	Department of Microbiology, Institute of Biomedical Science, University of Sao Paulo	Campos,A.C.A., Botosso,V.F., Souza,T.S., Moraes,C.T.P., Oliveira,D.B.L., Sant'Anna,O.A.B.E., Stewien,K.E. and Durigon,E.L.

EPI_ISL_2590419	Department of Microbiology, University of Sao Paulo Institute of Biomedical Science	Department of Microbiology, University of Sao Paulo Institute of Biomedical Science	Lima,H.N., Oliveira,D.B.L., Botosso,V.F., Stewien,K.E. and Durigon,E.L.
EPI_ISL_2590421	Department of Microbiology, Institute of Biomedical Science, University of Sao Paulo	Department of Microbiology, Institute of Biomedical Science, University of Sao Paulo	Campos,A.C.A., Botosso,V.F., Souza,T.S., Moraes,C.T.P., Oliveira,D.B.L., Sant'Anna,O.A.B.E., Stewien,K.E. and Durigon,E.L.
EPI_ISL_2590423	Department of Microbiology, University of Sao Paulo Institute of Biomedical Science	Department of Microbiology, University of Sao Paulo Institute of Biomedical Science	Lima,H.N., Oliveira,D.B.L., Botosso,V.F., Stewien,K.E. and Durigon,E.L.
EPI_ISL_2590425	Virology Branch, Butantan Institute	Virology Branch, Butantan Institute	Botosso,V.F., Peret,T.C.T., Thomazelli,L.M., Stewien,K.E. and Durigon,E.L.
EPI_ISL_2590427	Department of Microbiology, University of Sao Paulo Institute of Biomedical Science	Department of Microbiology, University of Sao Paulo Institute of Biomedical Science	Lima,H.N., Oliveira,D.B.L., Botosso,V.F., Stewien,K.E. and Durigon,E.L.
EPI_ISL_2590429	Virology Branch, Butantan Institute	Virology Branch, Butantan Institute	Botosso,V.F., Peret,T.C.T., Thomazelli,L.M., Stewien,K.E. and Durigon,E.L.
EPI_ISL_2590431	Department of Microbiology, Institute of Biomedical Science, University of Sao Paulo	Department of Microbiology, Institute of Biomedical Science, University of Sao Paulo	Campos,A.C.A., Botosso,V.F., Souza,T.S., Moraes,C.T.P., Oliveira,D.B.L., Sant'Anna,O.A.B.E., Stewien,K.E. and Durigon,E.L.
EPI_ISL_2590433	Virology Branch, Butantan Institute	Virology Branch, Butantan Institute	Botosso,V.F., Peret,T.C.T., Thomazelli,L.M., Stewien,K.E. and Durigon,E.L.
EPI_ISL_2590435, EPI_ISL_2590437	Department of Microbiology, Institute of Biomedical Science	Department of Microbiology, Institute of Biomedical Science	Moraes,C.T.P., Botosso,V.F., Sacramento,P., Comone,P., Souza,T.S., Oliveira,D.B.L., Stewien,K.E., Sant'Anna,O.A.B.E. and Durigon,E.L.
EPI_ISL_2590438, EPI_ISL_2590440	Department of Microbiology, Institute of Biomedical Science, University of Sao Paulo	Department of Microbiology, Institute of Biomedical Science, University of Sao Paulo	Campos,A.C.A., Botosso,V.F., Souza,T.S., Moraes,C.T.P., Oliveira,D.B.L., Sant'Anna,O.A.B.E., Stewien,K.E. and Durigon,E.L.
EPI_ISL_2590442, EPI_ISL_2590443	Department of Microbiology, Institute of Biomedical Science	Department of Microbiology, Institute of Biomedical Science	Moraes,C.T.P., Botosso,V.F., Sacramento,P., Comone,P., Souza,T.S., Oliveira,D.B.L., Stewien,K.E., Sant'Anna,O.A.B.E. and Durigon,E.L.
EPI_ISL_2590445	Department of Microbiology, Institute of Biomedical Science, University of Sao Paulo	Department of Microbiology, Institute of Biomedical Science, University of Sao Paulo	Campos,A.C.A., Botosso,V.F., Souza,T.S., Moraes,C.T.P., Oliveira,D.B.L., Sant'Anna,O.A.B.E., Stewien,K.E. and Durigon,E.L.
EPI_ISL_2590446	Department of Microbiology, University of Sao Paulo Institute of Biomedical Science	Department of Microbiology, University of Sao Paulo Institute of Biomedical Science	Lima,H.N., Oliveira,D.B.L., Botosso,V.F., Stewien,K.E. and Durigon,E.L.
EPI_ISL_2590448, EPI_ISL_2590449	Department of Microbiology, Institute of Biomedical Science, University of Sao Paulo	Department of Microbiology, Institute of Biomedical Science, University of Sao Paulo	Campos,A.C.A., Botosso,V.F., Souza,T.S., Moraes,C.T.P., Oliveira,D.B.L., Sant'Anna,O.A.B.E., Stewien,K.E. and Durigon,E.L.
EPI_ISL_2590451	Department of Microbiology, Institute of Biomedical Science	Department of Microbiology, Institute of Biomedical Science	Moraes,C.T.P., Botosso,V.F., Sacramento,P., Comone,P., Souza,T.S., Oliveira,D.B.L., Stewien,K.E., Sant'Anna,O.A.B.E. and Durigon,E.L.
EPI_ISL_2590453	Department of Microbiology, Institute of Biomedical Science, University of Sao Paulo	Department of Microbiology, Institute of Biomedical Science, University of Sao Paulo	Oliveira,D.B.L., Carvalho,A.C.L., Botosso,V.F., Sant' Anna,O.A.B.E., Stewien,K.E. and Durigon,E.L.
EPI_ISL_2590455, EPI_ISL_2590457	Department of Microbiology, Institute of Biomedical Science, University of Sao Paulo	Department of Microbiology, Institute of Biomedical Science, University of Sao Paulo	Campos,A.C.A., Botosso,V.F., Souza,T.S., Moraes,C.T.P., Oliveira,D.B.L., Sant'Anna,O.A.B.E., Stewien,K.E. and Durigon,E.L.
EPI_ISL_2590459	Department of Microbiology, University of Sao Paulo Institute of Biomedical Science	Department of Microbiology, University of Sao Paulo Institute of Biomedical Science	Lima,H.N., Oliveira,D.B.L., Botosso,V.F., Stewien,K.E. and Durigon,E.L.
EPI_ISL_2590461	Department of Microbiology, Institute of Biomedical Science, University of Sao Paulo	Department of Microbiology, Institute of Biomedical Science, University of Sao Paulo	Campos,A.C.A., Botosso,V.F., Souza,T.S., Moraes,C.T.P., Oliveira,D.B.L., Sant'Anna,O.A.B.E., Stewien,K.E. and Durigon,E.L.
EPI_ISL_2590463	Department of Microbiology, Institute of Biomedical Science, University of Sao Paulo	Department of Microbiology, Institute of Biomedical Science, University of Sao Paulo	Oliveira,D.B.L., Carvalho,A.C.L., Botosso,V.F., Sant' Anna,O.A.B.E., Stewien,K.E. and Durigon,E.L.
EPI_ISL_2590464, EPI_ISL_2590467, EPI_ISL_2590469, EPI_ISL_2590470, EPI_ISL_2590472, EPI_ISL_2590474	Department of Microbiology, University of Sao Paulo Institute of Biomedical Science	Department of Microbiology, University of Sao Paulo Institute of Biomedical Science	Lima,H.N., Oliveira,D.B.L., Botosso,V.F., Stewien,K.E. and Durigon,E.L.
EPI_ISL_2590476, EPI_ISL_2590477	Department of Microbiology, Institute of Biomedical Science, University of Sao Paulo	Department of Microbiology, Institute of Biomedical Science, University of Sao Paulo	Campos,A.C.A., Botosso,V.F., Souza,T.S., Moraes,C.T.P., Oliveira,D.B.L., Sant'Anna,O.A.B.E., Stewien,K.E. and Durigon,E.L.
EPI_ISL_2590483	Department of Microbiology, University of Sao Paulo Institute of Biomedical Science	Department of Microbiology, University of Sao Paulo Institute of Biomedical Science	Lima,H.N., Oliveira,D.B.L., Botosso,V.F., Stewien,K.E. and Durigon,E.L.
EPI_ISL_2590484, EPI_ISL_2590485, EPI_ISL_2590487	Department of Microbiology, Institute of Biomedical Science, University of Sao Paulo	Department of Microbiology, Institute of Biomedical Science, University of Sao Paulo	Campos,A.C.A., Botosso,V.F., Souza,T.S., Moraes,C.T.P., Oliveira,D.B.L., Sant'Anna,O.A.B.E., Stewien,K.E. and Durigon,E.L.
EPI_ISL_2590489, EPI_ISL_2590490	Virology Branch, Butantan Institute	Virology Branch, Butantan Institute	Botosso,V.F., Peret,T.C.T., Thomazelli,L.M., Stewien,K.E. and Durigon,E.L.
EPI_ISL_2590492, EPI_ISL_2590494	Department of Microbiology, Institute of Biomedical Science	Department of Microbiology, Institute of Biomedical Science	Moraes,C.T.P., Botosso,V.F., Sacramento,P., Comone,P., Souza,T.S., Oliveira,D.B.L., Stewien,K.E., Sant'Anna,O.A.B.E. and Durigon,E.L.
EPI_ISL_2590496	Department of Microbiology, University of Sao Paulo Institute of Biomedical Science	Department of Microbiology, University of Sao Paulo Institute of Biomedical Science	Lima,H.N., Oliveira,D.B.L., Botosso,V.F., Stewien,K.E. and Durigon,E.L.
EPI_ISL_2590498	Department of Microbiology, Institute of Biomedical Science, University of Sao Paulo	Department of Microbiology, Institute of Biomedical Science, University of Sao Paulo	Oliveira,D.B.L., Carvalho,A.C.L., Botosso,V.F., Sant' Anna,O.A.B.E., Stewien,K.E. and Durigon,E.L.
EPI_ISL_2590500	Department of Microbiology, Institute of Biomedical Science, University of Sao Paulo	Department of Microbiology, Institute of Biomedical Science, University of Sao Paulo	Campos,A.C.A., Botosso,V.F., Souza,T.S., Moraes,C.T.P., Oliveira,D.B.L., Sant'Anna,O.A.B.E., Stewien,K.E. and Durigon,E.L.
EPI_ISL_2590503	Department of Microbiology, Institute of Biomedical Science, University of Sao Paulo	Department of Microbiology, Institute of Biomedical Science, University of Sao Paulo	Oliveira,D.B.L., Carvalho,A.C.L., Botosso,V.F., Sant' Anna,O.A.B.E., Stewien,K.E. and Durigon,E.L.
EPI_ISL_2590504	Department of Microbiology, University of Sao Paulo Institute of Biomedical Science	Department of Microbiology, University of Sao Paulo Institute of Biomedical Science	Lima,H.N., Oliveira,D.B.L., Botosso,V.F., Stewien,K.E. and Durigon,E.L.
EPI_ISL_2590506, EPI_ISL_2590508, EPI_ISL_2590510, EPI_ISL_2590513, EPI_ISL_2590515, EPI_ISL_2590517, EPI_ISL_2590519, EPI_ISL_2590521, EPI_ISL_2590523, EPI_ISL_2590525, EPI_ISL_2590526, EPI_ISL_2590528, EPI_ISL_2590530, EPI_ISL_2590532			
see above	Virology Branch, Butantan Institute	Virology Branch, Butantan Institute	Botosso,V.F., Peret,T.C.T., Thomazelli,L.M., Stewien,K.E. and Durigon,E.L.
EPI_ISL_2590534	Department of Microbiology, Institute of Biomedical Science	Department of Microbiology, Institute of Biomedical Science	Moraes,C.T.P., Botosso,V.F., Sacramento,P., Comone,P., Souza,T.S., Oliveira,D.B.L., Stewien,K.E., Sant'Anna,O.A.B.E. and Durigon,E.L.
EPI_ISL_2590536	Microbiology, Adolfo Lutz Institut	Microbiology, Adolfo Lutz Institut	Souza,M.C.O., Matsumoto,T.K., Vaz-De-Lima,L.L.R.A., Sato,N.N., Salgado,M.M., Requejo,H.I.Z., Hong,M.A., Barbosa,M.L., Oliveira,C.A.F., Benelli,P.C., Shimokawa,R.Y., Pecchini,R., Berezin,E., Duarte,S.P., Schvartsman,C., Durigon,E.L. and Ueda,M.
EPI_ISL_2590538, EPI_ISL_2590539, EPI_ISL_2590541, EPI_ISL_2590543, EPI_ISL_2590545, EPI_ISL_2590546, EPI_ISL_2590548, EPI_ISL_2590550, EPI_ISL_2590552, EPI_ISL_2590555, EPI_ISL_2590556, EPI_ISL_2590558, EPI_ISL_2590560, EPI_ISL_2590562, EPI_ISL_2590564, EPI_ISL_2590566, EPI_ISL_2590568			
see above	Cell Biology, University of Sao Paulo School of Medicine	Cell Biology, University of Sao Paulo School of Medicine	Proenca-Modena,J.L., Paula,F.E., Iwamoto,M.A., Ansarah-Sobrinho,C., Saranzo,A.M., Santos,A.E., Cintra,O.A.L. and Arruda,E.
EPI_ISL_2590570, EPI_ISL_2590572	Microbiology, Adolfo Lutz Institut	Microbiology, Adolfo Lutz Institut	Souza,M.C.O., Matsumoto,T.K., Vaz-de-Lima,L.L.R., Sato,N.N., Salgado,M.M., Requejo,H.I.Z., Hong,M.A., Barbosa,M.L., Oliveira,C.A.F., Benelli,P.C., Shimokawa,R.Y., Pecchini,R., Berezin,E., Duarte,S.P., Schvartsman,C., Durigon,E.L. and Ueda,M.
EPI_ISL_2590573	Research & Development, Institute of Immunology	Research & Development, Institute of Immunology	Ivancic-Jelecki,J. and Forcic,D.
EPI_ISL_2590575, EPI_ISL_2590577, EPI_ISL_2590579, EPI_ISL_2590581, EPI_ISL_2590583, EPI_ISL_2590585, EPI_ISL_2590586, EPI_ISL_2590588, EPI_ISL_2590590, EPI_ISL_2590592, EPI_ISL_2590594, EPI_ISL_2590596, EPI_ISL_2590598, EPI_ISL_2590600, EPI_ISL_2590602, EPI_ISL_2590603, EPI_ISL_2590605, EPI_ISL_2590607, EPI_ISL_2590610, EPI_ISL_2590612, EPI_ISL_2590614, EPI_ISL_2590616, EPI_ISL_2590618, EPI_ISL_2590620, EPI_ISL_2590622, EPI_ISL_2590623, EPI_ISL_2590626, EPI_ISL_2590628, EPI_ISL_2590630, EPI_ISL_2590631, EPI_ISL_2590633, EPI_ISL_2590635, EPI_ISL_2590637, EPI_ISL_2590639, EPI_ISL_2590641, EPI_ISL_2590643, EPI_ISL_2590645, EPI_ISL_2590647, EPI_ISL_2590649, EPI_ISL_2590651, EPI_ISL_2590653, EPI_ISL_2590654, EPI_ISL_2590656, EPI_ISL_2590658, EPI_ISL_2590660, EPI_ISL_2590662, EPI_ISL_2590663, EPI_ISL_2590666, EPI_ISL_2590668, EPI_ISL_2590670, EPI_ISL_2590672, EPI_ISL_2590674, EPI_ISL_2590675, EPI_ISL_2590677, EPI_ISL_2590679, EPI_ISL_2590681			
see above	Division of Viral Infections, National Reference Center for Influenza, Robert Koch Institute	Division of Viral Infections, National Reference Center for Influenza, Robert Koch Institute	Reiche,J., Zadow,I., Lehmann,H., Tietze,S., Hafemann,S., Kluge,P. and Schweiger,B.
EPI_ISL_2590683, EPI_ISL_2590685, EPI_ISL_2590687, EPI_ISL_2590689, EPI_ISL_2590691, EPI_ISL_2590693, EPI_ISL_2590695	Virology Laboratory, Dr. Ricardo Gutierrez Children Hospital	Virology Laboratory, Dr. Ricardo Gutierrez Children Hospital	Viegas,M. and Mistchenko,A.S.
EPI_ISL_2590696, EPI_ISL_2590698, EPI_ISL_2590700	Immunology and Microbiology, Rega Institute for Medical Research	Immunology and Microbiology, Rega Institute for Medical Research	Houspie,L., Lemey,P., Keyaerts,E., Reijmen,E., Vergote,V., Vankeerberghen,A., Vaeyens,F., De Beenhouwer,H. and Van Ranst,M.
EPI_ISL_2590702, EPI_ISL_2590704, EPI_ISL_2590706, EPI_ISL_2590708, EPI_ISL_2590710, EPI_ISL_2590712, EPI_ISL_2590714, EPI_ISL_2590716, EPI_ISL_2590718, EPI_ISL_2590720, EPI_ISL_2590722, EPI_ISL_2590723, EPI_ISL_2590725, EPI_ISL_2590727, EPI_ISL_2590729, EPI_ISL_2590731, EPI_ISL_2590733, EPI_ISL_2590734, EPI_ISL_2590736			
see above	Virology Laboratory, Dr. Ricardo Gutierrez Children Hospital	Virology Laboratory, Dr. Ricardo Gutierrez Children Hospital	Viegas,M. and Mistchenko,A.S.
EPI_ISL_2590738	Virology Laboratory, Dr. Ricardo Gutierrez Children Hospital	Virology Laboratory, Dr. Ricardo Gutierrez Children Hospital	Viegas,M., Goya,S. and Mistchenko,A.S.
EPI_ISL_2590740, EPI_ISL_2590742, EPI_ISL_2590744, EPI_ISL_2590746, EPI_ISL_2590748, EPI_ISL_2590749, EPI_ISL_2590751, EPI_ISL_2590754, EPI_ISL_2590756, EPI_ISL_2590758, EPI_ISL_2590760, EPI_ISL_2590762, EPI_ISL_2590764, EPI_ISL_2590766			
see above	Department of Infectious, Parasitic and Immunomediated Diseases, Istituto Superiore di Sanita'	Department of Infectious, Parasitic and Immunomediated Diseases, Istituto Superiore di Sanita'	Montieri,S., Puzelli,S., Ciccozzi,M., Calzoletti,L. and Di Martino,A.
EPI_ISL_2590768	Department of Microbiology & Immunology, Rega Institute for Medical Research, University of Leuven, Laboratory of Clinical and Epidemiological Virology	Department of Microbiology & Immunology, Rega Institute for Medical Research, University of Leuven, Laboratory of Clinical and Epidemiological Virology	Zlateva,K.T., Lemey,P., Vandamme,A.-M. and Van Ranst,M.
EPI_ISL_2590770	Department of Microbiology and Immunology, Laboratory of Clinical and Epidemiological Virology, Rega Institute for Medical Research	Department of Microbiology and Immunology, Laboratory of Clinical and Epidemiological Virology, Rega Institute for Medical Research	Zlateva,K.T., Vijgen,L., Dekeersmaeker,N., Naranjo,C. and Van Ranst,M.
EPI_ISL_2590772	Department of Microbiology & Immunology, Rega Institute for Medical Research, University of Leuven, Laboratory of Clinical and Epidemiological Virology	Department of Microbiology & Immunology, Rega Institute for Medical Research, University of Leuven, Laboratory of Clinical and Epidemiological Virology	Zlateva,K.T., Lemey,P., Vandamme,A.-M. and Van Ranst,M.
EPI_ISL_2590774	Department of Microbiology and Immunology, Laboratory of Clinical and Epidemiological Virology, Rega Institute for Medical Research	Department of Microbiology and Immunology, Laboratory of Clinical and Epidemiological Virology, Rega Institute for Medical Research	Zlateva,K.T., Vijgen,L., Dekeersmaeker,N., Naranjo,C. and Van Ranst,M.
EPI_ISL_2590776	Department of Microbiology & Immunology, Rega Institute for Medical Research, University of Leuven, Laboratory of Clinical and Epidemiological Virology	Department of Microbiology & Immunology, Rega Institute for Medical Research, University of Leuven, Laboratory of Clinical and Epidemiological Virology	Zlateva,K.T., Lemey,P., Vandamme,A.-M. and Van Ranst,M.
EPI_ISL_2590778	Department of Microbiology and Immunology, Laboratory of Clinical and Epidemiological Virology, Rega Institute for Medical Research	Department of Microbiology and Immunology, Laboratory of Clinical and Epidemiological Virology, Rega Institute for Medical Research	Zlateva,K.T., Vijgen,L., Dekeersmaeker,N., Naranjo,C. and Van Ranst,M.
EPI_ISL_2590779, EPI_ISL_2590781, EPI_ISL_2590783, EPI_ISL_2590785	Department of Microbiology & Immunology, Rega Institute for Medical Research, University of Leuven, Laboratory of Clinical and Epidemiological Virology	Department of Microbiology & Immunology, Rega Institute for Medical Research, University of Leuven, Laboratory of Clinical and Epidemiological Virology	Zlateva,K.T., Lemey,P., Vandamme,A.-M. and Van Ranst,M.
EPI_ISL_2590787, EPI_ISL_2590789	Department of Microbiology and Immunology, Laboratory of Clinical and Epidemiological Virology, Rega Institute for Medical Research	Department of Microbiology and Immunology, Laboratory of Clinical and Epidemiological Virology, Rega Institute for Medical Research	Zlateva,K.T., Vijgen,L., Dekeersmaeker,N., Naranjo,C. and Van Ranst,M.
EPI_ISL_2590791, EPI_ISL_2590793, EPI_ISL_2590794, EPI_ISL_2590795, EPI_ISL_2590796, EPI_ISL_2590797, EPI_ISL_2590798	Department of Microbiology & Immunology, Rega Institute for Medical Research, University of Leuven, Laboratory of Clinical and Epidemiological Virology	Department of Microbiology & Immunology, Rega Institute for Medical Research, University of Leuven, Laboratory of Clinical and Epidemiological Virology	Zlateva,K.T., Lemey,P., Vandamme,A.-M. and Van Ranst,M.
EPI_ISL_2590799, EPI_ISL_2590800, EPI_ISL_2590801	Department of Microbiology and Immunology, Laboratory of Clinical and Epidemiological Virology, Rega Institute for Medical Research	Department of Microbiology and Immunology, Laboratory of Clinical and Epidemiological Virology, Rega Institute for Medical Research	Zlateva,K.T., Vijgen,L., Dekeersmaeker,N., Naranjo,C. and Van Ranst,M.
EPI_ISL_2590802	Department of Microbiology & Immunology, Rega Institute for Medical Research, University of Leuven, Laboratory of Clinical and Epidemiological Virology	Department of Microbiology & Immunology, Rega Institute for Medical Research, University of Leuven, Laboratory of Clinical and Epidemiological Virology	Zlateva,K.T., Lemey,P., Vandamme,A.-M. and Van Ranst,M.
EPI_ISL_2590803	Department of Microbiology and Immunology, Laboratory of Clinical and Epidemiological Virology, Rega Institute for Medical Research	Department of Microbiology and Immunology, Laboratory of Clinical and Epidemiological Virology, Rega Institute for Medical Research	Zlateva,K.T., Vijgen,L., Dekeersmaeker,N., Naranjo,C. and Van Ranst,M.
EPI_ISL_2590804	Department of Microbiology & Immunology, Rega Institute for Medical Research, University of Leuven, Laboratory of Clinical and Epidemiological Virology	Department of Microbiology & Immunology, Rega Institute for Medical Research, University of Leuven, Laboratory of Clinical and Epidemiological Virology	Zlateva,K.T., Lemey,P., Vandamme,A.-M. and Van Ranst,M.
EPI_ISL_2590806, EPI_ISL_2590808, EPI_ISL_2590810, EPI_ISL_2590812,	Virology Laboratory, Dr. Ricardo Gutierrez Children Hospital	Virology Laboratory, Dr. Ricardo Gutierrez Children Hospital	Viegas,M. and Mistchenko,A.S.

EPI_ISL_2590814, EPI_ISL_2590816, EPI_ISL_2590818, EPI_ISL_2590820, EPI_ISL_2590822			
EPI_ISL_2590824	Immunology and Microbiology, Rega Institute for Medical Research	Immunology and Microbiology, Rega Institute for Medical Research	Houspie,L., Lemey,P., Keyaerts,E., Reijmen,E., Vergote,V., Vankeerberghen,A., Vaeyens,F., De Beenhouwer,H. and Van Ranst,M.
EPI_ISL_2590828	Institute for Virology, University Duesseldorf	Institute for Virology, University Duesseldorf	Adams,O., Werczmircorwski,J. and Hengel,H.
EPI_ISL_2590830, EPI_ISL_2590832	Immunology and Microbiology, Rega Institute for Medical Research	Immunology and Microbiology, Rega Institute for Medical Research	Houspie,L., Lemey,P., Keyaerts,E., Reijmen,E., Vergote,V., Vankeerberghen,A., Vaeyens,F., De Beenhouwer,H. and Van Ranst,M.
EPI_ISL_2590834	Virology, Facultad de Ciencias	Virology, Facultad de Ciencias	Moura,F., Blanc,A. and Frabasile,S.
EPI_ISL_2590836, EPI_ISL_2590838, EPI_ISL_2590840, EPI_ISL_2590842, EPI_ISL_2590844, EPI_ISL_2590846, EPI_ISL_2590847, EPI_ISL_2590849, EPI_ISL_2590850	Department of Infectious, Parasitic and Immunomediated Diseases, Istituto Superiore di Sanita'	Department of Infectious, Parasitic and Immunomediated Diseases, Istituto Superiore di Sanita'	Montieri,S., Puzelli,S., Ciccozzi,M., Calzoletti,L. and Di Martino,A.
EPI_ISL_2590852	Virology Laboratory, Dr. Ricardo Gutierrez Children Hospital	Virology Laboratory, Dr. Ricardo Gutierrez Children Hospital	Viegas,M., Goya,S. and Mistchenko,A.S.
EPI_ISL_2590854	Virology, Facultad de Ciencias	Virology, Facultad de Ciencias	Moura,F., Blanc,A. and Frabasile,S.
EPI_ISL_2590857, EPI_ISL_2590859	Cell Biology, University of Sao Paulo School of Medicine	Cell Biology, University of Sao Paulo School of Medicine	Proenca-Modena,J.L., Paula,F.E., Iwamoto,M.A., Ansarah-Sobrinho,C., Saranzo,A.M., Santos,A.E., Cintra,O.A.L. and Arruda,E.
EPI_ISL_2590861, EPI_ISL_2590862	Division of Viral Infections, National Reference Center for Influenza, Robert Koch Institute	Division of Viral Infections, National Reference Center for Influenza, Robert Koch Institute	Reiche,J., Zadow,I., Lehmann,H., Tietze,S., Hafemann,S., Kluge,P. and Schweiger,B.
EPI_ISL_2590864, EPI_ISL_2590866	Department of Microbiology and Immunology, Laboratory of Clinical and Epidemiological Virology, Rega Institute for Medical Research	Department of Microbiology and Immunology, Laboratory of Clinical and Epidemiological Virology, Rega Institute for Medical Research	Zlateva,K.T., Vijgen,L., Dekeersmaeker,N., Naranjo,C. and Van Ranst,M.
EPI_ISL_2590867	Department of Microbiology & Immunology, Rega Institute for Medical Research, University of Leuven, Laboratory of Clinical and Epidemiological Virology	Department of Microbiology & Immunology, Rega Institute for Medical Research, University of Leuven, Laboratory of Clinical and Epidemiological Virology	Zlateva,K.T., Lemey,P., Vandamme,A.-M. and Van Ranst,M.
EPI_ISL_2590869, EPI_ISL_2590871	Department of Microbiology and Immunology, Laboratory of Clinical and Epidemiological Virology, Rega Institute for Medical Research	Department of Microbiology and Immunology, Laboratory of Clinical and Epidemiological Virology, Rega Institute for Medical Research	Zlateva,K.T., Vijgen,L., Dekeersmaeker,N., Naranjo,C. and Van Ranst,M.
EPI_ISL_2590873	Department of Microbiology & Immunology, Rega Institute for Medical Research, University of Leuven, Laboratory of Clinical and Epidemiological Virology	Department of Microbiology & Immunology, Rega Institute for Medical Research, University of Leuven, Laboratory of Clinical and Epidemiological Virology	Zlateva,K.T., Lemey,P., Vandamme,A.-M. and Van Ranst,M.
EPI_ISL_2590875	Department of Microbiology and Immunology, Laboratory of Clinical and Epidemiological Virology, Rega Institute for Medical Research	Department of Microbiology and Immunology, Laboratory of Clinical and Epidemiological Virology, Rega Institute for Medical Research	Zlateva,K.T., Vijgen,L., Dekeersmaeker,N., Naranjo,C. and Van Ranst,M.
EPI_ISL_2590877	Department of Microbiology & Immunology, Rega Institute for Medical Research, University of Leuven, Laboratory of Clinical and Epidemiological Virology	Department of Microbiology & Immunology, Rega Institute for Medical Research, University of Leuven, Laboratory of Clinical and Epidemiological Virology	Zlateva,K.T., Lemey,P., Vandamme,A.-M. and Van Ranst,M.
EPI_ISL_2590879	Department of Microbiology and Immunology, Laboratory of Clinical and Epidemiological Virology, Rega Institute for Medical Research	Department of Microbiology and Immunology, Laboratory of Clinical and Epidemiological Virology, Rega Institute for Medical Research	Zlateva,K.T., Vijgen,L., Dekeersmaeker,N., Naranjo,C. and Van Ranst,M.
EPI_ISL_2590881	Department of Microbiology & Immunology, Rega Institute for Medical Research, University of Leuven, Laboratory of Clinical and Epidemiological Virology	Department of Microbiology & Immunology, Rega Institute for Medical Research, University of Leuven, Laboratory of Clinical and Epidemiological Virology	Zlateva,K.T., Lemey,P., Vandamme,A.-M. and Van Ranst,M.
EPI_ISL_2590883, EPI_ISL_2590885	Department of Microbiology and Immunology, Laboratory of Clinical and Epidemiological Virology, Rega Institute for Medical Research	Department of Microbiology and Immunology, Laboratory of Clinical and Epidemiological Virology, Rega Institute for Medical Research	Zlateva,K.T., Vijgen,L., Dekeersmaeker,N., Naranjo,C. and Van Ranst,M.
EPI_ISL_2590886	Department of Microbiology & Immunology, Rega Institute for Medical Research, University of Leuven, Laboratory of Clinical and Epidemiological Virology	Department of Microbiology & Immunology, Rega Institute for Medical Research, University of Leuven, Laboratory of Clinical and Epidemiological Virology	Zlateva,K.T., Lemey,P., Vandamme,A.-M. and Van Ranst,M.
EPI_ISL_2590888	Department of Microbiology and Immunology, Laboratory of Clinical and Epidemiological Virology, Rega Institute for Medical Research	Department of Microbiology and Immunology, Laboratory of Clinical and Epidemiological Virology, Rega Institute for Medical Research	Zlateva,K.T., Vijgen,L., Dekeersmaeker,N., Naranjo,C. and Van Ranst,M.
EPI_ISL_2590890, EPI_ISL_2590892	Department of Microbiology & Immunology, Rega Institute for Medical Research, University of Leuven, Laboratory of Clinical and Epidemiological Virology	Department of Microbiology & Immunology, Rega Institute for Medical Research, University of Leuven, Laboratory of Clinical and Epidemiological Virology	Zlateva,K.T., Lemey,P., Vandamme,A.-M. and Van Ranst,M.
EPI_ISL_2590894, EPI_ISL_2590896	Institute for Virology, University Duesseldorf	Institute for Virology, University Duesseldorf	Adams,O., Werczmircorwski,J. and Hengel,H.
EPI_ISL_2590898, EPI_ISL_2590900	Virology Laboratory, Dr. Ricardo Gutierrez Children Hospital	Virology Laboratory, Dr. Ricardo Gutierrez Children Hospital	Viegas,M. and Mistchenko,A.S.
EPI_ISL_2590902	Centre de Recherche en Infectiologie, Centre Hospitalier Universitaire de Quebec (Pavillon CHUL)	Centre de Recherche en Infectiologie, Centre Hospitalier Universitaire de Quebec (Pavillon CHUL)	Gilca,R., De Serres,G., Tremblay,M., Leblanc,E., Bergeron,M.G., Dery,P. and Boivin,G.
EPI_ISL_2590904, EPI_ISL_2590906	Division of Viral Infections, National Reference Center for Influenza, Robert Koch Institute	Division of Viral Infections, National Reference Center for Influenza, Robert Koch Institute	Reiche,J., Zadow,I., Lehmann,H., Tietze,S., Hafemann,S., Kluge,P. and Schweiger,B.
EPI_ISL_2590908, EPI_ISL_2590910	Virology Branch, Butantan Institute	Virology Branch, Butantan Institute	Botosso,V.F., Peret,T.C.T., Thomazelli,L.M., Stewien,K.E. and Durigon,E.L.
EPI_ISL_2590912, EPI_ISL_2590914	Division of Viral Infections, National Reference Center for Influenza, Robert Koch Institute	Division of Viral Infections, National Reference Center for Influenza, Robert Koch Institute	Reiche,J., Zadow,I., Lehmann,H., Tietze,S., Hafemann,S., Kluge,P. and Schweiger,B.
EPI_ISL_2590918, EPI_ISL_2590920	Department of Microbiology and Immunology, Laboratory of Clinical and Epidemiological Virology, Rega Institute for Medical Research	Department of Microbiology and Immunology, Laboratory of Clinical and Epidemiological Virology, Rega Institute for Medical Research	Zlateva,K.T., Vijgen,L., Dekeersmaeker,N., Naranjo,C. and Van Ranst,M.
EPI_ISL_2590922, EPI_ISL_2590923, EPI_ISL_2590926	Department of Infectious, Parasitic and Immunomediated Diseases, Istituto Superiore di Sanita'	Department of Infectious, Parasitic and Immunomediated Diseases, Istituto Superiore di Sanita'	Montieri,S., Puzelli,S., Ciccozzi,M., Calzoletti,L. and Di Martino,A.
EPI_ISL_2590928	Department of Microbiology & Immunology, Rega Institute for Medical Research, University of Leuven, Laboratory of Clinical and Epidemiological Virology	Department of Microbiology & Immunology, Rega Institute for Medical Research, University of Leuven, Laboratory of Clinical and Epidemiological Virology	Zlateva,K.T., Lemey,P., Vandamme,A.-M. and Van Ranst,M.
EPI_ISL_2590929	Department of Microbiology and Immunology, Laboratory of Clinical and Epidemiological Virology, Rega Institute for Medical Research	Department of Microbiology and Immunology, Laboratory of Clinical and Epidemiological Virology, Rega Institute for Medical Research	Zlateva,K.T., Vijgen,L., Dekeersmaeker,N., Naranjo,C. and Van Ranst,M.
EPI_ISL_2590931, EPI_ISL_2590933, EPI_ISL_2590935, EPI_ISL_2590937, EPI_ISL_2590939	Department of Microbiology & Immunology, Rega Institute for Medical Research, University of Leuven, Laboratory of Clinical and Epidemiological Virology	Department of Microbiology & Immunology, Rega Institute for Medical Research, University of Leuven, Laboratory of Clinical and Epidemiological Virology	Zlateva,K.T., Lemey,P., Vandamme,A.-M. and Van Ranst,M.
EPI_ISL_2590941	Virology, Facultad de Ciencias	Virology, Facultad de Ciencias	Moura,F., Blanc,A. and Frabasile,S.
EPI_ISL_2590943	Lab of Emerging Viruses, Institute Pasteur of Shanghai, Chinese Academy of Sciences	Lab of Emerging Viruses, Institute Pasteur of Shanghai, Chinese Academy of Sciences	Wang,W., Sheng,J., Ren,P., Zhang,J., Lin,X., Hou,L. and Deubel,V.
EPI_ISL_2590945, EPI_ISL_2590947, EPI_ISL_2590949, EPI_ISL_2590951	Virology Laboratory, Dr. Ricardo Gutierrez Children Hospital	Virology Laboratory, Dr. Ricardo Gutierrez Children Hospital	Viegas,M. and Mistchenko,A.S.
EPI_ISL_2590955, EPI_ISL_2590957	Centre de Recherche en Infectiologie, Centre Hospitalier Universitaire de Quebec (Pavillon CHUL)	Centre de Recherche en Infectiologie, Centre Hospitalier Universitaire de Quebec (Pavillon CHUL)	Gilca,R., De Serres,G., Tremblay,M., Leblanc,E., Bergeron,M.G., Dery,P. and Boivin,G.
EPI_ISL_2590959, EPI_ISL_2590961, EPI_ISL_2590963, EPI_ISL_2590965, EPI_ISL_2590967	Department of Infectious, Parasitic and Immunomediated Diseases, Istituto Superiore di Sanita'	Department of Infectious, Parasitic and Immunomediated Diseases, Istituto Superiore di Sanita'	Montieri,S., Puzelli,S., Ciccozzi,M., Calzoletti,L. and Di Martino,A.
EPI_ISL_2590968	Department of Microbiology and Immunology, Laboratory of Clinical and Epidemiological Virology, Rega Institute for Medical Research	Department of Microbiology and Immunology, Laboratory of Clinical and Epidemiological Virology, Rega Institute for Medical Research	Zlateva,K.T., Vijgen,L., Dekeersmaeker,N., Naranjo,C. and Van Ranst,M.
EPI_ISL_2590970	Department of Microbiology & Immunology, Rega Institute for Medical Research, University of Leuven, Laboratory of Clinical and Epidemiological Virology	Department of Microbiology & Immunology, Rega Institute for Medical Research, University of Leuven, Laboratory of Clinical and Epidemiological Virology	Zlateva,K.T., Lemey,P., Vandamme,A.-M. and Van Ranst,M.
EPI_ISL_2590972	Department of Microbiology and Immunology, Laboratory of Clinical and Epidemiological Virology, Rega Institute for Medical Research	Department of Microbiology and Immunology, Laboratory of Clinical and Epidemiological Virology, Rega Institute for Medical Research	Zlateva,K.T., Vijgen,L., Dekeersmaeker,N., Naranjo,C. and Van Ranst,M.
EPI_ISL_2590974	Virology Laboratory, Dr. Ricardo Gutierrez Children Hospital	Virology Laboratory, Dr. Ricardo Gutierrez Children Hospital	Viegas,M. and Mistchenko,A.S.
EPI_ISL_2590975	Cell Biology, University of Sao Paulo School of Medicine	Cell Biology, University of Sao Paulo School of Medicine	Proenca-Modena,J.L., Paula,F.E., Iwamoto,M.A., Ansarah-Sobrinho,C., Saranzo,A.M., Santos,A.E., Cintra,O.A.L. and Arruda,E.
EPI_ISL_2590977, EPI_ISL_2590979	Virology, Facultad de Ciencias	Virology, Facultad de Ciencias	Moura,F., Blanc,A. and Frabasile,S.
EPI_ISL_2590981, EPI_ISL_2590983, EPI_ISL_2590985, EPI_ISL_2590988	Virology Branch, Butantan Institute	Virology Branch, Butantan Institute	Botosso,V.F., Peret,T.C.T., Thomazelli,L.M., Stewien,K.E. and Durigon,E.L.
EPI_ISL_2590989	Department of Microbiology, Institute of Biomedical Science	Department of Microbiology, Institute of Biomedical Science	Moraes,C.T.P., Botosso,V.F., Sacramento,P., Comone,P., Souza,T.S., Oliveira,D.B.L., Stewien,K.E., Sant'Anna,O.A.B.E. and Durigon,E.L.
EPI_ISL_2590991, EPI_ISL_2590997, EPI_ISL_2590998, EPI_ISL_2591000, EPI_ISL_2591002	Department of Microbiology & Immunology, Rega Institute for Medical Research, University of Leuven, Laboratory of Clinical and Epidemiological Virology	Department of Microbiology & Immunology, Rega Institute for Medical Research, University of Leuven, Laboratory of Clinical and Epidemiological Virology	Zlateva,K.T., Lemey,P., Vandamme,A.-M. and Van Ranst,M.
EPI_ISL_2591004	Virology Branch, Butantan Institute	Virology Branch, Butantan Institute	Botosso,V.F., Peret,T.C.T., Thomazelli,L.M., Stewien,K.E. and Durigon,E.L.
EPI_ISL_2591006, EPI_ISL_2591008	Cell Biology, University of Sao Paulo School of Medicine	Cell Biology, University of Sao Paulo School of Medicine	Proenca-Modena,J.L., Paula,F.E., Iwamoto,M.A., Ansarah-Sobrinho,C., Saranzo,A.M., Santos,A.E., Cintra,O.A.L. and Arruda,E.
EPI_ISL_2591010	Centre de Recherche en Infectiologie, Centre Hospitalier Universitaire de Quebec (Pavillon CHUL)	Centre de Recherche en Infectiologie, Centre Hospitalier Universitaire de Quebec (Pavillon CHUL)	Gilca,R., De Serres,G., Tremblay,M., Leblanc,E., Bergeron,M.G., Dery,P. and Boivin,G.
EPI_ISL_2591012	Department of Microbiology & Immunology, Rega Institute for Medical Research, University of Leuven, Laboratory of Clinical and Epidemiological Virology	Department of Microbiology & Immunology, Rega Institute for Medical Research, University of Leuven, Laboratory of Clinical and Epidemiological Virology	Zlateva,K.T., Lemey,P., Vandamme,A.-M. and Van Ranst,M.
EPI_ISL_2591014	Department of Microbiology and Immunolgy, Institute of Biology, State University of Campinas (UNICAMP)	Department of Microbiology and Immunolgy, Institute of Biology, State University of Campinas (UNICAMP)	da Silva,L.H.A., Spilki,F.R., Riccetto,A.G.L., de Almeida,R.S., Baracat,E.C.E. and Arns,C.W.
EPI_ISL_2591018, EPI_ISL_2591020, EPI_ISL_2591021	Department of Microbiology & Immunology, Rega Institute for Medical Research, University of Leuven, Laboratory of Clinical and Epidemiological Virology	Department of Microbiology & Immunology, Rega Institute for Medical Research, University of Leuven, Laboratory of Clinical and Epidemiological Virology	Zlateva,K.T., Lemey,P., Vandamme,A.-M. and Van Ranst,M.

EPI_ISL_2591024, EPI_ISL_2591025, EPI_ISL_2591028, EPI_ISL_2591029, EPI_ISL_2591031, EPI_ISL_2591033	Virology Branch, Butantan Institute	Virology Branch, Butantan Institute	Botosso,V.F., Peret,T.C.T., Thomazelli,L.M., Stewien,K.E. and Durigon,E.L.
EPI_ISL_2591035	Division of Viral Infections, National Reference Center for Influenza, Robert Koch Institute	Division of Viral Infections, National Reference Center for Influenza, Robert Koch Institute	Reiche,J., Zadow,I., Lehmann,H., Tietze,S., Hafemann,S., Kluge,P. and Schweiger,B.
EPI_ISL_2591037	Virology Branch, Butantan Institute	Virology Branch, Butantan Institute	Botosso,V.F., Peret,T.C.T., Thomazelli,L.M., Stewien,K.E. and Durigon,E.L.
EPI_ISL_2591039	Division of Viral Infections, National Reference Center for Influenza, Robert Koch Institute	Division of Viral Infections, National Reference Center for Influenza, Robert Koch Institute	Reiche,J., Zadow,I., Lehmann,H., Tietze,S., Hafemann,S., Kluge,P. and Schweiger,B.
EPI_ISL_2591041, EPI_ISL_2591043	Tsuguto Fujimoto National Institute of Infectious Diseases, Infectious Disease Surveillance Center	Tsuguto Fujimoto National Institute of Infectious Diseases, Infectious Disease Surveillance Center	Fujimoto,T., Hanaoka,N., Nakamura,M. and Kawabata,M.
EPI_ISL_2591045	Lab of Emerging Viruses, Institute Pasteur of Shanghai, Chinese Academy of Sciences	Lab of Emerging Viruses, Institute Pasteur of Shanghai, Chinese Academy of Sciences	Wang,W., Sheng,J., Ren,P., Zhang,J., Lin,X., Hou,L. and Deubel,V.
EPI_ISL_2591051	Centre de Recherche en Infectiologie, Centre Hospitalier Universitaire de Quebec (Pavillon CHUL)	Centre de Recherche en Infectiologie, Centre Hospitalier Universitaire de Quebec (Pavillon CHUL)	Gilca,R., De Serres,G., Tremblay,M., Leblanc,E., Bergeron,M.G., Dery,P. and Boivin,G.
EPI_ISL_2591053, EPI_ISL_2591055, EPI_ISL_2591057, EPI_ISL_2591059	Department of Microbiology and Immunology, Laboratory of Clinical and Epidemiological Virology, Rega Institute for Medical Research	Department of Microbiology and Immunology, Laboratory of Clinical and Epidemiological Virology, Rega Institute for Medical Research	Zlateva,K.T., Vijgen,L., Dekeersmaeker,N., Naranjo,C. and Van Ranst,M.
EPI_ISL_2591061	Virology Unit, Institut Pasteur in Cambodia	Virology Unit, Institut Pasteur in Cambodia	Arnott,A., Vong,S., Sek,M., Chu,S., Naughtin,M., Sovann,L., Buecher,C., Beaute,J., Rith,S., Borand,L., Asgari,N., Guillard,B., Touch,S., Deubel,V. and Buchy,P.
EPI_ISL_2591063	Department of Microbiology & Immunology, Rega Institute for Medical Research, University of Leuven, Laboratory of Clinical and Epidemiological Virology	Department of Microbiology & Immunology, Rega Institute for Medical Research, University of Leuven, Laboratory of Clinical and Epidemiological Virology	Zlateva,K.T., Lemey,P., Vandamme,A.-M. and Van Ranst,M.
EPI_ISL_2591065	Virology Unit, Institut Pasteur in Cambodia	Virology Unit, Institut Pasteur in Cambodia	Arnott,A., Vong,S., Sek,M., Chu,S., Naughtin,M., Sovann,L., Buecher,C., Beaute,J., Rith,S., Borand,L., Asgari,N., Guillard,B., Touch,S., Deubel,V. and Buchy,P.
EPI_ISL_2591067	Virology, NAMRU-56	Virology, NAMRU-56	Garcia,J., Sovero,M. and Halsey,E.S.
EPI_ISL_2591069, EPI_ISL_2591071, EPI_ISL_2591073, EPI_ISL_2591076, EPI_ISL_2591077, EPI_ISL_2591079	Department of Microbiology & Immunology, Rega Institute for Medical Research, University of Leuven, Laboratory of Clinical and Epidemiological Virology	Department of Microbiology & Immunology, Rega Institute for Medical Research, University of Leuven, Laboratory of Clinical and Epidemiological Virology	Zlateva,K.T., Lemey,P., Vandamme,A.-M. and Van Ranst,M.
EPI_ISL_2591081, EPI_ISL_2591083	Department of Infectious, Parasitic and Immunomediated Diseases, Istituto Superiore di Sanita'	Department of Infectious, Parasitic and Immunomediated Diseases, Istituto Superiore di Sanita'	Montieri,S., Puzelli,S., Ciccozzi,M., Calzoletti,L. and Di Martino,A.
EPI_ISL_2591088, EPI_ISL_2591089	Lab of Emerging Viruses, Institute Pasteur of Shanghai, Chinese Academy of Sciences	Lab of Emerging Viruses, Institute Pasteur of Shanghai, Chinese Academy of Sciences	Wang,W., Sheng,J., Ren,P., Zhang,J., Lin,X., Hou,L. and Deubel,V.
EPI_ISL_2591092	Virology, Institut Pasteur in Cambodia	Virology, Institut Pasteur in Cambodia	Chu,S., Buecher,C. and Buchy,P.
EPI_ISL_2591095	Cell Biology, University of Sao Paulo School of Medicine	Cell Biology, University of Sao Paulo School of Medicine	Proenca-Modena,J.L., Paula,F.E., Iwamoto,M.A., Ansarah-Sobrinho,C., Saranzo,A.M., Santos,A.E., Cintra,O.A.L. and Arruda,E.
EPI_ISL_2591097	Department of Microbiology, University of Sao Paulo Institute of Biomedical Science	Department of Microbiology, University of Sao Paulo Institute of Biomedical Science	Lima,H.N., Oliveira,D.B.L., Botosso,V.F., Stewien,K.E. and Durigon,E.L.
EPI_ISL_2591099	Microbiology, Adolfo Lutz Institut	Microbiology, Adolfo Lutz Institut	Souza,M.C.O., Matsumoto,T.K., Vaz-De-Lima,L.L.R.A., Sato,N.N., Salgado,M.M., Requejo,H.I.Z., Hong,M.A., Barbosa,M.L., Oliveira,C.A.F., Benelli,P.C., Shimokawa,R.Y., Pecchini,R., Berezin,E., Duarte,S.P., Schvartsman,C., Durigon,E.L. and Ueda,M.
EPI_ISL_2591101	Centre de Recherche en Infectiologie, Centre Hospitalier Universitaire de Quebec (Pavillon CHUL)	Centre de Recherche en Infectiologie, Centre Hospitalier Universitaire de Quebec (Pavillon CHUL)	Gilca,R., De Serres,G., Tremblay,M., Leblanc,E., Bergeron,M.G., Dery,P. and Boivin,G.
EPI_ISL_2591103	Division of Viral Infections, National Reference Center for Influenza, Robert Koch Institute	Division of Viral Infections, National Reference Center for Influenza, Robert Koch Institute	Reiche,J., Zadow,I., Lehmann,H., Tietze,S., Hafemann,S., Kluge,P. and Schweiger,B.
EPI_ISL_2591107	Microbiology, Adolfo Lutz Institut	Microbiology, Adolfo Lutz Institut	Souza,M.C.O., Matsumoto,T.K., Vaz-De-Lima,L.L.R.A., Sato,N.N., Salgado,M.M., Requejo,H.I.Z., Hong,M.A., Barbosa,M.L., Oliveira,C.A.F., Benelli,P.C., Shimokawa,R.Y., Pecchini,R., Berezin,E., Duarte,S.P., Schvartsman,C., Durigon,E.L. and Ueda,M.
EPI_ISL_2591111, EPI_ISL_2591113, EPI_ISL_2591115	Division of Viral Infections, National Reference Center for Influenza, Robert Koch Institute	Division of Viral Infections, National Reference Center for Influenza, Robert Koch Institute	Reiche,J., Zadow,I., Lehmann,H., Tietze,S., Hafemann,S., Kluge,P. and Schweiger,B.
EPI_ISL_2591121, EPI_ISL_2591123, EPI_ISL_2591125, EPI_ISL_2591127, EPI_ISL_2591129, EPI_ISL_2591131	Department of Microbiology & Immunology, Rega Institute for Medical Research, University of Leuven, Laboratory of Clinical and Epidemiological Virology	Department of Microbiology & Immunology, Rega Institute for Medical Research, University of Leuven, Laboratory of Clinical and Epidemiological Virology	Zlateva,K.T., Lemey,P., Vandamme,A.-M. and Van Ranst,M.
EPI_ISL_2591133	Departament of Microbiology, University of Sao Paulo Institute of Biomedical Science	Departament of Microbiology, University of Sao Paulo Institute of Biomedical Science	Lima,H.N., Oliveira,D.B.L., Durigon,E.L. and Stewien,K.E.
EPI_ISL_2591136, EPI_ISL_2591138, EPI_ISL_2591140, EPI_ISL_2591142, EPI_ISL_2591144, EPI_ISL_2591146, EPI_ISL_2591148, EPI_ISL_2591150, EPI_ISL_2591152	Department of Microbiology & Immunology, Rega Institute for Medical Research, University of Leuven, Laboratory of Clinical and Epidemiological Virology	Department of Microbiology & Immunology, Rega Institute for Medical Research, University of Leuven, Laboratory of Clinical and Epidemiological Virology	Zlateva,K.T., Lemey,P., Vandamme,A.-M. and Van Ranst,M.
EPI_ISL_2591154, EPI_ISL_2591156, EPI_ISL_2591157	Lab of Emerging Viruses, Institute Pasteur of Shanghai, Chinese Academy of Sciences	Lab of Emerging Viruses, Institute Pasteur of Shanghai, Chinese Academy of Sciences	Wang,W., Sheng,J., Ren,P., Zhang,J., Lin,X., Hou,L. and Deubel,V.
EPI_ISL_2591170	Virology, NAMRU-39	Virology, NAMRU-39	Garcia,J., Sovero,M. and Halsey,E.S.
EPI_ISL_2591172, EPI_ISL_2591174, EPI_ISL_2591176, EPI_ISL_2591178	Virology Branch, Butantan Institute	Virology Branch, Butantan Institute	Botosso,V.F., Peret,T.C.T., Thomazelli,L.M., Stewien,K.E. and Durigon,E.L.
EPI_ISL_2591180	Department of Microbiology, Institute of Biomedical Science, University of Sao Paulo	Department of Microbiology, Institute of Biomedical Science, University of Sao Paulo	Campos,A.C.A., Botosso,V.F., Souza,T.S., Moraes,C.T.P., Oliveira,D.B.L., Sant'Anna,O.A.B.E., Stewien,K.E. and Durigon,E.L.
EPI_ISL_2591182, EPI_ISL_2591184	Division of Viral Infections, National Reference Center for Influenza, Robert Koch Institute	Division of Viral Infections, National Reference Center for Influenza, Robert Koch Institute	Reiche,J., Zadow,I., Lehmann,H., Tietze,S., Hafemann,S., Kluge,P. and Schweiger,B.
EPI_ISL_2591199	Influenza Group, National Institute of Virology	Influenza Group, National Institute of Virology	Damle,R.G. and Chadha,M.S.
EPI_ISL_2591204	Virology, NAMRU-46	Virology, NAMRU-46	Garcia,J., Sovero,M. and Halsey,E.S.
EPI_ISL_2591205	Virology, NAMRU-42	Virology, NAMRU-42	Garcia,J., Sovero,M. and Halsey,E.S.
EPI_ISL_2591214	Department of Microbiology and Immunolgy, Institute of Biology, State University of Campinas (UNICAMP)	Department of Microbiology and Immunolgy, Institute of Biology, State University of Campinas (UNICAMP)	da Silva,L.H.A., Spilki,F.R., Riccetto,A.G.L., de Almeida,R.S., Baracat,E.C.E. and Arns,C.W.
EPI_ISL_2591216, EPI_ISL_2591218	Centre de Recherche en Infectiologie, Centre Hospitalier Universitaire de Quebec (Pavillon CHUL)	Centre de Recherche en Infectiologie, Centre Hospitalier Universitaire de Quebec (Pavillon CHUL)	Gilca,R., De Serres,G., Tremblay,M., Leblanc,E., Bergeron,M.G., Dery,P. and Boivin,G.
EPI_ISL_2591221	Virology Branch, Butantan Institute	Virology Branch, Butantan Institute	Botosso,V.F., Peret,T.C.T., Thomazelli,L.M., Stewien,K.E. and Durigon,E.L.
EPI_ISL_2591224	Department of Microbiology and Immunolgy, Institute of Biology, State University of Campinas (UNICAMP)	Department of Microbiology and Immunolgy, Institute of Biology, State University of Campinas (UNICAMP)	da Silva,L.H.A., Spilki,F.R., Riccetto,A.G.L., de Almeida,R.S., Baracat,E.C.E. and Arns,C.W.
EPI_ISL_2591226	Virology Branch, Butantan Institute	Virology Branch, Butantan Institute	Botosso,V.F., Peret,T.C.T., Thomazelli,L.M., Stewien,K.E. and Durigon,E.L.
EPI_ISL_2591228	Department of Microbiology, Institute of Biomedical Science, University of Sao Paulo	Department of Microbiology, Institute of Biomedical Science, University of Sao Paulo	Campos,A.C.A., Botosso,V.F., Souza,T.S., Moraes,C.T.P., Oliveira,D.B.L., Sant'Anna,O.A.B.E., Stewien,K.E. and Durigon,E.L.
EPI_ISL_2591229, EPI_ISL_2591231	Virology Branch, Butantan Institute	Virology Branch, Butantan Institute	Botosso,V.F., Peret,T.C.T., Thomazelli,L.M., Stewien,K.E. and Durigon,E.L.
EPI_ISL_2591233, EPI_ISL_2591235	Research & Development, Institute of Immunology	Research & Development, Institute of Immunology	Ivancic-Jelecki,J. and Forcic,D.
EPI_ISL_2591243, EPI_ISL_2591245	Clinical Microbiology, Trinity College Dublin	Clinical Microbiology, Trinity College Dublin	Salter,A.C., Crowley,B. and Ni Laoi,B.
EPI_ISL_2591246	Institute for Virology, University Duesseldorf	Institute for Virology, University Duesseldorf	Adams,O., Werczmircorwski,J. and Hengel,H.
EPI_ISL_2591249	Virology Branch, Butantan Institute	Virology Branch, Butantan Institute	Botosso,V.F., Peret,T.C.T., Thomazelli,L.M., Stewien,K.E. and Durigon,E.L.
EPI_ISL_2591250	Virology Branch, Butantan Institute	Virology Branch, Butantan Institute	Botosso,V.F., Sacramento,P., Comone,P., Oliveira,D.B.L., Souza,T.S., Moraes,C.T.P., Stewien,K.E., Sant'Anna,O.A.B.E. and Durigon,E.L.
EPI_ISL_2591251, EPI_ISL_2591252	Virology Branch, Butantan Institute	Virology Branch, Butantan Institute	Botosso,V.F., Peret,T.C.T., Thomazelli,L.M., Stewien,K.E. and Durigon,E.L.
EPI_ISL_2591253	Virology Branch, Butantan Institute	Virology Branch, Butantan Institute	Botosso,V.F., Sacramento,P., Comone,P., Oliveira,D.B.L., Souza,T.S., Moraes,C.T.P., Stewien,K.E., Sant'Anna,O.A.B.E. and Durigon,E.L.
EPI_ISL_2591254, EPI_ISL_2591255, EPI_ISL_2591256, EPI_ISL_2591257, EPI_ISL_2591258, EPI_ISL_2591259, EPI_ISL_2591260, EPI_ISL_2591262, EPI_ISL_2591263, EPI_ISL_2591265, EPI_ISL_2591267, EPI_ISL_2591269, EPI_ISL_2591271, EPI_ISL_2591273, EPI_ISL_2591275			
see above	Division of Viral Infections, National Reference Center for Influenza, Robert Koch Institute	Division of Viral Infections, National Reference Center for Influenza, Robert Koch Institute	Reiche,J., Zadow,I., Lehmann,H., Tietze,S., Hafemann,S., Kluge,P. and Schweiger,B.
EPI_ISL_2591278	Influenza Group, National Institute of Virology	Influenza Group, National Institute of Virology	Damle,R.G. and Chadha,M.S.
EPI_ISL_2591280, EPI_ISL_2591282	Clinical Microbiology, Trinity College Dublin	Clinical Microbiology, Trinity College Dublin	Salter,A.C., Crowley,B. and Ni Laoi,B.
EPI_ISL_2591286	Division of Viral Infections, National Reference Center for Influenza, Robert Koch Institute	Division of Viral Infections, National Reference Center for Influenza, Robert Koch Institute	Reiche,J., Zadow,I., Lehmann,H., Tietze,S., Hafemann,S., Kluge,P. and Schweiger,B.
EPI_ISL_2591288, EPI_ISL_2591290, EPI_ISL_2591292, EPI_ISL_2591294, EPI_ISL_2591296, EPI_ISL_2591298, EPI_ISL_2591300, EPI_ISL_2591302, EPI_ISL_2591304	Virology Branch, Butantan Institute	Virology Branch, Butantan Institute	Botosso,V.F., Peret,T.C.T., Thomazelli,L.M., Stewien,K.E. and Durigon,E.L.
EPI_ISL_2591306	Virology Branch, Butantan Institute	Virology Branch, Butantan Institute	Botosso,V.F., Sacramento,P., Comone,P., Oliveira,D.B.L., Souza,T.S., Moraes,C.T.P., Stewien,K.E., Sant'Anna,O.A.B.E. and Durigon,E.L.
EPI_ISL_2591308, EPI_ISL_2591310, EPI_ISL_2591312, EPI_ISL_2591313, EPI_ISL_2591316, EPI_ISL_2591317, EPI_ISL_2591319, EPI_ISL_2591321, EPI_ISL_2591323, EPI_ISL_2591325, EPI_ISL_2591327			
see above	Virology Branch, Butantan Institute	Virology Branch, Butantan Institute	Botosso,V.F., Peret,T.C.T., Thomazelli,L.M., Stewien,K.E. and Durigon,E.L.

EPI_ISL_2591328	Department of Microbiology, Institute of Biomedical Science, University of Sao Paulo	Department of Microbiology, Institute of Biomedical Science, University of Sao Paulo	Campos,A.C.A., Botosso,V.F., Souza,T.S., Moraes,C.T.P., Oliveira,D.B.L., Sant'Anna,O.A.B.E., Stewien,K.E. and Durigon,E.L.
EPI_ISL_2591330, EPI_ISL_2591332, EPI_ISL_2591334, EPI_ISL_2591336, EPI_ISL_2591338, EPI_ISL_2591340, EPI_ISL_2591341, EPI_ISL_2591343	Virology Branch, Butantan Institute	Virology Branch, Butantan Institute	Botosso,V.F., Peret,T.C.T., Thomazelli,L.M., Stewien,K.E. and Durigon,E.L.
EPI_ISL_2591345, EPI_ISL_2591347, EPI_ISL_2591349, EPI_ISL_2591351, EPI_ISL_2591353, EPI_ISL_2591355, EPI_ISL_2591357, EPI_ISL_2591359, EPI_ISL_2591361, EPI_ISL_2591362, EPI_ISL_2591364, EPI_ISL_2591366, EPI_ISL_2591368, EPI_ISL_2591370, EPI_ISL_2591372			
see above	Cell Biology, University of Sao Paulo School of Medicine	Cell Biology, University of Sao Paulo School of Medicine	Proenca-Modena,J.L., Paula,F.E., Iwamoto,M.A., Ansarah-Sobrinho,C., Saranzo,A.M., Santos,A.E., Cintra,O.A.L. and Arruda,E.
EPI_ISL_2591374	Centre de Recherche en Infectiologie, Centre Hospitalier Universitaire de Quebec (Pavillon CHUL)	Centre de Recherche en Infectiologie, Centre Hospitalier Universitaire de Quebec (Pavillon CHUL)	Gilca,R., De Serres,G., Tremblay,M., Leblanc,E., Bergeron,M.G., Dery,P. and Boivin,G.
EPI_ISL_2591376	Research & Development, Institute of Immunology	Research & Development, Institute of Immunology	Ivancic-Jelecki,J. and Forcic,D.
EPI_ISL_2591378, EPI_ISL_2591379, EPI_ISL_2591381, EPI_ISL_2591383, EPI_ISL_2591385, EPI_ISL_2591387, EPI_ISL_2591389, EPI_ISL_2591391, EPI_ISL_2591392, EPI_ISL_2591394, EPI_ISL_2591396, EPI_ISL_2591398, EPI_ISL_2591400, EPI_ISL_2591402, EPI_ISL_2591403, EPI_ISL_2591405, EPI_ISL_2591407, EPI_ISL_2591409, EPI_ISL_2591411, EPI_ISL_2591413, EPI_ISL_2591415, EPI_ISL_2591416, EPI_ISL_2591418, EPI_ISL_2591420, EPI_ISL_2591422, EPI_ISL_2591424			
see above	Division of Viral Infections, National Reference Center for Influenza, Robert Koch Institute	Division of Viral Infections, National Reference Center for Influenza, Robert Koch Institute	Reiche,J., Zadow,I., Lehmann,H., Tietze,S., Hafemann,S., Kluge,P. and Schweiger,B.
EPI_ISL_2591426, EPI_ISL_2591427	Influenza Group, National Institute of Virology	Influenza Group, National Institute of Virology	Damle,R.G. and Chadha,M.S.
EPI_ISL_2591429	Tsuguto Fujimoto National Institute of Infectious Diseases, Infectious Disease Surveillance Center	Tsuguto Fujimoto National Institute of Infectious Diseases, Infectious Disease Surveillance Center	Fujimoto,T., Hanaoka,N., Nakamura,M. and Kawabata,M.
EPI_ISL_2591431	Virology Laboratory, Dr. Ricardo Gutierrez Children Hospital	Virology Laboratory, Dr. Ricardo Gutierrez Children Hospital	Viegas,M. and Mistchenko,A.S.
EPI_ISL_2591433	Clinical Microbiology, Trinity College Dublin	Clinical Microbiology, Trinity College Dublin	Salter,A.C., Crowley,B. and Ni Laoi,B.
EPI_ISL_2591435	Department of Microbiology & Immunology, Rega Institute for Medical Research, University of Leuven, Laboratory of Clinical and Epidemiological Virology	Department of Microbiology & Immunology, Rega Institute for Medical Research, University of Leuven, Laboratory of Clinical and Epidemiological Virology	Zlateva,K.T., Lemey,P., Vandamme,A.-M. and Van Ranst,M.
EPI_ISL_2591443, EPI_ISL_2591445, EPI_ISL_2591447, EPI_ISL_2591449, EPI_ISL_2591450, EPI_ISL_2591452	Lab of Emerging Viruses, Institute Pasteur of Shanghai, Chinese Academy of Sciences	Lab of Emerging Viruses, Institute Pasteur of Shanghai, Chinese Academy of Sciences	Wang,W., Sheng,J., Ren,P., Zhang,J., Lin,X., Hou,L. and Deubel,V.
EPI_ISL_2591476, EPI_ISL_2591480, EPI_ISL_2591482, EPI_ISL_2591483	Miwako SaikNorth America / USA Yokohama City Institute of Public Health	Miwako SaikNorth America / USA Yokohama City Institute of Public Health	SaikNorth America / USA,M., Kawakami,C., Usuku,S., Sasao,T. and Okubo,I.
EPI_ISL_2591485, EPI_ISL_2591487, EPI_ISL_2591489	Departament of Microbiology, University of Sao Paulo Institute of Biomedical Science	Departament of Microbiology, University of Sao Paulo Institute of Biomedical Science	Lima,H.N., Oliveira,D.B.L., Durigon,E.L. and Stewien,K.E.
EPI_ISL_2591498	Virology, NAMRU-47	Virology, NAMRU-47	Garcia,J., Sovero,M. and Halsey,E.S.
EPI_ISL_2591500	Virology, NAMRU-65	Virology, NAMRU-65	Garcia,J., Sovero,M. and Halsey,E.S.
EPI_ISL_2591504	Virology, NAMRU-44	Virology, NAMRU-44	Garcia,J., Sovero,M. and Halsey,E.S.
EPI_ISL_2591510	Molecular Virology Unit, Virology and Microbiology Department, Fondazione IRCCS Policlinico San Matteo	Molecular Virology Unit, Virology and Microbiology Department, Fondazione IRCCS Policlinico San Matteo	Piralla,A., Percivalle,E. and Baldanti,F.
EPI_ISL_2591511, EPI_ISL_2591513, EPI_ISL_2591515, EPI_ISL_2591517, EPI_ISL_2591519, EPI_ISL_2591521, EPI_ISL_2591552, EPI_ISL_2591554, EPI_ISL_2591555, EPI_ISL_2591557, EPI_ISL_2591560, EPI_ISL_2591562, EPI_ISL_2591564, EPI_ISL_2591565, EPI_ISL_2591567, EPI_ISL_2591569, EPI_ISL_2591571, EPI_ISL_2591573, EPI_ISL_2591574, EPI_ISL_2591576, EPI_ISL_2591578, EPI_ISL_2591579, EPI_ISL_2591581, EPI_ISL_2591583, EPI_ISL_2591585, EPI_ISL_2591587, EPI_ISL_2591589, EPI_ISL_2591591, EPI_ISL_2591592, EPI_ISL_2591593, EPI_ISL_2591594, EPI_ISL_2591595, EPI_ISL_2591597, EPI_ISL_2591599, EPI_ISL_2591600, EPI_ISL_2591602, EPI_ISL_2591605, EPI_ISL_2591606, EPI_ISL_2591608, EPI_ISL_2591610, EPI_ISL_2591612, EPI_ISL_2591614, EPI_ISL_2591616, EPI_ISL_2591617, EPI_ISL_2591619, EPI_ISL_2591621, EPI_ISL_2591623			
see above	Miwako SaikNorth America / USA Yokohama City Institute of Public Health	Miwako SaikNorth America / USA Yokohama City Institute of Public Health	SaikNorth America / USA,M., Kawakami,C., Usuku,S., Sasao,T. and Okubo,I.
EPI_ISL_2591635, EPI_ISL_2591637, EPI_ISL_2591639, EPI_ISL_2591641, EPI_ISL_2591643	Virology, Institut Pasteur in Cambodia	Virology, Institut Pasteur in Cambodia	Chu,S., Buecher,C. and Buchy,P.
EPI_ISL_2591645, EPI_ISL_2591647, EPI_ISL_2591649, EPI_ISL_2591651, EPI_ISL_2591653, EPI_ISL_2591656, EPI_ISL_2591658, EPI_ISL_2591660, EPI_ISL_2591662, EPI_ISL_2591666, EPI_ISL_2591669			
see above	Influenza Group, National Institute of Virology	Influenza Group, National Institute of Virology	Damle,R.G. and Chadha,M.S.
EPI_ISL_2591671, EPI_ISL_2591673, EPI_ISL_2591675, EPI_ISL_2591676, EPI_ISL_2591678, EPI_ISL_2591680, EPI_ISL_2591682, EPI_ISL_2591684, EPI_ISL_2591686	Miwako SaikNorth America / USA Yokohama City Institute of Public Health	Miwako SaikNorth America / USA Yokohama City Institute of Public Health	SaikNorth America / USA,M., Kawakami,C., Usuku,S., Sasao,T. and Okubo,I.
EPI_ISL_2591688	Virology, NAMRU-63	Virology, NAMRU-63	Garcia,J., Sovero,M. and Halsey,E.S.
EPI_ISL_2591689	Virology, NAMRU-50	Virology, NAMRU-50	Garcia,J., Sovero,M. and Halsey,E.S.
EPI_ISL_2591690	Centre de Recherche en Infectiologie, Universite Laval, CHUL	Centre de Recherche en Infectiologie, Universite Laval, CHUL	Papenburg,J., Carbonneau,J., Hamelin,M.-E., Isabel,S., Ohoumanne,N., Dery,P., Paes,B.A., Corbeil,J., Bergeron,M.G., De Serres,G. and Boivin,G.
EPI_ISL_2591701, EPI_ISL_2591702, EPI_ISL_2591703, EPI_ISL_2591704, EPI_ISL_2591705, EPI_ISL_2591706, EPI_ISL_2591707, EPI_ISL_2591708, EPI_ISL_2591709, EPI_ISL_2591710, EPI_ISL_2591711, EPI_ISL_2591712, EPI_ISL_2591713, EPI_ISL_2591714			
see above	Lab of Emerging Viruses, Institute Pasteur of Shanghai, Chinese Academy of Sciences	Lab of Emerging Viruses, Institute Pasteur of Shanghai, Chinese Academy of Sciences	Wang,W., Sheng,J., Ren,P., Zhang,J., Lin,X., Hou,L. and Deubel,V.
EPI_ISL_2591734, EPI_ISL_2591735, EPI_ISL_2591736, EPI_ISL_2591737, EPI_ISL_2591738, EPI_ISL_2591755, EPI_ISL_2591756, EPI_ISL_2591757, EPI_ISL_2591758, EPI_ISL_2591759, EPI_ISL_2591760, EPI_ISL_2591761, EPI_ISL_2591762, EPI_ISL_2591763, EPI_ISL_2591764, EPI_ISL_2591765			
see above	Miwako SaikNorth America / USA Yokohama City Institute of Public Health	Miwako SaikNorth America / USA Yokohama City Institute of Public Health	SaikNorth America / USA,M., Kawakami,C., Usuku,S., Sasao,T. and Okubo,I.
EPI_ISL_2591813	Centre de Recherche en Infectiologie, Universite Laval, CHUL	Centre de Recherche en Infectiologie, Universite Laval, CHUL	Papenburg,J., Carbonneau,J., Hamelin,M.-E., Isabel,S., Ohoumanne,N., Dery,P., Paes,B.A., Corbeil,J., Bergeron,M.G., De Serres,G. and Boivin,G.
EPI_ISL_2591820, EPI_ISL_2591821, EPI_ISL_2591822, EPI_ISL_2591823, EPI_ISL_2591824, EPI_ISL_2591825, EPI_ISL_2591826, EPI_ISL_2591827, EPI_ISL_2591828, EPI_ISL_2591829, EPI_ISL_2591830, EPI_ISL_2591831, EPI_ISL_2591832			
see above	Miwako SaikNorth America / USA Yokohama City Institute of Public Health	Miwako SaikNorth America / USA Yokohama City Institute of Public Health	SaikNorth America / USA,M., Kawakami,C., Usuku,S., Sasao,T. and Okubo,I.
EPI_ISL_2591873	Centre de Recherche en Infectiologie, Universite Laval, CHUL	Centre de Recherche en Infectiologie, Universite Laval, CHUL	Papenburg,J., Carbonneau,J., Hamelin,M.-E., Isabel,S., Ohoumanne,N., Dery,P., Paes,B.A., Corbeil,J., Bergeron,M.G., De Serres,G. and Boivin,G.
EPI_ISL_2591893, EPI_ISL_2591894, EPI_ISL_2591895	Miwako SaikNorth America / USA Yokohama City Institute of Public Health	Miwako SaikNorth America / USA Yokohama City Institute of Public Health	SaikNorth America / USA,M., Kawakami,C., Usuku,S., Sasao,T. and Okubo,I.
EPI_ISL_2591974	Molecular Virology Unit, Virology and Microbiology Department, Fondazione IRCCS Policlinico San Matteo	Molecular Virology Unit, Virology and Microbiology Department, Fondazione IRCCS Policlinico San Matteo	Piralla,A., Percivalle,E. and Baldanti,F.
EPI_ISL_2591985, EPI_ISL_2591986, EPI_ISL_2591987, EPI_ISL_2591988, EPI_ISL_2591989	Miwako SaikNorth America / USA Yokohama City Institute of Public Health	Miwako SaikNorth America / USA Yokohama City Institute of Public Health	SaikNorth America / USA,M., Kawakami,C., Usuku,S., Sasao,T. and Okubo,I.
EPI_ISL_2592024, EPI_ISL_2592025, EPI_ISL_2592026, EPI_ISL_2592027, EPI_ISL_2592028, EPI_ISL_2592029, EPI_ISL_2592030, EPI_ISL_2592032, EPI_ISL_2592033, EPI_ISL_2592034, EPI_ISL_2592035, EPI_ISL_2592036, EPI_ISL_2592037, EPI_ISL_2592038, EPI_ISL_2592039, EPI_ISL_2592040, EPI_ISL_2592041, EPI_ISL_2592042, EPI_ISL_2592043, EPI_ISL_2592044, EPI_ISL_2592045, EPI_ISL_2592046, EPI_ISL_2592047, EPI_ISL_2592048, EPI_ISL_2592049, EPI_ISL_2592050, EPI_ISL_2592051, EPI_ISL_2592052, EPI_ISL_2592053, EPI_ISL_2592054, EPI_ISL_2592055, EPI_ISL_2592056, EPI_ISL_2592057, EPI_ISL_2592058, EPI_ISL_2592059, EPI_ISL_2592060, EPI_ISL_2592061, EPI_ISL_2592062, EPI_ISL_2592063, EPI_ISL_2592064, EPI_ISL_2592065, EPI_ISL_2592066, EPI_ISL_2592067, EPI_ISL_2592068, EPI_ISL_2592069, EPI_ISL_2592070, EPI_ISL_2592071, EPI_ISL_2592072, EPI_ISL_2592073, EPI_ISL_2592074, EPI_ISL_2592075, EPI_ISL_2592076, EPI_ISL_2592077, EPI_ISL_2592078, EPI_ISL_2592079, EPI_ISL_2592080, EPI_ISL_2592081, EPI_ISL_2592082, EPI_ISL_2592083, EPI_ISL_2592084, EPI_ISL_2592085			
see above	Departament of Microbiology, University of Sao Paulo Institute of Biomedical Science	Departament of Microbiology, University of Sao Paulo Institute of Biomedical Science	Lima,H.N., Oliveira,D.B.L., Durigon,E.L. and Stewien,K.E.
EPI_ISL_2592086	Virology Branch, Butantan Institute	Virology Branch, Butantan Institute	Botosso,V.F., Peret,T.C.T., Thomazelli,L.M., Stewien,K.E. and Durigon,E.L.
EPI_ISL_2592379, EPI_ISL_2592380, EPI_ISL_2592381, EPI_ISL_2592382, EPI_ISL_2592383	Influenza Group, National Institute of Virology	Influenza Group, National Institute of Virology	Damle,R.G. and Chadha,M.S.
EPI_ISL_2592424	Molecular Virology Unit, Virology and Microbiology Department, Fondazione IRCCS Policlinico San Matteo	Molecular Virology Unit, Virology and Microbiology Department, Fondazione IRCCS Policlinico San Matteo	Piralla,A., Percivalle,E. and Baldanti,F.
EPI_ISL_2592427, EPI_ISL_2592428, EPI_ISL_2592429, EPI_ISL_2592430, EPI_ISL_2592431, EPI_ISL_2592432	Shinichi Takao Hiroshima Prefectural Technology Research Institute, Center for Public Health and Environment	Shinichi Takao Hiroshima Prefectural Technology Research Institute, Center for Public Health and Environment	Takao,S. and Shimazu,Y.
EPI_ISL_2592437, EPI_ISL_2592438, EPI_ISL_2592439, EPI_ISL_2592440, EPI_ISL_2592441, EPI_ISL_2592442, EPI_ISL_2592443, EPI_ISL_2592444, EPI_ISL_2592445, EPI_ISL_2592446, EPI_ISL_2592447, EPI_ISL_2592448, EPI_ISL_2592449			
see above	Miwako SaikNorth America / USA Yokohama City Institute of Public Health	Miwako SaikNorth America / USA Yokohama City Institute of Public Health	SaikNorth America / USA,M., Kawakami,C., Usuku,S., Sasao,T. and Okubo,I.
EPI_ISL_2592492	J. Craig Venter Institute	J. Craig Venter Institute	Shabman,R., Fedorova,N., Puri,V., Shrivastava,S., Amedeo,P., Isom,R., Hu,L., Pickett,B., Novotny,M., Durbin,A., Rocchi,I., Williams,T., Hall,C.B., Tesini,B.L., Schnabel,K.C., Walsh,E.E. and Caserta,M.
EPI_ISL_2592493, EPI_ISL_2592494, EPI_ISL_2592495, EPI_ISL_2592496, EPI_ISL_2592497, EPI_ISL_2592498, EPI_ISL_2592499, EPI_ISL_2592500, EPI_ISL_2592501, EPI_ISL_2592502, EPI_ISL_2592503, EPI_ISL_2592504, EPI_ISL_2592505, EPI_ISL_2592506, EPI_ISL_2592507, EPI_ISL_2592508, EPI_ISL_2592509, EPI_ISL_2592510, EPI_ISL_2592511, EPI_ISL_2592512, EPI_ISL_2592513, EPI_ISL_2592514			
see above	Dopazo J., Centro Nacional de Biotecnologia - CSIC, BioInformatica, Universidad Autonoma	Dopazo J., Centro Nacional de Biotecnologia - CSIC, BioInformatica, Universidad Autonoma	Dopazo,J.
EPI_ISL_2592515	Influenza Group, National Institute of Virology	Influenza Group, National Institute of Virology	Yeolekar,L.R., Peret,T.C., Erdman,D.D. and Rao,B.L.
EPI_ISL_2592516, EPI_ISL_2592517, EPI_ISL_2592518, EPI_ISL_2592519, EPI_ISL_2592520, EPI_ISL_2592521, EPI_ISL_2592522, EPI_ISL_2592523, EPI_ISL_2592524, EPI_ISL_2592525, EPI_ISL_2592526, EPI_ISL_2592527, EPI_ISL_2592528			
see above	Virologia, Facultad de Ciencias	Virologia, Facultad de Ciencias	Frabasile,S., Facal,L. and Arbiza,J.
EPI_ISL_2592529	Pediatrics - Infectious Diseases, Medical College of Wisconsin	Pediatrics - Infectious Diseases, Medical College of Wisconsin	Rebuffo-Scheer,C., Bose,M.E., He,J., Khaja,S., Ulatowski,M., Beck,E.T., Fan,J., Kumar,S., Nelson,M.I. and Henrickson,K.J.
EPI_ISL_2592530	Virologia, Universidad EL BOSQUE	Virologia, Universidad EL BOSQUE	Avila,V., Calvo,E.P., Barbosa,J., Velandia-Romero,M.L. and Castellanos,J.E.
EPI_ISL_2592531, EPI_ISL_2592532, EPI_ISL_2592533, EPI_ISL_2592534, EPI_ISL_2592535, EPI_ISL_2592536	Virologia, Facultad de Ciencias	Virologia, Facultad de Ciencias	Frabasile,S., Facal,L. and Arbiza,J.
EPI_ISL_2592537, EPI_ISL_2592538	Pediatrics, Mackay Memorial Hospital	Pediatrics, Mackay Memorial Hospital	Chi,H., Hsiao,K.-L., Weng,L.-C., Chiu,N.-C., Huang,L.-M., Chiu,Y.-Y., Liu,C.-P. and Liu,H.-F.
EPI_ISL_2592539, EPI_ISL_2592540, EPI_ISL_2592541, EPI_ISL_2592542, EPI_ISL_2592543	Department of Epidemiology and Demography, Kenya Medical Research Institute (KEMRI) - Wellcome Trust Research Programme	Department of Epidemiology and Demography, Kenya Medical Research Institute (KEMRI) - Wellcome Trust Research Programme	Otieno,J.R., Agoti,C.N., Gitahi,C.W., Bett,A., Ngama,M., Medley,G.F., Cane,P.A. and Nokes,J.D.

EPI_ISL_2592544	Virologia, Universidad EL BOSQUE	Virologia, Universidad EL BOSQUE	Avila,V., Calvo,E.P., Barbosa,J., Velandia-Romero,M.L. and Castellanos,J.E.
EPI_ISL_2592545	Pediatrics, Mackay Memorial Hospital	Pediatrics, Mackay Memorial Hospital	Chi,H., Hsiao,K.-L., Weng,L.-C., Chiu,N.-C., Huang,L.-M., Chiu,Y.-Y., Liu,C.-P. and Liu,H.-F.
EPI_ISL_2592546	Virologia, Facultad de Ciencias	Virologia, Facultad de Ciencias	Frabasile,S., Facal,L. and Arbiza,J.
EPI_ISL_2592547	Pediatrics, Mackay Memorial Hospital	Pediatrics, Mackay Memorial Hospital	Chi,H., Hsiao,K.-L., Weng,L.-C., Chiu,N.-C., Huang,L.-M., Chiu,Y.-Y., Liu,C.-P. and Liu,H.-F.
EPI_ISL_2592548	Depto Microbiologia, Instituto de Ciencias Biomedicas, Universidade de Sao Paulo	Depto Microbiologia, Instituto de Ciencias Biomedicas, Universidade de Sao Paulo	Moura,F.E.A., Thomazelli,L.M., Candido,E.D.O., Florencio,C.M.G.D., Pereira,S.A.R., Oliveira,F.M.S., Alves,A.A., Ocadaque,C.J. and Durigon,E.L.
EPI_ISL_2592549, EPI_ISL_2592550	Virologia, Facultad de Ciencias	Virologia, Facultad de Ciencias	Frabasile,S., Facal,L. and Arbiza,J.
EPI_ISL_2592551, EPI_ISL_2592552, EPI_ISL_2592553, EPI_ISL_2592554, EPI_ISL_2592555, EPI_ISL_2592556	Department of Epidemiology and Demography, Kenya Medical Research Institute (KEMRI) - Wellcome Trust Research Programme	Department of Epidemiology and Demography, Kenya Medical Research Institute (KEMRI) - Wellcome Trust Research Programme	Otieno,J.R., Agoti,C.N., Gitahi,C.W., Bett,A., Ngama,M., Medley,G.F., Cane,P.A. and Nokes,J.D.
EPI_ISL_2592557	Microbiology Department, Virology Division, College of Medicine, Taif University	Microbiology Department, Virology Division, College of Medicine, Taif University	Al Aboud,D.M., Al Aboud,N.M., Al-Malky,M.I.R. and Abdel-Moneim,A.S.
EPI_ISL_2592558, EPI_ISL_2592559, EPI_ISL_2592560, EPI_ISL_2592561, EPI_ISL_2592562, EPI_ISL_2592563, EPI_ISL_2592564, EPI_ISL_2592565	Department of Epidemiology and Demography, Kenya Medical Research Institute (KEMRI) - Wellcome Trust Research Programme	Department of Epidemiology and Demography, Kenya Medical Research Institute (KEMRI) - Wellcome Trust Research Programme	Otieno,J.R., Agoti,C.N., Gitahi,C.W., Bett,A., Ngama,M., Medley,G.F., Cane,P.A. and Nokes,J.D.
EPI_ISL_2592566	Pediatrics, Mackay Memorial Hospital	Pediatrics, Mackay Memorial Hospital	Chi,H., Hsiao,K.-L., Weng,L.-C., Chiu,N.-C., Huang,L.-M., Chiu,Y.-Y., Liu,C.-P. and Liu,H.-F.
EPI_ISL_2592567, EPI_ISL_2592568, EPI_ISL_2592569, EPI_ISL_2592570, EPI_ISL_2592571, EPI_ISL_2592572	Broad Institute of MIT & Harvard	Broad Institute of MIT & Harvard	Newman,R.M., Zody,M.C., DeVincenzo,J.P., Grad,Y., Lipsitch,M., Murphy,R., Fitzgerald,M., Young,S., Gargeya,S., Poon,T.W., Charlebois,P., Weiner,B., Yang,X., Piper,M.E., McCowan,C., Ireland,A., Levin,J., Malboeuf,C., Qu,J., Chapman,S.B., Murphy,C., Wortman,J., Nusbaum,C. and Birren,B.
EPI_ISL_2592573	Virologia, Universidad EL BOSQUE	Virologia, Universidad EL BOSQUE	Avila,V., Calvo,E.P., Barbosa,J., Velandia-Romero,M.L. and Castellanos,J.E.
EPI_ISL_2592574, EPI_ISL_2592575, EPI_ISL_2592576, EPI_ISL_2592577, EPI_ISL_2592578, EPI_ISL_2592579, EPI_ISL_2592580, EPI_ISL_2592581, EPI_ISL_2592582, EPI_ISL_2592583	Pediatrics, Mackay Memorial Hospital	Pediatrics, Mackay Memorial Hospital	Chi,H., Hsiao,K.-L., Weng,L.-C., Chiu,N.-C., Huang,L.-M., Chiu,Y.-Y., Liu,C.-P. and Liu,H.-F.
EPI_ISL_2592584, EPI_ISL_2592585, EPI_ISL_2592586, EPI_ISL_2592587, EPI_ISL_2592588, EPI_ISL_2592589, EPI_ISL_2592590, EPI_ISL_2592591, EPI_ISL_2592592, EPI_ISL_2592593, EPI_ISL_2592594, EPI_ISL_2592595, EPI_ISL_2592596, EPI_ISL_2592597, EPI_ISL_2592598, EPI_ISL_2592599, EPI_ISL_2592600, EPI_ISL_2592601, EPI_ISL_2592602, EPI_ISL_2592603, EPI_ISL_2592604, EPI_ISL_2592605, EPI_ISL_2592606, EPI_ISL_2592607, EPI_ISL_2592608, EPI_ISL_2592609, EPI_ISL_2592610, EPI_ISL_2592611, EPI_ISL_2592612, EPI_ISL_2592613, EPI_ISL_2592614, EPI_ISL_2592615, EPI_ISL_2592616, EPI_ISL_2592617, EPI_ISL_2592618, EPI_ISL_2592619, EPI_ISL_2592620, EPI_ISL_2592621			
see above	Department of Epidemiology and Demography, Kenya Medical Research Institute (KEMRI) - Wellcome Trust Research Programme	Department of Epidemiology and Demography, Kenya Medical Research Institute (KEMRI) - Wellcome Trust Research Programme	Otieno,J.R., Agoti,C.N., Gitahi,C.W., Bett,A., Ngama,M., Medley,G.F., Cane,P.A. and Nokes,J.D.
EPI_ISL_2592622	Virologia, Universidad EL BOSQUE	Virologia, Universidad EL BOSQUE	Avila,V., Calvo,E.P., Barbosa,J., Velandia-Romero,M.L. and Castellanos,J.E.
EPI_ISL_2592623, EPI_ISL_2592624, EPI_ISL_2592625, EPI_ISL_2592626, EPI_ISL_2592627, EPI_ISL_2592628, EPI_ISL_2592629, EPI_ISL_2592631	Pediatrics, Mackay Memorial Hospital	Pediatrics, Mackay Memorial Hospital	Chi,H., Hsiao,K.-L., Weng,L.-C., Chiu,N.-C., Huang,L.-M., Chiu,Y.-Y., Liu,C.-P. and Liu,H.-F.
EPI_ISL_2592632	Department of Microbiology, All India Institute of Medical Sciences	Department of Microbiology, All India Institute of Medical Sciences	Parveen,S., Sullender,W.M. and Broor,S.
EPI_ISL_2592633	Department of Epidemiology and Demography, Kenya Medical Research Institute (KEMRI) - Wellcome Trust Research Programme	Department of Epidemiology and Demography, Kenya Medical Research Institute (KEMRI) - Wellcome Trust Research Programme	Otieno,J.R., Agoti,C.N., Gitahi,C.W., Bett,A., Ngama,M., Medley,G.F., Cane,P.A. and Nokes,J.D.
EPI_ISL_2592634, EPI_ISL_2592635, EPI_ISL_2592636, EPI_ISL_2592637, EPI_ISL_2592638	Measles Section, National Institute for Viral Disease Control and Prevention, China CDC	Measles Section, National Institute for Viral Disease Control and Prevention, China CDC	Zhang,Y., Xu,W., Shen,K., Beeler,J.A., Xie,Z., Sun,L., Lu,Q., Liu,C., Liang,G. and Anderson,L.J.
EPI_ISL_2592639, EPI_ISL_2592640	Pediatrics, Mackay Memorial Hospital	Pediatrics, Mackay Memorial Hospital	Chi,H., Hsiao,K.-L., Weng,L.-C., Chiu,N.-C., Huang,L.-M., Chiu,Y.-Y., Liu,C.-P. and Liu,H.-F.
EPI_ISL_2592641, EPI_ISL_2592642	Department of Microbiology and Immunolgy, Institute of Biology, State University of Campinas (UNICAMP)	Department of Microbiology and Immunolgy, Institute of Biology, State University of Campinas (UNICAMP)	da Silva,L.H.A., Spilki,F.R., Riccetto,A.G.L., de Almeida,R.S., Baracat,E.C.E. and Arns,C.W.
EPI_ISL_2592643, EPI_ISL_2592644, EPI_ISL_2592645	Pediatrics, Mackay Memorial Hospital	Pediatrics, Mackay Memorial Hospital	Chi,H., Hsiao,K.-L., Weng,L.-C., Chiu,N.-C., Huang,L.-M., Chiu,Y.-Y., Liu,C.-P. and Liu,H.-F.
EPI_ISL_2592646	Department of Microbiology and Immunolgy, Institute of Biology, State University of Campinas (UNICAMP)	Department of Microbiology and Immunolgy, Institute of Biology, State University of Campinas (UNICAMP)	da Silva,L.H.A., Spilki,F.R., Riccetto,A.G.L., de Almeida,R.S., Baracat,E.C.E. and Arns,C.W.
EPI_ISL_2592647	Pediatrics, Mackay Memorial Hospital	Pediatrics, Mackay Memorial Hospital	Chi,H., Hsiao,K.-L., Weng,L.-C., Chiu,N.-C., Huang,L.-M., Chiu,Y.-Y., Liu,C.-P. and Liu,H.-F.
EPI_ISL_2592648	Department of Microbiology and Immunolgy, Institute of Biology, State University of Campinas (UNICAMP)	Department of Microbiology and Immunolgy, Institute of Biology, State University of Campinas (UNICAMP)	da Silva,L.H.A., Spilki,F.R., Riccetto,A.G.L., de Almeida,R.S., Baracat,E.C.E. and Arns,C.W.
EPI_ISL_2592649, EPI_ISL_2592650, EPI_ISL_2592651	Department of Microbiology, All India Institute of Medical Sciences	Department of Microbiology, All India Institute of Medical Sciences	Parveen,S., Sullender,W.M. and Broor,S.
EPI_ISL_2592652, EPI_ISL_2592653, EPI_ISL_2592654, EPI_ISL_2592655, EPI_ISL_2592656, EPI_ISL_2592657, EPI_ISL_2592658	Department of Epidemiology and Demography, Kenya Medical Research Institute (KEMRI) - Wellcome Trust Research Programme	Department of Epidemiology and Demography, Kenya Medical Research Institute (KEMRI) - Wellcome Trust Research Programme	Otieno,J.R., Agoti,C.N., Gitahi,C.W., Bett,A., Ngama,M., Medley,G.F., Cane,P.A. and Nokes,J.D.
EPI_ISL_2592659	Depto Microbiologia, Instituto de Ciencias Biomedicas, Universidade de Sao Paulo	Depto Microbiologia, Instituto de Ciencias Biomedicas, Universidade de Sao Paulo	Moura,F.E.A., Thomazelli,L.M., Candido,E.D.O., Florencio,C.M.G.D., Pereira,S.A.R., Oliveira,F.M.S., Alves,A.A., Ocadaque,C.J. and Durigon,E.L.
EPI_ISL_2592660, EPI_ISL_2592661, EPI_ISL_2592662, EPI_ISL_2592663, EPI_ISL_2592664	Pediatrics, Mackay Memorial Hospital	Pediatrics, Mackay Memorial Hospital	Chi,H., Hsiao,K.-L., Weng,L.-C., Chiu,N.-C., Huang,L.-M., Chiu,Y.-Y., Liu,C.-P. and Liu,H.-F.
EPI_ISL_2592665	Chinese Academy of Medical Sciences & Peking Union Medical College	Chinese Academy of Medical Sciences & Peking Union Medical College	Jia,B., Xiao,Y., Wang,Y., Chen,L., Zhang,J., Ren,L. and Wang,J.
EPI_ISL_2592666	Depto Microbiologia, Instituto de Ciencias Biomedicas, Universidade de Sao Paulo	Depto Microbiologia, Instituto de Ciencias Biomedicas, Universidade de Sao Paulo	Moura,F.E.A., Thomazelli,L.M., Candido,E.D.O., Florencio,C.M.G.D., Pereira,S.A.R., Oliveira,F.M.S., Alves,A.A., Ocadaque,C.J. and Durigon,E.L.
EPI_ISL_2592667, EPI_ISL_2592668, EPI_ISL_2592669, EPI_ISL_2592670, EPI_ISL_2592671, EPI_ISL_2592672, EPI_ISL_2592673, EPI_ISL_2592674	Department of Epidemiology and Demography, Kenya Medical Research Institute (KEMRI) - Wellcome Trust Research Programme	Department of Epidemiology and Demography, Kenya Medical Research Institute (KEMRI) - Wellcome Trust Research Programme	Otieno,J.R., Agoti,C.N., Gitahi,C.W., Bett,A., Ngama,M., Medley,G.F., Cane,P.A. and Nokes,J.D.
EPI_ISL_2592675, EPI_ISL_2592676	Influenza Group, National Institute of Virology	Influenza Group, National Institute of Virology	Damle,R.G., Virdi,V.K. and Chadha,M.S.
EPI_ISL_2592677	Department of Biomedical Sciences for Health, University of Milan	Department of Biomedical Sciences for Health, University of Milan	Martinelli,M., Frati,E.R., Zappa,A., Ebranati,E., Bianchi,S., Pariani,E., Amendola,A., Zehender,G. and Tanzi,E.
EPI_ISL_2592678, EPI_ISL_2592679	Microbiology and Clinical Microbiology, Cerrahpasa Medical Faculty of Istanbul University	Microbiology and Clinical Microbiology, Cerrahpasa Medical Faculty of Istanbul University	Midilli,K., Yilmaz,G., Turkoglu,S., Kuskucu,M.A., Iskonova,B., Yalcin,I.N. and Altas,K.
EPI_ISL_2592680, EPI_ISL_2592681, EPI_ISL_2592682, EPI_ISL_2592683, EPI_ISL_2592684, EPI_ISL_2592685, EPI_ISL_2592686	Pediatrics, Mackay Memorial Hospital	Pediatrics, Mackay Memorial Hospital	Chi,H., Hsiao,K.-L., Weng,L.-C., Chiu,N.-C., Huang,L.-M., Chiu,Y.-Y., Liu,C.-P. and Liu,H.-F.
EPI_ISL_2592687, EPI_ISL_2592688	Chinese Academy of Medical Sciences & Peking Union Medical College	Chinese Academy of Medical Sciences & Peking Union Medical College	Jia,B., Xiao,Y., Wang,Y., Chen,L., Zhang,J., Ren,L. and Wang,J.
EPI_ISL_2592689	Microbiology, Adolfo Lutz Institut	Microbiology, Adolfo Lutz Institut	Souza,M.C.O., Matsumoto,T.K., Vaz-de-Lima,L.R.A., Sato,N.N., Salgado,M.M., Requejo,H.I.Z., Hong,M.A., Barbosa,M.L., Oliveira,C.A.F., Benelli,P.C., Shimokawa,R.Y., Pecchini,R., Berezin,E., Duarte,S.P., Schvartsman,C., Durigon,E.L. and Ueda,M.
EPI_ISL_2592690	Microbiology and Clinical Microbiology, Cerrahpasa Medical Faculty of Istanbul University	Microbiology and Clinical Microbiology, Cerrahpasa Medical Faculty of Istanbul University	Midilli,K., Yilmaz,G., Turkoglu,S., Kuskucu,M.A., Iskonova,B., Yalcin,I.N. and Altas,K.
EPI_ISL_2592691	Depto Microbiologia, Instituto de Ciencias Biomedicas, Universidade de Sao Paulo	Depto Microbiologia, Instituto de Ciencias Biomedicas, Universidade de Sao Paulo	Moura,F.E.A., Thomazelli,L.M., Candido,E.D.O., Florencio,C.M.G.D., Pereira,S.A.R., Oliveira,F.M.S., Alves,A.A., Ocadaque,C.J. and Durigon,E.L.
EPI_ISL_2592692	Microbiology and Clinical Microbiology, Cerrahpasa Medical Faculty of Istanbul University	Microbiology and Clinical Microbiology, Cerrahpasa Medical Faculty of Istanbul University	Midilli,K., Yilmaz,G., Turkoglu,S., Kuskucu,M.A., Iskonova,B., Yalcin,I.N. and Altas,K.
EPI_ISL_2592693, EPI_ISL_2592694, EPI_ISL_2592695, EPI_ISL_2592696	Depto Microbiologia, Instituto de Ciencias Biomedicas, Universidade de Sao Paulo	Depto Microbiologia, Instituto de Ciencias Biomedicas, Universidade de Sao Paulo	Moura,F.E.A., Thomazelli,L.M., Candido,E.D.O., Florencio,C.M.G.D., Pereira,S.A.R., Oliveira,F.M.S., Alves,A.A., Ocadaque,C.J. and Durigon,E.L.
EPI_ISL_2592697, EPI_ISL_2592698, EPI_ISL_2592699, EPI_ISL_2592700, EPI_ISL_2592701, EPI_ISL_2592702, EPI_ISL_2592703, EPI_ISL_2592704, EPI_ISL_2592705, EPI_ISL_2592706, EPI_ISL_2592707, EPI_ISL_2592708, EPI_ISL_2592709, EPI_ISL_2592710, EPI_ISL_2592711, EPI_ISL_2592712, EPI_ISL_2592713, EPI_ISL_2592714, EPI_ISL_2592715, EPI_ISL_2592716, EPI_ISL_2592717, EPI_ISL_2592718, EPI_ISL_2592719, EPI_ISL_2592720, EPI_ISL_2592721, EPI_ISL_2592722, EPI_ISL_2592723, EPI_ISL_2592724, EPI_ISL_2592725, EPI_ISL_2592726, EPI_ISL_2592727, EPI_ISL_2592728, EPI_ISL_2592729, EPI_ISL_2592730, EPI_ISL_2592731, EPI_ISL_2592732, EPI_ISL_2592733, EPI_ISL_2592734, EPI_ISL_2592735, EPI_ISL_2592736, EPI_ISL_2592737, EPI_ISL_2592738, EPI_ISL_2592739, EPI_ISL_2592740, EPI_ISL_2592741, EPI_ISL_2592742, EPI_ISL_2592743, EPI_ISL_2592744			
see above	Department of Epidemiology and Demography, Kenya Medical Research Institute (KEMRI) - Wellcome Trust Research Programme	Department of Epidemiology and Demography, Kenya Medical Research Institute (KEMRI) - Wellcome Trust Research Programme	Otieno,J.R., Agoti,C.N., Gitahi,C.W., Bett,A., Ngama,M., Medley,G.F., Cane,P.A. and Nokes,J.D.
EPI_ISL_2592745	Virology Division, Public Health Laboratory Services Branch, Centre for Health Protection, Department of Health	Virology Division, Public Health Laboratory Services Branch, Centre for Health Protection, Department of Health	Mak,G.C., Lau,C.S., Chiu,D.M.Y. and Lim,W.
EPI_ISL_2592746	Depto. de Virologia, Instituto Oswaldo Cruz, FIOCRUZ, Laboratorio de Virus Respiratorios e do Sarampo	Depto. de Virologia, Instituto Oswaldo Cruz, FIOCRUZ, Laboratorio de Virus Respiratorios e do Sarampo	Machado,D.B.B., Motta,F.C., Mesquita,M.M.A., Souza,T.M.L. and Siqueira,M.M.
EPI_ISL_2592747, EPI_ISL_2592748, EPI_ISL_2592749	Department of Epidemiology and Demography, Kenya Medical Research Institute (KEMRI) - Wellcome Trust Research Programme	Department of Epidemiology and Demography, Kenya Medical Research Institute (KEMRI) - Wellcome Trust Research Programme	Otieno,J.R., Agoti,C.N., Gitahi,C.W., Bett,A., Ngama,M., Medley,G.F., Cane,P.A. and Nokes,J.D.
EPI_ISL_2592750, EPI_ISL_2592751, EPI_ISL_2592752, EPI_ISL_2592753, EPI_ISL_2592754	Depto. de Virologia, Instituto Oswaldo Cruz, FIOCRUZ, Laboratorio de Virus Respiratorios e do Sarampo	Depto. de Virologia, Instituto Oswaldo Cruz, FIOCRUZ, Laboratorio de Virus Respiratorios e do Sarampo	Machado,D.B.B., Motta,F.C., Mesquita,M.M.A., Souza,T.M.L. and Siqueira,M.M.
EPI_ISL_2592755	Department of Epidemiology and Demography, Kenya Medical Research Institute (KEMRI) - Wellcome Trust Research Programme	Department of Epidemiology and Demography, Kenya Medical Research Institute (KEMRI) - Wellcome Trust Research Programme	Otieno,J.R., Agoti,C.N., Gitahi,C.W., Bett,A., Ngama,M., Medley,G.F., Cane,P.A. and Nokes,J.D.
EPI_ISL_2592756	Division of Immunology, Children's Hospital of Chongqing Medical University	Division of Immunology, Children's Hospital of Chongqing Medical University	Zhang,Z.-Y., Du,L.-N., Chen,X., Zhao,Y., Liu,E.-M., Yang,X.-Q. and Zhao,X.-D.
EPI_ISL_2592757	Depto. de Virologia, Instituto Oswaldo Cruz, FIOCRUZ, Laboratorio de Virus Respiratorios e do Sarampo	Depto. de Virologia, Instituto Oswaldo Cruz, FIOCRUZ, Laboratorio de Virus Respiratorios e do Sarampo	Machado,D.B.B., Motta,F.C., Mesquita,M.M.A., Souza,T.M.L. and Siqueira,M.M.
EPI_ISL_2592758, EPI_ISL_2592759	Department of Epidemiology and Demography, Kenya Medical Research Institute (KEMRI) - Wellcome Trust Research Programme	Department of Epidemiology and Demography, Kenya Medical Research Institute (KEMRI) - Wellcome Trust Research Programme	Otieno,J.R., Agoti,C.N., Gitahi,C.W., Bett,A., Ngama,M., Medley,G.F., Cane,P.A. and Nokes,J.D.
EPI_ISL_2592760	Division of Immunology, Children's Hospital of Chongqing Medical University	Division of Immunology, Children's Hospital of Chongqing Medical University	Zhang,Z.-Y., Du,L.-N., Chen,X., Zhao,Y., Liu,E.-M., Yang,X.-Q. and Zhao,X.-D.
EPI_ISL_2592761, EPI_ISL_2592762	Depto. de Virologia, Instituto Oswaldo Cruz, FIOCRUZ, Laboratorio de Virus	Depto. de Virologia, Instituto Oswaldo Cruz, FIOCRUZ, Laboratorio de Virus	Machado,D.B.B., Motta,F.C., Mesquita,M.M.A., Souza,T.M.L. and Siqueira,M.M.

	Respiratorios e do Sarampo	Respiratorios e do Sarampo	
EPI_ISL_2592763, EPI_ISL_2592764, EPI_ISL_2592765, EPI_ISL_2592766	Department of Epidemiology and Demography, Kenya Medical Research Institute (KEMRI) - Wellcome Trust Research Programme	Department of Epidemiology and Demography, Kenya Medical Research Institute (KEMRI) - Wellcome Trust Research Programme	Otieno,J.R., Agoti,C.N., Gitahi,C.W., Bett,A., Ngama,M., Medley,G.F., Cane,P.A. and Nokes,J.D.
EPI_ISL_2592767, EPI_ISL_2592768	Division of Immunology, Children's Hospital of Chongqing Medical University	Division of Immunology, Children's Hospital of Chongqing Medical University	Zhang,Z.-Y., Du,L.-N., Chen,X., Zhao,Y., Liu,E.-M., Yang,X.-Q. and Zhao,X.-D.
EPI_ISL_2592769	Virology Division, Public Health Laboratory Services Branch, Centre for Health Protection, Department of Health	Virology Division, Public Health Laboratory Services Branch, Centre for Health Protection, Department of Health	Mak,G.C., Lau,C.S., Chiu,D.M.Y. and Lim,W.
EPI_ISL_2592770, EPI_ISL_2592771	Division of Immunology, Children's Hospital of Chongqing Medical University	Division of Immunology, Children's Hospital of Chongqing Medical University	Zhang,Z.-Y., Du,L.-N., Chen,X., Zhao,Y., Liu,E.-M., Yang,X.-Q. and Zhao,X.-D.
EPI_ISL_2592772, EPI_ISL_2592773, EPI_ISL_2592774, EPI_ISL_2592775, EPI_ISL_2592776, EPI_ISL_2592777, EPI_ISL_2592778, EPI_ISL_2592779, EPI_ISL_2592780, EPI_ISL_2592781, EPI_ISL_2592782			
see above	Department of Epidemiology and Demography, Kenya Medical Research Institute (KEMRI) - Wellcome Trust Research Programme	Department of Epidemiology and Demography, Kenya Medical Research Institute (KEMRI) - Wellcome Trust Research Programme	Otieno,J.R., Agoti,C.N., Gitahi,C.W., Bett,A., Ngama,M., Medley,G.F., Cane,P.A. and Nokes,J.D.
EPI_ISL_2592783	Pediatrics - Infectious Diseases, Medical College of Wisconsin	Pediatrics - Infectious Diseases, Medical College of Wisconsin	Rebuffo-Scheer,C., Bose,M.E., He,J., Khaja,S., Ulatowski,M., Beck,E.T., Fan,J., Kumar,S., Nelson,M.I. and Henrickson,K.J.
EPI_ISL_2592784, EPI_ISL_2592785, EPI_ISL_2592786, EPI_ISL_2592787, EPI_ISL_2592788, EPI_ISL_2592789, EPI_ISL_2592790, EPI_ISL_2592791, EPI_ISL_2592792, EPI_ISL_2592793, EPI_ISL_2592794, EPI_ISL_2592795, EPI_ISL_2592796, EPI_ISL_2592797, EPI_ISL_2592798, EPI_ISL_2592799, EPI_ISL_2592800, EPI_ISL_2592801, EPI_ISL_2592802, EPI_ISL_2592803, EPI_ISL_2592804, EPI_ISL_2592805, EPI_ISL_2592806, EPI_ISL_2592807, EPI_ISL_2592808, EPI_ISL_2592809, EPI_ISL_2592810, EPI_ISL_2592811, EPI_ISL_2592812, EPI_ISL_2592813, EPI_ISL_2592814, EPI_ISL_2592815, EPI_ISL_2592816, EPI_ISL_2592817, EPI_ISL_2592818, EPI_ISL_2592819, EPI_ISL_2592820, EPI_ISL_2592821, EPI_ISL_2592822, EPI_ISL_2592823, EPI_ISL_2592824, EPI_ISL_2592825, EPI_ISL_2592826, EPI_ISL_2592827, EPI_ISL_2592828, EPI_ISL_2592829, EPI_ISL_2592830, EPI_ISL_2592831, EPI_ISL_2592832, EPI_ISL_2592833, EPI_ISL_2592834, EPI_ISL_2592835, EPI_ISL_2592836, EPI_ISL_2592837, EPI_ISL_2592838, EPI_ISL_2592839, EPI_ISL_2592840, EPI_ISL_2592841, EPI_ISL_2592842, EPI_ISL_2592843, EPI_ISL_2592844, EPI_ISL_2592845, EPI_ISL_2592846, EPI_ISL_2592847, EPI_ISL_2592848, EPI_ISL_2592849			
see above	Chinese Academy of Medical Sciences & Peking Union Medical College	Chinese Academy of Medical Sciences & Peking Union Medical College	Jia,B., Xiao,Y., Wang,Y., Chen,L., Zhang,J., Ren,L. and Wang,J.
EPI_ISL_2592850, EPI_ISL_2592851	Pediatrics, Mackay Memorial Hospital	Pediatrics, Mackay Memorial Hospital	Chi,H., Hsiao,K.-L., Weng,L.-C., Chiu,N.-C., Huang,L.-M., Chiu,Y.-Y., Liu,C.-P. and Liu,H.-F.
EPI_ISL_2592852	Department of Biomedical Sciences for Health, University of Milan	Department of Biomedical Sciences for Health, University of Milan	Martinelli,M., Frati,E.R., Zappa,A., Ebranati,E., Bianchi,S., Pariani,E., Amendola,A., Zehender,G. and Tanzi,E.
EPI_ISL_2592853	Depto Microbiologia, Instituto de Ciencias Biomedicas, Universidade de Sao Paulo	Depto Microbiologia, Instituto de Ciencias Biomedicas, Universidade de Sao Paulo	Moura,F.E.A., Thomazelli,L.M., Candido,E.D.O., Florencio,C.M.G.D., Pereira,S.A.R., Oliveira,F.M.S., Alves,A.A., Ocadaque,C.J. and Durigon,E.L.
EPI_ISL_2592854, EPI_ISL_2592855, EPI_ISL_2592856, EPI_ISL_2592857	Pediatrics, Mackay Memorial Hospital	Pediatrics, Mackay Memorial Hospital	Chi,H., Hsiao,K.-L., Weng,L.-C., Chiu,N.-C., Huang,L.-M., Chiu,Y.-Y., Liu,C.-P. and Liu,H.-F.
EPI_ISL_2592858, EPI_ISL_2592859, EPI_ISL_2592860	Department of Biomedical Sciences for Health, University of Milan	Department of Biomedical Sciences for Health, University of Milan	Martinelli,M., Frati,E.R., Zappa,A., Ebranati,E., Bianchi,S., Pariani,E., Amendola,A., Zehender,G. and Tanzi,E.
EPI_ISL_2592861	Laboratory of Virology, Capital Institute of Pediatrics	Laboratory of Virology, Capital Institute of Pediatrics	Cui,G., Deng,J., Zhu,R., Qian,Y., Sun,Y., Zhao,L. and Wang,F.
EPI_ISL_2592862	Chinese Academy of Medical Sciences & Peking Union Medical College	Chinese Academy of Medical Sciences & Peking Union Medical College	Jia,B., Xiao,Y., Wang,Y., Chen,L., Zhang,J., Ren,L. and Wang,J.
EPI_ISL_2592863	Pediatrics, Mackay Memorial Hospital	Pediatrics, Mackay Memorial Hospital	Chi,H., Hsiao,K.-L., Weng,L.-C., Chiu,N.-C., Huang,L.-M., Chiu,Y.-Y., Liu,C.-P. and Liu,H.-F.
EPI_ISL_2592864, EPI_ISL_2592865, EPI_ISL_2592866, EPI_ISL_2592867	Department of Biomedical Sciences for Health, University of Milan	Department of Biomedical Sciences for Health, University of Milan	Martinelli,M., Frati,E.R., Zappa,A., Ebranati,E., Bianchi,S., Pariani,E., Amendola,A., Zehender,G. and Tanzi,E.
EPI_ISL_2592868, EPI_ISL_2592869, EPI_ISL_2592870, EPI_ISL_2592871, EPI_ISL_2592872, EPI_ISL_2592873, EPI_ISL_2592874, EPI_ISL_2592875, EPI_ISL_2592876, EPI_ISL_2592877, EPI_ISL_2592878, EPI_ISL_2592879			
see above	Chinese Academy of Medical Sciences & Peking Union Medical College	Chinese Academy of Medical Sciences & Peking Union Medical College	Jia,B., Xiao,Y., Wang,Y., Chen,L., Zhang,J., Ren,L. and Wang,J.
EPI_ISL_2592880, EPI_ISL_2592881, EPI_ISL_2592882, EPI_ISL_2592883	Laboratory of Virology, Capital Institute of Pediatrics	Laboratory of Virology, Capital Institute of Pediatrics	Cui,G., Deng,J., Zhu,R., Qian,Y., Sun,Y., Zhao,L. and Wang,F.
EPI_ISL_2592884, EPI_ISL_2592885, EPI_ISL_2592886, EPI_ISL_2592887, EPI_ISL_2592888, EPI_ISL_2592889, EPI_ISL_2592890, EPI_ISL_2592891, EPI_ISL_2592892, EPI_ISL_2592893	Chinese Academy of Medical Sciences & Peking Union Medical College	Chinese Academy of Medical Sciences & Peking Union Medical College	Jia,B., Xiao,Y., Wang,Y., Chen,L., Zhang,J., Ren,L. and Wang,J.
EPI_ISL_2592894, EPI_ISL_2592895, EPI_ISL_2592896, EPI_ISL_2592897, EPI_ISL_2592898	Laboratory of Virology, Capital Institute of Pediatrics	Laboratory of Virology, Capital Institute of Pediatrics	Cui,G., Deng,J., Zhu,R., Qian,Y., Sun,Y., Zhao,L. and Wang,F.
EPI_ISL_2592899, EPI_ISL_2592900, EPI_ISL_2592901, EPI_ISL_2592902, EPI_ISL_2592903, EPI_ISL_2592904, EPI_ISL_2592905, EPI_ISL_2592906, EPI_ISL_2592907, EPI_ISL_2592908, EPI_ISL_2592909, EPI_ISL_2592910, EPI_ISL_2592911, EPI_ISL_2592912, EPI_ISL_2592913			
see above	Chinese Academy of Medical Sciences & Peking Union Medical College	Chinese Academy of Medical Sciences & Peking Union Medical College	Jia,B., Xiao,Y., Wang,Y., Chen,L., Zhang,J., Ren,L. and Wang,J.
EPI_ISL_2592914, EPI_ISL_2592915, EPI_ISL_2592916, EPI_ISL_2592917, EPI_ISL_2592918, EPI_ISL_2592919, EPI_ISL_2592920, EPI_ISL_2592921, EPI_ISL_2592922	Laboratory of Virology, Capital Institute of Pediatrics	Laboratory of Virology, Capital Institute of Pediatrics	Cui,G., Deng,J., Zhu,R., Qian,Y., Sun,Y., Zhao,L. and Wang,F.
EPI_ISL_2592923	Depto Microbiologia, Instituto de Ciencias Biomedicas, Universidade de Sao Paulo	Depto Microbiologia, Instituto de Ciencias Biomedicas, Universidade de Sao Paulo	Moura,F.E.A., Thomazelli,L.M., Candido,E.D.O., Florencio,C.M.G.D., Pereira,S.A.R., Oliveira,F.M.S., Alves,A.A., Ocadaque,C.J. and Durigon,E.L.
EPI_ISL_2592924, EPI_ISL_2592925, EPI_ISL_2592926	Microbiology, Adolfo Lutz Institut	Microbiology, Adolfo Lutz Institut	Souza,M.C.O., Matsumoto,T.K., Vaz-de-Lima,L.R.A., Sato,N.N., Salgado,M.M., Requejo,H.I.Z., Hong,M.A., Barbosa,M.L., Oliveira,C.A.F., Benelli,P.C., Shimokawa,R.Y., Pecchini,R., Berezin,E., Duarte,S.P., Schvartsman,C., Durigon,E.L. and Ueda,M.
EPI_ISL_2592927	Laboratory of Virology, Capital Institute of Pediatrics	Laboratory of Virology, Capital Institute of Pediatrics	Cui,G., Deng,J., Zhu,R., Qian,Y., Sun,Y., Zhao,L. and Wang,F.
EPI_ISL_2592928, EPI_ISL_2592929, EPI_ISL_2592930, EPI_ISL_2592931, EPI_ISL_2592932, EPI_ISL_2592933, EPI_ISL_2592934, EPI_ISL_2592935, EPI_ISL_2592936	Department of Epidemiology and Demography, Kenya Medical Research Institute (KEMRI) - Wellcome Trust Research Programme	Department of Epidemiology and Demography, Kenya Medical Research Institute (KEMRI) - Wellcome Trust Research Programme	Otieno,J.R., Agoti,C.N., Gitahi,C.W., Bett,A., Ngama,M., Medley,G.F., Cane,P.A. and Nokes,J.D.
EPI_ISL_2592937, EPI_ISL_2592938	Division of Immunology, Children's Hospital of Chongqing Medical University	Division of Immunology, Children's Hospital of Chongqing Medical University	Zhang,Z.-Y., Du,L.-N., Chen,X., Zhao,Y., Liu,E.-M., Yang,X.-Q. and Zhao,X.-D.
EPI_ISL_2592939, EPI_ISL_2592940, EPI_ISL_2592941, EPI_ISL_2592942, EPI_ISL_2592943, EPI_ISL_2592944, EPI_ISL_2592945, EPI_ISL_2592946	Department of Epidemiology and Demography, Kenya Medical Research Institute (KEMRI) - Wellcome Trust Research Programme	Department of Epidemiology and Demography, Kenya Medical Research Institute (KEMRI) - Wellcome Trust Research Programme	Otieno,J.R., Agoti,C.N., Gitahi,C.W., Bett,A., Ngama,M., Medley,G.F., Cane,P.A. and Nokes,J.D.
EPI_ISL_2592947	Division of Immunology, Children's Hospital of Chongqing Medical University	Division of Immunology, Children's Hospital of Chongqing Medical University	Zhang,Z.-Y., Du,L.-N., Chen,X., Zhao,Y., Liu,E.-M., Yang,X.-Q. and Zhao,X.-D.
EPI_ISL_2592948, EPI_ISL_2592949, EPI_ISL_2592950, EPI_ISL_2592951, EPI_ISL_2592952, EPI_ISL_2592953, EPI_ISL_2592954, EPI_ISL_2592955, EPI_ISL_2592956, EPI_ISL_2592957, EPI_ISL_2592958, EPI_ISL_2592959, EPI_ISL_2592960, EPI_ISL_2592961, EPI_ISL_2592962, EPI_ISL_2592963, EPI_ISL_2592964			
see above	Department of Epidemiology and Demography, Kenya Medical Research Institute (KEMRI) - Wellcome Trust Research Programme	Department of Epidemiology and Demography, Kenya Medical Research Institute (KEMRI) - Wellcome Trust Research Programme	Otieno,J.R., Agoti,C.N., Gitahi,C.W., Bett,A., Ngama,M., Medley,G.F., Cane,P.A. and Nokes,J.D.
EPI_ISL_2592965	Division of Immunology, Children's Hospital of Chongqing Medical University	Division of Immunology, Children's Hospital of Chongqing Medical University	Zhang,Z.-Y., Du,L.-N., Chen,X., Zhao,Y., Liu,E.-M., Yang,X.-Q. and Zhao,X.-D.
EPI_ISL_2592966, EPI_ISL_2592967	Department of Epidemiology and Demography, Kenya Medical Research Institute (KEMRI) - Wellcome Trust Research Programme	Department of Epidemiology and Demography, Kenya Medical Research Institute (KEMRI) - Wellcome Trust Research Programme	Otieno,J.R., Agoti,C.N., Gitahi,C.W., Bett,A., Ngama,M., Medley,G.F., Cane,P.A. and Nokes,J.D.
EPI_ISL_2592968	Depto. de Virologia, Instituto Oswaldo Cruz, FIOCRUZ, Laboratorio de Virus Respiratorios e do Sarampo	Depto. de Virologia, Instituto Oswaldo Cruz, FIOCRUZ, Laboratorio de Virus Respiratorios e do Sarampo	Machado,D.B.B., Motta,F.C., Mesquita,M.M.A., Souza,T.M.L. and Siqueira,M.M.
EPI_ISL_2592969, EPI_ISL_2592970, EPI_ISL_2592971, EPI_ISL_2592972, EPI_ISL_2592973	Department of Epidemiology and Demography, Kenya Medical Research Institute (KEMRI) - Wellcome Trust Research Programme	Department of Epidemiology and Demography, Kenya Medical Research Institute (KEMRI) - Wellcome Trust Research Programme	Otieno,J.R., Agoti,C.N., Gitahi,C.W., Bett,A., Ngama,M., Medley,G.F., Cane,P.A. and Nokes,J.D.
EPI_ISL_2592974	Division of Immunology, Children's Hospital of Chongqing Medical University	Division of Immunology, Children's Hospital of Chongqing Medical University	Zhang,Z.-Y., Du,L.-N., Chen,X., Zhao,Y., Liu,E.-M., Yang,X.-Q. and Zhao,X.-D.
EPI_ISL_2592975, EPI_ISL_2592976, EPI_ISL_2592977, EPI_ISL_2592978, EPI_ISL_2592979, EPI_ISL_2592980, EPI_ISL_2592981, EPI_ISL_2592982, EPI_ISL_2592983, EPI_ISL_2592984, EPI_ISL_2592985, EPI_ISL_2592986, EPI_ISL_2592987			
see above	Department of Epidemiology and Demography, Kenya Medical Research Institute (KEMRI) - Wellcome Trust Research Programme	Department of Epidemiology and Demography, Kenya Medical Research Institute (KEMRI) - Wellcome Trust Research Programme	Otieno,J.R., Agoti,C.N., Gitahi,C.W., Bett,A., Ngama,M., Medley,G.F., Cane,P.A. and Nokes,J.D.
EPI_ISL_2592988	Depto. de Virologia, Instituto Oswaldo Cruz, FIOCRUZ, Laboratorio de Virus Respiratorios e do Sarampo	Depto. de Virologia, Instituto Oswaldo Cruz, FIOCRUZ, Laboratorio de Virus Respiratorios e do Sarampo	Machado,D.B.B., Motta,F.C., Mesquita,M.M.A., Souza,T.M.L. and Siqueira,M.M.
EPI_ISL_2592989, EPI_ISL_2592990, EPI_ISL_2592991, EPI_ISL_2592992	Department of Epidemiology and Demography, Kenya Medical Research Institute (KEMRI) - Wellcome Trust Research Programme	Department of Epidemiology and Demography, Kenya Medical Research Institute (KEMRI) - Wellcome Trust Research Programme	Otieno,J.R., Agoti,C.N., Gitahi,C.W., Bett,A., Ngama,M., Medley,G.F., Cane,P.A. and Nokes,J.D.
EPI_ISL_2592993, EPI_ISL_2592994, EPI_ISL_2592995, EPI_ISL_2592996, EPI_ISL_2592997, EPI_ISL_2592998, EPI_ISL_2592999	Depto. de Virologia, Instituto Oswaldo Cruz, FIOCRUZ, Laboratorio de Virus Respiratorios e do Sarampo	Depto. de Virologia, Instituto Oswaldo Cruz, FIOCRUZ, Laboratorio de Virus Respiratorios e do Sarampo	Machado,D.B.B., Motta,F.C., Mesquita,M.M.A., Souza,T.M.L. and Siqueira,M.M.
EPI_ISL_2593000, EPI_ISL_2593001	Department of Epidemiology and Demography, Kenya Medical Research Institute (KEMRI) - Wellcome Trust Research Programme	Department of Epidemiology and Demography, Kenya Medical Research Institute (KEMRI) - Wellcome Trust Research Programme	Otieno,J.R., Agoti,C.N., Gitahi,C.W., Bett,A., Ngama,M., Medley,G.F., Cane,P.A. and Nokes,J.D.
EPI_ISL_2593002, EPI_ISL_2593003, EPI_ISL_2593004	Depto. de Virologia, Instituto Oswaldo Cruz, FIOCRUZ, Laboratorio de Virus Respiratorios e do Sarampo	Depto. de Virologia, Instituto Oswaldo Cruz, FIOCRUZ, Laboratorio de Virus Respiratorios e do Sarampo	Machado,D.B.B., Motta,F.C., Mesquita,M.M.A., Souza,T.M.L. and Siqueira,M.M.
EPI_ISL_2593005	Department of Epidemiology and Demography, Kenya Medical Research Institute (KEMRI) - Wellcome Trust Research Programme	Department of Epidemiology and Demography, Kenya Medical Research Institute (KEMRI) - Wellcome Trust Research Programme	Otieno,J.R., Agoti,C.N., Gitahi,C.W., Bett,A., Ngama,M., Medley,G.F., Cane,P.A. and Nokes,J.D.
EPI_ISL_2593006	Depto. de Virologia, Instituto Oswaldo Cruz, FIOCRUZ, Laboratorio de Virus Respiratorios e do Sarampo	Depto. de Virologia, Instituto Oswaldo Cruz, FIOCRUZ, Laboratorio de Virus Respiratorios e do Sarampo	Machado,D.B.B., Motta,F.C., Mesquita,M.M.A., Souza,T.M.L. and Siqueira,M.M.
EPI_ISL_2593007, EPI_ISL_2593008, EPI_ISL_2593009	Department of Epidemiology and Demography, Kenya Medical Research Institute (KEMRI) - Wellcome Trust Research Programme	Department of Epidemiology and Demography, Kenya Medical Research Institute (KEMRI) - Wellcome Trust Research Programme	Otieno,J.R., Agoti,C.N., Gitahi,C.W., Bett,A., Ngama,M., Medley,G.F., Cane,P.A. and Nokes,J.D.
EPI_ISL_2593010, EPI_ISL_2593011, EPI_ISL_2593012, EPI_ISL_2593013	Depto. de Virologia, Instituto Oswaldo Cruz, FIOCRUZ, Laboratorio de Virus Respiratorios e do Sarampo	Depto. de Virologia, Instituto Oswaldo Cruz, FIOCRUZ, Laboratorio de Virus Respiratorios e do Sarampo	Machado,D.B.B., Motta,F.C., Mesquita,M.M.A., Souza,T.M.L. and Siqueira,M.M.
EPI_ISL_2593014	Virology Division, Public Health Laboratory Services Branch, Centre for Health Protection, Department of Health	Virology Division, Public Health Laboratory Services Branch, Centre for Health Protection, Department of Health	Mak,G.C., Lau,C.S., Chiu,D.M.Y. and Lim,W.
EPI_ISL_2593015, EPI_ISL_2593016, EPI_ISL_2593017	Department of Epidemiology and Demography, Kenya Medical Research Institute (KEMRI) - Wellcome Trust Research Programme	Department of Epidemiology and Demography, Kenya Medical Research Institute (KEMRI) - Wellcome Trust Research Programme	Otieno,J.R., Agoti,C.N., Gitahi,C.W., Bett,A., Ngama,M., Medley,G.F., Cane,P.A. and Nokes,J.D.
EPI_ISL_2593018, EPI_ISL_2593019, EPI_ISL_2593020	Depto. de Virologia, Instituto Oswaldo Cruz, FIOCRUZ, Laboratorio de Virus Respiratorios e do Sarampo	Depto. de Virologia, Instituto Oswaldo Cruz, FIOCRUZ, Laboratorio de Virus Respiratorios e do Sarampo	Machado,D.B.B., Motta,F.C., Mesquita,M.M.A., Souza,T.M.L. and Siqueira,M.M.

EPI_ISL_2593021	Virology Division, Public Health Laboratory Services Branch, Centre for Health Protection, Department of Health	Virology Division, Public Health Laboratory Services Branch, Centre for Health Protection, Department of Health	Mak,G.C., Lau,C.S., Chiu,D.M.Y. and Lim,W.
EPI_ISL_2593022, EPI_ISL_2593023, EPI_ISL_2593024, EPI_ISL_2593025, EPI_ISL_2593026, EPI_ISL_2593027, EPI_ISL_2593028, EPI_ISL_2593029, EPI_ISL_2593030, EPI_ISL_2593031	Depto. de Virologia, Instituto Oswaldo Cruz, FIOCRUZ, Laboratorio de Virus Respiratorios e do Sarampo	Depto. de Virologia, Instituto Oswaldo Cruz, FIOCRUZ, Laboratorio de Virus Respiratorios e do Sarampo	Machado,D.B.B., Motta,F.C., Mesquita,M.M.A., Souza,T.M.L. and Siqueira,M.M.
EPI_ISL_2593032	Virology Division, Public Health Laboratory Services Branch, Centre for Health Protection, Department of Health	Virology Division, Public Health Laboratory Services Branch, Centre for Health Protection, Department of Health	Mak,G.C., Lau,C.S., Chiu,D.M.Y. and Lim,W.
EPI_ISL_2593033	Depto. de Virologia, Instituto Oswaldo Cruz, FIOCRUZ, Laboratorio de Virus Respiratorios e do Sarampo	Depto. de Virologia, Instituto Oswaldo Cruz, FIOCRUZ, Laboratorio de Virus Respiratorios e do Sarampo	Machado,D.B.B., Motta,F.C., Mesquita,M.M.A., Souza,T.M.L. and Siqueira,M.M.
EPI_ISL_2593034	Division of Immunology, Children's Hospital of Chongqing Medical University	Division of Immunology, Children's Hospital of Chongqing Medical University	Zhang,Z.-Y., Du,L.-N., Chen,X., Zhao,Y., Liu,E.-M., Yang,X.-Q. and Zhao,X.-D.
EPI_ISL_2593035	Virology Division, Public Health Laboratory Services Branch, Centre for Health Protection, Department of Health	Virology Division, Public Health Laboratory Services Branch, Centre for Health Protection, Department of Health	Mak,G.C., Lau,C.S., Chiu,D.M.Y. and Lim,W.
EPI_ISL_2593036	Virology, School of Public Health and Institute of Public Health Research, Tehran University of Medical Sciences	Virology, School of Public Health and Institute of Public Health Research, Tehran University of Medical Sciences	Mokhtari-Azad,T., Faghihloo,E., Salimi,V. and Rezaei,F.
EPI_ISL_2593037, EPI_ISL_2593038, EPI_ISL_2593039	Department of Epidemiology and Demography, Kenya Medical Research Institute (KEMRI) - Wellcome Trust Research Programme	Department of Epidemiology and Demography, Kenya Medical Research Institute (KEMRI) - Wellcome Trust Research Programme	Otieno,J.R., Agoti,C.N., Gitahi,C.W., Bett,A., Ngama,M., Medley,G.F., Cane,P.A. and Nokes,J.D.
EPI_ISL_2593040, EPI_ISL_2593041	Virology, School of Public Health and Institute of Public Health Research, Tehran University of Medical Sciences	Virology, School of Public Health and Institute of Public Health Research, Tehran University of Medical Sciences	Mokhtari-Azad,T., Faghihloo,E., Salimi,V. and Rezaei,F.
EPI_ISL_2593042	Department of Epidemiology and Demography, Kenya Medical Research Institute (KEMRI) - Wellcome Trust Research Programme	Department of Epidemiology and Demography, Kenya Medical Research Institute (KEMRI) - Wellcome Trust Research Programme	Otieno,J.R., Agoti,C.N., Gitahi,C.W., Bett,A., Ngama,M., Medley,G.F., Cane,P.A. and Nokes,J.D.
EPI_ISL_2593043	Division of Immunology, Children's Hospital of Chongqing Medical University	Division of Immunology, Children's Hospital of Chongqing Medical University	Zhang,Z.-Y., Du,L.-N., Chen,X., Zhao,Y., Liu,E.-M., Yang,X.-Q. and Zhao,X.-D.
EPI_ISL_2593044, EPI_ISL_2593045	Department of Epidemiology and Demography, Kenya Medical Research Institute (KEMRI) - Wellcome Trust Research Programme	Department of Epidemiology and Demography, Kenya Medical Research Institute (KEMRI) - Wellcome Trust Research Programme	Otieno,J.R., Agoti,C.N., Gitahi,C.W., Bett,A., Ngama,M., Medley,G.F., Cane,P.A. and Nokes,J.D.
EPI_ISL_2593046	Division of Immunology, Children's Hospital of Chongqing Medical University	Division of Immunology, Children's Hospital of Chongqing Medical University	Zhang,Z.-Y., Du,L.-N., Chen,X., Zhao,Y., Liu,E.-M., Yang,X.-Q. and Zhao,X.-D.
EPI_ISL_2593047, EPI_ISL_2593048	Department of Epidemiology and Demography, Kenya Medical Research Institute (KEMRI) - Wellcome Trust Research Programme	Department of Epidemiology and Demography, Kenya Medical Research Institute (KEMRI) - Wellcome Trust Research Programme	Otieno,J.R., Agoti,C.N., Gitahi,C.W., Bett,A., Ngama,M., Medley,G.F., Cane,P.A. and Nokes,J.D.
EPI_ISL_2593049	Dept Microbiology, Instituto de Ciencias Biomedicas, Universidade de Sao Paulo	Dept Microbiology, Instituto de Ciencias Biomedicas, Universidade de Sao Paulo	Moura,F.E.A., Perdigao,A.C.B., Ribeiro,J.F., Florencio,C.M.G.D., Oliveira,F.M.S., Pereira,S.A.R., Botosso,V.F., Siqueira,M.M., Thomazelli,L.M., Caldeira,R.N., Oliveira,D.B.L. and Durigon,E.L.
EPI_ISL_2593050, EPI_ISL_2593051, EPI_ISL_2593052, EPI_ISL_2593053, EPI_ISL_2593054, EPI_ISL_2593055, EPI_ISL_2593056, EPI_ISL_2593057, EPI_ISL_2593058, EPI_ISL_2593059, EPI_ISL_2593060, EPI_ISL_2593061, EPI_ISL_2593062, EPI_ISL_2593063, EPI_ISL_2593064, EPI_ISL_2593065, EPI_ISL_2593066, EPI_ISL_2593067, EPI_ISL_2593068, EPI_ISL_2593069, EPI_ISL_2593070, EPI_ISL_2593071, EPI_ISL_2593072, EPI_ISL_2593073, EPI_ISL_2593074, EPI_ISL_2593075, EPI_ISL_2593076, EPI_ISL_2593077, EPI_ISL_2593078, EPI_ISL_2593079, EPI_ISL_2593080, EPI_ISL_2593081, EPI_ISL_2593082, EPI_ISL_2593083, EPI_ISL_2593084, EPI_ISL_2593085, EPI_ISL_2593086, EPI_ISL_2593087, EPI_ISL_2593088, EPI_ISL_2593089, EPI_ISL_2593090, EPI_ISL_2593091, EPI_ISL_2593092, EPI_ISL_2593093, EPI_ISL_2593094, EPI_ISL_2593095			
see above	Chinese Academy of Medical Sciences & Peking Union Medical College	Chinese Academy of Medical Sciences & Peking Union Medical College	Jia,B., Xiao,Y., Wang,Y., Chen,L., Zhang,J., Ren,L. and Wang,J.
EPI_ISL_2593096	Virology Dep, School of Public Health and Institute of Public Health Research, Tehran University of Medical Sciences	Virology Dep, School of Public Health and Institute of Public Health Research, Tehran University of Medical Sciences	Mokhtari-Azad,T., Faghihloo,E., Salimi,V. and Rezaei,F.
EPI_ISL_2593097, EPI_ISL_2593098, EPI_ISL_2593099, EPI_ISL_2593100	Chinese Academy of Medical Sciences & Peking Union Medical College	Chinese Academy of Medical Sciences & Peking Union Medical College	Jia,B., Xiao,Y., Wang,Y., Chen,L., Zhang,J., Ren,L. and Wang,J.
EPI_ISL_2593101	WHO WPRO Measles Regional Reference Lab, Key Laboratory of Medical Virology Ministry of Health, National Institute for Viral Disease Control and Prevention, China Center for Disease Control and Prevention	WHO WPRO Measles Regional Reference Lab, Key Laboratory of Medical Virology Ministry of Health, National Institute for Viral Disease Control and Prevention, China Center for Disease Control and Prevention	Zhang,Y., Song,J. and Xu,W.
EPI_ISL_2593102	Pediatrics, Mackay Memorial Hospital	Pediatrics, Mackay Memorial Hospital	Chi,H., Hsiao,K.-L., Weng,L.-C., Chiu,N.-C., Huang,L.-M., Chiu,Y.-Y., Liu,C.-P. and Liu,H.-F.
EPI_ISL_2593103	Pediatrics, Show-Chwan Memorial Hospital	Pediatrics, Show-Chwan Memorial Hospital	Lee,C.-Y.
EPI_ISL_2593104, EPI_ISL_2593105	Depto Microbiologia, Instituto de Ciencias Biomedicas, Universidade de Sao Paulo	Depto Microbiologia, Instituto de Ciencias Biomedicas, Universidade de Sao Paulo	Moura,F.E.A., Thomazelli,L.M., Candido,E.D.O., Florencio,C.M.G.D., Pereira,S.A.R., Oliveira,F.M.S., Alves,A.A., Ocadaque,C.J. and Durigon,E.L.
EPI_ISL_2593106	Pediatrics, Mackay Memorial Hospital	Pediatrics, Mackay Memorial Hospital	Chi,H., Hsiao,K.-L., Weng,L.-C., Chiu,N.-C., Huang,L.-M., Chiu,Y.-Y., Liu,C.-P. and Liu,H.-F.
EPI_ISL_2593107	Pediatrics, Show-Chwan Memorial Hospital	Pediatrics, Show-Chwan Memorial Hospital	Lee,C.-Y.
EPI_ISL_2593108	Pediatrics, Mackay Memorial Hospital	Pediatrics, Mackay Memorial Hospital	Chi,H., Hsiao,K.-L., Weng,L.-C., Chiu,N.-C., Huang,L.-M., Chiu,Y.-Y., Liu,C.-P. and Liu,H.-F.
EPI_ISL_2593109, EPI_ISL_2593110, EPI_ISL_2593111	Pediatrics, Show-Chwan Memorial Hospital	Pediatrics, Show-Chwan Memorial Hospital	Lee,C.-Y.
EPI_ISL_2593112	Department of Biomedical Sciences for Health, University of Milan	Department of Biomedical Sciences for Health, University of Milan	Martinelli,M., Frati,E.R., Zappa,A., Ebranati,E., Bianchi,S., Pariani,E., Amendola,A., Zehender,G. and Tanzi,E.
EPI_ISL_2593113	Chinese Academy of Medical Sciences & Peking Union Medical College	Chinese Academy of Medical Sciences & Peking Union Medical College	Jia,B., Xiao,Y., Wang,Y., Chen,L., Zhang,J., Ren,L. and Wang,J.
EPI_ISL_2593114, EPI_ISL_2593115	Pediatrics, Mackay Memorial Hospital	Pediatrics, Mackay Memorial Hospital	Chi,H., Hsiao,K.-L., Weng,L.-C., Chiu,N.-C., Huang,L.-M., Chiu,Y.-Y., Liu,C.-P. and Liu,H.-F.
EPI_ISL_2593116, EPI_ISL_2593117	Pediatrics, Show-Chwan Memorial Hospital	Pediatrics, Show-Chwan Memorial Hospital	Lee,C.-Y.
EPI_ISL_2593118, EPI_ISL_2593119, EPI_ISL_2593120	Department of Biomedical Sciences for Health, University of Milan	Department of Biomedical Sciences for Health, University of Milan	Martinelli,M., Frati,E.R., Zappa,A., Ebranati,E., Bianchi,S., Pariani,E., Amendola,A., Zehender,G. and Tanzi,E.
EPI_ISL_2593121, EPI_ISL_2593122, EPI_ISL_2593123, EPI_ISL_2593124, EPI_ISL_2593125, EPI_ISL_2593126, EPI_ISL_2593127, EPI_ISL_2593128, EPI_ISL_2593129, EPI_ISL_2593130, EPI_ISL_2593131			
see above	Chinese Academy of Medical Sciences & Peking Union Medical College	Chinese Academy of Medical Sciences & Peking Union Medical College	Jia,B., Xiao,Y., Wang,Y., Chen,L., Zhang,J., Ren,L. and Wang,J.
EPI_ISL_2593132, EPI_ISL_2593133	Laboratory of Virology, Capital Institute of Pediatrics	Laboratory of Virology, Capital Institute of Pediatrics	Cui,G., Deng,J., Zhu,R., Qian,Y., Sun,Y., Zhao,L. and Wang,F.
EPI_ISL_2593134, EPI_ISL_2593135, EPI_ISL_2593136, EPI_ISL_2593137, EPI_ISL_2593138, EPI_ISL_2593139, EPI_ISL_2593140, EPI_ISL_2593141, EPI_ISL_2593142, EPI_ISL_2593143, EPI_ISL_2593144			
see above	Chinese Academy of Medical Sciences & Peking Union Medical College	Chinese Academy of Medical Sciences & Peking Union Medical College	Jia,B., Xiao,Y., Wang,Y., Chen,L., Zhang,J., Ren,L. and Wang,J.
EPI_ISL_2593145, EPI_ISL_2593146, EPI_ISL_2593147, EPI_ISL_2593148, EPI_ISL_2593149, EPI_ISL_2593150	Laboratory of Virology, Capital Institute of Pediatrics	Laboratory of Virology, Capital Institute of Pediatrics	Cui,G., Deng,J., Zhu,R., Qian,Y., Sun,Y., Zhao,L. and Wang,F.
EPI_ISL_2593151, EPI_ISL_2593152, EPI_ISL_2593153, EPI_ISL_2593154, EPI_ISL_2593155, EPI_ISL_2593156, EPI_ISL_2593157, EPI_ISL_2593158, EPI_ISL_2593159, EPI_ISL_2593160, EPI_ISL_2593161, EPI_ISL_2593162, EPI_ISL_2593163, EPI_ISL_2593164, EPI_ISL_2593165, EPI_ISL_2593166, EPI_ISL_2593167			
see above	Chinese Academy of Medical Sciences & Peking Union Medical College	Chinese Academy of Medical Sciences & Peking Union Medical College	Jia,B., Xiao,Y., Wang,Y., Chen,L., Zhang,J., Ren,L. and Wang,J.
EPI_ISL_2593168, EPI_ISL_2593169, EPI_ISL_2593170, EPI_ISL_2593171, EPI_ISL_2593172	Laboratory of Virology, Capital Institute of Pediatrics	Laboratory of Virology, Capital Institute of Pediatrics	Cui,G., Deng,J., Zhu,R., Qian,Y., Sun,Y., Zhao,L. and Wang,F.
EPI_ISL_2593173	Virology, University of Washington	Virology, University of Washington	Greninger,A.L., Shean,R.C. and Makhsous,N.
EPI_ISL_2593174, EPI_ISL_2593175, EPI_ISL_2593176, EPI_ISL_2593177, EPI_ISL_2593178, EPI_ISL_2593179	Respiratory Viruses Branch, Centers for Disease Control and Prevention	Respiratory Viruses Branch, Centers for Disease Control and Prevention	Peret,T.C.T.
EPI_ISL_2593180, EPI_ISL_2593181, EPI_ISL_2593182	Chinese Academy of Medical Sciences & Peking Union Medical College	Chinese Academy of Medical Sciences & Peking Union Medical College	Jia,B., Xiao,Y., Wang,Y., Chen,L., Zhang,J., Ren,L. and Wang,J.
EPI_ISL_2593183, EPI_ISL_2593184	Virology, School of Public Health and Institute of Public Health Research, Tehran University of Medical Sciences	Virology, School of Public Health and Institute of Public Health Research, Tehran University of Medical Sciences	Mokhtari-Azad,T., Faghihloo,E., Salimi,V. and Rezaei,F.
EPI_ISL_2593185	Depto. de Virologia, Instituto Oswaldo Cruz, FIOCRUZ, Laboratorio de Virus Respiratorios e do Sarampo	Depto. de Virologia, Instituto Oswaldo Cruz, FIOCRUZ, Laboratorio de Virus Respiratorios e do Sarampo	Machado,D.B.B., Motta,F.C., Mesquita,M.M.A., Souza,T.M.L. and Siqueira,M.M.
EPI_ISL_2593186, EPI_ISL_2593187	Division of Immunology, Children's Hospital of Chongqing Medical University	Division of Immunology, Children's Hospital of Chongqing Medical University	Zhang,Z.-Y., Du,L.-N., Chen,X., Zhao,Y., Liu,E.-M., Yang,X.-Q. and Zhao,X.-D.
EPI_ISL_2593188	Department of Epidemiology and Demography, Kenya Medical Research Institute (KEMRI) - Wellcome Trust Research Programme	Department of Epidemiology and Demography, Kenya Medical Research Institute (KEMRI) - Wellcome Trust Research Programme	Otieno,J.R., Agoti,C.N., Gitahi,C.W., Bett,A., Ngama,M., Medley,G.F., Cane,P.A. and Nokes,J.D.
EPI_ISL_2593189	Department of Clinical Laboratory, Fujian Provincial Hospital	Department of Clinical Laboratory, Fujian Provincial Hospital	Su,Y., Wu,Y., Tian,R. and Liang,G.
EPI_ISL_2593190	Department of Epidemiology and Demography, Kenya Medical Research Institute (KEMRI) - Wellcome Trust Research Programme	Department of Epidemiology and Demography, Kenya Medical Research Institute (KEMRI) - Wellcome Trust Research Programme	Otieno,J.R., Agoti,C.N., Gitahi,C.W., Bett,A., Ngama,M., Medley,G.F., Cane,P.A. and Nokes,J.D.
EPI_ISL_2593191	Epidemiology and Demography, KEMRI-Wellcome Trust Research Collaborative Programme	Epidemiology and Demography, KEMRI-Wellcome Trust Research Collaborative Programme	Agoti,C.N., Mayieka,L., Otieno,J.R., Ahmed,J.A., Fields,B., Waiboci,L.B., Nyoka,R., Eidex,R., Morano,N., Burton,W., Montgomery,J., Breiman,R. and Nokes,D.J.
EPI_ISL_2593192	Department of Epidemiology and Demography, Kenya Medical Research Institute (KEMRI) - Wellcome Trust Research Programme	Department of Epidemiology and Demography, Kenya Medical Research Institute (KEMRI) - Wellcome Trust Research Programme	Otieno,J.R., Agoti,C.N., Gitahi,C.W., Bett,A., Ngama,M., Medley,G.F., Cane,P.A. and Nokes,J.D.
EPI_ISL_2593193	Virology, School of Public Health and Institute of Public Health Research, Tehran University of Medical Sciences	Virology, School of Public Health and Institute of Public Health Research, Tehran University of Medical Sciences	Mokhtari-Azad,T., Faghihloo,E., Salimi,V. and Rezaei,F.
EPI_ISL_2593194	Department of Epidemiology and Demography, Kenya Medical Research Institute (KEMRI) - Wellcome Trust Research Programme	Department of Epidemiology and Demography, Kenya Medical Research Institute (KEMRI) - Wellcome Trust Research Programme	Otieno,J.R., Agoti,C.N., Gitahi,C.W., Bett,A., Ngama,M., Medley,G.F., Cane,P.A. and Nokes,J.D.
EPI_ISL_2593195	Epidemiology and Demography, KEMRI-Wellcome Trust Research Collaborative Programme	Epidemiology and Demography, KEMRI-Wellcome Trust Research Collaborative Programme	Agoti,C.N., Mayieka,L., Otieno,J.R., Ahmed,J.A., Fields,B., Waiboci,L.B., Nyoka,R., Eidex,R., Morano,N., Burton,W., Montgomery,J., Breiman,R. and Nokes,D.J.

EPI_ISL_2593196, EPI_ISL_2593197	Department of Epidemiology and Demography, Kenya Medical Research Institute (KEMRI) - Wellcome Trust Research Programme	Department of Epidemiology and Demography, Kenya Medical Research Institute (KEMRI) - Wellcome Trust Research Programme	Otieno,J.R., Agoti,C.N., Gitahi,C.W., Bett,A., Ngama,M., Medley,G.F., Cane,P.A. and Nokes,J.D.
EPI_ISL_2593198	Epidemiology and Demography, KEMRI-Wellcome Trust Research Collaborative Programme	Epidemiology and Demography, KEMRI-Wellcome Trust Research Collaborative Programme	Agoti,C.N., Mayieka,L., Otieno,J.R., Ahmed,J.A., Fields,B., Waiboci,L.B., Nyoka,R., Eidex,R., Morano,N., Burton,W., Montgomery,J., Breiman,R. and Nokes,D.J.
EPI_ISL_2593199	Virology, School of Public Health and Institute of Public Health Research, Tehran University of Medical Sciences	Virology, School of Public Health and Institute of Public Health Research, Tehran University of Medical Sciences	Mokhtari-Azad,T., Faghihloo,E., Salimi,V. and Rezaei,F.
EPI_ISL_2593200, EPI_ISL_2593201, EPI_ISL_2593202	Department of Epidemiology and Demography, Kenya Medical Research Institute (KEMRI) - Wellcome Trust Research Programme	Department of Epidemiology and Demography, Kenya Medical Research Institute (KEMRI) - Wellcome Trust Research Programme	Otieno,J.R., Agoti,C.N., Gitahi,C.W., Bett,A., Ngama,M., Medley,G.F., Cane,P.A. and Nokes,J.D.
EPI_ISL_2593203, EPI_ISL_2593204	Epidemiology and Demography, KEMRI-Wellcome Trust Research Collaborative Programme	Epidemiology and Demography, KEMRI-Wellcome Trust Research Collaborative Programme	Agoti,C.N., Mayieka,L., Otieno,J.R., Ahmed,J.A., Fields,B., Waiboci,L.B., Nyoka,R., Eidex,R., Morano,N., Burton,W., Montgomery,J., Breiman,R. and Nokes,D.J.
EPI_ISL_2593205	Department of Epidemiology and Demography, Kenya Medical Research Institute (KEMRI) - Wellcome Trust Research Programme	Department of Epidemiology and Demography, Kenya Medical Research Institute (KEMRI) - Wellcome Trust Research Programme	Otieno,J.R., Agoti,C.N., Gitahi,C.W., Bett,A., Ngama,M., Medley,G.F., Cane,P.A. and Nokes,J.D.
EPI_ISL_2593206	College of Medicine and Medical Research Institute, Chungbuk National University	College of Medicine and Medical Research Institute, Chungbuk National University	Baek,Y.H., Choi,E.H., Park,S.-J., Song,M.-S. and Choi,Y.-K.
EPI_ISL_2593207, EPI_ISL_2593208, EPI_ISL_2593209, EPI_ISL_2593210, EPI_ISL_2593211, EPI_ISL_2593212, EPI_ISL_2593213, EPI_ISL_2593214	Epidemiology and Demography, KEMRI-Wellcome Trust Research Collaborative Programme	Epidemiology and Demography, KEMRI-Wellcome Trust Research Collaborative Programme	Agoti,C.N., Mayieka,L., Otieno,J.R., Ahmed,J.A., Fields,B., Waiboci,L.B., Nyoka,R., Eidex,R., Morano,N., Burton,W., Montgomery,J., Breiman,R. and Nokes,D.J.
EPI_ISL_2593215	Department of Epidemiology and Demography, Kenya Medical Research Institute (KEMRI) - Wellcome Trust Research Programme	Department of Epidemiology and Demography, Kenya Medical Research Institute (KEMRI) - Wellcome Trust Research Programme	Otieno,J.R., Agoti,C.N., Gitahi,C.W., Bett,A., Ngama,M., Medley,G.F., Cane,P.A. and Nokes,J.D.
EPI_ISL_2593216, EPI_ISL_2593217, EPI_ISL_2593218, EPI_ISL_2593219, EPI_ISL_2593220, EPI_ISL_2593221, EPI_ISL_2593222, EPI_ISL_2593223, EPI_ISL_2593224	Epidemiology and Demography, KEMRI-Wellcome Trust Research Collaborative Programme	Epidemiology and Demography, KEMRI-Wellcome Trust Research Collaborative Programme	Agoti,C.N., Mayieka,L., Otieno,J.R., Ahmed,J.A., Fields,B., Waiboci,L.B., Nyoka,R., Eidex,R., Morano,N., Burton,W., Montgomery,J., Breiman,R. and Nokes,D.J.
EPI_ISL_2593225	College of Medicine and Medical Research Institute, Chungbuk National University	College of Medicine and Medical Research Institute, Chungbuk National University	Baek,Y.H., Choi,E.H., Park,S.-J., Song,M.-S. and Choi,Y.-K.
EPI_ISL_2593226, EPI_ISL_2593227, EPI_ISL_2593228	Epidemiology and Demography, KEMRI-Wellcome Trust Research Collaborative Programme	Epidemiology and Demography, KEMRI-Wellcome Trust Research Collaborative Programme	Agoti,C.N., Mayieka,L., Otieno,J.R., Ahmed,J.A., Fields,B., Waiboci,L.B., Nyoka,R., Eidex,R., Morano,N., Burton,W., Montgomery,J., Breiman,R. and Nokes,D.J.
EPI_ISL_2593229	Investigacion en Virologia y Biotecnologia, Instituto Conmemorativo Gorgas de Estudios de la Salud	Investigacion en Virologia y Biotecnologia, Instituto Conmemorativo Gorgas de Estudios de la Salud	Abrego,L., Delfraro,A., Franco,D., Castillo,C., Cano,M., Castillo,M., Castillo,J., Pascale,J. and Arbiza,J.
EPI_ISL_2593230, EPI_ISL_2593231, EPI_ISL_2593232	College of Medicine and Medical Research Institute, Chungbuk National University	College of Medicine and Medical Research Institute, Chungbuk National University	Baek,Y.H., Choi,E.H., Park,S.-J., Song,M.-S. and Choi,Y.-K.
EPI_ISL_2593233, EPI_ISL_2593234, EPI_ISL_2593235, EPI_ISL_2593236, EPI_ISL_2593237, EPI_ISL_2593238, EPI_ISL_2593239, EPI_ISL_2593240, EPI_ISL_2593241, EPI_ISL_2593242, EPI_ISL_2593243, EPI_ISL_2593244, EPI_ISL_2593245			
see above	Epidemiology and Demography, KEMRI-Wellcome Trust Research Collaborative Programme	Epidemiology and Demography, KEMRI-Wellcome Trust Research Collaborative Programme	Agoti,C.N., Mayieka,L., Otieno,J.R., Ahmed,J.A., Fields,B., Waiboci,L.B., Nyoka,R., Eidex,R., Morano,N., Burton,W., Montgomery,J., Breiman,R. and Nokes,D.J.
EPI_ISL_2593246	Department of Epidemiology and Demography, Kenya Medical Research Institute (KEMRI) - Wellcome Trust Research Programme	Department of Epidemiology and Demography, Kenya Medical Research Institute (KEMRI) - Wellcome Trust Research Programme	Otieno,J.R., Agoti,C.N., Gitahi,C.W., Bett,A., Ngama,M., Medley,G.F., Cane,P.A. and Nokes,J.D.
EPI_ISL_2593247, EPI_ISL_2593248	Epidemiology and Demography, KEMRI-Wellcome Trust Research Collaborative Programme	Epidemiology and Demography, KEMRI-Wellcome Trust Research Collaborative Programme	Agoti,C.N., Mayieka,L., Otieno,J.R., Ahmed,J.A., Fields,B., Waiboci,L.B., Nyoka,R., Eidex,R., Morano,N., Burton,W., Montgomery,J., Breiman,R. and Nokes,D.J.
EPI_ISL_2593249	Virology Division, Public Health Laboratory Services Branch, Centre for Health Protection, Department of Health	Virology Division, Public Health Laboratory Services Branch, Centre for Health Protection, Department of Health	Mak,G.C., Lau,C.S., Chiu,D.M.Y. and Lim,W.
EPI_ISL_2593250, EPI_ISL_2593251, EPI_ISL_2593252	Epidemiology and Demography, KEMRI-Wellcome Trust Research Collaborative Programme	Epidemiology and Demography, KEMRI-Wellcome Trust Research Collaborative Programme	Agoti,C.N., Mayieka,L., Otieno,J.R., Ahmed,J.A., Fields,B., Waiboci,L.B., Nyoka,R., Eidex,R., Morano,N., Burton,W., Montgomery,J., Breiman,R. and Nokes,D.J.
EPI_ISL_2593253, EPI_ISL_2593254, EPI_ISL_2593255	Department of Epidemiology and Demography, Kenya Medical Research Institute (KEMRI) - Wellcome Trust Research Programme	Department of Epidemiology and Demography, Kenya Medical Research Institute (KEMRI) - Wellcome Trust Research Programme	Otieno,J.R., Agoti,C.N., Gitahi,C.W., Bett,A., Ngama,M., Medley,G.F., Cane,P.A. and Nokes,J.D.
EPI_ISL_2593256	Epidemiology and Demography, KEMRI-Wellcome Trust Research Collaborative Programme	Epidemiology and Demography, KEMRI-Wellcome Trust Research Collaborative Programme	Agoti,C.N., Mayieka,L., Otieno,J.R., Ahmed,J.A., Fields,B., Waiboci,L.B., Nyoka,R., Eidex,R., Morano,N., Burton,W., Montgomery,J., Breiman,R. and Nokes,D.J.
EPI_ISL_2593257	Department of Epidemiology and Demography, Kenya Medical Research Institute (KEMRI) - Wellcome Trust Research Programme	Department of Epidemiology and Demography, Kenya Medical Research Institute (KEMRI) - Wellcome Trust Research Programme	Otieno,J.R., Agoti,C.N., Gitahi,C.W., Bett,A., Ngama,M., Medley,G.F., Cane,P.A. and Nokes,J.D.
EPI_ISL_2593258, EPI_ISL_2593259, EPI_ISL_2593260, EPI_ISL_2593261, EPI_ISL_2593262, EPI_ISL_2593263, EPI_ISL_2593264, EPI_ISL_2593265, EPI_ISL_2593266	Epidemiology and Demography, KEMRI-Wellcome Trust Research Collaborative Programme	Epidemiology and Demography, KEMRI-Wellcome Trust Research Collaborative Programme	Agoti,C.N., Mayieka,L., Otieno,J.R., Ahmed,J.A., Fields,B., Waiboci,L.B., Nyoka,R., Eidex,R., Morano,N., Burton,W., Montgomery,J., Breiman,R. and Nokes,D.J.
EPI_ISL_2593267	Department of Epidemiology and Demography, Kenya Medical Research Institute (KEMRI) - Wellcome Trust Research Programme	Department of Epidemiology and Demography, Kenya Medical Research Institute (KEMRI) - Wellcome Trust Research Programme	Otieno,J.R., Agoti,C.N., Gitahi,C.W., Bett,A., Ngama,M., Medley,G.F., Cane,P.A. and Nokes,J.D.
EPI_ISL_2593268, EPI_ISL_2593269, EPI_ISL_2593270, EPI_ISL_2593271, EPI_ISL_2593272, EPI_ISL_2593273, EPI_ISL_2593274, EPI_ISL_2593275	Epidemiology and Demography, KEMRI-Wellcome Trust Research Collaborative Programme	Epidemiology and Demography, KEMRI-Wellcome Trust Research Collaborative Programme	Agoti,C.N., Mayieka,L., Otieno,J.R., Ahmed,J.A., Fields,B., Waiboci,L.B., Nyoka,R., Eidex,R., Morano,N., Burton,W., Montgomery,J., Breiman,R. and Nokes,D.J.

We gratefully acknowledge the following Authors from the Originating laboratories responsible for obtaining the specimens, as well as the Submitting laboratories where the genome data were generated and shared via GISAID, on which this research is based.

All Submitters of data may be contacted directly via www.gisaid.org

Authors are sorted alphabetically.

Accession ID	Originating Laboratory	Submitting Laboratory	Authors
EPI_ISL_1074028, EPI_ISL_1074031, EPI_ISL_1074034, EPI_ISL_1074048, EPI_ISL_1074049, EPI_ISL_1074050, EPI_ISL_1074051, EPI_ISL_1074052, EPI_ISL_1074053, EPI_ISL_1074054, EPI_ISL_1074055, EPI_ISL_1074056, EPI_ISL_1074057, EPI_ISL_1074058, EPI_ISL_1074059, EPI_ISL_1074060, EPI_ISL_1074061, EPI_ISL_1074062, EPI_ISL_1074063, EPI_ISL_1074064, EPI_ISL_1074065, EPI_ISL_1074066, EPI_ISL_1074067, EPI_ISL_1074068, EPI_ISL_1074089, EPI_ISL_1074094, EPI_ISL_1074099, EPI_ISL_1074100, EPI_ISL_1074103, EPI_ISL_1074106, EPI_ISL_1074112, EPI_ISL_1074115, EPI_ISL_1074118, EPI_ISL_1074120, EPI_ISL_1074125, EPI_ISL_1074126, EPI_ISL_1074127, EPI_ISL_1074137, EPI_ISL_1074138, EPI_ISL_1074139, EPI_ISL_1074140, EPI_ISL_1074141, EPI_ISL_1074146, EPI_ISL_1074149, EPI_ISL_1074152, EPI_ISL_1074153, EPI_ISL_1074154, EPI_ISL_1074156, EPI_ISL_1074157, EPI_ISL_1074158, EPI_ISL_1074159, EPI_ISL_1074160, EPI_ISL_1074161, EPI_ISL_1074168, EPI_ISL_1074169, EPI_ISL_1074170, EPI_ISL_1074171, EPI_ISL_1074172, EPI_ISL_1074173, EPI_ISL_1074188, EPI_ISL_1074191, EPI_ISL_1074195, EPI_ISL_1074204, EPI_ISL_1074212, EPI_ISL_1074213, EPI_ISL_1074214, EPI_ISL_1074234, EPI_ISL_1074240, EPI_ISL_1074245, EPI_ISL_1074246, EPI_ISL_1074253, EPI_ISL_1074255, EPI_ISL_1074256, EPI_ISL_1074257, EPI_ISL_1074259, EPI_ISL_1074273, EPI_ISL_1074274, EPI_ISL_1074275, EPI_ISL_1074276, EPI_ISL_1074277, EPI_ISL_1074278, EPI_ISL_1074279, EPI_ISL_1074280, EPI_ISL_1074281, EPI_ISL_1074282			
see above	Virology Laboratory, Ricardo Gutiérrez Children's Hospital	Vanderbilt University Medical Center / Virology Laboratory, Ricardo Gutiérrez Children's Hospital	Goya, Stephanie; Lucion, Maria Florencia; Juarez, Maria del Valle; Shilts, Meghan; Gentile, Angela; Mistchenko, Alicia S.; Das, Suman# & Viegas, Mariana# (#contributed equally to this study)
EPI_ISL_10914989	VIC, Victorian Infectious Diseases Reference Laboratory	WHO Collaborating Centre for Reference and Research on Influenza	Xiaomin Dong, Annette Alafaci,Yi-Mo Deng, Naomi Komadina, Ammar Aziz
EPI_ISL_10954007, EPI_ISL_10954008	Respiratory Virus Unit, Specialised Microbiology and Laboratories Directorate, UK Health Security Agency	Respiratory Virus Unit, Specialised Microbiology and Laboratories Directorate, UK Health Security Agency	Zambon M, Talts T, Ellis J, Miah S, Platt S
EPI_ISL_11428309	Respiratory Virus Unit, Reference Services, UK Health Security Agency	Reference Services, UK Health Security Agency	Zambon M, Talts T, Ellis J, Miah S, Platt S
EPI_ISL_12544918, EPI_ISL_12544919	National Influenza Center, National Institute of Hygiene and Epidemiology (NIHE)	National Institute of Hygiene and Epidemiology (NIHE) National Influenza Centre, Virology Department	Ung Thi Hong Trang, Hoang Vu Mai Phuong, Nguyen Huy Hoang, Nguyen Le Khanh Hang, Le Thi Thanh, Nguyen Vu Son, Vuong Duc Cuong, Pham Thi Hien, Tran Thu Huong, Nguyen Co Thach, Nguyen Phuong Anh, Le Quynh Mai
EPI_ISL_12809004, EPI_ISL_12809056, EPI_ISL_12809166, EPI_ISL_12809168	ICMR-NATIONAL INSTITUTE OF VIROLOGY INFLUENZA	ICMR-National Institute of Virology Influenza	Dr. varsha potdar and NIC team
EPI_ISL_1437504, EPI_ISL_1437506, EPI_ISL_1437508, EPI_ISL_1437510	CEMIC	INEI-ANLIS "Carlos G. Malbrán". Servicio Virosis Respiratorias.	Elsa Baumeister, Andrea Pontoriero, Russo Mara, Martin Avaro, Estefania Benedetti, Ana Campos
EPI_ISL_1437512, EPI_ISL_1437514, EPI_ISL_1437516, EPI_ISL_1437518	Hospital Perrando	INEI-ANLIS "Carlos G. Malbrán". Servicio Virosis Respiratorias.	Elsa Baumeister, Andrea Pontoriero, Russo Mara, Martin Avaro, Estefania Benedetti, Ana Campos
EPI_ISL_1437520, EPI_ISL_1437522, EPI_ISL_1437523	Hospital de la Madre y el Niño	INEI-ANLIS "Carlos G. Malbrán". Servicio Virosis Respiratorias.	Elsa Baumeister, Andrea Pontoriero, Russo Mara, Martin Avaro, Estefania Benedetti, Ana Campos
EPI_ISL_1437525, EPI_ISL_1437527, EPI_ISL_1437529, EPI_ISL_1437538, EPI_ISL_1437539, EPI_ISL_1437541	Laboratorio de Salud Publica	INEI-ANLIS "Carlos G. Malbrán". Servicio Virosis Respiratorias.	Elsa Baumeister, Andrea Pontoriero, Russo Mara, Martin Avaro, Estefania Benedetti, Ana Campos
EPI_ISL_15055310, EPI_ISL_15055311, EPI_ISL_15055312, EPI_ISL_15055313, EPI_ISL_15055314, EPI_ISL_15067675, EPI_ISL_15067676, EPI_ISL_15067677, EPI_ISL_15067678, EPI_ISL_15067679	Virology Laboratory, Ricardo Gutiérrez Children's Hospital	Virology Laboratory, Ricardo Gutiérrez Children's Hospital	Acuña, Dolores; Goya, Stephanie; Nabaes Jodar, Mercedes S.; Lucion, M. Florencia; Juárez, María del Valle; Gentile, Ángela; Mistchenko, Alicia S.; Viegas, Mariana
EPI_ISL_15120692, EPI_ISL_15120694, EPI_ISL_15120703, EPI_ISL_15120706, EPI_ISL_15120713, EPI_ISL_15120715, EPI_ISL_15120716, EPI_ISL_15120717, EPI_ISL_15120719, EPI_ISL_15120721, EPI_ISL_15120723, EPI_ISL_15120726, EPI_ISL_15120727, EPI_ISL_15120728, EPI_ISL_15120729, EPI_ISL_15120730, EPI_ISL_15120738, EPI_ISL_15120739, EPI_ISL_15120743, EPI_ISL_15120745, EPI_ISL_15120770, EPI_ISL_15120771, EPI_ISL_15120772, EPI_ISL_15120773, EPI_ISL_15120774, EPI_ISL_15120775, EPI_ISL_15120776, EPI_ISL_15120777, EPI_ISL_15120778, EPI_ISL_15120780, EPI_ISL_15120781, EPI_ISL_15120782, EPI_ISL_15120783			
see above	National Institute of Hygiene, virology departement, National Influenza Center	Centers for Disease Control and Prevention, Coronavirus and Other Respiratory Viruses Division	Megha Aggarwal, Lijuan Wang, Ji In Park, Amanda Smith and Everardo Vega
EPI_ISL_15750183, EPI_ISL_15750184, EPI_ISL_15750185, EPI_ISL_15750186	Virology, Tehran University of Medical Sciences	Virology, Tehran University of Medical Sciences	Salimi,V., Tavakoli,F., Mokhtari-Azad,T., Ghavami,N.
EPI_ISL_15750506, EPI_ISL_15750507, EPI_ISL_15750508, EPI_ISL_15750509, EPI_ISL_15750510, EPI_ISL_15750511, EPI_ISL_15750512, EPI_ISL_15750513, EPI_ISL_15750514, EPI_ISL_15750515, EPI_ISL_15750516, EPI_ISL_15750517, EPI_ISL_15750518, EPI_ISL_15750519, EPI_ISL_15750520, EPI_ISL_15750521, EPI_ISL_15750522, EPI_ISL_15750523, EPI_ISL_15750524, EPI_ISL_15750525, EPI_ISL_15750526, EPI_ISL_15750527, EPI_ISL_15750528, EPI_ISL_15750529, EPI_ISL_15750530, EPI_ISL_15750531, EPI_ISL_15750532, EPI_ISL_15750533, EPI_ISL_15750534, EPI_ISL_15750535, EPI_ISL_15750536, EPI_ISL_15750537, EPI_ISL_15750538, EPI_ISL_15750539, EPI_ISL_15750540, EPI_ISL_15750541, EPI_ISL_15750542, EPI_ISL_15750543, EPI_ISL_15750544, EPI_ISL_15750545, EPI_ISL_15750546, EPI_ISL_15750547, EPI_ISL_15750548, EPI_ISL_15750549, EPI_ISL_15750550, EPI_ISL_15750551, EPI_ISL_15750552, EPI_ISL_15750553, EPI_ISL_15750554, EPI_ISL_15750555, EPI_ISL_15750556, EPI_ISL_15750557, EPI_ISL_15750558, EPI_ISL_15750559, EPI_ISL_15750560, EPI_ISL_15750561, EPI_ISL_15750562, EPI_ISL_15750563, EPI_ISL_15750564, EPI_ISL_15750565, EPI_ISL_15750566, EPI_ISL_15750567, EPI_ISL_15750568, EPI_ISL_15750569, EPI_ISL_15750570, EPI_ISL_15750571, EPI_ISL_15750572, EPI_ISL_15750573, EPI_ISL_15750574, EPI_ISL_15750575, EPI_ISL_15750576, EPI_ISL_15750577, EPI_ISL_15750578, EPI_ISL_15750579, EPI_ISL_15750580, EPI_ISL_15750581, EPI_ISL_15750582, EPI_ISL_15750583, EPI_ISL_15750584, EPI_ISL_15750585, EPI_ISL_15750586, EPI_ISL_15750587, EPI_ISL_15750588, EPI_ISL_15750589, EPI_ISL_15750590, EPI_ISL_15750591, EPI_ISL_15750592, EPI_ISL_15750593, EPI_ISL_15750594, EPI_ISL_15750595			
see above	Department of Pediatrics, Center of Excellence in Clinical Virology	Department of Pediatrics, Center of Excellence in Clinical Virology	Thongpan,I.
EPI_ISL_15750702, EPI_ISL_15750703, EPI_ISL_15750704, EPI_ISL_15750705, EPI_ISL_15750706, EPI_ISL_15750707, EPI_ISL_15750708, EPI_ISL_15750709, EPI_ISL_15750710, EPI_ISL_15750711, EPI_ISL_15750712, EPI_ISL_15750713, EPI_ISL_15750714, EPI_ISL_15750715, EPI_ISL_15750716, EPI_ISL_15750717, EPI_ISL_15750718, EPI_ISL_15750719, EPI_ISL_15750720, EPI_ISL_15750721, EPI_ISL_15750722, EPI_ISL_15750723, EPI_ISL_15750724, EPI_ISL_15750725, EPI_ISL_15750726, EPI_ISL_15750727, EPI_ISL_15750728, EPI_ISL_15750729, EPI_ISL_15750730, EPI_ISL_15750731, EPI_ISL_15750732, EPI_ISL_15750733, EPI_ISL_15750734, EPI_ISL_15750736, EPI_ISL_15750741, EPI_ISL_15750742, EPI_ISL_15750743, EPI_ISL_15750744, EPI_ISL_15750745, EPI_ISL_15750746, EPI_ISL_15750747, EPI_ISL_15750748, EPI_ISL_15750749, EPI_ISL_15750750, EPI_ISL_15750751, EPI_ISL_15750766			
see above	Beijing Center for Disease Prevention and Control, Immunological Prevention	Beijing Center for Disease Prevention and Control, Immunological Prevention	Luo,Q., Li,M., Huang,F.
EPI_ISL_15750840, EPI_ISL_15750841, EPI_ISL_15750842, EPI_ISL_15750843, EPI_ISL_15750844	State Key Laboratory of Pathogen and Biosecurity, Beijing Institute of Microbiology and Epidemiology	State Key Laboratory of Pathogen and Biosecurity, Beijing Institute of Microbiology and Epidemiology	Jia,N., Cao,W.-C., Hu,Y.-L., Ye,R.-Z., Que,T.-C., Xia,L.-Y., Cui,X.-M., Zhang,Y.-W., Jiang,J.-F., Wang,Q.-H., Wang,Q., Jia,N., Cao,W.-C., Hu,Y.-L., Ye,R.-Z., Que,T.-C., Xia,L.-Y., Cui,X.-M., Zhang,Y.-W., Jiang,J.-F., Wang,Q.-H., Wang,Q.
EPI_ISL_15751004, EPI_ISL_15751005, EPI_ISL_15751006, EPI_ISL_15751007, EPI_ISL_15751008, EPI_ISL_15751009, EPI_ISL_15751010, EPI_ISL_15751011, EPI_ISL_15751012, EPI_ISL_15751013, EPI_ISL_15751014, EPI_ISL_15751015, EPI_ISL_15751016, EPI_ISL_15751017, EPI_ISL_15751018, EPI_ISL_15751019, EPI_ISL_15751020, EPI_ISL_15751021, EPI_ISL_15751022, EPI_ISL_15751023, EPI_ISL_15751024, EPI_ISL_15751025, EPI_ISL_15751026, EPI_ISL_15751027, EPI_ISL_15751028, EPI_ISL_15751029, EPI_ISL_15751030, EPI_ISL_15751031, EPI_ISL_15751032, EPI_ISL_15751033, EPI_ISL_15751034, EPI_ISL_15751035, EPI_ISL_15751036, EPI_ISL_15751037, EPI_ISL_15751038, EPI_ISL_15751039, EPI_ISL_15751040, EPI_ISL_15751041, EPI_ISL_15751042, EPI_ISL_15751043, EPI_ISL_15751044, EPI_ISL_15751045, EPI_ISL_15751046, EPI_ISL_15751047, EPI_ISL_15751048, EPI_ISL_15751049, EPI_ISL_15751050, EPI_ISL_15751051, EPI_ISL_15751052, EPI_ISL_15751053, EPI_ISL_15751054, EPI_ISL_15751055, EPI_ISL_15751056, EPI_ISL_15751057, EPI_ISL_15751058, EPI_ISL_15751059, EPI_ISL_15751060, EPI_ISL_15751061, EPI_ISL_15751062, EPI_ISL_15751063, EPI_ISL_15751064, EPI_ISL_15751065, EPI_ISL_15751066, EPI_ISL_15751067, EPI_ISL_15751068, EPI_ISL_15751077, EPI_ISL_15751078, EPI_ISL_15751079, EPI_ISL_15751080, EPI_ISL_15751081, EPI_ISL_15751082, EPI_ISL_15751083, EPI_ISL_15751084, EPI_ISL_15751085, EPI_ISL_15751086, EPI_ISL_15751087, EPI_ISL_15751088, EPI_ISL_15751089, EPI_ISL_15751090, EPI_ISL_15751091			
see above	Beijing Center for Disease Prevention and Control, Immunological Prevention	Beijing Center for Disease Prevention and Control, Immunological Prevention	Luo,Q., Li,M., Huang,F.
EPI_ISL_15751153, EPI_ISL_15751154, EPI_ISL_15751155, EPI_ISL_15751156, EPI_ISL_15751157, EPI_ISL_15751158, EPI_ISL_15751159, EPI_ISL_15751160, EPI_ISL_15751161, EPI_ISL_15751162, EPI_ISL_15751163, EPI_ISL_15751164, EPI_ISL_15751165, EPI_ISL_15751166, EPI_ISL_15751167, EPI_ISL_15751168, EPI_ISL_15751169, EPI_ISL_15751170, EPI_ISL_15751171			
see above	Molecular Virology, Johns Hopkins School of Medicine	Molecular Virology, Johns Hopkins School of Medicine	Fall A, Elawar F, Hodcroft EB, Jallow MM, Toure CT, Barry MA, Kiori DE, Sy S, Diaw Y, Goudiaby D, Niang MN, Dia N, Fall,A., Jallow,M.M., Toure,C.T., Kiori,D.E., sy,S., Diaw,Y., Goudiaby,D., Niang,M.N., Dia,N.
EPI_ISL_15751232, EPI_ISL_15751233, EPI_ISL_15751236, EPI_ISL_15751237, EPI_ISL_15751238, EPI_ISL_15751239, EPI_ISL_15751240, EPI_ISL_15751241	Influenza Group, National Institute of Virology	Influenza Group, National Institute of Virology	Choudhary,M.L., Chaudhary,U., Choudhary,M.L., Chaudhary,U.
EPI_ISL_15752140, EPI_ISL_15752141, EPI_ISL_15752142, EPI_ISL_15752143, EPI_ISL_15752144, EPI_ISL_15752145, EPI_ISL_15752146, EPI_ISL_15752147, EPI_ISL_15752148, EPI_ISL_15752149, EPI_ISL_15752150, EPI_ISL_15752151, EPI_ISL_15752152, EPI_ISL_15752153, EPI_ISL_15752154, EPI_ISL_15752155, EPI_ISL_15752156, EPI_ISL_15752157, EPI_ISL_15752158, EPI_ISL_15752159, EPI_ISL_15752160, EPI_ISL_15752161, EPI_ISL_15752162, EPI_ISL_15752163, EPI_ISL_15752164, EPI_ISL_15752165, EPI_ISL_15752166, EPI_ISL_15752167, EPI_ISL_15752168, EPI_ISL_15752169, EPI_ISL_15752170, EPI_ISL_15752171, EPI_ISL_15752172, EPI_ISL_15752173, EPI_ISL_15752174, EPI_ISL_15752175, EPI_ISL_15752176, EPI_ISL_15752177, EPI_ISL_15752178, EPI_ISL_15752179, EPI_ISL_15752180, EPI_ISL_15752181, EPI_ISL_15752182, EPI_ISL_15752183, EPI_ISL_15752184, EPI_ISL_15752185, EPI_ISL_15752186, EPI_ISL_15752187, EPI_ISL_15752188, EPI_ISL_15752189, EPI_ISL_15752190, EPI_ISL_15752191, EPI_ISL_15752192, EPI_ISL_15752194, EPI_ISL_15752195, EPI_ISL_15752196, EPI_ISL_15752197, EPI_ISL_15752198, EPI_ISL_15752199, EPI_ISL_15752200, EPI_ISL_15752201, EPI_ISL_15752202, EPI_ISL_15752203, EPI_ISL_15752204, EPI_ISL_15752205, EPI_ISL_15752206, EPI_ISL_15752207, EPI_ISL_15752208, EPI_ISL_15752209, EPI_ISL_15752210, EPI_ISL_15752211, EPI_ISL_15752212, EPI_ISL_15752213, EPI_ISL_15752214, EPI_ISL_15752215, EPI_ISL_15752216, EPI_ISL_15752217, EPI_ISL_15752218, EPI_ISL_15752219, EPI_ISL_15752220, EPI_ISL_15752221, EPI_ISL_15752222, EPI_ISL_15752223, EPI_ISL_15752224, EPI_ISL_15752225, EPI_ISL_15752226, EPI_ISL_15752227, EPI_ISL_15752228, EPI_ISL_15752229, EPI_ISL_15752230, EPI_ISL_15752231, EPI_ISL_15752232, EPI_ISL_15752233, EPI_ISL_15752234, EPI_ISL_15752235, EPI_ISL_15752236, EPI_ISL_15752237, EPI_ISL_15752238, EPI_ISL_15752239, EPI_ISL_15752240, EPI_ISL_15752241, EPI_ISL_15752242, EPI_ISL_15752243, EPI_ISL_15752244, EPI_ISL_15752245, EPI_ISL_15752246, EPI_ISL_15752247, EPI_ISL_15752248, EPI_ISL_15752249, EPI_ISL_15752250, EPI_ISL_15752251, EPI_ISL_15752252, EPI_ISL_15752253, EPI_ISL_15752254, EPI_ISL_15752255, EPI_ISL_15752256, EPI_ISL_15752257, EPI_ISL_15752258, EPI_ISL_15752259, EPI_ISL_15752260, EPI_ISL_15752261, EPI_ISL_15752262, EPI_ISL_15752263, EPI_ISL_15752264, EPI_ISL_15752265, EPI_ISL_15752266			
see above	Microbial Sciences, AstraZeneca	Microbial Sciences, AstraZeneca	Tabor,D.E., Fernandes,F., Langedijk,A.C., Wilkins,D., Lebbink,R.J., Tovchigrechko,A., Ruzin,A., Kragten-Tabatabaie,L., Jin,H., Esser,M.T., Bont,L.J., Abram,M.E., Tabor,D.
EPI_ISL_15752719, EPI_ISL_15752720, EPI_ISL_15752723, EPI_ISL_15752724, EPI_ISL_15752725, EPI_ISL_15752726, EPI_ISL_15752727, EPI_ISL_15752728, EPI_ISL_15752734, EPI_ISL_15752736, EPI_ISL_15752737, EPI_ISL_15752738, EPI_ISL_15752739, EPI_ISL_15752740, EPI_ISL_15752742, EPI_ISL_15752744, EPI_ISL_15752745, EPI_ISL_15752747, EPI_ISL_15752749, EPI_ISL_15752750, EPI_ISL_15752751, EPI_ISL_15752753, EPI_ISL_15752754, EPI_ISL_15752755, EPI_ISL_15752756, EPI_ISL_15752757, EPI_ISL_15752758, EPI_ISL_15752759, EPI_ISL_15752760, EPI_ISL_15752761, EPI_ISL_15752762, EPI_ISL_15752764, EPI_ISL_15752765, EPI_ISL_15752766, EPI_ISL_15752767, EPI_ISL_15752769, EPI_ISL_15752771, EPI_ISL_15752772, EPI_ISL_15752774, EPI_ISL_15752777, EPI_ISL_15752778, EPI_ISL_15752779, EPI_ISL_15752780, EPI_ISL_15752781, EPI_ISL_15752784, EPI_ISL_15752785, EPI_ISL_15752789, EPI_ISL_15752790, EPI_ISL_15752791, EPI_ISL_15752792, EPI_ISL_15752793, EPI_ISL_15752794, EPI_ISL_15752795, EPI_ISL_15752796, EPI_ISL_15752797, EPI_ISL_15752798, EPI_ISL_15752803, EPI_ISL_15752804, EPI_ISL_15752805, EPI_ISL_15752807, EPI_ISL_15752808, EPI_ISL_15752810, EPI_ISL_15752811, EPI_ISL_15752812, EPI_ISL_15752816, EPI_ISL_15752819, EPI_ISL_15752820, EPI_ISL_15752822, EPI_ISL_15752823, EPI_ISL_15752825, EPI_ISL_15752828, EPI_ISL_15752829, EPI_ISL_15752830, EPI_ISL_15752831, EPI_ISL_15752833, EPI_ISL_15752834, EPI_ISL_15752835, EPI_ISL_15752836, EPI_ISL_15752841, EPI_ISL_15752847, EPI_ISL_15752851, EPI_ISL_15752857, EPI_ISL_15752858, EPI_ISL_15752865, EPI_ISL_15752868, EPI_ISL_15752869			
see above	Pneumococcal Research Group, Infections and Immunity	Pneumococcal Research Group, Infections and Immunity	Anderson,J., Oeum,M., Verkolf,E., Licciardi,P.V., Mulholland,K., Nguyen,C., Chow,K., Waller,G., Costa,A.M., Daley,A., Crawford,N.W., Babl,F.E., Duke,T., Do,L.A.H., Wurzel,D., Anderson,J., Oeum,M., Verkolf,E., Licciardi,P.V., Mulholland,K.E., Nguyen,C., Chow,K., Waller,G., Costa,A.-M., Daley,A.J., Crawford,N., Babl,F., Duke,T., Do,L.A.H., Wurzel,D.
EPI_ISL_15753258, EPI_ISL_15753267, EPI_ISL_15753268, EPI_ISL_15753269, EPI_ISL_15753270, EPI_ISL_15753275, EPI_ISL_15753276, EPI_ISL_15753277, EPI_ISL_15753281, EPI_ISL_15753283, EPI_ISL_15753286, EPI_ISL_15753287, EPI_ISL_15753288, EPI_ISL_15753289, EPI_ISL_15753296, EPI_ISL_15753298, EPI_ISL_15753299, EPI_ISL_15753301, EPI_ISL_15753302, EPI_ISL_15753306, EPI_ISL_15753308, EPI_ISL_15753317, EPI_ISL_15753322, EPI_ISL_15753328, EPI_ISL_15753330, EPI_ISL_15753337, EPI_ISL_15753341, EPI_ISL_15753342, EPI_ISL_15753348, EPI_ISL_15753349, EPI_ISL_15753353, EPI_ISL_15753354, EPI_ISL_15753355, EPI_ISL_15753360, EPI_ISL_15753361, EPI_ISL_15753364, EPI_ISL_15753365, EPI_ISL_15753367, EPI_ISL_15753368, EPI_ISL_15753369, EPI_ISL_15753373, EPI_ISL_15753374, EPI_ISL_15753376, EPI_ISL_15753378, EPI_ISL_15753380, EPI_ISL_15753381, EPI_ISL_15753383, EPI_ISL_15753388, EPI_ISL_15753389, EPI_ISL_15753392, EPI_ISL_15753393, EPI_ISL_15753396, EPI_ISL_15753398, EPI_ISL_15753403, EPI_ISL_15753405, EPI_ISL_15753412, EPI_ISL_15753413, EPI_ISL_15753415, EPI_ISL_15753417, EPI_ISL_15753418, EPI_ISL_15753423, EPI_ISL_15753425, EPI_ISL_15753426, EPI_ISL_15753427, EPI_ISL_15753428, EPI_ISL_15753431, EPI_ISL_15753432, EPI_ISL_15753434, EPI_ISL_15753435, EPI_ISL_15753440, EPI_ISL_15753442, EPI_ISL_15753443, EPI_ISL_15753444, EPI_ISL_15753450, EPI_ISL_15753453, EPI_ISL_15753455, EPI_ISL_15753458, EPI_ISL_15753463, EPI_ISL_15753464, EPI_ISL_15753466, EPI_ISL_15753468, EPI_ISL_15753469, EPI_ISL_15753479, EPI_ISL_15753481, EPI_ISL_15753488, EPI_ISL_15753493, EPI_ISL_15753495, EPI_ISL_15753497, EPI_ISL_15753501, EPI_ISL_15753507, EPI_ISL_15753511, EPI_ISL_15753514, EPI_ISL_15753518, EPI_ISL_15753523, EPI_ISL_15753527, EPI_ISL_15753532, EPI_ISL_15753534, EPI_ISL_15753536, EPI_ISL_15753537, EPI_ISL_15753544, EPI_ISL_15753546, EPI_ISL_15753549, EPI_ISL_15753553, EPI_ISL_15753556, EPI_ISL_15753558, EPI_ISL_15753561, EPI_ISL_15753565, EPI_ISL_15753566, EPI_ISL_15753569			
see above	Department of Paediatrics, University of Oxford	Department of Paediatrics, University of Oxford	Lin,G.-L., Drysdale,S.B., Snape,M.D., O'Connor,D., Brown,A., MacIntyre-Cockett,G., Mellado-Gomez,E., de Cesare,M., Bonsall,D., Ansari,M.A., Oner,D., Aerssens,J., Butler,C., Bont,L., Openshaw,P., Martinon-Torres,F., Nair,H., Bowden,R., Golubchik,T., Pollard,A.J., Lin,G.-L.
EPI_ISL_15753581, EPI_ISL_15753582, EPI_ISL_15753584, EPI_ISL_15753585, EPI_ISL_15753586, EPI_ISL_15753587	Clinical Laboratory, Zhongshan Center for Disease Control and Prevention	Clinical Laboratory, Zhongshan Center for Disease Control and Prevention	Lin,J., Wu,Y., Shi,W., Yang,S., Xie,Y.
EPI_ISL_15820233, EPI_ISL_15820234, EPI_ISL_15820235, EPI_ISL_15820236	University of Ghana, Medical Laboratory Sciences	University of Ghana, Medical Laboratory Sciences	Kafintu-Kwashie,A.A., Nii-Trebi,N.I., Obodai,E., Neizer,M., Adiku,T.K., Odoom,J.K., Nii-Trebi,N.I., Kafintu-Kwashie,A.A., Obodai,E., Neizer,M., Adiku,T.K., Odoom,J.K.
EPI_ISL_16051848, EPI_ISL_16051850, EPI_ISL_16051851, EPI_ISL_16051852, EPI_ISL_16051853, EPI_ISL_16051854, EPI_ISL_16051855, EPI_ISL_16051856, EPI_ISL_16051857, EPI_ISL_16051858, EPI_ISL_16051859, EPI_ISL_16051860, EPI_ISL_16051863, EPI_ISL_16051864, EPI_ISL_16051865, EPI_ISL_16051866, EPI_ISL_16051869, EPI_ISL_16051870, EPI_ISL_16051871, EPI_ISL_16051872, EPI_ISL_16051874, EPI_ISL_16051875, EPI_ISL_16051876, EPI_ISL_16051877, EPI_ISL_16051878, EPI_ISL_16051879, EPI_ISL_16051880, EPI_ISL_16051881, EPI_ISL_16051882, EPI_ISL_16051883, EPI_ISL_16051884, EPI_ISL_16051889, EPI_ISL_16282720, EPI_ISL_16282721, EPI_ISL_16282722, EPI_ISL_16282724, EPI_ISL_16282725, EPI_ISL_16282726, EPI_ISL_16282727, EPI_ISL_16282728, EPI_ISL_16282729, EPI_ISL_16282736, EPI_ISL_16282737, EPI_ISL_16282741, EPI_ISL_16282742, EPI_ISL_16282743, EPI_ISL_16282747, EPI_ISL_16282748, EPI_ISL_16282749, EPI_ISL_16282750, EPI_ISL_16282751			
see above	National Influenza Center, National Institute of Hygiene and Epidemiology (NIHE)	National Influenza Center, National Institute of Hygiene and Epidemiology (NIHE)	Ung Thi Hong Trang, Hoang Vu Mai Phuong, Nguyen Huy Hoang, Nguyen Le Khanh Hang, Le Thi Quynh Mai
EPI_ISL_1647456, EPI_ISL_1647457, EPI_ISL_1647458, EPI_ISL_1647459, EPI_ISL_1647460, EPI_ISL_1647461, EPI_ISL_1647462, EPI_ISL_1647463, EPI_ISL_1647464, EPI_ISL_1647465, EPI_ISL_1647466, EPI_ISL_1647467, EPI_ISL_1647468, EPI_ISL_1647469			

see above	Instituto Nacional de Saúde	WHO Influenza Centre for Reference and Research on Influenza	Angela Todd, Yi-Mo Deng, Almiro Rogerio Tivane, Naomi Komadina
EPI_ISL_16905445, EPI_ISL_16905447, EPI_ISL_16905448	National Institute of Hygiene, virology departement, National Influenza Center	Centers for Disease Control and Prevention, Coronavirus and Other Respiratory Viruses Division	Megha Aggarwal, Lijuan Wang, Ji In Park, Amanda Smith and Everardo Vega
EPI_ISL_16982627, EPI_ISL_16982628, EPI_ISL_16982632, EPI_ISL_16982633	Chinese Center for Disease Control and Prevention	Chinese Center for Disease Control and Prevention	Jiang,Y.
EPI_ISL_17167889, EPI_ISL_17168075, EPI_ISL_17168076, EPI_ISL_17168077, EPI_ISL_17168078, EPI_ISL_17168079, EPI_ISL_17168086, EPI_ISL_17168087, EPI_ISL_17168088, EPI_ISL_17168094, EPI_ISL_17168096, EPI_ISL_17168097, EPI_ISL_17168098, EPI_ISL_17168099, EPI_ISL_17168100, EPI_ISL_17168101, EPI_ISL_17168102, EPI_ISL_17168103, EPI_ISL_17168104, EPI_ISL_17168105, EPI_ISL_17168106, EPI_ISL_17168107, EPI_ISL_17168108, EPI_ISL_17168109, EPI_ISL_17168110, EPI_ISL_17168112, EPI_ISL_17168113, EPI_ISL_17168114, EPI_ISL_17168115, EPI_ISL_17168116, EPI_ISL_17168117, EPI_ISL_17168121, EPI_ISL_17168122, EPI_ISL_17168123, EPI_ISL_17168124, EPI_ISL_17168125, EPI_ISL_17168126, EPI_ISL_17168127, EPI_ISL_17168128, EPI_ISL_17168129, EPI_ISL_17168130, EPI_ISL_17168131, EPI_ISL_17168132, EPI_ISL_17168133, EPI_ISL_17168134, EPI_ISL_17168135, EPI_ISL_17168136, EPI_ISL_17168137, EPI_ISL_17168138, EPI_ISL_17168139, EPI_ISL_17168140, EPI_ISL_17168141, EPI_ISL_17168142, EPI_ISL_17168143, EPI_ISL_17168151, EPI_ISL_17168152, EPI_ISL_17168154, EPI_ISL_17168163, EPI_ISL_17168164, EPI_ISL_17168226, EPI_ISL_17168227, EPI_ISL_17168228, EPI_ISL_17168229, EPI_ISL_17168230, EPI_ISL_17168232			
see above	National Institute for Viral Disease Control and Prevention	National Institute for Viral Disease Control and Prevention	Song,J., Zhu,Z., Song,J., Mao,N., Cui,A., Xu,W. and Zhang,Y.
EPI_ISL_17213018, EPI_ISL_17213019, EPI_ISL_17213020, EPI_ISL_17213021, EPI_ISL_17213022, EPI_ISL_17213023, EPI_ISL_17213024, EPI_ISL_17213025, EPI_ISL_17213026, EPI_ISL_17213027, EPI_ISL_17213028, EPI_ISL_17213029, EPI_ISL_17213030, EPI_ISL_17213031, EPI_ISL_17213032, EPI_ISL_17213033, EPI_ISL_17213034, EPI_ISL_17213035, EPI_ISL_17213036, EPI_ISL_17213037, EPI_ISL_17213038, EPI_ISL_17213039, EPI_ISL_17213040, EPI_ISL_17213041, EPI_ISL_17213042, EPI_ISL_17213043, EPI_ISL_17213045, EPI_ISL_17213046, EPI_ISL_17213047, EPI_ISL_17213048, EPI_ISL_17213049, EPI_ISL_17213050, EPI_ISL_17213051, EPI_ISL_17213052, EPI_ISL_17213053, EPI_ISL_17213054, EPI_ISL_17213055, EPI_ISL_17213056, EPI_ISL_17213057, EPI_ISL_17213058, EPI_ISL_17213059, EPI_ISL_17213060, EPI_ISL_17213061, EPI_ISL_17213062, EPI_ISL_17213063, EPI_ISL_17213064, EPI_ISL_17213065, EPI_ISL_17213066			
see above	Molecular Biology/Microbiology Research Laboratory. Universidade Federal do Paraná	Molecular Biology/Microbiology Research Laboratory. Universidade Federal do Paraná	Jucelia Stadinicki, Maria do Carmo Debur, Heloisa Ihle Garcia Giamberardino, Sonia Mara Raboni
EPI_ISL_17215837, EPI_ISL_17215842, EPI_ISL_17215845, EPI_ISL_17215847, EPI_ISL_17215848, EPI_ISL_17215855, EPI_ISL_17215858, EPI_ISL_17215862, EPI_ISL_17215864, EPI_ISL_17215866, EPI_ISL_17215867, EPI_ISL_17215873, EPI_ISL_17215879, EPI_ISL_17215881, EPI_ISL_17215885, EPI_ISL_17215888, EPI_ISL_17215889, EPI_ISL_17215896, EPI_ISL_17215899, EPI_ISL_17215900, EPI_ISL_17215901, EPI_ISL_17215911, EPI_ISL_17215912, EPI_ISL_17215918, EPI_ISL_17215921, EPI_ISL_17215926, EPI_ISL_17215928, EPI_ISL_17215929, EPI_ISL_17215930, EPI_ISL_17215933, EPI_ISL_17215944, EPI_ISL_17215945, EPI_ISL_17215951, EPI_ISL_17215958, EPI_ISL_17215963, EPI_ISL_17215966, EPI_ISL_17215970, EPI_ISL_17215973, EPI_ISL_17215976, EPI_ISL_17215978, EPI_ISL_17215982, EPI_ISL_17215991, EPI_ISL_17215998, EPI_ISL_17215999, EPI_ISL_17216000, EPI_ISL_17216001, EPI_ISL_17216002, EPI_ISL_17216004, EPI_ISL_17216005, EPI_ISL_17216006, EPI_ISL_17216010, EPI_ISL_17216015, EPI_ISL_17216019, EPI_ISL_17216022, EPI_ISL_17216023, EPI_ISL_17216029, EPI_ISL_17216031, EPI_ISL_17216035, EPI_ISL_17216041, EPI_ISL_17216045, EPI_ISL_17216046, EPI_ISL_17216047, EPI_ISL_17216051, EPI_ISL_17216056, EPI_ISL_17216064, EPI_ISL_17216065, EPI_ISL_17216071, EPI_ISL_17216083, EPI_ISL_17216087, EPI_ISL_17216088, EPI_ISL_17216091, EPI_ISL_17216093, EPI_ISL_17216098, EPI_ISL_17216101, EPI_ISL_17216102, EPI_ISL_17216104, EPI_ISL_17216115, EPI_ISL_17216116, EPI_ISL_17216121, EPI_ISL_17216123, EPI_ISL_17216125, EPI_ISL_17216127, EPI_ISL_17216132, EPI_ISL_17216138, EPI_ISL_17216147, EPI_ISL_17216148, EPI_ISL_17216157, EPI_ISL_17216161, EPI_ISL_17216162, EPI_ISL_17216163, EPI_ISL_17216165, EPI_ISL_17216170, EPI_ISL_17216172, EPI_ISL_17216174, EPI_ISL_17216178, EPI_ISL_17216184, EPI_ISL_17216192, EPI_ISL_17216193, EPI_ISL_17216200, EPI_ISL_17216205, EPI_ISL_17216214, EPI_ISL_17216228, EPI_ISL_17216230, EPI_ISL_17216233, EPI_ISL_17216238, EPI_ISL_17216239, EPI_ISL_17216250, EPI_ISL_17216251, EPI_ISL_17216252, EPI_ISL_17216256, EPI_ISL_17216258, EPI_ISL_17216260, EPI_ISL_17216265, EPI_ISL_17216266, EPI_ISL_17216267, EPI_ISL_17216277, EPI_ISL_17216289, EPI_ISL_17216332, EPI_ISL_17216338, EPI_ISL_17216345, EPI_ISL_17216358, EPI_ISL_17216366, EPI_ISL_17216367, EPI_ISL_17216371, EPI_ISL_17216372, EPI_ISL_17216376, EPI_ISL_17216377, EPI_ISL_17216378, EPI_ISL_17216388, EPI_ISL_17216389, EPI_ISL_17216392, EPI_ISL_17216404, EPI_ISL_17216405, EPI_ISL_17216406, EPI_ISL_17216417, EPI_ISL_17216425, EPI_ISL_17216427, EPI_ISL_17216432, EPI_ISL_17216436, EPI_ISL_17216437, EPI_ISL_17216445, EPI_ISL_17216449, EPI_ISL_17216453, EPI_ISL_17216459, EPI_ISL_17216461, EPI_ISL_17216465, EPI_ISL_17216470, EPI_ISL_17216472, EPI_ISL_17216477, EPI_ISL_17216481, EPI_ISL_17216482, EPI_ISL_17216484, EPI_ISL_17216486, EPI_ISL_17216492, EPI_ISL_17216496, EPI_ISL_17216497, EPI_ISL_17216499, EPI_ISL_17216504, EPI_ISL_17216507, EPI_ISL_17216510, EPI_ISL_17216512, EPI_ISL_17216513, EPI_ISL_17216517, EPI_ISL_17216522, EPI_ISL_17216524, EPI_ISL_17216525, EPI_ISL_17216526, EPI_ISL_17216527, EPI_ISL_17216528, EPI_ISL_17216537, EPI_ISL_17216538, EPI_ISL_17216542, EPI_ISL_17216546, EPI_ISL_17216552, EPI_ISL_17216557, EPI_ISL_17216563, EPI_ISL_17216565, EPI_ISL_17216567, EPI_ISL_17216572, EPI_ISL_17216573, EPI_ISL_17216576, EPI_ISL_17216579, EPI_ISL_17216580, EPI_ISL_17216581, EPI_ISL_17216586, EPI_ISL_17216593, EPI_ISL_17216594, EPI_ISL_17216595, EPI_ISL_17216597, EPI_ISL_17216605, EPI_ISL_17216610, EPI_ISL_17216612, EPI_ISL_17216614, EPI_ISL_17216621, EPI_ISL_17216622, EPI_ISL_17216628, EPI_ISL_17216629, EPI_ISL_17216635, EPI_ISL_17216639, EPI_ISL_17216642, EPI_ISL_17216644, EPI_ISL_17216645, EPI_ISL_17216653, EPI_ISL_17216657, EPI_ISL_17216662, EPI_ISL_17216676, EPI_ISL_17216680, EPI_ISL_17216689, EPI_ISL_17216690, EPI_ISL_17216696, EPI_ISL_17216700, EPI_ISL_17216707, EPI_ISL_17216711, EPI_ISL_17216712, EPI_ISL_17216714, EPI_ISL_17216720, EPI_ISL_17216723, EPI_ISL_17216726, EPI_ISL_17216729, EPI_ISL_17216731, EPI_ISL_17216736, EPI_ISL_17216749, EPI_ISL_17216775, EPI_ISL_17216777, EPI_ISL_17216781, EPI_ISL_17216785, EPI_ISL_17216786, EPI_ISL_17216788, EPI_ISL_17216789, EPI_ISL_17216792, EPI_ISL_17216797, EPI_ISL_17216805, EPI_ISL_17216816, EPI_ISL_17216821, EPI_ISL_17216824, EPI_ISL_17216833, EPI_ISL_17216834, EPI_ISL_17216844, EPI_ISL_17216853, EPI_ISL_17216854, EPI_ISL_17216856, EPI_ISL_17216858, EPI_ISL_17216860, EPI_ISL_17216861, EPI_ISL_17216865, EPI_ISL_17216870, EPI_ISL_17216871, EPI_ISL_17216873, EPI_ISL_17216881, EPI_ISL_17216883, EPI_ISL_17216885, EPI_ISL_17216894, EPI_ISL_17216898, EPI_ISL_17216900, EPI_ISL_17216902, EPI_ISL_17216903, EPI_ISL_17216907, EPI_ISL_17216914, EPI_ISL_17216915, EPI_ISL_17216923, EPI_ISL_17216924, EPI_ISL_17216932, EPI_ISL_17216934, EPI_ISL_17216936, EPI_ISL_17216946, EPI_ISL_17216948, EPI_ISL_17216957, EPI_ISL_17216958, EPI_ISL_17216965, EPI_ISL_17216970, EPI_ISL_17216971, EPI_ISL_17216979, EPI_ISL_17216985, EPI_ISL_17216986, EPI_ISL_17216989, EPI_ISL_17216992, EPI_ISL_17216995, EPI_ISL_17216996, EPI_ISL_17217000, EPI_ISL_17217004, EPI_ISL_17217005, EPI_ISL_17217011, EPI_ISL_17217014, EPI_ISL_17217015, EPI_ISL_17217026, EPI_ISL_17217027, EPI_ISL_17217028, EPI_ISL_17217029, EPI_ISL_17217034, EPI_ISL_17217042, EPI_ISL_17217046, EPI_ISL_17217047, EPI_ISL_17217051, EPI_ISL_17217058, EPI_ISL_17217063, EPI_ISL_17217067, EPI_ISL_17217069, EPI_ISL_17217070, EPI_ISL_17217073, EPI_ISL_17217074, EPI_ISL_17217083, EPI_ISL_17217087, EPI_ISL_17217088, EPI_ISL_17217091, EPI_ISL_17217094, EPI_ISL_17217098, EPI_ISL_17217101, EPI_ISL_17217110, EPI_ISL_17217115, EPI_ISL_17217116, EPI_ISL_17217117, EPI_ISL_17217121, EPI_ISL_17217122, EPI_ISL_17217126, EPI_ISL_17217127, EPI_ISL_17217131, EPI_ISL_17217134, EPI_ISL_17217137, EPI_ISL_17217146, EPI_ISL_17217153, EPI_ISL_17217154, EPI_ISL_17217157, EPI_ISL_17217161, EPI_ISL_17217162, EPI_ISL_17217164, EPI_ISL_17217168, EPI_ISL_17217174, EPI_ISL_17217178, EPI_ISL_17217186, EPI_ISL_17217189, EPI_ISL_17217193, EPI_ISL_17217195, EPI_ISL_17217199, EPI_ISL_17217202, EPI_ISL_17217206, EPI_ISL_17217207, EPI_ISL_17217212, EPI_ISL_17217218, EPI_ISL_17217220, EPI_ISL_17217221, EPI_ISL_17217222, EPI_ISL_17217227, EPI_ISL_17217228, EPI_ISL_17217229, EPI_ISL_17217237, EPI_ISL_17217240, EPI_ISL_17217241, EPI_ISL_17217243, EPI_ISL_17217247, EPI_ISL_17217250, EPI_ISL_17217265, EPI_ISL_17217266, EPI_ISL_17217282, EPI_ISL_17217302, EPI_ISL_17217315, EPI_ISL_17217319, EPI_ISL_17217326, EPI_ISL_17217328, EPI_ISL_17217329, EPI_ISL_17217338, EPI_ISL_17217362, EPI_ISL_17217367, EPI_ISL_17217370, EPI_ISL_17217403, EPI_ISL_17217410, EPI_ISL_17217418, EPI_ISL_17217433, EPI_ISL_17217454, EPI_ISL_17217459, EPI_ISL_17217475, EPI_ISL_17217486, EPI_ISL_17217488, EPI_ISL_17217508, EPI_ISL_17217519, EPI_ISL_17217528, EPI_ISL_17217531, EPI_ISL_17217542, EPI_ISL_17217545, EPI_ISL_17217549, EPI_ISL_17217554, EPI_ISL_17217559, EPI_ISL_17217564, EPI_ISL_17217571, EPI_ISL_17217579, EPI_ISL_17217586, EPI_ISL_17217603, EPI_ISL_17217608, EPI_ISL_17217612, EPI_ISL_17217613, EPI_ISL_17217619, EPI_ISL_17217624, EPI_ISL_17217639, EPI_ISL_17217642, EPI_ISL_17217649, EPI_ISL_17217652, EPI_ISL_17217654, EPI_ISL_17217658, EPI_ISL_17217659, EPI_ISL_17217661, EPI_ISL_17217664, EPI_ISL_17217665, EPI_ISL_17217676, EPI_ISL_17217679, EPI_ISL_17217684, EPI_ISL_17217688, EPI_ISL_17217692, EPI_ISL_17217693, EPI_ISL_17217694, EPI_ISL_17217696, EPI_ISL_17217700, EPI_ISL_17217705, EPI_ISL_17217745, EPI_ISL_17217751, EPI_ISL_17217774, EPI_ISL_17217777, EPI_ISL_17217809, EPI_ISL_17217812, EPI_ISL_17217814, EPI_ISL_17217815, EPI_ISL_17217825, EPI_ISL_17217847, EPI_ISL_17217893, EPI_ISL_17217896, EPI_ISL_17217902, EPI_ISL_17217911, EPI_ISL_17217915, EPI_ISL_17217916, EPI_ISL_17217922, EPI_ISL_17217928, EPI_ISL_17217929, EPI_ISL_17217933, EPI_ISL_17217935, EPI_ISL_17217938, EPI_ISL_17217943, EPI_ISL_17217946, EPI_ISL_17217951, EPI_ISL_17217956, EPI_ISL_17217967, EPI_ISL_17217983, EPI_ISL_17218000, EPI_ISL_17218013, EPI_ISL_17218022, EPI_ISL_17218023, EPI_ISL_17218027, EPI_ISL_17218029, EPI_ISL_17218032, EPI_ISL_17218034, EPI_ISL_17218035, EPI_ISL_17218038, EPI_ISL_17218054, EPI_ISL_17218058, EPI_ISL_17218072, EPI_ISL_17218083, EPI_ISL_17218091, EPI_ISL_17218123, EPI_ISL_17218131, EPI_ISL_17218134, EPI_ISL_17218137, EPI_ISL_17218138, EPI_ISL_17218150, EPI_ISL_17218151, EPI_ISL_17218154, EPI_ISL_17218163, EPI_ISL_17218169, EPI_ISL_17218184, EPI_ISL_17218187, EPI_ISL_17218194, EPI_ISL_17218195, EPI_ISL_17218205, EPI_ISL_17218206, EPI_ISL_17218207, EPI_ISL_17218219, EPI_ISL_17218222, EPI_ISL_17218233, EPI_ISL_17218237, EPI_ISL_17218238, EPI_ISL_17218239, EPI_ISL_17218240, EPI_ISL_17218249, EPI_ISL_17218274, EPI_ISL_17218284, EPI_ISL_17218295, EPI_ISL_17218297, EPI_ISL_17218303, EPI_ISL_17218317, EPI_ISL_17218323, EPI_ISL_17218336, EPI_ISL_17218346, EPI_ISL_17218352, EPI_ISL_17218403, EPI_ISL_17218407, EPI_ISL_17218447, EPI_ISL_17218480, EPI_ISL_17218483, EPI_ISL_17218526, EPI_ISL_17218540, EPI_ISL_17218545, EPI_ISL_17218551, EPI_ISL_17218590, EPI_ISL_17218593, EPI_ISL_17218617, EPI_ISL_17218645, EPI_ISL_17218648, EPI_ISL_17218658, EPI_ISL_17218664, EPI_ISL_17218672, EPI_ISL_17218680, EPI_ISL_17218693, EPI_ISL_17218696, EPI_ISL_17218699, EPI_ISL_17218707, EPI_ISL_17218709, EPI_ISL_17218717, EPI_ISL_17218726, EPI_ISL_17218732, EPI_ISL_17218735, EPI_ISL_17218737, EPI_ISL_17218763, EPI_ISL_17218777, EPI_ISL_17218784, EPI_ISL_17218796, EPI_ISL_17218805, EPI_ISL_17218812, EPI_ISL_17218840, EPI_ISL_17218843, EPI_ISL_17218846, EPI_ISL_17218850, EPI_ISL_17218869, EPI_ISL_17218887, EPI_ISL_17218891, EPI_ISL_17218916, EPI_ISL_17218924, EPI_ISL_17218931, EPI_ISL_17218950, EPI_ISL_17218968, EPI_ISL_17218972, EPI_ISL_17218987, EPI_ISL_17218991, EPI_ISL_17218995, EPI_ISL_17219010, EPI_ISL_17219023, EPI_ISL_17219035, EPI_ISL_17219054, EPI_ISL_17219068, EPI_ISL_17219079, EPI_ISL_17219085, EPI_ISL_17219150, EPI_ISL_17219163, EPI_ISL_17219167, EPI_ISL_17219172, EPI_ISL_17219188, EPI_ISL_17219189, EPI_ISL_17219200, EPI_ISL_17219201, EPI_ISL_17219208, EPI_ISL_17219216, EPI_ISL_17219219, EPI_ISL_17219233, EPI_ISL_17219234, EPI_ISL_17219242, EPI_ISL_17219248, EPI_ISL_17219256, EPI_ISL_17219258, EPI_ISL_17219267, EPI_ISL_17219276, EPI_ISL_17219277, EPI_ISL_17219281, EPI_ISL_17219282, EPI_ISL_17219289, EPI_ISL_17219292, EPI_ISL_17219297, EPI_ISL_17219299, EPI_ISL_17219300, EPI_ISL_17219317, EPI_ISL_17219321, EPI_ISL_17219341, EPI_ISL_17219344, EPI_ISL_17219345, EPI_ISL_17219349, EPI_ISL_17219358, EPI_ISL_17219367, EPI_ISL_17219387, EPI_ISL_17219390, EPI_ISL_17219396, EPI_ISL_17219398, EPI_ISL_17219402, EPI_ISL_17219408, EPI_ISL_17219412, EPI_ISL_17219422, EPI_ISL_17219430, EPI_ISL_17219460, EPI_ISL_17219462, EPI_ISL_17219469, EPI_ISL_17219506, EPI_ISL_17219533, EPI_ISL_17219540, EPI_ISL_17219553, EPI_ISL_17219558, EPI_ISL_17219563, EPI_ISL_17219566, EPI_ISL_17219567, EPI_ISL_17219574, EPI_ISL_17219582, EPI_ISL_17219592, EPI_ISL_17219593, EPI_ISL_17219596, EPI_ISL_17219608, EPI_ISL_17219620, EPI_ISL_17219630, EPI_ISL_17219631, EPI_ISL_17219640, EPI_ISL_17219641, EPI_ISL_17219651, EPI_ISL_17219654, EPI_ISL_17219669, EPI_ISL_17219675, EPI_ISL_17219677, EPI_ISL_17219678, EPI_ISL_17219684, EPI_ISL_17219686, EPI_ISL_17219693, EPI_ISL_17219698, EPI_ISL_17219712, EPI_ISL_17219720, EPI_ISL_17219721, EPI_ISL_17219746, EPI_ISL_17219747, EPI_ISL_17219748, EPI_ISL_17219752, EPI_ISL_17219758, EPI_ISL_17219775, EPI_ISL_17219777, EPI_ISL_17219792, EPI_ISL_17219804, EPI_ISL_17219805, EPI_ISL_17219813, EPI_ISL_17219814, EPI_ISL_17219821, EPI_ISL_17219840, EPI_ISL_17219850, EPI_ISL_17219861, EPI_ISL_17219867, EPI_ISL_17219887, EPI_ISL_17219889, EPI_ISL_17219898, EPI_ISL_17219905, EPI_ISL_17219907, EPI_ISL_17219912, EPI_ISL_17219913, EPI_ISL_17219920, EPI_ISL_17219927, EPI_ISL_17219933, EPI_ISL_17219945, EPI_ISL_17219958, EPI_ISL_17219960, EPI_ISL_17219962, EPI_ISL_17219967, EPI_ISL_17219975, EPI_ISL_17219976, EPI_ISL_17219986, EPI_ISL_17220022, EPI_ISL_17220031, EPI_ISL_17220036, EPI_ISL_17220050, EPI_ISL_17220051, EPI_ISL_17220053, EPI_ISL_17220069, EPI_ISL_17220080, EPI_ISL_17220081, EPI_ISL_17220084, EPI_ISL_17220090, EPI_ISL_17220091, EPI_ISL_17220098, EPI_ISL_17220107, EPI_ISL_17220115, EPI_ISL_17220141, EPI_ISL_17220149, EPI_ISL_17220157, EPI_ISL_17220161, EPI_ISL_17220180, EPI_ISL_17220206, EPI_ISL_17220209, EPI_ISL_17220210, EPI_ISL_17220217, EPI_ISL_17220229, EPI_ISL_17220247, EPI_ISL_17220253, EPI_ISL_17220257, EPI_ISL_17220273, EPI_ISL_17220283, EPI_ISL_17220288, EPI_ISL_17220289, EPI_ISL_17220293, EPI_ISL_17220294, EPI_ISL_17220298, EPI_ISL_17220304, EPI_ISL_17220305, EPI_ISL_17220307, EPI_ISL_17220308, EPI_ISL_17220310, EPI_ISL_17220313, EPI_ISL_17220318, EPI_ISL_17220323, EPI_ISL_17220324, EPI_ISL_17220325, EPI_ISL_17220327, EPI_ISL_17220330, EPI_ISL_17220331, EPI_ISL_17220335, EPI_ISL_17220339, EPI_ISL_17220344, EPI_ISL_17220346, EPI_ISL_17220350, EPI_ISL_17220352, EPI_ISL_17220353, EPI_ISL_17220354, EPI_ISL_17220359, EPI_ISL_17220362, EPI_ISL_17220372, EPI_ISL_17220374, EPI_ISL_17220376, EPI_ISL_17220377, EPI_ISL_17220379, EPI_ISL_17220385, EPI_ISL_17220392, EPI_ISL_17220395, EPI_ISL_17220399, EPI_ISL_17220404, EPI_ISL_17220405, EPI_ISL_17220410, EPI_ISL_17220413, EPI_ISL_17220414, EPI_ISL_17220419, EPI_ISL_17220421, EPI_ISL_17220428, EPI_ISL_17220433, EPI_ISL_17220436, EPI_ISL_17220438, EPI_ISL_17220440, EPI_ISL_17220444, EPI_ISL_17220451, EPI_ISL_17220452, EPI_ISL_17220461, EPI_ISL_17220463, EPI_ISL_17220471, EPI_ISL_17220474, EPI_ISL_17220475, EPI_ISL_17220477, EPI_ISL_17220488, EPI_ISL_17220489, EPI_ISL_17220495, EPI_ISL_17220499, EPI_ISL_17220502, EPI_ISL_17220505, EPI_ISL_17220509, EPI_ISL_17220510, EPI_ISL_17220511, EPI_ISL_17220525, EPI_ISL_17220530, EPI_ISL_17220531, EPI_ISL_17220539, EPI_ISL_17220543, EPI_ISL_17220544, EPI_ISL_17220546, EPI_ISL_17220559, EPI_ISL_17220561, EPI_ISL_17220562, EPI_ISL_17220574, EPI_ISL_17220577, EPI_ISL_17220579, EPI_ISL_17220587, EPI_ISL_17220594, EPI_ISL_17220597, EPI_ISL_17220601, EPI_ISL_17220606, EPI_ISL_17220609, EPI_ISL_17220614, EPI_ISL_17220615, EPI_ISL_17220618, EPI_ISL_17220620, EPI_ISL_17220624, EPI_ISL_17220628, EPI_ISL_17220632, EPI_ISL_17220637, EPI_ISL_17220638, EPI_ISL_17220639, EPI_ISL_17220641, EPI_ISL_17220642, EPI_ISL_17220644, EPI_ISL_17220651, EPI_ISL_17220652, EPI_ISL_17220653, EPI_ISL_17220658, EPI_ISL_17220661, EPI_ISL_17220667, EPI_ISL_17220669, EPI_ISL_17220680, EPI_ISL_17220681, EPI_ISL_17220686, EPI_ISL_17220690, EPI_ISL_17220692, EPI_ISL_17220694, EPI_ISL_17220697, EPI_ISL_17220700, EPI_ISL_17220705, EPI_ISL_17220706, EPI_ISL_17220714, EPI_ISL_17220724, EPI_ISL_17220726, EPI_ISL_17220728, EPI_ISL_17220729, EPI_ISL_17220734, EPI_ISL_17220736, EPI_ISL_17220741, EPI_ISL_17220744, EPI_ISL_17220748, EPI_ISL_17220750, EPI_ISL_17220751, EPI_ISL_17220752, EPI_ISL_17220754, EPI_ISL_17220760, EPI_ISL_17220761, EPI_ISL_17220765, EPI_ISL_17220770, EPI_ISL_17220776, EPI_ISL_17220777, EPI_ISL_17220779, EPI_ISL_17220784, EPI_ISL_17220785, EPI_ISL_17220791, EPI_ISL_17220792, EPI_ISL_17220797, EPI_ISL_17220802, EPI_ISL_17220803, EPI_ISL_17220806, EPI_ISL_17220808, EPI_ISL_17220810, EPI_ISL_17220816, EPI_ISL_17220830, EPI_ISL_17220835, EPI_ISL_17220839, EPI_ISL_17220840, EPI_ISL_17220847, EPI_ISL_17220848, EPI_ISL_17220852, EPI_ISL_17220862, EPI_ISL_17220874, EPI_ISL_17220876, EPI_ISL_17220877, EPI_ISL_17220881, EPI_ISL_17220885, EPI_ISL_17220888, EPI_ISL_17220893, EPI_ISL_17220898, EPI_ISL_17220899, EPI_ISL_17220901, EPI_ISL_17220902, EPI_ISL_17220913, EPI_ISL_17220916, EPI_ISL_17220917, EPI_ISL_17220928, EPI_ISL_17220929, EPI_ISL_17220933, EPI_ISL_17220937, EPI_ISL_17220942, EPI_ISL_17220947, EPI_ISL_17220948, EPI_ISL_17220951, EPI_ISL_17220952, EPI_ISL_17220957, EPI_ISL_17220959, EPI_ISL_17220962, EPI_ISL_17220966, EPI_ISL_17220968, EPI_ISL_17220971, EPI_ISL_17220975, EPI_ISL_17220977, EPI_ISL_17220978, EPI_ISL_17220979, EPI_ISL_17220980, EPI_ISL_17220989, EPI_ISL_17220992, EPI_ISL_17220997, EPI_ISL_17221000, EPI_ISL_17221004, EPI_ISL_17221005, EPI_ISL_17221007, EPI_ISL_17221009, EPI_ISL_17221010, EPI_ISL_17221014, EPI_ISL_17221017, EPI_ISL_17221019, EPI_ISL_17221022, EPI_ISL_17221026, EPI_ISL_17221033, EPI_ISL_17221036, EPI_ISL_17221038, EPI_ISL_17221042, EPI_ISL_17221043, EPI_ISL_17221051, EPI_ISL_17221052, EPI_ISL_17221059, EPI_ISL_17221061, EPI_ISL_17221064, EPI_ISL_17221067, EPI_ISL_17221070, EPI_ISL_17221077, EPI_ISL_17221080, EPI_ISL_17221082, EPI_ISL_17221084, EPI_ISL_17221086, EPI_ISL_17221087, EPI_ISL_17221091, EPI_ISL_17221092, EPI_ISL_17221093, EPI_ISL_17221097, EPI_ISL_17221102, EPI_ISL_17221109, EPI_ISL_17221121, EPI_ISL_17221130, EPI_ISL_17221133, EPI_ISL_17221136, EPI_ISL_17221137, EPI_ISL_17221148, EPI_ISL_17221151, EPI_ISL_17221152, EPI_ISL_17221163, EPI_ISL_17221177, EPI_ISL_17221184, EPI_ISL_17221192, EPI_ISL_17221195, EPI_ISL_17221201, EPI_ISL_17221202, EPI_ISL_17221206, EPI_ISL_17221208, EPI_ISL_17221210, EPI_ISL_17221212, EPI_ISL_17221219, EPI_ISL_17221225, EPI_ISL_17221230, EPI_ISL_17221231, EPI_ISL_17221235, EPI_ISL_17221241, EPI_ISL_17221243, EPI_ISL_17221247, EPI_ISL_17221250, EPI_ISL_17221251, EPI_ISL_17221252, EPI_ISL_17221261, EPI_ISL_17221262, EPI_ISL_17221267, EPI_ISL_17221268, EPI_ISL_17221278, EPI_ISL_17221279, EPI_ISL_17221281, EPI_ISL_17221286, EPI_ISL_17221288, EPI_ISL_17221294, EPI_ISL_17221297, EPI_ISL_17221299, EPI_ISL_17221302, EPI_ISL_17221304, EPI_ISL_17221305, EPI_ISL_17221308, EPI_ISL_17221311, EPI_ISL_17221312, EPI_ISL_17221314, EPI_ISL_17221324, EPI_ISL_17221325, EPI_ISL_17221328, EPI_ISL_17221332, EPI_ISL_17221337, EPI_ISL_17221343, EPI_ISL_17221347, EPI_ISL_17221351, EPI_ISL_17221353, EPI_ISL_17221354, EPI_ISL_17221355, EPI_ISL_17221358, EPI_ISL_17221359, EPI_ISL_17221360, EPI_ISL_17221369, EPI_ISL_17221370, EPI_ISL_17221373, EPI_ISL_17221375, EPI_ISL_17221376, EPI_ISL_17221378, EPI_ISL_17221383, EPI_ISL_17221387, EPI_ISL_17221394, EPI_ISL_17221399, EPI_ISL_17221400, EPI_ISL_17221404, EPI_ISL_17221410, EPI_ISL_17221411, EPI_ISL_17221413, EPI_ISL_17221420, EPI_ISL_17221421, EPI_ISL_17221428, EPI_ISL_17221429, EPI_ISL_17221433, EPI_ISL_17221434, EPI_ISL_17221441, EPI_ISL_17221452, EPI_ISL_17221456, EPI_ISL_17221466, EPI_ISL_17221474, EPI_ISL_17221479, EPI_ISL_17221486, EPI_ISL_17221495, EPI_ISL_17221498, EPI_ISL_17221500, EPI_ISL_17221504, EPI_ISL_17221505, EPI_ISL_17221506, EPI_ISL_17221508, EPI_ISL_17221515, EPI_ISL_17221516, EPI_ISL_17221521, EPI_ISL_17221523, EPI_ISL_17221524, EPI_ISL_17221525, EPI_ISL_17221535			
see above	Astrazeneca Laboratory	Astrazeneca Laboratory	Wilkins,D., Langedijk,A.C., Lebbink,R.J., Morehouse,C.A., Abram,M.E., Ahani,B., Aksyuk,A.A., Baraldi,E., Brady,T., Chen,A.T., Chi,H., Choi,E.H., Cohen,R., Danilenko,D.M., Gopalakrishnan,V., Greenough,A., Heikkinen,T., Hosoya,M., Keller,C., Kelly,E.J., Kragten-Tabatabaie,L., Martinon-Torres,F., Mascarenas-de Los Santos,A.H., Nunes,M.C., Palomino,M.A., Papenburg,J., Pernica,J.M., Richmond,P., Stein,R.T., Tuffy,K.M., Verwey,C., Esser,M.T., Tabor,D.E. and Bont,L.J.
EPI_ISL_17221540, EPI_ISL_17221557, EPI_ISL_17221558, EPI_ISL_17221563, EPI_ISL_17221570, EPI_ISL_17221584, EPI_ISL_17221594, EPI_ISL_17221606, EPI_ISL_17221615, EPI_ISL_17221616, EPI_ISL_17221636, EPI_ISL_17221648, EPI_ISL_17221655, EPI_ISL_17221656, EPI_ISL_17221659			
see above	University of Ljubljana - Faculty of Medicine	University of Ljubljana - Faculty of Medicine	Jevsnik Virant,M., Lustrek,M. and Petrovec,M.
EPI_ISL_17308690, EPI_ISL_17308693, EPI_ISL_17308694, EPI_ISL_17308696, EPI_ISL_17308697, EPI_ISL_17308702, EPI_ISL_17308703, EPI_ISL_17308704, EPI_ISL_17308705, EPI_ISL_17308706, EPI_ISL_17308708, EPI_ISL_17308710, EPI_ISL_17308713, EPI_ISL_17308717, EPI_ISL_17308718, EPI_ISL_17308719, EPI_ISL_17308720, EPI_ISL_17308721, EPI_ISL_17308722			
see above	Laboratório de Virologia - Instituto Nacional de Saúde	Laboratório de Vírus Respiratórios e do Sarampo - Instituto Oswaldo Cruz, Laboratório de Virologia - Instituto Nacional de Saúde	Almiro Tivane, Paola Resende, Neuza Nguenha, Loira Machalele, Sadia Alí, Délcio Muteto, Aunésia Marrurele, Mirela Pale, Félix Gundane, Judite Salência, Marilda Siqueira
EPI_ISL_17508359, EPI_ISL_17508360, EPI_ISL_17508361, EPI_ISL_17508362, EPI_ISL_17508363, EPI_ISL_17508364, EPI_ISL_17508365, EPI_ISL_17508366, EPI_ISL_17508367, EPI_ISL_17508368, EPI_ISL_17508369, EPI_ISL_17508370, EPI_ISL_17508371, EPI_ISL_17508372, EPI_ISL_17508373, EPI_ISL_17508374, EPI_ISL_17508375, EPI_ISL_17508376, EPI_ISL_17508377, EPI_ISL_17508378, EPI_ISL_17508379, EPI_ISL_17508380, EPI_ISL_17508381, EPI_ISL_17508382, EPI_ISL_17508383, EPI_ISL_17508384, EPI_ISL_17508385, EPI_ISL_17508386, EPI_ISL_17508387, EPI_ISL_17508388, EPI_ISL_17508389, EPI_ISL_17508390, EPI_ISL_17508391, EPI_ISL_17508392, EPI_ISL_17508393, EPI_ISL_17508394, EPI_ISL_17508395, EPI_ISL_17508396, EPI_ISL_17508397, EPI_ISL_17508398, EPI_ISL_17508399, EPI_ISL_17508400, EPI_ISL_17508402, EPI_ISL_17508403, EPI_ISL_17508404, EPI_ISL_17508405, EPI_ISL_17508406, EPI_ISL_17508407, EPI_ISL_17508408, EPI_ISL_17508409, EPI_ISL_17508410, EPI_ISL_17508411, EPI_ISL_17508412, EPI_ISL_17508413, EPI_ISL_17508414, EPI_ISL_17508415, EPI_ISL_17508417, EPI_ISL_17508418, EPI_ISL_17508419, EPI_ISL_17508420, EPI_ISL_17508421, EPI_ISL_17508422, EPI_ISL_17508423, EPI_ISL_17508424, EPI_ISL_17508425, EPI_ISL_17508426, EPI_ISL_17508427, EPI_ISL_17508428, EPI_ISL_17508429, EPI_ISL_17508430, EPI_ISL_17508431, EPI_ISL_17508432, EPI_ISL_17508433, EPI_ISL_17508434, EPI_ISL_17508435, EPI_ISL_17508436, EPI_ISL_17508437, EPI_ISL_17508438, EPI_ISL_17508439, EPI_ISL_17508440, EPI_ISL_17508441, EPI_ISL_17508442, EPI_ISL_17508443, EPI_ISL_17508445, EPI_ISL_17508446, EPI_ISL_17508447, EPI_ISL_17508448, EPI_ISL_17508449, EPI_ISL_17508450, EPI_ISL_17508451, EPI_ISL_17508452, EPI_ISL_17508453, EPI_ISL_17508454, EPI_ISL_17508455, EPI_ISL_17508456, EPI_ISL_17508457, EPI_ISL_17508458, EPI_ISL_17508459, EPI_ISL_17508460, EPI_ISL_17508461, EPI_ISL_17508462, EPI_ISL_17508463, EPI_ISL_17508464, EPI_ISL_17508465, EPI_ISL_17508466, EPI_ISL_17508467, EPI_ISL_17508468, EPI_ISL_17508470, EPI_ISL_17508472, EPI_ISL_17508473			
see above	KEMRI-Wellcome Trust	KEMRI-Wellcome Trust	Lambisia,A.W., Lewa,C., Mutunga,M., Cheruiyot,R., Okanda,D., Makori,T., Githinji,G., Nokes,J.D. and Agoti,C.
EPI_ISL_17594434, EPI_ISL_17594435, EPI_ISL_17594436, EPI_ISL_17594437, EPI_ISL_17594438, EPI_ISL_17594439, EPI_ISL_17594440, EPI_ISL_17594444, EPI_ISL_17594445, EPI_ISL_17594446, EPI_ISL_17594448, EPI_ISL_17594449, EPI_ISL_17594450, EPI_ISL_17594451, EPI_ISL_17594452, EPI_ISL_17594453, EPI_ISL_17594454, EPI_ISL_17594455, EPI_ISL_17594459, EPI_ISL_17594460, EPI_ISL_17594461, EPI_ISL_17594462, EPI_ISL_17594463, EPI_ISL_17594466, EPI_ISL_17594468, EPI_ISL_17594469, EPI_ISL_17594470, EPI_ISL_17594471, EPI_ISL_17594472, EPI_ISL_17594473, EPI_ISL_17594474, EPI_ISL_17594476, EPI_ISL_17594477, EPI_ISL_17594478, EPI_ISL_17594479, EPI_ISL_17594480, EPI_ISL_17594482, EPI_ISL_17594483, EPI_ISL_17594484, EPI_ISL_17594485, EPI_ISL_17594486, EPI_ISL_17594487, EPI_ISL_17594488, EPI_ISL_17594489, EPI_ISL_17594490, EPI_ISL_17594492, EPI_ISL_17594493, EPI_ISL_17594511, EPI_ISL_17594512, EPI_ISL_17594513, EPI_ISL_17594514, EPI_ISL_17594515, EPI_ISL_17594517, EPI_ISL_17594540, EPI_ISL_17594585, EPI_ISL_17594586, EPI_ISL_17594588, EPI_ISL_17594653, EPI_ISL_17594654, EPI_ISL_17594658			
see above	Chang Bing Show Chwan Memorial Hospital	Chang Bing Show Chwan Memorial Hospital	Lee,C.Y.
EPI_ISL_17594666	Institute for Infectious Disease Control and Prevention, Henan Center for Disease Control and Prevention	Institute for Infectious Disease Control and Prevention, Henan Center for Disease Control and Prevention	Zhao,X.
EPI_ISL_17594672, EPI_ISL_17594673, EPI_ISL_17594674, EPI_ISL_17594675, EPI_ISL_17594676, EPI_ISL_17594678, EPI_ISL_17594679, EPI_ISL_17594680, EPI_ISL_17594681, EPI_ISL_17594682, EPI_ISL_17594683, EPI_ISL_17594684, EPI_ISL_17594686, EPI_ISL_17594688, EPI_ISL_17594689, EPI_ISL_17594691, EPI_ISL_17594692, EPI_ISL_17594693, EPI_ISL_17594695, EPI_ISL_17594697, EPI_ISL_17594698, EPI_ISL_17594699, EPI_ISL_17594700, EPI_ISL_17594702, EPI_ISL_17594703, EPI_ISL_17594705, EPI_ISL_17594706, EPI_ISL_17594710, EPI_ISL_17594711, EPI_ISL_17594714, EPI_ISL_17594715, EPI_ISL_17594716, EPI_ISL_17594720, EPI_ISL_17594723, EPI_ISL_17594725, EPI_ISL_17594738, EPI_ISL_17594744, EPI_ISL_17594745, EPI_ISL_17594748, EPI_ISL_17594750, EPI_ISL_17594760, EPI_ISL_17594761, EPI_ISL_17594762, EPI_ISL_17594763, EPI_ISL_17594764, EPI_ISL_17594765, EPI_ISL_17594766, EPI_ISL_17594768, EPI_ISL_17594770, EPI_ISL_17594774			
see above	Institute for Infectious Disease Control and Prevention, Henan Center for Disease Control and Prevention	Institute for Infectious Disease Control and Prevention, Henan Center for Disease Control and Prevention	Zhao,X., Teng,Z. and Zhang,X.
EPI_ISL_17782657	Kitasato University, Infection Control and Immunology	Kitasato University, Infection Control and Immunology	Ito,T., Sawada,A., Saito,A., Ishikura,K., Kawashima,H., Nakayama,T. and Katayama,K.
EPI_ISL_17797338, EPI_ISL_17797341, EPI_ISL_17797346, EPI_ISL_17797348, EPI_ISL_17797351, EPI_ISL_17797352, EPI_ISL_17797354, EPI_ISL_17797355, EPI_ISL_17797357, EPI_ISL_17797360, EPI_ISL_17797366, EPI_ISL_17797368, EPI_ISL_17797369, EPI_ISL_17797371, EPI_ISL_17797372, EPI_ISL_17797382, EPI_ISL_17797385, EPI_ISL_17797386, EPI_ISL_17797390, EPI_ISL_17797391, EPI_ISL_17797395, EPI_ISL_17797398, EPI_ISL_17797400, EPI_ISL_17797416, EPI_ISL_17797417, EPI_ISL_17797422, EPI_ISL_17797423, EPI_ISL_17797426, EPI_ISL_17797431, EPI_ISL_17797432, EPI_ISL_17797437, EPI_ISL_17797445, EPI_ISL_17797450, EPI_ISL_17797458, EPI_ISL_17797462, EPI_ISL_17797464, EPI_ISL_17797467, EPI_ISL_17797473, EPI_ISL_17797479, EPI_ISL_17797482, EPI_ISL_17797483, EPI_ISL_17797486, EPI_ISL_17797492, EPI_ISL_17797501, EPI_ISL_17797505, EPI_ISL_17797509, EPI_ISL_17797516, EPI_ISL_17797517, EPI_ISL_17797522, EPI_ISL_17797524			
see above	Chiang Mai University, Department of Microbiology	Chiang Mai University, Department of Microbiology	Lin,W.H.
EPI_ISL_17964306, EPI_ISL_17964307, EPI_ISL_17964309, EPI_ISL_17964311, EPI_ISL_17964312, EPI_ISL_17964313, EPI_ISL_17964315, EPI_ISL_17964316, EPI_ISL_17964317, EPI_ISL_17964321, EPI_ISL_17964322, EPI_ISL_17964323, EPI_ISL_17964324, EPI_ISL_17964326, EPI_ISL_17964328, EPI_ISL_17964329, EPI_ISL_17964330, EPI_ISL_17964332, EPI_ISL_17964333, EPI_ISL_17964334, EPI_ISL_17964336, EPI_ISL_17964337, EPI_ISL_17964338, EPI_ISL_17964339, EPI_ISL_17964340, EPI_ISL_17964342, EPI_ISL_17964344, EPI_ISL_17964345, EPI_ISL_17964347, EPI_ISL_17964355, EPI_ISL_17964359, EPI_ISL_17964360, EPI_ISL_17964363, EPI_ISL_17964365, EPI_ISL_17964366, EPI_ISL_17964367, EPI_ISL_17964370, EPI_ISL_17964372, EPI_ISL_17964374, EPI_ISL_17964375			

see above	University of Zambia Medical School	Department of Medical Microbiology, University Medical Centre Utrecht	Annefleur C. Langedijk, Bram Vrancken, Robert Jan Lebbink, Rachel C. Pieciak, Philippe Lemey, Louis J. Bont, Christopher J Gill
EPI_ISL_17975585	Sapienza University	Sapienza University	Pierangeli,A.
EPI_ISL_17995641, EPI_ISL_17995642, EPI_ISL_17995643, EPI_ISL_17995644, EPI_ISL_17995645, EPI_ISL_17995646, EPI_ISL_17995647, EPI_ISL_17995648, EPI_ISL_17995649, EPI_ISL_17995650, EPI_ISL_17995651, EPI_ISL_17995652, EPI_ISL_17995653, EPI_ISL_17995654, EPI_ISL_17995656, EPI_ISL_17995659, EPI_ISL_17995676, EPI_ISL_17995765			
see above	Robert Koch-Institute Nationales Referenzzentrum für Influenza	Robert Koch-Institute Nationales Referenzzentrum für Influenza	Sophie Kondgen, Janine Reiche
EPI_ISL_18090571, EPI_ISL_18090574, EPI_ISL_18090575, EPI_ISL_18090577, EPI_ISL_18090578, EPI_ISL_18090580, EPI_ISL_18090582, EPI_ISL_18090586, EPI_ISL_18090587, EPI_ISL_18090591, EPI_ISL_18090593, EPI_ISL_18090594, EPI_ISL_18090595, EPI_ISL_18090596, EPI_ISL_18090597, EPI_ISL_18090598, EPI_ISL_18090599, EPI_ISL_18090602, EPI_ISL_18090604, EPI_ISL_18090607, EPI_ISL_18090608, EPI_ISL_18090611, EPI_ISL_18090612, EPI_ISL_18090616, EPI_ISL_18090618, EPI_ISL_18090619, EPI_ISL_18090620, EPI_ISL_18090622, EPI_ISL_18090623, EPI_ISL_18090624, EPI_ISL_18090625, EPI_ISL_18090626, EPI_ISL_18090629, EPI_ISL_18090636, EPI_ISL_18090637, EPI_ISL_18090642, EPI_ISL_18090643, EPI_ISL_18090645, EPI_ISL_18090646, EPI_ISL_18090647, EPI_ISL_18090648, EPI_ISL_18090652, EPI_ISL_18090653, EPI_ISL_18090654, EPI_ISL_18090656, EPI_ISL_18090657, EPI_ISL_18090658, EPI_ISL_18090660, EPI_ISL_18090662, EPI_ISL_18090663, EPI_ISL_18090665, EPI_ISL_18090666, EPI_ISL_18090667, EPI_ISL_18090671, EPI_ISL_18090676, EPI_ISL_18090677, EPI_ISL_18090679, EPI_ISL_18090680, EPI_ISL_18090684, EPI_ISL_18090686, EPI_ISL_18090690, EPI_ISL_18090692, EPI_ISL_18090694			
see above	Westmead Institute for Medical Research & Sydney Infectious Disease Institute	Westmead Institute for Medical Research & Sydney Infectious Disease Institute	Pangesti,K.N.A., Ansari,H.R., Bayoumi,A., Kesson,A.M., Hill-Cawthorne,G.A. and Abd El Ghany,M.
EPI_ISL_18143718, EPI_ISL_18143719, EPI_ISL_18143720, EPI_ISL_18143722, EPI_ISL_18143723, EPI_ISL_18143724, EPI_ISL_18143725, EPI_ISL_18143726, EPI_ISL_18143727, EPI_ISL_18143729, EPI_ISL_18143731, EPI_ISL_18143732			
see above	Tohoku University School of Medicine	Tohoku University School of Medicine	Simusika,P., Okamoto,M., Sakamoto,M., Dapat,C., Imamura,T., Saito,M., Azam,S., Mwila,E., Monze,M. and Oshitani,H.
EPI_ISL_18310826, EPI_ISL_18310828, EPI_ISL_18310832, EPI_ISL_18310835, EPI_ISL_18310836, EPI_ISL_18310837, EPI_ISL_18310838, EPI_ISL_18310839, EPI_ISL_18310843, EPI_ISL_18310845, EPI_ISL_18310846, EPI_ISL_18310847, EPI_ISL_18310849, EPI_ISL_18310850, EPI_ISL_18310851, EPI_ISL_18310852, EPI_ISL_18310853, EPI_ISL_18310856, EPI_ISL_18310857, EPI_ISL_18310858, EPI_ISL_18310859, EPI_ISL_18310860, EPI_ISL_18310863, EPI_ISL_18310864, EPI_ISL_18310865, EPI_ISL_18310869, EPI_ISL_18310870, EPI_ISL_18310873, EPI_ISL_18310874, EPI_ISL_18310875, EPI_ISL_18310876, EPI_ISL_18310881, EPI_ISL_18310882, EPI_ISL_18310883, EPI_ISL_18310885, EPI_ISL_18310886, EPI_ISL_18310888, EPI_ISL_18310890, EPI_ISL_18310891, EPI_ISL_18310893, EPI_ISL_18310894, EPI_ISL_18310895, EPI_ISL_18310896, EPI_ISL_18310898, EPI_ISL_18310900, EPI_ISL_18310901, EPI_ISL_18310902, EPI_ISL_18310903, EPI_ISL_18310904, EPI_ISL_18310908, EPI_ISL_18310909, EPI_ISL_18310910, EPI_ISL_18310912, EPI_ISL_18310913, EPI_ISL_18310916, EPI_ISL_18310918			
see above	Global Health Research Center	Global Health Research Center	Umar,S., Yang,R., Wang,X., Liu,Y., Ke,P. and Qin,S.
EPI_ISL_18334087, EPI_ISL_18334093, EPI_ISL_18334101, EPI_ISL_18334107, EPI_ISL_18334123, EPI_ISL_18334145	Tianjin Children's Hospital, Tianjin Pediatric Research Institute	Tianjin Children's Hospital, Tianjin Pediatric Research Institute	Hou,M., Meng,C., Dong,L. and Wang,H.
EPI_ISL_18334239, EPI_ISL_18334251, EPI_ISL_18334252, EPI_ISL_18334254, EPI_ISL_18334258, EPI_ISL_18334260, EPI_ISL_18334264, EPI_ISL_18334265, EPI_ISL_18334271, EPI_ISL_18334276, EPI_ISL_18334279, EPI_ISL_18334287, EPI_ISL_18334288, EPI_ISL_18334289, EPI_ISL_18334293, EPI_ISL_18334298, EPI_ISL_18334300, EPI_ISL_18334303, EPI_ISL_18334306, EPI_ISL_18334308, EPI_ISL_18334309, EPI_ISL_18334310, EPI_ISL_18334315, EPI_ISL_18334321, EPI_ISL_18334322, EPI_ISL_18334324, EPI_ISL_18334326, EPI_ISL_18334331, EPI_ISL_18334332, EPI_ISL_18334347, EPI_ISL_18334350, EPI_ISL_18334351, EPI_ISL_18334353, EPI_ISL_18334355, EPI_ISL_18334358, EPI_ISL_18334360, EPI_ISL_18334368, EPI_ISL_18334370, EPI_ISL_18334372, EPI_ISL_18334375, EPI_ISL_18334377, EPI_ISL_18334384, EPI_ISL_18334395, EPI_ISL_18334396, EPI_ISL_18334398, EPI_ISL_18334401, EPI_ISL_18334407, EPI_ISL_18334408, EPI_ISL_18334409, EPI_ISL_18334412, EPI_ISL_18334413			
see above	National Influenza Center, Virology Laboratory	National Influenza Center, Virology Laboratory	Simusika,P., Okamoto,M., Dapat,C., Muleya,W., Saito,M., Azam,S., Mwape,I., Mwila,E., Monze,M. and Oshitani,H.
EPI_ISL_1834083, EPI_ISL_1834084, EPI_ISL_1834085, EPI_ISL_1834086, EPI_ISL_1834087, EPI_ISL_1834088, EPI_ISL_1834089, EPI_ISL_1834090, EPI_ISL_1834091, EPI_ISL_1834092, EPI_ISL_1834093, EPI_ISL_1834094, EPI_ISL_1834095, EPI_ISL_1834097, EPI_ISL_1834098, EPI_ISL_1834102, EPI_ISL_1834103, EPI_ISL_1834104, EPI_ISL_1834105, EPI_ISL_1834106, EPI_ISL_1834107, EPI_ISL_1834108, EPI_ISL_1834109, EPI_ISL_1834112, EPI_ISL_1834113, EPI_ISL_1834115, EPI_ISL_1834116, EPI_ISL_1834117, EPI_ISL_1834118, EPI_ISL_1834119, EPI_ISL_1834120, EPI_ISL_1834121, EPI_ISL_1834123, EPI_ISL_1834124, EPI_ISL_1834125, EPI_ISL_1834127, EPI_ISL_1834128, EPI_ISL_1834130, EPI_ISL_1834131, EPI_ISL_1834132, EPI_ISL_1834133, EPI_ISL_1834134, EPI_ISL_1834137, EPI_ISL_1834138, EPI_ISL_1834139, EPI_ISL_1834140, EPI_ISL_1834141, EPI_ISL_1834142, EPI_ISL_1834144, EPI_ISL_1834146, EPI_ISL_1834147, EPI_ISL_1834148			
see above	Royal Children's Hospital	WHO Influenza Centre for Reference and Research on Influenza	Angela Todd, Yi-Mo Deng, Annette Alafaci, Naomi Komadina
EPI_ISL_18396055, EPI_ISL_18396077, EPI_ISL_18396078, EPI_ISL_18396079, EPI_ISL_18396080, EPI_ISL_18396081, EPI_ISL_18396082, EPI_ISL_18396083, EPI_ISL_18396084, EPI_ISL_18396085, EPI_ISL_18396086, EPI_ISL_18396202, EPI_ISL_18396203, EPI_ISL_18396204, EPI_ISL_18396206, EPI_ISL_18396207, EPI_ISL_18396208, EPI_ISL_18396209, EPI_ISL_18396211			
see above	Niigata University (IDRC) Infectious Diseases Research Center	Niigata University (IDRC) Infectious Diseases Research Center	Wint Wint Phyu, Reiko Saito
EPI_ISL_18396212	Niigata University (IDRC) Infectious Diseases Research Center	Niigata University (IDRC) Infectious Diseases Research Center	1-757 Asahimachi-dori, Chuo-Ku, Niigata City 951-8510 Japan Wint Wint Phyu, Reiko Saito
EPI_ISL_18396249, EPI_ISL_18396250, EPI_ISL_18396251, EPI_ISL_18396252, EPI_ISL_18396253, EPI_ISL_18396254, EPI_ISL_18396255, EPI_ISL_18396256, EPI_ISL_18396257, EPI_ISL_18396258, EPI_ISL_18396336, EPI_ISL_18396337, EPI_ISL_18396338, EPI_ISL_18396339, EPI_ISL_18396340, EPI_ISL_18396341, EPI_ISL_18396342, EPI_ISL_18396343, EPI_ISL_18396344, EPI_ISL_18396345, EPI_ISL_18396347, EPI_ISL_18396348, EPI_ISL_18396350, EPI_ISL_18396351, EPI_ISL_18396354, EPI_ISL_18396355, EPI_ISL_18396358, EPI_ISL_18396359, EPI_ISL_18396360, EPI_ISL_18396361, EPI_ISL_18397025, EPI_ISL_18397042, EPI_ISL_18397057, EPI_ISL_18397058, EPI_ISL_18397181, EPI_ISL_18397188, EPI_ISL_18397189, EPI_ISL_18397190, EPI_ISL_18397656, EPI_ISL_18397657, EPI_ISL_18397658, EPI_ISL_18401430, EPI_ISL_18401431, EPI_ISL_18401432, EPI_ISL_18401433, EPI_ISL_18401434, EPI_ISL_18401435, EPI_ISL_18401436, EPI_ISL_18401437, EPI_ISL_18401438, EPI_ISL_18401439, EPI_ISL_18401440, EPI_ISL_18401441, EPI_ISL_18401442, EPI_ISL_18401443, EPI_ISL_18401444, EPI_ISL_18401445, EPI_ISL_18401446, EPI_ISL_18401447, EPI_ISL_18401448, EPI_ISL_18401449, EPI_ISL_18401450, EPI_ISL_18401451, EPI_ISL_18401455, EPI_ISL_18401460			
see above	Niigata University (IDRC) Infectious Diseases Research Center	Niigata University (IDRC) Infectious Diseases Research Center	Wint Wint Phyu, Reiko Saito
EPI_ISL_18436337, EPI_ISL_18436341, EPI_ISL_18436342, EPI_ISL_18436343, EPI_ISL_18436344, EPI_ISL_18436347, EPI_ISL_18436357, EPI_ISL_18436358, EPI_ISL_18436360, EPI_ISL_18436362, EPI_ISL_18436363, EPI_ISL_18436366, EPI_ISL_18436368, EPI_ISL_18436369, EPI_ISL_18436370, EPI_ISL_18436372, EPI_ISL_18436375, EPI_ISL_18436377, EPI_ISL_18436379, EPI_ISL_18436380, EPI_ISL_18436381, EPI_ISL_18436382, EPI_ISL_18436385, EPI_ISL_18436387, EPI_ISL_18436389, EPI_ISL_18436391, EPI_ISL_18436392, EPI_ISL_18436396, EPI_ISL_18436397, EPI_ISL_18436399, EPI_ISL_18436402, EPI_ISL_18436406, EPI_ISL_18436409, EPI_ISL_18436410, EPI_ISL_18436412, EPI_ISL_18436416, EPI_ISL_18436423, EPI_ISL_18436424, EPI_ISL_18436427, EPI_ISL_18436428, EPI_ISL_18436430, EPI_ISL_18436434, EPI_ISL_18436435, EPI_ISL_18436436, EPI_ISL_18436437, EPI_ISL_18436438, EPI_ISL_18436439, EPI_ISL_18436441, EPI_ISL_18436442, EPI_ISL_18436445, EPI_ISL_18436449, EPI_ISL_18436450, EPI_ISL_18436452, EPI_ISL_18436453, EPI_ISL_18436458, EPI_ISL_18436461, EPI_ISL_18436463, EPI_ISL_18436467, EPI_ISL_18436468, EPI_ISL_18436471, EPI_ISL_18436472, EPI_ISL_18436473, EPI_ISL_18436475, EPI_ISL_18436476, EPI_ISL_18436478, EPI_ISL_18436479, EPI_ISL_18436482, EPI_ISL_18436483, EPI_ISL_18436485, EPI_ISL_18436488, EPI_ISL_18436489, EPI_ISL_18436491, EPI_ISL_18436492, EPI_ISL_18436493, EPI_ISL_18436495, EPI_ISL_18436497, EPI_ISL_18436507, EPI_ISL_18436508, EPI_ISL_18436509, EPI_ISL_18436511, EPI_ISL_18436519, EPI_ISL_18436521, EPI_ISL_18436522, EPI_ISL_18436523, EPI_ISL_18436525, EPI_ISL_18436528, EPI_ISL_18436530, EPI_ISL_18436531, EPI_ISL_18436533, EPI_ISL_18436535, EPI_ISL_18436536, EPI_ISL_18436538, EPI_ISL_18436544, EPI_ISL_18436545, EPI_ISL_18436547, EPI_ISL_18436548, EPI_ISL_18436550, EPI_ISL_18436551, EPI_ISL_18436553, EPI_ISL_18436555, EPI_ISL_18436556, EPI_ISL_18436558			
see above	Beijing Institute of Microbiology and Epidemiology, State Key Laboratory of Pathogen and Biosecurity, Beijing Key Laboratory of Vector Borne and Natural Focus Infectious Diseases	Beijing Institute of Microbiology and Epidemiology, State Key Laboratory of Pathogen and Biosecurity, Beijing Key Laboratory of Vector Borne and Natural Focus Infectious Diseases	Fu,Y., Li,Q., Li,F., Huang,L., Zhang,H., Chen,X. and Xie,Z.
EPI_ISL_18482719, EPI_ISL_18482720, EPI_ISL_18482724, EPI_ISL_18482734, EPI_ISL_18482761, EPI_ISL_18482772, EPI_ISL_18482773, EPI_ISL_18482780, EPI_ISL_18482799, EPI_ISL_18482805, EPI_ISL_18482815			
see above	Beijing Children's Hospital, Capital Medical University, Laboratory of Infection and Virology	Beijing Children's Hospital, Capital Medical University, Laboratory of Infection and Virology	Li,F., Zhu,Y. and Chen,X.
EPI_ISL_18507283, EPI_ISL_18507293	Victorian Infectious Diseases Reference Laboratory, Translational Diagnostics	Victorian Infectious Diseases Reference Laboratory, Translational Diagnostics	Steinig JOACHIM,E.
EPI_ISL_2156812, EPI_ISL_2156813, EPI_ISL_2156814	Royal Children's Hospital	WHO Influenza Centre for Reference and Research on Influenza	Angela Todd, Yi-Mo Deng, Annette Alafaci, Naomi Komadina
EPI_ISL_2543814, EPI_ISL_2543815, EPI_ISL_2543817, EPI_ISL_2543818, EPI_ISL_2543819, EPI_ISL_2543824	Institut Pasteur de Cote d'Ivoire	WHO Collaborating Centre for Reference and Research on Influenza	Xiaomin Dong, Herve Kadjo, Yi-Mo Deng, Ammar Aziz, Naomi Komadina
EPI_ISL_2543828, EPI_ISL_2543830, EPI_ISL_2543832, EPI_ISL_2543841, EPI_ISL_2543844	National Center for Communicable Diseases	WHO Collaborating Centre for Reference and Research on Influenza	Xiaomin Dong, Darmaa Bardach, Yi-Mo Deng, Ammar Aziz, Naomi Komadina
EPI_ISL_2544052	Virology Laboratory, Dr. Ricardo Gutierrez Children Hospital	Virology Laboratory, Dr. Ricardo Gutierrez Children Hospital	Goya,S., Valinotto,L.E., Tittarelli,E., Rojo,G.L., Greninger,A., Zaiat,J., Marti,M., Mistchenko,A.S. and Viegas,M.
EPI_ISL_2558738, EPI_ISL_2558754, EPI_ISL_2558755, EPI_ISL_2558756, EPI_ISL_2558757, EPI_ISL_2558758	Public Health Laboratory, Minnesota Department of Health	Public Health Laboratory, Minnesota Department of Health	Wang,X., Thielen,B.K. and Lynfield,R.
EPI_ISL_2558770	Medicine, American University of Beirut	Medicine, American University of Beirut	Abou-El-Hassan,H. and Zaraket,H.
EPI_ISL_2570828	Department of Virology, Tehran University of Medical Sciences	Department of Virology, Tehran University of Medical Sciences	Salimi,V., Shatizadeh Malekshahi,S., Ghavami,N. and Mokhtari Azad,T.
EPI_ISL_2570919	Lab. of Molecular Virology, Research Institute of Influenza	Lab. of Molecular Virology, Research Institute of Influenza	Sintsova,K.S., Krivitskaya,V.Z., Sverlova,M.V., Petrova,E.R., Fadeev,A.V. and Komissarov,A.B.
EPI_ISL_2570929	Pediatrics, Show-Chwan Memorial Hospital	Pediatrics, Show-Chwan Memorial Hospital	Lee,C.-Y.
EPI_ISL_2570947	Centre for Infectious Diseases Virology, University of Heidelberg	Centre for Infectious Diseases Virology, University of Heidelberg	Ihling,C.M., Schnitzler,P., Heinrich,N., Mangu,C., Sudi,L., Souares,A., Gies,S., Sie,A., Coulibaly,B., Ouedraogo,A.T., Mordmueller,B., Held,J., Adegnika,A.A., Fernandez,J.F., Eckerle,I., May,J., Hogan,B., Eibach,D., Owusu-Dabo,E. and Tabatabai,J.
EPI_ISL_2571060, EPI_ISL_2571066, EPI_ISL_2571072, EPI_ISL_2571074, EPI_ISL_2571075, EPI_ISL_2571076, EPI_ISL_2571079, EPI_ISL_2571080	Centre for Research And Knowledge Transfer in Biotechnology, University of Zagreb	Centre for Research And Knowledge Transfer in Biotechnology, University of Zagreb	Slovic,A., Ivancic-Jelecki,J., Ljubin-Sternak,S., Mlinaric-Galinovic,G., Vilibic-Cavlek,T., Tabain,I. and Forcic,D.
EPI_ISL_2571094, EPI_ISL_2571098, EPI_ISL_2571100, EPI_ISL_2571109, EPI_ISL_2571112, EPI_ISL_2571131, EPI_ISL_2571138, EPI_ISL_2571139, EPI_ISL_2571144, EPI_ISL_2571145, EPI_ISL_2571147			
see above	University of Wuerzburg, Institute of Virology and Immunobiology	University of Wuerzburg, Institute of Virology and Immunobiology	Prifert,C., Hofmann,D. and Weissbrich,B.
EPI_ISL_2571171, EPI_ISL_2571172, EPI_ISL_2571173, EPI_ISL_2571174, EPI_ISL_2571175, EPI_ISL_2571176, EPI_ISL_2571177, EPI_ISL_2571178, EPI_ISL_2571179, EPI_ISL_2571180, EPI_ISL_2571183, EPI_ISL_2571184, EPI_ISL_2571185, EPI_ISL_2571186			
see above	Department of Virology, School of Public Health, Tehran University of Medical Sciences	Department of Virology, School of Public Health, Tehran University of Medical Sciences	Salimi,V., Samieipoor,Y., Ghavami,N. and Mokhtariazad,T.
EPI_ISL_2571235, EPI_ISL_2571237, EPI_ISL_2571239, EPI_ISL_2571242, EPI_ISL_2571243, EPI_ISL_2571244, EPI_ISL_2571245, EPI_ISL_2571248, EPI_ISL_2571249, EPI_ISL_2571251, EPI_ISL_2571252, EPI_ISL_2571256, EPI_ISL_2571257, EPI_ISL_2571258, EPI_ISL_2571261, EPI_ISL_2571262, EPI_ISL_2571263, EPI_ISL_2571264, EPI_ISL_2571268, EPI_ISL_2571269, EPI_ISL_2571272, EPI_ISL_2571273, EPI_ISL_2571274, EPI_ISL_2571275			
see above	Department of Infectious Diseases, National Health Institute Doutor Ricardo Jorge	Department of Infectious Diseases, National Health Institute Doutor Ricardo Jorge	Saez-Lopez,E., Cristovao,P., Costa,I., Pechirra,P., Conde,P. and Guiomar,R.
EPI_ISL_2571293, EPI_ISL_2571294, EPI_ISL_2571295, EPI_ISL_2571296, EPI_ISL_2571297, EPI_ISL_2571298, EPI_ISL_2571299, EPI_ISL_2571300, EPI_ISL_2571301, EPI_ISL_2571302, EPI_ISL_2571303, EPI_ISL_2571304, EPI_ISL_2571305, EPI_ISL_2571306, EPI_ISL_2571307, EPI_ISL_2571308, EPI_ISL_2571309, EPI_ISL_2571310, EPI_ISL_2571311, EPI_ISL_2571312, EPI_ISL_2571313, EPI_ISL_2571314, EPI_ISL_2571315, EPI_ISL_2571316, EPI_ISL_2571317, EPI_ISL_2571318, EPI_ISL_2571319, EPI_ISL_2571320, EPI_ISL_2571321, EPI_ISL_2571322, EPI_ISL_2571323, EPI_ISL_2571324, EPI_ISL_2571325, EPI_ISL_2571326, EPI_ISL_2571327, EPI_ISL_2571328, EPI_ISL_2571329, EPI_ISL_2571336, EPI_ISL_2571337, EPI_ISL_2571338, EPI_ISL_2571339, EPI_ISL_2571340, EPI_ISL_2571341, EPI_ISL_2571342, EPI_ISL_2571343, EPI_ISL_2571344, EPI_ISL_2571345, EPI_ISL_2571346, EPI_ISL_2571347, EPI_ISL_2571348, EPI_ISL_2571349			
see above	Microbiology, Hospital Universitari Vall d'Hebron	Microbiology, Hospital Universitari Vall d'Hebron	Gimferrer,L., Martin,Md.C., Pumarola,T. and Anton,A.
EPI_ISL_2571350, EPI_ISL_2571351, EPI_ISL_2571352, EPI_ISL_2571353, EPI_ISL_2571354, EPI_ISL_2571355, EPI_ISL_2571356, EPI_ISL_2571357, EPI_ISL_2571358, EPI_ISL_2571359	Department of Pediatrics, Center of Excellence in Clinical Virology, Chulalongkorn	Department of Pediatrics, Center of Excellence in Clinical Virology, Chulalongkorn	Thongpan,I.
EPI_ISL_2571360, EPI_ISL_2571363	Epidemiology and Immunogenetic of Viral Infections LR14SP02, University Hospital Sahloul	Epidemiology and Immunogenetic of Viral Infections LR14SP02, University Hospital Sahloul	Ataoui,I., Jerbi,A., BenHamida-Rebai,M., Ben Hadj Fredj,M., Fodha,I., Bennour,H., Hammouda,H., Khlifa,M., Brini,I., Boussoffara,R., Boussetta,K., Abroug,S. and Trabelsi,A.
EPI_ISL_2571381	Department of Virology, School of Public Health, Tehran University of Medical Sciences	Department of Virology, School of Public Health, Tehran University of Medical Sciences	Salimi,V., Samieipoor,Y., Ghavami,N. and Mokhtariazad,T.
EPI_ISL_2571384, EPI_ISL_2571385, EPI_ISL_2571386, EPI_ISL_2571388, EPI_ISL_2571389	College of Life Science and Technology, Jinan University	College of Life Science and Technology, Jinan University	Li,W., Wang,Y., Yu,B., Tan,Q., Zhou,J., Hu,J., Wu,Y., Wang,B. and Li,H.
EPI_ISL_2571400, EPI_ISL_2571404, EPI_ISL_2571405	Etiology and Epidemiology Department, Smorodintsev Research Institute of Influenza	Etiology and Epidemiology Department, Smorodintsev Research Institute of Influenza	Komissarova,K.S., Krivitskaya,V.Z., Sverlova,M.V., Petrova,E.R. and Ivanova,A.A.

EPI_ISL_2571406, EPI_ISL_2571407	Special Infectious Agents Unit, King Fahd Medical Research Center, King Abdulaziz University	Special Infectious Agents Unit, King Fahd Medical Research Center, King Abdulaziz University	El-Kafrawy,S.A., Alshareef,H. and Azhar,E.I.
EPI_ISL_2571493, EPI_ISL_2571494, EPI_ISL_2571495, EPI_ISL_2571496, EPI_ISL_2571497, EPI_ISL_2571498, EPI_ISL_2571499, EPI_ISL_2571500, EPI_ISL_2571501, EPI_ISL_2571502, EPI_ISL_2571503, EPI_ISL_2571504, EPI_ISL_2571505, EPI_ISL_2571506, EPI_ISL_2571507			
see above	Wint Wint Phyu Niigat University, International Health, Public Health	Wint Wint Phyu Niigat University, International Health, Public Health	Phyu,W.W. and Saito,R.
EPI_ISL_2571519, EPI_ISL_2571520, EPI_ISL_2571521, EPI_ISL_2571522, EPI_ISL_2571523, EPI_ISL_2571524, EPI_ISL_2571525, EPI_ISL_2571526, EPI_ISL_2571528, EPI_ISL_2571529, EPI_ISL_2571530			
see above	Medicine, American University of Beirut	Medicine, American University of Beirut	Abou-El-Hassan,H. and Zaraket,H.
EPI_ISL_2571531, EPI_ISL_2571532	Microbiology, Hospital Universitari Vall d'Hebron	Microbiology, Hospital Universitari Vall d'Hebron	Gimferrer,L., Martin,Md.C., Pumarola,T. and Anton,A.
EPI_ISL_2571569, EPI_ISL_2571570, EPI_ISL_2571571, EPI_ISL_2571573, EPI_ISL_2571574	College of Life Science and Technology, Jinan University	College of Life Science and Technology, Jinan University	Li,W., Wang,Y., Yu,B., Tan,Q., Zhou,J., Hu,J., Wu,Y., Wang,B. and Li,H.
EPI_ISL_2571578	Etiology and Epidemiology Department, Smorodintsev Research Institute of Influenza	Etiology and Epidemiology Department, Smorodintsev Research Institute of Influenza	Komissarova,K.S., Krivitskaya,V.Z., Sverlova,M.V., Petrova,E.R. and Ivanova,A.A.
EPI_ISL_2571580, EPI_ISL_2571581, EPI_ISL_2571582, EPI_ISL_2571583, EPI_ISL_2571584	Special Infectious Agents Unit, King Fahd Medical Research Center, King Abdulaziz University	Special Infectious Agents Unit, King Fahd Medical Research Center, King Abdulaziz University	El-Kafrawy,S.A., Alshareef,H. and Azhar,E.I.
EPI_ISL_2571598	Botany and Microbiology, College of Science, King Saud University, King Saud University	Botany and Microbiology, College of Science, King Saud University, King Saud University	Amer,H.M., Farrag,M.A., Hamad,M.E., Aziz,I.M. and Almajhdi,F.N.
EPI_ISL_2571605, EPI_ISL_2571606, EPI_ISL_2571607	Molecular Medicine Laboratory of Virology, Sapienza University	Molecular Medicine Laboratory of Virology, Sapienza University	Pierangeli,A.
EPI_ISL_2571614, EPI_ISL_2571615	Pediatrics, Show-Chwan Memorial Hospital	Pediatrics, Show-Chwan Memorial Hospital	Lee,C.-Y.
EPI_ISL_2571655	College of Life Science and Technology, Jinan University	College of Life Science and Technology, Jinan University	Li,W., Wang,Y., Yu,B., Tan,Q., Zhou,J., Hu,J., Wu,Y., Wang,B. and Li,H.
EPI_ISL_2571664, EPI_ISL_2571665	Special Infectious Agents Unit, King Fahd Medical Research Center, King Abdulaziz University	Special Infectious Agents Unit, King Fahd Medical Research Center, King Abdulaziz University	El-Kafrawy,S.A., Alshareef,H. and Azhar,E.I.
EPI_ISL_2571675	Molecular Virology, Blood Systems Research Institute	Molecular Virology, Blood Systems Research Institute	Altan,E. and Delwart,E.
EPI_ISL_2571739, EPI_ISL_2571741, EPI_ISL_2571742, EPI_ISL_2571743, EPI_ISL_2571749, EPI_ISL_2571751, EPI_ISL_2571752, EPI_ISL_2571754	Centre for Research And Knowledge Transfer in Biotechnology, University of Zagreb	Centre for Research And Knowledge Transfer in Biotechnology, University of Zagreb	Slovic,A., Ivancic-Jelecki,J., Ljubin-Sternak,S., Mlinaric-Galinovic,G., Vilibic-Cavlek,T., Tabain,I. and Forcic,D.
EPI_ISL_2571755, EPI_ISL_2571762, EPI_ISL_2571765, EPI_ISL_2571777, EPI_ISL_2571779, EPI_ISL_2571782, EPI_ISL_2571783, EPI_ISL_2571787, EPI_ISL_2571791	University of Wuerzburg, Institute of Virology and Immunobiology	University of Wuerzburg, Institute of Virology and Immunobiology	Prifert,C., Hofmann,D. and Weissbrich,B.
EPI_ISL_2571800, EPI_ISL_2571801, EPI_ISL_2571802, EPI_ISL_2571803, EPI_ISL_2571804, EPI_ISL_2571805, EPI_ISL_2571806, EPI_ISL_2571807, EPI_ISL_2571808, EPI_ISL_2571809, EPI_ISL_2571810, EPI_ISL_2571811, EPI_ISL_2571812, EPI_ISL_2571813, EPI_ISL_2571814, EPI_ISL_2571815, EPI_ISL_2571816			
see above	Department of Virology, School of Public Health, Tehran University of Medical Sciences	Department of Virology, School of Public Health, Tehran University of Medical Sciences	Salimi,V., Samieipoor,Y., Ghavami,N. and Mokhtariazad,T.
EPI_ISL_2571837, EPI_ISL_2571839, EPI_ISL_2571841, EPI_ISL_2571842, EPI_ISL_2571843, EPI_ISL_2571846, EPI_ISL_2571847, EPI_ISL_2571848, EPI_ISL_2571849, EPI_ISL_2571850, EPI_ISL_2571851, EPI_ISL_2571852, EPI_ISL_2571853			
see above	Department of Infectious Diseases, National Health Institute Doutor Ricardo Jorge	Department of Infectious Diseases, National Health Institute Doutor Ricardo Jorge	Saez-Lopez,E., Cristovao,P., Costa,I., Pechirra,P., Conde,P. and Guiomar,R.
EPI_ISL_2571857, EPI_ISL_2571858, EPI_ISL_2571859, EPI_ISL_2571860, EPI_ISL_2571861, EPI_ISL_2571862, EPI_ISL_2571863, EPI_ISL_2571864, EPI_ISL_2571865, EPI_ISL_2571866, EPI_ISL_2571867, EPI_ISL_2571868, EPI_ISL_2571869, EPI_ISL_2571870, EPI_ISL_2571871, EPI_ISL_2571872, EPI_ISL_2571873, EPI_ISL_2571874, EPI_ISL_2571875, EPI_ISL_2571876, EPI_ISL_2571877, EPI_ISL_2571878, EPI_ISL_2571879, EPI_ISL_2571880, EPI_ISL_2571881			
see above	Microbiology, Hospital Universitari Vall d'Hebron	Microbiology, Hospital Universitari Vall d'Hebron	Gimferrer,L., Martin,Md.C., Pumarola,T. and Anton,A.
EPI_ISL_2571882, EPI_ISL_2571883, EPI_ISL_2571884, EPI_ISL_2571885, EPI_ISL_2571886, EPI_ISL_2571887, EPI_ISL_2571888	Department of Pediatrics, Center of Excellence in Clinical Virology, Chulalongkorn	Department of Pediatrics, Center of Excellence in Clinical Virology, Chulalongkorn	Thongpan,I.
EPI_ISL_2571890, EPI_ISL_2571891, EPI_ISL_2571892	Epidemiology and Immunogenetic of Viral Infections LR14SP02, University Hospital Sahloul	Epidemiology and Immunogenetic of Viral Infections LR14SP02, University Hospital Sahloul	Ataoui,I., Jerbi,A., BenHamida-Rebai,M., Ben Hadj Fredj,M., Fodha,I., Bennour,H., Hammouda,H., Khlifa,M., Brini,I., Boussoffara,R., Boussetta,K., Abroug,S. and Trabelsi,A.
EPI_ISL_2571901	Department of Virology, School of Public Health, Tehran University of Medical Sciences	Department of Virology, School of Public Health, Tehran University of Medical Sciences	Salimi,V., Samieipoor,Y., Ghavami,N. and Mokhtariazad,T.
EPI_ISL_2571902, EPI_ISL_2571903	Special Infectious Agents Unit, King Fahd Medical Research Center, King Abdulaziz University	Special Infectious Agents Unit, King Fahd Medical Research Center, King Abdulaziz University	El-Kafrawy,S.A., Alshareef,H. and Azhar,E.I.
EPI_ISL_2571927, EPI_ISL_2571928, EPI_ISL_2571929, EPI_ISL_2571930, EPI_ISL_2571931, EPI_ISL_2571932, EPI_ISL_2571933, EPI_ISL_2571934, EPI_ISL_2571935, EPI_ISL_2571936, EPI_ISL_2571937, EPI_ISL_2571938, EPI_ISL_2571939, EPI_ISL_2571940, EPI_ISL_2571941, EPI_ISL_2571942, EPI_ISL_2571943			
see above	Wint Wint Phyu Niigat University, International Health, Public Health	Wint Wint Phyu Niigat University, International Health, Public Health	Phyu,W.W. and Saito,R.
EPI_ISL_2571945	Medicine, American University of Beirut	Medicine, American University of Beirut	Abou-El-Hassan,H. and Zaraket,H.
EPI_ISL_2571949, EPI_ISL_2571950	Molecular Medicine Laboratory of Virology, Sapienza University	Molecular Medicine Laboratory of Virology, Sapienza University	Pierangeli,A.
EPI_ISL_2571960	College of Life Science and Technology, Jinan University	College of Life Science and Technology, Jinan University	Li,W., Wang,Y., Yu,B., Tan,Q., Zhou,J., Hu,J., Wu,Y., Wang,B. and Li,H.
EPI_ISL_2571961	Special Infectious Agents Unit, King Fahd Medical Research Center, King Abdulaziz University	Special Infectious Agents Unit, King Fahd Medical Research Center, King Abdulaziz University	El-Kafrawy,S.A., Alshareef,H. and Azhar,E.I.
EPI_ISL_2571963	Botany and Microbiology, College of Science, King Saud University, King Saud University	Botany and Microbiology, College of Science, King Saud University, King Saud University	Amer,H.M., Farrag,M.A., Hamad,M.E., Aziz,I.M. and Almajhdi,F.N.
EPI_ISL_2571967	Centre for Infectious Diseases Research, Diagnostics and laboratory Surveillance, National Institute for Public Health and the Environment	Centre for Infectious Diseases Research, Diagnostics and laboratory Surveillance, National Institute for Public Health and the Environment	Meijer,A. and Overduin,P.
EPI_ISL_2572001, EPI_ISL_2572002, EPI_ISL_2572005	University of Wuerzburg, Institute of Virology and Immunobiology	University of Wuerzburg, Institute of Virology and Immunobiology	Prifert,C., Hofmann,D. and Weissbrich,B.
EPI_ISL_2572011	Department of Virology, School of Public Health, Tehran University of Medical Sciences	Department of Virology, School of Public Health, Tehran University of Medical Sciences	Salimi,V., Samieipoor,Y., Ghavami,N. and Mokhtariazad,T.
EPI_ISL_2572016, EPI_ISL_2572018, EPI_ISL_2572019, EPI_ISL_2572020, EPI_ISL_2572021	Department of Infectious Diseases, National Health Institute Doutor Ricardo Jorge	Department of Infectious Diseases, National Health Institute Doutor Ricardo Jorge	Saez-Lopez,E., Cristovao,P., Costa,I., Pechirra,P., Conde,P. and Guiomar,R.
EPI_ISL_2572029, EPI_ISL_2572030, EPI_ISL_2572031, EPI_ISL_2572032, EPI_ISL_2572033	Microbiology, Hospital Universitari Vall d'Hebron	Microbiology, Hospital Universitari Vall d'Hebron	Gimferrer,L., Martin,Md.C., Pumarola,T. and Anton,A.
EPI_ISL_2572035, EPI_ISL_2572036, EPI_ISL_2572037	Epidemiology and Immunogenetic of Viral Infections LR14SP02, University Hospital Sahloul	Epidemiology and Immunogenetic of Viral Infections LR14SP02, University Hospital Sahloul	Ataoui,I., Jerbi,A., BenHamida-Rebai,M., Ben Hadj Fredj,M., Fodha,I., Bennour,H., Hammouda,H., Khlifa,M., Brini,I., Boussoffara,R., Boussetta,K., Abroug,S. and Trabelsi,A.
EPI_ISL_2572044, EPI_ISL_2572045, EPI_ISL_2572046, EPI_ISL_2572047	Wint Wint Phyu Niigat University, International Health, Public Health	Wint Wint Phyu Niigat University, International Health, Public Health	Phyu,W.W. and Saito,R.
EPI_ISL_2572056	Centre for Research And Knowledge Transfer in Biotechnology, University of Zagreb	Centre for Research And Knowledge Transfer in Biotechnology, University of Zagreb	Slovic,A., Ivancic-Jelecki,J., Ljubin-Sternak,S., Mlinaric-Galinovic,G., Vilibic-Cavlek,T., Tabain,I. and Forcic,D.
EPI_ISL_2572058	Department of Infectious Diseases, National Health Institute Doutor Ricardo Jorge	Department of Infectious Diseases, National Health Institute Doutor Ricardo Jorge	Saez-Lopez,E., Cristovao,P., Costa,I., Pechirra,P., Conde,P. and Guiomar,R.
EPI_ISL_2572060, EPI_ISL_2572061, EPI_ISL_2572062	Microbiology, Hospital Universitari Vall d'Hebron	Microbiology, Hospital Universitari Vall d'Hebron	Gimferrer,L., Martin,Md.C., Pumarola,T. and Anton,A.
EPI_ISL_2572063	Department of Pediatrics, Center of Excellence in Clinical Virology, Chulalongkorn	Department of Pediatrics, Center of Excellence in Clinical Virology, Chulalongkorn	Thongpan,I.
EPI_ISL_2572074	Wint Wint Phyu Niigat University, International Health, Public Health	Wint Wint Phyu Niigat University, International Health, Public Health	Phyu,W.W. and Saito,R.
EPI_ISL_2572079	Microbiology, Hospital Universitari Vall d'Hebron	Microbiology, Hospital Universitari Vall d'Hebron	Gimferrer,L., Martin,Md.C., Pumarola,T. and Anton,A.
EPI_ISL_2572092, EPI_ISL_2572093	Wint Wint Phyu Niigat University, International Health, Public Health	Wint Wint Phyu Niigat University, International Health, Public Health	Phyu,W.W. and Saito,R.
EPI_ISL_2572105	Centre for Research And Knowledge Transfer in Biotechnology, University of Zagreb	Centre for Research And Knowledge Transfer in Biotechnology, University of Zagreb	Slovic,A., Ivancic-Jelecki,J., Ljubin-Sternak,S., Mlinaric-Galinovic,G., Vilibic-Cavlek,T., Tabain,I. and Forcic,D.
EPI_ISL_2572106	Department of Infectious Diseases, National Health Institute Doutor Ricardo Jorge	Department of Infectious Diseases, National Health Institute Doutor Ricardo Jorge	Saez-Lopez,E., Cristovao,P., Costa,I., Pechirra,P., Conde,P. and Guiomar,R.
EPI_ISL_2572205, EPI_ISL_2572206, EPI_ISL_2572207, EPI_ISL_2572208, EPI_ISL_2572218, EPI_ISL_2572225	College of Life Science and Technology, Jinan University	College of Life Science and Technology, Jinan University	Li,W., Wang,Y., Yu,B., Tan,Q., Zhou,J., Hu,J., Wu,Y., Wang,B. and Li,H.
EPI_ISL_2572291, EPI_ISL_2572298, EPI_ISL_2572299, EPI_ISL_2572304, EPI_ISL_2572305, EPI_ISL_2572307	University of Wuerzburg, Institute of Virology and Immunobiology	University of Wuerzburg, Institute of Virology and Immunobiology	Prifert,C., Hofmann,D. and Weissbrich,B.
EPI_ISL_2572331, EPI_ISL_2572332, EPI_ISL_2572333, EPI_ISL_2572334, EPI_ISL_2572335, EPI_ISL_2572336, EPI_ISL_2572337, EPI_ISL_2572338, EPI_ISL_2572339, EPI_ISL_2572340, EPI_ISL_2572343, EPI_ISL_2572344, EPI_ISL_2572345, EPI_ISL_2572346			
see above	Department of Virology, School of Public Health, Tehran University of Medical Sciences	Department of Virology, School of Public Health, Tehran University of Medical Sciences	Salimi,V., Samieipoor,Y., Ghavami,N. and Mokhtariazad,T.
EPI_ISL_2572395, EPI_ISL_2572397, EPI_ISL_2572399, EPI_ISL_2572402, EPI_ISL_2572403, EPI_ISL_2572404, EPI_ISL_2572405, EPI_ISL_2572408, EPI_ISL_2572409, EPI_ISL_2572411, EPI_ISL_2572412, EPI_ISL_2572416, EPI_ISL_2572417, EPI_ISL_2572418, EPI_ISL_2572421, EPI_ISL_2572422, EPI_ISL_2572423, EPI_ISL_2572424, EPI_ISL_2572428, EPI_ISL_2572429, EPI_ISL_2572432, EPI_ISL_2572433,			

EPI_ISL_2572434, EPI_ISL_2572435			
see above	Department of Infectious Diseases, National Health Institute Doutor Ricardo Jorge	Department of Infectious Diseases, National Health Institute Doutor Ricardo Jorge	Saez-Lopez,E., Cristovao,P., Costa,I., Pechirra,P., Conde,P. and Guiomar,R.
EPI_ISL_2572453, EPI_ISL_2572454, EPI_ISL_2572455, EPI_ISL_2572456, EPI_ISL_2572457, EPI_ISL_2572458, EPI_ISL_2572459, EPI_ISL_2572460, EPI_ISL_2572461, EPI_ISL_2572462, EPI_ISL_2572463, EPI_ISL_2572464, EPI_ISL_2572465, EPI_ISL_2572466, EPI_ISL_2572467, EPI_ISL_2572468, EPI_ISL_2572469, EPI_ISL_2572470, EPI_ISL_2572471, EPI_ISL_2572472, EPI_ISL_2572473, EPI_ISL_2572474, EPI_ISL_2572475, EPI_ISL_2572476, EPI_ISL_2572477, EPI_ISL_2572478, EPI_ISL_2572479, EPI_ISL_2572480, EPI_ISL_2572481, EPI_ISL_2572482, EPI_ISL_2572483, EPI_ISL_2572484, EPI_ISL_2572485, EPI_ISL_2572486, EPI_ISL_2572487, EPI_ISL_2572488, EPI_ISL_2572489, EPI_ISL_2572496, EPI_ISL_2572497, EPI_ISL_2572498, EPI_ISL_2572499, EPI_ISL_2572500, EPI_ISL_2572501, EPI_ISL_2572502, EPI_ISL_2572503, EPI_ISL_2572504, EPI_ISL_2572505, EPI_ISL_2572506, EPI_ISL_2572507, EPI_ISL_2572508, EPI_ISL_2572509			
see above	Microbiology, Hospital Universitari Vall d'Hebron	Microbiology, Hospital Universitari Vall d'Hebron	Gimferrer,L., Martin,Md.C., Pumarola,T. and Anton,A.
EPI_ISL_2572510, EPI_ISL_2572511, EPI_ISL_2572512, EPI_ISL_2572513, EPI_ISL_2572514, EPI_ISL_2572515, EPI_ISL_2572516, EPI_ISL_2572517, EPI_ISL_2572518, EPI_ISL_2572519	Department of Pediatrics, Center of Excellence in Clinical Virology, Chulalongkorn	Department of Pediatrics, Center of Excellence in Clinical Virology, Chulalongkorn	Thongpan,I.
EPI_ISL_2572520, EPI_ISL_2572523	Epidemiology and Immunogenetic of Viral Infections LR14SP02, University Hospital Sahloul	Epidemiology and Immunogenetic of Viral Infections LR14SP02, University Hospital Sahloul	Ataoui,I., Jerbi,A., BenHamida-Rebai,M., Ben Hadj Fredj,M., Fodha,I., Bennour,H., Hammouda,H., Khlifa,M., Brini,I., Boussoffara,R., Boussetta,K., Abroug,S. and Trabelsi,A.
EPI_ISL_2572541	Department of Virology, School of Public Health, Tehran University of Medical Sciences	Department of Virology, School of Public Health, Tehran University of Medical Sciences	Salimi,V., Samieipoor,Y., Ghavami,N. and Mokhtariazad,T.
EPI_ISL_2572544, EPI_ISL_2572545, EPI_ISL_2572546, EPI_ISL_2572548, EPI_ISL_2572549	College of Life Science and Technology, Jinan University	College of Life Science and Technology, Jinan University	Li,W., Wang,Y., Yu,B., Tan,Q., Zhou,J., Hu,J., Wu,Y., Wang,B. and Li,H.
EPI_ISL_2572560, EPI_ISL_2572564, EPI_ISL_2572565	Etiology and Epidemiology Department, Smorodintsev Research Institute of Influenza	Etiology and Epidemiology Department, Smorodintsev Research Institute of Influenza	Komissarova,K.S., Krivitskaya,V.Z., Sverlova,M.V., Petrova,E.R. and Ivanova,A.A.
EPI_ISL_2572566, EPI_ISL_2572567	Special Infectious Agents Unit, King Fahd Medical Research Center, King Abdulaziz University	Special Infectious Agents Unit, King Fahd Medical Research Center, King Abdulaziz University	El-Kafrawy,S.A., Alshareef,H. and Azhar,E.I.
EPI_ISL_2572653, EPI_ISL_2572654, EPI_ISL_2572655, EPI_ISL_2572656, EPI_ISL_2572657, EPI_ISL_2572658, EPI_ISL_2572659, EPI_ISL_2572660, EPI_ISL_2572661, EPI_ISL_2572662, EPI_ISL_2572663, EPI_ISL_2572664, EPI_ISL_2572665, EPI_ISL_2572666, EPI_ISL_2572667			
see above	Wint Wint Phyu Niigat University, International Health, Public Health	Wint Wint Phyu Niigat University, International Health, Public Health	Phyu,W.W. and Saito,R.
EPI_ISL_2572679, EPI_ISL_2572680, EPI_ISL_2572681, EPI_ISL_2572682, EPI_ISL_2572683, EPI_ISL_2572684, EPI_ISL_2572685, EPI_ISL_2572686, EPI_ISL_2572688, EPI_ISL_2572689, EPI_ISL_2572690			
see above	Medicine, American University of Beirut	Medicine, American University of Beirut	Abou-El-Hassan,H. and Zaraket,H.
EPI_ISL_2572691, EPI_ISL_2572692	Microbiology, Hospital Universitari Vall d'Hebron	Microbiology, Hospital Universitari Vall d'Hebron	Gimferrer,L., Martin,Md.C., Pumarola,T. and Anton,A.
EPI_ISL_2572729, EPI_ISL_2572730, EPI_ISL_2572731, EPI_ISL_2572733, EPI_ISL_2572734	College of Life Science and Technology, Jinan University	College of Life Science and Technology, Jinan University	Li,W., Wang,Y., Yu,B., Tan,Q., Zhou,J., Hu,J., Wu,Y., Wang,B. and Li,H.
EPI_ISL_2572738	Etiology and Epidemiology Department, Smorodintsev Research Institute of Influenza	Etiology and Epidemiology Department, Smorodintsev Research Institute of Influenza	Komissarova,K.S., Krivitskaya,V.Z., Sverlova,M.V., Petrova,E.R. and Ivanova,A.A.
EPI_ISL_2572740, EPI_ISL_2572741, EPI_ISL_2572742, EPI_ISL_2572743, EPI_ISL_2572744	Special Infectious Agents Unit, King Fahd Medical Research Center, King Abdulaziz University	Special Infectious Agents Unit, King Fahd Medical Research Center, King Abdulaziz University	El-Kafrawy,S.A., Alshareef,H. and Azhar,E.I.
EPI_ISL_2572758	Botany and Microbiology, College of Science, King Saud University, King Saud University	Botany and Microbiology, College of Science, King Saud University, King Saud University	Amer,H.M., Farrag,M.A., Hamad,M.E., Aziz,I.M. and Almajhdi,F.N.
EPI_ISL_2572765, EPI_ISL_2572766, EPI_ISL_2572767	Molecular Medicine Laboratory of Virology, Sapienza University	Molecular Medicine Laboratory of Virology, Sapienza University	Pierangeli,A.
EPI_ISL_2572774, EPI_ISL_2572775	Pediatrics, Show-Chwan Memorial Hospital	Pediatrics, Show-Chwan Memorial Hospital	Lee,C.-Y.
EPI_ISL_2572815	College of Life Science and Technology, Jinan University	College of Life Science and Technology, Jinan University	Li,W., Wang,Y., Yu,B., Tan,Q., Zhou,J., Hu,J., Wu,Y., Wang,B. and Li,H.
EPI_ISL_2572824, EPI_ISL_2572825	Special Infectious Agents Unit, King Fahd Medical Research Center, King Abdulaziz University	Special Infectious Agents Unit, King Fahd Medical Research Center, King Abdulaziz University	El-Kafrawy,S.A., Alshareef,H. and Azhar,E.I.
EPI_ISL_2572835	Molecular Virology, Blood Systems Research Institute	Molecular Virology, Blood Systems Research Institute	Altan,E. and Delwart,E.
EPI_ISL_2572899, EPI_ISL_2572901, EPI_ISL_2572902, EPI_ISL_2572903, EPI_ISL_2572909, EPI_ISL_2572911, EPI_ISL_2572912, EPI_ISL_2572914	Centre for Research And Knowledge Transfer in Biotechnology, University of Zagreb	Centre for Research And Knowledge Transfer in Biotechnology, University of Zagreb	Slovic,A., Ivancic-Jelecki,J., Ljubin-Sternak,S., Mlinaric-Galinovic,G., Vilibic-Cavlek,T., Tabain,I. and Forcic,D.
EPI_ISL_2572915, EPI_ISL_2572922, EPI_ISL_2572925, EPI_ISL_2572937, EPI_ISL_2572939, EPI_ISL_2572942, EPI_ISL_2572943, EPI_ISL_2572947, EPI_ISL_2572951	University of Wuerzburg, Institute of Virology and Immunobiology	University of Wuerzburg, Institute of Virology and Immunobiology	Prifert,C., Hofmann,D. and Weissbrich,B.
EPI_ISL_2572960, EPI_ISL_2572961, EPI_ISL_2572962, EPI_ISL_2572963, EPI_ISL_2572964, EPI_ISL_2572965, EPI_ISL_2572966, EPI_ISL_2572967, EPI_ISL_2572968, EPI_ISL_2572969, EPI_ISL_2572970, EPI_ISL_2572971, EPI_ISL_2572972, EPI_ISL_2572973, EPI_ISL_2572974, EPI_ISL_2572975, EPI_ISL_2572976			
see above	Department of Virology, School of Public Health, Tehran University of Medical Sciences	Department of Virology, School of Public Health, Tehran University of Medical Sciences	Salimi,V., Samieipoor,Y., Ghavami,N. and Mokhtariazad,T.
EPI_ISL_2572997, EPI_ISL_2572999, EPI_ISL_2573001, EPI_ISL_2573002, EPI_ISL_2573003, EPI_ISL_2573006, EPI_ISL_2573007, EPI_ISL_2573008, EPI_ISL_2573009, EPI_ISL_2573010, EPI_ISL_2573011, EPI_ISL_2573012, EPI_ISL_2573013			
see above	Department of Infectious Diseases, National Health Institute Doutor Ricardo Jorge	Department of Infectious Diseases, National Health Institute Doutor Ricardo Jorge	Saez-Lopez,E., Cristovao,P., Costa,I., Pechirra,P., Conde,P. and Guiomar,R.
EPI_ISL_2573017, EPI_ISL_2573018, EPI_ISL_2573019, EPI_ISL_2573020, EPI_ISL_2573021, EPI_ISL_2573022, EPI_ISL_2573023, EPI_ISL_2573024, EPI_ISL_2573025, EPI_ISL_2573026, EPI_ISL_2573027, EPI_ISL_2573028, EPI_ISL_2573029, EPI_ISL_2573030, EPI_ISL_2573031, EPI_ISL_2573032, EPI_ISL_2573033, EPI_ISL_2573034, EPI_ISL_2573035, EPI_ISL_2573036, EPI_ISL_2573037, EPI_ISL_2573038, EPI_ISL_2573039, EPI_ISL_2573040, EPI_ISL_2573041			
see above	Microbiology, Hospital Universitari Vall d'Hebron	Microbiology, Hospital Universitari Vall d'Hebron	Gimferrer,L., Martin,Md.C., Pumarola,T. and Anton,A.
EPI_ISL_2573042, EPI_ISL_2573043, EPI_ISL_2573044, EPI_ISL_2573045, EPI_ISL_2573046, EPI_ISL_2573047, EPI_ISL_2573048	Department of Pediatrics, Center of Excellence in Clinical Virology, Chulalongkorn	Department of Pediatrics, Center of Excellence in Clinical Virology, Chulalongkorn	Thongpan,I.
EPI_ISL_2573050, EPI_ISL_2573051, EPI_ISL_2573052	Epidemiology and Immunogenetic of Viral Infections LR14SP02, University Hospital Sahloul	Epidemiology and Immunogenetic of Viral Infections LR14SP02, University Hospital Sahloul	Ataoui,I., Jerbi,A., BenHamida-Rebai,M., Ben Hadj Fredj,M., Fodha,I., Bennour,H., Hammouda,H., Khlifa,M., Brini,I., Boussoffara,R., Boussetta,K., Abroug,S. and Trabelsi,A.
EPI_ISL_2573061	Department of Virology, School of Public Health, Tehran University of Medical Sciences	Department of Virology, School of Public Health, Tehran University of Medical Sciences	Salimi,V., Samieipoor,Y., Ghavami,N. and Mokhtariazad,T.
EPI_ISL_2573062, EPI_ISL_2573063	Special Infectious Agents Unit, King Fahd Medical Research Center, King Abdulaziz University	Special Infectious Agents Unit, King Fahd Medical Research Center, King Abdulaziz University	El-Kafrawy,S.A., Alshareef,H. and Azhar,E.I.
EPI_ISL_2573087, EPI_ISL_2573088, EPI_ISL_2573089, EPI_ISL_2573090, EPI_ISL_2573091, EPI_ISL_2573092, EPI_ISL_2573093, EPI_ISL_2573094, EPI_ISL_2573095, EPI_ISL_2573096, EPI_ISL_2573097, EPI_ISL_2573098, EPI_ISL_2573099, EPI_ISL_2573100, EPI_ISL_2573101, EPI_ISL_2573102, EPI_ISL_2573103			
see above	Wint Wint Phyu Niigat University, International Health, Public Health	Wint Wint Phyu Niigat University, International Health, Public Health	Phyu,W.W. and Saito,R.
EPI_ISL_2573105	Medicine, American University of Beirut	Medicine, American University of Beirut	Abou-El-Hassan,H. and Zaraket,H.
EPI_ISL_2573109, EPI_ISL_2573110	Molecular Medicine Laboratory of Virology, Sapienza University	Molecular Medicine Laboratory of Virology, Sapienza University	Pierangeli,A.
EPI_ISL_2573120	College of Life Science and Technology, Jinan University	College of Life Science and Technology, Jinan University	Li,W., Wang,Y., Yu,B., Tan,Q., Zhou,J., Hu,J., Wu,Y., Wang,B. and Li,H.
EPI_ISL_2573121	Special Infectious Agents Unit, King Fahd Medical Research Center, King Abdulaziz University	Special Infectious Agents Unit, King Fahd Medical Research Center, King Abdulaziz University	El-Kafrawy,S.A., Alshareef,H. and Azhar,E.I.
EPI_ISL_2573123	Botany and Microbiology, College of Science, King Saud University, King Saud University	Botany and Microbiology, College of Science, King Saud University, King Saud University	Amer,H.M., Farrag,M.A., Hamad,M.E., Aziz,I.M. and Almajhdi,F.N.
EPI_ISL_2573127	Centre for Infectious Diseases Research, Diagnostics and laboratory Surveillance, National Institute for Public Health and the Environment	Centre for Infectious Diseases Research, Diagnostics and laboratory Surveillance, National Institute for Public Health and the Environment	Meijer,A. and Overduin,P.
EPI_ISL_2573161, EPI_ISL_2573162, EPI_ISL_2573165	University of Wuerzburg, Institute of Virology and Immunobiology	University of Wuerzburg, Institute of Virology and Immunobiology	Prifert,C., Hofmann,D. and Weissbrich,B.
EPI_ISL_2573171	Department of Virology, School of Public Health, Tehran University of Medical Sciences	Department of Virology, School of Public Health, Tehran University of Medical Sciences	Salimi,V., Samieipoor,Y., Ghavami,N. and Mokhtariazad,T.
EPI_ISL_2573176, EPI_ISL_2573178, EPI_ISL_2573179, EPI_ISL_2573180, EPI_ISL_2573181	Department of Infectious Diseases, National Health Institute Doutor Ricardo Jorge	Department of Infectious Diseases, National Health Institute Doutor Ricardo Jorge	Saez-Lopez,E., Cristovao,P., Costa,I., Pechirra,P., Conde,P. and Guiomar,R.
EPI_ISL_2573189, EPI_ISL_2573190, EPI_ISL_2573191, EPI_ISL_2573192, EPI_ISL_2573193	Microbiology, Hospital Universitari Vall d'Hebron	Microbiology, Hospital Universitari Vall d'Hebron	Gimferrer,L., Martin,Md.C., Pumarola,T. and Anton,A.
EPI_ISL_2573195, EPI_ISL_2573196, EPI_ISL_2573197	Epidemiology and Immunogenetic of Viral Infections LR14SP02, University Hospital Sahloul	Epidemiology and Immunogenetic of Viral Infections LR14SP02, University Hospital Sahloul	Ataoui,I., Jerbi,A., BenHamida-Rebai,M., Ben Hadj Fredj,M., Fodha,I., Bennour,H., Hammouda,H., Khlifa,M., Brini,I., Boussoffara,R., Boussetta,K., Abroug,S. and Trabelsi,A.
EPI_ISL_2573204, EPI_ISL_2573205, EPI_ISL_2573206, EPI_ISL_2573207	Wint Wint Phyu Niigat University, International Health, Public Health	Wint Wint Phyu Niigat University, International Health, Public Health	Phyu,W.W. and Saito,R.
EPI_ISL_2573216	Centre for Research And Knowledge Transfer in Biotechnology, University of Zagreb	Centre for Research And Knowledge Transfer in Biotechnology, University of Zagreb	Slovic,A., Ivancic-Jelecki,J., Ljubin-Sternak,S., Mlinaric-Galinovic,G., Vilibic-Cavlek,T., Tabain,I. and Forcic,D.
EPI_ISL_2573218	Department of Infectious Diseases, National Health Institute Doutor Ricardo Jorge	Department of Infectious Diseases, National Health Institute Doutor Ricardo Jorge	Saez-Lopez,E., Cristovao,P., Costa,I., Pechirra,P., Conde,P. and Guiomar,R.
EPI_ISL_2573220, EPI_ISL_2573221, EPI_ISL_2573222	Microbiology, Hospital Universitari Vall d'Hebron	Microbiology, Hospital Universitari Vall d'Hebron	Gimferrer,L., Martin,Md.C., Pumarola,T. and Anton,A.

EPI_ISL_2573223	Department of Pediatrics, Center of Excellence in Clinical Virology, Chulalongkorn	Department of Pediatrics, Center of Excellence in Clinical Virology, Chulalongkorn	Thongpan,I.
EPI_ISL_2573234	Wint Wint Phyu Niigat University, International Health, Public Health	Wint Wint Phyu Niigat University, International Health, Public Health	Phyu,W.W. and Saito,R.
EPI_ISL_2573239	Microbiology, Hospital Universitari Vall d'Hebron	Microbiology, Hospital Universitari Vall d'Hebron	Gimferrer,L., Martin,Md.C., Pumarola,T. and Anton,A.
EPI_ISL_2573252, EPI_ISL_2573253	Wint Wint Phyu Niigat University, International Health, Public Health	Wint Wint Phyu Niigat University, International Health, Public Health	Phyu,W.W. and Saito,R.
EPI_ISL_2573265	Centre for Research And Knowledge Transfer in Biotechnology, University of Zagreb	Centre for Research And Knowledge Transfer in Biotechnology, University of Zagreb	Slovic,A., Ivancic-Jelecki,J., Ljubin-Sternak,S., Mlinaric-Galinovic,G., Vilibic-Cavlek,T., Tabain,I. and Forcic,D.
EPI_ISL_2573266	Department of Infectious Diseases, National Health Institute Doutor Ricardo Jorge	Department of Infectious Diseases, National Health Institute Doutor Ricardo Jorge	Saez-Lopez,E., Cristovao,P., Costa,I., Pechirra,P., Conde,P. and Guiomar,R.
EPI_ISL_2573365, EPI_ISL_2573366, EPI_ISL_2573367, EPI_ISL_2573368, EPI_ISL_2573378, EPI_ISL_2573385	College of Life Science and Technology, Jinan University	College of Life Science and Technology, Jinan University	Li,W., Wang,Y., Yu,B., Tan,Q., Zhou,J., Hu,J., Wu,Y., Wang,B. and Li,H.
EPI_ISL_2573547, EPI_ISL_2573548, EPI_ISL_2573551, EPI_ISL_2573606, EPI_ISL_2573613, EPI_ISL_2573617	Centre for Research And Knowledge Transfer in Biotechnology, University of Zagreb	Centre for Research And Knowledge Transfer in Biotechnology, University of Zagreb	Slovic,A., Ivancic-Jelecki,J., Ljubin-Sternak,S., Mlinaric-Galinovic,G., Vilibic-Cavlek,T., Tabain,I. and Forcic,D.
EPI_ISL_2573680, EPI_ISL_2573681, EPI_ISL_2573684, EPI_ISL_2573687, EPI_ISL_2573688, EPI_ISL_2573697, EPI_ISL_2573699, EPI_ISL_2573700, EPI_ISL_2573725, EPI_ISL_2573728, EPI_ISL_2573731, EPI_ISL_2573738, EPI_ISL_2573768, EPI_ISL_2573769			
see above	University of Wuerzburg, Institute of Virology and Immunobiology	University of Wuerzburg, Institute of Virology and Immunobiology	Prifert,C., Hofmann,D. and Weissbrich,B.
EPI_ISL_2573851, EPI_ISL_2573852, EPI_ISL_2573853	Department of Virology, School of Public Health, Tehran University of Medical Sciences	Department of Virology, School of Public Health, Tehran University of Medical Sciences	Salimi,V., Samieipoor,Y., Ghavami,N. and Mokhtariazad,T.
EPI_ISL_2573854, EPI_ISL_2573855	Department of Virology, Tehran University of Medical Sciences	Department of Virology, Tehran University of Medical Sciences	Salimi,V., Shatizadeh Malekshahi,S., Ghavami,N. and Mokhtari Azad,T.
EPI_ISL_2573856	Department of Virology, School of Public Health, Tehran University of Medical Sciences	Department of Virology, School of Public Health, Tehran University of Medical Sciences	Salimi,V., Samieipoor,Y., Ghavami,N. and Mokhtariazad,T.
EPI_ISL_2573857	Department of Virology, Tehran University of Medical Sciences	Department of Virology, Tehran University of Medical Sciences	Salimi,V., Shatizadeh Malekshahi,S., Ghavami,N. and Mokhtari Azad,T.
EPI_ISL_2573858, EPI_ISL_2573859	Department of Virology, School of Public Health, Tehran University of Medical Sciences	Department of Virology, School of Public Health, Tehran University of Medical Sciences	Salimi,V., Samieipoor,Y., Ghavami,N. and Mokhtariazad,T.
EPI_ISL_2573860	Department of Virology, Tehran University of Medical Sciences	Department of Virology, Tehran University of Medical Sciences	Salimi,V., Shatizadeh Malekshahi,S., Ghavami,N. and Mokhtari Azad,T.
EPI_ISL_2573861	Department of Virology, School of Public Health, Tehran University of Medical Sciences	Department of Virology, School of Public Health, Tehran University of Medical Sciences	Salimi,V., Samieipoor,Y., Ghavami,N. and Mokhtariazad,T.
EPI_ISL_2573862	Department of Virology, Tehran University of Medical Sciences	Department of Virology, Tehran University of Medical Sciences	Salimi,V., Shatizadeh Malekshahi,S., Ghavami,N. and Mokhtari Azad,T.
EPI_ISL_2573863, EPI_ISL_2573864, EPI_ISL_2573865, EPI_ISL_2573866	Department of Virology, School of Public Health, Tehran University of Medical Sciences	Department of Virology, School of Public Health, Tehran University of Medical Sciences	Salimi,V., Samieipoor,Y., Ghavami,N. and Mokhtariazad,T.
EPI_ISL_2573867	Department of Virology, Tehran University of Medical Sciences	Department of Virology, Tehran University of Medical Sciences	Salimi,V., Shatizadeh Malekshahi,S., Ghavami,N. and Mokhtari Azad,T.
EPI_ISL_2573868	Department of Virology, School of Public Health, Tehran University of Medical Sciences	Department of Virology, School of Public Health, Tehran University of Medical Sciences	Salimi,V., Samieipoor,Y., Ghavami,N. and Mokhtariazad,T.
EPI_ISL_2573869	Department of Virology, Tehran University of Medical Sciences	Department of Virology, Tehran University of Medical Sciences	Salimi,V., Shatizadeh Malekshahi,S., Ghavami,N. and Mokhtari Azad,T.
EPI_ISL_2573870	Department of Virology, School of Public Health, Tehran University of Medical Sciences	Department of Virology, School of Public Health, Tehran University of Medical Sciences	Salimi,V., Samieipoor,Y., Ghavami,N. and Mokhtariazad,T.
EPI_ISL_2573871	Department of Virology, Tehran University of Medical Sciences	Department of Virology, Tehran University of Medical Sciences	Salimi,V., Shatizadeh Malekshahi,S., Ghavami,N. and Mokhtari Azad,T.
EPI_ISL_2573872	Department of Virology, School of Public Health, Tehran University of Medical Sciences	Department of Virology, School of Public Health, Tehran University of Medical Sciences	Salimi,V., Samieipoor,Y., Ghavami,N. and Mokhtariazad,T.
EPI_ISL_2573873, EPI_ISL_2573874	Department of Virology, Tehran University of Medical Sciences	Department of Virology, Tehran University of Medical Sciences	Salimi,V., Shatizadeh Malekshahi,S., Ghavami,N. and Mokhtari Azad,T.
EPI_ISL_2573875, EPI_ISL_2573876	Department of Virology, School of Public Health, Tehran University of Medical Sciences	Department of Virology, School of Public Health, Tehran University of Medical Sciences	Salimi,V., Samieipoor,Y., Ghavami,N. and Mokhtariazad,T.
EPI_ISL_2573877	Department of Virology, Tehran University of Medical Sciences	Department of Virology, Tehran University of Medical Sciences	Salimi,V., Shatizadeh Malekshahi,S., Ghavami,N. and Mokhtari Azad,T.
EPI_ISL_2573881	Department of Virology, School of Public Health, Tehran University of Medical Sciences	Department of Virology, School of Public Health, Tehran University of Medical Sciences	Salimi,V., Samieipoor,Y., Ghavami,N. and Mokhtariazad,T.
EPI_ISL_2573882, EPI_ISL_2573883, EPI_ISL_2573884	Department of Virology, Tehran University of Medical Sciences	Department of Virology, Tehran University of Medical Sciences	Salimi,V., Shatizadeh Malekshahi,S., Ghavami,N. and Mokhtari Azad,T.
EPI_ISL_2573901, EPI_ISL_2573902, EPI_ISL_2573903, EPI_ISL_2573904, EPI_ISL_2573905, EPI_ISL_2573906, EPI_ISL_2573907, EPI_ISL_2573908, EPI_ISL_2573909, EPI_ISL_2573910	Molecular Medicine Laboratory of Virology, Sapienza University	Molecular Medicine Laboratory of Virology, Sapienza University	Pierangeli,A.
EPI_ISL_2574130, EPI_ISL_2574131, EPI_ISL_2574135, EPI_ISL_2574138, EPI_ISL_2574139, EPI_ISL_2574149, EPI_ISL_2574151, EPI_ISL_2574152, EPI_ISL_2574179, EPI_ISL_2574182, EPI_ISL_2574185, EPI_ISL_2574193, EPI_ISL_2574225, EPI_ISL_2574226			
see above	University of Wuerzburg, Institute of Virology and Immunobiology	University of Wuerzburg, Institute of Virology and Immunobiology	Prifert,C., Hofmann,D. and Weissbrich,B.
EPI_ISL_2574315, EPI_ISL_2574316, EPI_ISL_2574317	Department of Virology, School of Public Health, Tehran University of Medical Sciences	Department of Virology, School of Public Health, Tehran University of Medical Sciences	Salimi,V., Samieipoor,Y., Ghavami,N. and Mokhtariazad,T.
EPI_ISL_2574318, EPI_ISL_2574319	Department of Virology, Tehran University of Medical Sciences	Department of Virology, Tehran University of Medical Sciences	Salimi,V., Shatizadeh Malekshahi,S., Ghavami,N. and Mokhtari Azad,T.
EPI_ISL_2574320	Department of Virology, School of Public Health, Tehran University of Medical Sciences	Department of Virology, School of Public Health, Tehran University of Medical Sciences	Salimi,V., Samieipoor,Y., Ghavami,N. and Mokhtariazad,T.
EPI_ISL_2574322	Department of Virology, Tehran University of Medical Sciences	Department of Virology, Tehran University of Medical Sciences	Salimi,V., Shatizadeh Malekshahi,S., Ghavami,N. and Mokhtari Azad,T.
EPI_ISL_2574323, EPI_ISL_2574324	Department of Virology, School of Public Health, Tehran University of Medical Sciences	Department of Virology, School of Public Health, Tehran University of Medical Sciences	Salimi,V., Samieipoor,Y., Ghavami,N. and Mokhtariazad,T.
EPI_ISL_2574325	Department of Virology, Tehran University of Medical Sciences	Department of Virology, Tehran University of Medical Sciences	Salimi,V., Shatizadeh Malekshahi,S., Ghavami,N. and Mokhtari Azad,T.
EPI_ISL_2574326	Department of Virology, School of Public Health, Tehran University of Medical Sciences	Department of Virology, School of Public Health, Tehran University of Medical Sciences	Salimi,V., Samieipoor,Y., Ghavami,N. and Mokhtariazad,T.
EPI_ISL_2574327	Department of Virology, Tehran University of Medical Sciences	Department of Virology, Tehran University of Medical Sciences	Salimi,V., Shatizadeh Malekshahi,S., Ghavami,N. and Mokhtari Azad,T.
EPI_ISL_2574328, EPI_ISL_2574329, EPI_ISL_2574330, EPI_ISL_2574331	Department of Virology, School of Public Health, Tehran University of Medical Sciences	Department of Virology, School of Public Health, Tehran University of Medical Sciences	Salimi,V., Samieipoor,Y., Ghavami,N. and Mokhtariazad,T.
EPI_ISL_2574332	Department of Virology, Tehran University of Medical Sciences	Department of Virology, Tehran University of Medical Sciences	Salimi,V., Shatizadeh Malekshahi,S., Ghavami,N. and Mokhtari Azad,T.
EPI_ISL_2574333	Department of Virology, School of Public Health, Tehran University of Medical Sciences	Department of Virology, School of Public Health, Tehran University of Medical Sciences	Salimi,V., Samieipoor,Y., Ghavami,N. and Mokhtariazad,T.
EPI_ISL_2574335	Department of Virology, Tehran University of Medical Sciences	Department of Virology, Tehran University of Medical Sciences	Salimi,V., Shatizadeh Malekshahi,S., Ghavami,N. and Mokhtari Azad,T.
EPI_ISL_2574336	Department of Virology, School of Public Health, Tehran University of Medical Sciences	Department of Virology, School of Public Health, Tehran University of Medical Sciences	Salimi,V., Samieipoor,Y., Ghavami,N. and Mokhtariazad,T.
EPI_ISL_2574337	Department of Virology, Tehran University of Medical Sciences	Department of Virology, Tehran University of Medical Sciences	Salimi,V., Shatizadeh Malekshahi,S., Ghavami,N. and Mokhtari Azad,T.
EPI_ISL_2574338	Department of Virology, School of Public Health, Tehran University of Medical Sciences	Department of Virology, School of Public Health, Tehran University of Medical Sciences	Salimi,V., Samieipoor,Y., Ghavami,N. and Mokhtariazad,T.
EPI_ISL_2574339, EPI_ISL_2574340	Department of Virology, Tehran University of Medical Sciences	Department of Virology, Tehran University of Medical Sciences	Salimi,V., Shatizadeh Malekshahi,S., Ghavami,N. and Mokhtari Azad,T.
EPI_ISL_2574341, EPI_ISL_2574342	Department of Virology, School of Public Health, Tehran University of Medical Sciences	Department of Virology, School of Public Health, Tehran University of Medical Sciences	Salimi,V., Samieipoor,Y., Ghavami,N. and Mokhtariazad,T.
EPI_ISL_2574343	Department of Virology, Tehran University of Medical Sciences	Department of Virology, Tehran University of Medical Sciences	Salimi,V., Shatizadeh Malekshahi,S., Ghavami,N. and Mokhtari Azad,T.
EPI_ISL_2574348	Department of Virology, School of Public Health, Tehran University of Medical Sciences	Department of Virology, School of Public Health, Tehran University of Medical Sciences	Salimi,V., Samieipoor,Y., Ghavami,N. and Mokhtariazad,T.
EPI_ISL_2574349, EPI_ISL_2574350, EPI_ISL_2574351	Department of Virology, Tehran University of Medical Sciences	Department of Virology, Tehran University of Medical Sciences	Salimi,V., Shatizadeh Malekshahi,S., Ghavami,N. and Mokhtari Azad,T.
EPI_ISL_2574369, EPI_ISL_2574370, EPI_ISL_2574371, EPI_ISL_2574372, EPI_ISL_2574374, EPI_ISL_2574375, EPI_ISL_2574377, EPI_ISL_2574378, EPI_ISL_2574379, EPI_ISL_2574380	Molecular Medicine Laboratory of Virology, Sapienza University	Molecular Medicine Laboratory of Virology, Sapienza University	Pierangeli,A.
EPI_ISL_2574515, EPI_ISL_2574516	Epidemiology and Demography, KEMRI-Wellcome Trust Research Programme	Epidemiology and Demography, KEMRI-Wellcome Trust Research Programme	Kamau,E.M., Agoti,C.N., Owor,B., Lewa,C., Bett,A., Oketch,J., Cane,P.A. and Nokes,J.D.

EPI_ISL_2574776, EPI_ISL_2574778, EPI_ISL_2574779, EPI_ISL_2574780, EPI_ISL_2574781, EPI_ISL_2574782, EPI_ISL_2574783, EPI_ISL_2574784, EPI_ISL_2574785, EPI_ISL_2574786, EPI_ISL_2574787, EPI_ISL_2574788, EPI_ISL_2574789, EPI_ISL_2574790, EPI_ISL_2574791, EPI_ISL_2574792, EPI_ISL_2574793, EPI_ISL_2574794, EPI_ISL_2574796, EPI_ISL_2574797, EPI_ISL_2574798, EPI_ISL_2574799, EPI_ISL_2574800, EPI_ISL_2574801, EPI_ISL_2574802, EPI_ISL_2574803, EPI_ISL_2574804, EPI_ISL_2574805, EPI_ISL_2574806, EPI_ISL_2574807			
see above	Medicine, American University of Beirut	Medicine, American University of Beirut	Abou-El-Hassan,H. and Zaraket,H.
EPI_ISL_2574819, EPI_ISL_2574820, EPI_ISL_2574821, EPI_ISL_2574822, EPI_ISL_2574823, EPI_ISL_2574824, EPI_ISL_2574825, EPI_ISL_2574826, EPI_ISL_2574827, EPI_ISL_2574828, EPI_ISL_2574829, EPI_ISL_2574830, EPI_ISL_2574831, EPI_ISL_2574832, EPI_ISL_2574833, EPI_ISL_2574834			
see above	Wint Wint Phyu Niigat University, International Health, Public Health	Wint Wint Phyu Niigat University, International Health, Public Health	Phyu,W.W. and Saito,R.
EPI_ISL_2574899, EPI_ISL_2574900, EPI_ISL_2574902, EPI_ISL_2574903, EPI_ISL_2574904, EPI_ISL_2574905, EPI_ISL_2574907, EPI_ISL_2574908, EPI_ISL_2574910, EPI_ISL_2574912, EPI_ISL_2574913, EPI_ISL_2574914, EPI_ISL_2574915, EPI_ISL_2574916, EPI_ISL_2574917, EPI_ISL_2574918, EPI_ISL_2574919, EPI_ISL_2574920, EPI_ISL_2574921, EPI_ISL_2574922, EPI_ISL_2574925, EPI_ISL_2574926, EPI_ISL_2574929, EPI_ISL_2574931, EPI_ISL_2574932, EPI_ISL_2574933, EPI_ISL_2574934			
see above	Department of Infectious Diseases, National Health Institute Doutor Ricardo Jorge	Department of Infectious Diseases, National Health Institute Doutor Ricardo Jorge	Saez-Lopez,E., Cristovao,P., Costa,I., Pechirra,P., Conde,P. and Guiomar,R.
EPI_ISL_2574940, EPI_ISL_2574941	Etiology and Epidemiology Department, Smorodintsev Research Institute of Influenza	Etiology and Epidemiology Department, Smorodintsev Research Institute of Influenza	Komissarova,K.S., Krivitskaya,V.Z., Sverlova,M.V., Petrova,E.R. and Ivanova,A.A.
EPI_ISL_2574944, EPI_ISL_2574945	Laboratory of molecular virology, Research Institute of Influenza, Ministry of Healthcare of the Russian Federation	Laboratory of molecular virology, Research Institute of Influenza, Ministry of Healthcare of the Russian Federation	Komissarova,K.S., Krivitskaya,V.Z., Sverlova,M.V., Petrova,E.R., Fadeev,A.V. and Komissarov,A.B.
EPI_ISL_2574946, EPI_ISL_2574947, EPI_ISL_2574948, EPI_ISL_2574949, EPI_ISL_2574951, EPI_ISL_2574952, EPI_ISL_2574953, EPI_ISL_2574957, EPI_ISL_2574958, EPI_ISL_2574963	Etiology and Epidemiology Department, Smorodintsev Research Institute of Influenza	Etiology and Epidemiology Department, Smorodintsev Research Institute of Influenza	Komissarova,K.S., Krivitskaya,V.Z., Sverlova,M.V., Petrova,E.R. and Ivanova,A.A.
EPI_ISL_2574965, EPI_ISL_2574966	Lab. of Molecular Virology, Research Institute of Influenza	Lab. of Molecular Virology, Research Institute of Influenza	Sintsova,K.S., Krivitskaya,V.Z., Sverlova,M.V., Petrova,E.R., Fadeev,A.V. and Komissarov,A.B.
EPI_ISL_2574968, EPI_ISL_2574969	Special Infectious Agents Unit, King Fahd Medical Research Center, King Abdulaziz University	Special Infectious Agents Unit, King Fahd Medical Research Center, King Abdulaziz University	El-Kafrawy,S.A., Alshareef,H. and Azhar,E.I.
EPI_ISL_2574971	Botany and Microbiology, College of Science, King Saud University, King Saud University	Botany and Microbiology, College of Science, King Saud University, King Saud University	Amer,H.M., Farrag,M.A., Hamad,M.E., Aziz,I.M. and Almajhdi,F.N.
EPI_ISL_2575021, EPI_ISL_2575022, EPI_ISL_2575023, EPI_ISL_2575024, EPI_ISL_2575025, EPI_ISL_2575026, EPI_ISL_2575027, EPI_ISL_2575028, EPI_ISL_2575029, EPI_ISL_2575030, EPI_ISL_2575031, EPI_ISL_2575032, EPI_ISL_2575033, EPI_ISL_2575034, EPI_ISL_2575035, EPI_ISL_2575036, EPI_ISL_2575037, EPI_ISL_2575038, EPI_ISL_2575039, EPI_ISL_2575040, EPI_ISL_2575041, EPI_ISL_2575042, EPI_ISL_2575043, EPI_ISL_2575044, EPI_ISL_2575045, EPI_ISL_2575046, EPI_ISL_2575047, EPI_ISL_2575048, EPI_ISL_2575049, EPI_ISL_2575050, EPI_ISL_2575051, EPI_ISL_2575052, EPI_ISL_2575053, EPI_ISL_2575054, EPI_ISL_2575055, EPI_ISL_2575056, EPI_ISL_2575057, EPI_ISL_2575058, EPI_ISL_2575059, EPI_ISL_2575060, EPI_ISL_2575061, EPI_ISL_2575062, EPI_ISL_2575063, EPI_ISL_2575064, EPI_ISL_2575065, EPI_ISL_2575066, EPI_ISL_2575067, EPI_ISL_2575068, EPI_ISL_2575069, EPI_ISL_2575070, EPI_ISL_2575071, EPI_ISL_2575072, EPI_ISL_2575073, EPI_ISL_2575074, EPI_ISL_2575075, EPI_ISL_2575076, EPI_ISL_2575077, EPI_ISL_2575078, EPI_ISL_2575079, EPI_ISL_2575080, EPI_ISL_2575081, EPI_ISL_2575082, EPI_ISL_2575083, EPI_ISL_2575084, EPI_ISL_2575085, EPI_ISL_2575086, EPI_ISL_2575087, EPI_ISL_2575088, EPI_ISL_2575089, EPI_ISL_2575090, EPI_ISL_2575091, EPI_ISL_2575092, EPI_ISL_2575101, EPI_ISL_2575102, EPI_ISL_2575103, EPI_ISL_2575104, EPI_ISL_2575105, EPI_ISL_2575106, EPI_ISL_2575107, EPI_ISL_2575108, EPI_ISL_2575109, EPI_ISL_2575110, EPI_ISL_2575111, EPI_ISL_2575112, EPI_ISL_2575113, EPI_ISL_2575114, EPI_ISL_2575115, EPI_ISL_2575116, EPI_ISL_2575117, EPI_ISL_2575118			
see above	Microbiology, Hospital Universitari Vall d'Hebron	Microbiology, Hospital Universitari Vall d'Hebron	Gimferrer,L., Martin,Md.C., Pumarola,T. and Anton,A.
EPI_ISL_2575181	Centre for Infectious Diseases Virology, University of Heidelberg	Centre for Infectious Diseases Virology, University of Heidelberg	Ihling,C.M., Schnitzler,P., Heinrich,N., Mangu,C., Sudi,L., Souares,A., Gies,S., Sie,A., Coulibaly,B., Ouedraogo,A.T., Mordmueller,B., Held,J., Adegnika,A.A., Fernandez,J.F., Eckerle,I., May,J., Hogan,B., Eibach,D., Owusu-Dabo,E. and Tabatabai,J.
EPI_ISL_2575186, EPI_ISL_2575187, EPI_ISL_2575188, EPI_ISL_2575189, EPI_ISL_2575190	Department of Pediatrics, Center of Excellence in Clinical Virology, Chulalongkorn	Department of Pediatrics, Center of Excellence in Clinical Virology, Chulalongkorn	Thongpan,I.
EPI_ISL_2575235, EPI_ISL_2575240, EPI_ISL_2575242, EPI_ISL_2575245, EPI_ISL_2575246	Epidemiology and Immunogenetic of Viral Infections LR14SP02, University Hospital Sahloul	Epidemiology and Immunogenetic of Viral Infections LR14SP02, University Hospital Sahloul	Ataoui,I., Jerbi,A., BenHamida-Rebai,M., Ben Hadj Fredj,M., Fodha,I., Bennour,H., Hammouda,H., Khlifa,M., Brini,I., Boussoffara,R., Boussetta,K., Abroug,S. and Trabelsi,A.
EPI_ISL_2578712	Pediatrics, University of New Mexico	Pediatrics, University of New Mexico	Kothari,A., Kennedy,J.L., Schwalm,K.C., Putt,C., Denson,J.L. and Dinwiddie,D.L.
EPI_ISL_2578715, EPI_ISL_2578716, EPI_ISL_2578717	Marie Bashir Institute for Infectious Diseases and Biosecurity & Sydney Medical School, The University of Sydney, Westmead Institute for Medical Research	Marie Bashir Institute for Infectious Diseases and Biosecurity & Sydney Medical School, The University of Sydney, Westmead Institute for Medical Research	Eden,J.-S., Kok,J., Dwyer,D.E., Fernandez,M., Carter,I. and Holmes,E.C.
EPI_ISL_2578734	Epidemiology and Demography Department, KEMRI-Wellcome Trust Research Programme	Epidemiology and Demography Department, KEMRI-Wellcome Trust Research Programme	Otieno,J.R., Kamau,E.M., Oketch,J.W., Ngoi,J.M., Agoti,C.N., Gichuki,A.M., Otieno,G.P., Ngama,M., Cane,P.A., Kellam,P., Cotten,M., Lemey,P. and Nokes,D.J.
EPI_ISL_2578760, EPI_ISL_2578761, EPI_ISL_2578762, EPI_ISL_2578763, EPI_ISL_2578764, EPI_ISL_2578765	Department of Virology, Tehran University of Medical Sciences	Department of Virology, Tehran University of Medical Sciences	Salimi,V., Shatizadeh Malekshahi,S., Ghavami,N. and Mokhtari Azad,T.
EPI_ISL_2578767, EPI_ISL_2578768	Marie Bashir Institute for Infectious Diseases and Biosecurity & Sydney Medical School, The University of Sydney, Westmead Institute for Medical Research	Marie Bashir Institute for Infectious Diseases and Biosecurity & Sydney Medical School, The University of Sydney, Westmead Institute for Medical Research	Eden,J.-S., Kok,J., Dwyer,D.E., Fernandez,M., Carter,I. and Holmes,E.C.
EPI_ISL_2578777, EPI_ISL_2578778	J. Craig Venter Institute	J. Craig Venter Institute	Tan,G., Pickett,B., Fedorova,N., Amedeo,P., Hu,L., Christensen,J., Miller,J., Durbin,A., Williams,T., Arumemi,F., Cadiz,C., Alanis,R., Balmseda,A., Wiliams,T., Schiller,A., Patel,M., Kubale,J. and Gordon,A.
EPI_ISL_2578788, EPI_ISL_2578789	Marie Bashir Institute for Infectious Diseases and Biosecurity & Sydney Medical School, The University of Sydney, Westmead Institute for Medical Research	Marie Bashir Institute for Infectious Diseases and Biosecurity & Sydney Medical School, The University of Sydney, Westmead Institute for Medical Research	Eden,J.-S., Kok,J., Dwyer,D.E., Fernandez,M., Carter,I. and Holmes,E.C.
EPI_ISL_2578803	Department of Pediatrics, Center of Excellence in Clinical Virology, Chulalongkorn	Department of Pediatrics, Center of Excellence in Clinical Virology, Chulalongkorn	Thongpan,I.
EPI_ISL_2578811, EPI_ISL_2578812, EPI_ISL_2578813	Marie Bashir Institute for Infectious Diseases and Biosecurity & Sydney Medical School, The University of Sydney, Westmead Institute for Medical Research	Marie Bashir Institute for Infectious Diseases and Biosecurity & Sydney Medical School, The University of Sydney, Westmead Institute for Medical Research	Eden,J.-S., Kok,J., Dwyer,D.E., Fernandez,M., Carter,I. and Holmes,E.C.
EPI_ISL_2578816	J. Craig Venter Institute	J. Craig Venter Institute	Tan,G., Pickett,B., Fedorova,N., Amedeo,P., Hu,L., Christensen,J., Miller,J., Durbin,A., Williams,T., Arumemi,F., Cadiz,C., Alanis,R., Balmseda,A., Wiliams,T., Schiller,A., Patel,M., Kubale,J. and Gordon,A.
EPI_ISL_2578825, EPI_ISL_2578826	Marie Bashir Institute for Infectious Diseases and Biosecurity & Sydney Medical School, The University of Sydney, Westmead Institute for Medical Research	Marie Bashir Institute for Infectious Diseases and Biosecurity & Sydney Medical School, The University of Sydney, Westmead Institute for Medical Research	Eden,J.-S., Kok,J., Dwyer,D.E., Fernandez,M., Carter,I. and Holmes,E.C.
EPI_ISL_2578827, EPI_ISL_2578828, EPI_ISL_2578829, EPI_ISL_2578830, EPI_ISL_2578831, EPI_ISL_2578832, EPI_ISL_2578833, EPI_ISL_2578834, EPI_ISL_2578835, EPI_ISL_2578836, EPI_ISL_2578837, EPI_ISL_2578838, EPI_ISL_2578846, EPI_ISL_2578847			
see above	College of Life Science and Technology, Jinan University	College of Life Science and Technology, Jinan University	Li,W., Wang,Y., Yu,B., Tan,Q., Zhou,J., Hu,J., Wu,Y., Wang,B. and Li,H.
EPI_ISL_2578911, EPI_ISL_2578914, EPI_ISL_2578915, EPI_ISL_2578916	Laboratory of molecular virology, Research Institute of Influenza, Ministry of Healthcare of the Russian Federation	Laboratory of molecular virology, Research Institute of Influenza, Ministry of Healthcare of the Russian Federation	Komissarova,K.S., Krivitskaya,V.Z., Sverlova,M.V., Petrova,E.R., Fadeev,A.V. and Komissarov,A.B.
EPI_ISL_2578917, EPI_ISL_2578919, EPI_ISL_2578920, EPI_ISL_2578922	Etiology and Epidemiology Department, Smorodintsev Research Institute of Influenza	Etiology and Epidemiology Department, Smorodintsev Research Institute of Influenza	Komissarova,K.S., Krivitskaya,V.Z., Sverlova,M.V., Petrova,E.R. and Ivanova,A.A.
EPI_ISL_2578924	Laboratory of molecular virology, Research Institute of Influenza, Ministry of Healthcare of the Russian Federation	Laboratory of molecular virology, Research Institute of Influenza, Ministry of Healthcare of the Russian Federation	Komissarova,K.S., Krivitskaya,V.Z., Sverlova,M.V., Petrova,E.R., Fadeev,A.V. and Komissarov,A.B.
EPI_ISL_2578925, EPI_ISL_2578926, EPI_ISL_2578927, EPI_ISL_2578928, EPI_ISL_2578929, EPI_ISL_2578930	Special Infectious Agents Unit, King Fahd Medical Research Center, King Abdulaziz University	Special Infectious Agents Unit, King Fahd Medical Research Center, King Abdulaziz University	El-Kafrawy,S.A., Alshareef,H. and Azhar,E.I.
EPI_ISL_2578967	Etiology and Epidemiology Department, Smorodintsev Research Institute of Influenza	Etiology and Epidemiology Department, Smorodintsev Research Institute of Influenza	Komissarova,K.S., Krivitskaya,V.Z., Sverlova,M.V., Petrova,E.R. and Ivanova,A.A.
EPI_ISL_2578971	Bacteriology & Virology, University of Medical Sciences, Shiraz Medical School	Bacteriology & Virology, University of Medical Sciences, Shiraz Medical School	Mojarrad,S., Pirbonyeh,N., Moattari,A. and Babaei,A.
EPI_ISL_2579014, EPI_ISL_2579015, EPI_ISL_2579016	Department of Virology, Tehran University of Medical Sciences	Department of Virology, Tehran University of Medical Sciences	Salimi,V., Shatizadeh Malekshahi,S., Ghavami,N. and Mokhtari Azad,T.
EPI_ISL_2579154	Botany and Microbiology, College of Science, King Saud University, King Saud University	Botany and Microbiology, College of Science, King Saud University, King Saud University	Amer,H.M., Farrag,M.A., Hamad,M.E., Aziz,I.M. and Almajhdi,F.N.
EPI_ISL_2579177, EPI_ISL_2579178	Pediatrics, Show-Chwan Memorial Hospital	Pediatrics, Show-Chwan Memorial Hospital	Lee,C.-Y.
EPI_ISL_2579261	Department of Virology, Tehran University of Medical Sciences	Department of Virology, Tehran University of Medical Sciences	Salimi,V., Shatizadeh Malekshahi,S., Ghavami,N. and Mokhtari Azad,T.
EPI_ISL_2579274, EPI_ISL_2579275, EPI_ISL_2579276, EPI_ISL_2579277, EPI_ISL_2579278, EPI_ISL_2579279, EPI_ISL_2579280, EPI_ISL_2579282, EPI_ISL_2579289, EPI_ISL_2579291, EPI_ISL_2579292			
see above	College of Life Science and Technology, Jinan University	College of Life Science and Technology, Jinan University	Li,W., Wang,Y., Yu,B., Tan,Q., Zhou,J., Hu,J., Wu,Y., Wang,B. and Li,H.
EPI_ISL_2579334, EPI_ISL_2579335, EPI_ISL_2579336	Laboratory of molecular virology, Research Institute of Influenza, Ministry of Healthcare of the Russian Federation	Laboratory of molecular virology, Research Institute of Influenza, Ministry of Healthcare of the Russian Federation	Komissarova,K.S., Krivitskaya,V.Z., Sverlova,M.V., Petrova,E.R., Fadeev,A.V. and Komissarov,A.B.
EPI_ISL_2579339, EPI_ISL_2579340	Etiology and Epidemiology Department, Smorodintsev Research Institute of Influenza	Etiology and Epidemiology Department, Smorodintsev Research Institute of Influenza	Komissarova,K.S., Krivitskaya,V.Z., Sverlova,M.V., Petrova,E.R. and Ivanova,A.A.
EPI_ISL_2579342, EPI_ISL_2579343, EPI_ISL_2579344, EPI_ISL_2579345, EPI_ISL_2579346, EPI_ISL_2579347, EPI_ISL_2579348, EPI_ISL_2579349, EPI_ISL_2579350	Special Infectious Agents Unit, King Fahd Medical Research Center, King Abdulaziz University	Special Infectious Agents Unit, King Fahd Medical Research Center, King Abdulaziz University	El-Kafrawy,S.A., Alshareef,H. and Azhar,E.I.
EPI_ISL_2579381, EPI_ISL_2579382	Etiology and Epidemiology Department, Smorodintsev Research Institute of Influenza	Etiology and Epidemiology Department, Smorodintsev Research Institute of Influenza	Komissarova,K.S., Krivitskaya,V.Z., Sverlova,M.V., Petrova,E.R. and Ivanova,A.A.

EPI_ISL_2579383	Lab. of Molecular Virology, Research Institute of Influenza	Lab. of Molecular Virology, Research Institute of Influenza	Sintsova,K.S., Krivitskaya,V.Z., Sverlova,M.V., Petrova,E.R., Fadeev,A.V. and Komissarov,A.B.
EPI_ISL_2579400, EPI_ISL_2579401, EPI_ISL_2579402, EPI_ISL_2579403, EPI_ISL_2579404, EPI_ISL_2579405, EPI_ISL_2579406, EPI_ISL_2579407, EPI_ISL_2579408, EPI_ISL_2579409	Molecular Medicine Laboratory of Virology, Sapienza University	Molecular Medicine Laboratory of Virology, Sapienza University	Pierangeli,A.
EPI_ISL_2581792, EPI_ISL_2581917	Bacteriology & Virology, University of Medical Sciences, Shiraz Medical School	Bacteriology & Virology, University of Medical Sciences, Shiraz Medical School	Mojarrad,S., Pirbonyeh,N., Moattari,A. and Babaei,A.
EPI_ISL_2582177	Pediatrics, University of New Mexico	Pediatrics, University of New Mexico	Kothari,A., Kennedy,J.L., Schwalm,K.C., Putt,C., Denson,J.L. and Dinwiddie,D.L.
EPI_ISL_2582221, EPI_ISL_2582230, EPI_ISL_2582231	J. Craig Venter Institute	J. Craig Venter Institute	Tan,G., Pickett,B., Fedorova,N., Amedeo,P., Hu,L., Christensen,J., Miller,J., Durbin,A., Williams,T., Arumemi,F., Cadiz,C., Alanis,R., Balmseda,A., Wiliams,T., Schiller,A., Patel,M., Kubale,J. and Gordon,A.
EPI_ISL_2582233	Laboratory Medicine, UW Virology	Laboratory Medicine, UW Virology	Lin,M.J., Tait,A. and Greninger,A.L.
EPI_ISL_2582235, EPI_ISL_2582238	J. Craig Venter Institute	J. Craig Venter Institute	Tan,G., Pickett,B., Fedorova,N., Amedeo,P., Hu,L., Christensen,J., Miller,J., Durbin,A., Williams,T., Arumemi,F., Cadiz,C., Alanis,R., Balmseda,A., Wiliams,T., Schiller,A., Patel,M., Kubale,J. and Gordon,A.
EPI_ISL_2582239, EPI_ISL_2582241, EPI_ISL_2582244	Marie Bashir Institute for Infectious Diseases and Biosecurity & Sydney Medical School, The University of Sydney, Westmead Institute for Medical Research	Marie Bashir Institute for Infectious Diseases and Biosecurity & Sydney Medical School, The University of Sydney, Westmead Institute for Medical Research	Eden,J.-S., Kok,J., Dwyer,D.E., Fernandez,M., Carter,I. and Holmes,E.C.
EPI_ISL_2582246	Pediatrics, University of New Mexico	Pediatrics, University of New Mexico	Kothari,A., Kennedy,J.L., Schwalm,K.C., Putt,C., Denson,J.L. and Dinwiddie,D.L.
EPI_ISL_2582250, EPI_ISL_2582251, EPI_ISL_2582253	Marie Bashir Institute for Infectious Diseases and Biosecurity & Sydney Medical School, The University of Sydney, Westmead Institute for Medical Research	Marie Bashir Institute for Infectious Diseases and Biosecurity & Sydney Medical School, The University of Sydney, Westmead Institute for Medical Research	Eden,J.-S., Kok,J., Dwyer,D.E., Fernandez,M., Carter,I. and Holmes,E.C.
EPI_ISL_2582261	Pediatrics, University of New Mexico	Pediatrics, University of New Mexico	Kothari,A., Kennedy,J.L., Schwalm,K.C., Putt,C., Denson,J.L. and Dinwiddie,D.L.
EPI_ISL_2582264, EPI_ISL_2582265	Marie Bashir Institute for Infectious Diseases and Biosecurity & Sydney Medical School, The University of Sydney, Westmead Institute for Medical Research	Marie Bashir Institute for Infectious Diseases and Biosecurity & Sydney Medical School, The University of Sydney, Westmead Institute for Medical Research	Eden,J.-S., Kok,J., Dwyer,D.E., Fernandez,M., Carter,I. and Holmes,E.C.
EPI_ISL_2582271	J. Craig Venter Institute	J. Craig Venter Institute	Tan,G., Pickett,B., Fedorova,N., Amedeo,P., Hu,L., Christensen,J., Miller,J., Durbin,A., Williams,T., Arumemi,F., Cadiz,C., Alanis,R., Balmseda,A., Wiliams,T., Schiller,A., Patel,M., Kubale,J. and Gordon,A.
EPI_ISL_2582641, EPI_ISL_2582644	Marie Bashir Institute for Infectious Diseases and Biosecurity & Sydney Medical School, The University of Sydney, Westmead Institute for Medical Research	Marie Bashir Institute for Infectious Diseases and Biosecurity & Sydney Medical School, The University of Sydney, Westmead Institute for Medical Research	Eden,J.-S., Kok,J., Dwyer,D.E., Fernandez,M., Carter,I. and Holmes,E.C.
EPI_ISL_2582648, EPI_ISL_2582650, EPI_ISL_2582653	Pediatrics, University of New Mexico	Pediatrics, University of New Mexico	Kothari,A., Kennedy,J.L., Schwalm,K.C., Putt,C., Denson,J.L. and Dinwiddie,D.L.
EPI_ISL_2582678	Epidemiology and Demography Department, KEMRI-Wellcome Trust Research Programme	Epidemiology and Demography Department, KEMRI-Wellcome Trust Research Programme	Otieno,J.R., Kamau,E.M., Oketch,J.W., Ngoi,J.M., Agoti,C.N., Gichuki,A.M., Otieno,G.P., Ngama,M., Cane,P.A., Kellam,P., Cotten,M., Lemey,P. and Nokes,D.J.
EPI_ISL_2582771, EPI_ISL_2582841	Marie Bashir Institute for Infectious Diseases and Biosecurity & Sydney Medical School, The University of Sydney, Westmead Institute for Medical Research	Marie Bashir Institute for Infectious Diseases and Biosecurity & Sydney Medical School, The University of Sydney, Westmead Institute for Medical Research	Eden,J.-S., Kok,J., Dwyer,D.E., Fernandez,M., Carter,I. and Holmes,E.C.
EPI_ISL_2582864	Gansu Center for Disease Control and Prevention, Pathogen Laboratory	Gansu Center for Disease Control and Prevention, Pathogen Laboratory	Qiao,R., Chen,J., Wu,H. and Yu,D.
EPI_ISL_2582906	Department of Pediatrics, Center of Excellence in Clinical Virology, Chulalongkorn	Department of Pediatrics, Center of Excellence in Clinical Virology, Chulalongkorn	Thongpan,I.
EPI_ISL_2583033	Epidemiology and Demography Department, KEMRI-Wellcome Trust Research Programme	Epidemiology and Demography Department, KEMRI-Wellcome Trust Research Programme	Otieno,J.R., Kamau,E.M., Oketch,J.W., Ngoi,J.M., Agoti,C.N., Gichuki,A.M., Otieno,G.P., Ngama,M., Cane,P.A., Kellam,P., Cotten,M., Lemey,P. and Nokes,D.J.
EPI_ISL_2583038, EPI_ISL_2583042, EPI_ISL_2583061, EPI_ISL_2583062, EPI_ISL_2583063	Pediatrics, University of New Mexico	Pediatrics, University of New Mexico	Kothari,A., Kennedy,J.L., Schwalm,K.C., Putt,C., Denson,J.L. and Dinwiddie,D.L.
EPI_ISL_2583067	Molecular Virology, Blood Systems Research Institute	Molecular Virology, Blood Systems Research Institute	Altan,E. and Delwart,E.
EPI_ISL_2583070	Epidemiology and Demography Department, KEMRI-Wellcome Trust Research Programme	Epidemiology and Demography Department, KEMRI-Wellcome Trust Research Programme	Otieno,J.R., Kamau,E.M., Oketch,J.W., Ngoi,J.M., Agoti,C.N., Gichuki,A.M., Otieno,G.P., Ngama,M., Cane,P.A., Kellam,P., Cotten,M., Lemey,P. and Nokes,D.J.
EPI_ISL_2583076	Pediatrics, University of New Mexico	Pediatrics, University of New Mexico	Kothari,A., Kennedy,J.L., Schwalm,K.C., Putt,C., Denson,J.L. and Dinwiddie,D.L.
EPI_ISL_2583089, EPI_ISL_2583091	Epidemiology and Demography Department, KEMRI-Wellcome Trust Research Programme	Epidemiology and Demography Department, KEMRI-Wellcome Trust Research Programme	Otieno,J.R., Kamau,E.M., Oketch,J.W., Ngoi,J.M., Agoti,C.N., Gichuki,A.M., Otieno,G.P., Ngama,M., Cane,P.A., Kellam,P., Cotten,M., Lemey,P. and Nokes,D.J.
EPI_ISL_2587497, EPI_ISL_2587499, EPI_ISL_2587501	Environmental and Global Health, University of Florida	Environmental and Global Health, University of Florida	Lednicky,J.A., Loeb,J.C., Bonny,T.S., Pan,M., Jiang,X., Eiguren-Fernandez,A., Hering,S.V., Fan,Z.H. and Wu,C.-Y.
EPI_ISL_2588026, EPI_ISL_2588027, EPI_ISL_2588028, EPI_ISL_2588190, EPI_ISL_2588197	Centre for Infectious Disease Research, Diagnostics and Screening (IDS) Department of Endemic and Emerging Viruses (EEV), RIVM	Centre for Infectious Disease Research, Diagnostics and Screening (IDS) Department of Endemic and Emerging Viruses (EEV), RIVM	Janssens,T.K.S., Zhan,S.H., Overduin,P., Alamouti,S.M., Kwok,B.S., Schmitz,D., Qadir,M.A. and Meijer,A.
EPI_ISL_2588211, EPI_ISL_2588212	Miwako SaikNorth America / USA Yokohama City Institute of Public Health	Miwako SaikNorth America / USA Yokohama City Institute of Public Health	SaikNorth America / USA,M., Kawakami,C., Usuku,S., Sasao,T. and Okubo,I.
EPI_ISL_2588222	Centre for Infectious Disease Research, Diagnostics and Screening (IDS) Department of Endemic and Emerging Viruses (EEV), RIVM	Centre for Infectious Disease Research, Diagnostics and Screening (IDS) Department of Endemic and Emerging Viruses (EEV), RIVM	Janssens,T.K.S., Zhan,S.H., Overduin,P., Alamouti,S.M., Kwok,B.S., Schmitz,D., Qadir,M.A. and Meijer,A.
EPI_ISL_2588338, EPI_ISL_2588339, EPI_ISL_2588340, EPI_ISL_2588341, EPI_ISL_2588342, EPI_ISL_2588343	Miwako SaikNorth America / USA Yokohama City Institute of Public Health	Miwako SaikNorth America / USA Yokohama City Institute of Public Health	SaikNorth America / USA,M., Kawakami,C., Usuku,S., Sasao,T. and Okubo,I.
EPI_ISL_2588344	Centre for Infectious Disease Research, Diagnostics and Screening (IDS) Department of Endemic and Emerging Viruses (EEV), RIVM	Centre for Infectious Disease Research, Diagnostics and Screening (IDS) Department of Endemic and Emerging Viruses (EEV), RIVM	Janssens,T.K.S., Zhan,S.H., Overduin,P., Alamouti,S.M., Kwok,B.S., Schmitz,D., Qadir,M.A. and Meijer,A.
EPI_ISL_2588421, EPI_ISL_2588422, EPI_ISL_2588423, EPI_ISL_2588424, EPI_ISL_2588425, EPI_ISL_2588426, EPI_ISL_2588427, EPI_ISL_2588428, EPI_ISL_2588429, EPI_ISL_2588430, EPI_ISL_2588431, EPI_ISL_2588432, EPI_ISL_2588433, EPI_ISL_2588434, EPI_ISL_2588435			
see above	Miwako SaikNorth America / USA Yokohama City Institute of Public Health	Miwako SaikNorth America / USA Yokohama City Institute of Public Health	SaikNorth America / USA,M., Kawakami,C., Usuku,S., Sasao,T. and Okubo,I.
EPI_ISL_2588443, EPI_ISL_2588444, EPI_ISL_2588445, EPI_ISL_2588446	Centre for Infectious Disease Research, Diagnostics and Screening (IDS) Department of Endemic and Emerging Viruses (EEV), RIVM	Centre for Infectious Disease Research, Diagnostics and Screening (IDS) Department of Endemic and Emerging Viruses (EEV), RIVM	Janssens,T.K.S., Zhan,S.H., Overduin,P., Alamouti,S.M., Kwok,B.S., Schmitz,D., Qadir,M.A. and Meijer,A.
EPI_ISL_2588794	Virology Laboratory, Dr. Ricardo Gutierrez Children Hospital	Virology Laboratory, Dr. Ricardo Gutierrez Children Hospital	Goya,S., Rojo,G.L., Valinotto,L.E., Mistchenko,A.S. and Viegas,M.
EPI_ISL_2588795	Virology Laboratory, Dr. Ricardo Gutierrez Children Hospital	Virology Laboratory, Dr. Ricardo Gutierrez Children Hospital	Goya,S., Valinotto,L.E., Tittarelli,E., Rojo,G.L., Greninger,A., Zaiat,J., Marti,M., Mistchenko,A.S. and Viegas,M.
EPI_ISL_2588801, EPI_ISL_2588802, EPI_ISL_2588803, EPI_ISL_2588804, EPI_ISL_2588805, EPI_ISL_2588806, EPI_ISL_2588807, EPI_ISL_2588808, EPI_ISL_2588809, EPI_ISL_2588810, EPI_ISL_2588811, EPI_ISL_2588812, EPI_ISL_2588814, EPI_ISL_2588815, EPI_ISL_2588821			
see above	Manipal Institute of Virology (MIV), Manipal Academy of Higher Education (MAHE)	Manipal Institute of Virology (MIV), Manipal Academy of Higher Education (MAHE)	Suresh,B.S., Santhosha,D., Rushil,B., Preetiparna,P., Mevis,F., Ameen,A. and Arunkumar,G.
EPI_ISL_2588827	Virology Laboratory, Dr. Ricardo Gutierrez Children Hospital	Virology Laboratory, Dr. Ricardo Gutierrez Children Hospital	Goya,S., Valinotto,L.E., Tittarelli,E., Rojo,G.L., Greninger,A., Zaiat,J., Marti,M., Mistchenko,A.S. and Viegas,M.
EPI_ISL_2591046, EPI_ISL_2591085, EPI_ISL_2591191, EPI_ISL_2591193, EPI_ISL_2591195, EPI_ISL_2591491, EPI_ISL_2591493, EPI_ISL_2591495, EPI_ISL_2591497	Virology, National Centre of Infectious and Parasitic Diseases	Virology, National Centre of Infectious and Parasitic Diseases	Korsun,N.S. and Angelova,S.G.
EPI_ISL_2591506	A' Dept. of Microbiology, Aristotle University of Thessaloniki, Medical School, Thessaloniki	A' Dept. of Microbiology, Aristotle University of Thessaloniki, Medical School, Thessaloniki	Papa,A., Pappa,S., Tsergouli,K. and Haidopoulou,K.
EPI_ISL_2591747, EPI_ISL_2591748, EPI_ISL_2591749, EPI_ISL_2591750, EPI_ISL_2591751	Virology, National Centre of Infectious and Parasitic Diseases	Virology, National Centre of Infectious and Parasitic Diseases	Korsun,N.S. and Angelova,S.G.
EPI_ISL_2591752, EPI_ISL_2591753, EPI_ISL_2591754	A' Dept. of Microbiology, Aristotle University of Thessaloniki, Medical School, Thessaloniki	A' Dept. of Microbiology, Aristotle University of Thessaloniki, Medical School, Thessaloniki	Papa,A., Pappa,S., Tsergouli,K. and Haidopoulou,K.
EPI_ISL_2591984	Miwako SaikNorth America / USA Yokohama City Institute of Public Health	Miwako SaikNorth America / USA Yokohama City Institute of Public Health	SaikNorth America / USA,M., Kawakami,C., Usuku,S., Sasao,T. and Okubo,I.
EPI_ISL_2592087, EPI_ISL_2592088, EPI_ISL_2592089	Virology, National Centre of Infectious and Parasitic Diseases	Virology, National Centre of Infectious and Parasitic Diseases	Korsun,N.S. and Angelova,S.G.
EPI_ISL_2592369, EPI_ISL_2592370, EPI_ISL_2592371, EPI_ISL_2592372, EPI_ISL_2592373, EPI_ISL_2592374, EPI_ISL_2592375, EPI_ISL_2592376, EPI_ISL_2592377	A' Dept. of Microbiology, Aristotle University of Thessaloniki, Medical School, Thessaloniki	A' Dept. of Microbiology, Aristotle University of Thessaloniki, Medical School, Thessaloniki	Papa,A., Pappa,S., Tsergouli,K. and Haidopoulou,K.
EPI_ISL_2592378	Manipal Institute of Virology (MIV), Manipal Academy of Higher Education (MAHE)	Manipal Institute of Virology (MIV), Manipal Academy of Higher Education (MAHE)	Suresh,B.S., Santhosha,D., Rushil,B., Preetiparna,P., Mevis,F., Ameen,A. and Arunkumar,G.
EPI_ISL_2595695	Bacteriology & Virology, University of Medical Sciences, Shiraz Medical School	Bacteriology & Virology, University of Medical Sciences, Shiraz Medical School	Mojarrad,S., Pirbonyeh,N., Moattari,A. and Babaei,A.
EPI_ISL_2595696	Pediatrics, University of New Mexico	Pediatrics, University of New Mexico	Kothari,A., Kennedy,J.L., Schwalm,K.C., Putt,C., Denson,J.L. and Dinwiddie,D.L.

EPI_ISL_2595697	Epidemiology and Demography Department, KEMRI-Wellcome Trust Research Programme	Epidemiology and Demography Department, KEMRI-Wellcome Trust Research Programme	Otieno,J.R., Kamau,E.M., Oketch,J.W., Ngoi,J.M., Agoti,C.N., Gichuki,A.M., Otieno,G.P., Ngama,M., Cane,P.A., Kellam,P., Cotten,M., Lemey,P. and Nokes,D.J.
EPI_ISL_2595698	Botany and Microbiology, College of Science, King Saud University, King Saud University	Botany and Microbiology, College of Science, King Saud University, King Saud University	Amer,H.M., Farrag,M.A., Hamad,M.E., Aziz,I.M. and Almajhdi,F.N.
EPI_ISL_2595699, EPI_ISL_2595700, EPI_ISL_2595701	Pediatrics, University of New Mexico	Pediatrics, University of New Mexico	Kothari,A., Kennedy,J.L., Schwalm,K.C., Putt,C., Denson,J.L. and Dinwiddie,D.L.
EPI_ISL_2595702	Epidemiology and Demography Department, KEMRI-Wellcome Trust Research Programme	Epidemiology and Demography Department, KEMRI-Wellcome Trust Research Programme	Otieno,J.R., Kamau,E.M., Oketch,J.W., Ngoi,J.M., Agoti,C.N., Gichuki,A.M., Otieno,G.P., Ngama,M., Cane,P.A., Kellam,P., Cotten,M., Lemey,P. and Nokes,D.J.
EPI_ISL_2595703, EPI_ISL_2595704, EPI_ISL_2595705	Pediatrics, University of New Mexico	Pediatrics, University of New Mexico	Kothari,A., Kennedy,J.L., Schwalm,K.C., Putt,C., Denson,J.L. and Dinwiddie,D.L.
EPI_ISL_2595706, EPI_ISL_2595707, EPI_ISL_2595708, EPI_ISL_2595709, EPI_ISL_2595710, EPI_ISL_2595711, EPI_ISL_2595712	Marie Bashir Institute for Infectious Diseases and Biosecurity & Sydney Medical School, The University of Sydney, Westmead Institute for Medical Research	Marie Bashir Institute for Infectious Diseases and Biosecurity & Sydney Medical School, The University of Sydney, Westmead Institute for Medical Research	Eden,J.-S., Kok,J., Dwyer,D.E., Fernandez,M., Carter,I. and Holmes,E.C.
EPI_ISL_2595713	Department of Pediatrics, Center of Excellence in Clinical Virology, Chulalongkorn	Department of Pediatrics, Center of Excellence in Clinical Virology, Chulalongkorn	Thongpan,I.
EPI_ISL_2595714, EPI_ISL_2595715, EPI_ISL_2595716	Marie Bashir Institute for Infectious Diseases and Biosecurity & Sydney Medical School, The University of Sydney, Westmead Institute for Medical Research	Marie Bashir Institute for Infectious Diseases and Biosecurity & Sydney Medical School, The University of Sydney, Westmead Institute for Medical Research	Eden,J.-S., Kok,J., Dwyer,D.E., Fernandez,M., Carter,I. and Holmes,E.C.
EPI_ISL_2595717	J. Craig Venter Institute	J. Craig Venter Institute	Tan,G., Pickett,B., Fedorova,N., Amedeo,P., Hu,L., Christensen,J., Miller,J., Durbin,A., Williams,T., Arumemi,F., Cadiz,C., Alanis,R., Balmseda,A., Wiliams,T., Schiller,A., Patel,M., Kubale,J. and Gordon,A.
EPI_ISL_2595718, EPI_ISL_2595719	Marie Bashir Institute for Infectious Diseases and Biosecurity & Sydney Medical School, The University of Sydney, Westmead Institute for Medical Research	Marie Bashir Institute for Infectious Diseases and Biosecurity & Sydney Medical School, The University of Sydney, Westmead Institute for Medical Research	Eden,J.-S., Kok,J., Dwyer,D.E., Fernandez,M., Carter,I. and Holmes,E.C.
EPI_ISL_2595720, EPI_ISL_2595721	Special Infectious Agents Unit, King Fahd Medical Research Center, King Abdulaziz University	Special Infectious Agents Unit, King Fahd Medical Research Center, King Abdulaziz University	El-Kafrawy,S.A., Alshareef,H. and Azhar,E.I.
EPI_ISL_2595722	Department of Pediatrics, Center of Excellence in Clinical Virology, Chulalongkorn	Department of Pediatrics, Center of Excellence in Clinical Virology, Chulalongkorn	Thongpan,I.
EPI_ISL_2595723	Gansu Center for Disease Control and Prevention, Pathogen Laboratory	Gansu Center for Disease Control and Prevention, Pathogen Laboratory	Qiao,R., Chen,J., Wu,H. and Yu,D.
EPI_ISL_2595724	Laboratory Medicine, UW Virology	Laboratory Medicine, UW Virology	Lin,M.J., Tait,A. and Greninger,A.L.
EPI_ISL_412458	Centro de Salud Sócrates Flores Vivas	University of Edinburgh	Gordon, A., Alanis,R., Balmseda,A., Schiller,A., Patel,M., Kubale,J., Tan,G., Pickett,B., Fedorova,N., Amedeo,P., Hu,L., Christensen,J., Miller,J., Durbin,A., Williams,T., Arumemi,F., Cadiz,C., Williams, T.
EPI_ISL_412643	Virology Laboratory Ricardo Gutiérrez Children's Hospital	Virology Laboratory Ricardo Gutiérrez Children's Hospital	Goya, Stephanie.; Nabaes Jodar, Mercedes S.; Valinotto, Laura E.; Rojo, Gabriel L.; Zaiat, Jonathan; Marti, Marcelo A.; Mistchenko, Alicia S.; Viegas, M.
EPI_ISL_412857	Virology Laboratory, Ricardo Gutiérrez Children's Hospital	Virology Laboratory, Ricardo Gutiérrez Children's Hospital / Vanderbilt University Medical Center	Goya, Stephanie; Lucion, Maria Florencia; Juarez, Maria del Valle; Shilts, Meghan; Gentile, Angela; Mistchenko, Alicia S.; Das, Suman & Viegas, Mariana
EPI_ISL_412859	WHO National Influenza Centre Russian Federation	WHO National Influenza Centre Russian Federation	Komissarova K.,Fadeev A., Komissarov A., Krivitskaya V.
EPI_ISL_412865	Respiratory Virus Unit, Microbiology Services Colindale, Public Health England	Microbiology Services Colindale, Public Health England	Zambon M
EPI_ISL_412866	Respiratory Virus Unit, Microbiology Services Colindale, Public Health England	Microbiology Services Colindale, Public Health England	Zambon M.
EPI_ISL_412874	National Reference Laboratory "Influenza and ARD"	National Reference Laboratory "Influenza and ARD"	Korsun N, Angelova S, Trifonova I
EPI_ISL_412911	Institut Pasteur du Cambodge	University of Edinburgh	Karlsson, E., Kim L., Viseth S.,Horwood P, Dussart P., Tan,G., Pickett,B., Fedorova,N., Amedeo,P., Hu,L., Christensen,J., Miller,J., Durbin,A., Williams,T.
EPI_ISL_413353	Institut Pasteur de Madagascar	Institut Pasteur de Madagascar	Jean-Michel HERAUD
EPI_ISL_413644, EPI_ISL_413645, EPI_ISL_413646, EPI_ISL_413917	Bahrami Children Hospital, Tehran University of Medical Sciences	Virology Department, School of Public Health, Tehran University of Medical Sciences	Vahid Salimi, Somayeh Shatizadeh Malekshahi, Talat Mokhtari-Azad
EPI_ISL_413918	Bahrami Children Hospital, Tehran University of Medical Sciences	Virology Department, School of Public Health, Tehran University of Medical Sciences,	Vahid Salimi, Somayeh Shatizadeh Malekshahi, Talat Mokhtari-Azad
EPI_ISL_413919, EPI_ISL_413920, EPI_ISL_413921, EPI_ISL_413923, EPI_ISL_413929, EPI_ISL_413932, EPI_ISL_413933	Bahrami Children Hospital, Tehran University of Medical Sciences	Virology Department, School of Public Health, Tehran University of Medical Sciences	Vahid Salimi, Somayeh Shatizadeh Malekshahi, Talat Mokhtari-Azad
EPI_ISL_413934	WHO National Influenza Centre Russian Federation	WHO National Influenza Centre Russian Federation	Komissarova K.,Fadeev A., Komissarov A., Krivitskaya V
EPI_ISL_413935, EPI_ISL_413936	WHO National Influenza Centre Russian Federation	WHO National Influenza Centre Russian Federation	Komissarova K.,Fadeev A., Komissarov A., Krivitskaya V.
EPI_ISL_413937	Bahrami Children Hospital, Tehran University of Medical Sciences	Virology Department, School of Public Health, Tehran University of Medical Sciences	Vahid Salimi, Somayeh Shatizadeh Malekshahi, Talat Mokhtari-Azad
EPI_ISL_413938, EPI_ISL_413939, EPI_ISL_413940, EPI_ISL_413941, EPI_ISL_413942, EPI_ISL_413943, EPI_ISL_413944, EPI_ISL_413945, EPI_ISL_413946, EPI_ISL_413947, EPI_ISL_413948, EPI_ISL_413950, EPI_ISL_413951, EPI_ISL_413952, EPI_ISL_413953, EPI_ISL_413954			
see above	WHO National Influenza Centre Russian Federation	WHO National Influenza Centre Russian Federation	Komissarova K.,Fadeev A., Komissarov A., Krivitskaya V.
EPI_ISL_413985, EPI_ISL_413986, EPI_ISL_413987, EPI_ISL_413988, EPI_ISL_413989	Bahrami Children Hospital, Tehran University of Medical Sciences	Virology Department, School of Public Health, Tehran University of Medical Sciences	Vahid Salimi, Somayeh Shatizadeh Malekshahi, Talat Mokhtari-Azad
EPI_ISL_413990	Bahrami Children Hospital, Tehran University of Medical Sciences	Virology Department, School of Public Health, Tehran University of Medical Sciences, Tehran, Iran	Vahid Salimi, Somayeh Shatizadeh Malekshahi, Talat Mokhtari-Azad
EPI_ISL_413991	Bahrami Children Hospital, Tehran University of Medical Sciences	Virology Department, School of Public Health, Tehran University of Medical Sciences	Vahid Salimi, Somayeh Shatizadeh Malekshahi, Talat Mokhtari-Azad
EPI_ISL_413992	Bahrami Children Hospital, Tehran University of Medical Sciences,	Virology Department, School of Public Health, Tehran University of Medical Sciences	Vahid Salimi, Somayeh Shatizadeh Malekshahi, Talat Mokhtari-Azad
EPI_ISL_413993	Bahrami Children Hospital, Tehran University of Medical Sciences	Virology Department, School of Public Health, Tehran University of Medical Sciences, Tehran, Iran	Vahid Salimi, Somayeh Shatizadeh Malekshahi, Talat Mokhtari-Azad
EPI_ISL_413994	Bahrami Children Hospital, Tehran University of Medical Sciences	Virology Department, School of Public Health, Tehran University of Medical Sciences	Vahid Salimi, Somayeh Shatizadeh Malekshahi, Talat Mokhtari-Azad
EPI_ISL_413995	Bahrami Children Hospital, Tehran University of Medical Sciences	Virology Department, School of Public Health, Tehran University of Medical Sciences, Tehran, Iran	Vahid Salimi, Somayeh Shatizadeh Malekshahi, Talat Mokhtari-Azad
EPI_ISL_413998, EPI_ISL_414000, EPI_ISL_414001, EPI_ISL_414002, EPI_ISL_414003	WHO National Influenza Centre Russian Federation	WHO National Influenza Centre Russian Federation	Komissarova K.,Fadeev A., Komissarov A., Krivitskaya V.
EPI_ISL_4602779	VIC, Victorian Infectious Diseases Reference Laboratory	WHO Collaborating Centre for Reference and Research on Influenza	Xiaomin Dong, Annette Alafaci,Yi-Mo Deng, Ammar Aziz, Naomi Komadina
EPI_ISL_549271	LACEN/ES	Laboratory of Respiratory Viruses and Measles, Oswaldo Cruz Institute, FIOCRUZ	Lucas Vianna, Lays Volpini, Marilda Siqueira, Iuri Louro, Paola Resende
EPI_ISL_549272, EPI_ISL_549273, EPI_ISL_549274	Laboratorio Central de Saude Publica do Estado do Espirito Santo (LACEN/ES)	Laboratory of Respiratory Viruses and Measles, Oswaldo Cruz Institute, FIOCRUZ	Lucas Vianna, Lays Volpini, Marilda Siqueira, Iuri Louro, Paola Resende
EPI_ISL_549275	LACEN/ES	Laboratory of Respiratory Viruses and Measles, Oswaldo Cruz Institute, FIOCRUZ	Lucas Vianna, Lays Volpini, Marilda Siqueira, Iuri Louro, Paola Resende
EPI_ISL_549276, EPI_ISL_549277, EPI_ISL_549278, EPI_ISL_549279	Laboratorio Central de Saude Publica do Estado do Espirito Santo (LACEN/ES)	Laboratory of Respiratory Viruses and Measles, Oswaldo Cruz Institute, FIOCRUZ	Lucas Vianna, Lays Volpini, Marilda Siqueira, Iuri Louro, Paola Resende
EPI_ISL_549280	LACEN/ES	Laboratory of Respiratory Viruses and Measles, Oswaldo Cruz Institute, FIOCRUZ	Lucas Vianna, Lays Volpini, Marilda Siqueira, Iuri Louro, Paola Resende
EPI_ISL_549281, EPI_ISL_549282, EPI_ISL_549283, EPI_ISL_549284, EPI_ISL_549285, EPI_ISL_549286, EPI_ISL_549287, EPI_ISL_549288, EPI_ISL_549289, EPI_ISL_549290, EPI_ISL_549291, EPI_ISL_549292, EPI_ISL_549293			
see above	Laboratorio Central de Saude Publica do Estado do Espirito Santo (LACEN/ES)	Laboratory of Respiratory Viruses and Measles, Oswaldo Cruz Institute, FIOCRUZ	Lucas Vianna, Lays Volpini, Marilda Siqueira, Iuri Louro, Paola Resende
EPI_ISL_549294	LACEN/ES	Laboratory of Respiratory Viruses and Measles, Oswaldo Cruz Institute, FIOCRUZ	Lucas Vianna, Lays Volpini, Marilda Siqueira, Iuri Louro, Paola Resende
EPI_ISL_549295, EPI_ISL_549296, EPI_ISL_549297, EPI_ISL_549298	Laboratorio Central de Saude Publica do Estado do Espirito Santo (LACEN/ES)	Laboratory of Respiratory Viruses and Measles, Oswaldo Cruz Institute, FIOCRUZ	Lucas Vianna, Lays Volpini, Marilda Siqueira, Iuri Louro, Paola Resende
EPI_ISL_549299	LACEN/ES	Laboratory of Respiratory Viruses and Measles, Oswaldo Cruz Institute, FIOCRUZ	Lucas Vianna, Lays Volpini, Marilda Siqueira, Iuri Louro, Paola Resende

EPI_ISL_549300, EPI_ISL_549301, EPI_ISL_549302, EPI_ISL_549303, EPI_ISL_549304, EPI_ISL_549305, EPI_ISL_549306	Laboratorio Central de Saude Publica do Estado do Espirito Santo (LACEN/ES)	Laboratory of Respiratory Viruses and Measles, Oswaldo Cruz Institute, FIOCRUZ	Lucas Vianna, Lays Volpini, Marilda Siqueira, Iuri Louro, Paola Resende
EPI_ISL_5930150	Institut Pasteur de Cote d'ivoire	WHO Collaborating Centre for Reference and Research on Influenza	Xiaomin Dong, Herve Kadjo, Yi-Mo Deng, Ammar Aziz, Naomi Komadina
EPI_ISL_6208710, EPI_ISL_6208712, EPI_ISL_6208715, EPI_ISL_6208716, EPI_ISL_6208718	Egypt, Central Public Health Laboratory (CPHL)	WHO Collaborating Centre for Reference and Research on Influenza	Xiaomin Dong,Yi-Mo Deng, Amel Naguib, Naomi Komadina
EPI_ISL_6494811, EPI_ISL_6494815, EPI_ISL_6494816, EPI_ISL_6494818, EPI_ISL_6494819, EPI_ISL_6494820, EPI_ISL_6494821, EPI_ISL_6494822, EPI_ISL_6494823, EPI_ISL_6494824, EPI_ISL_732337, EPI_ISL_732338, EPI_ISL_732339, EPI_ISL_732340, EPI_ISL_732341, EPI_ISL_732342, EPI_ISL_732344, EPI_ISL_732345, EPI_ISL_732346, EPI_ISL_732347, EPI_ISL_732348, EPI_ISL_732357, EPI_ISL_732359, EPI_ISL_732360, EPI_ISL_732361, EPI_ISL_732368, EPI_ISL_732369, EPI_ISL_732370, EPI_ISL_732372			
see above	Respiratory Virus Unit, National Infection Service, Public Health England	National Infection Service, Public Health England	Zambon M, Talts T, Ellis J, Miah S, Platt S
EPI_ISL_9665061, EPI_ISL_9665287, EPI_ISL_9665343, EPI_ISL_9665479, EPI_ISL_9665637, EPI_ISL_9665769, EPI_ISL_9665941, EPI_ISL_9666164, EPI_ISL_9666295, EPI_ISL_9667434, EPI_ISL_9667506, EPI_ISL_9670828			
see above	Ricardo Gutierrez Children's Hospital, Virology Laboratory, sequencing area.	Ricardo Gutierrez Children's Hospital, Virology Laboratory, sequencing area.	Acuña, Dolores; Goya, Stephanie; Nabaes Jodar, Mercedes S.; Grandis, Érica; María Cristina Alvarez López, María Elina Acevedo, Oscar Jacquez, Sofía Alexay, Mariángeles Barreda Fink, María Emilia Villegas, Raquel Barquez, Estela Chascón, Jorgelina Caruso, Karina Zacarías, Cristian Díaz, Oscar Luna, Cristian Turchiaro, Julián Cipelli, Guillermo Thomas, Carla Medina, Natalia Labarta. Mistchenko, Alicia S.; Viegas, Mariana.

We gratefully acknowledge the following Authors from the Originating laboratories responsible for obtaining the specimens, as well as the Submitting laboratories where the genome data were generated and shared via GISAID, on which this research is based.

All Submitters of data may be contacted directly via www.gisaid.org

Authors are sorted alphabetically.

Accession ID	Originating Laboratory	Submitting Laboratory	Authors
EPI_ISL_11055718	Edendale Gateway clinic	National Institute for Communicable Diseases of the National Health Laboratory Service	Amoako DG, Everatt J, Kekana D, Scheepers C, Mohale T, Ntuli N, Mahlangu B, Mnguni A, Ismail A, Bhiman JN, Wolter N
EPI_ISL_11055719, EPI_ISL_11055720	Edendale hospital	National Institute for Communicable Diseases of the National Health Laboratory Service	Amoako DG, Everatt J, Kekana D, Scheepers C, Mohale T, Ntuli N, Mahlangu B, Mnguni A, Ismail A, Bhiman JN, Wolter N
EPI_ISL_11055721	Edendale Gateway clinic	National Institute for Communicable Diseases of the National Health Laboratory Service	Amoako DG, Everatt J, Kekana D, Scheepers C, Mohale T, Ntuli N, Mahlangu B, Mnguni A, Ismail A, Bhiman JN, Wolter N
EPI_ISL_11055722, EPI_ISL_11055723	Edendale hospital	National Institute for Communicable Diseases of the National Health Laboratory Service	Amoako DG, Everatt J, Kekana D, Scheepers C, Mohale T, Ntuli N, Mahlangu B, Mnguni A, Ismail A, Bhiman JN, Wolter N
EPI_ISL_11055724, EPI_ISL_11055725, EPI_ISL_11055726	Edendale Gateway clinic	National Institute for Communicable Diseases of the National Health Laboratory Service	Amoako DG, Everatt J, Kekana D, Scheepers C, Mohale T, Ntuli N, Mahlangu B, Mnguni A, Ismail A, Bhiman JN, Wolter N
EPI_ISL_11055727, EPI_ISL_11055728, EPI_ISL_11055729, EPI_ISL_11055730, EPI_ISL_11055731	Edendale hospital	National Institute for Communicable Diseases of the National Health Laboratory Service	Amoako DG, Everatt J, Kekana D, Scheepers C, Mohale T, Ntuli N, Mahlangu B, Mnguni A, Ismail A, Bhiman JN, Wolter N
EPI_ISL_11055732, EPI_ISL_11055734, EPI_ISL_11055768	Edendale Gateway clinic	National Institute for Communicable Diseases of the National Health Laboratory Service	Amoako DG, Everatt J, Kekana D, Scheepers C, Mohale T, Ntuli N, Mahlangu B, Mnguni A, Ismail A, Bhiman JN, Wolter N
EPI_ISL_12808997, EPI_ISL_12808998, EPI_ISL_12809001, EPI_ISL_12809005, EPI_ISL_12809006, EPI_ISL_12809007, EPI_ISL_12809008, EPI_ISL_12809054, EPI_ISL_12809057, EPI_ISL_12809058, EPI_ISL_12809060, EPI_ISL_12809061, EPI_ISL_12809062, EPI_ISL_12809063, EPI_ISL_12809064, EPI_ISL_12809076, EPI_ISL_12809077, EPI_ISL_12809078, EPI_ISL_12809079, EPI_ISL_12809093, EPI_ISL_12809111, EPI_ISL_12809131, EPI_ISL_12809145, EPI_ISL_12809146, EPI_ISL_12809160, EPI_ISL_12809162, EPI_ISL_12809163			
see above	ICMR-NATIONAL INSTITUTE OF VIROLOGY INFLUENZA	ICMR-National Institute of Virology Influenza	Dr. varsha potdar and NIC team
EPI_ISL_12870543, EPI_ISL_12870544, EPI_ISL_12870545	Agincourt clinic	National Institute for Communicable Diseases of the National Health Laboratory Service	Amoako DG, Everatt J, Kekana D, Scheepers C, Mohale T, Ntuli N, Mahlangu B, Mnguni A, Ismail A, Bhiman JN, Wolter N
EPI_ISL_12870547	Rahima Moosa Mother and child Hospital	National Institute for Communicable Diseases of the National Health Laboratory Service	Amoako DG, Everatt J, Kekana D, Scheepers C, Mohale T, Ntuli N, Mahlangu B, Mnguni A, Ismail A, Bhiman JN, Wolter N
EPI_ISL_12870552, EPI_ISL_12870553, EPI_ISL_12870554	Edendale hospital	National Institute for Communicable Diseases of the National Health Laboratory Service	Amoako DG, Everatt J, Kekana D, Scheepers C, Mohale T, Ntuli N, Mahlangu B, Mnguni A, Ismail A, Bhiman JN, Wolter N
EPI_ISL_13297850, EPI_ISL_13297987	Sciensano, Department of infectious diseases, Laboratory of viral diseases	Sciensano, Department of infectious diseases, Laboratory of viral diseases	François E. Dufrasne, Sarah Denayer, Steven Van Gucht, Cyril Barbezange
EPI_ISL_14084089, EPI_ISL_14084090, EPI_ISL_14084091	Complexo Hospitalario Universitario de Vigo. Microbiology Department	Complexo Hospitalario Universitario de Vigo. Microbiology Department	Daviña, Carlos; Pizcueta, Juan; Perez, Sonia
EPI_ISL_14769848	Rahima Moosa	National Institute for Communicable Diseases of the National Health Laboratory Service	Amoako DG, Everatt J, Kekana D, Mahlangu B, Stock N, Ntuli N, Mnguni A, Motsatsi G, Nzimande A, Ismail A, Bhiman JN, Wolter N
EPI_ISL_14769850	Red Cross	National Institute for Communicable Diseases of the National Health Laboratory Service	Amoako DG, Everatt J, Kekana D, Mahlangu B, Stock N, Ntuli N, Mnguni A, Motsatsi G, Nzimande A, Ismail A, Bhiman JN, Wolter N
EPI_ISL_15055326, EPI_ISL_15055327, EPI_ISL_15055328, EPI_ISL_15067691, EPI_ISL_15067692, EPI_ISL_15067693	Virology Laboratory, Ricardo Gutiérrez Children's Hospital	Virology Laboratory, Ricardo Gutiérrez Children's Hospital	Acuña, Dolores; Goya, Stephanie; Nabaes Jodar, Mercedes S.; Lucion, M. Florencia; Juárez, María del Valle; Gentile, Ángela; Mistchenko, Alicia S.; Viegas, Mariana
EPI_ISL_15153773	Servicio de Microbiología Hospital Universitario La Paz	IdiPAZ Research Institute	Fernando Lázaro, Iván Bloise, Borja de la Hoz, Marcos López Ortiz, Jesús Mingorance Cruz, Elie Dahdouh
EPI_ISL_15421344	Department of Infectious Diseases in Humans, Viral Diseases Laboratory, Sciensano, Brussels	Department of Infectious Diseases in Humans, Viral Diseases Laboratory, Sciensano, Brussels	François Dufrasne, Sarh Denayer, Steven Van Gucht, Cyril Barbezange
EPI_ISL_15728615	Edendale	National Institute for Communicable Diseases of the National Health Laboratory Service	Everatt J, Kekana D, Mahlangu B, Stock N, Ntozini B, Ntuli N, Mnguni A, Nzimande A, Ismail A, Bhiman JN, Wolter N
EPI_ISL_15728616	Klerksdorp	National Institute for Communicable Diseases of the National Health Laboratory Service	Everatt J, Kekana D, Mahlangu B, Stock N, Ntozini B, Ntuli N, Mnguni A, Nzimande A, Ismail A, Bhiman JN, Wolter N
EPI_ISL_15728617	Mapulaneng	National Institute for Communicable Diseases of the National Health Laboratory Service	Everatt J, Kekana D, Mahlangu B, Stock N, Ntozini B, Ntuli N, Mnguni A, Nzimande A, Ismail A, Bhiman JN, Wolter N
EPI_ISL_15728618	Matikwane	National Institute for Communicable Diseases of the National Health Laboratory Service	Everatt J, Kekana D, Mahlangu B, Stock N, Ntozini B, Ntuli N, Mnguni A, Nzimande A, Ismail A, Bhiman JN, Wolter N
EPI_ISL_15750126	Eurasian Institute of Zoonotic Infections, The Federal Research Center of Fundamental and Translational Medicine	Eurasian Institute of Zoonotic Infections, The Federal Research Center of Fundamental and Translational Medicine	Solomatina,M.V., Kurskaya,O.G., Kabilov,M.R., Tupikin,A.E., Saroyan,T.A., Derko,A.A., Dubovitskiy,N.A., Sobolev,I.A., Shestopalov,A.M., Sharshov,K.A.
EPI_ISL_15820248, EPI_ISL_15820249, EPI_ISL_15820250, EPI_ISL_15820251, EPI_ISL_15820252, EPI_ISL_15820253, EPI_ISL_15820254, EPI_ISL_15820255	Hygiene, IRCCS Ospedale Policlinico San Martino	Hygiene, IRCCS Ospedale Policlinico San Martino	Stefanelli,F., Domnich,A., Bruzzone,B., Trombetta,C.-S., Ricucci,V., Orsi,A., Icardi,G.
EPI_ISL_15895018, EPI_ISL_15895019, EPI_ISL_15895020, EPI_ISL_15895021	Laboratorio Central de Saude Publica do Estado do Rio Grande do Sul (LACEN/RS)	Laboratory of Respiratory Viruses and Measles, Oswaldo Cruz Institute, FIOCRUZ	Paola Resende, Fernando Motta, Elisa Cavalcante Pereira, Bruna Mendonça da Silva, Jéssica Graça Macedo de Carvalho, Larissa Macedo Pinto, Victor Guimaraes, Ighor Leonardo Arantes, Leticia Scalioni, Anderson Brandao Leite, Marilda Siqueira on behalf of the Fiocruz COVID-19 Genomic Surveillance Network
EPI_ISL_15895037, EPI_ISL_15895040, EPI_ISL_15895043, EPI_ISL_15895044	Laboratorio Central de Saude Publica do Estado do Espirito Santo (LACEN/ES)	Laboratory of Respiratory Viruses and Measles, Oswaldo Cruz Institute, FIOCRUZ	Paola Resende, Fernando Motta, Elisa Cavalcante Pereira, Ighor Arantes, Bruna Mendonça da Silva, Jéssica Graça Macedo de Carvalho, Larissa Macedo Pinto, Victor Guimaraes, Leticia Scalioni, Rodrigo Ribeiro Rodrigues, Marilda Siqueira on behalf of the Fiocruz COVID-19 Genomic Surveillance Network
EPI_ISL_15895046, EPI_ISL_15895048, EPI_ISL_15895049, EPI_ISL_15895050, EPI_ISL_15895051, EPI_ISL_15895052, EPI_ISL_15895053, EPI_ISL_15895054	Laboratorio Central de Saude Publica do Estado do Rio Grande do Sul (LACEN/RS)	Laboratory of Respiratory Viruses and Measles, Oswaldo Cruz Institute, FIOCRUZ	Paola Resende, Fernando Motta, Elisa Cavalcante Pereira, Bruna Mendonça da Silva, Jéssica Graça Macedo de Carvalho, Larissa Macedo Pinto, Victor Guimaraes, Ighor Leonardo Arantes, Leticia Scalioni, Anderson Brandao Leite, Marilda Siqueira on behalf of the Fiocruz COVID-19 Genomic Surveillance Network
EPI_ISL_15895070, EPI_ISL_15895073, EPI_ISL_15895076, EPI_ISL_15895082, EPI_ISL_15895083, EPI_ISL_15895085, EPI_ISL_15895086, EPI_ISL_15895087, EPI_ISL_15895088, EPI_ISL_15895089, EPI_ISL_15895091, EPI_ISL_15895092			
see above	Laboratorio Central de Saude Publica do Estado do Espirito Santo (LACEN/ES)	Laboratory of Respiratory Viruses and Measles, Oswaldo Cruz Institute, FIOCRUZ	Paola Resende, Fernando Motta, Elisa Cavalcante Pereira, Ighor Arantes, Bruna Mendonça da Silva, Jéssica Graça Macedo de Carvalho, Larissa Macedo Pinto, Victor Guimaraes, Leticia Scalioni, Rodrigo Ribeiro Rodrigues, Marilda Siqueira on behalf of the Fiocruz COVID-19 Genomic Surveillance Network
EPI_ISL_15895096, EPI_ISL_15895098, EPI_ISL_15895099, EPI_ISL_15895100, EPI_ISL_15895101, EPI_ISL_15895102, EPI_ISL_15895103, EPI_ISL_15895105, EPI_ISL_15895106, EPI_ISL_15895107, EPI_ISL_15895108, EPI_ISL_15895110, EPI_ISL_15895114, EPI_ISL_15895115, EPI_ISL_15895116, EPI_ISL_15895117, EPI_ISL_15895118, EPI_ISL_15895119, EPI_ISL_15895120, EPI_ISL_15895136, EPI_ISL_15895137			
see above	Laboratorio Central de Saude Publica do Estado do Parana (LACEN/PR)	Laboratory of Respiratory Viruses and Measles, Oswaldo Cruz Institute, FIOCRUZ	Paola Resende, Fernando Motta, Elisa Cavalcante Pereira, Ighor Arantes, Bruna Mendonça da Silva, Jéssica Graça Macedo de Carvalho, Larissa Macedo Pinto, Victor Guimaraes, Leticia Scalioni, Irina Riediger, Marilda Siqueira on behalf of the Fiocruz COVID-19 Genomic Surveillance Network
EPI_ISL_15896125	VIC, RCH Molecular Microbiology Dept. (Bio21)	WHO Collaborating Centre for Reference and Research on Influenza	Xiaomin Dong, Steven Edwards, Yi-Mo Deng, Ammar Aziz, Ian Barr
EPI_ISL_15896127	QLD, Queensland Childrens Hospital	WHO Collaborating Centre for Reference and Research on Influenza	Xiaomin Dong, Steven Edwards, Yi-Mo Deng, Ammar Aziz, Ian Barr
EPI_ISL_15896129	VIC, RCH Molecular Microbiology Dept. (Bio21)	WHO Collaborating Centre for Reference and Research on Influenza	Xiaomin Dong, Steven Edwards, Yi-Mo Deng, Ammar Aziz, Ian Barr
EPI_ISL_15896130, EPI_ISL_15896131, EPI_ISL_15896132	QLD, Queensland Childrens Hospital	WHO Collaborating Centre for Reference and Research on Influenza	Xiaomin Dong, Steven Edwards, Yi-Mo Deng, Ammar Aziz, Ian Barr
EPI_ISL_15896133, EPI_ISL_15896134	NT, Royal Darwin Hospital	WHO Collaborating Centre for Reference and Research on Influenza	Xiaomin Dong, Steven Edwards, Yi-Mo Deng, Ammar Aziz, Ian Barr
EPI_ISL_15896136	QLD, Queensland Childrens Hospital	WHO Collaborating Centre for Reference and Research on Influenza	Xiaomin Dong, Steven Edwards, Yi-Mo Deng, Ammar Aziz, Ian Barr
EPI_ISL_15896139, EPI_ISL_15896141, EPI_ISL_15896143	NT, Royal Darwin Hospital	WHO Collaborating Centre for Reference and Research on Influenza	Xiaomin Dong, Steven Edwards, Yi-Mo Deng, Ammar Aziz, Ian Barr
EPI_ISL_15896144, EPI_ISL_15896145, EPI_ISL_15896146	QLD, Queensland Childrens Hospital	WHO Collaborating Centre for Reference and	Xiaomin Dong, Steven Edwards, Yi-Mo Deng, Ammar Aziz, Ian Barr

		Research on Influenza	
EPI_ISL_15896148, EPI_ISL_15896149, EPI_ISL_15896150, EPI_ISL_15896151, EPI_ISL_15896152	VIC, RCH Molecular Microbiology Dept. (Bio21)	WHO Collaborating Centre for Reference and Research on Influenza	Xiaomin Dong, Steven Edwards, Yi-Mo Deng, Ammar Aziz, Ian Barr
EPI_ISL_15896155	VIC, Royal Children's Hospital	WHO Collaborating Centre for Reference and Research on Influenza	Xiaomin Dong, Steven Edwards, Yi-Mo Deng, Ammar Aziz, Ian Barr
EPI_ISL_15896156	NT, Royal Darwin Hospital	WHO Collaborating Centre for Reference and Research on Influenza	Xiaomin Dong, Steven Edwards, Yi-Mo Deng, Ammar Aziz, Ian Barr
EPI_ISL_15896158, EPI_ISL_15896159	VIC, RCH Molecular Microbiology Dept. (Bio21)	WHO Collaborating Centre for Reference and Research on Influenza	Xiaomin Dong, Steven Edwards, Yi-Mo Deng, Ammar Aziz, Ian Barr
EPI_ISL_15896160, EPI_ISL_15896161	NT, Royal Darwin Hospital	WHO Collaborating Centre for Reference and Research on Influenza	Xiaomin Dong, Steven Edwards, Yi-Mo Deng, Ammar Aziz, Ian Barr
EPI_ISL_15896162, EPI_ISL_15896164	QLD, Queensland Childrens Hospital	WHO Collaborating Centre for Reference and Research on Influenza	Xiaomin Dong, Steven Edwards, Yi-Mo Deng, Ammar Aziz, Ian Barr
EPI_ISL_15896166, EPI_ISL_15896167	VIC, RCH Molecular Microbiology Dept. (Bio21)	WHO Collaborating Centre for Reference and Research on Influenza	Xiaomin Dong, Steven Edwards, Yi-Mo Deng, Ammar Aziz, Ian Barr
EPI_ISL_15896168, EPI_ISL_15896169, EPI_ISL_15896170, EPI_ISL_15896171	NT, Royal Darwin Hospital	WHO Collaborating Centre for Reference and Research on Influenza	Xiaomin Dong, Steven Edwards, Yi-Mo Deng, Ammar Aziz, Ian Barr
EPI_ISL_15896172	QLD, Queensland Childrens Hospital	WHO Collaborating Centre for Reference and Research on Influenza	Xiaomin Dong, Steven Edwards, Yi-Mo Deng, Ammar Aziz, Ian Barr
EPI_ISL_15896175, EPI_ISL_15896176, EPI_ISL_15896177, EPI_ISL_15896178, EPI_ISL_15896179, EPI_ISL_15896181, EPI_ISL_15896182, EPI_ISL_15896183	VIC, RCH Molecular Microbiology Dept. (Bio21)	WHO Collaborating Centre for Reference and Research on Influenza	Xiaomin Dong, Steven Edwards, Yi-Mo Deng, Ammar Aziz, Ian Barr
EPI_ISL_15896186, EPI_ISL_15896187, EPI_ISL_15896188, EPI_ISL_15896189, EPI_ISL_15896193	NT, Royal Darwin Hospital	WHO Collaborating Centre for Reference and Research on Influenza	Xiaomin Dong, Steven Edwards, Yi-Mo Deng, Ammar Aziz, Ian Barr
EPI_ISL_15896194, EPI_ISL_15896195	VIC, RCH Molecular Microbiology Dept. (Bio21)	WHO Collaborating Centre for Reference and Research on Influenza	Xiaomin Dong, Steven Edwards, Yi-Mo Deng, Ammar Aziz, Ian Barr
EPI_ISL_15896198, EPI_ISL_15896199, EPI_ISL_15896202, EPI_ISL_15896203	NT, Royal Darwin Hospital	WHO Collaborating Centre for Reference and Research on Influenza	Xiaomin Dong, Steven Edwards, Yi-Mo Deng, Ammar Aziz, Ian Barr
EPI_ISL_15896211, EPI_ISL_15896213, EPI_ISL_15896218	VIC, RCH Molecular Microbiology Dept. (Bio21)	WHO Collaborating Centre for Reference and Research on Influenza	Xiaomin Dong, Steven Edwards, Yi-Mo Deng, Ammar Aziz, Ian Barr
EPI_ISL_15896222	NT, Royal Darwin Hospital	WHO Collaborating Centre for Reference and Research on Influenza	Xiaomin Dong, Steven Edwards, Yi-Mo Deng, Ammar Aziz, Ian Barr
EPI_ISL_15896234	QLD, Queensland Childrens Hospital	WHO Collaborating Centre for Reference and Research on Influenza	Xiaomin Dong, Steven Edwards, Yi-Mo Deng, Ammar Aziz, Ian Barr
EPI_ISL_15896257	NT, Royal Darwin Hospital	WHO Collaborating Centre for Reference and Research on Influenza	Xiaomin Dong, Steven Edwards, Yi-Mo Deng, Ammar Aziz, Ian Barr
EPI_ISL_15896294	VIC, RCH Molecular Microbiology Dept. (Bio21)	WHO Collaborating Centre for Reference and Research on Influenza	Xiaomin Dong, Steven Edwards, Yi-Mo Deng, Ammar Aziz, Ian Barr
EPI_ISL_15953175	Niigata University (IDRC)Infectious Diseases Research Center	Niigata University (IDRC)Infectious Diseases Research Center	Sayaka Yoshioka, Wint Wint Phyu, Irina Chon, Reiko Saito
EPI_ISL_15953176, EPI_ISL_15953177, EPI_ISL_15953178, EPI_ISL_15953179, EPI_ISL_15953180, EPI_ISL_15953181, EPI_ISL_15953234, EPI_ISL_15953393, EPI_ISL_15953394, EPI_ISL_15953468, EPI_ISL_15953469, EPI_ISL_15953471, EPI_ISL_15953472, EPI_ISL_15953474, EPI_ISL_15953476, EPI_ISL_15953478, EPI_ISL_15953479, EPI_ISL_15953480, EPI_ISL_15953481, EPI_ISL_15953483, EPI_ISL_15953485, EPI_ISL_15953486, EPI_ISL_15953488, EPI_ISL_15953490, EPI_ISL_15953492, EPI_ISL_15953494, EPI_ISL_15953497, EPI_ISL_15953498, EPI_ISL_15953499, EPI_ISL_15953686, EPI_ISL_15953948, EPI_ISL_15954275, EPI_ISL_15954574, EPI_ISL_15954707, EPI_ISL_15954709, EPI_ISL_15954711, EPI_ISL_15954715, EPI_ISL_15954717, EPI_ISL_15954720, EPI_ISL_15954722, EPI_ISL_15954725, EPI_ISL_15954727, EPI_ISL_15954752, EPI_ISL_15955487, EPI_ISL_15955488, EPI_ISL_15955489, EPI_ISL_15967288, EPI_ISL_15967289, EPI_ISL_15967290, EPI_ISL_15967291, EPI_ISL_15967337, EPI_ISL_15967338, EPI_ISL_15967339, EPI_ISL_15967340, EPI_ISL_15967341, EPI_ISL_15967450, EPI_ISL_15967451, EPI_ISL_15967564, EPI_ISL_15967714, EPI_ISL_15968088, EPI_ISL_15968168, EPI_ISL_15968273, EPI_ISL_15969109, EPI_ISL_15969408, EPI_ISL_15969409, EPI_ISL_15969442, EPI_ISL_15969443, EPI_ISL_15969610, EPI_ISL_15969611, EPI_ISL_15969613, EPI_ISL_15969714, EPI_ISL_15969794, EPI_ISL_15969795, EPI_ISL_15969893, EPI_ISL_15969894, EPI_ISL_15969895, EPI_ISL_15969896, EPI_ISL_15969897			
see above	Niigata University (IDRC) Infectious Diseases Research Center	Niigata University (IDRC) Infectious Diseases Research Center	Sayaka Yoshioka, Wint Wint Phyu, Irina Chon, Reiko Saito
EPI_ISL_16132328	University of WashingtonLaboratory Medicine and Pathology, UW Medicine	University of WashingtonLaboratory Medicine and Pathology, UW Medicine	Goya,S., Sereewit,J., Pfalmer,D., Nguyen,T., Sobolik,E.B. and Greninger,A.L.
EPI_ISL_16132330, EPI_ISL_16132331, EPI_ISL_16132332, EPI_ISL_16132333, EPI_ISL_16132334, EPI_ISL_16132336, EPI_ISL_16132338, EPI_ISL_16132339, EPI_ISL_16132340, EPI_ISL_16132341, EPI_ISL_16132342, EPI_ISL_16132343, EPI_ISL_16132344, EPI_ISL_16132345, EPI_ISL_16132346, EPI_ISL_16132347, EPI_ISL_16132348, EPI_ISL_16132349, EPI_ISL_16132350, EPI_ISL_16132351, EPI_ISL_16132353, EPI_ISL_16132354, EPI_ISL_16132355, EPI_ISL_16132356, EPI_ISL_16132357, EPI_ISL_16132360, EPI_ISL_16132361, EPI_ISL_16132362, EPI_ISL_16132363, EPI_ISL_16132364, EPI_ISL_16132365, EPI_ISL_16132366, EPI_ISL_16132368, EPI_ISL_16132369, EPI_ISL_16132370, EPI_ISL_16132371, EPI_ISL_16132372, EPI_ISL_16132376, EPI_ISL_16132377, EPI_ISL_16132378			
see above	Infectious Disease Program, Broad Institute of Harvard and MIT	Infectious Disease Program, Broad Institute of Harvard and MIT	Adams,G., Uddin,R., Messer,K., Dobbins,S., Kotzen,B., Siddle,K.J., Petros,B., Paull,J., Brock-Fisher,T., Levine,Z., Krasilnikova,L., Negrete-Arenas,F., Tomkins-Tinch,C., Chaluvadi,S., Marmol,C., DeRuff,K., Loreth,C., Birren,B.W., Park,D.J., MacInnis,B.L., Sabeti,P.C., Rosenberg,E., Turbett,S., Lemieux,J.E., Adams,G., Uddin,R., Messer,K., Dobbins,S., Kotzen,B., Siddle,K.J., Petros,B., Paull,J., Brock-Fisher,T., Levine,Z., Krasilnikova,L., Negrete-Arenas,F., Tomkins-Tinch,C., Chaluvadi,S., Marmol,C., DeRuff,K., Loreth,C., Birren,B.W., Park,D.J., MacInnis,B.L., Sabeti,P.C., Rosenberg,E., Turbett,S., Lemieux,J.E.
EPI_ISL_16132384, EPI_ISL_16132385, EPI_ISL_16132386, EPI_ISL_16132387, EPI_ISL_16132388, EPI_ISL_16132389, EPI_ISL_16132390, EPI_ISL_16132391, EPI_ISL_16132392, EPI_ISL_16132393, EPI_ISL_16132394, EPI_ISL_16132395, EPI_ISL_16132396, EPI_ISL_16132397, EPI_ISL_16132398, EPI_ISL_16132399, EPI_ISL_16132400, EPI_ISL_16132401, EPI_ISL_16132402, EPI_ISL_16132403, EPI_ISL_16132404, EPI_ISL_16132405, EPI_ISL_16132406, EPI_ISL_16132407, EPI_ISL_16132408, EPI_ISL_16132409, EPI_ISL_16132410, EPI_ISL_16132411, EPI_ISL_16132412			
see above	University of WashingtonLaboratory Medicine and Pathology, UW Medicine	University of WashingtonLaboratory Medicine and Pathology, UW Medicine	Goya,S., Sereewit,J., Pfalmer,D., Nguyen,T., Sobolik,E.B. and Greninger,A.L.
EPI_ISL_16251628, EPI_ISL_16251629, EPI_ISL_16251631, EPI_ISL_16251632, EPI_ISL_16251633, EPI_ISL_16251634, EPI_ISL_16251635, EPI_ISL_16251638, EPI_ISL_16251639, EPI_ISL_16251640, EPI_ISL_16251641, EPI_ISL_16251643, EPI_ISL_16251646, EPI_ISL_16251647, EPI_ISL_16251650, EPI_ISL_16251651, EPI_ISL_16251652, EPI_ISL_16251653, EPI_ISL_16251654, EPI_ISL_16251656, EPI_ISL_16251657, EPI_ISL_16251658, EPI_ISL_16251661, EPI_ISL_16251663, EPI_ISL_16251664, EPI_ISL_16251665, EPI_ISL_16251668, EPI_ISL_16251671, EPI_ISL_16251672, EPI_ISL_16251673, EPI_ISL_16251676, EPI_ISL_16251677, EPI_ISL_16251678, EPI_ISL_16251679, EPI_ISL_16251680, EPI_ISL_16251682, EPI_ISL_16251683, EPI_ISL_16289019, EPI_ISL_16289021, EPI_ISL_16289023, EPI_ISL_16289024, EPI_ISL_16289025, EPI_ISL_16289026, EPI_ISL_16289027, EPI_ISL_16289028, EPI_ISL_16289029, EPI_ISL_16289030, EPI_ISL_16289031, EPI_ISL_16289032, EPI_ISL_16289033, EPI_ISL_16289034, EPI_ISL_16289035, EPI_ISL_16289038, EPI_ISL_16289039, EPI_ISL_16289040, EPI_ISL_16289041, EPI_ISL_16289042, EPI_ISL_16289044, EPI_ISL_16289045, EPI_ISL_16289046, EPI_ISL_16289047, EPI_ISL_16289048			
see above	Microbiology Department. Complexo Hospitalario Universitario de Vigo	Microbiology Department. Complexo Hospitalario Universitario de Vigo	Daviña C, Martinez L, Perez-Castro S
EPI_ISL_16533870, EPI_ISL_16533871, EPI_ISL_16533873, EPI_ISL_16533874	Center for Virology, Medical University of Vienna	Center for Virology, Medical University of Vienna	Jeremy V. Camp, Monika Redlberger-Fritz
EPI_ISL_16609707, EPI_ISL_16609710, EPI_ISL_16609731, EPI_ISL_16609733, EPI_ISL_16609734, EPI_ISL_16609741, EPI_ISL_16609742, EPI_ISL_16609746, EPI_ISL_16609747, EPI_ISL_16609749, EPI_ISL_16609750, EPI_ISL_16609752, EPI_ISL_16609754, EPI_ISL_16609755, EPI_ISL_16609758, EPI_ISL_16609760, EPI_ISL_16609764, EPI_ISL_16609766, EPI_ISL_16672796, EPI_ISL_16672797, EPI_ISL_16672798, EPI_ISL_16672826, EPI_ISL_16672827, EPI_ISL_16672828, EPI_ISL_16672829, EPI_ISL_16672831, EPI_ISL_16672833, EPI_ISL_16672836, EPI_ISL_16672837, EPI_ISL_16672838, EPI_ISL_16672839, EPI_ISL_16672840, EPI_ISL_16672841, EPI_ISL_16672842, EPI_ISL_16672843, EPI_ISL_16672844, EPI_ISL_16672845, EPI_ISL_16672846, EPI_ISL_16672847, EPI_ISL_16672851, EPI_ISL_16672852, EPI_ISL_16672853, EPI_ISL_16672882, EPI_ISL_16672887, EPI_ISL_16672889, EPI_ISL_16672892, EPI_ISL_16672893, EPI_ISL_16672894, EPI_ISL_16672895, EPI_ISL_16672896, EPI_ISL_16672897, EPI_ISL_16672898, EPI_ISL_16672899, EPI_ISL_16672900, EPI_ISL_16672901, EPI_ISL_16672902, EPI_ISL_16672903, EPI_ISL_16672919, EPI_ISL_16672920, EPI_ISL_16672926, EPI_ISL_16672927, EPI_ISL_16672928, EPI_ISL_16672930, EPI_ISL_16672931, EPI_ISL_16672934, EPI_ISL_16672938, EPI_ISL_16672940, EPI_ISL_16672942, EPI_ISL_16672943, EPI_ISL_16672944, EPI_ISL_16672946, EPI_ISL_16672947, EPI_ISL_16672948, EPI_ISL_16672949, EPI_ISL_16672950, EPI_ISL_16672951, EPI_ISL_16672952, EPI_ISL_16672953, EPI_ISL_16672954, EPI_ISL_16672955, EPI_ISL_16672956, EPI_ISL_16672957, EPI_ISL_16672958, EPI_ISL_16672959, EPI_ISL_16672960, EPI_ISL_16672961, EPI_ISL_16672962, EPI_ISL_16672963, EPI_ISL_16672964, EPI_ISL_16672965, EPI_ISL_16672966, EPI_ISL_16672967, EPI_ISL_16672968, EPI_ISL_16672969, EPI_ISL_16672970, EPI_ISL_16672971, EPI_ISL_16672972, EPI_ISL_16672973, EPI_ISL_16672974, EPI_ISL_16672975, EPI_ISL_16672976, EPI_ISL_16672977, EPI_ISL_16672978, EPI_ISL_16672979, EPI_ISL_16672980, EPI_ISL_16672981, EPI_ISL_16672982, EPI_ISL_16672983, EPI_ISL_16672984, EPI_ISL_16672985, EPI_ISL_16672986, EPI_ISL_16672987, EPI_ISL_16672988, EPI_ISL_16672989, EPI_ISL_16672990, EPI_ISL_16672991, EPI_ISL_16672992, EPI_ISL_16672993, EPI_ISL_16672994, EPI_ISL_16672995, EPI_ISL_16672996, EPI_ISL_16672997, EPI_ISL_16672998			
see above	Respiratory Virus Unit / Reference Microbiology Services / UK Health Security Agency	Reference Microbiology Services / UK Health Security Agency	Zambon M. Talts T. Mosscrop L. Miah S
EPI_ISL_16681178	King Saud University	King Saud University	Alzayed,R.A., Aziz,I.M., Dudin,G.A., Alsaleh,A.N., Ahmed,A.A. and Almajhdi,F.N.
EPI_ISL_16681182, EPI_ISL_16681183	Broad Institute of Harvard and Massachusetts Institute of Technology	Broad Institute of Harvard and Massachusetts Institute of Technology	Adams,G., Uddin,R., Messer,K., Dobbins,S., Kotzen,B., Siddle,K.J., Petros,B., Paull,J., Brock-Fisher,T., Levine,Z., Krasilnikova,L., Negrete-Arenas,F., Tomkins-Tinch,C., Chaluvadi,S., Marmol,C., DeRuff,K., Loreth,C., Birren,B.W., Park,D.J., MacInnis,B.L., Sabeti,P.C., Rosenberg,E., Turbett,S. and Lemieux,J.E.
EPI_ISL_16681187, EPI_ISL_16681196	Kenya Medical Research Institute-Wellcome Trust	Kenya Medical Research Institute-Wellcome Trust	Lambisia,A.W., Makori,T.O., Mutunga,M., Lewa,C., Githinji,G., Agoti,C.N. and Nokes,J.D.
EPI_ISL_16681201	Broad Institute of Harvard and Massachusetts Institute of Technology	Broad Institute of Harvard and Massachusetts Institute of Technology	Adams,G., Uddin,R., Messer,K., Dobbins,S., Kotzen,B., Siddle,K.J., Petros,B., Paull,J., Brock-Fisher,T., Levine,Z., Krasilnikova,L., Negrete-Arenas,F., Tomkins-Tinch,C., Chaluvadi,S., Marmol,C., DeRuff,K., Loreth,C., Birren,B.W., Park,D.J., MacInnis,B.L., Sabeti,P.C., Rosenberg,E., Turbett,S. and Lemieux,J.E.
EPI_ISL_16681210	King Saud University	King Saud University	Alzayed,R.A., Aziz,I.M., Dudin,G.A., Alsaleh,A.N., Ahmed,A.A. and Almajhdi,F.N.
EPI_ISL_16681217, EPI_ISL_16681226	Kenya Medical Research Institute-Wellcome Trust	Kenya Medical Research Institute-Wellcome Trust	Lambisia,A.W., Makori,T.O., Mutunga,M., Lewa,C., Githinji,G., Agoti,C.N. and Nokes,J.D.
EPI_ISL_16681239, EPI_ISL_16681240, EPI_ISL_16681247, EPI_ISL_16681257, EPI_ISL_16681258, EPI_ISL_16681259, EPI_ISL_16681261, EPI_ISL_16681270, EPI_ISL_16681271, EPI_ISL_16681273, EPI_ISL_16681283, EPI_ISL_16681284, EPI_ISL_16681285			
see above	Broad Institute of Harvard and Massachusetts Institute of Technology	Broad Institute of Harvard and Massachusetts Institute of Technology	Adams,G., Uddin,R., Messer,K., Dobbins,S., Kotzen,B., Siddle,K.J., Petros,B., Paull,J., Brock-Fisher,T., Levine,Z., Krasilnikova,L., Negrete-Arenas,F., Tomkins-Tinch,C., Chaluvadi,S., Marmol,C., DeRuff,K., Loreth,C., Birren,B.W., Park,D.J., MacInnis,B.L., Sabeti,P.C., Rosenberg,E., Turbett,S. and Lemieux,J.E.
EPI_ISL_16681289	Kenya Medical Research Institute-Wellcome Trust	Kenya Medical Research Institute-Wellcome Trust	Lambisia,A.W., Makori,T.O., Mutunga,M., Lewa,C., Githinji,G., Agoti,C.N. and Nokes,J.D.
EPI_ISL_16681296, EPI_ISL_16681297, EPI_ISL_16681298, EPI_ISL_16681300, EPI_ISL_16681301, EPI_ISL_16681308, EPI_ISL_16681309, EPI_ISL_16681310, EPI_ISL_16681311, EPI_ISL_16681323, EPI_ISL_16681325, EPI_ISL_16681326, EPI_ISL_16681336, EPI_ISL_16681337, EPI_ISL_16681339, EPI_ISL_16681340, EPI_ISL_16681341, EPI_ISL_16681349			
see above	Broad Institute of Harvard and Massachusetts	Broad Institute of Harvard and Massachusetts	Adams,G., Uddin,R., Messer,K., Dobbins,S., Kotzen,B., Siddle,K.J., Petros,B., Paull,J., Brock-Fisher,T., Levine,Z., Krasilnikova,L., Negrete-Arenas,F., Tomkins-Tinch,C., Chaluvadi,S., Marmol,C., DeRuff,K., Loreth,C., Birren,B.W., Park,D.J.,

	Institute of Technology	Institute of Technology	MacInnis,B.L., Sabeti,P.C., Rosenberg,E., Turbett,S. and Lemieux,J.E.
EPI_ISL_16681352	King Saud University	King Saud University	Alzayed,R.A., Aziz,I.M., Dudin,G.A., Alsaleh,A.N., Ahmed,A.A. and Almajhdi,F.N.
EPI_ISL_16681359	Kenya Medical Research Institute-Wellcome Trust	Kenya Medical Research Institute-Wellcome Trust	Lambisia,A.W., Makori,T.O., Mutunga,M., Lewa,C., Githinji,G., Agoti,C.N. and Nokes,J.D.
EPI_ISL_16681360	Broad Institute of Harvard and Massachusetts Institute of Technology	Broad Institute of Harvard and Massachusetts Institute of Technology	Adams,G., Uddin,R., Messer,K., Dobbins,S., Kotzen,B., Siddle,K.J., Petros,B., Paull,J., Brock-Fisher,T., Levine,Z., Krasilnikova,L., Negrete-Arenas,F., Tomkins-Tinch,C., Chaluvadi,S., Marmol,C., DeRuff,K., Loreth,C., Birren,B.W., Park,D.J., MacInnis,B.L., Sabeti,P.C., Rosenberg,E., Turbett,S. and Lemieux,J.E.
EPI_ISL_16681362	Kenya Medical Research Institute-Wellcome Trust	Kenya Medical Research Institute-Wellcome Trust	Lambisia,A.W., Makori,T.O., Mutunga,M., Lewa,C., Githinji,G., Agoti,C.N. and Nokes,J.D.
EPI_ISL_16681403	King Saud University	King Saud University	Alzayed,R.A., Aziz,I.M., Dudin,G.A., Alsaleh,A.N., Ahmed,A.A. and Almajhdi,F.N.
EPI_ISL_16681408	Broad Institute of Harvard and Massachusetts Institute of Technology	Broad Institute of Harvard and Massachusetts Institute of Technology	Adams,G., Uddin,R., Messer,K., Dobbins,S., Kotzen,B., Siddle,K.J., Petros,B., Paull,J., Brock-Fisher,T., Levine,Z., Krasilnikova,L., Negrete-Arenas,F., Tomkins-Tinch,C., Chaluvadi,S., Marmol,C., DeRuff,K., Loreth,C., Birren,B.W., Park,D.J., MacInnis,B.L., Sabeti,P.C., Rosenberg,E., Turbett,S. and Lemieux,J.E.
EPI_ISL_16708990, EPI_ISL_16708991, EPI_ISL_16708992, EPI_ISL_16708993, EPI_ISL_16708995, EPI_ISL_16708996, EPI_ISL_16708997, EPI_ISL_16708998, EPI_ISL_16709002, EPI_ISL_16709004, EPI_ISL_16709005, EPI_ISL_16709009, EPI_ISL_16709011			
see above	University Hospitals of Leicester NHS Trust Pathology Services / Leicester Royal Infirmary	Reference Microbiology Services / UK Health Security Agency	Williams T. Zambon M. Talts T. Mosscrop L. Holmes C. Miah S
EPI_ISL_16714280, EPI_ISL_16714281, EPI_ISL_16714282, EPI_ISL_16714284, EPI_ISL_16714285, EPI_ISL_16714286, EPI_ISL_16714288, EPI_ISL_16714290, EPI_ISL_16714292, EPI_ISL_16714293, EPI_ISL_16714295, EPI_ISL_16714296, EPI_ISL_16714297, EPI_ISL_16714299, EPI_ISL_16714300, EPI_ISL_16714301, EPI_ISL_16714302, EPI_ISL_16714303, EPI_ISL_16714304, EPI_ISL_16714308, EPI_ISL_16714309, EPI_ISL_16714310, EPI_ISL_16714311, EPI_ISL_16714312, EPI_ISL_16714314, EPI_ISL_16714316, EPI_ISL_16714317, EPI_ISL_16714318, EPI_ISL_16714320, EPI_ISL_16714321, EPI_ISL_16714322, EPI_ISL_16714324, EPI_ISL_16714325, EPI_ISL_16714326, EPI_ISL_16714329, EPI_ISL_16714331, EPI_ISL_16714337, EPI_ISL_16714341, EPI_ISL_16714343, EPI_ISL_16714344, EPI_ISL_16714346, EPI_ISL_16714347, EPI_ISL_16714350, EPI_ISL_16714354, EPI_ISL_16714355, EPI_ISL_16714360, EPI_ISL_16714361, EPI_ISL_16714362, EPI_ISL_16714363, EPI_ISL_16714365, EPI_ISL_16714366, EPI_ISL_16714367, EPI_ISL_16714368, EPI_ISL_16714372, EPI_ISL_16714373, EPI_ISL_16714374, EPI_ISL_16714376, EPI_ISL_16714379, EPI_ISL_16714381, EPI_ISL_16714383, EPI_ISL_16714388, EPI_ISL_16714390, EPI_ISL_16714391, EPI_ISL_16714392, EPI_ISL_16714393, EPI_ISL_16714395, EPI_ISL_16714396, EPI_ISL_16714397, EPI_ISL_16714398, EPI_ISL_16714399, EPI_ISL_16714400, EPI_ISL_16714401, EPI_ISL_16714402, EPI_ISL_16714403, EPI_ISL_16714404, EPI_ISL_16714405, EPI_ISL_16714406, EPI_ISL_16714407, EPI_ISL_16714409, EPI_ISL_16714411, EPI_ISL_16714412, EPI_ISL_16714414, EPI_ISL_16714415, EPI_ISL_16714416, EPI_ISL_16714417, EPI_ISL_16714419, EPI_ISL_16714420, EPI_ISL_16714425, EPI_ISL_16714428, EPI_ISL_16714430, EPI_ISL_16714431, EPI_ISL_16714432, EPI_ISL_16714433, EPI_ISL_16714434, EPI_ISL_16714435, EPI_ISL_16714436, EPI_ISL_16714437, EPI_ISL_16714438, EPI_ISL_16714439, EPI_ISL_16714440, EPI_ISL_16714441, EPI_ISL_16714442, EPI_ISL_16714443, EPI_ISL_16714444, EPI_ISL_16714445, EPI_ISL_16714446, EPI_ISL_16714447, EPI_ISL_16714448, EPI_ISL_16714449, EPI_ISL_16714450, EPI_ISL_16714451, EPI_ISL_16714452, EPI_ISL_16714455, EPI_ISL_16714456, EPI_ISL_16714457, EPI_ISL_16714458, EPI_ISL_16714459, EPI_ISL_16714461, EPI_ISL_16714462, EPI_ISL_16714464, EPI_ISL_16714465, EPI_ISL_16714467, EPI_ISL_16714468, EPI_ISL_16714471, EPI_ISL_16714473			
see above	University Hospital Southampton / Southampton Specialist Virology Centre	Reference Microbiology Services / UK Health Security Agency	Williams T. Zambon M. Talts T. Mosscrop L. Pelosi E. Miah S
EPI_ISL_16714494, EPI_ISL_16714495, EPI_ISL_16714496, EPI_ISL_16714497, EPI_ISL_16714498, EPI_ISL_16714499, EPI_ISL_16714500, EPI_ISL_16714501, EPI_ISL_16714502, EPI_ISL_16714503, EPI_ISL_16714504, EPI_ISL_16714505, EPI_ISL_16714506, EPI_ISL_16714507, EPI_ISL_16714508, EPI_ISL_16714509, EPI_ISL_16714510, EPI_ISL_16714511, EPI_ISL_16714512, EPI_ISL_16714513, EPI_ISL_16714514, EPI_ISL_16714515, EPI_ISL_16714516, EPI_ISL_16714517, EPI_ISL_16714518, EPI_ISL_16714519, EPI_ISL_16714520, EPI_ISL_16714521, EPI_ISL_16714522, EPI_ISL_16714523, EPI_ISL_16714524, EPI_ISL_16714525, EPI_ISL_16714526, EPI_ISL_16714527, EPI_ISL_16714528, EPI_ISL_16714529, EPI_ISL_16714530, EPI_ISL_16714531, EPI_ISL_16714532, EPI_ISL_16714533, EPI_ISL_16714534, EPI_ISL_16714535, EPI_ISL_16714536, EPI_ISL_16714537, EPI_ISL_16714538, EPI_ISL_16714539, EPI_ISL_16714540, EPI_ISL_16714541, EPI_ISL_16714542, EPI_ISL_16714543, EPI_ISL_16714544, EPI_ISL_16714545, EPI_ISL_16714546, EPI_ISL_16714547, EPI_ISL_16714548, EPI_ISL_16714549, EPI_ISL_16714550, EPI_ISL_16714551, EPI_ISL_16714552, EPI_ISL_16714553, EPI_ISL_16714554, EPI_ISL_16714555, EPI_ISL_16714556, EPI_ISL_16714557, EPI_ISL_16714558, EPI_ISL_16714559, EPI_ISL_16714560, EPI_ISL_16714561, EPI_ISL_16714562, EPI_ISL_16714563, EPI_ISL_16714564, EPI_ISL_16714565, EPI_ISL_16714566, EPI_ISL_16714567, EPI_ISL_16714568, EPI_ISL_16714569, EPI_ISL_16714570, EPI_ISL_16714571, EPI_ISL_16714575, EPI_ISL_16714577, EPI_ISL_16714579, EPI_ISL_16714582, EPI_ISL_16714588, EPI_ISL_16714591, EPI_ISL_16714593, EPI_ISL_16714617, EPI_ISL_16714619, EPI_ISL_16714688, EPI_ISL_16714689, EPI_ISL_16714690, EPI_ISL_16714691, EPI_ISL_16714692, EPI_ISL_16714693, EPI_ISL_16714694, EPI_ISL_16714695, EPI_ISL_16714696, EPI_ISL_16714697, EPI_ISL_16714698, EPI_ISL_16714699, EPI_ISL_16714700, EPI_ISL_16714701, EPI_ISL_16714702, EPI_ISL_16714703, EPI_ISL_16714704, EPI_ISL_16714740, EPI_ISL_16714743			
see above	Respiratory Virus Unit / Reference Microbiology Services / UK Health Security Agency	Reference Microbiology Services / UK Health Security Agency	Zambon M. Talts T. Mosscrop L. Miah S
EPI_ISL_16714745, EPI_ISL_16714747, EPI_ISL_16714749	VIC, RCH Molecular Microbiology Dept. (Bio21)	WHO Collaborating Centre for Reference and Research on Influenza	Xiaomin Dong, Steven Edwards, Yi-Mo Deng, Ammar Aziz, Ian Barr
EPI_ISL_16714760, EPI_ISL_16714770, EPI_ISL_16714774, EPI_ISL_16714780	NT, Royal Darwin Hospital	WHO Collaborating Centre for Reference and Research on Influenza	Xiaomin Dong, Steven Edwards, Yi-Mo Deng, Ammar Aziz, Ian Barr
EPI_ISL_16714781, EPI_ISL_16714786, EPI_ISL_16714789, EPI_ISL_16714790, EPI_ISL_16714791, EPI_ISL_16714795, EPI_ISL_16714797, EPI_ISL_16714799, EPI_ISL_16714800	VIC, Royal Children's Hospital Victoria Australia	WHO Collaborating Centre for Reference and Research on Influenza	Xiaomin Dong, Steven Edwards, Yi-Mo Deng, Ammar Aziz, Ian Barr
EPI_ISL_16714803, EPI_ISL_16714809, EPI_ISL_16714810	NT, Royal Darwin Hospital	WHO Collaborating Centre for Reference and Research on Influenza	Xiaomin Dong, Steven Edwards, Yi-Mo Deng, Ammar Aziz, Ian Barr
EPI_ISL_16737029	Respiratory Virus Unit / Reference Microbiology Services / UK Health Security Agency	Reference Microbiology Services / UK Health Security Agency	Zambon M. Talts T. Mosscrop L. Miah S
EPI_ISL_16737030, EPI_ISL_16737033, EPI_ISL_16737038	University Hospital Southampton / Southampton Specialist Virology Centre	Reference Microbiology Services / UK Health Security Agency	Williams T. Zambon M. Talts T. Mosscrop L. Pelosi E. Miah S
EPI_ISL_16746475	Respiratory Virus Unit / Reference Microbiology Services / UK Health Security Agency	Reference Microbiology Services / UK Health Security Agency	Zambon M. Talts T. Mosscrop L. Miah S
EPI_ISL_16839032, EPI_ISL_16839033, EPI_ISL_16839034, EPI_ISL_16839035, EPI_ISL_16839036, EPI_ISL_16839037, EPI_ISL_16839038	West of Scotland Specialist Virology Centre	West of Scotland Specialist Virology Centre	Lynne Ferguson, Imogen Johnston-Menzies, Rory Gunson
EPI_ISL_16842706, EPI_ISL_16842848, EPI_ISL_16842849, EPI_ISL_16842850, EPI_ISL_16842851, EPI_ISL_16842852, EPI_ISL_16842853, EPI_ISL_16842854, EPI_ISL_16842855, EPI_ISL_16842856, EPI_ISL_16842857, EPI_ISL_16842858, EPI_ISL_16842859, EPI_ISL_16842860, EPI_ISL_16842861, EPI_ISL_16842862, EPI_ISL_16842863, EPI_ISL_16844016, EPI_ISL_16844017, EPI_ISL_16844018, EPI_ISL_16844019, EPI_ISL_16844020, EPI_ISL_16844026, EPI_ISL_16844028			
see above	Niigata University (IDRC) Infectious Diseases Research Center	Niigata University (IDRC) Infectious Diseases Research Center	Sayaka Yoshioka, Wint Wint Phyu, Irina Chon, Reiko Saito
EPI_ISL_16959528	ESR - Wallaceville	Institute of Environmental Science and Research	Klarysse Berquist, Lauren Jelley, Una Ren, Meaghan O'Neill
EPI_ISL_16960100, EPI_ISL_16960101	Middlemore Hospital	Institute of Environmental Science and Research	Klarysse Berquist, Lauren Jelley, Una Ren, Meaghan O'Neill
EPI_ISL_16960103	Wellington SCL	Institute of Environmental Science and Research	Klarysse Berquist, Lauren Jelley, Una Ren, Meaghan O'Neill
EPI_ISL_16960111, EPI_ISL_16960112, EPI_ISL_16960114, EPI_ISL_16960115	Middlemore Hospital	Institute of Environmental Science and Research	Klarysse Berquist, Lauren Jelley, Una Ren, Meaghan O'Neill
EPI_ISL_16960118, EPI_ISL_16960125, EPI_ISL_16960129	Southern Community Lab - Dunedin	Institute of Environmental Science and Research	Klarysse Berquist, Lauren Jelley, Una Ren, Meaghan O'Neill
EPI_ISL_16960130, EPI_ISL_16960131, EPI_ISL_16960132, EPI_ISL_16960136, EPI_ISL_16960139	LabPlus	Institute of Environmental Science and Research	Klarysse Berquist, Lauren Jelley, Una Ren, Meaghan O'Neill
EPI_ISL_16960149	Canterbury Health Laboratory	Institute of Environmental Science and Research	Klarysse Berquist, Lauren Jelley, Una Ren, Meaghan O'Neill
EPI_ISL_17066776, EPI_ISL_17066777	VIC, Royal Children's Hospital Victoria Australia	WHO Collaborating Centre for Reference and Research on Influenza	Xiaomin Dong, Steven Edwards, Yi-Mo Deng, Ammar Aziz, Ian Barr
EPI_ISL_17066784	NT, Royal Darwin Hospital	WHO Collaborating Centre for Reference and Research on Influenza	Xiaomin Dong, Steven Edwards, Yi-Mo Deng, Ammar Aziz, Ian Barr
EPI_ISL_17066788, EPI_ISL_17066793, EPI_ISL_17066795, EPI_ISL_17066802, EPI_ISL_17066813, EPI_ISL_17066817	QLD, Pathology Queensland - Royal Brisbane and Women's Hospital	WHO Collaborating Centre for Reference and Research on Influenza	Xiaomin Dong, Steven Edwards, Yi-Mo Deng, Ammar Aziz, Ian Barr
EPI_ISL_17066819, EPI_ISL_17066820, EPI_ISL_17066821	NT, Royal Darwin Hospital	WHO Collaborating Centre for Reference and Research on Influenza	Xiaomin Dong, Steven Edwards, Yi-Mo Deng, Ammar Aziz, Ian Barr
EPI_ISL_17066824, EPI_ISL_17066827, EPI_ISL_17066828, EPI_ISL_17066831, EPI_ISL_17066833, EPI_ISL_17066834	VIC, Royal Children's Hospital Victoria Australia	WHO Collaborating Centre for Reference and Research on Influenza	Xiaomin Dong, Steven Edwards, Yi-Mo Deng, Ammar Aziz, Ian Barr
EPI_ISL_17127009, EPI_ISL_17127010	Servicio de Microbiologia, Hospital Universitario Virgen del Rocio	Plataforma de Medicina Computacional, Fundación Progreso y Salud	José Antonio Lepe, Javier Pérez-Florido, María Lara Jiménez
EPI_ISL_17216312, EPI_ISL_17216669, EPI_ISL_17217289, EPI_ISL_17217321, EPI_ISL_17217667, EPI_ISL_17217703, EPI_ISL_17217859, EPI_ISL_17217912, EPI_ISL_17218380, EPI_ISL_17218769, EPI_ISL_17219098, EPI_ISL_17219288, EPI_ISL_17219322, EPI_ISL_17219626, EPI_ISL_17219636, EPI_ISL_17219931, EPI_ISL_17219998, EPI_ISL_17220517, EPI_ISL_17220604, EPI_ISL_17221487			
see above	Astrazeneca Laboratory	Astrazeneca Laboratory	Wilkins,D., Langedijk,A.C., Lebbink,R.J., Morehouse,C.A., Abram,M.E., Ahani,B., Aksyuk,A.A., Baraldi,E., Brady,T., Chen,A.T., Chi,H., Choi,E.H., Cohen,R., Danilenko,D.M., Gopalakrishnan,V., Greenough,A., Heikkinen,T., Hosoya,M., Keller,C., Kelly,E.J., Kragten-Tabatabaie,L., Martinon-Torres,F., Mascarenas-de Los Santos,A.H., Nunes,M.C., Palomino,M.A., Papenburg,J., Pernica,J.M., Richmond,P., Stein,R.T., Tuffy,K.M., Verwey,C., Esser,M.T., Tabor,D.E. and Bont,L.J.
EPI_ISL_17221538, EPI_ISL_17221542, EPI_ISL_17221543, EPI_ISL_17221544, EPI_ISL_17221545, EPI_ISL_17221547, EPI_ISL_17221548, EPI_ISL_17221549, EPI_ISL_17221551, EPI_ISL_17221552, EPI_ISL_17221553, EPI_ISL_17221554, EPI_ISL_17221555, EPI_ISL_17221556, EPI_ISL_17221559, EPI_ISL_17221560, EPI_ISL_17221562, EPI_ISL_17221564, EPI_ISL_17221565, EPI_ISL_17221566, EPI_ISL_17221568, EPI_ISL_17221569, EPI_ISL_17221571, EPI_ISL_17221572, EPI_ISL_17221573, EPI_ISL_17221574, EPI_ISL_17221575, EPI_ISL_17221576, EPI_ISL_17221577, EPI_ISL_17221579, EPI_ISL_17221581, EPI_ISL_17221583, EPI_ISL_17221587, EPI_ISL_17221588, EPI_ISL_17221589, EPI_ISL_17221591, EPI_ISL_17221592, EPI_ISL_17221595, EPI_ISL_17221596, EPI_ISL_17221598, EPI_ISL_17221599, EPI_ISL_17221600, EPI_ISL_17221601, EPI_ISL_17221602, EPI_ISL_17221603, EPI_ISL_17221604, EPI_ISL_17221608, EPI_ISL_17221609, EPI_ISL_17221610, EPI_ISL_17221611, EPI_ISL_17221612, EPI_ISL_17221613, EPI_ISL_17221614, EPI_ISL_17221617, EPI_ISL_17221618, EPI_ISL_17221619, EPI_ISL_17221620, EPI_ISL_17221621, EPI_ISL_17221622, EPI_ISL_17221624, EPI_ISL_17221626, EPI_ISL_17221627, EPI_ISL_17221628, EPI_ISL_17221630, EPI_ISL_17221631, EPI_ISL_17221632, EPI_ISL_17221633, EPI_ISL_17221635, EPI_ISL_17221638, EPI_ISL_17221640, EPI_ISL_17221641, EPI_ISL_17221642, EPI_ISL_17221643, EPI_ISL_17221645, EPI_ISL_17221646, EPI_ISL_17221650, EPI_ISL_17221651, EPI_ISL_17221652, EPI_ISL_17221653, EPI_ISL_17221657, EPI_ISL_17221661, EPI_ISL_17221663, EPI_ISL_17221664, EPI_ISL_17221665			
see above	University of Ljubljana - Faculty of Medicine	University of Ljubljana - Faculty of Medicine	Jevsnik Virant,M., Lustrek,M. and Petrovec,M.
EPI_ISL_17253616, EPI_ISL_17253618, EPI_ISL_17253625	National Public Health Institute of Slovakia	Laboratory of Genomics and Bioinformatics, Comenius University Science Park	Szemes,Tomáš;Kaliňáková,Anna;Kotvasová,Barbora;Ševčíková,Lucia;Vrabľová,Terézia;Sedláčková,Tatiana;Rusňáková,Diana;Böhmer,Miroslav;Budiš,Jaroslav;Styk,Jakub;Lipková,Nikola;Forgáčová,Michaela;Bokorová,Silvia;Mišenko,Pavol
EPI_ISL_17258467, EPI_ISL_17258527, EPI_ISL_17258528, EPI_ISL_17258529, EPI_ISL_17258530, EPI_ISL_17258531, EPI_ISL_17258532, EPI_ISL_17258533, EPI_ISL_17258534, EPI_ISL_17258535, EPI_ISL_17258536, EPI_ISL_17258537, EPI_ISL_17258538			
see above	Respiratory Virus Unit / Reference Microbiology Services / UK Health Security Agency	Reference Microbiology Services / UK Health Security Agency	Zambon M. Talts T. Mosscrop L. Miah S
EPI_ISL_17276529, EPI_ISL_17276530, EPI_ISL_17276531, EPI_ISL_17276538, EPI_ISL_17276541, EPI_ISL_17276542, EPI_ISL_17276546, EPI_ISL_17276548, EPI_ISL_17276553, EPI_ISL_17276555, EPI_ISL_17276556, EPI_ISL_17276558, EPI_ISL_17276564, EPI_ISL_17276566, EPI_ISL_17276567, EPI_ISL_17276568, EPI_ISL_17276570, EPI_ISL_17276588, EPI_ISL_17276600, EPI_ISL_17276601, EPI_ISL_17276602, EPI_ISL_17276603, EPI_ISL_17276604, EPI_ISL_17276605, EPI_ISL_17276606, EPI_ISL_17276607, EPI_ISL_17276608, EPI_ISL_17276609, EPI_ISL_17276610, EPI_ISL_17276611, EPI_ISL_17276612, EPI_ISL_17276613			
see above	Respiratory Virus Unit , Reference Microbiology	Reference Microbiology Services , UK Health	Zambon M. Talts T. Mosscrop L. Miah S

	Services , UK Health Security Agency	Security Agency	
EPI_ISL_17280741, EPI_ISL_17280742, EPI_ISL_17280743, EPI_ISL_17280744, EPI_ISL_17280745, EPI_ISL_17280756, EPI_ISL_17280757	Niigata University (IDRC) Infectious Diseases Research Center	Niigata University (IDRC) Infectious Diseases Research Center	Sayaka Yoshioka, Irina Chon, Reiko Saito
EPI_ISL_17287524	Respiratory Virus Unit, Reference Microbiology Services, UK Health Security Agency	Reference Microbiology Services, UK Health Security Agency	Zambon M. Talts T. Mosscrop L. Miah S
EPI_ISL_17297222, EPI_ISL_17297227, EPI_ISL_17297231, EPI_ISL_17297233, EPI_ISL_17297234, EPI_ISL_17297242, EPI_ISL_17297247, EPI_ISL_17297253	Respiratory Virus Unit / Reference Microbiology Services / UK Health Security Agency	Reference Microbiology Services / UK Health Security Agency	Zambon M. Talts T. Mosscrop L. Miah S
EPI_ISL_17367998, EPI_ISL_17367999	IVY4 Central Lab, Vanderbilt University Medical Center	Lauring Lab, University of Michigan, Department of Microbiology and Immunology	Gilbert
EPI_ISL_17368000	IVY5 Central Lab, Vanderbilt University Medical Center	Lauring Lab, University of Michigan, Department of Microbiology and Immunology	Gilbert
EPI_ISL_17368001, EPI_ISL_17368002	IVY4 Central Lab, Vanderbilt University Medical Center	Lauring Lab, University of Michigan, Department of Microbiology and Immunology	Gilbert
EPI_ISL_17368003, EPI_ISL_17368004	IVY5 Central Lab, Vanderbilt University Medical Center	Lauring Lab, University of Michigan, Department of Microbiology and Immunology	Gilbert
EPI_ISL_17368005, EPI_ISL_17368006, EPI_ISL_17368007, EPI_ISL_17368008, EPI_ISL_17368009, EPI_ISL_17368010, EPI_ISL_17368011, EPI_ISL_17368012, EPI_ISL_17368013, EPI_ISL_17368014, EPI_ISL_17368015, EPI_ISL_17368016, EPI_ISL_17368017, EPI_ISL_17368018, EPI_ISL_17368019, EPI_ISL_17368020, EPI_ISL_17368021, EPI_ISL_17368022, EPI_ISL_17368023, EPI_ISL_17368024, EPI_ISL_17368025, EPI_ISL_17368026, EPI_ISL_17368027, EPI_ISL_17368028, EPI_ISL_17368029, EPI_ISL_17368030, EPI_ISL_17368031, EPI_ISL_17368032, EPI_ISL_17368033, EPI_ISL_17368034, EPI_ISL_17368035, EPI_ISL_17368036			
see above	IVY4 Central Lab, Vanderbilt University Medical Center	Lauring Lab, University of Michigan, Department of Microbiology and Immunology	Gilbert
EPI_ISL_17368037, EPI_ISL_17368038, EPI_ISL_17368039, EPI_ISL_17368040	Edendale	National Institute for Communicable Diseases of the National Health Laboratory Service	Everatt J, Kekana D, Mahlangu B, Stock N, Ntozini B, Ntuli N, Mnguni A, Nzimande A, Ismail A, Bhiman JN, Wolter N
EPI_ISL_17368041, EPI_ISL_17368042	Klerksdorp	National Institute for Communicable Diseases of the National Health Laboratory Service	Everatt J, Kekana D, Mahlangu B, Stock N, Ntozini B, Ntuli N, Mnguni A, Nzimande A, Ismail A, Bhiman JN, Wolter N
EPI_ISL_17368043, EPI_ISL_17368044	Mapulaneng	National Institute for Communicable Diseases of the National Health Laboratory Service	Everatt J, Kekana D, Mahlangu B, Stock N, Ntozini B, Ntuli N, Mnguni A, Nzimande A, Ismail A, Bhiman JN, Wolter N
EPI_ISL_17394605, EPI_ISL_17394606, EPI_ISL_17394607, EPI_ISL_17394608, EPI_ISL_17394609, EPI_ISL_17394610, EPI_ISL_17394611, EPI_ISL_17394612, EPI_ISL_17394613, EPI_ISL_17394614, EPI_ISL_17394615, EPI_ISL_17394616, EPI_ISL_17394617, EPI_ISL_17394618, EPI_ISL_17394619, EPI_ISL_17394620, EPI_ISL_17394621, EPI_ISL_17394622, EPI_ISL_17394623, EPI_ISL_17394624, EPI_ISL_17394625, EPI_ISL_17394626, EPI_ISL_17394627, EPI_ISL_17394628, EPI_ISL_17394629, EPI_ISL_17394630, EPI_ISL_17394631, EPI_ISL_17394632, EPI_ISL_17394633, EPI_ISL_17394634, EPI_ISL_17394635, EPI_ISL_17394636, EPI_ISL_17394637, EPI_ISL_17394638, EPI_ISL_17394639, EPI_ISL_17394640, EPI_ISL_17394641			
see above	IVY5 Central Lab, Vanderbilt University Medical Center	Lauring Lab, University of Michigan, Department of Microbiology and Immunology	Gilbert
EPI_ISL_17401569, EPI_ISL_17401570, EPI_ISL_17401571, EPI_ISL_17401572, EPI_ISL_17401573, EPI_ISL_17401574, EPI_ISL_17401575, EPI_ISL_17401578, EPI_ISL_17401579, EPI_ISL_17401580, EPI_ISL_17401581, EPI_ISL_17401583, EPI_ISL_17401584			
see above	Hainan Medical University, Hainan Medical University-The University of Hong Kong Joint Laboratory of Tropical Infectious Diseases	Hainan Medical University, Hainan Medical University-The University of Hong Kong Joint Laboratory of Tropical Infectious Diseases	Jia,Y. and Yin,F.
EPI_ISL_17402456	Willis-Knighton Medical Center Hospital Laboratory	LSUHS Emerging Viral Threat Laboratory	Lorie M. Atkins, April N. Johnson, Ricardo Costa, Nellie Perez, Mariana Alvarado, Alexander Mijalis, Jennifer L. Carroll, Rona S. Scott, Andrew D. Yurochko, Christopher G. Kevil, John A. Vanchiere, Krista Queen, Joseph A. Bocchini, Jarrod Sawyer
EPI_ISL_17481634, EPI_ISL_17481635, EPI_ISL_17481643, EPI_ISL_17481644, EPI_ISL_17481646, EPI_ISL_17481661	National Public Health Institute of Slovakia	Laboratory of Genomics and Bioinformatics, Comenius University Science Park	Szemes,Tomas; Kalinakova,Anna; Kotvasova,Barbora; Sevcikova,Lucia; Vrablova,Terezia; Sedlackova,Tatiana; Rusnakova,Diana; Bohmer,Miroslav; Budis,Jaroslav; Styk,Jakub; Lipkova,Nikola; Forgacova,Michaela; Bokorova,Silvia; Misenko,Pavol
EPI_ISL_17559305, EPI_ISL_17559306, EPI_ISL_17559307, EPI_ISL_17559308, EPI_ISL_17559309, EPI_ISL_17559310, EPI_ISL_17559311, EPI_ISL_17559312, EPI_ISL_17559313	IVY5 Central Lab, Vanderbilt University Medical Center	Lauring Lab, University of Michigan, Department of Microbiology and Immunology	Gilbert
EPI_ISL_17559319	Red Cross	National Institute for Communicable Diseases of the National Health Laboratory Service	Everatt J, Kekana D, Mahlangu B, Stock N, Ntozini B, Ntuli N, Mnguni A, Nzimande A, Ismail A, Bhiman JN, Wolter N
EPI_ISL_17559321	East ridge Clinic	National Institute for Communicable Diseases of the National Health Laboratory Service	Everatt J, Kekana D, Mahlangu B, Stock N, Ntozini B, Ntuli N, Mnguni A, Nzimande A, Ismail A, Bhiman JN, Wolter N
EPI_ISL_17559323	Edendale	National Institute for Communicable Diseases of the National Health Laboratory Service	Everatt J, Kekana D, Mahlangu B, Stock N, Ntozini B, Ntuli N, Mnguni A, Nzimande A, Ismail A, Bhiman JN, Wolter N
EPI_ISL_17559324, EPI_ISL_17559327	Rahima Moosa	National Institute for Communicable Diseases of the National Health Laboratory Service	Everatt J, Kekana D, Mahlangu B, Stock N, Ntozini B, Ntuli N, Mnguni A, Nzimande A, Ismail A, Bhiman JN, Wolter N
EPI_ISL_17559328, EPI_ISL_17559329	Red Cross	National Institute for Communicable Diseases of the National Health Laboratory Service	Everatt J, Kekana D, Mahlangu B, Stock N, Ntozini B, Ntuli N, Mnguni A, Nzimande A, Ismail A, Bhiman JN, Wolter N
EPI_ISL_17559330, EPI_ISL_17559332, EPI_ISL_17559333, EPI_ISL_17559334	Rahima Moosa	National Institute for Communicable Diseases of the National Health Laboratory Service	Everatt J, Kekana D, Mahlangu B, Stock N, Ntozini B, Ntuli N, Mnguni A, Nzimande A, Ismail A, Bhiman JN, Wolter N
EPI_ISL_17559337	East ridge Clinic	National Institute for Communicable Diseases of the National Health Laboratory Service	Everatt J, Kekana D, Mahlangu B, Stock N, Ntozini B, Ntuli N, Mnguni A, Nzimande A, Ismail A, Bhiman JN, Wolter N
EPI_ISL_17559339, EPI_ISL_17559340, EPI_ISL_17559341, EPI_ISL_17559342, EPI_ISL_17559343	Mitchells Plain	National Institute for Communicable Diseases of the National Health Laboratory Service	Everatt J, Kekana D, Mahlangu B, Stock N, Ntozini B, Ntuli N, Mnguni A, Nzimande A, Ismail A, Bhiman JN, Wolter N
EPI_ISL_17559344, EPI_ISL_17559345, EPI_ISL_17559346, EPI_ISL_17559347	Red Cross	National Institute for Communicable Diseases of the National Health Laboratory Service	Everatt J, Kekana D, Mahlangu B, Stock N, Ntozini B, Ntuli N, Mnguni A, Nzimande A, Ismail A, Bhiman JN, Wolter N
EPI_ISL_17559349	Rahima Moosa	National Institute for Communicable Diseases of the National Health Laboratory Service	Everatt J, Kekana D, Mahlangu B, Stock N, Ntozini B, Ntuli N, Mnguni A, Nzimande A, Ismail A, Bhiman JN, Wolter N
EPI_ISL_17559351	Red Cross	National Institute for Communicable Diseases of the National Health Laboratory Service	Everatt J, Kekana D, Mahlangu B, Stock N, Ntozini B, Ntuli N, Mnguni A, Nzimande A, Ismail A, Bhiman JN, Wolter N
EPI_ISL_17559352, EPI_ISL_17559354, EPI_ISL_17559355, EPI_ISL_17559363, EPI_ISL_17559364	Rahima Moosa	National Institute for Communicable Diseases of the National Health Laboratory Service	Everatt J, Kekana D, Mahlangu B, Stock N, Ntozini B, Ntuli N, Mnguni A, Nzimande A, Ismail A, Bhiman JN, Wolter N
EPI_ISL_17559366, EPI_ISL_17559367	Red Cross	National Institute for Communicable Diseases of the National Health Laboratory Service	Everatt J, Kekana D, Mahlangu B, Stock N, Ntozini B, Ntuli N, Mnguni A, Nzimande A, Ismail A, Bhiman JN, Wolter N
EPI_ISL_17559368	Mitchells Plain	National Institute for Communicable Diseases of the National Health Laboratory Service	Everatt J, Kekana D, Mahlangu B, Stock N, Ntozini B, Ntuli N, Mnguni A, Nzimande A, Ismail A, Bhiman JN, Wolter N
EPI_ISL_17559369	Red Cross	National Institute for Communicable Diseases of the National Health Laboratory Service	Everatt J, Kekana D, Mahlangu B, Stock N, Ntozini B, Ntuli N, Mnguni A, Nzimande A, Ismail A, Bhiman JN, Wolter N
EPI_ISL_17559370, EPI_ISL_17559371	Rahima Moosa	National Institute for Communicable Diseases of the National Health Laboratory Service	Everatt J, Kekana D, Mahlangu B, Stock N, Ntozini B, Ntuli N, Mnguni A, Nzimande A, Ismail A, Bhiman JN, Wolter N
EPI_ISL_17559372	Mitchells Plain	National Institute for Communicable Diseases of the National Health Laboratory Service	Everatt J, Kekana D, Mahlangu B, Stock N, Ntozini B, Ntuli N, Mnguni A, Nzimande A, Ismail A, Bhiman JN, Wolter N
EPI_ISL_17559373	East ridge Clinic	National Institute for Communicable Diseases of the National Health Laboratory Service	Everatt J, Kekana D, Mahlangu B, Stock N, Ntozini B, Ntuli N, Mnguni A, Nzimande A, Ismail A, Bhiman JN, Wolter N
EPI_ISL_17559374, EPI_ISL_17559375	Mitchells Plain	National Institute for Communicable Diseases of the National Health Laboratory Service	Everatt J, Kekana D, Mahlangu B, Stock N, Ntozini B, Ntuli N, Mnguni A, Nzimande A, Ismail A, Bhiman JN, Wolter N
EPI_ISL_17559376	Red Cross	National Institute for Communicable Diseases of the National Health Laboratory Service	Everatt J, Kekana D, Mahlangu B, Stock N, Ntozini B, Ntuli N, Mnguni A, Nzimande A, Ismail A, Bhiman JN, Wolter N
EPI_ISL_17559377	Rahima Moosa	National Institute for Communicable Diseases of the National Health Laboratory Service	Everatt J, Kekana D, Mahlangu B, Stock N, Ntozini B, Ntuli N, Mnguni A, Nzimande A, Ismail A, Bhiman JN, Wolter N
EPI_ISL_17559378	Red Cross	National Institute for Communicable Diseases of the National Health Laboratory Service	Everatt J, Kekana D, Mahlangu B, Stock N, Ntozini B, Ntuli N, Mnguni A, Nzimande A, Ismail A, Bhiman JN, Wolter N
EPI_ISL_17559379	Rahima Moosa	National Institute for Communicable Diseases of the National Health Laboratory Service	Everatt J, Kekana D, Mahlangu B, Stock N, Ntozini B, Ntuli N, Mnguni A, Nzimande A, Ismail A, Bhiman JN, Wolter N
EPI_ISL_17559380	Edendale Gateway Clinic	National Institute for Communicable Diseases of the National Health Laboratory Service	Everatt J, Kekana D, Mahlangu B, Stock N, Ntozini B, Ntuli N, Mnguni A, Nzimande A, Ismail A, Bhiman JN, Wolter N

EPI_ISL_17559385	Red Cross	National Institute for Communicable Diseases of the National Health Laboratory Service	Everatt J, Kekana D, Mahlangu B, Stock N, Ntozini B, Ntuli N, Mnguni A, Nzimande A, Ismail A, Bhiman JN, Wolter N
EPI_ISL_17559387, EPI_ISL_17559388, EPI_ISL_17559389, EPI_ISL_17559390, EPI_ISL_17559391, EPI_ISL_17559393, EPI_ISL_17559395	Rahima Moosa	National Institute for Communicable Diseases of the National Health Laboratory Service	Everatt J, Kekana D, Mahlangu B, Stock N, Ntozini B, Ntuli N, Mnguni A, Nzimande A, Ismail A, Bhiman JN, Wolter N
EPI_ISL_17559397, EPI_ISL_17559399	East ridge Clinic	National Institute for Communicable Diseases of the National Health Laboratory Service	Everatt J, Kekana D, Mahlangu B, Stock N, Ntozini B, Ntuli N, Mnguni A, Nzimande A, Ismail A, Bhiman JN, Wolter N
EPI_ISL_17559401	Red Cross	National Institute for Communicable Diseases of the National Health Laboratory Service	Everatt J, Kekana D, Mahlangu B, Stock N, Ntozini B, Ntuli N, Mnguni A, Nzimande A, Ismail A, Bhiman JN, Wolter N
EPI_ISL_17559402, EPI_ISL_17559403	Tintswalo	National Institute for Communicable Diseases of the National Health Laboratory Service	Everatt J, Kekana D, Mahlangu B, Stock N, Ntozini B, Ntuli N, Mnguni A, Nzimande A, Ismail A, Bhiman JN, Wolter N
EPI_ISL_17583025, EPI_ISL_17583026, EPI_ISL_17583027, EPI_ISL_17583028, EPI_ISL_17583029, EPI_ISL_17583030, EPI_ISL_17583663, EPI_ISL_17583664, EPI_ISL_17583665, EPI_ISL_17583666, EPI_ISL_17583667, EPI_ISL_17583668, EPI_ISL_17583669, EPI_ISL_17583670			
see above	West of Scotland Specialist Virology Centre	West of Scotland Specialist Virology Centre	Lynne Ferguson, Imogen Johnston-Menzies, Rory Gunson
EPI_ISL_17597699, EPI_ISL_17597700, EPI_ISL_17597701, EPI_ISL_17597703, EPI_ISL_17597706, EPI_ISL_17597713, EPI_ISL_17597718	IVY4 Central Lab, Vanderbilt University Medical Center	Lauring Lab, University of Michigan, Department of Microbiology and Immunology	Gilbert
EPI_ISL_17597725, EPI_ISL_17597731, EPI_ISL_17597746, EPI_ISL_17597757, EPI_ISL_17597759, EPI_ISL_17597778	IVY5 Central Lab, Vanderbilt University Medical Center	Lauring Lab, University of Michigan, Department of Microbiology and Immunology	Gilbert
EPI_ISL_17694305, EPI_ISL_17694306, EPI_ISL_17694307, EPI_ISL_17694308, EPI_ISL_17694309, EPI_ISL_17694310, EPI_ISL_17694311, EPI_ISL_17694312, EPI_ISL_17694313, EPI_ISL_17694314, EPI_ISL_17694315, EPI_ISL_17694316, EPI_ISL_17694317			
see above	Division Virologia, Laboratorio de Salud Publica, Tucuman	INEI-ANLIS "Dr. Carlos G. Malbran" Servicio de Virosis Respiratorias	Elsa Baumeister, Andrea Pontoriero, Russo Mara, Martin Avaro, Estefania Benedetti, Maria Elena Dattero, Chamorro Ariana, Garcia Valdez Jackeline, Erika Machicado Macias, Fabian Pardon.
EPI_ISL_17694318	Hospital "Prof. Dr. Juan P. Garrahan"	INEI-ANLIS "Dr. Carlos G. Malbran" Servicio de Virosis Respiratorias	Elsa Baumeister, Andrea Pontoriero, Russo Mara, Martin Avaro, Estefania Benedetti, Maria Elena Dattero, Chamorro Ariana, Garcia Valdez Jackeline, Erika Machicado Macias, Fabian Pardon.
EPI_ISL_17694325, EPI_ISL_17694326, EPI_ISL_17694332, EPI_ISL_17694336, EPI_ISL_17694337	Respiratory Virus Unit / Reference Microbiology Services / UK Health Security Agency	Reference Microbiology Services / UK Health Security Agency	Zambon M. Talts T. Mosscrop L. Miah S
EPI_ISL_17773648, EPI_ISL_17773649, EPI_ISL_17773650, EPI_ISL_17773652, EPI_ISL_17773653, EPI_ISL_17773654, EPI_ISL_17773655, EPI_ISL_17773656, EPI_ISL_17773657, EPI_ISL_17773658, EPI_ISL_17773660, EPI_ISL_17773661			
see above	Laboratorio Central de Saude Publica do Distrito Federal (LACEN/DF)	Laboratory of Respiratory Viruses and Measles, Oswaldo Cruz Institute, FIOCRUZ	Paola Resende, Fernando Motta, Elisa Cavalcante Pereira, Leticia Ferreira Lima, Bruna Mendonça da Silva, Jéssica Graça Macedo de Carvalho, Larissa Macedo Pinto, Victor Guimaraes, Leticia Scalioni, Rodrigo Ribeiro Rodrigues, Marilda Siqueira on behalf of the Fiocruz COVID-19 Genomic Surveillance Network
EPI_ISL_17773665	Universidade Federal de São Paulo, Departamento de Medicina, Disciplina de Doenças Infecciosas e Parasitárias. Laboratório de Virologia	Laboratory of Respiratory Viruses and Measles, Oswaldo Cruz Institute, FIOCRUZ	Paola Resende, Fernando Motta, Elisa Cavalcante Pereira, Leticia Ferreira Lima, Bruna Mendonça da Silva, Jéssica Graça Macedo de Carvalho, Larissa Macedo Pinto, Victor Guimaraes, Leticia Scalioni, Rodrigo Ribeiro Rodrigues, Nancy Bellei, Marilda Siqueira on behalf of the Fiocruz COVID-19 Genomic Surveillance Network
EPI_ISL_17778166, EPI_ISL_17778298, EPI_ISL_17778391	Laboratorio Central de Saude Publica do Distrito Federal (LACEN/DF)	Instituto Oswaldo Cruz FIOCRUZ - Laboratory of Respiratory Viruses and Measles (LVRS)	Paola Resende, Fernando Motta, Elisa Cavalcante Pereira, Leticia Ferreira Lima, Bruna Mendonça da Silva, Jéssica Graça Macedo de Carvalho, Larissa Macedo Pinto, Victor Guimaraes, Leticia Scalioni, Rodrigo Ribeiro Rodrigues, Marilda Siqueira on behalf of the Fiocruz COVID-19 Genomic Surveillance Network
EPI_ISL_17797313, EPI_ISL_17797315, EPI_ISL_17797316, EPI_ISL_17797317, EPI_ISL_17797318, EPI_ISL_17797319, EPI_ISL_17797323, EPI_ISL_17797324, EPI_ISL_17797325, EPI_ISL_17797328	Centre Pasteur in Cameroon, National Reference Laboratory for Chikungunya and Dengue	Centre Pasteur in Cameroon, National Reference Laboratory for Chikungunya and Dengue	Moumbeket Yifomnjou,M.H. and Njouom,R.
EPI_ISL_17808729, EPI_ISL_17808730	Central public health laboratory of the state of Amapa	Evandro Chagas Institute, Laboratory of Respiratory Viruses, National InfluenzaCenter	Mirleide Santos; Fernando Tavares; Edivaldo Junior; Luana Barbagelata; Amanda Mendes; Wanderley Dias; Delana Melo; Agata Monique; Edvaldo Penha;
EPI_ISL_17808737, EPI_ISL_17808739, EPI_ISL_17808740, EPI_ISL_17808741, EPI_ISL_17808742, EPI_ISL_17808743, EPI_ISL_17808744, EPI_ISL_17808745, EPI_ISL_17808746, EPI_ISL_17808747, EPI_ISL_17808748, EPI_ISL_17808749, EPI_ISL_17808750, EPI_ISL_17808751, EPI_ISL_17808752, EPI_ISL_17808753, EPI_ISL_17808754, EPI_ISL_17808755, EPI_ISL_17808756, EPI_ISL_17808757, EPI_ISL_17808758, EPI_ISL_17808759, EPI_ISL_17808760, EPI_ISL_17808761, EPI_ISL_17808762, EPI_ISL_17808763, EPI_ISL_17808764, EPI_ISL_17808765, EPI_ISL_17808766, EPI_ISL_17808767, EPI_ISL_17808768, EPI_ISL_17808769, EPI_ISL_17808770, EPI_ISL_17808771, EPI_ISL_17808773, EPI_ISL_17808774, EPI_ISL_17808775, EPI_ISL_17808776, EPI_ISL_17808777, EPI_ISL_17808778, EPI_ISL_17808779, EPI_ISL_17808780, EPI_ISL_17808781, EPI_ISL_17808782, EPI_ISL_17808783, EPI_ISL_17808784, EPI_ISL_17808785, EPI_ISL_17808786, EPI_ISL_17808787, EPI_ISL_17808788, EPI_ISL_17808789, EPI_ISL_17808790, EPI_ISL_17808792, EPI_ISL_17808793, EPI_ISL_17808794, EPI_ISL_17808795, EPI_ISL_17808797, EPI_ISL_17808798, EPI_ISL_17808799, EPI_ISL_17808800, EPI_ISL_17808802, EPI_ISL_17808803, EPI_ISL_17808804, EPI_ISL_17808805, EPI_ISL_17808806, EPI_ISL_17808807, EPI_ISL_17808808, EPI_ISL_17808809, EPI_ISL_17808810, EPI_ISL_17808811, EPI_ISL_17808812, EPI_ISL_17808814, EPI_ISL_17808815, EPI_ISL_17808816, EPI_ISL_17808817, EPI_ISL_17808819, EPI_ISL_17808820, EPI_ISL_17808822, EPI_ISL_17808824, EPI_ISL_17808826, EPI_ISL_17808827, EPI_ISL_17808828, EPI_ISL_17808831, EPI_ISL_17808832, EPI_ISL_17808834, EPI_ISL_17808835, EPI_ISL_17808836, EPI_ISL_17808837			
see above	Valley Wise Health	Arizona State University	Holland, LaRinda A.; Holland, Steven C.; Smith, Matthew F.; Leonard, Victoria R.; Murugan, Vel; Nordstrom, Lora; Mulrow, Mary; Salgado, Raquel; White, Michael; Lim, Efrem S.
EPI_ISL_17950088, EPI_ISL_17950089, EPI_ISL_17950091, EPI_ISL_17950094, EPI_ISL_17950095, EPI_ISL_17950097, EPI_ISL_17950098, EPI_ISL_17950099, EPI_ISL_17950100, EPI_ISL_17950101, EPI_ISL_17950102, EPI_ISL_17950103, EPI_ISL_17950105, EPI_ISL_17950107, EPI_ISL_17950108, EPI_ISL_17950110, EPI_ISL_17950111, EPI_ISL_17950112, EPI_ISL_17950114, EPI_ISL_17950115, EPI_ISL_17950116, EPI_ISL_17950117, EPI_ISL_17950118, EPI_ISL_17950119, EPI_ISL_17950120, EPI_ISL_17950122, EPI_ISL_17950124, EPI_ISL_17950125, EPI_ISL_17950126, EPI_ISL_17950127, EPI_ISL_17950129, EPI_ISL_17950130, EPI_ISL_17950131, EPI_ISL_17950132, EPI_ISL_17950133, EPI_ISL_17950134, EPI_ISL_17950135			
see above	The First Affiliated Hospital of Bengbu Medical College	The First Affiliated Hospital of Bengbu Medical College	Huang,L.
EPI_ISL_17950137, EPI_ISL_17950138, EPI_ISL_17950139, EPI_ISL_17950140, EPI_ISL_17950141, EPI_ISL_17950142, EPI_ISL_17950143, EPI_ISL_17950144, EPI_ISL_17950145, EPI_ISL_17950146, EPI_ISL_17950147, EPI_ISL_17950148, EPI_ISL_17950149, EPI_ISL_17950151, EPI_ISL_17950152, EPI_ISL_17950153, EPI_ISL_17950154, EPI_ISL_17950155, EPI_ISL_17950156, EPI_ISL_17950157, EPI_ISL_17950158, EPI_ISL_17950159, EPI_ISL_17950163, EPI_ISL_17950164, EPI_ISL_17950165, EPI_ISL_17950166, EPI_ISL_17950167, EPI_ISL_17950171, EPI_ISL_17950172, EPI_ISL_17950173, EPI_ISL_17950174, EPI_ISL_17950175, EPI_ISL_17950176, EPI_ISL_17950177, EPI_ISL_17950178, EPI_ISL_17950179, EPI_ISL_17950180, EPI_ISL_17950183, EPI_ISL_17950186, EPI_ISL_17950188, EPI_ISL_17950189, EPI_ISL_17950191, EPI_ISL_17950192, EPI_ISL_17950193, EPI_ISL_17950194, EPI_ISL_17950196, EPI_ISL_17950197, EPI_ISL_17950199, EPI_ISL_17950202, EPI_ISL_17950203, EPI_ISL_17950204, EPI_ISL_17950205, EPI_ISL_17950206, EPI_ISL_17950208, EPI_ISL_17950209, EPI_ISL_17950211, EPI_ISL_17950212, EPI_ISL_17950213, EPI_ISL_17950214, EPI_ISL_17950216, EPI_ISL_17950217, EPI_ISL_17950219, EPI_ISL_17950220, EPI_ISL_17950221, EPI_ISL_17950222, EPI_ISL_17950224, EPI_ISL_17950225, EPI_ISL_17950226, EPI_ISL_17950228, EPI_ISL_17950229, EPI_ISL_17950230, EPI_ISL_17950232, EPI_ISL_17950233, EPI_ISL_17950234, EPI_ISL_17950235, EPI_ISL_17950237, EPI_ISL_17950238, EPI_ISL_17950239, EPI_ISL_17950240, EPI_ISL_17950242, EPI_ISL_17950243, EPI_ISL_17950245, EPI_ISL_17950246, EPI_ISL_17950247, EPI_ISL_17950249, EPI_ISL_17950250, EPI_ISL_17950251, EPI_ISL_17950252			
see above	Arizona State University	Arizona State University	Holland,S.C., Holland,L.A., Smith,M.F., Leonard,V.R., Murugan,V., Nordstrom,L., Mulrow,M., Salgado,R., White,M. and Lim,E.S.
EPI_ISL_17950996, EPI_ISL_17950997, EPI_ISL_17950998, EPI_ISL_17950999, EPI_ISL_17951000, EPI_ISL_17951001, EPI_ISL_17951002, EPI_ISL_17951003, EPI_ISL_17951004, EPI_ISL_17951005, EPI_ISL_17951006, EPI_ISL_17951007, EPI_ISL_17951008, EPI_ISL_17951009, EPI_ISL_17951010, EPI_ISL_17951011, EPI_ISL_17951012, EPI_ISL_17951013, EPI_ISL_17951014, EPI_ISL_17951015, EPI_ISL_17951016, EPI_ISL_17951017, EPI_ISL_17951018, EPI_ISL_17951019, EPI_ISL_17951020, EPI_ISL_17951021, EPI_ISL_17951022, EPI_ISL_17951023, EPI_ISL_17951024, EPI_ISL_17951025, EPI_ISL_17951026, EPI_ISL_17951027, EPI_ISL_17951028, EPI_ISL_17951029, EPI_ISL_17951030, EPI_ISL_17951031, EPI_ISL_17951032, EPI_ISL_17951033, EPI_ISL_17951034, EPI_ISL_17951035, EPI_ISL_17951036, EPI_ISL_17951037, EPI_ISL_17951038, EPI_ISL_17951039, EPI_ISL_17951040, EPI_ISL_17951041, EPI_ISL_17951042, EPI_ISL_17951043, EPI_ISL_17951044, EPI_ISL_17951045, EPI_ISL_17951046			
see above	Molecular Biology Laboratory, Pedro de Elizalde Hospital	Virology Laboratory, Ricardo Gutiérrez Children's Hospital	Acuña, Dolores; Goya, Stephanie; Nabaes Jodar, Mercedes S.; Montoto, L; Wenk, G; Sevilla, ME; Miño, L; Valeri, C; Bokser, V; Mistchenko, Alicia S.; Viegas, Mariana
EPI_ISL_17953807, EPI_ISL_17953808, EPI_ISL_17953809, EPI_ISL_17953810, EPI_ISL_17953811, EPI_ISL_17953812, EPI_ISL_17953813, EPI_ISL_17953814, EPI_ISL_17953815, EPI_ISL_17953817, EPI_ISL_17953818, EPI_ISL_17953820, EPI_ISL_17953822, EPI_ISL_17953823, EPI_ISL_17953824, EPI_ISL_17953825, EPI_ISL_17953826, EPI_ISL_17953827, EPI_ISL_17953828, EPI_ISL_17953829, EPI_ISL_17953830, EPI_ISL_17953832, EPI_ISL_17953834, EPI_ISL_17953835, EPI_ISL_17953836, EPI_ISL_17953837, EPI_ISL_17953838, EPI_ISL_17953839, EPI_ISL_17953840, EPI_ISL_17953841, EPI_ISL_17953842, EPI_ISL_17953843, EPI_ISL_17953844, EPI_ISL_17953845, EPI_ISL_17953846, EPI_ISL_17953847, EPI_ISL_17953849, EPI_ISL_17953850, EPI_ISL_17953851, EPI_ISL_17953852, EPI_ISL_17953853, EPI_ISL_17953854, EPI_ISL_17953855, EPI_ISL_17953856, EPI_ISL_17953857, EPI_ISL_17953858, EPI_ISL_17953859, EPI_ISL_17953860, EPI_ISL_17953861, EPI_ISL_17953862, EPI_ISL_17953863, EPI_ISL_17953864, EPI_ISL_17953865, EPI_ISL_17953866, EPI_ISL_17953867, EPI_ISL_17953868, EPI_ISL_17953870, EPI_ISL_17953871, EPI_ISL_17953872, EPI_ISL_17953873, EPI_ISL_17953874, EPI_ISL_17953875, EPI_ISL_17953876, EPI_ISL_17953877, EPI_ISL_17953878, EPI_ISL_17953880, EPI_ISL_17953881, EPI_ISL_17953882, EPI_ISL_17953883, EPI_ISL_17953884, EPI_ISL_17953885, EPI_ISL_17953886, EPI_ISL_17953887, EPI_ISL_17953889, EPI_ISL_17953890, EPI_ISL_17953891, EPI_ISL_17953892, EPI_ISL_17953893, EPI_ISL_17953894, EPI_ISL_17953895, EPI_ISL_17953896, EPI_ISL_17953897, EPI_ISL_17953899, EPI_ISL_17953901, EPI_ISL_17953902, EPI_ISL_17953903, EPI_ISL_17953904, EPI_ISL_17953905, EPI_ISL_17953906, EPI_ISL_17953907, EPI_ISL_17953908, EPI_ISL_17953909, EPI_ISL_17953910, EPI_ISL_17953911, EPI_ISL_17953912, EPI_ISL_17953913, EPI_ISL_17953914, EPI_ISL_17953915, EPI_ISL_17953916, EPI_ISL_17953917, EPI_ISL_17953918, EPI_ISL_17953919, EPI_ISL_17953920, EPI_ISL_17953921			
see above	State Key Laboratory for Diagnosis and Treatment of Infectious Diseases, First Affiliated Hospital, School of Medicine, Zhejiang University	State Key Laboratory for Diagnosis and Treatment of Infectious Diseases, First Affiliated Hospital, School of Medicine, Zhejiang University	Guo,Y.
EPI_ISL_17975551, EPI_ISL_17975552, EPI_ISL_17975557, EPI_ISL_17975564, EPI_ISL_17975568, EPI_ISL_17975572, EPI_ISL_17975581, EPI_ISL_17975582, EPI_ISL_17975583, EPI_ISL_17975586, EPI_ISL_17975589, EPI_ISL_17975595, EPI_ISL_17975598, EPI_ISL_17975602, EPI_ISL_17975607, EPI_ISL_17975610, EPI_ISL_17975613, EPI_ISL_17975617, EPI_ISL_17975623, EPI_ISL_17975625, EPI_ISL_17975626, EPI_ISL_17975629, EPI_ISL_17975631, EPI_ISL_17975633, EPI_ISL_17975642			
see above	Sapienza University	Sapienza University	Pierangeli,A.
EPI_ISL_17992452, EPI_ISL_17992454, EPI_ISL_17992456, EPI_ISL_17992458	The South African Red Cross Society - Western Cape Provincial Office	National Institute for Communicable Diseases of the National Health Laboratory Service	Everatt J, Kekana D, Mahlangu B, Stock N, Ntozini B, Ntuli N, Mnguni A, Nzimande A, Ismail A, Bhiman JN, Wolter N
EPI_ISL_17992460, EPI_ISL_17992462, EPI_ISL_17992464	Rahima Moosa Mother and Child Hospital	National Institute for Communicable Diseases of the National Health Laboratory Service	Everatt J, Kekana D, Mahlangu B, Stock N, Ntozini B, Ntuli N, Mnguni A, Nzimande A, Ismail A, Bhiman JN, Wolter N
EPI_ISL_17992468	The South African Red Cross Society - Western Cape Provincial Office	National Institute for Communicable Diseases of the National Health Laboratory Service	Everatt J, Kekana D, Mahlangu B, Stock N, Ntozini B, Ntuli N, Mnguni A, Nzimande A, Ismail A, Bhiman JN, Wolter N
EPI_ISL_17992470, EPI_ISL_17992473	Mitchells Plain Hospital	National Institute for Communicable Diseases of the National Health Laboratory Service	Everatt J, Kekana D, Mahlangu B, Stock N, Ntozini B, Ntuli N, Mnguni A, Nzimande A, Ismail A, Bhiman JN, Wolter N
EPI_ISL_17992475, EPI_ISL_17992477, EPI_ISL_17992479, EPI_ISL_17992481, EPI_ISL_17992483, EPI_ISL_17992485	The South African Red Cross Society - Western Cape Provincial Office	National Institute for Communicable Diseases of the National Health Laboratory Service	Everatt J, Kekana D, Mahlangu B, Stock N, Ntozini B, Ntuli N, Mnguni A, Nzimande A, Ismail A, Bhiman JN, Wolter N
EPI_ISL_17992487	Mitchells Plain Hospital	National Institute for Communicable Diseases of the National Health Laboratory Service	Everatt J, Kekana D, Mahlangu B, Stock N, Ntozini B, Ntuli N, Mnguni A, Nzimande A, Ismail A, Bhiman JN, Wolter N
EPI_ISL_17992489, EPI_ISL_17992492, EPI_ISL_17992494	Rahima Moosa Mother and Child Hospital	National Institute for Communicable Diseases of the National Health Laboratory Service	Everatt J, Kekana D, Mahlangu B, Stock N, Ntozini B, Ntuli N, Mnguni A, Nzimande A, Ismail A, Bhiman JN, Wolter N
EPI_ISL_17992496	Matikwana Hospital	National Institute for Communicable Diseases of the National Health Laboratory Service	Everatt J, Kekana D, Mahlangu B, Stock N, Ntozini B, Ntuli N, Mnguni A, Nzimande A, Ismail A, Bhiman JN, Wolter N
EPI_ISL_17992498	Rahima Moosa Mother and Child Hospital	National Institute for Communicable Diseases of the National Health Laboratory Service	Everatt J, Kekana D, Mahlangu B, Stock N, Ntozini B, Ntuli N, Mnguni A, Nzimande A, Ismail A, Bhiman JN, Wolter N
EPI_ISL_17995701, EPI_ISL_17995702, EPI_ISL_17995703, EPI_ISL_17995704, EPI_ISL_17995705, EPI_ISL_17995706, EPI_ISL_17995707, EPI_ISL_17995708, EPI_ISL_17995709, EPI_ISL_17995710, EPI_ISL_17995711, EPI_ISL_17995712, EPI_ISL_17995713, EPI_ISL_17995714, EPI_ISL_17995715, EPI_ISL_17995716, EPI_ISL_17995717, EPI_ISL_17995718, EPI_ISL_17995719, EPI_ISL_17995720,			

EPI_ISL_17995721, EPI_ISL_17995722, EPI_ISL_17995723, EPI_ISL_17995724, EPI_ISL_17995725, EPI_ISL_17995726, EPI_ISL_17995727, EPI_ISL_17995728, EPI_ISL_17995729, EPI_ISL_17995730, EPI_ISL_17995731, EPI_ISL_17995732, EPI_ISL_17995733, EPI_ISL_17995734, EPI_ISL_17995735, EPI_ISL_17995736, EPI_ISL_17995737, EPI_ISL_17995738, EPI_ISL_17995739, EPI_ISL_17995740, EPI_ISL_17995741, EPI_ISL_17995742, EPI_ISL_17995743, EPI_ISL_17995744, EPI_ISL_17995745, EPI_ISL_17995746, EPI_ISL_17995747, EPI_ISL_17995748, EPI_ISL_17995749, EPI_ISL_17995750, EPI_ISL_17995751, EPI_ISL_17995752, EPI_ISL_17995753, EPI_ISL_17995754, EPI_ISL_17995755, EPI_ISL_17995756, EPI_ISL_17995757, EPI_ISL_17995758, EPI_ISL_17995759, EPI_ISL_17995769, EPI_ISL_17995770, EPI_ISL_17995771, EPI_ISL_17995772, EPI_ISL_17995773, EPI_ISL_17995774, EPI_ISL_17995775, EPI_ISL_17995776, EPI_ISL_17995777, EPI_ISL_17995778			
see above	Robert Koch-Institute Nationales Referenzzentrum für Influenza	Robert Koch-Institute Nationales Referenzzentrum für Influenza	Sophie Kondgen, Janine Reiche
EPI_ISL_18042516, EPI_ISL_18042517, EPI_ISL_18042518, EPI_ISL_18042519, EPI_ISL_18042520	Research Institute for Tropical Medicine, Department of Health Compound, Virology Section	WHO Collaborating Centre for Reference and Research on Influenza	Jonjee Morin, Catherine Dacasin, Vina Lea Arguelles, Xiaomin Dong, Steven Edwards, Yi-Mo Deng, Clyde Dapat, Ian Barr
EPI_ISL_18054250, EPI_ISL_18054251, EPI_ISL_18054252, EPI_ISL_18054253, EPI_ISL_18054254, EPI_ISL_18054261, EPI_ISL_18054262, EPI_ISL_18054265, EPI_ISL_18054269, EPI_ISL_18054270, EPI_ISL_18054272, EPI_ISL_18054273			
see above	Hangzhou Center for Disease Control and Prevention, Microbiology Laboratory	Hangzhou Center for Disease Control and Prevention, Microbiology Laboratory	Chen,L.
EPI_ISL_18054275, EPI_ISL_18054276, EPI_ISL_18054277, EPI_ISL_18054278, EPI_ISL_18054281, EPI_ISL_18054285, EPI_ISL_18054286, EPI_ISL_18054287, EPI_ISL_18054288, EPI_ISL_18054290, EPI_ISL_18054292, EPI_ISL_18054295, EPI_ISL_18054296, EPI_ISL_18054297, EPI_ISL_18054299, EPI_ISL_18054300, EPI_ISL_18054302, EPI_ISL_18054303, EPI_ISL_18054306, EPI_ISL_18054307, EPI_ISL_18054308, EPI_ISL_18054309, EPI_ISL_18054310, EPI_ISL_18054312, EPI_ISL_18054313, EPI_ISL_18054314, EPI_ISL_18054315, EPI_ISL_18054316, EPI_ISL_18054317, EPI_ISL_18054318, EPI_ISL_18054319, EPI_ISL_18054320, EPI_ISL_18054321, EPI_ISL_18054325, EPI_ISL_18054326, EPI_ISL_18054330, EPI_ISL_18054331, EPI_ISL_18054332, EPI_ISL_18054333, EPI_ISL_18054334, EPI_ISL_18054336, EPI_ISL_18054337, EPI_ISL_18054338, EPI_ISL_18054339, EPI_ISL_18054341, EPI_ISL_18054343, EPI_ISL_18054345, EPI_ISL_18054349, EPI_ISL_18054350, EPI_ISL_18054351, EPI_ISL_18054353, EPI_ISL_18054358, EPI_ISL_18054361, EPI_ISL_18054362, EPI_ISL_18054363, EPI_ISL_18054367, EPI_ISL_18054368, EPI_ISL_18054369, EPI_ISL_18054370, EPI_ISL_18054371, EPI_ISL_18054372, EPI_ISL_18054374, EPI_ISL_18054376, EPI_ISL_18054377, EPI_ISL_18054379, EPI_ISL_18054380, EPI_ISL_18054381, EPI_ISL_18054382			
see above	Kenya Medical Research Institute - KEMRI Wellcome Trust Research Programme	Kenya Medical Research Institute - KEMRI Wellcome Trust Research Programme	Lambisia,A.W., Lewa,C., Mutunga,M., Okanda,D., Githinji,G., Nokes,J.D. and Agoti,C.N.
EPI_ISL_18082330, EPI_ISL_18082410, EPI_ISL_18082411, EPI_ISL_18082412, EPI_ISL_18082643	Niigata University (IDRC) Infectious Diseases Research Center	Niigata University (IDRC) Infectious Diseases Research Center	Sayaka Yoshioka, Reiko Saito
EPI_ISL_18089334	Department of Virology, National Institute of Health, Islamabad, Pakistan	Department of Virology, National Institute of Health, Islamabad, Pakistan	Massab Umair, Syed Adnan Haider, Qasim Ali, Muhammad Ammar, Zunera Jamal, and Muhammad Salman
EPI_ISL_18094390, EPI_ISL_18094391, EPI_ISL_18094392	Department of Virology, National Institute of Health, Islamabad, Pakistan	Department of Virology, National Institute of Health, Islamabad, Pakistan	Massab Umair, Syed Adnan Haider, Muhammad Ammar, Zunera Jamal, and Muhammad Salman
EPI_ISL_18110959, EPI_ISL_18110960, EPI_ISL_18110961, EPI_ISL_18110962, EPI_ISL_18110963, EPI_ISL_18110964, EPI_ISL_18110965, EPI_ISL_18110966, EPI_ISL_18110969, EPI_ISL_18110970	Queen's University Belfast	Queen's University Belfast	Danielle Allen
EPI_ISL_18110983, EPI_ISL_18110984	Regional Virus Laboratory, Belfast Health and Social Care Trust (BHSCT)	Queen's University Belfast	Danielle Allen
EPI_ISL_18143456, EPI_ISL_18143463, EPI_ISL_18143522	The Brotman Baty Institute for Precision Medicine	The Brotman Baty Institute for Precision Medicine	Frazar,C.D., Lee,J., Ryke,E., Gamboa,L., McDermot,E., Stone,J., Kolar,T., Han,P.D., Sibley,T.R., Truong,M., Reinhart,D., Wolf,C.R., Duchin,J., Boeckh,M., Englund,J.A., Lutz,B.R., Waghmare,A., Viboud,C., Starita,L.M., Chu,H.Y., Bedford,T. and Shendure,J.
EPI_ISL_18204747, EPI_ISL_18204754, EPI_ISL_18204756, EPI_ISL_18204758, EPI_ISL_18204760, EPI_ISL_18204763, EPI_ISL_18204765, EPI_ISL_18204767, EPI_ISL_18204771, EPI_ISL_18204773, EPI_ISL_18204775, EPI_ISL_18204777, EPI_ISL_18204780, EPI_ISL_18204782, EPI_ISL_18204784, EPI_ISL_18204786, EPI_ISL_18204788, EPI_ISL_18204838, EPI_ISL_18204856, EPI_ISL_18204861, EPI_ISL_18204867, EPI_ISL_18204869, EPI_ISL_18204886			
see above	Microbiology Department. Complexo Hospitalario Universitario de Vigo	Microbiology Department. Complexo Hospitalario Universitario de Vigo	Daviña C, Martinez L, Perez-Castro S
EPI_ISL_18228265, EPI_ISL_18228290, EPI_ISL_18228291, EPI_ISL_18228292, EPI_ISL_18228293, EPI_ISL_18228294, EPI_ISL_18228295, EPI_ISL_18240645, EPI_ISL_18240646, EPI_ISL_18240647, EPI_ISL_18240648, EPI_ISL_18240649, EPI_ISL_18240650, EPI_ISL_18240651			
see above	Institut Pasteur de Dakar	Institut Pasteur de Dakar	Jallow, Mamadou Malado; Diagne, Moussa Moise; Sankhe, Safietou; Mendy, Marie Pedapa; Ndiaye, Ndiende Koba; Sy, Sara; Kiori, Davy; Goudiaby, Deborah; Dia, Ndongo
EPI_ISL_18262238, EPI_ISL_18262240	Instituto de Medicina Tropical Alexander von Humboldt, Universidad Peruana Cayetano Heredia	Laboratorio de Genómica Microbiana, Universidad Peruana Cayetano Heredia	Ericka Meza, Diego Cuicapuza, Anne Martínez-Ventura, Brenda Ayzanoa, Janet Huancachoque, César Ugarte, Carlos Zamudio, Pablo Tsukayama
EPI_ISL_18265062, EPI_ISL_18265063, EPI_ISL_18265064, EPI_ISL_18265065, EPI_ISL_18265066, EPI_ISL_18265067, EPI_ISL_18272375, EPI_ISL_18272376, EPI_ISL_18272377, EPI_ISL_18272378, EPI_ISL_18272379, EPI_ISL_18272380, EPI_ISL_18272382, EPI_ISL_18272386, EPI_ISL_18272387, EPI_ISL_18272388, EPI_ISL_18272389, EPI_ISL_18272390, EPI_ISL_18272391, EPI_ISL_18272392, EPI_ISL_18272393, EPI_ISL_18272394, EPI_ISL_18272398, EPI_ISL_18272399, EPI_ISL_18272400, EPI_ISL_18272401, EPI_ISL_18272402, EPI_ISL_18272403, EPI_ISL_18272404, EPI_ISL_18272405, EPI_ISL_18277074, EPI_ISL_18277075, EPI_ISL_18277076, EPI_ISL_18277077, EPI_ISL_18277078, EPI_ISL_18277079, EPI_ISL_18277080, EPI_ISL_18277081, EPI_ISL_18277082, EPI_ISL_18277083, EPI_ISL_18277084, EPI_ISL_18277085, EPI_ISL_18277086, EPI_ISL_18277087, EPI_ISL_18277132, EPI_ISL_18277134, EPI_ISL_18277135			
see above	West of Scotland Specialist Virology Centre	West of Scotland Specialist Virology Centre	Lynne Ferguson, Imogen Johnston-Menzies, Rory Gunson
EPI_ISL_18279085	National Institute of Public Health	National Institute of Public Health	Chhorvann CHHEA, Darapheak CHAU, Sitha PRUM, Visal CHHE, Sokha DUL, Sengly MANH, Phally VY, Borann SAR, Chanthap LON, Jessica E. Manning, Sophana Chea, Savuth CHIN
EPI_ISL_18320972, EPI_ISL_18320973, EPI_ISL_18320974, EPI_ISL_18320975	Division Virologia, Laboratorio de Salud Publica, Tucuman	INEI-ANLIS "Dr. Carlos G. Malbran" Servicio de Virosis Respiratorias	Russo Mara, Martin Avaro, Estefania Benedetti, Maria Elena Dattero, Chamorro Ariana, Garcia Valdez Jackeline, Fabian Pardon, Erika Machicado Macias, Andrea Pontoriero.
EPI_ISL_18320976	Hospital Perrando	INEI-ANLIS "Dr. Carlos G. Malbran" Servicio de Virosis Respiratorias	Russo Mara, Martin Avaro, Estefania Benedetti, Maria Elena Dattero, Chamorro Ariana, Garcia Valdez Jackeline, Fabian Pardon, Erika Machicado Macias, Andrea Pontoriero.
EPI_ISL_18320977, EPI_ISL_18320978, EPI_ISL_18320979, EPI_ISL_18320980	Division Virologia, Laboratorio de Salud Publica, Tucuman	INEI-ANLIS "Dr. Carlos G. Malbran" Servicio de Virosis Respiratorias	Russo Mara, Martin Avaro, Estefania Benedetti, Maria Elena Dattero, Chamorro Ariana, Garcia Valdez Jackeline, Fabian Pardon, Erika Machicado Macias, Andrea Pontoriero.
EPI_ISL_18320981	Hospital de la Madre y el Niño de La Rioja	INEI-ANLIS "Dr. Carlos G. Malbran" Servicio de Virosis Respiratorias	Russo Mara, Martin Avaro, Estefania Benedetti, Maria Elena Dattero, Chamorro Ariana, Garcia Valdez Jackeline, Fabian Pardon, Erika Machicado Macias, Andrea Pontoriero.
EPI_ISL_18320982	Hospital de la Madre y el Niño de Formosa	INEI-ANLIS "Dr. Carlos G. Malbran" Servicio de Virosis Respiratorias	Russo Mara, Martin Avaro, Estefania Benedetti, Maria Elena Dattero, Chamorro Ariana, Garcia Valdez Jackeline, Fabian Pardon, Erika Machicado Macias, Andrea Pontoriero.
EPI_ISL_18320983, EPI_ISL_18320984	Division Virologia, Laboratorio de Salud Publica, Tucuman	INEI-ANLIS "Dr. Carlos G. Malbran" Servicio de Virosis Respiratorias	Russo Mara, Martin Avaro, Estefania Benedetti, Maria Elena Dattero, Chamorro Ariana, Garcia Valdez Jackeline, Fabian Pardon, Erika Machicado Macias, Andrea Pontoriero.
EPI_ISL_18320985	Hospital Nacional Profesor Alejandro Posadas	INEI-ANLIS "Dr. Carlos G. Malbran" Servicio de Virosis Respiratorias	Russo Mara, Martin Avaro, Estefania Benedetti, Maria Elena Dattero, Chamorro Ariana, Garcia Valdez Jackeline, Fabian Pardon, Erika Machicado Macias, Andrea Pontoriero.
EPI_ISL_18320986	Hospital de la Madre y el Niño de La Rioja	INEI-ANLIS "Dr. Carlos G. Malbran" Servicio de Virosis Respiratorias	Russo Mara, Martin Avaro, Estefania Benedetti, Maria Elena Dattero, Chamorro Ariana, Garcia Valdez Jackeline, Fabian Pardon, Erika Machicado Macias, Andrea Pontoriero.
EPI_ISL_18320987	Hospital Perrando	INEI-ANLIS "Dr. Carlos G. Malbran" Servicio de Virosis Respiratorias	Russo Mara, Martin Avaro, Estefania Benedetti, Maria Elena Dattero, Chamorro Ariana, Garcia Valdez Jackeline, Fabian Pardon, Erika Machicado Macias, Andrea Pontoriero.
EPI_ISL_18320988, EPI_ISL_18320989	Laboratorio Central. Subsecretaria de Salud de Neuquén	INEI-ANLIS "Dr. Carlos G. Malbran" Servicio de Virosis Respiratorias	Russo Mara, Martin Avaro, Estefania Benedetti, Maria Elena Dattero, Chamorro Ariana, Garcia Valdez Jackeline, Fabian Pardon, Erika Machicado Macias, Andrea Pontoriero.
EPI_ISL_18320990	Hospital Garrahan	INEI-ANLIS "Dr. Carlos G. Malbran" Servicio de Virosis Respiratorias	Russo Mara, Martin Avaro, Estefania Benedetti, Maria Elena Dattero, Chamorro Ariana, Garcia Valdez Jackeline, Fabian Pardon, Erika Machicado Macias, Andrea Pontoriero.
EPI_ISL_18320991	Hospital de la Madre y el Niño de La Rioja	INEI-ANLIS "Dr. Carlos G. Malbran" Servicio de Virosis Respiratorias	Russo Mara, Martin Avaro, Estefania Benedetti, Maria Elena Dattero, Chamorro Ariana, Garcia Valdez Jackeline, Fabian Pardon, Erika Machicado Macias, Andrea Pontoriero.
EPI_ISL_18320992	Division Virologia, Laboratorio de Salud Publica, Tucuman	INEI-ANLIS "Dr. Carlos G. Malbran" Servicio de Virosis Respiratorias	Russo Mara, Martin Avaro, Estefania Benedetti, Maria Elena Dattero, Chamorro Ariana, Garcia Valdez Jackeline, Fabian Pardon, Erika Machicado Macias, Andrea Pontoriero.
EPI_ISL_18320993	Laboratorio de Salud Pública de San Luis	INEI-ANLIS "Dr. Carlos G. Malbran" Servicio de Virosis Respiratorias	Russo Mara, Martin Avaro, Estefania Benedetti, Maria Elena Dattero, Chamorro Ariana, Garcia Valdez Jackeline, Fabian Pardon, Erika Machicado Macias, Andrea Pontoriero.
EPI_ISL_18320994	Laboratorio Central. Subsecretaria de Salud de Neuquén	INEI-ANLIS "Dr. Carlos G. Malbran" Servicio de Virosis Respiratorias	Russo Mara, Martin Avaro, Estefania Benedetti, Maria Elena Dattero, Chamorro Ariana, Garcia Valdez Jackeline, Fabian Pardon, Erika Machicado Macias, Andrea Pontoriero.
EPI_ISL_18320995, EPI_ISL_18320996	Hospital Perrando	INEI-ANLIS "Dr. Carlos G. Malbran" Servicio de Virosis Respiratorias	Russo Mara, Martin Avaro, Estefania Benedetti, Maria Elena Dattero, Chamorro Ariana, Garcia Valdez Jackeline, Fabian Pardon, Erika Machicado Macias, Andrea Pontoriero.
EPI_ISL_18320997, EPI_ISL_18320998	Hospital Nacional Profesor Alejandro Posadas	INEI-ANLIS "Dr. Carlos G. Malbran" Servicio de Virosis Respiratorias	Russo Mara, Martin Avaro, Estefania Benedetti, Maria Elena Dattero, Chamorro Ariana, Garcia Valdez Jackeline, Fabian Pardon, Erika Machicado Macias, Andrea Pontoriero.
EPI_ISL_18320999	Hospital Regional Río Gallegos- Laboratorio Biología Molecular	INEI-ANLIS "Dr. Carlos G. Malbran" Servicio de Virosis Respiratorias	Russo Mara, Martin Avaro, Estefania Benedetti, Maria Elena Dattero, Chamorro Ariana, Garcia Valdez Jackeline, Fabian Pardon, Erika Machicado Macias, Andrea Pontoriero.
EPI_ISL_18321000	Hospital de Emergencias "Papa Francisco" - UPA9	INEI-ANLIS "Dr. Carlos G. Malbran" Servicio de Virosis Respiratorias	Russo Mara, Martin Avaro, Estefania Benedetti, Maria Elena Dattero, Chamorro Ariana, Garcia Valdez Jackeline, Fabian Pardon, Erika Machicado Macias, Andrea Pontoriero.
EPI_ISL_18321001	Hospital de la Madre y el Niño de La Rioja	INEI-ANLIS "Dr. Carlos G. Malbran" Servicio de Virosis Respiratorias	Russo Mara, Martin Avaro, Estefania Benedetti, Maria Elena Dattero, Chamorro Ariana, Garcia Valdez Jackeline, Fabian Pardon, Erika Machicado Macias, Andrea Pontoriero.
EPI_ISL_18321002	Laboratorio de Salud Pública de San Luis	INEI-ANLIS "Dr. Carlos G. Malbran" Servicio de Virosis Respiratorias	Russo Mara, Martin Avaro, Estefania Benedetti, Maria Elena Dattero, Chamorro Ariana, Garcia Valdez Jackeline, Fabian Pardon, Erika Machicado Macias, Andrea Pontoriero.
EPI_ISL_18321003	Hospital Garrahan	INEI-ANLIS "Dr. Carlos G. Malbran" Servicio de Virosis Respiratorias	Russo Mara, Martin Avaro, Estefania Benedetti, Maria Elena Dattero, Chamorro Ariana, Garcia Valdez Jackeline, Fabian Pardon, Erika Machicado Macias, Andrea Pontoriero.
EPI_ISL_18321008, EPI_ISL_18321009, EPI_ISL_18321010, EPI_ISL_18321011, EPI_ISL_18321012, EPI_ISL_18321013, EPI_ISL_18321014, EPI_ISL_18321015, EPI_ISL_18321016, EPI_ISL_18321017, EPI_ISL_18321018, EPI_ISL_18321019, EPI_ISL_18321020, EPI_ISL_18321034, EPI_ISL_18321035, EPI_ISL_18321036, EPI_ISL_18321037, EPI_ISL_18321038, EPI_ISL_18321039, EPI_ISL_18321040, EPI_ISL_18321041, EPI_ISL_18321042, EPI_ISL_18321043, EPI_ISL_18321044, EPI_ISL_18321045, EPI_ISL_18321046, EPI_ISL_18321047, EPI_ISL_18321048, EPI_ISL_18321049, EPI_ISL_18321050, EPI_ISL_18321051, EPI_ISL_18321052, EPI_ISL_18321053, EPI_ISL_18321054, EPI_ISL_18321055, EPI_ISL_18321078, EPI_ISL_18321079, EPI_ISL_18321080, EPI_ISL_18321081, EPI_ISL_18321082,			

EPI_ISL_18321083, EPI_ISL_18321084, EPI_ISL_18321085, EPI_ISL_18321086, EPI_ISL_18321088, EPI_ISL_18321089, EPI_ISL_18321090, EPI_ISL_18321091, EPI_ISL_18321092, EPI_ISL_18321093, EPI_ISL_18321094, EPI_ISL_18321095, EPI_ISL_18321096, EPI_ISL_18321097, EPI_ISL_18321098, EPI_ISL_18321099, EPI_ISL_18321100, EPI_ISL_18321101			
see above	National Virus Reference Laboratory	National Virus Reference Laboratory	Michael Carr, Charlene Bennett, Jonathan Dean, Daniel Hare, Cillian F De Gascun
EPI_ISL_18321533, EPI_ISL_18321558	Servicio de Microbiología Complejo Hospitalario Universitario Nuestra Señora de Candelaria	Institute of Technology and Renewable Energy (ITER) Genomics Division	Julia,Alcoba-Florez;Rafaela,González-Montelongo;Diego,García-Martínez de Artola;Adrián,Muñoz-Barrera;Helena,Gil-Campesino;Oscar,Diez-Gil;Jose Miguel,Lorenzo-Salazar;Carlos,Flores
EPI_ISL_18329452, EPI_ISL_18329453, EPI_ISL_18329454, EPI_ISL_18329455, EPI_ISL_18329456, EPI_ISL_18329457, EPI_ISL_18329458, EPI_ISL_18329459, EPI_ISL_18329460, EPI_ISL_18329462	Microbiology and Virology Depertment, Fondazione IRCCS Policlinico San Matteo, Pavia	Microbiology and Virology Depertment, Fondazione IRCCS Policlinico San Matteo, Pavia	Gugliemo Ferrari, Federica Giardina, Antonino Pitrolo, Fausto Baldanti
EPI_ISL_18334081, EPI_ISL_18334085, EPI_ISL_18334088, EPI_ISL_18334094, EPI_ISL_18334098, EPI_ISL_18334102, EPI_ISL_18334104, EPI_ISL_18334109, EPI_ISL_18334125, EPI_ISL_18334128, EPI_ISL_18334129, EPI_ISL_18334130, EPI_ISL_18334133, EPI_ISL_18334151, EPI_ISL_18334156, EPI_ISL_18334158, EPI_ISL_18334159, EPI_ISL_18334161, EPI_ISL_18334162			
see above	Tianjin Children's Hospital, Tianjin Pediatric Research Institute	Tianjin Children's Hospital, Tianjin Pediatric Research Institute	Hou,M., Meng,C., Dong,L. and Wang,H.
EPI_ISL_18334244, EPI_ISL_18334259, EPI_ISL_18334263, EPI_ISL_18334267, EPI_ISL_18334268, EPI_ISL_18334273, EPI_ISL_18334275, EPI_ISL_18334277, EPI_ISL_18334283, EPI_ISL_18334297, EPI_ISL_18334302, EPI_ISL_18334304, EPI_ISL_18334312, EPI_ISL_18334313, EPI_ISL_18334316, EPI_ISL_18334318, EPI_ISL_18334328, EPI_ISL_18334336, EPI_ISL_18334338, EPI_ISL_18334349, EPI_ISL_18334359, EPI_ISL_18334369, EPI_ISL_18334376, EPI_ISL_18334387, EPI_ISL_18334389, EPI_ISL_18334394, EPI_ISL_18334403, EPI_ISL_18334405, EPI_ISL_18334406, EPI_ISL_18334411			
see above	National Influenza Center, Virology Laboratory	National Influenza Center, Virology Laboratory	Simusika,P., Okamoto,M., Dapat,C., Muleya,W., Saito,M., Azam,S., Mwape,I., Mwila,E., Monze,M. and Oshitani,H.
EPI_ISL_18339703, EPI_ISL_18339705, EPI_ISL_18339707, EPI_ISL_18339710, EPI_ISL_18339712, EPI_ISL_18339713, EPI_ISL_18339714, EPI_ISL_18339715, EPI_ISL_18339718, EPI_ISL_18339722, EPI_ISL_18339726, EPI_ISL_18339728, EPI_ISL_18339729, EPI_ISL_18339733, EPI_ISL_18339735, EPI_ISL_18339736, EPI_ISL_18339738, EPI_ISL_18339739, EPI_ISL_18339744, EPI_ISL_18339746, EPI_ISL_18339751, EPI_ISL_18339754, EPI_ISL_18339759, EPI_ISL_18339761, EPI_ISL_18339762, EPI_ISL_18339763			
see above	Department of Microbiology, Jawaharlal Institute of Postgraduate Medical Education and Research	Department of Microbiology, Jawaharlal Institute of Postgraduate Medical Education and Research	Shameema,M., Dhodapkar,R., Sharmila,P.F.M. and Venkatesh,C.
EPI_ISL_18356570, EPI_ISL_18356572, EPI_ISL_18356577, EPI_ISL_18356580, EPI_ISL_18356581, EPI_ISL_18356583, EPI_ISL_18356585, EPI_ISL_18356588, EPI_ISL_18356592, EPI_ISL_18356593, EPI_ISL_18356596, EPI_ISL_18356599, EPI_ISL_18356600, EPI_ISL_18356601, EPI_ISL_18356602, EPI_ISL_18356604, EPI_ISL_18356606, EPI_ISL_18356608, EPI_ISL_18356611, EPI_ISL_18356612, EPI_ISL_18356616, EPI_ISL_18356617, EPI_ISL_18356618, EPI_ISL_18356619, EPI_ISL_18356622, EPI_ISL_18356626, EPI_ISL_18356627, EPI_ISL_18356628, EPI_ISL_18356631, EPI_ISL_18356633, EPI_ISL_18356634, EPI_ISL_18356637, EPI_ISL_18356642, EPI_ISL_18356643, EPI_ISL_18356644, EPI_ISL_18356648, EPI_ISL_18356650, EPI_ISL_18356652, EPI_ISL_18356656, EPI_ISL_18356659, EPI_ISL_18356663, EPI_ISL_18356664, EPI_ISL_18356665, EPI_ISL_18356666, EPI_ISL_18356667, EPI_ISL_18356669, EPI_ISL_18356675, EPI_ISL_18356678, EPI_ISL_18356681, EPI_ISL_18356683			
see above	Hospital Central de Asturias, Microbiologia	Hospital Central de Asturias, Microbiologia	Rojo-Alba,S., Perez-Martinez,Z., Boga,J., Alvarez-Arguelles,M.E., Gonzalez-Alba,J.M. and Melon,S.
EPI_ISL_18374651, EPI_ISL_18374653	Edendale Hospital	National Institute for Communicable Diseases of the National Health Laboratory Service	Everatt J, Kekana D, Mahlangu B, Stock N, Ntozini B, Ntuli N, Mnguni A, Nzimande A, Ismail A, Bhiman JN, Wolter N
EPI_ISL_18374654	Edendale Gateway Clinic SRH	National Institute for Communicable Diseases of the National Health Laboratory Service	Everatt J, Kekana D, Mahlangu B, Stock N, Ntozini B, Ntuli N, Mnguni A, Nzimande A, Ismail A, Bhiman JN, Wolter N
EPI_ISL_18374655	Jouberton Clinic	National Institute for Communicable Diseases of the National Health Laboratory Service	Everatt J, Kekana D, Mahlangu B, Stock N, Ntozini B, Ntuli N, Mnguni A, Nzimande A, Ismail A, Bhiman JN, Wolter N
EPI_ISL_18374660, EPI_ISL_18374661, EPI_ISL_18374662	Klerksdorp	National Institute for Communicable Diseases of the National Health Laboratory Service	Everatt J, Kekana D, Mahlangu B, Stock N, Ntozini B, Ntuli N, Mnguni A, Nzimande A, Ismail A, Bhiman JN, Wolter N
EPI_ISL_18374665	Mitchells Plain Hospital	National Institute for Communicable Diseases of the National Health Laboratory Service	Everatt J, Kekana D, Mahlangu B, Stock N, Ntozini B, Ntuli N, Mnguni A, Nzimande A, Ismail A, Bhiman JN, Wolter N
EPI_ISL_18374666	National Institute for Communicable Diseases of the National Health Laboratory Service	National Institute for Communicable Diseases of the National Health Laboratory Service	Everatt J, Kekana D, Mahlangu B, Stock N, Ntozini B, Ntuli N, Mnguni A, Nzimande A, Ismail A, Bhiman JN, Wolter N
EPI_ISL_18374667, EPI_ISL_18374668, EPI_ISL_18374672, EPI_ISL_18374673, EPI_ISL_18374675, EPI_ISL_18374676, EPI_ISL_18374678	Rahima Moosa Mother and Child Hospital	National Institute for Communicable Diseases of the National Health Laboratory Service	Everatt J, Kekana D, Mahlangu B, Stock N, Ntozini B, Ntuli N, Mnguni A, Nzimande A, Ismail A, Bhiman JN, Wolter N
EPI_ISL_18374680, EPI_ISL_18374681, EPI_ISL_18374682, EPI_ISL_18374683, EPI_ISL_18374684, EPI_ISL_18374685, EPI_ISL_18374686, EPI_ISL_18374687, EPI_ISL_18374689, EPI_ISL_18374690	The South African Red Cross Society	National Institute for Communicable Diseases of the National Health Laboratory Service	Everatt J, Kekana D, Mahlangu B, Stock N, Ntozini B, Ntuli N, Mnguni A, Nzimande A, Ismail A, Bhiman JN, Wolter N
EPI_ISL_18374691	Tambo Memorial Hospital	National Institute for Communicable Diseases of the National Health Laboratory Service	Everatt J, Kekana D, Mahlangu B, Stock N, Ntozini B, Ntuli N, Mnguni A, Nzimande A, Ismail A, Bhiman JN, Wolter N
EPI_ISL_18374692	Tintswalo Hospital	National Institute for Communicable Diseases of the National Health Laboratory Service	Everatt J, Kekana D, Mahlangu B, Stock N, Ntozini B, Ntuli N, Mnguni A, Nzimande A, Ismail A, Bhiman JN, Wolter N
EPI_ISL_18387876, EPI_ISL_18387878, EPI_ISL_18387879, EPI_ISL_18387880, EPI_ISL_18387881, EPI_ISL_18387883, EPI_ISL_18387885, EPI_ISL_18387886, EPI_ISL_18387888, EPI_ISL_18387890, EPI_ISL_18387891, EPI_ISL_18387892, EPI_ISL_18387893, EPI_ISL_18387894, EPI_ISL_18387895, EPI_ISL_18387896, EPI_ISL_18387897, EPI_ISL_18387899, EPI_ISL_18387901, EPI_ISL_18387903, EPI_ISL_18387904, EPI_ISL_18387906, EPI_ISL_18387908, EPI_ISL_18387909, EPI_ISL_18387911, EPI_ISL_18387912, EPI_ISL_18387913, EPI_ISL_18387914, EPI_ISL_18387915, EPI_ISL_18387916, EPI_ISL_18387919, EPI_ISL_18387921, EPI_ISL_18387922, EPI_ISL_18387923, EPI_ISL_18387925, EPI_ISL_18387926, EPI_ISL_18387927, EPI_ISL_18387928, EPI_ISL_18387929, EPI_ISL_18387930, EPI_ISL_18387931, EPI_ISL_18387933, EPI_ISL_18387934, EPI_ISL_18387937, EPI_ISL_18387938, EPI_ISL_18387939, EPI_ISL_18387941, EPI_ISL_18387943, EPI_ISL_18387944, EPI_ISL_18387946, EPI_ISL_18387948, EPI_ISL_18387949, EPI_ISL_18387950, EPI_ISL_18387953, EPI_ISL_18387955, EPI_ISL_18387956, EPI_ISL_18387958, EPI_ISL_18387959, EPI_ISL_18387960, EPI_ISL_18387962, EPI_ISL_18387963			
see above	The Brotman Baty Institute for Precision Medicine	The Brotman Baty Institute for Precision Medicine	Frazar,C.D., Lee,J., Ryke,E., Gamboa,L., McDermot,E., Kolar,T., Han,P.D., Sibley,T.R., Reinhart,D., Grindstaff,S., Truong,M., Babu,T.M., Feldstein,L.R., Saydah,S., Briggs-Hagen,M., Casto,A., Ehmen,B., Englund,J.A., Fortmann,S.P., Kuntz,J.L., Lockwood,T., Midgley,C.M., Mularski,R.A., Ogilvie,T., Reich,S., Schmidt,M.A., Smith,N., Starita,L., Stone,J., Vandermeer,M., Weil,A.A., Wolf,C.R., Chu,H.Y. and Naleway,A.L.
EPI_ISL_18387964	Department of Communicable Diseases, Interactive Research School for Health Affairs	Department of Communicable Diseases, Interactive Research School for Health Affairs	Mandviwala,A. and Patil,H.
EPI_ISL_18396210	Niigata University (IDRC) Infectious Diseases Research Center	Niigata University (IDRC) Infectious Diseases Research Center	Wint Wint Phyu, Reiko Saito
EPI_ISL_18401461, EPI_ISL_18401557, EPI_ISL_18401558, EPI_ISL_18401674	Niigata University (IDRC) Infectious Diseases Research Center	Niigata University (IDRC) Infectious Diseases Research Center	Sayaka Yoshioka, Reiko Saito
EPI_ISL_18446761, EPI_ISL_18446762, EPI_ISL_18446765, EPI_ISL_18446766, EPI_ISL_18446767, EPI_ISL_18446768, EPI_ISL_18446769, EPI_ISL_18446770, EPI_ISL_18446771, EPI_ISL_18446772, EPI_ISL_18446773, EPI_ISL_18446774, EPI_ISL_18446775, EPI_ISL_18446776, EPI_ISL_18446777, EPI_ISL_18446778, EPI_ISL_18446779, EPI_ISL_18446780, EPI_ISL_18446781, EPI_ISL_18446782, EPI_ISL_18446783, EPI_ISL_18446784, EPI_ISL_18446785, EPI_ISL_18446786, EPI_ISL_18446787, EPI_ISL_18446788, EPI_ISL_18446789, EPI_ISL_18446790, EPI_ISL_18446791, EPI_ISL_18446793, EPI_ISL_18446794, EPI_ISL_18446795, EPI_ISL_18446796, EPI_ISL_18446797, EPI_ISL_18446798, EPI_ISL_18446799, EPI_ISL_18446800, EPI_ISL_18446802, EPI_ISL_18446803, EPI_ISL_18446804, EPI_ISL_18446805, EPI_ISL_18446806, EPI_ISL_18446807, EPI_ISL_18446808, EPI_ISL_18446809			
see above	Vittore Buzzi Children Hospital, Pediatric Department	Laboratory of Infectious Diseases, Department of Biomedical and Clinical Sciences, University of Milan	Alessia Lai, Annalisa Bergna, Valentina Fabiano, Carla della Ventura, Giulia Fumagalli, Alessandra Mari, Martina Loiodice, Gian Vincenzo Zuccotti, Gianguglielmo Zehender
EPI_ISL_18452361, EPI_ISL_18452363, EPI_ISL_18452364, EPI_ISL_18452365, EPI_ISL_18452366, EPI_ISL_18452367, EPI_ISL_18452368, EPI_ISL_18452369, EPI_ISL_18452370, EPI_ISL_18452371	The Brotman Baty Institute for Precision Medicine	The Brotman Baty Institute for Precision Medicine	Frazar,C.D., Lee,J., Ryke,E., Gamboa,L., McDermot,E., Kolar,T., Han,P.D., Sibley,T.R., Reinhart,D., Grindstaff,S., Truong,M., Babu,T.M., Feldstein,L.R., Saydah,S., Briggs-Hagen,M., Casto,A., Ehmen,B., Englund,J.A., Fortmann,S.P., Kuntz,J.L., Lockwood,T., Midgley,C.M., Mularski,R.A., Ogilvie,T., Reich,S., Schmidt,M.A., Smith,N., Starita,L., Stone,J., Vandermeer,M., Weil,A.A., Wolf,C.R., Chu,H.Y. and Naleway,A.L.
EPI_ISL_18463162, EPI_ISL_18463165, EPI_ISL_18463167, EPI_ISL_18463170, EPI_ISL_18463181, EPI_ISL_18463183, EPI_ISL_18463184, EPI_ISL_18463185, EPI_ISL_18463186	Hospital Universitario Severo Ochoa	Instituto de Salud Carlos III	Iglesias-Caballero, M. Camarero, S. Pozo, F. Casas, I. Jiménez, P. Zaballos, A.Varona, S. Monzón, S.Cuesta, I. García-García, M.L.
EPI_ISL_18469654	Hospital Santa María Nai	Instituto de Salud Carlos III	Iglesias-Caballero, M. Camarero, S. Pozo, F. Casas, I. Jiménez, P. Zaballos, A.Varona, S. Monzón, S.Cuesta, I. García, J.
EPI_ISL_18469655, EPI_ISL_18469656, EPI_ISL_18469657, EPI_ISL_18469658, EPI_ISL_18469659, EPI_ISL_18469660, EPI_ISL_18469661, EPI_ISL_18469662, EPI_ISL_18469663, EPI_ISL_18469664, EPI_ISL_18469665, EPI_ISL_18469666, EPI_ISL_18469667			
see above	Hospital Universitario Severo Ochoa	Instituto de Salud Carlos III	Iglesias-Caballero, M. Camarero, S. Pozo, F. Casas, I. Jiménez, P. Zaballos, A.Varona, S. Monzón, S.Cuesta, I. García-García, M.L.
EPI_ISL_18469668	Hospital Universitario La Paz	Instituto de Salud Carlos III	Iglesias-Caballero, M. Camarero, S. Pozo, F. Casas, I. Jiménez, P. Zaballos, A.Varona, S. Monzón, S.Cuesta, Calvo, C.
EPI_ISL_18469669, EPI_ISL_18469670, EPI_ISL_18469671	Hospital Universitario Severo Ochoa	Instituto de Salud Carlos III	Iglesias-Caballero, M. Camarero, S. Pozo, F. Casas, I. Jiménez, P. Zaballos, A.Varona, S. Monzón, S.Cuesta, I. García-García, M.L.
EPI_ISL_18469672, EPI_ISL_18469673	Hospital Universitario La Paz	Instituto de Salud Carlos III	Iglesias-Caballero, M. Camarero, S. Pozo, F. Casas, I. Jiménez, P. Zaballos, A.Varona, S. Monzón, S.Cuesta, I. Calvo, C.
EPI_ISL_18469674, EPI_ISL_18469675, EPI_ISL_18469676, EPI_ISL_18469677, EPI_ISL_18469678, EPI_ISL_18469679, EPI_ISL_18469680, EPI_ISL_18469681, EPI_ISL_18469682, EPI_ISL_18469683, EPI_ISL_18469684, EPI_ISL_18469685, EPI_ISL_18469686, EPI_ISL_18469687, EPI_ISL_18469688, EPI_ISL_18469689, EPI_ISL_18469690, EPI_ISL_18469691, EPI_ISL_18469692, EPI_ISL_18469693			
see above	Hospital Universitario Severo Ochoa	Instituto de Salud Carlos III	Iglesias-Caballero, M. Camarero, S. Pozo, F. Casas, I. Jiménez, P. Zaballos, A.Varona, S. Monzón, S.Cuesta, I. García-García, M.L.
EPI_ISL_18469694	Hospital Universitario La Paz	Instituto de Salud Carlos III	Iglesias-Caballero, M. Camarero, S. Pozo, F. Casas, I. Jiménez, P. Zaballos, A.Varona, S. Monzón, S.Cuesta, I. Calvo, C.
EPI_ISL_18469695, EPI_ISL_18469696, EPI_ISL_18469697, EPI_ISL_18469698, EPI_ISL_18469699, EPI_ISL_18469700, EPI_ISL_18469701, EPI_ISL_18469702, EPI_ISL_18469703, EPI_ISL_18469704, EPI_ISL_18469705, EPI_ISL_18469706, EPI_ISL_18469707, EPI_ISL_18469708, EPI_ISL_18469709, EPI_ISL_18469710, EPI_ISL_18469711, EPI_ISL_18469712, EPI_ISL_18469713, EPI_ISL_18469715, EPI_ISL_18469716, EPI_ISL_18469717, EPI_ISL_18469718, EPI_ISL_18469719			
see above	Hospital Universitario Severo Ochoa	Instituto de Salud Carlos III	Iglesias-Caballero, M. Camarero, S. Pozo, F. Casas, I. Jiménez, P. Zaballos, A.Varona, S. Monzón, S.Cuesta, I. García-García, M.L.
EPI_ISL_18507281, EPI_ISL_18507287, EPI_ISL_18507288, EPI_ISL_18507291, EPI_ISL_18507292, EPI_ISL_18507294, EPI_ISL_18507295, EPI_ISL_18507296, EPI_ISL_18507297	Victorian Infectious Diseases Reference Laboratory, Translational Diagnostics	Victorian Infectious Diseases Reference Laboratory, Translational Diagnostics	Steinig JOACHIM,E.
EPI_ISL_18509832, EPI_ISL_18509835, EPI_ISL_18509838, EPI_ISL_18509840, EPI_ISL_18509842, EPI_ISL_18509844, EPI_ISL_18509846, EPI_ISL_18509849, EPI_ISL_18509884, EPI_ISL_18509895, EPI_ISL_18509897, EPI_ISL_18509899, EPI_ISL_18509901, EPI_ISL_18509903, EPI_ISL_18510219			
see above	Institut Pasteur de Dakar	Institut Pasteur de Dakar	Jallow,Mamadou Malado; Diagne,Moussa Moise; Sankhe,Safietou; Diop,Seynabou Mbaye Ba Souna; Mendy,Marie Pedepa; Sy,Sara; Ndiaye,Ndiende Koba; Kiory, Davy Evrard; Goudiaby,Deborah; Dia,Ndongo
EPI_ISL_18522759, EPI_ISL_18522767	Servicio Microbiología Hospital La Paz	Servicio Microbiología Hospital La Paz	Fernando Lázaro, Iván Bloise, Pablo Prieto Casado, Francisco López Rodrigo, Jesús Mingorance Cruz, Elie Dahdouh
EPI_ISL_18537458	RELAB - BIOGROUP - Plateau technique St Denis	National Reference Center for Viruses of	Léa Avon, Marion Barbet, Marine Bernard, Emma Bezot, Angela Brisebarre, Océane Dehan, Flora Donati, Vanessa Guimaraes, Banujaa Jeyarajah, Florian Préjean, Yannis Rahou, Sylvie van der Werf, Frédéric Lemoine, Samar Berreira

		Respiratory Infections, Institut Pasteur, Paris	Ibraim, Jérôme Bourret, Kévin Da Silva, Maud Vanpeene, Vincent Enouf, Marie-Anne Rameix-Welti, Yanis CHAIB
EPI_ISL_18537459	Sentinelles Ile-de-France	National Reference Center for Viruses of Respiratory Infections, Institut Pasteur, Paris	Léa Avon, Marion Barbet, Marine Bernard, Emma Bezot, Angela Brisebarre, Océane Dehan, Flora Donati, Vanessa Guimaraes, Banujaa Jeyarajah, Florian Préjean, Yannis Rahou, Sylvie van der Werf, Frédéric Lemoine, Samar Berreira Ibraim, Jérôme Bourret, Kévin Da Silva, Maud Vanpeene, Vincent Enouf, Marie-Anne Rameix-Welti, Patricia LUBELSKI
EPI_ISL_18537460	Sentinelles Province	National Reference Center for Viruses of Respiratory Infections, Institut Pasteur, Paris	Léa Avon, Marion Barbet, Marine Bernard, Emma Bezot, Angela Brisebarre, Océane Dehan, Flora Donati, Vanessa Guimaraes, Banujaa Jeyarajah, Florian Préjean, Yannis Rahou, Sylvie van der Werf, Frédéric Lemoine, Samar Berreira Ibraim, Jérôme Bourret, Kévin Da Silva, Maud Vanpeene, Vincent Enouf, Marie-Anne Rameix-Welti, Eric VAN MELKEBEKE
EPI_ISL_18537461	Sentinelles Province	National Reference Center for Viruses of Respiratory Infections, Institut Pasteur, Paris	Léa Avon, Marion Barbet, Marine Bernard, Emma Bezot, Angela Brisebarre, Océane Dehan, Flora Donati, Vanessa Guimaraes, Banujaa Jeyarajah, Florian Préjean, Yannis Rahou, Sylvie van der Werf, Frédéric Lemoine, Samar Berreira Ibraim, Jérôme Bourret, Kévin Da Silva, Maud Vanpeene, Vincent Enouf, Marie-Anne Rameix-Welti, Gwenaelle MAHE
EPI_ISL_18537462	Sentinelles Ile-de-France	National Reference Center for Viruses of Respiratory Infections, Institut Pasteur, Paris	Léa Avon, Marion Barbet, Marine Bernard, Emma Bezot, Angela Brisebarre, Océane Dehan, Flora Donati, Vanessa Guimaraes, Banujaa Jeyarajah, Florian Préjean, Yannis Rahou, Sylvie van der Werf, Frédéric Lemoine, Samar Berreira Ibraim, Jérôme Bourret, Kévin Da Silva, Maud Vanpeene, Vincent Enouf, Marie-Anne Rameix-Welti, Frédéric URBAIN
EPI_ISL_18537463, EPI_ISL_18537464	RELAB - BIOGROUP - Plateau technique St Denis	National Reference Center for Viruses of Respiratory Infections, Institut Pasteur, Paris	Léa Avon, Marion Barbet, Marine Bernard, Emma Bezot, Angela Brisebarre, Océane Dehan, Flora Donati, Vanessa Guimaraes, Banujaa Jeyarajah, Florian Préjean, Yannis Rahou, Sylvie van der Werf, Frédéric Lemoine, Samar Berreira Ibraim, Jérôme Bourret, Kévin Da Silva, Maud Vanpeene, Vincent Enouf, Marie-Anne Rameix-Welti, Yanis CHAIB
EPI_ISL_18537465	Sentinelles Ile-de-France	National Reference Center for Viruses of Respiratory Infections, Institut Pasteur, Paris	Léa Avon, Marion Barbet, Marine Bernard, Emma Bezot, Angela Brisebarre, Océane Dehan, Flora Donati, Vanessa Guimaraes, Banujaa Jeyarajah, Florian Préjean, Yannis Rahou, Sylvie van der Werf, Frédéric Lemoine, Samar Berreira Ibraim, Jérôme Bourret, Kévin Da Silva, Maud Vanpeene, Vincent Enouf, Marie-Anne Rameix-Welti, Marie-Anne COSSON
EPI_ISL_18537468	RELAB - BIOGROUP - Plateau technique St Denis	National Reference Center for Viruses of Respiratory Infections, Institut Pasteur, Paris	Léa Avon, Marion Barbet, Marine Bernard, Emma Bezot, Angela Brisebarre, Océane Dehan, Flora Donati, Vanessa Guimaraes, Banujaa Jeyarajah, Florian Préjean, Yannis Rahou, Sylvie van der Werf, Frédéric Lemoine, Samar Berreira Ibraim, Jérôme Bourret, Kévin Da Silva, Maud Vanpeene, Vincent Enouf, Marie-Anne Rameix-Welti, Yanis CHAIB
EPI_ISL_18537469	Hôpital Ambroise Paré - Service de Microbiologie et hygiène	National Reference Center for Viruses of Respiratory Infections, Institut Pasteur, Paris	Léa Avon, Marion Barbet, Marine Bernard, Emma Bezot, Angela Brisebarre, Océane Dehan, Flora Donati, Vanessa Guimaraes, Banujaa Jeyarajah, Florian Préjean, Yannis Rahou, Sylvie van der Werf, Frédéric Lemoine, Samar Berreira Ibraim, Jérôme Bourret, Kévin Da Silva, Maud Vanpeene, Vincent Enouf, Marie-Anne Rameix-Welti, Elyanne GAULT
EPI_ISL_18537472, EPI_ISL_18537474, EPI_ISL_18537479, EPI_ISL_18537480	RELAB - BIOGROUP - Plateau technique St Denis	National Reference Center for Viruses of Respiratory Infections, Institut Pasteur, Paris	Léa Avon, Marion Barbet, Marine Bernard, Emma Bezot, Angela Brisebarre, Océane Dehan, Flora Donati, Vanessa Guimaraes, Banujaa Jeyarajah, Florian Préjean, Yannis Rahou, Sylvie van der Werf, Frédéric Lemoine, Samar Berreira Ibraim, Jérôme Bourret, Kévin Da Silva, Maud Vanpeene, Vincent Enouf, Marie-Anne Rameix-Welti, Yanis CHAIB
EPI_ISL_18537482	Sentinelles Ile-de-France	National Reference Center for Viruses of Respiratory Infections, Institut Pasteur, Paris	Léa Avon, Marion Barbet, Marine Bernard, Emma Bezot, Angela Brisebarre, Océane Dehan, Flora Donati, Vanessa Guimaraes, Banujaa Jeyarajah, Florian Préjean, Yannis Rahou, Sylvie van der Werf, Frédéric Lemoine, Samar Berreira Ibraim, Jérôme Bourret, Kévin Da Silva, Maud Vanpeene, Vincent Enouf, Marie-Anne Rameix-Welti, MARIE ANNE DAUMONT
EPI_ISL_18537483, EPI_ISL_18537484, EPI_ISL_18537487, EPI_ISL_18537488, EPI_ISL_18537489	RELAB - BIOGROUP - Plateau technique St Denis	National Reference Center for Viruses of Respiratory Infections, Institut Pasteur, Paris	Léa Avon, Marion Barbet, Marine Bernard, Emma Bezot, Angela Brisebarre, Océane Dehan, Flora Donati, Vanessa Guimaraes, Banujaa Jeyarajah, Florian Préjean, Yannis Rahou, Sylvie van der Werf, Frédéric Lemoine, Samar Berreira Ibraim, Jérôme Bourret, Kévin Da Silva, Maud Vanpeene, Vincent Enouf, Marie-Anne Rameix-Welti, Yanis CHAIB
EPI_ISL_18537494	Hôpital Robert Debre, Service Microbiologie	National Reference Center for Viruses of Respiratory Infections, Institut Pasteur, Paris	Léa Avon, Marion Barbet, Marine Bernard, Emma Bezot, Angela Brisebarre, Océane Dehan, Flora Donati, Vanessa Guimaraes, Banujaa Jeyarajah, Florian Préjean, Yannis Rahou, Sylvie van der Werf, Frédéric Lemoine, Samar Berreira Ibraim, Jérôme Bourret, Kévin Da Silva, Maud Vanpeene, Vincent Enouf, Marie-Anne Rameix-Welti, Stéphane BONACORSI
EPI_ISL_18537495, EPI_ISL_18537496, EPI_ISL_18537497	RELAB - BIOGROUP - Plateau technique St Denis	National Reference Center for Viruses of Respiratory Infections, Institut Pasteur, Paris	Léa Avon, Marion Barbet, Marine Bernard, Emma Bezot, Angela Brisebarre, Océane Dehan, Flora Donati, Vanessa Guimaraes, Banujaa Jeyarajah, Florian Préjean, Yannis Rahou, Sylvie van der Werf, Frédéric Lemoine, Samar Berreira Ibraim, Jérôme Bourret, Kévin Da Silva, Maud Vanpeene, Vincent Enouf, Marie-Anne Rameix-Welti, Yanis CHAIB
EPI_ISL_18537503	RELAB - Laboratoire Biomer	National Reference Center for Viruses of Respiratory Infections, Institut Pasteur, Paris	Léa Avon, Marion Barbet, Marine Bernard, Emma Bezot, Angela Brisebarre, Océane Dehan, Flora Donati, Vanessa Guimaraes, Banujaa Jeyarajah, Florian Préjean, Yannis Rahou, Sylvie van der Werf, Frédéric Lemoine, Samar Berreira Ibraim, Jérôme Bourret, Kévin Da Silva, Maud Vanpeene, Vincent Enouf, Marie-Anne Rameix-Welti, Alexandra JACQUES
EPI_ISL_18537504	RELAB - BIOGROUP - Plateau technique St Denis	National Reference Center for Viruses of Respiratory Infections, Institut Pasteur, Paris	Léa Avon, Marion Barbet, Marine Bernard, Emma Bezot, Angela Brisebarre, Océane Dehan, Flora Donati, Vanessa Guimaraes, Banujaa Jeyarajah, Florian Préjean, Yannis Rahou, Sylvie van der Werf, Frédéric Lemoine, Samar Berreira Ibraim, Jérôme Bourret, Kévin Da Silva, Maud Vanpeene, Vincent Enouf, Marie-Anne Rameix-Welti, Yanis CHAIB
EPI_ISL_18537505	RELAB - Laboratoire Biomer	National Reference Center for Viruses of Respiratory Infections, Institut Pasteur, Paris	Léa Avon, Marion Barbet, Marine Bernard, Emma Bezot, Angela Brisebarre, Océane Dehan, Flora Donati, Vanessa Guimaraes, Banujaa Jeyarajah, Florian Préjean, Yannis Rahou, Sylvie van der Werf, Frédéric Lemoine, Samar Berreira Ibraim, Jérôme Bourret, Kévin Da Silva, Maud Vanpeene, Vincent Enouf, Marie-Anne Rameix-Welti, Alexandra JACQUES
EPI_ISL_18537506, EPI_ISL_18537507, EPI_ISL_18537508, EPI_ISL_18537510, EPI_ISL_18537511, EPI_ISL_18537512	RELAB - BIOGROUP - Plateau technique St Denis	National Reference Center for Viruses of Respiratory Infections, Institut Pasteur, Paris	Léa Avon, Marion Barbet, Marine Bernard, Emma Bezot, Angela Brisebarre, Océane Dehan, Flora Donati, Vanessa Guimaraes, Banujaa Jeyarajah, Florian Préjean, Yannis Rahou, Sylvie van der Werf, Frédéric Lemoine, Samar Berreira Ibraim, Jérôme Bourret, Kévin Da Silva, Maud Vanpeene, Vincent Enouf, Marie-Anne Rameix-Welti, Yanis CHAIB
EPI_ISL_9690098, EPI_ISL_9690342, EPI_ISL_9691187, EPI_ISL_9691376	Ricardo Gutierrez Children's Hospital, Virology Laboratory, sequencing area.	Ricardo Gutierrez Children's Hospital, Virology Laboratory, sequencing area.	Acuña, Dolores; Goya, Stephanie; Nabaes Jodar, Mercedes S.; Grandis, Érica; María Cristina Alvarez López, María Elina Acevedo, Oscar Jacquez, Sofía Alexay, Mariángeles Barreda Fink, María Emilia Villegas, Raquel Barquez, Estela Chascón, Jorgelina Caruso, Karina Zacarías, Cristian Díaz, Oscar Luna, Cristian Turchiaro, Julián Cipelli, Guillermo Thomas, Carla Medina, Natalia Labarta. Mistchenko, Alicia S.; Viegas, Mariana.

We gratefully acknowledge the following Authors from the Originating laboratories responsible for obtaining the specimens, as well as the Submitting laboratories where the genome data were generated and shared via GISAID, on which this research is based.

All Submitters of data may be contacted directly via www.gisaid.org

Authors are sorted alphabetically.

Accession ID	Originating Laboratory	Submitting Laboratory	Authors
EPI_ISL_10914987	VIC, Victorian Infectious Diseases Reference Laboratory	WHO Collaborating Centre for Reference and Research on Influenza	Xiaomin Dong, Annette Alafaci,Yi-Mo Deng, Naomi Komadina, Ammar Aziz
EPI_ISL_10954009	Respiratory Virus Unit, Specialised Microbiology and Laboratories Directorate, UK Health Security Agency	Respiratory Virus Unit, Specialised Microbiology and Laboratories Directorate, UK Health Security Agency	Zambon M, Talts T, Ellis J, Miah S, Platt S
EPI_ISL_11050303	Phra Nakhon Si Ayutthaya Hospital	National Institute of Health, Department of Medical Sciences, Ministry of Public Health, Thailand	Siripaporn Phuygun; Pakorn Piromtong; Thanutsapa Thanadachakul; Natchaya Khiadsang; Sunthareeya Waicharoen; Malinee Chittaganpitch; Pilailuk Akkapaiboon Okada
EPI_ISL_11050304	Nakornping Hospital	National Institute of Health, Department of Medical Sciences, Ministry of Public Health, Thailand	Siripaporn Phuygun; Pakorn Piromtong; Thanutsapa Thanadachakul; Natchaya Khiadsang; Sunthareeya Waicharoen; Malinee Chittaganpitch; Pilailuk Akkapaiboon Okada
EPI_ISL_11055769	Rahima Moosa Mother and child Hospital	National Institute for Communicable Diseases of the National Health Laboratory Service	Amoako DG, Everatt J, Kekana D, Scheepers C, Mohale T, Ntuli N, Mahlangu B, Mnguni A, Ismail A, Bhiman JN, Wolter N
EPI_ISL_11055770	Edendale Gateway clinic	National Institute for Communicable Diseases of the National Health Laboratory Service	Amoako DG, Everatt J, Kekana D, Scheepers C, Mohale T, Ntuli N, Mahlangu B, Mnguni A, Ismail A, Bhiman JN, Wolter N
EPI_ISL_11055771	Rahima Moosa Mother and child Hospital	National Institute for Communicable Diseases of the National Health Laboratory Service	Amoako DG, Everatt J, Kekana D, Scheepers C, Mohale T, Ntuli N, Mahlangu B, Mnguni A, Ismail A, Bhiman JN, Wolter N
EPI_ISL_11055787	Agincourt clinic	National Institute for Communicable Diseases of the National Health Laboratory Service	Amoako DG, Everatt J, Kekana D, Scheepers C, Mohale T, Ntuli N, Mahlangu B, Mnguni A, Ismail A, Bhiman JN, Wolter N
EPI_ISL_11055792, EPI_ISL_11055793, EPI_ISL_11055794	Red cross childrens hospital	National Institute for Communicable Diseases of the National Health Laboratory Service	Amoako DG, Everatt J, Kekana D, Scheepers C, Mohale T, Ntuli N, Mahlangu B, Mnguni A, Ismail A, Bhiman JN, Wolter N
EPI_ISL_11055795	Klerksdorp	National Institute for Communicable Diseases of the National Health Laboratory Service	Amoako DG, Everatt J, Kekana D, Scheepers C, Mohale T, Ntuli N, Mahlangu B, Mnguni A, Ismail A, Bhiman JN, Wolter N
EPI_ISL_11055796	Jouberton clinic	National Institute for Communicable Diseases of the National Health Laboratory Service	Amoako DG, Everatt J, Kekana D, Scheepers C, Mohale T, Ntuli N, Mahlangu B, Mnguni A, Ismail A, Bhiman JN, Wolter N
EPI_ISL_11055797, EPI_ISL_11055798, EPI_ISL_11055801, EPI_ISL_11055802	Red cross childrens hospital	National Institute for Communicable Diseases of the National Health Laboratory Service	Amoako DG, Everatt J, Kekana D, Scheepers C, Mohale T, Ntuli N, Mahlangu B, Mnguni A, Ismail A, Bhiman JN, Wolter N
EPI_ISL_11055803	Edendale hospital	National Institute for Communicable Diseases of the National Health Laboratory Service	Amoako DG, Everatt J, Kekana D, Scheepers C, Mohale T, Ntuli N, Mahlangu B, Mnguni A, Ismail A, Bhiman JN, Wolter N
EPI_ISL_11055804	Mitchell's plain distric hospital	National Institute for Communicable Diseases of the National Health Laboratory Service	Amoako DG, Everatt J, Kekana D, Scheepers C, Mohale T, Ntuli N, Mahlangu B, Mnguni A, Ismail A, Bhiman JN, Wolter N
EPI_ISL_11055805	Klerksdorp	National Institute for Communicable Diseases of the National Health Laboratory Service	Amoako DG, Everatt J, Kekana D, Scheepers C, Mohale T, Ntuli N, Mahlangu B, Mnguni A, Ismail A, Bhiman JN, Wolter N
EPI_ISL_11428314	Respiratory Virus Unit, Reference Services, UK Health Security Agency	Reference Services, UK Health Security Agency	Zambon M, Talts T, Ellis J, Miah S, Platt S
EPI_ISL_11817018, EPI_ISL_11817022, EPI_ISL_11817029, EPI_ISL_11817031, EPI_ISL_11817033, EPI_ISL_11817034, EPI_ISL_11817035, EPI_ISL_11817037, EPI_ISL_11817040, EPI_ISL_11817044, EPI_ISL_11817047, EPI_ISL_11817048, EPI_ISL_11817050, EPI_ISL_11817052, EPI_ISL_11817054, EPI_ISL_11817055, EPI_ISL_11817057, EPI_ISL_11817058, EPI_ISL_11817059, EPI_ISL_11817060, EPI_ISL_11817063, EPI_ISL_11817064, EPI_ISL_11817067, EPI_ISL_11817068, EPI_ISL_11817073, EPI_ISL_11817074, EPI_ISL_11817077, EPI_ISL_11817079, EPI_ISL_11817081, EPI_ISL_11817084, EPI_ISL_11817085			
see above	QLD, Royal Brisbane and Woman's Hospital	WHO Collaborating Centre for Reference and Research on Influenza	Xiaomin Dong, Yi-Mo Deng, Ammar Aziz, Naomi Komadina
EPI_ISL_11817087, EPI_ISL_11817089, EPI_ISL_11817093, EPI_ISL_11817098	VIC, Royal Children's Hospital	WHO Collaborating Centre for Reference and Research on Influenza	Xiaomin Dong, Annette Alafaci,Yi-Mo Deng, Ammar Aziz, Naomi Komadina
EPI_ISL_11817110, EPI_ISL_11817111, EPI_ISL_11817112, EPI_ISL_11817113, EPI_ISL_11817114, EPI_ISL_11817115, EPI_ISL_11817116, EPI_ISL_11817117, EPI_ISL_11817118, EPI_ISL_11817119, EPI_ISL_11817120, EPI_ISL_11817121, EPI_ISL_11817122, EPI_ISL_11817123, EPI_ISL_11817124, EPI_ISL_11817125, EPI_ISL_11817126, EPI_ISL_11817127, EPI_ISL_11817128, EPI_ISL_11817129, EPI_ISL_11817130, EPI_ISL_11817131, EPI_ISL_11817132, EPI_ISL_11817133, EPI_ISL_11817134, EPI_ISL_11817135, EPI_ISL_11817136, EPI_ISL_11817137, EPI_ISL_11817138, EPI_ISL_11817139			
see above	VIC, Monash Medical Centre	WHO Collaborating Centre for Reference and Research on Influenza	Xiaomin Dong, Michelle Francis, Tony Korman, Yi-Mo Deng, Ammar Aziz, Naomi Komadina
EPI_ISL_12529654	Klerksdorp	National Institute for Communicable Diseases of the National Health Laboratory Service	Amoako DG, Everatt J, Kekana D, Scheepers C, Mohale T, Ntuli N, Mahlangu B, Mnguni A, Ismail A, Bhiman JN, Wolter N
EPI_ISL_12529655	Rahima Moosa Mother and child Hospital	National Institute for Communicable Diseases of the National Health Laboratory Service	Amoako DG, Everatt J, Kekana D, Scheepers C, Mohale T, Ntuli N, Mahlangu B, Mnguni A, Ismail A, Bhiman JN, Wolter N
EPI_ISL_12529656	Edendale hospital	National Institute for Communicable Diseases of the National Health Laboratory Service	Amoako DG, Everatt J, Kekana D, Scheepers C, Mohale T, Ntuli N, Mahlangu B, Mnguni A, Ismail A, Bhiman JN, Wolter N
EPI_ISL_12529657	Klerksdorp	National Institute for Communicable Diseases of the National Health Laboratory Service	Amoako DG, Everatt J, Kekana D, Scheepers C, Mohale T, Ntuli N, Mahlangu B, Mnguni A, Ismail A, Bhiman JN, Wolter N
EPI_ISL_12529658, EPI_ISL_12529659	Red cross childrens hospital	National Institute for Communicable Diseases of the National Health Laboratory Service	Amoako DG, Everatt J, Kekana D, Scheepers C, Mohale T, Ntuli N, Mahlangu B, Mnguni A, Ismail A, Bhiman JN, Wolter N
EPI_ISL_12529660	Edendale hospital	National Institute for Communicable Diseases of the National Health Laboratory Service	Amoako DG, Everatt J, Kekana D, Scheepers C, Mohale T, Ntuli N, Mahlangu B, Mnguni A, Ismail A, Bhiman JN, Wolter N
EPI_ISL_12544917	National Influenza Center, National Institute of Hygiene and Epidemiology (NIHE)	National Institute of Hygiene and Epidemiology (NIHE) National Influenza Centre, Virology Department	Ung Thi Hong Trang, Hoang Vu Mai Phuong, Nguyen Huy Hoang, Nguyen Le Khanh Hang, Le Thi Thanh, Nguyen Vu Son, Vuong Duc Cuong, Pham Thi Hien, Tran Thu Huong, Nguyen Co Thach, Nguyen Phuong Anh, Le Quynh Mai
EPI_ISL_12970400, EPI_ISL_12970402, EPI_ISL_12970404, EPI_ISL_12970406, EPI_ISL_12970410, EPI_ISL_12970424	Department of Virology, Research Institute for Tropical Medicine	RVDT/RVD/DVD/CDC	Lijuan Wang, Mayan U. Lumandas, Vina Lea Arguelles, Jonjee Morin, Roman Tatusov and Everardo Vega
EPI_ISL_13231399, EPI_ISL_13231406, EPI_ISL_13231411, EPI_ISL_13231414, EPI_ISL_13231416, EPI_ISL_13231420, EPI_ISL_13231421, EPI_ISL_13231423, EPI_ISL_13231424, EPI_ISL_13231426, EPI_ISL_13231427, EPI_ISL_13231430, EPI_ISL_13231431, EPI_ISL_13231432, EPI_ISL_13231433, EPI_ISL_13231437, EPI_ISL_13231438, EPI_ISL_13231440			
see above	Israel Central Virology laboratory, Ministry of Health	Israel Central Virology laboratory, Ministry of Health	Neta S. Zuckerman, Efrat Bucris, Miranda Geva, Hagar Morad, Or Zilbertzan, Ilana S. Fratty, Ital Nemet, Nofar Atari, Limor Kliker, Ella Mendelson, Michal Mandelboim
EPI_ISL_13469901, EPI_ISL_13469935, EPI_ISL_13469936, EPI_ISL_13469937, EPI_ISL_13469938, EPI_ISL_13469939, EPI_ISL_13469940, EPI_ISL_13469941, EPI_ISL_13469944, EPI_ISL_13469945, EPI_ISL_13469946, EPI_ISL_13470021, EPI_ISL_13470192, EPI_ISL_13472081			
see above	ICMR-National Institute of Virology Influenza	ICMR-National Institute of Virology Influenza	Varsha Potdar and NIC
EPI_ISL_13603192	ICMR-National Institute of Virology Influenza	ICMR-National Institute of Virology Influenza	Varsha Potdar and NIC team
EPI_ISL_13603193	ICMR-National Institute of Virology Influenza	ICMR-National Institute of Virology Influenza	Varsha Potdar and NIC Team
EPI_ISL_13603194	ICMR-National Institute of Virology Influenza	ICMR-National Institute of Virology Influenza	Varsha Potdar and NIC team
EPI_ISL_13603195, EPI_ISL_13603196, EPI_ISL_13603198, EPI_ISL_13603199, EPI_ISL_13603200, EPI_ISL_13603201, EPI_ISL_13603202, EPI_ISL_13603203	ICMR-National Institute of Virology Influenza	ICMR-National Institute of Virology Influenza	Varsha Potdar and NIC
EPI_ISL_14084066, EPI_ISL_14084067, EPI_ISL_14084068, EPI_ISL_14084070, EPI_ISL_14084072, EPI_ISL_14084073, EPI_ISL_14084074, EPI_ISL_14084075, EPI_ISL_14084076, EPI_ISL_14084077, EPI_ISL_14084078, EPI_ISL_14084079, EPI_ISL_14084080, EPI_ISL_14084082, EPI_ISL_14084085, EPI_ISL_14084086, EPI_ISL_14084087			
see above	Complexo Hospitalario Universitario de Vigo. Microbiology Department	Complexo Hospitalario Universitario de Vigo. Microbiology Department	Daviña, Carlos; Pizcueta, Juan; Perez, Sonia
EPI_ISL_1437543	Instituto Medico Platense	INEI-ANLIS "Carlos G. Malbrán". Servicio Virosis Respiratorias.	Elsa Baumeister, Andrea Pontoriero, Russo Mara, Martin Avaro, Estefania Benedetti, Ana Campos
EPI_ISL_1437553, EPI_ISL_1437555, EPI_ISL_1437557, EPI_ISL_1437559, EPI_ISL_1437561, EPI_ISL_1437562, EPI_ISL_1437564, EPI_ISL_1437573	Laboratorio de Salud Publica	INEI-ANLIS "Carlos G. Malbrán". Servicio Virosis Respiratorias.	Elsa Baumeister, Andrea Pontoriero, Russo Mara, Martin Avaro, Estefania Benedetti, Ana Campos
EPI_ISL_15055331, EPI_ISL_15055332, EPI_ISL_15055333, EPI_ISL_15055334, EPI_ISL_15055335, EPI_ISL_15055336, EPI_ISL_15055337, EPI_ISL_15055338, EPI_ISL_15055339, EPI_ISL_15055340, EPI_ISL_15055341, EPI_ISL_15055342, EPI_ISL_15055343, EPI_ISL_15055344, EPI_ISL_15055345, EPI_ISL_15055346, EPI_ISL_15055347, EPI_ISL_15055348, EPI_ISL_15055349, EPI_ISL_15055350, EPI_ISL_15055351, EPI_ISL_15055352, EPI_ISL_15055353, EPI_ISL_15055354, EPI_ISL_15055355, EPI_ISL_15055356, EPI_ISL_15055357, EPI_ISL_15055358, EPI_ISL_15067696, EPI_ISL_15067697, EPI_ISL_15067698, EPI_ISL_15067699, EPI_ISL_15067700, EPI_ISL_15067701, EPI_ISL_15067702, EPI_ISL_15067703, EPI_ISL_15067704, EPI_ISL_15067705, EPI_ISL_15067706, EPI_ISL_15067707, EPI_ISL_15067708, EPI_ISL_15067709, EPI_ISL_15067710, EPI_ISL_15067711, EPI_ISL_15067712, EPI_ISL_15067713, EPI_ISL_15067714, EPI_ISL_15067715, EPI_ISL_15067716, EPI_ISL_15067717, EPI_ISL_15067718, EPI_ISL_15067719, EPI_ISL_15067720, EPI_ISL_15067721, EPI_ISL_15067722, EPI_ISL_15067723			
see above	Virology Laboratory, Ricardo Gutiérrez Children's Hospital	Virology Laboratory, Ricardo Gutiérrez Children's Hospital	Acuña, Dolores; Goya, Stephanie; Nabaes Jodar, Mercedes S.; Lucion, M. Florencia; Juárez, María del Valle; Gentile, Ángela; Mistchenko, Alicia S.; Viegas, Mariana
EPI_ISL_15120784, EPI_ISL_15120787, EPI_ISL_15120788, EPI_ISL_15120789, EPI_ISL_15120790, EPI_ISL_15120791, EPI_ISL_15120792, EPI_ISL_15120793, EPI_ISL_15120794, EPI_ISL_15120795, EPI_ISL_15120796, EPI_ISL_15120797, EPI_ISL_15120798, EPI_ISL_15120799			
see above	National Institute of Hygiene, virology departement, National	Centers for Disease Control and Prevention, Coronavirus and	Megha Aggarwal, Lijuan Wang, Ji In Park, Amanda Smith and Everardo Vega

	Influenza Center	Other Respiratory Viruses Division	
EPI_ISL_15750158, EPI_ISL_15750159, EPI_ISL_15750160	Virology, Tehran University of Medical Sciences	Virology, Tehran University of Medical Sciences	Salimi,V., Tavakoli,F., Mokhtari-Azad,T., Ghavami,N.
EPI_ISL_15750192, EPI_ISL_15750193, EPI_ISL_15750194, EPI_ISL_15750195	Inserm UMR1137, Universite de Paris	Inserm UMR1137, Universite de Paris	Coppee,R., Chenane,H.R., Bridier-Nahmias,A., Tcherakian,C., Catherinot,E., Collin,G., Lebourgeois,S., Visseaux,B., Descamps,D., Vasse,M., Farfour,E., Coppee,R., Chenane,H.R., Bridier-Nahmias,A., Collin,G., Lebourgeois,S., Visseaux,B., Descamps,D.
EPI_ISL_15750201, EPI_ISL_15750202, EPI_ISL_15750203	Department of Clinical Laboratory, Children's Hospital of Fudan University	Department of Clinical Laboratory, Children's Hospital of Fudan University	Ran,J.
EPI_ISL_15750204, EPI_ISL_15750205, EPI_ISL_15750206, EPI_ISL_15750207, EPI_ISL_15750208, EPI_ISL_15750209, EPI_ISL_15750210, EPI_ISL_15750211, EPI_ISL_15750212, EPI_ISL_15750213, EPI_ISL_15750214, EPI_ISL_15750215, EPI_ISL_15750216, EPI_ISL_15750217, EPI_ISL_15750218, EPI_ISL_15750219, EPI_ISL_15750220, EPI_ISL_15750221, EPI_ISL_15750222, EPI_ISL_15750223, EPI_ISL_15750224, EPI_ISL_15750225, EPI_ISL_15750226, EPI_ISL_15750227, EPI_ISL_15750228, EPI_ISL_15750229, EPI_ISL_15750230, EPI_ISL_15750231, EPI_ISL_15750232, EPI_ISL_15750233, EPI_ISL_15750234, EPI_ISL_15750235, EPI_ISL_15750236, EPI_ISL_15750237, EPI_ISL_15750238, EPI_ISL_15750239, EPI_ISL_15750240, EPI_ISL_15750241, EPI_ISL_15750242, EPI_ISL_15750243, EPI_ISL_15750244, EPI_ISL_15750245, EPI_ISL_15750246			
see above	Department of Community Medicine, Faculty of Medicine	Department of Community Medicine, Faculty of Medicine	Malasao,R., Chaiut,W., Tantipetcharawan,W., Tongphung,R., Charoensri,N., Takarn,P., Sudjaritruk,T., Maneekarn,N., Malasao,R., Chaiut,W., Tantipetcharawan,W., Tongphung,R., Charoensri,N., Takarn,P., Sudjaritruk,T., Maneekarn,N.
EPI_ISL_15750299, EPI_ISL_15750300, EPI_ISL_15750301, EPI_ISL_15750302, EPI_ISL_15750303, EPI_ISL_15750304, EPI_ISL_15750305, EPI_ISL_15750306	WHO WPRO Regional Reference Measles/Rubella Laboratory, National Institute for Viral Disease Control and Prevention	WHO WPRO Regional Reference Measles/Rubella Laboratory, National Institute for Viral Disease Control and Prevention	Song,J., Zhang,Y., Song,J., Zhang,Y.
EPI_ISL_15750404, EPI_ISL_15750405, EPI_ISL_15750406, EPI_ISL_15750407, EPI_ISL_15750408, EPI_ISL_15750409, EPI_ISL_15750410, EPI_ISL_15750411, EPI_ISL_15750412, EPI_ISL_15750413, EPI_ISL_15750414, EPI_ISL_15750415, EPI_ISL_15750416, EPI_ISL_15750417, EPI_ISL_15750418, EPI_ISL_15750419, EPI_ISL_15750420, EPI_ISL_15750421, EPI_ISL_15750422, EPI_ISL_15750423, EPI_ISL_15750424, EPI_ISL_15750425, EPI_ISL_15750426, EPI_ISL_15750427, EPI_ISL_15750428, EPI_ISL_15750429, EPI_ISL_15750430, EPI_ISL_15750431, EPI_ISL_15750478, EPI_ISL_15750479, EPI_ISL_15750480, EPI_ISL_15750481, EPI_ISL_15750482, EPI_ISL_15750483, EPI_ISL_15750484, EPI_ISL_15750485, EPI_ISL_15750486, EPI_ISL_15750487, EPI_ISL_15750488, EPI_ISL_15750491, EPI_ISL_15750492, EPI_ISL_15750493, EPI_ISL_15750494, EPI_ISL_15750495, EPI_ISL_15750496, EPI_ISL_15750497, EPI_ISL_15750498, EPI_ISL_15750499, EPI_ISL_15750500, EPI_ISL_15750501, EPI_ISL_15750502, EPI_ISL_15750503, EPI_ISL_15750504, EPI_ISL_15750505, EPI_ISL_15750618, EPI_ISL_15750619, EPI_ISL_15750620, EPI_ISL_15750621, EPI_ISL_15750622			
see above	Department of Pediatrics, Center of Excellence in Clinical Virology	Department of Pediatrics, Center of Excellence in Clinical Virology	Thongpan,I.
EPI_ISL_15750901, EPI_ISL_15750902, EPI_ISL_15750903, EPI_ISL_15750904, EPI_ISL_15750905, EPI_ISL_15750906, EPI_ISL_15750907, EPI_ISL_15750908, EPI_ISL_15750909, EPI_ISL_15750910, EPI_ISL_15750911, EPI_ISL_15750912, EPI_ISL_15750913, EPI_ISL_15750914, EPI_ISL_15750915, EPI_ISL_15750916, EPI_ISL_15750917, EPI_ISL_15750918, EPI_ISL_15750919, EPI_ISL_15750920, EPI_ISL_15750921, EPI_ISL_15750922, EPI_ISL_15750923, EPI_ISL_15750924, EPI_ISL_15750925, EPI_ISL_15750926, EPI_ISL_15750927, EPI_ISL_15750928, EPI_ISL_15750929, EPI_ISL_15750930, EPI_ISL_15750931, EPI_ISL_15750932, EPI_ISL_15750933			
see above	Beijing Center for Disease Prevention and Control, Immunological Prevention	Beijing Center for Disease Prevention and Control, Immunological Prevention	Qin,L., Li,M., Huang,F.
EPI_ISL_15750941, EPI_ISL_15750942, EPI_ISL_15750943, EPI_ISL_15750944, EPI_ISL_15750945, EPI_ISL_15750946, EPI_ISL_15750947, EPI_ISL_15750948, EPI_ISL_15750949, EPI_ISL_15750950, EPI_ISL_15750951, EPI_ISL_15750952, EPI_ISL_15750953, EPI_ISL_15750954			
see above	Beijing Center for Disease Prevention and Control, Immunological Prevention	Beijing Center for Disease Prevention and Control, Immunological Prevention	Luo,Q., Li,M., Huang,F.
EPI_ISL_15751361, EPI_ISL_15751362, EPI_ISL_15751363, EPI_ISL_15751364, EPI_ISL_15751365, EPI_ISL_15751366, EPI_ISL_15751367, EPI_ISL_15751368, EPI_ISL_15751369, EPI_ISL_15751370, EPI_ISL_15751371, EPI_ISL_15751372, EPI_ISL_15751373, EPI_ISL_15751374, EPI_ISL_15751375, EPI_ISL_15751376, EPI_ISL_15751377, EPI_ISL_15751378, EPI_ISL_15751379, EPI_ISL_15751380, EPI_ISL_15751381, EPI_ISL_15751382, EPI_ISL_15751383, EPI_ISL_15751384, EPI_ISL_15751385, EPI_ISL_15751386, EPI_ISL_15751387, EPI_ISL_15751388			
see above	Microbiology, Hospices Civiles de Lyon	Microbiology, Hospices Civiles de Lyon	Vidoni,L.
EPI_ISL_15751422	Pathology, Stanford University School of Medicine	Pathology, Stanford University School of Medicine	Huang,C., Doan,T., Sahoo,M.K., Pinsky,B.A.
EPI_ISL_15751423, EPI_ISL_15751424, EPI_ISL_15751425, EPI_ISL_15751426, EPI_ISL_15751427, EPI_ISL_15751428, EPI_ISL_15751429, EPI_ISL_15751430	Molecular Virology, Johns Hopkins School of Medicine	Molecular Virology, Johns Hopkins School of Medicine	Fall A, Elawar F, Hodcroft EB, Jallow MM, Toure CT, Barry MA, Kiori DE, Sy S, Diaw Y, Goudiaby D, Niang MN, Dia N, Fall,A., Jallow,M.M., Toure,C.T., Kiori,D.E., sy,S., Diaw,Y., Goudiaby,D., Niang,M.N., Dia,N.
EPI_ISL_15751442, EPI_ISL_15751443, EPI_ISL_15751444, EPI_ISL_15751445, EPI_ISL_15751446, EPI_ISL_15751447, EPI_ISL_15751448, EPI_ISL_15751449, EPI_ISL_15751450, EPI_ISL_15751451, EPI_ISL_15751452, EPI_ISL_15751453, EPI_ISL_15751454, EPI_ISL_15751455, EPI_ISL_15751456, EPI_ISL_15751457, EPI_ISL_15751458, EPI_ISL_15751459, EPI_ISL_15751460, EPI_ISL_15751461, EPI_ISL_15751462, EPI_ISL_15751463, EPI_ISL_15751464, EPI_ISL_15751465, EPI_ISL_15751466, EPI_ISL_15751467, EPI_ISL_15751468, EPI_ISL_15751469, EPI_ISL_15751470, EPI_ISL_15751471, EPI_ISL_15751472, EPI_ISL_15751473, EPI_ISL_15751474, EPI_ISL_15751475, EPI_ISL_15751476, EPI_ISL_15751477, EPI_ISL_15751478, EPI_ISL_15751479, EPI_ISL_15751480, EPI_ISL_15751481, EPI_ISL_15751482, EPI_ISL_15751483, EPI_ISL_15751484, EPI_ISL_15751485, EPI_ISL_15751486, EPI_ISL_15751487, EPI_ISL_15751488, EPI_ISL_15751489, EPI_ISL_15751490, EPI_ISL_15751491, EPI_ISL_15751492, EPI_ISL_15751493, EPI_ISL_15751494, EPI_ISL_15751495, EPI_ISL_15751496, EPI_ISL_15751497, EPI_ISL_15751498, EPI_ISL_15751499, EPI_ISL_15751500, EPI_ISL_15751501, EPI_ISL_15751502, EPI_ISL_15751503, EPI_ISL_15751504, EPI_ISL_15751505, EPI_ISL_15751506, EPI_ISL_15751507, EPI_ISL_15751508, EPI_ISL_15751509, EPI_ISL_15751510, EPI_ISL_15751511, EPI_ISL_15751512, EPI_ISL_15751513, EPI_ISL_15751514, EPI_ISL_15751515, EPI_ISL_15751516, EPI_ISL_15751517, EPI_ISL_15751518, EPI_ISL_15751519, EPI_ISL_15751520, EPI_ISL_15751521, EPI_ISL_15751522, EPI_ISL_15751523, EPI_ISL_15751524, EPI_ISL_15751525, EPI_ISL_15751526, EPI_ISL_15751527, EPI_ISL_15751528, EPI_ISL_15751529, EPI_ISL_15751530, EPI_ISL_15751531, EPI_ISL_15751532, EPI_ISL_15751533, EPI_ISL_15751534, EPI_ISL_15751535, EPI_ISL_15751536, EPI_ISL_15751537, EPI_ISL_15751538, EPI_ISL_15751539, EPI_ISL_15751540, EPI_ISL_15751541, EPI_ISL_15751542, EPI_ISL_15751543, EPI_ISL_15751544, EPI_ISL_15751545, EPI_ISL_15751546, EPI_ISL_15751547, EPI_ISL_15751548, EPI_ISL_15751549, EPI_ISL_15751550, EPI_ISL_15751551, EPI_ISL_15751552, EPI_ISL_15751553, EPI_ISL_15751554, EPI_ISL_15751555, EPI_ISL_15751556, EPI_ISL_15751557, EPI_ISL_15751558, EPI_ISL_15751559, EPI_ISL_15751560, EPI_ISL_15751561, EPI_ISL_15751562, EPI_ISL_15751563, EPI_ISL_15751564, EPI_ISL_15751565, EPI_ISL_15751566, EPI_ISL_15751567, EPI_ISL_15751568, EPI_ISL_15751569, EPI_ISL_15751570, EPI_ISL_15751571, EPI_ISL_15751572, EPI_ISL_15751573, EPI_ISL_15751574, EPI_ISL_15751575, EPI_ISL_15751576, EPI_ISL_15751577, EPI_ISL_15751578, EPI_ISL_15751579			
see above	Microbiology, Hospices Civiles de Lyon	Microbiology, Hospices Civiles de Lyon	Vidoni,L.
EPI_ISL_15752267, EPI_ISL_15752268, EPI_ISL_15752274, EPI_ISL_15752303, EPI_ISL_15752310, EPI_ISL_15752314, EPI_ISL_15752319, EPI_ISL_15752321, EPI_ISL_15752322, EPI_ISL_15752327, EPI_ISL_15752339, EPI_ISL_15752341, EPI_ISL_15752342, EPI_ISL_15752346, EPI_ISL_15752348, EPI_ISL_15752349, EPI_ISL_15752350, EPI_ISL_15752352, EPI_ISL_15752356, EPI_ISL_15752357, EPI_ISL_15752358, EPI_ISL_15752360, EPI_ISL_15752361, EPI_ISL_15752362, EPI_ISL_15752363, EPI_ISL_15752366, EPI_ISL_15752373, EPI_ISL_15752374, EPI_ISL_15752375, EPI_ISL_15752376, EPI_ISL_15752377, EPI_ISL_15752380, EPI_ISL_15752381, EPI_ISL_15752382, EPI_ISL_15752383, EPI_ISL_15752384, EPI_ISL_15752385, EPI_ISL_15752387, EPI_ISL_15752388, EPI_ISL_15752389, EPI_ISL_15752390, EPI_ISL_15752391, EPI_ISL_15752392, EPI_ISL_15752394, EPI_ISL_15752395, EPI_ISL_15752396, EPI_ISL_15752397, EPI_ISL_15752398, EPI_ISL_15752399, EPI_ISL_15752400, EPI_ISL_15752401, EPI_ISL_15752402, EPI_ISL_15752403, EPI_ISL_15752404, EPI_ISL_15752405, EPI_ISL_15752406, EPI_ISL_15752407, EPI_ISL_15752408, EPI_ISL_15752411, EPI_ISL_15752412, EPI_ISL_15752414, EPI_ISL_15752415, EPI_ISL_15752416, EPI_ISL_15752417, EPI_ISL_15752418, EPI_ISL_15752419, EPI_ISL_15752420, EPI_ISL_15752421, EPI_ISL_15752423, EPI_ISL_15752427, EPI_ISL_15752428, EPI_ISL_15752429, EPI_ISL_15752430, EPI_ISL_15752431, EPI_ISL_15752433, EPI_ISL_15752434, EPI_ISL_15752435, EPI_ISL_15752442, EPI_ISL_15752443, EPI_ISL_15752446, EPI_ISL_15752447, EPI_ISL_15752448, EPI_ISL_15752449, EPI_ISL_15752450, EPI_ISL_15752451, EPI_ISL_15752452, EPI_ISL_15752453, EPI_ISL_15752454, EPI_ISL_15752455, EPI_ISL_15752456, EPI_ISL_15752461, EPI_ISL_15752462, EPI_ISL_15752463, EPI_ISL_15752466, EPI_ISL_15752467, EPI_ISL_15752473, EPI_ISL_15752474, EPI_ISL_15752476, EPI_ISL_15752477, EPI_ISL_15752478, EPI_ISL_15752479, EPI_ISL_15752480, EPI_ISL_15752481, EPI_ISL_15752482, EPI_ISL_15752484, EPI_ISL_15752486, EPI_ISL_15752487, EPI_ISL_15752488, EPI_ISL_15752489, EPI_ISL_15752491, EPI_ISL_15752492, EPI_ISL_15752496, EPI_ISL_15752498, EPI_ISL_15752505, EPI_ISL_15752507, EPI_ISL_15752509, EPI_ISL_15752511, EPI_ISL_15752515, EPI_ISL_15752516, EPI_ISL_15752522, EPI_ISL_15752524, EPI_ISL_15752531, EPI_ISL_15752533, EPI_ISL_15752534, EPI_ISL_15752535, EPI_ISL_15752537, EPI_ISL_15752539, EPI_ISL_15752542, EPI_ISL_15752547			
see above	Microbial Sciences, AstraZeneca	Microbial Sciences, AstraZeneca	Tabor,D.E., Fernandes,F., Langedijk,A.C., Wilkins,D., Lebbink,R.J., Tovchigrechko,A., Ruzin,A., Kragten-Tabatabaie,L., Jin,H., Esser,M.T., Bont,L.J., Abram,M.E., Tabor,D.
EPI_ISL_15752551, EPI_ISL_15752560, EPI_ISL_15752571, EPI_ISL_15752575, EPI_ISL_15752578, EPI_ISL_15752579, EPI_ISL_15752580, EPI_ISL_15752581, EPI_ISL_15752582, EPI_ISL_15752583, EPI_ISL_15752584, EPI_ISL_15752586, EPI_ISL_15752587, EPI_ISL_15752590, EPI_ISL_15752593, EPI_ISL_15752594, EPI_ISL_15752601, EPI_ISL_15752603, EPI_ISL_15752604, EPI_ISL_15752606, EPI_ISL_15752607, EPI_ISL_15752609, EPI_ISL_15752611, EPI_ISL_15752612, EPI_ISL_15752613, EPI_ISL_15752614, EPI_ISL_15752615, EPI_ISL_15752616, EPI_ISL_15752617, EPI_ISL_15752618, EPI_ISL_15752619, EPI_ISL_15752620, EPI_ISL_15752621, EPI_ISL_15752622, EPI_ISL_15752623, EPI_ISL_15752624, EPI_ISL_15752625, EPI_ISL_15752626, EPI_ISL_15752627, EPI_ISL_15752628, EPI_ISL_15752629, EPI_ISL_15752630, EPI_ISL_15752631, EPI_ISL_15752632, EPI_ISL_15752633, EPI_ISL_15752634, EPI_ISL_15752635, EPI_ISL_15752636, EPI_ISL_15752637, EPI_ISL_15752638, EPI_ISL_15752639, EPI_ISL_15752640, EPI_ISL_15752641, EPI_ISL_15752642, EPI_ISL_15752648, EPI_ISL_15752657, EPI_ISL_15752658, EPI_ISL_15752667, EPI_ISL_15752668, EPI_ISL_15752672, EPI_ISL_15752682, EPI_ISL_15752683, EPI_ISL_15752685, EPI_ISL_15752691, EPI_ISL_15752703, EPI_ISL_15752707, EPI_ISL_15752709, EPI_ISL_15752710, EPI_ISL_15752711, EPI_ISL_15752712, EPI_ISL_15752714			
see above	Pneumococcal Research Group, Infections and Immunity	Pneumococcal Research Group, Infections and Immunity	Anderson,J., Oeum,M., Verkolf,E., Licciardi,P.V., Mulholland,K., Nguyen,C., Chow,K., Waller,G., Costa,A.M., Daley,A., Crawford,N.W., Babl,F.E., Duke,T., Do,L.A.H., Wurzel,D., Anderson,J., Oeum,M., Verkolf,E., Licciardi,P.V., Mulholland,K.E., Nguyen,C., Chow,K., Waller,G., Costa,A.-M., Daley,A.J., Crawford,N., Babl,F., Duke,T., Do,L.A.H., Wurzel,D.
EPI_ISL_15752871, EPI_ISL_15752872, EPI_ISL_15752873, EPI_ISL_15752874, EPI_ISL_15752875, EPI_ISL_15752876, EPI_ISL_15752877, EPI_ISL_15752878, EPI_ISL_15752879, EPI_ISL_15752880, EPI_ISL_15752881, EPI_ISL_15752882, EPI_ISL_15752883, EPI_ISL_15752884, EPI_ISL_15752885, EPI_ISL_15752886, EPI_ISL_15752887, EPI_ISL_15752888, EPI_ISL_15752889, EPI_ISL_15752890, EPI_ISL_15752891, EPI_ISL_15752892, EPI_ISL_15752893, EPI_ISL_15752894, EPI_ISL_15752895, EPI_ISL_15752896, EPI_ISL_15752897, EPI_ISL_15752898, EPI_ISL_15752899, EPI_ISL_15752900, EPI_ISL_15752901, EPI_ISL_15752902, EPI_ISL_15752903, EPI_ISL_15752904, EPI_ISL_15752905, EPI_ISL_15752906, EPI_ISL_15752907, EPI_ISL_15752908, EPI_ISL_15752909, EPI_ISL_15752910, EPI_ISL_15752911, EPI_ISL_15752912, EPI_ISL_15752913, EPI_ISL_15752914, EPI_ISL_15752915, EPI_ISL_15752916, EPI_ISL_15752917, EPI_ISL_15752918, EPI_ISL_15752919, EPI_ISL_15752920, EPI_ISL_15752921, EPI_ISL_15752922, EPI_ISL_15752923, EPI_ISL_15752924, EPI_ISL_15752925, EPI_ISL_15752926, EPI_ISL_15752927, EPI_ISL_15752928, EPI_ISL_15752929, EPI_ISL_15752930, EPI_ISL_15752931, EPI_ISL_15752932, EPI_ISL_15752933, EPI_ISL_15752934, EPI_ISL_15752935, EPI_ISL_15752936, EPI_ISL_15752937, EPI_ISL_15752938, EPI_ISL_15752939, EPI_ISL_15752940, EPI_ISL_15752941, EPI_ISL_15752942, EPI_ISL_15752943, EPI_ISL_15752944, EPI_ISL_15752945, EPI_ISL_15752946, EPI_ISL_15752947, EPI_ISL_15752948, EPI_ISL_15752949, EPI_ISL_15752950, EPI_ISL_15752951, EPI_ISL_15752952, EPI_ISL_15752953, EPI_ISL_15752954, EPI_ISL_15752955, EPI_ISL_15752956, EPI_ISL_15752957, EPI_ISL_15752958, EPI_ISL_15752959, EPI_ISL_15752960, EPI_ISL_15752961, EPI_ISL_15752962, EPI_ISL_15752963, EPI_ISL_15752964, EPI_ISL_15752965, EPI_ISL_15752966, EPI_ISL_15752967, EPI_ISL_15752968, EPI_ISL_15752969, EPI_ISL_15752970, EPI_ISL_15752971, EPI_ISL_15752972, EPI_ISL_15752973, EPI_ISL_15752974, EPI_ISL_15752975, EPI_ISL_15752976			
see above	Epidemiology and Demography, KEMRI-Wellcome Trust Research Programme	Epidemiology and Demography, KEMRI-Wellcome Trust Research Programme	Kamau,E., Mutunga,M., Mwema,A., Agoti,C.N., Nokes,D.J., Kamau,E., Mutunga,M., Mwema,A., Agoti,C.N., Nokes,D.J.
EPI_ISL_15752977, EPI_ISL_15752979, EPI_ISL_15752980, EPI_ISL_15752981, EPI_ISL_15752982, EPI_ISL_15752985, EPI_ISL_15752986, EPI_ISL_15752987, EPI_ISL_15752988, EPI_ISL_15752990, EPI_ISL_15752992, EPI_ISL_15752993, EPI_ISL_15752994, EPI_ISL_15752995, EPI_ISL_15752996, EPI_ISL_15752997, EPI_ISL_15752998, EPI_ISL_15752999, EPI_ISL_15753000, EPI_ISL_15753001, EPI_ISL_15753005, EPI_ISL_15753007, EPI_ISL_15753008, EPI_ISL_15753009, EPI_ISL_15753011, EPI_ISL_15753012, EPI_ISL_15753014, EPI_ISL_15753015, EPI_ISL_15753016, EPI_ISL_15753017, EPI_ISL_15753019, EPI_ISL_15753021, EPI_ISL_15753022, EPI_ISL_15753023, EPI_ISL_15753026, EPI_ISL_15753028, EPI_ISL_15753029, EPI_ISL_15753030, EPI_ISL_15753035, EPI_ISL_15753036, EPI_ISL_15753040, EPI_ISL_15753041, EPI_ISL_15753043, EPI_ISL_15753044, EPI_ISL_15753045, EPI_ISL_15753048, EPI_ISL_15753049, EPI_ISL_15753050, EPI_ISL_15753051, EPI_ISL_15753052, EPI_ISL_15753054, EPI_ISL_15753056, EPI_ISL_15753058, EPI_ISL_15753059, EPI_ISL_15753060, EPI_ISL_15753061, EPI_ISL_15753062, EPI_ISL_15753064, EPI_ISL_15753066, EPI_ISL_15753068, EPI_ISL_15753069, EPI_ISL_15753072, EPI_ISL_15753073, EPI_ISL_15753074, EPI_ISL_15753075, EPI_ISL_15753076, EPI_ISL_15753077, EPI_ISL_15753078, EPI_ISL_15753079, EPI_ISL_15753080, EPI_ISL_15753083, EPI_ISL_15753084, EPI_ISL_15753086, EPI_ISL_15753087, EPI_ISL_15753088, EPI_ISL_15753092, EPI_ISL_15753093, EPI_ISL_15753094, EPI_ISL_15753097, EPI_ISL_15753098, EPI_ISL_15753099, EPI_ISL_15753101, EPI_ISL_15753103, EPI_ISL_15753104, EPI_ISL_15753105, EPI_ISL_15753107, EPI_ISL_15753109, EPI_ISL_15753110, EPI_ISL_15753111, EPI_ISL_15753112, EPI_ISL_15753115, EPI_ISL_15753116, EPI_ISL_15753117, EPI_ISL_15753119, EPI_ISL_15753122, EPI_ISL_15753124, EPI_ISL_15753127, EPI_ISL_15753129, EPI_ISL_15753132, EPI_ISL_15753133, EPI_ISL_15753134, EPI_ISL_15753135, EPI_ISL_15753137, EPI_ISL_15753139, EPI_ISL_15753140, EPI_ISL_15753141, EPI_ISL_15753142, EPI_ISL_15753144, EPI_ISL_15753145, EPI_ISL_15753146, EPI_ISL_15753147, EPI_ISL_15753148, EPI_ISL_15753150, EPI_ISL_15753151, EPI_ISL_15753152, EPI_ISL_15753153, EPI_ISL_15753155, EPI_ISL_15753158, EPI_ISL_15753161, EPI_ISL_15753164, EPI_ISL_15753165, EPI_ISL_15753166, EPI_ISL_15753167, EPI_ISL_15753168, EPI_ISL_15753170, EPI_ISL_15753171, EPI_ISL_15753172, EPI_ISL_15753175, EPI_ISL_15753178, EPI_ISL_15753179, EPI_ISL_15753181, EPI_ISL_15753182, EPI_ISL_15753184, EPI_ISL_15753185, EPI_ISL_15753186, EPI_ISL_15753188, EPI_ISL_15753189, EPI_ISL_15753192, EPI_ISL_15753193, EPI_ISL_15753195, EPI_ISL_15753196, EPI_ISL_15753198, EPI_ISL_15753201, EPI_ISL_15753202, EPI_ISL_15753203, EPI_ISL_15753204, EPI_ISL_15753205, EPI_ISL_15753208, EPI_ISL_15753209, EPI_ISL_15753210, EPI_ISL_15753211, EPI_ISL_15753212, EPI_ISL_15753213, EPI_ISL_15753214, EPI_ISL_15753217, EPI_ISL_15753219, EPI_ISL_15753223, EPI_ISL_15753224, EPI_ISL_15753225, EPI_ISL_15753228, EPI_ISL_15753230, EPI_ISL_15753231, EPI_ISL_15753232, EPI_ISL_15753233, EPI_ISL_15753234, EPI_ISL_15753237, EPI_ISL_15753238, EPI_ISL_15753239, EPI_ISL_15753241, EPI_ISL_15753243, EPI_ISL_15753245, EPI_ISL_15753246, EPI_ISL_15753247, EPI_ISL_15753248, EPI_ISL_15753250			
see above	Department of Paediatrics, University of Oxford	Department of Paediatrics, University of Oxford	Lin,G.-L., Drysdale,S.B., Snape,M.D., O'Connor,D., Brown,A., MacIntyre-Cockett,G., Mellado-Gomez,E., de Cesare,M., Bonsall,D., Ansari,M.A., Oner,D., Aerssens,J., Butler,C., Bont,L., Openshaw,P., Martinon-Torres,F., Nair,H., Bowden,R., Golubchik,T., Pollard,A.J., Lin,G.-L.
EPI_ISL_15753571	Eurasian Institute of zoonotic infections, The Federal Research Center of Fundamental and Translational Medicine	Eurasian Institute of zoonotic infections, The Federal Research Center of Fundamental and Translational Medicine	Dubovitskiy,N.A., Sobolev,I.A., Kurskaya,O.G., Anoshina,A.V., Leonova,N.V., Murashkina,T.A., Solomatina,M.V., Derko,A.A., Saroyan,T.A., Kabilov,M.R., Tupikin,A.E., Sharshov,K.A., Shestopalov,A.M.
EPI_ISL_15753572, EPI_ISL_15753573	Eurasian Institute of zoonotic infections, The Federal Research Center of Fundamental and Translational Medicine	Eurasian Institute of zoonotic infections, The Federal Research Center of Fundamental and Translational Medicine	Dubovitskiy,N.A., Sobolev,I.A., Kurskaya,O.G., Simkina,O.A., Komissarova,T.V., Murashkina,T.A., Solomatina,M.V., Derko,A.A., Saroyan,T.A., Kabilov,M.R., Tupikin,A.E., Sharshov,K.A., Shestopalov,A.M.
EPI_ISL_15753578, EPI_ISL_15753579, EPI_ISL_15753580	Eurasian Institute of zoonotic infections, The Federal Research Center of Fundamental and Translational Medicine	Eurasian Institute of zoonotic infections, The Federal Research Center of Fundamental and Translational Medicine	Dubovitskiy,N.A., Sobolev,I.A., Kurskaya,O.G., Anoshina,A.V., Leonova,N.V., Murashkina,T.A., Solomatina,M.V., Derko,A.A., Saroyan,T.A., Kabilov,M.R., Tupikin,A.E., Sharshov,K.A., Shestopalov,A.M.
EPI_ISL_15772002, EPI_ISL_15772004, EPI_ISL_15772006, EPI_ISL_15772008, EPI_ISL_15772009, EPI_ISL_15772011, EPI_ISL_15772013, EPI_ISL_15772014, EPI_ISL_15772016, EPI_ISL_15772018, EPI_ISL_15772020, EPI_ISL_15772022, EPI_ISL_15772024, EPI_ISL_15772026, EPI_ISL_15772027, EPI_ISL_15772029, EPI_ISL_15772031, EPI_ISL_15772033, EPI_ISL_15772034, EPI_ISL_15772036, EPI_ISL_15772038, EPI_ISL_15772040, EPI_ISL_15772041, EPI_ISL_15772043, EPI_ISL_15772045, EPI_ISL_15772046, EPI_ISL_15772048, EPI_ISL_15772050, EPI_ISL_15772052, EPI_ISL_15772054, EPI_ISL_15772056, EPI_ISL_15772057, EPI_ISL_15772059, EPI_ISL_15772061, EPI_ISL_15772063, EPI_ISL_15772064, EPI_ISL_15772066, EPI_ISL_15772068, EPI_ISL_15772070, EPI_ISL_15772071, EPI_ISL_15772073, EPI_ISL_15772075, EPI_ISL_15772076, EPI_ISL_15772078, EPI_ISL_15772080, EPI_ISL_15772081, EPI_ISL_15772083, EPI_ISL_15772085, EPI_ISL_15772087, EPI_ISL_15772089, EPI_ISL_15772091, EPI_ISL_15772093, EPI_ISL_15772094, EPI_ISL_15772096, EPI_ISL_15772098, EPI_ISL_15772100, EPI_ISL_15772102, EPI_ISL_15772104, EPI_ISL_15772106, EPI_ISL_15772107, EPI_ISL_15772109, EPI_ISL_15772111, EPI_ISL_15772114, EPI_ISL_15772115, EPI_ISL_15772117, EPI_ISL_15772119, EPI_ISL_15772121, EPI_ISL_15772123, EPI_ISL_15772124, EPI_ISL_15772126, EPI_ISL_15772128, EPI_ISL_15772130, EPI_ISL_15772132, EPI_ISL_15772134, EPI_ISL_15772135, EPI_ISL_15772138, EPI_ISL_15772139, EPI_ISL_15772141, EPI_ISL_15772143, EPI_ISL_15772145, EPI_ISL_15772147, EPI_ISL_15772149, EPI_ISL_15772151, EPI_ISL_15772153, EPI_ISL_15772155, EPI_ISL_15772156, EPI_ISL_15772158, EPI_ISL_15772160, EPI_ISL_15772162, EPI_ISL_15772164, EPI_ISL_15772165, EPI_ISL_15772167, EPI_ISL_15772169, EPI_ISL_15772172, EPI_ISL_15772173, EPI_ISL_15772175, EPI_ISL_15772177, EPI_ISL_15772179, EPI_ISL_15772181, EPI_ISL_15772182, EPI_ISL_15772184, EPI_ISL_15772186, EPI_ISL_15772188, EPI_ISL_15772189			
see above	Pathology and Laboratory Medicine, Children's Mercy	Pathology and Laboratory Medicine, Children's Mercy	Adhikari,B., Hassan,F., Harrison,C., Dien Bard,J., Dunn,J., Kehl,S. and Selvarangan,R.
EPI_ISL_15773985, EPI_ISL_15773988, EPI_ISL_15773989, EPI_ISL_15773996, EPI_ISL_15774002, EPI_ISL_15774003, EPI_ISL_15774004, EPI_ISL_15774005, EPI_ISL_15774019, EPI_ISL_15774028, EPI_ISL_15774034, EPI_ISL_15774036, EPI_ISL_15774037, EPI_ISL_15774040, EPI_ISL_15774041, EPI_ISL_15774044, EPI_ISL_15774048, EPI_ISL_15774051, EPI_ISL_15774052, EPI_ISL_15774055, EPI_ISL_15774057, EPI_ISL_15774061, EPI_ISL_15774069			
see above	QLD, Royal Brisbane and Woman's Hospital	WHO Collaborating Centre for Reference and Research on Influenza	Xiaomin Dong, Yi-Mo Deng, Ammar Aziz, Naomi Komadina
EPI_ISL_15820244, EPI_ISL_15820245, EPI_ISL_15820246, EPI_ISL_15820247	Hygiene, IRCCS Ospedale Policlinico San Martino	Hygiene, IRCCS Ospedale Policlinico San Martino	Stefanelli,F., Domnich,A., Bruzzone,B., Trombetta,C.-S., Ricucci,V., Orsi,A., Icardi,G.
EPI_ISL_15820269	Tokyo Metropolitan Institute of Public Health, Department of Microbiology	Tokyo Metropolitan Institute of Public Health, Department of Microbiology	Kasuya,F., Mori,K., Suzuki,J., Sadamasu,K., Kasuya,F., Mori,K., Suzuki,J., Sadamasu,K.
EPI_ISL_15895059	Laboratorio Central de Saude Publica do Estado do Rio Grande do Sul (LACEN/RS)	Laboratory of Respiratory Viruses and Measles, Oswaldo Cruz Institute, FIOCRUZ	Paola Resende, Fernando Motta, Elisa Cavalcante Pereira, Bruna Mendonça da Silva, Jéssica Graça Macedo de Carvalho, Larissa Macedo Pinto, Victor Guimaraes, Ighor Leonardo Arantes, Leticia Scalioni, Anderson Brandao Leite, Marilda Siqueira on behalf of the Fiocruz COVID-19 Genomic Surveillance Network

EPI_ISL_15895128	Universidade Federal de São Paulo, Departamento de Medicina, Disciplina de Doenças Infecciosas e Parasitárias. Laboratório de Virologia	Laboratory of Respiratory Viruses and Measles, Oswaldo Cruz Institute, FIOCRUZ	Paola Resende, Fernando Motta, Elisa Cavalcante Pereira, Ighor Arantes, Bruna Mendonça da Silva, Jéssica Graça Macedo de Carvalho, Larissa Macedo Pinto, Victor Guimaraes, Leticia Scalioni, Ana Helena Perosa, Gabriela Rodrigues Barbosa, Nancy Bellei e Marilda Siqueira on behalf of the Fiocruz COVID-19 Genomic Surveillance Network
EPI_ISL_16003692, EPI_ISL_16003693, EPI_ISL_16003694, EPI_ISL_16003695, EPI_ISL_16003696, EPI_ISL_16003697, EPI_ISL_16003698, EPI_ISL_16003699, EPI_ISL_16003700, EPI_ISL_16003701, EPI_ISL_16003702, EPI_ISL_16003703, EPI_ISL_16003704, EPI_ISL_16003705, EPI_ISL_16003706, EPI_ISL_16003707, EPI_ISL_16003709, EPI_ISL_16003710, EPI_ISL_16003711, EPI_ISL_16003712			
see above	Niigata University (IDRC) Infectious Diseases Research Center	Niigata University (IDRC) Infectious Diseases Research Center	Sayaka Yoshioka, Wint Wint Phyu, Irina Chon, Reiko Saito
EPI_ISL_16003713	Niigata University (IDRC) Infectious Diseases Research Center	vNiigata University (IDRC) Infectious Diseases Research Center	Sayaka Yoshioka, Wint Wint Phyu, Irina Chon, Reiko Saito
EPI_ISL_16003718, EPI_ISL_16003719, EPI_ISL_16003720, EPI_ISL_16003721, EPI_ISL_16003722, EPI_ISL_16003724, EPI_ISL_16003725, EPI_ISL_16003726, EPI_ISL_16003727, EPI_ISL_16003728, EPI_ISL_16003797, EPI_ISL_16003798, EPI_ISL_16003799, EPI_ISL_16003800, EPI_ISL_16003802, EPI_ISL_16003804, EPI_ISL_16003824, EPI_ISL_16003826, EPI_ISL_16003828, EPI_ISL_16003830, EPI_ISL_16003832, EPI_ISL_16003833, EPI_ISL_16003880, EPI_ISL_16003882, EPI_ISL_16003885, EPI_ISL_16003887, EPI_ISL_16003889, EPI_ISL_16003891, EPI_ISL_16003894, EPI_ISL_16003895, EPI_ISL_16003897, EPI_ISL_16003898, EPI_ISL_16003904, EPI_ISL_16003905, EPI_ISL_16004072, EPI_ISL_16013972, EPI_ISL_16013973, EPI_ISL_16013974, EPI_ISL_16013975, EPI_ISL_16013976, EPI_ISL_16013977, EPI_ISL_16013978, EPI_ISL_16013979, EPI_ISL_16013980, EPI_ISL_16013981, EPI_ISL_16013983, EPI_ISL_16013984, EPI_ISL_16013985, EPI_ISL_16013988, EPI_ISL_16013989, EPI_ISL_16013991, EPI_ISL_16013992, EPI_ISL_16013994, EPI_ISL_16014111, EPI_ISL_16014366, EPI_ISL_16014367, EPI_ISL_16014443, EPI_ISL_16014444, EPI_ISL_16014446, EPI_ISL_16014447, EPI_ISL_16014528, EPI_ISL_16014533, EPI_ISL_16014534, EPI_ISL_16014540, EPI_ISL_16014541, EPI_ISL_16014542, EPI_ISL_16014576, EPI_ISL_16014577, EPI_ISL_16014578			
see above	Niigata University (IDRC) Infectious Diseases Research Center	Niigata University (IDRC) Infectious Diseases Research Center	Sayaka Yoshioka, Wint Wint Phyu, Irina Chon, Reiko Saito
EPI_ISL_16051892, EPI_ISL_16051893, EPI_ISL_16051894, EPI_ISL_16051895, EPI_ISL_16051896, EPI_ISL_16051897, EPI_ISL_16051898, EPI_ISL_16051899, EPI_ISL_16051900, EPI_ISL_16051918, EPI_ISL_16051919, EPI_ISL_16051920, EPI_ISL_16051921, EPI_ISL_16051922, EPI_ISL_16051923, EPI_ISL_16051924, EPI_ISL_16051925, EPI_ISL_16051926, EPI_ISL_16051927, EPI_ISL_16051935, EPI_ISL_16051936, EPI_ISL_16051937, EPI_ISL_16051938, EPI_ISL_16051939, EPI_ISL_16051940, EPI_ISL_16051941			
see above	National Influenza Center, National Institute of Hygiene and Epidemiology (NIHE)	National Influenza Center, National Institute of Hygiene and Epidemiology (NIHE)	Ung Thi Hong Trang, Hoang Vu Mai Phuong, Nguyen Huy Hoang, Nguyen Le Khanh Hang, Le Thi Quynh Mai
EPI_ISL_16132315, EPI_ISL_16132317, EPI_ISL_16132318, EPI_ISL_16132319, EPI_ISL_16132320, EPI_ISL_16132322, EPI_ISL_16132323, EPI_ISL_16132324, EPI_ISL_16132327	University of WashingtonLaboratory Medicine and Pathology, UW Medicine	University of WashingtonLaboratory Medicine and Pathology, UW Medicine	Goya,S., Sereewit,J., Pfalmer,D., Nguyen,T., Sobolik,E.B. and Greninger,A.L.
EPI_ISL_16289246, EPI_ISL_16289248, EPI_ISL_16289249, EPI_ISL_16289253, EPI_ISL_16289271, EPI_ISL_16289279, EPI_ISL_16289294	National Influenza Center, National Institute of Hygiene and Epidemiology (NIHE)	National Influenza Center, National Institute of Hygiene and Epidemiology (NIHE)	Ung Thi Hong Trang, Hoang Vu Mai Phuong, Nguyen Huy Hoang, Nguyen Le Khanh Hang, Le Thi Quynh Mai
EPI_ISL_1647488	Instituto Nacional de Saúde	WHO Influenza Centre for Reference and Research on Influenza	Angela Todd, Yi-Mo Deng, Almiro Rogerio Tivane, Naomi Komadina
EPI_ISL_1647490, EPI_ISL_1647491, EPI_ISL_1647492, EPI_ISL_1647493, EPI_ISL_1647494, EPI_ISL_1647495, EPI_ISL_1647496, EPI_ISL_1647497, EPI_ISL_1647498, EPI_ISL_1647499, EPI_ISL_1647500, EPI_ISL_1647501, EPI_ISL_1647502, EPI_ISL_1647503, EPI_ISL_1647504, EPI_ISL_1647505, EPI_ISL_1647506, EPI_ISL_1647507, EPI_ISL_1647508, EPI_ISL_1647509, EPI_ISL_1647510, EPI_ISL_1647511, EPI_ISL_1647512, EPI_ISL_1647513, EPI_ISL_1647514, EPI_ISL_1647515, EPI_ISL_1647516, EPI_ISL_1647517, EPI_ISL_1647518, EPI_ISL_1647519, EPI_ISL_1647520, EPI_ISL_1647521, EPI_ISL_1647522, EPI_ISL_1647523, EPI_ISL_1647524, EPI_ISL_1647525, EPI_ISL_1647526, EPI_ISL_1647527, EPI_ISL_1647528, EPI_ISL_1647529, EPI_ISL_1647530, EPI_ISL_1647531, EPI_ISL_1647532, EPI_ISL_1647533, EPI_ISL_1647534, EPI_ISL_1647535, EPI_ISL_1647536, EPI_ISL_1647537, EPI_ISL_1647538, EPI_ISL_1647539, EPI_ISL_1647540, EPI_ISL_1647541, EPI_ISL_1647542, EPI_ISL_1647543, EPI_ISL_1647544, EPI_ISL_1647545, EPI_ISL_1647546, EPI_ISL_1647547, EPI_ISL_1647548, EPI_ISL_1647549, EPI_ISL_1647550, EPI_ISL_1647551, EPI_ISL_1647552, EPI_ISL_1647553, EPI_ISL_1647554, EPI_ISL_1647555, EPI_ISL_1647556, EPI_ISL_1647557, EPI_ISL_1647558, EPI_ISL_1647559, EPI_ISL_1647560, EPI_ISL_1647561, EPI_ISL_1647562, EPI_ISL_1647563, EPI_ISL_1647564, EPI_ISL_1647565, EPI_ISL_1647566, EPI_ISL_1647567, EPI_ISL_1647568, EPI_ISL_1647569, EPI_ISL_1647570, EPI_ISL_1647571, EPI_ISL_1647572, EPI_ISL_1647573, EPI_ISL_1647574, EPI_ISL_1647575, EPI_ISL_1647576, EPI_ISL_1647577, EPI_ISL_1647578, EPI_ISL_1647579, EPI_ISL_1647580, EPI_ISL_1647581, EPI_ISL_1647582, EPI_ISL_1647583, EPI_ISL_1647584, EPI_ISL_1647585, EPI_ISL_1647586, EPI_ISL_1647587, EPI_ISL_1647588, EPI_ISL_1647589, EPI_ISL_1647590, EPI_ISL_1647591, EPI_ISL_1647592, EPI_ISL_1647593, EPI_ISL_1647594, EPI_ISL_1647595, EPI_ISL_1647596, EPI_ISL_1647597, EPI_ISL_1647598, EPI_ISL_1647599, EPI_ISL_1647600			
see above	Respiratory Virus Unit, National Infection Service, Public Health England	National Infection Service, Public Health England	Zambon M, Talts T, Ellis J, Miah S, Platt S
EPI_ISL_16533855, EPI_ISL_16533857, EPI_ISL_16533858	Center for Virology, Medical University of Vienna	Center for Virology, Medical University of Vienna	Jeremy V. Camp, Monika Redlberger-Fritz
EPI_ISL_1653993, EPI_ISL_1653994, EPI_ISL_1653995, EPI_ISL_1653996, EPI_ISL_1653997, EPI_ISL_1653998, EPI_ISL_1653999	Royal Children's Hospital	WHO Influenza Centre for Reference and Research on Influenza	Angela Todd, Yi-Mo Deng, Annette Alafaci, Naomi Komadina
EPI_ISL_16681177, EPI_ISL_16681189, EPI_ISL_16681194	Children's Hospital of Fudan University	Children's Hospital of Fudan University	Jia,R.
EPI_ISL_16681195	Kenya Medical Research Institute-Wellcome Trust	Kenya Medical Research Institute-Wellcome Trust	Lambisia,A.W., Makori,T.O., Mutunga,M., Lewa,C., Githinji,G., Agoti,C.N. and Nokes,J.D.
EPI_ISL_16681203	Children's Hospital of Fudan University	Children's Hospital of Fudan University	Jia,R.
EPI_ISL_16681206	King Saud University	King Saud University	Alzayed,R.M., Aziz,I.M., Dudin,G.A., Alsaleh,A.N., Ahmed,A.A., Hussain,T. and Almajhdi,F.N.
EPI_ISL_16681207	Children's Hospital of Fudan University	Children's Hospital of Fudan University	Jia,R.
EPI_ISL_16681208	Kenya Medical Research Institute-Wellcome Trust	Kenya Medical Research Institute-Wellcome Trust	Lambisia,A.W., Makori,T.O., Mutunga,M., Lewa,C., Githinji,G., Agoti,C.N. and Nokes,J.D.
EPI_ISL_16681216, EPI_ISL_16681224	Children's Hospital of Fudan University	Children's Hospital of Fudan University	Jia,R.
EPI_ISL_16681225	King Saud University	King Saud University	Alzayed,R.M., Aziz,I.M., Dudin,G.A., Alsaleh,A.N., Ahmed,A.A. and Almajhdi,F.N.
EPI_ISL_16681227	Kenya Medical Research Institute-Wellcome Trust	Kenya Medical Research Institute-Wellcome Trust	Lambisia,A.W., Makori,T.O., Mutunga,M., Lewa,C., Githinji,G., Agoti,C.N. and Nokes,J.D.
EPI_ISL_16681228, EPI_ISL_16681234, EPI_ISL_16681245	Children's Hospital of Fudan University	Children's Hospital of Fudan University	Jia,R.
EPI_ISL_16681251, EPI_ISL_16681252, EPI_ISL_16681253	Kenya Medical Research Institute-Wellcome Trust	Kenya Medical Research Institute-Wellcome Trust	Lambisia,A.W., Makori,T.O., Mutunga,M., Lewa,C., Githinji,G., Agoti,C.N. and Nokes,J.D.
EPI_ISL_16681255, EPI_ISL_16681264	Children's Hospital of Fudan University	Children's Hospital of Fudan University	Jia,R.
EPI_ISL_16681265, EPI_ISL_16681267, EPI_ISL_16681269, EPI_ISL_16681276, EPI_ISL_16681277	Kenya Medical Research Institute-Wellcome Trust	Kenya Medical Research Institute-Wellcome Trust	Lambisia,A.W., Makori,T.O., Mutunga,M., Lewa,C., Githinji,G., Agoti,C.N. and Nokes,J.D.
EPI_ISL_16681278	Children's Hospital of Fudan University	Children's Hospital of Fudan University	Jia,R.
EPI_ISL_16681279, EPI_ISL_16681282, EPI_ISL_16681290, EPI_ISL_16681291, EPI_ISL_16681292	Kenya Medical Research Institute-Wellcome Trust	Kenya Medical Research Institute-Wellcome Trust	Lambisia,A.W., Makori,T.O., Mutunga,M., Lewa,C., Githinji,G., Agoti,C.N. and Nokes,J.D.
EPI_ISL_16681294	Children's Hospital of Fudan University	Children's Hospital of Fudan University	Jia,R.
EPI_ISL_16681303, EPI_ISL_16681305, EPI_ISL_16681307, EPI_ISL_16681314, EPI_ISL_16681315, EPI_ISL_16681316, EPI_ISL_16681317, EPI_ISL_16681318, EPI_ISL_16681329, EPI_ISL_16681330, EPI_ISL_16681331, EPI_ISL_16681332			
see above	Kenya Medical Research Institute-Wellcome Trust	Kenya Medical Research Institute-Wellcome Trust	Lambisia,A.W., Makori,T.O., Mutunga,M., Lewa,C., Githinji,G., Agoti,C.N. and Nokes,J.D.
EPI_ISL_16681335	King Saud University	King Saud University	Alzayed,R.M., Aziz,I.M., Dudin,G.A., Alsaleh,A.N., Ahmed,A.A., Hussain,T. and Almajhdi,F.N.
EPI_ISL_16681342, EPI_ISL_16681343, EPI_ISL_16681345, EPI_ISL_16681347, EPI_ISL_16681350	Kenya Medical Research Institute-Wellcome Trust	Kenya Medical Research Institute-Wellcome Trust	Lambisia,A.W., Makori,T.O., Mutunga,M., Lewa,C., Githinji,G., Agoti,C.N. and Nokes,J.D.
EPI_ISL_16681351	Children's Hospital of Fudan University	Children's Hospital of Fudan University	Jia,R.
EPI_ISL_16681361	King Saud University	King Saud University	Alzayed,R.M., Aziz,I.M., Dudin,G.A., Alsaleh,A.N., Ahmed,A.A., Hussain,T. and Almajhdi,F.N.
EPI_ISL_16681364, EPI_ISL_16681368	Children's Hospital of Fudan University	Children's Hospital of Fudan University	Jia,R.
EPI_ISL_16681369	Kenya Medical Research Institute-Wellcome Trust	Kenya Medical Research Institute-Wellcome Trust	Lambisia,A.W., Makori,T.O., Mutunga,M., Lewa,C., Githinji,G., Agoti,C.N. and Nokes,J.D.
EPI_ISL_16681378, EPI_ISL_16681392	Children's Hospital of Fudan University	Children's Hospital of Fudan University	Jia,R.
EPI_ISL_16681398	Kenya Medical Research Institute-Wellcome Trust	Kenya Medical Research Institute-Wellcome Trust	Lambisia,A.W., Makori,T.O., Mutunga,M., Lewa,C., Githinji,G., Agoti,C.N. and Nokes,J.D.
EPI_ISL_16681399	Children's Hospital of Fudan University	Children's Hospital of Fudan University	Jia,R.
EPI_ISL_16681410	King Saud University	King Saud University	Alzayed,R.M., Aziz,I.M., Dudin,G.A., Alsaleh,A.N., Ahmed,A.A., Hussain,T. and Almajhdi,F.N.
EPI_ISL_16681411, EPI_ISL_16681416	Children's Hospital of Fudan University	Children's Hospital of Fudan University	Jia,R.
EPI_ISL_16681417	Kenya Medical Research Institute-Wellcome Trust	Kenya Medical Research Institute-Wellcome Trust	Lambisia,A.W., Makori,T.O., Mutunga,M., Lewa,C., Githinji,G., Agoti,C.N. and Nokes,J.D.
EPI_ISL_16708973, EPI_ISL_16708974, EPI_ISL_16708978, EPI_ISL_16708979, EPI_ISL_16708983, EPI_ISL_16708988, EPI_ISL_16708994, EPI_ISL_16708999, EPI_ISL_16709000, EPI_ISL_16709001, EPI_ISL_16709003, EPI_ISL_16709006, EPI_ISL_16709007, EPI_ISL_16709008, EPI_ISL_16709010			
see above	University Hospitals of Leicester NHS Trust Pathology Services / Leicester Royal Infirmary	Reference Microbiology Services / UK Health Security Agency	Williams T. Zambon M. Talts T. Mosscrop L. Holmes C. Miah S
EPI_ISL_16714278, EPI_ISL_16714279, EPI_ISL_16714283, EPI_ISL_16714287, EPI_ISL_16714289, EPI_ISL_16714291, EPI_ISL_16714294, EPI_ISL_16714298, EPI_ISL_16714307, EPI_ISL_16714313, EPI_ISL_16714315, EPI_ISL_16714319, EPI_ISL_16714323, EPI_ISL_16714327, EPI_ISL_16714328, EPI_ISL_16714330, EPI_ISL_16714332, EPI_ISL_16714333, EPI_ISL_16714334, EPI_ISL_16714335, EPI_ISL_16714336, EPI_ISL_16714338, EPI_ISL_16714339, EPI_ISL_16714340, EPI_ISL_16714342, EPI_ISL_16714345, EPI_ISL_16714348, EPI_ISL_16714349, EPI_ISL_16714351, EPI_ISL_16714352, EPI_ISL_16714353, EPI_ISL_16714356, EPI_ISL_16714357, EPI_ISL_16714358, EPI_ISL_16714359, EPI_ISL_16714364, EPI_ISL_16714369, EPI_ISL_16714370, EPI_ISL_16714371, EPI_ISL_16714375, EPI_ISL_16714377, EPI_ISL_16714378, EPI_ISL_16714380, EPI_ISL_16714382, EPI_ISL_16714384, EPI_ISL_16714385, EPI_ISL_16714386, EPI_ISL_16714387, EPI_ISL_16714389, EPI_ISL_16714394, EPI_ISL_16714408, EPI_ISL_16714410, EPI_ISL_16714413, EPI_ISL_16714418, EPI_ISL_16714421, EPI_ISL_16714422, EPI_ISL_16714423, EPI_ISL_16714424, EPI_ISL_16714426, EPI_ISL_16714427, EPI_ISL_16714429			
see above	University Hospital Southampton / Southampton Specialist Virology Centre	Reference Microbiology Services / UK Health Security Agency	Williams T. Zambon M. Talts T. Mosscrop L. Pelosi E. Miah S
EPI_ISL_16714595, EPI_ISL_16714596, EPI_ISL_16714597, EPI_ISL_16714598, EPI_ISL_16714599, EPI_ISL_16714600, EPI_ISL_16714602, EPI_ISL_16714603, EPI_ISL_16714604, EPI_ISL_16714605, EPI_ISL_16714607, EPI_ISL_16714608, EPI_ISL_16714609, EPI_ISL_16714610, EPI_ISL_16714612, EPI_ISL_16714613, EPI_ISL_16714614, EPI_ISL_16714615, EPI_ISL_16714616, EPI_ISL_16714618, EPI_ISL_16714620, EPI_ISL_16714621, EPI_ISL_16714622, EPI_ISL_16714623, EPI_ISL_16714624, EPI_ISL_16714625, EPI_ISL_16714626, EPI_ISL_16714627, EPI_ISL_16714628, EPI_ISL_16714629, EPI_ISL_16714630, EPI_ISL_16714631, EPI_ISL_16714632, EPI_ISL_16714633, EPI_ISL_16714634, EPI_ISL_16714635, EPI_ISL_16714636, EPI_ISL_16714637, EPI_ISL_16714638, EPI_ISL_16714639, EPI_ISL_16714640, EPI_ISL_16714641, EPI_ISL_16714642, EPI_ISL_16714643, EPI_ISL_16714644, EPI_ISL_16714645, EPI_ISL_16714646, EPI_ISL_16714647, EPI_ISL_16714648, EPI_ISL_16714649, EPI_ISL_16714650, EPI_ISL_16714651, EPI_ISL_16714652, EPI_ISL_16714653, EPI_ISL_16714654, EPI_ISL_16714655, EPI_ISL_16714656, EPI_ISL_16714657, EPI_ISL_16714658, EPI_ISL_16714659, EPI_ISL_16714660, EPI_ISL_16714661, EPI_ISL_16714662, EPI_ISL_16714663, EPI_ISL_16714664, EPI_ISL_16714665, EPI_ISL_16714666, EPI_ISL_16714667, EPI_ISL_16714668, EPI_ISL_16714669, EPI_ISL_16714670, EPI_ISL_16714671, EPI_ISL_16714672, EPI_ISL_16714673, EPI_ISL_16714674, EPI_ISL_16714675, EPI_ISL_16714676, EPI_ISL_16714677, EPI_ISL_16714678, EPI_ISL_16714679, EPI_ISL_16714680, EPI_ISL_16714681, EPI_ISL_16714682, EPI_ISL_16714683, EPI_ISL_16714684, EPI_ISL_16714685, EPI_ISL_16714686, EPI_ISL_16714687			
see above	Respiratory Virus Unit / Reference Microbiology Services / UK Health Security Agency	Reference Microbiology Services / UK Health Security Agency	Zambon M. Talts T. Mosscrop L. Miah S
EPI_ISL_16737031, EPI_ISL_16737032, EPI_ISL_16737034, EPI_ISL_16737035, EPI_ISL_16737036, EPI_ISL_16737037	University Hospital Southampton / Southampton Specialist Virology Centre	Reference Microbiology Services / UK Health Security Agency	Williams T. Zambon M. Talts T. Mosscrop L. Pelosi E. Miah S
EPI_ISL_16959469	Ranfurly Medical Centre, Auckland	Institute of Environmental Science and Research	Klarysse Berquist, Lauren Jelley, Una Ren, Meaghan O'Neill
EPI_ISL_16959470	Rolleston Central Health, Christchurch	Institute of Environmental Science and Research	Klarysse Berquist, Lauren Jelley, Una Ren, Meaghan O'Neill

EPI_ISL_16959471	Upper Hutt Health Centre	Institute of Environmental Science and Research	Klarysse Berquist, Lauren Jelley, Una Ren, Meaghan O'Neill
EPI_ISL_16959472	Westland Medical Centre	Institute of Environmental Science and Research	Klarysse Berquist, Lauren Jelley, Una Ren, Meaghan O'Neill
EPI_ISL_16959475	LabPlus	Institute of Environmental Science and Research	Klarysse Berquist, Lauren Jelley, Una Ren, Meaghan O'Neill
EPI_ISL_16959476, EPI_ISL_16959477, EPI_ISL_16959478, EPI_ISL_16959479, EPI_ISL_16959480, EPI_ISL_16959481, EPI_ISL_16959482, EPI_ISL_16959484, EPI_ISL_16959485, EPI_ISL_16959486, EPI_ISL_16959487, EPI_ISL_16959488, EPI_ISL_16959489, EPI_ISL_16959491, EPI_ISL_16959492, EPI_ISL_16959493, EPI_ISL_16959494, EPI_ISL_16959496, EPI_ISL_16959497, EPI_ISL_16959498, EPI_ISL_16959499, EPI_ISL_16959501, EPI_ISL_16959502, EPI_ISL_16959503, EPI_ISL_16959504, EPI_ISL_16959505, EPI_ISL_16959506, EPI_ISL_16959508, EPI_ISL_16959510, EPI_ISL_16959511, EPI_ISL_16959512, EPI_ISL_16959513, EPI_ISL_16959515, EPI_ISL_16959516, EPI_ISL_16959517, EPI_ISL_16959518, EPI_ISL_16959519, EPI_ISL_16959520, EPI_ISL_16959521, EPI_ISL_16959523, EPI_ISL_16959524, EPI_ISL_16959525, EPI_ISL_16959526, EPI_ISL_16959527, EPI_ISL_16959529			
see above	ESR - Wallaceville	Institute of Environmental Science and Research	Klarysse Berquist, Lauren Jelley, Una Ren, Meaghan O'Neill
EPI_ISL_16959544	Middlemore Hospital	Institute of Environmental Science and Research	Klarysse Berquist, Lauren Jelley, Una Ren, Meaghan O'Neill
EPI_ISL_16959552, EPI_ISL_16959553, EPI_ISL_16959554, EPI_ISL_16959555	Hawkes Bay District Health Board	Institute of Environmental Science and Research	Klarysse Berquist, Lauren Jelley, Una Ren, Meaghan O'Neill
EPI_ISL_16959556, EPI_ISL_16959558, EPI_ISL_16959559, EPI_ISL_16959560	Wellington SCL	Institute of Environmental Science and Research	Klarysse Berquist, Lauren Jelley, Una Ren, Meaghan O'Neill
EPI_ISL_16959586, EPI_ISL_16959589, EPI_ISL_16959590, EPI_ISL_16959610, EPI_ISL_16959611, EPI_ISL_16959624, EPI_ISL_16959631, EPI_ISL_16959650, EPI_ISL_16959654, EPI_ISL_16959657, EPI_ISL_16959658, EPI_ISL_16959675, EPI_ISL_16959678, EPI_ISL_16959689, EPI_ISL_16959696, EPI_ISL_16959701, EPI_ISL_16959704, EPI_ISL_16959705, EPI_ISL_16959707, EPI_ISL_16959716, EPI_ISL_16959717, EPI_ISL_16959723, EPI_ISL_16959726, EPI_ISL_16959729			
see above	Middlemore Hospital	Institute of Environmental Science and Research	Klarysse Berquist, Lauren Jelley, Una Ren, Meaghan O'Neill
EPI_ISL_16959739, EPI_ISL_16959740	Southern Community Lab - Dunedin	Institute of Environmental Science and Research	Klarysse Berquist, Lauren Jelley, Una Ren, Meaghan O'Neill
EPI_ISL_16959752, EPI_ISL_16959753, EPI_ISL_16959755, EPI_ISL_16959760, EPI_ISL_16959761, EPI_ISL_16959763	Middlemore Hospital	Institute of Environmental Science and Research	Klarysse Berquist, Lauren Jelley, Una Ren, Meaghan O'Neill
EPI_ISL_16959784, EPI_ISL_16959785, EPI_ISL_16959786, EPI_ISL_16959787, EPI_ISL_16959789, EPI_ISL_16959790, EPI_ISL_16959791, EPI_ISL_16959793, EPI_ISL_16959794, EPI_ISL_16959795, EPI_ISL_16959796, EPI_ISL_16959797, EPI_ISL_16959798, EPI_ISL_16959799, EPI_ISL_16959800, EPI_ISL_16959801, EPI_ISL_16959802, EPI_ISL_16959804, EPI_ISL_16959805, EPI_ISL_16959807, EPI_ISL_16959808, EPI_ISL_16959809, EPI_ISL_16959811, EPI_ISL_16959812, EPI_ISL_16959813, EPI_ISL_16959815, EPI_ISL_16959816, EPI_ISL_16959817			
see above	Wellington SCL	Institute of Environmental Science and Research	Klarysse Berquist, Lauren Jelley, Una Ren, Meaghan O'Neill
EPI_ISL_16959823, EPI_ISL_16959824, EPI_ISL_16959825, EPI_ISL_16959827, EPI_ISL_16959828, EPI_ISL_16959837, EPI_ISL_16959838, EPI_ISL_16959839, EPI_ISL_16959840	Middlemore Hospital	Institute of Environmental Science and Research	Klarysse Berquist, Lauren Jelley, Una Ren, Meaghan O'Neill
EPI_ISL_16959841, EPI_ISL_16959842, EPI_ISL_16959843, EPI_ISL_16959844, EPI_ISL_16959845, EPI_ISL_16959848, EPI_ISL_16959850	Canterbury Health Laboratory	Institute of Environmental Science and Research	Klarysse Berquist, Lauren Jelley, Una Ren, Meaghan O'Neill
EPI_ISL_16959853, EPI_ISL_16959854, EPI_ISL_16959855	Labplus	Institute of Environmental Science and Research	Klarysse Berquist, Lauren Jelley, Una Ren, Meaghan O'Neill
EPI_ISL_16959857, EPI_ISL_16959859, EPI_ISL_16959864, EPI_ISL_16959865, EPI_ISL_16959868	Southern Community Lab - Dunedin	Institute of Environmental Science and Research	Klarysse Berquist, Lauren Jelley, Una Ren, Meaghan O'Neill
EPI_ISL_16959874, EPI_ISL_16959875, EPI_ISL_16959876, EPI_ISL_16959877, EPI_ISL_16959878	Middlemore Hospital	Institute of Environmental Science and Research	Klarysse Berquist, Lauren Jelley, Una Ren, Meaghan O'Neill
EPI_ISL_16959880, EPI_ISL_16959884, EPI_ISL_16959888, EPI_ISL_16959890	Labplus	Institute of Environmental Science and Research	Klarysse Berquist, Lauren Jelley, Una Ren, Meaghan O'Neill
EPI_ISL_16959891, EPI_ISL_16959892, EPI_ISL_16959893, EPI_ISL_16959894, EPI_ISL_16959896, EPI_ISL_16959897, EPI_ISL_16959898, EPI_ISL_16959899, EPI_ISL_16959900, EPI_ISL_16959901, EPI_ISL_16959902, EPI_ISL_16959903, EPI_ISL_16959904, EPI_ISL_16959906, EPI_ISL_16959908, EPI_ISL_16959909, EPI_ISL_16959910, EPI_ISL_16959911, EPI_ISL_16959912, EPI_ISL_16959913, EPI_ISL_16959914, EPI_ISL_16959916, EPI_ISL_16959917			
see above	Wellington SCL	Institute of Environmental Science and Research	Klarysse Berquist, Lauren Jelley, Una Ren, Meaghan O'Neill
EPI_ISL_16959921	PathLab Bay of Plenty	Institute of Environmental Science and Research	Klarysse Berquist, Lauren Jelley, Una Ren, Meaghan O'Neill
EPI_ISL_16959929, EPI_ISL_16959930, EPI_ISL_16959931, EPI_ISL_16959932	Canterbury Health Laboratory	Institute of Environmental Science and Research	Klarysse Berquist, Lauren Jelley, Una Ren, Meaghan O'Neill
EPI_ISL_16959933, EPI_ISL_16959934, EPI_ISL_16959935	Hawkes Bay District Health Board	Institute of Environmental Science and Research	Klarysse Berquist, Lauren Jelley, Una Ren, Meaghan O'Neill
EPI_ISL_16959936, EPI_ISL_16959937, EPI_ISL_16959938, EPI_ISL_16959939, EPI_ISL_16959940, EPI_ISL_16959941	Middlemore Hospital	Institute of Environmental Science and Research	Klarysse Berquist, Lauren Jelley, Una Ren, Meaghan O'Neill
EPI_ISL_16959947, EPI_ISL_16959950, EPI_ISL_16959956, EPI_ISL_16959958	LabPlus	Institute of Environmental Science and Research	Klarysse Berquist, Lauren Jelley, Una Ren, Meaghan O'Neill
EPI_ISL_16959984	MedLab South Nelson	Institute of Environmental Science and Research	Klarysse Berquist, Lauren Jelley, Una Ren, Meaghan O'Neill
EPI_ISL_16959986, EPI_ISL_16959987, EPI_ISL_16959988, EPI_ISL_16959989, EPI_ISL_16959990, EPI_ISL_16959991, EPI_ISL_16959992	Canterbury Health Laboratory	Institute of Environmental Science and Research	Klarysse Berquist, Lauren Jelley, Una Ren, Meaghan O'Neill
EPI_ISL_16959993, EPI_ISL_16959994, EPI_ISL_16959996, EPI_ISL_16959998	Southern Community Lab - Dunedin	Institute of Environmental Science and Research	Klarysse Berquist, Lauren Jelley, Una Ren, Meaghan O'Neill
EPI_ISL_16960000, EPI_ISL_16960001, EPI_ISL_16960006, EPI_ISL_16960009	Waikato Hospital	Institute of Environmental Science and Research	Klarysse Berquist, Lauren Jelley, Una Ren, Meaghan O'Neill
EPI_ISL_16960014, EPI_ISL_16960015, EPI_ISL_16960016, EPI_ISL_16960017	Middlemore Hospital	Institute of Environmental Science and Research	Klarysse Berquist, Lauren Jelley, Una Ren, Meaghan O'Neill
EPI_ISL_16960018, EPI_ISL_16960019, EPI_ISL_16960020	Hawkes Bay District Health Board	Institute of Environmental Science and Research	Klarysse Berquist, Lauren Jelley, Una Ren, Meaghan O'Neill
EPI_ISL_16960022, EPI_ISL_16960027, EPI_ISL_16960028, EPI_ISL_16960031, EPI_ISL_16960033, EPI_ISL_16960035, EPI_ISL_16960040, EPI_ISL_16960041, EPI_ISL_16960043, EPI_ISL_16960044, EPI_ISL_16960053, EPI_ISL_16960060, EPI_ISL_16960063, EPI_ISL_16960067, EPI_ISL_16960069, EPI_ISL_16960075, EPI_ISL_16960078, EPI_ISL_16960079, EPI_ISL_16960080, EPI_ISL_16960083, EPI_ISL_16960087, EPI_ISL_16960090, EPI_ISL_16960091			
see above	LabPlus	Institute of Environmental Science and Research	Klarysse Berquist, Lauren Jelley, Una Ren, Meaghan O'Neill
EPI_ISL_16960092, EPI_ISL_16960093, EPI_ISL_16960094, EPI_ISL_16960095, EPI_ISL_16960096, EPI_ISL_16960097, EPI_ISL_16960098	Middlemore Hospital	Institute of Environmental Science and Research	Klarysse Berquist, Lauren Jelley, Una Ren, Meaghan O'Neill
EPI_ISL_16960102	Wellington SCL	Institute of Environmental Science and Research	Klarysse Berquist, Lauren Jelley, Una Ren, Meaghan O'Neill
EPI_ISL_16960105, EPI_ISL_16960106, EPI_ISL_16960107, EPI_ISL_16960108, EPI_ISL_16960109, EPI_ISL_16960110	Canterbury Health Laboratory	Institute of Environmental Science and Research	Klarysse Berquist, Lauren Jelley, Una Ren, Meaghan O'Neill
EPI_ISL_16960116	Middlemore Hospital	Institute of Environmental Science and Research	Klarysse Berquist, Lauren Jelley, Una Ren, Meaghan O'Neill
EPI_ISL_16960119, EPI_ISL_16960120, EPI_ISL_16960121, EPI_ISL_16960122, EPI_ISL_16960123, EPI_ISL_16960124, EPI_ISL_16960126, EPI_ISL_16960127, EPI_ISL_16960128	Southern Community Lab - Dunedin	Institute of Environmental Science and Research	Klarysse Berquist, Lauren Jelley, Una Ren, Meaghan O'Neill
EPI_ISL_16960133, EPI_ISL_16960134, EPI_ISL_16960135, EPI_ISL_16960137, EPI_ISL_16960138, EPI_ISL_16960141, EPI_ISL_16960142, EPI_ISL_16960143, EPI_ISL_16960144, EPI_ISL_16960145, EPI_ISL_16960146			
see above	LabPlus	Institute of Environmental Science and Research	Klarysse Berquist, Lauren Jelley, Una Ren, Meaghan O'Neill
EPI_ISL_16960147, EPI_ISL_16960148	Canterbury Health Laboratory	Institute of Environmental Science and Research	Klarysse Berquist, Lauren Jelley, Una Ren, Meaghan O'Neill
EPI_ISL_16960150	Wellington SCL	Institute of Environmental Science and Research	Klarysse Berquist, Lauren Jelley, Una Ren, Meaghan O'Neill
EPI_ISL_17089180, EPI_ISL_17089181, EPI_ISL_17089182, EPI_ISL_17089185, EPI_ISL_17089186, EPI_ISL_17089188, EPI_ISL_17089189, EPI_ISL_17089190, EPI_ISL_17089191	University of Washington - Laboratory Medicine	University of Washington - Laboratory Medicine	Sereewit,J., Xie,H., Roychoudhury,P. and Greninger,A.L.
EPI_ISL_17104707	National Institute of Public Health	National Institute of Public Health	Chhorvann CHHEA, Darapheak CHAU, Sitha PRUM, Visal CHHE, Sokha DUL, Sengly MANH, Phally VY, Borann SAR, Chanthap LON, Jessica E. Manning, Sophana Chea, Savuth CHIN
EPI_ISL_17167890, EPI_ISL_17167891, EPI_ISL_17167892, EPI_ISL_17167893, EPI_ISL_17167894, EPI_ISL_17167895, EPI_ISL_17167896, EPI_ISL_17167897, EPI_ISL_17167898, EPI_ISL_17167899, EPI_ISL_17167900, EPI_ISL_17167901, EPI_ISL_17167902, EPI_ISL_17167903, EPI_ISL_17167904, EPI_ISL_17167905, EPI_ISL_17167906, EPI_ISL_17167907, EPI_ISL_17167908, EPI_ISL_17167909, EPI_ISL_17167910, EPI_ISL_17167911, EPI_ISL_17167912, EPI_ISL_17167913, EPI_ISL_17167914, EPI_ISL_17167915, EPI_ISL_17167916, EPI_ISL_17167917, EPI_ISL_17167918, EPI_ISL_17167919, EPI_ISL_17167920, EPI_ISL_17167921, EPI_ISL_17167922, EPI_ISL_17167923, EPI_ISL_17167924, EPI_ISL_17167925, EPI_ISL_17167926, EPI_ISL_17167927, EPI_ISL_17167928, EPI_ISL_17167929, EPI_ISL_17167930, EPI_ISL_17167931, EPI_ISL_17167932, EPI_ISL_17167933, EPI_ISL_17167934, EPI_ISL_17167935, EPI_ISL_17167936, EPI_ISL_17167937, EPI_ISL_17167938, EPI_ISL_17167939, EPI_ISL_17167940, EPI_ISL_17167941, EPI_ISL_17167942, EPI_ISL_17167943, EPI_ISL_17167944, EPI_ISL_17167945, EPI_ISL_17167946, EPI_ISL_17167947, EPI_ISL_17167948, EPI_ISL_17167949, EPI_ISL_17167950, EPI_ISL_17167951, EPI_ISL_17167952, EPI_ISL_17167953, EPI_ISL_17167954, EPI_ISL_17167955, EPI_ISL_17167956, EPI_ISL_17167957, EPI_ISL_17167958, EPI_ISL_17167959, EPI_ISL_17167960, EPI_ISL_17167961, EPI_ISL_17167962, EPI_ISL_17167963, EPI_ISL_17167964, EPI_ISL_17167965, EPI_ISL_17167966, EPI_ISL_17167967, EPI_ISL_17167968, EPI_ISL_17167969, EPI_ISL_17167970, EPI_ISL_17167971, EPI_ISL_17167972, EPI_ISL_17167973, EPI_ISL_17167974, EPI_ISL_17167975, EPI_ISL_17167976, EPI_ISL_17167977, EPI_ISL_17167978, EPI_ISL_17167979, EPI_ISL_17167980, EPI_ISL_17167981, EPI_ISL_17167982, EPI_ISL_17167983, EPI_ISL_17167984, EPI_ISL_17167985, EPI_ISL_17167986, EPI_ISL_17167987, EPI_ISL_17167988, EPI_ISL_17167989, EPI_ISL_17167990, EPI_ISL_17167991, EPI_ISL_17167992, EPI_ISL_17167993, EPI_ISL_17167994, EPI_ISL_17167995, EPI_ISL_17167996, EPI_ISL_17167997, EPI_ISL_17167998, EPI_ISL_17167999, EPI_ISL_17168000, EPI_ISL_17168001, EPI_ISL_17168002, EPI_ISL_17168003, EPI_ISL_17168004, EPI_ISL_17168005, EPI_ISL_17168006, EPI_ISL_17168007, EPI_ISL_17168008, EPI_ISL_17168009, EPI_ISL_17168010, EPI_ISL_17168011, EPI_ISL_17168023, EPI_ISL_17168024, EPI_ISL_17168025, EPI_ISL_17168026, EPI_ISL_17168027, EPI_ISL_17168028, EPI_ISL_17168029, EPI_ISL_17168030, EPI_ISL_17168031, EPI_ISL_17168032, EPI_ISL_17168033, EPI_ISL_17168034, EPI_ISL_17168035, EPI_ISL_17168036, EPI_ISL_17168037, EPI_ISL_17168038, EPI_ISL_17168039, EPI_ISL_17168040, EPI_ISL_17168041, EPI_ISL_17168042, EPI_ISL_17168043, EPI_ISL_17168044, EPI_ISL_17168045, EPI_ISL_17168046, EPI_ISL_17168047, EPI_ISL_17168048, EPI_ISL_17168049, EPI_ISL_17168050, EPI_ISL_17168051, EPI_ISL_17168052, EPI_ISL_17168053, EPI_ISL_17168054, EPI_ISL_17168055, EPI_ISL_17168056, EPI_ISL_17168057, EPI_ISL_17168058, EPI_ISL_17168059, EPI_ISL_17168060, EPI_ISL_17168061, EPI_ISL_17168062, EPI_ISL_17168063, EPI_ISL_17168064, EPI_ISL_17168065, EPI_ISL_17168066, EPI_ISL_17168067, EPI_ISL_17168068, EPI_ISL_17168069, EPI_ISL_17168070, EPI_ISL_17168071, EPI_ISL_17168072, EPI_ISL_17168073, EPI_ISL_17168074, EPI_ISL_17168165, EPI_ISL_17168168, EPI_ISL_17168169, EPI_ISL_17168170, EPI_ISL_17168171, EPI_ISL_17168172, EPI_ISL_17168173, EPI_ISL_17168174, EPI_ISL_17168175, EPI_ISL_17168176, EPI_ISL_17168177, EPI_ISL_17168178, EPI_ISL_17168179, EPI_ISL_17168180, EPI_ISL_17168181, EPI_ISL_17168182, EPI_ISL_17168183, EPI_ISL_17168184, EPI_ISL_17168185, EPI_ISL_17168186, EPI_ISL_17168187, EPI_ISL_17168188, EPI_ISL_17168189, EPI_ISL_17168190, EPI_ISL_17168191, EPI_ISL_17168192, EPI_ISL_17168193, EPI_ISL_17168194, EPI_ISL_17168195, EPI_ISL_17168196, EPI_ISL_17168197, EPI_ISL_17168198, EPI_ISL_17168199, EPI_ISL_17168200, EPI_ISL_17168201, EPI_ISL_17168202, EPI_ISL_17168203, EPI_ISL_17168204, EPI_ISL_17168205, EPI_ISL_17168206, EPI_ISL_17168207, EPI_ISL_17168208, EPI_ISL_17168209, EPI_ISL_17168210, EPI_ISL_17168211, EPI_ISL_17168212, EPI_ISL_17168213, EPI_ISL_17168214, EPI_ISL_17168215, EPI_ISL_17168216, EPI_ISL_17168217, EPI_ISL_17168233, EPI_ISL_17168234, EPI_ISL_17168235, EPI_ISL_17168236, EPI_ISL_17168237, EPI_ISL_17168238, EPI_ISL_17168239, EPI_ISL_17168240, EPI_ISL_17168241, EPI_ISL_17168250, EPI_ISL_17168251, EPI_ISL_17168252, EPI_ISL_17168253, EPI_ISL_17168254, EPI_ISL_17168255, EPI_ISL_17168256, EPI_ISL_17168258			
see above	National Institute for Viral Disease Control and Prevention	National Institute for Viral Disease Control and Prevention	Song,J., Zhu,Z., Song,J., Mao,N., Cui,A., Xu,W. and Zhang,Y.
EPI_ISL_17215509, EPI_ISL_17215510, EPI_ISL_17215511, EPI_ISL_17215513, EPI_ISL_17215515, EPI_ISL_17215516, EPI_ISL_17215518, EPI_ISL_17215519, EPI_ISL_17215520, EPI_ISL_17215521, EPI_ISL_17215522, EPI_ISL_17215524, EPI_ISL_17215525, EPI_ISL_17215526, EPI_ISL_17215527, EPI_ISL_17215528, EPI_ISL_17215529, EPI_ISL_17215530, EPI_ISL_17215531, EPI_ISL_17215532, EPI_ISL_17215533, EPI_ISL_17215534, EPI_ISL_17215535, EPI_ISL_17215536, EPI_ISL_17215537, EPI_ISL_17215539, EPI_ISL_17215540, EPI_ISL_17215541, EPI_ISL_17215542, EPI_ISL_17215543, EPI_ISL_17215544, EPI_ISL_17215545, EPI_ISL_17215546, EPI_ISL_17215547, EPI_ISL_17215595			
see above	State Key Laboratory of Respiratory Disease, Guangzhou Institute of Respiratory Health	State Key Laboratory of Respiratory Disease, Guangzhou Institute of Respiratory Health	Zhao,J., Wang,Y., Wei,P. and Xiao,J.
EPI_ISL_17215844, EPI_ISL_17215846, EPI_ISL_17215849, EPI_ISL_17215850, EPI_ISL_17215854, EPI_ISL_17215856, EPI_ISL_17215857, EPI_ISL_17215859, EPI_ISL_17215860, EPI_ISL_17215861, EPI_ISL_17215870, EPI_ISL_17215871, EPI_ISL_17215872, EPI_ISL_17215874, EPI_ISL_17215877, EPI_ISL_17215878, EPI_ISL_17215883, EPI_ISL_17215884, EPI_ISL_17215886, EPI_ISL_17215887, EPI_ISL_17215893, EPI_ISL_17215894, EPI_ISL_17215895, EPI_ISL_17215898, EPI_ISL_17215904, EPI_ISL_17215905, EPI_ISL_17215906, EPI_ISL_17215907, EPI_ISL_17215915, EPI_ISL_17215916, EPI_ISL_17215917, EPI_ISL_17215940, EPI_ISL_17215942, EPI_ISL_17215946, EPI_ISL_17215950, EPI_ISL_17215952, EPI_ISL_17215953, EPI_ISL_17215965, EPI_ISL_17215981, EPI_ISL_17215984, EPI_ISL_17215985, EPI_ISL_17215989, EPI_ISL_17215993, EPI_ISL_17216007, EPI_ISL_17216008, EPI_ISL_17216009, EPI_ISL_17216012, EPI_ISL_17216013, EPI_ISL_17216020, EPI_ISL_17216026, EPI_ISL_17216027, EPI_ISL_17216032, EPI_ISL_17216033, EPI_ISL_17216034, EPI_ISL_17216036, EPI_ISL_17216037, EPI_ISL_17216038, EPI_ISL_17216049, EPI_ISL_17216057, EPI_ISL_17216059, EPI_ISL_17216063, EPI_ISL_17216078, EPI_ISL_17216084, EPI_ISL_17216090, EPI_ISL_17216094, EPI_ISL_17216095, EPI_ISL_17216105, EPI_ISL_17216106, EPI_ISL_17216107, EPI_ISL_17216108, EPI_ISL_17216109, EPI_ISL_17216110, EPI_ISL_17216111, EPI_ISL_17216112, EPI_ISL_17216113, EPI_ISL_17216119, EPI_ISL_17216120, EPI_ISL_17216130, EPI_ISL_17216131, EPI_ISL_17216135,			

EPI_ISL_17216136, EPI_ISL_17216139, EPI_ISL_17216140, EPI_ISL_17216144, EPI_ISL_17216146, EPI_ISL_17216149, EPI_ISL_17216151, EPI_ISL_17216154, EPI_ISL_17216155, EPI_ISL_17216158, EPI_ISL_17216166, EPI_ISL_17216167, EPI_ISL_17216175, EPI_ISL_17216176, EPI_ISL_17216179, EPI_ISL_17216180, EPI_ISL_17216187, EPI_ISL_17216190, EPI_ISL_17216191, EPI_ISL_17216202, EPI_ISL_17216204, EPI_ISL_17216207, EPI_ISL_17216212, EPI_ISL_17216232, EPI_ISL_17216241, EPI_ISL_17216242, EPI_ISL_17216246, EPI_ISL_17216247, EPI_ISL_17216248, EPI_ISL_17216249, EPI_ISL_17216253, EPI_ISL_17216255, EPI_ISL_17216263, EPI_ISL_17216264, EPI_ISL_17216271, EPI_ISL_17216273, EPI_ISL_17216274, EPI_ISL_17216281, EPI_ISL_17216301, EPI_ISL_17216308, EPI_ISL_17216313, EPI_ISL_17216317, EPI_ISL_17216324, EPI_ISL_17216327, EPI_ISL_17216328, EPI_ISL_17216334, EPI_ISL_17216336, EPI_ISL_17216343, EPI_ISL_17216379, EPI_ISL_17216382, EPI_ISL_17216390, EPI_ISL_17216391, EPI_ISL_17216396, EPI_ISL_17216397, EPI_ISL_17216398, EPI_ISL_17216399, EPI_ISL_17216400, EPI_ISL_17216401, EPI_ISL_17216409, EPI_ISL_17216410, EPI_ISL_17216411, EPI_ISL_17216414, EPI_ISL_17216415, EPI_ISL_17216450, EPI_ISL_17216451, EPI_ISL_17216454, EPI_ISL_17216455, EPI_ISL_17216462, EPI_ISL_17216463, EPI_ISL_17216464, EPI_ISL_17216467, EPI_ISL_17216468, EPI_ISL_17216475, EPI_ISL_17216476, EPI_ISL_17216479, EPI_ISL_17216480, EPI_ISL_17216483, EPI_ISL_17216485, EPI_ISL_17216487, EPI_ISL_17216488, EPI_ISL_17216489, EPI_ISL_17216491, EPI_ISL_17216493, EPI_ISL_17216494, EPI_ISL_17216495, EPI_ISL_17216501, EPI_ISL_17216502, EPI_ISL_17216503, EPI_ISL_17216505, EPI_ISL_17216506, EPI_ISL_17216514, EPI_ISL_17216515, EPI_ISL_17216516, EPI_ISL_17216518, EPI_ISL_17216519, EPI_ISL_17216520, EPI_ISL_17216530, EPI_ISL_17216531, EPI_ISL_17216532, EPI_ISL_17216533, EPI_ISL_17216534, EPI_ISL_17216539, EPI_ISL_17216540, EPI_ISL_17216543, EPI_ISL_17216544, EPI_ISL_17216548, EPI_ISL_17216549, EPI_ISL_17216553, EPI_ISL_17216554, EPI_ISL_17216556, EPI_ISL_17216559, EPI_ISL_17216560, EPI_ISL_17216561, EPI_ISL_17216569, EPI_ISL_17216570, EPI_ISL_17216571, EPI_ISL_17216577, EPI_ISL_17216578, EPI_ISL_17216583, EPI_ISL_17216584, EPI_ISL_17216585, EPI_ISL_17216588, EPI_ISL_17216590, EPI_ISL_17216591, EPI_ISL_17216592, EPI_ISL_17216599, EPI_ISL_17216600, EPI_ISL_17216601, EPI_ISL_17216603, EPI_ISL_17216604, EPI_ISL_17216607, EPI_ISL_17216609, EPI_ISL_17216615, EPI_ISL_17216616, EPI_ISL_17216617, EPI_ISL_17216620, EPI_ISL_17216623, EPI_ISL_17216624, EPI_ISL_17216625, EPI_ISL_17216631, EPI_ISL_17216632, EPI_ISL_17216637, EPI_ISL_17216638, EPI_ISL_17216640, EPI_ISL_17216641, EPI_ISL_17216648, EPI_ISL_17216675, EPI_ISL_17216681, EPI_ISL_17216683, EPI_ISL_17216693, EPI_ISL_17216698, EPI_ISL_17216703, EPI_ISL_17216706, EPI_ISL_17216717, EPI_ISL_17216719, EPI_ISL_17216722, EPI_ISL_17216725, EPI_ISL_17216727, EPI_ISL_17216728, EPI_ISL_17216742, EPI_ISL_17216743, EPI_ISL_17216744, EPI_ISL_17216745, EPI_ISL_17216746, EPI_ISL_17216747, EPI_ISL_17216751, EPI_ISL_17216756, EPI_ISL_17216757, EPI_ISL_17216758, EPI_ISL_17216767, EPI_ISL_17216770, EPI_ISL_17216780, EPI_ISL_17216795, EPI_ISL_17216796, EPI_ISL_17216802, EPI_ISL_17216809, EPI_ISL_17216811, EPI_ISL_17216832, EPI_ISL_17216836, EPI_ISL_17216837, EPI_ISL_17216838, EPI_ISL_17216839, EPI_ISL_17216840, EPI_ISL_17216841, EPI_ISL_17216846, EPI_ISL_17216847, EPI_ISL_17216848, EPI_ISL_17216862, EPI_ISL_17216863, EPI_ISL_17216864, EPI_ISL_17216866, EPI_ISL_17216867, EPI_ISL_17216868, EPI_ISL_17216874, EPI_ISL_17216876, EPI_ISL_17216877, EPI_ISL_17216886, EPI_ISL_17216887, EPI_ISL_17216897, EPI_ISL_17216904, EPI_ISL_17216905, EPI_ISL_17216916, EPI_ISL_17216918, EPI_ISL_17216919, EPI_ISL_17216920, EPI_ISL_17216928, EPI_ISL_17216929, EPI_ISL_17216931, EPI_ISL_17216938, EPI_ISL_17216940, EPI_ISL_17216942, EPI_ISL_17216943, EPI_ISL_17216944, EPI_ISL_17216945, EPI_ISL_17216955, EPI_ISL_17216956, EPI_ISL_17216959, EPI_ISL_17216960, EPI_ISL_17216964, EPI_ISL_17216967, EPI_ISL_17216968, EPI_ISL_17216974, EPI_ISL_17216976, EPI_ISL_17216980, EPI_ISL_17216981, EPI_ISL_17216983, EPI_ISL_17216987, EPI_ISL_17216988, EPI_ISL_17216991, EPI_ISL_17216993, EPI_ISL_17217002, EPI_ISL_17217003, EPI_ISL_17217006, EPI_ISL_17217007, EPI_ISL_17217009, EPI_ISL_17217012, EPI_ISL_17217013, EPI_ISL_17217016, EPI_ISL_17217017, EPI_ISL_17217018, EPI_ISL_17217020, EPI_ISL_17217024, EPI_ISL_17217033, EPI_ISL_17217053, EPI_ISL_17217057, EPI_ISL_17217060, EPI_ISL_17217061, EPI_ISL_17217062, EPI_ISL_17217064, EPI_ISL_17217065, EPI_ISL_17217066, EPI_ISL_17217076, EPI_ISL_17217106, EPI_ISL_17217114, EPI_ISL_17217128, EPI_ISL_17217129, EPI_ISL_17217130, EPI_ISL_17217158, EPI_ISL_17217166, EPI_ISL_17217167, EPI_ISL_17217171, EPI_ISL_17217175, EPI_ISL_17217183, EPI_ISL_17217184, EPI_ISL_17217187, EPI_ISL_17217188, EPI_ISL_17217190, EPI_ISL_17217191, EPI_ISL_17217197, EPI_ISL_17217198, EPI_ISL_17217200, EPI_ISL_17217201, EPI_ISL_17217205, EPI_ISL_17217209, EPI_ISL_17217210, EPI_ISL_17217213, EPI_ISL_17217214, EPI_ISL_17217215, EPI_ISL_17217224, EPI_ISL_17217225, EPI_ISL_17217230, EPI_ISL_17217231, EPI_ISL_17217233, EPI_ISL_17217244, EPI_ISL_17217245, EPI_ISL_17217246, EPI_ISL_17217249, EPI_ISL_17217255, EPI_ISL_17217256, EPI_ISL_17217262, EPI_ISL_17217264, EPI_ISL_17217270, EPI_ISL_17217271, EPI_ISL_17217272, EPI_ISL_17217273, EPI_ISL_17217277, EPI_ISL_17217291, EPI_ISL_17217293, EPI_ISL_17217299, EPI_ISL_17217323, EPI_ISL_17217330, EPI_ISL_17217331, EPI_ISL_17217332, EPI_ISL_17217339, EPI_ISL_17217341, EPI_ISL_17217342, EPI_ISL_17217347, EPI_ISL_17217348, EPI_ISL_17217356, EPI_ISL_17217358, EPI_ISL_17217359, EPI_ISL_17217369, EPI_ISL_17217371, EPI_ISL_17217387, EPI_ISL_17217388, EPI_ISL_17217414, EPI_ISL_17217416, EPI_ISL_17217419, EPI_ISL_17217431, EPI_ISL_17217435, EPI_ISL_17217438, EPI_ISL_17217441, EPI_ISL_17217442, EPI_ISL_17217468, EPI_ISL_17217477, EPI_ISL_17217481, EPI_ISL_17217499, EPI_ISL_17217500, EPI_ISL_17217501, EPI_ISL_17217506, EPI_ISL_17217509, EPI_ISL_17217510, EPI_ISL_17217518, EPI_ISL_17217529, EPI_ISL_17217530, EPI_ISL_17217532, EPI_ISL_17217539, EPI_ISL_17217540, EPI_ISL_17217543, EPI_ISL_17217547, EPI_ISL_17217548, EPI_ISL_17217551, EPI_ISL_17217557, EPI_ISL_17217560, EPI_ISL_17217561, EPI_ISL_17217565, EPI_ISL_17217567, EPI_ISL_17217568, EPI_ISL_17217574, EPI_ISL_17217575, EPI_ISL_17217577, EPI_ISL_17217578, EPI_ISL_17217583, EPI_ISL_17217584, EPI_ISL_17217585, EPI_ISL_17217589, EPI_ISL_17217596, EPI_ISL_17217598, EPI_ISL_17217601, EPI_ISL_17217605, EPI_ISL_17217609, EPI_ISL_17217610, EPI_ISL_17217614, EPI_ISL_17217615, EPI_ISL_17217625, EPI_ISL_17217626, EPI_ISL_17217635, EPI_ISL_17217636, EPI_ISL_17217641, EPI_ISL_17217643, EPI_ISL_17217669, EPI_ISL_17217673, EPI_ISL_17217675, EPI_ISL_17217677, EPI_ISL_17217678, EPI_ISL_17217681, EPI_ISL_17217682, EPI_ISL_17217683, EPI_ISL_17217690, EPI_ISL_17217691, EPI_ISL_17217695, EPI_ISL_17217698, EPI_ISL_17217699, EPI_ISL_17217701, EPI_ISL_17217704, EPI_ISL_17217710, EPI_ISL_17217716, EPI_ISL_17217717, EPI_ISL_17217721, EPI_ISL_17217728, EPI_ISL_17217747, EPI_ISL_17217755, EPI_ISL_17217763, EPI_ISL_17217767, EPI_ISL_17217775, EPI_ISL_17217776, EPI_ISL_17217808, EPI_ISL_17217826, EPI_ISL_17217827, EPI_ISL_17217829, EPI_ISL_17217833, EPI_ISL_17217850, EPI_ISL_17217852, EPI_ISL_17217855, EPI_ISL_17217861, EPI_ISL_17217877, EPI_ISL_17217879, EPI_ISL_17217885, EPI_ISL_17217889, EPI_ISL_17217890, EPI_ISL_17217891, EPI_ISL_17217892, EPI_ISL_17217904, EPI_ISL_17217905, EPI_ISL_17217906, EPI_ISL_17217907, EPI_ISL_17217908, EPI_ISL_17217913, EPI_ISL_17217917, EPI_ISL_17217918, EPI_ISL_17217926, EPI_ISL_17217939, EPI_ISL_17217940, EPI_ISL_17217948, EPI_ISL_17217949, EPI_ISL_17217955, EPI_ISL_17217964, EPI_ISL_17217973, EPI_ISL_17217980, EPI_ISL_17217981, EPI_ISL_17217991, EPI_ISL_17217992, EPI_ISL_17218005, EPI_ISL_17218006, EPI_ISL_17218008, EPI_ISL_17218016, EPI_ISL_17218019, EPI_ISL_17218039, EPI_ISL_17218065, EPI_ISL_17218067, EPI_ISL_17218070, EPI_ISL_17218071, EPI_ISL_17218086, EPI_ISL_17218089, EPI_ISL_17218092, EPI_ISL_17218100, EPI_ISL_17218127, EPI_ISL_17218128, EPI_ISL_17218130, EPI_ISL_17218144, EPI_ISL_17218145, EPI_ISL_17218148, EPI_ISL_17218149, EPI_ISL_17218158, EPI_ISL_17218159, EPI_ISL_17218171, EPI_ISL_17218176, EPI_ISL_17218197, EPI_ISL_17218210, EPI_ISL_17218212, EPI_ISL_17218214, EPI_ISL_17218216, EPI_ISL_17218220, EPI_ISL_17218221, EPI_ISL_17218223, EPI_ISL_17218224, EPI_ISL_17218226, EPI_ISL_17218227, EPI_ISL_17218228, EPI_ISL_17218230, EPI_ISL_17218231, EPI_ISL_17218236, EPI_ISL_17218241, EPI_ISL_17218242, EPI_ISL_17218251, EPI_ISL_17218255, EPI_ISL_17218257, EPI_ISL_17218261, EPI_ISL_17218263, EPI_ISL_17218264, EPI_ISL_17218269, EPI_ISL_17218270, EPI_ISL_17218279, EPI_ISL_17218280, EPI_ISL_17218281, EPI_ISL_17218286, EPI_ISL_17218287, EPI_ISL_17218290, EPI_ISL_17218291, EPI_ISL_17218319, EPI_ISL_17218320, EPI_ISL_17218340, EPI_ISL_17218343, EPI_ISL_17218360, EPI_ISL_17218361, EPI_ISL_17218364, EPI_ISL_17218370, EPI_ISL_17218376, EPI_ISL_17218386, EPI_ISL_17218389, EPI_ISL_17218458, EPI_ISL_17218464, EPI_ISL_17218466, EPI_ISL_17218479, EPI_ISL_17218485, EPI_ISL_17218486, EPI_ISL_17218511, EPI_ISL_17218513, EPI_ISL_17218514, EPI_ISL_17218519, EPI_ISL_17218524, EPI_ISL_17218535, EPI_ISL_17218555, EPI_ISL_17218561, EPI_ISL_17218564, EPI_ISL_17218572, EPI_ISL_17218575, EPI_ISL_17218587, EPI_ISL_17218589, EPI_ISL_17218595, EPI_ISL_17218609, EPI_ISL_17218612, EPI_ISL_17218630, EPI_ISL_17218640, EPI_ISL_17218643, EPI_ISL_17218652, EPI_ISL_17218654, EPI_ISL_17218661, EPI_ISL_17218663, EPI_ISL_17218666, EPI_ISL_17218674, EPI_ISL_17218687, EPI_ISL_17218689, EPI_ISL_17218691, EPI_ISL_17218702, EPI_ISL_17218705, EPI_ISL_17218708, EPI_ISL_17218713, EPI_ISL_17218714, EPI_ISL_17218723, EPI_ISL_17218725, EPI_ISL_17218738, EPI_ISL_17218760, EPI_ISL_17218762, EPI_ISL_17218765, EPI_ISL_17218766, EPI_ISL_17218774, EPI_ISL_17218778, EPI_ISL_17218781, EPI_ISL_17218794, EPI_ISL_17218802, EPI_ISL_17218803, EPI_ISL_17218806, EPI_ISL_17218808, EPI_ISL_17218809, EPI_ISL_17218818, EPI_ISL_17218828, EPI_ISL_17218830, EPI_ISL_17218838, EPI_ISL_17218847, EPI_ISL_17218849, EPI_ISL_17218852, EPI_ISL_17218856, EPI_ISL_17218859, EPI_ISL_17218865, EPI_ISL_17218871, EPI_ISL_17218872, EPI_ISL_17218875, EPI_ISL_17218876, EPI_ISL_17218879, EPI_ISL_17218888, EPI_ISL_17218890, EPI_ISL_17218896, EPI_ISL_17218898, EPI_ISL_17218904, EPI_ISL_17218906, EPI_ISL_17218907, EPI_ISL_17218912, EPI_ISL_17218913, EPI_ISL_17218914, EPI_ISL_17218917, EPI_ISL_17218926, EPI_ISL_17218927, EPI_ISL_17218932, EPI_ISL_17218933, EPI_ISL_17218947, EPI_ISL_17218949, EPI_ISL_17218953, EPI_ISL_17218970, EPI_ISL_17218993, EPI_ISL_17218996, EPI_ISL_17219042, EPI_ISL_17219057, EPI_ISL_17219058, EPI_ISL_17219059, EPI_ISL_17219065, EPI_ISL_17219067, EPI_ISL_17219076, EPI_ISL_17219077, EPI_ISL_17219083, EPI_ISL_17219092, EPI_ISL_17219094, EPI_ISL_17219134, EPI_ISL_17219151, EPI_ISL_17219154, EPI_ISL_17219157, EPI_ISL_17219161, EPI_ISL_17219162, EPI_ISL_17219170, EPI_ISL_17219171, EPI_ISL_17219177, EPI_ISL_17219178, EPI_ISL_17219179, EPI_ISL_17219183, EPI_ISL_17219184, EPI_ISL_17219186, EPI_ISL_17219192, EPI_ISL_17219220, EPI_ISL_17219237, EPI_ISL_17219238, EPI_ISL_17219244, EPI_ISL_17219247, EPI_ISL_17219252, EPI_ISL_17219253, EPI_ISL_17219261, EPI_ISL_17219271, EPI_ISL_17219275, EPI_ISL_17219279, EPI_ISL_17219286, EPI_ISL_17219291, EPI_ISL_17219295, EPI_ISL_17219296, EPI_ISL_17219298, EPI_ISL_17219301, EPI_ISL_17219302, EPI_ISL_17219304, EPI_ISL_17219305, EPI_ISL_17219306, EPI_ISL_17219307, EPI_ISL_17219311, EPI_ISL_17219314, EPI_ISL_17219315, EPI_ISL_17219318, EPI_ISL_17219319, EPI_ISL_17219327, EPI_ISL_17219328, EPI_ISL_17219342, EPI_ISL_17219347, EPI_ISL_17219353, EPI_ISL_17219359, EPI_ISL_17219368, EPI_ISL_17219370, EPI_ISL_17219381, EPI_ISL_17219384, EPI_ISL_17219386, EPI_ISL_17219394, EPI_ISL_17219416, EPI_ISL_17219421, EPI_ISL_17219424, EPI_ISL_17219425, EPI_ISL_17219443, EPI_ISL_17219446, EPI_ISL_17219456, EPI_ISL_17219459, EPI_ISL_17219461, EPI_ISL_17219482, EPI_ISL_17219499, EPI_ISL_17219526, EPI_ISL_17219527, EPI_ISL_17219528, EPI_ISL_17219530, EPI_ISL_17219531, EPI_ISL_17219538, EPI_ISL_17219541, EPI_ISL_17219548, EPI_ISL_17219554, EPI_ISL_17219556, EPI_ISL_17219573, EPI_ISL_17219613, EPI_ISL_17219627, EPI_ISL_17219632, EPI_ISL_17219635, EPI_ISL_17219637, EPI_ISL_17219638, EPI_ISL_17219643, EPI_ISL_17219645, EPI_ISL_17219647, EPI_ISL_17219655, EPI_ISL_17219656, EPI_ISL_17219659, EPI_ISL_17219662, EPI_ISL_17219663, EPI_ISL_17219666, EPI_ISL_17219671, EPI_ISL_17219672, EPI_ISL_17219680, EPI_ISL_17219682, EPI_ISL_17219690, EPI_ISL_17219694, EPI_ISL_17219695, EPI_ISL_17219696, EPI_ISL_17219707, EPI_ISL_17219713, EPI_ISL_17219715, EPI_ISL_17219722, EPI_ISL_17219726, EPI_ISL_17219727, EPI_ISL_17219741, EPI_ISL_17219773, EPI_ISL_17219776, EPI_ISL_17219778, EPI_ISL_17219785, EPI_ISL_17219786, EPI_ISL_17219791, EPI_ISL_17219796, EPI_ISL_17219803, EPI_ISL_17219820, EPI_ISL_17219827, EPI_ISL_17219834, EPI_ISL_17219862, EPI_ISL_17219866, EPI_ISL_17219874, EPI_ISL_17219878, EPI_ISL_17219882, EPI_ISL_17219890, EPI_ISL_17219895, EPI_ISL_17219896, EPI_ISL_17219899, EPI_ISL_17219902, EPI_ISL_17219903, EPI_ISL_17219906, EPI_ISL_17219908, EPI_ISL_17219910, EPI_ISL_17219932, EPI_ISL_17219938, EPI_ISL_17219939, EPI_ISL_17219940, EPI_ISL_17219946, EPI_ISL_17219947, EPI_ISL_17219949, EPI_ISL_17219955, EPI_ISL_17219957, EPI_ISL_17219963, EPI_ISL_17219965, EPI_ISL_17219968, EPI_ISL_17219969, EPI_ISL_17219981, EPI_ISL_17219982, EPI_ISL_17219990, EPI_ISL_17219991, EPI_ISL_17219993, EPI_ISL_17219994, EPI_ISL_17219995, EPI_ISL_17219996, EPI_ISL_17220000, EPI_ISL_17220004, EPI_ISL_17220006, EPI_ISL_17220010, EPI_ISL_17220012, EPI_ISL_17220014, EPI_ISL_17220015, EPI_ISL_17220026, EPI_ISL_17220027, EPI_ISL_17220029, EPI_ISL_17220030, EPI_ISL_17220032, EPI_ISL_17220035, EPI_ISL_17220040, EPI_ISL_17220041, EPI_ISL_17220042, EPI_ISL_17220052, EPI_ISL_17220054, EPI_ISL_17220055, EPI_ISL_17220064, EPI_ISL_17220065, EPI_ISL_17220070, EPI_ISL_17220072, EPI_ISL_17220102, EPI_ISL_17220103, EPI_ISL_17220108, EPI_ISL_17220110, EPI_ISL_17220122, EPI_ISL_17220128, EPI_ISL_17220129, EPI_ISL_17220136, EPI_ISL_17220140, EPI_ISL_17220143, EPI_ISL_17220148, EPI_ISL_17220155, EPI_ISL_17220156, EPI_ISL_17220159, EPI_ISL_17220160, EPI_ISL_17220186, EPI_ISL_17220195, EPI_ISL_17220196, EPI_ISL_17220212, EPI_ISL_17220214, EPI_ISL_17220216, EPI_ISL_17220222, EPI_ISL_17220228, EPI_ISL_17220232, EPI_ISL_17220233, EPI_ISL_17220241, EPI_ISL_17220242, EPI_ISL_17220243, EPI_ISL_17220245, EPI_ISL_17220248, EPI_ISL_17220249, EPI_ISL_17220277, EPI_ISL_17220278, EPI_ISL_17220280, EPI_ISL_17220291, EPI_ISL_17220295, EPI_ISL_17220296, EPI_ISL_17220297, EPI_ISL_17220299, EPI_ISL_17220301, EPI_ISL_17220309, EPI_ISL_17220311, EPI_ISL_17220312, EPI_ISL_17220315, EPI_ISL_17220316, EPI_ISL_17220317, EPI_ISL_17220320, EPI_ISL_17220321, EPI_ISL_17220322, EPI_ISL_17220329, EPI_ISL_17220332, EPI_ISL_17220333, EPI_ISL_17220334, EPI_ISL_17220336, EPI_ISL_17220337, EPI_ISL_17220338, EPI_ISL_17220341, EPI_ISL_17220347, EPI_ISL_17220348, EPI_ISL_17220355, EPI_ISL_17220356, EPI_ISL_17220357, EPI_ISL_17220360, EPI_ISL_17220361, EPI_ISL_17220366, EPI_ISL_17220367, EPI_ISL_17220368, EPI_ISL_17220369, EPI_ISL_17220371, EPI_ISL_17220383, EPI_ISL_17220384, EPI_ISL_17220390, EPI_ISL_17220391, EPI_ISL_17220396, EPI_ISL_17220397, EPI_ISL_17220398, EPI_ISL_17220407, EPI_ISL_17220408, EPI_ISL_17220409, EPI_ISL_17220411, EPI_ISL_17220412, EPI_ISL_17220415, EPI_ISL_17220416, EPI_ISL_17220417, EPI_ISL_17220418, EPI_ISL_17220423, EPI_ISL_17220424, EPI_ISL_17220425, EPI_ISL_17220426, EPI_ISL_17220429, EPI_ISL_17220430, EPI_ISL_17220431, EPI_ISL_17220432, EPI_ISL_17220442, EPI_ISL_17220443, EPI_ISL_17220448, EPI_ISL_17220449, EPI_ISL_17220450, EPI_ISL_17220453, EPI_ISL_17220454, EPI_ISL_17220455, EPI_ISL_17220460, EPI_ISL_17220464, EPI_ISL_17220469, EPI_ISL_17220478, EPI_ISL_17220503, EPI_ISL_17220504, EPI_ISL_17220506, EPI_ISL_17220507, EPI_ISL_17220508, EPI_ISL_17220514, EPI_ISL_17220548, EPI_ISL_17220549, EPI_ISL_17220550, EPI_ISL_17220551, EPI_ISL_17220553, EPI_ISL_17220557, EPI_ISL_17220558, EPI_ISL_17220563, EPI_ISL_17220571, EPI_ISL_17220572, EPI_ISL_17220573, EPI_ISL_17220589, EPI_ISL_17220595, EPI_ISL_17220622, EPI_ISL_17220623, EPI_ISL_17220626, EPI_ISL_17220627, EPI_ISL_17220635, EPI_ISL_17220636, EPI_ISL_17220645, EPI_ISL_17220646, EPI_ISL_17220647, EPI_ISL_17220648, EPI_ISL_17220649, EPI_ISL_17220650, EPI_ISL_17220656, EPI_ISL_17220657, EPI_ISL_17220662, EPI_ISL_17220663, EPI_ISL_17220664, EPI_ISL_17220670, EPI_ISL_17220672, EPI_ISL_17220673, EPI_ISL_17220674, EPI_ISL_17220675, EPI_ISL_17220676, EPI_ISL_17220679, EPI_ISL_17220683, EPI_ISL_17220687, EPI_ISL_17220688, EPI_ISL_17220695, EPI_ISL_17220696, EPI_ISL_17220698, EPI_ISL_17220699, EPI_ISL_17220702, EPI_ISL_17220703, EPI_ISL_17220710, EPI_ISL_17220712, EPI_ISL_17220713, EPI_ISL_17220720, EPI_ISL_17220721, EPI_ISL_17220722, EPI_ISL_17220742, EPI_ISL_17220743, EPI_ISL_17220745, EPI_ISL_17220746, EPI_ISL_17220747, EPI_ISL_17220757, EPI_ISL_17220759, EPI_ISL_17220762, EPI_ISL_17220763, EPI_ISL_17220764, EPI_ISL_17220767, EPI_ISL_17220768, EPI_ISL_17220769, EPI_ISL_17220773, EPI_ISL_17220774, EPI_ISL_17220775, EPI_ISL_17220782, EPI_ISL_17220814, EPI_ISL_17220815, EPI_ISL_17220817, EPI_ISL_17220818, EPI_ISL_17220821, EPI_ISL_17220849, EPI_ISL_17220854, EPI_ISL_17220864, EPI_ISL_17220868, EPI_ISL_17220869, EPI_ISL_17220875, EPI_ISL_17220878, EPI_ISL_17220879, EPI_ISL_17220880, EPI_ISL_17220887, EPI_ISL_17220889, EPI_ISL_17220891, EPI_ISL_17220894, EPI_ISL_17220895, EPI_ISL_17220896, EPI_ISL_17220903, EPI_ISL_17220904, EPI_ISL_17220905, EPI_ISL_17220906, EPI_ISL_17220907, EPI_ISL_17220908, EPI_ISL_17220921, EPI_ISL_17220938, EPI_ISL_17220939, EPI_ISL_17220940, EPI_ISL_17220941, EPI_ISL_17220954, EPI_ISL_17220955, EPI_ISL_17220956, EPI_ISL_17220960, EPI_ISL_17220961, EPI_ISL_17220964, EPI_ISL_17220965, EPI_ISL_17220970, EPI_ISL_17220972, EPI_ISL_17220974, EPI_ISL_17220982, EPI_ISL_17220983, EPI_ISL_17220985, EPI_ISL_17220986, EPI_ISL_17220993, EPI_ISL_17220994, EPI_ISL_17220998, EPI_ISL_17221001, EPI_ISL_17221002, EPI_ISL_17221012, EPI_ISL_17221013, EPI_ISL_17221015, EPI_ISL_17221016, EPI_ISL_17221023, EPI_ISL_17221024, EPI_ISL_17221028, EPI_ISL_17221029, EPI_ISL_17221030, EPI_ISL_17221045, EPI_ISL_17221046, EPI_ISL_17221047, EPI_ISL_17221055, EPI_ISL_17221066, EPI_ISL_17221069, EPI_ISL_17221074, EPI_ISL_17221075, EPI_ISL_17221076, EPI_ISL_17221079, EPI_ISL_17221081, EPI_ISL_17221089, EPI_ISL_17221098, EPI_ISL_17221099, EPI_ISL_17221100, EPI_ISL_17221101, EPI_ISL_17221106, EPI_ISL_17221107, EPI_ISL_17221108, EPI_ISL_17221112, EPI_ISL_17221113, EPI_ISL_17221115, EPI_ISL_17221116, EPI_ISL_17221120, EPI_ISL_17221122, EPI_ISL_17221124, EPI_ISL_17221139, EPI_ISL_17221162, EPI_ISL_17221167, EPI_ISL_17221187, EPI_ISL_17221196, EPI_ISL_17221197, EPI_ISL_17221198, EPI_ISL_17221203, EPI_ISL_17221204, EPI_ISL_17221211, EPI_ISL_17221215, EPI_ISL_17221216, EPI_ISL_17221217, EPI_ISL_17221222, EPI_ISL_17221223, EPI_ISL_17221224, EPI_ISL_17221226, EPI_ISL_17221228, EPI_ISL_17221232, EPI_ISL_17221233, EPI_ISL_17221234, EPI_ISL_17221242, EPI_ISL_17221244, EPI_ISL_17221253, EPI_ISL_17221254, EPI_ISL_17221255, EPI_ISL_17221256, EPI_ISL_17221257, EPI_ISL_17221258, EPI_ISL_17221263, EPI_ISL_17221264, EPI_ISL_17221272, EPI_ISL_17221273, EPI_ISL_17221277, EPI_ISL_17221285, EPI_ISL_17221287, EPI_ISL_17221289, EPI_ISL_17221290, EPI_ISL_17221291, EPI_ISL_17221292, EPI_ISL_17221293, EPI_ISL_17221295, EPI_ISL_17221301, EPI_ISL_17221307, EPI_ISL_17221316, EPI_ISL_17221318, EPI_ISL_17221319, EPI_ISL_17221326, EPI_ISL_17221329, EPI_ISL_17221333, EPI_ISL_17221334, EPI_ISL_17221338, EPI_ISL_17221340, EPI_ISL_17221341, EPI_ISL_17221342, EPI_ISL_17221344, EPI_ISL_17221346, EPI_ISL_17221348, EPI_ISL_17221349, EPI_ISL_17221350, EPI_ISL_17221363, EPI_ISL_17221364, EPI_ISL_17221365, EPI_ISL_17221366, EPI_ISL_17221367, EPI_ISL_17221368, EPI_ISL_17221374, EPI_ISL_17221377, EPI_ISL_17221379, EPI_ISL_17221380, EPI_ISL_17221382, EPI_ISL_17221385, EPI_ISL_17221390, EPI_ISL_17221391, EPI_ISL_17221392, EPI_ISL_17221393, EPI_ISL_17221395, EPI_ISL_17221396, EPI_ISL_17221398, EPI_ISL_17221401, EPI_ISL_17221403, EPI_ISL_17221405, EPI_ISL_17221406, EPI_ISL_17221407, EPI_ISL_17221408, EPI_ISL_17221412, EPI_ISL_17221414, EPI_ISL_17221417, EPI_ISL_17221418, EPI_ISL_17221422, EPI_ISL_17221423, EPI_ISL_17221431, EPI_ISL_17221437, EPI_ISL_17221444, EPI_ISL_17221450, EPI_ISL_17221451, EPI_ISL_17221453, EPI_ISL_17221454, EPI_ISL_17221462, EPI_ISL_17221463, EPI_ISL_17221480, EPI_ISL_17221482, EPI_ISL_17221484, EPI_ISL_17221490, EPI_ISL_17221491, EPI_ISL_17221493, EPI_ISL_17221497, EPI_ISL_17221507, EPI_ISL_17221510, EPI_ISL_17221532, EPI_ISL_17221533, EPI_ISL_17221534, EPI_ISL_17221537			
see above	Astrazeneca Laboratory	Astrazeneca Laboratory	Wilkins,D., Langedijk,A.C., Lebbink,R.J., Morehouse,C.A., Abram,M.E., Ahani,B., Aksyuk,A.A., Baraldi,E., Brady,T., Chen,A.T., Chi,H., Choi,E.H., Cohen,R., Danilenko,D.M., Gopalakrishnan,V., Greenough,A., Heikkinen,T., Hosoya,M., Keller,C., Kelly,E.J., Kragten-Tabatabaie,L., Martinon-Torres,F., Mascarenas-de Los Santos,A.H., Nunes,M.C., Palomino,M.A., Papenburg,J., Pernica,J.M., Richmond,P., Stein,R.T., Tuffy,K.M., Verwey,C., Esser,M.T., Tabor,D.E. and Bont,L.J.
EPI_ISL_17221667, EPI_ISL_17221668, EPI_ISL_17221669, EPI_ISL_17221670, EPI_ISL_17221672, EPI_ISL_17221673, EPI_ISL_17221675, EPI_ISL_17221677, EPI_ISL_17221678	University of Washington - Virology	University of Washington - Virology	Sereewit,J., Xie,H., Roychoudhury,P. and Greninger,A.L.
EPI_ISL_17287519, EPI_ISL_17287526	Respiratory Virus Unit, Reference Microbiology Services, UK Health Security Agency	Reference Microbiology Services, UK Health Security Agency	Zambon M. Talts T. Mosscrop L. Miah S
EPI_ISL_17383429, EPI_ISL_17383431, EPI_ISL_17383432, EPI_ISL_17383433, EPI_ISL_17383434, EPI_ISL_17383439, EPI_ISL_17383440, EPI_ISL_17383441, EPI_ISL_17383443, EPI_ISL_17383444, EPI_ISL_17383445, EPI_ISL_17383449, EPI_ISL_17383450, EPI_ISL_17383451, EPI_ISL_17383456, EPI_ISL_17383457, EPI_ISL_17383459, EPI_ISL_17383460, EPI_ISL_17383461, EPI_ISL_17383463, EPI_ISL_17383464, EPI_ISL_17383468, EPI_ISL_17383469, EPI_ISL_17383470, EPI_ISL_17383475, EPI_ISL_17383476, EPI_ISL_17383477, EPI_ISL_17383478, EPI_ISL_17383479, EPI_ISL_17383483, EPI_ISL_17383486, EPI_ISL_17383488, EPI_ISL_17383489, EPI_ISL_17383493, EPI_ISL_17383494, EPI_ISL_17383497, EPI_ISL_17383498, EPI_ISL_17383499, EPI_ISL_17383500, EPI_ISL_17383502, EPI_ISL_17383503, EPI_ISL_17383504, EPI_ISL_17383506, EPI_ISL_17383507, EPI_ISL_17383508, EPI_ISL_17383510, EPI_ISL_17383512, EPI_ISL_17383516, EPI_ISL_17383520			
see above	Faculty of Medicine, University of Ljubljana	Faculty of Medicine, University of Ljubljana	Jevsnik Virant,M., Lustrek,M. and Petrovec,M.
EPI_ISL_17401582	Hainan Medical University, Hainan Medical University-The University of Hong Kong Joint Laboratory of Tropical Infectious Diseases	Hainan Medical University, Hainan Medical University-The University of Hong Kong Joint Laboratory of Tropical Infectious Diseases	Jia,Y. and Yin,F.
EPI_ISL_17417577, EPI_ISL_17417587, EPI_ISL_17417588, EPI_ISL_17417590, EPI_ISL_17417593, EPI_ISL_17417605	Centers for Disease Control and Prevention (CDC)	Centers for Disease Control and Prevention (CDC)	Wang,L., Lumandas,M.U., Arguelles,V.L., Morin,J., Aggarwal,M., Tatusov,R. and Vega,E.
EPI_ISL_17559469	Laboratory of Respiratory Viruses and Measles, Oswaldo Cruz Institute, FIOCRUZ	Laboratorio de Alta Complexidade do Instituto Fernandes Figueira - LACIFF	Paola Resende, Fernando Motta, Elisa Cavalcante Pereira, Bruna Mendonça da Silva, Jéssica Graça Macedo de Carvalho, Larissa Macedo Pinto, Victor Guimaraes, Letícia Lima, Letícia Scalioni, Marilda Siqueira on behalf of the Fiocruz COVID-19 Genomic Surveillance Network
EPI_ISL_17583312, EPI_ISL_17583313, EPI_ISL_17583314	West of Scotland Specialist Virology Centre	West of Scotland Specialist Virology Centre	Lynne Ferguson, Imogen Johnston-Menzies, Rory Gunson
EPI_ISL_17594406, EPI_ISL_17594407, EPI_ISL_17594421, EPI_ISL_17594457, EPI_ISL_17594458, EPI_ISL_17594491, EPI_ISL_17594494, EPI_ISL_17594495, EPI_ISL_17594496, EPI_ISL_17594518, EPI_ISL_17594525, EPI_ISL_17594529, EPI_ISL_17594530, EPI_ISL_17594531, EPI_ISL_17594552, EPI_ISL_17594562, EPI_ISL_17594563, EPI_ISL_17594581, EPI_ISL_17594582, EPI_ISL_17594583, EPI_ISL_17594584, EPI_ISL_17594587			
see above	Chang Bing Show Chwan Memorial Hospital	Chang Bing Show Chwan Memorial Hospital	Lee,C.Y.
EPI_ISL_17597842	Laboratório de Virologia - Instituto Nacional de Saúde	Centers for Disease Control and Prevention - United States of America, Laboratório de Virologia - Instituto Nacional de Saúde	Almiro Tivane, Everardo Vega, Amanda Smith, Lijuan Wang, Neuza Nguenha, Loira Machalele, Sadia Alí, Délcio Muteto, Mirela Pale, Aunésia Marrurele, Félix Gundane, Judite Salência
EPI_ISL_1760384, EPI_ISL_1760385, EPI_ISL_1760386, EPI_ISL_1760393, EPI_ISL_1760394, EPI_ISL_1760401, EPI_ISL_1760406, EPI_ISL_1760407, EPI_ISL_1760408, EPI_ISL_1760415, EPI_ISL_1760417, EPI_ISL_1760420, EPI_ISL_1760424, EPI_ISL_1760432, EPI_ISL_1760434, EPI_ISL_1760438, EPI_ISL_1760441, EPI_ISL_1760442, EPI_ISL_1760443, EPI_ISL_1760444			
see above	Royal Children's Hospital, 50 Flemington Rd., Park	WHO Influenza Centre for Reference and Research on Influenza	Angela Todd, Yi-Mo Deng, Annette Alafaci, Naomi Komadina
EPI_ISL_17694319, EPI_ISL_17694320, EPI_ISL_17694321, EPI_ISL_17694322	Division Virologia, Laboratorio de Salud Publica, Tucuman	INEI-ANLIS "Dr. Carlos G. Malbran" Servicio de Virosis Respiratorias	Elsa Baumeister, Andrea Pontoriero, Russo Mara, Martin Avaro, Estefania Benedetti, Maria Elena Dattero, Chamorro Ariana, Garcia Valdez Jackeline, Erika Machicado Macias, Fabian Pardon.
EPI_ISL_17782658	Kitasato University, Infection Control and Immunology	Kitasato University, Infection Control and Immunology	Ito,T., Sawada,A., Saito,A., Ishikura,K., Kawashima,H., Nakayama,T. and Katayama,K.
EPI_ISL_17950090, EPI_ISL_17950092, EPI_ISL_17950093, EPI_ISL_17950096, EPI_ISL_17950104, EPI_ISL_17950106, EPI_ISL_17950109, EPI_ISL_17950113, EPI_ISL_17950123, EPI_ISL_17950128	The First Affiliated Hospital of Bengbu Medical College	The First Affiliated Hospital of Bengbu Medical College	Huang,L.
EPI_ISL_17953819, EPI_ISL_17953821, EPI_ISL_17953848, EPI_ISL_17953869, EPI_ISL_17953879, EPI_ISL_17953888, EPI_ISL_17953898, EPI_ISL_17953900	State Key Laboratory for Diagnosis and Treatment of Infectious Diseases, First Affiliated Hospital, School of Medicine, Zhejiang University	State Key Laboratory for Diagnosis and Treatment of Infectious Diseases, First Affiliated Hospital, School of Medicine, Zhejiang University	Guo,Y.
EPI_ISL_17964351, EPI_ISL_17964353, EPI_ISL_17964354, EPI_ISL_17964357, EPI_ISL_17964358, EPI_ISL_17964361, EPI_ISL_17964362, EPI_ISL_17964364, EPI_ISL_17964376	University of Zambia Medical School	Department of Medical Microbiology, University Medical Centre Utrecht	Annefleur C. Langedijk, Bram Vrancken, Robert Jan Lebbink, Rachel C. Pieciak, Philippe Lemey, Louis J. Bont, Christopher J Gill

EPI_ISL_17975546, EPI_ISL_17975548, EPI_ISL_17975550, EPI_ISL_17975554, EPI_ISL_17975558, EPI_ISL_17975561, EPI_ISL_17975570, EPI_ISL_17975573, EPI_ISL_17975575, EPI_ISL_17975584, EPI_ISL_17975590, EPI_ISL_17975597, EPI_ISL_17975601, EPI_ISL_17975605, EPI_ISL_17975614, EPI_ISL_17975618, EPI_ISL_17975621, EPI_ISL_17975622, EPI_ISL_17975643			
see above	Sapienza University	Sapienza University	Pierangeli,A.
EPI_ISL_18005798, EPI_ISL_18005799, EPI_ISL_18005801, EPI_ISL_18005802, EPI_ISL_18005803, EPI_ISL_18005804, EPI_ISL_18005805, EPI_ISL_18005806, EPI_ISL_18005807, EPI_ISL_18005808, EPI_ISL_18005809, EPI_ISL_18005810, EPI_ISL_18005811, EPI_ISL_18005812, EPI_ISL_18005813, EPI_ISL_18005814, EPI_ISL_18005816, EPI_ISL_18005832, EPI_ISL_18005836, EPI_ISL_18005837, EPI_ISL_18005838, EPI_ISL_18005839, EPI_ISL_18005840, EPI_ISL_18005841, EPI_ISL_18005842, EPI_ISL_18005843, EPI_ISL_18005844, EPI_ISL_18005845, EPI_ISL_18005846, EPI_ISL_18005849, EPI_ISL_18005850, EPI_ISL_18005859, EPI_ISL_18005860, EPI_ISL_18005863, EPI_ISL_18005864, EPI_ISL_18005867, EPI_ISL_18005868, EPI_ISL_18005869, EPI_ISL_18005870, EPI_ISL_18005871, EPI_ISL_18005872, EPI_ISL_18005873, EPI_ISL_18005874, EPI_ISL_18005875, EPI_ISL_18005890, EPI_ISL_18005891, EPI_ISL_18005892, EPI_ISL_18005893, EPI_ISL_18005894, EPI_ISL_18005895, EPI_ISL_18005933, EPI_ISL_18005934, EPI_ISL_18005952			
see above	Robert Koch-Institute Nationales Referenzzentrum für Influenza	Robert Koch-Institute Nationales Referenzzentrum für Influenza	Sophie Kondgen, Janine Reiche
EPI_ISL_18103610, EPI_ISL_18103611, EPI_ISL_18103612, EPI_ISL_18103613, EPI_ISL_18103614, EPI_ISL_18103615, EPI_ISL_18103616, EPI_ISL_18103617, EPI_ISL_18103618, EPI_ISL_18103619, EPI_ISL_18103620, EPI_ISL_18103621, EPI_ISL_18103622, EPI_ISL_18103623, EPI_ISL_18103624, EPI_ISL_18103625, EPI_ISL_18103626, EPI_ISL_18103627, EPI_ISL_18103628, EPI_ISL_18103629, EPI_ISL_18103630			
see above	Centre Pasteur du Cameroun	Centre Pasteur du Cameroun	Moumbeket Yifomnjou,M.H. and Njouom,R.
EPI_ISL_18110967, EPI_ISL_18110968	Queen's University Belfast	Queen's University Belfast	Danielle Allen
EPI_ISL_18119456, EPI_ISL_18119466, EPI_ISL_18119476, EPI_ISL_18119483, EPI_ISL_18119489, EPI_ISL_18119491, EPI_ISL_18119493, EPI_ISL_18119495, EPI_ISL_18119507, EPI_ISL_18119508, EPI_ISL_18119510, EPI_ISL_18119511, EPI_ISL_18119515, EPI_ISL_18119516, EPI_ISL_18119523			
see above	Kenya Medical Research Institute - KEMRI Wellcome Trust Research Programme	Kenya Medical Research Institute - KEMRI Wellcome Trust Research Programme	Odoyo,S.O., Lambisia,A.W., Murunga,N., Lewa,C.S., Maina,G., Okanda,D., Mutunga,M., Agoti,C.N., Nokes,J.D. and Githinji,G.L.
EPI_ISL_18128341, EPI_ISL_18128342, EPI_ISL_18128343, EPI_ISL_18128344	Republic University, Science Faculty, Virology Section	Republic University, Science Faculty, Virology Section	Ramos,N., Panzera,Y., Frabasile,S., Tomas,G., Calleros,L., Marandino,A., Goni,N., Techera,C., Grecco,S., Fuques,E., Coppola,L., Ramas,V., Morel,M.N., Mogdasy,C., Chiparelli,H., Arbiza,J., Perez,R. and Delfraro,A.
EPI_ISL_18143446, EPI_ISL_18143448	The Brotman Baty Institute for Precision Medicine	The Brotman Baty Institute for Precision Medicine	Frazar,C.D., Lee,J., Gamboa,L., McDermot,E., Stone,J., Kolar,T., Han,P.D., Sibley,T.R., Truong,M., Reinhart,D., Wolf,C.R., Boeckh,M., Englund,J.A., Lutz,B.R., Waghmare,A., Viboud,C., Starita,L.M., Shendure,J., Bedford,T. and Chu,H.Y.
EPI_ISL_18143450	The Brotman Baty Institute for Precision Medicine	The Brotman Baty Institute for Precision Medicine	Frazar,C.D., Lee,J., Ryke,E., Gamboa,L., McDermot,E., Stone,J., Kolar,T., Han,P.D., Sibley,T.R., Truong,M., Reinhart,D., Wolf,C.R., Duchin,J., Boeckh,M., Englund,J.A., Lutz,B.R., Waghmare,A., Viboud,C., Starita,L.M., Chu,H.Y., Bedford,T. and Shendure,J.
EPI_ISL_18143452	The Brotman Baty Institute for Precision Medicine	The Brotman Baty Institute for Precision Medicine	Frazar,C.D., Lee,J., Gamboa,L., McDermot,E., Stone,J., Kolar,T., Han,P.D., Sibley,T.R., Truong,M., Reinhart,D., Wolf,C.R., Boeckh,M., Englund,J.A., Lutz,B.R., Waghmare,A., Viboud,C., Starita,L.M., Shendure,J., Bedford,T. and Chu,H.Y.
EPI_ISL_18143453	The Brotman Baty Institute for Precision Medicine	The Brotman Baty Institute for Precision Medicine	Frazar,C.D., Lee,J., Ryke,E., Gamboa,L., McDermot,E., Stone,J., Kolar,T., Han,P.D., Sibley,T.R., Truong,M., Reinhart,D., Wolf,C.R., Duchin,J., Boeckh,M., Englund,J.A., Lutz,B.R., Waghmare,A., Viboud,C., Starita,L.M., Chu,H.Y., Bedford,T. and Shendure,J.
EPI_ISL_18143455, EPI_ISL_18143458, EPI_ISL_18143459, EPI_ISL_18143461	The Brotman Baty Institute for Precision Medicine	The Brotman Baty Institute for Precision Medicine	Frazar,C.D., Lee,J., Gamboa,L., McDermot,E., Stone,J., Kolar,T., Han,P.D., Sibley,T.R., Truong,M., Reinhart,D., Wolf,C.R., Boeckh,M., Englund,J.A., Lutz,B.R., Waghmare,A., Viboud,C., Starita,L.M., Shendure,J., Bedford,T. and Chu,H.Y.
EPI_ISL_18143462, EPI_ISL_18143466, EPI_ISL_18143468, EPI_ISL_18143470, EPI_ISL_18143473	The Brotman Baty Institute for Precision Medicine	The Brotman Baty Institute for Precision Medicine	Frazar,C.D., Lee,J., Ryke,E., Gamboa,L., McDermot,E., Stone,J., Kolar,T., Han,P.D., Sibley,T.R., Truong,M., Reinhart,D., Wolf,C.R., Duchin,J., Boeckh,M., Englund,J.A., Lutz,B.R., Waghmare,A., Viboud,C., Starita,L.M., Chu,H.Y., Bedford,T. and Shendure,J.
EPI_ISL_18143474	The Brotman Baty Institute for Precision Medicine	The Brotman Baty Institute for Precision Medicine	Frazar,C.D., Lee,J., Gamboa,L., McDermot,E., Stone,J., Kolar,T., Han,P.D., Sibley,T.R., Truong,M., Reinhart,D., Wolf,C.R., Boeckh,M., Englund,J.A., Lutz,B.R., Waghmare,A., Viboud,C., Starita,L.M., Shendure,J., Bedford,T. and Chu,H.Y.
EPI_ISL_18143475, EPI_ISL_18143481, EPI_ISL_18143482	The Brotman Baty Institute for Precision Medicine	The Brotman Baty Institute for Precision Medicine	Frazar,C.D., Lee,J., Ryke,E., Gamboa,L., McDermot,E., Stone,J., Kolar,T., Han,P.D., Sibley,T.R., Truong,M., Reinhart,D., Wolf,C.R., Duchin,J., Boeckh,M., Englund,J.A., Lutz,B.R., Waghmare,A., Viboud,C., Starita,L.M., Chu,H.Y., Bedford,T. and Shendure,J.
EPI_ISL_18143485	The Brotman Baty Institute for Precision Medicine	The Brotman Baty Institute for Precision Medicine	Frazar,C.D., Lee,J., Gamboa,L., McDermot,E., Stone,J., Kolar,T., Han,P.D., Sibley,T.R., Truong,M., Reinhart,D., Wolf,C.R., Boeckh,M., Englund,J.A., Lutz,B.R., Waghmare,A., Viboud,C., Starita,L.M., Shendure,J., Bedford,T. and Chu,H.Y.
EPI_ISL_18143487, EPI_ISL_18143488	The Brotman Baty Institute for Precision Medicine	The Brotman Baty Institute for Precision Medicine	Frazar,C.D., Lee,J., Ryke,E., Gamboa,L., McDermot,E., Stone,J., Kolar,T., Han,P.D., Sibley,T.R., Truong,M., Reinhart,D., Wolf,C.R., Duchin,J., Boeckh,M., Englund,J.A., Lutz,B.R., Waghmare,A., Viboud,C., Starita,L.M., Chu,H.Y., Bedford,T. and Shendure,J.
EPI_ISL_18143491	The Brotman Baty Institute for Precision Medicine	The Brotman Baty Institute for Precision Medicine	Frazar,C.D., Lee,J., Gamboa,L., McDermot,E., Stone,J., Kolar,T., Han,P.D., Sibley,T.R., Truong,M., Reinhart,D., Wolf,C.R., Boeckh,M., Englund,J.A., Lutz,B.R., Waghmare,A., Viboud,C., Starita,L.M., Shendure,J., Bedford,T. and Chu,H.Y.
EPI_ISL_18143492, EPI_ISL_18143493	The Brotman Baty Institute for Precision Medicine	The Brotman Baty Institute for Precision Medicine	Frazar,C.D., Lee,J., Ryke,E., Gamboa,L., McDermot,E., Stone,J., Kolar,T., Han,P.D., Sibley,T.R., Truong,M., Reinhart,D., Wolf,C.R., Duchin,J., Boeckh,M., Englund,J.A., Lutz,B.R., Waghmare,A., Viboud,C., Starita,L.M., Chu,H.Y., Bedford,T. and Shendure,J.
EPI_ISL_18143498, EPI_ISL_18143504	The Brotman Baty Institute for Precision Medicine	The Brotman Baty Institute for Precision Medicine	Frazar,C.D., Lee,J., Gamboa,L., McDermot,E., Stone,J., Kolar,T., Han,P.D., Sibley,T.R., Truong,M., Reinhart,D., Wolf,C.R., Boeckh,M., Englund,J.A., Lutz,B.R., Waghmare,A., Viboud,C., Starita,L.M., Shendure,J., Bedford,T. and Chu,H.Y.
EPI_ISL_18143505	The Brotman Baty Institute for Precision Medicine	The Brotman Baty Institute for Precision Medicine	Frazar,C.D., Lee,J., Ryke,E., Gamboa,L., McDermot,E., Stone,J., Kolar,T., Han,P.D., Sibley,T.R., Truong,M., Reinhart,D., Wolf,C.R., Duchin,J., Boeckh,M., Englund,J.A., Lutz,B.R., Waghmare,A., Viboud,C., Starita,L.M., Chu,H.Y., Bedford,T. and Shendure,J.
EPI_ISL_18143516	The Brotman Baty Institute for Precision Medicine	The Brotman Baty Institute for Precision Medicine	Frazar,C.D., Lee,J., Gamboa,L., McDermot,E., Stone,J., Kolar,T., Han,P.D., Sibley,T.R., Truong,M., Reinhart,D., Wolf,C.R., Boeckh,M., Englund,J.A., Lutz,B.R., Waghmare,A., Viboud,C., Starita,L.M., Shendure,J., Bedford,T. and Chu,H.Y.
EPI_ISL_18143519, EPI_ISL_18143521, EPI_ISL_18143523, EPI_ISL_18143525	The Brotman Baty Institute for Precision Medicine	The Brotman Baty Institute for Precision Medicine	Frazar,C.D., Lee,J., Ryke,E., Gamboa,L., McDermot,E., Stone,J., Kolar,T., Han,P.D., Sibley,T.R., Truong,M., Reinhart,D., Wolf,C.R., Duchin,J., Boeckh,M., Englund,J.A., Lutz,B.R., Waghmare,A., Viboud,C., Starita,L.M., Chu,H.Y., Bedford,T. and Shendure,J.
EPI_ISL_18143526	The Brotman Baty Institute for Precision Medicine	The Brotman Baty Institute for Precision Medicine	Frazar,C.D., Lee,J., Gamboa,L., McDermot,E., Stone,J., Kolar,T., Han,P.D., Sibley,T.R., Truong,M., Reinhart,D., Wolf,C.R., Boeckh,M., Englund,J.A., Lutz,B.R., Waghmare,A., Viboud,C., Starita,L.M., Shendure,J., Bedford,T. and Chu,H.Y.
EPI_ISL_18143534	The Brotman Baty Institute for Precision Medicine	The Brotman Baty Institute for Precision Medicine	Frazar,C.D., Lee,J., Ryke,E., Gamboa,L., McDermot,E., Stone,J., Kolar,T., Han,P.D., Sibley,T.R., Truong,M., Reinhart,D., Wolf,C.R., Duchin,J., Boeckh,M., Englund,J.A., Lutz,B.R., Waghmare,A., Viboud,C., Starita,L.M., Chu,H.Y., Bedford,T. and Shendure,J.
EPI_ISL_18143535	The Brotman Baty Institute for Precision Medicine	The Brotman Baty Institute for Precision Medicine	Frazar,C.D., Lee,J., Gamboa,L., McDermot,E., Stone,J., Kolar,T., Han,P.D., Sibley,T.R., Truong,M., Reinhart,D., Wolf,C.R., Boeckh,M., Englund,J.A., Lutz,B.R., Waghmare,A., Viboud,C., Starita,L.M., Shendure,J., Bedford,T. and Chu,H.Y.
EPI_ISL_18143541, EPI_ISL_18143543, EPI_ISL_18143545	The Brotman Baty Institute for Precision Medicine	The Brotman Baty Institute for Precision Medicine	Frazar,C.D., Lee,J., Ryke,E., Gamboa,L., McDermot,E., Stone,J., Kolar,T., Han,P.D., Sibley,T.R., Truong,M., Reinhart,D., Wolf,C.R., Duchin,J., Boeckh,M., Englund,J.A., Lutz,B.R., Waghmare,A., Viboud,C., Starita,L.M., Chu,H.Y., Bedford,T. and Shendure,J.
EPI_ISL_18143549, EPI_ISL_18143552	The Brotman Baty Institute for Precision Medicine	The Brotman Baty Institute for Precision Medicine	Frazar,C.D., Lee,J., Gamboa,L., McDermot,E., Stone,J., Kolar,T., Han,P.D., Sibley,T.R., Truong,M., Reinhart,D., Wolf,C.R., Boeckh,M., Englund,J.A., Lutz,B.R., Waghmare,A., Viboud,C., Starita,L.M., Shendure,J., Bedford,T. and Chu,H.Y.
EPI_ISL_18143558, EPI_ISL_18143559	The Brotman Baty Institute for Precision Medicine	The Brotman Baty Institute for Precision Medicine	Frazar,C.D., Lee,J., Ryke,E., Gamboa,L., McDermot,E., Stone,J., Kolar,T., Han,P.D., Sibley,T.R., Truong,M., Reinhart,D., Wolf,C.R., Duchin,J., Boeckh,M., Englund,J.A., Lutz,B.R., Waghmare,A., Viboud,C., Starita,L.M., Chu,H.Y., Bedford,T. and Shendure,J.
EPI_ISL_18143561	The Brotman Baty Institute for Precision Medicine	The Brotman Baty Institute for Precision Medicine	Frazar,C.D., Lee,J., Gamboa,L., McDermot,E., Stone,J., Kolar,T., Han,P.D., Sibley,T.R., Truong,M., Reinhart,D., Wolf,C.R., Boeckh,M., Englund,J.A., Lutz,B.R., Waghmare,A., Viboud,C., Starita,L.M., Shendure,J., Bedford,T. and Chu,H.Y.
EPI_ISL_18143565	The Brotman Baty Institute for Precision Medicine	The Brotman Baty Institute for Precision Medicine	Frazar,C.D., Lee,J., Ryke,E., Gamboa,L., McDermot,E., Stone,J., Kolar,T., Han,P.D., Sibley,T.R., Truong,M., Reinhart,D., Wolf,C.R., Duchin,J., Boeckh,M., Englund,J.A., Lutz,B.R., Waghmare,A., Viboud,C., Starita,L.M., Chu,H.Y., Bedford,T. and Shendure,J.
EPI_ISL_18143567	The Brotman Baty Institute for Precision Medicine	The Brotman Baty Institute for Precision Medicine	Frazar,C.D., Lee,J., Gamboa,L., McDermot,E., Stone,J., Kolar,T., Han,P.D., Sibley,T.R., Truong,M., Reinhart,D., Wolf,C.R., Boeckh,M., Englund,J.A., Lutz,B.R., Waghmare,A., Viboud,C., Starita,L.M., Shendure,J., Bedford,T. and Chu,H.Y.
EPI_ISL_18143568	The Brotman Baty Institute for Precision Medicine	The Brotman Baty Institute for Precision Medicine	Frazar,C.D., Lee,J., Ryke,E., Gamboa,L., McDermot,E., Stone,J., Kolar,T., Han,P.D., Sibley,T.R., Truong,M., Reinhart,D., Wolf,C.R., Duchin,J., Boeckh,M., Englund,J.A., Lutz,B.R., Waghmare,A., Viboud,C., Starita,L.M., Chu,H.Y., Bedford,T. and Shendure,J.
EPI_ISL_18143569, EPI_ISL_18143570, EPI_ISL_18143574, EPI_ISL_18143577	The Brotman Baty Institute for Precision Medicine	The Brotman Baty Institute for Precision Medicine	Frazar,C.D., Lee,J., Gamboa,L., McDermot,E., Stone,J., Kolar,T., Han,P.D., Sibley,T.R., Truong,M., Reinhart,D., Wolf,C.R., Boeckh,M., Englund,J.A., Lutz,B.R., Waghmare,A., Viboud,C., Starita,L.M., Shendure,J., Bedford,T. and Chu,H.Y.
EPI_ISL_18143579	The Brotman Baty Institute for Precision Medicine	The Brotman Baty Institute for Precision Medicine	Frazar,C.D., Lee,J., Ryke,E., Gamboa,L., McDermot,E., Stone,J., Kolar,T., Han,P.D., Sibley,T.R., Truong,M., Reinhart,D., Wolf,C.R., Duchin,J., Boeckh,M., Englund,J.A., Lutz,B.R., Waghmare,A., Viboud,C., Starita,L.M., Chu,H.Y., Bedford,T. and Shendure,J.
EPI_ISL_18143581, EPI_ISL_18143584	The Brotman Baty Institute for Precision Medicine	The Brotman Baty Institute for Precision Medicine	Frazar,C.D., Lee,J., Gamboa,L., McDermot,E., Stone,J., Kolar,T., Han,P.D., Sibley,T.R., Truong,M., Reinhart,D., Wolf,C.R., Boeckh,M., Englund,J.A., Lutz,B.R., Waghmare,A., Viboud,C., Starita,L.M., Shendure,J., Bedford,T. and Chu,H.Y.
EPI_ISL_18143586	The Brotman Baty Institute for Precision Medicine	The Brotman Baty Institute for Precision Medicine	Frazar,C.D., Lee,J., Ryke,E., Gamboa,L., McDermot,E., Stone,J., Kolar,T., Han,P.D., Sibley,T.R., Truong,M., Reinhart,D., Wolf,C.R., Duchin,J., Boeckh,M., Englund,J.A., Lutz,B.R., Waghmare,A., Viboud,C., Starita,L.M., Chu,H.Y., Bedford,T. and Shendure,J.
EPI_ISL_18143587	The Brotman Baty Institute for Precision Medicine	The Brotman Baty Institute for Precision Medicine	Frazar,C.D., Lee,J., Gamboa,L., McDermot,E., Stone,J., Kolar,T., Han,P.D., Sibley,T.R., Truong,M., Reinhart,D., Wolf,C.R., Boeckh,M., Englund,J.A., Lutz,B.R., Waghmare,A., Viboud,C., Starita,L.M., Shendure,J., Bedford,T. and Chu,H.Y.
EPI_ISL_18143588	The Brotman Baty Institute for Precision Medicine	The Brotman Baty Institute for Precision Medicine	Frazar,C.D., Lee,J., Ryke,E., Gamboa,L., McDermot,E., Stone,J., Kolar,T., Han,P.D., Sibley,T.R., Truong,M., Reinhart,D., Wolf,C.R., Duchin,J., Boeckh,M., Englund,J.A., Lutz,B.R., Waghmare,A., Viboud,C., Starita,L.M., Chu,H.Y., Bedford,T. and Shendure,J.
EPI_ISL_18143590	The Brotman Baty Institute for Precision Medicine	The Brotman Baty Institute for Precision Medicine	Frazar,C.D., Lee,J., Gamboa,L., McDermot,E., Stone,J., Kolar,T., Han,P.D., Sibley,T.R., Truong,M., Reinhart,D., Wolf,C.R., Boeckh,M., Englund,J.A., Lutz,B.R., Waghmare,A., Viboud,C., Starita,L.M., Shendure,J., Bedford,T. and Chu,H.Y.
EPI_ISL_18143593, EPI_ISL_18143594, EPI_ISL_18143595	The Brotman Baty Institute for Precision Medicine	The Brotman Baty Institute for Precision Medicine	Frazar,C.D., Lee,J., Ryke,E., Gamboa,L., McDermot,E., Stone,J., Kolar,T., Han,P.D., Sibley,T.R., Truong,M., Reinhart,D., Wolf,C.R., Duchin,J., Boeckh,M., Englund,J.A., Lutz,B.R., Waghmare,A., Viboud,C., Starita,L.M., Chu,H.Y., Bedford,T. and Shendure,J.
EPI_ISL_18143596, EPI_ISL_18143597, EPI_ISL_18143598, EPI_ISL_18143602	The Brotman Baty Institute for Precision Medicine	The Brotman Baty Institute for Precision Medicine	Frazar,C.D., Lee,J., Gamboa,L., McDermot,E., Stone,J., Kolar,T., Han,P.D., Sibley,T.R., Truong,M., Reinhart,D., Wolf,C.R., Boeckh,M., Englund,J.A., Lutz,B.R., Waghmare,A., Viboud,C., Starita,L.M., Shendure,J., Bedford,T. and Chu,H.Y.
EPI_ISL_18143603, EPI_ISL_18143612, EPI_ISL_18143616	The Brotman Baty Institute for Precision Medicine	The Brotman Baty Institute for Precision Medicine	Frazar,C.D., Lee,J., Ryke,E., Gamboa,L., McDermot,E., Stone,J., Kolar,T., Han,P.D., Sibley,T.R., Truong,M., Reinhart,D., Wolf,C.R., Duchin,J., Boeckh,M., Englund,J.A., Lutz,B.R., Waghmare,A., Viboud,C., Starita,L.M., Chu,H.Y., Bedford,T. and Shendure,J.
EPI_ISL_18143617	The Brotman Baty Institute for Precision Medicine	The Brotman Baty Institute for Precision Medicine	Frazar,C.D., Lee,J., Gamboa,L., McDermot,E., Stone,J., Kolar,T., Han,P.D., Sibley,T.R., Truong,M., Reinhart,D., Wolf,C.R., Boeckh,M., Englund,J.A., Lutz,B.R., Waghmare,A., Viboud,C., Starita,L.M.,

			Shendure,J., Bedford,T. and Chu,H.Y.
EPI_ISL_18143619, EPI_ISL_18143627, EPI_ISL_18143628	The Brotman Baty Institute for Precision Medicine	The Brotman Baty Institute for Precision Medicine	Frazar,C.D., Lee,J., Ryke,E., Gamboa,L., McDermot,E., Stone,J., Kolar,T., Han,P.D., Sibley,T.R., Truong,M., Reinhart,D., Wolf,C.R., Duchin,J., Boeckh,M., Englund,J.A., Lutz,B.R., Waghmare,A., Viboud,C., Starita,L.M., Chu,H.Y., Bedford,T. and Shendure,J.
EPI_ISL_18143630	The Brotman Baty Institute for Precision Medicine	The Brotman Baty Institute for Precision Medicine	Frazar,C.D., Lee,J., Gamboa,L., McDermot,E., Stone,J., Kolar,T., Han,P.D., Sibley,T.R., Truong,M., Reinhart,D., Wolf,C.R., Boeckh,M., Englund,J.A., Lutz,B.R., Waghmare,A., Viboud,C., Starita,L.M., Shendure,J., Bedford,T. and Chu,H.Y.
EPI_ISL_18143631, EPI_ISL_18143632, EPI_ISL_18143633, EPI_ISL_18143634, EPI_ISL_18143635, EPI_ISL_18143637	The Brotman Baty Institute for Precision Medicine	The Brotman Baty Institute for Precision Medicine	Frazar,C.D., Lee,J., Ryke,E., Gamboa,L., McDermot,E., Stone,J., Kolar,T., Han,P.D., Sibley,T.R., Truong,M., Reinhart,D., Wolf,C.R., Duchin,J., Boeckh,M., Englund,J.A., Lutz,B.R., Waghmare,A., Viboud,C., Starita,L.M., Chu,H.Y., Bedford,T. and Shendure,J.
EPI_ISL_18143646	The Brotman Baty Institute for Precision Medicine	The Brotman Baty Institute for Precision Medicine	Frazar,C.D., Lee,J., Gamboa,L., McDermot,E., Stone,J., Kolar,T., Han,P.D., Sibley,T.R., Truong,M., Reinhart,D., Wolf,C.R., Boeckh,M., Englund,J.A., Lutz,B.R., Waghmare,A., Viboud,C., Starita,L.M., Shendure,J., Bedford,T. and Chu,H.Y.
EPI_ISL_18143650, EPI_ISL_18143653, EPI_ISL_18143659, EPI_ISL_18143661	The Brotman Baty Institute for Precision Medicine	The Brotman Baty Institute for Precision Medicine	Frazar,C.D., Lee,J., Ryke,E., Gamboa,L., McDermot,E., Stone,J., Kolar,T., Han,P.D., Sibley,T.R., Truong,M., Reinhart,D., Wolf,C.R., Duchin,J., Boeckh,M., Englund,J.A., Lutz,B.R., Waghmare,A., Viboud,C., Starita,L.M., Chu,H.Y., Bedford,T. and Shendure,J.
EPI_ISL_18143665, EPI_ISL_18143666, EPI_ISL_18143667	The Brotman Baty Institute for Precision Medicine	The Brotman Baty Institute for Precision Medicine	Frazar,C.D., Lee,J., Gamboa,L., McDermot,E., Stone,J., Kolar,T., Han,P.D., Sibley,T.R., Truong,M., Reinhart,D., Wolf,C.R., Boeckh,M., Englund,J.A., Lutz,B.R., Waghmare,A., Viboud,C., Starita,L.M., Shendure,J., Bedford,T. and Chu,H.Y.
EPI_ISL_18143669, EPI_ISL_18143671, EPI_ISL_18143673, EPI_ISL_18143674	The Brotman Baty Institute for Precision Medicine	The Brotman Baty Institute for Precision Medicine	Frazar,C.D., Lee,J., Ryke,E., Gamboa,L., McDermot,E., Stone,J., Kolar,T., Han,P.D., Sibley,T.R., Truong,M., Reinhart,D., Wolf,C.R., Duchin,J., Boeckh,M., Englund,J.A., Lutz,B.R., Waghmare,A., Viboud,C., Starita,L.M., Chu,H.Y., Bedford,T. and Shendure,J.
EPI_ISL_18143676	The Brotman Baty Institute for Precision Medicine	The Brotman Baty Institute for Precision Medicine	Frazar,C.D., Lee,J., Gamboa,L., McDermot,E., Stone,J., Kolar,T., Han,P.D., Sibley,T.R., Truong,M., Reinhart,D., Wolf,C.R., Boeckh,M., Englund,J.A., Lutz,B.R., Waghmare,A., Viboud,C., Starita,L.M., Shendure,J., Bedford,T. and Chu,H.Y.
EPI_ISL_18143678, EPI_ISL_18143681, EPI_ISL_18143682, EPI_ISL_18143683	The Brotman Baty Institute for Precision Medicine	The Brotman Baty Institute for Precision Medicine	Frazar,C.D., Lee,J., Ryke,E., Gamboa,L., McDermot,E., Stone,J., Kolar,T., Han,P.D., Sibley,T.R., Truong,M., Reinhart,D., Wolf,C.R., Duchin,J., Boeckh,M., Englund,J.A., Lutz,B.R., Waghmare,A., Viboud,C., Starita,L.M., Chu,H.Y., Bedford,T. and Shendure,J.
EPI_ISL_18143684, EPI_ISL_18143689, EPI_ISL_18143690	The Brotman Baty Institute for Precision Medicine	The Brotman Baty Institute for Precision Medicine	Frazar,C.D., Lee,J., Gamboa,L., McDermot,E., Stone,J., Kolar,T., Han,P.D., Sibley,T.R., Truong,M., Reinhart,D., Wolf,C.R., Boeckh,M., Englund,J.A., Lutz,B.R., Waghmare,A., Viboud,C., Starita,L.M., Shendure,J., Bedford,T. and Chu,H.Y.
EPI_ISL_18143691	The Brotman Baty Institute for Precision Medicine	The Brotman Baty Institute for Precision Medicine	Frazar,C.D., Lee,J., Ryke,E., Gamboa,L., McDermot,E., Stone,J., Kolar,T., Han,P.D., Sibley,T.R., Truong,M., Reinhart,D., Wolf,C.R., Duchin,J., Boeckh,M., Englund,J.A., Lutz,B.R., Waghmare,A., Viboud,C., Starita,L.M., Chu,H.Y., Bedford,T. and Shendure,J.
EPI_ISL_18143693, EPI_ISL_18143696, EPI_ISL_18143697	The Brotman Baty Institute for Precision Medicine	The Brotman Baty Institute for Precision Medicine	Frazar,C.D., Lee,J., Gamboa,L., McDermot,E., Stone,J., Kolar,T., Han,P.D., Sibley,T.R., Truong,M., Reinhart,D., Wolf,C.R., Boeckh,M., Englund,J.A., Lutz,B.R., Waghmare,A., Viboud,C., Starita,L.M., Shendure,J., Bedford,T. and Chu,H.Y.
EPI_ISL_18143698, EPI_ISL_18143702, EPI_ISL_18143705, EPI_ISL_18143708, EPI_ISL_18143710, EPI_ISL_18143712	The Brotman Baty Institute for Precision Medicine	The Brotman Baty Institute for Precision Medicine	Frazar,C.D., Lee,J., Ryke,E., Gamboa,L., McDermot,E., Stone,J., Kolar,T., Han,P.D., Sibley,T.R., Truong,M., Reinhart,D., Wolf,C.R., Duchin,J., Boeckh,M., Englund,J.A., Lutz,B.R., Waghmare,A., Viboud,C., Starita,L.M., Chu,H.Y., Bedford,T. and Shendure,J.
EPI_ISL_18143713, EPI_ISL_18143716	The Brotman Baty Institute for Precision Medicine	The Brotman Baty Institute for Precision Medicine	Frazar,C.D., Lee,J., Gamboa,L., McDermot,E., Stone,J., Kolar,T., Han,P.D., Sibley,T.R., Truong,M., Reinhart,D., Wolf,C.R., Boeckh,M., Englund,J.A., Lutz,B.R., Waghmare,A., Viboud,C., Starita,L.M., Shendure,J., Bedford,T. and Chu,H.Y.
EPI_ISL_18272383, EPI_ISL_18272384, EPI_ISL_18272385, EPI_ISL_18272395, EPI_ISL_18272396, EPI_ISL_18272397, EPI_ISL_18272406, EPI_ISL_18272407, EPI_ISL_18272408, EPI_ISL_18272409, EPI_ISL_18272410, EPI_ISL_18272411, EPI_ISL_18272412, EPI_ISL_18272413, EPI_ISL_18272414, EPI_ISL_18272415, EPI_ISL_18277120, EPI_ISL_18277121, EPI_ISL_18277122, EPI_ISL_18277123, EPI_ISL_18277124, EPI_ISL_18277125, EPI_ISL_18277126, EPI_ISL_18277127			
see above	West of Scotland Specialist Virology Centre	West of Scotland Specialist Virology Centre	Lynne Ferguson, Imogen Johnston-Menzies, Rory Gunson
EPI_ISL_18334082, EPI_ISL_18334083, EPI_ISL_18334084, EPI_ISL_18334089, EPI_ISL_18334090, EPI_ISL_18334091, EPI_ISL_18334097, EPI_ISL_18334099, EPI_ISL_18334103, EPI_ISL_18334110, EPI_ISL_18334112, EPI_ISL_18334115, EPI_ISL_18334117, EPI_ISL_18334120, EPI_ISL_18334122, EPI_ISL_18334124, EPI_ISL_18334126, EPI_ISL_18334131, EPI_ISL_18334135, EPI_ISL_18334136, EPI_ISL_18334139, EPI_ISL_18334140, EPI_ISL_18334141, EPI_ISL_18334144, EPI_ISL_18334146, EPI_ISL_18334147, EPI_ISL_18334148, EPI_ISL_18334150, EPI_ISL_18334152, EPI_ISL_18334153, EPI_ISL_18334155			
see above	Tianjin Children's Hospital, Tianjin Pediatric Research Institute	Tianjin Children's Hospital, Tianjin Pediatric Research Institute	Hou,M., Meng,C., Dong,L. and Wang,H.
EPI_ISL_18334242, EPI_ISL_18334245, EPI_ISL_18334249, EPI_ISL_18334250, EPI_ISL_18334253, EPI_ISL_18334255, EPI_ISL_18334257, EPI_ISL_18334261, EPI_ISL_18334262, EPI_ISL_18334272, EPI_ISL_18334274, EPI_ISL_18334278, EPI_ISL_18334280, EPI_ISL_18334281, EPI_ISL_18334282, EPI_ISL_18334284, EPI_ISL_18334290, EPI_ISL_18334291, EPI_ISL_18334294, EPI_ISL_18334301, EPI_ISL_18334305, EPI_ISL_18334307, EPI_ISL_18334311, EPI_ISL_18334317, EPI_ISL_18334320, EPI_ISL_18334327, EPI_ISL_18334329, EPI_ISL_18334330, EPI_ISL_18334334, EPI_ISL_18334335, EPI_ISL_18334339, EPI_ISL_18334340, EPI_ISL_18334344, EPI_ISL_18334345, EPI_ISL_18334348, EPI_ISL_18334352, EPI_ISL_18334354, EPI_ISL_18334356, EPI_ISL_18334362, EPI_ISL_18334364, EPI_ISL_18334365, EPI_ISL_18334366, EPI_ISL_18334367, EPI_ISL_18334371, EPI_ISL_18334374, EPI_ISL_18334378, EPI_ISL_18334379, EPI_ISL_18334380, EPI_ISL_18334381, EPI_ISL_18334383, EPI_ISL_18334386, EPI_ISL_18334390, EPI_ISL_18334391, EPI_ISL_18334393, EPI_ISL_18334399, EPI_ISL_18334400, EPI_ISL_18334402, EPI_ISL_18334404, EPI_ISL_18334410			
see above	National Influenza Center, Virology Laboratory	National Influenza Center, Virology Laboratory	Simusika,P., Okamoto,M., Dapat,C., Muleya,W., Saito,M., Azam,S., Mwape,I., Mwila,E., Monze,M. and Oshitani,H.
EPI_ISL_1834174, EPI_ISL_1834175, EPI_ISL_1834176, EPI_ISL_1834177, EPI_ISL_1834178, EPI_ISL_1834179, EPI_ISL_1834180, EPI_ISL_1834181, EPI_ISL_1834182, EPI_ISL_1834183, EPI_ISL_1834184			
see above	Respiratory Virus Unit, National Infection Service, Public Health England	National Infection Service, Public Health England	Zambon M, Talts T, Ellis J, Miah S, Platt S
EPI_ISL_18356576, EPI_ISL_18356578, EPI_ISL_18356582, EPI_ISL_18356587, EPI_ISL_18356590, EPI_ISL_18356591, EPI_ISL_18356594, EPI_ISL_18356595, EPI_ISL_18356597, EPI_ISL_18356603, EPI_ISL_18356605, EPI_ISL_18356607, EPI_ISL_18356614, EPI_ISL_18356615, EPI_ISL_18356621, EPI_ISL_18356623, EPI_ISL_18356629, EPI_ISL_18356630, EPI_ISL_18356635, EPI_ISL_18356636, EPI_ISL_18356651, EPI_ISL_18356670, EPI_ISL_18356676, EPI_ISL_18356682			
see above	Hospital Central de Asturias, Microbiologia	Hospital Central de Asturias, Microbiologia	Rojo-Alba,S., Perez-Martinez,Z., Boga,J., Alvarez-Arguelles,M.E., Gonzalez-Alba,J.M. and Melon,S.
EPI_ISL_18406013, EPI_ISL_18406014, EPI_ISL_18406015, EPI_ISL_18406016, EPI_ISL_18406047, EPI_ISL_18406048, EPI_ISL_18406049, EPI_ISL_18406050, EPI_ISL_18406051, EPI_ISL_18406052, EPI_ISL_18406053, EPI_ISL_18406054, EPI_ISL_18406055, EPI_ISL_18406056, EPI_ISL_18406057, EPI_ISL_18406058, EPI_ISL_18406059, EPI_ISL_18406060, EPI_ISL_18406061, EPI_ISL_18406062, EPI_ISL_18406063, EPI_ISL_18406064, EPI_ISL_18406066, EPI_ISL_18406067			
see above	Niigata University (IDRC) Infectious Diseases Research Center	Niigata University (IDRC) Infectious Diseases Research Center	Wint Wint Phyu, Reiko Saito
EPI_ISL_18446810, EPI_ISL_18446811, EPI_ISL_18446812, EPI_ISL_18446814, EPI_ISL_18446815, EPI_ISL_18446816, EPI_ISL_18446817, EPI_ISL_18446818, EPI_ISL_18446819, EPI_ISL_18446820, EPI_ISL_18446821, EPI_ISL_18446822, EPI_ISL_18446823, EPI_ISL_18446824, EPI_ISL_18446825, EPI_ISL_18446826, EPI_ISL_18446827, EPI_ISL_18446828, EPI_ISL_18446829, EPI_ISL_18446830, EPI_ISL_18446832, EPI_ISL_18446833, EPI_ISL_18446834, EPI_ISL_18446835, EPI_ISL_18446837, EPI_ISL_18446838, EPI_ISL_18446840, EPI_ISL_18446841, EPI_ISL_18446842, EPI_ISL_18446843, EPI_ISL_18446844, EPI_ISL_18446845, EPI_ISL_18446846, EPI_ISL_18446847, EPI_ISL_18446848			
see above	Vittore Buzzi Children Hospital, Pediatric Department	Laboratory of Infectious Diseases, Department of Biomedical and Clinical Sciences, University of Milan	Alessia Lai, Annalisa Bergna, Valentina Fabiano, Carla della Ventura, Giulia Fumagalli, Alessandra Mari, Martina Loiodice, Gian Vincenzo Zuccotti, Gianguglielmo Zehender
EPI_ISL_18463166, EPI_ISL_18463168, EPI_ISL_18463169, EPI_ISL_18463175, EPI_ISL_18463189, EPI_ISL_18463191, EPI_ISL_18463192	Hospital Universitario Severo Ochoa	Instituto de Salud Carlos III	Iglesias-Caballero, M. Camarero, S. Pozo, F. Casas, I. Jiménez, P. Zaballos, A.Varona, S. Monzón, S.Cuesta, I. García-García, M.L.
EPI_ISL_18470191	Hospital Santa María Nai	Instituto de Salud Carlos III	Iglesias-Caballero, M. Camarero, S. Pozo, F. Casas, I. Jiménez, P. Zaballos, A.Varona, S. Monzón, S.Cuesta, I. García, J.
EPI_ISL_18470198	Hospital Universitario Severo Ochoa	Instituto de Salud Carlos III	Iglesias-Caballero, M. Camarero, S. Pozo, F. Casas, I. Jiménez, P. Zaballos, A.Varona, S. Monzón, S.Cuesta, I. García-García, M.L.
EPI_ISL_18470203	Hospital Universitario Severo Ochoa	Instituto de Salud Carlos III	Iglesias-Caballero, M. Camarero, S. Pozo, F. Casas, I. Jiménez, P. Zaballos, A.Varona, S. Monzón, S.Cuesta, I. Calvo, C.
EPI_ISL_18474127	Hospital Santa María Nai	Instituto de Salud Carlos III	Iglesias-Caballero, M. Camarero, S. Pozo, F. Casas, I. Jiménez, P. Zaballos, A.Varona, S. Monzón, S.Cuesta, I. García, J.
EPI_ISL_18482716, EPI_ISL_18482721, EPI_ISL_18482726, EPI_ISL_18482727, EPI_ISL_18482732, EPI_ISL_18482733, EPI_ISL_18482737, EPI_ISL_18482738, EPI_ISL_18482739, EPI_ISL_18482742, EPI_ISL_18482743, EPI_ISL_18482745, EPI_ISL_18482747, EPI_ISL_18482750, EPI_ISL_18482755, EPI_ISL_18482758, EPI_ISL_18482764, EPI_ISL_18482768, EPI_ISL_18482770, EPI_ISL_18482774, EPI_ISL_18482783, EPI_ISL_18482789, EPI_ISL_18482790, EPI_ISL_18482794, EPI_ISL_18482796, EPI_ISL_18482798, EPI_ISL_18482803, EPI_ISL_18482806, EPI_ISL_18482812, EPI_ISL_18482818			
see above	Beijing Children's Hospital, Capital Medical University, Laboratory of Infection and Virology	Beijing Children's Hospital, Capital Medical University, Laboratory of Infection and Virology	Li,F., Zhu,Y. and Chen,X.
EPI_ISL_2156816, EPI_ISL_2156818, EPI_ISL_2156819	Royal Children's Hospital	WHO Influenza Centre for Reference and Research on Influenza	Angela Todd, Yi-Mo Deng, Annette Alafaci, Naomi Komadina
EPI_ISL_2543763	Royal Children's Hospital	WHO Collaborating Centre for Reference and Research on Influenza	Xiaomin Dong, Annette Alafaci,Yi-Mo Deng, Ammar Aziz, Naomi Komadina
EPI_ISL_2543848	Monash Medical Centre	WHO Collaborating Centre for Reference and Research on Influenza	Xiaomin Dong, Michelle Francis, Tony Korman, Yi-Mo Deng, Ammar Aziz, Naomi Komadina
EPI_ISL_2543849, EPI_ISL_2543850, EPI_ISL_2543851, EPI_ISL_2543852, EPI_ISL_2543853	Royal Children's Hospital	WHO Collaborating Centre for Reference and Research on Influenza	Xiaomin Dong, Annette Alafaci,Yi-Mo Deng, Ammar Aziz, Naomi Komadina
EPI_ISL_2543890	NIC, National Institute of Health	WHO Collaborating Centre for Reference and Research on Influenza	Xiaomin Dong, Pilailuk Akkapaiboon Okada, Yi-Mo Deng, Ammar Aziz, Naomi Komadina
EPI_ISL_2558987	Department of Infectious Diseases and Pathobiology, Institute of Virology and Immunology (IVI)	Department of Infectious Diseases and Pathobiology, Institute of Virology and Immunology (IVI)	Thao,T.T.N., Labroussaa,F., Ebert,N., Stalder,H., Dijkman,R., Jores,J., Thiel,V., Bittel,P., Suter-Riniker,F. and Kelly,J.
EPI_ISL_2575367	Suguru Takeuchi Nagoya University Graduate School of Medicine, Pediatrics	Suguru Takeuchi Nagoya University Graduate School of Medicine, Pediatrics	Takeuchi,S., Kawada,J. and Ito,Y.
EPI_ISL_2575571, EPI_ISL_2575606, EPI_ISL_2575615	Department of Experimental Modeling and Pathogenesis of Infectious Diseases, Federal Research Center of Fundamental and Translational Medicine	Department of Experimental Modeling and Pathogenesis of Infectious Diseases, Federal Research Center of Fundamental and Translational Medicine	Dubovitskiy,N.A., Sobolev,I.A., Kurskaya,O.G., Sharshov,K.A., Anoshina,A.V., Leonova,N.V., Murashkina,T.A., Solomatina,M.V., Derko,A.A., Saroyan,T.A., Kabilov,M.R., Alikina,T.Y. and Shestopalov,A.M.
EPI_ISL_2575957, EPI_ISL_2575971, EPI_ISL_2575972, EPI_ISL_2575973, EPI_ISL_2575974, EPI_ISL_2576441, EPI_ISL_2576442, EPI_ISL_2576443, EPI_ISL_2576489, EPI_ISL_2576490, EPI_ISL_2576491, EPI_ISL_2576492, EPI_ISL_2576493, EPI_ISL_2576494, EPI_ISL_2576495, EPI_ISL_2576496, EPI_ISL_2576497, EPI_ISL_2576498, EPI_ISL_2576499, EPI_ISL_2576500, EPI_ISL_2576501, EPI_ISL_2576502, EPI_ISL_2576503, EPI_ISL_2576504, EPI_ISL_2576505, EPI_ISL_2576506, EPI_ISL_2576507, EPI_ISL_2578532, EPI_ISL_2578534, EPI_ISL_2578535, EPI_ISL_2578536, EPI_ISL_2578537, EPI_ISL_2578538, EPI_ISL_2578539, EPI_ISL_2578540, EPI_ISL_2578541, EPI_ISL_2578542, EPI_ISL_2578543, EPI_ISL_2578656			
see above	Wint Wint Phyu Niigat University, International Health, Public Health	Wint Wint Phyu Niigat University, International Health, Public Health	Phyu,W.W. and Saito,R.
EPI_ISL_2584738	Laboratory Medicine, UW Virology	Laboratory Medicine, UW Virology	Lin,M.J., Tait,A. and Greninger,A.L.
EPI_ISL_2839180, EPI_ISL_2839185, EPI_ISL_2839193, EPI_ISL_2839366, EPI_ISL_2839368, EPI_ISL_2839374	Centre for Infectious Diseases and Microbiology Laboratory Services	Centre for Infectious Diseases and Microbiology Laboratory Services	"John-Sebastian Eden, Jen Kok, Dominic Dwyer, Edward Holmes, Philip Britton, Alison Kesson, Elena Cutmore, Rachel Tulloch, Bethany Horsburgh"
EPI_ISL_2839376	PathWest Laboratory Medicine WA Microbial Surveillance Unit	PathWest Laboratory Medicine WA Microbial Surveillance Unit	"Chisha Sikazwe, Avram Levy, David Smith, Chris Blyth, Alice Michie, Cara Minney-Smith, David Speers"
EPI_ISL_2839393, EPI_ISL_2839394, EPI_ISL_2839416, EPI_ISL_2839417	Centre for Infectious Diseases and Microbiology Laboratory Services	Centre for Infectious Diseases and Microbiology Laboratory Services	"John-Sebastian Eden, Jen Kok, Dominic Dwyer, Edward Holmes, Philip Britton, Alison Kesson, Elena Cutmore, Rachel Tulloch, Bethany Horsburgh"

EPI_ISL_2839434	Departments of Clinical Microbiology and Infectious Diseases	Centre for Infectious Diseases and Microbiology Laboratory Services	"John-Sebastian Eden, Jen Kok, Dominic Dwyer, Edward Holmes, Philip Britton, Alison Kesson, Elena Cutmore, Rachel Tulloch, Bethany Horsburgh"
EPI_ISL_2839435, EPI_ISL_2839436, EPI_ISL_2839437, EPI_ISL_2839442, EPI_ISL_2839449	Centre for Infectious Diseases and Microbiology Laboratory Services	Centre for Infectious Diseases and Microbiology Laboratory Services	"John-Sebastian Eden, Jen Kok, Dominic Dwyer, Edward Holmes, Philip Britton, Alison Kesson, Elena Cutmore, Rachel Tulloch, Bethany Horsburgh"
EPI_ISL_2839450	Departments of Clinical Microbiology and Infectious Diseases	Centre for Infectious Diseases and Microbiology Laboratory Services	"John-Sebastian Eden, Jen Kok, Dominic Dwyer, Edward Holmes, Philip Britton, Alison Kesson, Elena Cutmore, Rachel Tulloch, Bethany Horsburgh"
EPI_ISL_2839452	Centre for Infectious Diseases and Microbiology Laboratory Services	Centre for Infectious Diseases and Microbiology Laboratory Services	"John-Sebastian Eden, Jen Kok, Dominic Dwyer, Edward Holmes, Philip Britton, Alison Kesson, Elena Cutmore, Rachel Tulloch, Bethany Horsburgh"
EPI_ISL_2839453	PathWest Laboratory Medicine WA Microbial Surveillance Unit	PathWest Laboratory Medicine WA Microbial Surveillance Unit	"Chisha Sikazwe, Avram Levy, David Smith, Chris Blyth, Alice Michie, Cara Minney-Smith, David Speers"
EPI_ISL_2839454	Centre for Infectious Diseases and Microbiology Laboratory Services	Centre for Infectious Diseases and Microbiology Laboratory Services	"John-Sebastian Eden, Jen Kok, Dominic Dwyer, Edward Holmes, Philip Britton, Alison Kesson, Elena Cutmore, Rachel Tulloch, Bethany Horsburgh"
EPI_ISL_2839455, EPI_ISL_2839456	Departments of Clinical Microbiology and Infectious Diseases	Centre for Infectious Diseases and Microbiology Laboratory Services	"John-Sebastian Eden, Jen Kok, Dominic Dwyer, Edward Holmes, Philip Britton, Alison Kesson, Elena Cutmore, Rachel Tulloch, Bethany Horsburgh"
EPI_ISL_2839457	Centre for Infectious Diseases and Microbiology Laboratory Services	Centre for Infectious Diseases and Microbiology Laboratory Services	"John-Sebastian Eden, Jen Kok, Dominic Dwyer, Edward Holmes, Philip Britton, Alison Kesson, Elena Cutmore, Rachel Tulloch, Bethany Horsburgh"
EPI_ISL_2896251, EPI_ISL_2896254	NIC, National Institute of Health	WHO Collaborating Centre for Reference and Research on Influenza	Xiaomin Dong, Pilailuk Akkapaiboon Okada, Yi-Mo Deng, Ammar Aziz, Naomi Komadina
EPI_ISL_2989828	Royal Children's Hospital	WHO Collaborating Centre for Reference and Research on Influenza	Xiaomin Dong, Annette Alafaci,Yi-Mo Deng, Ammar Aziz, Naomi Komadina
EPI_ISL_4203848, EPI_ISL_4204345	Sub Departamento Genetica Molecular	Instituto de Salud Publica de Chile Seccion Genetica Molecular	Jorge Fernandez
EPI_ISL_5522628, EPI_ISL_5522629	Institut Pasteur de Côte d'Ivoire	WHO Collaborating Centre for Reference and Research on Influenza	Xiaomin Dong, Herve Kadjo, Yi-Mo Deng, Ammar Aziz, Naomi Komadina
EPI_ISL_5930147	Institut Pasteur de Cote d'ivoire	WHO Collaborating Centre for Reference and Research on Influenza	Xiaomin Dong, Herve Kadjo, Yi-Mo Deng, Ammar Aziz, Naomi Komadina
EPI_ISL_5930151	Royal Children's Hospital	WHO Collaborating Centre for Reference and Research on Influenza	Xiaomin Dong, Annette Alafaci,Yi-Mo Deng, Ammar Aziz, Naomi Komadina
EPI_ISL_6174137	Cote d'ivoire, Institut Pasteur de Cote d'ivoire	WHO Collaborating Centre for Reference and Research on Influenza	Xiaomin Dong, Herve Kadjo, Yi-Mo Deng, Ammar Aziz, Naomi Komadina
EPI_ISL_6174138, EPI_ISL_6208709, EPI_ISL_6208711, EPI_ISL_6208713, EPI_ISL_6208714, EPI_ISL_6208717, EPI_ISL_6208721, EPI_ISL_6208722, EPI_ISL_6208728	Egypt, Central Public Health Laboratory (CPHL)	WHO Collaborating Centre for Reference and Research on Influenza	Xiaomin Dong,Yi-Mo Deng, Amel Naguib, Naomi Komadina
EPI_ISL_6268437, EPI_ISL_6268455, EPI_ISL_6268456, EPI_ISL_6268457, EPI_ISL_6268594, EPI_ISL_6268595, EPI_ISL_6268596, EPI_ISL_6268597, EPI_ISL_6268598, EPI_ISL_6268599, EPI_ISL_6268606, EPI_ISL_6268607, EPI_ISL_6268611, EPI_ISL_6268612, EPI_ISL_6268617, EPI_ISL_6268618, EPI_ISL_6268620, EPI_ISL_6268621, EPI_ISL_6268622, EPI_ISL_6268623, EPI_ISL_6268625, EPI_ISL_6268626, EPI_ISL_6268627, EPI_ISL_6268628, EPI_ISL_6314463, EPI_ISL_6314464, EPI_ISL_6314465, EPI_ISL_6314466, EPI_ISL_6314467, EPI_ISL_6314468, EPI_ISL_6314469, EPI_ISL_6314470, EPI_ISL_6314471, EPI_ISL_6314585, EPI_ISL_6314640, EPI_ISL_6314641, EPI_ISL_6314642, EPI_ISL_6314658, EPI_ISL_6314659, EPI_ISL_6314660, EPI_ISL_6314661, EPI_ISL_6314662, EPI_ISL_6314663			
see above	Department of Respiratory Medicine, Wuhan Children's Hospital	CAS Key Laboratory of Special Pathogens and Biosafety, Chinese Academy of Sciences	Decheng Wang, Yi Yan, Ying Li, Xiaoxia Lu, Di Liu
EPI_ISL_732356, EPI_ISL_732358, EPI_ISL_732362, EPI_ISL_732363, EPI_ISL_732364, EPI_ISL_732365, EPI_ISL_732366, EPI_ISL_732367, EPI_ISL_732371, EPI_ISL_732373	Respiratory Virus Unit, National Infection Service, Public Health England	National Infection Service, Public Health England	Zambon M, Talts T, Ellis J, Miah S, Platt S
EPI_ISL_9003920	National Institute for Communicable Diseases of the National Health Laboratory Service	National Institute for Communicable Diseases of the National Health Laboratory Service	Amoako DG, Everatt J, Mohale T, Ntuli N, Mahlangu B, Mnguni A, Ismail A, Bhiman JN, Wolter N
EPI_ISL_9248382	Laboratorio de Salud Publica	Instituto Nacional de Enfermedades Infecciosas ANLIS "Carlos G. Malbran"	Elsa Baumeister, Andrea Pontoriero, Russo Mara, Martin Avaro, Estefania Benedetti, Maria Elena Dattero, Ana Campos, Fabian Pardon, Erika Machicado Macias, Carlos Vargas
EPI_ISL_9257522, EPI_ISL_9257599, EPI_ISL_9257845, EPI_ISL_9257940	Laboratorio de Salud Publica	Instituto Nacional Enfermedades Infecciosas ANLIS "Carlos G. Malbran"	Elsa Baumeister, Andrea Pontoriero, Russo Mara, Martin Avaro, Estefania Benedetti, Maria Elena Dattero, Ana Campos, Fabian Pardon, Erika Machicado Macias, Carlos Vargas
EPI_ISL_9347043, EPI_ISL_9347044, EPI_ISL_9347045	Laboratorio de Salud Publica	Instituto Nacional Enfermedades Infecciosas "Carlos G. Malbran"	Elsa Baumeister, Andrea Pontoriero, Russo Mara, Martin Avaro, Estefania Benedetti, Maria Elena Dattero, Ana Campos, Fabian Pardon, Erika Machicado Macias, Carlos Vargas
EPI_ISL_9347046	Hospital Perrando	Instituto Nacional Enfermedades Infecciosas "Carlos G. Malbran"	Elsa Baumeister, Andrea Pontoriero, Russo Mara, Martin Avaro, Estefania Benedetti, Maria Elena Dattero, Ana Campos, Fabian Pardon, Erika Machicado Macias, Carlos Vargas
EPI_ISL_9347071, EPI_ISL_9347074, EPI_ISL_9347113	Laboratorio de Salud Publica	Instituto Nacional Enfermedades Infecciosas "Carlos G. Malbran"	Elsa Baumeister, Andrea Pontoriero, Russo Mara, Martin Avaro, Estefania Benedetti, Maria Elena Dattero, Ana Campos, Fabian Pardon, Erika Machicado Macias, Carlos Vargas
EPI_ISL_9695692, EPI_ISL_9695803, EPI_ISL_9695812, EPI_ISL_9695868, EPI_ISL_9695951, EPI_ISL_9696131, EPI_ISL_9696161, EPI_ISL_9696231	Ricardo Gutierrez Children's Hospital, Virology Laboratory, sequencing area.	Ricardo Gutierrez Children's Hospital, Virology Laboratory, sequencing area.	Acuña, Dolores; Goya, Stephanie; Nabaes Jodar, Mercedes S.; Grandis, Érica; María Cristina Alvarez López, María Elina Acevedo, Oscar Jacquez, Sofía Alexay, Mariángeles Barreda Fink, María Emilia Villegas, Raquel Barquez, Estela Chascón, Jorgelina Caruso, Karina Zacarías, Cristian Díaz, Oscar Luna, Cristian Turchiaro, Julián Cipelli, Guillermo Thomas, Carla Medina, Natalia Labarta. Mistchenko, Alicia S.; Viegas, Mariana.
EPI_ISL_9696323, EPI_ISL_9696363	Ricardo Gutierrez Children's Hospital, Virology Laboratory, sequencing area	Ricardo Gutierrez Children's Hospital, Virology Laboratory, sequencing area	Acuña, Dolores; Goya, Stephanie; Nabaes Jodar, Mercedes S.; Grandis, Érica; María Cristina Alvarez López, María Elina Acevedo, Oscar Jacquez, Sofía Alexay, Mariángeles Barreda Fink, María Emilia Villegas, Raquel Barquez, Estela Chascón, Jorgelina Caruso, Karina Zacarías, Cristian Díaz, Oscar Luna, Cristian Turchiaro, Julián Cipelli, Guillermo Thomas, Carla Medina, Natalia Labarta. Mistchenko, Alicia S.; Viegas, Mariana.
EPI_ISL_9696386, EPI_ISL_9696417	Ricardo Gutierrez Children's Hospital, Virology Laboratory, sequencing area.	Ricardo Gutierrez Children's Hospital, Virology Laboratory, sequencing area.	Acuña, Dolores; Goya, Stephanie; Nabaes Jodar, Mercedes S.; Grandis, Érica; María Cristina Alvarez López, María Elina Acevedo, Oscar Jacquez, Sofía Alexay, Mariángeles Barreda Fink, María Emilia Villegas, Raquel Barquez, Estela Chascón, Jorgelina Caruso, Karina Zacarías, Cristian Díaz, Oscar Luna, Cristian Turchiaro, Julián Cipelli, Guillermo Thomas, Carla Medina, Natalia Labarta. Mistchenko, Alicia S.; Viegas, Mariana.
EPI_ISL_9697022, EPI_ISL_9697241	Ricardo Gutierrez Children's Hospital, Virology Laboratory, sequencing area	Ricardo Gutierrez Children's Hospital, Virology Laboratory, sequencing area	Acuña, Dolores; Goya, Stephanie; Nabaes Jodar, Mercedes S.; Grandis, Érica; María Cristina Alvarez López, María Elina Acevedo, Oscar Jacquez, Sofía Alexay, Mariángeles Barreda Fink, María Emilia Villegas, Raquel Barquez, Estela Chascón, Jorgelina Caruso, Karina Zacarías, Cristian Díaz, Oscar Luna, Cristian Turchiaro, Julián Cipelli, Guillermo Thomas, Carla Medina, Natalia Labarta. Mistchenko, Alicia S.; Viegas, Mariana.
EPI_ISL_9697599, EPI_ISL_9697975	Ricardo Gutierrez Children's Hospital, Virology Laboratory, sequencing area.	Ricardo Gutierrez Children's Hospital, Virology Laboratory, sequencing area.	Acuña, Dolores; Goya, Stephanie; Nabaes Jodar, Mercedes S.; Grandis, Érica; María Cristina Alvarez López, María Elina Acevedo, Oscar Jacquez, Sofía Alexay, Mariángeles Barreda Fink, María Emilia Villegas, Raquel Barquez, Estela Chascón, Jorgelina Caruso, Karina Zacarías, Cristian Díaz, Oscar Luna, Cristian Turchiaro, Julián Cipelli, Guillermo Thomas, Carla Medina, Natalia Labarta. Mistchenko, Alicia S.; Viegas, Mariana.
EPI_ISL_9698012	Ricardo Gutierrez Children's Hospital, Virology Laboratory, sequencing area	Ricardo Gutierrez Children's Hospital, Virology Laboratory, sequencing area	Acuña, Dolores; Goya, Stephanie; Nabaes Jodar, Mercedes S.; Grandis, Érica; María Cristina Alvarez López, María Elina Acevedo, Oscar Jacquez, Sofía Alexay, Mariángeles Barreda Fink, María Emilia Villegas, Raquel Barquez, Estela Chascón, Jorgelina Caruso, Karina Zacarías, Cristian Díaz, Oscar Luna, Cristian Turchiaro, Julián Cipelli, Guillermo Thomas, Carla Medina, Natalia Labarta. Mistchenko, Alicia S.; Viegas, Mariana
EPI_ISL_9698053, EPI_ISL_9698101	Ricardo Gutierrez Children's Hospital, Virology Laboratory, sequencing area.	Ricardo Gutierrez Children's Hospital, Virology Laboratory, sequencing area.	Acuña, Dolores; Goya, Stephanie; Nabaes Jodar, Mercedes S.; Grandis, Érica; María Cristina Alvarez López, María Elina Acevedo, Oscar Jacquez, Sofía Alexay, Mariángeles Barreda Fink, María Emilia Villegas, Raquel Barquez, Estela Chascón, Jorgelina Caruso, Karina Zacarías, Cristian Díaz, Oscar Luna, Cristian Turchiaro, Julián Cipelli, Guillermo Thomas, Carla Medina, Natalia Labarta. Mistchenko, Alicia S.; Viegas, Mariana.
EPI_ISL_9698339	Ricardo Gutierrez Children's Hospital, Virology Laboratory, sequencing area	Ricardo Gutierrez Children's Hospital, Virology Laboratory, sequencing area	Acuña, Dolores; Goya, Stephanie; Nabaes Jodar, Mercedes S.; Grandis, Érica; María Cristina Alvarez López, María Elina Acevedo, Oscar Jacquez, Sofía Alexay, Mariángeles Barreda Fink, María Emilia Villegas, Raquel Barquez, Estela Chascón, Jorgelina Caruso, Karina Zacarías, Cristian Díaz, Oscar Luna, Cristian Turchiaro, Julián Cipelli, Guillermo Thomas, Carla Medina, Natalia Labarta. Mistchenko, Alicia S.; Viegas, Mariana.
EPI_ISL_9698397, EPI_ISL_9698606	Ricardo Gutierrez Children's Hospital, Virology Laboratory, sequencing area.	Ricardo Gutierrez Children's Hospital, Virology Laboratory, sequencing area.	Acuña, Dolores; Goya, Stephanie; Nabaes Jodar, Mercedes S.; Grandis, Érica; María Cristina Alvarez López, María Elina Acevedo, Oscar Jacquez, Sofía Alexay, Mariángeles Barreda Fink, María Emilia Villegas, Raquel Barquez, Estela Chascón, Jorgelina Caruso, Karina Zacarías, Cristian Díaz, Oscar Luna, Cristian Turchiaro, Julián Cipelli, Guillermo Thomas, Carla Medina, Natalia Labarta. Mistchenko, Alicia S.; Viegas, Mariana.
EPI_ISL_9699485, EPI_ISL_9699534, EPI_ISL_9700031	Ricardo Gutierrez Children's Hospital, Virology Laboratory, sequencing area	Ricardo Gutierrez Children's Hospital, Virology Laboratory, sequencing area	Acuña, Dolores; Goya, Stephanie; Nabaes Jodar, Mercedes S.; Grandis, Érica; María Cristina Alvarez López, María Elina Acevedo, Oscar Jacquez, Sofía Alexay, Mariángeles Barreda Fink, María Emilia Villegas, Raquel Barquez, Estela Chascón, Jorgelina Caruso, Karina Zacarías, Cristian Díaz, Oscar Luna, Cristian Turchiaro, Julián Cipelli, Guillermo Thomas, Carla Medina, Natalia Labarta. Mistchenko, Alicia S.; Viegas, Mariana.
EPI_ISL_9700162	Ricardo Gutierrez Children's Hospital, Virology Laboratory, sequencing area.	Ricardo Gutierrez Children's Hospital, Virology Laboratory, sequencing area.	Acuña, Dolores; Goya, Stephanie; Nabaes Jodar, Mercedes S.; Grandis, Érica; María Cristina Alvarez López, María Elina Acevedo, Oscar Jacquez, Sofía Alexay, Mariángeles Barreda Fink, María Emilia Villegas, Raquel Barquez, Estela Chascón, Jorgelina Caruso, Karina Zacarías, Cristian Díaz, Oscar Luna, Cristian Turchiaro, Julián Cipelli, Guillermo Thomas, Carla Medina, Natalia Labarta. Mistchenko, Alicia S.; Viegas, Mariana.
EPI_ISL_9700328	Ricardo Gutierrez Children's Hospital, Virology Laboratory, sequencing area	Ricardo Gutierrez Children's Hospital, Virology Laboratory, sequencing area	Acuña, Dolores; Goya, Stephanie; Nabaes Jodar, Mercedes S.; Grandis, Érica; María Cristina Alvarez López, María Elina Acevedo, Oscar Jacquez, Sofía Alexay, Mariángeles Barreda Fink, María Emilia Villegas, Raquel Barquez, Estela Chascón, Jorgelina Caruso, Karina Zacarías, Cristian Díaz, Oscar Luna, Cristian Turchiaro, Julián Cipelli, Guillermo Thomas, Carla Medina, Natalia Labarta. Mistchenko, Alicia S.; Viegas, Mariana.
EPI_ISL_9700417, EPI_ISL_9700517, EPI_ISL_9701163, EPI_ISL_9701389, EPI_ISL_9701490, EPI_ISL_9701848, EPI_ISL_9701878, EPI_ISL_9701919, EPI_ISL_9701978, EPI_ISL_9702028, EPI_ISL_9702029, EPI_ISL_9702030			
see above	Ricardo Gutierrez Children's Hospital, Virology Laboratory, sequencing area.	Ricardo Gutierrez Children's Hospital, Virology Laboratory, sequencing area.	Acuña, Dolores; Goya, Stephanie; Nabaes Jodar, Mercedes S.; Grandis, Érica; María Cristina Alvarez López, María Elina Acevedo, Oscar Jacquez, Sofía Alexay, Mariángeles Barreda Fink, María Emilia Villegas, Raquel Barquez, Estela Chascón, Jorgelina Caruso, Karina Zacarías, Cristian Díaz, Oscar Luna, Cristian Turchiaro, Julián Cipelli, Guillermo Thomas, Carla Medina, Natalia Labarta. Mistchenko, Alicia S.; Viegas, Mariana.
EPI_ISL_9702035, EPI_ISL_9702358, EPI_ISL_9702360, EPI_ISL_9702415	Ricardo Gutierrez Children's Hospital, Virology Laboratory, sequencing area	Ricardo Gutierrez Children's Hospital, Virology Laboratory, sequencing area	Acuña, Dolores; Goya, Stephanie; Nabaes Jodar, Mercedes S.; Grandis, Érica; María Cristina Alvarez López, María Elina Acevedo, Oscar Jacquez, Sofía Alexay, Mariángeles Barreda Fink, María Emilia Villegas, Raquel Barquez, Estela Chascón, Jorgelina Caruso, Karina Zacarías, Cristian Díaz, Oscar Luna, Cristian Turchiaro, Julián Cipelli, Guillermo Thomas, Carla Medina, Natalia Labarta.

			Mistchenko, Alicia S.; Viegas, Mariana.
EPI_ISL_9702416, EPI_ISL_9702418, EPI_ISL_9702434, EPI_ISL_9702436, EPI_ISL_9702572, EPI_ISL_9702600, EPI_ISL_9702601, EPI_ISL_9702603, EPI_ISL_9702614, EPI_ISL_9702616	Ricardo Gutierrez Children's Hospital, Virology Laboratory, sequencing area.	Ricardo Gutierrez Children's Hospital, Virology Laboratory, sequencing area.	Acuña, Dolores; Goya, Stephanie; Nabaes Jodar, Mercedes S.; Grandis, Érica; María Cristina Alvarez López, María Elina Acevedo, Oscar Jacquez, Sofía Alexay, Mariángeles Barreda Fink, María Emilia Villegas, Raquel Barquez, Estela Chascón, Jorgelina Caruso, Karina Zacarías, Cristian Díaz, Oscar Luna, Cristian Turchiaro, Julián Cipelli, Guillermo Thomas, Carla Medina, Natalia Labarta. Mistchenko, Alicia S.; Viegas, Mariana.
EPI_ISL_9702617	Ricardo Gutierrez Children's Hospital, Virology Laboratory, sequencing area	Ricardo Gutierrez Children's Hospital, Virology Laboratory, sequencing area	Acuña, Dolores; Goya, Stephanie; Nabaes Jodar, Mercedes S.; Grandis, Érica; María Cristina Alvarez López, María Elina Acevedo, Oscar Jacquez, Sofía Alexay, Mariángeles Barreda Fink, María Emilia Villegas, Raquel Barquez, Estela Chascón, Jorgelina Caruso, Karina Zacarías, Cristian Díaz, Oscar Luna, Cristian Turchiaro, Julián Cipelli, Guillermo Thomas, Carla Medina, Natalia Labarta. Mistchenko, Alicia S.; Viegas, Mariana.
EPI_ISL_9702629, EPI_ISL_9702941, EPI_ISL_9702970	Ricardo Gutierrez Children's Hospital, Virology Laboratory, sequencing area.	Ricardo Gutierrez Children's Hospital, Virology Laboratory, sequencing area.	Acuña, Dolores; Goya, Stephanie; Nabaes Jodar, Mercedes S.; Grandis, Érica; María Cristina Alvarez López, María Elina Acevedo, Oscar Jacquez, Sofía Alexay, Mariángeles Barreda Fink, María Emilia Villegas, Raquel Barquez, Estela Chascón, Jorgelina Caruso, Karina Zacarías, Cristian Díaz, Oscar Luna, Cristian Turchiaro, Julián Cipelli, Guillermo Thomas, Carla Medina, Natalia Labarta. Mistchenko, Alicia S.; Viegas, Mariana.
EPI_ISL_9703005	Ricardo Gutierrez Children's Hospital, Virology Laboratory, sequencing area	Ricardo Gutierrez Children's Hospital, Virology Laboratory, sequencing area	Acuña, Dolores; Goya, Stephanie; Nabaes Jodar, Mercedes S.; Grandis, Érica; María Cristina Alvarez López, María Elina Acevedo, Oscar Jacquez, Sofía Alexay, Mariángeles Barreda Fink, María Emilia Villegas, Raquel Barquez, Estela Chascón, Jorgelina Caruso, Karina Zacarías, Cristian Díaz, Oscar Luna, Cristian Turchiaro, Julián Cipelli, Guillermo Thomas, Carla Medina, Natalia Labarta. Mistchenko, Alicia S.; Viegas, Mariana.
EPI_ISL_9703023, EPI_ISL_9703062	Ricardo Gutierrez Children's Hospital, Virology Laboratory, sequencing area.	Ricardo Gutierrez Children's Hospital, Virology Laboratory, sequencing area.	Acuña, Dolores; Goya, Stephanie; Nabaes Jodar, Mercedes S.; Grandis, Érica; María Cristina Alvarez López, María Elina Acevedo, Oscar Jacquez, Sofía Alexay, Mariángeles Barreda Fink, María Emilia Villegas, Raquel Barquez, Estela Chascón, Jorgelina Caruso, Karina Zacarías, Cristian Díaz, Oscar Luna, Cristian Turchiaro, Julián Cipelli, Guillermo Thomas, Carla Medina, Natalia Labarta. Mistchenko, Alicia S.; Viegas, Mariana.
EPI_ISL_9703110, EPI_ISL_9703111, EPI_ISL_9703513, EPI_ISL_9703515	Ricardo Gutierrez Children's Hospital, Virology Laboratory, sequencing area	Ricardo Gutierrez Children's Hospital, Virology Laboratory, sequencing area	Acuña, Dolores; Goya, Stephanie; Nabaes Jodar, Mercedes S.; Grandis, Érica; María Cristina Alvarez López, María Elina Acevedo, Oscar Jacquez, Sofía Alexay, Mariángeles Barreda Fink, María Emilia Villegas, Raquel Barquez, Estela Chascón, Jorgelina Caruso, Karina Zacarías, Cristian Díaz, Oscar Luna, Cristian Turchiaro, Julián Cipelli, Guillermo Thomas, Carla Medina, Natalia Labarta. Mistchenko, Alicia S.; Viegas, Mariana.
EPI_ISL_9703602, EPI_ISL_9703604, EPI_ISL_9703605	Ricardo Gutierrez Children's Hospital, Virology Laboratory, sequencing area.	Ricardo Gutierrez Children's Hospital, Virology Laboratory, sequencing area.	Acuña, Dolores; Goya, Stephanie; Nabaes Jodar, Mercedes S.; Grandis, Érica; María Cristina Alvarez López, María Elina Acevedo, Oscar Jacquez, Sofía Alexay, Mariángeles Barreda Fink, María Emilia Villegas, Raquel Barquez, Estela Chascón, Jorgelina Caruso, Karina Zacarías, Cristian Díaz, Oscar Luna, Cristian Turchiaro, Julián Cipelli, Guillermo Thomas, Carla Medina, Natalia Labarta. Mistchenko, Alicia S.; Viegas, Mariana.
EPI_ISL_9703793	Ricardo Gutierrez Children's Hospital, Virology Laboratory, sequencing area	Ricardo Gutierrez Children's Hospital, Virology Laboratory, sequencing area	Acuña, Dolores; Goya, Stephanie; Nabaes Jodar, Mercedes S.; Grandis, Érica; María Cristina Alvarez López, María Elina Acevedo, Oscar Jacquez, Sofía Alexay, Mariángeles Barreda Fink, María Emilia Villegas, Raquel Barquez, Estela Chascón, Jorgelina Caruso, Karina Zacarías, Cristian Díaz, Oscar Luna, Cristian Turchiaro, Julián Cipelli, Guillermo Thomas, Carla Medina, Natalia Labarta. Mistchenko, Alicia S.; Viegas, Mariana.
EPI_ISL_9703795, EPI_ISL_9703796, EPI_ISL_9703797, EPI_ISL_9703839, EPI_ISL_9703840, EPI_ISL_9703920, EPI_ISL_9704079, EPI_ISL_9704129, EPI_ISL_9705195, EPI_ISL_9705233, EPI_ISL_9705374			
see above	Ricardo Gutierrez Children's Hospital, Virology Laboratory, sequencing area.	Ricardo Gutierrez Children's Hospital, Virology Laboratory, sequencing area.	Acuña, Dolores; Goya, Stephanie; Nabaes Jodar, Mercedes S.; Grandis, Érica; María Cristina Alvarez López, María Elina Acevedo, Oscar Jacquez, Sofía Alexay, Mariángeles Barreda Fink, María Emilia Villegas, Raquel Barquez, Estela Chascón, Jorgelina Caruso, Karina Zacarías, Cristian Díaz, Oscar Luna, Cristian Turchiaro, Julián Cipelli, Guillermo Thomas, Carla Medina, Natalia Labarta. Mistchenko, Alicia S.; Viegas, Mariana.

We gratefully acknowledge the following Authors from the Originating laboratories responsible for obtaining the specimens, as well as the Submitting laboratories where the genome data were generated and shared via GISAID, on which this research is based.

All Submitters of data may be contacted directly via www.gisaid.org

Authors are sorted alphabetically.

Accession ID	Originating Laboratory	Submitting Laboratory	Authors
EPI_ISL_1074025, EPI_ISL_1074026, EPI_ISL_1074027, EPI_ISL_1074030, EPI_ISL_1074032, EPI_ISL_1074033, EPI_ISL_1074069, EPI_ISL_1074070, EPI_ISL_1074071, EPI_ISL_1074072, EPI_ISL_1074073, EPI_ISL_1074074, EPI_ISL_1074075, EPI_ISL_1074076, EPI_ISL_1074077, EPI_ISL_1074078, EPI_ISL_1074079, EPI_ISL_1074080, EPI_ISL_1074081, EPI_ISL_1074082, EPI_ISL_1074083, EPI_ISL_1074084, EPI_ISL_1074085, EPI_ISL_1074086, EPI_ISL_1074087, EPI_ISL_1074091, EPI_ISL_1074092, EPI_ISL_1074093, EPI_ISL_1074095, EPI_ISL_1074096, EPI_ISL_1074097, EPI_ISL_1074101, EPI_ISL_1074102, EPI_ISL_1074107, EPI_ISL_1074109, EPI_ISL_1074110, EPI_ISL_1074116, EPI_ISL_1074117, EPI_ISL_1074119, EPI_ISL_1074121, EPI_ISL_1074122, EPI_ISL_1074123, EPI_ISL_1074128, EPI_ISL_1074129, EPI_ISL_1074130, EPI_ISL_1074131, EPI_ISL_1074132, EPI_ISL_1074133, EPI_ISL_1074134, EPI_ISL_1074142, EPI_ISL_1074143, EPI_ISL_1074144, EPI_ISL_1074147, EPI_ISL_1074148, EPI_ISL_1074155, EPI_ISL_1074162, EPI_ISL_1074163, EPI_ISL_1074174, EPI_ISL_1074175, EPI_ISL_1074176, EPI_ISL_1074177, EPI_ISL_1074178, EPI_ISL_1074179, EPI_ISL_1074180, EPI_ISL_1074181, EPI_ISL_1074182, EPI_ISL_1074185, EPI_ISL_1074186, EPI_ISL_1074189, EPI_ISL_1074192, EPI_ISL_1074193, EPI_ISL_1074196, EPI_ISL_1074197, EPI_ISL_1074198, EPI_ISL_1074199, EPI_ISL_1074200, EPI_ISL_1074201, EPI_ISL_1074202, EPI_ISL_1074203, EPI_ISL_1074205, EPI_ISL_1074206, EPI_ISL_1074207, EPI_ISL_1074215, EPI_ISL_1074216, EPI_ISL_1074217, EPI_ISL_1074218, EPI_ISL_1074219, EPI_ISL_1074220, EPI_ISL_1074221, EPI_ISL_1074222, EPI_ISL_1074223, EPI_ISL_1074224, EPI_ISL_1074225, EPI_ISL_1074226, EPI_ISL_1074229, EPI_ISL_1074230, EPI_ISL_1074231, EPI_ISL_1074232, EPI_ISL_1074235, EPI_ISL_1074236, EPI_ISL_1074237, EPI_ISL_1074238, EPI_ISL_1074239, EPI_ISL_1074241, EPI_ISL_1074242, EPI_ISL_1074247, EPI_ISL_1074251, EPI_ISL_1074252, EPI_ISL_1074254, EPI_ISL_1074258, EPI_ISL_1074261, EPI_ISL_1074263, EPI_ISL_1074264, EPI_ISL_1074265, EPI_ISL_1074266, EPI_ISL_1074268, EPI_ISL_1074269, EPI_ISL_1074283, EPI_ISL_1074284, EPI_ISL_1074285, EPI_ISL_1074286, EPI_ISL_1074287, EPI_ISL_1074288, EPI_ISL_1074289, EPI_ISL_1074290, EPI_ISL_1074291, EPI_ISL_1074292, EPI_ISL_1074293, EPI_ISL_1074294, EPI_ISL_1074295, EPI_ISL_1074296, EPI_ISL_1074297, EPI_ISL_1074298, EPI_ISL_1074299, EPI_ISL_1074300			
see above	Virology Laboratory, Ricardo Gutiérrez Children's Hospital	Vanderbilt University Medical Center / Virology Laboratory, Ricardo Gutiérrez Children's Hospital	Goya, Stephanie; Lucion, Maria Florencia; Juarez, Maria del Valle; Shilts, Meghan; Gentile, Angela; Mistchenko, Alicia S.; Das, Suman# & Viegas, Mariana# (#contributed equally to this study)
EPI_ISL_10914986	VIC, Victorian Infectious Diseases Reference Laboratory	WHO Collaborating Centre for Reference and Research on Influenza	Xiaomin Dong, Annette Alafaci,Yi-Mo Deng, Naomi Komadina, Ammar Aziz
EPI_ISL_11428307, EPI_ISL_11428308, EPI_ISL_11428310, EPI_ISL_11428311, EPI_ISL_11428312, EPI_ISL_11428313	Respiratory Virus Unit, Reference Services, UK Health Security Agency	Reference Services, UK Health Security Agency	Zambon M, Talts T, Ellis J, Miah S, Platt S
EPI_ISL_12544916	National Influenza Center, National Institute of Hygiene and Epidemiology (NIHE)	National Institute of Hygiene and Epidemiology (NIHE) National Influenza Centre, Virology Department	Ung Thi Hong Trang, Hoang Vu Mai Phuong, Nguyen Huy Hoang, Nguyen Le Khanh Hang, Le Thi Thanh, Nguyen Vu Son, Vuong Duc Cuong, Pham Thi Hien, Tran Thu Huong, Nguyen Co Thach, Nguyen Phuong Anh, Le Quynh Mai
EPI_ISL_1437545	Hospital Perrando	INEI-ANLIS "Carlos G. Malbrán". Servicio Virosis Respiratorias.	Elsa Baumeister, Andrea Pontoriero, Russo Mara, Martin Avaro, Estefania Benedetti, Ana Campos
EPI_ISL_1437546, EPI_ISL_1437548, EPI_ISL_1437550	Hospital de la Madre y el Niño	INEI-ANLIS "Carlos G. Malbrán". Servicio Virosis Respiratorias.	Elsa Baumeister, Andrea Pontoriero, Russo Mara, Martin Avaro, Estefania Benedetti, Ana Campos
EPI_ISL_1437552, EPI_ISL_1437566, EPI_ISL_1437568, EPI_ISL_1437569, EPI_ISL_1437571	Laboratorio de Salud Publica	INEI-ANLIS "Carlos G. Malbrán". Servicio Virosis Respiratorias.	Elsa Baumeister, Andrea Pontoriero, Russo Mara, Martin Avaro, Estefania Benedetti, Ana Campos
EPI_ISL_15055329, EPI_ISL_15055330, EPI_ISL_15067694, EPI_ISL_15067695	Virology Laboratory, Ricardo Gutiérrez Children's Hospital	Virology Laboratory, Ricardo Gutiérrez Children's Hospital	Acuña, Dolores; Goya, Stephanie; Nabaes Jodar, Mercedes S.; Lucion, M. Florencia; Juárez, María del Valle; Gentile, Ángela; Mistchenko, Alicia S.; Viegas, Mariana
EPI_ISL_15120684, EPI_ISL_15120685, EPI_ISL_15120686, EPI_ISL_15120687, EPI_ISL_15120691, EPI_ISL_15120693, EPI_ISL_15120695, EPI_ISL_15120696, EPI_ISL_15120697, EPI_ISL_15120698, EPI_ISL_15120699, EPI_ISL_15120700, EPI_ISL_15120701, EPI_ISL_15120702, EPI_ISL_15120704, EPI_ISL_15120705, EPI_ISL_15120707, EPI_ISL_15120708, EPI_ISL_15120709, EPI_ISL_15120710, EPI_ISL_15120711, EPI_ISL_15120712, EPI_ISL_15120714, EPI_ISL_15120718, EPI_ISL_15120720, EPI_ISL_15120722, EPI_ISL_15120724, EPI_ISL_15120725, EPI_ISL_15120732, EPI_ISL_15120733, EPI_ISL_15120734, EPI_ISL_15120735, EPI_ISL_15120736, EPI_ISL_15120737, EPI_ISL_15120740, EPI_ISL_15120741, EPI_ISL_15120742, EPI_ISL_15120744, EPI_ISL_15120746, EPI_ISL_15120747, EPI_ISL_15120748, EPI_ISL_15120749, EPI_ISL_15120750, EPI_ISL_15120751, EPI_ISL_15120752, EPI_ISL_15120753, EPI_ISL_15120754, EPI_ISL_15120755, EPI_ISL_15120756, EPI_ISL_15120757, EPI_ISL_15120758, EPI_ISL_15120759, EPI_ISL_15120760, EPI_ISL_15120761, EPI_ISL_15120762, EPI_ISL_15120763, EPI_ISL_15120764, EPI_ISL_15120765, EPI_ISL_15120766, EPI_ISL_15120767, EPI_ISL_15120768, EPI_ISL_15120769, EPI_ISL_15120779			
see above	National Institute of Hygiene, virology departement, National Influenza Center	Centers for Disease Control and Prevention, Coronavirus and Other Respiratory Viruses Division	Megha Aggarwal, Lijuan Wang, Ji In Park, Amanda Smith and Everardo Vega
EPI_ISL_15750161, EPI_ISL_15750162, EPI_ISL_15750163, EPI_ISL_15750164, EPI_ISL_15750165, EPI_ISL_15750166	Virology, Tehran University of Medical Sciences	Virology, Tehran University of Medical Sciences	Salimi,V., Tavakoli,F., Mokhtari-Azad,T., Ghavami,N.
EPI_ISL_15750489, EPI_ISL_15750490	Department of Pediatrics, Center of Excellence in Clinical Virology	Department of Pediatrics, Center of Excellence in Clinical Virology	Thongpan,I.
EPI_ISL_15750596, EPI_ISL_15750597, EPI_ISL_15750598, EPI_ISL_15750599, EPI_ISL_15750600, EPI_ISL_15750601, EPI_ISL_15750602, EPI_ISL_15750603, EPI_ISL_15750604, EPI_ISL_15750605, EPI_ISL_15750606, EPI_ISL_15750826, EPI_ISL_15750827			
see above	Department of virology, Post Graduate Institute of Medical Education & Research	Department of virology, Post Graduate Institute of Medical Education & Research	Sarkar,S., Ratho,R.K., Singh,M.
EPI_ISL_15750833, EPI_ISL_15750834, EPI_ISL_15750835, EPI_ISL_15750836, EPI_ISL_15750837, EPI_ISL_15750838, EPI_ISL_15750839	State Key Laboratory of Pathogen and Biosecurity, Beijing Institute of Microbiology and Epidemiology	State Key Laboratory of Pathogen and Biosecurity, Beijing Institute of Microbiology and Epidemiology	Jia,N., Cao,W.-C., Hu,Y.-L., Ye,R.-Z., Que,T.-C., Xia,L.-Y., Cui,X.-M., Zhang,Y.-W., Jiang,J.-F., Wang,Q.-H., Wang,Q., Jia,N., Cao,W.-C., Hu,Y.-L., Ye,R.-Z., Que,T.-C., Xia,L.-Y., Cui,X.-M., Zhang,Y.-W., Jiang,J.-F., Wang,Q.-H., Wang,Q.
EPI_ISL_15750845, EPI_ISL_15750846, EPI_ISL_15750847, EPI_ISL_15750848, EPI_ISL_15750849, EPI_ISL_15750850, EPI_ISL_15750851, EPI_ISL_15750852, EPI_ISL_15750853, EPI_ISL_15750854, EPI_ISL_15750855, EPI_ISL_15750856, EPI_ISL_15750857, EPI_ISL_15750858, EPI_ISL_15750859, EPI_ISL_15750860, EPI_ISL_15750861, EPI_ISL_15750862, EPI_ISL_15750863, EPI_ISL_15750864, EPI_ISL_15750865, EPI_ISL_15750866, EPI_ISL_15750867, EPI_ISL_15750868, EPI_ISL_15750869, EPI_ISL_15750870, EPI_ISL_15750871, EPI_ISL_15750872, EPI_ISL_15750873, EPI_ISL_15750874, EPI_ISL_15750875, EPI_ISL_15750876, EPI_ISL_15750877, EPI_ISL_15750878, EPI_ISL_15750879, EPI_ISL_15750880, EPI_ISL_15750881, EPI_ISL_15750882, EPI_ISL_15750883, EPI_ISL_15750884, EPI_ISL_15750885, EPI_ISL_15750886, EPI_ISL_15750887, EPI_ISL_15750888, EPI_ISL_15750889, EPI_ISL_15750890, EPI_ISL_15750891, EPI_ISL_15750892, EPI_ISL_15750893, EPI_ISL_15750894, EPI_ISL_15750895, EPI_ISL_15750896, EPI_ISL_15750897, EPI_ISL_15750898, EPI_ISL_15750899, EPI_ISL_15750900			
see above	Beijing Center for Disease Prevention and Control, Immunological Prevention	Beijing Center for Disease Prevention and Control, Immunological Prevention	Qin,L., Li,M., Huang,F.
EPI_ISL_15750934, EPI_ISL_15750935, EPI_ISL_15750936, EPI_ISL_15750937, EPI_ISL_15750938, EPI_ISL_15750939, EPI_ISL_15750940	Beijing Center for Disease Prevention and Control, Immunological Prevention	Beijing Center for Disease Prevention and Control, Immunological Prevention	Luo,Q., Li,M., Huang,F.
EPI_ISL_15751117, EPI_ISL_15751118, EPI_ISL_15751119, EPI_ISL_15751120, EPI_ISL_15751121, EPI_ISL_15751122, EPI_ISL_15751123, EPI_ISL_15751124, EPI_ISL_15751125, EPI_ISL_15751126, EPI_ISL_15751127, EPI_ISL_15751128, EPI_ISL_15751129, EPI_ISL_15751130, EPI_ISL_15751131, EPI_ISL_15751132, EPI_ISL_15751133, EPI_ISL_15751134, EPI_ISL_15751135, EPI_ISL_15751136, EPI_ISL_15751137, EPI_ISL_15751138, EPI_ISL_15751139			
see above	Molecular Virology, Johns Hopkins School of Medicine	Molecular Virology, Johns Hopkins School of Medicine	Fall A, Elawar F, Hodcroft EB, Jallow MM, Toure CT, Barry MA, Kiori DE, Sy S, Diaw Y, Goudiaby D, Niang MN, Dia N, Fall,A., Jallow,M.M., Toure,C.T., Kiori,D.E., sy,S., Diaw,Y., Goudiaby,D., Niang,M.N., Dia,N.
EPI_ISL_15751218, EPI_ISL_15751219, EPI_ISL_15751220, EPI_ISL_15751224, EPI_ISL_15751225, EPI_ISL_15751227	Influenza Group, National Institute of Virology	Influenza Group, National Institute of Virology	Choudhary,M.L., Chaudhary,U., Choudhary,M.L., Chaudhary,U.
EPI_ISL_15751431, EPI_ISL_15751432, EPI_ISL_15751433, EPI_ISL_15751434, EPI_ISL_15751435, EPI_ISL_15751436, EPI_ISL_15751437, EPI_ISL_15751438, EPI_ISL_15751439, EPI_ISL_15751440, EPI_ISL_15751441			
see above	Molecular Virology, Johns Hopkins School of Medicine	Molecular Virology, Johns Hopkins School of Medicine	Fall A, Elawar F, Hodcroft EB, Jallow MM, Toure CT, Barry MA, Kiori DE, Sy S, Diaw Y, Goudiaby D, Niang MN, Dia N, Fall,A., Jallow,M.M., Toure,C.T., Kiori,D.E., sy,S., Diaw,Y., Goudiaby,D., Niang,M.N., Dia,N.
EPI_ISL_15752269, EPI_ISL_15752270, EPI_ISL_15752271, EPI_ISL_15752272, EPI_ISL_15752273, EPI_ISL_15752275, EPI_ISL_15752276, EPI_ISL_15752277, EPI_ISL_15752278, EPI_ISL_15752279, EPI_ISL_15752280, EPI_ISL_15752281, EPI_ISL_15752282, EPI_ISL_15752283, EPI_ISL_15752284, EPI_ISL_15752285, EPI_ISL_15752286, EPI_ISL_15752287, EPI_ISL_15752288, EPI_ISL_15752289, EPI_ISL_15752290, EPI_ISL_15752291, EPI_ISL_15752292, EPI_ISL_15752293, EPI_ISL_15752294, EPI_ISL_15752295, EPI_ISL_15752296, EPI_ISL_15752297, EPI_ISL_15752298, EPI_ISL_15752299, EPI_ISL_15752300, EPI_ISL_15752301, EPI_ISL_15752302, EPI_ISL_15752304, EPI_ISL_15752305, EPI_ISL_15752306, EPI_ISL_15752307, EPI_ISL_15752308, EPI_ISL_15752309, EPI_ISL_15752311, EPI_ISL_15752312, EPI_ISL_15752313, EPI_ISL_15752315, EPI_ISL_15752316, EPI_ISL_15752317, EPI_ISL_15752318, EPI_ISL_15752320, EPI_ISL_15752323, EPI_ISL_15752324, EPI_ISL_15752325, EPI_ISL_15752326, EPI_ISL_15752328, EPI_ISL_15752329, EPI_ISL_15752330, EPI_ISL_15752331, EPI_ISL_15752332, EPI_ISL_15752333, EPI_ISL_15752334, EPI_ISL_15752335, EPI_ISL_15752336, EPI_ISL_15752337, EPI_ISL_15752338, EPI_ISL_15752340, EPI_ISL_15752343, EPI_ISL_15752344, EPI_ISL_15752345, EPI_ISL_15752347, EPI_ISL_15752351, EPI_ISL_15752353, EPI_ISL_15752354, EPI_ISL_15752355, EPI_ISL_15752359, EPI_ISL_15752364, EPI_ISL_15752365, EPI_ISL_15752367, EPI_ISL_15752368, EPI_ISL_15752369, EPI_ISL_15752370, EPI_ISL_15752371, EPI_ISL_15752372, EPI_ISL_15752378, EPI_ISL_15752379, EPI_ISL_15752386, EPI_ISL_15752393, EPI_ISL_15752409, EPI_ISL_15752410, EPI_ISL_15752413, EPI_ISL_15752422, EPI_ISL_15752424, EPI_ISL_15752425, EPI_ISL_15752426, EPI_ISL_15752432, EPI_ISL_15752436, EPI_ISL_15752437, EPI_ISL_15752438, EPI_ISL_15752439, EPI_ISL_15752440, EPI_ISL_15752441, EPI_ISL_15752444, EPI_ISL_15752445, EPI_ISL_15752457, EPI_ISL_15752458, EPI_ISL_15752459, EPI_ISL_15752460, EPI_ISL_15752465, EPI_ISL_15752468, EPI_ISL_15752469, EPI_ISL_15752470, EPI_ISL_15752471, EPI_ISL_15752472, EPI_ISL_15752475, EPI_ISL_15752483, EPI_ISL_15752485, EPI_ISL_15752490, EPI_ISL_15752493, EPI_ISL_15752494, EPI_ISL_15752495, EPI_ISL_15752497, EPI_ISL_15752499, EPI_ISL_15752500, EPI_ISL_15752501, EPI_ISL_15752502, EPI_ISL_15752503, EPI_ISL_15752504, EPI_ISL_15752506, EPI_ISL_15752508, EPI_ISL_15752510, EPI_ISL_15752512, EPI_ISL_15752513, EPI_ISL_15752514, EPI_ISL_15752517, EPI_ISL_15752518, EPI_ISL_15752519, EPI_ISL_15752520, EPI_ISL_15752521, EPI_ISL_15752523, EPI_ISL_15752525, EPI_ISL_15752526, EPI_ISL_15752527, EPI_ISL_15752528, EPI_ISL_15752529, EPI_ISL_15752530, EPI_ISL_15752532, EPI_ISL_15752536, EPI_ISL_15752538, EPI_ISL_15752540, EPI_ISL_15752541, EPI_ISL_15752543, EPI_ISL_15752544, EPI_ISL_15752545, EPI_ISL_15752546, EPI_ISL_15752548, EPI_ISL_15752549			
see above	Microbial Sciences, AstraZeneca	Microbial Sciences, AstraZeneca	Tabor,D.E., Fernandes,F., Langedijk,A.C., Wilkins,D., Lebbink,R.J., Tovchigrechko,A., Ruzin,A., Kragten-Tabatabaie,L., Jin,H., Esser,M.T., Bont,L.J., Abram,M.E., Tabor,D.
EPI_ISL_15752550, EPI_ISL_15752552, EPI_ISL_15752553, EPI_ISL_15752554, EPI_ISL_15752555, EPI_ISL_15752556, EPI_ISL_15752557, EPI_ISL_15752558, EPI_ISL_15752559, EPI_ISL_15752561, EPI_ISL_15752562, EPI_ISL_15752563, EPI_ISL_15752564, EPI_ISL_15752565, EPI_ISL_15752566, EPI_ISL_15752567, EPI_ISL_15752568, EPI_ISL_15752569, EPI_ISL_15752570, EPI_ISL_15752572, EPI_ISL_15752573, EPI_ISL_15752574, EPI_ISL_15752576, EPI_ISL_15752577, EPI_ISL_15752585, EPI_ISL_15752588, EPI_ISL_15752589, EPI_ISL_15752591, EPI_ISL_15752592, EPI_ISL_15752595, EPI_ISL_15752596, EPI_ISL_15752597, EPI_ISL_15752598, EPI_ISL_15752599, EPI_ISL_15752600, EPI_ISL_15752602, EPI_ISL_15752605, EPI_ISL_15752608, EPI_ISL_15752610, EPI_ISL_15752659, EPI_ISL_15752661, EPI_ISL_15752663, EPI_ISL_15752680, EPI_ISL_15752684, EPI_ISL_15752686, EPI_ISL_15752687, EPI_ISL_15752688, EPI_ISL_15752689, EPI_ISL_15752690, EPI_ISL_15752692, EPI_ISL_15752693, EPI_ISL_15752694, EPI_ISL_15752695, EPI_ISL_15752696, EPI_ISL_15752697, EPI_ISL_15752698, EPI_ISL_15752699, EPI_ISL_15752700, EPI_ISL_15752701, EPI_ISL_15752702, EPI_ISL_15752704, EPI_ISL_15752705, EPI_ISL_15752706, EPI_ISL_15752708, EPI_ISL_15752713, EPI_ISL_15752715, EPI_ISL_15752716, EPI_ISL_15752717, EPI_ISL_15752718			
see above	Pneumococcal Research Group, Infections and Immunity	Pneumococcal Research Group, Infections and Immunity	Anderson,J., Oeum,M., Verkolf,E., Licciardi,P.V., Mulholland,K., Nguyen,C., Chow,K., Waller,G., Costa,A.M., Daley,A., Crawford,N.W., Babl,F.E., Duke,T., Do,L.A.H., Wurzel,D., Anderson,J., Oeum,M., Verkolf,E., Licciardi,P.V., Mulholland,K.E., Nguyen,C., Chow,K., Waller,G., Costa,A.-M., Daley,A.J., Crawford,N., Babl,F., Duke,T., Do,L.A.H., Wurzel,D.
EPI_ISL_15752870	Epidemiology and Demography, KEMRI-Wellcome Trust Research Programme	Epidemiology and Demography, KEMRI-Wellcome Trust Research Programme	Kamau,E., Mutunga,M., Mwema,A., Agoti,C.N., Nokes,D.J., Kamau,E., Mutunga,M., Mwema,A., Agoti,C.N., Nokes,D.J.
EPI_ISL_15752978, EPI_ISL_15752984, EPI_ISL_15753002, EPI_ISL_15753003, EPI_ISL_15753006, EPI_ISL_15753010, EPI_ISL_15753013, EPI_ISL_15753018, EPI_ISL_15753020, EPI_ISL_15753024, EPI_ISL_15753025, EPI_ISL_15753027, EPI_ISL_15753032, EPI_ISL_15753037, EPI_ISL_15753038, EPI_ISL_15753039, EPI_ISL_15753046, EPI_ISL_15753053, EPI_ISL_15753055, EPI_ISL_15753067, EPI_ISL_15753071, EPI_ISL_15753082, EPI_ISL_15753085, EPI_ISL_15753089, EPI_ISL_15753090, EPI_ISL_15753091, EPI_ISL_15753096, EPI_ISL_15753100, EPI_ISL_15753106, EPI_ISL_15753114, EPI_ISL_15753118, EPI_ISL_15753121, EPI_ISL_15753125, EPI_ISL_15753126, EPI_ISL_15753128, EPI_ISL_15753138, EPI_ISL_15753154, EPI_ISL_15753156, EPI_ISL_15753159, EPI_ISL_15753162, EPI_ISL_15753163, EPI_ISL_15753174, EPI_ISL_15753177, EPI_ISL_15753180, EPI_ISL_15753187, EPI_ISL_15753190, EPI_ISL_15753194, EPI_ISL_15753197, EPI_ISL_15753199, EPI_ISL_15753206, EPI_ISL_15753207, EPI_ISL_15753215, EPI_ISL_15753220, EPI_ISL_15753221, EPI_ISL_15753226, EPI_ISL_15753229, EPI_ISL_15753236, EPI_ISL_15753240, EPI_ISL_15753242, EPI_ISL_15753244, EPI_ISL_15753249, EPI_ISL_15753251			
see above	Department of Paediatrics, University of Oxford	Department of Paediatrics, University of Oxford	Lin,G.-L., Drysdale,S.B., Snape,M.D., O'Connor,D., Brown,A., MacIntyre-Cockett,G., Mellado-Gomez,E., de Cesare,M., Bonsall,D., Ansari,M.A., Oner,D., Aerssens,J., Butler,C., Bont,L., Openshaw,P., Martinon-Torres,F., Nair,H., Bowden,R., Golubchik,T., Pollard,A.J., Lin,G.-L.
EPI_ISL_15772196	Laboratory Medicine, UW Virology	Laboratory Medicine, UW Virology	Sereewit,J., Xie,H. and Greninger,A.
EPI_ISL_15820226, EPI_ISL_15820227, EPI_ISL_15820228, EPI_ISL_15820229, EPI_ISL_15820230, EPI_ISL_15820231, EPI_ISL_15820232	University of Ghana, Medical Laboratory Sciences	University of Ghana, Medical Laboratory Sciences	Kafintu-Kwashie,A.A., Nii-Trebi,N.I., Obodai,E., Neizer,M., Adiku,T.K., Odoom,J.K., Nii-Trebi,N.I., Kafintu-Kwashie,A.A., Obodai,E., Neizer,M., Adiku,T.K., Odoom,J.K.
EPI_ISL_15820256	Beijing Center for Disease Prevention and Control, Immunological Prevention	Beijing Center for Disease Prevention and Control, Immunological Prevention	Qin,L., Li,M., Huang,F.
EPI_ISL_16051901, EPI_ISL_16051902, EPI_ISL_16051903, EPI_ISL_16051904, EPI_ISL_16051905, EPI_ISL_16051906, EPI_ISL_16051907, EPI_ISL_16051908, EPI_ISL_16051909, EPI_ISL_16051910, EPI_ISL_16051911, EPI_ISL_16051912, EPI_ISL_16051913, EPI_ISL_16051914, EPI_ISL_16051915, EPI_ISL_16051916, EPI_ISL_16051917, EPI_ISL_16051929, EPI_ISL_16051930, EPI_ISL_16051931, EPI_ISL_16051932, EPI_ISL_16051933, EPI_ISL_16051934			
see above	National Influenza Center, National Institute of Hygiene and Epidemiology (NIHE)	National Influenza Center, National Institute of Hygiene and Epidemiology (NIHE)	Ung Thi Hong Trang, Hoang Vu Mai Phuong, Nguyen Huy Hoang, Nguyen Le Khanh Hang, Le Thi Quynh Mai
EPI_ISL_1647470, EPI_ISL_1647471, EPI_ISL_1647472, EPI_ISL_1647473, EPI_ISL_1647474, EPI_ISL_1647475, EPI_ISL_1647476, EPI_ISL_1647477, EPI_ISL_1647478, EPI_ISL_1647479, EPI_ISL_1647480, EPI_ISL_1647481, EPI_ISL_1647482, EPI_ISL_1647483, EPI_ISL_1647484, EPI_ISL_1647485, EPI_ISL_1647486, EPI_ISL_1647487, EPI_ISL_1647489			

see above | Instituto Nacional de Saúde | WHO Influenza Centre for Reference and Research on Influenza | Angela Todd, Yi-Mo Deng, Almiro Rogerio Tivane, Naomi Komadina

EPI_ISL_16905446, EPI_ISL_16905449, EPI_ISL_16905450 | National Institute of Hygiene, virology departement, National Influenza Center | Centers for Disease Control and Prevention, Coronavirus and Other Respiratory Viruses Division | Megha Aggarwal, Lijuan Wang, Ji In Park, Amanda Smith and Everardo Vega

EPI_ISL_17168012, EPI_ISL_17168013, EPI_ISL_17168014, EPI_ISL_17168015, EPI_ISL_17168016, EPI_ISL_17168017, EPI_ISL_17168018, EPI_ISL_17168019, EPI_ISL_17168020, EPI_ISL_17168021, EPI_ISL_17168022, EPI_ISL_17168242, EPI_ISL_17168243, EPI_ISL_17168244, EPI_ISL_17168245, EPI_ISL_17168247, EPI_ISL_17168248, EPI_ISL_17168249, EPI_ISL_17168257

see above | National Institute for Viral Disease Control and Prevention | National Institute for Viral Disease Control and Prevention | Song,J., Zhu,Z., Song,J., Mao,N., Cui,A., Xu,W. and Zhang,Y.

EPI_ISL_17215836, EPI_ISL_17215838, EPI_ISL_17215839, EPI_ISL_17215841, EPI_ISL_17215843, EPI_ISL_17215853, EPI_ISL_17215863, EPI_ISL_17215865, EPI_ISL_17215868, EPI_ISL_17215869, EPI_ISL_17215876, EPI_ISL_17215880, EPI_ISL_17215882, EPI_ISL_17215890, EPI_ISL_17215903, EPI_ISL_17215910, EPI_ISL_17215913, EPI_ISL_17215919, EPI_ISL_17215922, EPI_ISL_17215923,
EPI_ISL_17215924, EPI_ISL_17215925, EPI_ISL_17215931, EPI_ISL_17215932, EPI_ISL_17215935, EPI_ISL_17215939, EPI_ISL_17215941, EPI_ISL_17215947, EPI_ISL_17215948, EPI_ISL_17215949, EPI_ISL_17215955, EPI_ISL_17215959, EPI_ISL_17215960, EPI_ISL_17215962, EPI_ISL_17215968, EPI_ISL_17215969, EPI_ISL_17215971,
EPI_ISL_17215975, EPI_ISL_17215979, EPI_ISL_17215980, EPI_ISL_17215987, EPI_ISL_17215988, EPI_ISL_17215994, EPI_ISL_17215995, EPI_ISL_17215996, EPI_ISL_17215997, EPI_ISL_17216003, EPI_ISL_17216011, EPI_ISL_17216014, EPI_ISL_17216016, EPI_ISL_17216018, EPI_ISL_17216021, EPI_ISL_17216028, EPI_ISL_17216030, EPI_ISL_17216039, EPI_ISL_17216040, EPI_ISL_17216042,
EPI_ISL_17216053, EPI_ISL_17216054, EPI_ISL_17216055, EPI_ISL_17216060, EPI_ISL_17216061, EPI_ISL_17216062, EPI_ISL_17216066, EPI_ISL_17216067, EPI_ISL_17216068, EPI_ISL_17216069, EPI_ISL_17216075, EPI_ISL_17216079, EPI_ISL_17216080, EPI_ISL_17216086, EPI_ISL_17216096, EPI_ISL_17216097, EPI_ISL_17216100, EPI_ISL_17216117, EPI_ISL_17216126,
EPI_ISL_17216134, EPI_ISL_17216141, EPI_ISL_17216152, EPI_ISL_17216156, EPI_ISL_17216159, EPI_ISL_17216169, EPI_ISL_17216171, EPI_ISL_17216181, EPI_ISL_17216194, EPI_ISL_17216195, EPI_ISL_17216196, EPI_ISL_17216197, EPI_ISL_17216201, EPI_ISL_17216208, EPI_ISL_17216211, EPI_ISL_17216218, EPI_ISL_17216222, EPI_ISL_17216223, EPI_ISL_17216224, EPI_ISL_17216231,
EPI_ISL_17216234, EPI_ISL_17216235, EPI_ISL_17216237, EPI_ISL_17216240, EPI_ISL_17216244, EPI_ISL_17216254, EPI_ISL_17216259, EPI_ISL_17216261, EPI_ISL_17216262, EPI_ISL_17216268, EPI_ISL_17216270, EPI_ISL_17216272, EPI_ISL_17216275, EPI_ISL_17216276, EPI_ISL_17216279, EPI_ISL_17216282, EPI_ISL_17216285, EPI_ISL_17216287, EPI_ISL_17216290, EPI_ISL_17216291,
EPI_ISL_17216295, EPI_ISL_17216299, EPI_ISL_17216303, EPI_ISL_17216305, EPI_ISL_17216306, EPI_ISL_17216311, EPI_ISL_17216319, EPI_ISL_17216330, EPI_ISL_17216331, EPI_ISL_17216335, EPI_ISL_17216339, EPI_ISL_17216340, EPI_ISL_17216344, EPI_ISL_17216348, EPI_ISL_17216350, EPI_ISL_17216354, EPI_ISL_17216355, EPI_ISL_17216360, EPI_ISL_17216361, EPI_ISL_17216362,
EPI_ISL_17216368, EPI_ISL_17216370, EPI_ISL_17216373, EPI_ISL_17216381, EPI_ISL_17216384, EPI_ISL_17216385, EPI_ISL_17216387, EPI_ISL_17216393, EPI_ISL_17216394, EPI_ISL_17216403, EPI_ISL_17216407, EPI_ISL_17216412, EPI_ISL_17216416, EPI_ISL_17216421, EPI_ISL_17216423, EPI_ISL_17216424, EPI_ISL_17216428, EPI_ISL_17216433, EPI_ISL_17216434,
EPI_ISL_17216441, EPI_ISL_17216442, EPI_ISL_17216443, EPI_ISL_17216447, EPI_ISL_17216448, EPI_ISL_17216457, EPI_ISL_17216458, EPI_ISL_17216460, EPI_ISL_17216466, EPI_ISL_17216498, EPI_ISL_17216500, EPI_ISL_17216511, EPI_ISL_17216521, EPI_ISL_17216523, EPI_ISL_17216529, EPI_ISL_17216550, EPI_ISL_17216551, EPI_ISL_17216562, EPI_ISL_17216564, EPI_ISL_17216568,
EPI_ISL_17216575, EPI_ISL_17216582, EPI_ISL_17216611, EPI_ISL_17216646, EPI_ISL_17216649, EPI_ISL_17216656, EPI_ISL_17216658, EPI_ISL_17216660, EPI_ISL_17216661, EPI_ISL_17216666, EPI_ISL_17216673, EPI_ISL_17216674, EPI_ISL_17216678, EPI_ISL_17216679, EPI_ISL_17216686, EPI_ISL_17216688, EPI_ISL_17216694, EPI_ISL_17216695, EPI_ISL_17216697, EPI_ISL_17216699,
EPI_ISL_17216701, EPI_ISL_17216704, EPI_ISL_17216709, EPI_ISL_17216713, EPI_ISL_17216716, EPI_ISL_17216732, EPI_ISL_17216733, EPI_ISL_17216734, EPI_ISL_17216738, EPI_ISL_17216739, EPI_ISL_17216740, EPI_ISL_17216752, EPI_ISL_17216753, EPI_ISL_17216754, EPI_ISL_17216760, EPI_ISL_17216763, EPI_ISL_17216764, EPI_ISL_17216765, EPI_ISL_17216768, EPI_ISL_17216769,
EPI_ISL_17216771, EPI_ISL_17216774, EPI_ISL_17216776, EPI_ISL_17216782, EPI_ISL_17216783, EPI_ISL_17216793, EPI_ISL_17216794, EPI_ISL_17216799, EPI_ISL_17216800, EPI_ISL_17216801, EPI_ISL_17216807, EPI_ISL_17216808, EPI_ISL_17216810, EPI_ISL_17216813, EPI_ISL_17216814, EPI_ISL_17216815, EPI_ISL_17216817, EPI_ISL_17216818, EPI_ISL_17216819, EPI_ISL_17216820,
EPI_ISL_17216826, EPI_ISL_17216827, EPI_ISL_17216828, EPI_ISL_17216830, EPI_ISL_17216842, EPI_ISL_17216849, EPI_ISL_17216851, EPI_ISL_17216857, EPI_ISL_17216859, EPI_ISL_17216869, EPI_ISL_17216872, EPI_ISL_17216878, EPI_ISL_17216879, EPI_ISL_17216880, EPI_ISL_17216882, EPI_ISL_17216884, EPI_ISL_17216890, EPI_ISL_17216891, EPI_ISL_17216892, EPI_ISL_17216896,
EPI_ISL_17216899, EPI_ISL_17216906, EPI_ISL_17216908, EPI_ISL_17216910, EPI_ISL_17216911, EPI_ISL_17216913, EPI_ISL_17216917, EPI_ISL_17216921, EPI_ISL_17216927, EPI_ISL_17216930, EPI_ISL_17216935, EPI_ISL_17216937, EPI_ISL_17216949, EPI_ISL_17216950, EPI_ISL_17216954, EPI_ISL_17216963, EPI_ISL_17216969, EPI_ISL_17216972, EPI_ISL_17216977, EPI_ISL_17216997,
EPI_ISL_17217001, EPI_ISL_17217021, EPI_ISL_17217022, EPI_ISL_17217023, EPI_ISL_17217030, EPI_ISL_17217031, EPI_ISL_17217032, EPI_ISL_17217037, EPI_ISL_17217038, EPI_ISL_17217040, EPI_ISL_17217041, EPI_ISL_17217044, EPI_ISL_17217045, EPI_ISL_17217048, EPI_ISL_17217049, EPI_ISL_17217052, EPI_ISL_17217056, EPI_ISL_17217059, EPI_ISL_17217068, EPI_ISL_17217071,
EPI_ISL_17217072, EPI_ISL_17217075, EPI_ISL_17217077, EPI_ISL_17217078, EPI_ISL_17217079, EPI_ISL_17217080, EPI_ISL_17217081, EPI_ISL_17217084, EPI_ISL_17217086, EPI_ISL_17217089, EPI_ISL_17217092, EPI_ISL_17217104, EPI_ISL_17217105, EPI_ISL_17217109, EPI_ISL_17217111, EPI_ISL_17217112, EPI_ISL_17217119, EPI_ISL_17217123, EPI_ISL_17217124, EPI_ISL_17217125,
EPI_ISL_17217132, EPI_ISL_17217136, EPI_ISL_17217139, EPI_ISL_17217140, EPI_ISL_17217141, EPI_ISL_17217142, EPI_ISL_17217143, EPI_ISL_17217145, EPI_ISL_17217149, EPI_ISL_17217150, EPI_ISL_17217151, EPI_ISL_17217152, EPI_ISL_17217156, EPI_ISL_17217159, EPI_ISL_17217160, EPI_ISL_17217163, EPI_ISL_17217169, EPI_ISL_17217170, EPI_ISL_17217176, EPI_ISL_17217177,
EPI_ISL_17217179, EPI_ISL_17217180, EPI_ISL_17217182, EPI_ISL_17217192, EPI_ISL_17217194, EPI_ISL_17217203, EPI_ISL_17217204, EPI_ISL_17217208, EPI_ISL_17217217, EPI_ISL_17217226, EPI_ISL_17217234, EPI_ISL_17217236, EPI_ISL_17217238, EPI_ISL_17217242, EPI_ISL_17217253, EPI_ISL_17217257, EPI_ISL_17217260, EPI_ISL_17217267, EPI_ISL_17217281, EPI_ISL_17217286,
EPI_ISL_17217287, EPI_ISL_17217290, EPI_ISL_17217295, EPI_ISL_17217297, EPI_ISL_17217305, EPI_ISL_17217306, EPI_ISL_17217307, EPI_ISL_17217313, EPI_ISL_17217314, EPI_ISL_17217316, EPI_ISL_17217318, EPI_ISL_17217320, EPI_ISL_17217333, EPI_ISL_17217345, EPI_ISL_17217353, EPI_ISL_17217354, EPI_ISL_17217366, EPI_ISL_17217372, EPI_ISL_17217374, EPI_ISL_17217377,
EPI_ISL_17217381, EPI_ISL_17217384, EPI_ISL_17217385, EPI_ISL_17217390, EPI_ISL_17217394, EPI_ISL_17217397, EPI_ISL_17217400, EPI_ISL_17217401, EPI_ISL_17217405, EPI_ISL_17217409, EPI_ISL_17217411, EPI_ISL_17217412, EPI_ISL_17217420, EPI_ISL_17217422, EPI_ISL_17217425, EPI_ISL_17217428, EPI_ISL_17217434, EPI_ISL_17217439, EPI_ISL_17217440, EPI_ISL_17217445,
EPI_ISL_17217447, EPI_ISL_17217449, EPI_ISL_17217456, EPI_ISL_17217460, EPI_ISL_17217463, EPI_ISL_17217466, EPI_ISL_17217470, EPI_ISL_17217472, EPI_ISL_17217473, EPI_ISL_17217474, EPI_ISL_17217482, EPI_ISL_17217483, EPI_ISL_17217487, EPI_ISL_17217489, EPI_ISL_17217490, EPI_ISL_17217494, EPI_ISL_17217496, EPI_ISL_17217497, EPI_ISL_17217504, EPI_ISL_17217505,
EPI_ISL_17217511, EPI_ISL_17217516, EPI_ISL_17217521, EPI_ISL_17217524, EPI_ISL_17217527, EPI_ISL_17217534, EPI_ISL_17217536, EPI_ISL_17217537, EPI_ISL_17217538, EPI_ISL_17217558, EPI_ISL_17217573, EPI_ISL_17217590, EPI_ISL_17217591, EPI_ISL_17217594, EPI_ISL_17217604, EPI_ISL_17217607, EPI_ISL_17217621, EPI_ISL_17217622, EPI_ISL_17217629, EPI_ISL_17217631,
EPI_ISL_17217633, EPI_ISL_17217638, EPI_ISL_17217648, EPI_ISL_17217650, EPI_ISL_17217651, EPI_ISL_17217653, EPI_ISL_17217656, EPI_ISL_17217657, EPI_ISL_17217660, EPI_ISL_17217663, EPI_ISL_17217706, EPI_ISL_17217707, EPI_ISL_17217711, EPI_ISL_17217714, EPI_ISL_17217715, EPI_ISL_17217722, EPI_ISL_17217725, EPI_ISL_17217731, EPI_ISL_17217734, EPI_ISL_17217737,
EPI_ISL_17217740, EPI_ISL_17217743, EPI_ISL_17217749, EPI_ISL_17217752, EPI_ISL_17217757, EPI_ISL_17217758, EPI_ISL_17217761, EPI_ISL_17217765, EPI_ISL_17217770, EPI_ISL_17217771, EPI_ISL_17217772, EPI_ISL_17217778, EPI_ISL_17217781, EPI_ISL_17217784, EPI_ISL_17217785, EPI_ISL_17217789, EPI_ISL_17217791, EPI_ISL_17217793, EPI_ISL_17217794, EPI_ISL_17217797,
EPI_ISL_17217798, EPI_ISL_17217803, EPI_ISL_17217806, EPI_ISL_17217810, EPI_ISL_17217813, EPI_ISL_17217816, EPI_ISL_17217817, EPI_ISL_17217821, EPI_ISL_17217824, EPI_ISL_17217831, EPI_ISL_17217835, EPI_ISL_17217837, EPI_ISL_17217840, EPI_ISL_17217843, EPI_ISL_17217846, EPI_ISL_17217849, EPI_ISL_17217851, EPI_ISL_17217854, EPI_ISL_17217860, EPI_ISL_17217862,
EPI_ISL_17217863, EPI_ISL_17217866, EPI_ISL_17217867, EPI_ISL_17217874, EPI_ISL_17217883, EPI_ISL_17217898, EPI_ISL_17217910, EPI_ISL_17217919, EPI_ISL_17217923, EPI_ISL_17217930, EPI_ISL_17217931, EPI_ISL_17217932, EPI_ISL_17217936, EPI_ISL_17217944, EPI_ISL_17217952, EPI_ISL_17217953, EPI_ISL_17217958, EPI_ISL_17217962, EPI_ISL_17217963,
EPI_ISL_17217965, EPI_ISL_17217966, EPI_ISL_17217970, EPI_ISL_17217984, EPI_ISL_17217987, EPI_ISL_17217988, EPI_ISL_17217993, EPI_ISL_17217997, EPI_ISL_17218001, EPI_ISL_17218002, EPI_ISL_17218009, EPI_ISL_17218010, EPI_ISL_17218014, EPI_ISL_17218021, EPI_ISL_17218024, EPI_ISL_17218025, EPI_ISL_17218026, EPI_ISL_17218030, EPI_ISL_17218031, EPI_ISL_17218033,
EPI_ISL_17218036, EPI_ISL_17218040, EPI_ISL_17218042, EPI_ISL_17218043, EPI_ISL_17218046, EPI_ISL_17218051, EPI_ISL_17218056, EPI_ISL_17218057, EPI_ISL_17218060, EPI_ISL_17218062, EPI_ISL_17218068, EPI_ISL_17218069, EPI_ISL_17218073, EPI_ISL_17218074, EPI_ISL_17218079, EPI_ISL_17218082, EPI_ISL_17218085, EPI_ISL_17218094, EPI_ISL_17218095, EPI_ISL_17218098,
EPI_ISL_17218099, EPI_ISL_17218102, EPI_ISL_17218108, EPI_ISL_17218111, EPI_ISL_17218112, EPI_ISL_17218115, EPI_ISL_17218120, EPI_ISL_17218124, EPI_ISL_17218126, EPI_ISL_17218132, EPI_ISL_17218136, EPI_ISL_17218139, EPI_ISL_17218141, EPI_ISL_17218153, EPI_ISL_17218155, EPI_ISL_17218161, EPI_ISL_17218164, EPI_ISL_17218165, EPI_ISL_17218170, EPI_ISL_17218175,
EPI_ISL_17218177, EPI_ISL_17218178, EPI_ISL_17218181, EPI_ISL_17218186, EPI_ISL_17218188, EPI_ISL_17218189, EPI_ISL_17218193, EPI_ISL_17218196, EPI_ISL_17218200, EPI_ISL_17218201, EPI_ISL_17218204, EPI_ISL_17218247, EPI_ISL_17218271, EPI_ISL_17218283, EPI_ISL_17218292, EPI_ISL_17218294, EPI_ISL_17218296, EPI_ISL_17218298, EPI_ISL_17218299,
EPI_ISL_17218302, EPI_ISL_17218304, EPI_ISL_17218306, EPI_ISL_17218307, EPI_ISL_17218313, EPI_ISL_17218314, EPI_ISL_17218318, EPI_ISL_17218322, EPI_ISL_17218329, EPI_ISL_17218332, EPI_ISL_17218335, EPI_ISL_17218348, EPI_ISL_17218369, EPI_ISL_17218377, EPI_ISL_17218379, EPI_ISL_17218383, EPI_ISL_17218392, EPI_ISL_17218394, EPI_ISL_17218397, EPI_ISL_17218404,
EPI_ISL_17218415, EPI_ISL_17218416, EPI_ISL_17218420, EPI_ISL_17218422, EPI_ISL_17218426, EPI_ISL_17218427, EPI_ISL_17218432, EPI_ISL_17218436, EPI_ISL_17218441, EPI_ISL_17218446, EPI_ISL_17218450, EPI_ISL_17218454, EPI_ISL_17218455, EPI_ISL_17218467, EPI_ISL_17218472, EPI_ISL_17218473, EPI_ISL_17218475, EPI_ISL_17218492, EPI_ISL_17218495, EPI_ISL_17218500,
EPI_ISL_17218501, EPI_ISL_17218503, EPI_ISL_17218508, EPI_ISL_17218517, EPI_ISL_17218529, EPI_ISL_17218530, EPI_ISL_17218536, EPI_ISL_17218539, EPI_ISL_17218542, EPI_ISL_17218543, EPI_ISL_17218546, EPI_ISL_17218552, EPI_ISL_17218553, EPI_ISL_17218558, EPI_ISL_17218559, EPI_ISL_17218567, EPI_ISL_17218569, EPI_ISL_17218583, EPI_ISL_17218586, EPI_ISL_17218596,
EPI_ISL_17218601, EPI_ISL_17218608, EPI_ISL_17218615, EPI_ISL_17218618, EPI_ISL_17218621, EPI_ISL_17218623, EPI_ISL_17218628, EPI_ISL_17218631, EPI_ISL_17218636, EPI_ISL_17218655, EPI_ISL_17218670, EPI_ISL_17218681, EPI_ISL_17218686, EPI_ISL_17218716, EPI_ISL_17218731, EPI_ISL_17218787, EPI_ISL_17218815, EPI_ISL_17218824, EPI_ISL_17218825, EPI_ISL_17218831,
EPI_ISL_17218833, EPI_ISL_17218837, EPI_ISL_17218868, EPI_ISL_17218909, EPI_ISL_17218920, EPI_ISL_17218922, EPI_ISL_17218935, EPI_ISL_17218938, EPI_ISL_17218941, EPI_ISL_17218942, EPI_ISL_17218946, EPI_ISL_17218955, EPI_ISL_17218962, EPI_ISL_17218967, EPI_ISL_17218975, EPI_ISL_17218984, EPI_ISL_17218986, EPI_ISL_17218989, EPI_ISL_17218990, EPI_ISL_17218997,
EPI_ISL_17218999, EPI_ISL_17219007, EPI_ISL_17219008, EPI_ISL_17219011, EPI_ISL_17219018, EPI_ISL_17219027, EPI_ISL_17219036, EPI_ISL_17219040, EPI_ISL_17219043, EPI_ISL_17219048, EPI_ISL_17219055, EPI_ISL_17219073, EPI_ISL_17219074, EPI_ISL_17219086, EPI_ISL_17219087, EPI_ISL_17219096, EPI_ISL_17219099, EPI_ISL_17219108, EPI_ISL_17219109, EPI_ISL_17219111,
EPI_ISL_17219117, EPI_ISL_17219121, EPI_ISL_17219122, EPI_ISL_17219124, EPI_ISL_17219139, EPI_ISL_17219140, EPI_ISL_17219142, EPI_ISL_17219145, EPI_ISL_17219147, EPI_ISL_17219148, EPI_ISL_17219158, EPI_ISL_17219164, EPI_ISL_17219165, EPI_ISL_17219166, EPI_ISL_17219169, EPI_ISL_17219176, EPI_ISL_17219193, EPI_ISL_17219196, EPI_ISL_17219197, EPI_ISL_17219202,
EPI_ISL_17219203, EPI_ISL_17219210, EPI_ISL_17219211, EPI_ISL_17219213, EPI_ISL_17219217, EPI_ISL_17219218, EPI_ISL_17219225, EPI_ISL_17219226, EPI_ISL_17219227, EPI_ISL_17219230, EPI_ISL_17219235, EPI_ISL_17219239, EPI_ISL_17219245, EPI_ISL_17219268, EPI_ISL_17219312, EPI_ISL_17219324, EPI_ISL_17219326, EPI_ISL_17219330, EPI_ISL_17219334, EPI_ISL_17219335,
EPI_ISL_17219338, EPI_ISL_17219343, EPI_ISL_17219352, EPI_ISL_17219354, EPI_ISL_17219360, EPI_ISL_17219363, EPI_ISL_17219364, EPI_ISL_17219373, EPI_ISL_17219374, EPI_ISL_17219377, EPI_ISL_17219380, EPI_ISL_17219388, EPI_ISL_17219391, EPI_ISL_17219392, EPI_ISL_17219399, EPI_ISL_17219401, EPI_ISL_17219407, EPI_ISL_17219409, EPI_ISL_17219411, EPI_ISL_17219413,
EPI_ISL_17219431, EPI_ISL_17219432, EPI_ISL_17219434, EPI_ISL_17219438, EPI_ISL_17219439, EPI_ISL_17219448, EPI_ISL_17219451, EPI_ISL_17219452, EPI_ISL_17219464, EPI_ISL_17219465, EPI_ISL_17219472, EPI_ISL_17219477, EPI_ISL_17219478, EPI_ISL_17219488, EPI_ISL_17219490, EPI_ISL_17219496, EPI_ISL_17219498, EPI_ISL_17219500, EPI_ISL_17219504,
EPI_ISL_17219505, EPI_ISL_17219509, EPI_ISL_17219510, EPI_ISL_17219514, EPI_ISL_17219516, EPI_ISL_17219518, EPI_ISL_17219520, EPI_ISL_17219525, EPI_ISL_17219532, EPI_ISL_17219534, EPI_ISL_17219535, EPI_ISL_17219542, EPI_ISL_17219561, EPI_ISL_17219564, EPI_ISL_17219568, EPI_ISL_17219571, EPI_ISL_17219572, EPI_ISL_17219578, EPI_ISL_17219581, EPI_ISL_17219585,
EPI_ISL_17219588, EPI_ISL_17219595, EPI_ISL_17219597, EPI_ISL_17219603, EPI_ISL_17219607, EPI_ISL_17219609, EPI_ISL_17219617, EPI_ISL_17219619, EPI_ISL_17219625, EPI_ISL_17219639, EPI_ISL_17219648, EPI_ISL_17219652, EPI_ISL_17219653, EPI_ISL_17219665, EPI_ISL_17219688, EPI_ISL_17219689, EPI_ISL_17219699, EPI_ISL_17219705, EPI_ISL_17219709, EPI_ISL_17219711,
EPI_ISL_17219719, EPI_ISL_17219724, EPI_ISL_17219729, EPI_ISL_17219733, EPI_ISL_17219734, EPI_ISL_17219737, EPI_ISL_17219738, EPI_ISL_17219745, EPI_ISL_17219750, EPI_ISL_17219753, EPI_ISL_17219756, EPI_ISL_17219760, EPI_ISL_17219761, EPI_ISL_17219764, EPI_ISL_17219765, EPI_ISL_17219766, EPI_ISL_17219769, EPI_ISL_17219770, EPI_ISL_17219771, EPI_ISL_17219781,
EPI_ISL_17219790, EPI_ISL_17219797, EPI_ISL_17219798, EPI_ISL_17219801, EPI_ISL_17219806, EPI_ISL_17219808, EPI_ISL_17219811, EPI_ISL_17219815, EPI_ISL_17219817, EPI_ISL_17219822, EPI_ISL_17219823, EPI_ISL_17219824, EPI_ISL_17219829, EPI_ISL_17219832, EPI_ISL_17219836, EPI_ISL_17219838, EPI_ISL_17219841, EPI_ISL_17219842, EPI_ISL_17219846,
EPI_ISL_17219852, EPI_ISL_17219855, EPI_ISL_17219856, EPI_ISL_17219860, EPI_ISL_17219865, EPI_ISL_17219869, EPI_ISL_17219870, EPI_ISL_17219891, EPI_ISL_17219914, EPI_ISL_17219916, EPI_ISL_17219919, EPI_ISL_17219922, EPI_ISL_17219925, EPI_ISL_17219929, EPI_ISL_17219934, EPI_ISL_17219935, EPI_ISL_17219942, EPI_ISL_17219943, EPI_ISL_17219944, EPI_ISL_17219953,
EPI_ISL_17219974, EPI_ISL_17219977, EPI_ISL_17219978, EPI_ISL_17219979, EPI_ISL_17219984, EPI_ISL_17219985, EPI_ISL_17219988, EPI_ISL_17219989, EPI_ISL_17220020, EPI_ISL_17220044, EPI_ISL_17220047, EPI_ISL_17220049, EPI_ISL_17220056, EPI_ISL_17220059, EPI_ISL_17220062, EPI_ISL_17220074, EPI_ISL_17220078, EPI_ISL_17220079, EPI_ISL_17220082,
EPI_ISL_17220085, EPI_ISL_17220088, EPI_ISL_17220093, EPI_ISL_17220094, EPI_ISL_17220097, EPI_ISL_17220100, EPI_ISL_17220109, EPI_ISL_17220112, EPI_ISL_17220117, EPI_ISL_17220118, EPI_ISL_17220123, EPI_ISL_17220126, EPI_ISL_17220131, EPI_ISL_17220134, EPI_ISL_17220138, EPI_ISL_17220144, EPI_ISL_17220145, EPI_ISL_17220151, EPI_ISL_17220152, EPI_ISL_17220162,
EPI_ISL_17220163, EPI_ISL_17220164, EPI_ISL_17220170, EPI_ISL_17220171, EPI_ISL_17220172, EPI_ISL_17220177, EPI_ISL_17220178, EPI_ISL_17220181, EPI_ISL_17220182, EPI_ISL_17220183, EPI_ISL_17220185, EPI_ISL_17220187, EPI_ISL_17220190, EPI_ISL_17220197, EPI_ISL_17220198, EPI_ISL_17220221, EPI_ISL_17220224, EPI_ISL_17220226, EPI_ISL_17220235,
EPI_ISL_17220251, EPI_ISL_17220255, EPI_ISL_17220259, EPI_ISL_17220262, EPI_ISL_17220266, EPI_ISL_17220268, EPI_ISL_17220271, EPI_ISL_17220274, EPI_ISL_17220276, EPI_ISL_17220281, EPI_ISL_17220282, EPI_ISL_17220285, EPI_ISL_17220286, EPI_ISL_17220292, EPI_ISL_17220300, EPI_ISL_17220303, EPI_ISL_17220306, EPI_ISL_17220314, EPI_ISL_17220326, EPI_ISL_17220328,
EPI_ISL_17220342, EPI_ISL_17220343, EPI_ISL_17220358, EPI_ISL_17220373, EPI_ISL_17220378, EPI_ISL_17220380, EPI_ISL_17220382, EPI_ISL_17220386, EPI_ISL_17220389, EPI_ISL_17220393, EPI_ISL_17220400, EPI_ISL_17220402, EPI_ISL_17220406, EPI_ISL_17220439, EPI_ISL_17220441, EPI_ISL_17220456, EPI_ISL_17220457, EPI_ISL_17220458, EPI_ISL_17220459,
EPI_ISL_17220462, EPI_ISL_17220465, EPI_ISL_17220466, EPI_ISL_17220473, EPI_ISL_17220476, EPI_ISL_17220479, EPI_ISL_17220482, EPI_ISL_17220483, EPI_ISL_17220486, EPI_ISL_17220487, EPI_ISL_17220490, EPI_ISL_17220491, EPI_ISL_17220494, EPI_ISL_17220496, EPI_ISL_17220498, EPI_ISL_17220500, EPI_ISL_17220512, EPI_ISL_17220513, EPI_ISL_17220516, EPI_ISL_17220521,
EPI_ISL_17220522, EPI_ISL_17220523, EPI_ISL_17220527, EPI_ISL_17220533, EPI_ISL_17220535, EPI_ISL_17220536, EPI_ISL_17220538, EPI_ISL_17220541, EPI_ISL_17220542, EPI_ISL_17220547, EPI_ISL_17220556, EPI_ISL_17220560, EPI_ISL_17220564, EPI_ISL_17220565, EPI_ISL_17220569, EPI_ISL_17220575, EPI_ISL_17220576, EPI_ISL_17220578, EPI_ISL_17220581,
EPI_ISL_17220582, EPI_ISL_17220583, EPI_ISL_17220590, EPI_ISL_17220591, EPI_ISL_17220592, EPI_ISL_17220593, EPI_ISL_17220598, EPI_ISL_17220600, EPI_ISL_17220605, EPI_ISL_17220608, EPI_ISL_17220611, EPI_ISL_17220616, EPI_ISL_17220617, EPI_ISL_17220629, EPI_ISL_17220631, EPI_ISL_17220633, EPI_ISL_17220640, EPI_ISL_17220654, EPI_ISL_17220659, EPI_ISL_17220666,
EPI_ISL_17220682, EPI_ISL_17220689, EPI_ISL_17220691, EPI_ISL_17220707, EPI_ISL_17220708, EPI_ISL_17220711, EPI_ISL_17220715, EPI_ISL_17220716, EPI_ISL_17220718, EPI_ISL_17220725, EPI_ISL_17220727, EPI_ISL_17220730, EPI_ISL_17220737, EPI_ISL_17220738, EPI_ISL_17220739, EPI_ISL_17220753, EPI_ISL_17220755, EPI_ISL_17220758, EPI_ISL_17220766,
EPI_ISL_17220778, EPI_ISL_17220780, EPI_ISL_17220783, EPI_ISL_17220786, EPI_ISL_17220787, EPI_ISL_17220788, EPI_ISL_17220789, EPI_ISL_17220794, EPI_ISL_17220795, EPI_ISL_17220796, EPI_ISL_17220799, EPI_ISL_17220800, EPI_ISL_17220805, EPI_ISL_17220809, EPI_ISL_17220811, EPI_ISL_17220812, EPI_ISL_17220819, EPI_ISL_17220820, EPI_ISL_17220823, EPI_ISL_17220824,
EPI_ISL_17220825, EPI_ISL_17220832, EPI_ISL_17220833, EPI_ISL_17220834, EPI_ISL_17220836, EPI_ISL_17220837, EPI_ISL_17220842, EPI_ISL_17220844, EPI_ISL_17220850, EPI_ISL_17220851, EPI_ISL_17220853, EPI_ISL_17220857, EPI_ISL_17220858, EPI_ISL_17220859, EPI_ISL_17220860, EPI_ISL_17220865, EPI_ISL_17220871, EPI_ISL_17220873, EPI_ISL_17220883, EPI_ISL_17220890,
EPI_ISL_17220897, EPI_ISL_17220900, EPI_ISL_17220909, EPI_ISL_17220911, EPI_ISL_17220912, EPI_ISL_17220918, EPI_ISL_17220922, EPI_ISL_17220923, EPI_ISL_17220926, EPI_ISL_17220931, EPI_ISL_17220932, EPI_ISL_17220935, EPI_ISL_17220936, EPI_ISL_17220944, EPI_ISL_17220946, EPI_ISL_17220949, EPI_ISL_17220950, EPI_ISL_17220953, EPI_ISL_17220967, EPI_ISL_17220973,
EPI_ISL_17220987, EPI_ISL_17220988, EPI_ISL_17220990, EPI_ISL_17220996, EPI_ISL_17220999, EPI_ISL_17221003, EPI_ISL_17221008, EPI_ISL_17221020, EPI_ISL_17221025, EPI_ISL_17221027, EPI_ISL_17221032, EPI_ISL_17221034, EPI_ISL_17221035, EPI_ISL_17221039, EPI_ISL_17221041, EPI_ISL_17221056, EPI_ISL_17221057, EPI_ISL_17221062, EPI_ISL_17221063, EPI_ISL_17221068,
EPI_ISL_17221071, EPI_ISL_17221073, EPI_ISL_17221085, EPI_ISL_17221088, EPI_ISL_17221090, EPI_ISL_17221094, EPI_ISL_17221095, EPI_ISL_17221103, EPI_ISL_17221110, EPI_ISL_17221111, EPI_ISL_17221114, EPI_ISL_17221118, EPI_ISL_17221125, EPI_ISL_17221127, EPI_ISL_17221128, EPI_ISL_17221129, EPI_ISL_17221132, EPI_ISL_17221134, EPI_ISL_17221135,
EPI_ISL_17221138, EPI_ISL_17221141, EPI_ISL_17221144, EPI_ISL_17221145, EPI_ISL_17221155, EPI_ISL_17221156, EPI_ISL_17221157, EPI_ISL_17221170, EPI_ISL_17221171, EPI_ISL_17221174, EPI_ISL_17221175, EPI_ISL_17221176, EPI_ISL_17221178, EPI_ISL_17221182, EPI_ISL_17221183, EPI_ISL_17221186, EPI_ISL_17221188, EPI_ISL_17221191, EPI_ISL_17221205, EPI_ISL_17221207,
EPI_ISL_17221213, EPI_ISL_17221227, EPI_ISL_17221229, EPI_ISL_17221237, EPI_ISL_17221238, EPI_ISL_17221240, EPI_ISL_17221245, EPI_ISL_17221248, EPI_ISL_17221249, EPI_ISL_17221260, EPI_ISL_17221266, EPI_ISL_17221270, EPI_ISL_17221271, EPI_ISL_17221274, EPI_ISL_17221275, EPI_ISL_17221280, EPI_ISL_17221282, EPI_ISL_17221298, EPI_ISL_17221303, EPI_ISL_17221309,
EPI_ISL_17221313, EPI_ISL_17221315, EPI_ISL_17221317, EPI_ISL_17221320, EPI_ISL_17221322, EPI_ISL_17221335, EPI_ISL_17221336, EPI_ISL_17221356, EPI_ISL_17221357, EPI_ISL_17221361, EPI_ISL_17221362, EPI_ISL_17221372, EPI_ISL_17221381, EPI_ISL_17221384, EPI_ISL_17221397, EPI_ISL_17221402, EPI_ISL_17221416, EPI_ISL_17221419, EPI_ISL_17221425, EPI_ISL_17221426,
EPI_ISL_17221427, EPI_ISL_17221438, EPI_ISL_17221439, EPI_ISL_17221440, EPI_ISL_17221443, EPI_ISL_17221446, EPI_ISL_17221447, EPI_ISL_17221448, EPI_ISL_17221455, EPI_ISL_17221458, EPI_ISL_17221459, EPI_ISL_17221460, EPI_ISL_17221464, EPI_ISL_17221467, EPI_ISL_17221468, EPI_ISL_17221470, EPI_ISL_17221475, EPI_ISL_17221476, EPI_ISL_17221477,
EPI_ISL_17221481, EPI_ISL_17221485, EPI_ISL_17221489, EPI_ISL_17221492, EPI_ISL_17221494, EPI_ISL_17221502, EPI_ISL_17221503, EPI_ISL_17221513, EPI_ISL_17221514, EPI_ISL_17221522, EPI_ISL_17221526, EPI_ISL_17221527, EPI_ISL_17221529, EPI_ISL_17221531, EPI_ISL_17221536

see above | Astrazeneca Laboratory | Astrazeneca Laboratory | Wilkins,D., Langedijk,A.C., Lebbink,R.J., Morehouse,C.A., Abram,M.E., Ahani,B., Aksyuk,A.A., Baraldi,E., Brady,T., Chen,A.T., Chi,H., Choi,E.H., Cohen,R., Danilenko,D.M., Gopalakrishnan,V., Greenough,A., Heikkinen,T., Hosoya,M., Keller,C., Kelly,E.J., Kragten-Tabatabaie,L., Martinon-Torres,F., Mascarenas-de Los Santos,A.H., Nunes,M.C., Palomino,M.A., Papenburg,J., Pernica,J.M., Richmond,P., Stein,R.T., Tuffy,K.M., Verwey,C., Esser,M.T., Tabor,D.E. and Bont,L.J.

EPI_ISL_17238898, EPI_ISL_17238899, EPI_ISL_17238900, EPI_ISL_17238901, EPI_ISL_17238902, EPI_ISL_17238903, EPI_ISL_17238904, EPI_ISL_17238905, EPI_ISL_17238906, EPI_ISL_17238907, EPI_ISL_17238908, EPI_ISL_17238909, EPI_ISL_17238910, EPI_ISL_17238911, EPI_ISL_17238912, EPI_ISL_17238913, EPI_ISL_17238914, EPI_ISL_17238915, EPI_ISL_17238916, EPI_ISL_17238917,
EPI_ISL_17238918, EPI_ISL_17238919, EPI_ISL_17238920, EPI_ISL_17238921, EPI_ISL_17238922, EPI_ISL_17238923, EPI_ISL_17238924, EPI_ISL_17238925, EPI_ISL_17238926, EPI_ISL_17238927, EPI_ISL_17238928, EPI_ISL_17238929, EPI_ISL_17238930, EPI_ISL_17238931, EPI_ISL_17238932, EPI_ISL_17238933, EPI_ISL_17238934, EPI_ISL_17238935, EPI_ISL_17238936, EPI_ISL_17238937,
EPI_ISL_17238938, EPI_ISL_17238939, EPI_ISL_17238940, EPI_ISL_17238941, EPI_ISL_17238942, EPI_ISL_17238943, EPI_ISL_17238944, EPI_ISL_17238945, EPI_ISL_17238946, EPI_ISL_17238947, EPI_ISL_17238948, EPI_ISL_17238949, EPI_ISL_17238950, EPI_ISL_17238951, EPI_ISL_17238952, EPI_ISL_17238953, EPI_ISL_17238954, EPI_ISL_17238955, EPI_ISL_17238956, EPI_ISL_17238957,
EPI_ISL_17238958, EPI_ISL_17238959, EPI_ISL_17238960, EPI_ISL_17238961, EPI_ISL_17238962, EPI_ISL_17238963, EPI_ISL_17238964, EPI_ISL_17238965, EPI_ISL_17238966, EPI_ISL_17238967, EPI_ISL_17238968, EPI_ISL_17238969, EPI_ISL_17238970, EPI_ISL_17238971, EPI_ISL_17238972, EPI_ISL_17238973, EPI_ISL_17238974

see above | Molecular Biology/Microbiology Research Laboratory. Universidade Federal do Paraná | Molecular Biology/Microbiology Research Laboratory. Universidade Federal do Paraná | Jucelia Stadinicki, Maria do Carmo Debur, Heloisa Ihle Garcia Giamberardino, Sonia Mara Raboni

EPI_ISL_17383430, EPI_ISL_17383435, EPI_ISL_17383436, EPI_ISL_17383437, EPI_ISL_17383438, EPI_ISL_17383442, EPI_ISL_17383446, EPI_ISL_17383447, EPI_ISL_17383448, EPI_ISL_17383452, EPI_ISL_17383453, EPI_ISL_17383454, EPI_ISL_17383455, EPI_ISL_17383458, EPI_ISL_17383462, EPI_ISL_17383465, EPI_ISL_17383466, EPI_ISL_17383467, EPI_ISL_17383471, EPI_ISL_17383472,
EPI_ISL_17383473, EPI_ISL_17383474, EPI_ISL_17383480, EPI_ISL_17383481, EPI_ISL_17383482, EPI_ISL_17383484, EPI_ISL_17383485, EPI_ISL_17383487, EPI_ISL_17383490, EPI_ISL_17383491, EPI_ISL_17383492, EPI_ISL_17383495, EPI_ISL_17383496, EPI_ISL_17383501, EPI_ISL_17383505, EPI_ISL_17383509, EPI_ISL_17383511, EPI_ISL_17383513, EPI_ISL_17383514, EPI_ISL_17383515,
EPI_ISL_17383517, EPI_ISL_17383518, EPI_ISL_17383519

see above | Faculty of Medicine, University of Ljubljana | Faculty of Medicine, University of Ljubljana | Jevsnik Virant,M., Lustrek,M. and Petrovec,M.

EPI_ISL_17594408, EPI_ISL_17594409, EPI_ISL_17594410, EPI_ISL_17594411, EPI_ISL_17594413, EPI_ISL_17594414, EPI_ISL_17594415, EPI_ISL_17594420, EPI_ISL_17594497, EPI_ISL_17594498, EPI_ISL_17594499, EPI_ISL_17594501, EPI_ISL_17594502, EPI_ISL_17594503, EPI_ISL_17594504, EPI_ISL_17594505, EPI_ISL_17594506, EPI_ISL_17594507, EPI_ISL_17594508, EPI_ISL_17594509,
EPI_ISL_17594510, EPI_ISL_17594516, EPI_ISL_17594519, EPI_ISL_17594520, EPI_ISL_17594521, EPI_ISL_17594522, EPI_ISL_17594523, EPI_ISL_17594524, EPI_ISL_17594532, EPI_ISL_17594533, EPI_ISL_17594534, EPI_ISL_17594535, EPI_ISL_17594536, EPI_ISL_17594537, EPI_ISL_17594538, EPI_ISL_17594564, EPI_ISL_17594565, EPI_ISL_17594566, EPI_ISL_17594567, EPI_ISL_17594568,
EPI_ISL_17594569, EPI_ISL_17594570, EPI_ISL_17594571, EPI_ISL_17594572, EPI_ISL_17594573, EPI_ISL_17594574, EPI_ISL_17594576, EPI_ISL_17594577, EPI_ISL_17594578, EPI_ISL_17594579, EPI_ISL_17594580, EPI_ISL_17594589, EPI_ISL_17594590, EPI_ISL_17594591, EPI_ISL_17594595, EPI_ISL_17594597

see above | Chang Bing Show Chwan Memorial Hospital | Chang Bing Show Chwan Memorial Hospital | Lee,C.Y.

EPI_ISL_17597871, EPI_ISL_17597881, EPI_ISL_17597898 | Laboratório de Virologia - Instituto Nacional de Saúde | Laboratório de Vírus Respiratórios e do Sarampo - Instituto Oswaldo Cruz, Laboratório de Virologia - Instituto Nacional de Saúde | Almiro Tivane, Paola Resende, Neuza Nguenha, Loira Machalele, Sadia Alí, Délcio Muteto, Aunésia Marrurele, Mirela Pale, Félix Gundane, Judite Salência, Marilda Siqueira

EPI_ISL_1760383, EPI_ISL_1760387, EPI_ISL_1760388, EPI_ISL_1760389, EPI_ISL_1760390, EPI_ISL_1760391, EPI_ISL_1760392, EPI_ISL_1760395, EPI_ISL_1760396, EPI_ISL_1760397, EPI_ISL_1760398, EPI_ISL_1760399, EPI_ISL_1760400, EPI_ISL_1760402, EPI_ISL_1760403, EPI_ISL_1760404, EPI_ISL_1760405, EPI_ISL_1760409, EPI_ISL_1760410, EPI_ISL_1760411, EPI_ISL_1760412, EPI_ISL_1760413,
EPI_ISL_1760414, EPI_ISL_1760416, EPI_ISL_1760418, EPI_ISL_1760419, EPI_ISL_1760421, EPI_ISL_1760422, EPI_ISL_1760423, EPI_ISL_1760425, EPI_ISL_1760426, EPI_ISL_1760427, EPI_ISL_1760428, EPI_ISL_1760429, EPI_ISL_1760430, EPI_ISL_1760431, EPI_ISL_1760433, EPI_ISL_1760435, EPI_ISL_1760436, EPI_ISL_1760437, EPI_ISL_1760439, EPI_ISL_1760440

see above | Royal Children's Hospital, 50 Flemington Rd., Park | WHO Influenza Centre for Reference and Research on Influenza | Angela Todd, Yi-Mo Deng, Annette Alafaci, Naomi Komadina

EPI_ISL_17975547, EPI_ISL_17975549, EPI_ISL_17975563, EPI_ISL_17975566, EPI_ISL_17975569, EPI_ISL_17975576, EPI_ISL_17975577, EPI_ISL_17975596, EPI_ISL_17975599, EPI_ISL_17975600, EPI_ISL_17975606, EPI_ISL_17975615, EPI_ISL_17975632, EPI_ISL_17975641

see above | Sapienza University | Sapienza University | Pierangeli,A.

EPI_ISL_18005800, EPI_ISL_18005815, EPI_ISL_18005817, EPI_ISL_18005818, EPI_ISL_18005819, EPI_ISL_18005820, EPI_ISL_18005821, EPI_ISL_18005822, EPI_ISL_18005823, EPI_ISL_18005824, EPI_ISL_18005825, EPI_ISL_18005826, EPI_ISL_18005827, EPI_ISL_18005828, EPI_ISL_18005829, EPI_ISL_18005830, EPI_ISL_18005831, EPI_ISL_18005833, EPI_ISL_18005834, EPI_ISL_18005835,
EPI_ISL_18005925, EPI_ISL_18005935

see above | Robert Koch-Institute Nationales Referenzzentrum für Influenza | Robert Koch-Institute Nationales Referenzzentrum für Influenza | Sophie Kondgen, Janine Reiche

EPI_ISL_18090572, EPI_ISL_18090576, EPI_ISL_18090579, EPI_ISL_18090581, EPI_ISL_18090583, EPI_ISL_18090584, EPI_ISL_18090585, EPI_ISL_18090588, EPI_ISL_18090589, EPI_ISL_18090590, EPI_ISL_18090592, EPI_ISL_18090600, EPI_ISL_18090601, EPI_ISL_18090603, EPI_ISL_18090605, EPI_ISL_18090609, EPI_ISL_18090610, EPI_ISL_18090613, EPI_ISL_18090614, EPI_ISL_18090615,
EPI_ISL_18090617, EPI_ISL_18090621, EPI_ISL_18090627, EPI_ISL_18090628, EPI_ISL_18090630, EPI_ISL_18090631, EPI_ISL_18090632, EPI_ISL_18090633, EPI_ISL_18090634, EPI_ISL_18090635, EPI_ISL_18090638, EPI_ISL_18090639, EPI_ISL_18090640, EPI_ISL_18090641, EPI_ISL_18090644, EPI_ISL_18090649, EPI_ISL_18090650, EPI_ISL_18090651, EPI_ISL_18090655, EPI_ISL_18090659,
EPI_ISL_18090661, EPI_ISL_18090664, EPI_ISL_18090668, EPI_ISL_18090669, EPI_ISL_18090670, EPI_ISL_18090672, EPI_ISL_18090673, EPI_ISL_18090674, EPI_ISL_18090675, EPI_ISL_18090681, EPI_ISL_18090683, EPI_ISL_18090685, EPI_ISL_18090687, EPI_ISL_18090688, EPI_ISL_18090691, EPI_ISL_18090693

see above | Westmead Institute for Medical Research & Sydney Infectious Disease Institute | Westmead Institute for Medical Research & Sydney Infectious Disease Institute | Pangesti,K.N.A., Ansari,H.R., Bayoumi,A., Kesson,A.M., Hill-Cawthorne,G.A. and Abd El Ghany,M.

EPI_ISL_18119455, EPI_ISL_18119461, EPI_ISL_18119464, EPI_ISL_18119471, EPI_ISL_18119475, EPI_ISL_18119477, EPI_ISL_18119478, EPI_ISL_18119479, EPI_ISL_18119482, EPI_ISL_18119488, EPI_ISL_18119492, EPI_ISL_18119494, EPI_ISL_18119496, EPI_ISL_18119497, EPI_ISL_18119505, EPI_ISL_18119514, EPI_ISL_18119517, EPI_ISL_18119520, EPI_ISL_18119524, EPI_ISL_18119528, EPI_ISL_18119535			
see above	Kenya Medical Research Institute - KEMRI Wellcome Trust Research Programme	Kenya Medical Research Institute - KEMRI Wellcome Trust Research Programme	Odoyo,S.O., Lambisia,A.W., Murunga,N., Lewa,C.S., Maina,G., Okanda,D., Mutunga,M., Agoti,C.N., Nokes,J.D. and Githinji,G.L.
EPI_ISL_18310827, EPI_ISL_18310829, EPI_ISL_18310830, EPI_ISL_18310831, EPI_ISL_18310833, EPI_ISL_18310834, EPI_ISL_18310840, EPI_ISL_18310841, EPI_ISL_18310842, EPI_ISL_18310844, EPI_ISL_18310848, EPI_ISL_18310854, EPI_ISL_18310855, EPI_ISL_18310861, EPI_ISL_18310862, EPI_ISL_18310866, EPI_ISL_18310867, EPI_ISL_18310868, EPI_ISL_18310871, EPI_ISL_18310872, EPI_ISL_18310877, EPI_ISL_18310878, EPI_ISL_18310879, EPI_ISL_18310880, EPI_ISL_18310884, EPI_ISL_18310887, EPI_ISL_18310889, EPI_ISL_18310892, EPI_ISL_18310897, EPI_ISL_18310899, EPI_ISL_18310905, EPI_ISL_18310906, EPI_ISL_18310907, EPI_ISL_18310911, EPI_ISL_18310914, EPI_ISL_18310915, EPI_ISL_18310917			
see above	Global Health Research Center	Global Health Research Center	Umar,S., Yang,R., Wang,X., Liu,Y., Ke,P. and Qin,S.
EPI_ISL_18334095, EPI_ISL_18334118, EPI_ISL_18334138, EPI_ISL_18334143	Tianjin Children's Hospital, Tianjin Pediatric Research Institute	Tianjin Children's Hospital, Tianjin Pediatric Research Institute	Hou,M., Meng,C., Dong,L. and Wang,H.
EPI_ISL_18334164, EPI_ISL_18334165, EPI_ISL_18334168, EPI_ISL_18334170, EPI_ISL_18334172, EPI_ISL_18334174, EPI_ISL_18334181, EPI_ISL_18334182, EPI_ISL_18334183, EPI_ISL_18334184, EPI_ISL_18334186, EPI_ISL_18334188, EPI_ISL_18334189, EPI_ISL_18334192, EPI_ISL_18334193, EPI_ISL_18334197, EPI_ISL_18334198, EPI_ISL_18334200, EPI_ISL_18334202, EPI_ISL_18334203, EPI_ISL_18334207, EPI_ISL_18334208, EPI_ISL_18334209, EPI_ISL_18334210, EPI_ISL_18334211, EPI_ISL_18334212, EPI_ISL_18334217, EPI_ISL_18334218, EPI_ISL_18334219, EPI_ISL_18334223, EPI_ISL_18334224, EPI_ISL_18334226, EPI_ISL_18334227, EPI_ISL_18334228, EPI_ISL_18334229, EPI_ISL_18334233, EPI_ISL_18334234, EPI_ISL_18334235			
see above	Baylor College of Medicine, Division of Pediatric Tropical Medicine	Baylor College of Medicine, Division of Pediatric Tropical Medicine	Avadhanula,V., Agustinho,D.P., Menon,V.K., Chemaly,R.F., Shah,D.P., Qin,X., Surathu,A., Doddapaneni,H., Muzny,D.M., Metcalf,G.A., Gregeen,S.J., Gibbs,R.A., Petrosino,J.J., Sedlazeck,F.J. and Piedra,P.A.
EPI_ISL_18402126, EPI_ISL_18402127, EPI_ISL_18402128, EPI_ISL_18402130, EPI_ISL_18402131, EPI_ISL_18402132, EPI_ISL_18402134, EPI_ISL_18402135, EPI_ISL_18402136, EPI_ISL_18402137, EPI_ISL_18402168, EPI_ISL_18402170, EPI_ISL_18402171, EPI_ISL_18402172, EPI_ISL_18402173, EPI_ISL_18402174, EPI_ISL_18402175, EPI_ISL_18402176, EPI_ISL_18402177, EPI_ISL_18402179, EPI_ISL_18402180, EPI_ISL_18405788, EPI_ISL_18405789, EPI_ISL_18405790, EPI_ISL_18405791, EPI_ISL_18405792, EPI_ISL_18405793, EPI_ISL_18405794, EPI_ISL_18405795, EPI_ISL_18405796, EPI_ISL_18405797, EPI_ISL_18405798, EPI_ISL_18405799, EPI_ISL_18405800, EPI_ISL_18405801, EPI_ISL_18405802, EPI_ISL_18405803, EPI_ISL_18405804, EPI_ISL_18405805, EPI_ISL_18405806, EPI_ISL_18405807, EPI_ISL_18405808, EPI_ISL_18405809, EPI_ISL_18405810, EPI_ISL_18405811, EPI_ISL_18405812, EPI_ISL_18405813, EPI_ISL_18405814, EPI_ISL_18405815, EPI_ISL_18405816, EPI_ISL_18405817, EPI_ISL_18405818, EPI_ISL_18405819, EPI_ISL_18405820, EPI_ISL_18405821, EPI_ISL_18405822, EPI_ISL_18405823, EPI_ISL_18405824, EPI_ISL_18405825, EPI_ISL_18405826, EPI_ISL_18405827, EPI_ISL_18405828, EPI_ISL_18405829, EPI_ISL_18405830, EPI_ISL_18405831, EPI_ISL_18405833, EPI_ISL_18405834, EPI_ISL_18405835, EPI_ISL_18405836, EPI_ISL_18405939, EPI_ISL_18405949			
see above	Niigata University (IDRC) Infectious Diseases Research Center	Niigata University (IDRC) Infectious Diseases Research Center	Wint Wint Phyu, Reiko Saito
EPI_ISL_18482722, EPI_ISL_18482725, EPI_ISL_18482754, EPI_ISL_18482763, EPI_ISL_18482810	Beijing Children's Hospital, Capital Medical University, Laboratory of Infection and Virology	Beijing Children's Hospital, Capital Medical University, Laboratory of Infection and Virology	Li,F., Zhu,Y. and Chen,X.
EPI_ISL_2156815, EPI_ISL_2156817, EPI_ISL_2156820	Royal Children's Hospital	WHO Influenza Centre for Reference and Research on Influenza	Angela Todd, Yi-Mo Deng, Annette Alafaci, Naomi Komadina
EPI_ISL_2543854, EPI_ISL_2543855, EPI_ISL_2543856, EPI_ISL_2543857, EPI_ISL_2543858, EPI_ISL_2543859	Institut Pasteur de Cote d'Ivoire	WHO Collaborating Centre for Reference and Research on Influenza	Xiaomin Dong, Herve Kadjo, Yi-Mo Deng, Ammar Aziz, Naomi Komadina
EPI_ISL_2543860, EPI_ISL_2543861, EPI_ISL_2543862, EPI_ISL_2543863, EPI_ISL_2543864, EPI_ISL_2543865, EPI_ISL_2543866, EPI_ISL_2543867, EPI_ISL_2543868, EPI_ISL_2543869, EPI_ISL_2543870, EPI_ISL_2543871, EPI_ISL_2543872, EPI_ISL_2543873, EPI_ISL_2543874, EPI_ISL_2543875, EPI_ISL_2543876, EPI_ISL_2543877, EPI_ISL_2543878, EPI_ISL_2543879, EPI_ISL_2543880, EPI_ISL_2543881, EPI_ISL_2543882, EPI_ISL_2543883, EPI_ISL_2543884, EPI_ISL_2543885, EPI_ISL_2543886, EPI_ISL_2543887, EPI_ISL_2543888, EPI_ISL_2543889			
see above	National Center for Communicable Diseases	WHO Collaborating Centre for Reference and Research on Influenza	Xiaomin Dong, Darmaa Bardach, Yi-Mo Deng, Ammar Aziz, Naomi Komadina
EPI_ISL_2544069, EPI_ISL_2544071	Epidemiology and Demography, KEMRI-Wellcome Trust Research Programme	Epidemiology and Demography, KEMRI-Wellcome Trust Research Programme	Kamau,E.M., Agoti,C.N., Lewa,C.S., Cane,P.A., Bett,A., Medley,G.F. and Nokes,J.D.
EPI_ISL_2544087	Translational Medicine Institute, Affiliated the First People's Hospital of Chenzhou, University of South China	Translational Medicine Institute, Affiliated the First People's Hospital of Chenzhou, University of South China	Fan,R.Y., Qu,X.W. and Fan,C.P.
EPI_ISL_2544116, EPI_ISL_2544119	Microbiology, Hospital Universitari Vall d'Hebron	Microbiology, Hospital Universitari Vall d'Hebron	Gimferrer,L., Campins,M., Codina,M.G., Martin,M.D.C., Fuentes,F., Esperalba,J., Bruguera,A., Vilca,L.M., Armadans,L., Vall,A., Pumarola,T. and Anton,A.
EPI_ISL_2544121, EPI_ISL_2544126	Reference Microbiology, Public Health England National Infection Services	Reference Microbiology, Public Health England National Infection Services	Valappil,M., Talts,T., Ellis,J., Sails,A., Eltringham,G., Waugh,S., Gould,K., Harrison,I., Pebody,R. and Zambon,M.
EPI_ISL_2544142, EPI_ISL_2544143, EPI_ISL_2544145	J. Craig Venter Institute	J. Craig Venter Institute	Das,S.R., Halpin,R.A., Shilts,M., Puri,V., Akopov,A., Fedorova,N., Stockwell,T., Amedeo,P., Bishop,B., Katzel,D., Schobel,S., Shrivastava,S. and Hartert,T.
EPI_ISL_2544147	Lab Medicine, UW	Lab Medicine, UW	Greninger,A.L., Makhsous,N., Kuypers,J.M., Shean,R.C. and Jerome,K.R.
EPI_ISL_2544173, EPI_ISL_2544174, EPI_ISL_2544177	J. Craig Venter Institute	J. Craig Venter Institute	Shabman,R., Das,S.R., Shilts,M., Fedorova,N., Puri,V., Shrivastava,S., Amedeo,P., Williams,M., Barratt,K., Mitchell,J. and Jennings,L.
EPI_ISL_2544182, EPI_ISL_2544183	Virology Laboratory, Dr. Ricardo Gutierrez Children Hospital	Virology Laboratory, Dr. Ricardo Gutierrez Children Hospital	Rojo,G.L., Goya,S., Orellana,M., Sancilio,A., Rodriguez Perez,A., Montali,C., Garcia,C., Sosa,L., Musto,A., Hamilton,G., Alvarez,D., Castello,A. and Viegas,M.
EPI_ISL_2544184	Virology Laboratory, Dr. Ricardo Gutierrez Children Hospital	Virology Laboratory, Dr. Ricardo Gutierrez Children Hospital	Goya,S., Valinotto,L.E., Tittarelli,E., Rojo,G.L., Greninger,A., Luso,S., Natale,M., Mistchenko,A.S. and Viegas,M.
EPI_ISL_2544185	Virology Laboratory, Dr. Ricardo Gutierrez Children Hospital	Virology Laboratory, Dr. Ricardo Gutierrez Children Hospital	Goya,S., Valinotto,L.E., Tittarelli,E., Rojo,G.L., Greninger,A., Zaiat,J., Marti,M., Mistchenko,A.S. and Viegas,M.
EPI_ISL_2544186	Virology Laboratory, Dr. Ricardo Gutierrez Children Hospital	Virology Laboratory, Dr. Ricardo Gutierrez Children Hospital	Goya,S., Rojo,G.L., Valinotto,L.E., Mistchenko,A.S. and Viegas,M.
EPI_ISL_2558777	Public Health Laboratory, Minnesota Department of Health	Public Health Laboratory, Minnesota Department of Health	Wang,X., Thielen,B.K. and Lynfield,R.
EPI_ISL_2558779, EPI_ISL_2558785, EPI_ISL_2558786	Kazuya Shirato National Institute of Infectious Diseases, Virology III	Kazuya Shirato National Institute of Infectious Diseases, Virology III	Shirato,K., Sato,K., Dapat,I., Nao,N., Omiya,S., Matsuyama,S., Takeda,M. and Nishimura,H.
EPI_ISL_2558825, EPI_ISL_2558850	Depto Microbiologia, Instituto de Ciencias Biomedicas, Universidade de Sao Paulo	Depto Microbiologia, Instituto de Ciencias Biomedicas, Universidade de Sao Paulo	Moura,F.E.A., Thomazelli,L.M., Campelo,F.S., Delfraro,A., Arbiza,J. and Durigon,E.L.
EPI_ISL_2558884, EPI_ISL_2558886, EPI_ISL_2558887, EPI_ISL_2558888	Kazuya Shirato National Institute of Infectious Diseases, Virology III	Kazuya Shirato National Institute of Infectious Diseases, Virology III	Shirato,K., Sato,K., Dapat,I., Nao,N., Omiya,S., Matsuyama,S., Takeda,M. and Nishimura,H.
EPI_ISL_2558891	Pediatrics, Mackay Memorial Hospital	Pediatrics, Mackay Memorial Hospital	Chi,H., Hsiao,K.-L., Weng,L.-C., Chiu,N.-C., Huang,L.-M., Chiu,Y.-Y., Liu,C.-P. and Liu,H.-F.
EPI_ISL_2558892	Pediatrics, Show-Chwan Memorial Hospital	Pediatrics, Show-Chwan Memorial Hospital	Lee,C.-Y.
EPI_ISL_2558893	Pediatrics, Mackay Memorial Hospital	Pediatrics, Mackay Memorial Hospital	Chi,H., Hsiao,K.-L., Weng,L.-C., Chiu,N.-C., Huang,L.-M., Chiu,Y.-Y., Liu,C.-P. and Liu,H.-F.
EPI_ISL_2558897, EPI_ISL_2558898, EPI_ISL_2558899, EPI_ISL_2558900, EPI_ISL_2558901, EPI_ISL_2558902, EPI_ISL_2558903, EPI_ISL_2558905, EPI_ISL_2558908	Kazuya Shirato National Institute of Infectious Diseases, Virology III	Kazuya Shirato National Institute of Infectious Diseases, Virology III	Shirato,K., Sato,K., Dapat,I., Nao,N., Omiya,S., Matsuyama,S., Takeda,M. and Nishimura,H.
EPI_ISL_2558927	Lab Medicine, UW	Lab Medicine, UW	Greninger,A.L., Makhsous,N., Kuypers,J.M., Shean,R.C. and Jerome,K.R.
EPI_ISL_2558937	Centre for Research And Knowledge Transfer in Biotechnology, University of Zagreb	Centre for Research And Knowledge Transfer in Biotechnology, University of Zagreb	Jagusic,M., Slovic,A., Santak,M., Kosutic-Gulija,T., Ivancic-Jelecki,J. and Forcic,D.
EPI_ISL_2558971	Lab Medicine, UW	Lab Medicine, UW	Greninger,A.L., Makhsous,N., Kuypers,J.M., Shean,R.C. and Jerome,K.R.
EPI_ISL_2558972	Pediatrics, Show-Chwan Memorial Hospital	Pediatrics, Show-Chwan Memorial Hospital	Lee,C.-Y.
EPI_ISL_2558982, EPI_ISL_2558983	Kazuya Shirato National Institute of Infectious Diseases, Virology III	Kazuya Shirato National Institute of Infectious Diseases, Virology III	Shirato,K., Sato,K., Dapat,I., Nao,N., Omiya,S., Matsuyama,S., Takeda,M. and Nishimura,H.
EPI_ISL_2558985	Koichi Hashimoto Fukushima Medical University, Department of Pediatrics, School of Medicine	Koichi Hashimoto Fukushima Medical University, Department of Pediatrics, School of Medicine	Fukuyama,Y., Takeshita,F., Norito,S., Hashimoto,K. and Hosoya,M.
EPI_ISL_2559081, EPI_ISL_2559082, EPI_ISL_2559083, EPI_ISL_2559084, EPI_ISL_2559085, EPI_ISL_2559086, EPI_ISL_2559087, EPI_ISL_2559088, EPI_ISL_2559089, EPI_ISL_2559090, EPI_ISL_2559091, EPI_ISL_2559092, EPI_ISL_2559093, EPI_ISL_2559094, EPI_ISL_2559095, EPI_ISL_2559096, EPI_ISL_2559097, EPI_ISL_2559098, EPI_ISL_2559099, EPI_ISL_2559100, EPI_ISL_2559102, EPI_ISL_2559103, EPI_ISL_2559105, EPI_ISL_2559107, EPI_ISL_2559109			
see above	Kazuya Shirato National Institute of Infectious Diseases, Virology III	Kazuya Shirato National Institute of Infectious Diseases, Virology III	Shirato,K., Sato,K., Dapat,I., Nao,N., Omiya,S., Matsuyama,S., Takeda,M. and Nishimura,H.
EPI_ISL_2559123	Medicine, American University of Beirut	Medicine, American University of Beirut	Abou-El-Hassan,H. and Zaraket,H.
EPI_ISL_2559130	Depto Microbiologia, Instituto de Ciencias Biomedicas, Universidade de Sao Paulo	Depto Microbiologia, Instituto de Ciencias Biomedicas, Universidade de Sao Paulo	Moura,F.E.A., Thomazelli,L.M., Campelo,F.S., Delfraro,A., Arbiza,J. and Durigon,E.L.
EPI_ISL_2559141, EPI_ISL_2559143, EPI_ISL_2559145	Centre for Research And Knowledge Transfer in Biotechnology, University of Zagreb	Centre for Research And Knowledge Transfer in Biotechnology, University of Zagreb	Slovic,A., Ivancic-Jelecki,J., Ljubin-Sternak,S., Mlinaric-Galinovic,G., Vilibic-Cavlek,T., Tabain,I. and Forcic,D.
EPI_ISL_2559155	University of Wuerzburg, Institute of Virology and Immunobiology	University of Wuerzburg, Institute of Virology and Immunobiology	Prifert,C., Hofmann,D. and Weissbrich,B.
EPI_ISL_2559158	Centre for Research And Knowledge Transfer in Biotechnology, University of Zagreb	Centre for Research And Knowledge Transfer in Biotechnology, University of Zagreb	Slovic,A., Ivancic-Jelecki,J., Ljubin-Sternak,S., Mlinaric-Galinovic,G., Vilibic-Cavlek,T., Tabain,I. and Forcic,D.
EPI_ISL_2559161, EPI_ISL_2559162	Virology, Evandro Chagas Institute	Virology, Evandro Chagas Institute	Santos,V.M., Ferreira,J.A., Lima,J.F., Barbagelata,L.S., Souza,E.M.A., Goncalves,M.S., SoNorth America / USA,E.C. Jr., Costa,I.B., Santos,M.C., SoNorth America / USA,R.C.M. and Mello,W.A.
EPI_ISL_2559177	University of Wuerzburg, Institute of Virology and Immunobiology	University of Wuerzburg, Institute of Virology and Immunobiology	Prifert,C., Hofmann,D. and Weissbrich,B.
EPI_ISL_2559178	Pediatrics, Show-Chwan Memorial Hospital	Pediatrics, Show-Chwan Memorial Hospital	Lee,C.-Y.
EPI_ISL_2559224	Department of Infectious Diseases, National Health Institute Doutor Ricardo Jorge	Department of Infectious Diseases, National Health Institute Doutor Ricardo Jorge	Saez-Lopez,E., Cristovao,P., Costa,I., Pechirra,P., Conde,P. and Guiomar,R.
EPI_ISL_2559225	Centre for Research And Knowledge Transfer in Biotechnology, University of Zagreb	Centre for Research And Knowledge Transfer in Biotechnology, University of Zagreb	Slovic,A., Ivancic-Jelecki,J., Ljubin-Sternak,S., Mlinaric-Galinovic,G., Vilibic-Cavlek,T., Tabain,I. and Forcic,D.
EPI_ISL_2559226, EPI_ISL_2559227, EPI_ISL_2559228, EPI_ISL_2559229	Department of Infectious Diseases, National Health Institute Doutor Ricardo Jorge	Department of Infectious Diseases, National Health Institute Doutor Ricardo Jorge	Saez-Lopez,E., Cristovao,P., Costa,I., Pechirra,P., Conde,P. and Guiomar,R.
EPI_ISL_2559252, EPI_ISL_2559272, EPI_ISL_2559274	Virology, Evandro Chagas Institute	Virology, Evandro Chagas Institute	Santos,V.M., Ferreira,J.A., Lima,J.F., Barbagelata,L.S., Souza,E.M.A., Goncalves,M.S., SoNorth America / USA,E.C. Jr., Costa,I.B., Santos,M.C., SoNorth America / USA,R.C.M. and Mello,W.A.
EPI_ISL_2559320	Public Health Laboratory, Minnesota Department of Health	Public Health Laboratory, Minnesota Department of Health	Wang,X., Thielen,B.K. and Lynfield,R.
EPI_ISL_2559400	Institute of Virology and AIDS Research, First Hospital of Jilin University	Institute of Virology and AIDS Research, First Hospital of Jilin University	Zheng,Y., Liu,L., Wang,S., Li,Z., Hou,M., Li,J., Yu,X.-F., Zhang,W. and Hua,S.
EPI_ISL_2559401, EPI_ISL_2559403, EPI_ISL_2559405	University of Wuerzburg, Institute of Virology and Immunobiology	University of Wuerzburg, Institute of Virology and Immunobiology	Prifert,C., Hofmann,D. and Weissbrich,B.
EPI_ISL_2559407	Epidemiology and Immunogenetic of Viral Infections LR14SP02, University	Epidemiology and Immunogenetic of Viral Infections LR14SP02, University	Ataoui,I., Jerbi,A., BenHamida-Rebai,M., Ben Hadj Fredj,M., Fodha,I., Bennour,H., Hammouda,H., Khlifa,M., Brini,I., Boussoffara,R., Boussetta,K., Abroug,S. and Trabelsi,A.

	Hospital Sahloul	Hospital Sahloul	
EPI_ISL_2559409	Department of Infectious Diseases, National Health Institute Doutor Ricardo Jorge	Department of Infectious Diseases, National Health Institute Doutor Ricardo Jorge	Saez-Lopez,E., Cristovao,P., Costa,I., Pechirra,P., Conde,P. and Guiomar,R.
EPI_ISL_2559411	Centre for Research And Knowledge Transfer in Biotechnology, University of Zagreb	Centre for Research And Knowledge Transfer in Biotechnology, University of Zagreb	Slovic,A., Ivancic-Jelecki,J., Ljubin-Sternak,S., Mlinaric-Galinovic,G., Vilibic-Cavlek,T., Tabain,I. and Forcic,D.
EPI_ISL_2559413, EPI_ISL_2559415, EPI_ISL_2559417, EPI_ISL_2559418	University of Wuerzburg, Institute of Virology and Immunobiology	University of Wuerzburg, Institute of Virology and Immunobiology	Prifert,C., Hofmann,D. and Weissbrich,B.
EPI_ISL_2559420, EPI_ISL_2559422, EPI_ISL_2559424, EPI_ISL_2559426, EPI_ISL_2559428, EPI_ISL_2559430, EPI_ISL_2559432, EPI_ISL_2559434, EPI_ISL_2559436	Department of Infectious Diseases, National Health Institute Doutor Ricardo Jorge	Department of Infectious Diseases, National Health Institute Doutor Ricardo Jorge	Saez-Lopez,E., Cristovao,P., Costa,I., Pechirra,P., Conde,P. and Guiomar,R.
EPI_ISL_2559449	University of Wuerzburg, Institute of Virology and Immunobiology	University of Wuerzburg, Institute of Virology and Immunobiology	Prifert,C., Hofmann,D. and Weissbrich,B.
EPI_ISL_2559453	Kazuya Shirato National Institute of Infectious Diseases, Virology III	Kazuya Shirato National Institute of Infectious Diseases, Virology III	Shirato,K., Sato,K., Dapat,I., Nao,N., Omiya,S., Matsuyama,S., Takeda,M. and Nishimura,H.
EPI_ISL_2559480, EPI_ISL_2559481, EPI_ISL_2559482, EPI_ISL_2559483, EPI_ISL_2559484, EPI_ISL_2559485, EPI_ISL_2559486	Virology, Evandro Chagas Institute	Virology, Evandro Chagas Institute	Santos,V.M., Ferreira,J.A., Lima,J.F., Barbagelata,L.S., Souza,E.M.A., Goncalves,M.S., SoNorth America / USA,E.C. Jr., Costa,I.B., Santos,M.C., SoNorth America / USA,R.C.M. and Mello,W.A.
EPI_ISL_2559490	Pediatrics, Show-Chwan Memorial Hospital	Pediatrics, Show-Chwan Memorial Hospital	Lee,C.-Y.
EPI_ISL_2559494	Depto Microbiologia, Instituto de Ciencias Biomedicas, Universidade de Sao Paulo	Depto Microbiologia, Instituto de Ciencias Biomedicas, Universidade de Sao Paulo	Moura,F.E.A., Thomazelli,L.M., Campelo,F.S., Delfraro,A., Arbiza,J. and Durigon,E.L.
EPI_ISL_2559517	Centre for Research And Knowledge Transfer in Biotechnology, University of Zagreb	Centre for Research And Knowledge Transfer in Biotechnology, University of Zagreb	Slovic,A., Ivancic-Jelecki,J., Ljubin-Sternak,S., Mlinaric-Galinovic,G., Vilibic-Cavlek,T., Tabain,I. and Forcic,D.
EPI_ISL_2559645, EPI_ISL_2559646	Institute of Virology and AIDS Research, First Hospital of Jilin University	Institute of Virology and AIDS Research, First Hospital of Jilin University	Zheng,Y., Liu,L., Wang,S., Li,Z., Hou,M., Li,J., Yu,X.-F., Zhang,W. and Hua,S.
EPI_ISL_2559647	Centre for Research And Knowledge Transfer in Biotechnology, University of Zagreb	Centre for Research And Knowledge Transfer in Biotechnology, University of Zagreb	Slovic,A., Ivancic-Jelecki,J., Ljubin-Sternak,S., Mlinaric-Galinovic,G., Vilibic-Cavlek,T., Tabain,I. and Forcic,D.
EPI_ISL_2559648, EPI_ISL_2559649, EPI_ISL_2559650, EPI_ISL_2559651, EPI_ISL_2559652, EPI_ISL_2559653, EPI_ISL_2559654, EPI_ISL_2559655	University of Wuerzburg, Institute of Virology and Immunobiology	University of Wuerzburg, Institute of Virology and Immunobiology	Prifert,C., Hofmann,D. and Weissbrich,B.
EPI_ISL_2559656, EPI_ISL_2559657, EPI_ISL_2559658, EPI_ISL_2559659, EPI_ISL_2559660, EPI_ISL_2559661, EPI_ISL_2559662	Centre for Research And Knowledge Transfer in Biotechnology, University of Zagreb	Centre for Research And Knowledge Transfer in Biotechnology, University of Zagreb	Slovic,A., Ivancic-Jelecki,J., Ljubin-Sternak,S., Mlinaric-Galinovic,G., Vilibic-Cavlek,T., Tabain,I. and Forcic,D.
EPI_ISL_2559663, EPI_ISL_2559664, EPI_ISL_2559665, EPI_ISL_2559666, EPI_ISL_2559667, EPI_ISL_2559668	University of Wuerzburg, Institute of Virology and Immunobiology	University of Wuerzburg, Institute of Virology and Immunobiology	Prifert,C., Hofmann,D. and Weissbrich,B.
EPI_ISL_2559669, EPI_ISL_2559670, EPI_ISL_2559671, EPI_ISL_2559672, EPI_ISL_2559673, EPI_ISL_2559674	Department of Infectious Diseases, National Health Institute Doutor Ricardo Jorge	Department of Infectious Diseases, National Health Institute Doutor Ricardo Jorge	Saez-Lopez,E., Cristovao,P., Costa,I., Pechirra,P., Conde,P. and Guiomar,R.
EPI_ISL_2559675, EPI_ISL_2559676	Medicine, American University of Beirut	Medicine, American University of Beirut	Abou-El-Hassan,H. and Zaraket,H.
EPI_ISL_2559677, EPI_ISL_2559678, EPI_ISL_2559679, EPI_ISL_2559680, EPI_ISL_2559681, EPI_ISL_2559682	Centre for Research And Knowledge Transfer in Biotechnology, University of Zagreb	Centre for Research And Knowledge Transfer in Biotechnology, University of Zagreb	Slovic,A., Ivancic-Jelecki,J., Ljubin-Sternak,S., Mlinaric-Galinovic,G., Vilibic-Cavlek,T., Tabain,I. and Forcic,D.
EPI_ISL_2559683, EPI_ISL_2559684, EPI_ISL_2559685, EPI_ISL_2559686, EPI_ISL_2559687, EPI_ISL_2559688	University of Wuerzburg, Institute of Virology and Immunobiology	University of Wuerzburg, Institute of Virology and Immunobiology	Prifert,C., Hofmann,D. and Weissbrich,B.
EPI_ISL_2559689, EPI_ISL_2559690, EPI_ISL_2559691, EPI_ISL_2559692, EPI_ISL_2559693, EPI_ISL_2559694, EPI_ISL_2559695, EPI_ISL_2559696, EPI_ISL_2559697, EPI_ISL_2559698, EPI_ISL_2559699, EPI_ISL_2559700, EPI_ISL_2559701, EPI_ISL_2559702, EPI_ISL_2559703, EPI_ISL_2559704, EPI_ISL_2559710, EPI_ISL_2559711			
see above	Department of Infectious Diseases, National Health Institute Doutor Ricardo Jorge	Department of Infectious Diseases, National Health Institute Doutor Ricardo Jorge	Saez-Lopez,E., Cristovao,P., Costa,I., Pechirra,P., Conde,P. and Guiomar,R.
EPI_ISL_2559721	Virology, Evandro Chagas Institute	Virology, Evandro Chagas Institute	Santos,V.M., Ferreira,J.A., Lima,J.F., Barbagelata,L.S., Souza,E.M.A., Goncalves,M.S., SoNorth America / USA,E.C. Jr., Costa,I.B., Santos,M.C., SoNorth America / USA,R.C.M. and Mello,W.A.
EPI_ISL_2559750, EPI_ISL_2559751	Pediatrics, Mackay Memorial Hospital	Pediatrics, Mackay Memorial Hospital	Chi,H., Hsiao,K.-L., Weng,L.-C., Chiu,N.-C., Huang,L.-M., Chiu,Y.-Y., Liu,C.-P. and Liu,H.-F.
EPI_ISL_2559752	Pediatrics, Show-Chwan Memorial Hospital	Pediatrics, Show-Chwan Memorial Hospital	Lee,C.-Y.
EPI_ISL_2559771, EPI_ISL_2559772, EPI_ISL_2559773, EPI_ISL_2559774, EPI_ISL_2559775	Public Health Laboratory, Minnesota Department of Health	Public Health Laboratory, Minnesota Department of Health	Wang,X., Thielen,B.K. and Lynfield,R.
EPI_ISL_2559815	Pediatrics, Mackay Memorial Hospital	Pediatrics, Mackay Memorial Hospital	Chi,H., Hsiao,K.-L., Weng,L.-C., Chiu,N.-C., Huang,L.-M., Chiu,Y.-Y., Liu,C.-P. and Liu,H.-F.
EPI_ISL_2559816, EPI_ISL_2559817	Pediatrics, Show-Chwan Memorial Hospital	Pediatrics, Show-Chwan Memorial Hospital	Lee,C.-Y.
EPI_ISL_2559846, EPI_ISL_2559847, EPI_ISL_2559848, EPI_ISL_2559849, EPI_ISL_2559850, EPI_ISL_2559851, EPI_ISL_2559852, EPI_ISL_2559853, EPI_ISL_2559854, EPI_ISL_2559855, EPI_ISL_2559856, EPI_ISL_2559857			
see above	Kazuya Shirato National Institute of Infectious Diseases, Virology III	Kazuya Shirato National Institute of Infectious Diseases, Virology III	Shirato,K., Sato,K., Dapat,I., Nao,N., Omiya,S., Matsuyama,S., Takeda,M. and Nishimura,H.
EPI_ISL_2559866, EPI_ISL_2559867, EPI_ISL_2559868, EPI_ISL_2559869, EPI_ISL_2559870, EPI_ISL_2559871, EPI_ISL_2559872, EPI_ISL_2559873, EPI_ISL_2559874, EPI_ISL_2559875, EPI_ISL_2559876, EPI_ISL_2559877, EPI_ISL_2559878, EPI_ISL_2559879			
see above	Virology, Evandro Chagas Institute	Virology, Evandro Chagas Institute	Santos,V.M., Ferreira,J.A., Lima,J.F., Barbagelata,L.S., Souza,E.M.A., Goncalves,M.S., SoNorth America / USA,E.C. Jr., Costa,I.B., Santos,M.C., SoNorth America / USA,R.C.M. and Mello,W.A.
EPI_ISL_2559913	Centre for Interdisciplinary Research in Basic Science	Centre for Interdisciplinary Research in Basic Science	Haider,M.S.H., Naqvi,I.H., Broor,S., Ahmed,A. and Parveen,S.
EPI_ISL_2559925	Depto Microbiologia, Instituto de Ciencias Biomedicas, Universidade de Sao Paulo	Depto Microbiologia, Instituto de Ciencias Biomedicas, Universidade de Sao Paulo	Moura,F.E.A., Thomazelli,L.M., Campelo,F.S., Delfraro,A., Arbiza,J. and Durigon,E.L.
EPI_ISL_2559929	University of Wuerzburg, Institute of Virology and Immunobiology	University of Wuerzburg, Institute of Virology and Immunobiology	Prifert,C., Hofmann,D. and Weissbrich,B.
EPI_ISL_2560178, EPI_ISL_2560179, EPI_ISL_2560180	Institute of Virology and AIDS Research, First Hospital of Jilin University	Institute of Virology and AIDS Research, First Hospital of Jilin University	Zheng,Y., Liu,L., Wang,S., Li,Z., Hou,M., Li,J., Yu,X.-F., Zhang,W. and Hua,S.
EPI_ISL_2560181	Centre for Research And Knowledge Transfer in Biotechnology, University of Zagreb	Centre for Research And Knowledge Transfer in Biotechnology, University of Zagreb	Slovic,A., Ivancic-Jelecki,J., Ljubin-Sternak,S., Mlinaric-Galinovic,G., Vilibic-Cavlek,T., Tabain,I. and Forcic,D.
EPI_ISL_2560182, EPI_ISL_2560183, EPI_ISL_2560184, EPI_ISL_2560185, EPI_ISL_2560186, EPI_ISL_2560187, EPI_ISL_2560188, EPI_ISL_2560189, EPI_ISL_2560190, EPI_ISL_2560191, EPI_ISL_2560192, EPI_ISL_2560193, EPI_ISL_2560194, EPI_ISL_2560195, EPI_ISL_2560196, EPI_ISL_2560197, EPI_ISL_2560198, EPI_ISL_2560199, EPI_ISL_2560200, EPI_ISL_2560201, EPI_ISL_2560202, EPI_ISL_2560203, EPI_ISL_2560204			
see above	University of Wuerzburg, Institute of Virology and Immunobiology	University of Wuerzburg, Institute of Virology and Immunobiology	Prifert,C., Hofmann,D. and Weissbrich,B.
EPI_ISL_2560205, EPI_ISL_2560206, EPI_ISL_2560207, EPI_ISL_2560208, EPI_ISL_2560209	Centre for Research And Knowledge Transfer in Biotechnology, University of Zagreb	Centre for Research And Knowledge Transfer in Biotechnology, University of Zagreb	Slovic,A., Ivancic-Jelecki,J., Ljubin-Sternak,S., Mlinaric-Galinovic,G., Vilibic-Cavlek,T., Tabain,I. and Forcic,D.
EPI_ISL_2560210, EPI_ISL_2560211, EPI_ISL_2560212, EPI_ISL_2560213, EPI_ISL_2560214, EPI_ISL_2560215, EPI_ISL_2560216, EPI_ISL_2560217, EPI_ISL_2560218, EPI_ISL_2560219, EPI_ISL_2560220, EPI_ISL_2560221, EPI_ISL_2560222, EPI_ISL_2560223, EPI_ISL_2560224, EPI_ISL_2560225, EPI_ISL_2560226, EPI_ISL_2560227			
see above	University of Wuerzburg, Institute of Virology and Immunobiology	University of Wuerzburg, Institute of Virology and Immunobiology	Prifert,C., Hofmann,D. and Weissbrich,B.
EPI_ISL_2560228, EPI_ISL_2560229, EPI_ISL_2560230, EPI_ISL_2560231, EPI_ISL_2560232, EPI_ISL_2560233, EPI_ISL_2560234, EPI_ISL_2560235, EPI_ISL_2560236, EPI_ISL_2560237, EPI_ISL_2560238, EPI_ISL_2560239, EPI_ISL_2560240, EPI_ISL_2560241, EPI_ISL_2560242, EPI_ISL_2560243, EPI_ISL_2560244, EPI_ISL_2560245			
see above	Department of Infectious Diseases, National Health Institute Doutor Ricardo Jorge	Department of Infectious Diseases, National Health Institute Doutor Ricardo Jorge	Saez-Lopez,E., Cristovao,P., Costa,I., Pechirra,P., Conde,P. and Guiomar,R.
EPI_ISL_2560246, EPI_ISL_2560247	Biochemistry, King Saud University	Biochemistry, King Saud University	Al Hassinah,S.M., Ahmed,A., Alsenaidy,A.M., Haq,S.H. and Somily,A.M.
EPI_ISL_2560248, EPI_ISL_2560249, EPI_ISL_2560250, EPI_ISL_2560251	Medicine, American University of Beirut	Medicine, American University of Beirut	Abou-El-Hassan,H. and Zaraket,H.
EPI_ISL_2560252, EPI_ISL_2560253, EPI_ISL_2560254, EPI_ISL_2560255, EPI_ISL_2560256, EPI_ISL_2560257, EPI_ISL_2560258, EPI_ISL_2560259, EPI_ISL_2560260, EPI_ISL_2560261, EPI_ISL_2560262, EPI_ISL_2560263, EPI_ISL_2560264, EPI_ISL_2560265, EPI_ISL_2560266, EPI_ISL_2560267, EPI_ISL_2560268			
see above	Centre for Research And Knowledge Transfer in Biotechnology, University of Zagreb	Centre for Research And Knowledge Transfer in Biotechnology, University of Zagreb	Slovic,A., Ivancic-Jelecki,J., Ljubin-Sternak,S., Mlinaric-Galinovic,G., Vilibic-Cavlek,T., Tabain,I. and Forcic,D.
EPI_ISL_2560269, EPI_ISL_2560270, EPI_ISL_2560271, EPI_ISL_2560272, EPI_ISL_2560273, EPI_ISL_2560274, EPI_ISL_2560275, EPI_ISL_2560276, EPI_ISL_2560277, EPI_ISL_2560278, EPI_ISL_2560279, EPI_ISL_2560280, EPI_ISL_2560281, EPI_ISL_2560282, EPI_ISL_2560283, EPI_ISL_2560284			
see above	University of Wuerzburg, Institute of Virology and Immunobiology	University of Wuerzburg, Institute of Virology and Immunobiology	Prifert,C., Hofmann,D. and Weissbrich,B.
EPI_ISL_2560285	Medicine, American University of Beirut	Medicine, American University of Beirut	Abou-El-Hassan,H. and Zaraket,H.
EPI_ISL_2560286, EPI_ISL_2560287, EPI_ISL_2560288, EPI_ISL_2560289, EPI_ISL_2560290, EPI_ISL_2560291, EPI_ISL_2560292, EPI_ISL_2560293, EPI_ISL_2560294, EPI_ISL_2560295, EPI_ISL_2560296, EPI_ISL_2560297, EPI_ISL_2560298, EPI_ISL_2560299, EPI_ISL_2560300, EPI_ISL_2560301, EPI_ISL_2560302, EPI_ISL_2560303, EPI_ISL_2560304, EPI_ISL_2560305, EPI_ISL_2560306, EPI_ISL_2560307, EPI_ISL_2560308, EPI_ISL_2560309, EPI_ISL_2560310, EPI_ISL_2560311, EPI_ISL_2560312, EPI_ISL_2560313, EPI_ISL_2560314, EPI_ISL_2560337, EPI_ISL_2560338, EPI_ISL_2560339, EPI_ISL_2560340, EPI_ISL_2560341, EPI_ISL_2560342			
see above	Department of Infectious Diseases, National Health Institute Doutor Ricardo Jorge	Department of Infectious Diseases, National Health Institute Doutor Ricardo Jorge	Saez-Lopez,E., Cristovao,P., Costa,I., Pechirra,P., Conde,P. and Guiomar,R.
EPI_ISL_2560524, EPI_ISL_2560525, EPI_ISL_2560526, EPI_ISL_2560527	Pediatrics, Mackay Memorial Hospital	Pediatrics, Mackay Memorial Hospital	Chi,H., Hsiao,K.-L., Weng,L.-C., Chiu,N.-C., Huang,L.-M., Chiu,Y.-Y., Liu,C.-P. and Liu,H.-F.

EPI_ISL_2560528	Pediatrics, Show-Chwan Memorial Hospital	Pediatrics, Show-Chwan Memorial Hospital	Lee,C.-Y.
EPI_ISL_2560529	Pediatrics, Mackay Memorial Hospital	Pediatrics, Mackay Memorial Hospital	Chi,H., Hsiao,K.-L., Weng,L.-C., Chiu,N.-C., Huang,L.-M., Chiu,Y.-Y., Liu,C.-P. and Liu,H.-F.
EPI_ISL_2560530, EPI_ISL_2560531, EPI_ISL_2560532	Pediatrics, Show-Chwan Memorial Hospital	Pediatrics, Show-Chwan Memorial Hospital	Lee,C.-Y.
EPI_ISL_2560533	Pediatrics, Mackay Memorial Hospital	Pediatrics, Mackay Memorial Hospital	Chi,H., Hsiao,K.-L., Weng,L.-C., Chiu,N.-C., Huang,L.-M., Chiu,Y.-Y., Liu,C.-P. and Liu,H.-F.
EPI_ISL_2560534, EPI_ISL_2560535	Pediatrics, Show-Chwan Memorial Hospital	Pediatrics, Show-Chwan Memorial Hospital	Lee,C.-Y.
EPI_ISL_2560544, EPI_ISL_2560545, EPI_ISL_2560546	Depto Microbiologia, Instituto de Ciencias Biomedicas, Universidade de Sao Paulo	Depto Microbiologia, Instituto de Ciencias Biomedicas, Universidade de Sao Paulo	Thomazelli,L.M., Oliveira,D.B.L., Colmanetti,T., Vieira,S.E., Paulis,M., Ferronato,A.E., Martinez,M.B. and Durigon,E.L.
EPI_ISL_2560557, EPI_ISL_2560583, EPI_ISL_2560617, EPI_ISL_2560620	Depto Microbiologia, Instituto de Ciencias Biomedicas, Universidade de Sao Paulo	Depto Microbiologia, Instituto de Ciencias Biomedicas, Universidade de Sao Paulo	Moura,F.E.A., Thomazelli,L.M., Campelo,F.S., Delfraro,A., Arbiza,J. and Durigon,E.L.
EPI_ISL_2560682, EPI_ISL_2560683, EPI_ISL_2560684	Pediatrics, Show-Chwan Memorial Hospital	Pediatrics, Show-Chwan Memorial Hospital	Lee,C.-Y.
EPI_ISL_2560685	Pediatrics, Mackay Memorial Hospital	Pediatrics, Mackay Memorial Hospital	Chi,H., Hsiao,K.-L., Weng,L.-C., Chiu,N.-C., Huang,L.-M., Chiu,Y.-Y., Liu,C.-P. and Liu,H.-F.
EPI_ISL_2560686, EPI_ISL_2560687	Pediatrics, Show-Chwan Memorial Hospital	Pediatrics, Show-Chwan Memorial Hospital	Lee,C.-Y.
EPI_ISL_2560688, EPI_ISL_2560689, EPI_ISL_2560690, EPI_ISL_2560691	Pediatrics, Mackay Memorial Hospital	Pediatrics, Mackay Memorial Hospital	Chi,H., Hsiao,K.-L., Weng,L.-C., Chiu,N.-C., Huang,L.-M., Chiu,Y.-Y., Liu,C.-P. and Liu,H.-F.
EPI_ISL_2560692, EPI_ISL_2560693, EPI_ISL_2560694, EPI_ISL_2560695	Pediatrics, Show-Chwan Memorial Hospital	Pediatrics, Show-Chwan Memorial Hospital	Lee,C.-Y.
EPI_ISL_2560696, EPI_ISL_2560697	Pediatrics, Mackay Memorial Hospital	Pediatrics, Mackay Memorial Hospital	Chi,H., Hsiao,K.-L., Weng,L.-C., Chiu,N.-C., Huang,L.-M., Chiu,Y.-Y., Liu,C.-P. and Liu,H.-F.
EPI_ISL_2560698	Pediatrics, Show-Chwan Memorial Hospital	Pediatrics, Show-Chwan Memorial Hospital	Lee,C.-Y.
EPI_ISL_2560801, EPI_ISL_2560802	Kazuya Shirato National Institute of Infectious Diseases, Virology III	Kazuya Shirato National Institute of Infectious Diseases, Virology III	Shirato,K., Sato,K., Dapat,I., Nao,N., Omiya,S., Matsuyama,S., Takeda,M. and Nishimura,H.
EPI_ISL_2560803, EPI_ISL_2560806	Lab Medicine, UW	Lab Medicine, UW	Greninger,A.L., Makhsous,N., Kuypers,J.M., Shean,R.C. and Jerome,K.R.
EPI_ISL_2560807	Kazuya Shirato National Institute of Infectious Diseases, Virology III	Kazuya Shirato National Institute of Infectious Diseases, Virology III	Shirato,K., Sato,K., Dapat,I., Nao,N., Omiya,S., Matsuyama,S., Takeda,M. and Nishimura,H.
EPI_ISL_2560808, EPI_ISL_2560809, EPI_ISL_2560815, EPI_ISL_2560837, EPI_ISL_2560839	Lab Medicine, UW	Lab Medicine, UW	Greninger,A.L., Makhsous,N., Kuypers,J.M., Shean,R.C. and Jerome,K.R.
EPI_ISL_2561282, EPI_ISL_2561283, EPI_ISL_2561284, EPI_ISL_2561285, EPI_ISL_2561286, EPI_ISL_2561287, EPI_ISL_2561288, EPI_ISL_2561289, EPI_ISL_2561290, EPI_ISL_2561291	Virology, Evandro Chagas Institute	Virology, Evandro Chagas Institute	Santos,V.M., Ferreira,J.A., Lima,J.F., Barbagelata,L.S., Souza,E.M.A., Goncalves,M.S., SoNorth America / USA,E.C. Jr., Costa,I.B., Santos,M.C., SoNorth America / USA,R.C.M. and Mello,W.A.
EPI_ISL_2561292	Depto Microbiologia, Instituto de Ciencias Biomedicas, Universidade de Sao Paulo	Depto Microbiologia, Instituto de Ciencias Biomedicas, Universidade de Sao Paulo	Moura,F.E.A., Thomazelli,L.M., Campelo,F.S., Delfraro,A., Arbiza,J. and Durigon,E.L.
EPI_ISL_2561293, EPI_ISL_2561294, EPI_ISL_2561295	Virology, Evandro Chagas Institute	Virology, Evandro Chagas Institute	Santos,V.M., Ferreira,J.A., Lima,J.F., Barbagelata,L.S., Souza,E.M.A., Goncalves,M.S., SoNorth America / USA,E.C. Jr., Costa,I.B., Santos,M.C., SoNorth America / USA,R.C.M. and Mello,W.A.
EPI_ISL_2561296	Depto Microbiologia, Instituto de Ciencias Biomedicas, Universidade de Sao Paulo	Depto Microbiologia, Instituto de Ciencias Biomedicas, Universidade de Sao Paulo	Thomazelli,L.M., Oliveira,D.B.L., Colmanetti,T., Vieira,S.E., Paulis,M., Ferronato,A.E., Martinez,M.B. and Durigon,E.L.
EPI_ISL_2561297, EPI_ISL_2561298	Institute of Virology and AIDS Research, First Hospital of Jilin University	Institute of Virology and AIDS Research, First Hospital of Jilin University	Zheng,Y., Liu,L., Wang,S., Li,Z., Hou,M., Li,J., Yu,X.-F., Zhang,W. and Hua,S.
EPI_ISL_2561299	Centre for Research And Knowledge Transfer in Biotechnology, University of Zagreb	Centre for Research And Knowledge Transfer in Biotechnology, University of Zagreb	Slovic,A., Ivancic-Jelecki,J., Ljubin-Sternak,S., Mlinaric-Galinovic,G., Vilibic-Cavlek,T., Tabain,I. and Forcic,D.
EPI_ISL_2561300, EPI_ISL_2561301, EPI_ISL_2561302, EPI_ISL_2561303, EPI_ISL_2561304, EPI_ISL_2561305, EPI_ISL_2561306, EPI_ISL_2561307, EPI_ISL_2561308, EPI_ISL_2561309, EPI_ISL_2561310, EPI_ISL_2561311, EPI_ISL_2561312, EPI_ISL_2561313, EPI_ISL_2561314, EPI_ISL_2561315, EPI_ISL_2561316, EPI_ISL_2561317, EPI_ISL_2561318, EPI_ISL_2561319, EPI_ISL_2561320, EPI_ISL_2561321, EPI_ISL_2561322, EPI_ISL_2561323, EPI_ISL_2561324, EPI_ISL_2561325, EPI_ISL_2561326, EPI_ISL_2561327, EPI_ISL_2561328, EPI_ISL_2561329			
see above	University of Wuerzburg, Institute of Virology and Immunobiology	University of Wuerzburg, Institute of Virology and Immunobiology	Prifert,C., Hofmann,D. and Weissbrich,B.
EPI_ISL_2561330	Centre for Interdisciplinary Research in Basic Science	Centre for Interdisciplinary Research in Basic Science	Haider,M.S.H., Naqvi,I.H., Broor,S., Ahmed,A. and Parveen,S.
EPI_ISL_2561331, EPI_ISL_2561332, EPI_ISL_2561333	Medicine, American University of Beirut	Medicine, American University of Beirut	Abou-El-Hassan,H. and Zaraket,H.
EPI_ISL_2561334, EPI_ISL_2561335, EPI_ISL_2561336, EPI_ISL_2561337	Pediatrics, Show-Chwan Memorial Hospital	Pediatrics, Show-Chwan Memorial Hospital	Lee,C.-Y.
EPI_ISL_2561338, EPI_ISL_2561339, EPI_ISL_2561340, EPI_ISL_2561341	Pediatrics, Mackay Memorial Hospital	Pediatrics, Mackay Memorial Hospital	Chi,H., Hsiao,K.-L., Weng,L.-C., Chiu,N.-C., Huang,L.-M., Chiu,Y.-Y., Liu,C.-P. and Liu,H.-F.
EPI_ISL_2561342, EPI_ISL_2561343, EPI_ISL_2561344	Epidemiology and Immunogenetic of Viral Infections LR14SP02, University Hospital Sahloul	Epidemiology and Immunogenetic of Viral Infections LR14SP02, University Hospital Sahloul	Ataoui,I., Jerbi,A., BenHamida-Rebai,M., Ben Hadj Fredj,M., Fodha,I., Bennour,H., Hammouda,H., Khlifa,M., Brini,I., Boussoffara,R., Boussetta,K., Abroug,S. and Trabelsi,A.
EPI_ISL_2561345, EPI_ISL_2561346, EPI_ISL_2561347, EPI_ISL_2561348, EPI_ISL_2561349, EPI_ISL_2561350, EPI_ISL_2561351	Centre for Research And Knowledge Transfer in Biotechnology, University of Zagreb	Centre for Research And Knowledge Transfer in Biotechnology, University of Zagreb	Slovic,A., Ivancic-Jelecki,J., Ljubin-Sternak,S., Mlinaric-Galinovic,G., Vilibic-Cavlek,T., Tabain,I. and Forcic,D.
EPI_ISL_2561352, EPI_ISL_2561353, EPI_ISL_2561354, EPI_ISL_2561355, EPI_ISL_2561356, EPI_ISL_2561357, EPI_ISL_2561358, EPI_ISL_2561359, EPI_ISL_2561360, EPI_ISL_2561361, EPI_ISL_2561362, EPI_ISL_2561363, EPI_ISL_2561364, EPI_ISL_2561365, EPI_ISL_2561366, EPI_ISL_2561367, EPI_ISL_2561368, EPI_ISL_2561369, EPI_ISL_2561370, EPI_ISL_2561371, EPI_ISL_2561372, EPI_ISL_2561373, EPI_ISL_2561374			
see above	University of Wuerzburg, Institute of Virology and Immunobiology	University of Wuerzburg, Institute of Virology and Immunobiology	Prifert,C., Hofmann,D. and Weissbrich,B.
EPI_ISL_2561375	Kazuya Shirato National Institute of Infectious Diseases, Virology III	Kazuya Shirato National Institute of Infectious Diseases, Virology III	Shirato,K., Sato,K., Dapat,I., Nao,N., Omiya,S., Matsuyama,S., Takeda,M. and Nishimura,H.
EPI_ISL_2561376, EPI_ISL_2561377, EPI_ISL_2561378, EPI_ISL_2561379, EPI_ISL_2561380, EPI_ISL_2561381	Medicine, American University of Beirut	Medicine, American University of Beirut	Abou-El-Hassan,H. and Zaraket,H.
EPI_ISL_2561382, EPI_ISL_2561383, EPI_ISL_2561384, EPI_ISL_2561385, EPI_ISL_2561386, EPI_ISL_2561387, EPI_ISL_2561388	Department of Infectious Diseases, National Health Institute Doutor Ricardo Jorge	Department of Infectious Diseases, National Health Institute Doutor Ricardo Jorge	Saez-Lopez,E., Cristovao,P., Costa,I., Pechirra,P., Conde,P. and Guiomar,R.
EPI_ISL_2561389, EPI_ISL_2561390, EPI_ISL_2561391	Biochemistry, King Saud University	Biochemistry, King Saud University	Al Hassinah,S.M., Ahmed,A., Alsenaidy,A.M., Haq,S.H. and Somily,A.M.
EPI_ISL_2561392, EPI_ISL_2561393, EPI_ISL_2561394, EPI_ISL_2561395, EPI_ISL_2561396	Pediatrics, Show-Chwan Memorial Hospital	Pediatrics, Show-Chwan Memorial Hospital	Lee,C.-Y.
EPI_ISL_2561397	Pediatrics, Mackay Memorial Hospital	Pediatrics, Mackay Memorial Hospital	Chi,H., Hsiao,K.-L., Weng,L.-C., Chiu,N.-C., Huang,L.-M., Chiu,Y.-Y., Liu,C.-P. and Liu,H.-F.
EPI_ISL_2561398, EPI_ISL_2561399, EPI_ISL_2561400	Pediatrics, Show-Chwan Memorial Hospital	Pediatrics, Show-Chwan Memorial Hospital	Lee,C.-Y.
EPI_ISL_2561401	Lab Medicine, UW	Lab Medicine, UW	Greninger,A.L., Makhsous,N., Kuypers,J.M., Shean,R.C. and Jerome,K.R.
EPI_ISL_2561402	Kazuya Shirato National Institute of Infectious Diseases, Virology III	Kazuya Shirato National Institute of Infectious Diseases, Virology III	Shirato,K., Sato,K., Dapat,I., Nao,N., Omiya,S., Matsuyama,S., Takeda,M. and Nishimura,H.
EPI_ISL_2561403, EPI_ISL_2561404, EPI_ISL_2561405, EPI_ISL_2561406, EPI_ISL_2561407, EPI_ISL_2561408, EPI_ISL_2561409, EPI_ISL_2561410, EPI_ISL_2561411, EPI_ISL_2561412, EPI_ISL_2561413, EPI_ISL_2561414			
see above	Medicine, American University of Beirut	Medicine, American University of Beirut	Abou-El-Hassan,H. and Zaraket,H.
EPI_ISL_2561415, EPI_ISL_2561416, EPI_ISL_2561417, EPI_ISL_2561418, EPI_ISL_2561419, EPI_ISL_2561420, EPI_ISL_2561421, EPI_ISL_2561422, EPI_ISL_2561423	Centre for Research And Knowledge Transfer in Biotechnology, University of Zagreb	Centre for Research And Knowledge Transfer in Biotechnology, University of Zagreb	Slovic,A., Ivancic-Jelecki,J., Ljubin-Sternak,S., Mlinaric-Galinovic,G., Vilibic-Cavlek,T., Tabain,I. and Forcic,D.
EPI_ISL_2561424, EPI_ISL_2561425, EPI_ISL_2561426, EPI_ISL_2561427, EPI_ISL_2561428, EPI_ISL_2561429, EPI_ISL_2561430, EPI_ISL_2561431, EPI_ISL_2561432, EPI_ISL_2561433, EPI_ISL_2561434, EPI_ISL_2561435, EPI_ISL_2561436, EPI_ISL_2561437, EPI_ISL_2561438, EPI_ISL_2561439, EPI_ISL_2561440, EPI_ISL_2561441, EPI_ISL_2561442, EPI_ISL_2561443, EPI_ISL_2561444, EPI_ISL_2561445, EPI_ISL_2561446, EPI_ISL_2561447, EPI_ISL_2561448, EPI_ISL_2561449, EPI_ISL_2561450, EPI_ISL_2561451, EPI_ISL_2561452			
see above	University of Wuerzburg, Institute of Virology and Immunobiology	University of Wuerzburg, Institute of Virology and Immunobiology	Prifert,C., Hofmann,D. and Weissbrich,B.
EPI_ISL_2561453	Maximum Containment Laboratory, National Institue of Virology	Maximum Containment Laboratory, National Institue of Virology	Yadav,P.D.
EPI_ISL_2561454, EPI_ISL_2561455	Kazuya Shirato National Institute of Infectious Diseases, Virology III	Kazuya Shirato National Institute of Infectious Diseases, Virology III	Shirato,K., Sato,K., Dapat,I., Nao,N., Omiya,S., Matsuyama,S., Takeda,M. and Nishimura,H.
EPI_ISL_2561456, EPI_ISL_2561457, EPI_ISL_2561458, EPI_ISL_2561459, EPI_ISL_2561460, EPI_ISL_2561461, EPI_ISL_2561462, EPI_ISL_2561463, EPI_ISL_2561464, EPI_ISL_2561465, EPI_ISL_2561466, EPI_ISL_2561467, EPI_ISL_2561468, EPI_ISL_2561469, EPI_ISL_2561470			
see above	Medicine, American University of Beirut	Medicine, American University of Beirut	Abou-El-Hassan,H. and Zaraket,H.
EPI_ISL_2561471, EPI_ISL_2561472, EPI_ISL_2561473, EPI_ISL_2561474	Department of Infectious Diseases, National Health Institute Doutor Ricardo Jorge	Department of Infectious Diseases, National Health Institute Doutor Ricardo Jorge	Saez-Lopez,E., Cristovao,P., Costa,I., Pechirra,P., Conde,P. and Guiomar,R.
EPI_ISL_2561475, EPI_ISL_2561476, EPI_ISL_2561477	Pediatrics, Show-Chwan Memorial Hospital	Pediatrics, Show-Chwan Memorial Hospital	Lee,C.-Y.
EPI_ISL_2561478, EPI_ISL_2561479, EPI_ISL_2561480, EPI_ISL_2561481, EPI_ISL_2561482, EPI_ISL_2561483, EPI_ISL_2561484, EPI_ISL_2561485, EPI_ISL_2561486, EPI_ISL_2561487, EPI_ISL_2561488, EPI_ISL_2561489, EPI_ISL_2561490, EPI_ISL_2561491, EPI_ISL_2561492, EPI_ISL_2561493, EPI_ISL_2561494, EPI_ISL_2561495, EPI_ISL_2561496, EPI_ISL_2561497, EPI_ISL_2561498, EPI_ISL_2561499, EPI_ISL_2561500			
see above	Medicine, American University of Beirut	Medicine, American University of Beirut	Abou-El-Hassan,H. and Zaraket,H.
EPI_ISL_2561501, EPI_ISL_2561502, EPI_ISL_2561503, EPI_ISL_2561504, EPI_ISL_2561505, EPI_ISL_2561506, EPI_ISL_2561507, EPI_ISL_2561508, EPI_ISL_2561509, EPI_ISL_2561510, EPI_ISL_2561511, EPI_ISL_2561512, EPI_ISL_2561513, EPI_ISL_2561514, EPI_ISL_2561515, EPI_ISL_2561516, EPI_ISL_2561517, EPI_ISL_2561518, EPI_ISL_2561519, EPI_ISL_2561520			

see above	Department of Infectious Diseases, National Health Institute Doutor Ricardo Jorge	Department of Infectious Diseases, National Health Institute Doutor Ricardo Jorge	Saez-Lopez,E., Cristovao,P., Costa,I., Pechirra,P., Conde,P. and Guiomar,R.
EPI_ISL_2561521, EPI_ISL_2561522, EPI_ISL_2561523, EPI_ISL_2561524, EPI_ISL_2561525, EPI_ISL_2561526, EPI_ISL_2561527, EPI_ISL_2561528, EPI_ISL_2561529, EPI_ISL_2561530, EPI_ISL_2561531, EPI_ISL_2561532, EPI_ISL_2561533, EPI_ISL_2561534, EPI_ISL_2561535, EPI_ISL_2561536, EPI_ISL_2561537, EPI_ISL_2561538, EPI_ISL_2561539, EPI_ISL_2561540, EPI_ISL_2561541, EPI_ISL_2561542, EPI_ISL_2561543, EPI_ISL_2561544, EPI_ISL_2561545, EPI_ISL_2561546, EPI_ISL_2561547, EPI_ISL_2561548, EPI_ISL_2561549, EPI_ISL_2561550, EPI_ISL_2561551, EPI_ISL_2561552, EPI_ISL_2561553, EPI_ISL_2561554, EPI_ISL_2561555, EPI_ISL_2561556, EPI_ISL_2561557, EPI_ISL_2561558, EPI_ISL_2561559, EPI_ISL_2561560, EPI_ISL_2561561, EPI_ISL_2561562, EPI_ISL_2561563, EPI_ISL_2561564, EPI_ISL_2561565, EPI_ISL_2561566, EPI_ISL_2561567, EPI_ISL_2561568, EPI_ISL_2561569, EPI_ISL_2561570, EPI_ISL_2561571, EPI_ISL_2561572, EPI_ISL_2561573, EPI_ISL_2561574, EPI_ISL_2561575, EPI_ISL_2561576, EPI_ISL_2561577, EPI_ISL_2561578, EPI_ISL_2561579, EPI_ISL_2561580, EPI_ISL_2561581, EPI_ISL_2561582, EPI_ISL_2561583, EPI_ISL_2561584, EPI_ISL_2561585, EPI_ISL_2561586, EPI_ISL_2561587, EPI_ISL_2561588, EPI_ISL_2561589, EPI_ISL_2561590, EPI_ISL_2561591, EPI_ISL_2561592, EPI_ISL_2561593, EPI_ISL_2561594, EPI_ISL_2561595, EPI_ISL_2561596, EPI_ISL_2561597, EPI_ISL_2561598, EPI_ISL_2561599, EPI_ISL_2561600, EPI_ISL_2561601, EPI_ISL_2561602, EPI_ISL_2561603, EPI_ISL_2561604, EPI_ISL_2561605, EPI_ISL_2561606, EPI_ISL_2561607, EPI_ISL_2561608, EPI_ISL_2561609, EPI_ISL_2561610, EPI_ISL_2561611, EPI_ISL_2561612, EPI_ISL_2561613, EPI_ISL_2561614, EPI_ISL_2561615, EPI_ISL_2561616, EPI_ISL_2561617, EPI_ISL_2561618, EPI_ISL_2561619, EPI_ISL_2561620, EPI_ISL_2561621, EPI_ISL_2561622, EPI_ISL_2561623, EPI_ISL_2561624, EPI_ISL_2561625, EPI_ISL_2561626, EPI_ISL_2561627, EPI_ISL_2561628, EPI_ISL_2561629, EPI_ISL_2561630, EPI_ISL_2561631, EPI_ISL_2561632, EPI_ISL_2561633, EPI_ISL_2561634, EPI_ISL_2561635, EPI_ISL_2561636, EPI_ISL_2561637, EPI_ISL_2561638, EPI_ISL_2561639, EPI_ISL_2561640, EPI_ISL_2561641, EPI_ISL_2561642			
see above	Public Health Laboratory, Minnesota Department of Health	Public Health Laboratory, Minnesota Department of Health	Wang,X., Thielen,B.K. and Lynfield,R.
EPI_ISL_2575417	Kazuya Shirato National Institute of Infectious Diseases, Virology III	Kazuya Shirato National Institute of Infectious Diseases, Virology III	Shirato,K., Sato,K., Dapat,I., Nao,N., Omiya,S., Matsuyama,S., Takeda,M. and Nishimura,H.
EPI_ISL_2575423	J. Craig Venter Institute	J. Craig Venter Institute	Tan,G., Pickett,B., Fedorova,N., Amedeo,P., Hu,L., Christensen,J., Miller,J., Durbin,A., Williams,T., Arumemi,F., Cadiz,C., Alanis,R., Balmseda,A., Wiliams,T., Schiller,A., Patel,M., Kubale,J. and Gordon,A.
EPI_ISL_2575424	Marie Bashir Institute for Infectious Diseases and Biosecurity & Sydney Medical School, The University of Sydney, Westmead Institute for Medical Research	Marie Bashir Institute for Infectious Diseases and Biosecurity & Sydney Medical School, The University of Sydney, Westmead Institute for Medical Research	Eden,J.-S., Kok,J., Dwyer,D.E., Fernandez,M., Carter,I. and Holmes,E.C.
EPI_ISL_2575425	Virus Research Group, Beijing Pediatric Research Institute, Beijing Children's Hospital, Capital Medical University	Virus Research Group, Beijing Pediatric Research Institute, Beijing Children's Hospital, Capital Medical University	Xu,L. and Xie,Z.
EPI_ISL_2575427, EPI_ISL_2575428, EPI_ISL_2575429, EPI_ISL_2575430	J. Craig Venter Institute	J. Craig Venter Institute	Tan,G., Pickett,B., Fedorova,N., Amedeo,P., Hu,L., Christensen,J., Miller,J., Durbin,A., Williams,T., Arumemi,F., Cadiz,C., Alanis,R., Balmseda,A., Wiliams,T., Schiller,A., Patel,M., Kubale,J. and Gordon,A.
EPI_ISL_2575432	Marie Bashir Institute for Infectious Diseases and Biosecurity & Sydney Medical School, The University of Sydney, Westmead Institute for Medical Research	Marie Bashir Institute for Infectious Diseases and Biosecurity & Sydney Medical School, The University of Sydney, Westmead Institute for Medical Research	Eden,J.-S., Kok,J., Dwyer,D.E., Fernandez,M., Carter,I. and Holmes,E.C.
EPI_ISL_2575433, EPI_ISL_2575434	Department of Pediatrics, Center of Excellence in Clinical Virology, Faculty of Medicine, Chulalongkorn University	Department of Pediatrics, Center of Excellence in Clinical Virology, Faculty of Medicine, Chulalongkorn University	Thongpan,I., Mauleekoonphairoj,J., Vichiwattana,P., Korkong,S., Vongpunsawad,S. and Poovorawan,Y.
EPI_ISL_2575462	Epidemiology and Demography, KEMRI-Wellcome Trust Research Programme	Epidemiology and Demography, KEMRI-Wellcome Trust Research Programme	Kamau,E.M., Agoti,C.N., Lewa,C.S., Cane,P.A., Bett,A., Medley,G.F. and Nokes,J.D.
EPI_ISL_2575463	J. Craig Venter Institute	J. Craig Venter Institute	Tan,G., Pickett,B., Fedorova,N., Amedeo,P., Hu,L., Christensen,J., Miller,J., Durbin,A., Williams,T., Arumemi,F., Cadiz,C., Alanis,R., Balmseda,A., Wiliams,T., Schiller,A., Patel,M., Kubale,J. and Gordon,A.
EPI_ISL_2575465	Pediatrics, University of New Mexico	Pediatrics, University of New Mexico	Dinwiddle,D.
EPI_ISL_2575471	Michiko Okamoto Graduate School of Medicine, Tohoku University, Virology	Michiko Okamoto Graduate School of Medicine, Tohoku University, Virology	Okamoto,M. and Oshitani,H.
EPI_ISL_2575473	Microbiology, Hospital Universitari Vall d'Hebron	Microbiology, Hospital Universitari Vall d'Hebron	Gimferrer,L., Martin,Md.C., Pumarola,T. and Anton,A.
EPI_ISL_2575490	Marie Bashir Institute for Infectious Diseases and Biosecurity & Sydney Medical School, The University of Sydney, Westmead Institute for Medical Research	Marie Bashir Institute for Infectious Diseases and Biosecurity & Sydney Medical School, The University of Sydney, Westmead Institute for Medical Research	Eden,J.-S., Kok,J., Dwyer,D.E., Fernandez,M., Carter,I. and Holmes,E.C.
EPI_ISL_2575491	J. Craig Venter Institute	J. Craig Venter Institute	Tan,G., Pickett,B., Fedorova,N., Amedeo,P., Hu,L., Christensen,J., Miller,J., Durbin,A., Williams,T., Arumemi,F., Cadiz,C., Alanis,R., Balmseda,A., Wiliams,T., Schiller,A., Patel,M., Kubale,J. and Gordon,A.
EPI_ISL_2575494, EPI_ISL_2575495	Marie Bashir Institute for Infectious Diseases and Biosecurity & Sydney Medical School, The University of Sydney, Westmead Institute for Medical Research	Marie Bashir Institute for Infectious Diseases and Biosecurity & Sydney Medical School, The University of Sydney, Westmead Institute for Medical Research	Eden,J.-S., Kok,J., Dwyer,D.E., Fernandez,M., Carter,I. and Holmes,E.C.
EPI_ISL_2575496, EPI_ISL_2575497	Pediatrics, University of New Mexico	Pediatrics, University of New Mexico	Dinwiddle,D.
EPI_ISL_2575498	J. Craig Venter Institute	J. Craig Venter Institute	Tan,G., Pickett,B., Fedorova,N., Amedeo,P., Hu,L., Christensen,J., Miller,J., Durbin,A., Williams,T., Arumemi,F., Cadiz,C., Alanis,R., Balmseda,A., Wiliams,T., Schiller,A., Patel,M., Kubale,J. and Gordon,A.
EPI_ISL_2575499	Department of Experimental Modeling and Infectious Diseases Pathogenesis, Federal Research Center of Fundamental and Translational Medicine	Department of Experimental Modeling and Infectious Diseases Pathogenesis, Federal Research Center of Fundamental and Translational Medicine	Kurskaya,O.G., Sobolev,I.A., Sharshov,K.A., Alexeev,A.Y., Murashkina,T.A., Kabilov,M.R., Alikina,T.Y. and Shestopalov,A.M.
EPI_ISL_2575500	Department of Experimental Modeling and Infectious Diseases Pathogenesis, Federal Research Center of Fundamental and Translational Medicine	Department of Experimental Modeling and Infectious Diseases Pathogenesis, Federal Research Center of Fundamental and Translational Medicine	Sobolev,I.A., Kurskaya,O.G., Sharshov,K.A., Alexeev,A.Y., Murashkina,T.A., Kabilov,M.R., Alikina,T.Y. and Shestopalov,A.M.
EPI_ISL_2575506	Marie Bashir Institute for Infectious Diseases and Biosecurity & Sydney Medical School, The University of Sydney, Westmead Institute for Medical Research	Marie Bashir Institute for Infectious Diseases and Biosecurity & Sydney Medical School, The University of Sydney, Westmead Institute for Medical Research	Eden,J.-S., Kok,J., Dwyer,D.E., Fernandez,M., Carter,I. and Holmes,E.C.
EPI_ISL_2575507	J. Craig Venter Institute	J. Craig Venter Institute	Tan,G., Pickett,B., Fedorova,N., Amedeo,P., Hu,L., Christensen,J., Miller,J., Durbin,A., Williams,T., Arumemi,F., Cadiz,C., Alanis,R., Balmseda,A., Wiliams,T., Schiller,A., Patel,M., Kubale,J. and Gordon,A.
EPI_ISL_2575508, EPI_ISL_2575509, EPI_ISL_2575510, EPI_ISL_2575511	Marie Bashir Institute for Infectious Diseases and Biosecurity & Sydney Medical School, The University of Sydney, Westmead Institute for Medical Research	Marie Bashir Institute for Infectious Diseases and Biosecurity & Sydney Medical School, The University of Sydney, Westmead Institute for Medical Research	Eden,J.-S., Kok,J., Dwyer,D.E., Fernandez,M., Carter,I. and Holmes,E.C.
EPI_ISL_2575512	Michiko Okamoto Graduate School of Medicine, Tohoku University, Virology	Michiko Okamoto Graduate School of Medicine, Tohoku University, Virology	Okamoto,M. and Oshitani,H.
EPI_ISL_2575518	Pediatrics, University of New Mexico	Pediatrics, University of New Mexico	Dinwiddle,D.
EPI_ISL_2575520, EPI_ISL_2575521	Virology Section, Department of Microbiology, King George's Medical University	Virology Section, Department of Microbiology, King George's Medical University	Singh,A.K., Jain,A., Jain,B., Dangi,T., Verma,A.K., Dwivedi,M. and Kushwaha,R.
EPI_ISL_2575522, EPI_ISL_2575523, EPI_ISL_2575524	Pediatrics, University of New Mexico	Pediatrics, University of New Mexico	Dinwiddle,D.
EPI_ISL_2575536	Marie Bashir Institute for Infectious Diseases and Biosecurity & Sydney Medical School, The University of Sydney, Westmead Institute for Medical Research	Marie Bashir Institute for Infectious Diseases and Biosecurity & Sydney Medical School, The University of Sydney, Westmead Institute for Medical Research	Eden,J.-S., Kok,J., Dwyer,D.E., Fernandez,M., Carter,I. and Holmes,E.C.
EPI_ISL_2575537	Virology, King Institute of Preventive Medicine and Research	Virology, King Institute of Preventive Medicine and Research	SureshBabu B,V., Gunasekaran,P., Kaveri,K., Venkataraman,P., Magesh,S., Kiruba,R., Mohana,S. and SenthilRaja,R.
EPI_ISL_2575538	Marie Bashir Institute for Infectious Diseases and Biosecurity & Sydney Medical School, The University of Sydney, Westmead Institute for Medical Research	Marie Bashir Institute for Infectious Diseases and Biosecurity & Sydney Medical School, The University of Sydney, Westmead Institute for Medical Research	Eden,J.-S., Kok,J., Dwyer,D.E., Fernandez,M., Carter,I. and Holmes,E.C.
EPI_ISL_2575539, EPI_ISL_2575540	Virology, King Institute of Preventive Medicine and Research	Virology, King Institute of Preventive Medicine and Research	SureshBabu B,V., Gunasekaran,P., Kaveri,K., Venkataraman,P., Magesh,S., Kiruba,R., Mohana,S. and SenthilRaja,R.
EPI_ISL_2575542	Epidemiology and Demography, KEMRI-Wellcome Trust Research Programme	Epidemiology and Demography, KEMRI-Wellcome Trust Research Programme	Kamau,E.M., Agoti,C.N., Lewa,C.S., Cane,P.A., Bett,A., Medley,G.F. and Nokes,J.D.
EPI_ISL_2575546, EPI_ISL_2575547	Department of Experimental Modeling and Infectious Diseases Pathogenesis, Federal Research Center of Fundamental and Translational Medicine	Department of Experimental Modeling and Infectious Diseases Pathogenesis, Federal Research Center of Fundamental and Translational Medicine	Kurskaya,O.G., Sobolev,I.A., Sharshov,K.A., Alexeev,A.Y., Murashkina,T.A., Kabilov,M.R., Alikina,T.Y. and Shestopalov,A.M.
EPI_ISL_2575548	J. Craig Venter Institute	J. Craig Venter Institute	Tan,G., Pickett,B., Fedorova,N., Amedeo,P., Hu,L., Christensen,J., Miller,J., Durbin,A., Williams,T., Arumemi,F., Cadiz,C., Alanis,R., Balmseda,A., Wiliams,T., Schiller,A., Patel,M., Kubale,J. and Gordon,A.
EPI_ISL_2575549	Marie Bashir Institute for Infectious Diseases and Biosecurity & Sydney Medical School, The University of Sydney, Westmead Institute for Medical Research	Marie Bashir Institute for Infectious Diseases and Biosecurity & Sydney Medical School, The University of Sydney, Westmead Institute for Medical Research	Eden,J.-S., Kok,J., Dwyer,D.E., Fernandez,M., Carter,I. and Holmes,E.C.
EPI_ISL_2575567, EPI_ISL_2575570, EPI_ISL_2575580, EPI_ISL_2575581, EPI_ISL_2575582, EPI_ISL_2575583, EPI_ISL_2575584, EPI_ISL_2575585, EPI_ISL_2575586, EPI_ISL_2575587	J. Craig Venter Institute	J. Craig Venter Institute	Tan,G., Pickett,B., Fedorova,N., Amedeo,P., Hu,L., Christensen,J., Miller,J., Durbin,A., Williams,T., Arumemi,F., Cadiz,C., Alanis,R., Balmseda,A., Wiliams,T., Schiller,A., Patel,M., Kubale,J. and Gordon,A.
EPI_ISL_2575588, EPI_ISL_2575589, EPI_ISL_2575590	Marie Bashir Institute for Infectious Diseases and Biosecurity & Sydney Medical School, The University of Sydney, Westmead Institute for Medical Research	Marie Bashir Institute for Infectious Diseases and Biosecurity & Sydney Medical School, The University of Sydney, Westmead Institute for Medical Research	Eden,J.-S., Kok,J., Dwyer,D.E., Fernandez,M., Carter,I. and Holmes,E.C.
EPI_ISL_2575593	Kazuya Shirato National Institute of Infectious Diseases, Virology III	Kazuya Shirato National Institute of Infectious Diseases, Virology III	Shirato,K., Sato,K., Dapat,I., Nao,N., Omiya,S., Matsuyama,S., Takeda,M. and Nishimura,H.
EPI_ISL_2575594, EPI_ISL_2575595, EPI_ISL_2575596	J. Craig Venter Institute	J. Craig Venter Institute	Tan,G., Pickett,B., Fedorova,N., Amedeo,P., Hu,L., Christensen,J., Miller,J., Durbin,A., Williams,T., Arumemi,F., Cadiz,C., Alanis,R., Balmseda,A., Wiliams,T., Schiller,A., Patel,M., Kubale,J. and Gordon,A.
EPI_ISL_2575597, EPI_ISL_2575598, EPI_ISL_2575599, EPI_ISL_2575600, EPI_ISL_2575602, EPI_ISL_2575603, EPI_ISL_2575604, EPI_ISL_2575605, EPI_ISL_2575607, EPI_ISL_2575608, EPI_ISL_2575609, EPI_ISL_2575610, EPI_ISL_2575611, EPI_ISL_2575612, EPI_ISL_2575613, EPI_ISL_2575614			
see above	Marie Bashir Institute for Infectious Diseases and Biosecurity & Sydney Medical School, The University of Sydney, Westmead Institute for Medical Research	Marie Bashir Institute for Infectious Diseases and Biosecurity & Sydney Medical School, The University of Sydney, Westmead Institute for Medical Research	Eden,J.-S., Kok,J., Dwyer,D.E., Fernandez,M., Carter,I. and Holmes,E.C.
EPI_ISL_2575618, EPI_ISL_2575619, EPI_ISL_2575620	J. Craig Venter Institute	J. Craig Venter Institute	Tan,G., Pickett,B., Fedorova,N., Amedeo,P., Hu,L., Christensen,J., Miller,J., Durbin,A., Williams,T., Arumemi,F., Cadiz,C., Alanis,R., Balmseda,A., Wiliams,T., Schiller,A., Patel,M., Kubale,J. and Gordon,A.

EPI_ISL_2575621, EPI_ISL_2575623	Marie Bashir Institute for Infectious Diseases and Biosecurity & Sydney Medical School, The University of Sydney, Westmead Institute for Medical Research	Marie Bashir Institute for Infectious Diseases and Biosecurity & Sydney Medical School, The University of Sydney, Westmead Institute for Medical Research	Eden,J.-S., Kok,J., Dwyer,D.E., Fernandez,M., Carter,I. and Holmes,E.C.
EPI_ISL_2575652, EPI_ISL_2575653, EPI_ISL_2575654	Epidemiology and Demography, KEMRI-Wellcome Trust Research Programme	Epidemiology and Demography, KEMRI-Wellcome Trust Research Programme	Kamau,E.M., Agoti,C.N., Lewa,C.S., Cane,P.A., Nokes,J.D. and Agoti,C.N.
EPI_ISL_2575680	Wint Wint Phyu Niigat University, International Health, Public Health	Wint Wint Phyu Niigat University, International Health, Public Health	Phyu,W.W. and Saito,R.
EPI_ISL_2575682	Akinobu Hibino Niigata University, International Health, Public Health	Akinobu Hibino Niigata University, International Health, Public Health	Hibino,A., Saito,R., Shoubugawa,Y., Makiya,T. and Takefuta,K.
EPI_ISL_2575686	Akinobu Hibino Niigata University, International Health, Public Health	Akinobu Hibino Niigata University, International Health, Public Health	Hibino,A., Saito,R., Shoubugawa,Y. and Tomimoto,K.
EPI_ISL_2575687	WHO WPRO Measles Regional Reference Lab, Key Laboratory of Medical Virology Ministry of Health, National Institute for Viral Disease Control and Prevention, China Center for Disease Control and Prevention	WHO WPRO Measles Regional Reference Lab, Key Laboratory of Medical Virology Ministry of Health, National Institute for Viral Disease Control and Prevention, China Center for Disease Control and Prevention	Song,J., Zhang,Y. and Xu,W.
EPI_ISL_2575690	Akinobu Hibino Niigata University, International Health, Public Health	Akinobu Hibino Niigata University, International Health, Public Health	Hibino,A., Saito,R., Shoubugawa,Y. and Tomimoto,K.
EPI_ISL_2575691	Akinobu Hibino Niigata University, International Health, Public Health	Akinobu Hibino Niigata University, International Health, Public Health	Hibino,A., Saito,R., Shoubugawa,Y. and Nagata,N.
EPI_ISL_2575704, EPI_ISL_2575705	Microbiology, Hospital Universitari Vall d'Hebron	Microbiology, Hospital Universitari Vall d'Hebron	Gimferrer,L., Martin,Md.C., Pumarola,T. and Anton,A.
EPI_ISL_2575711	WHO WPRO Measles Regional Reference Lab, Key Laboratory of Medical Virology Ministry of Health, National Institute for Viral Disease Control and Prevention, China Center for Disease Control and Prevention	WHO WPRO Measles Regional Reference Lab, Key Laboratory of Medical Virology Ministry of Health, National Institute for Viral Disease Control and Prevention, China Center for Disease Control and Prevention	Song,J., Zhang,Y. and Xu,W.
EPI_ISL_2575712	Akinobu Hibino Niigata University, International Health, Public Health	Akinobu Hibino Niigata University, International Health, Public Health	Hibino,A., Saito,R., Shoubugawa,Y. and Kaji,H.
EPI_ISL_2575733, EPI_ISL_2575734, EPI_ISL_2575735, EPI_ISL_2575736, EPI_ISL_2575737, EPI_ISL_2575738, EPI_ISL_2575739	Microbiology, Hospital Universitari Vall d'Hebron	Microbiology, Hospital Universitari Vall d'Hebron	Gimferrer,L., Martin,Md.C., Pumarola,T. and Anton,A.
EPI_ISL_2575746	WHO WPRO Measles Regional Reference Lab, Key Laboratory of Medical Virology Ministry of Health, National Institute for Viral Disease Control and Prevention, China Center for Disease Control and Prevention	WHO WPRO Measles Regional Reference Lab, Key Laboratory of Medical Virology Ministry of Health, National Institute for Viral Disease Control and Prevention, China Center for Disease Control and Prevention	Song,J., Zhang,Y. and Xu,W.
EPI_ISL_2575747	Virology, University of Ibadan, College of Medicine (UCH)	Virology, University of Ibadan, College of Medicine (UCH)	Ogunsemowo,O.S., Odaibo,G.N. and Olaleye,D.O.
EPI_ISL_2575748	Department of Pediatrics, Center of Excellence in Clinical Virology, Chulalongkorn	Department of Pediatrics, Center of Excellence in Clinical Virology, Chulalongkorn	Thongpan,I.
EPI_ISL_2575749, EPI_ISL_2575750	Wint Wint Phyu Niigat University, International Health, Public Health	Wint Wint Phyu Niigat University, International Health, Public Health	Phyu,W.W. and Saito,R.
EPI_ISL_2575768, EPI_ISL_2575769, EPI_ISL_2575770, EPI_ISL_2575771, EPI_ISL_2575772, EPI_ISL_2575773, EPI_ISL_2575774, EPI_ISL_2575775	Epidemiology and Demography, KEMRI-Wellcome Trust Research Programme	Epidemiology and Demography, KEMRI-Wellcome Trust Research Programme	Kamau,E.M., Agoti,C.N., Lewa,C.S., Cane,P.A., Bett,A., Medley,G.F. and Nokes,J.D.
EPI_ISL_2575805	Epidemiology and Demography, KEMRI-Wellcome Trust Research Programme	Epidemiology and Demography, KEMRI-Wellcome Trust Research Programme	Kamau,E.M., Agoti,C.N., Lewa,C.S., Cane,P.A., Nokes,J.D. and Agoti,C.N.
EPI_ISL_2575813	Epidemiology and Demography, KEMRI-Wellcome Trust Research Programme	Epidemiology and Demography, KEMRI-Wellcome Trust Research Programme	Kamau,E.M., Agoti,C.N., Owor,B., Lewa,C., Bett,A., Oketch,J., Cane,P.A. and Nokes,J.D.
EPI_ISL_2575828	Bacteriology & Virology, University of Medical Sciences, Shiraz Medical School	Bacteriology & Virology, University of Medical Sciences, Shiraz Medical School	Mojarrad,S., Pirbonyeh,N., Moattari,A. and Babaei,A.
EPI_ISL_2575844, EPI_ISL_2575845, EPI_ISL_2575848, EPI_ISL_2575849, EPI_ISL_2575850, EPI_ISL_2575851, EPI_ISL_2575852, EPI_ISL_2575853, EPI_ISL_2575854, EPI_ISL_2575855, EPI_ISL_2575856, EPI_ISL_2575857, EPI_ISL_2575858, EPI_ISL_2575859, EPI_ISL_2575860, EPI_ISL_2575861, EPI_ISL_2575862, EPI_ISL_2575863			
see above	Microbiology, Hospital Universitari Vall d'Hebron	Microbiology, Hospital Universitari Vall d'Hebron	Gimferrer,L., Martin,Md.C., Pumarola,T. and Anton,A.
EPI_ISL_2575903, EPI_ISL_2575904, EPI_ISL_2575905	Epidemiology and Demography, KEMRI-Wellcome Trust Research Programme	Epidemiology and Demography, KEMRI-Wellcome Trust Research Programme	Kamau,E.M., Agoti,C.N., Lewa,C.S., Cane,P.A., Bett,A., Medley,G.F. and Nokes,J.D.
EPI_ISL_2575906, EPI_ISL_2575907, EPI_ISL_2575908, EPI_ISL_2575909	Epidemiology and Demography, KEMRI-Wellcome Trust Research Programme	Epidemiology and Demography, KEMRI-Wellcome Trust Research Programme	Kamau,E.M., Agoti,C.N., Lewa,C.S., Cane,P.A., Nokes,J.D. and Agoti,C.N.
EPI_ISL_2575934, EPI_ISL_2575935, EPI_ISL_2575936, EPI_ISL_2575937, EPI_ISL_2575938	WHO WPRO Measles Regional Reference Lab, Key Laboratory of Medical Virology Ministry of Health, National Institute for Viral Disease Control and Prevention, China Center for Disease Control and Prevention	WHO WPRO Measles Regional Reference Lab, Key Laboratory of Medical Virology Ministry of Health, National Institute for Viral Disease Control and Prevention, China Center for Disease Control and Prevention	Song,J., Zhang,Y. and Xu,W.
EPI_ISL_2575939	Department of Public Health Laboratory Sciences, West China School of Public Health, Sichuan University	Department of Public Health Laboratory Sciences, West China School of Public Health, Sichuan University	Hu,W.P. and Pei,F.X.
EPI_ISL_2575940, EPI_ISL_2575941, EPI_ISL_2575942, EPI_ISL_2575943, EPI_ISL_2575944	WHO WPRO Measles Regional Reference Lab, Key Laboratory of Medical Virology Ministry of Health, National Institute for Viral Disease Control and Prevention, China Center for Disease Control and Prevention	WHO WPRO Measles Regional Reference Lab, Key Laboratory of Medical Virology Ministry of Health, National Institute for Viral Disease Control and Prevention, China Center for Disease Control and Prevention	Song,J., Zhang,Y. and Xu,W.
EPI_ISL_2575945	Akinobu Hibino Niigata University, International Health, Public Health	Akinobu Hibino Niigata University, International Health, Public Health	Hibino,A., Saito,R., Shoubugawa,Y. and Kaji,H.
EPI_ISL_2575946	Virology, University of Ibadan, College of Medicine (UCH)	Virology, University of Ibadan, College of Medicine (UCH)	Ogunsemowo,O.S., Odaibo,G.N. and Olaleye,D.O.
EPI_ISL_2575947, EPI_ISL_2575948, EPI_ISL_2575949	Department of Pediatrics, Center of Excellence in Clinical Virology, Chulalongkorn	Department of Pediatrics, Center of Excellence in Clinical Virology, Chulalongkorn	Thongpan,I.
EPI_ISL_2575950	Wint Wint Phyu Niigat University, International Health, Public Health	Wint Wint Phyu Niigat University, International Health, Public Health	Phyu,W.W. and Saito,R.
EPI_ISL_2575951, EPI_ISL_2575952, EPI_ISL_2575953, EPI_ISL_2575954, EPI_ISL_2575955, EPI_ISL_2575956	Department of Pediatrics, Center of Excellence in Clinical Virology, Chulalongkorn	Department of Pediatrics, Center of Excellence in Clinical Virology, Chulalongkorn	Thongpan,I.
EPI_ISL_2575969, EPI_ISL_2575970	Wint Wint Phyu Niigat University, International Health, Public Health	Wint Wint Phyu Niigat University, International Health, Public Health	Phyu,W.W. and Saito,R.
EPI_ISL_2575975	Medical Virology, Faculty of Public Health, Tehran University of Medical Sciences	Medical Virology, Faculty of Public Health, Tehran University of Medical Sciences	Arjeyni,Y., Faghihloo,E. and Mokhtari Azad,T.
EPI_ISL_2575995, EPI_ISL_2575996, EPI_ISL_2575997, EPI_ISL_2575998	Virology, University of Ibadan, College of Medicine (UCH)	Virology, University of Ibadan, College of Medicine (UCH)	Ogunsemowo,O.S., Odaibo,G.N. and Olaleye,D.O.
EPI_ISL_2576000, EPI_ISL_2576001	Centre for Infectious Diseases Virology, University of Heidelberg	Centre for Infectious Diseases Virology, University of Heidelberg	Ihling,C.M., Schnitzler,P., Heinrich,N., Mangu,C., Sudi,L., Souares,A., Gies,S., Sie,A., Coulibaly,B., Ouedraogo,A.T., Mordmueller,B., Held,J., Adegnika,A.A., Fernandez,J.F., Eckerle,I., May,J., Hogan,B., Eibach,D., Owusu-Dabo,E. and Tabatabai,J.
EPI_ISL_2576050	Virology Laboratory, Dr. Ricardo Gutierrez Children Hospital	Virology Laboratory, Dr. Ricardo Gutierrez Children Hospital	Rojo,G.L., Goya,S., Orellana,M., Sancilio,A., Rodriguez Perez,A., Montali,C., Garcia,C., Sosa,L., Musto,A., Hamilton,G., Alvarez,D., Castello,A. and Viegas,M.
EPI_ISL_2576057	Bacteriology & Virology, University of Medical Sciences, Shiraz Medical School	Bacteriology & Virology, University of Medical Sciences, Shiraz Medical School	Mojarrad,S., Pirbonyeh,N., Moattari,A. and Babaei,A.
EPI_ISL_2576076, EPI_ISL_2576077	Epidemiology and Demography, KEMRI-Wellcome Trust Research Programme	Epidemiology and Demography, KEMRI-Wellcome Trust Research Programme	Kamau,E.M., Agoti,C.N., Lewa,C.S., Cane,P.A., Bett,A., Medley,G.F. and Nokes,J.D.
EPI_ISL_2576078, EPI_ISL_2576079, EPI_ISL_2576080	Epidemiology and Demography, KEMRI-Wellcome Trust Research Programme	Epidemiology and Demography, KEMRI-Wellcome Trust Research Programme	Kamau,E.M., Agoti,C.N., Lewa,C.S., Cane,P.A., Nokes,J.D. and Agoti,C.N.
EPI_ISL_2576103	Bacteriology & Virology, University of Medical Sciences, Shiraz Medical School	Bacteriology & Virology, University of Medical Sciences, Shiraz Medical School	Mojarrad,S., Pirbonyeh,N., Moattari,A. and Babaei,A.
EPI_ISL_2576180, EPI_ISL_2576182, EPI_ISL_2576183	Virology Laboratory, Dr. Ricardo Gutierrez Children Hospital	Virology Laboratory, Dr. Ricardo Gutierrez Children Hospital	Rojo,G.L., Goya,S., Orellana,M., Sancilio,A., Rodriguez Perez,A., Montali,C., Garcia,C., Sosa,L., Musto,A., Hamilton,G., Alvarez,D., Castello,A. and Viegas,M.
EPI_ISL_2576185, EPI_ISL_2576186	Virology, University of Ibadan, College of Medicine (UCH)	Virology, University of Ibadan, College of Medicine (UCH)	Ogunsemowo,O.S., Odaibo,G.N. and Olaleye,D.O.
EPI_ISL_2576188, EPI_ISL_2576189, EPI_ISL_2576190, EPI_ISL_2576191	Department of Clinical Laboratory, Fujian Provincial Hospital	Department of Clinical Laboratory, Fujian Provincial Hospital	Su,Y., Wu,Y., Tian,R. and Liang,G.
EPI_ISL_2576192	Michiko Okamoto Tohoku University Graduate School of Medicine, Virology	Michiko Okamoto Tohoku University Graduate School of Medicine, Virology	Okamoto,M. and Dapat,C.
EPI_ISL_2576198, EPI_ISL_2576199, EPI_ISL_2576203, EPI_ISL_2576204, EPI_ISL_2576205, EPI_ISL_2576206, EPI_ISL_2576207, EPI_ISL_2576208, EPI_ISL_2576209, EPI_ISL_2576210, EPI_ISL_2576211, EPI_ISL_2576212, EPI_ISL_2576213, EPI_ISL_2576214, EPI_ISL_2576215, EPI_ISL_2576216			
see above	Epidemiology and Demography, KEMRI-Wellcome Trust Research Programme	Epidemiology and Demography, KEMRI-Wellcome Trust Research Programme	Kamau,E.M., Agoti,C.N., Lewa,C.S., Cane,P.A., Bett,A., Medley,G.F. and Nokes,J.D.
EPI_ISL_2576217, EPI_ISL_2576218, EPI_ISL_2576219, EPI_ISL_2576220, EPI_ISL_2576221	Epidemiology and Demography, KEMRI-Wellcome Trust Research Programme	Epidemiology and Demography, KEMRI-Wellcome Trust Research Programme	Kamau,E.M., Agoti,C.N., Lewa,C.S., Cane,P.A., Nokes,J.D. and Agoti,C.N.
EPI_ISL_2576228	Michiko Okamoto Tohoku University Graduate School of Medicine, Virology	Michiko Okamoto Tohoku University Graduate School of Medicine, Virology	Okamoto,M. and Dapat,C.
EPI_ISL_2576229	Epidemiology and Demography, KEMRI-Wellcome Trust Research Programme	Epidemiology and Demography, KEMRI-Wellcome Trust Research Programme	Kamau,E.M., Agoti,C.N., Owor,B., Lewa,C., Bett,A., Oketch,J., Cane,P.A. and Nokes,J.D.
EPI_ISL_2576239, EPI_ISL_2576240, EPI_ISL_2576241, EPI_ISL_2576242, EPI_ISL_2576243, EPI_ISL_2576244, EPI_ISL_2576245, EPI_ISL_2576250, EPI_ISL_2576251, EPI_ISL_2576252, EPI_ISL_2576253, EPI_ISL_2576254, EPI_ISL_2576255, EPI_ISL_2576256, EPI_ISL_2576257, EPI_ISL_2576258, EPI_ISL_2576259, EPI_ISL_2576260, EPI_ISL_2576261, EPI_ISL_2576262, EPI_ISL_2576263, EPI_ISL_2576264, EPI_ISL_2576265, EPI_ISL_2576266, EPI_ISL_2576267, EPI_ISL_2576268, EPI_ISL_2576269, EPI_ISL_2576270, EPI_ISL_2576271, EPI_ISL_2576272, EPI_ISL_2576273, EPI_ISL_2576274, EPI_ISL_2576275, EPI_ISL_2576276, EPI_ISL_2576277, EPI_ISL_2576278, EPI_ISL_2576279, EPI_ISL_2576280, EPI_ISL_2576281, EPI_ISL_2576282, EPI_ISL_2576283, EPI_ISL_2576284, EPI_ISL_2576285, EPI_ISL_2576286, EPI_ISL_2576287, EPI_ISL_2576288, EPI_ISL_2576289, EPI_ISL_2576290, EPI_ISL_2576291			
see above	Microbiology, Hospital Universitari Vall d'Hebron	Microbiology, Hospital Universitari Vall d'Hebron	Gimferrer,L., Martin,Md.C., Pumarola,T. and Anton,A.
EPI_ISL_2576292	Michiko Okamoto Tohoku University Graduate School of Medicine, Virology	Michiko Okamoto Tohoku University Graduate School of Medicine, Virology	Okamoto,M. and Dapat,C.
EPI_ISL_2576295	Department of Pediatrics, Center of Excellence in Clinical Virology, Faculty of Medicine, Chulalongkorn University	Department of Pediatrics, Center of Excellence in Clinical Virology, Faculty of Medicine, Chulalongkorn University	Thongpan,I., Mauleekoonphairoj,J., Vichiwattana,P., Korkong,S., Vongpunsawad,S. and Poovorawan,Y.
EPI_ISL_2576297, EPI_ISL_2576359, EPI_ISL_2576360, EPI_ISL_2576361, EPI_ISL_2576362, EPI_ISL_2576363, EPI_ISL_2576364, EPI_ISL_2576365	WHO WPRO Measles Regional Reference Lab, Key Laboratory of Medical Virology Ministry of Health, National Institute for Viral Disease Control and Prevention, China Center for Disease Control and Prevention	WHO WPRO Measles Regional Reference Lab, Key Laboratory of Medical Virology Ministry of Health, National Institute for Viral Disease Control and Prevention, China Center for Disease Control and Prevention	Song,J., Zhang,Y. and Xu,W.

EPI_ISL_2576366, EPI_ISL_2576367	Department of Public Health Laboratory Sciences, West China School of Public Health, Sichuan University	Department of Public Health Laboratory Sciences, West China School of Public Health, Sichuan University	Hu,W.P. and Pei,F.X.
EPI_ISL_2576368, EPI_ISL_2576369, EPI_ISL_2576370, EPI_ISL_2576371, EPI_ISL_2576372, EPI_ISL_2576373, EPI_ISL_2576374, EPI_ISL_2576375, EPI_ISL_2576376, EPI_ISL_2576377	WHO WPRO Measles Regional Reference Lab, Key Laboratory of Medical Virology Ministry of Health, National Institute for Viral Disease Control and Prevention, China Center for Disease Control and Prevention	WHO WPRO Measles Regional Reference Lab, Key Laboratory of Medical Virology Ministry of Health, National Institute for Viral Disease Control and Prevention, China Center for Disease Control and Prevention	Song,J., Zhang,Y. and Xu,W.
EPI_ISL_2576378	Department of Public Health Laboratory Sciences, West China School of Public Health, Sichuan University	Department of Public Health Laboratory Sciences, West China School of Public Health, Sichuan University	Hu,W.P. and Pei,F.X.
EPI_ISL_2576379, EPI_ISL_2576380	WHO WPRO Measles Regional Reference Lab, Key Laboratory of Medical Virology Ministry of Health, National Institute for Viral Disease Control and Prevention, China Center for Disease Control and Prevention	WHO WPRO Measles Regional Reference Lab, Key Laboratory of Medical Virology Ministry of Health, National Institute for Viral Disease Control and Prevention, China Center for Disease Control and Prevention	Song,J., Zhang,Y. and Xu,W.
EPI_ISL_2576381	Department of Public Health Laboratory Sciences, West China School of Public Health, Sichuan University	Department of Public Health Laboratory Sciences, West China School of Public Health, Sichuan University	Hu,W.P. and Pei,F.X.
EPI_ISL_2576382, EPI_ISL_2576383, EPI_ISL_2576384, EPI_ISL_2576385, EPI_ISL_2576386, EPI_ISL_2576387	WHO WPRO Measles Regional Reference Lab, Key Laboratory of Medical Virology Ministry of Health, National Institute for Viral Disease Control and Prevention, China Center for Disease Control and Prevention	WHO WPRO Measles Regional Reference Lab, Key Laboratory of Medical Virology Ministry of Health, National Institute for Viral Disease Control and Prevention, China Center for Disease Control and Prevention	Song,J., Zhang,Y. and Xu,W.
EPI_ISL_2576388, EPI_ISL_2576389, EPI_ISL_2576390, EPI_ISL_2576391, EPI_ISL_2576392, EPI_ISL_2576393	Department of Pediatrics, Center of Excellence in Clinical Virology, Faculty of Medicine, Chulalongkorn University	Department of Pediatrics, Center of Excellence in Clinical Virology, Faculty of Medicine, Chulalongkorn University	Thongpan,I., Mauleekoonphairoj,J., Vichiwattana,P., Korkong,S., Vongpunsawad,S. and Poovorawan,Y.
EPI_ISL_2576394	Akinobu Hibino Niigata University, International Health, Public Health	Akinobu Hibino Niigata University, International Health, Public Health	Hibino,A., Saito,R., Shoubugawa,Y. and Nagata,N.
EPI_ISL_2576395, EPI_ISL_2576396	Akinobu Hibino Niigata University, International Health, Public Health	Akinobu Hibino Niigata University, International Health, Public Health	Hibino,A., Saito,R., Shoubugawa,Y., Azhar,S. and Rohaizat,H.
EPI_ISL_2576397, EPI_ISL_2576398, EPI_ISL_2576399	Department of Pediatrics, Center of Excellence in Clinical Virology, Faculty of Medicine, Chulalongkorn University	Department of Pediatrics, Center of Excellence in Clinical Virology, Faculty of Medicine, Chulalongkorn University	Thongpan,I., Mauleekoonphairoj,J., Vichiwattana,P., Korkong,S., Vongpunsawad,S. and Poovorawan,Y.
EPI_ISL_2576400, EPI_ISL_2576401, EPI_ISL_2576402, EPI_ISL_2576403, EPI_ISL_2576404, EPI_ISL_2576405, EPI_ISL_2576406, EPI_ISL_2576407, EPI_ISL_2576408, EPI_ISL_2576409, EPI_ISL_2576410, EPI_ISL_2576411, EPI_ISL_2576412, EPI_ISL_2576413, EPI_ISL_2576414, EPI_ISL_2576415, EPI_ISL_2576416, EPI_ISL_2576417, EPI_ISL_2576418, EPI_ISL_2576419, EPI_ISL_2576420, EPI_ISL_2576421, EPI_ISL_2576422, EPI_ISL_2576423, EPI_ISL_2576424, EPI_ISL_2576425, EPI_ISL_2576426, EPI_ISL_2576427, EPI_ISL_2576428, EPI_ISL_2576429, EPI_ISL_2576430, EPI_ISL_2576431, EPI_ISL_2576432, EPI_ISL_2576433, EPI_ISL_2576434, EPI_ISL_2576435, EPI_ISL_2576436, EPI_ISL_2576437, EPI_ISL_2576438, EPI_ISL_2576439			
see above	Department of Pediatrics, Center of Excellence in Clinical Virology, Chulalongkorn	Department of Pediatrics, Center of Excellence in Clinical Virology, Chulalongkorn	Thongpan,I.
EPI_ISL_2576440, EPI_ISL_2576482, EPI_ISL_2576483, EPI_ISL_2576484	Wint Wint Phyu Niigat University, International Health, Public Health	Wint Wint Phyu Niigat University, International Health, Public Health	Phyu,W.W. and Saito,R.
EPI_ISL_2576485	Department of Pediatrics, Center of Excellence in Clinical Virology, Chulalongkorn	Department of Pediatrics, Center of Excellence in Clinical Virology, Chulalongkorn	Thongpan,I.
EPI_ISL_2576486	Wint Wint Phyu Niigat University, International Health, Public Health	Wint Wint Phyu Niigat University, International Health, Public Health	Phyu,W.W. and Saito,R.
EPI_ISL_2576487	Department of Pediatrics, Center of Excellence in Clinical Virology, Chulalongkorn	Department of Pediatrics, Center of Excellence in Clinical Virology, Chulalongkorn	Thongpan,I.
EPI_ISL_2576488	Wint Wint Phyu Niigat University, International Health, Public Health	Wint Wint Phyu Niigat University, International Health, Public Health	Phyu,W.W. and Saito,R.
EPI_ISL_2576511, EPI_ISL_2576512, EPI_ISL_2576513	Centre for Infectious Diseases Virology, University of Heidelberg	Centre for Infectious Diseases Virology, University of Heidelberg	Ihling,C.M., Schnitzler,P., Heinrich,N., Mangu,C., Sudi,L., Souares,A., Gies,S., Sie,A., Coulibaly,B., Ouedraogo,A.T., Mordmueller,B., Held,J., Adegnika,A.A., Fernandez,J.F., Eckerle,I., May,J., Hogan,B., Eibach,D., Owusu-Dabo,E. and Tabatabai,J.
EPI_ISL_2576519, EPI_ISL_2576520, EPI_ISL_2576521, EPI_ISL_2576522, EPI_ISL_2576523	Department of Clinical Laboratory, Fujian Provincial Hospital	Department of Clinical Laboratory, Fujian Provincial Hospital	Su,Y., Wu,Y., Tian,R. and Liang,G.
EPI_ISL_2576524, EPI_ISL_2576525, EPI_ISL_2576526, EPI_ISL_2576527, EPI_ISL_2576528, EPI_ISL_2576529, EPI_ISL_2576530	Michiko Okamoto Tohoku University Graduate School of Medicine, Virology	Michiko Okamoto Tohoku University Graduate School of Medicine, Virology	Okamoto,M. and Dapat,C.
EPI_ISL_2576531	Bacteriology & Virology, University of Medical Sciences, Shiraz Medical School	Bacteriology & Virology, University of Medical Sciences, Shiraz Medical School	Mojarrad,S., Pirbonyeh,N., Moattari,A. and Babaei,A.
EPI_ISL_2576584, EPI_ISL_2576585, EPI_ISL_2576586, EPI_ISL_2576587, EPI_ISL_2576588, EPI_ISL_2576589, EPI_ISL_2576590, EPI_ISL_2576591, EPI_ISL_2576592, EPI_ISL_2576593, EPI_ISL_2576594			
see above	Virology Laboratory, Dr. Ricardo Gutierrez Children Hospital	Virology Laboratory, Dr. Ricardo Gutierrez Children Hospital	Rojo,G.L., Goya,S., Orellana,M., Sancilio,A., Rodriguez Perez,A., Montali,C., Garcia,C., Sosa,L., Musto,A., Hamilton,G., Alvarez,D., Castello,A. and Viegas,M.
EPI_ISL_2576595	Epidemiology and Demography, KEMRI-Wellcome Trust Research Programme	Epidemiology and Demography, KEMRI-Wellcome Trust Research Programme	Kamau,E.M., Agoti,C.N., Lewa,C.S., Cane,P.A., Bett,A., Medley,G.F. and Nokes,J.D.
EPI_ISL_2576596	Epidemiology and Demography, KEMRI-Wellcome Trust Research Programme	Epidemiology and Demography, KEMRI-Wellcome Trust Research Programme	Kamau,E.M., Agoti,C.N., Lewa,C.S., Cane,P.A., Nokes,J.D. and Agoti,C.N.
EPI_ISL_2576599, EPI_ISL_2576612	Epidemiology and Demography, KEMRI-Wellcome Trust Research Programme	Epidemiology and Demography, KEMRI-Wellcome Trust Research Programme	Kamau,E.M., Agoti,C.N., Lewa,C.S., Cane,P.A., Bett,A., Medley,G.F. and Nokes,J.D.
EPI_ISL_2576613, EPI_ISL_2576614, EPI_ISL_2576617, EPI_ISL_2576618	Epidemiology and Demography, KEMRI-Wellcome Trust Research Programme	Epidemiology and Demography, KEMRI-Wellcome Trust Research Programme	Kamau,E.M., Agoti,C.N., Lewa,C.S., Cane,P.A., Nokes,J.D. and Agoti,C.N.
EPI_ISL_2576627, EPI_ISL_2576628, EPI_ISL_2576629, EPI_ISL_2576630, EPI_ISL_2576631, EPI_ISL_2576632, EPI_ISL_2576633, EPI_ISL_2576634	Epidemiology and Demography, KEMRI-Wellcome Trust Research Programme	Epidemiology and Demography, KEMRI-Wellcome Trust Research Programme	Kamau,E.M., Agoti,C.N., Lewa,C.S., Cane,P.A., Bett,A., Medley,G.F. and Nokes,J.D.
EPI_ISL_2576635, EPI_ISL_2576636, EPI_ISL_2576637, EPI_ISL_2576638, EPI_ISL_2576639, EPI_ISL_2576640, EPI_ISL_2576641, EPI_ISL_2576642, EPI_ISL_2576643, EPI_ISL_2576644, EPI_ISL_2576645, EPI_ISL_2576646, EPI_ISL_2576647, EPI_ISL_2576648, EPI_ISL_2576649, EPI_ISL_2576650, EPI_ISL_2576651, EPI_ISL_2576652, EPI_ISL_2576653, EPI_ISL_2576654, EPI_ISL_2576655, EPI_ISL_2576656, EPI_ISL_2576657, EPI_ISL_2576658, EPI_ISL_2576659, EPI_ISL_2576660, EPI_ISL_2576673, EPI_ISL_2576674			
see above	Epidemiology and Demography, KEMRI-Wellcome Trust Research Programme	Epidemiology and Demography, KEMRI-Wellcome Trust Research Programme	Kamau,E.M., Agoti,C.N., Lewa,C.S., Cane,P.A., Nokes,J.D. and Agoti,C.N.
EPI_ISL_2576686	Department of Clinical Laboratory, Fujian Provincial Hospital	Department of Clinical Laboratory, Fujian Provincial Hospital	Su,Y., Wu,Y., Tian,R. and Liang,G.
EPI_ISL_2576689, EPI_ISL_2576690, EPI_ISL_2576691, EPI_ISL_2576692, EPI_ISL_2576694, EPI_ISL_2576695, EPI_ISL_2576696	Epidemiology and Demography, KEMRI-Wellcome Trust Research Programme	Epidemiology and Demography, KEMRI-Wellcome Trust Research Programme	Kamau,E.M., Agoti,C.N., Lewa,C.S., Cane,P.A., Nokes,J.D. and Agoti,C.N.
EPI_ISL_2576738	Virology, University of Ibadan, College of Medicine (UCH)	Virology, University of Ibadan, College of Medicine (UCH)	Ogunsemowo,O.O., Odaibo,G.N. and Olaleye,D.O.
EPI_ISL_2576740	Department of Clinical Laboratory, Fujian Provincial Hospital	Department of Clinical Laboratory, Fujian Provincial Hospital	Su,Y., Wu,Y., Tian,R. and Liang,G.
EPI_ISL_2576741	Michiko Okamoto Tohoku University Graduate School of Medicine, Virology	Michiko Okamoto Tohoku University Graduate School of Medicine, Virology	Okamoto,M. and Dapat,C.
EPI_ISL_2576742	Department of Clinical Laboratory, Fujian Provincial Hospital	Department of Clinical Laboratory, Fujian Provincial Hospital	Su,Y., Wu,Y., Tian,R. and Liang,G.
EPI_ISL_2576751, EPI_ISL_2576752, EPI_ISL_2576753	Virology, University of Ibadan, College of Medicine (UCH)	Virology, University of Ibadan, College of Medicine (UCH)	Ogunsemowo,O.S., Odaibo,G.N. and Olaleye,D.O.
EPI_ISL_2576762	Unidad de Biologia Viral, Instituto de Salud Carlos III	Unidad de Biologia Viral, Instituto de Salud Carlos III	Trento,A., Rodriguez-Fernandez,R., Gonzalez,M.I., Gonzalez,F., Mas,V., Vazquez,M., Palomo,C. and Melero,J.A.
EPI_ISL_2576763	Michiko Okamoto Tohoku University Graduate School of Medicine, Virology	Michiko Okamoto Tohoku University Graduate School of Medicine, Virology	Okamoto,M. and Dapat,C.
EPI_ISL_2576764	Bacteriology & Virology, University of Medical Sciences, Shiraz Medical School	Bacteriology & Virology, University of Medical Sciences, Shiraz Medical School	Mojarrad,S., Pirbonyeh,N., Moattari,A. and Babaei,A.
EPI_ISL_2576769, EPI_ISL_2576770	Epidemiology and Demography, KEMRI-Wellcome Trust Research Programme	Epidemiology and Demography, KEMRI-Wellcome Trust Research Programme	Kamau,E.M., Agoti,C.N., Owor,B., Lewa,C., Bett,A., Oketch,J., Cane,P.A. and Nokes,J.D.
EPI_ISL_2576783	Virology, University of Ibadan, College of Medicine (UCH)	Virology, University of Ibadan, College of Medicine (UCH)	Ogunsemowo,O., Odaibo,G.N. and Olaleye,D.O.
EPI_ISL_2576784, EPI_ISL_2576786	Epidemiology and Demography, KEMRI-Wellcome Trust Research Programme	Epidemiology and Demography, KEMRI-Wellcome Trust Research Programme	Kamau,E.M., Agoti,C.N., Owor,B., Lewa,C., Bett,A., Oketch,J., Cane,P.A. and Nokes,J.D.
EPI_ISL_2576805	WHO WPRO Measles Regional Reference Lab, Key Laboratory of Medical Virology Ministry of Health, National Institute for Viral Disease Control and Prevention, China Center for Disease Control and Prevention	WHO WPRO Measles Regional Reference Lab, Key Laboratory of Medical Virology Ministry of Health, National Institute for Viral Disease Control and Prevention, China Center for Disease Control and Prevention	Song,J., Zhang,Y. and Xu,W.
EPI_ISL_2576806, EPI_ISL_2576807, EPI_ISL_2576808, EPI_ISL_2576809	Michiko Okamoto Tohoku University Graduate School of Medicine, Virology	Michiko Okamoto Tohoku University Graduate School of Medicine, Virology	Okamoto,M. and Dapat,C.
EPI_ISL_2576810	Department of Clinical Laboratory, Fujian Provincial Hospital	Department of Clinical Laboratory, Fujian Provincial Hospital	Su,Y., Wu,Y., Tian,R. and Liang,G.
EPI_ISL_2576811	Michiko Okamoto Tohoku University Graduate School of Medicine, Virology	Michiko Okamoto Tohoku University Graduate School of Medicine, Virology	Okamoto,M. and Dapat,C.
EPI_ISL_2576824, EPI_ISL_2576825, EPI_ISL_2576826, EPI_ISL_2576827, EPI_ISL_2576828, EPI_ISL_2576829, EPI_ISL_2576830, EPI_ISL_2576831, EPI_ISL_2576832, EPI_ISL_2576833, EPI_ISL_2576834, EPI_ISL_2576835, EPI_ISL_2576836, EPI_ISL_2576837, EPI_ISL_2576838, EPI_ISL_2576839, EPI_ISL_2576840			
see above	Department of Pediatrics, Center of Excellence in Clinical Virology, Faculty of Medicine, Chulalongkorn University	Department of Pediatrics, Center of Excellence in Clinical Virology, Faculty of Medicine, Chulalongkorn University	Thongpan,I., Mauleekoonphairoj,J., Vichiwattana,P., Korkong,S., Vongpunsawad,S. and Poovorawan,Y.
EPI_ISL_2576891	Epidemiology and Demography, KEMRI-Wellcome Trust Research Programme	Epidemiology and Demography, KEMRI-Wellcome Trust Research Programme	Kamau,E.M., Agoti,C.N., Lewa,C.S., Cane,P.A., Bett,A., Medley,G.F. and Nokes,J.D.
EPI_ISL_2576892	Epidemiology and Demography, KEMRI-Wellcome Trust Research Programme	Epidemiology and Demography, KEMRI-Wellcome Trust Research Programme	Kamau,E.M., Agoti,C.N., Lewa,C.S., Cane,P.A., Nokes,J.D. and Agoti,C.N.
EPI_ISL_2576894, EPI_ISL_2576895, EPI_ISL_2576896, EPI_ISL_2576897, EPI_ISL_2576898, EPI_ISL_2576899, EPI_ISL_2576900, EPI_ISL_2576901, EPI_ISL_2576902, EPI_ISL_2576903, EPI_ISL_2576904, EPI_ISL_2576905, EPI_ISL_2576906, EPI_ISL_2576907, EPI_ISL_2576908, EPI_ISL_2576909, EPI_ISL_2576910, EPI_ISL_2577029, EPI_ISL_2577030, EPI_ISL_2577031, EPI_ISL_2577032, EPI_ISL_2577033, EPI_ISL_2577034			
see above	Virology Laboratory, Dr. Ricardo Gutierrez Children Hospital	Virology Laboratory, Dr. Ricardo Gutierrez Children Hospital	Rojo,G.L., Goya,S., Orellana,M., Sancilio,A., Rodriguez Perez,A., Montali,C., Garcia,C., Sosa,L., Musto,A., Hamilton,G., Alvarez,D., Castello,A. and Viegas,M.
EPI_ISL_2577035	Centre for Virology, Pushpagiri Institute of Medical Science and Research Centre	Centre for Virology, Pushpagiri Institute of Medical Science and Research Centre	Jose,S., Jagan,O.A., John,N.S., George,S., Chandy,S., Bai,S. and Manoharan,A.

EPI_ISL_2577036, EPI_ISL_2577037, EPI_ISL_2577038, EPI_ISL_2577039, EPI_ISL_2577040, EPI_ISL_2577041, EPI_ISL_2577042, EPI_ISL_2577043, EPI_ISL_2577044	Virology Laboratory, Dr. Ricardo Gutierrez Children Hospital	Virology Laboratory, Dr. Ricardo Gutierrez Children Hospital	Rojo,G.L., Goya,S., Orellana,M., Sancilio,A., Rodriguez Perez,A., Montali,C., Garcia,C., Sosa,L., Musto,A., Hamilton,G., Alvarez,D., Castello,A. and Viegas,M.
EPI_ISL_2577045, EPI_ISL_2577046, EPI_ISL_2577058, EPI_ISL_2577059	Epidemiology and Demography, KEMRI-Wellcome Trust Research Programme	Epidemiology and Demography, KEMRI-Wellcome Trust Research Programme	Kamau,E.M., Agoti,C.N., Lewa,C.S., Cane,P.A., Bett,A., Medley,G.F. and Nokes,J.D.
EPI_ISL_2577060	Epidemiology and Demography, KEMRI-Wellcome Trust Research Programme	Epidemiology and Demography, KEMRI-Wellcome Trust Research Programme	Kamau,E.M., Agoti,C.N., Lewa,C.S., Cane,P.A., Nokes,J.D. and Agoti,C.N.
EPI_ISL_2577064, EPI_ISL_2577065, EPI_ISL_2577066	Epidemiology and Demography, KEMRI-Wellcome Trust Research Programme	Epidemiology and Demography, KEMRI-Wellcome Trust Research Programme	Kamau,E.M., Agoti,C.N., Lewa,C.S., Cane,P.A., Bett,A., Medley,G.F. and Nokes,J.D.
EPI_ISL_2577067, EPI_ISL_2577068	Epidemiology and Demography, KEMRI-Wellcome Trust Research Programme	Epidemiology and Demography, KEMRI-Wellcome Trust Research Programme	Kamau,E.M., Agoti,C.N., Lewa,C.S., Cane,P.A., Nokes,J.D. and Agoti,C.N.
EPI_ISL_2577106, EPI_ISL_2577107, EPI_ISL_2577108, EPI_ISL_2577109, EPI_ISL_2577110, EPI_ISL_2577111, EPI_ISL_2577112, EPI_ISL_2577113, EPI_ISL_2577114, EPI_ISL_2577115, EPI_ISL_2577116, EPI_ISL_2577117, EPI_ISL_2577118, EPI_ISL_2577119, EPI_ISL_2577120, EPI_ISL_2577121, EPI_ISL_2577122, EPI_ISL_2577123, EPI_ISL_2577124, EPI_ISL_2577125, EPI_ISL_2577126			
see above	Epidemiology and Demography, KEMRI-Wellcome Trust Research Programme	Epidemiology and Demography, KEMRI-Wellcome Trust Research Programme	Kamau,E.M., Agoti,C.N., Lewa,C.S., Cane,P.A., Bett,A., Medley,G.F. and Nokes,J.D.
EPI_ISL_2577127	Epidemiology and Demography, KEMRI-Wellcome Trust Research Programme	Epidemiology and Demography, KEMRI-Wellcome Trust Research Programme	Kamau,E.M., Agoti,C.N., Lewa,C.S., Cane,P.A., Nokes,J.D. and Agoti,C.N.
EPI_ISL_2577128	Epidemiology and Demography, KEMRI-Wellcome Trust Research Programme	Epidemiology and Demography, KEMRI-Wellcome Trust Research Programme	Kamau,E.M., Agoti,C.N., Lewa,C.S., Cane,P.A., Bett,A., Medley,G.F. and Nokes,J.D.
EPI_ISL_2577129, EPI_ISL_2577130	Epidemiology and Demography, KEMRI-Wellcome Trust Research Programme	Epidemiology and Demography, KEMRI-Wellcome Trust Research Programme	Kamau,E.M., Agoti,C.N., Lewa,C.S., Cane,P.A., Nokes,J.D. and Agoti,C.N.
EPI_ISL_2577131	Epidemiology and Demography, KEMRI-Wellcome Trust Research Programme	Epidemiology and Demography, KEMRI-Wellcome Trust Research Programme	Kamau,E.M., Agoti,C.N., Lewa,C.S., Cane,P.A., Bett,A., Medley,G.F. and Nokes,J.D.
EPI_ISL_2577132, EPI_ISL_2577133, EPI_ISL_2577134, EPI_ISL_2577135, EPI_ISL_2577136, EPI_ISL_2577137, EPI_ISL_2577138, EPI_ISL_2577139, EPI_ISL_2577140, EPI_ISL_2577141, EPI_ISL_2577142, EPI_ISL_2577143, EPI_ISL_2577144, EPI_ISL_2577145, EPI_ISL_2577146, EPI_ISL_2577147, EPI_ISL_2577148, EPI_ISL_2577149, EPI_ISL_2577150, EPI_ISL_2577151, EPI_ISL_2577152, EPI_ISL_2577153			
see above	Epidemiology and Demography, KEMRI-Wellcome Trust Research Programme	Epidemiology and Demography, KEMRI-Wellcome Trust Research Programme	Kamau,E.M., Agoti,C.N., Lewa,C.S., Cane,P.A., Nokes,J.D. and Agoti,C.N.
EPI_ISL_2577165, EPI_ISL_2577166, EPI_ISL_2577167, EPI_ISL_2577168, EPI_ISL_2577169, EPI_ISL_2577170	Centre for Infectious Diseases Research, Diagnostics and laboratory Surveillance, National Institute for Public Health and the Environment	Centre for Infectious Diseases Research, Diagnostics and laboratory Surveillance, National Institute for Public Health and the Environment	Meijer,A. and Overduin,P.
EPI_ISL_2577171, EPI_ISL_2577174, EPI_ISL_2577175, EPI_ISL_2577176	Epidemiology and Demography, KEMRI-Wellcome Trust Research Programme	Epidemiology and Demography, KEMRI-Wellcome Trust Research Programme	Kamau,E.M., Agoti,C.N., Lewa,C.S., Cane,P.A., Nokes,J.D. and Agoti,C.N.
EPI_ISL_2577177	Centre for Infectious Diseases Research, Diagnostics and laboratory Surveillance, National Institute for Public Health and the Environment	Centre for Infectious Diseases Research, Diagnostics and laboratory Surveillance, National Institute for Public Health and the Environment	Meijer,A. and Overduin,P.
EPI_ISL_2577203	Department of Clinical Laboratory, Fujian Provincial Hospital	Department of Clinical Laboratory, Fujian Provincial Hospital	Su,Y., Wu,Y., Tian,R. and Liang,G.
EPI_ISL_2577204, EPI_ISL_2577205	WHO WPRO Measles Regional Reference Lab, Key Laboratory of Medical Virology Ministry of Health, National Institute for Viral Disease Control and Prevention, China Center for Disease Control and Prevention	WHO WPRO Measles Regional Reference Lab, Key Laboratory of Medical Virology Ministry of Health, National Institute for Viral Disease Control and Prevention, China Center for Disease Control and Prevention	Song,J., Zhang,Y. and Xu,W.
EPI_ISL_2577206, EPI_ISL_2577207	Michiko Okamoto Tohoku University Graduate School of Medicine, Virology	Michiko Okamoto Tohoku University Graduate School of Medicine, Virology	Okamoto,M. and Dapat,C.
EPI_ISL_2577208, EPI_ISL_2577209	Department of Clinical Laboratory, Fujian Provincial Hospital	Department of Clinical Laboratory, Fujian Provincial Hospital	Su,Y., Wu,Y., Tian,R. and Liang,G.
EPI_ISL_2577210, EPI_ISL_2577211, EPI_ISL_2577212, EPI_ISL_2577213	Michiko Okamoto Tohoku University Graduate School of Medicine, Virology	Michiko Okamoto Tohoku University Graduate School of Medicine, Virology	Okamoto,M. and Dapat,C.
EPI_ISL_2577232	Virology, University of Ibadan, College of Medicine (UCH)	Virology, University of Ibadan, College of Medicine (UCH)	Ogunsemowo,O., Odaibo,G.N. and Olaleye,D.O.
EPI_ISL_2577233	Virology, University of Ibadan, College of Medicine (UCH)	Virology, University of Ibadan, College of Medicine (UCH)	Ogunsemowo,O.O., Odaibo,G.N. and Olaleye,D.O.
EPI_ISL_2577249	Department of Clinical Laboratory, Fujian Provincial Hospital	Department of Clinical Laboratory, Fujian Provincial Hospital	Su,Y., Wu,Y., Tian,R. and Liang,G.
EPI_ISL_2577250	Unidad de Biologia Viral, Instituto de Salud Carlos III	Unidad de Biologia Viral, Instituto de Salud Carlos III	Trento,A., Rodriguez-Fernandez,R., Gonzalez,M.I., Gonzalez,F., Mas,V., Vazquez,M., Palomo,C. and Melero,J.A.
EPI_ISL_2577251	Department of Clinical Laboratory, Fujian Provincial Hospital	Department of Clinical Laboratory, Fujian Provincial Hospital	Su,Y., Wu,Y., Tian,R. and Liang,G.
EPI_ISL_2577252, EPI_ISL_2577253, EPI_ISL_2577254, EPI_ISL_2577255, EPI_ISL_2577256, EPI_ISL_2577257, EPI_ISL_2577258, EPI_ISL_2577259	Michiko Okamoto Tohoku University Graduate School of Medicine, Virology	Michiko Okamoto Tohoku University Graduate School of Medicine, Virology	Okamoto,M. and Dapat,C.
EPI_ISL_2577260	Department of Clinical Laboratory, Fujian Provincial Hospital	Department of Clinical Laboratory, Fujian Provincial Hospital	Su,Y., Wu,Y., Tian,R. and Liang,G.
EPI_ISL_2577261, EPI_ISL_2577262, EPI_ISL_2577263, EPI_ISL_2577264	Michiko Okamoto Tohoku University Graduate School of Medicine, Virology	Michiko Okamoto Tohoku University Graduate School of Medicine, Virology	Okamoto,M. and Dapat,C.
EPI_ISL_2577270	Epidemiology and Demography, KEMRI-Wellcome Trust Research Programme	Epidemiology and Demography, KEMRI-Wellcome Trust Research Programme	Kamau,E.M., Agoti,C.N., Owor,B., Lewa,C., Bett,A., Oketch,J., Cane,P.A. and Nokes,J.D.
EPI_ISL_2577271	Virology, University of Ibadan, College of Medicine (UCH)	Virology, University of Ibadan, College of Medicine (UCH)	Ogunsemowo,O., Odaibo,G.N. and Olaleye,D.O.
EPI_ISL_2577272, EPI_ISL_2577273, EPI_ISL_2577274	Epidemiology and Demography, KEMRI-Wellcome Trust Research Programme	Epidemiology and Demography, KEMRI-Wellcome Trust Research Programme	Kamau,E.M., Agoti,C.N., Owor,B., Lewa,C., Bett,A., Oketch,J., Cane,P.A. and Nokes,J.D.
EPI_ISL_2577275, EPI_ISL_2577276, EPI_ISL_2577277	Centre for Infectious Diseases Research, Diagnostics and laboratory Surveillance, National Institute for Public Health and the Environment	Centre for Infectious Diseases Research, Diagnostics and laboratory Surveillance, National Institute for Public Health and the Environment	Meijer,A. and Overduin,P.
EPI_ISL_2577290	Epidemiology and Demography, KEMRI-Wellcome Trust Research Programme	Epidemiology and Demography, KEMRI-Wellcome Trust Research Programme	Kamau,E.M., Agoti,C.N., Owor,B., Lewa,C., Bett,A., Oketch,J., Cane,P.A. and Nokes,J.D.
EPI_ISL_2577291, EPI_ISL_2577300	Marie Bashir Institute for Infectious Diseases and Biosecurity & Sydney Medical School, The University of Sydney, Westmead Institute for Medical Research	Marie Bashir Institute for Infectious Diseases and Biosecurity & Sydney Medical School, The University of Sydney, Westmead Institute for Medical Research	Eden,J.-S., Kok,J., Dwyer,D.E., Fernandez,M., Carter,I. and Holmes,E.C.
EPI_ISL_2577309, EPI_ISL_2577310	Michiko Okamoto Graduate School of Medicine, Tohoku University, Virology	Michiko Okamoto Graduate School of Medicine, Tohoku University, Virology	Okamoto,M. and Oshitani,H.
EPI_ISL_2577311, EPI_ISL_2577312	Marie Bashir Institute for Infectious Diseases and Biosecurity & Sydney Medical School, The University of Sydney, Westmead Institute for Medical Research	Marie Bashir Institute for Infectious Diseases and Biosecurity & Sydney Medical School, The University of Sydney, Westmead Institute for Medical Research	Eden,J.-S., Kok,J., Dwyer,D.E., Fernandez,M., Carter,I. and Holmes,E.C.
EPI_ISL_2577326	Reference Microbiology, Public Health England National Infection Services	Reference Microbiology, Public Health England National Infection Services	Valappil,M., Talts,T., Ellis,J., Sails,A., Eltringham,G., Waugh,S., Gould,K., Harrison,I., Pebody,R. and Zambon,M.
EPI_ISL_2577327, EPI_ISL_2577328, EPI_ISL_2577329, EPI_ISL_2577330	Marie Bashir Institute for Infectious Diseases and Biosecurity & Sydney Medical School, The University of Sydney, Westmead Institute for Medical Research	Marie Bashir Institute for Infectious Diseases and Biosecurity & Sydney Medical School, The University of Sydney, Westmead Institute for Medical Research	Eden,J.-S., Kok,J., Dwyer,D.E., Fernandez,M., Carter,I. and Holmes,E.C.
EPI_ISL_2577331	Michiko Okamoto Graduate School of Medicine, Tohoku University, Virology	Michiko Okamoto Graduate School of Medicine, Tohoku University, Virology	Okamoto,M. and Oshitani,H.
EPI_ISL_2577335, EPI_ISL_2577336, EPI_ISL_2577337, EPI_ISL_2577338, EPI_ISL_2577339	Marie Bashir Institute for Infectious Diseases and Biosecurity & Sydney Medical School, The University of Sydney, Westmead Institute for Medical Research	Marie Bashir Institute for Infectious Diseases and Biosecurity & Sydney Medical School, The University of Sydney, Westmead Institute for Medical Research	Eden,J.-S., Kok,J., Dwyer,D.E., Fernandez,M., Carter,I. and Holmes,E.C.
EPI_ISL_2577361	Department of Clinical Laboratory, Fujian Provincial Hospital	Department of Clinical Laboratory, Fujian Provincial Hospital	Su,Y., Wu,Y., Tian,R. and Liang,G.
EPI_ISL_2577362, EPI_ISL_2577363	WHO WPRO Measles Regional Reference Lab, Key Laboratory of Medical Virology Ministry of Health, National Institute for Viral Disease Control and Prevention, China Center for Disease Control and Prevention	WHO WPRO Measles Regional Reference Lab, Key Laboratory of Medical Virology Ministry of Health, National Institute for Viral Disease Control and Prevention, China Center for Disease Control and Prevention	Song,J., Zhang,Y. and Xu,W.
EPI_ISL_2577364	Department of Clinical Laboratory, Fujian Provincial Hospital	Department of Clinical Laboratory, Fujian Provincial Hospital	Su,Y., Wu,Y., Tian,R. and Liang,G.
EPI_ISL_2577365, EPI_ISL_2577366	WHO WPRO Measles Regional Reference Lab, Key Laboratory of Medical Virology Ministry of Health, National Institute for Viral Disease Control and Prevention, China Center for Disease Control and Prevention	WHO WPRO Measles Regional Reference Lab, Key Laboratory of Medical Virology Ministry of Health, National Institute for Viral Disease Control and Prevention, China Center for Disease Control and Prevention	Song,J., Zhang,Y. and Xu,W.
EPI_ISL_2577367	Department of Clinical Laboratory, Fujian Provincial Hospital	Department of Clinical Laboratory, Fujian Provincial Hospital	Su,Y., Wu,Y., Tian,R. and Liang,G.
EPI_ISL_2577368	WHO WPRO Measles Regional Reference Lab, Key Laboratory of Medical Virology Ministry of Health, National Institute for Viral Disease Control and Prevention, China Center for Disease Control and Prevention	WHO WPRO Measles Regional Reference Lab, Key Laboratory of Medical Virology Ministry of Health, National Institute for Viral Disease Control and Prevention, China Center for Disease Control and Prevention	Song,J., Zhang,Y. and Xu,W.
EPI_ISL_2577369, EPI_ISL_2577370, EPI_ISL_2577371, EPI_ISL_2577372, EPI_ISL_2577373, EPI_ISL_2577374	Michiko Okamoto Tohoku University Graduate School of Medicine, Virology	Michiko Okamoto Tohoku University Graduate School of Medicine, Virology	Okamoto,M. and Dapat,C.
EPI_ISL_2577375, EPI_ISL_2577376	Department of Clinical Laboratory, Fujian Provincial Hospital	Department of Clinical Laboratory, Fujian Provincial Hospital	Su,Y., Wu,Y., Tian,R. and Liang,G.
EPI_ISL_2577377, EPI_ISL_2577378, EPI_ISL_2577379, EPI_ISL_2577380, EPI_ISL_2577381	Michiko Okamoto Tohoku University Graduate School of Medicine, Virology	Michiko Okamoto Tohoku University Graduate School of Medicine, Virology	Okamoto,M. and Dapat,C.
EPI_ISL_2577384	Michiko Okamoto Graduate School of Medicine, Tohoku University, Virology	Michiko Okamoto Graduate School of Medicine, Tohoku University, Virology	Okamoto,M. and Oshitani,H.
EPI_ISL_2577385	Reference Microbiology, Public Health England National Infection Services	Reference Microbiology, Public Health England National Infection Services	Valappil,M., Talts,T., Ellis,J., Sails,A., Eltringham,G., Waugh,S., Gould,K., Harrison,I., Pebody,R. and Zambon,M.
EPI_ISL_2577386, EPI_ISL_2577387	Marie Bashir Institute for Infectious Diseases and Biosecurity & Sydney Medical School, The University of Sydney, Westmead Institute for Medical Research	Marie Bashir Institute for Infectious Diseases and Biosecurity & Sydney Medical School, The University of Sydney, Westmead Institute for Medical Research	Eden,J.-S., Kok,J., Dwyer,D.E., Fernandez,M., Carter,I. and Holmes,E.C.
EPI_ISL_2577396	Reference Microbiology, Public Health England National Infection Services	Reference Microbiology, Public Health England National Infection Services	Valappil,M., Talts,T., Ellis,J., Sails,A., Eltringham,G., Waugh,S., Gould,K., Harrison,I., Pebody,R. and Zambon,M.
EPI_ISL_2577397, EPI_ISL_2577398, EPI_ISL_2577399	J. Craig Venter Institute	J. Craig Venter Institute	Tan,G., Pickett,B., Fedorova,N., Amedeo,P., Hu,L., Christensen,J., Miller,J., Durbin,A., Williams,T., Arumemi,F., Cadiz,C., Alanis,R., Balmseda,A., Wiliams,T., Schiller,A., Patel,M.,

			Kubale,J. and Gordon,A.
EPI_ISL_2577400, EPI_ISL_2577401, EPI_ISL_2577402, EPI_ISL_2577409, EPI_ISL_2577410	Marie Bashir Institute for Infectious Diseases and Biosecurity & Sydney Medical School, The University of Sydney, Westmead Institute for Medical Research	Marie Bashir Institute for Infectious Diseases and Biosecurity & Sydney Medical School, The University of Sydney, Westmead Institute for Medical Research	Eden,J.-S., Kok,J., Dwyer,D.E., Fernandez,M., Carter,I. and Holmes,E.C.
EPI_ISL_2577411, EPI_ISL_2577412, EPI_ISL_2577413	J. Craig Venter Institute	J. Craig Venter Institute	Tan,G., Pickett,B., Fedorova,N., Amedeo,P., Hu,L., Christensen,J., Miller,J., Durbin,A., Williams,T., Arumemi,F., Cadiz,C., Alanis,R., Balmseda,A., Wiliams,T., Schiller,A., Patel,M., Kubale,J. and Gordon,A.
EPI_ISL_2577414, EPI_ISL_2577415, EPI_ISL_2577427, EPI_ISL_2577428	Marie Bashir Institute for Infectious Diseases and Biosecurity & Sydney Medical School, The University of Sydney, Westmead Institute for Medical Research	Marie Bashir Institute for Infectious Diseases and Biosecurity & Sydney Medical School, The University of Sydney, Westmead Institute for Medical Research	Eden,J.-S., Kok,J., Dwyer,D.E., Fernandez,M., Carter,I. and Holmes,E.C.
EPI_ISL_2577429	J. Craig Venter Institute	J. Craig Venter Institute	Tan,G., Pickett,B., Fedorova,N., Amedeo,P., Hu,L., Christensen,J., Miller,J., Durbin,A., Williams,T., Arumemi,F., Cadiz,C., Alanis,R., Balmseda,A., Wiliams,T., Schiller,A., Patel,M., Kubale,J. and Gordon,A.
EPI_ISL_2577430, EPI_ISL_2577438, EPI_ISL_2577439, EPI_ISL_2577440	Marie Bashir Institute for Infectious Diseases and Biosecurity & Sydney Medical School, The University of Sydney, Westmead Institute for Medical Research	Marie Bashir Institute for Infectious Diseases and Biosecurity & Sydney Medical School, The University of Sydney, Westmead Institute for Medical Research	Eden,J.-S., Kok,J., Dwyer,D.E., Fernandez,M., Carter,I. and Holmes,E.C.
EPI_ISL_2577441, EPI_ISL_2577442, EPI_ISL_2577443	J. Craig Venter Institute	J. Craig Venter Institute	Tan,G., Pickett,B., Fedorova,N., Amedeo,P., Hu,L., Christensen,J., Miller,J., Durbin,A., Williams,T., Arumemi,F., Cadiz,C., Alanis,R., Balmseda,A., Wiliams,T., Schiller,A., Patel,M., Kubale,J. and Gordon,A.
EPI_ISL_2577444	Marie Bashir Institute for Infectious Diseases and Biosecurity & Sydney Medical School, The University of Sydney, Westmead Institute for Medical Research	Marie Bashir Institute for Infectious Diseases and Biosecurity & Sydney Medical School, The University of Sydney, Westmead Institute for Medical Research	Eden,J.-S., Kok,J., Dwyer,D.E., Fernandez,M., Carter,I. and Holmes,E.C.
EPI_ISL_2577986, EPI_ISL_2577987, EPI_ISL_2577988, EPI_ISL_2577989, EPI_ISL_2577990, EPI_ISL_2577991, EPI_ISL_2577992, EPI_ISL_2577993, EPI_ISL_2577994, EPI_ISL_2577995, EPI_ISL_2577996, EPI_ISL_2577997			
see above	WHO WPRO Measles Regional Reference Lab, Key Laboratory of Medical Virology Ministry of Health, National Institute for Viral Disease Control and Prevention, China Center for Disease Control and Prevention	WHO WPRO Measles Regional Reference Lab, Key Laboratory of Medical Virology Ministry of Health, National Institute for Viral Disease Control and Prevention, China Center for Disease Control and Prevention	Song,J., Zhang,Y. and Xu,W.
EPI_ISL_2577998	Unidad de Biologia Viral, Instituto de Salud Carlos III	Unidad de Biologia Viral, Instituto de Salud Carlos III	Trento,A., Rodriguez-Fernandez,R., Gonzalez,M.I., Gonzalez,F., Mas,V., Vazquez,M., Palomo,C. and Melero,J.A.
EPI_ISL_2577999, EPI_ISL_2578000, EPI_ISL_2578001	Department of Public Health Laboratory Sciences, West China School of Public Health, Sichuan University	Department of Public Health Laboratory Sciences, West China School of Public Health, Sichuan University	Hu,W.P. and Pei,F.X.
EPI_ISL_2578002	Unidad de Biologia Viral, Instituto de Salud Carlos III	Unidad de Biologia Viral, Instituto de Salud Carlos III	Trento,A., Rodriguez-Fernandez,R., Gonzalez,M.I., Gonzalez,F., Mas,V., Vazquez,M., Palomo,C. and Melero,J.A.
EPI_ISL_2578003	Centre for Research And Knowledge Transfer in Biotechnology, University of Zagreb	Centre for Research And Knowledge Transfer in Biotechnology, University of Zagreb	Ivancic-Jelecki,J., Slovic,A., Ljubin-Sternak,S., Mlinaric-Galinovic,G. and Forcic,D.
EPI_ISL_2578004	WHO WPRO Measles Regional Reference Lab, Key Laboratory of Medical Virology Ministry of Health, National Institute for Viral Disease Control and Prevention, China Center for Disease Control and Prevention	WHO WPRO Measles Regional Reference Lab, Key Laboratory of Medical Virology Ministry of Health, National Institute for Viral Disease Control and Prevention, China Center for Disease Control and Prevention	Song,J., Zhang,Y. and Xu,W.
EPI_ISL_2578005	Centre for Research And Knowledge Transfer in Biotechnology, University of Zagreb	Centre for Research And Knowledge Transfer in Biotechnology, University of Zagreb	Ivancic-Jelecki,J., Slovic,A., Ljubin-Sternak,S., Mlinaric-Galinovic,G. and Forcic,D.
EPI_ISL_2578006	WHO WPRO Measles Regional Reference Lab, Key Laboratory of Medical Virology Ministry of Health, National Institute for Viral Disease Control and Prevention, China Center for Disease Control and Prevention	WHO WPRO Measles Regional Reference Lab, Key Laboratory of Medical Virology Ministry of Health, National Institute for Viral Disease Control and Prevention, China Center for Disease Control and Prevention	Song,J., Zhang,Y. and Xu,W.
EPI_ISL_2578007	Centre for Research And Knowledge Transfer in Biotechnology, University of Zagreb	Centre for Research And Knowledge Transfer in Biotechnology, University of Zagreb	Ivancic-Jelecki,J., Slovic,A., Ljubin-Sternak,S., Mlinaric-Galinovic,G. and Forcic,D.
EPI_ISL_2578008, EPI_ISL_2578009, EPI_ISL_2578010	WHO WPRO Measles Regional Reference Lab, Key Laboratory of Medical Virology Ministry of Health, National Institute for Viral Disease Control and Prevention, China Center for Disease Control and Prevention	WHO WPRO Measles Regional Reference Lab, Key Laboratory of Medical Virology Ministry of Health, National Institute for Viral Disease Control and Prevention, China Center for Disease Control and Prevention	Song,J., Zhang,Y. and Xu,W.
EPI_ISL_2578011	Centre for Research And Knowledge Transfer in Biotechnology, University of Zagreb	Centre for Research And Knowledge Transfer in Biotechnology, University of Zagreb	Ivancic-Jelecki,J., Slovic,A., Ljubin-Sternak,S., Mlinaric-Galinovic,G. and Forcic,D.
EPI_ISL_2578012, EPI_ISL_2578013	Department of Public Health Laboratory Sciences, West China School of Public Health, Sichuan University	Department of Public Health Laboratory Sciences, West China School of Public Health, Sichuan University	Hu,W.P. and Pei,F.X.
EPI_ISL_2578014	WHO WPRO Measles Regional Reference Lab, Key Laboratory of Medical Virology Ministry of Health, National Institute for Viral Disease Control and Prevention, China Center for Disease Control and Prevention	WHO WPRO Measles Regional Reference Lab, Key Laboratory of Medical Virology Ministry of Health, National Institute for Viral Disease Control and Prevention, China Center for Disease Control and Prevention	Song,J., Zhang,Y. and Xu,W.
EPI_ISL_2578015	Centre for Research And Knowledge Transfer in Biotechnology, University of Zagreb	Centre for Research And Knowledge Transfer in Biotechnology, University of Zagreb	Ivancic-Jelecki,J., Slovic,A., Ljubin-Sternak,S., Mlinaric-Galinovic,G. and Forcic,D.
EPI_ISL_2578016, EPI_ISL_2578017, EPI_ISL_2578018	WHO WPRO Measles Regional Reference Lab, Key Laboratory of Medical Virology Ministry of Health, National Institute for Viral Disease Control and Prevention, China Center for Disease Control and Prevention	WHO WPRO Measles Regional Reference Lab, Key Laboratory of Medical Virology Ministry of Health, National Institute for Viral Disease Control and Prevention, China Center for Disease Control and Prevention	Song,J., Zhang,Y. and Xu,W.
EPI_ISL_2578019	Unidad de Biologia Viral, Instituto de Salud Carlos III	Unidad de Biologia Viral, Instituto de Salud Carlos III	Trento,A., Rodriguez-Fernandez,R., Gonzalez,M.I., Gonzalez,F., Mas,V., Vazquez,M., Palomo,C. and Melero,J.A.
EPI_ISL_2578020	WHO WPRO Measles Regional Reference Lab, Key Laboratory of Medical Virology Ministry of Health, National Institute for Viral Disease Control and Prevention, China Center for Disease Control and Prevention	WHO WPRO Measles Regional Reference Lab, Key Laboratory of Medical Virology Ministry of Health, National Institute for Viral Disease Control and Prevention, China Center for Disease Control and Prevention	Song,J., Zhang,Y. and Xu,W.
EPI_ISL_2578021	Unidad de Biologia Viral, Instituto de Salud Carlos III	Unidad de Biologia Viral, Instituto de Salud Carlos III	Trento,A., Rodriguez-Fernandez,R., Gonzalez,M.I., Gonzalez,F., Mas,V., Vazquez,M., Palomo,C. and Melero,J.A.
EPI_ISL_2578022	WHO WPRO Measles Regional Reference Lab, Key Laboratory of Medical Virology Ministry of Health, National Institute for Viral Disease Control and Prevention, China Center for Disease Control and Prevention	WHO WPRO Measles Regional Reference Lab, Key Laboratory of Medical Virology Ministry of Health, National Institute for Viral Disease Control and Prevention, China Center for Disease Control and Prevention	Song,J., Zhang,Y. and Xu,W.
EPI_ISL_2578023	Centre for Research And Knowledge Transfer in Biotechnology, University of Zagreb	Centre for Research And Knowledge Transfer in Biotechnology, University of Zagreb	Ivancic-Jelecki,J., Slovic,A., Ljubin-Sternak,S., Mlinaric-Galinovic,G. and Forcic,D.
EPI_ISL_2578024, EPI_ISL_2578025	WHO WPRO Measles Regional Reference Lab, Key Laboratory of Medical Virology Ministry of Health, National Institute for Viral Disease Control and Prevention, China Center for Disease Control and Prevention	WHO WPRO Measles Regional Reference Lab, Key Laboratory of Medical Virology Ministry of Health, National Institute for Viral Disease Control and Prevention, China Center for Disease Control and Prevention	Song,J., Zhang,Y. and Xu,W.
EPI_ISL_2578026	Centre for Research And Knowledge Transfer in Biotechnology, University of Zagreb	Centre for Research And Knowledge Transfer in Biotechnology, University of Zagreb	Ivancic-Jelecki,J., Slovic,A., Ljubin-Sternak,S., Mlinaric-Galinovic,G. and Forcic,D.
EPI_ISL_2578027	WHO WPRO Measles Regional Reference Lab, Key Laboratory of Medical Virology Ministry of Health, National Institute for Viral Disease Control and Prevention, China Center for Disease Control and Prevention	WHO WPRO Measles Regional Reference Lab, Key Laboratory of Medical Virology Ministry of Health, National Institute for Viral Disease Control and Prevention, China Center for Disease Control and Prevention	Song,J., Zhang,Y. and Xu,W.
EPI_ISL_2578028	Centre for Research And Knowledge Transfer in Biotechnology, University of Zagreb	Centre for Research And Knowledge Transfer in Biotechnology, University of Zagreb	Ivancic-Jelecki,J., Slovic,A., Ljubin-Sternak,S., Mlinaric-Galinovic,G. and Forcic,D.
EPI_ISL_2578029, EPI_ISL_2578030	WHO WPRO Measles Regional Reference Lab, Key Laboratory of Medical Virology Ministry of Health, National Institute for Viral Disease Control and Prevention, China Center for Disease Control and Prevention	WHO WPRO Measles Regional Reference Lab, Key Laboratory of Medical Virology Ministry of Health, National Institute for Viral Disease Control and Prevention, China Center for Disease Control and Prevention	Song,J., Zhang,Y. and Xu,W.
EPI_ISL_2578031, EPI_ISL_2578032, EPI_ISL_2578033, EPI_ISL_2578034, EPI_ISL_2578035, EPI_ISL_2578036	Centre for Research And Knowledge Transfer in Biotechnology, University of Zagreb	Centre for Research And Knowledge Transfer in Biotechnology, University of Zagreb	Ivancic-Jelecki,J., Slovic,A., Ljubin-Sternak,S., Mlinaric-Galinovic,G. and Forcic,D.
EPI_ISL_2578037	Epidemiology and Demography, KEMRI-Wellcome Trust Research Programme	Epidemiology and Demography, KEMRI-Wellcome Trust Research Programme	Kamau,E.M., Agoti,C.N., Owor,B., Lewa,C., Bett,A., Oketch,J., Cane,P.A. and Nokes,J.D.
EPI_ISL_2578038	WHO WPRO Measles Regional Reference Lab, Key Laboratory of Medical Virology Ministry of Health, National Institute for Viral Disease Control and Prevention, China Center for Disease Control and Prevention	WHO WPRO Measles Regional Reference Lab, Key Laboratory of Medical Virology Ministry of Health, National Institute for Viral Disease Control and Prevention, China Center for Disease Control and Prevention	Song,J., Zhang,Y. and Xu,W.
EPI_ISL_2578039	Centre for Infectious Diseases Research, Diagnostics and laboratory Surveillance, National Institute for Public Health and the Environment	Centre for Infectious Diseases Research, Diagnostics and laboratory Surveillance, National Institute for Public Health and the Environment	Meijer,A. and Overduin,P.
EPI_ISL_2578040	Centre for Research And Knowledge Transfer in Biotechnology, University of Zagreb	Centre for Research And Knowledge Transfer in Biotechnology, University of Zagreb	Ivancic-Jelecki,J., Slovic,A., Ljubin-Sternak,S., Mlinaric-Galinovic,G. and Forcic,D.
EPI_ISL_2578041, EPI_ISL_2578042	WHO WPRO Measles Regional Reference Lab, Key Laboratory of Medical Virology Ministry of Health, National Institute for Viral Disease Control and Prevention, China Center for Disease Control and Prevention	WHO WPRO Measles Regional Reference Lab, Key Laboratory of Medical Virology Ministry of Health, National Institute for Viral Disease Control and Prevention, China Center for Disease Control and Prevention	Song,J., Zhang,Y. and Xu,W.
EPI_ISL_2578043, EPI_ISL_2578044, EPI_ISL_2578045, EPI_ISL_2578046	Centre for Research And Knowledge Transfer in Biotechnology, University of Zagreb	Centre for Research And Knowledge Transfer in Biotechnology, University of Zagreb	Ivancic-Jelecki,J., Slovic,A., Ljubin-Sternak,S., Mlinaric-Galinovic,G. and Forcic,D.

EPI_ISL_2578047, EPI_ISL_2578048, EPI_ISL_2578049, EPI_ISL_2578050, EPI_ISL_2578051, EPI_ISL_2578052, EPI_ISL_2578053, EPI_ISL_2578054, EPI_ISL_2578055	WHO WPRO Measles Regional Reference Lab, Key Laboratory of Medical Virology Ministry of Health, National Institute for Viral Disease Control and Prevention, China Center for Disease Control and Prevention	WHO WPRO Measles Regional Reference Lab, Key Laboratory of Medical Virology Ministry of Health, National Institute for Viral Disease Control and Prevention, China Center for Disease Control and Prevention	Song,J., Zhang,Y. and Xu,W.
EPI_ISL_2578056	Centre for Research And Knowledge Transfer in Biotechnology, University of Zagreb	Centre for Research And Knowledge Transfer in Biotechnology, University of Zagreb	Ivancic-Jelecki,J., Slovic,A., Ljubin-Sternak,S., Mlinaric-Galinovic,G. and Forcic,D.
EPI_ISL_2578057, EPI_ISL_2578058, EPI_ISL_2578059	WHO WPRO Measles Regional Reference Lab, Key Laboratory of Medical Virology Ministry of Health, National Institute for Viral Disease Control and Prevention, China Center for Disease Control and Prevention	WHO WPRO Measles Regional Reference Lab, Key Laboratory of Medical Virology Ministry of Health, National Institute for Viral Disease Control and Prevention, China Center for Disease Control and Prevention	Song,J., Zhang,Y. and Xu,W.
EPI_ISL_2578060, EPI_ISL_2578061, EPI_ISL_2578062, EPI_ISL_2578063	Centre for Research And Knowledge Transfer in Biotechnology, University of Zagreb	Centre for Research And Knowledge Transfer in Biotechnology, University of Zagreb	Ivancic-Jelecki,J., Slovic,A., Ljubin-Sternak,S., Mlinaric-Galinovic,G. and Forcic,D.
EPI_ISL_2578064, EPI_ISL_2578065	WHO WPRO Measles Regional Reference Lab, Key Laboratory of Medical Virology Ministry of Health, National Institute for Viral Disease Control and Prevention, China Center for Disease Control and Prevention	WHO WPRO Measles Regional Reference Lab, Key Laboratory of Medical Virology Ministry of Health, National Institute for Viral Disease Control and Prevention, China Center for Disease Control and Prevention	Song,J., Zhang,Y. and Xu,W.
EPI_ISL_2578066	Centre for Research And Knowledge Transfer in Biotechnology, University of Zagreb	Centre for Research And Knowledge Transfer in Biotechnology, University of Zagreb	Ivancic-Jelecki,J., Slovic,A., Ljubin-Sternak,S., Mlinaric-Galinovic,G. and Forcic,D.
EPI_ISL_2578067	WHO WPRO Measles Regional Reference Lab, Key Laboratory of Medical Virology Ministry of Health, National Institute for Viral Disease Control and Prevention, China Center for Disease Control and Prevention	WHO WPRO Measles Regional Reference Lab, Key Laboratory of Medical Virology Ministry of Health, National Institute for Viral Disease Control and Prevention, China Center for Disease Control and Prevention	Song,J., Zhang,Y. and Xu,W.
EPI_ISL_2578068, EPI_ISL_2578069	Centre for Research And Knowledge Transfer in Biotechnology, University of Zagreb	Centre for Research And Knowledge Transfer in Biotechnology, University of Zagreb	Ivancic-Jelecki,J., Slovic,A., Ljubin-Sternak,S., Mlinaric-Galinovic,G. and Forcic,D.
EPI_ISL_2578070	WHO WPRO Measles Regional Reference Lab, Key Laboratory of Medical Virology Ministry of Health, National Institute for Viral Disease Control and Prevention, China Center for Disease Control and Prevention	WHO WPRO Measles Regional Reference Lab, Key Laboratory of Medical Virology Ministry of Health, National Institute for Viral Disease Control and Prevention, China Center for Disease Control and Prevention	Song,J., Zhang,Y. and Xu,W.
EPI_ISL_2578071	Centre for Research And Knowledge Transfer in Biotechnology, University of Zagreb	Centre for Research And Knowledge Transfer in Biotechnology, University of Zagreb	Ivancic-Jelecki,J., Slovic,A., Ljubin-Sternak,S., Mlinaric-Galinovic,G. and Forcic,D.
EPI_ISL_2578072	Department of Public Health Laboratory Sciences, West China School of Public Health, Sichuan University	Department of Public Health Laboratory Sciences, West China School of Public Health, Sichuan University	Hu,W.P. and Pei,F.X.
EPI_ISL_2578073, EPI_ISL_2578074, EPI_ISL_2578075	WHO WPRO Measles Regional Reference Lab, Key Laboratory of Medical Virology Ministry of Health, National Institute for Viral Disease Control and Prevention, China Center for Disease Control and Prevention	WHO WPRO Measles Regional Reference Lab, Key Laboratory of Medical Virology Ministry of Health, National Institute for Viral Disease Control and Prevention, China Center for Disease Control and Prevention	Song,J., Zhang,Y. and Xu,W.
EPI_ISL_2578076	Centre for Research And Knowledge Transfer in Biotechnology, University of Zagreb	Centre for Research And Knowledge Transfer in Biotechnology, University of Zagreb	Ivancic-Jelecki,J., Slovic,A., Ljubin-Sternak,S., Mlinaric-Galinovic,G. and Forcic,D.
EPI_ISL_2578077, EPI_ISL_2578078, EPI_ISL_2578079, EPI_ISL_2578080	WHO WPRO Measles Regional Reference Lab, Key Laboratory of Medical Virology Ministry of Health, National Institute for Viral Disease Control and Prevention, China Center for Disease Control and Prevention	WHO WPRO Measles Regional Reference Lab, Key Laboratory of Medical Virology Ministry of Health, National Institute for Viral Disease Control and Prevention, China Center for Disease Control and Prevention	Song,J., Zhang,Y. and Xu,W.
EPI_ISL_2578081	Centre for Research And Knowledge Transfer in Biotechnology, University of Zagreb	Centre for Research And Knowledge Transfer in Biotechnology, University of Zagreb	Ivancic-Jelecki,J., Slovic,A., Ljubin-Sternak,S., Mlinaric-Galinovic,G. and Forcic,D.
EPI_ISL_2578082, EPI_ISL_2578083	WHO WPRO Measles Regional Reference Lab, Key Laboratory of Medical Virology Ministry of Health, National Institute for Viral Disease Control and Prevention, China Center for Disease Control and Prevention	WHO WPRO Measles Regional Reference Lab, Key Laboratory of Medical Virology Ministry of Health, National Institute for Viral Disease Control and Prevention, China Center for Disease Control and Prevention	Song,J., Zhang,Y. and Xu,W.
EPI_ISL_2578084	Epidemiology and Demography, KEMRI-Wellcome Trust Research Programme	Epidemiology and Demography, KEMRI-Wellcome Trust Research Programme	Kamau,E.M., Agoti,C.N., Owor,B., Lewa,C., Bett,A., Oketch,J., Cane,P.A. and Nokes,J.D.
EPI_ISL_2578085, EPI_ISL_2578086, EPI_ISL_2578087	WHO WPRO Measles Regional Reference Lab, Key Laboratory of Medical Virology Ministry of Health, National Institute for Viral Disease Control and Prevention, China Center for Disease Control and Prevention	WHO WPRO Measles Regional Reference Lab, Key Laboratory of Medical Virology Ministry of Health, National Institute for Viral Disease Control and Prevention, China Center for Disease Control and Prevention	Song,J., Zhang,Y. and Xu,W.
EPI_ISL_2578088	Centre for Research And Knowledge Transfer in Biotechnology, University of Zagreb	Centre for Research And Knowledge Transfer in Biotechnology, University of Zagreb	Ivancic-Jelecki,J., Slovic,A., Ljubin-Sternak,S., Mlinaric-Galinovic,G. and Forcic,D.
EPI_ISL_2578089, EPI_ISL_2578090, EPI_ISL_2578091	WHO WPRO Measles Regional Reference Lab, Key Laboratory of Medical Virology Ministry of Health, National Institute for Viral Disease Control and Prevention, China Center for Disease Control and Prevention	WHO WPRO Measles Regional Reference Lab, Key Laboratory of Medical Virology Ministry of Health, National Institute for Viral Disease Control and Prevention, China Center for Disease Control and Prevention	Song,J., Zhang,Y. and Xu,W.
EPI_ISL_2578092, EPI_ISL_2578093	Centre for Research And Knowledge Transfer in Biotechnology, University of Zagreb	Centre for Research And Knowledge Transfer in Biotechnology, University of Zagreb	Ivancic-Jelecki,J., Slovic,A., Ljubin-Sternak,S., Mlinaric-Galinovic,G. and Forcic,D.
EPI_ISL_2578094, EPI_ISL_2578095, EPI_ISL_2578096	WHO WPRO Measles Regional Reference Lab, Key Laboratory of Medical Virology Ministry of Health, National Institute for Viral Disease Control and Prevention, China Center for Disease Control and Prevention	WHO WPRO Measles Regional Reference Lab, Key Laboratory of Medical Virology Ministry of Health, National Institute for Viral Disease Control and Prevention, China Center for Disease Control and Prevention	Song,J., Zhang,Y. and Xu,W.
EPI_ISL_2578097	Centre for Research And Knowledge Transfer in Biotechnology, University of Zagreb	Centre for Research And Knowledge Transfer in Biotechnology, University of Zagreb	Ivancic-Jelecki,J., Slovic,A., Ljubin-Sternak,S., Mlinaric-Galinovic,G. and Forcic,D.
EPI_ISL_2578098, EPI_ISL_2578099, EPI_ISL_2578100	WHO WPRO Measles Regional Reference Lab, Key Laboratory of Medical Virology Ministry of Health, National Institute for Viral Disease Control and Prevention, China Center for Disease Control and Prevention	WHO WPRO Measles Regional Reference Lab, Key Laboratory of Medical Virology Ministry of Health, National Institute for Viral Disease Control and Prevention, China Center for Disease Control and Prevention	Song,J., Zhang,Y. and Xu,W.
EPI_ISL_2578101	Virology Section, Department of Microbiology, King George's Medical University	Virology Section, Department of Microbiology, King George's Medical University	Singh,A.K., Jain,A., Jain,B., Dangi,T., Verma,A.K., Dwivedi,M. and Kushwaha,R.
EPI_ISL_2578102, EPI_ISL_2578103, EPI_ISL_2578104, EPI_ISL_2578105	WHO WPRO Measles Regional Reference Lab, Key Laboratory of Medical Virology Ministry of Health, National Institute for Viral Disease Control and Prevention, China Center for Disease Control and Prevention	WHO WPRO Measles Regional Reference Lab, Key Laboratory of Medical Virology Ministry of Health, National Institute for Viral Disease Control and Prevention, China Center for Disease Control and Prevention	Song,J., Zhang,Y. and Xu,W.
EPI_ISL_2578106, EPI_ISL_2578107, EPI_ISL_2578108, EPI_ISL_2578109, EPI_ISL_2578110, EPI_ISL_2578111, EPI_ISL_2578112, EPI_ISL_2578113, EPI_ISL_2578114, EPI_ISL_2578115, EPI_ISL_2578116, EPI_ISL_2578117, EPI_ISL_2578118, EPI_ISL_2578119, EPI_ISL_2578120, EPI_ISL_2578121, EPI_ISL_2578122, EPI_ISL_2578123, EPI_ISL_2578124, EPI_ISL_2578125, EPI_ISL_2578126, EPI_ISL_2578127, EPI_ISL_2578128, EPI_ISL_2578129, EPI_ISL_2578130, EPI_ISL_2578131, EPI_ISL_2578132, EPI_ISL_2578133, EPI_ISL_2578134, EPI_ISL_2578135, EPI_ISL_2578136, EPI_ISL_2578137			
see above	Virology Laboratory, Dr. Ricardo Gutierrez Children Hospital	Virology Laboratory, Dr. Ricardo Gutierrez Children Hospital	Rojo,G.L., Goya,S., Orellana,M., Sancilio,A., Rodriguez Perez,A., Montali,C., Garcia,C., Sosa,L., Musto,A., Hamilton,G., Alvarez,D., Castello,A. and Viegas,M.
EPI_ISL_2578138, EPI_ISL_2578139	Michiko Okamoto Tohoku University Graduate School of Medicine, Virology	Michiko Okamoto Tohoku University Graduate School of Medicine, Virology	Okamoto,M. and Dapat,C.
EPI_ISL_2578140, EPI_ISL_2578141, EPI_ISL_2578142, EPI_ISL_2578143, EPI_ISL_2578144, EPI_ISL_2578145, EPI_ISL_2578146, EPI_ISL_2578147	Virology Laboratory, Dr. Ricardo Gutierrez Children Hospital	Virology Laboratory, Dr. Ricardo Gutierrez Children Hospital	Rojo,G.L., Goya,S., Orellana,M., Sancilio,A., Rodriguez Perez,A., Montali,C., Garcia,C., Sosa,L., Musto,A., Hamilton,G., Alvarez,D., Castello,A. and Viegas,M.
EPI_ISL_2578148	Michiko Okamoto Tohoku University Graduate School of Medicine, Virology	Michiko Okamoto Tohoku University Graduate School of Medicine, Virology	Okamoto,M. and Dapat,C.
EPI_ISL_2578149	Virology Laboratory, Dr. Ricardo Gutierrez Children Hospital	Virology Laboratory, Dr. Ricardo Gutierrez Children Hospital	Rojo,G.L., Goya,S., Orellana,M., Sancilio,A., Rodriguez Perez,A., Montali,C., Garcia,C., Sosa,L., Musto,A., Hamilton,G., Alvarez,D., Castello,A. and Viegas,M.
EPI_ISL_2578150	Michiko Okamoto Tohoku University Graduate School of Medicine, Virology	Michiko Okamoto Tohoku University Graduate School of Medicine, Virology	Okamoto,M. and Dapat,C.
EPI_ISL_2578151	Virology Laboratory, Dr. Ricardo Gutierrez Children Hospital	Virology Laboratory, Dr. Ricardo Gutierrez Children Hospital	Rojo,G.L., Goya,S., Orellana,M., Sancilio,A., Rodriguez Perez,A., Montali,C., Garcia,C., Sosa,L., Musto,A., Hamilton,G., Alvarez,D., Castello,A. and Viegas,M.
EPI_ISL_2578152	Michiko Okamoto Tohoku University Graduate School of Medicine, Virology	Michiko Okamoto Tohoku University Graduate School of Medicine, Virology	Okamoto,M. and Dapat,C.
EPI_ISL_2578153	Virology Laboratory, Dr. Ricardo Gutierrez Children Hospital	Virology Laboratory, Dr. Ricardo Gutierrez Children Hospital	Rojo,G.L., Goya,S., Orellana,M., Sancilio,A., Rodriguez Perez,A., Montali,C., Garcia,C., Sosa,L., Musto,A., Hamilton,G., Alvarez,D., Castello,A. and Viegas,M.
EPI_ISL_2578154	Michiko Okamoto Graduate School of Medicine, Tohoku University, Virology	Michiko Okamoto Graduate School of Medicine, Tohoku University, Virology	Okamoto,M. and Oshitani,H.
EPI_ISL_2578155, EPI_ISL_2578156	Michiko Okamoto Tohoku University Graduate School of Medicine, Virology	Michiko Okamoto Tohoku University Graduate School of Medicine, Virology	Okamoto,M. and Dapat,C.
EPI_ISL_2578157	Michiko Okamoto Graduate School of Medicine, Tohoku University, Virology	Michiko Okamoto Graduate School of Medicine, Tohoku University, Virology	Okamoto,M. and Oshitani,H.
EPI_ISL_2578158, EPI_ISL_2578159	Michiko Okamoto Tohoku University Graduate School of Medicine, Virology	Michiko Okamoto Tohoku University Graduate School of Medicine, Virology	Okamoto,M. and Dapat,C.
EPI_ISL_2578160	Virology Laboratory, Dr. Ricardo Gutierrez Children Hospital	Virology Laboratory, Dr. Ricardo Gutierrez Children Hospital	Rojo,G.L., Goya,S., Orellana,M., Sancilio,A., Rodriguez Perez,A., Montali,C., Garcia,C., Sosa,L., Musto,A., Hamilton,G., Alvarez,D., Castello,A. and Viegas,M.
EPI_ISL_2578161	Marie Bashir Institute for Infectious Diseases and Biosecurity & Sydney Medical School, The University of Sydney, Westmead Institute for Medical Research	Marie Bashir Institute for Infectious Diseases and Biosecurity & Sydney Medical School, The University of Sydney, Westmead Institute for Medical Research	Eden,J.-S., Kok,J., Dwyer,D.E., Fernandez,M., Carter,I. and Holmes,E.C.
EPI_ISL_2578162, EPI_ISL_2578163	Michiko Okamoto Tohoku University Graduate School of Medicine, Virology	Michiko Okamoto Tohoku University Graduate School of Medicine, Virology	Okamoto,M. and Dapat,C.
EPI_ISL_2578164	Virology Laboratory, Dr. Ricardo Gutierrez Children Hospital	Virology Laboratory, Dr. Ricardo Gutierrez Children Hospital	Rojo,G.L., Goya,S., Orellana,M., Sancilio,A., Rodriguez Perez,A., Montali,C., Garcia,C., Sosa,L., Musto,A., Hamilton,G., Alvarez,D., Castello,A. and Viegas,M.
EPI_ISL_2578165	Centre for Virology, Pushpagiri Institute of Medical Science and Research Centre, Pushpagir Medical College Hospital	Centre for Virology, Pushpagiri Institute of Medical Science and Research Centre, Pushpagir Medical College Hospital	George,S., Jagan,O.A., Jose,S., Chandy,S., Bai,S. and Manoharan,A.
EPI_ISL_2578166	Department of Pediatrics, Center of Excellence in Clinical Virology, Faculty of	Department of Pediatrics, Center of Excellence in Clinical Virology, Faculty of	Thongpan,I., Mauleekoonphairoj,J., Vichiwattana,P., Korkong,S., Vongpunsawad,S. and Poovorawan,Y.

	Medicine, Chulalongkorn University	Medicine, Chulalongkorn University	
EPI_ISL_2578167	Michiko Okamoto Graduate School of Medicine, Tohoku University, Virology	Michiko Okamoto Graduate School of Medicine, Tohoku University, Virology	Okamoto,M. and Oshitani,H.
EPI_ISL_2578168	Epidemiology and Demography, KEMRI-Wellcome Trust Research Programme	Epidemiology and Demography, KEMRI-Wellcome Trust Research Programme	Kamau,E.M., Agoti,C.N., Owor,B., Lewa,C., Bett,A., Oketch,J., Cane,P.A. and Nokes,J.D.
EPI_ISL_2578169, EPI_ISL_2578170, EPI_ISL_2578171	Department of Pediatrics, Center of Excellence in Clinical Virology, Faculty of Medicine, Chulalongkorn University	Department of Pediatrics, Center of Excellence in Clinical Virology, Faculty of Medicine, Chulalongkorn University	Thongpan,I., Mauleekoonphairoj,J., Vichiwattana,P., Korkong,S., Vongpunsawad,S. and Poovorawan,Y.
EPI_ISL_2578172	WHO WPRO Measles Regional Reference Lab, Key Laboratory of Medical Virology Ministry of Health, National Institute for Viral Disease Control and Prevention, China Center for Disease Control and Prevention	WHO WPRO Measles Regional Reference Lab, Key Laboratory of Medical Virology Ministry of Health, National Institute for Viral Disease Control and Prevention, China Center for Disease Control and Prevention	Song,J., Zhang,Y. and Xu,W.
EPI_ISL_2578173	Epidemiology and Demography, KEMRI-Wellcome Trust Research Programme	Epidemiology and Demography, KEMRI-Wellcome Trust Research Programme	Kamau,E.M., Agoti,C.N., Owor,B., Lewa,C., Bett,A., Oketch,J., Cane,P.A. and Nokes,J.D.
EPI_ISL_2578174, EPI_ISL_2578175, EPI_ISL_2578176	Department of Pediatrics, Center of Excellence in Clinical Virology, Faculty of Medicine, Chulalongkorn University	Department of Pediatrics, Center of Excellence in Clinical Virology, Faculty of Medicine, Chulalongkorn University	Thongpan,I., Mauleekoonphairoj,J., Vichiwattana,P., Korkong,S., Vongpunsawad,S. and Poovorawan,Y.
EPI_ISL_2578177	WHO WPRO Measles Regional Reference Lab, Key Laboratory of Medical Virology Ministry of Health, National Institute for Viral Disease Control and Prevention, China Center for Disease Control and Prevention	WHO WPRO Measles Regional Reference Lab, Key Laboratory of Medical Virology Ministry of Health, National Institute for Viral Disease Control and Prevention, China Center for Disease Control and Prevention	Song,J., Zhang,Y. and Xu,W.
EPI_ISL_2578178, EPI_ISL_2578179, EPI_ISL_2578180	Epidemiology and Demography, KEMRI-Wellcome Trust Research Programme	Epidemiology and Demography, KEMRI-Wellcome Trust Research Programme	Kamau,E.M., Agoti,C.N., Owor,B., Lewa,C., Bett,A., Oketch,J., Cane,P.A. and Nokes,J.D.
EPI_ISL_2578181	Epidemiology and Demography, KEMRI-Wellcome Trust Research Programme	Epidemiology and Demography, KEMRI-Wellcome Trust Research Programme	Kamau,E.M., Agoti,C.N., Lewa,C.S., Cane,P.A., Bett,A., Medley,G.F. and Nokes,J.D.
EPI_ISL_2578182, EPI_ISL_2578183, EPI_ISL_2578184, EPI_ISL_2578185, EPI_ISL_2578186, EPI_ISL_2578187, EPI_ISL_2578188, EPI_ISL_2578189	Epidemiology and Demography, KEMRI-Wellcome Trust Research Programme	Epidemiology and Demography, KEMRI-Wellcome Trust Research Programme	Kamau,E.M., Agoti,C.N., Owor,B., Lewa,C., Bett,A., Oketch,J., Cane,P.A. and Nokes,J.D.
EPI_ISL_2578190	Department of Clinical Laboratory, Fujian Provincial Hospital	Department of Clinical Laboratory, Fujian Provincial Hospital	Su,Y., Wu,Y., Tian,R. and Liang,G.
EPI_ISL_2578191	WHO WPRO Measles Regional Reference Lab, Key Laboratory of Medical Virology Ministry of Health, National Institute for Viral Disease Control and Prevention, China Center for Disease Control and Prevention	WHO WPRO Measles Regional Reference Lab, Key Laboratory of Medical Virology Ministry of Health, National Institute for Viral Disease Control and Prevention, China Center for Disease Control and Prevention	Song,J., Zhang,Y. and Xu,W.
EPI_ISL_2578192, EPI_ISL_2578193, EPI_ISL_2578194, EPI_ISL_2578195	Epidemiology and Demography, KEMRI-Wellcome Trust Research Programme	Epidemiology and Demography, KEMRI-Wellcome Trust Research Programme	Kamau,E.M., Agoti,C.N., Owor,B., Lewa,C., Bett,A., Oketch,J., Cane,P.A. and Nokes,J.D.
EPI_ISL_2578196	Department of Clinical Laboratory, Fujian Provincial Hospital	Department of Clinical Laboratory, Fujian Provincial Hospital	Su,Y., Wu,Y., Tian,R. and Liang,G.
EPI_ISL_2578197, EPI_ISL_2578198, EPI_ISL_2578199, EPI_ISL_2578200, EPI_ISL_2578201, EPI_ISL_2578202, EPI_ISL_2578203, EPI_ISL_2578204, EPI_ISL_2578205	Epidemiology and Demography, KEMRI-Wellcome Trust Research Programme	Epidemiology and Demography, KEMRI-Wellcome Trust Research Programme	Kamau,E.M., Agoti,C.N., Owor,B., Lewa,C., Bett,A., Oketch,J., Cane,P.A. and Nokes,J.D.
EPI_ISL_2578206	Centre for Infectious Diseases Research, Diagnostics and laboratory Surveillance, National Institute for Public Health and the Environment	Centre for Infectious Diseases Research, Diagnostics and laboratory Surveillance, National Institute for Public Health and the Environment	Meijer,A. and Overduin,P.
EPI_ISL_2578207	Epidemiology and Demography, KEMRI-Wellcome Trust Research Programme	Epidemiology and Demography, KEMRI-Wellcome Trust Research Programme	Kamau,E.M., Agoti,C.N., Lewa,C.S., Cane,P.A., Bett,A., Medley,G.F. and Nokes,J.D.
EPI_ISL_2578208, EPI_ISL_2578209	Epidemiology and Demography, KEMRI-Wellcome Trust Research Programme	Epidemiology and Demography, KEMRI-Wellcome Trust Research Programme	Kamau,E.M., Agoti,C.N., Owor,B., Lewa,C., Bett,A., Oketch,J., Cane,P.A. and Nokes,J.D.
EPI_ISL_2578210	Centre for Infectious Diseases Research, Diagnostics and laboratory Surveillance, National Institute for Public Health and the Environment	Centre for Infectious Diseases Research, Diagnostics and laboratory Surveillance, National Institute for Public Health and the Environment	Meijer,A. and Overduin,P.
EPI_ISL_2578211, EPI_ISL_2578212, EPI_ISL_2578213	Virology Laboratory, Dr. Ricardo Gutierrez Children Hospital	Virology Laboratory, Dr. Ricardo Gutierrez Children Hospital	Rojo,G.L., Goya,S., Orellana,M., Sancilio,A., Rodriguez Perez,A., Montali,C., Garcia,C., Sosa,L., Musto,A., Hamilton,G., Alvarez,D., Castello,A. and Viegas,M.
EPI_ISL_2578214	Epidemiology and Demography, KEMRI-Wellcome Trust Research Programme	Epidemiology and Demography, KEMRI-Wellcome Trust Research Programme	Kamau,E.M., Agoti,C.N., Lewa,C.S., Cane,P.A., Bett,A., Medley,G.F. and Nokes,J.D.
EPI_ISL_2578215, EPI_ISL_2578216	Virology Laboratory, Dr. Ricardo Gutierrez Children Hospital	Virology Laboratory, Dr. Ricardo Gutierrez Children Hospital	Rojo,G.L., Goya,S., Orellana,M., Sancilio,A., Rodriguez Perez,A., Montali,C., Garcia,C., Sosa,L., Musto,A., Hamilton,G., Alvarez,D., Castello,A. and Viegas,M.
EPI_ISL_2578217	Centre for Infectious Diseases Research, Diagnostics and laboratory Surveillance, National Institute for Public Health and the Environment	Centre for Infectious Diseases Research, Diagnostics and laboratory Surveillance, National Institute for Public Health and the Environment	Meijer,A. and Overduin,P.
EPI_ISL_2578218, EPI_ISL_2578219, EPI_ISL_2578220, EPI_ISL_2578221, EPI_ISL_2578222, EPI_ISL_2578223, EPI_ISL_2578224, EPI_ISL_2578225	Virology Laboratory, Dr. Ricardo Gutierrez Children Hospital	Virology Laboratory, Dr. Ricardo Gutierrez Children Hospital	Rojo,G.L., Goya,S., Orellana,M., Sancilio,A., Rodriguez Perez,A., Montali,C., Garcia,C., Sosa,L., Musto,A., Hamilton,G., Alvarez,D., Castello,A. and Viegas,M.
EPI_ISL_2578226	Epidemiology and Demography, KEMRI-Wellcome Trust Research Programme	Epidemiology and Demography, KEMRI-Wellcome Trust Research Programme	Kamau,E.M., Agoti,C.N., Owor,B., Lewa,C., Bett,A., Oketch,J., Cane,P.A. and Nokes,J.D.
EPI_ISL_2578227, EPI_ISL_2578228, EPI_ISL_2578229, EPI_ISL_2578230	Virology Laboratory, Dr. Ricardo Gutierrez Children Hospital	Virology Laboratory, Dr. Ricardo Gutierrez Children Hospital	Rojo,G.L., Goya,S., Orellana,M., Sancilio,A., Rodriguez Perez,A., Montali,C., Garcia,C., Sosa,L., Musto,A., Hamilton,G., Alvarez,D., Castello,A. and Viegas,M.
EPI_ISL_2578231, EPI_ISL_2578232	Epidemiology and Demography, KEMRI-Wellcome Trust Research Programme	Epidemiology and Demography, KEMRI-Wellcome Trust Research Programme	Kamau,E.M., Agoti,C.N., Owor,B., Lewa,C., Bett,A., Oketch,J., Cane,P.A. and Nokes,J.D.
EPI_ISL_2578233	Department of Pediatrics, Center of Excellence in Clinical Virology, Faculty of Medicine, Chulalongkorn University	Department of Pediatrics, Center of Excellence in Clinical Virology, Faculty of Medicine, Chulalongkorn University	Thongpan,I., Mauleekoonphairoj,J., Vichiwattana,P., Korkong,S., Vongpunsawad,S. and Poovorawan,Y.
EPI_ISL_2578234	Centre for Infectious Diseases Virology, University of Heidelberg	Centre for Infectious Diseases Virology, University of Heidelberg	Ihling,C.M., Schnitzler,P., Heinrich,N., Mangu,C., Sudi,L., Souares,A., Gies,S., Sie,A., Coulibaly,B., Ouedraogo,A.T., Mordmueller,B., Held,J., Adegnika,A.A., Fernandez,J.F., Eckerle,I., May,J., Hogan,B., Eibach,D., Owusu-Dabo,E. and Tabatabai,J.
EPI_ISL_2578235	Virology Laboratory, Dr. Ricardo Gutierrez Children Hospital	Virology Laboratory, Dr. Ricardo Gutierrez Children Hospital	Rojo,G.L., Goya,S., Orellana,M., Sancilio,A., Rodriguez Perez,A., Montali,C., Garcia,C., Sosa,L., Musto,A., Hamilton,G., Alvarez,D., Castello,A. and Viegas,M.
EPI_ISL_2578236	Epidemiology and Demography, KEMRI-Wellcome Trust Research Programme	Epidemiology and Demography, KEMRI-Wellcome Trust Research Programme	Kamau,E.M., Agoti,C.N., Lewa,C.S., Cane,P.A., Bett,A., Medley,G.F. and Nokes,J.D.
EPI_ISL_2578237	Epidemiology and Demography, KEMRI-Wellcome Trust Research Programme	Epidemiology and Demography, KEMRI-Wellcome Trust Research Programme	Kamau,E.M., Agoti,C.N., Owor,B., Lewa,C., Bett,A., Oketch,J., Cane,P.A. and Nokes,J.D.
EPI_ISL_2578238	Centre for Infectious Diseases Virology, University of Heidelberg	Centre for Infectious Diseases Virology, University of Heidelberg	Ihling,C.M., Schnitzler,P., Heinrich,N., Mangu,C., Sudi,L., Souares,A., Gies,S., Sie,A., Coulibaly,B., Ouedraogo,A.T., Mordmueller,B., Held,J., Adegnika,A.A., Fernandez,J.F., Eckerle,I., May,J., Hogan,B., Eibach,D., Owusu-Dabo,E. and Tabatabai,J.
EPI_ISL_2578239	Marie Bashir Institute for Infectious Diseases and Biosecurity & Sydney Medical School, The University of Sydney, Westmead Institute for Medical Research	Marie Bashir Institute for Infectious Diseases and Biosecurity & Sydney Medical School, The University of Sydney, Westmead Institute for Medical Research	Eden,J.-S., Kok,J., Dwyer,D.E., Fernandez,M., Carter,I. and Holmes,E.C.
EPI_ISL_2578240	Centre for Infectious Diseases Research, Diagnostics and laboratory Surveillance, National Institute for Public Health and the Environment	Centre for Infectious Diseases Research, Diagnostics and laboratory Surveillance, National Institute for Public Health and the Environment	Meijer,A. and Overduin,P.
EPI_ISL_2578241, EPI_ISL_2578242, EPI_ISL_2578243, EPI_ISL_2578244, EPI_ISL_2578245, EPI_ISL_2578246, EPI_ISL_2578247, EPI_ISL_2578248, EPI_ISL_2578249, EPI_ISL_2578250	Department of Pediatrics, Center of Excellence in Clinical Virology, Faculty of Medicine, Chulalongkorn University	Department of Pediatrics, Center of Excellence in Clinical Virology, Faculty of Medicine, Chulalongkorn University	Thongpan,I., Mauleekoonphairoj,J., Vichiwattana,P., Korkong,S., Vongpunsawad,S. and Poovorawan,Y.
EPI_ISL_2578251, EPI_ISL_2578252, EPI_ISL_2578253, EPI_ISL_2578254, EPI_ISL_2578255	Michiko Okamoto Tohoku University Graduate School of Medicine, Virology	Michiko Okamoto Tohoku University Graduate School of Medicine, Virology	Okamoto,M. and Dapat,C.
EPI_ISL_2578256	Centre for Infectious Diseases Research, Diagnostics and laboratory Surveillance, National Institute for Public Health and the Environment	Centre for Infectious Diseases Research, Diagnostics and laboratory Surveillance, National Institute for Public Health and the Environment	Meijer,A. and Overduin,P.
EPI_ISL_2578257, EPI_ISL_2578258	Michiko Okamoto Tohoku University Graduate School of Medicine, Virology	Michiko Okamoto Tohoku University Graduate School of Medicine, Virology	Okamoto,M. and Dapat,C.
EPI_ISL_2578259	Department of Pediatrics, Center of Excellence in Clinical Virology, Faculty of Medicine, Chulalongkorn University	Department of Pediatrics, Center of Excellence in Clinical Virology, Faculty of Medicine, Chulalongkorn University	Thongpan,I., Mauleekoonphairoj,J., Vichiwattana,P., Korkong,S., Vongpunsawad,S. and Poovorawan,Y.
EPI_ISL_2578261, EPI_ISL_2578262, EPI_ISL_2578263, EPI_ISL_2578264, EPI_ISL_2578265	Microbiology, Hospital Universitari Vall d'Hebron	Microbiology, Hospital Universitari Vall d'Hebron	Gimferrer,L., Martin,Md.C., Pumarola,T. and Anton,A.
EPI_ISL_2578266	Epidemiology and Demography, KEMRI-Wellcome Trust Research Programme	Epidemiology and Demography, KEMRI-Wellcome Trust Research Programme	Kamau,E.M., Agoti,C.N., Lewa,C.S., Cane,P.A., Bett,A., Medley,G.F. and Nokes,J.D.
EPI_ISL_2578267, EPI_ISL_2578268, EPI_ISL_2578269, EPI_ISL_2578270, EPI_ISL_2578271, EPI_ISL_2578272, EPI_ISL_2578274, EPI_ISL_2578275	Microbiology, Hospital Universitari Vall d'Hebron	Microbiology, Hospital Universitari Vall d'Hebron	Gimferrer,L., Martin,Md.C., Pumarola,T. and Anton,A.
EPI_ISL_2578276	Epidemiology and Demography, KEMRI-Wellcome Trust Research Programme	Epidemiology and Demography, KEMRI-Wellcome Trust Research Programme	Kamau,E.M., Agoti,C.N., Lewa,C.S., Cane,P.A., Bett,A., Medley,G.F. and Nokes,J.D.
EPI_ISL_2578277	Epidemiology and Demography, KEMRI-Wellcome Trust Research Programme	Epidemiology and Demography, KEMRI-Wellcome Trust Research Programme	Kamau,E.M., Agoti,C.N., Owor,B., Lewa,C., Bett,A., Oketch,J., Cane,P.A. and Nokes,J.D.
EPI_ISL_2578278	Microbiology, Hospital Universitari Vall d'Hebron	Microbiology, Hospital Universitari Vall d'Hebron	Gimferrer,L., Martin,Md.C., Pumarola,T. and Anton,A.
EPI_ISL_2578279	Epidemiology and Demography, KEMRI-Wellcome Trust Research Programme	Epidemiology and Demography, KEMRI-Wellcome Trust Research Programme	Kamau,E.M., Agoti,C.N., Lewa,C.S., Cane,P.A., Bett,A., Medley,G.F. and Nokes,J.D.
EPI_ISL_2578280, EPI_ISL_2578281, EPI_ISL_2578282	Microbiology, Hospital Universitari Vall d'Hebron	Microbiology, Hospital Universitari Vall d'Hebron	Gimferrer,L., Martin,Md.C., Pumarola,T. and Anton,A.
EPI_ISL_2578283	Epidemiology and Demography, KEMRI-Wellcome Trust Research Programme	Epidemiology and Demography, KEMRI-Wellcome Trust Research Programme	Kamau,E.M., Agoti,C.N., Owor,B., Lewa,C., Bett,A., Oketch,J., Cane,P.A. and Nokes,J.D.
EPI_ISL_2578284, EPI_ISL_2578285	Microbiology, Hospital Universitari Vall d'Hebron	Microbiology, Hospital Universitari Vall d'Hebron	Gimferrer,L., Martin,Md.C., Pumarola,T. and Anton,A.
EPI_ISL_2578286	Epidemiology and Demography, KEMRI-Wellcome Trust Research Programme	Epidemiology and Demography, KEMRI-Wellcome Trust Research Programme	Kamau,E.M., Agoti,C.N., Owor,B., Lewa,C., Bett,A., Oketch,J., Cane,P.A. and Nokes,J.D.
EPI_ISL_2578287	Microbiology, Hospital Universitari Vall d'Hebron	Microbiology, Hospital Universitari Vall d'Hebron	Gimferrer,L., Martin,Md.C., Pumarola,T. and Anton,A.

EPI_ISL_2578288, EPI_ISL_2578290	Epidemiology and Demography, KEMRI-Wellcome Trust Research Programme	Epidemiology and Demography, KEMRI-Wellcome Trust Research Programme	Kamau,E.M., Agoti,C.N., Owor,B., Lewa,C., Bett,A., Oketch,J., Cane,P.A. and Nokes,J.D.
EPI_ISL_2578291, EPI_ISL_2578292	Microbiology, Hospital Universitari Vall d'Hebron	Microbiology, Hospital Universitari Vall d'Hebron	Gimferrer,L., Martin,Md.C., Pumarola,T. and Anton,A.
EPI_ISL_2578293	Epidemiology and Demography, KEMRI-Wellcome Trust Research Programme	Epidemiology and Demography, KEMRI-Wellcome Trust Research Programme	Kamau,E.M., Agoti,C.N., Lewa,C.S., Cane,P.A., Bett,A., Medley,G.F. and Nokes,J.D.
EPI_ISL_2578294, EPI_ISL_2578295, EPI_ISL_2578296	Epidemiology and Demography, KEMRI-Wellcome Trust Research Programme	Epidemiology and Demography, KEMRI-Wellcome Trust Research Programme	Kamau,E.M., Agoti,C.N., Owor,B., Lewa,C., Bett,A., Oketch,J., Cane,P.A. and Nokes,J.D.
EPI_ISL_2578297	Microbiology, Hospital Universitari Vall d'Hebron	Microbiology, Hospital Universitari Vall d'Hebron	Gimferrer,L., Martin,Md.C., Pumarola,T. and Anton,A.
EPI_ISL_2578298	Epidemiology and Demography, KEMRI-Wellcome Trust Research Programme	Epidemiology and Demography, KEMRI-Wellcome Trust Research Programme	Kamau,E.M., Agoti,C.N., Lewa,C.S., Cane,P.A., Bett,A., Medley,G.F. and Nokes,J.D.
EPI_ISL_2578299, EPI_ISL_2578300	Epidemiology and Demography, KEMRI-Wellcome Trust Research Programme	Epidemiology and Demography, KEMRI-Wellcome Trust Research Programme	Kamau,E.M., Agoti,C.N., Owor,B., Lewa,C., Bett,A., Oketch,J., Cane,P.A. and Nokes,J.D.
EPI_ISL_2578301, EPI_ISL_2578303, EPI_ISL_2578304, EPI_ISL_2578305	Epidemiology and Demography, KEMRI-Wellcome Trust Research Programme	Epidemiology and Demography, KEMRI-Wellcome Trust Research Programme	Kamau,E.M., Agoti,C.N., Lewa,C.S., Cane,P.A., Nokes,J.D. and Agoti,C.N.
EPI_ISL_2578306	Microbiology, Hospital Universitari Vall d'Hebron	Microbiology, Hospital Universitari Vall d'Hebron	Gimferrer,L., Martin,Md.C., Pumarola,T. and Anton,A.
EPI_ISL_2578307, EPI_ISL_2578308, EPI_ISL_2578309	Epidemiology and Demography, KEMRI-Wellcome Trust Research Programme	Epidemiology and Demography, KEMRI-Wellcome Trust Research Programme	Kamau,E.M., Agoti,C.N., Lewa,C.S., Cane,P.A., Nokes,J.D. and Agoti,C.N.
EPI_ISL_2578310	Microbiology, Hospital Universitari Vall d'Hebron	Microbiology, Hospital Universitari Vall d'Hebron	Gimferrer,L., Martin,Md.C., Pumarola,T. and Anton,A.
EPI_ISL_2578311, EPI_ISL_2578312, EPI_ISL_2578313, EPI_ISL_2578314, EPI_ISL_2578315, EPI_ISL_2578316	Epidemiology and Demography, KEMRI-Wellcome Trust Research Programme	Epidemiology and Demography, KEMRI-Wellcome Trust Research Programme	Kamau,E.M., Agoti,C.N., Lewa,C.S., Cane,P.A., Nokes,J.D. and Agoti,C.N.
EPI_ISL_2578318, EPI_ISL_2578319, EPI_ISL_2578320	Microbiology, Hospital Universitari Vall d'Hebron	Microbiology, Hospital Universitari Vall d'Hebron	Gimferrer,L., Martin,Md.C., Pumarola,T. and Anton,A.
EPI_ISL_2578321, EPI_ISL_2578322, EPI_ISL_2578323	Epidemiology and Demography, KEMRI-Wellcome Trust Research Programme	Epidemiology and Demography, KEMRI-Wellcome Trust Research Programme	Kamau,E.M., Agoti,C.N., Lewa,C.S., Cane,P.A., Nokes,J.D. and Agoti,C.N.
EPI_ISL_2578324, EPI_ISL_2578325, EPI_ISL_2578326, EPI_ISL_2578327	Microbiology, Hospital Universitari Vall d'Hebron	Microbiology, Hospital Universitari Vall d'Hebron	Gimferrer,L., Martin,Md.C., Pumarola,T. and Anton,A.
EPI_ISL_2578328, EPI_ISL_2578329, EPI_ISL_2578330	Epidemiology and Demography, KEMRI-Wellcome Trust Research Programme	Epidemiology and Demography, KEMRI-Wellcome Trust Research Programme	Kamau,E.M., Agoti,C.N., Lewa,C.S., Cane,P.A., Nokes,J.D. and Agoti,C.N.
EPI_ISL_2578331	Microbiology, Hospital Universitari Vall d'Hebron	Microbiology, Hospital Universitari Vall d'Hebron	Gimferrer,L., Martin,Md.C., Pumarola,T. and Anton,A.
EPI_ISL_2578332	Epidemiology and Demography, KEMRI-Wellcome Trust Research Programme	Epidemiology and Demography, KEMRI-Wellcome Trust Research Programme	Kamau,E.M., Agoti,C.N., Lewa,C.S., Cane,P.A., Nokes,J.D. and Agoti,C.N.
EPI_ISL_2578334	Epidemiology and Demography, KEMRI-Wellcome Trust Research Programme	Epidemiology and Demography, KEMRI-Wellcome Trust Research Programme	Kamau,E.M., Agoti,C.N., Owor,B., Lewa,C., Bett,A., Oketch,J., Cane,P.A. and Nokes,J.D.
EPI_ISL_2578335, EPI_ISL_2578336	Epidemiology and Demography, KEMRI-Wellcome Trust Research Programme	Epidemiology and Demography, KEMRI-Wellcome Trust Research Programme	Kamau,E.M., Agoti,C.N., Lewa,C.S., Cane,P.A., Nokes,J.D. and Agoti,C.N.
EPI_ISL_2578337, EPI_ISL_2578338	Epidemiology and Demography, KEMRI-Wellcome Trust Research Programme	Epidemiology and Demography, KEMRI-Wellcome Trust Research Programme	Kamau,E.M., Agoti,C.N., Owor,B., Lewa,C., Bett,A., Oketch,J., Cane,P.A. and Nokes,J.D.
EPI_ISL_2578339	Epidemiology and Demography, KEMRI-Wellcome Trust Research Programme	Epidemiology and Demography, KEMRI-Wellcome Trust Research Programme	Kamau,E.M., Agoti,C.N., Lewa,C.S., Cane,P.A., Nokes,J.D. and Agoti,C.N.
EPI_ISL_2578340	Epidemiology and Demography, KEMRI-Wellcome Trust Research Programme	Epidemiology and Demography, KEMRI-Wellcome Trust Research Programme	Kamau,E.M., Agoti,C.N., Lewa,C.S., Cane,P.A., Bett,A., Medley,G.F. and Nokes,J.D.
EPI_ISL_2578341, EPI_ISL_2578342	Laboratorios de Virus Respiratorio e do Sarampo, Instituto Oswaldo Cruz	Laboratorios de Virus Respiratorio e do Sarampo, Instituto Oswaldo Cruz	Resende,P.
EPI_ISL_2578343, EPI_ISL_2578344, EPI_ISL_2578345, EPI_ISL_2578346, EPI_ISL_2578347	Epidemiology and Demography, KEMRI-Wellcome Trust Research Programme	Epidemiology and Demography, KEMRI-Wellcome Trust Research Programme	Kamau,E.M., Agoti,C.N., Lewa,C.S., Cane,P.A., Nokes,J.D. and Agoti,C.N.
EPI_ISL_2578349	Department of Pediatrics, Center of Excellence in Clinical Virology, Chulalongkorn	Department of Pediatrics, Center of Excellence in Clinical Virology, Chulalongkorn	Thongpan,I.
EPI_ISL_2578350	Microbiology, Hospital Universitari Vall d'Hebron	Microbiology, Hospital Universitari Vall d'Hebron	Gimferrer,L., Martin,Md.C., Pumarola,T. and Anton,A.
EPI_ISL_2578351	Department of Pediatrics, Center of Excellence in Clinical Virology, Chulalongkorn	Department of Pediatrics, Center of Excellence in Clinical Virology, Chulalongkorn	Thongpan,I.
EPI_ISL_2578352	J. Craig Venter Institute	J. Craig Venter Institute	Tan,G., Pickett,B., Fedorova,N., Amedeo,P., Hu,L., Christensen,J., Miller,J., Durbin,A., Williams,T., Arumemi,F., Cadiz,C., Alanis,R., Balmseda,A., Wiliams,T., Schiller,A., Patel,M., Kubale,J. and Gordon,A.
EPI_ISL_2578353, EPI_ISL_2578354, EPI_ISL_2578355, EPI_ISL_2578356, EPI_ISL_2578357, EPI_ISL_2578358, EPI_ISL_2578359, EPI_ISL_2578360	Department of Pediatrics, Center of Excellence in Clinical Virology, Chulalongkorn	Department of Pediatrics, Center of Excellence in Clinical Virology, Chulalongkorn	Thongpan,I.
EPI_ISL_2578361, EPI_ISL_2578362	J. Craig Venter Institute	J. Craig Venter Institute	Tan,G., Pickett,B., Fedorova,N., Amedeo,P., Hu,L., Christensen,J., Miller,J., Durbin,A., Williams,T., Arumemi,F., Cadiz,C., Alanis,R., Balmseda,A., Wiliams,T., Schiller,A., Patel,M., Kubale,J. and Gordon,A.
EPI_ISL_2578363	Microbiology, Hospital Universitari Vall d'Hebron	Microbiology, Hospital Universitari Vall d'Hebron	Gimferrer,L., Martin,Md.C., Pumarola,T. and Anton,A.
EPI_ISL_2578365	J. Craig Venter Institute	J. Craig Venter Institute	Tan,G., Pickett,B., Fedorova,N., Amedeo,P., Hu,L., Christensen,J., Miller,J., Durbin,A., Williams,T., Arumemi,F., Cadiz,C., Alanis,R., Balmseda,A., Wiliams,T., Schiller,A., Patel,M., Kubale,J. and Gordon,A.
EPI_ISL_2578366, EPI_ISL_2578367, EPI_ISL_2578368, EPI_ISL_2578369, EPI_ISL_2578370, EPI_ISL_2578371, EPI_ISL_2578372, EPI_ISL_2578373, EPI_ISL_2578374, EPI_ISL_2578375, EPI_ISL_2578376, EPI_ISL_2578377, EPI_ISL_2578378, EPI_ISL_2578380			
see above	Microbiology, Hospital Universitari Vall d'Hebron	Microbiology, Hospital Universitari Vall d'Hebron	Gimferrer,L., Martin,Md.C., Pumarola,T. and Anton,A.
EPI_ISL_2578381	Marie Bashir Institute for Infectious Diseases and Biosecurity & Sydney Medical School, The University of Sydney, Westmead Institute for Medical Research	Marie Bashir Institute for Infectious Diseases and Biosecurity & Sydney Medical School, The University of Sydney, Westmead Institute for Medical Research	Eden,J.-S., Kok,J., Dwyer,D.E., Fernandez,M., Carter,I. and Holmes,E.C.
EPI_ISL_2578382, EPI_ISL_2578383, EPI_ISL_2578384, EPI_ISL_2578385, EPI_ISL_2578386, EPI_ISL_2578387, EPI_ISL_2578388	Microbiology, Hospital Universitari Vall d'Hebron	Microbiology, Hospital Universitari Vall d'Hebron	Gimferrer,L., Martin,Md.C., Pumarola,T. and Anton,A.
EPI_ISL_2578389, EPI_ISL_2578390	Epidemiology and Demography, KEMRI-Wellcome Trust Research Programme	Epidemiology and Demography, KEMRI-Wellcome Trust Research Programme	Kamau,E.M., Agoti,C.N., Lewa,C.S., Cane,P.A., Bett,A., Medley,G.F. and Nokes,J.D.
EPI_ISL_2578391, EPI_ISL_2578392, EPI_ISL_2578393, EPI_ISL_2578394, EPI_ISL_2578396, EPI_ISL_2578397	Microbiology, Hospital Universitari Vall d'Hebron	Microbiology, Hospital Universitari Vall d'Hebron	Gimferrer,L., Martin,Md.C., Pumarola,T. and Anton,A.
EPI_ISL_2578398	Epidemiology and Demography, KEMRI-Wellcome Trust Research Programme	Epidemiology and Demography, KEMRI-Wellcome Trust Research Programme	Kamau,E.M., Agoti,C.N., Lewa,C.S., Cane,P.A., Nokes,J.D. and Agoti,C.N.
EPI_ISL_2578399, EPI_ISL_2578400, EPI_ISL_2578401, EPI_ISL_2578402, EPI_ISL_2578403, EPI_ISL_2578404, EPI_ISL_2578405	Microbiology, Hospital Universitari Vall d'Hebron	Microbiology, Hospital Universitari Vall d'Hebron	Gimferrer,L., Martin,Md.C., Pumarola,T. and Anton,A.
EPI_ISL_2578406	Epidemiology and Demography, KEMRI-Wellcome Trust Research Programme	Epidemiology and Demography, KEMRI-Wellcome Trust Research Programme	Kamau,E.M., Agoti,C.N., Lewa,C.S., Cane,P.A., Nokes,J.D. and Agoti,C.N.
EPI_ISL_2578407	Microbiology, Hospital Universitari Vall d'Hebron	Microbiology, Hospital Universitari Vall d'Hebron	Gimferrer,L., Martin,Md.C., Pumarola,T. and Anton,A.
EPI_ISL_2578408	Epidemiology and Demography, KEMRI-Wellcome Trust Research Programme	Epidemiology and Demography, KEMRI-Wellcome Trust Research Programme	Kamau,E.M., Agoti,C.N., Lewa,C.S., Cane,P.A., Nokes,J.D. and Agoti,C.N.
EPI_ISL_2578409, EPI_ISL_2578410, EPI_ISL_2578411, EPI_ISL_2578413, EPI_ISL_2578414, EPI_ISL_2578415, EPI_ISL_2578416, EPI_ISL_2578417	Microbiology, Hospital Universitari Vall d'Hebron	Microbiology, Hospital Universitari Vall d'Hebron	Gimferrer,L., Martin,Md.C., Pumarola,T. and Anton,A.
EPI_ISL_2578418, EPI_ISL_2578419	Epidemiology and Demography, KEMRI-Wellcome Trust Research Programme	Epidemiology and Demography, KEMRI-Wellcome Trust Research Programme	Kamau,E.M., Agoti,C.N., Lewa,C.S., Cane,P.A., Nokes,J.D. and Agoti,C.N.
EPI_ISL_2578420, EPI_ISL_2578421, EPI_ISL_2578422, EPI_ISL_2578423	Microbiology, Hospital Universitari Vall d'Hebron	Microbiology, Hospital Universitari Vall d'Hebron	Gimferrer,L., Martin,Md.C., Pumarola,T. and Anton,A.
EPI_ISL_2578424	Epidemiology and Demography, KEMRI-Wellcome Trust Research Programme	Epidemiology and Demography, KEMRI-Wellcome Trust Research Programme	Kamau,E.M., Agoti,C.N., Lewa,C.S., Cane,P.A., Nokes,J.D. and Agoti,C.N.
EPI_ISL_2578425, EPI_ISL_2578426, EPI_ISL_2578428, EPI_ISL_2578429, EPI_ISL_2578430, EPI_ISL_2578431	Microbiology, Hospital Universitari Vall d'Hebron	Microbiology, Hospital Universitari Vall d'Hebron	Gimferrer,L., Martin,Md.C., Pumarola,T. and Anton,A.
EPI_ISL_2578432, EPI_ISL_2578433	Epidemiology and Demography, KEMRI-Wellcome Trust Research Programme	Epidemiology and Demography, KEMRI-Wellcome Trust Research Programme	Kamau,E.M., Agoti,C.N., Lewa,C.S., Cane,P.A., Nokes,J.D. and Agoti,C.N.
EPI_ISL_2578434	Department of Pediatrics, Center of Excellence in Clinical Virology, Chulalongkorn	Department of Pediatrics, Center of Excellence in Clinical Virology, Chulalongkorn	Thongpan,I.
EPI_ISL_2578435, EPI_ISL_2578436, EPI_ISL_2578437, EPI_ISL_2578438, EPI_ISL_2578439, EPI_ISL_2578440, EPI_ISL_2578441, EPI_ISL_2578443, EPI_ISL_2578444	Epidemiology and Demography, KEMRI-Wellcome Trust Research Programme	Epidemiology and Demography, KEMRI-Wellcome Trust Research Programme	Kamau,E.M., Agoti,C.N., Lewa,C.S., Cane,P.A., Nokes,J.D. and Agoti,C.N.
EPI_ISL_2578445, EPI_ISL_2578446, EPI_ISL_2578447	Department of Pediatrics, Center of Excellence in Clinical Virology, Chulalongkorn	Department of Pediatrics, Center of Excellence in Clinical Virology, Chulalongkorn	Thongpan,I.
EPI_ISL_2578448	Wint Wint Phyu Niigat University, International Health, Public Health	Wint Wint Phyu Niigat University, International Health, Public Health	Phyu,W.W. and Saito,R.
EPI_ISL_2578449, EPI_ISL_2578450, EPI_ISL_2578451, EPI_ISL_2578452, EPI_ISL_2578453	Department of Pediatrics, Center of Excellence in Clinical Virology, Chulalongkorn	Department of Pediatrics, Center of Excellence in Clinical Virology, Chulalongkorn	Thongpan,I.
EPI_ISL_2578454	Wint Wint Phyu Niigat University, International Health, Public Health	Wint Wint Phyu Niigat University, International Health, Public Health	Phyu,W.W. and Saito,R.
EPI_ISL_2578455, EPI_ISL_2578456, EPI_ISL_2578457, EPI_ISL_2578459, EPI_ISL_2578460	Department of Pediatrics, Center of Excellence in Clinical Virology, Chulalongkorn	Department of Pediatrics, Center of Excellence in Clinical Virology, Chulalongkorn	Thongpan,I.

EPI_ISL_2578461	Wint Wint Phyu Niigat University, International Health, Public Health	Wint Wint Phyu Niigat University, International Health, Public Health	Phyu,W.W. and Saito,R.
EPI_ISL_2578462, EPI_ISL_2578463, EPI_ISL_2578464	Department of Pediatrics, Center of Excellence in Clinical Virology, Chulalongkorn	Department of Pediatrics, Center of Excellence in Clinical Virology, Chulalongkorn	Thongpan,I.
EPI_ISL_2578465	Wint Wint Phyu Niigat University, International Health, Public Health	Wint Wint Phyu Niigat University, International Health, Public Health	Phyu,W.W. and Saito,R.
EPI_ISL_2578466, EPI_ISL_2578467, EPI_ISL_2578468, EPI_ISL_2578469	Department of Pediatrics, Center of Excellence in Clinical Virology, Chulalongkorn	Department of Pediatrics, Center of Excellence in Clinical Virology, Chulalongkorn	Thongpan,I.
EPI_ISL_2578470	Wint Wint Phyu Niigat University, International Health, Public Health	Wint Wint Phyu Niigat University, International Health, Public Health	Phyu,W.W. and Saito,R.
EPI_ISL_2578471, EPI_ISL_2578473	Department of Pediatrics, Center of Excellence in Clinical Virology, Chulalongkorn	Department of Pediatrics, Center of Excellence in Clinical Virology, Chulalongkorn	Thongpan,I.
EPI_ISL_2578474, EPI_ISL_2578475, EPI_ISL_2578476	Wint Wint Phyu Niigat University, International Health, Public Health	Wint Wint Phyu Niigat University, International Health, Public Health	Phyu,W.W. and Saito,R.
EPI_ISL_2578477	Department of Pediatrics, Center of Excellence in Clinical Virology, Chulalongkorn	Department of Pediatrics, Center of Excellence in Clinical Virology, Chulalongkorn	Thongpan,I.
EPI_ISL_2578478, EPI_ISL_2578479	Wint Wint Phyu Niigat University, International Health, Public Health	Wint Wint Phyu Niigat University, International Health, Public Health	Phyu,W.W. and Saito,R.
EPI_ISL_2578480, EPI_ISL_2578481, EPI_ISL_2578482	Department of Pediatrics, Center of Excellence in Clinical Virology, Chulalongkorn	Department of Pediatrics, Center of Excellence in Clinical Virology, Chulalongkorn	Thongpan,I.
EPI_ISL_2578483, EPI_ISL_2578484	Microbiology, Hospital Universitari Vall d'Hebron	Microbiology, Hospital Universitari Vall d'Hebron	Gimferrer,L., Martin,Md.C., Pumarola,T. and Anton,A.
EPI_ISL_2578485	Department of Pediatrics, Center of Excellence in Clinical Virology, Chulalongkorn	Department of Pediatrics, Center of Excellence in Clinical Virology, Chulalongkorn	Thongpan,I.
EPI_ISL_2578487	Microbiology, Hospital Universitari Vall d'Hebron	Microbiology, Hospital Universitari Vall d'Hebron	Gimferrer,L., Martin,Md.C., Pumarola,T. and Anton,A.
EPI_ISL_2578488	Department of Pediatrics, Center of Excellence in Clinical Virology, Chulalongkorn	Department of Pediatrics, Center of Excellence in Clinical Virology, Chulalongkorn	Thongpan,I.
EPI_ISL_2578489, EPI_ISL_2578490, EPI_ISL_2578491, EPI_ISL_2578492, EPI_ISL_2578493	Microbiology, Hospital Universitari Vall d'Hebron	Microbiology, Hospital Universitari Vall d'Hebron	Gimferrer,L., Martin,Md.C., Pumarola,T. and Anton,A.
EPI_ISL_2578494	Department of Pediatrics, Center of Excellence in Clinical Virology, Chulalongkorn	Department of Pediatrics, Center of Excellence in Clinical Virology, Chulalongkorn	Thongpan,I.
EPI_ISL_2578495, EPI_ISL_2578496, EPI_ISL_2578497	Microbiology, Hospital Universitari Vall d'Hebron	Microbiology, Hospital Universitari Vall d'Hebron	Gimferrer,L., Martin,Md.C., Pumarola,T. and Anton,A.
EPI_ISL_2578498	Department of Pediatrics, Center of Excellence in Clinical Virology, Chulalongkorn	Department of Pediatrics, Center of Excellence in Clinical Virology, Chulalongkorn	Thongpan,I.
EPI_ISL_2578499, EPI_ISL_2578500, EPI_ISL_2578501, EPI_ISL_2578503, EPI_ISL_2578504, EPI_ISL_2578505, EPI_ISL_2578506, EPI_ISL_2578507, EPI_ISL_2578508, EPI_ISL_2578509	Microbiology, Hospital Universitari Vall d'Hebron	Microbiology, Hospital Universitari Vall d'Hebron	Gimferrer,L., Martin,Md.C., Pumarola,T. and Anton,A.
EPI_ISL_2578510	Wint Wint Phyu Niigat University, International Health, Public Health	Wint Wint Phyu Niigat University, International Health, Public Health	Phyu,W.W. and Saito,R.
EPI_ISL_2578511, EPI_ISL_2578512, EPI_ISL_2578513, EPI_ISL_2578514, EPI_ISL_2578515, EPI_ISL_2578516, EPI_ISL_2578517, EPI_ISL_2578519, EPI_ISL_2578520, EPI_ISL_2578521, EPI_ISL_2578522, EPI_ISL_2578523, EPI_ISL_2578524, EPI_ISL_2578525, EPI_ISL_2578526, EPI_ISL_2578527, EPI_ISL_2578528, EPI_ISL_2578529, EPI_ISL_2578530, EPI_ISL_2578531			
see above	Microbiology, Hospital Universitari Vall d'Hebron	Microbiology, Hospital Universitari Vall d'Hebron	Gimferrer,L., Martin,Md.C., Pumarola,T. and Anton,A.
EPI_ISL_2578611	Pediatric Clinic 1, Department of Pathophysiology and Transplantation, University of Milan and Fondazione IRCCS Ca Granda	Pediatric Clinic 1, Department of Pathophysiology and Transplantation, University of Milan and Fondazione IRCCS Ca Granda	Esposito,S., Zampiero,A., Piralla,A. and Principi,N.
EPI_ISL_2584489, EPI_ISL_2584491, EPI_ISL_2584492, EPI_ISL_2584494, EPI_ISL_2584496, EPI_ISL_2584497, EPI_ISL_2584498, EPI_ISL_2584499, EPI_ISL_2584500, EPI_ISL_2584504	Epidemiology and Demography, KEMRI-Wellcome Trust	Epidemiology and Demography, KEMRI-Wellcome Trust	Kamau,E., Otieno,J.R., Murunga,N., Nyiro,J.U., Oketch,J.W., Ngoi,J.M., de Laurent,Z.R., Mwema,A., Agoti,C.N. and Nokes,D.J.
EPI_ISL_2584508, EPI_ISL_2584509, EPI_ISL_2584510	Centre for Infectious Disease Research, Diagnostics and Screening (IDS) Department of Endemic and Emerging Viruses (EEV), RIVM	Centre for Infectious Disease Research, Diagnostics and Screening (IDS) Department of Endemic and Emerging Viruses (EEV), RIVM	Janssens,T.K.S., Zhan,S.H., Overduin,P., Alamouti,S.M., Kwok,B.S., Schmitz,D., Qadir,M.A. and Meijer,A.
EPI_ISL_2584511, EPI_ISL_2584512, EPI_ISL_2584513, EPI_ISL_2584514	J. Craig Venter Institute	J. Craig Venter Institute	Shabman,R., Das,S.R., Shilts,M., Fedorova,N., Puri,V., Shrivastava,S., Amedeo,P., Williams,M., Barratt,K., Mitchell,J. and Jennings,L.
EPI_ISL_2584568	J. Craig Venter Institute	J. Craig Venter Institute	Das,S.R., Halpin,R.A., Shilts,M., Puri,V., Akopov,A., Fedorova,N., Stockwell,T., Amedeo,P., Bishop,B., Katzel,D., Schobel,S., Shrivastava,S. and Hartert,T.
EPI_ISL_2584591, EPI_ISL_2584592, EPI_ISL_2584593, EPI_ISL_2584594	Epidemiology and Demography, KEMRI-Wellcome Trust	Epidemiology and Demography, KEMRI-Wellcome Trust	Kamau,E., Otieno,J.R., Murunga,N., Nyiro,J.U., Oketch,J.W., Ngoi,J.M., de Laurent,Z.R., Mwema,A., Agoti,C.N. and Nokes,D.J.
EPI_ISL_2584635	J. Craig Venter Institute	J. Craig Venter Institute	Das,S.R., Halpin,R.A., Shilts,M., Puri,V., Akopov,A., Fedorova,N., Stockwell,T., Amedeo,P., Bishop,B., Katzel,D., Schobel,S., Shrivastava,S. and Hartert,T.
EPI_ISL_2584639	Biologia Molecular y Validacion de Tecnicas, Instituto de Diagnostico y Referencia Epidemiologicos (InDRE)	Biologia Molecular y Validacion de Tecnicas, Instituto de Diagnostico y Referencia Epidemiologicos (InDRE)	Munoz-Medina,J.E., Monroy-Munoz,I.E., Santos Coy-Arechavaleta,A., Meza-Chavez,A., Angeles-Martinez,J., Anguiano-Hernandez,Y.M., Santacruz-Tinoco,C.E., Gonzalez-Ibarra,J., Martinez-Miguel,B., Alvarado-Yaah,J.E., Palomec-Nava,I.D., Ortiz-Alcantara,J.M., Garces-Ayala,F., Ramirez-Gonzalez,J.E., Diaz-Quinonez,J.A. and Gonzalez-Bonilla,C.R.
EPI_ISL_2584648	J. Craig Venter Institute	J. Craig Venter Institute	Das,S.R., Halpin,R.A., Shilts,M., Puri,V., Akopov,A., Fedorova,N., Stockwell,T., Amedeo,P., Bishop,B., Katzel,D., Schobel,S., Shrivastava,S. and Hartert,T.
EPI_ISL_2584651, EPI_ISL_2584653, EPI_ISL_2584654, EPI_ISL_2584657, EPI_ISL_2584661, EPI_ISL_2584664, EPI_ISL_2584665, EPI_ISL_2584666, EPI_ISL_2584667, EPI_ISL_2584668, EPI_ISL_2584669, EPI_ISL_2584670, EPI_ISL_2584673, EPI_ISL_2584674, EPI_ISL_2584676, EPI_ISL_2584677, EPI_ISL_2584680, EPI_ISL_2584688, EPI_ISL_2584689, EPI_ISL_2584690, EPI_ISL_2584691, EPI_ISL_2584693, EPI_ISL_2584699, EPI_ISL_2584706, EPI_ISL_2584708, EPI_ISL_2584711, EPI_ISL_2584715, EPI_ISL_2584717, EPI_ISL_2584718, EPI_ISL_2584719, EPI_ISL_2584720, EPI_ISL_2584721, EPI_ISL_2584722, EPI_ISL_2584723, EPI_ISL_2584724, EPI_ISL_2584725			
see above	Epidemiology and Demography, KEMRI-Wellcome Trust	Epidemiology and Demography, KEMRI-Wellcome Trust	Kamau,E., Otieno,J.R., Murunga,N., Nyiro,J.U., Oketch,J.W., Ngoi,J.M., de Laurent,Z.R., Mwema,A., Agoti,C.N. and Nokes,D.J.
EPI_ISL_2584742	J. Craig Venter Institute	J. Craig Venter Institute	Shabman,R., Das,S.R., Shilts,M., Fedorova,N., Puri,V., Shrivastava,S., Amedeo,P., Williams,M., Barratt,K., Mitchell,J. and Jennings,L.
EPI_ISL_2584773, EPI_ISL_2584774	Epidemiology and Demography, KEMRI-Wellcome Trust	Epidemiology and Demography, KEMRI-Wellcome Trust	Kamau,E., Otieno,J.R., Murunga,N., Nyiro,J.U., Oketch,J.W., Ngoi,J.M., de Laurent,Z.R., Mwema,A., Agoti,C.N. and Nokes,D.J.
EPI_ISL_2584833	Centre for Infectious Disease Research, Diagnostics and Screening (IDS) Department of Endemic and Emerging Viruses (EEV), RIVM	Centre for Infectious Disease Research, Diagnostics and Screening (IDS) Department of Endemic and Emerging Viruses (EEV), RIVM	Janssens,T.K.S., Zhan,S.H., Overduin,P., Alamouti,S.M., Kwok,B.S., Schmitz,D., Qadir,M.A. and Meijer,A.
EPI_ISL_2584856	Epidemiology and Demography, KEMRI-Wellcome Trust	Epidemiology and Demography, KEMRI-Wellcome Trust	Kamau,E., Otieno,J.R., Murunga,N., Nyiro,J.U., Oketch,J.W., Ngoi,J.M., de Laurent,Z.R., Mwema,A., Agoti,C.N. and Nokes,D.J.
EPI_ISL_2584860	J. Craig Venter Institute	J. Craig Venter Institute	Shabman,R., Das,S.R., Shilts,M., Fedorova,N., Puri,V., Shrivastava,S., Amedeo,P., Williams,M., Barratt,K., Mitchell,J. and Jennings,L.
EPI_ISL_2584862	Michiko Okamoto Tohoku University Graduate School of Medicine, Virology	Michiko Okamoto Tohoku University Graduate School of Medicine, Virology	Okamoto,M., Malasaor,R. and Oshitani,H.
EPI_ISL_2584866, EPI_ISL_2584867, EPI_ISL_2584868, EPI_ISL_2584869, EPI_ISL_2584871, EPI_ISL_2584872, EPI_ISL_2584881, EPI_ISL_2584882, EPI_ISL_2584883, EPI_ISL_2584884, EPI_ISL_2584885, EPI_ISL_2584886, EPI_ISL_2584887, EPI_ISL_2584888, EPI_ISL_2584889			
see above	Epidemiology and Demography, KEMRI-Wellcome Trust	Epidemiology and Demography, KEMRI-Wellcome Trust	Kamau,E., Otieno,J.R., Murunga,N., Nyiro,J.U., Oketch,J.W., Ngoi,J.M., de Laurent,Z.R., Mwema,A., Agoti,C.N. and Nokes,D.J.
EPI_ISL_2584910	Centre for Infectious Disease Research, Diagnostics and Screening (IDS) Department of Endemic and Emerging Viruses (EEV), RIVM	Centre for Infectious Disease Research, Diagnostics and Screening (IDS) Department of Endemic and Emerging Viruses (EEV), RIVM	Janssens,T.K.S., Zhan,S.H., Overduin,P., Alamouti,S.M., Kwok,B.S., Schmitz,D., Qadir,M.A. and Meijer,A.
EPI_ISL_2584912, EPI_ISL_2584913, EPI_ISL_2584914, EPI_ISL_2584915, EPI_ISL_2584916, EPI_ISL_2584917, EPI_ISL_2584918, EPI_ISL_2584919, EPI_ISL_2584920, EPI_ISL_2584921, EPI_ISL_2584922, EPI_ISL_2584923, EPI_ISL_2584924, EPI_ISL_2584925, EPI_ISL_2584926, EPI_ISL_2584927, EPI_ISL_2584928, EPI_ISL_2584929, EPI_ISL_2584930, EPI_ISL_2584931, EPI_ISL_2584932, EPI_ISL_2584933, EPI_ISL_2584934, EPI_ISL_2584935			
see above	Epidemiology and Demography, KEMRI-Wellcome Trust	Epidemiology and Demography, KEMRI-Wellcome Trust	Kamau,E., Otieno,J.R., Murunga,N., Nyiro,J.U., Oketch,J.W., Ngoi,J.M., de Laurent,Z.R., Mwema,A., Agoti,C.N. and Nokes,D.J.
EPI_ISL_2584938, EPI_ISL_2584939	J. Craig Venter Institute	J. Craig Venter Institute	Shabman,R., Das,S.R., Shilts,M., Fedorova,N., Puri,V., Shrivastava,S., Amedeo,P., Williams,M., Barratt,K., Mitchell,J. and Jennings,L.
EPI_ISL_2584955, EPI_ISL_2584956, EPI_ISL_2584958, EPI_ISL_2584959, EPI_ISL_2584960, EPI_ISL_2584961, EPI_ISL_2584963, EPI_ISL_2584964, EPI_ISL_2584965, EPI_ISL_2584967, EPI_ISL_2584968, EPI_ISL_2584969, EPI_ISL_2584970, EPI_ISL_2584972, EPI_ISL_2584973, EPI_ISL_2584975, EPI_ISL_2584976, EPI_ISL_2584977, EPI_ISL_2584978, EPI_ISL_2584980, EPI_ISL_2584981, EPI_ISL_2584982, EPI_ISL_2584983, EPI_ISL_2584985			
see above	Epidemiology and Demography, KEMRI-Wellcome Trust	Epidemiology and Demography, KEMRI-Wellcome Trust	Kamau,E., Otieno,J.R., Murunga,N., Nyiro,J.U., Oketch,J.W., Ngoi,J.M., de Laurent,Z.R., Mwema,A., Agoti,C.N. and Nokes,D.J.
EPI_ISL_2584988	J. Craig Venter Institute	J. Craig Venter Institute	Shabman,R., Das,S.R., Shilts,M., Fedorova,N., Puri,V., Shrivastava,S., Amedeo,P., Williams,M., Barratt,K., Mitchell,J. and Jennings,L.
EPI_ISL_2584994	Division of Biosafety Evaluation and Control, Korea National Institute of Health	Division of Biosafety Evaluation and Control, Korea National Institute of Health	Yun,M.-R., Lee,W.-J., Kim,A.-R., Lee,H.S., Kim,K., Kim,S.S., Kim,Y.-J. and Kim,D.-W.
EPI_ISL_2585017, EPI_ISL_2585018, EPI_ISL_2585019, EPI_ISL_2585020, EPI_ISL_2585021, EPI_ISL_2585022, EPI_ISL_2585023, EPI_ISL_2585025, EPI_ISL_2585026, EPI_ISL_2585027, EPI_ISL_2585028, EPI_ISL_2585029, EPI_ISL_2585031, EPI_ISL_2585032, EPI_ISL_2585033, EPI_ISL_2585036, EPI_ISL_2585037, EPI_ISL_2585039, EPI_ISL_2585040, EPI_ISL_2585041, EPI_ISL_2585042, EPI_ISL_2585043, EPI_ISL_2585044, EPI_ISL_2585045, EPI_ISL_2585047, EPI_ISL_2585049, EPI_ISL_2585050, EPI_ISL_2585051, EPI_ISL_2585052, EPI_ISL_2585053, EPI_ISL_2585054, EPI_ISL_2585056, EPI_ISL_2585057, EPI_ISL_2585058, EPI_ISL_2585059, EPI_ISL_2585060, EPI_ISL_2585061			
see above	Epidemiology and Demography, KEMRI-Wellcome Trust	Epidemiology and Demography, KEMRI-Wellcome Trust	Kamau,E., Otieno,J.R., Murunga,N., Nyiro,J.U., Oketch,J.W., Ngoi,J.M., de Laurent,Z.R., Mwema,A., Agoti,C.N. and Nokes,D.J.
EPI_ISL_2585064, EPI_ISL_2585065, EPI_ISL_2585066, EPI_ISL_2585067	J. Craig Venter Institute	J. Craig Venter Institute	Shabman,R., Das,S.R., Shilts,M., Fedorova,N., Puri,V., Shrivastava,S., Amedeo,P., Williams,M., Barratt,K., Mitchell,J. and Jennings,L.
EPI_ISL_2585096, EPI_ISL_2585098, EPI_ISL_2585099, EPI_ISL_2585100, EPI_ISL_2585101, EPI_ISL_2585102, EPI_ISL_2585103, EPI_ISL_2585104, EPI_ISL_2585105, EPI_ISL_2585106, EPI_ISL_2585107, EPI_ISL_2585108, EPI_ISL_2585110, EPI_ISL_2585111, EPI_ISL_2585112, EPI_ISL_2585113, EPI_ISL_2585114, EPI_ISL_2585115, EPI_ISL_2585116, EPI_ISL_2585117, EPI_ISL_2585118, EPI_ISL_2585119, EPI_ISL_2585120, EPI_ISL_2585121, EPI_ISL_2585123, EPI_ISL_2585124, EPI_ISL_2585125, EPI_ISL_2585126, EPI_ISL_2585127, EPI_ISL_2585128, EPI_ISL_2585130, EPI_ISL_2585131, EPI_ISL_2585132, EPI_ISL_2585133, EPI_ISL_2585134, EPI_ISL_2585135, EPI_ISL_2585136, EPI_ISL_2585137, EPI_ISL_2585139, EPI_ISL_2585140, EPI_ISL_2585156, EPI_ISL_2585157, EPI_ISL_2585158, EPI_ISL_2585160, EPI_ISL_2585161, EPI_ISL_2585162, EPI_ISL_2585163, EPI_ISL_2585164, EPI_ISL_2585165, EPI_ISL_2585167, EPI_ISL_2585168, EPI_ISL_2585169, EPI_ISL_2585171, EPI_ISL_2585172, EPI_ISL_2585173, EPI_ISL_2585174, EPI_ISL_2585175, EPI_ISL_2585176, EPI_ISL_2585177, EPI_ISL_2585178, EPI_ISL_2585179, EPI_ISL_2585180, EPI_ISL_2585181, EPI_ISL_2585182, EPI_ISL_2585183, EPI_ISL_2585184, EPI_ISL_2585185, EPI_ISL_2585186, EPI_ISL_2585187, EPI_ISL_2585188			
see above	Epidemiology and Demography, KEMRI-Wellcome Trust	Epidemiology and Demography, KEMRI-Wellcome Trust	Kamau,E., Otieno,J.R., Murunga,N., Nyiro,J.U., Oketch,J.W., Ngoi,J.M., de Laurent,Z.R., Mwema,A., Agoti,C.N. and Nokes,D.J.

EPI_ISL_2585191, EPI_ISL_2585192	J. Craig Venter Institute	J. Craig Venter Institute	Shabman,R., Das,S.R., Shilts,M., Fedorova,N., Puri,V., Shrivastava,S., Amedeo,P., Williams,M., Barratt,K., Mitchell,J. and Jennings,L.
EPI_ISL_2588651	Virology Laboratory, Dr. Ricardo Gutierrez Children Hospital	Virology Laboratory, Dr. Ricardo Gutierrez Children Hospital	Goya,S., Rojo,G.L., Valinotto,L.E., Mistchenko,A.S. and Viegas,M.
EPI_ISL_2588654	Virology Laboratory, Dr. Ricardo Gutierrez Children Hospital	Virology Laboratory, Dr. Ricardo Gutierrez Children Hospital	Goya,S., Valinotto,L.E., Tittarelli,E., Rojo,G.L., Greninger,A., Luso,S., Natale,M., Mistchenko,A.S. and Viegas,M.
EPI_ISL_2588655	Virology Laboratory, Dr. Ricardo Gutierrez Children Hospital	Virology Laboratory, Dr. Ricardo Gutierrez Children Hospital	Goya,S., Rojo,G.L., Valinotto,L.E., Mistchenko,A.S. and Viegas,M.
EPI_ISL_2588659, EPI_ISL_2588664	Key Laboratory of Emergency Detection for Public Health of Zhejiang Province, Zhejiang Provincial Centre for Disease Control and Prevention	Key Laboratory of Emergency Detection for Public Health of Zhejiang Province, Zhejiang Provincial Centre for Disease Control and Prevention	Li,C.-X., Li,W., Zhou,J., Zhang,B., Feng,Y., Xu,C.-P., Lu,Y.-Y., Holmes,E.C. and Shi,M.
EPI_ISL_2588666	Virology Laboratory, Dr. Ricardo Gutierrez Children Hospital	Virology Laboratory, Dr. Ricardo Gutierrez Children Hospital	Goya,S., Valinotto,L.E., Tittarelli,E., Rojo,G.L., Greninger,A., Luso,S., Natale,M., Mistchenko,A.S. and Viegas,M.
EPI_ISL_2588667	Mami Nagashima Tokyo Metropolitan Institute of Public Health, Microbiology	Mami Nagashima Tokyo Metropolitan Institute of Public Health, Microbiology	Hasegawa,M., Okazaki,T., Sakamoto,T., Murata,R., Nagashima,M., Shinkai,T. and Sadamasu,K.
EPI_ISL_2588669	Key Laboratory of Emergency Detection for Public Health of Zhejiang Province, Zhejiang Provincial Centre for Disease Control and Prevention	Key Laboratory of Emergency Detection for Public Health of Zhejiang Province, Zhejiang Provincial Centre for Disease Control and Prevention	Li,C.-X., Li,W., Zhou,J., Zhang,B., Feng,Y., Xu,C.-P., Lu,Y.-Y., Holmes,E.C. and Shi,M.
EPI_ISL_2588670, EPI_ISL_2588674, EPI_ISL_2588676, EPI_ISL_2588677, EPI_ISL_2588678, EPI_ISL_2588680, EPI_ISL_2588681, EPI_ISL_2588682, EPI_ISL_2588690, EPI_ISL_2588691, EPI_ISL_2588692, EPI_ISL_2588693, EPI_ISL_2588703, EPI_ISL_2588704, EPI_ISL_2588705, EPI_ISL_2588706, EPI_ISL_2588707, EPI_ISL_2588708, EPI_ISL_2588709, EPI_ISL_2588710, EPI_ISL_2588711, EPI_ISL_2588712, EPI_ISL_2588713, EPI_ISL_2588714, EPI_ISL_2588715, EPI_ISL_2588716			
see above	Virology Laborotary, Beijing Pediatric Research Institute, Beijing Children's Hospital, Capital Medical University, National Center for Children's Health	Virology Laborotary, Beijing Pediatric Research Institute, Beijing Children's Hospital, Capital Medical University, National Center for Children's Health	Chen,X., Guo,J. and Xie,Z.
EPI_ISL_2588720	Virology Laboratory, Dr. Ricardo Gutierrez Children Hospital	Virology Laboratory, Dr. Ricardo Gutierrez Children Hospital	Goya,S., Valinotto,L.E., Tittarelli,E., Rojo,G.L., Greninger,A., Luso,S., Natale,M., Mistchenko,A.S. and Viegas,M.
EPI_ISL_2588721, EPI_ISL_2588722	Virology Laboratory, Dr. Ricardo Gutierrez Children Hospital	Virology Laboratory, Dr. Ricardo Gutierrez Children Hospital	Goya,S., Valinotto,L.E., Tittarelli,E., Rojo,G.L., Greninger,A., Zaiat,J., Marti,M., Mistchenko,A.S. and Viegas,M.
EPI_ISL_2588726, EPI_ISL_2588727, EPI_ISL_2588728, EPI_ISL_2588729, EPI_ISL_2588730, EPI_ISL_2588731, EPI_ISL_2588732, EPI_ISL_2588733, EPI_ISL_2588735, EPI_ISL_2588743, EPI_ISL_2588749, EPI_ISL_2588750, EPI_ISL_2588751, EPI_ISL_2588752, EPI_ISL_2588753, EPI_ISL_2588754, EPI_ISL_2588755, EPI_ISL_2588756, EPI_ISL_2588757, EPI_ISL_2588758, EPI_ISL_2588759, EPI_ISL_2588760, EPI_ISL_2588761, EPI_ISL_2588762, EPI_ISL_2588763, EPI_ISL_2588764, EPI_ISL_2588765, EPI_ISL_2588766, EPI_ISL_2588767, EPI_ISL_2588768, EPI_ISL_2588769, EPI_ISL_2588770, EPI_ISL_2588771, EPI_ISL_2588772, EPI_ISL_2588773, EPI_ISL_2588774, EPI_ISL_2588775, EPI_ISL_2588776, EPI_ISL_2588777, EPI_ISL_2588778, EPI_ISL_2588779, EPI_ISL_2588780			
see above	Virology Laborotary, Beijing Pediatric Research Institute, Beijing Children's Hospital, Capital Medical University, National Center for Children's Health	Virology Laborotary, Beijing Pediatric Research Institute, Beijing Children's Hospital, Capital Medical University, National Center for Children's Health	Chen,X., Guo,J. and Xie,Z.
EPI_ISL_2588785	Mami Nagashima Tokyo Metropolitan Institute of Public Health, Microbiology	Mami Nagashima Tokyo Metropolitan Institute of Public Health, Microbiology	Hasegawa,M., Okazaki,T., Sakamoto,T., Murata,R., Nagashima,M., Shinkai,T. and Sadamasu,K.
EPI_ISL_412460, EPI_ISL_412461, EPI_ISL_412863	Virology Laboratory, Ricardo Gutiérrez Children's Hospital	Virology Laboratory, Ricardo Gutiérrez Children's Hospital / Vanderbilt University Medical Center	Goya, Stephanie; Lucion, Maria Florencia; Juarez, Maria del Valle; Shilts, Meghan; Gentile, Angela; Mistchenko, Alicia S.; Das, Suman & Viegas, Mariana
EPI_ISL_412864	Virology Laboratory, Ricardo Gutiérrez Children's Hospital.	Virology Laboratory, Ricardo Gutiérrez Children's Hospital / Vanderbilt University Medical Center.	Goya, Stephanie; Lucion, Maria Florencia; Juarez, Maria del Valle; Shilts, Meghan; Gentile, Angela; Mistchenko, Alicia S.; Das, Suman & Viegas, Mariana
EPI_ISL_412867, EPI_ISL_412868	Respiratory Virus Unit, Microbiology Services Colindale, Public Health England	Microbiology Services Colindale, Public Health England	Zambon M
EPI_ISL_413293, EPI_ISL_413352	Institut Pasteur de Madagascar	Institut Pasteur de Madagascar	Jean-Michel HERAUD
EPI_ISL_414004	WHO National Influenza Centre Russian Federation	WHO National Influenza Centre Russian Federation	Komissarova K.,Fadeev A., Komissarov A., Krivitskaya V.
EPI_ISL_4569432	VIC, Victorian Infectious Diseases Reference Laboratory	WHO Collaborating Centre for Reference and Research on Influenza	Xiaomin Dong, Annette Alafaci,Yi-Mo Deng, Ammar Aziz, Naomi Komadina
EPI_ISL_4848359	Respiratory Virus Unit, National Infection Service, Public Health England	Public Health England, Respiratory Virus Unit, National Infection Service	Zambon M, Talts T, Ellis J, Miah S, Platt S
EPI_ISL_549307, EPI_ISL_549308, EPI_ISL_549309	Laboratorio Central de Saude Publica do Estado do Espirito Santo (LACEN/ES)	Laboratory of Respiratory Viruses and Measles, Oswaldo Cruz Institute, FIOCRUZ	Lucas Vianna, Lays Volpini, Marilda Siqueira, Iuri Louro, Paola Resende
EPI_ISL_549310	LACEN/ES	Laboratory of Respiratory Viruses and Measles, Oswaldo Cruz Institute, FIOCRUZ	Lucas Vianna, Lays Volpini, Marilda Siqueira, Iuri Louro, Paola Resende
EPI_ISL_549311, EPI_ISL_549312, EPI_ISL_549313, EPI_ISL_549314	Laboratorio Central de Saude Publica do Estado do Espirito Santo (LACEN/ES)	Laboratory of Respiratory Viruses and Measles, Oswaldo Cruz Institute, FIOCRUZ	Lucas Vianna, Lays Volpini, Marilda Siqueira, Iuri Louro, Paola Resende
EPI_ISL_549315, EPI_ISL_549316, EPI_ISL_549317, EPI_ISL_549318	LACEN/ES	Laboratory of Respiratory Viruses and Measles, Oswaldo Cruz Institute, FIOCRUZ	Lucas Vianna, Lays Volpini, Marilda Siqueira, Iuri Louro, Paola Resende
EPI_ISL_549319, EPI_ISL_549320	Laboratorio Central de Saude Publica do Estado do Espirito Santo (LACEN/ES)	Laboratory of Respiratory Viruses and Measles, Oswaldo Cruz Institute, FIOCRUZ	Lucas Vianna, Lays Volpini, Marilda Siqueira, Iuri Louro, Paola Resende
EPI_ISL_549321, EPI_ISL_549322	LACEN/ES	Laboratory of Respiratory Viruses and Measles, Oswaldo Cruz Institute, FIOCRUZ	Lucas Vianna, Lays Volpini, Marilda Siqueira, Iuri Louro, Paola Resende
EPI_ISL_549323, EPI_ISL_549324, EPI_ISL_549325	Laboratorio Central de Saude Publica do Estado do Espirito Santo (LACEN/ES)	Laboratory of Respiratory Viruses and Measles, Oswaldo Cruz Institute, FIOCRUZ	Lucas Vianna, Lays Volpini, Marilda Siqueira, Iuri Louro, Paola Resende
EPI_ISL_549326, EPI_ISL_549327	LACEN/ES	Laboratory of Respiratory Viruses and Measles, Oswaldo Cruz Institute, FIOCRUZ	Lucas Vianna, Lays Volpini, Marilda Siqueira, Iuri Louro, Paola Resende
EPI_ISL_5522625, EPI_ISL_5522626, EPI_ISL_5522627	Institut Pasteur de Côte d'Ivoire	WHO Collaborating Centre for Reference and Research on Influenza	Xiaomin Dong, Herve Kadjo, Yi-Mo Deng, Ammar Aziz, Naomi Komadina
EPI_ISL_5930144, EPI_ISL_5930145, EPI_ISL_5930146, EPI_ISL_5930148	Institut Pasteur de Cote d'ivoire	WHO Collaborating Centre for Reference and Research on Influenza	Xiaomin Dong, Herve Kadjo, Yi-Mo Deng, Ammar Aziz, Naomi Komadina
EPI_ISL_6174132, EPI_ISL_6174133, EPI_ISL_6174134	Cote d'ivoire, Institut Pasteur de Cote d'ivoire	WHO Collaborating Centre for Reference and Research on Influenza	Xiaomin Dong, Herve Kadjo, Yi-Mo Deng, Ammar Aziz, Naomi Komadina
EPI_ISL_6266390	WHO Collaborating Centre for Reference and Research on Influenza	WHO Collaborating Centre for Reference and Research on Influenza	Xiaomin Dong, Herve Kadjo, Yi-Mo Deng, Ammar Aziz, Naomi Komadina
EPI_ISL_6494786, EPI_ISL_6494789, EPI_ISL_6494790, EPI_ISL_6494791, EPI_ISL_6494795, EPI_ISL_6494797, EPI_ISL_6494798, EPI_ISL_6494799, EPI_ISL_6494801, EPI_ISL_6494802, EPI_ISL_6494803, EPI_ISL_6494804, EPI_ISL_6494810, EPI_ISL_6494812, EPI_ISL_6494813, EPI_ISL_6494814, EPI_ISL_6494817, EPI_ISL_6494825, EPI_ISL_6494826, EPI_ISL_6494827, EPI_ISL_6495005, EPI_ISL_6495006, EPI_ISL_732343, EPI_ISL_732349, EPI_ISL_732350, EPI_ISL_732351, EPI_ISL_732352, EPI_ISL_732353, EPI_ISL_732354, EPI_ISL_732355			
see above	Respiratory Virus Unit, National Infection Service, Public Health England	National Infection Service, Public Health England	Zambon M, Talts T, Ellis J, Miah S, Platt S
EPI_ISL_9692635, EPI_ISL_9694009, EPI_ISL_9694316, EPI_ISL_9694669	Ricardo Gutierrez Children's Hospital, Virology Laboratory, sequencing area.	Ricardo Gutierrez Children's Hospital, Virology Laboratory, sequencing area.	Acuña, Dolores; Goya, Stephanie; Nabaes Jodar, Mercedes S.; Grandis, Érica; María Cristina Alvarez López, María Elina Acevedo, Oscar Jacquez, Sofía Alexay, Mariángeles Barreda Fink, María Emilia Villegas, Raquel Barquez, Estela Chascón, Jorgelina Caruso, Karina Zacarías, Cristian Díaz, Oscar Luna, Cristian Turchiaro, Julián Cipelli, Guillermo Thomas, Carla Medina, Natalia Labarta. Mistchenko, Alicia S.; Viegas, Mariana.
EPI_ISL_9695176	Ricardo Gutierrez Children's Hospital, Virology Laboratory, sequencing area.	Ricardo Gutierrez Children's Hospital, Virology Laboratory, sequencing area.	Acuña, Dolores; Goya, Stephanie; Nabaes Jodar, Mercedes S.; Grandis, Érica; María Cristina Alvarez López, María Elina Acevedo, Oscar Jacquez, Sofía Alexay, Mariángeles Barreda Fink, María Emilia Villegas, Raquel Barquez, Estela Chascón, Jorgelina Caruso, Karina Zacarías, Cristian Díaz, Oscar Luna, Cristian Turchiaro, Julián Cipelli, Guillermo Thomas, Carla Medina, Natalia Labarta. Mistchenko, Alicia S.; Viegas, Mariana
EPI_ISL_9695537	Ricardo Gutierrez Children's Hospital, Virology Laboratory, sequencing area.	Ricardo Gutierrez Children's Hospital, Virology Laboratory, sequencing area.	Acuña, Dolores; Goya, Stephanie; Nabaes Jodar, Mercedes S.; Grandis, Érica; María Cristina Alvarez López, María Elina Acevedo, Oscar Jacquez, Sofía Alexay, Mariángeles Barreda Fink, María Emilia Villegas, Raquel Barquez, Estela Chascón, Jorgelina Caruso, Karina Zacarías, Cristian Díaz, Oscar Luna, Cristian Turchiaro, Julián Cipelli, Guillermo Thomas, Carla Medina, Natalia Labarta. Mistchenko, Alicia S.; Viegas, Mariana.

We gratefully acknowledge the following Authors from the Originating laboratories responsible for obtaining the specimens, as well as the Submitting laboratories where the genome data were generated and shared via GISAID, on which this research is based.

All Submitters of data may be contacted directly via www.gisaid.org

Authors are sorted alphabetically.

Accession ID	Originating Laboratory	Submitting Laboratory	Authors
EPI_ISL_11019781, EPI_ISL_11019782, EPI_ISL_11019880	NAKORNPING HOSPITAL	National Institute of Health, Department of Medical Sciences, Ministry of Public Health, Thailand	Siripaporn Phuygun; Pakorn Piromtong; Thanutsapa Thanadachakul; Natchaya Khiadsang; Sunthareeya Waicharoen; Malinee Chittaganpitch; Pilailuk Akkapaiboon Okada
EPI_ISL_11055715	Rahima Moosa Mother and child Hospital	National Institute for Communicable Diseases of the National Health Laboratory Service	Amoako DG, Everatt J, Kekana D, Scheepers C, Mohale T, Ntuli N, Mahlangu B, Mnguni A, Ismail A, Bhiman JN, Wolter N
EPI_ISL_11055716, EPI_ISL_11055717	Agincourt clinic	National Institute for Communicable Diseases of the National Health Laboratory Service	Amoako DG, Everatt J, Kekana D, Scheepers C, Mohale T, Ntuli N, Mahlangu B, Mnguni A, Ismail A, Bhiman JN, Wolter N
EPI_ISL_11055735	Matikwana hospital	National Institute for Communicable Diseases of the National Health Laboratory Service	Amoako DG, Everatt J, Kekana D, Scheepers C, Mohale T, Ntuli N, Mahlangu B, Mnguni A, Ismail A, Bhiman JN, Wolter N
EPI_ISL_11055736	Eastridge clinic	National Institute for Communicable Diseases of the National Health Laboratory Service	Amoako DG, Everatt J, Kekana D, Scheepers C, Mohale T, Ntuli N, Mahlangu B, Mnguni A, Ismail A, Bhiman JN, Wolter N
EPI_ISL_11055737	Agincourt clinic	National Institute for Communicable Diseases of the National Health Laboratory Service	Amoako DG, Everatt J, Kekana D, Scheepers C, Mohale T, Ntuli N, Mahlangu B, Mnguni A, Ismail A, Bhiman JN, Wolter N
EPI_ISL_11055738	Mapulaneng hospital	National Institute for Communicable Diseases of the National Health Laboratory Service	Amoako DG, Everatt J, Kekana D, Scheepers C, Mohale T, Ntuli N, Mahlangu B, Mnguni A, Ismail A, Bhiman JN, Wolter N
EPI_ISL_11055740, EPI_ISL_11055741, EPI_ISL_11055742, EPI_ISL_11055743	Red cross childrens hospital	National Institute for Communicable Diseases of the National Health Laboratory Service	Amoako DG, Everatt J, Kekana D, Scheepers C, Mohale T, Ntuli N, Mahlangu B, Mnguni A, Ismail A, Bhiman JN, Wolter N
EPI_ISL_11055744	Mitchell's plain distric hospital	National Institute for Communicable Diseases of the National Health Laboratory Service	Amoako DG, Everatt J, Kekana D, Scheepers C, Mohale T, Ntuli N, Mahlangu B, Mnguni A, Ismail A, Bhiman JN, Wolter N
EPI_ISL_11055745, EPI_ISL_11055746, EPI_ISL_11055747	Red cross childrens hospital	National Institute for Communicable Diseases of the National Health Laboratory Service	Amoako DG, Everatt J, Kekana D, Scheepers C, Mohale T, Ntuli N, Mahlangu B, Mnguni A, Ismail A, Bhiman JN, Wolter N
EPI_ISL_11055748	Eastridge clinic	National Institute for Communicable Diseases of the National Health Laboratory Service	Amoako DG, Everatt J, Kekana D, Scheepers C, Mohale T, Ntuli N, Mahlangu B, Mnguni A, Ismail A, Bhiman JN, Wolter N
EPI_ISL_11055749	Red cross childrens hospital	National Institute for Communicable Diseases of the National Health Laboratory Service	Amoako DG, Everatt J, Kekana D, Scheepers C, Mohale T, Ntuli N, Mahlangu B, Mnguni A, Ismail A, Bhiman JN, Wolter N
EPI_ISL_11055750	Eastridge clinic	National Institute for Communicable Diseases of the National Health Laboratory Service	Amoako DG, Everatt J, Kekana D, Scheepers C, Mohale T, Ntuli N, Mahlangu B, Mnguni A, Ismail A, Bhiman JN, Wolter N
EPI_ISL_11055751	Red cross childrens hospital	National Institute for Communicable Diseases of the National Health Laboratory Service	Amoako DG, Everatt J, Kekana D, Scheepers C, Mohale T, Ntuli N, Mahlangu B, Mnguni A, Ismail A, Bhiman JN, Wolter N
EPI_ISL_11055752, EPI_ISL_11055753, EPI_ISL_11055754, EPI_ISL_11055755, EPI_ISL_11055756, EPI_ISL_11055757, EPI_ISL_11055759, EPI_ISL_11055760	Mitchell's plain distric hospital	National Institute for Communicable Diseases of the National Health Laboratory Service	Amoako DG, Everatt J, Kekana D, Scheepers C, Mohale T, Ntuli N, Mahlangu B, Mnguni A, Ismail A, Bhiman JN, Wolter N
EPI_ISL_11055762, EPI_ISL_11055763, EPI_ISL_11055765, EPI_ISL_11055766	Red cross childrens hospital	National Institute for Communicable Diseases of the National Health Laboratory Service	Amoako DG, Everatt J, Kekana D, Scheepers C, Mohale T, Ntuli N, Mahlangu B, Mnguni A, Ismail A, Bhiman JN, Wolter N
EPI_ISL_11428293, EPI_ISL_11428294, EPI_ISL_11428295, EPI_ISL_11428296, EPI_ISL_11428297, EPI_ISL_11428298, EPI_ISL_11428299, EPI_ISL_11428300, EPI_ISL_11428301, EPI_ISL_11428302, EPI_ISL_11428303, EPI_ISL_11428304, EPI_ISL_11428305, EPI_ISL_11428306, EPI_ISL_11428315, EPI_ISL_11428316, EPI_ISL_11428317, EPI_ISL_11428318, EPI_ISL_11428319, EPI_ISL_11428320, EPI_ISL_11428321, EPI_ISL_11428322, EPI_ISL_11428323, EPI_ISL_11428324, EPI_ISL_11428325, EPI_ISL_11428326, EPI_ISL_11428327, EPI_ISL_11428328			
see above	Respiratory Virus Unit, Reference Services, UK Health Security Agency	Reference Services, UK Health Security Agency	Zambon M, Talts T, Ellis J, Miah S, Platt S
EPI_ISL_11817019, EPI_ISL_11817020, EPI_ISL_11817021, EPI_ISL_11817023, EPI_ISL_11817024, EPI_ISL_11817025, EPI_ISL_11817026, EPI_ISL_11817027, EPI_ISL_11817028, EPI_ISL_11817030, EPI_ISL_11817032, EPI_ISL_11817036, EPI_ISL_11817038, EPI_ISL_11817039, EPI_ISL_11817041, EPI_ISL_11817042, EPI_ISL_11817043, EPI_ISL_11817045, EPI_ISL_11817046, EPI_ISL_11817049, EPI_ISL_11817051, EPI_ISL_11817053, EPI_ISL_11817056, EPI_ISL_11817061, EPI_ISL_11817062, EPI_ISL_11817065, EPI_ISL_11817066, EPI_ISL_11817069, EPI_ISL_11817070, EPI_ISL_11817071, EPI_ISL_11817072, EPI_ISL_11817075, EPI_ISL_11817076, EPI_ISL_11817078, EPI_ISL_11817080, EPI_ISL_11817082, EPI_ISL_11817083, EPI_ISL_11817086			
see above	QLD, Royal Brisbane and Woman's Hospital	WHO Collaborating Centre for Reference and Research on Influenza	Xiaomin Dong, Yi-Mo Deng, Ammar Aziz, Naomi Komadina
EPI_ISL_11817088, EPI_ISL_11817090, EPI_ISL_11817091, EPI_ISL_11817092, EPI_ISL_11817094, EPI_ISL_11817095, EPI_ISL_11817096, EPI_ISL_11817097, EPI_ISL_11817099, EPI_ISL_11817100, EPI_ISL_11817101, EPI_ISL_11817102, EPI_ISL_11817103, EPI_ISL_11817104, EPI_ISL_11817105, EPI_ISL_11817106, EPI_ISL_11817107, EPI_ISL_11817108, EPI_ISL_11817109, EPI_ISL_11823065			
see above	VIC, Royal Children's Hospital	WHO Collaborating Centre for Reference and Research on Influenza	Xiaomin Dong, Annette Alafaci,Yi-Mo Deng, Ammar Aziz, Naomi Komadina
EPI_ISL_12529634, EPI_ISL_12529635	Rahima Moosa Mother and child Hospital	National Institute for Communicable Diseases of the National Health Laboratory Service	Amoako DG, Everatt J, Kekana D, Scheepers C, Mohale T, Ntuli N, Mahlangu B, Mnguni A, Ismail A, Bhiman JN, Wolter N
EPI_ISL_12529636	Agincourt clinic	National Institute for Communicable Diseases of the National Health Laboratory Service	Amoako DG, Everatt J, Kekana D, Scheepers C, Mohale T, Ntuli N, Mahlangu B, Mnguni A, Ismail A, Bhiman JN, Wolter N
EPI_ISL_12529637, EPI_ISL_12529638	Rahima Moosa Mother and child Hospital	National Institute for Communicable Diseases of the National Health Laboratory Service	Amoako DG, Everatt J, Kekana D, Scheepers C, Mohale T, Ntuli N, Mahlangu B, Mnguni A, Ismail A, Bhiman JN, Wolter N
EPI_ISL_12529639, EPI_ISL_12529640	Mapulaneng hospital	National Institute for Communicable Diseases of the National Health Laboratory Service	Amoako DG, Everatt J, Kekana D, Scheepers C, Mohale T, Ntuli N, Mahlangu B, Mnguni A, Ismail A, Bhiman JN, Wolter N
EPI_ISL_12529641	Agincourt clinic	National Institute for Communicable Diseases of the National Health Laboratory Service	Amoako DG, Everatt J, Kekana D, Scheepers C, Mohale T, Ntuli N, Mahlangu B, Mnguni A, Ismail A, Bhiman JN, Wolter N
EPI_ISL_12529642	Rahima Moosa Mother and child Hospital	National Institute for Communicable Diseases of the National Health Laboratory Service	Amoako DG, Everatt J, Kekana D, Scheepers C, Mohale T, Ntuli N, Mahlangu B, Mnguni A, Ismail A, Bhiman JN, Wolter N
EPI_ISL_12529643	Red cross childrens hospital	National Institute for Communicable Diseases of the National Health Laboratory Service	Amoako DG, Everatt J, Kekana D, Scheepers C, Mohale T, Ntuli N, Mahlangu B, Mnguni A, Ismail A, Bhiman JN, Wolter N
EPI_ISL_12529644	Rahima Moosa Mother and child Hospital	National Institute for Communicable Diseases of the National Health Laboratory Service	Amoako DG, Everatt J, Kekana D, Scheepers C, Mohale T, Ntuli N, Mahlangu B, Mnguni A, Ismail A, Bhiman JN, Wolter N
EPI_ISL_12529645, EPI_ISL_12529646	Tintswalo Hospital	National Institute for Communicable Diseases of the National Health Laboratory Service	Amoako DG, Everatt J, Kekana D, Scheepers C, Mohale T, Ntuli N, Mahlangu B, Mnguni A, Ismail A, Bhiman JN, Wolter N
EPI_ISL_12529647	Matikwana hospital	National Institute for Communicable Diseases of the National Health Laboratory Service	Amoako DG, Everatt J, Kekana D, Scheepers C, Mohale T, Ntuli N, Mahlangu B, Mnguni A, Ismail A, Bhiman JN, Wolter N
EPI_ISL_12529648	Mitchell's plain distric hospital	National Institute for Communicable Diseases of the National Health Laboratory Service	Amoako DG, Everatt J, Kekana D, Scheepers C, Mohale T, Ntuli N, Mahlangu B, Mnguni A, Ismail A, Bhiman JN, Wolter N
EPI_ISL_12809009, EPI_ISL_12809055, EPI_ISL_12809059, EPI_ISL_12809161, EPI_ISL_12809164, EPI_ISL_12809165, EPI_ISL_12809167, EPI_ISL_12809267, EPI_ISL_12809268, EPI_ISL_12809269, EPI_ISL_12809270, EPI_ISL_12809271, EPI_ISL_12809272			
see above	ICMR-NATIONAL INSTITUTE OF VIROLOGY INFLUENZA	ICMR-National Institute of Virology Influenza	Dr. varsha potdar and NIC team
EPI_ISL_12970401, EPI_ISL_12970403, EPI_ISL_12970405, EPI_ISL_12970407, EPI_ISL_12970408, EPI_ISL_12970409, EPI_ISL_12970411, EPI_ISL_12970412, EPI_ISL_12970413, EPI_ISL_12970414, EPI_ISL_12970415, EPI_ISL_12970416, EPI_ISL_12970417, EPI_ISL_12970418, EPI_ISL_12970419, EPI_ISL_12970420, EPI_ISL_12970421, EPI_ISL_12970422, EPI_ISL_12970423, EPI_ISL_12970425, EPI_ISL_12970426, EPI_ISL_12970427, EPI_ISL_12970428			
see above	Department of Virology, Research Institute for Tropical Medicine	RVDT/RVD/DVD/CDC	Lijuan Wang, Mayan U. Lumandas, Vina Lea Arguelles, Jonjee Morin, Roman Tatusov and Everardo Vega
EPI_ISL_14018007, EPI_ISL_14018008, EPI_ISL_14039044, EPI_ISL_14039045, EPI_ISL_14039046, EPI_ISL_14039047, EPI_ISL_14039048, EPI_ISL_14039049, EPI_ISL_14039050	Infectious Diseases Research Collaboration (IDRC), Uganda	Chan Zuckerberg Biohub, USA	Greenhouse,Bryan; Kamya,Moses; Mwakibete,Lusajo; Rek,John; Rodriguez-Barraquer,Isabel; Ssewanyana,Isaac; Takahashi,Saki; Tato,Cristina
EPI_ISL_14170476	National Laboratory Service, Department of Virology; University of the Witwatersrand	National Institute for Communicable Diseases Center for Respiratory Diseases and Meningitis	Prince Mabilo, Hloniphile Mthiyane, Andiswa Simane, Kathleen Subramoney, Florette K. Treurnicht
EPI_ISL_14170477, EPI_ISL_14170847, EPI_ISL_14171764, EPI_ISL_14172564, EPI_ISL_14173335, EPI_ISL_14174342, EPI_ISL_14204579, EPI_ISL_14204580, EPI_ISL_14204581, EPI_ISL_14204582, EPI_ISL_14204583, EPI_ISL_14204584, EPI_ISL_14204585, EPI_ISL_14204586, EPI_ISL_14204587, EPI_ISL_14204588, EPI_ISL_14204589, EPI_ISL_14204590, EPI_ISL_14204591, EPI_ISL_14204592, EPI_ISL_14204593, EPI_ISL_14204594, EPI_ISL_14204595, EPI_ISL_14204596, EPI_ISL_14204597, EPI_ISL_14204598, EPI_ISL_14204599, EPI_ISL_14204600, EPI_ISL_14204601, EPI_ISL_14204602, EPI_ISL_14204603			
see above	Department of Virology; National Laboratory Service; University of the Witwatersrand	Department of Virology; National Laboratory Service; University of the Witwatersrand	Prince Mabilo, Hloniphile Mthiyane, Andiswa Simane, Kathleen Subramoney, Florette K. Treurnicht
EPI_ISL_1437530, EPI_ISL_1437532, EPI_ISL_1437534, EPI_ISL_1437536	Laboratorio de Salud Publica	INEI-ANLIS "Carlos G. Malbrán". Servicio Virosis Respiratorias.	Elsa Baumeister, Andrea Pontoriero, Russo Mara, Martin Avaro, Estefania Benedetti, Ana Campos
EPI_ISL_15055315, EPI_ISL_15055316, EPI_ISL_15055317, EPI_ISL_15055318, EPI_ISL_15055319, EPI_ISL_15055320, EPI_ISL_15055321, EPI_ISL_15055322, EPI_ISL_15055323, EPI_ISL_15055324, EPI_ISL_15055325, EPI_ISL_15067680, EPI_ISL_15067681, EPI_ISL_15067682, EPI_ISL_15067683, EPI_ISL_15067684, EPI_ISL_15067685, EPI_ISL_15067686, EPI_ISL_15067687, EPI_ISL_15067688, EPI_ISL_15067689, EPI_ISL_15067690			

see above	Virology Laboratory, Ricardo Gutiérrez Children's Hospital	Virology Laboratory, Ricardo Gutiérrez Children's Hospital	Acuña, Dolores; Goya, Stephanie; Nabaes Jodar, Mercedes S.; Lucion, M. Florencia; Juárez, María del Valle; Gentile, Ángela; Mistchenko, Alicia S.; Viegas, Mariana
EPI_ISL_15120785, EPI_ISL_15120786	National Institute of Hygiene, virology departement, National Influenza Center	Centers for Disease Control and Prevention, Coronavirus and Other Respiratory Viruses Division	Megha Aggarwal, Lijuan Wang, Ji In Park, Amanda Smith and Everardo Vega
EPI_ISL_1520381, EPI_ISL_1520382, EPI_ISL_1520383, EPI_ISL_1520384, EPI_ISL_1520385, EPI_ISL_1520386, EPI_ISL_1520387, EPI_ISL_1520388, EPI_ISL_1520389, EPI_ISL_1520390, EPI_ISL_1520391, EPI_ISL_1520392, EPI_ISL_1520393, EPI_ISL_1520394, EPI_ISL_1520395, EPI_ISL_1520396, EPI_ISL_1520397, EPI_ISL_1520398, EPI_ISL_1520399, EPI_ISL_1520400, EPI_ISL_1520401, EPI_ISL_1520402, EPI_ISL_1520403, EPI_ISL_1520404, EPI_ISL_1520405, EPI_ISL_1520406, EPI_ISL_1520407, EPI_ISL_1520408, EPI_ISL_1520409, EPI_ISL_1520410, EPI_ISL_1520411, EPI_ISL_1520412, EPI_ISL_1520413, EPI_ISL_1520414, EPI_ISL_1520415, EPI_ISL_1520416, EPI_ISL_1520417, EPI_ISL_1520418, EPI_ISL_1520419, EPI_ISL_1520420, EPI_ISL_1520421, EPI_ISL_1520422, EPI_ISL_1520423, EPI_ISL_1520424, EPI_ISL_1520425, EPI_ISL_1520426, EPI_ISL_1520427, EPI_ISL_1520428, EPI_ISL_1520429, EPI_ISL_1520430, EPI_ISL_1520431, EPI_ISL_1520432, EPI_ISL_1520433, EPI_ISL_1520434, EPI_ISL_1520435, EPI_ISL_1520436, EPI_ISL_1520437, EPI_ISL_1520438, EPI_ISL_1520439, EPI_ISL_1520440			
see above	Respiratory Virus Unit, National Infection Service, Public Health England	National Infection Service, Public Health England	Zambon M, Talts T, Ellis J, Miah S, Platt S
EPI_ISL_15750127, EPI_ISL_15750128	Department of virology, Post Graduate Institute of Medical Education & Research	Department of virology, Post Graduate Institute of Medical Education & Research	Sarkar,S., Shuchi,S., Ratho,R.K., Mohi,G.
EPI_ISL_15750167, EPI_ISL_15750168, EPI_ISL_15750169, EPI_ISL_15750170, EPI_ISL_15750171, EPI_ISL_15750172, EPI_ISL_15750173, EPI_ISL_15750174, EPI_ISL_15750175, EPI_ISL_15750176, EPI_ISL_15750177, EPI_ISL_15750178, EPI_ISL_15750179, EPI_ISL_15750180, EPI_ISL_15750181, EPI_ISL_15750182			
see above	Virology, Tehran University of Medical Sciences	Virology, Tehran University of Medical Sciences	Salimi,V., Tavakoli,F., Mokhtari-Azad,T., Ghavami,N.
EPI_ISL_15750187, EPI_ISL_15750188, EPI_ISL_15750189, EPI_ISL_15750190, EPI_ISL_15750191, EPI_ISL_15750196, EPI_ISL_15750197, EPI_ISL_15750198, EPI_ISL_15750199, EPI_ISL_15750200	Inserm UMR1137, Universite de Paris	Inserm UMR1137, Universite de Paris	Coppee,R., Chenane,H.R., Bridier-Nahmias,A., Tcherakian,C., Catherinot,E., Collin,G., Lebourgeois,S., Visseaux,B., Descamps,D., Vasse,M., Farfour,E., Coppee,R., Chenane,H.R., Bridier-Nahmias,A., Collin,G., Lebourgeois,S., Visseaux,B., Descamps,D.
EPI_ISL_15750247, EPI_ISL_15750248, EPI_ISL_15750249, EPI_ISL_15750250, EPI_ISL_15750251, EPI_ISL_15750252, EPI_ISL_15750253, EPI_ISL_15750254, EPI_ISL_15750255, EPI_ISL_15750256, EPI_ISL_15750257, EPI_ISL_15750258, EPI_ISL_15750259, EPI_ISL_15750260, EPI_ISL_15750261, EPI_ISL_15750262, EPI_ISL_15750263, EPI_ISL_15750264, EPI_ISL_15750265, EPI_ISL_15750266, EPI_ISL_15750267, EPI_ISL_15750268, EPI_ISL_15750269, EPI_ISL_15750270, EPI_ISL_15750271, EPI_ISL_15750272, EPI_ISL_15750273, EPI_ISL_15750274, EPI_ISL_15750275, EPI_ISL_15750276, EPI_ISL_15750277, EPI_ISL_15750278, EPI_ISL_15750279, EPI_ISL_15750280, EPI_ISL_15750281, EPI_ISL_15750282, EPI_ISL_15750283, EPI_ISL_15750284, EPI_ISL_15750285, EPI_ISL_15750286, EPI_ISL_15750287, EPI_ISL_15750288, EPI_ISL_15750289, EPI_ISL_15750290, EPI_ISL_15750291, EPI_ISL_15750292, EPI_ISL_15750293, EPI_ISL_15750294, EPI_ISL_15750295, EPI_ISL_15750296, EPI_ISL_15750297			
see above	Department of Community Medicine, Faculty of Medicine	Department of Community Medicine, Faculty of Medicine	Malasao,R., Chaiut,W., Tantipetcharawan,W., Tongphung,R., Charoensri,N., Takarn,P., Sudjaritruk,T., Maneekarn,N., Malasao,R., Chaiut,W., Tantipetcharawan,W., Tongphung,R., Charoensri,N., Takarn,P., Sudjaritruk,T., Maneekarn,N.
EPI_ISL_15750298	Virology Unit, Istituto Zooprofilattico Sperimentale Abruzzo e Molise	Virology Unit, Istituto Zooprofilattico Sperimentale Abruzzo e Molise	Curini,V., Marcacci,M., Camma,C., Lorusso,A., Boutiba-Ben Boubaker,I.
EPI_ISL_15750307, EPI_ISL_15750308, EPI_ISL_15750309, EPI_ISL_15750310, EPI_ISL_15750311, EPI_ISL_15750312, EPI_ISL_15750313, EPI_ISL_15750314, EPI_ISL_15750315, EPI_ISL_15750316, EPI_ISL_15750317, EPI_ISL_15750318, EPI_ISL_15750319, EPI_ISL_15750320, EPI_ISL_15750321, EPI_ISL_15750322, EPI_ISL_15750323, EPI_ISL_15750324, EPI_ISL_15750325, EPI_ISL_15750326, EPI_ISL_15750327, EPI_ISL_15750328, EPI_ISL_15750329, EPI_ISL_15750330, EPI_ISL_15750331, EPI_ISL_15750332, EPI_ISL_15750333, EPI_ISL_15750334, EPI_ISL_15750335, EPI_ISL_15750336, EPI_ISL_15750337, EPI_ISL_15750338, EPI_ISL_15750339, EPI_ISL_15750340, EPI_ISL_15750341, EPI_ISL_15750342, EPI_ISL_15750343, EPI_ISL_15750344, EPI_ISL_15750345, EPI_ISL_15750346, EPI_ISL_15750347, EPI_ISL_15750348, EPI_ISL_15750349, EPI_ISL_15750350, EPI_ISL_15750351, EPI_ISL_15750352, EPI_ISL_15750353, EPI_ISL_15750354, EPI_ISL_15750355, EPI_ISL_15750356, EPI_ISL_15750357, EPI_ISL_15750358, EPI_ISL_15750359, EPI_ISL_15750360, EPI_ISL_15750361, EPI_ISL_15750362, EPI_ISL_15750363, EPI_ISL_15750364, EPI_ISL_15750365, EPI_ISL_15750366, EPI_ISL_15750367, EPI_ISL_15750368, EPI_ISL_15750369, EPI_ISL_15750370, EPI_ISL_15750371, EPI_ISL_15750372, EPI_ISL_15750373, EPI_ISL_15750374, EPI_ISL_15750375, EPI_ISL_15750376, EPI_ISL_15750377, EPI_ISL_15750378, EPI_ISL_15750379, EPI_ISL_15750380, EPI_ISL_15750381, EPI_ISL_15750382, EPI_ISL_15750383, EPI_ISL_15750384, EPI_ISL_15750385, EPI_ISL_15750386, EPI_ISL_15750387, EPI_ISL_15750388, EPI_ISL_15750389, EPI_ISL_15750390, EPI_ISL_15750391, EPI_ISL_15750392, EPI_ISL_15750393, EPI_ISL_15750394, EPI_ISL_15750395, EPI_ISL_15750396, EPI_ISL_15750397, EPI_ISL_15750398, EPI_ISL_15750399, EPI_ISL_15750400, EPI_ISL_15750401, EPI_ISL_15750402, EPI_ISL_15750403, EPI_ISL_15750432, EPI_ISL_15750433, EPI_ISL_15750434, EPI_ISL_15750435, EPI_ISL_15750436, EPI_ISL_15750437, EPI_ISL_15750438, EPI_ISL_15750439, EPI_ISL_15750440, EPI_ISL_15750441, EPI_ISL_15750442, EPI_ISL_15750443, EPI_ISL_15750444, EPI_ISL_15750445, EPI_ISL_15750446, EPI_ISL_15750447, EPI_ISL_15750448, EPI_ISL_15750449, EPI_ISL_15750450, EPI_ISL_15750451, EPI_ISL_15750452, EPI_ISL_15750453, EPI_ISL_15750454, EPI_ISL_15750455, EPI_ISL_15750456, EPI_ISL_15750457, EPI_ISL_15750458, EPI_ISL_15750459, EPI_ISL_15750460, EPI_ISL_15750461, EPI_ISL_15750462, EPI_ISL_15750463, EPI_ISL_15750464, EPI_ISL_15750465, EPI_ISL_15750466, EPI_ISL_15750467, EPI_ISL_15750468, EPI_ISL_15750469, EPI_ISL_15750470, EPI_ISL_15750471, EPI_ISL_15750472, EPI_ISL_15750473, EPI_ISL_15750474, EPI_ISL_15750475, EPI_ISL_15750476, EPI_ISL_15750477, EPI_ISL_15750623, EPI_ISL_15750624, EPI_ISL_15750625, EPI_ISL_15750626, EPI_ISL_15750627, EPI_ISL_15750628, EPI_ISL_15750629, EPI_ISL_15750630, EPI_ISL_15750631, EPI_ISL_15750632, EPI_ISL_15750633, EPI_ISL_15750634, EPI_ISL_15750635, EPI_ISL_15750636, EPI_ISL_15750637, EPI_ISL_15750638, EPI_ISL_15750639, EPI_ISL_15750640, EPI_ISL_15750641, EPI_ISL_15750642, EPI_ISL_15750643, EPI_ISL_15750644, EPI_ISL_15750645, EPI_ISL_15750646, EPI_ISL_15750647, EPI_ISL_15750648, EPI_ISL_15750649, EPI_ISL_15750650, EPI_ISL_15750651, EPI_ISL_15750652, EPI_ISL_15750653, EPI_ISL_15750654, EPI_ISL_15750655, EPI_ISL_15750656, EPI_ISL_15750657, EPI_ISL_15750658, EPI_ISL_15750659, EPI_ISL_15750660, EPI_ISL_15750661, EPI_ISL_15750662, EPI_ISL_15750663, EPI_ISL_15750664, EPI_ISL_15750665, EPI_ISL_15750666, EPI_ISL_15750667, EPI_ISL_15750668, EPI_ISL_15750669, EPI_ISL_15750670, EPI_ISL_15750671, EPI_ISL_15750672, EPI_ISL_15750673, EPI_ISL_15750674, EPI_ISL_15750675, EPI_ISL_15750676, EPI_ISL_15750677, EPI_ISL_15750678, EPI_ISL_15750679			
see above	Department of Pediatrics, Center of Excellence in Clinical Virology	Department of Pediatrics, Center of Excellence in Clinical Virology	Thongpan,I.
EPI_ISL_15750682, EPI_ISL_15750683, EPI_ISL_15750684, EPI_ISL_15750685, EPI_ISL_15750686, EPI_ISL_15750687, EPI_ISL_15750688, EPI_ISL_15750689, EPI_ISL_15750690, EPI_ISL_15750691, EPI_ISL_15750692, EPI_ISL_15750693, EPI_ISL_15750698, EPI_ISL_15750699, EPI_ISL_15750978, EPI_ISL_15750979, EPI_ISL_15750980, EPI_ISL_15750981, EPI_ISL_15750982, EPI_ISL_15750983, EPI_ISL_15750984, EPI_ISL_15750985, EPI_ISL_15750986, EPI_ISL_15750988, EPI_ISL_15750989, EPI_ISL_15750990, EPI_ISL_15750991, EPI_ISL_15750992, EPI_ISL_15750993, EPI_ISL_15750994			
see above	Beijing Center for Disease Prevention and Control, Immunological Prevention	Beijing Center for Disease Prevention and Control, Immunological Prevention	Luo,Q., Li,M., Huang,F.
EPI_ISL_15751389, EPI_ISL_15751390, EPI_ISL_15751391, EPI_ISL_15751392, EPI_ISL_15751393, EPI_ISL_15751394, EPI_ISL_15751395, EPI_ISL_15751396, EPI_ISL_15751397, EPI_ISL_15751398, EPI_ISL_15751399, EPI_ISL_15751400, EPI_ISL_15751401, EPI_ISL_15751402, EPI_ISL_15751403, EPI_ISL_15751404, EPI_ISL_15751405, EPI_ISL_15751406, EPI_ISL_15751407, EPI_ISL_15751408, EPI_ISL_15751409, EPI_ISL_15751410, EPI_ISL_15751411, EPI_ISL_15751412, EPI_ISL_15751413, EPI_ISL_15751414, EPI_ISL_15751415, EPI_ISL_15751416, EPI_ISL_15751417, EPI_ISL_15751418, EPI_ISL_15751419, EPI_ISL_15751420, EPI_ISL_15751421			
see above	Pediatrics, Show-Chwan Memorial Hospital	Pediatrics, Show-Chwan Memorial Hospital	Lee,C.Y., Wu,T.H., Fang,Y.P., Chang,J.C., Wang,H.C., Lin,S.J., Mai,C.H., Chang,Y.C., Chou,T.Y., Lee,C.-Y.
EPI_ISL_15751580, EPI_ISL_15751581, EPI_ISL_15751582, EPI_ISL_15751583, EPI_ISL_15751584, EPI_ISL_15751585, EPI_ISL_15751586, EPI_ISL_15751587, EPI_ISL_15751588, EPI_ISL_15751589, EPI_ISL_15751590, EPI_ISL_15751591, EPI_ISL_15751592, EPI_ISL_15751593, EPI_ISL_15751594, EPI_ISL_15751595, EPI_ISL_15751596, EPI_ISL_15751597, EPI_ISL_15751598, EPI_ISL_15751599, EPI_ISL_15751600, EPI_ISL_15751601, EPI_ISL_15751602, EPI_ISL_15751603, EPI_ISL_15751604, EPI_ISL_15751605, EPI_ISL_15751606, EPI_ISL_15751607, EPI_ISL_15751608, EPI_ISL_15751609, EPI_ISL_15751610, EPI_ISL_15751611, EPI_ISL_15751612, EPI_ISL_15751613, EPI_ISL_15751614, EPI_ISL_15751615, EPI_ISL_15751616, EPI_ISL_15751617, EPI_ISL_15751618, EPI_ISL_15751619, EPI_ISL_15751620, EPI_ISL_15751621, EPI_ISL_15751622, EPI_ISL_15751623, EPI_ISL_15751624, EPI_ISL_15751625, EPI_ISL_15751626, EPI_ISL_15751627, EPI_ISL_15751628, EPI_ISL_15751629, EPI_ISL_15751630, EPI_ISL_15751631, EPI_ISL_15751632, EPI_ISL_15751633, EPI_ISL_15751634, EPI_ISL_15751635, EPI_ISL_15751636, EPI_ISL_15751637, EPI_ISL_15751638, EPI_ISL_15751639, EPI_ISL_15751640, EPI_ISL_15751641, EPI_ISL_15751642, EPI_ISL_15751643, EPI_ISL_15751644, EPI_ISL_15751645, EPI_ISL_15751646, EPI_ISL_15751647, EPI_ISL_15751648, EPI_ISL_15751649, EPI_ISL_15751650, EPI_ISL_15751651, EPI_ISL_15751652, EPI_ISL_15751653, EPI_ISL_15751654, EPI_ISL_15751655, EPI_ISL_15751656, EPI_ISL_15751657, EPI_ISL_15751658, EPI_ISL_15751659, EPI_ISL_15751660, EPI_ISL_15751661, EPI_ISL_15751662, EPI_ISL_15751663, EPI_ISL_15751664, EPI_ISL_15751665, EPI_ISL_15751666, EPI_ISL_15751667, EPI_ISL_15751668, EPI_ISL_15751669, EPI_ISL_15751670, EPI_ISL_15751671, EPI_ISL_15751672, EPI_ISL_15751673, EPI_ISL_15751674, EPI_ISL_15751675, EPI_ISL_15751676, EPI_ISL_15751677, EPI_ISL_15751678, EPI_ISL_15751679, EPI_ISL_15751680, EPI_ISL_15751681, EPI_ISL_15751682, EPI_ISL_15751683, EPI_ISL_15751684, EPI_ISL_15751685, EPI_ISL_15751686, EPI_ISL_15751687, EPI_ISL_15751688, EPI_ISL_15751689, EPI_ISL_15751690, EPI_ISL_15751691, EPI_ISL_15751692, EPI_ISL_15751693, EPI_ISL_15751694, EPI_ISL_15751695, EPI_ISL_15751696, EPI_ISL_15751697, EPI_ISL_15751698, EPI_ISL_15751699, EPI_ISL_15751700, EPI_ISL_15751701, EPI_ISL_15751702, EPI_ISL_15751703, EPI_ISL_15751704, EPI_ISL_15751705, EPI_ISL_15751706, EPI_ISL_15751707, EPI_ISL_15751708, EPI_ISL_15751709, EPI_ISL_15751710, EPI_ISL_15751711, EPI_ISL_15751712, EPI_ISL_15751713, EPI_ISL_15751714, EPI_ISL_15751715, EPI_ISL_15751716, EPI_ISL_15751717, EPI_ISL_15751718, EPI_ISL_15751719, EPI_ISL_15751720, EPI_ISL_15751721, EPI_ISL_15751722, EPI_ISL_15751723, EPI_ISL_15751724, EPI_ISL_15751725, EPI_ISL_15751726, EPI_ISL_15751727, EPI_ISL_15751728, EPI_ISL_15751729, EPI_ISL_15751730, EPI_ISL_15751731, EPI_ISL_15751732, EPI_ISL_15751733, EPI_ISL_15751734, EPI_ISL_15751735, EPI_ISL_15751736, EPI_ISL_15751737, EPI_ISL_15751738, EPI_ISL_15751739, EPI_ISL_15751740, EPI_ISL_15751741, EPI_ISL_15751742, EPI_ISL_15751743, EPI_ISL_15751744, EPI_ISL_15751745, EPI_ISL_15751746, EPI_ISL_15751747, EPI_ISL_15751748, EPI_ISL_15751749, EPI_ISL_15751750, EPI_ISL_15751751, EPI_ISL_15751752, EPI_ISL_15751753, EPI_ISL_15751754, EPI_ISL_15751755, EPI_ISL_15751756, EPI_ISL_15751757, EPI_ISL_15751758, EPI_ISL_15751759, EPI_ISL_15751760, EPI_ISL_15751761, EPI_ISL_15751762, EPI_ISL_15751763, EPI_ISL_15751764, EPI_ISL_15751765, EPI_ISL_15751766, EPI_ISL_15751767, EPI_ISL_15751768, EPI_ISL_15751769, EPI_ISL_15751770, EPI_ISL_15751771, EPI_ISL_15751772, EPI_ISL_15751773, EPI_ISL_15751774, EPI_ISL_15751775, EPI_ISL_15751776, EPI_ISL_15751777, EPI_ISL_15751778, EPI_ISL_15751779, EPI_ISL_15751780, EPI_ISL_15751781, EPI_ISL_15751782, EPI_ISL_15751783, EPI_ISL_15751784, EPI_ISL_15751785, EPI_ISL_15751786, EPI_ISL_15751787, EPI_ISL_15751788, EPI_ISL_15751789, EPI_ISL_15751790, EPI_ISL_15751791, EPI_ISL_15751792, EPI_ISL_15751793, EPI_ISL_15751794, EPI_ISL_15751795, EPI_ISL_15751796, EPI_ISL_15751797, EPI_ISL_15751798, EPI_ISL_15751799, EPI_ISL_15751800, EPI_ISL_15751801, EPI_ISL_15751802, EPI_ISL_15751803, EPI_ISL_15751804, EPI_ISL_15751805, EPI_ISL_15751806, EPI_ISL_15751807, EPI_ISL_15751808, EPI_ISL_15751809, EPI_ISL_15751810, EPI_ISL_15751811, EPI_ISL_15751812, EPI_ISL_15751813, EPI_ISL_15751814, EPI_ISL_15751815, EPI_ISL_15751816, EPI_ISL_15751817, EPI_ISL_15751818, EPI_ISL_15751819, EPI_ISL_15751820, EPI_ISL_15751821, EPI_ISL_15751822, EPI_ISL_15751823, EPI_ISL_15751824, EPI_ISL_15751825, EPI_ISL_15751826, EPI_ISL_15751827, EPI_ISL_15751828, EPI_ISL_15751829, EPI_ISL_15751830, EPI_ISL_15751831, EPI_ISL_15751832, EPI_ISL_15751833, EPI_ISL_15751834, EPI_ISL_15751835, EPI_ISL_15751836, EPI_ISL_15751837, EPI_ISL_15751838, EPI_ISL_15751839, EPI_ISL_15751840, EPI_ISL_15751841, EPI_ISL_15751842, EPI_ISL_15751843, EPI_ISL_15751844, EPI_ISL_15751845, EPI_ISL_15751846, EPI_ISL_15751847, EPI_ISL_15751848, EPI_ISL_15751849, EPI_ISL_15751850, EPI_ISL_15751851, EPI_ISL_15751852, EPI_ISL_15751853, EPI_ISL_15751854, EPI_ISL_15751855, EPI_ISL_15751856, EPI_ISL_15751857, EPI_ISL_15751858, EPI_ISL_15751859, EPI_ISL_15751860, EPI_ISL_15751861, EPI_ISL_15751862, EPI_ISL_15751863, EPI_ISL_15751864, EPI_ISL_15751865, EPI_ISL_15751866, EPI_ISL_15751867, EPI_ISL_15751868, EPI_ISL_15751869, EPI_ISL_15751870, EPI_ISL_15751871, EPI_ISL_15751872, EPI_ISL_15751873, EPI_ISL_15751874, EPI_ISL_15751875, EPI_ISL_15751876, EPI_ISL_15751877, EPI_ISL_15751878, EPI_ISL_15751879, EPI_ISL_15751880, EPI_ISL_15751881, EPI_ISL_15751882, EPI_ISL_15751883, EPI_ISL_15751884, EPI_ISL_15751885, EPI_ISL_15751886, EPI_ISL_15751887, EPI_ISL_15751888, EPI_ISL_15751889, EPI_ISL_15751890, EPI_ISL_15751891, EPI_ISL_15751892, EPI_ISL_15751893, EPI_ISL_15751894, EPI_ISL_15751895, EPI_ISL_15751896, EPI_ISL_15751897, EPI_ISL_15751898, EPI_ISL_15751899, EPI_ISL_15751900, EPI_ISL_15751901, EPI_ISL_15751902, EPI_ISL_15751903, EPI_ISL_15751904, EPI_ISL_15751905, EPI_ISL_15751906, EPI_ISL_15751907, EPI_ISL_15751908, EPI_ISL_15751909, EPI_ISL_15751910, EPI_ISL_15751911, EPI_ISL_15751912, EPI_ISL_15751913, EPI_ISL_15751914, EPI_ISL_15751915, EPI_ISL_15751916, EPI_ISL_15751917, EPI_ISL_15751918, EPI_ISL_15751919, EPI_ISL_15751920, EPI_ISL_15751921, EPI_ISL_15751922, EPI_ISL_15751923, EPI_ISL_15751924, EPI_ISL_15751925, EPI_ISL_15751926, EPI_ISL_15751927, EPI_ISL_15751928, EPI_ISL_15751929, EPI_ISL_15751930, EPI_ISL_15751931, EPI_ISL_15751932, EPI_ISL_15751933, EPI_ISL_15751934, EPI_ISL_15751935, EPI_ISL_15751936, EPI_ISL_15751937, EPI_ISL_15751938, EPI_ISL_15751939, EPI_ISL_15751940, EPI_ISL_15751941, EPI_ISL_15751942			
see above	Microbiology, Hospices Civiles de Lyon	Microbiology, Hospices Civiles de Lyon	Vidoni,L.
EPI_ISL_15752721, EPI_ISL_15752722, EPI_ISL_15752729, EPI_ISL_15752730, EPI_ISL_15752731, EPI_ISL_15752732, EPI_ISL_15752733, EPI_ISL_15752735, EPI_ISL_15752741, EPI_ISL_15752746, EPI_ISL_15752748, EPI_ISL_15752752, EPI_ISL_15752763, EPI_ISL_15752768, EPI_ISL_15752770, EPI_ISL_15752773, EPI_ISL_15752775, EPI_ISL_15752776, EPI_ISL_15752782, EPI_ISL_15752783, EPI_ISL_15752786, EPI_ISL_15752787, EPI_ISL_15752788, EPI_ISL_15752799, EPI_ISL_15752800, EPI_ISL_15752801, EPI_ISL_15752802, EPI_ISL_15752806, EPI_ISL_15752809, EPI_ISL_15752814, EPI_ISL_15752815, EPI_ISL_15752817, EPI_ISL_15752818, EPI_ISL_15752821, EPI_ISL_15752824, EPI_ISL_15752826, EPI_ISL_15752827, EPI_ISL_15752832, EPI_ISL_15752837, EPI_ISL_15752838, EPI_ISL_15752839, EPI_ISL_15752840, EPI_ISL_15752842, EPI_ISL_15752843, EPI_ISL_15752844, EPI_ISL_15752845, EPI_ISL_15752846, EPI_ISL_15752848, EPI_ISL_15752849, EPI_ISL_15752850, EPI_ISL_15752852, EPI_ISL_15752853, EPI_ISL_15752854, EPI_ISL_15752855, EPI_ISL_15752856, EPI_ISL_15752859, EPI_ISL_15752860, EPI_ISL_15752861, EPI_ISL_15752862, EPI_ISL_15752863, EPI_ISL_15752864, EPI_ISL_15752866, EPI_ISL_15752867			
see above	Pneumococcal Research Group, Infections and Immunity	Pneumococcal Research Group, Infections and Immunity	Anderson,J., Oeum,M., Verkolf,E., Licciardi,P.V., Mulholland,K., Nguyen,C., Chow,K., Waller,G., Costa,A.M., Daley,A., Crawford,N.W., Babl,F.E., Duke,T., Do,L.A.H., Wurzel,D., Anderson,J., Oeum,M., Verkolf,E., Licciardi,P.V., Mulholland,K.E., Nguyen,C., Chow,K., Waller,G., Costa,A.-M., Daley,A.J., Crawford,N., Babl,F., Duke,T., Do,L.A.H., Wurzel,D.
EPI_ISL_15753254, EPI_ISL_15753255, EPI_ISL_15753256, EPI_ISL_15753257, EPI_ISL_15753259, EPI_ISL_15753260, EPI_ISL_15753263, EPI_ISL_15753264, EPI_ISL_15753265, EPI_ISL_15753266, EPI_ISL_15753271, EPI_ISL_15753272, EPI_ISL_15753273, EPI_ISL_15753274, EPI_ISL_15753279, EPI_ISL_15753280, EPI_ISL_15753282, EPI_ISL_15753284, EPI_ISL_15753285, EPI_ISL_15753290, EPI_ISL_15753291, EPI_ISL_15753292, EPI_ISL_15753293, EPI_ISL_15753294, EPI_ISL_15753295, EPI_ISL_15753297, EPI_ISL_15753300, EPI_ISL_15753303, EPI_ISL_15753305, EPI_ISL_15753307, EPI_ISL_15753309, EPI_ISL_15753310, EPI_ISL_15753311, EPI_ISL_15753314, EPI_ISL_15753315, EPI_ISL_15753316, EPI_ISL_15753318, EPI_ISL_15753319, EPI_ISL_15753320, EPI_ISL_15753321, EPI_ISL_15753323, EPI_ISL_15753324, EPI_ISL_15753325, EPI_ISL_15753326, EPI_ISL_15753327, EPI_ISL_15753331, EPI_ISL_15753332, EPI_ISL_15753333, EPI_ISL_15753334, EPI_ISL_15753336, EPI_ISL_15753338, EPI_ISL_15753340, EPI_ISL_15753343, EPI_ISL_15753344, EPI_ISL_15753345, EPI_ISL_15753346, EPI_ISL_15753350, EPI_ISL_15753351, EPI_ISL_15753356, EPI_ISL_15753357, EPI_ISL_15753358, EPI_ISL_15753362, EPI_ISL_15753363, EPI_ISL_15753370, EPI_ISL_15753371, EPI_ISL_15753372, EPI_ISL_15753375, EPI_ISL_15753377, EPI_ISL_15753379, EPI_ISL_15753384, EPI_ISL_15753386, EPI_ISL_15753387, EPI_ISL_15753390, EPI_ISL_15753394, EPI_ISL_15753395, EPI_ISL_15753397, EPI_ISL_15753399, EPI_ISL_15753400, EPI_ISL_15753402, EPI_ISL_15753407, EPI_ISL_15753408, EPI_ISL_15753414, EPI_ISL_15753416, EPI_ISL_15753419, EPI_ISL_15753420, EPI_ISL_15753422, EPI_ISL_15753424, EPI_ISL_15753429, EPI_ISL_15753430, EPI_ISL_15753433, EPI_ISL_15753437, EPI_ISL_15753439, EPI_ISL_15753446, EPI_ISL_15753447, EPI_ISL_15753448, EPI_ISL_15753449, EPI_ISL_15753451, EPI_ISL_15753452, EPI_ISL_15753454, EPI_ISL_15753457, EPI_ISL_15753459, EPI_ISL_15753460, EPI_ISL_15753461, EPI_ISL_15753462, EPI_ISL_15753465, EPI_ISL_15753467, EPI_ISL_15753470, EPI_ISL_15753471, EPI_ISL_15753472, EPI_ISL_15753473, EPI_ISL_15753474, EPI_ISL_15753476, EPI_ISL_15753477, EPI_ISL_15753478, EPI_ISL_15753480, EPI_ISL_15753482, EPI_ISL_15753483, EPI_ISL_15753484, EPI_ISL_15753485, EPI_ISL_15753486, EPI_ISL_15753490, EPI_ISL_15753491, EPI_ISL_15753492, EPI_ISL_15753494, EPI_ISL_15753496, EPI_ISL_15753498, EPI_ISL_15753499, EPI_ISL_15753500, EPI_ISL_15753502, EPI_ISL_15753503, EPI_ISL_15753504, EPI_ISL_15753505, EPI_ISL_15753508, EPI_ISL_15753509, EPI_ISL_15753510, EPI_ISL_15753512, EPI_ISL_15753515, EPI_ISL_15753516, EPI_ISL_15753517, EPI_ISL_15753519, EPI_ISL_15753520, EPI_ISL_15753521, EPI_ISL_15753524, EPI_ISL_15753525, EPI_ISL_15753526, EPI_ISL_15753528, EPI_ISL_15753529, EPI_ISL_15753530, EPI_ISL_15753531, EPI_ISL_15753535, EPI_ISL_15753538, EPI_ISL_15753539, EPI_ISL_15753540, EPI_ISL_15753541, EPI_ISL_15753542, EPI_ISL_15753545, EPI_ISL_15753547, EPI_ISL_15753548, EPI_ISL_15753550, EPI_ISL_15753551, EPI_ISL_15753552, EPI_ISL_15753554, EPI_ISL_15753555, EPI_ISL_15753557, EPI_ISL_15753559, EPI_ISL_15753560, EPI_ISL_15753562, EPI_ISL_15753563, EPI_ISL_15753564, EPI_ISL_15753568			
see above	Department of Paediatrics, University of Oxford	Department of Paediatrics, University of Oxford	Lin,G.-L., Drysdale,S.B., Snape,M.D., O'Connor,D., Brown,A., MacIntyre-Cockett,G., Mellado-Gomez,E., de Cesare,M., Bonsall,D., Ansari,M.A., Oner,D., Aerssens,J., Butler,C., Bont,L., Openshaw,P., Martinon-Torres,F., Nair,H., Bowden,R., Golubchik,T., Pollard,A.J., Lin,G.-L.
EPI_ISL_15753570, EPI_ISL_15753574	Eurasian Institute of zoonotic infections, The Federal Research Center of Fundamental and Translational Medicine	Eurasian Institute of zoonotic infections, The Federal Research Center of Fundamental and Translational Medicine	Dubovitskiy,N.A., Sobolev,I.A., Kurskaya,O.G., Anoshina,A.V., Leonova,N.V., Murashkina,T.A., Solomatina,M.V., Derko,A.A., Saroyan,T.A., Kabilov,M.R., Tupikin,A.E., Sharshov,K.A., Shestopalov,A.M.
EPI_ISL_15753575, EPI_ISL_15753576, EPI_ISL_15753577	Eurasian Institute of zoonotic infections, The Federal Research Center of Fundamental and Translational Medicine	Eurasian Institute of zoonotic infections, The Federal Research Center of Fundamental and Translational Medicine	Dubovitskiy,N.A., Sobolev,I.A., Kurskaya,O.G., Simkina,O.A., Komissarova,T.V., Murashkina,T.A., Solomatina,M.V., Derko,A.A., Saroyan,T.A., Kabilov,M.R., Tupikin,A.E., Sharshov,K.A., Shestopalov,A.M.
EPI_ISL_15771669, EPI_ISL_15771670, EPI_ISL_15771671, EPI_ISL_15771673, EPI_ISL_15771675, EPI_ISL_15771677, EPI_ISL_15771678, EPI_ISL_15771681, EPI_ISL_15771683, EPI_ISL_15771685, EPI_ISL_15771687, EPI_ISL_15771689, EPI_ISL_15771690, EPI_ISL_15771692, EPI_ISL_15771694, EPI_ISL_15771696, EPI_ISL_15771698, EPI_ISL_15771700, EPI_ISL_15771702, EPI_ISL_15771703, EPI_ISL_15771705, EPI_ISL_15771707, EPI_ISL_15771709, EPI_ISL_15771711, EPI_ISL_15771713, EPI_ISL_15771715, EPI_ISL_15771717, EPI_ISL_15771718, EPI_ISL_15771720, EPI_ISL_15771721, EPI_ISL_15771723, EPI_ISL_15771725, EPI_ISL_15771727, EPI_ISL_15771756, EPI_ISL_15771758, EPI_ISL_15771759, EPI_ISL_15771761, EPI_ISL_15771763, EPI_ISL_15771764, EPI_ISL_15771766, EPI_ISL_15771768, EPI_ISL_15771770, EPI_ISL_15771772, EPI_ISL_15771774, EPI_ISL_15771776, EPI_ISL_15771778, EPI_ISL_15771779, EPI_ISL_15771782, EPI_ISL_15771783, EPI_ISL_15771786, EPI_ISL_15771788, EPI_ISL_15771790, EPI_ISL_15771792, EPI_ISL_15771794, EPI_ISL_15771795, EPI_ISL_15771797, EPI_ISL_15771800, EPI_ISL_15771801, EPI_ISL_15771803, EPI_ISL_15771805, EPI_ISL_15771806, EPI_ISL_15771808, EPI_ISL_15771810, EPI_ISL_15771812, EPI_ISL_15771814, EPI_ISL_15771816, EPI_ISL_15771817, EPI_ISL_15771857, EPI_ISL_15771858, EPI_ISL_15771861, EPI_ISL_15771863, EPI_ISL_15771865, EPI_ISL_15771866, EPI_ISL_15771868, EPI_ISL_15771870, EPI_ISL_15771872, EPI_ISL_15771874, EPI_ISL_15771875, EPI_ISL_15771877, EPI_ISL_15771879, EPI_ISL_15771881, EPI_ISL_15771883, EPI_ISL_15771884, EPI_ISL_15771886, EPI_ISL_15771888, EPI_ISL_15771890, EPI_ISL_15771892, EPI_ISL_15771893, EPI_ISL_15771895, EPI_ISL_15771897, EPI_ISL_15771899, EPI_ISL_15771901, EPI_ISL_15771902, EPI_ISL_15771904, EPI_ISL_15771906, EPI_ISL_15771908, EPI_ISL_15771910, EPI_ISL_15771912, EPI_ISL_15771913, EPI_ISL_15771915, EPI_ISL_15771917, EPI_ISL_15771918, EPI_ISL_15771920, EPI_ISL_15771922, EPI_ISL_15771937, EPI_ISL_15771939, EPI_ISL_15771941, EPI_ISL_15771943, EPI_ISL_15771944, EPI_ISL_15771946, EPI_ISL_15771948, EPI_ISL_15771950, EPI_ISL_15771952, EPI_ISL_15771954, EPI_ISL_15771956, EPI_ISL_15771958, EPI_ISL_15771959, EPI_ISL_15771961, EPI_ISL_15771963, EPI_ISL_15771965, EPI_ISL_15771967, EPI_ISL_15771969, EPI_ISL_15771971, EPI_ISL_15771972, EPI_ISL_15771974, EPI_ISL_15771976, EPI_ISL_15771978, EPI_ISL_15771979, EPI_ISL_15771981, EPI_ISL_15771983, EPI_ISL_15771985, EPI_ISL_15771987, EPI_ISL_15771989, EPI_ISL_15771991, EPI_ISL_15771993, EPI_ISL_15771995, EPI_ISL_15771997, EPI_ISL_15771998, EPI_ISL_15772000			
see above	Pathology and Laboratory Medicine, Children's Mercy	Pathology and Laboratory Medicine, Children's Mercy	Adhikari,B., Hassan,F., Harrison,C., Dien Bard,J., Dunn,J., Kehl,S. and Selvarangan,R.
EPI_ISL_15773984, EPI_ISL_15773986, EPI_ISL_15773987, EPI_ISL_15773990, EPI_ISL_15773991, EPI_ISL_15773992, EPI_ISL_15773993, EPI_ISL_15773994, EPI_ISL_15773995, EPI_ISL_15773997, EPI_ISL_15773998, EPI_ISL_15773999, EPI_ISL_15774000, EPI_ISL_15774001, EPI_ISL_15774006, EPI_ISL_15774007, EPI_ISL_15774008, EPI_ISL_15774009, EPI_ISL_15774010, EPI_ISL_15774011, EPI_ISL_15774012, EPI_ISL_15774013, EPI_ISL_15774014, EPI_ISL_15774015, EPI_ISL_15774016, EPI_ISL_15774017, EPI_ISL_15774018, EPI_ISL_15774020, EPI_ISL_15774021, EPI_ISL_15774022, EPI_ISL_15774023, EPI_ISL_15774024, EPI_ISL_15774025, EPI_ISL_15774026, EPI_ISL_15774027, EPI_ISL_15774029, EPI_ISL_15774030, EPI_ISL_15774031, EPI_ISL_15774032, EPI_ISL_15774033, EPI_ISL_15774035, EPI_ISL_15774038, EPI_ISL_15774039, EPI_ISL_15774042, EPI_ISL_15774043, EPI_ISL_15774045, EPI_ISL_15774046, EPI_ISL_15774047, EPI_ISL_15774049, EPI_ISL_15774050, EPI_ISL_15774053, EPI_ISL_15774054, EPI_ISL_15774056, EPI_ISL_15774058, EPI_ISL_15774059, EPI_ISL_15774060, EPI_ISL_15774062, EPI_ISL_15774063, EPI_ISL_15774064, EPI_ISL_15774065, EPI_ISL_15774066, EPI_ISL_15774067, EPI_ISL_15774068, EPI_ISL_15774070, EPI_ISL_15774071			
see above	QLD, Royal Brisbane and Woman's Hospital	WHO Collaborating Centre for Reference and Research on Influenza	Xiaomin Dong, Yi-Mo Deng, Ammar Aziz, Naomi Komadina
EPI_ISL_15820270, EPI_ISL_15820271	Tokyo Metropolitan Institute of Public Health, Department of Microbiology	Tokyo Metropolitan Institute of Public Health, Department of Microbiology	Kasuya,F., Mori,K., Suzuki,J., Sadamasu,K., Kasuya,F., Mori,K., Suzuki,J., Sadamasu,K.
EPI_ISL_15894966, EPI_ISL_15894968, EPI_ISL_15894969, EPI_ISL_15894970, EPI_ISL_15894971, EPI_ISL_15894972, EPI_ISL_15894973, EPI_ISL_15894974, EPI_ISL_15894976, EPI_ISL_15894978, EPI_ISL_15894979, EPI_ISL_15894980, EPI_ISL_15894981, EPI_ISL_15894983, EPI_ISL_15894984, EPI_ISL_15894985, EPI_ISL_15894986, EPI_ISL_15894987, EPI_ISL_15894988, EPI_ISL_15894989, EPI_ISL_15894990, EPI_ISL_15894991, EPI_ISL_15894993, EPI_ISL_15894994, EPI_ISL_15894995, EPI_ISL_15894996, EPI_ISL_15894997, EPI_ISL_15894998, EPI_ISL_15895000, EPI_ISL_15895003, EPI_ISL_15895004, EPI_ISL_15895005, EPI_ISL_15895007, EPI_ISL_15895008, EPI_ISL_15895010, EPI_ISL_15895011, EPI_ISL_15895012, EPI_ISL_15895013, EPI_ISL_15895014, EPI_ISL_15895015,			

EPI_ISL_15895016, EPI_ISL_15895017			
see above	Laboratorio Central de Saude Publica do Estado do Rio Grande do Sul (LACEN/RS)	Laboratory of Respiratory Viruses and Measles, Oswaldo Cruz Institute, FIOCRUZ	Paola Resende, Fernando Motta, Elisa Cavalcante Pereira, Bruna Mendonça da Silva, Jéssica Graça Macedo de Carvalho, Larissa Macedo Pinto, Victor Guimaraes, Ighor Leonardo Arantes, Leticia Scalioni, Anderson Brandao Leite, Marilda Siqueira on behalf of the Fiocruz COVID-19 Genomic Surveillance Network
EPI_ISL_15895124	Universidade Federal de São Paulo, Departamento de Medicina, Disciplina de Doenças Infecciosas e Parasitárias. Laboratório de Virologia	Laboratory of Respiratory Viruses and Measles, Oswaldo Cruz Institute, FIOCRUZ	Paola Resende, Fernando Motta, Elisa Cavalcante Pereira, Ighor Arantes, Bruna Mendonça da Silva, Jéssica Graça Macedo de Carvalho, Larissa Macedo Pinto, Victor Guimaraes, Leticia Scalioni, Ana Helena Perosa, Gabriela Rodrigues Barbosa, Nancy Bellei e Marilda Siqueira on behalf of the Fiocruz COVID-19 Genomic Surveillance Network
EPI_ISL_15895129	Instituto Fernandes Figueira (IFF/FIOCRUZ)	Laboratory of Respiratory Viruses and Measles, Oswaldo Cruz Institute, FIOCRUZ	Paola Resende, Luciana Appolinario, Fernando Motta, Elisa Cavalcante Pereira, Ighor Arantes, Alice Sampaio Rocha, Bruna Mendonça da Silva, Jéssica Graça Macedo de Carvalho, Larissa Macedo Pinto, Victor Guimaraes, Leticia Scalioni, Zilton Vasconcelos, Silvia Leite, Marilda Siqueira on behalf of the Fiocruz COVID-19 Genomic Surveillance Network
EPI_ISL_15895130	Laboratorio Central de Saude Publica do Estado do Amapa (LACEN/AP)	Laboratory of Respiratory Viruses and Measles, Oswaldo Cruz Institute, FIOCRUZ	Paola Resende, Fernando Motta, Elisa Cavalcante Pereira, Ighor Arantes, Bruna Mendonça da Silva, Jéssica Graça Macedo de Carvalho, Larissa Macedo Pinto, Victor Guimaraes, Leticia Scalioni, Andreia Santos Costa, Marcia Socorro Pereira Cavalcante, Anne Caroline da Silva Soledade, Lindomar dos Anjos Silva, Marilda Siqueira on behalf of the Fiocruz COVID-19 Genomic Surveillance Network
EPI_ISL_15895131	Instituto Fernandes Figueira (IFF/FIOCRUZ)	Laboratory of Respiratory Viruses and Measles, Oswaldo Cruz Institute, FIOCRUZ	Paola Resende, Luciana Appolinario, Fernando Motta, Elisa Cavalcante Pereira, Ighor Arantes, Alice Sampaio Rocha, Bruna Mendonça da Silva, Jéssica Graça Macedo de Carvalho, Larissa Macedo Pinto, Victor Guimaraes, Leticia Scalioni, Zilton Vasconcelos, Silvia Leite, Marilda Siqueira on behalf of the Fiocruz COVID-19 Genomic Surveillance Network
EPI_ISL_15895133, EPI_ISL_15895134	Universidade Federal de São Paulo, Departamento de Medicina, Disciplina de Doenças Infecciosas e Parasitárias. Laboratório de Virologia	Laboratory of Respiratory Viruses and Measles, Oswaldo Cruz Institute, FIOCRUZ	Paola Resende, Fernando Motta, Elisa Cavalcante Pereira, Ighor Arantes, Bruna Mendonça da Silva, Jéssica Graça Macedo de Carvalho, Larissa Macedo Pinto, Victor Guimaraes, Leticia Scalioni, Ana Helena Perosa, Gabriela Rodrigues Barbosa, Nancy Bellei e Marilda Siqueira on behalf of the Fiocruz COVID-19 Genomic Surveillance Network
EPI_ISL_15895140, EPI_ISL_15895141	Laboratory of Respiratory Viruses and Measles, Oswaldo Cruz Institute, FIOCRUZ	Laboratory of Respiratory Viruses and Measles, Oswaldo Cruz Institute, FIOCRUZ	Paola Resende, Luciana Appolinario, Fernando Motta, Elisa Cavalcante Pereira, Ighor Arantes, Alice Sampaio Rocha, Bruna Mendonça da Silva, Jéssica Graça Macedo de Carvalho, Larissa Macedo Pinto, Victor Guimaraes, Leticia Scalioni, Marilda Siqueira on behalf of the Fiocruz COVID-19 Genomic Surveillance Network
EPI_ISL_15895142	Universidade Federal de São Paulo, Departamento de Medicina, Disciplina de Doenças Infecciosas e Parasitárias. Laboratório de Virologia	Laboratory of Respiratory Viruses and Measles, Oswaldo Cruz Institute, FIOCRUZ	Paola Resende, Fernando Motta, Elisa Cavalcante Pereira, Ighor Arantes, Bruna Mendonça da Silva, Jéssica Graça Macedo de Carvalho, Larissa Macedo Pinto, Victor Guimaraes, Leticia Scalioni, Ana Helena Perosa, Gabriela Rodrigues Barbosa, Nancy Bellei e Marilda Siqueira on behalf of the Fiocruz COVID-19 Genomic Surveillance Network
EPI_ISL_15969898, EPI_ISL_15969921, EPI_ISL_15969922, EPI_ISL_15969923, EPI_ISL_15969924, EPI_ISL_15969926, EPI_ISL_15969927, EPI_ISL_15969928, EPI_ISL_15969929, EPI_ISL_15969931, EPI_ISL_15970064, EPI_ISL_15970065, EPI_ISL_15970066, EPI_ISL_15970067, EPI_ISL_15970068, EPI_ISL_15970069, EPI_ISL_15970070, EPI_ISL_15970304, EPI_ISL_15970720, EPI_ISL_15970961, EPI_ISL_15979223, EPI_ISL_15979224, EPI_ISL_15979225, EPI_ISL_15979226, EPI_ISL_15979227, EPI_ISL_15979228, EPI_ISL_15979229, EPI_ISL_15979730, EPI_ISL_15979731, EPI_ISL_15979732, EPI_ISL_15980233, EPI_ISL_15980436, EPI_ISL_15980516, EPI_ISL_15980517, EPI_ISL_15980518, EPI_ISL_15980519, EPI_ISL_15980520, EPI_ISL_15980521, EPI_ISL_15980522, EPI_ISL_15980523, EPI_ISL_15980604, EPI_ISL_15980605, EPI_ISL_15980606, EPI_ISL_15980607, EPI_ISL_15980608, EPI_ISL_15980612, EPI_ISL_15980613, EPI_ISL_15980614, EPI_ISL_16003269, EPI_ISL_16003271, EPI_ISL_16003272, EPI_ISL_16003274, EPI_ISL_16003276, EPI_ISL_16003278, EPI_ISL_16003280, EPI_ISL_16003283, EPI_ISL_16003284, EPI_ISL_16003286, EPI_ISL_16003288, EPI_ISL_16003289, EPI_ISL_16003290, EPI_ISL_16003291, EPI_ISL_16003292, EPI_ISL_16003294, EPI_ISL_16003296, EPI_ISL_16003298, EPI_ISL_16003300, EPI_ISL_16003302, EPI_ISL_16003307, EPI_ISL_16003309, EPI_ISL_16003311, EPI_ISL_16003312, EPI_ISL_16003313, EPI_ISL_16003314, EPI_ISL_16003315, EPI_ISL_16003316, EPI_ISL_16003317, EPI_ISL_16003318, EPI_ISL_16003319, EPI_ISL_16003320, EPI_ISL_16003321, EPI_ISL_16003322, EPI_ISL_16003323, EPI_ISL_16003324, EPI_ISL_16003325, EPI_ISL_16003326, EPI_ISL_16003327, EPI_ISL_16003328, EPI_ISL_16003329, EPI_ISL_16003331, EPI_ISL_16003332, EPI_ISL_16003333, EPI_ISL_16003334, EPI_ISL_16003335, EPI_ISL_16003336, EPI_ISL_16003337, EPI_ISL_16003357, EPI_ISL_16003358, EPI_ISL_16003359, EPI_ISL_16003388, EPI_ISL_16003416, EPI_ISL_16003417, EPI_ISL_16003418			
see above	Niigata University (IDRC) Infectious Diseases Research Center	Niigata University (IDRC) Infectious Diseases Research Center	Sayaka Yoshioka, Wint Wint Phyu, Irina Chon, Reiko Saito
EPI_ISL_16051837, EPI_ISL_16051838, EPI_ISL_16051839, EPI_ISL_16051840, EPI_ISL_16051841, EPI_ISL_16051842, EPI_ISL_16051843, EPI_ISL_16051844, EPI_ISL_16051845, EPI_ISL_16051846, EPI_ISL_16051847, EPI_ISL_16051849, EPI_ISL_16051861, EPI_ISL_16051862, EPI_ISL_16051867, EPI_ISL_16051868, EPI_ISL_16051873, EPI_ISL_16051885, EPI_ISL_16051886, EPI_ISL_16051887, EPI_ISL_16051888, EPI_ISL_16051890, EPI_ISL_16051891, EPI_ISL_16282709, EPI_ISL_16282710, EPI_ISL_16282711, EPI_ISL_16282715, EPI_ISL_16282716, EPI_ISL_16282717, EPI_ISL_16282718, EPI_ISL_16282719, EPI_ISL_16282732, EPI_ISL_16282733, EPI_ISL_16282734, EPI_ISL_16282739, EPI_ISL_16282740, EPI_ISL_16282745, EPI_ISL_16282758, EPI_ISL_16282759, EPI_ISL_16282760, EPI_ISL_16282761, EPI_ISL_16282762, EPI_ISL_16282763			
see above	National Influenza Center, National Institute of Hygiene and Epidemiology (NIHE)	National Influenza Center, National Institute of Hygiene and Epidemiology (NIHE)	Ung Thi Hong Trang, Hoang Vu Mai Phuong, Nguyen Huy Hoang, Nguyen Le Khanh Hang, Le Thi Quynh Mai
EPI_ISL_1647383, EPI_ISL_1647384, EPI_ISL_1647385, EPI_ISL_1647386, EPI_ISL_1647387, EPI_ISL_1647388, EPI_ISL_1647389, EPI_ISL_1647390, EPI_ISL_1647391, EPI_ISL_1647392, EPI_ISL_1647393, EPI_ISL_1647394, EPI_ISL_1647395, EPI_ISL_1647396, EPI_ISL_1647397, EPI_ISL_1647398, EPI_ISL_1647399, EPI_ISL_1647400, EPI_ISL_1647401, EPI_ISL_1647402, EPI_ISL_1647403, EPI_ISL_1647404, EPI_ISL_1647405, EPI_ISL_1647406, EPI_ISL_1647407, EPI_ISL_1647408, EPI_ISL_1647409, EPI_ISL_1647410, EPI_ISL_1647411, EPI_ISL_1647412, EPI_ISL_1647413, EPI_ISL_1647414, EPI_ISL_1647415, EPI_ISL_1647416, EPI_ISL_1647417, EPI_ISL_1647418, EPI_ISL_1647419, EPI_ISL_1647420, EPI_ISL_1647421, EPI_ISL_1647422			
see above	Respiratory Virus Unit, National Infection Service, Public Health England	National Infection Service, Public Health England	Zambon M, Talts T, Ellis J, Miah S, Platt S
EPI_ISL_16533868, EPI_ISL_16533869	Center for Virology, Medical University of Vienna	Center for Virology, Medical University of Vienna	Jeremy V. Camp, Monika Redlberger-Fritz
EPI_ISL_1653937	Royal Children's Hospital	WHO Influenza Centre for Reference and Research on Influenza	Angela Todd, Yi-Mo Deng, Annette Alafaci, Naomi Komadina
EPI_ISL_1653938, EPI_ISL_1653939	Royal Children's Hospital	WHO Influenza Centre for Reference and Research on Influenza	Jean Moselen, Yi-Mo Deng, Annette Alafaci, Naomi Komadina
EPI_ISL_1653940	Royal Children's Hospital	WHO Influenza Centre for Reference and Research on Influenza	Angela Todd, Yi-Mo Deng, Annette Alafaci, Naomi Komadina
EPI_ISL_1653941, EPI_ISL_1653942	Royal Children's Hospital	WHO Influenza Centre for Reference and Research on Influenza	Jean Moselen, Yi-Mo Deng, Annette Alafaci, Naomi Komadina
EPI_ISL_1653943	Royal Children's Hospital	WHO Influenza Centre for Reference and Research on Influenza	Angela Todd, Yi-Mo Deng, Annette Alafaci, Naomi Komadina
EPI_ISL_1653944	Royal Children's Hospital	WHO Influenza Centre for Reference and Research on Influenza	Jean Moselen, Yi-Mo Deng, Annette Alafaci, Naomi Komadina
EPI_ISL_1653945	Royal Children's Hospital	WHO Influenza Centre for Reference and Research on Influenza	Angela Todd, Yi-Mo Deng, Annette Alafaci, Naomi Komadina
EPI_ISL_1653946	Royal Children's Hospital	WHO Influenza Centre for Reference and Research on Influenza	Jean Moselen, Yi-Mo Deng, Annette Alafaci, Naomi Komadina
EPI_ISL_1653947	Royal Children's Hospital	WHO Influenza Centre for Reference and Research on Influenza	Angela Todd, Yi-Mo Deng, Annette Alafaci, Naomi Komadina
EPI_ISL_1653948	Royal Children's Hospital	WHO Influenza Centre for Reference and Research on Influenza	Jean Moselen, Yi-Mo Deng, Annette Alafaci, Naomi Komadina
EPI_ISL_1653949, EPI_ISL_1653950, EPI_ISL_1653951, EPI_ISL_1653952, EPI_ISL_1653953, EPI_ISL_1653954, EPI_ISL_1653955, EPI_ISL_1653956, EPI_ISL_1653957, EPI_ISL_1653958, EPI_ISL_1653959, EPI_ISL_1653960, EPI_ISL_1653961, EPI_ISL_1653962, EPI_ISL_1653963, EPI_ISL_1653964, EPI_ISL_1653965, EPI_ISL_1653966, EPI_ISL_1653967, EPI_ISL_1653968, EPI_ISL_1653969, EPI_ISL_1653970, EPI_ISL_1653971, EPI_ISL_1653972, EPI_ISL_1653973, EPI_ISL_1653974, EPI_ISL_1653975, EPI_ISL_1653976, EPI_ISL_1653977, EPI_ISL_1653978, EPI_ISL_1653979, EPI_ISL_1653980, EPI_ISL_1653981, EPI_ISL_1653982, EPI_ISL_1653983, EPI_ISL_1653984, EPI_ISL_1653985, EPI_ISL_1653986, EPI_ISL_1653987, EPI_ISL_1653988, EPI_ISL_1653989, EPI_ISL_1653990, EPI_ISL_1653991, EPI_ISL_1653992			
see above	Royal Children's Hospital	WHO Influenza Centre for Reference and Research on Influenza	Angela Todd, Yi-Mo Deng, Annette Alafaci, Naomi Komadina
EPI_ISL_16681174	Children's Hospital of Fudan University	Children's Hospital of Fudan University	Jia,R.
EPI_ISL_16681175	Kenya Medical Research Institute-Wellcome Trust	Kenya Medical Research Institute-Wellcome Trust	Lambisia,A.W., Makori,T.O., Mutunga,M., Lewa,C., Githinji,G., Agoti,C.N. and Nokes,J.D.
EPI_ISL_16681180	Children's Hospital of Fudan University	Children's Hospital of Fudan University	Jia,R.
EPI_ISL_16681181	Kenya Medical Research Institute-Wellcome Trust	Kenya Medical Research Institute-Wellcome Trust	Lambisia,A.W., Makori,T.O., Mutunga,M., Lewa,C., Githinji,G., Agoti,C.N. and Nokes,J.D.
EPI_ISL_16681185, EPI_ISL_16681190	Children's Hospital of Fudan University	Children's Hospital of Fudan University	Jia,R.
EPI_ISL_16681191	Kenya Medical Research Institute-Wellcome Trust	Kenya Medical Research Institute-Wellcome Trust	Lambisia,A.W., Makori,T.O., Mutunga,M., Lewa,C., Githinji,G., Agoti,C.N. and Nokes,J.D.
EPI_ISL_16681193	King Saud University	King Saud University	Alzayed,R.M., Aziz,I.M., Dudin,G.A., Alsaleh,A.N., Ahmed,A.A. and Almajhdi,F.N.
EPI_ISL_16681197, EPI_ISL_16681212	Children's Hospital of Fudan University	Children's Hospital of Fudan University	Jia,R.
EPI_ISL_16681213	Kenya Medical Research Institute-Wellcome Trust	Kenya Medical Research Institute-Wellcome Trust	Lambisia,A.W., Makori,T.O., Mutunga,M., Lewa,C., Githinji,G., Agoti,C.N. and Nokes,J.D.
EPI_ISL_16681219, EPI_ISL_16681230	Children's Hospital of Fudan University	Children's Hospital of Fudan University	Jia,R.
EPI_ISL_16681232	Kenya Medical Research Institute-Wellcome Trust	Kenya Medical Research Institute-Wellcome Trust	Lambisia,A.W., Makori,T.O., Mutunga,M., Lewa,C., Githinji,G., Agoti,C.N. and Nokes,J.D.
EPI_ISL_16681235	Children's Hospital of Fudan University	Children's Hospital of Fudan University	Jia,R.
EPI_ISL_16681236	Kenya Medical Research Institute-Wellcome Trust	Kenya Medical Research Institute-Wellcome Trust	Lambisia,A.W., Makori,T.O., Mutunga,M., Lewa,C., Githinji,G., Agoti,C.N. and Nokes,J.D.
EPI_ISL_16681238	King Saud University	King Saud University	Alzayed,R.M., Aziz,I.M., Dudin,G.A., Alsaleh,A.N., Ahmed,A.A. and Almajhdi,F.N.
EPI_ISL_16681241	Children's Hospital of Fudan University	Children's Hospital of Fudan University	Jia,R.
EPI_ISL_16681244	Kenya Medical Research Institute-Wellcome Trust	Kenya Medical Research Institute-Wellcome Trust	Lambisia,A.W., Makori,T.O., Mutunga,M., Lewa,C., Githinji,G., Agoti,C.N. and Nokes,J.D.
EPI_ISL_16681250	Children's Hospital of Fudan University	Children's Hospital of Fudan University	Jia,R.
EPI_ISL_16681254, EPI_ISL_16681262	Kenya Medical Research Institute-Wellcome Trust	Kenya Medical Research Institute-Wellcome Trust	Lambisia,A.W., Makori,T.O., Mutunga,M., Lewa,C., Githinji,G., Agoti,C.N. and Nokes,J.D.
EPI_ISL_16681263, EPI_ISL_16681268, EPI_ISL_16681275, EPI_ISL_16681280	Children's Hospital of Fudan University	Children's Hospital of Fudan University	Jia,R.
EPI_ISL_16681281	Kenya Medical Research Institute-Wellcome Trust	Kenya Medical Research Institute-Wellcome Trust	Lambisia,A.W., Makori,T.O., Mutunga,M., Lewa,C., Githinji,G., Agoti,C.N. and Nokes,J.D.
EPI_ISL_16681288	Children's Hospital of Fudan University	Children's Hospital of Fudan University	Jia,R.
EPI_ISL_16681293	Kenya Medical Research Institute-Wellcome Trust	Kenya Medical Research Institute-Wellcome Trust	Lambisia,A.W., Makori,T.O., Mutunga,M., Lewa,C., Githinji,G., Agoti,C.N. and Nokes,J.D.
EPI_ISL_16681321	King Saud University	King Saud University	Alzayed,R.M., Aziz,I.M., Dudin,G.A., Alsaleh,A.N., Ahmed,A.A. and Almajhdi,F.N.
EPI_ISL_16681354	Children's Hospital of Fudan University	Children's Hospital of Fudan University	Jia,R.
EPI_ISL_16681355	Kenya Medical Research Institute-Wellcome Trust	Kenya Medical Research Institute-Wellcome Trust	Lambisia,A.W., Makori,T.O., Mutunga,M., Lewa,C., Githinji,G., Agoti,C.N. and Nokes,J.D.
EPI_ISL_16681358	Children's Hospital of Fudan University	Children's Hospital of Fudan University	Jia,R.
EPI_ISL_16681371	Kenya Medical Research Institute-Wellcome Trust	Kenya Medical Research Institute-Wellcome Trust	Lambisia,A.W., Makori,T.O., Mutunga,M., Lewa,C., Githinji,G., Agoti,C.N. and Nokes,J.D.
EPI_ISL_16681372, EPI_ISL_16681374	Children's Hospital of Fudan University	Children's Hospital of Fudan University	Jia,R.
EPI_ISL_16681375	Kenya Medical Research Institute-Wellcome Trust	Kenya Medical Research Institute-Wellcome Trust	Lambisia,A.W., Makori,T.O., Mutunga,M., Lewa,C., Githinji,G., Agoti,C.N. and Nokes,J.D.
EPI_ISL_16681377	King Saud University	King Saud University	Alzayed,R.M., Aziz,I.M., Dudin,G.A., Alsaleh,A.N., Ahmed,A.A. and Almajhdi,F.N.
EPI_ISL_16681380	Children's Hospital of Fudan University	Children's Hospital of Fudan University	Jia,R.
EPI_ISL_16681382, EPI_ISL_16681385	Kenya Medical Research Institute-Wellcome Trust	Kenya Medical Research Institute-Wellcome Trust	Lambisia,A.W., Makori,T.O., Mutunga,M., Lewa,C., Githinji,G., Agoti,C.N. and Nokes,J.D.
EPI_ISL_16681387	Children's Hospital of Fudan University	Children's Hospital of Fudan University	Jia,R.

EPI_ISL_16681388	King Saud University	King Saud University	Alzayed,R.M., Aziz,I.M., Dudin,G.A., Alsaleh,A.N., Ahmed,A.A., Hussain,T. and Almajhdi,F.N.
EPI_ISL_16681390	Children's Hospital of Fudan University	Children's Hospital of Fudan University	Jia,R.
EPI_ISL_16681391	Kenya Medical Research Institute-Wellcome Trust	Kenya Medical Research Institute-Wellcome Trust	Lambisia,A.W., Makori,T.O., Mutunga,M., Lewa,C., Githinji,G., Agoti,C.N. and Nokes,J.D.
EPI_ISL_16681395	Children's Hospital of Fudan University	Children's Hospital of Fudan University	Jia,R.
EPI_ISL_16681396	Kenya Medical Research Institute-Wellcome Trust	Kenya Medical Research Institute-Wellcome Trust	Lambisia,A.W., Makori,T.O., Mutunga,M., Lewa,C., Githinji,G., Agoti,C.N. and Nokes,J.D.
EPI_ISL_16681400	Children's Hospital of Fudan University	Children's Hospital of Fudan University	Jia,R.
EPI_ISL_16681401	Kenya Medical Research Institute-Wellcome Trust	Kenya Medical Research Institute-Wellcome Trust	Lambisia,A.W., Makori,T.O., Mutunga,M., Lewa,C., Githinji,G., Agoti,C.N. and Nokes,J.D.
EPI_ISL_16681405	Children's Hospital of Fudan University	Children's Hospital of Fudan University	Jia,R.
EPI_ISL_16681407, EPI_ISL_16681409	Kenya Medical Research Institute-Wellcome Trust	Kenya Medical Research Institute-Wellcome Trust	Lambisia,A.W., Makori,T.O., Mutunga,M., Lewa,C., Githinji,G., Agoti,C.N. and Nokes,J.D.
EPI_ISL_16708970, EPI_ISL_16708971, EPI_ISL_16708972, EPI_ISL_16708975, EPI_ISL_16708976, EPI_ISL_16708977, EPI_ISL_16708980, EPI_ISL_16708981, EPI_ISL_16708982, EPI_ISL_16708984, EPI_ISL_16708985, EPI_ISL_16708986, EPI_ISL_16708987, EPI_ISL_16708989			
see above	University Hospitals of Leicester NHS Trust Pathology Services / Leicester Royal Infirmary	Reference Microbiology Services / UK Health Security Agency	Williams T. Zambon M. Talts T. Mosscrop L. Holmes C. Miah S
EPI_ISL_16714474, EPI_ISL_16714475, EPI_ISL_16714476, EPI_ISL_16714477, EPI_ISL_16714478, EPI_ISL_16714479, EPI_ISL_16714480, EPI_ISL_16714481, EPI_ISL_16714482, EPI_ISL_16714483, EPI_ISL_16714484, EPI_ISL_16714485, EPI_ISL_16714486, EPI_ISL_16714487, EPI_ISL_16714488, EPI_ISL_16714489, EPI_ISL_16714490, EPI_ISL_16714491, EPI_ISL_16714492, EPI_ISL_16714493, EPI_ISL_16714601, EPI_ISL_16714606, EPI_ISL_16714611			
see above	Respiratory Virus Unit / Reference Microbiology Services / UK Health Security Agency	Reference Microbiology Services / UK Health Security Agency	Zambon M. Talts T. Mosscrop L. Miah S
EPI_ISL_16959495, EPI_ISL_16959500, EPI_ISL_16959507, EPI_ISL_16959514, EPI_ISL_16959522	ESR - Wallaceville	Institute of Environmental Science and Research	Klarysse Berquist, Lauren Jelley, Una Ren, Meaghan O'Neill
EPI_ISL_16959530, EPI_ISL_16959531, EPI_ISL_16959532, EPI_ISL_16959533, EPI_ISL_16959534, EPI_ISL_16959535, EPI_ISL_16959536, EPI_ISL_16959537, EPI_ISL_16959538, EPI_ISL_16959539, EPI_ISL_16959540, EPI_ISL_16959541, EPI_ISL_16959542, EPI_ISL_16959543, EPI_ISL_16959545, EPI_ISL_16959546, EPI_ISL_16959547, EPI_ISL_16959548, EPI_ISL_16959549, EPI_ISL_16959550, EPI_ISL_16959563, EPI_ISL_16959564, EPI_ISL_16959567, EPI_ISL_16959568, EPI_ISL_16959569, EPI_ISL_16959571, EPI_ISL_16959572, EPI_ISL_16959574, EPI_ISL_16959575, EPI_ISL_16959576, EPI_ISL_16959577, EPI_ISL_16959579, EPI_ISL_16959580, EPI_ISL_16959581, EPI_ISL_16959582, EPI_ISL_16959583, EPI_ISL_16959584, EPI_ISL_16959585, EPI_ISL_16959587, EPI_ISL_16959588, EPI_ISL_16959591, EPI_ISL_16959594			
see above	Middlemore Hospital	Institute of Environmental Science and Research	Klarysse Berquist, Lauren Jelley, Una Ren, Meaghan O'Neill
EPI_ISL_16959595, EPI_ISL_16959597	PathLab Bay of Plenty	Institute of Environmental Science and Research	Klarysse Berquist, Lauren Jelley, Una Ren, Meaghan O'Neill
EPI_ISL_16959598, EPI_ISL_16959600, EPI_ISL_16959603, EPI_ISL_16959604, EPI_ISL_16959605, EPI_ISL_16959606, EPI_ISL_16959607, EPI_ISL_16959608, EPI_ISL_16959609, EPI_ISL_16959612, EPI_ISL_16959613, EPI_ISL_16959615, EPI_ISL_16959616, EPI_ISL_16959617, EPI_ISL_16959618, EPI_ISL_16959619, EPI_ISL_16959620, EPI_ISL_16959622, EPI_ISL_16959625, EPI_ISL_16959626, EPI_ISL_16959627, EPI_ISL_16959628, EPI_ISL_16959629, EPI_ISL_16959630, EPI_ISL_16959632, EPI_ISL_16959634, EPI_ISL_16959635			
see above	Middlemore Hospital	Institute of Environmental Science and Research	Klarysse Berquist, Lauren Jelley, Una Ren, Meaghan O'Neill
EPI_ISL_16959636, EPI_ISL_16959637, EPI_ISL_16959638, EPI_ISL_16959639	MedLab South Nelson	Institute of Environmental Science and Research	Klarysse Berquist, Lauren Jelley, Una Ren, Meaghan O'Neill
EPI_ISL_16959641, EPI_ISL_16959642, EPI_ISL_16959643, EPI_ISL_16959644, EPI_ISL_16959645, EPI_ISL_16959646, EPI_ISL_16959647, EPI_ISL_16959648, EPI_ISL_16959649, EPI_ISL_16959651, EPI_ISL_16959652, EPI_ISL_16959653, EPI_ISL_16959655, EPI_ISL_16959656, EPI_ISL_16959659, EPI_ISL_16959660, EPI_ISL_16959661, EPI_ISL_16959663, EPI_ISL_16959664, EPI_ISL_16959665, EPI_ISL_16959666, EPI_ISL_16959667, EPI_ISL_16959668, EPI_ISL_16959670, EPI_ISL_16959671, EPI_ISL_16959673, EPI_ISL_16959674, EPI_ISL_16959676, EPI_ISL_16959677, EPI_ISL_16959679, EPI_ISL_16959680, EPI_ISL_16959681, EPI_ISL_16959682, EPI_ISL_16959683, EPI_ISL_16959685, EPI_ISL_16959686, EPI_ISL_16959688, EPI_ISL_16959690, EPI_ISL_16959691, EPI_ISL_16959692, EPI_ISL_16959693, EPI_ISL_16959694, EPI_ISL_16959695, EPI_ISL_16959697, EPI_ISL_16959698, EPI_ISL_16959699, EPI_ISL_16959700, EPI_ISL_16959702, EPI_ISL_16959706, EPI_ISL_16959708, EPI_ISL_16959709, EPI_ISL_16959710, EPI_ISL_16959711, EPI_ISL_16959712, EPI_ISL_16959713, EPI_ISL_16959714, EPI_ISL_16959715, EPI_ISL_16959718, EPI_ISL_16959719, EPI_ISL_16959720, EPI_ISL_16959721, EPI_ISL_16959722, EPI_ISL_16959724, EPI_ISL_16959725, EPI_ISL_16959727, EPI_ISL_16959728, EPI_ISL_16959730, EPI_ISL_16959731, EPI_ISL_16959732, EPI_ISL_16959733, EPI_ISL_16959734, EPI_ISL_16959735			
see above	Middlemore Hospital	Institute of Environmental Science and Research	Klarysse Berquist, Lauren Jelley, Una Ren, Meaghan O'Neill
EPI_ISL_16959736, EPI_ISL_16959737, EPI_ISL_16959741, EPI_ISL_16959742	Southern Community Lab - Dunedin	Institute of Environmental Science and Research	Klarysse Berquist, Lauren Jelley, Una Ren, Meaghan O'Neill
EPI_ISL_16959743, EPI_ISL_16959744, EPI_ISL_16959745, EPI_ISL_16959746, EPI_ISL_16959747, EPI_ISL_16959748, EPI_ISL_16959749, EPI_ISL_16959750, EPI_ISL_16959751, EPI_ISL_16959754, EPI_ISL_16959757, EPI_ISL_16959758, EPI_ISL_16959759, EPI_ISL_16959762, EPI_ISL_16959764, EPI_ISL_16959765, EPI_ISL_16959766, EPI_ISL_16959767, EPI_ISL_16959768, EPI_ISL_16959769, EPI_ISL_16959770, EPI_ISL_16959772, EPI_ISL_16959773, EPI_ISL_16959774, EPI_ISL_16959775, EPI_ISL_16959776, EPI_ISL_16959777, EPI_ISL_16959778, EPI_ISL_16959779, EPI_ISL_16959780, EPI_ISL_16959781, EPI_ISL_16959782			
see above	Middlemore Hospital	Institute of Environmental Science and Research	Klarysse Berquist, Lauren Jelley, Una Ren, Meaghan O'Neill
EPI_ISL_16959783, EPI_ISL_16959788, EPI_ISL_16959806	Wellington SCL	Institute of Environmental Science and Research	Klarysse Berquist, Lauren Jelley, Una Ren, Meaghan O'Neill
EPI_ISL_16959818, EPI_ISL_16959819, EPI_ISL_16959820, EPI_ISL_16959821, EPI_ISL_16959822, EPI_ISL_16959829, EPI_ISL_16959831, EPI_ISL_16959832, EPI_ISL_16959833, EPI_ISL_16959834, EPI_ISL_16959835, EPI_ISL_16959836			
see above	Middlemore Hospital	Institute of Environmental Science and Research	Klarysse Berquist, Lauren Jelley, Una Ren, Meaghan O'Neill
EPI_ISL_16959846, EPI_ISL_16959847, EPI_ISL_16959849, EPI_ISL_16959851	Canterbury Health Laboratory	Institute of Environmental Science and Research	Klarysse Berquist, Lauren Jelley, Una Ren, Meaghan O'Neill
EPI_ISL_16959852, EPI_ISL_16959856	Labplus	Institute of Environmental Science and Research	Klarysse Berquist, Lauren Jelley, Una Ren, Meaghan O'Neill
EPI_ISL_16959858, EPI_ISL_16959860, EPI_ISL_16959861, EPI_ISL_16959862, EPI_ISL_16959863, EPI_ISL_16959866	Southern Community Lab - Dunedin	Institute of Environmental Science and Research	Klarysse Berquist, Lauren Jelley, Una Ren, Meaghan O'Neill
EPI_ISL_16959871, EPI_ISL_16959872, EPI_ISL_16959873	Middlemore Hospital	Institute of Environmental Science and Research	Klarysse Berquist, Lauren Jelley, Una Ren, Meaghan O'Neill
EPI_ISL_16959879, EPI_ISL_16959881, EPI_ISL_16959882, EPI_ISL_16959883, EPI_ISL_16959885, EPI_ISL_16959886, EPI_ISL_16959887, EPI_ISL_16959889	Labplus	Institute of Environmental Science and Research	Klarysse Berquist, Lauren Jelley, Una Ren, Meaghan O'Neill
EPI_ISL_16959905	Wellington SCL	Institute of Environmental Science and Research	Klarysse Berquist, Lauren Jelley, Una Ren, Meaghan O'Neill
EPI_ISL_16959918, EPI_ISL_16959919, EPI_ISL_16959922, EPI_ISL_16959923, EPI_ISL_16959924, EPI_ISL_16959926, EPI_ISL_16959927	PathLab Bay of Plenty	Institute of Environmental Science and Research	Klarysse Berquist, Lauren Jelley, Una Ren, Meaghan O'Neill
EPI_ISL_16959928	Canterbury Health Laboratory	Institute of Environmental Science and Research	Klarysse Berquist, Lauren Jelley, Una Ren, Meaghan O'Neill
EPI_ISL_16959942, EPI_ISL_16959943, EPI_ISL_16959945	Middlemore Hospital	Institute of Environmental Science and Research	Klarysse Berquist, Lauren Jelley, Una Ren, Meaghan O'Neill
EPI_ISL_16959946, EPI_ISL_16959948, EPI_ISL_16959949, EPI_ISL_16959951, EPI_ISL_16959953, EPI_ISL_16959954, EPI_ISL_16959957	LabPlus	Institute of Environmental Science and Research	Klarysse Berquist, Lauren Jelley, Una Ren, Meaghan O'Neill
EPI_ISL_16959959, EPI_ISL_16959960, EPI_ISL_16959961, EPI_ISL_16959962, EPI_ISL_16959963, EPI_ISL_16959964, EPI_ISL_16959965, EPI_ISL_16959966, EPI_ISL_16959969, EPI_ISL_16959970, EPI_ISL_16959971, EPI_ISL_16959972, EPI_ISL_16959973, EPI_ISL_16959975, EPI_ISL_16959976, EPI_ISL_16959977, EPI_ISL_16959978, EPI_ISL_16959979, EPI_ISL_16959980, EPI_ISL_16959981, EPI_ISL_16959982, EPI_ISL_16959983, EPI_ISL_16959985			
see above	MedLab South Nelson	Institute of Environmental Science and Research	Klarysse Berquist, Lauren Jelley, Una Ren, Meaghan O'Neill
EPI_ISL_16959995, EPI_ISL_16959997	Southern Community Lab - Dunedin	Institute of Environmental Science and Research	Klarysse Berquist, Lauren Jelley, Una Ren, Meaghan O'Neill
EPI_ISL_16959999, EPI_ISL_16960002, EPI_ISL_16960003, EPI_ISL_16960005, EPI_ISL_16960008	Waikato Hospital	Institute of Environmental Science and Research	Klarysse Berquist, Lauren Jelley, Una Ren, Meaghan O'Neill
EPI_ISL_16960010, EPI_ISL_16960011, EPI_ISL_16960012, EPI_ISL_16960013	Middlemore Hospital	Institute of Environmental Science and Research	Klarysse Berquist, Lauren Jelley, Una Ren, Meaghan O'Neill
EPI_ISL_16960021, EPI_ISL_16960023, EPI_ISL_16960024, EPI_ISL_16960025, EPI_ISL_16960026, EPI_ISL_16960029, EPI_ISL_16960030, EPI_ISL_16960032, EPI_ISL_16960034, EPI_ISL_16960036, EPI_ISL_16960037, EPI_ISL_16960038, EPI_ISL_16960039, EPI_ISL_16960042, EPI_ISL_16960045, EPI_ISL_16960046, EPI_ISL_16960047, EPI_ISL_16960048, EPI_ISL_16960049, EPI_ISL_16960050, EPI_ISL_16960052, EPI_ISL_16960054, EPI_ISL_16960055, EPI_ISL_16960056, EPI_ISL_16960057, EPI_ISL_16960058, EPI_ISL_16960059, EPI_ISL_16960061, EPI_ISL_16960062, EPI_ISL_16960064, EPI_ISL_16960065, EPI_ISL_16960066, EPI_ISL_16960068, EPI_ISL_16960070, EPI_ISL_16960071, EPI_ISL_16960072, EPI_ISL_16960073, EPI_ISL_16960074, EPI_ISL_16960076, EPI_ISL_16960077, EPI_ISL_16960081, EPI_ISL_16960082, EPI_ISL_16960084, EPI_ISL_16960085, EPI_ISL_16960086, EPI_ISL_16960088, EPI_ISL_16960089			
see above	LabPlus	Institute of Environmental Science and Research	Klarysse Berquist, Lauren Jelley, Una Ren, Meaghan O'Neill
EPI_ISL_16960099, EPI_ISL_16960152	Middlemore Hospital	Institute of Environmental Science and Research	Klarysse Berquist, Lauren Jelley, Una Ren, Meaghan O'Neill
EPI_ISL_17089183, EPI_ISL_17089184	University of Washington - Laboratory Medicine	University of Washington - Laboratory Medicine	Sereewit,J., Xie,H., Roychoudhury,P. and Greninger,A.L.
EPI_ISL_17168080, EPI_ISL_17168081, EPI_ISL_17168082, EPI_ISL_17168083, EPI_ISL_17168084, EPI_ISL_17168085, EPI_ISL_17168089, EPI_ISL_17168090, EPI_ISL_17168091, EPI_ISL_17168092, EPI_ISL_17168093, EPI_ISL_17168095, EPI_ISL_17168118, EPI_ISL_17168119, EPI_ISL_17168120, EPI_ISL_17168144, EPI_ISL_17168145, EPI_ISL_17168146, EPI_ISL_17168147, EPI_ISL_17168148, EPI_ISL_17168149, EPI_ISL_17168150, EPI_ISL_17168153, EPI_ISL_17168155, EPI_ISL_17168156, EPI_ISL_17168157, EPI_ISL_17168158, EPI_ISL_17168159, EPI_ISL_17168160, EPI_ISL_17168161, EPI_ISL_17168162, EPI_ISL_17168166, EPI_ISL_17168167, EPI_ISL_17168218, EPI_ISL_17168219, EPI_ISL_17168220, EPI_ISL_17168221, EPI_ISL_17168222, EPI_ISL_17168223, EPI_ISL_17168224, EPI_ISL_17168225, EPI_ISL_17168231, EPI_ISL_17168246			
see above	National Institute for Viral Disease Control and Prevention	National Institute for Viral Disease Control and Prevention	Song,J., Zhu,Z., Song,J., Mao,N., Cui,A., Xu,W. and Zhang,Y.
EPI_ISL_17215508, EPI_ISL_17215514, EPI_ISL_17215523, EPI_ISL_17215538, EPI_ISL_17215548, EPI_ISL_17215549	State Key Laboratory of Respiratory Disease, Guangzhou Institute of Respiratory Health	State Key Laboratory of Respiratory Disease, Guangzhou Institute of Respiratory Health	Zhao,J., Wang,Y., Wei,P. and Xiao,J.
EPI_ISL_17215851, EPI_ISL_17215852, EPI_ISL_17215875, EPI_ISL_17215891, EPI_ISL_17215892, EPI_ISL_17215897, EPI_ISL_17215902, EPI_ISL_17215908, EPI_ISL_17215909, EPI_ISL_17215914, EPI_ISL_17215920, EPI_ISL_17215927, EPI_ISL_17215934, EPI_ISL_17215938, EPI_ISL_17215943, EPI_ISL_17215954, EPI_ISL_17215957, EPI_ISL_17215961, EPI_ISL_17215967, EPI_ISL_17215972, EPI_ISL_17215974, EPI_ISL_17215977, EPI_ISL_17215983, EPI_ISL_17215986, EPI_ISL_17215990, EPI_ISL_17215992, EPI_ISL_17216017, EPI_ISL_17216024, EPI_ISL_17216025, EPI_ISL_17216044, EPI_ISL_17216050, EPI_ISL_17216058, EPI_ISL_17216070, EPI_ISL_17216072, EPI_ISL_17216073, EPI_ISL_17216074, EPI_ISL_17216076, EPI_ISL_17216077, EPI_ISL_17216085, EPI_ISL_17216089, EPI_ISL_17216092, EPI_ISL_17216103, EPI_ISL_17216122, EPI_ISL_17216128, EPI_ISL_17216142, EPI_ISL_17216143, EPI_ISL_17216153, EPI_ISL_17216168, EPI_ISL_17216183, EPI_ISL_17216199, EPI_ISL_17216209, EPI_ISL_17216210, EPI_ISL_17216213, EPI_ISL_17216216, EPI_ISL_17216217, EPI_ISL_17216220, EPI_ISL_17216221, EPI_ISL_17216226, EPI_ISL_17216227, EPI_ISL_17216229, EPI_ISL_17216236, EPI_ISL_17216243, EPI_ISL_17216245, EPI_ISL_17216257, EPI_ISL_17216269, EPI_ISL_17216278, EPI_ISL_17216280, EPI_ISL_17216283, EPI_ISL_17216284, EPI_ISL_17216286, EPI_ISL_17216288, EPI_ISL_17216292, EPI_ISL_17216293, EPI_ISL_17216294, EPI_ISL_17216296, EPI_ISL_17216298, EPI_ISL_17216300, EPI_ISL_17216304, EPI_ISL_17216307, EPI_ISL_17216309, EPI_ISL_17216310, EPI_ISL_17216314, EPI_ISL_17216316, EPI_ISL_17216320, EPI_ISL_17216321, EPI_ISL_17216323, EPI_ISL_17216325, EPI_ISL_17216326, EPI_ISL_17216341, EPI_ISL_17216342, EPI_ISL_17216347, EPI_ISL_17216349, EPI_ISL_17216352, EPI_ISL_17216353, EPI_ISL_17216356, EPI_ISL_17216357, EPI_ISL_17216363, EPI_ISL_17216365, EPI_ISL_17216386, EPI_ISL_17216402, EPI_ISL_17216408, EPI_ISL_17216413, EPI_ISL_17216418, EPI_ISL_17216419, EPI_ISL_17216422, EPI_ISL_17216426, EPI_ISL_17216429, EPI_ISL_17216430, EPI_ISL_17216431, EPI_ISL_17216435, EPI_ISL_17216439, EPI_ISL_17216440, EPI_ISL_17216446, EPI_ISL_17216469, EPI_ISL_17216473, EPI_ISL_17216478, EPI_ISL_17216508, EPI_ISL_17216509, EPI_ISL_17216535, EPI_ISL_17216536, EPI_ISL_17216541, EPI_ISL_17216587, EPI_ISL_17216598, EPI_ISL_17216602, EPI_ISL_17216619, EPI_ISL_17216627, EPI_ISL_17216633, EPI_ISL_17216636, EPI_ISL_17216650, EPI_ISL_17216651, EPI_ISL_17216654, EPI_ISL_17216655, EPI_ISL_17216663, EPI_ISL_17216668, EPI_ISL_17216670, EPI_ISL_17216671, EPI_ISL_17216684, EPI_ISL_17216685, EPI_ISL_17216691, EPI_ISL_17216708, EPI_ISL_17216715, EPI_ISL_17216721, EPI_ISL_17216735, EPI_ISL_17216741, EPI_ISL_17216750, EPI_ISL_17216755, EPI_ISL_17216759, EPI_ISL_17216761, EPI_ISL_17216772, EPI_ISL_17216773, EPI_ISL_17216778, EPI_ISL_17216779, EPI_ISL_17216787, EPI_ISL_17216790, EPI_ISL_17216791, EPI_ISL_17216803, EPI_ISL_17216804, EPI_ISL_17216806, EPI_ISL_17216812, EPI_ISL_17216823, EPI_ISL_17216825, EPI_ISL_17216835, EPI_ISL_17216843, EPI_ISL_17216850, EPI_ISL_17216852, EPI_ISL_17216855, EPI_ISL_17216875, EPI_ISL_17216888, EPI_ISL_17216889, EPI_ISL_17216893, EPI_ISL_17216895, EPI_ISL_17216909, EPI_ISL_17216925, EPI_ISL_17216926, EPI_ISL_17216933, EPI_ISL_17216941, EPI_ISL_17216951, EPI_ISL_17216953, EPI_ISL_17216961, EPI_ISL_17216973, EPI_ISL_17216984, EPI_ISL_17216999, EPI_ISL_17217010, EPI_ISL_17217019, EPI_ISL_17217025, EPI_ISL_17217035, EPI_ISL_17217039, EPI_ISL_17217043, EPI_ISL_17217050, EPI_ISL_17217054, EPI_ISL_17217055, EPI_ISL_17217082, EPI_ISL_17217085, EPI_ISL_17217090, EPI_ISL_17217093, EPI_ISL_17217095, EPI_ISL_17217096, EPI_ISL_17217097, EPI_ISL_17217099, EPI_ISL_17217102, EPI_ISL_17217103, EPI_ISL_17217107, EPI_ISL_17217108, EPI_ISL_17217113, EPI_ISL_17217120, EPI_ISL_17217133, EPI_ISL_17217135, EPI_ISL_17217138, EPI_ISL_17217144, EPI_ISL_17217147, EPI_ISL_17217148, EPI_ISL_17217155, EPI_ISL_17217165, EPI_ISL_17217172, EPI_ISL_17217173, EPI_ISL_17217181, EPI_ISL_17217185, EPI_ISL_17217211, EPI_ISL_17217216, EPI_ISL_17217251, EPI_ISL_17217252, EPI_ISL_17217258, EPI_ISL_17217259, EPI_ISL_17217261, EPI_ISL_17217269, EPI_ISL_17217274, EPI_ISL_17217275, EPI_ISL_17217278, EPI_ISL_17217279, EPI_ISL_17217283, EPI_ISL_17217285, EPI_ISL_17217288, EPI_ISL_17217292, EPI_ISL_17217294, EPI_ISL_17217296, EPI_ISL_17217298, EPI_ISL_17217301, EPI_ISL_17217303, EPI_ISL_17217304, EPI_ISL_17217308, EPI_ISL_17217309, EPI_ISL_17217310, EPI_ISL_17217311, EPI_ISL_17217312, EPI_ISL_17217324, EPI_ISL_17217325, EPI_ISL_17217335, EPI_ISL_17217336, EPI_ISL_17217337, EPI_ISL_17217340, EPI_ISL_17217343, EPI_ISL_17217344, EPI_ISL_17217346, EPI_ISL_17217350, EPI_ISL_17217351, EPI_ISL_17217355, EPI_ISL_17217360, EPI_ISL_17217361, EPI_ISL_17217363, EPI_ISL_17217364, EPI_ISL_17217368, EPI_ISL_17217373, EPI_ISL_17217375, EPI_ISL_17217376, EPI_ISL_17217378, EPI_ISL_17217379, EPI_ISL_17217380, EPI_ISL_17217382, EPI_ISL_17217383, EPI_ISL_17217386, EPI_ISL_17217389, EPI_ISL_17217391, EPI_ISL_17217392, EPI_ISL_17217393, EPI_ISL_17217395, EPI_ISL_17217396, EPI_ISL_17217398, EPI_ISL_17217399, EPI_ISL_17217402, EPI_ISL_17217404, EPI_ISL_17217406, EPI_ISL_17217407, EPI_ISL_17217408, EPI_ISL_17217413, EPI_ISL_17217415, EPI_ISL_17217417, EPI_ISL_17217421, EPI_ISL_17217423, EPI_ISL_17217424, EPI_ISL_17217426, EPI_ISL_17217427, EPI_ISL_17217429, EPI_ISL_17217430, EPI_ISL_17217432, EPI_ISL_17217436, EPI_ISL_17217437, EPI_ISL_17217443, EPI_ISL_17217444, EPI_ISL_17217446, EPI_ISL_17217448,			

EPI_ISL_17217450, EPI_ISL_17217451, EPI_ISL_17217452, EPI_ISL_17217453, EPI_ISL_17217455, EPI_ISL_17217457, EPI_ISL_17217458, EPI_ISL_17217461, EPI_ISL_17217462, EPI_ISL_17217464, EPI_ISL_17217465, EPI_ISL_17217467, EPI_ISL_17217469, EPI_ISL_17217471, EPI_ISL_17217476, EPI_ISL_17217478, EPI_ISL_17217479, EPI_ISL_17217480, EPI_ISL_17217484, EPI_ISL_17217485, EPI_ISL_17217491, EPI_ISL_17217492, EPI_ISL_17217493, EPI_ISL_17217495, EPI_ISL_17217498, EPI_ISL_17217502, EPI_ISL_17217503, EPI_ISL_17217507, EPI_ISL_17217512, EPI_ISL_17217513, EPI_ISL_17217514, EPI_ISL_17217515, EPI_ISL_17217517, EPI_ISL_17217520, EPI_ISL_17217522, EPI_ISL_17217523, EPI_ISL_17217525, EPI_ISL_17217526, EPI_ISL_17217533, EPI_ISL_17217535, EPI_ISL_17217541, EPI_ISL_17217545, EPI_ISL_17217546, EPI_ISL_17217550, EPI_ISL_17217553, EPI_ISL_17217555, EPI_ISL_17217562, EPI_ISL_17217570, EPI_ISL_17217576, EPI_ISL_17217580, EPI_ISL_17217581, EPI_ISL_17217582, EPI_ISL_17217587, EPI_ISL_17217588, EPI_ISL_17217592, EPI_ISL_17217593, EPI_ISL_17217595, EPI_ISL_17217597, EPI_ISL_17217599, EPI_ISL_17217600, EPI_ISL_17217602, EPI_ISL_17217606, EPI_ISL_17217616, EPI_ISL_17217617, EPI_ISL_17217618, EPI_ISL_17217620, EPI_ISL_17217623, EPI_ISL_17217627, EPI_ISL_17217628, EPI_ISL_17217630, EPI_ISL_17217632, EPI_ISL_17217634, EPI_ISL_17217637, EPI_ISL_17217640, EPI_ISL_17217644, EPI_ISL_17217645, EPI_ISL_17217646, EPI_ISL_17217647, EPI_ISL_17217655, EPI_ISL_17217666, EPI_ISL_17217668, EPI_ISL_17217670, EPI_ISL_17217671, EPI_ISL_17217672, EPI_ISL_17217674, EPI_ISL_17217680, EPI_ISL_17217685, EPI_ISL_17217686, EPI_ISL_17217687, EPI_ISL_17217689, EPI_ISL_17217697, EPI_ISL_17217702, EPI_ISL_17217708, EPI_ISL_17217709, EPI_ISL_17217712, EPI_ISL_17217713, EPI_ISL_17217718, EPI_ISL_17217719, EPI_ISL_17217720, EPI_ISL_17217723, EPI_ISL_17217724, EPI_ISL_17217726, EPI_ISL_17217727, EPI_ISL_17217729, EPI_ISL_17217730, EPI_ISL_17217732, EPI_ISL_17217733, EPI_ISL_17217735, EPI_ISL_17217736, EPI_ISL_17217738, EPI_ISL_17217739, EPI_ISL_17217741, EPI_ISL_17217742, EPI_ISL_17217744, EPI_ISL_17217746, EPI_ISL_17217748, EPI_ISL_17217750, EPI_ISL_17217753, EPI_ISL_17217754, EPI_ISL_17217756, EPI_ISL_17217759, EPI_ISL_17217760, EPI_ISL_17217762, EPI_ISL_17217764, EPI_ISL_17217766, EPI_ISL_17217768, EPI_ISL_17217769, EPI_ISL_17217773, EPI_ISL_17217779, EPI_ISL_17217780, EPI_ISL_17217782, EPI_ISL_17217783, EPI_ISL_17217786, EPI_ISL_17217787, EPI_ISL_17217788, EPI_ISL_17217790, EPI_ISL_17217792, EPI_ISL_17217795, EPI_ISL_17217796, EPI_ISL_17217799, EPI_ISL_17217800, EPI_ISL_17217801, EPI_ISL_17217802, EPI_ISL_17217804, EPI_ISL_17217805, EPI_ISL_17217807, EPI_ISL_17217811, EPI_ISL_17217818, EPI_ISL_17217819, EPI_ISL_17217820, EPI_ISL_17217822, EPI_ISL_17217823, EPI_ISL_17217828, EPI_ISL_17217830, EPI_ISL_17217832, EPI_ISL_17217834, EPI_ISL_17217836, EPI_ISL_17217838, EPI_ISL_17217839, EPI_ISL_17217841, EPI_ISL_17217842, EPI_ISL_17217844, EPI_ISL_17217845, EPI_ISL_17217848, EPI_ISL_17217853, EPI_ISL_17217856, EPI_ISL_17217857, EPI_ISL_17217858, EPI_ISL_17217864, EPI_ISL_17217865, EPI_ISL_17217868, EPI_ISL_17217869, EPI_ISL_17217870, EPI_ISL_17217871, EPI_ISL_17217872, EPI_ISL_17217873, EPI_ISL_17217875, EPI_ISL_17217876, EPI_ISL_17217878, EPI_ISL_17217880, EPI_ISL_17217881, EPI_ISL_17217882, EPI_ISL_17217884, EPI_ISL_17217886, EPI_ISL_17217887, EPI_ISL_17217888, EPI_ISL_17217894, EPI_ISL_17217895, EPI_ISL_17217897, EPI_ISL_17217899, EPI_ISL_17217900, EPI_ISL_17217901, EPI_ISL_17217903, EPI_ISL_17217914, EPI_ISL_17217920, EPI_ISL_17217921, EPI_ISL_17217924, EPI_ISL_17217927, EPI_ISL_17217934, EPI_ISL_17217937, EPI_ISL_17217941, EPI_ISL_17217942, EPI_ISL_17217947, EPI_ISL_17217950, EPI_ISL_17217954, EPI_ISL_17217957, EPI_ISL_17217960, EPI_ISL_17217961, EPI_ISL_17217968, EPI_ISL_17217969, EPI_ISL_17217971, EPI_ISL_17217972, EPI_ISL_17217974, EPI_ISL_17217975, EPI_ISL_17217976, EPI_ISL_17217977, EPI_ISL_17217978, EPI_ISL_17217979, EPI_ISL_17217982, EPI_ISL_17217985, EPI_ISL_17217986, EPI_ISL_17217989, EPI_ISL_17217990, EPI_ISL_17217994, EPI_ISL_17217995, EPI_ISL_17217996, EPI_ISL_17217998, EPI_ISL_17217999, EPI_ISL_17218003, EPI_ISL_17218004, EPI_ISL_17218007, EPI_ISL_17218011, EPI_ISL_17218012, EPI_ISL_17218015, EPI_ISL_17218017, EPI_ISL_17218018, EPI_ISL_17218020, EPI_ISL_17218028, EPI_ISL_17218037, EPI_ISL_17218041, EPI_ISL_17218044, EPI_ISL_17218045, EPI_ISL_17218047, EPI_ISL_17218048, EPI_ISL_17218049, EPI_ISL_17218050, EPI_ISL_17218052, EPI_ISL_17218053, EPI_ISL_17218055, EPI_ISL_17218059, EPI_ISL_17218061, EPI_ISL_17218063, EPI_ISL_17218064, EPI_ISL_17218066, EPI_ISL_17218075, EPI_ISL_17218076, EPI_ISL_17218077, EPI_ISL_17218078, EPI_ISL_17218080, EPI_ISL_17218081, EPI_ISL_17218084, EPI_ISL_17218087, EPI_ISL_17218088, EPI_ISL_17218090, EPI_ISL_17218093, EPI_ISL_17218096, EPI_ISL_17218097, EPI_ISL_17218101, EPI_ISL_17218103, EPI_ISL_17218104, EPI_ISL_17218105, EPI_ISL_17218106, EPI_ISL_17218107, EPI_ISL_17218109, EPI_ISL_17218110, EPI_ISL_17218113, EPI_ISL_17218114, EPI_ISL_17218116, EPI_ISL_17218117, EPI_ISL_17218118, EPI_ISL_17218119, EPI_ISL_17218121, EPI_ISL_17218125, EPI_ISL_17218129, EPI_ISL_17218133, EPI_ISL_17218135, EPI_ISL_17218140, EPI_ISL_17218142, EPI_ISL_17218143, EPI_ISL_17218146, EPI_ISL_17218147, EPI_ISL_17218157, EPI_ISL_17218160, EPI_ISL_17218162, EPI_ISL_17218166, EPI_ISL_17218167, EPI_ISL_17218168, EPI_ISL_17218172, EPI_ISL_17218173, EPI_ISL_17218174, EPI_ISL_17218179, EPI_ISL_17218180, EPI_ISL_17218182, EPI_ISL_17218183, EPI_ISL_17218185, EPI_ISL_17218190, EPI_ISL_17218191, EPI_ISL_17218192, EPI_ISL_17218198, EPI_ISL_17218199, EPI_ISL_17218202, EPI_ISL_17218203, EPI_ISL_17218208, EPI_ISL_17218209, EPI_ISL_17218211, EPI_ISL_17218213, EPI_ISL_17218215, EPI_ISL_17218217, EPI_ISL_17218218, EPI_ISL_17218225, EPI_ISL_17218232, EPI_ISL_17218234, EPI_ISL_17218243, EPI_ISL_17218244, EPI_ISL_17218245, EPI_ISL_17218246, EPI_ISL_17218248, EPI_ISL_17218250, EPI_ISL_17218252, EPI_ISL_17218253, EPI_ISL_17218254, EPI_ISL_17218256, EPI_ISL_17218258, EPI_ISL_17218259, EPI_ISL_17218260, EPI_ISL_17218262, EPI_ISL_17218265, EPI_ISL_17218266, EPI_ISL_17218267, EPI_ISL_17218268, EPI_ISL_17218272, EPI_ISL_17218273, EPI_ISL_17218275, EPI_ISL_17218276, EPI_ISL_17218277, EPI_ISL_17218282, EPI_ISL_17218288, EPI_ISL_17218289, EPI_ISL_17218293, EPI_ISL_17218300, EPI_ISL_17218301, EPI_ISL_17218305, EPI_ISL_17218308, EPI_ISL_17218309, EPI_ISL_17218310, EPI_ISL_17218311, EPI_ISL_17218312, EPI_ISL_17218315, EPI_ISL_17218316, EPI_ISL_17218325, EPI_ISL_17218326, EPI_ISL_17218327, EPI_ISL_17218330, EPI_ISL_17218333, EPI_ISL_17218337, EPI_ISL_17218339, EPI_ISL_17218345, EPI_ISL_17218349, EPI_ISL_17218351, EPI_ISL_17218354, EPI_ISL_17218355, EPI_ISL_17218357, EPI_ISL_17218358, EPI_ISL_17218363, EPI_ISL_17218366, EPI_ISL_17218367, EPI_ISL_17218372, EPI_ISL_17218373, EPI_ISL_17218374, EPI_ISL_17218382, EPI_ISL_17218385, EPI_ISL_17218388, EPI_ISL_17218391, EPI_ISL_17218395, EPI_ISL_17218398, EPI_ISL_17218400, EPI_ISL_17218401, EPI_ISL_17218406, EPI_ISL_17218408, EPI_ISL_17218410, EPI_ISL_17218411, EPI_ISL_17218413, EPI_ISL_17218417, EPI_ISL_17218419, EPI_ISL_17218423, EPI_ISL_17218425, EPI_ISL_17218429, EPI_ISL_17218430, EPI_ISL_17218433, EPI_ISL_17218435, EPI_ISL_17218438, EPI_ISL_17218439, EPI_ISL_17218443, EPI_ISL_17218444, EPI_ISL_17218448, EPI_ISL_17218451, EPI_ISL_17218452, EPI_ISL_17218457, EPI_ISL_17218460, EPI_ISL_17218461, EPI_ISL_17218463, EPI_ISL_17218469, EPI_ISL_17218470, EPI_ISL_17218476, EPI_ISL_17218477, EPI_ISL_17218482, EPI_ISL_17218488, EPI_ISL_17218489, EPI_ISL_17218491, EPI_ISL_17218494, EPI_ISL_17218497, EPI_ISL_17218498, EPI_ISL_17218504, EPI_ISL_17218506, EPI_ISL_17218507, EPI_ISL_17218510, EPI_ISL_17218516, EPI_ISL_17218520, EPI_ISL_17218527, EPI_ISL_17218532, EPI_ISL_17218533, EPI_ISL_17218534, EPI_ISL_17218537, EPI_ISL_17218548, EPI_ISL_17218549, EPI_ISL_17218556, EPI_ISL_17218562, EPI_ISL_17218565, EPI_ISL_17218568, EPI_ISL_17218571, EPI_ISL_17218574, EPI_ISL_17218577, EPI_ISL_17218578, EPI_ISL_17218580, EPI_ISL_17218581, EPI_ISL_17218584, EPI_ISL_17218592, EPI_ISL_17218598, EPI_ISL_17218599, EPI_ISL_17218602, EPI_ISL_17218604, EPI_ISL_17218605, EPI_ISL_17218606, EPI_ISL_17218611, EPI_ISL_17218614, EPI_ISL_17218620, EPI_ISL_17218624, EPI_ISL_17218626, EPI_ISL_17218627, EPI_ISL_17218633, EPI_ISL_17218634, EPI_ISL_17218637, EPI_ISL_17218639, EPI_ISL_17218646, EPI_ISL_17218651, EPI_ISL_17218660, EPI_ISL_17218675, EPI_ISL_17218677, EPI_ISL_17218683, EPI_ISL_17218684, EPI_ISL_17218690, EPI_ISL_17218695, EPI_ISL_17218698, EPI_ISL_17218701, EPI_ISL_17218703, EPI_ISL_17218719, EPI_ISL_17218720, EPI_ISL_17218722, EPI_ISL_17218728, EPI_ISL_17218729, EPI_ISL_17218734, EPI_ISL_17218740, EPI_ISL_17218768, EPI_ISL_17218771, EPI_ISL_17218775, EPI_ISL_17218780, EPI_ISL_17218782, EPI_ISL_17218785, EPI_ISL_17218788, EPI_ISL_17218790, EPI_ISL_17218791, EPI_ISL_17218793, EPI_ISL_17218797, EPI_ISL_17218799, EPI_ISL_17218800, EPI_ISL_17218811, EPI_ISL_17218813, EPI_ISL_17218817, EPI_ISL_17218819, EPI_ISL_17218821, EPI_ISL_17218822, EPI_ISL_17218834, EPI_ISL_17218836, EPI_ISL_17218841, EPI_ISL_17218844, EPI_ISL_17218853, EPI_ISL_17218855, EPI_ISL_17218857, EPI_ISL_17218860, EPI_ISL_17218862, EPI_ISL_17218863, EPI_ISL_17218866, EPI_ISL_17218873, EPI_ISL_17218878, EPI_ISL_17218881, EPI_ISL_17218882, EPI_ISL_17218884, EPI_ISL_17218885, EPI_ISL_17218893, EPI_ISL_17218894, EPI_ISL_17218897, EPI_ISL_17218900, EPI_ISL_17218902, EPI_ISL_17218903, EPI_ISL_17218905, EPI_ISL_17218910, EPI_ISL_17218919, EPI_ISL_17218923, EPI_ISL_17218929, EPI_ISL_17218930, EPI_ISL_17218936, EPI_ISL_17218939, EPI_ISL_17218944, EPI_ISL_17218945, EPI_ISL_17218952, EPI_ISL_17218956, EPI_ISL_17218958, EPI_ISL_17218959, EPI_ISL_17218961, EPI_ISL_17218964, EPI_ISL_17218965, EPI_ISL_17218971, EPI_ISL_17218974, EPI_ISL_17218977, EPI_ISL_17218978, EPI_ISL_17218980, EPI_ISL_17218981, EPI_ISL_17218983, EPI_ISL_17219000, EPI_ISL_17219002, EPI_ISL_17219004, EPI_ISL_17219005, EPI_ISL_17219012, EPI_ISL_17219014, EPI_ISL_17219015, EPI_ISL_17219017, EPI_ISL_17219020, EPI_ISL_17219021, EPI_ISL_17219024, EPI_ISL_17219026, EPI_ISL_17219030, EPI_ISL_17219032, EPI_ISL_17219033, EPI_ISL_17219038, EPI_ISL_17219039, EPI_ISL_17219045, EPI_ISL_17219046, EPI_ISL_17219049, EPI_ISL_17219051, EPI_ISL_17219052, EPI_ISL_17219061, EPI_ISL_17219062, EPI_ISL_17219064, EPI_ISL_17219070, EPI_ISL_17219071, EPI_ISL_17219080, EPI_ISL_17219082, EPI_ISL_17219089, EPI_ISL_17219091, EPI_ISL_17219095, EPI_ISL_17219101, EPI_ISL_17219102, EPI_ISL_17219104, EPI_ISL_17219105, EPI_ISL_17219107, EPI_ISL_17219113, EPI_ISL_17219114, EPI_ISL_17219116, EPI_ISL_17219118, EPI_ISL_17219119, EPI_ISL_17219125, EPI_ISL_17219127, EPI_ISL_17219128, EPI_ISL_17219130, EPI_ISL_17219131, EPI_ISL_17219133, EPI_ISL_17219136, EPI_ISL_17219137, EPI_ISL_17219141, EPI_ISL_17219143, EPI_ISL_17219144, EPI_ISL_17219146, EPI_ISL_17219149, EPI_ISL_17219152, EPI_ISL_17219153, EPI_ISL_17219155, EPI_ISL_17219156, EPI_ISL_17219159, EPI_ISL_17219160, EPI_ISL_17219174, EPI_ISL_17219175, EPI_ISL_17219180, EPI_ISL_17219181, EPI_ISL_17219182, EPI_ISL_17219185, EPI_ISL_17219187, EPI_ISL_17219190, EPI_ISL_17219191, EPI_ISL_17219194, EPI_ISL_17219195, EPI_ISL_17219198, EPI_ISL_17219199, EPI_ISL_17219204, EPI_ISL_17219205, EPI_ISL_17219206, EPI_ISL_17219207, EPI_ISL_17219209, EPI_ISL_17219212, EPI_ISL_17219214, EPI_ISL_17219215, EPI_ISL_17219221, EPI_ISL_17219222, EPI_ISL_17219223, EPI_ISL_17219224, EPI_ISL_17219228, EPI_ISL_17219229, EPI_ISL_17219231, EPI_ISL_17219232, EPI_ISL_17219236, EPI_ISL_17219243, EPI_ISL_17219250, EPI_ISL_17219255, EPI_ISL_17219257, EPI_ISL_17219260, EPI_ISL_17219262, EPI_ISL_17219263, EPI_ISL_17219264, EPI_ISL_17219266, EPI_ISL_17219269, EPI_ISL_17219270, EPI_ISL_17219272, EPI_ISL_17219273, EPI_ISL_17219274, EPI_ISL_17219278, EPI_ISL_17219283, EPI_ISL_17219284, EPI_ISL_17219285, EPI_ISL_17219287, EPI_ISL_17219290, EPI_ISL_17219293, EPI_ISL_17219294, EPI_ISL_17219303, EPI_ISL_17219308, EPI_ISL_17219309, EPI_ISL_17219310, EPI_ISL_17219313, EPI_ISL_17219316, EPI_ISL_17219320, EPI_ISL_17219323, EPI_ISL_17219325, EPI_ISL_17219329, EPI_ISL_17219331, EPI_ISL_17219332, EPI_ISL_17219333, EPI_ISL_17219336, EPI_ISL_17219339, EPI_ISL_17219340, EPI_ISL_17219346, EPI_ISL_17219348, EPI_ISL_17219350, EPI_ISL_17219355, EPI_ISL_17219356, EPI_ISL_17219357, EPI_ISL_17219362, EPI_ISL_17219365, EPI_ISL_17219369, EPI_ISL_17219371, EPI_ISL_17219375, EPI_ISL_17219376, EPI_ISL_17219378, EPI_ISL_17219379, EPI_ISL_17219383, EPI_ISL_17219393, EPI_ISL_17219395, EPI_ISL_17219400, EPI_ISL_17219404, EPI_ISL_17219405, EPI_ISL_17219406, EPI_ISL_17219410, EPI_ISL_17219414, EPI_ISL_17219415, EPI_ISL_17219418, EPI_ISL_17219419, EPI_ISL_17219420, EPI_ISL_17219426, EPI_ISL_17219427, EPI_ISL_17219429, EPI_ISL_17219435, EPI_ISL_17219437, EPI_ISL_17219440, EPI_ISL_17219441, EPI_ISL_17219444, EPI_ISL_17219445, EPI_ISL_17219447, EPI_ISL_17219449, EPI_ISL_17219450, EPI_ISL_17219453, EPI_ISL_17219454, EPI_ISL_17219455, EPI_ISL_17219457, EPI_ISL_17219463, EPI_ISL_17219466, EPI_ISL_17219468, EPI_ISL_17219470, EPI_ISL_17219473, EPI_ISL_17219474, EPI_ISL_17219475, EPI_ISL_17219476, EPI_ISL_17219480, EPI_ISL_17219481, EPI_ISL_17219483, EPI_ISL_17219484, EPI_ISL_17219485, EPI_ISL_17219489, EPI_ISL_17219491, EPI_ISL_17219493, EPI_ISL_17219494, EPI_ISL_17219495, EPI_ISL_17219497, EPI_ISL_17219501, EPI_ISL_17219502, EPI_ISL_17219503, EPI_ISL_17219508, EPI_ISL_17219511, EPI_ISL_17219512, EPI_ISL_17219513, EPI_ISL_17219517, EPI_ISL_17219521, EPI_ISL_17219523, EPI_ISL_17219524, EPI_ISL_17219536, EPI_ISL_17219537, EPI_ISL_17219539, EPI_ISL_17219543, EPI_ISL_17219544, EPI_ISL_17219545, EPI_ISL_17219546, EPI_ISL_17219547, EPI_ISL_17219549, EPI_ISL_17219550, EPI_ISL_17219551, EPI_ISL_17219552, EPI_ISL_17219555, EPI_ISL_17219557, EPI_ISL_17219559, EPI_ISL_17219560, EPI_ISL_17219562, EPI_ISL_17219565, EPI_ISL_17219569, EPI_ISL_17219570, EPI_ISL_17219575, EPI_ISL_17219576, EPI_ISL_17219577, EPI_ISL_17219579, EPI_ISL_17219583, EPI_ISL_17219586, EPI_ISL_17219587, EPI_ISL_17219590, EPI_ISL_17219591, EPI_ISL_17219598, EPI_ISL_17219599, EPI_ISL_17219600, EPI_ISL_17219601, EPI_ISL_17219604, EPI_ISL_17219606, EPI_ISL_17219610, EPI_ISL_17219612, EPI_ISL_17219614, EPI_ISL_17219616, EPI_ISL_17219622, EPI_ISL_17219623, EPI_ISL_17219624, EPI_ISL_17219628, EPI_ISL_17219629, EPI_ISL_17219633, EPI_ISL_17219634, EPI_ISL_17219642, EPI_ISL_17219644, EPI_ISL_17219646, EPI_ISL_17219649, EPI_ISL_17219650, EPI_ISL_17219657, EPI_ISL_17219658, EPI_ISL_17219660, EPI_ISL_17219661, EPI_ISL_17219664, EPI_ISL_17219667, EPI_ISL_17219668, EPI_ISL_17219670, EPI_ISL_17219673, EPI_ISL_17219674, EPI_ISL_17219676, EPI_ISL_17219679, EPI_ISL_17219681, EPI_ISL_17219683, EPI_ISL_17219685, EPI_ISL_17219691, EPI_ISL_17219697, EPI_ISL_17219700, EPI_ISL_17219701, EPI_ISL_17219702, EPI_ISL_17219703, EPI_ISL_17219704, EPI_ISL_17219706, EPI_ISL_17219708, EPI_ISL_17219710, EPI_ISL_17219714, EPI_ISL_17219716, EPI_ISL_17219717, EPI_ISL_17219718, EPI_ISL_17219723, EPI_ISL_17219725, EPI_ISL_17219728, EPI_ISL_17219730, EPI_ISL_17219731, EPI_ISL_17219732, EPI_ISL_17219735, EPI_ISL_17219736, EPI_ISL_17219739, EPI_ISL_17219740, EPI_ISL_17219742, EPI_ISL_17219743, EPI_ISL_17219744, EPI_ISL_17219749, EPI_ISL_17219751, EPI_ISL_17219754, EPI_ISL_17219755, EPI_ISL_17219757, EPI_ISL_17219759, EPI_ISL_17219762, EPI_ISL_17219763, EPI_ISL_17219767, EPI_ISL_17219768, EPI_ISL_17219772, EPI_ISL_17219774, EPI_ISL_17219779, EPI_ISL_17219780, EPI_ISL_17219782, EPI_ISL_17219783, EPI_ISL_17219784, EPI_ISL_17219787, EPI_ISL_17219788, EPI_ISL_17219789, EPI_ISL_17219793, EPI_ISL_17219794, EPI_ISL_17219795, EPI_ISL_17219799, EPI_ISL_17219800, EPI_ISL_17219802, EPI_ISL_17219807, EPI_ISL_17219809, EPI_ISL_17219810, EPI_ISL_17219812, EPI_ISL_17219816, EPI_ISL_17219818, EPI_ISL_17219819, EPI_ISL_17219825, EPI_ISL_17219826, EPI_ISL_17219830, EPI_ISL_17219831, EPI_ISL_17219833, EPI_ISL_17219835, EPI_ISL_17219837, EPI_ISL_17219839, EPI_ISL_17219843, EPI_ISL_17219844, EPI_ISL_17219845, EPI_ISL_17219847, EPI_ISL_17219848, EPI_ISL_17219849, EPI_ISL_17219851, EPI_ISL_17219853, EPI_ISL_17219854, EPI_ISL_17219857, EPI_ISL_17219858, EPI_ISL_17219859, EPI_ISL_17219863, EPI_ISL_17219864, EPI_ISL_17219868, EPI_ISL_17219871, EPI_ISL_17219872, EPI_ISL_17219873, EPI_ISL_17219875, EPI_ISL_17219876, EPI_ISL_17219877, EPI_ISL_17219879, EPI_ISL_17219880, EPI_ISL_17219881, EPI_ISL_17219883, EPI_ISL_17219884, EPI_ISL_17219885, EPI_ISL_17219886, EPI_ISL_17219888, EPI_ISL_17219892, EPI_ISL_17219893, EPI_ISL_17219894, EPI_ISL_17219897, EPI_ISL_17219900, EPI_ISL_17219904, EPI_ISL_17219909, EPI_ISL_17219911, EPI_ISL_17219915, EPI_ISL_17219917, EPI_ISL_17219918, EPI_ISL_17219921, EPI_ISL_17219923, EPI_ISL_17219924, EPI_ISL_17219926, EPI_ISL_17219928, EPI_ISL_17219930, EPI_ISL_17219936, EPI_ISL_17219937, EPI_ISL_17219941, EPI_ISL_17219950, EPI_ISL_17219951, EPI_ISL_17219952, EPI_ISL_17219954, EPI_ISL_17219956, EPI_ISL_17219959, EPI_ISL_17219961, EPI_ISL_17219964, EPI_ISL_17219966, EPI_ISL_17219970, EPI_ISL_17219971, EPI_ISL_17219972, EPI_ISL_17219973, EPI_ISL_17219980, EPI_ISL_17219987, EPI_ISL_17219992, EPI_ISL_17219997, EPI_ISL_17219999, EPI_ISL_17220001, EPI_ISL_17220002, EPI_ISL_17220003, EPI_ISL_17220005, EPI_ISL_17220007, EPI_ISL_17220008, EPI_ISL_17220009, EPI_ISL_17220011, EPI_ISL_17220013, EPI_ISL_17220016, EPI_ISL_17220017, EPI_ISL_17220018, EPI_ISL_17220019, EPI_ISL_17220021, EPI_ISL_17220023, EPI_ISL_17220024, EPI_ISL_17220025, EPI_ISL_17220033, EPI_ISL_17220034, EPI_ISL_17220037, EPI_ISL_17220038, EPI_ISL_17220039, EPI_ISL_17220043, EPI_ISL_17220045, EPI_ISL_17220046, EPI_ISL_17220048, EPI_ISL_17220057, EPI_ISL_17220058, EPI_ISL_17220060, EPI_ISL_17220061, EPI_ISL_17220063, EPI_ISL_17220066, EPI_ISL_17220068, EPI_ISL_17220071, EPI_ISL_17220073, EPI_ISL_17220075, EPI_ISL_17220076, EPI_ISL_17220077, EPI_ISL_17220083, EPI_ISL_17220086, EPI_ISL_17220087, EPI_ISL_17220089, EPI_ISL_17220092, EPI_ISL_17220095, EPI_ISL_17220096, EPI_ISL_17220099, EPI_ISL_17220101, EPI_ISL_17220104, EPI_ISL_17220105, EPI_ISL_17220106, EPI_ISL_17220111, EPI_ISL_17220113, EPI_ISL_17220114, EPI_ISL_17220116, EPI_ISL_17220119, EPI_ISL_17220120, EPI_ISL_17220121, EPI_ISL_17220124, EPI_ISL_17220125, EPI_ISL_17220127, EPI_ISL_17220130, EPI_ISL_17220132, EPI_ISL_17220133, EPI_ISL_17220135, EPI_ISL_17220137, EPI_ISL_17220139, EPI_ISL_17220142, EPI_ISL_17220146, EPI_ISL_17220150, EPI_ISL_17220153, EPI_ISL_17220154, EPI_ISL_17220158, EPI_ISL_17220165, EPI_ISL_17220166, EPI_ISL_17220167, EPI_ISL_17220168, EPI_ISL_17220169, EPI_ISL_17220173, EPI_ISL_17220174, EPI_ISL_17220175, EPI_ISL_17220176, EPI_ISL_17220179, EPI_ISL_17220184, EPI_ISL_17220188, EPI_ISL_17220189, EPI_ISL_17220191, EPI_ISL_17220192, EPI_ISL_17220194, EPI_ISL_17220199, EPI_ISL_17220200, EPI_ISL_17220201, EPI_ISL_17220202, EPI_ISL_17220203, EPI_ISL_17220204, EPI_ISL_17220205, EPI_ISL_17220207, EPI_ISL_17220208, EPI_ISL_17220211, EPI_ISL_17220213, EPI_ISL_17220215, EPI_ISL_17220218, EPI_ISL_17220219, EPI_ISL_17220220, EPI_ISL_17220223, EPI_ISL_17220227, EPI_ISL_17220230, EPI_ISL_17220231, EPI_ISL_17220234, EPI_ISL_17220236, EPI_ISL_17220237, EPI_ISL_17220239, EPI_ISL_17220240, EPI_ISL_17220244, EPI_ISL_17220246, EPI_ISL_17220250, EPI_ISL_17220252, EPI_ISL_17220254, EPI_ISL_17220256, EPI_ISL_17220258, EPI_ISL_17220260, EPI_ISL_17220261, EPI_ISL_17220263, EPI_ISL_17220264, EPI_ISL_17220265, EPI_ISL_17220267, EPI_ISL_17220269, EPI_ISL_17220270, EPI_ISL_17220275, EPI_ISL_17220284, EPI_ISL_17220287, EPI_ISL_17220290, EPI_ISL_17220302, EPI_ISL_17220319, EPI_ISL_17220340, EPI_ISL_17220345, EPI_ISL_17220349, EPI_ISL_17220351, EPI_ISL_17220363, EPI_ISL_17220364, EPI_ISL_17220370, EPI_ISL_17220375, EPI_ISL_17220381, EPI_ISL_17220387, EPI_ISL_17220388, EPI_ISL_17220401, EPI_ISL_17220403, EPI_ISL_17220420, EPI_ISL_17220422, EPI_ISL_17220427, EPI_ISL_17220434, EPI_ISL_17220437, EPI_ISL_17220445, EPI_ISL_17220446, EPI_ISL_17220447, EPI_ISL_17220467, EPI_ISL_17220468, EPI_ISL_17220470, EPI_ISL_17220472, EPI_ISL_17220480, EPI_ISL_17220481, EPI_ISL_17220484, EPI_ISL_17220485, EPI_ISL_17220492, EPI_ISL_17220493, EPI_ISL_17220501, EPI_ISL_17220515, EPI_ISL_17220518, EPI_ISL_17220519, EPI_ISL_17220520, EPI_ISL_17220524, EPI_ISL_17220526, EPI_ISL_17220528, EPI_ISL_17220529, EPI_ISL_17220532, EPI_ISL_17220534, EPI_ISL_17220540, EPI_ISL_17220545, EPI_ISL_17220554, EPI_ISL_17220566, EPI_ISL_17220567, EPI_ISL_17220568, EPI_ISL_17220580, EPI_ISL_17220584, EPI_ISL_17220585, EPI_ISL_17220586, EPI_ISL_17220588, EPI_ISL_17220596, EPI_ISL_17220599, EPI_ISL_17220602, EPI_ISL_17220603, EPI_ISL_17220607, EPI_ISL_17220610, EPI_ISL_17220612, EPI_ISL_17220613, EPI_ISL_17220619, EPI_ISL_17220621, EPI_ISL_17220625, EPI_ISL_17220630, EPI_ISL_17220634, EPI_ISL_17220643, EPI_ISL_17220655, EPI_ISL_17220660, EPI_ISL_17220665, EPI_ISL_17220671, EPI_ISL_17220678, EPI_ISL_17220684, EPI_ISL_17220685, EPI_ISL_17220693, EPI_ISL_17220701, EPI_ISL_17220704, EPI_ISL_17220709, EPI_ISL_17220717, EPI_ISL_17220719, EPI_ISL_17220723, EPI_ISL_17220732, EPI_ISL_17220733, EPI_ISL_17220735, EPI_ISL_17220740, EPI_ISL_17220749, EPI_ISL_17220756, EPI_ISL_17220771, EPI_ISL_17220772, EPI_ISL_17220781, EPI_ISL_17220790, EPI_ISL_17220793, EPI_ISL_17220798, EPI_ISL_17220801, EPI_ISL_17220804, EPI_ISL_17220807, EPI_ISL_17220813, EPI_ISL_17220822, EPI_ISL_17220826, EPI_ISL_17220827, EPI_ISL_17220828, EPI_ISL_17220829, EPI_ISL_17220831, EPI_ISL_17220838, EPI_ISL_17220841, EPI_ISL_17220843, EPI_ISL_17220846, EPI_ISL_17220855, EPI_ISL_17220856, EPI_ISL_17220861, EPI_ISL_17220863, EPI_ISL_17220866, EPI_ISL_17220867, EPI_ISL_17220870, EPI_ISL_17220882, EPI_ISL_17220884, EPI_ISL_17220886, EPI_ISL_17220892, EPI_ISL_17220914, EPI_ISL_17220915, EPI_ISL_17220919, EPI_ISL_17220920, EPI_ISL_17220924, EPI_ISL_17220925, EPI_ISL_17220927, EPI_ISL_17220930, EPI_ISL_17220934, EPI_ISL_17220943, EPI_ISL_17220945, EPI_ISL_17220958, EPI_ISL_17220963, EPI_ISL_17220969, EPI_ISL_17220976, EPI_ISL_17220981, EPI_ISL_17220991, EPI_ISL_17220995, EPI_ISL_17221006, EPI_ISL_17221011, EPI_ISL_17221018, EPI_ISL_17221021, EPI_ISL_17221031, EPI_ISL_17221037, EPI_ISL_17221040, EPI_ISL_17221044, EPI_ISL_17221048, EPI_ISL_17221049, EPI_ISL_17221050, EPI_ISL_17221053, EPI_ISL_17221054, EPI_ISL_17221058, EPI_ISL_17221060, EPI_ISL_17221065, EPI_ISL_17221072, EPI_ISL_17221078, EPI_ISL_17221083, EPI_ISL_17221096, EPI_ISL_17221104, EPI_ISL_17221105, EPI_ISL_17221117, EPI_ISL_17221123, EPI_ISL_17221126, EPI_ISL_17221131, EPI_ISL_17221140, EPI_ISL_17221142, EPI_ISL_17221143, EPI_ISL_17221146, EPI_ISL_17221147, EPI_ISL_17221149, EPI_ISL_17221150, EPI_ISL_17221153, EPI_ISL_17221154, EPI_ISL_17221158, EPI_ISL_17221159, EPI_ISL_17221160, EPI_ISL_17221161, EPI_ISL_17221164, EPI_ISL_17221165, EPI_ISL_17221166, EPI_ISL_17221168, EPI_ISL_17221169, EPI_ISL_17221172, EPI_ISL_17221173, EPI_ISL_17221179, EPI_ISL_17221180, EPI_ISL_17221181, EPI_ISL_17221185, EPI_ISL_17221189, EPI_ISL_17221190, EPI_ISL_17221193, EPI_ISL_17221194, EPI_ISL_17221199, EPI_ISL_17221200, EPI_ISL_17221209, EPI_ISL_17221214, EPI_ISL_17221218, EPI_ISL_17221220, EPI_ISL_17221236, EPI_ISL_17221239, EPI_ISL_17221246, EPI_ISL_17221259, EPI_ISL_17221265, EPI_ISL_17221269, EPI_ISL_17221276, EPI_ISL_17221283, EPI_ISL_17221284, EPI_ISL_17221296, EPI_ISL_17221300, EPI_ISL_17221306, EPI_ISL_17221310, EPI_ISL_17221321, EPI_ISL_17221323, EPI_ISL_17221327, EPI_ISL_17221330, EPI_ISL_17221331, EPI_ISL_17221339, EPI_ISL_17221345, EPI_ISL_17221352, EPI_ISL_17221371, EPI_ISL_17221386, EPI_ISL_17221388, EPI_ISL_17221409, EPI_ISL_17221415, EPI_ISL_17221424, EPI_ISL_17221430, EPI_ISL_17221432, EPI_ISL_17221435, EPI_ISL_17221436, EPI_ISL_17221442, EPI_ISL_17221449, EPI_ISL_17221457, EPI_ISL_17221461, EPI_ISL_17221465, EPI_ISL_17221469, EPI_ISL_17221471, EPI_ISL_17221472, EPI_ISL_17221473, EPI_ISL_17221478, EPI_ISL_17221483, EPI_ISL_17221488, EPI_ISL_17221496, EPI_ISL_17221499, EPI_ISL_17221501, EPI_ISL_17221509, EPI_ISL_17221511, EPI_ISL_17221512, EPI_ISL_17221517, EPI_ISL_17221518, EPI_ISL_17221519, EPI_ISL_17221520, EPI_ISL_17221528, EPI_ISL_17221530			
see above	Astrazeneca Laboratory	Astrazeneca Laboratory	Wilkins,D., Langedijk,A.C., Lebbink,R.J., Morehouse,C.A., Abram,M.E., Ahani,B., Aksyuk,A.A., Baraldi,E., Brady,T., Chen,A.T., Chi,H., Choi,E.H., Cohen,R., Danilenko,D.M., Gopalakrishnan,V., Greenough,A., Heikkinen,T., Hosoya,M., Keller,C., Kelly,E.J., Kragten-Tabatabaie,L., Martinon-Torres,F., Mascarenas-de Los Santos,A.H., Nunes,M.C., Palomino,M.A., Papenburg,J., Pernica,J.M., Richmond,P., Stein,R.T., Tuffy,K.M., Verwey,C., Esser,M.T., Tabor,D.E. and Bont,L.J.
EPI_ISL_17221539, EPI_ISL_17221541, EPI_ISL_17221546, EPI_ISL_17221550, EPI_ISL_17221561, EPI_ISL_17221567, EPI_ISL_17221578, EPI_ISL_17221580, EPI_ISL_17221582, EPI_ISL_17221585, EPI_ISL_17221586, EPI_ISL_17221590, EPI_ISL_17221593, EPI_ISL_17221597, EPI_ISL_17221605, EPI_ISL_17221607, EPI_ISL_17221623, EPI_ISL_17221625, EPI_ISL_17221629, EPI_ISL_17221634, EPI_ISL_17221637, EPI_ISL_17221639, EPI_ISL_17221644, EPI_ISL_17221647, EPI_ISL_17221649, EPI_ISL_17221654, EPI_ISL_17221658, EPI_ISL_17221660, EPI_ISL_17221662, EPI_ISL_17221666			
see above	University of Ljubljana - Faculty of Medicine	University of Ljubljana - Faculty of Medicine	Jevsnik Virant,M., Lustrek,M. and Petrovec,M.
EPI_ISL_17221671, EPI_ISL_17221674	University of Washington - Virology	University of Washington - Virology	Sereewit,J., Xie,H., Roychoudhury,P. and Greninger,A.L.
EPI_ISL_17226496, EPI_ISL_17226497, EPI_ISL_17226498, EPI_ISL_17226499, EPI_ISL_17226500, EPI_ISL_17226501, EPI_ISL_17226502	Hainan Medical University	Hainan Medical University	Tian,X., Yin,F. and Xiao,M.
EPI_ISL_17308687, EPI_ISL_17308688, EPI_ISL_17308689, EPI_ISL_17308691, EPI_ISL_17308692, EPI_ISL_17308695, EPI_ISL_17308698, EPI_ISL_17308699, EPI_ISL_17308700, EPI_ISL_17308701, EPI_ISL_17308707, EPI_ISL_17308709, EPI_ISL_17308711, EPI_ISL_17308712, EPI_ISL_17308714, EPI_ISL_17308715, EPI_ISL_17308716, EPI_ISL_17308724, EPI_ISL_17308725, EPI_ISL_17308728			
see above	Laboratório de Virologia - Instituto Nacional de Saúde	Centers for Disease Control and Prevention - United States of America, Laboratório de Virologia - Instituto Nacional de Saúde	Almiro Tivane, Everardo Vega, Amanda Smith, Lijuan Wang, Neuza Nguenha, Loira Machalele, Sadia Alí, Délcio Muteto, Mirela Pale, Aunésia Marrurele, Félix Gundane, Judite Salência
EPI_ISL_17401576, EPI_ISL_17401577	Hainan Medical University, Hainan Medical University-The University of Hong Kong Joint Laboratory of Tropical Infectious Diseases	Hainan Medical University, Hainan Medical University-The University of Hong Kong Joint Laboratory of Tropical Infectious Diseases	Jia,Y. and Yin,F.
EPI_ISL_17417578, EPI_ISL_17417579, EPI_ISL_17417580, EPI_ISL_17417581, EPI_ISL_17417582, EPI_ISL_17417583, EPI_ISL_17417584, EPI_ISL_17417585, EPI_ISL_17417586, EPI_ISL_17417589, EPI_ISL_17417591, EPI_ISL_17417592, EPI_ISL_17417594, EPI_ISL_17417595, EPI_ISL_17417596, EPI_ISL_17417597, EPI_ISL_17417598, EPI_ISL_17417599, EPI_ISL_17417600, EPI_ISL_17417601, EPI_ISL_17417602, EPI_ISL_17417603, EPI_ISL_17417604			
see above	Centers for Disease Control and Prevention (CDC)	Centers for Disease Control and Prevention (CDC)	Wang,L., Lumandas,M.U., Arguelles,V.L., Morin,J., Aggarwal,M., Tatusov,R. and Vega,E.
EPI_ISL_17559466, EPI_ISL_17559467, EPI_ISL_17559468	Laboratory of Respiratory Viruses and Measles, Oswaldo Cruz Institute, FIOCRUZ	Laboratorio de Alta Complexidade do Instituto Fernandes Figueira - LACIFF	Paola Resende, Fernando Motta, Elisa Cavalcante Pereira, Bruna Mendonça da Silva, Jéssica Graça Macedo de Carvalho, Larissa Macedo Pinto, Victor Guimaraes, Letícia Lima, Letícia Scalioni, Marilda Siqueira on behalf of the Fiocruz COVID-19 Genomic Surveillance Network
EPI_ISL_17594417, EPI_ISL_17594424, EPI_ISL_17594425, EPI_ISL_17594426, EPI_ISL_17594427, EPI_ISL_17594456, EPI_ISL_17594526, EPI_ISL_17594527, EPI_ISL_17594528, EPI_ISL_17594539, EPI_ISL_17594542, EPI_ISL_17594575, EPI_ISL_17594592, EPI_ISL_17594593, EPI_ISL_17594594, EPI_ISL_17594599, EPI_ISL_17594600, EPI_ISL_17594601, EPI_ISL_17594602, EPI_ISL_17594603, EPI_ISL_17594604, EPI_ISL_17594605, EPI_ISL_17594606, EPI_ISL_17594607, EPI_ISL_17594608, EPI_ISL_17594609, EPI_ISL_17594610, EPI_ISL_17594611, EPI_ISL_17594612, EPI_ISL_17594613, EPI_ISL_17594614, EPI_ISL_17594615, EPI_ISL_17594617, EPI_ISL_17594618, EPI_ISL_17594619, EPI_ISL_17594620, EPI_ISL_17594621, EPI_ISL_17594622, EPI_ISL_17594623, EPI_ISL_17594624, EPI_ISL_17594625, EPI_ISL_17594626, EPI_ISL_17594627, EPI_ISL_17594628, EPI_ISL_17594629, EPI_ISL_17594630, EPI_ISL_17594631, EPI_ISL_17594632, EPI_ISL_17594633, EPI_ISL_17594635, EPI_ISL_17594638, EPI_ISL_17594639, EPI_ISL_17594640, EPI_ISL_17594641, EPI_ISL_17594642, EPI_ISL_17594643, EPI_ISL_17594644, EPI_ISL_17594645, EPI_ISL_17594646, EPI_ISL_17594647, EPI_ISL_17594648, EPI_ISL_17594649, EPI_ISL_17594650, EPI_ISL_17594651, EPI_ISL_17594652, EPI_ISL_17594655, EPI_ISL_17594656, EPI_ISL_17594660, EPI_ISL_17594662, EPI_ISL_17594663, EPI_ISL_17594664			
see above	Chang Bing Show Chwan Memorial Hospital	Chang Bing Show Chwan Memorial Hospital	Lee,C.Y.
EPI_ISL_17594667	Institute for Infectious Disease Control and Prevention, Henan Center for Disease Control and Prevention	Institute for Infectious Disease Control and Prevention, Henan Center for Disease Control and Prevention	Zhao,X.
EPI_ISL_17594668, EPI_ISL_17594670, EPI_ISL_17594671, EPI_ISL_17594694, EPI_ISL_17594708, EPI_ISL_17594717, EPI_ISL_17594718, EPI_ISL_17594719, EPI_ISL_17594721, EPI_ISL_17594722, EPI_ISL_17594724, EPI_ISL_17594726, EPI_ISL_17594727, EPI_ISL_17594728, EPI_ISL_17594729, EPI_ISL_17594730, EPI_ISL_17594731, EPI_ISL_17594732, EPI_ISL_17594733, EPI_ISL_17594734, EPI_ISL_17594735, EPI_ISL_17594736, EPI_ISL_17594737, EPI_ISL_17594739, EPI_ISL_17594740, EPI_ISL_17594741, EPI_ISL_17594742, EPI_ISL_17594743, EPI_ISL_17594746, EPI_ISL_17594747, EPI_ISL_17594749, EPI_ISL_17594751, EPI_ISL_17594752, EPI_ISL_17594753, EPI_ISL_17594754, EPI_ISL_17594755, EPI_ISL_17594756, EPI_ISL_17594757, EPI_ISL_17594758, EPI_ISL_17594767, EPI_ISL_17594769, EPI_ISL_17594771, EPI_ISL_17594772, EPI_ISL_17594773, EPI_ISL_17594775, EPI_ISL_17594776, EPI_ISL_17594777, EPI_ISL_17594778, EPI_ISL_17594779, EPI_ISL_17594780			
see above	Institute for Infectious Disease Control and Prevention, Henan Center for Disease Control and Prevention	Institute for Infectious Disease Control and Prevention, Henan Center for Disease Control and Prevention	Zhao,X., Teng,Z. and Zhang,X.
EPI_ISL_17797314, EPI_ISL_17797320, EPI_ISL_17797321, EPI_ISL_17797322, EPI_ISL_17797326, EPI_ISL_17797327, EPI_ISL_17797329, EPI_ISL_17797330, EPI_ISL_17797331	Centre Pasteur in Cameroon, National Reference Laboratory for Chikungunya and Dengue	Centre Pasteur in Cameroon, National Reference Laboratory for Chikungunya and Dengue	Moumbeket Yifomnjou,M.H. and Njouom,R.
EPI_ISL_17797334, EPI_ISL_17797335, EPI_ISL_17797336, EPI_ISL_17797339, EPI_ISL_17797342, EPI_ISL_17797343, EPI_ISL_17797344, EPI_ISL_17797345, EPI_ISL_17797347, EPI_ISL_17797349, EPI_ISL_17797356, EPI_ISL_17797358, EPI_ISL_17797359, EPI_ISL_17797361, EPI_ISL_17797364, EPI_ISL_17797367, EPI_ISL_17797373, EPI_ISL_17797374, EPI_ISL_17797375, EPI_ISL_17797376,			

EPI_ISL_17797377, EPI_ISL_17797378, EPI_ISL_17797379, EPI_ISL_17797381, EPI_ISL_17797383, EPI_ISL_17797388, EPI_ISL_17797393, EPI_ISL_17797397, EPI_ISL_17797399, EPI_ISL_17797401, EPI_ISL_17797402, EPI_ISL_17797404, EPI_ISL_17797405, EPI_ISL_17797406, EPI_ISL_17797407, EPI_ISL_17797408, EPI_ISL_17797409, EPI_ISL_17797410, EPI_ISL_17797411, EPI_ISL_17797412, EPI_ISL_17797413, EPI_ISL_17797414, EPI_ISL_17797415, EPI_ISL_17797418, EPI_ISL_17797419, EPI_ISL_17797428, EPI_ISL_17797430, EPI_ISL_17797434, EPI_ISL_17797435, EPI_ISL_17797438, EPI_ISL_17797440, EPI_ISL_17797442, EPI_ISL_17797443, EPI_ISL_17797444, EPI_ISL_17797447, EPI_ISL_17797448, EPI_ISL_17797449, EPI_ISL_17797451, EPI_ISL_17797452, EPI_ISL_17797455, EPI_ISL_17797456, EPI_ISL_17797459, EPI_ISL_17797460, EPI_ISL_17797461, EPI_ISL_17797463, EPI_ISL_17797465, EPI_ISL_17797466, EPI_ISL_17797468, EPI_ISL_17797470, EPI_ISL_17797472, EPI_ISL_17797474, EPI_ISL_17797477, EPI_ISL_17797478, EPI_ISL_17797481, EPI_ISL_17797484, EPI_ISL_17797485, EPI_ISL_17797491, EPI_ISL_17797493, EPI_ISL_17797494, EPI_ISL_17797495, EPI_ISL_17797498, EPI_ISL_17797499, EPI_ISL_17797503, EPI_ISL_17797507, EPI_ISL_17797508, EPI_ISL_17797510, EPI_ISL_17797511, EPI_ISL_17797512, EPI_ISL_17797513, EPI_ISL_17797514, EPI_ISL_17797515, EPI_ISL_17797518, EPI_ISL_17797519, EPI_ISL_17797520, EPI_ISL_17797521, EPI_ISL_17797523, EPI_ISL_17797525			
see above	Chiang Mai University, Department of Microbiology	Chiang Mai University, Department of Microbiology	Lin,W.H.
EPI_ISL_17953816, EPI_ISL_17953831, EPI_ISL_17953833	State Key Laboratory for Diagnosis and Treatment of Infectious Diseases, First Affiliated Hospital, School of Medicine, Zhejiang University	State Key Laboratory for Diagnosis and Treatment of Infectious Diseases, First Affiliated Hospital, School of Medicine, Zhejiang University	Guo,Y.
EPI_ISL_17964308, EPI_ISL_17964310, EPI_ISL_17964314, EPI_ISL_17964318, EPI_ISL_17964319, EPI_ISL_17964320, EPI_ISL_17964325, EPI_ISL_17964327, EPI_ISL_17964331, EPI_ISL_17964335, EPI_ISL_17964341, EPI_ISL_17964343, EPI_ISL_17964346, EPI_ISL_17964348, EPI_ISL_17964349, EPI_ISL_17964350, EPI_ISL_17964352, EPI_ISL_17964356, EPI_ISL_17964368, EPI_ISL_17964369, EPI_ISL_17964371, EPI_ISL_17964373			
see above	University of Zambia Medical School	Department of Medical Microbiology, University Medical Centre Utrecht	Annefleur C. Langedijk, Bram Vrancken, Robert Jan Lebbink, Rachel C. Pieciak, Philippe Lemey, Louis J. Bont, Christopher J Gill
EPI_ISL_17975537, EPI_ISL_17975538, EPI_ISL_17975539, EPI_ISL_17975540, EPI_ISL_17975543, EPI_ISL_17975545, EPI_ISL_17975553, EPI_ISL_17975556, EPI_ISL_17975571, EPI_ISL_17975588, EPI_ISL_17975591, EPI_ISL_17975604, EPI_ISL_17975609, EPI_ISL_17975612, EPI_ISL_17975616, EPI_ISL_17975620, EPI_ISL_17975627, EPI_ISL_17975628, EPI_ISL_17975634, EPI_ISL_17975637, EPI_ISL_17975639			
see above	Sapienza University	Sapienza University	Pierangeli,A.
EPI_ISL_17975644, EPI_ISL_17975645, EPI_ISL_17975646, EPI_ISL_17975647, EPI_ISL_17975648	United Arab Emirates University	United Arab Emirates University	Alsamri,M.T., Narchi,H., George,J.A. and Alsuwaidi,A.R.
EPI_ISL_17995637, EPI_ISL_17995655, EPI_ISL_17995657, EPI_ISL_17995658, EPI_ISL_17995660, EPI_ISL_17995661, EPI_ISL_17995662, EPI_ISL_17995663, EPI_ISL_17995664, EPI_ISL_17995665, EPI_ISL_17995677, EPI_ISL_17995678, EPI_ISL_17995679, EPI_ISL_17995680, EPI_ISL_17995681, EPI_ISL_17995682, EPI_ISL_17995683, EPI_ISL_17995684, EPI_ISL_17995685, EPI_ISL_17995686, EPI_ISL_17995687, EPI_ISL_17995688, EPI_ISL_17995689, EPI_ISL_17995690, EPI_ISL_17995691, EPI_ISL_17995692, EPI_ISL_17995693, EPI_ISL_17995694, EPI_ISL_17995695, EPI_ISL_17995696, EPI_ISL_17995697, EPI_ISL_17995698, EPI_ISL_17995699, EPI_ISL_17995700, EPI_ISL_17995760, EPI_ISL_17995761, EPI_ISL_17995762, EPI_ISL_17995763, EPI_ISL_17995764, EPI_ISL_17995766, EPI_ISL_17995767, EPI_ISL_17995768			
see above	Robert Koch-Institute Nationales Referenzzentrum für Influenza	Robert Koch-Institute Nationales Referenzzentrum für Influenza	Sophie Kondgen, Janine Reiche
EPI_ISL_18054247, EPI_ISL_18054248, EPI_ISL_18054249, EPI_ISL_18054255, EPI_ISL_18054256, EPI_ISL_18054257, EPI_ISL_18054258, EPI_ISL_18054259, EPI_ISL_18054260, EPI_ISL_18054263, EPI_ISL_18054264, EPI_ISL_18054266, EPI_ISL_18054267, EPI_ISL_18054268, EPI_ISL_18054271, EPI_ISL_18054274			
see above	Hangzhou Center for Disease Control and Prevention, Microbiology Laboratory	Hangzhou Center for Disease Control and Prevention, Microbiology Laboratory	Chen,L.
EPI_ISL_18054283, EPI_ISL_18054284, EPI_ISL_18054301, EPI_ISL_18054348, EPI_ISL_18054355, EPI_ISL_18054375, EPI_ISL_18054378, EPI_ISL_18054383	Kenya Medical Research Institute - KEMRI Wellcome Trust Research Programme	Kenya Medical Research Institute - KEMRI Wellcome Trust Research Programme	Lambisia,A.W., Lewa,C., Mutunga,M., Okanda,D., Githinji,G., Nokes,J.D. and Agoti,C.N.
EPI_ISL_18143445, EPI_ISL_18143447, EPI_ISL_18143451, EPI_ISL_18143454, EPI_ISL_18143457, EPI_ISL_18143460, EPI_ISL_18143464, EPI_ISL_18143465, EPI_ISL_18143467, EPI_ISL_18143469, EPI_ISL_18143471, EPI_ISL_18143472, EPI_ISL_18143476, EPI_ISL_18143478, EPI_ISL_18143479, EPI_ISL_18143480, EPI_ISL_18143483, EPI_ISL_18143484, EPI_ISL_18143486, EPI_ISL_18143489, EPI_ISL_18143490, EPI_ISL_18143494, EPI_ISL_18143495, EPI_ISL_18143496, EPI_ISL_18143497, EPI_ISL_18143499, EPI_ISL_18143500, EPI_ISL_18143502, EPI_ISL_18143503, EPI_ISL_18143506, EPI_ISL_18143507, EPI_ISL_18143508, EPI_ISL_18143509, EPI_ISL_18143510, EPI_ISL_18143511, EPI_ISL_18143512, EPI_ISL_18143513, EPI_ISL_18143514, EPI_ISL_18143517, EPI_ISL_18143518, EPI_ISL_18143520, EPI_ISL_18143527, EPI_ISL_18143528, EPI_ISL_18143529, EPI_ISL_18143530, EPI_ISL_18143531, EPI_ISL_18143533, EPI_ISL_18143536, EPI_ISL_18143537, EPI_ISL_18143539, EPI_ISL_18143540, EPI_ISL_18143542, EPI_ISL_18143544, EPI_ISL_18143547, EPI_ISL_18143548, EPI_ISL_18143553, EPI_ISL_18143554, EPI_ISL_18143555, EPI_ISL_18143556, EPI_ISL_18143557, EPI_ISL_18143560, EPI_ISL_18143562, EPI_ISL_18143564, EPI_ISL_18143566, EPI_ISL_18143571, EPI_ISL_18143572, EPI_ISL_18143575, EPI_ISL_18143576, EPI_ISL_18143578, EPI_ISL_18143583, EPI_ISL_18143585, EPI_ISL_18143589, EPI_ISL_18143591, EPI_ISL_18143592, EPI_ISL_18143599, EPI_ISL_18143600, EPI_ISL_18143601, EPI_ISL_18143604, EPI_ISL_18143605, EPI_ISL_18143606, EPI_ISL_18143607, EPI_ISL_18143608, EPI_ISL_18143609, EPI_ISL_18143610, EPI_ISL_18143611, EPI_ISL_18143613, EPI_ISL_18143614, EPI_ISL_18143615, EPI_ISL_18143618, EPI_ISL_18143620, EPI_ISL_18143621, EPI_ISL_18143622, EPI_ISL_18143623, EPI_ISL_18143624, EPI_ISL_18143626, EPI_ISL_18143629, EPI_ISL_18143636, EPI_ISL_18143638, EPI_ISL_18143639, EPI_ISL_18143640, EPI_ISL_18143641, EPI_ISL_18143643, EPI_ISL_18143644, EPI_ISL_18143645, EPI_ISL_18143647, EPI_ISL_18143648, EPI_ISL_18143649, EPI_ISL_18143651, EPI_ISL_18143652, EPI_ISL_18143654, EPI_ISL_18143655, EPI_ISL_18143656, EPI_ISL_18143657, EPI_ISL_18143658, EPI_ISL_18143660, EPI_ISL_18143662, EPI_ISL_18143663, EPI_ISL_18143664, EPI_ISL_18143668, EPI_ISL_18143672, EPI_ISL_18143675, EPI_ISL_18143677, EPI_ISL_18143679, EPI_ISL_18143680, EPI_ISL_18143685, EPI_ISL_18143686, EPI_ISL_18143687, EPI_ISL_18143688, EPI_ISL_18143692, EPI_ISL_18143694, EPI_ISL_18143695, EPI_ISL_18143699, EPI_ISL_18143700, EPI_ISL_18143701, EPI_ISL_18143703, EPI_ISL_18143704, EPI_ISL_18143707, EPI_ISL_18143709, EPI_ISL_18143711, EPI_ISL_18143714, EPI_ISL_18143715			
see above	The Brotman Baty Institute for Precision Medicine	The Brotman Baty Institute for Precision Medicine	Frazar,C.D., Lee,J., Ryke,E., Gamboa,L., McDermot,E., Stone,J., Kolar,T., Han,P.D., Sibley,T.R., Truong,M., Reinhart,D., Wolf,C.R., Boeckh,M., Englund,J.A., Lutz,B.R., Waghmare,A., Viboud,C., Starita,L.M., Shendure,J., Bedford,T. and Chu,H.Y.
EPI_ISL_18143717, EPI_ISL_18143721, EPI_ISL_18143728, EPI_ISL_18143730	Tohoku University School of Medicine	Tohoku University School of Medicine	Simusika,P., Okamoto,M., Sakamoto,M., Dapat,C., Imamura,T., Saito,M., Azam,S., Mwila,E., Monze,M. and Oshitani,H.
EPI_ISL_18272381	West of Scotland Specialist Virology Centre	West of Scotland Specialist Virology Centre	Lynne Ferguson, Imogen Johnston-Menzies, Rory Gunson
EPI_ISL_18334079	Kitasato University, Infection Control and Immunology, Yukie Katayama Omura Satoshi Memorial Institute	Kitasato University, Infection Control and Immunology, Yukie Katayama Omura Satoshi Memorial Institute	Takai-Todaka,R., Ito,T., Haga,K., Ishiyama,R., Igarashi,T., Katayama,Y., Nakayama,T. and Katayama,K.
EPI_ISL_18334080, EPI_ISL_18334100, EPI_ISL_18334105, EPI_ISL_18334106, EPI_ISL_18334119, EPI_ISL_18334127, EPI_ISL_18334134, EPI_ISL_18334137, EPI_ISL_18334142, EPI_ISL_18334149	Tianjin Children's Hospital, Tianjin Pediatric Research Institute	Tianjin Children's Hospital, Tianjin Pediatric Research Institute	Hou,M., Meng,C., Dong,L. and Wang,H.
EPI_ISL_18334238, EPI_ISL_18334240, EPI_ISL_18334241, EPI_ISL_18334243, EPI_ISL_18334246, EPI_ISL_18334247, EPI_ISL_18334248, EPI_ISL_18334256, EPI_ISL_18334266, EPI_ISL_18334269, EPI_ISL_18334270, EPI_ISL_18334285, EPI_ISL_18334286, EPI_ISL_18334292, EPI_ISL_18334295, EPI_ISL_18334296, EPI_ISL_18334299, EPI_ISL_18334314, EPI_ISL_18334319, EPI_ISL_18334323, EPI_ISL_18334325, EPI_ISL_18334333, EPI_ISL_18334337, EPI_ISL_18334341, EPI_ISL_18334342, EPI_ISL_18334343, EPI_ISL_18334346, EPI_ISL_18334357, EPI_ISL_18334361, EPI_ISL_18334363, EPI_ISL_18334373, EPI_ISL_18334382, EPI_ISL_18334385, EPI_ISL_18334388, EPI_ISL_18334392, EPI_ISL_18334397			
see above	National Influenza Center, Virology Laboratory	National Influenza Center, Virology Laboratory	Simusika,P., Okamoto,M., Dapat,C., Muleya,W., Saito,M., Azam,S., Mwape,I., Mwila,E., Monze,M. and Oshitani,H.
EPI_ISL_18339706, EPI_ISL_18339709, EPI_ISL_18339711, EPI_ISL_18339717, EPI_ISL_18339719, EPI_ISL_18339721, EPI_ISL_18339723, EPI_ISL_18339725, EPI_ISL_18339730, EPI_ISL_18339732, EPI_ISL_18339740, EPI_ISL_18339742, EPI_ISL_18339743, EPI_ISL_18339747, EPI_ISL_18339749, EPI_ISL_18339750, EPI_ISL_18339752, EPI_ISL_18339755, EPI_ISL_18339757, EPI_ISL_18339758, EPI_ISL_18339765			
see above	Department of Microbiology, Jawaharlal Institute of Postgraduate Medical Education and Research	Department of Microbiology, Jawaharlal Institute of Postgraduate Medical Education and Research	Shameema,M., Dhodapkar,R., Sharmila,P.F.M. and Venkatesh,C.
EPI_ISL_1834096, EPI_ISL_1834099, EPI_ISL_1834100, EPI_ISL_1834101, EPI_ISL_1834110, EPI_ISL_1834114, EPI_ISL_1834126, EPI_ISL_1834129, EPI_ISL_1834135, EPI_ISL_1834136, EPI_ISL_1834143, EPI_ISL_1834145, EPI_ISL_1834149			
see above	Royal Children's Hospital	WHO Influenza Centre for Reference and Research on Influenza	Angela Todd, Yi-Mo Deng, Annette Alafaci, Naomi Komadina
EPI_ISL_1834150, EPI_ISL_1834151, EPI_ISL_1834152, EPI_ISL_1834153, EPI_ISL_1834154, EPI_ISL_1834155, EPI_ISL_1834156, EPI_ISL_1834157, EPI_ISL_1834158, EPI_ISL_1834159, EPI_ISL_1834160, EPI_ISL_1834161, EPI_ISL_1834162, EPI_ISL_1834163, EPI_ISL_1834164, EPI_ISL_1834165, EPI_ISL_1834166, EPI_ISL_1834167, EPI_ISL_1834168, EPI_ISL_1834169, EPI_ISL_1834170, EPI_ISL_1834171, EPI_ISL_1834172, EPI_ISL_1834173			
see above	Respiratory Virus Unit, National Infection Service, Public Health England	National Infection Service, Public Health England	Zambon M, Talts T, Ellis J, Miah S, Platt S
EPI_ISL_18356647	Hospital Central de Asturias, Microbiologia	Hospital Central de Asturias, Microbiologia	Rojo-Alba,S., Perez-Martinez,Z., Boga,J., Alvarez-Arguelles,M.E., Gonzalez-Alba,J.M. and Melon,S.
EPI_ISL_18401452, EPI_ISL_18401454, EPI_ISL_18401456, EPI_ISL_18401457	Niigata University (IDRC) Infectious Diseases Research Center	Niigata University (IDRC) Infectious Diseases Research Center	Wint Wint Phyu, Reiko Saito
EPI_ISL_18436332, EPI_ISL_18436335, EPI_ISL_18436345, EPI_ISL_18436346, EPI_ISL_18436348, EPI_ISL_18436350, EPI_ISL_18436352, EPI_ISL_18436353, EPI_ISL_18436354, EPI_ISL_18436355, EPI_ISL_18436361, EPI_ISL_18436364, EPI_ISL_18436371, EPI_ISL_18436376, EPI_ISL_18436378, EPI_ISL_18436388, EPI_ISL_18436390, EPI_ISL_18436395, EPI_ISL_18436398, EPI_ISL_18436400, EPI_ISL_18436405, EPI_ISL_18436408, EPI_ISL_18436411, EPI_ISL_18436413, EPI_ISL_18436414, EPI_ISL_18436417, EPI_ISL_18436418, EPI_ISL_18436419, EPI_ISL_18436429, EPI_ISL_18436431, EPI_ISL_18436444, EPI_ISL_18436446, EPI_ISL_18436457, EPI_ISL_18436464, EPI_ISL_18436465, EPI_ISL_18436470, EPI_ISL_18436474, EPI_ISL_18436480, EPI_ISL_18436484, EPI_ISL_18436487, EPI_ISL_18436490, EPI_ISL_18436498, EPI_ISL_18436499, EPI_ISL_18436502, EPI_ISL_18436505, EPI_ISL_18436506, EPI_ISL_18436513, EPI_ISL_18436516, EPI_ISL_18436517, EPI_ISL_18436524, EPI_ISL_18436532, EPI_ISL_18436541, EPI_ISL_18436542, EPI_ISL_18436549			
see above	Beijing Institute of Microbiology and Epidemiology, State Key Laboratory of Pathogen and Biosecurity, Beijing Key Laboratory of Vector Borne and Natural Focus Infectious Diseases	Beijing Institute of Microbiology and Epidemiology, State Key Laboratory of Pathogen and Biosecurity, Beijing Key Laboratory of Vector Borne and Natural Focus Infectious Diseases	Fu,Y., Li,Q., Li,F., Huang,L., Zhang,H., Chen,X. and Xie,Z.
EPI_ISL_18447450	National Centre of Infectious and Parasitic Diseases National Laboratory "Influenza and ARD"	National Centre of Infectious and Parasitic Diseases National Laboratory "Influenza and ARD"	Ivelina Trifonova; Neli Korsun; Iveta Madzharova
EPI_ISL_18463174, EPI_ISL_18463177, EPI_ISL_18463178, EPI_ISL_18463188, EPI_ISL_18463190	Hospital Universitario Severo Ochoa	Instituto de Salud Carlos III	Iglesias-Caballero, M. Camarero, S. Pozo, F. Casas, I. Jiménez, P. Zaballos, A.Varona, S. Monzón, S.Cuesta, I. García-García, M.L.
[truncated: 548,438 more chars]
