# Supplementary material for: Differential impact of COVID-19 non-pharmaceutical interventions on the epidemiological dynamics of respiratory syncytial virus subtypes A and B
Source: Sci Rep. 2024 Jun 24;14:14527. doi: 10.1038/s41598-024-64624-1 (PMC11196647; doi:10.1038/s41598-024-64624-1)
Supplement: Supplementary file 2 — Supplementary Information 2. [file 41598_2024_64624_MOESM2_ESM.docx]

Differential impact of COVID-19 non-pharmaceutical interventions on the epidemiological dynamics of respiratory syncytial virus subtypes A and B

Inga Holmdahl^1^, Samantha J. Bents^2^*, Rachel E. Baker^3^, Jean-Sebastien Casalegno^4^, Nídia Sequeira Trovão^2^, Sang Woo Park^1^, Jessica E. Metcalf^1^, Cécile Viboud^2^, Bryan Grenfell^1^

1. Department of Ecology and Evolutionary Biology, Princeton University, Princeton, NJ, USA
2. Fogarty International Center, National Institutes of Health, Bethesda, MD, USA
3. School of Public Health, Brown University, Providence, Rhode Island, USA
4. Virology Laboratory, CNR des Virus des Infections Respiratoires, Institut des Agents Infectieux, Hôpital de la Croix Rousse, Hospices Civils de Lyon, Lyon, France

*joint first authors

*Corresponding author: Samantha Bents, email: [sbents@alumni.princeton.edu](mailto:sbents@alumni.princeton.edu), phone: 715-417-2857

**Supplementary Materials**

**Model Equations**

The model is made up of a set of differential equations that determine transmission between compartments.

$$\frac{dX}{dt} = \mu- (\lambda_{A}+\lambda_{B}+\mu)X + \omega(X_{A}+X_{B})$$

$$\frac{dP_{A}}{dt}= \lambda_{A}X - (\nu+\mu)P_{A}$$

$$\frac{dP_{B}}{dt}= \lambda_{B}X - (\nu+\mu)P_{B}$$

$$\frac{dX_{A}}{dt}=\nu{(P}_{A}+Y_{A}) - (\sigma_{ho}\lambda_{A}+\sigma_{he}\lambda_{B}+\omega+\mu)X_{A}+{\omega X}_{AB}$$

$$\frac{dX_{B}}{dt}=\nu{(P}_{B}+Y_{B}) -(\sigma_{he}\lambda_{A}+\sigma_{ho}\lambda_{B}+\omega+\mu)X_{B}+{\omega X}_{AB}$$

$$\frac{dP_{BA}}{dt}=\sigma_{he}\lambda_{A}X_{B}-(\nu+\mu)P_{BA}$$

$$\frac{dP_{AB}}{dt}=\sigma_{he}\lambda_{B}X_{A}-(\nu+\mu)P_{AB}$$

$$\frac{dY_{A}}{dt}=\sigma_{ho}\lambda_{A}X_{A}-(\nu+\mu)Y_{A}$$

$$\frac{dY_{B}}{dt}=\sigma_{ho}\lambda_{B}X_{B}-(\nu+\mu)Y_{B}$$

$$\frac{dX_{AB}}{dt}= \nu(P_{AB}+P_{BA}+Y_{AB}+Y_{BA})-(\sigma_{he}\sigma_{ho}(\lambda_{A}+\lambda_{B}) -2\omega{+\mu)X}_{AB}$$

$$\frac{dY_{AB}}{dt}=\sigma_{he}\sigma_{ho}\lambda_{B}X_{AB}-(\nu+\mu)Y_{AB}$$

$$\frac{dY_{BA}}{dt}=\sigma_{he}\sigma_{ho}\lambda_{A}X_{BA}-(\nu+\mu)Y_{BA}$$

$$\lambda_{A}=\beta_{A}(P_{A}+\eta(P_{BA}+Y_{A}+Y_{BA}))$$

$$\lambda_{B}=\beta_{B}(P_{B}+\eta(P_{AB}+Y_{B}+Y_{AB}))$$

$\beta_{A}=\rho b_{A}(a$cos ($2\pi(t-\phi))+1)$)

$$\beta_{B}=r\beta_{A}$$

#### Supplementary Tables

*Table S1. Model states*

| *X* | *Uninfected, no immunity* |
| --- | --- |
| *P_A_* | *Primary infection with subtype A* |
| *P_B_* | *Primary infection with subtype B* |
| *X_A_* | *Uninfected, previously infected with subtype A* |
| *X_B_* | *Uninfected, previously infected with subtype B* |
| *P_AB_* | *Primary infection with subtype B, previously infected with subtype A* |
| *P_BA_* | *Primary infection with subtype A, previously infected with subtype B* |
| *Y_A_* | *Subsequent infection with subtype A, previously infected with subtype A* |
| *Y_B_* | *Subsequent infection with subtype B, previously infected with subtype B* |
| *X_AB_* | *Uninfected, previously infected with subtypes A and B* |
| *Y_AB_* | *Subsequent infection with subtype B, previously infected with subtypes A and B* |
| *Y_BA_* | *Subsequent infection with subtype A, previously infected with subtypes A and B* |
| $\lambda_{A}$ | *Force of infection of group A acting on naive hosts* |
| $\lambda_{B}$ | *Force of infection of group B acting on naive hosts* |
| $\beta_{A}$ | *Seasonally varying transmission coefficient for group A* |
| $\beta_{B}$ | *Seasonally varying transmission coefficient for group B* |
| $\rho$ | *Time varying reduction in transmission coefficient due to NPIs* |

*Table S2. Model parameters*

| Parameter | Definition | Estimate | |
| --- | --- | --- | --- |
| $r$ | Subtype B transmission relative to transmission by subtype A | 0.9159 | |
| $\eta$ | Infectiousness of subsequent infections relative to primary | 0.4126 | |
| $\sigma_{heterotypic}$ | Relative susceptibility to heterologous infection | 0.8426 | |
| $\sigma_{homotypic}$ | Relative susceptibility to homologous (secondary) infection | 0.3569 | |
| $\nu$ | Rate of recovery (1/duration of infectiousness) | 0.0247 years^-1^ | |
| $\omega$ | Rate of waning of immunity (1/duration of immunity) | 0.51 years^-1^ | |
| $b_{A}$ | Average transmission coefficient of subtype A | UK | 0.312 |
|  |  | Finland | 0.273 |
| $\mu$ | Birth/death rate | UK | 0.014 years^-1^ |
|  |  | Finland | 0.012  years^-1^ |
| $\phi$ | Point in the year of peak transmission | UK | 0.929 |
|  |  | Finland | 0.97 |
| $a$ | Amplitude of transmission coefficient | UK | 0.815 |
|  |  | Finland | 0.347 |
| $s_{f}$ | Scaling factor | UK | 397.3 |
|  |  | Finland | 6.638 |

####

#### **Supplementary Figures**

**Figure S1**

*30% results:*
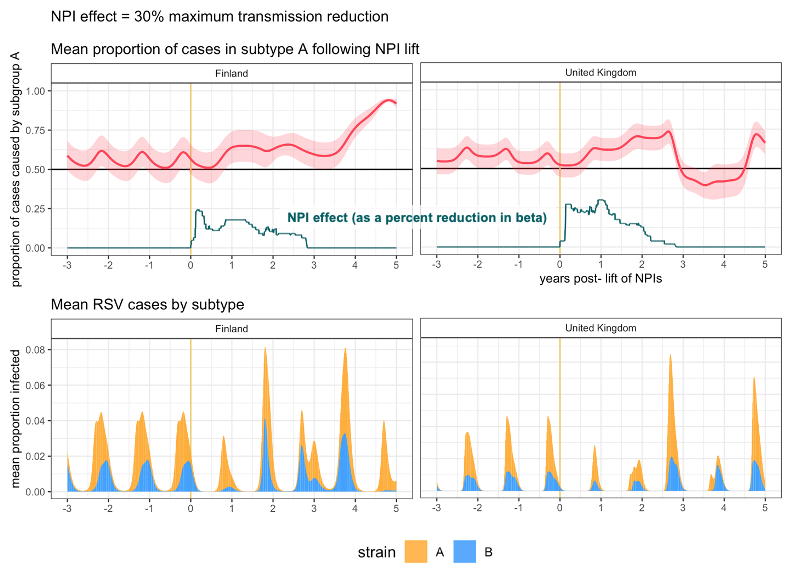


**Figure S2**

*50% results:*


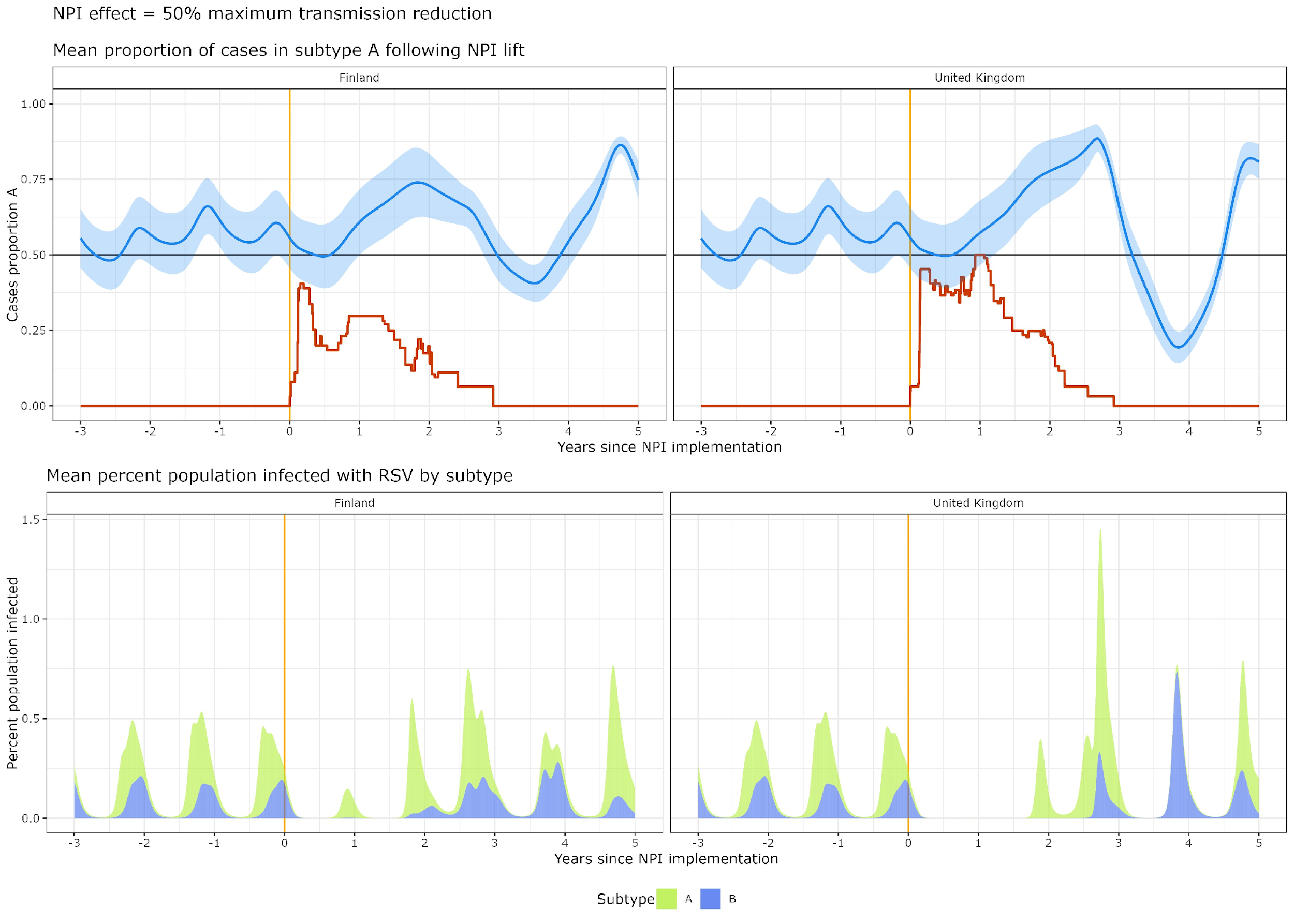


**Figure S3**


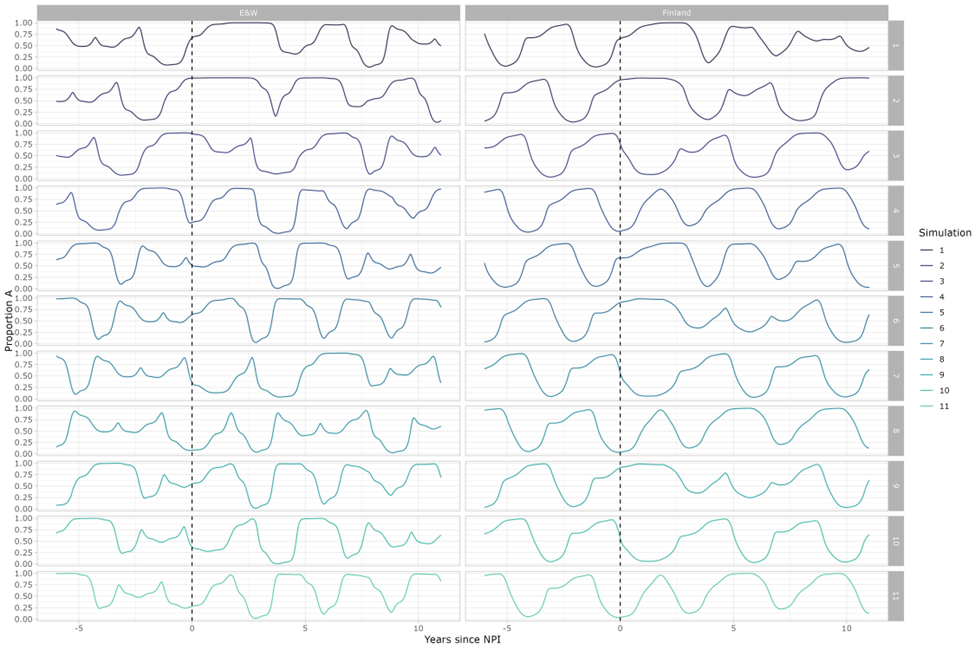


Individual simulations 1-11 showing the proportion of RSV infections that are subtype A in England & Wales and Finland.

**Figure S4**


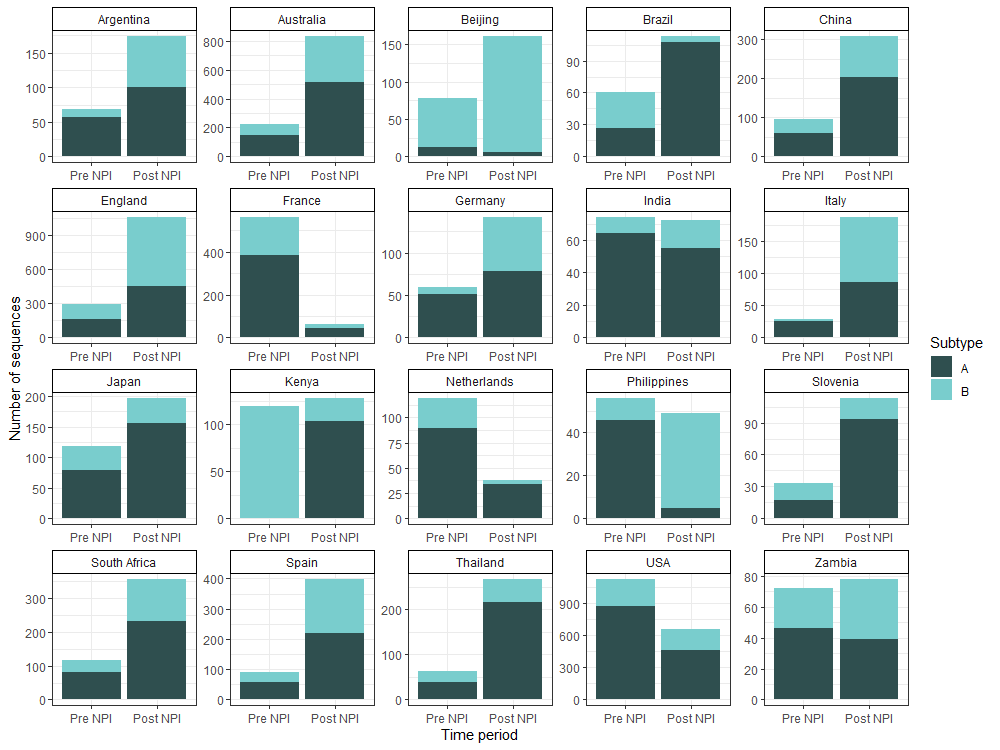


The number of RSV A and B samples reported in the GISAID data one year before the pandemic (pre NPI) and three years after the implementation of NPIs (post NPI) for each of the 20 locations included in the analysis.

**Figure S5**


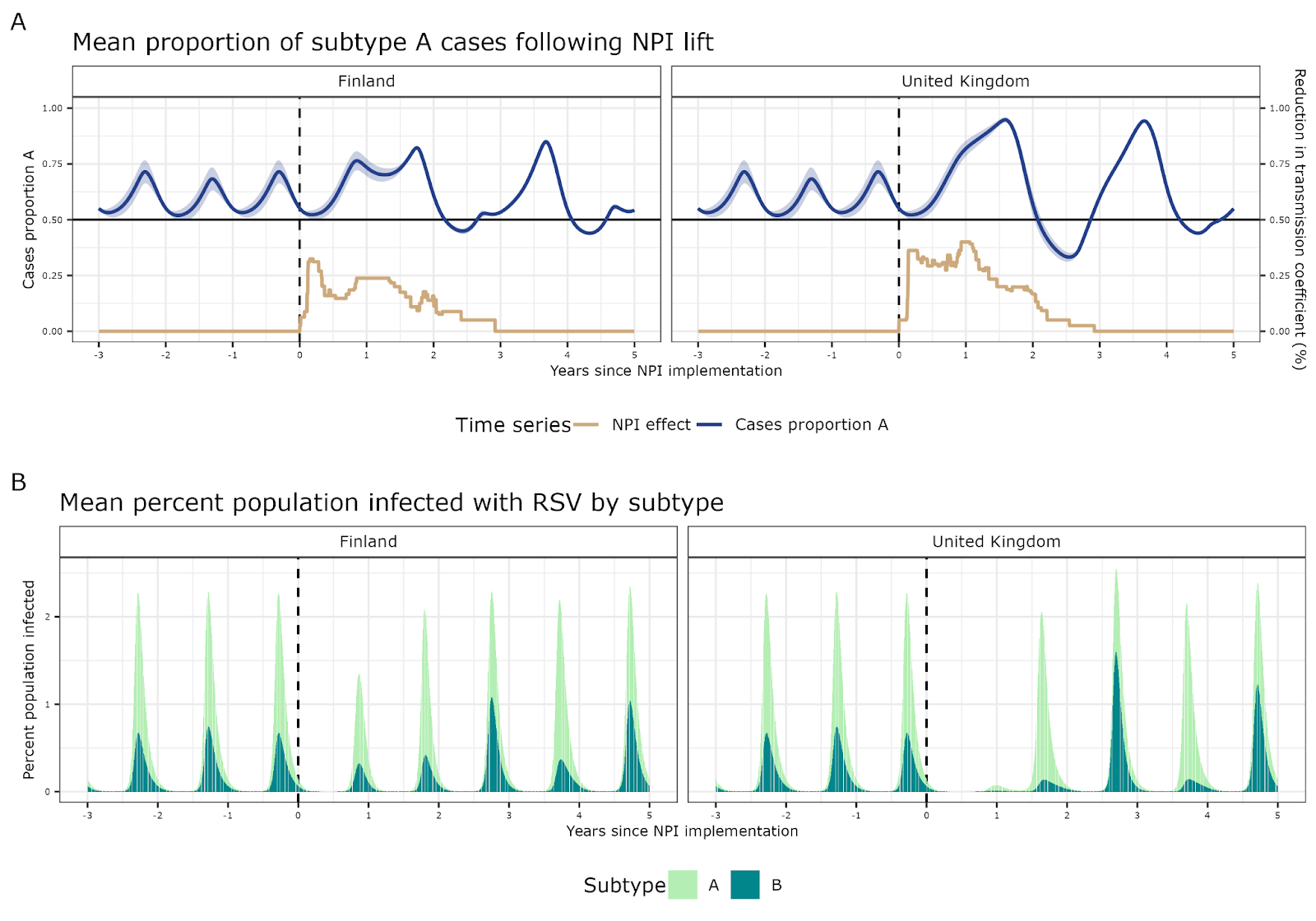
Here we change the immunity structure so that all individuals wane at rate 2w regardless of whether they have homotypic or heterotypic immunity and predict the impact of NPIs in both locations.
